# Association between targeted somatic mutation (TSM) signatures and HGS‐OvCa progression

**DOI:** 10.1002/cam4.825

**Published:** 2016-08-03

**Authors:** Robyn A. Lindley, Patrick Humbert, Cliff Larner, Eric H. Akmeemana, Christopher R. R. Pendlebury

**Affiliations:** ^1^ Department of PathologyUniversity of MelbourneParkvilleVictoria3010Australia; ^2^ GMDx Pty LtdMelbourneVictoria3000Australia; ^3^ Department of Biochemistry and Molecular BiologyUniversity of MelbourneParkvilleVictoria3010Australia; ^4^ Cell Cycle and Cancer Genetics LaboratoryPeter MacCallum Cancer CentreEast MelbourneVictoria3002Australia; ^5^ Sir Peter MacCallum Department of OncologyUniversity of MelbourneParkvilleVictoria3010Australia; ^6^ Swinburne University of TechnologyHawthornVictoria3132Australia; ^7^ HealthIQ Pty LtdMelbourneVictoria3000Australia; ^8^ AusBioCloud Pty LtdMelbourneVictoria3000Australia

**Keywords:** Cancer genetics, codon context, deaminase, ovarian cancer, targeted somatic mutation

## Abstract

Evidence already exists that the activation‐induced cytidine deaminase (AID/APOBEC) and the adenosine deaminase (ADAR) families of enzymes are implicated as powerful mutagens in oncogenic processes in many somatic tissues. Each deaminase is identified by the DNA or RNA nucleotide sequence (“motif”) surrounding the nucleotide targeted for deamination. The primary objective of this study is to develop an *in silico* approach to identify nucleotide sequence changes of the target motifs of key deaminases during oncogenesis. If successful, a secondary objective is to investigate if such changes are associated with disease progression indicators that include disease stage and progression‐free survival time. Using a discovery cohort of 194 high‐grade serous ovarian adenocarcinoma (HGS‐OvCa) exomes, the results confirm the ability of the novel *in silico* approach used to identify changes in the preferred target motifs for AID, APOBEC3G, APOBEC3B, and ADAR1 during oncogenesis. Using this approach, a set of new cancer‐progression associated signatures (C‐PASs) were identified. Furthermore, it was found that the C‐PAS identified can be used to differentiate between the cohort of patients that remained progression‐free for longer than 60 months, from those in which disease progressed within 60 months (sensitivity 95%, specificity 90%). The spectrum of outcomes observed here could provide a foundation for future clinical assessment of susceptibility variants in ovarian, and several other cancers as disease progresses. The ability of the *in silico* methodology used to identify changes in deaminase motifs during oncogenesis also suggests new links between immune system function and tumorigenesis.

## Introduction

The human genome encodes enzymes that deaminate single‐stranded DNA (ssDNA) or double stranded RNA (dsRNA) during transcription. They constitute antiviral defenses which few viruses survive because they become heavily mutated. Mutagenic deaminase activity also occurs in a controlled “beneficial” fashion during the antigen‐driven somatic hypermutation processes of immunoglobulin (Ig) genes expressed in B lymphocytes in Germinal Centres (GCs). When deaminase activity is dysregulated in somatic tissues, the same enzymes can attack nucleic acids causing the accumulation of unwanted *de novo* mutations that may result in cancer 1, 2, 3, 4.

There are two different deaminase families: cytidine deaminases, and adenosine deaminases. The cytidine deaminase family converts cytidines to uracil (C‐to‐U) in ssDNA. The most widely studied is activation‐induced cytidine deaminase (AID) which is expressed primarily in activated B cells undergoing Ig somatic hypermutation (SHM) and Ig class switch recombination. AID activity has been identified in early mutagenic events leading to cancers in nonlymphoid cells 5. AID targets both the transcribed strand (TS) and the nontranscribed strand (NTS) of ssDNA exposed during transcription 6, 7, 8. In a relevant example, the estrogen receptor complex binds to the AID promotor region causing a 20‐fold increase in AID production in breast and ovarian tissue 9. Estrogen protagonists such as the widely prescribed Tamoxifen, are therefore effective at inhibiting AID production in somatic tissue. AID preferentially targets the trinucleotide motifs WRC/GYW (where W = A/T, R = A/G and Y = T/C) for cytidine deamination 10.

The apolipoprotein B mRNA‐editing enzyme‐catalytic polypeptide‐like cytidine (APOBEC) deaminases, of which AID is a member, form a family of 11 or more orthologous cytidine deaminases. The human APOBEC3 deaminase family has a range of functions that protect against pathogens, such as retroviruses and DNA viruses with single‐stranded intermediates during, and post, transcription. APOBEC3G and APOBEC3B have been widely studied and they are expressed at significant levels in most tissues. APOBEC3G potently restricts the mobility of retrotransposons and pathogenic retroviruses such as human immunodeficiency virus (HIV) 11. APOBEC3G preferentially targets cytosine dinucleotides (CC/GG).

Several studies suggest that APOBEC3B‐catalyzed deaminations are responsible for a large proportion of mutations in a range of cancers, and that these also provide a chronic source of DNA damage in breast cancer and ovarian cancers 12, 13, 14. APOBEC3B preferentially targets the dinucleotide local sequence TC/GA.

The adenosine deaminases (ADARs) bind to dsRNA, converting adenosine to inosine (A‐to‐I) which now codes as guanosine during translation 15. ADAR1, ADAR2, and ADAR3 genes have been identified in humans. While ADAR1 preferentially targets WA sites in dsRNA, different splice variants of ADAR1 can exhibit different deaminase binding domains for adenosine targeting. Binding domains are essential for the function of many proteins. Here, the deaminase binding domain (DBD) refers to the protein domain of each deaminase which binds to a specific DNA or RNA nucleotide sequence (“motif”) that flanks the target cytosine or adenosine nucleotide for deamination. Furthermore, ADARs can self‐edit their active DBDs, and thereby alter their function 16. The resulting polymorphisms cause changes in the deaminase “motifs” targeted 17. Such variants also exhibit the potential for alternative ADAR targeting preferences during tumor progression.

A previous study of *TP53* mutations occurring in breast cancer has shown that the deaminase targeting preferences are highly specific, and that the molecular mechanisms involved rely upon the codon reading frame structure at the level of ssDNA in the nucleus during transcription 8. The molecular mechanisms involved also distinguish between cytidines on the “top” or nontranscribed strand (NTS), from those on the “bottom” or transcribed strand (TS) 8. Since dysregulated cytidine and adenosine deaminase activity is both highly targeted and heavily implicated in oncogenic processes, here we take a fundamentally new approach by focusing on the processes governing their behavior as disease progresses.

The newly discovered somatic mutation process involving the cytidine and adenosine deaminases during transcription is referred to as Targeted Somatic Mutation (TSM). To summarize, uncorrected (or “rogue”) TSM results in *de novo* mutations that: (1) occur at a particular nucleotide sequence, or “motif” that infers the DBD of a particular deaminase; (2) preferentially targets a particular site within the structure of the mutated codon (MC); and (3) results predominantly in one type of nucleotide change. An example of a TSM is a G‐to‐A transition, occurring off a GYW motif that is associated with AID activity, and occurring at the second nucleotide site within the MC structure (MC2, read 5‐prime to 3‐prime). The resulting transition is annotated as ‘G>A, off GYW at MC2′.

The specificity of cytidine and adenosine deaminases is thus governed by the codon‐contexted motifs defining the inferred DBDs for each isoform of a family member. APOBEC3G and APOBEC3B have two deaminase domains and AID has a single deaminase domain. Different isoforms of the respective DBDs determine the targeting preferences during deamination. In one study, it was concluded that the anti‐HIV activity of APOBEC3H is regulated by processes that result in different isoforms of the DBDs, and splice variants 18. Other studies reported that different APOBEC family DBD isoforms play an important role in modulating deamination 19, 20. More recently, it has also been reported that different isoforms of DBDs emerge as cancer progresses 21. Therefore, the emergence of different DBDs during oncogenesis is now an accepted concept in cancer progression studies.

It is hypothesized that new DBDs (as inferred by their preferred binding “motif”) for the main deaminases emerge as ovarian cancer progresses. The primary objective is to identify changes in nucleotide motifs for mutations associated with the mutagenic activity of some key deaminases (i.e., inferring new isoforms of DBDs), that are associated with disease progression indicators such as tumor stage, and progression‐free survival times. Given success at this step, a second objective is to investigate if such changes can be used to predict progression‐free survival, or stage progression of disease. This is important because, the potential motif specificity of the inferred DBDs might provide a way of defining new drug targets to inhibit both the rate and type of de novo mutations arising during oncogenesis.

To achieve these objectives, a cohort of 194 TCGA (The Cancer Genome Atlas) high‐grade serous ovarian adenocarcinomas (HGS‐OvCa) that have been previously studied, are used as a discovery cohort 22. Ovarian cancer is one disease for which a predictive diagnostic could be beneficial for clinicians. The average 5‐year survival is around 44% 23. As most deaths are the result of late‐stage diagnosis, understanding the contributing somatic genetic factors is important for developing new prognostic screening strategies.

## Materials and Methods

### Data source

Whole‐exome somatic mutation data were sourced from a cohort of 429 clinically annotated stage II‐IV HGS‐OvCa samples archived by The Cancer Genome Atlas of matched germline and tumor samples 22. The source of tumor and germline tissue is indicated by the code following the third hyphen in TCGA‐XX‐XXX‐YYY‐ZZZ 24 DNA was sequenced following exome capture on Illumina or SOLiD platforms. To produce aligned BAM files and call variants, TCGA processed the BAM files by aligning the sequence data to National Centre for Biotechnology I NCBI Build 36 of the human reference using BWA 0.5.9, and deduplicated using Picard 1.29.

Samples were selected from the 429 TCGA HGS‐OvCa samples using two selection criteria: (1) each sample included ≥60 single‐nucleotide variations (SNVs) to ensure that each sample had a sufficient number of mutations for profiling; and, (2) the clinical dataset included age at diagnosis, overall survival (in months), vital status, tumor stage, and tumor grade. In selecting samples with ≥60 single‐nucleotide variations (SNVs) as part of our data model, we sought a balance between decreasing predictive power by reducing the number of samples included in this study, and the need to increase the accuracy in the DBD (as defined by target motif) call rate. Of the 194 samples meeting these criteria, 172 were Caucasian, 15 were African‐American, one was American Alaskan Native, four were Asian, and two were of unknown ethnicity. Patient ages ranged from 39 to 84 years (median 60 years). The vast majority (98%) were diagnosed at late stage (at Stages III‐IV). At the time of TCGA sample procurement 47% (*n *=* *106) were deceased (Table S1).

Using the TCGA patient barcode as an identifier, cDNA mutation data for each sample were accessed via the Sanger Institute's Catalogue of Somatic Mutations (COSMIC) Whole Genome database v.72 on 1 August 2014. This step was included as COSMIC provides *vcf* cDNA file formats that can be directly imported into a desktop application developed by us for data analysis. Insertions, deletions, dinucleotide mutations, mutations with mismatched or missing information in any of the data fields were excluded. The remaining pooled dataset included 18,563 SNVs. Of these, there are 4446 (24.0%) silent mutations, 12,240 (65.9%) missense mutations, and 1877 (10.1%) are noted as unknown.

### Data treatment

The sample mutation.vcf files were directly uploaded for analysis using an Educational Research In Codon‐context (ERIC) Version 1.6 developed by us. This desktop application is scripted in Excel VBA, to automate the process of analyzing and compiling the mutation data for analysis. The program routine uses all Ensembl gene transcripts 25 to identify the nine nucleotide sequence context surrounding each mutation, and to determine the position of each mutation within the nucleotide structure of the MC. The resulting additional information for each mutation is included in the Table S2. ERIC v.1.6 was then used to call and tabulate mutations falling on predefined motifs of interest. Once the nucleotides of the selected motifs are entered into the configuration settings, all mutations off each motif were tabulated into a 3 × 3 table revealing the distribution of mutations (e.g., G‐to‐A/C/T), and their respective positions within the MC (i.e., positions MC1/2/3, read 5′ to 3′). The resulting 3 × 3 table is referred to as a TSM table.

### Data analysis

The first step was to verify the AID/APOBEC3G/APOBEC3B and ADAR families of deaminases as the likely source of many of the mutations in the pooled dataset. Motifs previously found to be associated with the mutagenic behavior of these deaminases were used to tabulate TSM tables. Nonoverlapping targeting preferences (“motifs”) include WRCG/CGYW for AID, and CCG/CGG for APOBEC3G 10. For ADAR activity, the motif WAY is selected, and for APOBEC3B, the motif TCG/CGA 14 was used. It is important to note that although different isoforms of the DBDs are known to exist for each deaminase, motifs inferred for the dominant DBDs of each deaminase have been chosen to ensure that there is no overlap among the selected deaminases (Table 1). The Chi square level of statistical significance for deviation from the expected distribution of mutations for each 3 × 3 table showing mutation type, and the location of mutations within the MC for each motif is included (*P *<* *0.001, 8 df).

**Table 1:** Targeted somatic mutation (TSM) profiles of mutations occurring at motifs of key deaminases associated with somatic mutagenesis for 194 high‐grade serous ovarian adenocarcinoma (HGS‐OvCa) exome samples

Deaminasefamily	Keymotif	Mutationtype	Mutated Codon Target Site (5′ to 3′)			Significance*P*‐value
			MC1	MC2	MC3	
AID	WRCG	C>A	7	17	17	
		C>G	17	16	34	1.54E‐97
		C>T	**138**	59	**141**	
	CGYW	G>A	58	**150**	69	
G>C	7	16	21	2.60E‐93
		G>T	11	15	13	
APOBEC3G	CCG	C>A	20	10	20	
		C>G	26	12	23	1.52E‐110
		C>T	**172**	69	130	
	CGG	G>A	88	**142**	136	
		G>C	22	27	16	3.58E‐88
		G>T	18	16	24	
APOBEC3B	TCG	C>A	17	3	28	
		C>G	26	12	11	1.77E‐48
		C>T	**90**	42	**83**	
	CGA	G>A	**135**	81	44	
		G>C	16	12	7	3.14E‐82
		G>T	27	18	7	
ADAR	WAY	A>C	27	58	21	
		A>G	110	**191**	71	4.46E‐61
		A>T	59	81	47	

For each motif, the preferred target sites are in bold. In the motifs defined, W = A/T, R  =  A/G, and Y = T/C. MC1, MC2, and MC3 refer to the position of the mutations within the mutated codon (MC), read 5‐prime (5′) to 3‐prime (3′). The Chi square level of statistical significance for deviation from the expected distribution of mutations in a 3 × 3 TSM table by type of mutation (×3) and location within the MC (×3) for each motif is shown in the right hand column (*P *<* *0.001, 8 df). The mutation data used to produce Table 1 are shown in the Table S2.). AID, activation‐induced cytidine deaminase; ADAR, adenosine deaminase; MC, mutated codon.

**Figure 1:**
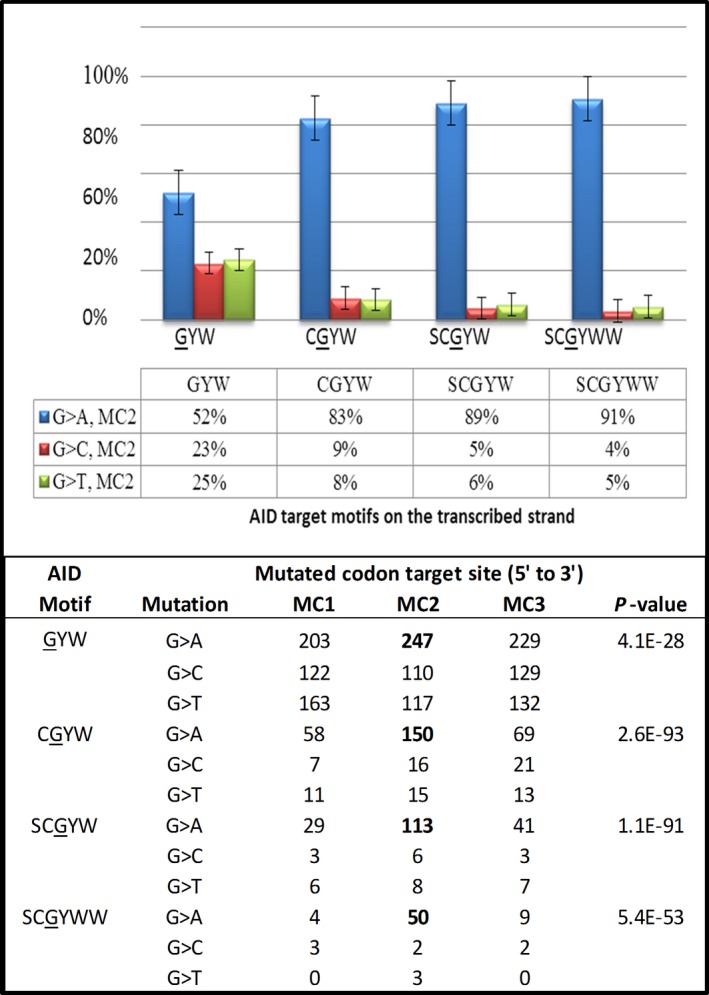
Graph showing how the targeting specificity for G‐to‐A transitions occurring in 194 TCGA high‐grade serous ovarian adenocarcinoma (HGS‐OvCa) samples is increased as the number of nucleotides defining the target motif is incrementally increased from three nucleotides (3N) to six nucleotides (6N). All of the selected motifs show a preference for targeting the second nucleotide position within the mutated codon (i.e., MC2 sites), and the dominant resulting mutation is a G‐to‐A transition. The percentage of each type of mutation off guanosine (G) at MC2 sites is shown. A targeted somatic mutation (TSM) 3 × 3 table for each motif, and showing all possible mutations and target sites within the MC is included. For the motifs, W = A/T, S = C/G and Y = T/C. MC1, MC2 and MC3 refer to the position of the mutations within the MC, read 5‐prime (5′) to 3‐prime (3′). The Chi square level of statistical significance *(P *<* *0.001, 8 df) for deviation from the expected distribution of mutations by type and location within the MC for each motif is shown in the column on the far right.

As all AID/APOBEC/ADAR family members are known to have multiple active DBDs, the chimeric nature of the key deaminases was then verified using the following investigative approach. As a first step and by way of example, the influence of changes in the 5′ and 3′ nucleotide context were demonstrated by starting with the AID preferred target motif GYW. Using ERIC v.1.6, and starting with GYW, the number of nucleotides were incrementally increased from the motif 3N to 6N. The results for each extension GYW (3N), CGYW (4N), SCGYW (5N), and SCGYWW (6N) were tabulated. Each additional nucleotide was only added as an extension if both the targeting preference and the resulting dominant type of mutation remained the same. All other possible extensions of the target motif were discarded. The results are shown in Figure 1. However, in adopting this approach, it should be noted that it cannot be confirmed that the resulting 6N motif allows binding of a particular isoform of AID without further experimentation.

Second, a table was constructed to investigate how changes in the 5′ and 3′ context of the targeting preferences for different motifs for AID and ADAR family members might alter the type of mutation and/or preferred target site of mutations within the MC. This approach also provides indirect verification of the polymorphic nature of these deaminases, and helps us to understand how changes in the 5′ or 3′ nucleotide context might alter the preferred target site(s) and/or the type of mutation produced (Table 2).

**Table 2:** Table of targeted somatic mutation (TSM) targets for motifs associated with ADAR and AID deaminase activity to demonstrate how targeting preferences are changed when the 5‐prime and 3‐prime nuclear context is altered for 194 high‐grade serous ovarian adenocarcinoma (HGS‐OvCa) tumors

DeaminaseEnzyme	TargetMotif	PreferredMutation	Target codonPosition	Sig. Level(*P* value)
ADARs	RAWA	A>T	MC1	1.37E‐09
	CWA	A>G	MC2	5.21E‐55
	GWA	A>G	MC3	9.02E‐17
	AWA	A>G	MC1	6.10E‐17
	AWG	A>G	MC2	1.91E‐47
	AWT	A>G	MC3	3.44E‐05
AID	WRCGS	C>T	MC1	5.00E‐67
	XWRCT	C>G	MC1	5.63E‐09
	WR**C**AW	C>T	MC3	2.90E‐12
	WGGYW	G>T	MC1	4.77E‐08
	SCGYW	G>A	MC2	1.13E‐91
	STGYW	G>A	MC3	4.83E‐15

W = A/T, S = C/G, R = A/G, Y = T/C and X = C/A. MC1, MC2, and MC3 refer to the position of the mutations within the mutated codon (MC), read 5‐prime (5′) to 3‐prime (3′). The Chi square level of statistical significance for deviation from the expected distribution of mutations in a 3 × 3 TSM table by type of mutation (×3) and location within the MC (×3) for each motif is shown in the right hand column (*P *<* *0.001, 8 df). AID, activation‐induced cytidine deaminase; ADAR, adenosine deaminase.

These two steps are included to ensure that the TSM profiling method is able to identify different isoforms of DBDs, and to indicate the likely optimum length of mutation targets. A wider analysis of a more complete set of DBD isoforms, and to validate unique DBDs as belonging to a single deaminase is outside of the scope of this study.

### Identifying cancer‐progression associated signatures (C‐PAS)

The “motifs” (i.e., the inferred DBDs) that have been identified as being associated with disease progression factors are referred to as Cancer‐Progression Associated Signatures (C‐PAS). To be classified as a C‐PAS, candidate motifs must satisfy two criteria. First, the 3 × 3 TSM table for a candidate C‐PAS must show a highly significant targeting preference (*P *<* *0.0001, 8 df). This ensured that the test motif is strongly associated with the mutagenic activity of a single DBD, and that the resulting TSM pattern did not arise by chance alone.

Second, the set of C‐PASs selected must show a significant difference between the average numbers of targeted mutations by progression status (significant at *P *<* *0.01). The available clinical data linked to disease progression status include the stage at diagnosis, and progression‐free survival time. As most patients were diagnosed at late stage, the samples were analyzed as three groups: Stage IIA‐IIIB (*n *=* *22), IIIC (*n *=* *142), and IV (*n *=* *30). Progression‐free survival time refers to the number of months that a patient was reported as “progression‐free” by the managing clinician, and commencing from the time of the initial diagnosis. The two cohorts compared were those that were living progression‐free for 60 months or more (*n *=* *14), and those that did not (*n *=* *96).

ERIC v.1.6 was used to identify a set of motifs meeting the two C‐PAS selection criteria. As it was not feasible to consider all possible nucleotide combinations, only variants of target motifs associated with the mutagenic activity of the key deaminases found in most (if not all) somatic tissues were considered (i.e., motifs for AID, APOBEC3G, APOBEC3B, and ADAR1). Fifteen DBDs satisfied the selection criteria, and were subsequently classified as C‐PASs (Table 3).

**Table 3:** Table showing the list of motifs for the Cancer‐Progression Associated Signatures (C‐PASs) associated with AID, APOBEC3B, APOBEC3G, and ADAR deaminase activity for 110 high grade serous ovarian adenocarcinoma tumors (HGS‐OvCa), and their association with recurrence and tumor stage indicators

Deaminasefamily	Targetmotif	Targetsite	*P*‐value	Living/Disease Free >60 months(*n* = 10)	Recurred/Progressed in <60 months(*n* = 100)	Stage		
						IIA‐IIIB(*n* = 22)	IIIC(*n* = 142)	IV(*n* = 30)
AID	WRCGSS	C>T MC1	5.2E‐39	0	12	5	35	5
APOBEC3B	TCGA	C>T MC1	5.8E‐12	1	7	8	13	4
	ATCS	C>T MC3	9.8E‐26	0	11	6	51	6
APOBEC3G	GCGGC	C>T MC1	1.1E‐25	0	7	1	26	3
	CCGX	C>T MC1	1.8E‐57	2	24	9	72	11
	ZCCG	C>T MC1	2.6E‐71	2	27	6	77	17
	SGGRR	G>A MC1	1.6E‐31	0	9	3	45	8
	TCCG	C>T MC1	3.3E‐45	0	4	3	29	5
	GCGC	G>A MC2	2.6E‐44	0	11	2	51	6
	CCGGC	G>A MC2	5.6E‐11	0	6	4	9	3
ADARs	RAWA	A>T MC1	1.4E‐09	0	9	6	29	11
	WTAW	A>G MC1	1.6E‐09	0	8	2	24	4
	SARA	A>G MC1	1.5E‐13	0	17	2	51	3
	TWTY	T>C MC2	1.1E‐10	0	16	8	47	5
	TWTY	T>C MC3	1.1E‐10	0	19	4	27	5
Total				5	187	69	586	96
Average/sample				0.5	1.87	3.1	4.1	3.2

The number of mutations for each motif is shown for patients living progression‐free for more than 60 months (*n *=* *10), and for those whose progression‐free survival time is less than 60 months (*n *=* *100). The number of mutations for each motif by tumor stage is shown in the last three columns. For overall comparison, the average number of mutations per sample is tabulated for each cohort. W = A/T, S = C/G, R = A/G, Y = T/C, X = C/A, and Z = G/T. MC1, MC2 and MC3 refer to the position of the mutations within the mutated codon (MC), read 5‐prime (5′) to 3‐prime (3′). The Chi square level of statistical significance for deviation from the expected distribution of mutations in a 3 × 3 targeted somatic mutation (TSM) table by type of mutation (×3) and location within the MC (×3) for each motif is shown in the right hand column (*P *<* *0.001, 8 df). AID, activation‐induced cytidine deaminase; ADAR, adenosine deaminase.

### Prognostication

**Figure 2:**
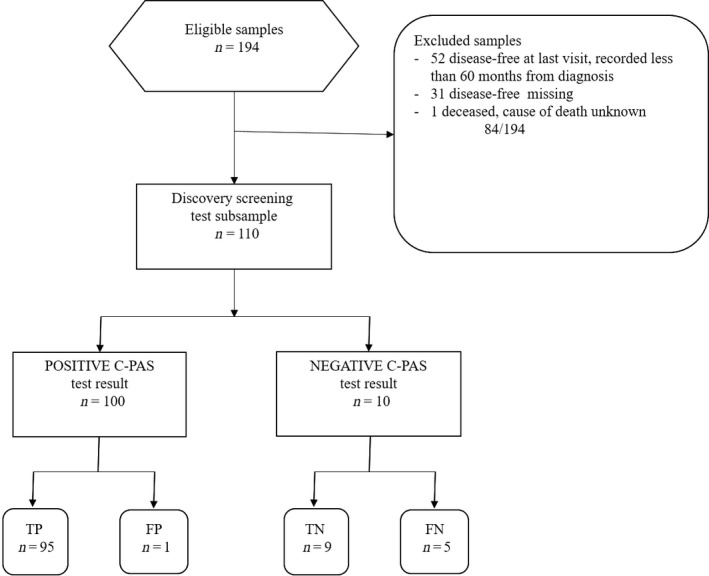
Standards for Reporting of Diagnostic Accuracy (STARD) flow diagram of the cancer‐progression associated signatures (C‐PASs) study discovery cohort of 194 high‐grade serous ovarian adenocarcinoma (HGS‐OvCa) exome samples.

**Figure 3:**
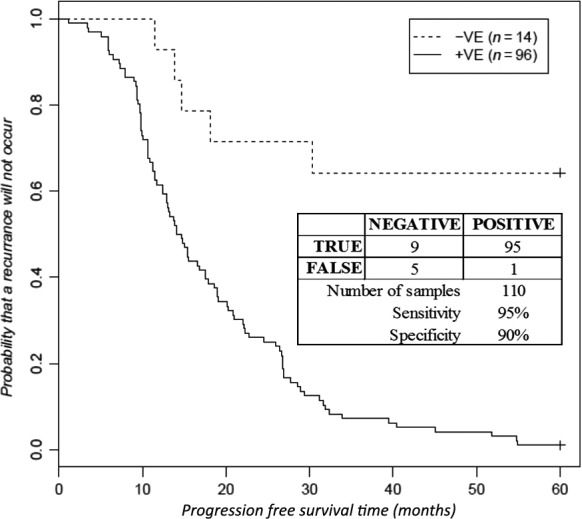
A Kaplan–Meier plot predicting progression‐free survival probabilities for 110 high‐grade serous ovarian adenocarcinoma (HGS‐OvCa) samples with a positive, or negative Cancer‐Progression Association Signature (C‐PAS) test result. A C‐PAS test outcome is denoted as positive if one or more mutations is detected at any of the C‐PAS motifs shown in Table 3. Similarly, a C‐PAS test result is denoted as negative if no mutations are found to occur at any of these C‐PAS test motifs. The Cox *P*‐value is 1.57E‐05, and the Log‐Rank *P* value is 7.86E‐07. The associated sensitivity (95%) and specificity (90%) measures are included in the inset. The mutation data and the test result for each sample are shown in the Table S1.

The set of C‐PAS identified were then used as a novel genetic assay to evaluate their collective prognostic potential for predicting progression‐free times, or stage progression. For each sample, a POSITIVE test result was indicated if one or more mutations by any one of the C‐PASs was identified. Similarly, a NEGATIVE test result was recorded for samples with no mutations off any of the C‐PASs identified. The test outcome for each sample was entered in the Clinical Data sheet (Table S1). The tabulated results are shown in Table 3. A Standards for Reporting of Diagnostic Accuracy (STARD) flow diagram of the cancer‐progression associated signatures (C‐PASs) study discovery cohort is shown in Figure 2 26. The results are also summarized in a Kaplan–Meier plot (Fig. 3) predicting the probability of recurrence for each test cohort for the 60 months following initial diagnosis. Statistical and survival analysis was conducted using R 27, 28. The Cox *P* and the Log‐Rank *P* values were calculated. Sensitivity and specificity measures for a test predicting disease progression were also calculated.

### Advantages and potential caveats of this study

The main advantage of the methods adopted for this study is the capacity of the TSM methodology to characterize the targeted nature, and underlying molecular processes involved in somatic mutagenesis. However, there are always uncertainties associated with *in‐silico*‐based clinical predictions. There are also limitations in the TCGA data which includes a lack of personal information and personal cancer history, lack of samples with an early stage diagnosis (Stage 1–II), and lack of progression‐free survival times. There are also confounding errors resulting from differences in the sequencing platforms used to generate the exome sequence data, and the methods used to call variants.

## Results

### HGS‐OvCa TSM profiles identifies differences in deaminase targeting preferences

The TSM profiles of motifs for AID (WRCG/CGYW), APOBEC3G (CCG/CGG), APOBEC3B (TCG/CGA), and ADAR1 (WAY) show a statistically significant bias for the sites preferentially targeted within the MC trinucleotide structure, and for the dominant type of mutation (Table 1, W = A/T, Y = T/C, and R = A/G). The Chi Square level of statistical significance for deviation from the expected for mutations off each motif in a 3 × 3 dataset is shown. In all cases, the observed deviation from the null hypothesis is highly significant (*P *<* *0.001, 8 df*)*.

For AID, APOBEC3G, and APOBEC3B motifs, there is a statistically significant MC1 and MC3 bias for C‐to‐T transitions. For motifs associated with AID and APOBEC3G mutagenic activity, cytidine deamination on the TS results in a dominant number of G‐to‐A transitions at MC2 sites. In contrast, for APOBEC3B, there is a strong preference for G‐to‐A transitions to target MC1 sites. For the ADAR motif WAY, the dominant mutations are A‐to‐G transitions at MC2 sites.

### DBD specificity is best defined by motifs with 4–6 nucleotides

A significant improvement in the targeting specificity of motifs for the catalytic domain of AID is observed as the number of nucleotides flanking the motif are incrementally increased from 3N to 6N (Fig. 1). The proportion of G‐to‐A transitions at MC2 sites increases from 52% of all mutations of guanosine for the 3N motif GYW, to 91% of all mutations of guanosine for the 6N motif SCGYWW. A table showing the mutation distributions for all possible mutations of guanosine (G‐to‐A/C/T), and target sites within the MC is included. Similar results have been found for APOBEC3G, APOBEC3B and ADAR targeting specificity (data not shown).

### Different isoforms of DBDs result in target site discrimination

The shift in targeting preferences of a range of ADAR motifs with a WA or AW base composition were compared. While it is recognized that there is some overlap between these motifs, the shifting target preferences observed are highly significant (Table 2). A shift in the location of the preferred target for putative isoforms of AID deaminase domains using the trinucleotide base motifs WRC/GYW were also compared (Table 2). In each example, the Chi Square level of statistical significance for deviation from the expected is shown for mutations off each motif in a 3 × 3 dataset (*P *<* *0.001, 8 df).

For each of the variations made to the nucleotide context of the inferred DBDs, the result is either a change in the dominant type of mutation produced, a shift in the preferred target site within the MC nucleotide structure, or both of these. For example, for the motif CWA an A‐to‐G transition preferentially targets MC2 sites. However when the “C” (cytidine) is replaced by “G” (guanosine), the result is an A‐to‐G transition targeting MC3 sites. Changes to the nucleotide motif context for other deaminases also demonstrate the kinetic nature of target site discrimination by mutation type and/or the target site within the MC (data not shown). Examples of changes made to the nucleotide context for inferred DBDs that did not result in either a change in the dominant type of resulting mutation, or the MC target site are not included.

Based on these findings and the work of others, the HGS‐OvCa data were next used as a training dataset to identify which specific inferred DBDs might be associated with cancer progression factors.

### Identifying cancer‐progression associated signatures (C‐PASs)

The 15 C‐PASs identified as satisfying the two selection criteria, and associated with AID, APOBEC3G, APOBEC3B, and ADAR deaminase activity, are each defined by a 4N‐5N inferred DBD that is responsible for introducing the *de novo* mutation (Table 3).

The total number of mutations per sample for the cohort of living patients that remained progression‐free for 60 months or more is 95.00 (*n = *10), whereas the average number of mutations per sample for the cohort of patients that did not remain progression‐free for 60 months or more is 97.55 (*n = *100). In the cohort of living patients that remained progression‐free for 60 months or more, the average number of mutations off C‐PASs per sample is 0.50. In patients with a progression‐free survival time of less 60 months, the average number of mutations per sample is increased more than threefold to 1.87. In comparing the average number of C‐PAS mutations per sample for each cohort, the Pearson product moment correlation (*r*) is 0.68 (significant at the *P *<* *0.001 level).

When the number of C‐PAS mutations per sample is compared by stage, it is found that the average number per sample for Stages IIA‐IIIB is 3.1, for Stage IIIC it is 4.1, and 3.2 for Stage IV (Table 3). In comparing the average number of C‐PAS mutations per sample for Stages IIA‐IIIB compared to Stage IIIC, the Pearson product moment correlation *r* is 0.28 (not significant at the *P *<* *0.05 level). However, it is found that the average number of C‐PAS mutations per sample for Stage IIIC is greater in comparison to Stage IV samples. Pearson product moment correlation *r* is 0.69 (significant at the *P* < 0.001 level).

### Using C‐PASs for prognostication

Although there are some limitations in the methods used to call the SNVs, the set of C‐PASs identified was used as a “first pass” reference test to predict progression‐free survival. The number of mutations occurring at motifs for each of the identified C‐PASs was tabulated for each sample. A negative test result was recorded if no mutations occurred at any of the identified C‐PASs, and as positive if there was one or more.

A Kaplan–Meier plot predicting progression‐free survival times for HGS‐OvCa samples with a positive test result, and compared to the cohort with a negative test result revealed that the difference between the two cohorts is highly significant (Fig. 2). The Cox *P*‐value is 1.57E‐05, and the Log‐Rank *P* value is 7.86E‐07. The sensitivity measure is 95%, and the specificity is 90%. The samples not included in these results are 52 samples that were progression‐free at their last visit which was recorded less than 60 months from the initial diagnosis, 31 samples with progression‐free survival data missing, and one that was deceased with the cause of death unknown (sample TCGA‐13‐1481). After 30 months, only 12% of patients with a positive C‐PAS test result remained progression‐free. After 60 months, only 1 patient of the 96 with a positive test result was progression‐free.

## Discussion

This study provides further confirmation of the importance of considering somatic mutation targeting in the context of a codon structure during transcription. The results demonstrate that the codon structure, and the 5′ and 3′ nucleotide sequence context are both critically important for furthering our knowledge of the molecular processes driving dysregulated deamination in somatic tissue. The resulting TSM profiles confirm the specificity of the targeting preferences for 4N‐6N motifs associated with the activity of previously studied deaminases, and thus provide an indication of the oligonucleotide sequence of the inferred DBDs that are responsible for *de novo* somatic mutations arising during oncogenesis. In order to suppress the rate at which *de novo* mutations occur, the inferred DBDs identified in a TSM profile for an individual are therefore potential new drug targets.

The observed targeting preferences for the deaminases studied are in general agreement with the results of a previous study of *TP53* gene mutations using pooled breast cancer mutation data 8. However, an unexpected finding was an observed difference between the targeting preferences for motifs associated with AID and APOBEC3G deaminase activity. In the *TP53* breast cancer data, the majority of C‐to‐T transitions at motifs associated with AID and APOBEC3G deaminase activity were found to preferentially target MC1 sites, with few or no transitions occurring at MC2 or MC3 sites. In the HGS‐OvCa samples used in this study, the majority of C‐to‐T transitions off motifs associated with AID and APOBEC3G deaminase activity were found to preferentially target both MC1 and MC3 sites**.** As most of the HGS‐OvCa samples analyzed in this study were diagnosed at late stage, and breast cancer is usually diagnosed at an earlier stage, further investigation is required to establish if this shift in cytidine targeting preference for C‐to‐T transitions from MC1 to MC3 sites for HGS‐OvCa samples is associated with a late‐stage diagnosis.

The mutation targeting preferences off motifs associated with ADAR deaminase activity are consistent with the previous study of *TP53* gene mutations for pooled breast cancer data 8. Both datasets reveal a dominant ADAR target preference for A‐to‐G transitions at MC2 sites. However, further molecular evidence is needed to establish whether or not the motifs for this targeting preference is due exclusively to an ADAR1 DBD isoform, and whether or not an ADAR2 DBD is targeting the MC1 sites. It is also possible that an immunologically related splice variant form of ADAR1 is preferentially targeting the MC1 sites 29. Both ADAR1 and ADAR2 are found in many tissues. Dysregulation of ADAR1 and ADAR2 expression and conformation have been linked to cancer phenotypes, and a general decrease in RNA editing is associated with disease progression 30. More recently, the gene for ADAR1 has been identified as a tumor promotor, and the gene for ADAR2 as a tumor suppressor 31. Thus, understanding how ADAR editing patterns are regulated, and how these alter TSM profiles will be important for advancing our knowledge of the role of ADARs during oncogenesis, even before tumor development. This will require using both molecular experimentation and *in silico* analyses.

In this study it was also demonstrated for the first time that the motif CGA associated with APOBEC3B deaminase activity and resulting in G‐to‐A transitions, preferentially targets MC1 sites (Table 2), whereas for the motifs CGG associated with APOBEC3G, and CGYW associated with AID, the resulting dominant G‐to‐A transitions preferentially target MC2 sites. Further investigation is required to determine if this distinction in target site discrimination can be used to differentiate between the deaminase activity of APOBEC3B and AID/APOBEC3G deaminases.

The fine specificity and the TSM kinetics shown in Table 2 and Figure 1 is in general agreement with *in vitro* studies that have established that nucleotides 5′ and 3′ of a target cytidine, can strongly influence the type of mutations produced, as well as their efficiency in terms of the relative number of potential target sites deaminated 32, 33. The results add further support to the hypothesis that DBD isoforms involving one or more nucleotide changes will result in target site discrimination, and that the resulting transformations can be used to identify new DBD isoforms arising during oncogenesis.

Another key finding of this study, is that the C‐PASs identified in Table 3 can provide the basis for the development of a novel genetic test to predict the likelihood of disease progression. A Kaplan–Meier plot comparing the progression‐free survival probabilities for cohorts with either a negative or a positive test result are used to estimate the probability of progression of disease within 5 years after the initial diagnosis (Fig. 3). The results of this study are consistent with another recent study of TCGA HGS‐OvCa samples that used four expression subtypes and survival gene expression signatures 34. While the results showed an ability to predict outcomes based on a response to therapy, the methods used did not further our understanding of the changes giving rise to new DBDs during oncogenesis. In contrast, the results reported in this study demonstrate the potential clinical utility of TSM profiling for identifying C‐PAS, and for predicting the probability of disease progression. However, it is important to note that the cohort of 14 patients yielding a negative test result included five false negatives. In comparison, there is only one false‐positive test result among 96 samples. It is speculated that there may be other factors responsible for promoting disease progression. Another explanation is the use of different methods to obtain the matched germline samples to call SNVs using exome capture techniques and resulting in some mutations occurring at C‐PAS sites being excluded from the mutation dataset. While it is not within the scope of this study, future studies may also examine the role of the C‐PASs identified here in conjunction with the genes targeted for mutation. In examining the individual profiles of the five false‐negative cases, it was noticed that sample TCGA‐29‐1696 had 4 G‐to‐C transversions, all of which were off a GG motif at an MC1 site of the *KRAS* gene at c.34 that may impact its functions. None of the other 193 samples have any mutations in the *KRAS* gene.

In contrast, the data did not show a meaningful association with stage progression. The unexpected decrease in the average number of C‐PAS mutations per sample from Stage IIIC to Stage IV shown in Table 3 is statistically significant (*P *<* *0.01), and might be explained by the tissue sampling methods used to call SNVs. In this regard the SNVs called in apparently normal or healthy tissue can impact cancer data or their interpretation. It has been found that half or more SNVs in cancers could have arisen prior to the development of a tumor 35. Recently, it has also been reported that more than a quarter of the apparently healthy human epidermal skin cells harbor cancer‐causing somatic mutations 36. In our view, once TSM processes become active *via* an at present unknown “switching mechanism” involving an immune‐like response in apparently normal tissue, then it is likely that individual somatic cells in a tissue will harbor a diverse range of genetic changes. Apparently healthy tissue in late Stage IV cancer may therefore harbor many of the mutations associated with the C‐PASs identified in Stage IIIC. In this case, the tissue matching methods used by the TCGA to call SNVs, will exclude those variants identified in both samples. This means that many of the mutations associated with C‐PASs may be excluded from the mutation datasets produced, and may partly explain the observed decrease from Stage IIIC to IV (Table 3). Much further work is needed to understand what triggers rogue deaminase activity, and its association with stage progression factors during tumorigenesis. Moreover, possible proapoptotic/tumor suppressor roles of such genetic changes in tumor cells should also be investigated.

In conclusion, TSM profiling presents us with a fundamentally new genomic analysis toolkit for identifying some important differences in isomorphic forms of DBDs arising in an individual during oncogenesis. TSM profiling can be used to provide important information for the diagnosis of disease associated with “rogue” deaminase activity. More importantly, TSM profiling methods can characterize the genetic mutation links between SHM, immune system function and oncogenic processes, and risk stratification involving a dysregulated AID, APOBEC and ADAR response in somatic tissue. While the results reported in this study demonstrate the potential utility of adopting a TSM approach based on codon‐contexted information, much further work is required to validate the links between the inferred changes in the DBDs identified, changes in immune system function and phenotypic changes as disease progresses. Caveats on the TCGA dataset used here as a discovery dataset have been noted. Limitations in the number of samples available with matching clinical data, and differences among the sequencing platforms an variant call methods used, all cast some doubt on the reproducibility of the results. While we provide the first evidence showing that changes in DBD occur and may in fact predict progression/recurrence of disease, caution is required in interpreting the results. Further studies are required to confirm or reject the hypotheses and data reported here, to validate clinically useful DBDs, and to provide independent measures of specificity and sensitivity.

## Conflict of Interest

None declared.

## Supporting information


**Table S1**. Clinical Data. (Lindley et al., 2015).

**Table S2**. Mutation Data. (Lindley et al., 2015).

**Table S3**. Mutation Data. (Lindley et al., 2015) TSM Mutations.
