# Supplementary material for: Association between targeted somatic mutation (TSM) signatures and HGS‐OvCa progression
Source: Cancer Med. 2016 Aug 3;5(9):2629–40. doi: 10.1002/cam4.825 (PMC5055158; doi:10.1002/cam4.825)
Supplement: Supplementary file 1 — Table S1. Clinical Data. (Lindley et al., 2015). [file CAM4-5-2629-s001.pdf]

Supporting Information Table S1. Clinical Data. (Lindley et al, 2015)

CLINICAL DATA									
Patient Barcode	Age at Diagnosis (yrs)	Vital Status	Tumor Stage	Tumor Grade	Overall Survival	Progression Free Survival (mths)	Progression Free Status	Total Mutations	Test Result
TCGA-29-1784	55.37	LIVING	IIIC	G3	5.34	5.34	DiseaseFree	122	NEGATIVE
TCGA-20-1685	45.88	LIVING	IIIC	G3	16.62	16.62	DiseaseFree	79	NEGATIVE
TCGA-61-1915	50.46	LIVING	IIC	G3	67.70	67.70	DiseaseFree	118	NEGATIVE
TCGA-13-0890	56.90	LIVING	IIIC	G3	67.80	67.80	DiseaseFree	57	NEGATIVE
TCGA-09-2050	65.72	LIVING	IIA	G2	70.92	70.92	DiseaseFree	109	NEGATIVE
TCGA-04-1367	50.89	LIVING	IIIC	G3	71.28	71.28	DiseaseFree	89	NEGATIVE
TCGA-61-2094	63.75	LIVING	IIIC	G3	71.67	71.67	DiseaseFree	56	NEGATIVE
TCGA-13-0886	67.73	LIVING	IIIC	G3	74.07	74.07	DiseaseFree	70	NEGATIVE
TCGA-23-1118	45.12	LIVING	IIIC	G3	85.77	85.77	DiseaseFree	64	NEGATIVE
TCGA-23-2078	66.36	LIVING	IIIC	G3	87.25	87.25	DiseaseFree	103	NEGATIVE
TCGA-13-0885	70.58	LIVING	IIIC	G3	91.15	91.15	DiseaseFree	171	NEGATIVE
TCGA-13-0793	40.29	LIVING	IV	G3	28.62	11.51	Recurred/Progressed	57	NEGATIVE
TCGA-13-0761	51.22	LIVING	IV	G3	33.97	13.84	Recurred/Progressed	106	NEGATIVE
TCGA-61-2095	54.23	DECEASED	IIIC	G2	61.54	14.66	Recurred/Progressed	91	NEGATIVE
TCGA-13-1499	56.71	LIVING	IIIC	G3	37.28	18.10	Recurred/Progressed	65	NEGATIVE
TCGA-04-1361	57.15	LIVING	IIIB	G3	32.43	30.36	Recurred/Progressed	124	NEGATIVE
TCGA-23-1809	63.32	LIVING	IIC	G3	0.52	0.52	DiseaseFree	70	POSITIVE
TCGA-61-1895	52.21	LIVING	IIIB	G3	1.54	1.54	DiseaseFree	66	POSITIVE
TCGA-13-1512	49.75	LIVING	IIIC	G3	2.33	2.33	DiseaseFree	57	POSITIVE
TCGA-13-1510	62.21	LIVING	IIIC	G3	2.56	2.56	DiseaseFree	112	POSITIVE
TCGA-20-1687	46.47	LIVING	IV	G3	2.62	2.62	DiseaseFree	94	POSITIVE
TCGA-20-1686	75.58	LIVING	IIIC	G3	2.92	2.92	DiseaseFree	87	POSITIVE
TCGA-23-1111	63.48	LIVING	IIIC	G3	3.21	3.21	DiseaseFree	61	POSITIVE
TCGA-24-1470	54.81	LIVING	IIIB	G3	3.41	3.41	DiseaseFree	82	POSITIVE
TCGA-24-1843	67.04	LIVING	IIIC	G3	3.48	3.48	DiseaseFree	97	POSITIVE
TCGA-24-1844	64.50	LIVING	IIIC	G3	3.70	3.70	DiseaseFree	175	POSITIVE
TCGA-24-1845	42.74	LIVING	IIIC	G3	3.80	3.80	DiseaseFree	168	POSITIVE
TCGA-13-1509	64.34	LIVING	IV	G3	4.07	4.07	DiseaseFree	80	POSITIVE

TCGA-13-1505	63.67	LIVING	IIIC	G3	4.20	4.20	DiseaseFree	58	POSITIVE
TCGA-23-1120	60.68	LIVING	IIIC	G3	4.26	4.26	DiseaseFree	73	POSITIVE
TCGA-24-1846	45.30	LIVING	IIIC	G3	4.36	4.36	DiseaseFree	121	POSITIVE
TCGA-23-1116	83.46	LIVING	IIIC	G3	4.56	4.56	DiseaseFree	58	POSITIVE
TCGA-13-1507	77.93	LIVING	IIIC	G3	4.75	4.75	DiseaseFree	86	POSITIVE
TCGA-24-1850	72.24	LIVING	IIIC	G3	5.48	5.48	DiseaseFree	89	POSITIVE
TCGA-13-0924	45.70	LIVING	IV	G3	5.67	5.67	DiseaseFree	62	POSITIVE
TCGA-24-1849	80.97	LIVING	IIIC	G3	5.74	5.74	DiseaseFree	92	POSITIVE
TCGA-61-1900	51.95	LIVING	IIIB	G3	5.80	5.80	DiseaseFree	110	POSITIVE
TCGA-09-2044	77.34	LIVING	IIB	G3	6.10	6.19	DiseaseFree	84	POSITIVE
TCGA-24-1423	61.04	LIVING	IIIC	G3	6.23	6.23	DiseaseFree	63	POSITIVE
TCGA-13-0923	74.19	LIVING	IIIC	G3	6.39	6.39	DiseaseFree	138	POSITIVE
TCGA-29-1783	58.31	LIVING	IIIC	G3	7.18	7.18	DiseaseFree	72	POSITIVE
TCGA-24-1417	54.18	LIVING	IV	G3	7.80	7.80	DiseaseFree	58	POSITIVE
TCGA-29-1781	69.60	LIVING	IIIC	G3	8.33	8.33	DiseaseFree	110	POSITIVE
TCGA-61-1899	81.32	LIVING	IIIC	G3	8.69	8.69	DiseaseFree	65	POSITIVE
TCGA-23-1029	46.27	LIVING	IIIC	G3	8.79	8.79	DiseaseFree	87	POSITIVE
TCGA-24-1469	71.06	LIVING	IIIC	G3	9.08	9.08	DiseaseFree	153	POSITIVE
TCGA-61-1904	60.29	LIVING	IIIC	G3	9.18	9.18	DiseaseFree	102	POSITIVE
TCGA-24-1847	45.33	LIVING	IV	G3	10.75	10.75	DiseaseFree	96	POSITIVE
TCGA-09-2056	62.64	LIVING	IIIC	G3	12.43	12.43	DiseaseFree	73	POSITIVE
TCGA-13-0919	52.98	LIVING	IIIC	G3	14.85	14.85	DiseaseFree	52	POSITIVE
TCGA-13-0916	49.83	LIVING	IIIC	G3	21.08	21.08	DiseaseFree	72	POSITIVE
TCGA-29-1769	40.42	LIVING	IIIC	G3	22.92	22.92	DiseaseFree	94	POSITIVE
TCGA-20-0991	78.69	LIVING	IIB	G3	25.25	25.25	DiseaseFree	83	POSITIVE
TCGA-36-1577	43.30	LIVING	IIC	G2	25.67	25.67	DiseaseFree	50	POSITIVE
TCGA-61-2012	81.72	LIVING	IIC	G2	30.62	30.62	DiseaseFree	114	POSITIVE
TCGA-13-0762	65.64	LIVING	IIIC	G3	32.16	32.16	DiseaseFree	146	POSITIVE
TCGA-29-1711	45.16	LIVING	IIIC	G2	34.52	34.52	DiseaseFree	116	POSITIVE
TCGA-29-1699	57.89	DECEASED	IIIC	G3	36.26	36.26	DiseaseFree	95	POSITIVE
TCGA-61-1910	56.92	LIVING	IIC	G3	37.05	37.05	DiseaseFree	155	POSITIVE
TCGA-61-1737	42.55	LIVING	IV	G3	44.23	44.23	DiseaseFree	91	POSITIVE
TCGA-13-0906	50.36	LIVING	IIIC	G3	44.82	44.82	DiseaseFree	83	POSITIVE

TCGA-13-0905	51.39	LIVING	IIIC	G3	47.70	47.70	DiseaseFree	62	POSITIVE
TCGA-25-2391	57.36	DECEASED	IIIC	G3	48.92	48.92	DiseaseFree	54	POSITIVE
TCGA-04-1336	55.53	LIVING	IIIB	G3	49.02	49.02	DiseaseFree	60	POSITIVE
TCGA-61-1913	48.12	LIVING	IIIB	G3	49.08	49.08	DiseaseFree	61	POSITIVE
TCGA-13-0900	59.47	LIVING	IIIC	G3	56.59	56.59	DiseaseFree	90	POSITIVE
TCGA-04-1347	81.36	LIVING	IV	G3	62.89	62.89	DiseaseFree	116	POSITIVE
TCGA-13-1481	76.43	DECEASED	IIIC	G2	86.82	86.82	DiseaseFree	104	POSITIVE
TCGA-13-0755	75.17	DECEASED	IV	G3	2.46	1.11	Recurred/Progressed	97	POSITIVE
TCGA-61-2009	65.12	LIVING	IIIC	G3	39.87	3.44	Recurred/Progressed	65	POSITIVE
TCGA-13-0714	55.10	DECEASED	IV	G3	6.10	3.51	Recurred/Progressed	67	POSITIVE
TCGA-04-1356	62.61	DECEASED	IIC	G3	49.15	5.11	Recurred/Progressed	66	POSITIVE
TCGA-61-1733	71.72	LIVING	IIIC	G3	31.80	5.84	Recurred/Progressed	124	POSITIVE
TCGA-29-1698	53.73	LIVING	IIIC	G3	68.10	5.90	Recurred/Progressed	30	POSITIVE
TCGA-30-1891	61.22	DECEASED	IIIC	G2	29.97	5.93	Recurred/Progressed	71	POSITIVE
TCGA-25-1318	54.12	DECEASED	IIIC	G3	34.89	6.07	Recurred/Progressed	114	POSITIVE
TCGA-24-1431	67.83	DECEASED	IIIC	G3	19.11	6.52	Recurred/Progressed	59	POSITIVE
TCGA-30-1856	56.51	DECEASED	IIIC	G3	15.64	7.18	Recurred/Progressed	74	POSITIVE
TCGA-04-1362	59.58	DECEASED	IIC	G3	44.20	7.31	Recurred/Progressed	67	POSITIVE
TCGA-30-1714	68.58	DECEASED	IV	G3	37.97	7.90	Recurred/Progressed	74	POSITIVE
TCGA-61-1906	55.81	DECEASED	IIIC	G3	34.23	7.93	Recurred/Progressed	130	POSITIVE
TCGA-09-0369	56.73	DECEASED	IIIC	G3	35.44	8.95	Recurred/Progressed	138	POSITIVE
TCGA-13-0791	58.57	LIVING	IIIC	G3	38.82	9.21	Recurred/Progressed	69	POSITIVE
TCGA-29-1705	48.03	DECEASED	IIIC	G2	18.20	9.28	Recurred/Progressed	58	POSITIVE
TCGA-61-1738	60.16	DECEASED	IIIC	G3	35.93	9.28	Recurred/Progressed	150	POSITIVE
TCGA-29-1702	84.68	DECEASED	IIIC	G3	23.87	9.34	Recurred/Progressed	63	POSITIVE
TCGA-24-2035	65.35	DECEASED	IIIC	G3	28.10	9.41	Recurred/Progressed	83	POSITIVE
TCGA-13-0795	66.94	LIVING	IIIC	G3	18.56	9.70	Recurred/Progressed	71	POSITIVE
TCGA-13-0920	65.93	LIVING	IIIC	G3	13.77	9.74	Recurred/Progressed	111	POSITIVE
TCGA-13-0904	63.86	LIVING	IIIC	G3	47.18	9.77	Recurred/Progressed	115	POSITIVE
TCGA-04-1638	57.50	DECEASED	IV	G3	55.25	9.77	Recurred/Progressed	62	POSITIVE
TCGA-29-1701	56.81	DECEASED	IIIC	G3	16.89	9.77	Recurred/Progressed	59	POSITIVE
TCGA-61-2113	54.01	DECEASED	IIC	G3	22.39	9.77	Recurred/Progressed	75	POSITIVE
TCGA-24-0975	58.68	DECEASED	IIIC	G3	21.74	9.87	Recurred/Progressed	55	POSITIVE

TCGA-25-2393	81.05	DECEASED	IIIC	G3	37.93	10.00	Recurred/Progressed	56	POSITIVE
TCGA-24-1464	70.89	DECEASED	IIIC	G3	12.43	10.59	Recurred/Progressed	55	POSITIVE
TCGA-29-1703	56.50	LIVING	IIIC	G2	55.08	10.62	Recurred/Progressed	99	POSITIVE
TCGA-24-2290	56.24	DECEASED	IIIC	G3	36.13	10.62	Recurred/Progressed	116	POSITIVE
TCGA-23-1110	42.15	LIVING	IIIC	G3	54.36	10.69	Recurred/Progressed	102	POSITIVE
TCGA-24-1474	57.27	DECEASED	IIIC	G3	22.13	10.85	Recurred/Progressed	61	POSITIVE
TCGA-29-1775	51.90	LIVING	IIIC	G2	12.33	11.18	Recurred/Progressed	124	POSITIVE
TCGA-29-1696	43.34	DECEASED	IIIC	G2	33.84	11.21	Recurred/Progressed	170	POSITIVE
TCGA-04-1530	68.53	DECEASED	IIIC	G3	118.75	11.48	Recurred/Progressed	63	POSITIVE
TCGA-13-1488	59.52	DECEASED	IV	G3	70.62	11.51	Recurred/Progressed	126	POSITIVE
TCGA-13-1501	50.49	LIVING	IV	G3	31.57	11.67	Recurred/Progressed	142	POSITIVE
TCGA-29-1770	54.83	LIVING	IIIC	G2	24.30	12.36	Recurred/Progressed	98	POSITIVE
TCGA-04-1338	78.87	LIVING	IIIC	G3	46.49	12.46	Recurred/Progressed	135	POSITIVE
TCGA-25-1313	62.96	DECEASED	IV	G3	26.85	12.92	Recurred/Progressed	145	POSITIVE
TCGA-25-1326	61.20	DECEASED	IIIC	G3	40.92	12.95	Recurred/Progressed	141	POSITIVE
TCGA-24-1563	66.50	DECEASED	IIIC	G3	47.57	12.98	Recurred/Progressed	75	POSITIVE
TCGA-13-0893	48.31	DECEASED	IIIC	G3	41.31	13.18	Recurred/Progressed	81	POSITIVE
TCGA-24-1616	56.95	DECEASED	IIIC	G3	38.13	13.28	Recurred/Progressed	60	POSITIVE
TCGA-23-1117	42.99	DECEASED	IIIC	G3	33.21	13.70	Recurred/Progressed	208	POSITIVE
TCGA-24-2024	72.53	DECEASED	IIIC	G3	58.00	13.80	Recurred/Progressed	79	POSITIVE
TCGA-29-1763	43.89	LIVING	IIC	G2	66.59	14.03	Recurred/Progressed	124	POSITIVE
TCGA-24-0979	53.68	DECEASED	IV	G3	41.44	14.03	Recurred/Progressed	91	POSITIVE
TCGA-23-1122	53.53	DECEASED	IIIC	G3	38.98	14.66	Recurred/Progressed	117	POSITIVE
TCGA-23-1022	67.94	DECEASED	IIIC	G3	49.54	14.75	Recurred/Progressed	184	POSITIVE
TCGA-04-1331	79.04	DECEASED	IIIC	G3	43.80	15.05	Recurred/Progressed	93	POSITIVE
TCGA-09-1665	74.00	DECEASED	IIIC	G2	41.48	15.34	Recurred/Progressed	94	POSITIVE
TCGA-61-2109	40.80	DECEASED	IIIC	G3	20.82	15.44	Recurred/Progressed	61	POSITIVE
TCGA-29-1785	55.94	DECEASED	IIIC	G3	36.20	15.51	Recurred/Progressed	162	POSITIVE
TCGA-24-1435	57.83	DECEASED	IIIC	G3	43.41	16.56	Recurred/Progressed	77	POSITIVE
TCGA-13-0887	42.93	DECEASED	IIIC	G3	66.46	16.82	Recurred/Progressed	110	POSITIVE
TCGA-13-1498	73.48	LIVING	IIIC	G3	37.51	17.48	Recurred/Progressed	116	POSITIVE
TCGA-24-2280	74.95	LIVING	IIIC	G3	70.26	17.51	Recurred/Progressed	151	POSITIVE
TCGA-29-1768	50.40	DECEASED	IV	G3	31.21	17.93	Recurred/Progressed	57	POSITIVE

TCGA-24-2289	69.02	DECEASED	IV	G3	67.18	18.62	Recurred/Progressed	149	POSITIVE
TCGA-25-2398	71.96	DECEASED	IIIC	G3	44.89	18.92	Recurred/Progressed	52	POSITIVE
TCGA-25-2400	76.13	DECEASED	IIIC	G3	41.90	18.92	Recurred/Progressed	77	POSITIVE
TCGA-09-2051	42.88	LIVING	IIIC	G3	62.92	19.11	Recurred/Progressed	94	POSITIVE
TCGA-29-1691	51.13	DECEASED	IIIC	G2	48.20	20.10	Recurred/Progressed	197	POSITIVE
TCGA-20-1683	65.77	LIVING	IIIC	G3	25.31	20.26	Recurred/Progressed	105	POSITIVE
TCGA-23-1114	55.92	DECEASED	IIIC	G3	68.49	20.79	Recurred/Progressed	110	POSITIVE
TCGA-23-1124	63.03	DECEASED	IIIC	G3	57.97	21.02	Recurred/Progressed	145	POSITIVE
TCGA-29-1766	74.96	DECEASED	IIIC	G2	39.28	22.03	Recurred/Progressed	75	POSITIVE
TCGA-24-1103	50.91	DECEASED	IIIC	G3	53.93	22.20	Recurred/Progressed	82	POSITIVE
TCGA-10-0930	70.51	DECEASED	IIIC	G3	34.10	22.30	Recurred/Progressed	304	POSITIVE
TCGA-29-1693	72.05	LIVING	IIIC	G3	101.48	22.75	Recurred/Progressed	103	POSITIVE
TCGA-61-1907	63.56	LIVING	IIIC	G3	31.38	24.52	Recurred/Progressed	130	POSITIVE
TCGA-29-1764	49.35	LIVING	IIIC	G2	62.75	25.97	Recurred/Progressed	86	POSITIVE
TCGA-13-1489	70.60	LIVING	IIIC	G2	82.66	26.43	Recurred/Progressed	54	POSITIVE
TCGA-13-1497	48.02	LIVING	IIIC	G3	54.16	26.66	Recurred/Progressed	132	POSITIVE
TCGA-20-0990	74.59	LIVING	IIIC	G3	24.07	26.72	Recurred/Progressed	81	POSITIVE
TCGA-13-0792	40.35	LIVING	IIIC	G3	36.62	26.75	Recurred/Progressed	57	POSITIVE
TCGA-13-0883	61.36	DECEASED	IIIC	G3	68.72	26.82	Recurred/Progressed	67	POSITIVE
TCGA-04-1655	49.28	DECEASED	IIIB	G2	45.21	26.85	Recurred/Progressed	66	POSITIVE
TCGA-61-2008	40.51	LIVING	IIC	G2	30.69	26.95	Recurred/Progressed	61	POSITIVE
TCGA-61-1725	40.49	LIVING	IIIC	G3	31.44	27.77	Recurred/Progressed	64	POSITIVE
TCGA-13-0913	53.62	LIVING	IIIC	G3	29.90	28.56	Recurred/Progressed	79	POSITIVE
TCGA-04-1652	76.98	DECEASED	IIIC	G2	31.87	28.95	Recurred/Progressed	176	POSITIVE
TCGA-30-1718	44.93	DECEASED	IIIC	G3	51.77	29.38	Recurred/Progressed	125	POSITIVE
TCGA-09-2049	64.14	LIVING	IIIC	G3	112.07	31.21	Recurred/Progressed	120	POSITIVE
TCGA-24-1104	56.22	DECEASED	IV	G3	63.57	31.61	Recurred/Progressed	58	POSITIVE
TCGA-24-1463	70.50	DECEASED	IIIC	G3	72.72	31.84	Recurred/Progressed	59	POSITIVE
TCGA-04-1651	53.78	DECEASED	IIIC	G3	36.07	32.39	Recurred/Progressed	130	POSITIVE
TCGA-04-1542	52.78	DECEASED	IIIB	G2	83.97	33.93	Recurred/Progressed	67	POSITIVE
TCGA-13-0884	39.96	DECEASED	IIIC	G3	105.31	39.44	Recurred/Progressed	89	POSITIVE
TCGA-23-2077	45.35	LIVING	IIIC	G3	115.57	40.39	Recurred/Progressed	72	POSITIVE
TCGA-29-1762	59.94	DECEASED	IV	G2	86.36	45.05	Recurred/Progressed	77	POSITIVE

TCGA-29-2427	60.11	LIVING	IIIC	G3	62.30	51.84	Recurred/Progressed	70	POSITIVE
TCGA-04-1649	74.42	LIVING	IIIC	G3	64.46	54.82	Recurred/Progressed	69	POSITIVE
TCGA-61-1914	65.33	LIVING	IIIC	G3	56.52	54.95	Recurred/Progressed	110	POSITIVE
TCGA-13-0760	63.23	DECEASED	IV	G3	10.10	Missing	DiseaseFree	186	POSITIVE
TCGA-13-0807	54.49	DECEASED	IIIC	G3	13.57	Missing	DiseaseFree	61	POSITIVE
TCGA-13-1496	65.80	DECEASED	IIIC	G3	3.41	Missing	DiseaseFree	64	POSITIVE
TCGA-24-1422	82.83	DECEASED	IIIC	G3	0.75	Missing	DiseaseFree	128	POSITIVE
TCGA-25-2392	75.80	DECEASED	IV	G3	1.02	Missing	DiseaseFree	104	POSITIVE
TCGA-25-2401	64.96	DECEASED	IIIC	G3	2.95	Missing	DiseaseFree	65	POSITIVE
TCGA-29-1695	62.72	DECEASED	IIIC	G2	40.30	Missing	DiseaseFree	74	POSITIVE
TCGA-13-1408	59.27	LIVING	IIIC	G3	5.90	Missing	Recurred/Progressed	56	NEGATIVE
TCGA-29-1776	63.06	LIVING	IIIC	G3	11.80	Missing	Recurred/Progressed	47	NEGATIVE
TCGA-09-1674	79.12	LIVING	IIIC	G3	8.52	Missing	Recurred/Progressed	157	POSITIVE
TCGA-13-0903	43.03	LIVING	IV	G3	47.25	Missing	Recurred/Progressed	58	POSITIVE
TCGA-29-1777	47.55	LIVING	IIIC	G2	12.26	Missing	Recurred/Progressed	155	POSITIVE
TCGA-61-1998	48.91	LIVING	IIIC	G3	5.61	Missing	Recurred/Progressed	95	POSITIVE
TCGA-04-1337	78.42	DECEASED	IIIC	G2	2.03	Missing	Recurred/Progressed	48	POSITIVE
TCGA-04-1342	80.82	DECEASED	IV	G2	18.46	Missing	Recurred/Progressed	73	POSITIVE
TCGA-04-1343	72.41	DECEASED	IV	G3	11.84	Missing	Recurred/Progressed	62	POSITIVE
TCGA-23-1021	45.16	DECEASED	IV	G3	47.41	Missing	Recurred/Progressed	83	POSITIVE
TCGA-23-1031	60.89	DECEASED	IV	G3	18.82	Missing	Recurred/Progressed	107	POSITIVE
TCGA-23-1032	73.19	DECEASED	IV	G3	2.75	Missing	Recurred/Progressed	97	POSITIVE
TCGA-23-1123	59.04	DECEASED	IIIC	G3	33.38	Missing	Recurred/Progressed	176	POSITIVE
TCGA-24-1604	67.04	DECEASED	IIIC	G3	88.10	Missing	Recurred/Progressed	60	POSITIVE
TCGA-24-2262	57.15	DECEASED	IIIC	G3	0.36	Missing	Recurred/Progressed	69	POSITIVE
TCGA-24-2267	58.34	DECEASED	IIB	G3	47.41	Missing	Recurred/Progressed	115	POSITIVE
TCGA-24-2288	70.77	DECEASED	IIIC	G3	0.82	Missing	Recurred/Progressed	105	POSITIVE
TCGA-25-2042	60.62	DECEASED	IIIC	G3	12.98	Missing	Recurred/Progressed	74	POSITIVE
TCGA-29-1690	66.62	DECEASED	IIIC	G2	47.48	Missing	Recurred/Progressed	54	POSITIVE
TCGA-29-1761	80.38	DECEASED	IIIC	G3	17.31	Missing	Recurred/Progressed	201	POSITIVE
TCGA-30-1855	61.35	DECEASED	IIIC	G3	2.46	Missing	Recurred/Progressed	129	POSITIVE
TCGA-30-1857	64.91	DECEASED	IV	G3	0.13	Missing	Recurred/Progressed	96	POSITIVE
TCGA-61-1740	71.68	DECEASED	IIIC	G3	2.62	Missing	Recurred/Progressed	212	POSITIVE

TCGA-61-2102	74.66	DECEASED	IIIC	G3	6.56	Missing	Recurred/Progressed	62	POSITIVE
