# Supplementary material for: Association between targeted somatic mutation (TSM) signatures and HGS‐OvCa progression
Source: Cancer Med. 2016 Aug 3;5(9):2629–40. doi: 10.1002/cam4.825 (PMC5055158; doi:10.1002/cam4.825)
Supplement: Supplementary file 2 — Table S2. Mutation Data. (Lindley et al., 2015). [file CAM4-5-2629-s002.pdf]

Supporting Information: Table S2. Mutation Data. (Lindley et al, 2015)

Sample	Gene	Transcript	AA Mutation	CDS Mutation	Validated	5'codon	Mutation	3'Codon	MC	WT	MUT
TCGA-13-0913	AADACL4	ENST00000376221	p.K175Q		Unverified	CTG	AAG	GCC	1	A	C
TCGA-25-2392	ACIN1	ENST00000262710	p.Q752P	c.2255A>C	Verified	ACT	CAG	ACC	2	A	C
TCGA-13-0890	ADAMTS20	ENST00000389420	p.H542P	c.1625A>C	Verified	CGT	CAT	GGG	2	A	C
TCGA-13-0885	ADAMTS9	ENST00000498707	p.Q916P	c.2747A>C	Verified	gat	caa	aga	2	A	C
TCGA-13-0795	ADPRH	ENST00000357003	p.Q218P	c.653A>C	Verified	CTT	CAA	CAC	2	A	C
TCGA-29-1775	AHCTF1	ENST00000391829	p.Q2180P	c.6539A>C	Unverified	GCA	CAA	TCA	2	A	C
TCGA-13-0807	AKAP4	ENST00000358526	p.E291A	c.872A>C	Verified	gga	gag	gag	2	A	C
TCGA-24-1844	AKNA	ENST00000307564	p.P1338P	c.4014A>C	Unverified	CCT	CCA	GCC	3	A	C
TCGA-13-1497	ALG2	ENST00000476832	p.H193P	c.578A>C	Verified	TCT	CAC	ATA	2	A	C
TCGA-29-1777	ALK	ENST00000389048	p.P1142P	c.3426A>C	Unverified	gac	cca	agc	3	A	C
TCGA-13-1481	ANGPTL4	ENST00000301455	p.N177H	c.529A>C	Verified	CAC	AAT	GTC	1	A	C
TCGA-24-2262	ANKFY1	ENST00000574367	p.R1006R	c.3016A>C	Verified	CTC	AGA	GGC	1	A	C
TCGA-24-0975	ANKRD30A	ENST00000361713	p.Q1260P	c.3779A>C	Unverified	CAG	CAG	GAG	2	A	C
TCGA-24-2288	ANKRD7	ENST00000357099	p.K270Q	c.808A>C	Unverified	AAG	AAG	AAA	1	A	C
TCGA-61-1906	AP3D1	ENST00000345016	p.D1075A	c.3224A>C	Unverified	GTC	GAT	GGC	2	A	C
TCGA-61-1906	AP3D1	ENST00000345016	p.D1075A	c.3224A>C	Unverified	GTC	GAT	GGC	2	A	C
TCGA-61-2012	ARL4C	ENST00000390645	p.Q188P	c.563A>C	Unverified	aag	cag	aag	2	A	C
TCGA-24-1845	ASTN2	ENST00000361209	p.T567P	c.1699A>C	Unverified	AAG	ACC	GAT	1	A	C
TCGA-30-1857	ATF7	ENST00000328463	p.H310P	c.929A>C	Unverified	CAG	CAC	CCT	2	A	C
TCGA-30-1857	ATF7_ENST(	ENST00000548446	p.H310P	c.929A>C	Unverified	CAG	CAC	CCT	2	A	C
TCGA-13-0791	ATXN3L	ENST00000380622	p.T146T	c.438A>C	Verified	gat	aca	tgc	3	A	C
TCGA-13-0900	BCL7A	ENST00000538010	p.R195R	c.583A>C	Verified	CAG	AGG	GGC	1	A	C
TCGA-13-1488	BMX	ENST00000357607	p.S212R	c.634A>C	Verified	acc	agt	cta	1	A	C
TCGA-23-1122	C10orf18	ENST00000328090	p.T1647T	c.4941A>C	Verified	TCT	ACA	CAG	3	A	C
TCGA-61-1907	C10orf92	ENST00000263170	p.T166P	c.496A>C	Unverified	GCC	ACA	AAG	1	A	C
TCGA-61-1725	C12orf40	ENST00000324616	p.E369A	c.1106A>C	Unverified	CCA	GAA	AAA	2	A	C
TCGA-13-0885	C13orf26	ENST00000380473	p.T77P	c.229A>C	Verified	TAC	ACT	TGG	1	A	C
TCGA-61-1998	C14orf102	ENST00000354366	p.H438P	c.1313A>C	Unverified	ATT	CAC	AGT	2	A	C
TCGA-04-1338	C15orf42	ENST00000268138	p.E840A	c.2519A>C	Unverified	CCT	GAA	TCT	2	A	C
TCGA-23-1029	C17orf28	ENST00000425042	p.S98R	c.292A>C	Unverified	TGC	AGC	CGG	1	A	C
TCGA-25-1326	C17orf87	ENST00000574081	p.P122P	c.366A>C	Verified	ATC	CCA	AGC	3	A	C
TCGA-29-1768	C19orf18	ENST00000314391	p.E130A	c.389A>C	Unverified	GCT	GAG	GAA	2	A	C

TCGA-04-1331	C1orf173	ENST00000326665	p.E666A	c.1997A>C	Verified	TTT	GAG	AAT	2	A	C
TCGA-23-1124	C3orf36	ENST00000408895	p.R54R	c.160A>C	Verified	CTC	AGG	AAG	1	A	C
TCGA-13-0906	C4A_ENST0(	ENST00000428956	p.Q1111P	c.3332A>C	Unverified	CAG	CAG	CAG	2	A	C
TCGA-13-0906	C4B	ENST00000428956	p.Q1111P	c.3332A>C	Unverified	CAG	CAG	CAG	2	A	C
TCGA-24-1844	C6orf211	ENST00000367294	p.A17A	c.51A>C	Unverified	TTT	GCA	TAT	3	A	C
TCGA-23-1110	C7_ENST000	ENST00000313164	p.E585A	c.1754A>C	Verified	GAT	GAA	GGT	2	A	C
TCGA-23-1120	CASP9	ENST00000333868	p.Q245P	c.734A>C	Unverified	ctg	cag	ttc	2	A	C
TCGA-29-1764	CC2D1B	ENST00000371586	p.A373A	c.1119A>C	Unverified	CCA	GCA	ACC	3	A	C
TCGA-61-2012	CCDC141	ENST00000343876	p.E600A	c.1799A>C	Verified	GAA	GAG	CGA	2	A	C
TCGA-10-0930	CCDC80	ENST00000206423	p.K656Q	c.1966A>C	Verified	AGG	AAA	ATC	1	A	C
TCGA-10-0930	CCDC80	ENST00000206423	p.K656Q	c.1966A>C	Verified	AGG	AAA	ATC	1	A	C
TCGA-09-2044	CD109	ENST00000287097	p.R1114R	c.3340A>C	Verified	TGG	AGA	GCA	1	A	C
TCGA-04-1651	CD1C	ENST00000368170	p.S328S	c.984A>C	Unverified	TGC	TCA	TAT	3	A	C
TCGA-04-1651	CD1C	ENST00000368170	p.S328S	c.984A>C	Unverified	TGC	TCA	TAT	3	A	C
TCGA-13-0905	CECR1	ENST00000399837	p.E194A	c.581A>C	Verified	CCG	GAG	GTG	2	A	C
TCGA-24-2280	CELF6	ENST00000287202	p.R390S	c.1170A>C	Unverified	CAG	AGA	GAA	3	A	C
TCGA-13-1505	CEP164	ENST00000278935	p.H1452P	c.4355A>C	Verified	GAG	CAC	AAC	2	A	C
TCGA-61-1914	CEP70	ENST00000264982	p.E333A	c.998A>C	Unverified	GAT	GAG	CCC	2	A	C
TCGA-20-1687	CHD2	NM_001271.1	p.D677A	c.2030A>C	Unverified	gaa	gac	cat	2	A	C
TCGA-20-1687	CHD2_ENST(	ENST00000394196	p.D677A	c.2030A>C	Unverified	GAA	GAC	CAT	2	A	C
TCGA-13-0906	CHUK	ENST00000370397	p.K237Q	c.709A>C	Verified	AAG	AAG	GAT	1	A	C
TCGA-24-2267	CLEC11A	ENST00000250340	p.H281P	c.842A>C	Verified	CCG	CAT	CCG	2	A	C
TCGA-23-1031	CNOT1	ENST00000317147	p.N1377H	c.4129A>C	Verified	CTG	AAT	CCA	1	A	C
TCGA-24-2267	CNTRL	ENST00000238341	p.Q482P	c.1445A>C	Verified	ATA	CAA	CTA	2	A	C
TCGA-23-1117	COL15A1	ENST00000375001	p.K920Q	c.2758A>C	Verified	CTC	AAG	GGT	1	A	C
TCGA-23-1117	COL15A1	ENST00000375001	p.K920Q	c.2758A>C	Verified	CTC	AAG	GGT	1	A	C
TCGA-61-1910	COL4A5	ENST00000361603	p.S41R	c.121A>C	Unverified	tgc	agt	ggc	1	A	C
TCGA-13-0884	COL6A3	ENST00000295550	p.T3076T	c.9228A>C	Verified	AGT	ACA	AAG	3	A	C
TCGA-29-1698	COMMD9	ENST00000263401	p.I153L	c.457A>C	Unverified	AAG	ATC	CAA	1	A	C
TCGA-29-1777	CRISPLD1	ENST00000262207	p.T13T	c.39A>C	Unverified	ACC	ACA	GTG	3	A	C
TCGA-24-1849	CRMP1	ENST00000324989	p.I356L	c.1066A>C	Unverified	ACC	ATT	GCG	1	A	C
TCGA-61-1907	CSMD3	ENST00000297405	p.K320Q	c.958A>C	Unverified	aac	aaa	aac	1	A	C
TCGA-61-1907	CSMD3_ENS	ENST00000343508	p.K280Q	c.838A>C	Unverified	AAC	AAA	AAC	1	A	C
TCGA-23-1029	CST4	ENST00000217423	p.K114Q	c.340A>C	Unverified	CAG	AAG	AAA	1	A	C

TCGA-25-1326	CTNNAL1	ENST00000325551	p.S387R	c.1159A>C	Verified	atc	agt	cac	1	A	C
TCGA-13-0755	CTTNBP2	ENST00000160373	p.G721G	c.2163A>C	Verified	CAG	GGA	AAT	3	A	C
TCGA-61-2095	CYP2A6	ENST00000301141	p.N407H	c.1219A>C	Unverified	TCC	AAC	CCC	1	A	C
TCGA-04-1542	DAGLA	ENST00000257215	p.D717A	c.2150A>C	Verified	GCA	GAC	CAC	2	A	C
TCGA-13-0904	DDB2	ENST00000256996	p.T338P	c.1012A>C	Verified	CTC	ACA	CCC	1	A	C
TCGA-29-1781	DICER1	ENST00000343455	p.E275A	c.824A>C	Unverified	tta	gaa	gaa	2	A	C
TCGA-29-1696	DIO3	ENST00000359323	p.N138H	c.412A>C	Unverified	CTC	AAT	TTC	1	A	C
TCGA-29-1696	DIO3	ENST00000359323	p.N138H	c.412A>C	Unverified	CTC	AAT	TTC	1	A	C
TCGA-29-1696	DIO3_ENST(	ENST00000510508	p.N164H	c.490A>C	Unverified	CTC	AAT	TTC	1	A	C
TCGA-29-1696	DIO3_ENST(	ENST00000510508	p.N164H	c.490A>C	Unverified	CTC	AAT	TTC	1	A	C
TCGA-04-1336	DLG5	ENST00000372391	p.R1339R	c.4015A>C	Verified	CGG	AGA	AAG	1	A	C
TCGA-29-1764	DMBT1	ENST00000368909	p.R1161R	c.3481A>C	Unverified	TGC	AGG	CAG	1	A	C
TCGA-29-1770	DMBT1	ENST00000368909	p.I2097L	c.6289A>C	Unverified	TCC	ATT	CGC	1	A	C
TCGA-29-1764	DMBT1_ENS	ENST00000368909	p.R1161R	c.3481A>C	Unverified	TGC	AGG	CAG	1	A	C
TCGA-29-1770	DMBT1_ENS	ENST00000368909	p.I2097L	c.6289A>C	Unverified	TCC	ATT	CGC	1	A	C
TCGA-29-1764	DMBT1_ENS	ENST00000368915	p.R1161R	c.3481A>C	Unverified	TGC	AGG	CAG	1	A	C
TCGA-29-1770	DMBT1_ENS	ENST00000368915	p.I2226L	c.6676A>C	Unverified	TCC	ATT	CGC	1	A	C
TCGA-24-1563	DMXL2	ENST00000251076	p.P485P	c.1455A>C	Verified	ATG	CCA	CTG	3	A	C
TCGA-29-1784	DNAH17	ENST00000300671	p.H3620P	c.10859A>C	Unverified	AAG	CAC	ACA	2	A	C
TCGA-13-0885	DNAH5	ENST00000265104	p.N3204H	c.9610A>C	Verified	ATG	AAT	ACT	1	A	C
TCGA-29-2427	DNAH8	NM_001371.1	p.Q2843P	c.8528A>C	Verified	ttc	cag	ata	2	A	C
TCGA-04-1343	DPYD	ENST00000370192	p.P462P	c.1386A>C	Verified	CTC	CCA	GAA	3	A	C
TCGA-23-1124	DSCAM	ENST00000400454	p.D1690A	c.5069A>C	Verified	GCT	GAC	TTT	2	A	C
TCGA-09-2044	DSN1	ENST00000373750	p.T118T	c.354A>C	Verified	ATC	ACA	GAG	3	A	C
TCGA-23-1124	DYNC1I1	ENST00000324972	p.T566P	c.1696A>C	Verified	GAC	ACC	GAG	1	A	C
TCGA-30-1718	EFTUD1	ENST00000268206	p.Q931P	c.2792A>C	Unverified	CTA	CAA	GAT	2	A	C
TCGA-61-1910	EHD4	ENST00000220325	p.I168L	c.502A>C	Unverified	CGC	ATC	AGC	1	A	C
TCGA-23-1123	ENSG00000:	ENST00000157775	p.Q157P	c.470A>C	Verified	GCG	CAG	CCC	2	A	C
TCGA-23-1123	ENSG00000:	ENST00000157775	p.Q157P	c.470A>C	Verified	GCG	CAG	CCC	2	A	C
TCGA-23-1117	ENSG00000:	ENST00000322049	p.A249A	c.747A>C	Verified	TGT	GCA	GTC	3	A	C
TCGA-23-1117	ENSG00000:	ENST00000322049	p.A249A	c.747A>C	Verified	TGT	GCA	GTC	3	A	C
TCGA-30-1855	ENSG00000:	ENST00000439556	p.Q307P	c.920A>C	Unverified	AAG	CAA	TGT	2	A	C
TCGA-09-2056	EP400	ENST00000389561	p.H1938P	c.5813A>C	Verified	ACT	CAC	AGC	2	A	C
TCGA-25-1326	EPC1	ENST00000263062	p.H732P	c.2195A>C	Verified	TCC	CAC	AGT	2	A	C

TCGA-61-1904	EPDR1	ENST00000559325	p.H97P	c.290A>C	Unverified	AGC	CAC	TCT	2	A	C
TCGA-23-2077	EPHB2	NM_017449	p.A142A	c.426A>C	Unverified	att	gca	gcc	3	A	C
TCGA-25-1318	EPHB3	ENST00000330394	p.I560L	c.1678A>C	Unverified	ctc	atc	gtg	1	A	C
TCGA-25-1318	EPHB3	ENST00000330394	p.I560L	c.1678A>C	Unverified	ctc	atc	gtg	1	A	C
TCGA-29-1769	ERC2	ENST00000288221	p.Q939P	c.2816A>C	Unverified	TCG	CAA	CAT	2	A	C
TCGA-29-1769	ERC2_ENST(	ENST00000288221	p.Q939P	c.2816A>C	Unverified	TCG	CAA	CAT	2	A	C
TCGA-04-1338	ERCC6L	ENST00000334463	p.Q1246P	c.3737A>C	Verified	AAG	CAA	CTT	2	A	C
TCGA-13-0762	EXDL2	ENST00000409014	p.D148A	c.443A>C	Verified	GAT	GAC	CAC	2	A	C
TCGA-13-0762	EXDL2	ENST00000409014	p.D148A	c.443A>C	Verified	GAT	GAC	CAC	2	A	C
TCGA-29-1764	EYS	ENST00000342421	p.T363T	c.1089A>C	Unverified	ttt	aca	gat	3	A	C
TCGA-29-1764	EYS_ENST00	ENST00000370621	p.T363T	c.1089A>C	Unverified	TTT	ACA	GAT	3	A	C
TCGA-29-1703	FAM111A	ENST00000528737	p.K315Q	c.943A>C	Unverified	TTC	AAG	AAA	1	A	C
TCGA-23-1123	FAM171B	ENST00000304698	p.H762P	c.2285A>C	Verified	AGG	CAC	ATC	2	A	C
TCGA-23-1123	FAM171B	ENST00000304698	p.H762P	c.2285A>C	Verified	AGG	CAC	ATC	2	A	C
TCGA-24-1422	FAM53B	ENST00000337318	p.T249T	c.747A>C	Unverified	AGC	ACA	CCT	3	A	C
TCGA-13-0923	FARSB	ENST00000281828	p.N503H	c.1507A>C	Verified	TAC	AAC	AAG	1	A	C
TCGA-24-1843	FAT2	ENST00000261800	p.I1404L	c.4210A>C	Unverified	GTC	ATT	GCC	1	A	C
TCGA-24-1843	FAT3_ENST(	ENST00000298047_v61	p.K3860Q	c.11578A>C	Unverified	TTC	AAA	CTA	1	A	C
TCGA-24-1843	FAT3_ENST(	ENST00000409404	p.K3860Q	c.11578A>C	Unverified	TTC	AAA	CTA	1	A	C
TCGA-13-0755	FLG	ENST00000368799	p.R3483S	c.10449A>C	Verified	TCC	AGA	AGT	3	A	C
TCGA-61-2113	FLG	ENST00000368799	p.S522S	c.1566A>C	Verified	GGG	TCA	AGC	3	A	C
TCGA-13-1507	FLG2	ENST00000388718	p.Q1859P	c.5576A>C	Verified	TCT	CAA	TCT	2	A	C
TCGA-13-1510	FLVCR1	ENST00000366971	p.D266A	c.797A>C	Unverified	AAT	GAC	ACA	2	A	C
TCGA-13-0903	FOS	ENST00000303562	p.N245H	c.733A>C	Verified	CTC	AAT	GAC	1	A	C
TCGA-23-1110	FOXA2	ENST00000377115	p.N38H	c.112A>C	Verified	atg	aac	ggc	1	A	C
TCGA-30-1857	FOXM1	ENST00000342628	p.G194G	c.582A>C	Unverified	GAT	GGA	CTG	3	A	C
TCGA-30-1857	FOXP2	ENST00000408937	p.H584P	c.1751A>C	Unverified	CTG	CAC	AAG	2	A	C
TCGA-23-1117	GABPA	ENST00000354828	p.Q97P	c.290A>C	Verified	gta	cag	gta	2	A	C
TCGA-23-1117	GABPA	ENST00000354828	p.Q97P	c.290A>C	Verified	gta	cag	gta	2	A	C
TCGA-29-1785	GCN1L1	ENST00000300648	p.R2168R	c.6502A>C	Unverified	ATG	AGG	CAA	1	A	C
TCGA-29-1785	GCN1L1	ENST00000300648	p.R2168R	c.6502A>C	Unverified	ATG	AGG	CAA	1	A	C
TCGA-20-0990	GDPD2	ENST00000374382	p.T240P	c.718A>C	Verified	AAC	ACC	CTG	1	A	C
TCGA-20-1685	GLB1L2	ENST00000339772	p.R482R	c.1444A>C	Unverified	CTG	AGG	ATC	1	A	C
TCGA-13-1488	GLYATL1	ENST00000300079	p.R47R	c.139A>C	Verified	CTG	AGG	ATA	1	A	C

TCGA-25-2391	GNAT1	ENST00000232461	p.K47Q	c.139A>C	Unverified	gtc	aag	cag	1	A	C
TCGA-04-1337	GNPAT	ENST00000366647	p.R270S	c.810A>C	Verified	AAA	AGA	GAA	3	A	C
TCGA-09-2049	GOLGB1	ENST00000340645	p.Q1074P	c.3221A>C	Verified	CTA	CAG	CAT	2	A	C
TCGA-29-1761	GOLGB1	ENST00000340645	p.E2230A	c.6689A>C	Unverified	AAA	GAA	GAT	2	A	C
TCGA-30-1718	GPR174	ENST00000276077	p.T329T	c.987A>C	Unverified	atg	aca	cct	3	A	C
TCGA-30-1856	GPR32	ENST00000270590	p.T332P	c.994A>C	Unverified	TTG	ACT	TCT	1	A	C
TCGA-61-2109	GPRIN3	ENST00000333209	p.K310Q	c.928A>C	Unverified	ATC	AAG	GAA	1	A	C
TCGA-20-0990	GPX6	ENST00000361902	p.Q26P	c.77A>C	Verified	CCT	CAA	AAT	2	A	C
TCGA-29-1775	GRIA2	ENST00000296526	p.S154R	c.460A>C	Unverified	GAC	AGT	GAC	1	A	C
TCGA-29-1775	GRIA2_ENST	ENST00000264426	p.S154R	c.460A>C	Unverified	GAC	AGT	GAC	1	A	C
TCGA-04-1651	GRM7	ENST00000389335	p.R744R	c.2230A>C	Unverified	GCC	AGA	GGG	1	A	C
TCGA-04-1651	GRM7	ENST00000389335	p.R744R	c.2230A>C	Unverified	GCC	AGA	GGG	1	A	C
TCGA-09-0369	HENMT1	ENST00000370032	p.N105H	c.313A>C	Verified	CTG	AAT	TTG	1	A	C
TCGA-09-0369	HENMT1	ENST00000370032	p.N105H	c.313A>C	Verified	CTG	AAT	TTG	1	A	C
TCGA-13-0755	HERC1	ENST00000443617	p.A2669A	c.8007A>C	Verified	CCT	GCA	TCC	3	A	C
TCGA-24-1417	HIVEP1	ENST00000379388	p.A2345A	c.7035A>C	Unverified	ACA	GCA	GCG	3	A	C
TCGA-29-1775	HLA-E	ENST00000376630	p.D217A	c.650A>C	Unverified	TCT	GAC	CAT	2	A	C
TCGA-29-2427	HLA-G	ENST00000360323	p.Q300P	c.899A>C	Verified	AAG	CAG	TCT	2	A	C
TCGA-04-1356	HMCN1	ENST00000271588	p.T192T	c.576A>C	Verified	TCT	ACA	AGT	3	A	C
TCGA-13-1496	HOXC6	ENST00000243108	p.I147L	c.439A>C	Verified	CAG	ATC	TAC	1	A	C
TCGA-13-1512	HOXC8	ENST00000040584	p.K241Q	c.721A>C	Verified	AAC	AAG	GAC	1	A	C
TCGA-24-2035	HS3ST3A1	ENST00000284110	p.T236P	c.706A>C	Verified	GAC	ACC	AAG	1	A	C
TCGA-20-0991	IDE	ENST00000265986	p.D698A	c.2093A>C	Verified	AAA	GAT	GAG	2	A	C
TCGA-04-1530	IDI2	ENST00000277517	p.Q113P	c.338A>C	Unverified	CTG	CAA	GCA	2	A	C
TCGA-23-1122	IFT140	ENST00000426508	p.A363A	c.1089A>C	Verified	GGG	GCA	GAG	3	A	C
TCGA-23-1117	IL1RAPL1	ENST00000378993	p.E312A	c.935A>C	Verified	GGG	GAA	CAG	2	A	C
TCGA-23-1117	IL1RAPL1	ENST00000378993	p.E312A	c.935A>C	Verified	GGG	GAA	CAG	2	A	C
TCGA-29-1698	IL1RAPL1	ENST00000378993	p.H350P	c.1049A>C	Unverified	CTT	CAT	AAA	2	A	C
TCGA-29-1698	IL1RAPL1_EI	ENST00000378993	p.H350P	c.1049A>C	Unverified	CTT	CAT	AAA	2	A	C
TCGA-23-1022	INA	ENST00000369849	p.S433R	c.1297A>C	Verified	CTC	AGT	GCT	1	A	C
TCGA-04-1342	IRGQ	ENST00000422989	p.T286P	c.856A>C	Unverified	GCC	ACC	ACC	1	A	C
TCGA-23-1118	ITGAX	ENST00000268296	p.K535Q	c.1603A>C	Verified	gac	aag	ctg	1	A	C
TCGA-13-1498	ITGB2	ENST00000397852	p.I60L	c.178A>C	Verified	TCC	ATT	CGC	1	A	C
TCGA-23-1022	ITK	ENST00000422843	p.D202A	c.605A>C	Verified	CTG	GAC	AGT	2	A	C

TCGA-13-1488	ITPR1	ENST00000456211	p.R728R	c.2182A>C	Verified	GCG	AGG	ATG	1	A	C
TCGA-04-1361	ITPR2	ENST00000381340	p.T2334P	c.7000A>C	Verified	TTC	ACC	CGT	1	A	C
TCGA-04-1361	ITPR2	ENST00000381340	p.T2334P	c.7000A>C	Verified	TTC	ACC	CGT	1	A	C
TCGA-04-1362	IVL	ENST00000368764	p.E102A	c.305A>C	Verified	GCA	GAA	AAC	2	A	C
TCGA-09-2049	KCMF1	ENST00000409785	p.A314A	c.942A>C	Verified	CGT	GCA	GAC	3	A	C
TCGA-23-1111	KCND2	ENST00000331113	p.P378P	c.1134A>C	Unverified	GTG	CCA	AAA	3	A	C
TCGA-61-1913	KDELC2	ENST00000323468	p.K500Q	c.1498A>C	Unverified	AGG	AAA	AAG	1	A	C
TCGA-24-1417	KDM2A	ENST00000529006	p.I794L	c.2380A>C	Unverified	aag	atc	cgg	1	A	C
TCGA-61-2009	KIAA1324L	ENST00000297222	p.T124T	c.372A>C	Verified	AAG	ACA	CAG	3	A	C
TCGA-61-1725	KIAA1407	ENST00000295878	p.Q820P	c.2459A>C	Unverified	CTA	CAG	TAC	2	A	C
TCGA-04-1655	KIF27	ENST00000297814	p.A338A	c.1014A>C	Unverified	AGA	GCA	CGG	3	A	C
TCGA-24-1470	KIF3C	ENST00000264712	p.N210H	c.628A>C	Verified	ATG	AAT	GAG	1	A	C
TCGA-09-2049	KIFC2	ENST00000301332	p.P418P	c.1254A>C	Verified	AGG	CCA	GGG	3	A	C
TCGA-23-1022	KLHL34	ENST00000379499	p.K580Q	c.1738A>C	Verified	CTC	AAG	TGG	1	A	C
TCGA-61-1740	KPNA5	ENST00000368564	p.P351P	c.1053A>C	Unverified	agc	cca	aag	3	A	C
TCGA-29-1766	KRTAP10-10	ENST00000380095	p.D83A	c.248A>C	Unverified	CCG	GAT	TGC	2	A	C
TCGA-04-1336	KRTAP13-3	ENST00000390690	p.M87L	c.259A>C	Verified	CAC	ATG	CTC	1	A	C
TCGA-09-2056	L3MBTL2	ENST00000216237	p.Q625P	c.1874A>C	Unverified	AAA	CAG	TTT	2	A	C
TCGA-29-1784	LCTL	ENST00000341509	p.K129Q	c.385A>C	Unverified	AAC	AAG	AAG	1	A	C
TCGA-25-2392	LIMCH1	ENST00000313860	p.N1066H	c.3196A>C	Verified	CTG	AAC	TGT	1	A	C
TCGA-29-1781	LMBRD2	ENST00000296603	p.G681G	c.2043A>C	Unverified	GGT	GGA	CGA	3	A	C
TCGA-25-2401	LPAR6	ENST00000345941	p.A149A	c.447A>C	Verified	AGT	GCA	CCC	3	A	C
TCGA-61-2102	LRBA	ENST00000357115	p.H2304P	c.6911A>C	Verified	ACT	CAT	TAC	2	A	C
TCGA-61-1906	LRCH1	ENST00000389798	p.H282P	c.845A>C	Unverified	GTT	CAC	ATA	2	A	C
TCGA-61-1906	LRCH1	ENST00000389798	p.H282P	c.845A>C	Unverified	GTT	CAC	ATA	2	A	C
TCGA-61-1906	LRCH1_ENST	ENST00000311191	p.H282P	c.845A>C	Unverified	GTT	CAC	ATA	2	A	C
TCGA-61-1906	LRCH1_ENST	ENST00000311191	p.H282P	c.845A>C	Unverified	GTT	CAC	ATA	2	A	C
TCGA-29-1699	LRRK2	SU_LRRK2	p.E1150A	c.3449A>C	Unverified	ctt	gag	gct	2	A	C
TCGA-29-1699	LRRK2_ENST	ENST00000298910	p.E1150A	c.3449A>C	Unverified	CTT	GAG	GCT	2	A	C
TCGA-13-0900	LRRN3	ENST00000308478	p.N150H	c.448A>C	Verified	CAC	AAC	TTG	1	A	C
TCGA-61-1895	LUZP1	ENST00000374623	p.S472S	c.1416A>C	Unverified	CCC	TCA	GTG	3	A	C
TCGA-29-1691	LYAR	ENST00000343470	p.A8A	c.24A>C	Unverified	AAT	GCA	TGT	3	A	C
TCGA-09-2050	MAGI2	ENST00000354212	p.T137P	c.409A>C	Verified	CGC	ACG	GTG	1	A	C
TCGA-29-1769	MAP2	ENST00000360351	p.R599S	c.1797A>C	Unverified	ACT	AGA	GAA	3	A	C

TCGA-23-1116	MARS2	ENST00000282276	p.D357A	c.1070A>C	Unverified	GTG	GAT	CCT	2	A	C
TCGA-61-2009	MAST3	SU_MAST3	p.E352A	c.1055A>C	Unverified	ctg	gag	gag	2	A	C
TCGA-13-1509	MBD5	ENST00000407073	p.G1302G	c.3906A>C	Verified	TTT	GGA	GAG	3	A	C
TCGA-24-2267	MEFV	ENST00000219596	p.H739P	c.2216A>C	Unverified	TCC	CAC	ATC	2	A	C
TCGA-61-1915	MEI1	ENST00000300398	p.T864T	c.2592A>C	Unverified	GAC	ACA	GCT	3	A	C
TCGA-13-0906	MGA	XM_031689.7	p.Q2884P	c.8651A>C	Unverified	aat	caa	caa	2	A	C
TCGA-29-1768	MGC42105	ENST00000326035	p.R120R	c.360A>C	Unverified	TCC	CGA	GAA	3	A	C
TCGA-24-1844	MMP1	ENST00000315274	p.Q186P	c.557A>C	Unverified	TTT	CAA	CCA	2	A	C
TCGA-61-1914	MRO	ENST00000256425	p.I131L	c.391A>C	Unverified	TTC	ATA	GAT	1	A	C
TCGA-61-1914	MRO_ENST(	ENST00000436348	p.I145L	c.433A>C	Unverified	TTC	ATA	GAT	1	A	C
TCGA-29-1775	MTAC2D1	ENST00000360594	p.S318S	c.954A>C	Unverified	TCA	TCA	ACA	3	A	C
TCGA-13-0884	MXRA5	ENST00000381114	p.H87P	c.260A>C	Verified	ATT	CAC	GGC	2	A	C
TCGA-13-1408	MYBPC3	ENST00000399249	p.K812Q	c.2434A>C	Unverified	AAG	AAG	AAG	1	A	C
TCGA-24-2280	MYBPHL	ENST00000357155	p.Q124P	c.371A>C	Unverified	TAC	CAA	CTC	2	A	C
TCGA-24-1470	MYCBP2	NM_015057.1	p.R1641S	c.4923A>C	Verified	agg	aga	gaa	3	A	C
TCGA-29-1766	MYH3	ENST00000226209	p.R1177R	c.3529A>C	Unverified	CGC	AGG	GAC	1	A	C
TCGA-61-2012	MYO15A	ENST00000205890	p.T3122T	c.9366A>C	Verified	ATC	ACA	GAC	3	A	C
TCGA-29-1693	MYO5B	ENST00000285039	p.E1206A	c.3617A>C	Unverified	CTG	GAG	TCA	2	A	C
TCGA-13-1497	NAP1L1	ENST00000393263	p.K276Q	c.826A>C	Verified	AAG	AAG	CAG	1	A	C
TCGA-61-1904	NARF	NM_031968.1	p.H413P	c.1238A>C	Unverified	ttc	cac	ttt	2	A	C
TCGA-24-1850	NBAS	ENST00000281513	p.P769P	c.2307A>C	Unverified	TCT	CCA	CAT	3	A	C
TCGA-61-1904	NCKAP5L_EI	ENST00000335999	p.T29P	c.85A>C	Unverified	GGC	ACC	TGC	1	A	C
TCGA-04-1331	NCOA7	ENST00000368357	p.T705T	c.2115A>C	Verified	TAC	ACA	TTC	3	A	C
TCGA-13-1499	NEB	ENST00000409198	p.H2207P	c.6620A>C	Verified	CAG	CAC	CCG	2	A	C
TCGA-30-1855	NEDD4	ENST00000338963	p.Q92P	c.275A>C	Unverified	CTA	CAG	CCA	2	A	C
TCGA-61-1740	NF1	ENST00000358273	p.T1730P	c.5188A>C	Unverified	GCC	ACC	TTG	1	A	C
TCGA-61-1740	NF1_ENST0(	ENST00000358273	p.T1730P	c.5188A>C	Unverified	GCC	ACC	TTG	1	A	C
TCGA-24-1847	NFS1	ENST00000374092	p.A311A	c.933A>C	Unverified	GTG	GCA	CAG	3	A	C
TCGA-24-2288	NLRP13	ENST00000342929	p.Q115P	c.344A>C	Unverified	CTG	CAA	GAT	2	A	C
TCGA-24-2280	NLRP8	ENST00000291971	p.I124L	c.370A>C	Unverified	GCC	ATT	CTG	1	A	C
TCGA-13-0760	NOC2L	ENST00000327044	p.N565H	c.1693A>C	Verified	GCC	AAC	TAC	1	A	C
TCGA-13-1498	NR5A2	ENST00000367362	p.N426H	c.1276A>C	Verified	aac	aac	ctc	1	A	C
TCGA-30-1856	NRXN2	ENST00000377551	p.Q1108P	c.3323A>C	Unverified	aac	cag	ggc	2	A	C
TCGA-29-2427	NTNG2	ENST00000393229	p.T105P	c.313A>C	Unverified	GCC	ACC	TAC	1	A	C

TCGA-13-1488	NUF2	ENST00000367900	p.S86R	c.256A>C	Verified	TTC	AGC	AAT	1	A	C
TCGA-13-0890	NUP210L	ENST00000368559	p.Q935P	c.2804A>C	Verified	GAG	CAG	GGT	2	A	C
TCGA-20-0991	OR13C8	ENST00000335040	p.T246T	c.738A>C	Verified	CTG	ACA	GTG	3	A	C
TCGA-61-1740	OR1A1	ENST00000304094	p.N84H	c.250A>C	Unverified	GCC	AAC	CAT	1	A	C
TCGA-23-1117	OR51T1	ENST00000380378	p.A276A	c.828A>C	Verified	TGT	GCA	GTC	3	A	C
TCGA-23-1117	OR51T1	ENST00000380378	p.A276A	c.828A>C	Verified	TGT	GCA	GTC	3	A	C
TCGA-13-1481	OSBPL1A	ENST00000319481	p.T285P	c.853A>C	Verified	CTG	ACT	CAA	1	A	C
TCGA-29-1763	OTOGL_ENS	ENST00000458043_v65	p.?	c.1561-2A>C	Unverified	CAG	AAT	CTT	1	A	C
TCGA-23-1124	OTX2	ENST00000339475	p.Q109P	c.326A>C	Verified	cag	cag	aat	2	A	C
TCGA-04-1356	P2RY11	ENST00000321826	p.Q304P	c.911A>C	Verified	TAC	CAG	GTG	2	A	C
TCGA-29-1764	PABPC1	ENST00000318607	p.N394H	c.1180A>C	Unverified	CCC	AAC	CCT	1	A	C
TCGA-25-2392	PABPC1L	ENST00000217073	p.Q292P	c.875A>C	Verified	TAC	CAG	GGT	2	A	C
TCGA-29-1764	PAK1	ENST00000356341	p.A412A	c.1236A>C	Unverified	TGT	GCA	CAG	3	A	C
TCGA-29-1764	PAK1_ENST(	ENST00000278568	p.A412A	c.1236A>C	Unverified	TGT	GCA	CAG	3	A	C
TCGA-20-0991	PANK4	ENST00000378466	p.D174A	c.521A>C	Verified	TCC	GAC	CCT	2	A	C
TCGA-13-0893	PARP2	NM_005484.2	p.D133A	c.398A>C	Verified	ctt	gac	aaa	2	A	C
TCGA-13-0905	PCNT	ENST00000359568	p.H855P	c.2564A>C	Verified	CTC	CAC	GTG	2	A	C
TCGA-24-1103	PDE8A	ENST00000310298	p.E746A	c.2237A>C	Verified	TCG	GAA	GAA	2	A	C
TCGA-04-1361	PFKFB1	ENST00000375006	p.P3P	c.9A>C	Verified	TCT	CCA	GAG	3	A	C
TCGA-04-1361	PFKFB1	ENST00000375006	p.P3P	c.9A>C	Verified	TCT	CCA	GAG	3	A	C
TCGA-04-1347	PHC3	NM_024947.2	p.Q337P	c.1010A>C	Verified	ctc	cag	aac	2	A	C
TCGA-13-0890	PHF21B	ENST00000313237	p.D89A	c.266A>C	Verified	CGG	GAC	CGG	2	A	C
TCGA-30-1855	PIK3R1	ENST00000521381	p.P717P	c.2151A>C	Unverified	tac	cca	gta	3	A	C
TCGA-30-1855	PIK3R1_ENS	ENST00000320694	p.P417P	c.1251A>C	Unverified	TAC	CCA	GTA	3	A	C
TCGA-30-1855	PIK3R1_ENS	ENST00000336483	p.P447P	c.1341A>C	Unverified	TAC	CCA	GTA	3	A	C
TCGA-13-0760	PIK3R4	ENST00000356763	p.E1094A	c.3281A>C	Verified	aag	gag	gac	2	A	C
TCGA-25-2393	PIWIL1	ENST00000245255	p.S660S	c.1980A>C	Verified	TTC	TCA	CGC	3	A	C
TCGA-61-1907	PKHD1	ENST00000371117	p.K1072Q	c.3214A>C	Unverified	TGC	AAA	GTT	1	A	C
TCGA-61-1907	PKHD1_ENS	ENST00000340994	p.K1072Q	c.3214A>C	Unverified	TGC	AAA	GTT	1	A	C
TCGA-13-0793	PKIA	ENST00000352966	p.T17T	c.51A>C	Verified	AGA	ACA	GGT	3	A	C
TCGA-24-0979	PLD5	ENST00000366545	p.A382A	c.1146A>C	Verified	CAG	GCA	GAT	3	A	C
TCGA-09-1674	PMEL	ENST00000552882	p.S36S	c.108A>C	Unverified	GTC	TCA	AGG	3	A	C
TCGA-04-1356	PODN	ENST00000312553	p.N197H	c.589A>C	Unverified	CTC	AAT	TAC	1	A	C
TCGA-04-1356	PPAN-P2RY1	ENST00000393796	p.Q724P	c.2171A>C	Verified	TAC	CAG	GTG	2	A	C

TCGA-24-1843	PPP3CA	ENST00000394854	p.K399Q	c.1195A>C	Unverified	aac	aag	atc	1	A	C
TCGA-24-1845	PRG4	ENST00000445192	p.T401P	c.1201A>C	Unverified	ACC	ACC	AAG	1	A	C
TCGA-13-0887	PRKCE	ENST00000306156	p.Q197P	c.590A>C	Verified	AAG	CAG	GGA	2	A	C
TCGA-13-0792	PRKCZ_ENST	ENST00000378567	p.D251A	c.752A>C	Unverified	CAG	GAC	TTT	2	A	C
TCGA-25-2401	PROC	ENST00000234071	p.Q3P	c.8A>C	Unverified	TGG	CAG	CTC	2	A	C
TCGA-04-1331	PSMA4	ENST00000044462	p.D57A	c.170A>C	Unverified	CTT	GAT	GAA	2	A	C
TCGA-10-0930	PSMC3	ENST00000298852	p.N207H	c.619A>C	Verified	ATG	AAC	CAC	1	A	C
TCGA-10-0930	PSMC3	ENST00000298852	p.N207H	c.619A>C	Verified	ATG	AAC	CAC	1	A	C
TCGA-04-1337	PTPN12	ENST00000248594	p.D26A	c.77A>C	Unverified	GAG	GAC	AAC	2	A	C
TCGA-29-1761	Q8NH32_HU	ENST00000321415	p.I13L	c.37A>C	Unverified	ATC	ATC	CTG	1	A	C
TCGA-29-1763	RAP1GAP	ENST00000374765	p.P29P	c.87A>C	Unverified	ATT	CCA	TAC	3	A	C
TCGA-29-1763	RAP1GAP_E	ENST00000374757	p.P171P	c.513A>C	Unverified	ATT	CCA	TAC	3	A	C
TCGA-29-1763	RAP1GAP_E	ENST00000374761	p.P60P	c.180A>C	Unverified	ATT	CCA	TAC	3	A	C
TCGA-13-0793	RBM12B	ENST00000399300	p.P670P	c.2010A>C	Unverified	CGG	CCA	CCT	3	A	C
TCGA-13-1499	RBM26	ENST00000267229	p.S487S	c.1461A>C	Verified	GAC	TCA	GAA	3	A	C
TCGA-29-1781	RECQL	ENST00000444129	p.P126P	c.378A>C	Unverified	TTA	CCA	GCA	3	A	C
TCGA-29-1775	RIMS1	ENST00000521978	p.Q943P	c.2828A>C	Unverified	GAA	CAG	CAA	2	A	C
TCGA-13-0887	RIOK3	ENST00000339486	p.K456Q	c.1366A>C	Verified	GTC	AAG	GAA	1	A	C
TCGA-61-1907	RIT2	ENST00000326695	p.G30G	c.90A>C	Unverified	ggg	gga	gtt	3	A	C
TCGA-09-2049	RNASE7	ENST00000298690	p.N127H	c.379A>C	Verified	CAG	AAC	AAG	1	A	C
TCGA-23-1031	RPL10	ENST00000369817	p.M184L	c.550A>C	Unverified	GAC	ATG	GTG	1	A	C
TCGA-29-1693	RSPH1	ENST00000291536	p.G87G	c.261A>C	Unverified	gat	gga	tcc	3	A	C
TCGA-20-1683	SCG2	ENST00000305409	p.S555R	c.1663A>C	Unverified	GGC	AGC	TCT	1	A	C
TCGA-09-2051	SEC16A	ENST00000313050	p.G2115G	c.6345A>C	Unverified	CCT	GGA	AAG	3	A	C
TCGA-24-1847	SEC23B	ENST00000377465	p.I744L	c.2230A>C	Unverified	CCC	ATC	CTA	1	A	C
TCGA-61-2012	SERPINB4	ENST00000341074	p.Q52P	c.155A>C	Verified	caa	caa	att	2	A	C
TCGA-25-2392	SH2B1	ENST00000337120	p.E349A	c.1046A>C	Verified	GTG	GAA	GGT	2	A	C
TCGA-29-1696	SLC10A1	ENST00000216540	p.K177Q	c.529A>C	Unverified	CTC	AAA	TCC	1	A	C
TCGA-29-1696	SLC10A1	ENST00000216540	p.K177Q	c.529A>C	Unverified	CTC	AAA	TCC	1	A	C
TCGA-13-0887	SLC12A8_EN	ENST00000393469	p.M33L	c.97A>C	Verified	TTC	ATG	TGG	1	A	C
TCGA-04-1542	SLC5A7	ENST00000264047	p.S297S	c.891A>C	Verified	GCA	TCA	ACA	3	A	C
TCGA-23-1021	SLC9A7	ENST00000328306	p.T312P	c.934A>C	Verified	aac	act	cac	1	A	C
TCGA-25-1313	SLC9A9	ENST00000316549	p.I589L	c.1765A>C	Verified	gcc	ata	aat	1	A	C
TCGA-04-1651	SON	ENST00000356577	p.I8L	c.22A>C	Unverified	CAG	ATT	TTT	1	A	C

TCGA-04-1651	SON	ENST00000356577	p.I8L	c.22A>C	Unverified	CAG	ATT	TTT	1	A	C
TCGA-04-1651	SON_ENST0	ENST00000300278	p.I8L	c.22A>C	Unverified	CAG	ATT	TTT	1	A	C
TCGA-04-1651	SON_ENST0	ENST00000300278	p.I8L	c.22A>C	Unverified	CAG	ATT	TTT	1	A	C
TCGA-24-1104	SOX6	ENST00000316399	p.R93R	c.279A>C	Verified	TTC	CGA	AAT	3	A	C
TCGA-13-0762	SPATA13	ENST00000382095	p.T378P	c.1132A>C	Verified	ctc	aca	cca	1	A	C
TCGA-13-0762	SPATA13	ENST00000382095	p.T378P	c.1132A>C	Verified	ctc	aca	cca	1	A	C
TCGA-24-1422	SPATA2	ENST00000289431	p.N504H	c.1510A>C	Unverified	CCC	AAC	AAC	1	A	C
TCGA-20-0990	SPG11	ENST00000261866	p.K604Q	c.1810A>C	Verified	AGC	AAA	CAC	1	A	C
TCGA-29-1770	SPG11	ENST00000261866	p.Q1400P	c.4199A>C	Unverified	ATT	CAA	GAC	2	A	C
TCGA-30-1714	SPHAR	ENST00000366688	p.N51H	c.151A>C	Unverified	TTC	AAT	GTC	1	A	C
TCGA-13-0760	SPOCK1	ENST00000394945	p.K201Q	c.601A>C	Verified	GAC	AAG	GAG	1	A	C
TCGA-29-1761	SPTBN1_EN	ENST00000333896	p.Q20P	c.59A>C	Unverified	GGG	CAG	GTG	2	A	C
TCGA-13-0906	SRP72	ENST00000342756	p.A392A	c.1176A>C	Verified	AAA	GCA	TGT	3	A	C
TCGA-13-0760	ST14	ENST00000278742	p.H656P	c.1967A>C	Verified	GCA	CAC	TGC	2	A	C
TCGA-29-1781	STARD3	ENST00000336308	p.D48A	c.143A>C	Unverified	TCT	GAT	GTC	2	A	C
TCGA-61-1740	STOX2	ENST00000308497	p.G702G	c.2106A>C	Unverified	GAC	ACA	CTG	3	A	C
TCGA-61-1740	STOX2_ENS	ENST00000308497	p.G678G	c.2034A>C	Unverified	AAC	GGA	CGC	3	A	C
TCGA-20-0991	SUN2	ENST00000405510	p.T192P	c.574A>C	Unverified	CTG	ACC	ACA	1	A	C
TCGA-24-1563	SV2A	ENST00000369146	p.S393S	c.1179A>C	Verified	TTC	TCA	GTA	3	A	C
TCGA-04-1362	SVEP1	ENST00000401783	p.A2939A	c.8817A>C	Verified	GAT	GCA	GAG	3	A	C
TCGA-25-2398	SYNE2	ENST00000358025	p.Q6731P	c.20192A>C	Verified	ATG	CAA	CTG	2	A	C
TCGA-24-1846	TAS2R7	ENST00000240687	p.R164R	c.490A>C	Unverified	AAG	AGG	AAA	1	A	C
TCGA-29-1775	TC2N	ENST00000340892	p.S382S	c.1146A>C	Unverified	TCA	TCA	ACA	3	A	C
TCGA-25-2042	TCF21	ENST00000237316	p.Q80P	c.239A>C	Unverified	GTC	CAG	CGC	2	A	C
TCGA-25-2042	TGM2	ENST00000361475	p.Q69P	c.206A>C	Unverified	AGC	CAG	GAG	2	A	C
TCGA-61-1913	TLL2	ENST00000357947	p.H833P	c.2498A>C	Unverified	GAC	CAC	CTG	2	A	C
TCGA-09-1674	TMEM182	ENST00000412401	p.A109A	c.327A>C	Unverified	TCT	GCA	GTT	3	A	C
TCGA-04-1338	TMEM33	ENST00000504986	p.Q234P	c.701A>C	Verified	CTC	CAG	AGC	2	A	C
TCGA-24-2267	TNFAIP8	ENST00000504771	p.E100A	c.299A>C	Verified	ATG	GAG	AAA	2	A	C
TCGA-23-1124	TNS3	ENST00000311160	p.K284Q	c.850A>C	Verified	AGC	AAA	GAT	1	A	C
TCGA-13-0905	TRAF3IP2	ENST00000368761	p.D205A	c.614A>C	Verified	CAG	GAT	GTC	2	A	C
TCGA-61-2095	TREH	ENST00000264029	p.N334H	c.1000A>C	Unverified	CCC	AAC	TCG	1	A	C
TCGA-13-1512	TRIM26	ENST00000453195	p.K225Q	c.673A>C	Verified	TTC	AAG	AGC	1	A	C
TCGA-29-1691	TRIM55	ENST00000315962	p.H90P	c.269A>C	Unverified	aga	cat	ggg	2	A	C

TCGA-24-1846	TRIOBP	ENST00000407319	p.T422P	c.1264A>C	Unverified	GTC	ACC	CCG	1	A	C
TCGA-13-0923	TRIP11	ENST00000267622	p.E539A	c.1616A>C	Verified	GAT	GAA	AAA	2	A	C
TCGA-23-2078	TRPC4	ENST00000379705	p.D841A	c.2522A>C	Unverified	ACC	GAT	ATC	2	A	C
TCGA-20-1683	TRRAP	ENST00000355540	p.R210R	c.630A>C	Unverified	ACT	CGA	ACA	3	A	C
TCGA-20-1683	TRRAP_ENST	ENST00000359863	p.R210R	c.630A>C	Unverified	ACT	CGA	ACA	3	A	C
TCGA-10-0930	TSGA13	ENST00000356588	p.S14S	c.42A>C	Verified	AAA	TCA	AAG	3	A	C
TCGA-10-0930	TSGA13	ENST00000356588	p.S14S	c.42A>C	Verified	AAA	TCA	AAG	3	A	C
TCGA-09-2051	TTC15	ENST00000382110	p.T418P	c.1252A>C	Unverified	CTC	ACC	AGC	1	A	C
TCGA-61-1907	TTC40	ENST00000368586	p.T2005P	c.6013A>C	Unverified	GCC	ACA	AAG	1	A	C
TCGA-23-1122	TTN	NM_003319	p.H6291P	c.18872A>C	Verified	aag	cac	atg	2	A	C
TCGA-24-1469	TTN	NM_003319	p.G19553G	c.58659A>C	Verified	gaa	gga	tgt	3	A	C
TCGA-23-1122	TTN_ENST0(	ENST00000356127	p.H12788P	c.38363A>C	Verified	AAG	CAC	ATG	2	A	C
TCGA-24-1469	TTN_ENST0(	ENST00000356127	p.G26048G	c.78144A>C	Verified	GAA	GGA	TGT	3	A	C
TCGA-13-1507	TUBGCP4	ENST00000564079	p.K402Q	c.1204A>C	Verified	CAC	AAG	GTA	1	A	C
TCGA-24-1435	TYR	ENST00000263321	p.D317A	c.950A>C	Verified	GCT	GAT	GTA	2	A	C
TCGA-30-1714	UBE2O	ENST00000319380	p.H648P	c.1943A>C	Unverified	GAC	CAC	CCT	2	A	C
TCGA-30-1714	UBE2O_ENS	ENST00000319380	p.H648P	c.1943A>C	Unverified	GAC	CAC	CCT	2	A	C
TCGA-13-1481	UBR4	ENST00000375254	p.E2169A	c.6506A>C	Verified	TCT	GAG	GTG	2	A	C
TCGA-24-2280	UGT1A5	ENST00000373414	p.Q86P	c.257A>C	Unverified	ACC	CAG	GAC	2	A	C
TCGA-13-0885	UNC13C	ENST00000260323	p.P722P	c.2166A>C	Verified	TCT	CCA	TGC	3	A	C
TCGA-61-1907	UROS	ENST00000368786	p.K7Q	c.19A>C	Unverified	CTG	AAG	GAT	1	A	C
TCGA-13-1496	USP15	NM_006313.1	p.D653A	c.1958A>C	Verified	gaa	gat	tca	2	A	C
TCGA-29-1775	USP37	ENST00000258399	p.Q817P	c.2450A>C	Unverified	GCT	CAG	AGC	2	A	C
TCGA-61-1998	VAV2	ENST00000406606	p.N369H	c.1105A>C	Unverified	ATC	AAT	GAA	1	A	C
TCGA-36-1577	VLDLR	ENST00000382100	p.D145A	c.434A>C	Verified	GAA	GAT	GAA	2	A	C
TCGA-24-1435	VWA3B	ENST00000477737	p.A446A	c.1338A>C	Verified	CAT	GCA	AAA	3	A	C
TCGA-29-1775	WDR26	ENST00000295024	p.A115A	c.345A>C	Unverified	CAT	GCA	GAA	3	A	C
TCGA-29-1775	WDR26_ENS	ENST00000414423	p.A262A	c.786A>C	Unverified	CAT	GCA	GAA	3	A	C
TCGA-61-2008	WDR27_ENS	ENST00000333572	p.?	c.1130-2A>C	Verified	AAG	GAT	TTC	2	A	C
TCGA-61-2008	WDR27_ENS	ENST00000333572	p.?	c.1130-2A>C	Verified	AAG	GAT	TTC	2	A	C
TCGA-61-2008	WDR27_ENS	ENST00000448612	p.?	c.1130-2A>C	Verified	AAG	GAT	TTC	2	A	C
TCGA-25-2398	WDR55	ENST00000358337	p.T272P	c.814A>C	Verified	TCC	ACT	GAT	1	A	C
TCGA-20-0991	WDR55	ENST00000358337	p.D2A	c.5A>C	Unverified	ATG	GAC	CGC	2	A	C
TCGA-09-0369	WHSC1	ENST00000382891	p.R1334R	c.4000A>C	Verified	GTC	AGA	AGC	1	A	C

TCGA-09-0369	WHSC1	ENST00000382891	p.R1334R	c.4000A>C	Verified	GTC	AGA	AGC	1	A	C
TCGA-61-1907	WWC1	ENST00000265293	p.Q186P	c.557A>C	Unverified	CTG	CAG	TTC	2	A	C
TCGA-29-1691	XPNPEP1	ENST00000369680	p.D594A	c.1781A>C	Unverified	AGG	GAT	GTG	2	A	C
TCGA-29-1691	XPNPEP1_EI	ENST00000502935	p.D637A	c.1910A>C	Unverified	AGG	GAT	GTG	2	A	C
TCGA-29-1693	YEATS2	ENST00000305135	p.Q505P	c.1514A>C	Unverified	AAG	CAG	CCG	2	A	C
TCGA-23-1110	ZCCHC4	ENST00000302874	p.K167Q	c.499A>C	Verified	AAC	AAG	AAG	1	A	C
TCGA-13-0884	ZFHX4	ENST00000521891	p.N2629H	c.7885A>C	Verified	TCC	AAT	CCT	1	A	C
TCGA-24-1847	ZFPM2	ENST00000407775	p.R311R	c.933A>C	Unverified	GCT	CGA	GCT	3	A	C
TCGA-25-2392	ZMYND12	ENST00000372565	p.I49L	c.145A>C	Verified	AGC	ATC	CAT	1	A	C
TCGA-61-1737	ZNF18	ENST00000322748	p.S172S	c.516A>C	Unverified	TCC	TCA	GGG	3	A	C
TCGA-09-1665	ZNF211	ENST00000240731	p.I314L	c.940A>C	Verified	TTC	ATT	ATA	1	A	C
TCGA-24-1846	ZNF22	ENST00000298299	p.E171A	c.512A>C	Unverified	AGT	GAA	TGT	2	A	C
TCGA-23-1114	ZNF224	ENST00000336976	p.I270L	c.808A>C	Unverified	TTC	ATT	CAC	1	A	C
TCGA-20-0990	ZNF236	ENST00000253159	p.T87P	c.259A>C	Verified	TGC	ACC	CAC	1	A	C
TCGA-04-1367	ZNF503	ENST00000372524	p.G624G	c.1872A>C	Verified	ACC	GGA	CCG	3	A	C
TCGA-30-1855	ZNF625	ENST00000355738	p.Q241P	c.722A>C	Unverified	AAG	CAA	TGT	2	A	C
TCGA-23-1022	ZNF91_ENST	ENST00000300619	p.E942A	c.2825A>C	Verified	GAA	GAA	TGT	2	A	C
TCGA-24-1474	ZXDA	ENST00000358697	p.H320P	c.959A>C	Verified	agg	cac	ctg	2	A	C
TCGA-04-1338	A2M	ENST00000318602	p.Q1051H	c.3153A>C	Unverified	GCC	CAA	GCT	3	A	C
TCGA-61-1906	ABCA10	ENST00000269081	p.K1113T	c.3338A>C	Unverified	AAT	AAA	ACC	2	A	C
TCGA-61-1906	ABCA10	ENST00000269081	p.K1113T	c.3338A>C	Unverified	AAT	AAA	ACC	2	A	C
TCGA-23-1122	ABCA6	ENST00000284425	p.Y271S	c.812A>C	Unverified	ATC	TAT	GCT	2	A	C
TCGA-61-1740	ADAMTS6_E	ENST00000381055	p.Y662S	c.1985A>C	Unverified	TTC	TAC	ACT	2	A	C
TCGA-04-1336	ADAMTSL1	NM_139238.1	p.K193T	c.578A>C	Verified	tat	aaa	tcc	2	A	C
TCGA-04-1336	ADAMTSL1_	ENST00000380548	p.K193T	c.578A>C	Verified	TAT	AAA	TCC	2	A	C
TCGA-24-1845	AHR	ENST00000242057	p.K37T	c.110A>C	Unverified	tcc	aag	cgg	2	A	C
TCGA-23-1116	ALS2CR12	ENST00000286190	p.Q133H	c.399A>C	Verified	GAG	CAA	ATC	3	A	C
TCGA-61-1904	ANGEL1	ENST00000251089	p.L358L	c.1074A>C	Unverified	GAG	CTA	CTT	3	A	C
TCGA-24-1845	ANKRD30A	ENST00000361713	p.Q845H	c.2535A>C	Unverified	GAA	CAA	ACA	3	A	C
TCGA-25-2401	ANKS1A	ENST00000360359	p.Q264H	c.792A>C	Verified	GTG	CAA	ATC	3	A	C
TCGA-09-0369	ANO2	ENST00000327087	p.K202N	c.606A>C	Verified	ATC	AAA	GTT	3	A	C
TCGA-09-0369	ANO2	ENST00000327087	p.K202N	c.606A>C	Verified	ATC	AAA	GTT	3	A	C
TCGA-61-1740	ANUBL1	ENST00000344646	p.S678R	c.2032A>C	Unverified	GCT	AGT	AGC	1	A	C
TCGA-13-0755	ANXA2	ENST00000332680	p.T141P	c.421A>C	Verified	GGA	ACC	GAC	1	A	C

TCGA-13-0923	API5	ENST00000378852	p.Q81H	c.243A>C	Verified	CGT	CAA	GCA	3	A	C
TCGA-13-1509	ARHGAP31	ENST00000264245	p.N776T	c.2327A>C	Verified	GGC	AAT	CTG	2	A	C
TCGA-13-0890	ARHGEF10_	ENST00000349830	p.E164D	c.492A>C	Verified	CCA	GAA	GTC	3	A	C
TCGA-13-0920	ARHGEF19	ENST00000270747	p.K788T	c.2363A>C	Verified	aat	aag	cga	2	A	C
TCGA-24-1604	ARHGEF38	ENST00000265154	p.K4N	c.12A>C	Verified	CCC	AAA	GAA	3	A	C
TCGA-24-1470	ARID2	ENST00000334344	p.K466T	c.1397A>C	Verified	GTA	AAA	CTC	2	A	C
TCGA-61-1915	ARL3_ENST(	ENST00000260746	p.I20I	c.60A>C	Unverified	AGA	ATA	CTT	3	A	C
TCGA-25-2393	ARL6IP5	ENST00000273258	p.K151N	c.453A>C	Verified	AAT	AAA	ATG	3	A	C
TCGA-61-1904	ARSF	ENST00000381127	p.N314H	c.940A>C	Unverified	gat	aat	gtg	1	A	C
TCGA-09-2050	ASB18	NM_212556.1	p.N57T	c.170A>C	Verified	gcc	aac	gtg	2	A	C
TCGA-24-1469	ASPH	ENST00000379454	p.T252P	c.754A>C	Verified	GAT	ACA	GAT	1	A	C
TCGA-13-1497	ASS1	ENST00000372393	p.K412T	c.1235A>C	Verified	GCC	AAA	TAG	2	A	C
TCGA-61-2102	ATP10D	ENST00000273859	p.N304T	c.911A>C	Verified	AAC	AAC	AGT	2	A	C
TCGA-23-1120	AXIN2	ENST00000307078	p.K123T	c.368A>C	Unverified	CTG	AAG	GAT	2	A	C
TCGA-61-2109	BACH2	ENST00000257749	p.Y528S	c.1583A>C	Unverified	TCC	TAC	GCG	2	A	C
TCGA-29-1784	BFSP1	ENST00000377873	p.E182D	c.546A>C	Unverified	CTG	GAA	GTT	3	A	C
TCGA-13-0900	BICC1	ENST00000373886	p.K242T	c.725A>C	Verified	TTT	AAA	CAG	2	A	C
TCGA-61-2109	BIRC6	ENST00000421745	p.L1631L	c.4893A>C	Unverified	CAG	CTA	CAG	3	A	C
TCGA-24-1604	BMP2	ENST00000378827	p.I145L	c.433A>C	Verified	ttt	atc	acc	1	A	C
TCGA-25-2042	BMX	ENST00000357607	p.K313Q	c.937A>C	Verified	caa	aag	gga	1	A	C
TCGA-09-2050	BST2	ENST00000252593	p.Q110H	c.330A>C	Verified	GGA	CAA	AAG	3	A	C
TCGA-24-2289	C10orf71_EI	ENST00000374144	p.S275R	c.823A>C	Unverified	AAT	AGT	GCT	1	A	C
TCGA-24-0975	C10orf96	ENST00000333254	p.K110Q	c.328A>C	Verified	ATT	AAG	GAA	1	A	C
TCGA-61-1913	C11orf74	ENST00000334307	p.L16F	c.48A>C	Unverified	CAA	TTA	ATC	3	A	C
TCGA-04-1542	C17orf42	ENST00000581216	p.I171I	c.513A>C	Verified	ATC	ATA	TCT	3	A	C
TCGA-25-2042	C1orf88	ENST00000369738	p.K124T	c.371A>C	Verified	CCA	AAG	GAC	2	A	C
TCGA-23-1021	C20orf133	ENST00000217246	p.Y232S	c.695A>C	Verified	ATC	TAC	AAA	2	A	C
TCGA-13-1488	C6	ENST00000263413	p.T576P	c.1726A>C	Verified	AGT	ACC	TGT	1	A	C
TCGA-04-1342	C9orf129	ENST00000375419	p.T124P	c.370A>C	Unverified	TAT	ACA	ATC	1	A	C
TCGA-61-2113	C9orf78	ENST00000372447	p.K6T	c.17A>C	Unverified	CGG	AAG	ATT	2	A	C
TCGA-04-1331	CA12	ENST00000178638	p.N150T	c.449A>C	Verified	TAT	AAC	TCA	2	A	C
TCGA-23-1110	CACNA1S	ENST00000362061	p.K162T	c.485A>C	Verified	GTC	AAG	GCC	2	A	C
TCGA-24-2280	CADPS	ENST00000283269	p.Q877H	c.2631A>C	Unverified	TCC	CAA	GTG	3	A	C
TCGA-25-1326	CAPN10	ENST00000354082	p.K66T	c.197A>C	Verified	GTG	AAG	CAG	2	A	C

TCGA-23-1022	CAPS2	ENST00000328705	p.K296N	c.888A>C	Verified	ATG	AAA	CTG	3	A	C
TCGA-24-2267	CC2D2B	ENST00000344386	p.E294D	c.882A>C	Verified	ACA	GAA	TTT	3	A	C
TCGA-24-1435	CCDC146	ENST00000285871	p.Q471H	c.1413A>C	Verified	GAA	CAA	AAG	3	A	C
TCGA-13-0906	CCDC57	ENST00000445854	p.K212N	c.636A>C	Verified	CAC	AAA	GAG	3	A	C
TCGA-23-1117	CDC6	ENST00000209728	p.L112L	c.336A>C	Verified	gaa	cta	gcc	3	A	C
TCGA-23-1117	CDC6	ENST00000209728	p.L112L	c.336A>C	Verified	gaa	cta	gcc	3	A	C
TCGA-13-0900	CDH5	ENST00000341529	p.N79T	c.236A>C	Verified	AAG	AAT	GCC	2	A	C
TCGA-09-2049	CES4A	ENST00000398354	p.Y84S	c.251A>C	Verified	ACC	TAC	CCG	2	A	C
TCGA-09-0369	CHEK1	ENST00000438015	p.N63H	c.187A>C	Unverified	CTA	AAT	CAT	1	A	C
TCGA-09-0369	CHEK1	ENST00000438015	p.N63H	c.187A>C	Unverified	CTA	AAT	CAT	1	A	C
TCGA-23-1120	CHMP4A	ENST00000347519	p.Y160S	c.479A>C	Verified	GCC	TAC	CAG	2	A	C
TCGA-13-1509	CNTNAP2	ENST00000361727	p.N391H	c.1171A>C	Verified	CTT	AAC	CAG	1	A	C
TCGA-23-1124	CPB2	ENST00000181383	p.K414Q	c.1240A>C	Verified	TCT	AAA	ATA	1	A	C
TCGA-04-1638	CRISP2	ENST00000339139	p.S113R	c.337A>C	Unverified	CAA	AGC	TGG	1	A	C
TCGA-23-1110	CUBN	ENST00000377833	p.K510T	c.1529A>C	Verified	GGA	AAG	GTC	2	A	C
TCGA-61-2009	DCT	ENST00000377028	p.L145F	c.435A>C	Verified	GCC	TTA	GAT	3	A	C
TCGA-13-0760	DDX3X	ENST00000399959	p.N551H	c.1651A>C	Verified	ATA	AAT	ATT	1	A	C
TCGA-25-1326	DDX4	ENST00000505374	p.N11T	c.32A>C	Verified	ATC	AAC	CCT	2	A	C
TCGA-24-2280	DGKA	ENST00000331886	p.N601T	c.1802A>C	Unverified	cta	aac	atc	2	A	C
TCGA-13-0885	DHX34	ENST00000328771	p.K176T	c.527A>C	Verified	ctg	aag	gag	2	A	C
TCGA-24-2289	DIP2A	ENST00000417564	p.K1134N	c.3402A>C	Unverified	CCA	AAA	AAG	3	A	C
TCGA-04-1356	DNAH17	ENST00000300671	p.N1174H	c.3520A>C	Verified	GCA	AAT	ACC	1	A	C
TCGA-10-0930	DNAH3	ENST00000261383	p.E972D	c.2916A>C	Verified	ACC	GAA	ACG	3	A	C
TCGA-10-0930	DNAH3	ENST00000261383	p.E972D	c.2916A>C	Verified	ACC	GAA	ACG	3	A	C
TCGA-20-1687	DST	ENST00000370765	p.Y2366S	c.7097A>C	Unverified	ATA	TAT	CTT	2	A	C
TCGA-20-1687	DST_ENST0(	ENST00000370765	p.Y2366S	c.7097A>C	Unverified	ATA	TAT	CTT	2	A	C
TCGA-13-1510	EDARADD	ENST00000334232	p.K128Q	c.382A>C	Unverified	ATA	AAG	CTG	1	A	C
TCGA-13-1488	EMR3	ENST00000253673	p.N111T	c.332A>C	Verified	AGT	AAT	TCC	2	A	C
TCGA-13-1489	ENAH	ENST00000366844	p.R402R	c.1204A>C	Verified	CTT	AGG	AAA	1	A	C
TCGA-61-1738	ENSG00002	ENST00000400226	p.K530N	c.1590A>C	Unverified	CAG	AAA	ATC	3	A	C
TCGA-61-1738	ENSG00002	ENST00000400226	p.K530N	c.1590A>C	Unverified	CAG	AAA	ATC	3	A	C
TCGA-29-2427	ENSG00002	ENST00000528157	p.K170Q	c.508A>C	Unverified	CCT	AAA	TAT	1	A	C
TCGA-61-1910	EPC1	ENST00000263062	p.N205H	c.613A>C	Unverified	ACA	AAT	GAT	1	A	C
TCGA-61-1904	EPHA4	ENST00000281821	p.Y246S	c.737A>C	Unverified	ATG	TAC	TGT	2	A	C

TCGA-61-1904	EPHA4_ENST	ENST00000281821	p.Y246S	c.737A>C	Unverified	ATG	TAC	TGT	2	A	C
TCGA-61-1725	EPS8	ENST00000281172	p.N395H	c.1183A>C	Unverified	TTA	AAT	TAT	1	A	C
TCGA-24-1844	ERC2	ENST00000288221	p.R660R	c.1978A>C	Unverified	AAA	AGG	GAT	1	A	C
TCGA-24-1844	ERC2_ENST(	ENST00000288221	p.R660R	c.1978A>C	Unverified	AAA	AGG	GAT	1	A	C
TCGA-61-2102	ERGIC2	ENST00000360150	p.R133R	c.397A>C	Verified	AGT	AGG	CTA	1	A	C
TCGA-24-1850	ETFDH	ENST00000511912	p.Y310S	c.929A>C	Unverified	CTC	TAT	CAT	2	A	C
TCGA-24-1563	ETV5	ENST00000306376	p.N77H	c.229A>C	Verified	GAT	AAC	CTG	1	A	C
TCGA-23-1029	FANCM	ENST00000267430	p.N1186T	c.3557A>C	Unverified	GAC	AAT	AAT	2	A	C
TCGA-61-1998	FAT1	ENST00000441802	p.L1625F	c.4875A>C	Unverified	GAA	TTA	GAT	3	A	C
TCGA-25-1313	FLAD1	ENST00000292180	p.I436L	c.1306A>C	Unverified	TAT	ATC	CGC	1	A	C
TCGA-13-0906	FLNB	ENST00000295956	p.T1065P	c.3193A>C	Unverified	GAT	ACC	AAA	1	A	C
TCGA-24-1417	FNIP1	ENST00000510461	p.E415D	c.1245A>C	Unverified	GGA	GAA	CCT	3	A	C
TCGA-29-1695	FTSJD1	ENST00000338099	p.K3T	c.8A>C	Unverified	AGT	AAG	TGC	2	A	C
TCGA-23-1122	GHRHR	ENST00000326139	p.N216T	c.647A>C	Verified	ACC	AAC	TTC	2	A	C
TCGA-61-1725	GLCE	ENST00000261858	p.K546Q	c.1636A>C	Unverified	CTT	AAA	GCC	1	A	C
TCGA-24-1423	GOLGA3	ENST00000204726	p.N258T	c.773A>C	Verified	GGG	AAT	TCT	2	A	C
TCGA-09-2051	GOLGA4	ENST00000361924	p.K648T	c.1943A>C	Unverified	GAA	AAG	TGT	2	A	C
TCGA-24-2024	GPR82	ENST00000302548	p.L110F	c.330A>C	Verified	ATT	TTA	AGT	3	A	C
TCGA-24-2024	GPR82_ENST	ENST00000302548	p.L110F	c.330A>C	Verified	ATT	TTA	AGT	3	A	C
TCGA-13-0916	GRIK2_ENST	ENST00000421544	p.K876T	c.2627A>C	Verified	TTA	AAA	CAT	2	A	C
TCGA-24-1846	GRIN2A	ENST00000396573	p.N992T	c.2975A>C	Unverified	tcc	aac	cct	2	A	C
TCGA-29-1695	GRM8	ENST00000339582	p.T786P	c.2356A>C	Unverified	TTT	ACC	ATG	1	A	C
TCGA-29-1691	HAPLN4	ENST00000291481	p.Y112S	c.335A>C	Unverified	AGC	TAC	CGT	2	A	C
TCGA-23-1111	HMOX2	ENST00000219700	p.Y115S	c.344A>C	Unverified	GAG	TAT	TTC	2	A	C
TCGA-13-1509	HPS3	ENST00000296051	p.K937N	c.2811A>C	Verified	TGG	AAA	AAA	3	A	C
TCGA-13-0760	HSP90AA1	ENST00000334701	p.K180T	c.539A>C	Unverified	GAC	AAA	ATC	2	A	C
TCGA-61-1915	IGSF22	ENST00000319338	p.K353T	c.1058A>C	Unverified	TCC	AAG	AAA	2	A	C
TCGA-61-1915	IGSF22_ENS	ENST00000513874	p.K353T	c.1058A>C	Unverified	TCC	AAG	AAA	2	A	C
TCGA-29-1698	ISCU	ENST00000392807	p.K85T	c.254A>C	Unverified	GTG	AAA	GGA	2	A	C
TCGA-23-1122	IVD	ENST00000487418	p.N43T	c.128A>C	Unverified	ATC	AAT	GGG	2	A	C
TCGA-30-1891	KCTD19	ENST00000304372	p.E240D	c.720A>C	Unverified	GTG	GAA	ATT	3	A	C
TCGA-24-1845	KIAA0467_E	ENST00000372442	p.K1818T	c.5453A>C	Unverified	GAA	AAG	GAG	2	A	C
TCGA-04-1649	KIAA0528	ENST00000333957	p.I618I	c.1854A>C	Unverified	AAG	ATA	AAT	3	A	C
TCGA-04-1649	KIAA0528_E	ENST00000544930	p.I433I	c.1299A>C	Unverified	AAG	ATA	AAT	3	A	C

TCGA-23-1122	KIF4A	ENST00000374403	p.K444T	c.1331A>C	Verified	tgc	aaa	ctg	2	A	C
TCGA-23-1110	KRT35	ENST00000393989	p.N440T	c.1319A>C	Verified	ACA	AAC	TGC	2	A	C
TCGA-04-1337	L1CAM	ENST00000370060	p.M1012L	c.3034A>C	Unverified	ACT	ATG	GCC	1	A	C
TCGA-25-2401	LAMA1	ENST00000389658	p.K1723T	c.5168A>C	Verified	CTC	AAG	GCT	2	A	C
TCGA-25-2400	LAMA3	ENST00000313654	p.I788L	c.2362A>C	Verified	gtt	att	ctg	1	A	C
TCGA-23-1116	LAMC1	ENST00000258341	p.K1594T	c.4781A>C	Verified	AGG	AAG	ACC	2	A	C
TCGA-25-1313	LAPTM4A	ENST00000175091	p.I45L	c.133A>C	Verified	GCA	ATT	TTG	1	A	C
TCGA-29-1695	LRBA	ENST00000357115	p.K2117N	c.6351A>C	Unverified	AAA	AAA	ATC	3	A	C
TCGA-30-1855	LRRC8A	ENST00000372599	p.K246T	c.737A>C	Unverified	GAG	AAG	GTG	2	A	C
TCGA-13-1501	MAB21L1	ENST00000379919	p.K175N	c.525A>C	Verified	TTT	AAA	TGC	3	A	C
TCGA-13-1501	MAB21L1	ENST00000379919	p.K175N	c.525A>C	Verified	TTT	AAA	TGC	3	A	C
TCGA-24-1431	MAN2A2	ENST00000360468	p.Q624H	c.1872A>C	Unverified	CTC	CAA	GTG	3	A	C
TCGA-29-2427	MANF	ENST00000528157	p.K170Q	c.508A>C	Unverified	CCT	AAA	TAT	1	A	C
TCGA-13-1499	MAP4K3	ENST00000263881	p.Q473H	c.1419A>C	Verified	tcc	caa	gtt	3	A	C
TCGA-20-0991	MC5R	ENST00000324750	p.K27N	c.81A>C	Unverified	GTC	AAA	AAC	3	A	C
TCGA-30-1718	MFAP3	ENST00000322602	p.N351T	c.1052A>C	Unverified	TGT	AAC	TAC	2	A	C
TCGA-24-2267	MITF	ENST00000394351	p.N323T	c.968A>C	Verified	GAG	AAC	TGC	2	A	C
TCGA-13-0755	MLL3	ENST00000262189	p.Y366S	c.1097A>C	Verified	CAC	TAT	CAT	2	A	C
TCGA-29-1711	MLL5	ENST00000257745	p.M906L	c.2716A>C	Unverified	CCT	ATG	GAC	1	A	C
TCGA-29-1711	MLL5	ENST00000257745	p.M906L	c.2716A>C	Unverified	CCT	ATG	GAC	1	A	C
TCGA-13-0923	MOGAT3	ENST00000223114	p.K14N	c.42A>C	Unverified	TCC	AAA	ACC	3	A	C
TCGA-61-1904	MORC1	ENST00000232603	p.K860T	c.2579A>C	Unverified	AAC	AAA	AAG	2	A	C
TCGA-61-2095	MUC4	ENST00000346145	p.Y1092S	c.3275A>C	Unverified	GGC	TAC	TGT	2	A	C
TCGA-61-2095	MUC4_ENST	ENST00000405167	p.Y5200S	c.15599A>C	Unverified	GGC	TAC	TGT	2	A	C
TCGA-61-1910	MYH13	ENST00000252172	p.M214L	c.640A>C	Unverified	AAA	ATG	CAG	1	A	C
TCGA-61-1910	MYH13_ENS	ENST00000570743	p.M214L	c.640A>C	Unverified	AAA	ATG	CAG	1	A	C
TCGA-13-1497	MYH4	ENST00000255381	p.T927P	c.2779A>C	Verified	GTA	ACT	GAA	1	A	C
TCGA-23-1118	MYO18B	ENST00000335473	p.E2086D	c.6258A>C	Verified	GAA	GAA	GTG	3	A	C
TCGA-25-1326	MYO5C	ENST00000261839	p.E1117D	c.3351A>C	Verified	ATT	GAA	GAT	3	A	C
TCGA-13-1497	MYOM1	ENST00000356443	p.N1147H	c.3439A>C	Verified	GTA	AAT	GTG	1	A	C
TCGA-13-0714	MYST3	ENST00000265713	p.E673D	c.2019A>C	Verified	CGT	GAA	GGC	3	A	C
TCGA-13-1498	NOL4	ENST00000261592	p.T172P	c.514A>C	Verified	GGA	ACA	GAT	1	A	C
TCGA-04-1530	NOL8	ENST00000442668	p.N414T	c.1241A>C	Verified	AAA	AAT	AGA	2	A	C
TCGA-09-2050	NONO	ENST00000276079	p.L93L	c.279A>C	Verified	aaa	cta	ttt	3	A	C

TCGA-61-1913	NUP210	ENST00000254508	p.V610V	c.1830A>C	Unverified	CGG	GTA	AAG	3	A	C
TCGA-04-1342	OR2AG2	ENST00000338569	p.Q270H	c.810A>C	Verified	AAA	CAA	GAC	3	A	C
TCGA-09-2051	OR2T2	ENST00000342927	p.V305V	c.915A>C	Unverified	AAA	GTA	CTA	3	A	C
TCGA-23-1022	OR4D6	ENST00000300127	p.Y287S	c.860A>C	Verified	ATC	TAT	TCC	2	A	C
TCGA-13-0900	OR4E2	ENST00000408935	p.L4L	c.12A>C	Verified	AGT	CTA	AAC	3	A	C
TCGA-13-0905	OR6Y1	ENST00000302617	p.I214L	c.640A>C	Verified	GCT	ATT	CCT	1	A	C
TCGA-13-0795	OSTalpha	ENST00000296327	p.L268L	c.804A>C	Verified	GCC	CTA	CAG	3	A	C
TCGA-13-0885	PABPCP2	ENST00000359464	p.L187F	c.561A>C	Verified	GCT	TTA	GCT	3	A	C
TCGA-13-0793	PBRM1_ENS	ENST00000296302	p.I193I	c.579A>C	Unverified	GCC	ATA	GTT	3	A	C
TCGA-23-1116	PCDHA8	ENST00000378123	p.K343N	c.1029A>C	Unverified	GAT	AAA	AAT	3	A	C
TCGA-23-1120	PDE12	ENST00000311180	p.V270V	c.810A>C	Unverified	GTG	GTA	GAG	3	A	C
TCGA-24-1844	PIKFYVE	ENST00000264380	p.N310T	c.929A>C	Unverified	ACT	AAC	CTG	2	A	C
TCGA-13-0913	PJA2	ENST00000361189	p.K46N	c.138A>C	Verified	TTT	AAA	CCA	3	A	C
TCGA-13-0760	PLCB2	ENST00000260402	p.K71T	c.212A>C	Verified	GGG	AAG	TTT	2	A	C
TCGA-20-0991	PLCB4	NM_000933.2	p.R167R	c.499A>C	Verified	gtt	agg	agt	1	A	C
TCGA-24-1563	POTEJ	ENST00000409602	p.Q559H	c.1677A>C	Unverified	GAA	CAA	AAT	3	A	C
TCGA-24-1563	POTEJ_ENST	ENST00000409602_v68	p.Q559H	c.1677A>C	Unverified	GAA	CAA	AAT	3	A	C
TCGA-13-0762	PRKDC	NM_006904	p.N1662H	c.4984A>C	Verified	ttt	aat	aca	1	A	C
TCGA-13-0762	PRKDC	NM_006904	p.N1662H	c.4984A>C	Verified	ttt	aat	aca	1	A	C
TCGA-13-0900	PTCHD3	ENST00000438700	p.K685T	c.2054A>C	Verified	GAA	AAA	AAT	2	A	C
TCGA-24-2267	PUS10	ENST00000316752	p.K70N	c.210A>C	Verified	AAG	AAA	ATT	3	A	C
TCGA-23-2077	PXDN	ENST00000252804	p.Y1176S	c.3527A>C	Verified	GTC	TAC	TGC	2	A	C
TCGA-13-0760	RAB3IP	ENST00000550536	p.K5T	c.14A>C	Verified	aaa	aag	atg	2	A	C
TCGA-24-1104	RABGAP1	NM_012197.2	p.V40V	c.120A>C	Verified	tta	gta	ggg	3	A	C
TCGA-13-0755	RAD54L2	ENST00000409535	p.S681R	c.2041A>C	Verified	AAT	AGC	AAG	1	A	C
TCGA-09-2050	RAG1	ENST00000299440	p.K969T	c.2906A>C	Verified	AAC	AAA	CTG	2	A	C
TCGA-13-0924	RBM14	ENST00000310137	p.L608F	c.1824A>C	Verified	CGT	TTA	GCC	3	A	C
TCGA-61-1900	RFPL3	ENST00000397468	p.T147P	c.439A>C	Unverified	TTT	ACC	TGT	1	A	C
TCGA-61-1900	RFPL3_ENST	ENST00000249007	p.T176P	c.526A>C	Unverified	TTT	ACC	TGT	1	A	C
TCGA-24-0975	RFX1	ENST00000254325	p.N551T	c.1652A>C	Unverified	ACC	AAC	GGC	2	A	C
TCGA-29-1698	RGS7	ENST00000366565	p.Y290S	c.869A>C	Unverified	CAG	TAT	TTA	2	A	C
TCGA-29-1698	RGS7_ENST(	ENST00000366565	p.Y290S	c.869A>C	Unverified	CAG	TAT	TTA	2	A	C
TCGA-13-1498	RIF1	ENST00000444746	p.E1319D	c.3957A>C	Verified	gtt	gaa	ggc	3	A	C
TCGA-13-0807	RIMS2	ENST00000507740	p.Y658S	c.1973A>C	Verified	TGG	TAC	AAA	2	A	C

TCGA-13-0807	RIMS2_ENS	ENST00000436393	p.Y644S	c.1931A>C	Verified	TGG	TAC	AAA	2	A	C
TCGA-20-0990	RNASE9	ENST00000338904	p.K154Q	c.460A>C	Verified	TGT	AAA	TAC	1	A	C
TCGA-61-2102	RNASE9	ENST00000338904	p.I127L	c.379A>C	Verified	GGA	ATT	AGG	1	A	C
TCGA-20-1683	RNF20	ENST00000389120	p.T358P	c.1072A>C	Unverified	act	aca	caa	1	A	C
TCGA-23-1031	RNF213	ENST00000336301	p.I568L	c.1702A>C	Verified	GCT	ATA	AGC	1	A	C
TCGA-29-1783	ROBO1	ENST00000464233	p.E1236D	c.3708A>C	Unverified	GAT	GAA	CGA	3	A	C
TCGA-29-1783	ROBO1_ENS	ENST00000464233	p.E1236D	c.3708A>C	Unverified	GAT	GAA	CGA	3	A	C
TCGA-29-1783	ROBO1_ENS	ENST00000464233	p.E1236D	c.3708A>C	Unverified	GAT	GAA	CGA	3	A	C
TCGA-29-1783	ROBO1_ENS	ENST00000495273	p.E1191D	c.3573A>C	Unverified	GAT	GAA	CGA	3	A	C
TCGA-61-1725	RPGRIP1L	ENST00000379925	p.L735F	c.2205A>C	Unverified	CGA	TTA	AGA	3	A	C
TCGA-24-1846	RXFP3	ENST00000330120	p.K398T	c.1193A>C	Unverified	CGC	AAG	GCG	2	A	C
TCGA-13-1507	RYR2	ENST00000366574	p.K1745T	c.5234A>C	Verified	AAA	AAA	CAC	2	A	C
TCGA-24-2289	SBF2	ENST00000256190	p.N766T	c.2297A>C	Unverified	AAA	AAC	AAG	2	A	C
TCGA-13-0761	SCIN	ENST00000297029	p.K672T	c.2015A>C	Verified	CTG	AAG	TCT	2	A	C
TCGA-13-0761	SCIN	ENST00000297029	p.K672T	c.2015A>C	Verified	CTG	AAG	TCT	2	A	C
TCGA-29-1775	SCN11A	ENST00000302328	p.T372P	c.1114A>C	Unverified	ACT	ACT	GGG	1	A	C
TCGA-13-0885	SDR16C5	ENST00000303749	p.I42L	c.124A>C	Verified	GAA	ATA	GTC	1	A	C
TCGA-13-0903	SEC23B	ENST00000377465	p.T116P	c.346A>C	Verified	TCT	ACA	ATT	1	A	C
TCGA-24-1849	SEMA4B	ENST00000332496	p.Y160S	c.479A>C	Unverified	acc	tac	atc	2	A	C
TCGA-13-0890	SIK3	SU_QSK	p.L773F	c.2319A>C	Verified	agg	tta	agg	3	A	C
TCGA-04-1343	SLC24A2	ENST00000341998	p.Y515S	c.1544A>C	Verified	TCT	TAC	TTG	2	A	C
TCGA-29-1711	SLC5A7	ENST00000264047	p.S356R	c.1066A>C	Unverified	GCA	AGT	TCC	1	A	C
TCGA-29-1711	SLC5A7	ENST00000264047	p.S356R	c.1066A>C	Unverified	GCA	AGT	TCC	1	A	C
TCGA-61-1740	SLC9A10	ENST00000305815	p.L277F	c.831A>C	Unverified	CTT	TTA	AAT	3	A	C
TCGA-25-1326	SLITRK2	ENST00000447897	p.K512N	c.1536A>C	Verified	gtg	aaa	ggg	3	A	C
TCGA-25-2042	SNX6	NM_152233.1	p.R278R	c.832A>C	Unverified	aca	aga	aaa	1	A	C
TCGA-13-0795	SPIN3	NM_001010862.1	p.N48T	c.143A>C	Verified	ggg	aac	atc	2	A	C
TCGA-24-1469	SPTA1	ENST00000368148	p.K679N	c.2037A>C	Verified	CAG	AAA	GGG	3	A	C
TCGA-24-2288	SRCAP	ENST00000262518	p.L1507L	c.4521A>C	Unverified	ACT	CTA	GGT	3	A	C
TCGA-61-1907	SRPK2	ENST00000357311	p.N267T	c.800A>C	Unverified	aaa	aac	aaa	2	A	C
TCGA-29-1702	SRSF7	ENST00000313117	p.L37F	c.111A>C	Unverified	CCT	TTA	AGA	3	A	C
TCGA-23-1021	STK38	ENST00000229812	p.K332T	c.995A>C	Verified	tat	aag	aag	2	A	C
TCGA-24-2289	SVIL	ENST00000355867	p.V327V	c.981A>C	Unverified	TCC	GTA	ACT	3	A	C
TCGA-24-1845	SZT2	ENST00000372442	p.K1818T	c.5453A>C	Unverified	GAA	AAG	GAG	2	A	C

TCGA-24-1845	SZT2_ENST0	ENST00000562955	p.K2660T	c.7979A>C	Unverified	GAA	AAG	GAG	2	A	C
TCGA-30-1714	TCEAL4	NM_024863.3	p.K189T	c.566A>C	Unverified	gga	aag	aca	2	A	C
TCGA-24-1435	TCP11	ENST00000373979	p.T426P	c.1276A>C	Verified	AAA	ACC	CTC	1	A	C
TCGA-13-0791	TGFBR2	NM_003242	p.K468Q	c.1402A>C	Verified	gta	aaa	gat	1	A	C
TCGA-13-0900	TMEM177	ENST00000272521	p.N277T	c.830A>C	Verified	GGG	AAC	ATC	2	A	C
TCGA-24-1845	TMEM98	ENST00000394642	p.L62F	c.186A>C	Unverified	GAG	TTA	GAA	3	A	C
TCGA-09-1665	TOMM70A	ENST00000284320	p.K188T	c.563A>C	Verified	GTG	AAA	GCT	2	A	C
TCGA-61-2095	TRAM1L1	ENST00000310754	p.N216T	c.647A>C	Unverified	TTG	AAT	CAT	2	A	C
TCGA-29-1783	TRO	ENST00000319167	p.I523I	c.1569A>C	Unverified	ggc	ata	ctg	3	A	C
TCGA-29-1783	TRO_ENST0(	ENST00000173898	p.I523I	c.1569A>C	Unverified	GGC	ATA	CTG	3	A	C
TCGA-13-0885	TSHZ2	ENST00000371497	p.E638D	c.1914A>C	Verified	AGT	GAA	ACA	3	A	C
TCGA-29-1769	TTC21B	ENST00000243344	p.K220T	c.659A>C	Unverified	ATG	AAA	TTA	2	A	C
TCGA-61-1915	TTN	NM_003319	p.Y12727S	c.38180A>C	Unverified	ggc	tat	ttc	2	A	C
TCGA-61-1915	TTN_ENST0(	ENST00000342175	p.Y12919S	c.38756A>C	Unverified	GGC	TAT	TTC	2	A	C
TCGA-61-1915	TTN_ENST0(	ENST00000342992	p.Y19224S	c.57671A>C	Unverified	GGC	TAT	TTC	2	A	C
TCGA-61-1915	TTN_ENST0(	ENST00000356127	p.Y19222S	c.57665A>C	Unverified	GGC	TAT	TTC	2	A	C
TCGA-61-1915	TTN_ENST0(	ENST00000359218	p.Y12852S	c.38555A>C	Unverified	GGC	TAT	TTC	2	A	C
TCGA-13-1488	USP4	ENST00000265560	p.K186N	c.558A>C	Verified	aac	aaa	tac	3	A	C
TCGA-61-2009	WDFY3	ENST00000295888	p.Y411S	c.1232A>C	Verified	ATT	TAC	ATG	2	A	C
TCGA-61-1907	WNK1	NM_018979	p.N2045T	c.6134A>C	Unverified	cag	aat	cta	2	A	C
TCGA-61-1907	WNK1_ENST	ENST00000537687	p.N2305T	c.6914A>C	Unverified	CAG	AAT	CTA	2	A	C
TCGA-13-0761	XPC	ENST00000285021	p.K387T	c.1160A>C	Verified	AAC	AAG	GGA	2	A	C
TCGA-13-0761	XPC	ENST00000285021	p.K387T	c.1160A>C	Verified	AAC	AAG	GGA	2	A	C
TCGA-13-1499	XRCC6	ENST00000360079	p.E418D	c.1254A>C	Verified	GAA	GAA	GAG	3	A	C
TCGA-25-2042	ZBTB25	ENST00000394715	p.Q69H	c.207A>C	Verified	ATC	CAA	CCT	3	A	C
TCGA-23-1116	ZGPAT	ENST00000328969	p.Y139S	c.416A>C	Unverified	TAC	TAC	AGC	2	A	C
TCGA-25-1313	ZNF132	ENST00000254166	p.K675N	c.2025A>C	Unverified	CAG	AAA	GTT	3	A	C
TCGA-25-1326	ZNF14	ENST00000344099	p.R64R	c.190A>C	Verified	CGA	AGA	TGT	1	A	C
TCGA-23-1118	ZNF222	ENST00000187879	p.Q84H	c.252A>C	Verified	TCT	CAA	GAT	3	A	C
TCGA-24-2289	ZNF227	ENST00000313040	p.N366T	c.1097A>C	Unverified	TCA	AAT	TTT	2	A	C
TCGA-24-2267	ZNF425	ENST00000378061	p.K468Q	c.1402A>C	Unverified	CAA	AAG	CCC	1	A	C
TCGA-29-1775	ZNF573	ENST00000339503	p.K214T	c.641A>C	Unverified	GAA	AAA	CCA	2	A	C
TCGA-13-0900	ZNF654_ENS	ENST00000309495	p.K567T	c.1700A>C	Verified	AAG	AAA	TGT	2	A	C
TCGA-61-1998	ZNF791	ENST00000343325	p.K167N	c.501A>C	Verified	GGA	AAA	ACC	3	A	C

TCGA-13-1488	ABCB4	ENST00000359206	p.D576G	c.1727A>G	Verified	CTG	GAT	AAG	2	A	G
TCGA-04-1331	ABHD13	ENST00000375898	p.P223P	c.669A>G	Verified	ATA	CCA	CAT	3	A	G
TCGA-23-1114	AC073082.1	ENST00000378305	p.P36P	c.108A>G	Unverified	ACA	CCA	GAA	3	A	G
TCGA-23-1114	AC073082.1_	ENST00000378305_v68	p.P36P	c.108A>G	Unverified	ACA	CCA	GAA	3	A	G
TCGA-24-2035	ACP6	ENST00000369238	p.K236E	c.706A>G	Verified	GTG	AAG	GAC	1	A	G
TCGA-29-1777	ACSM1	ENST00000307493	p.S318S	c.954A>G	Unverified	GTA	TCA	TCT	3	A	G
TCGA-09-1665	ACSS3	ENST00000548058	p.G500G	c.1500A>G	Verified	TTA	GGA	AAT	3	A	G
TCGA-13-0714	ACTC1	ENST00000290378	p.S241G	c.721A>G	Verified	AAG	AGC	TAT	1	A	G
TCGA-13-0900	ADAMTS16_	ENST00000274181	p.T1148A	c.3442A>G	Unverified	CAG	ACG	AGG	1	A	G
TCGA-23-1031	ADCY9	ENST00000294016	p.M1197V	c.3589A>G	Verified	AGG	ATG	GAC	1	A	G
TCGA-13-0887	ADI1	ENST00000327435	p.K144E	c.430A>G	Verified	ACG	AAG	GCC	1	A	G
TCGA-29-1781	ADNP	ENST00000349014	p.H563R	c.1688A>G	Unverified	ATC	CAT	CTC	2	A	G
TCGA-13-0883	AHCYL2	ENST00000325006	p.D287G	c.860A>G	Verified	GAA	GAT	GAC	2	A	G
TCGA-30-1855	AIM2	ENST00000368130	p.H130R	c.389A>G	Unverified	CCT	CAT	GTT	2	A	G
TCGA-29-1764	AK5	ENST00000354567	p.D352G	c.1055A>G	Unverified	GAA	GAT	CAG	2	A	G
TCGA-23-1122	ALCAM	ENST00000306107	p.P441P	c.1323A>G	Verified	TTT	CCA	AAG	3	A	G
TCGA-13-1497	ALDH3A2	ENST00000581518	p.I371V	c.1111A>G	Verified	ctc	atc	aaa	1	A	G
TCGA-24-2262	ALG1	ENST00000262374	p.Q339R	c.1016A>G	Verified	TTC	CAG	CAC	2	A	G
TCGA-13-0920	ANAPC4	ENST00000315368	p.R632R	c.1896A>G	Verified	gtc	aga	aga	3	A	G
TCGA-23-1117	ANPEP	ENST00000300060	p.G143G	c.429A>G	Verified	GTG	GGA	GGC	3	A	G
TCGA-23-1117	ANPEP	ENST00000300060	p.G143G	c.429A>G	Verified	GTG	GGA	GGC	3	A	G
TCGA-04-1338	APC	ENST00000457016	p.E1286G	c.3857A>G	Unverified	GAT	GAA	ATA	2	A	G
TCGA-24-2024	APC	ENST00000457016	p.Q1429R	c.4286A>G	Verified	GGA	CAA	ACC	2	A	G
TCGA-10-0930	APCDD1L	ENST00000371149	p.I50V	c.148A>G	Verified	gcg	atc	ctg	1	A	G
TCGA-10-0930	APCDD1L	ENST00000371149	p.I50V	c.148A>G	Verified	gcg	atc	ctg	1	A	G
TCGA-61-1895	AQP11	ENST00000313578	p.S149G	c.445A>G	Unverified	GTC	AGC	GAG	1	A	G
TCGA-13-0890	ARHGEF15	ENST00000361926	p.E754G	c.2261A>G	Verified	tat	gag	gac	2	A	G
TCGA-24-2280	ARHGEF17	ENST00000263674	p.R1838R	c.5514A>G	Unverified	aac	cga	gtc	3	A	G
TCGA-29-1777	ARL5B	ENST00000377275	p.N78D	c.232A>G	Unverified	tgg	aac	aca	1	A	G
TCGA-24-0979	ASAP1	ENST00000357668	p.D720G	c.2159A>G	Verified	GAT	GAT	CTG	2	A	G
TCGA-23-2077	ASAP1	ENST00000357668	p.G937G	c.2811A>G	Verified	CCT	GGA	GAC	3	A	G
TCGA-24-1844	ATAD3C	ENST00000378785	p.P178P	c.534A>G	Unverified	GGG	CCA	CCA	3	A	G
TCGA-61-1914	ATIC_ENST0	ENST00000236959	p.G345G	c.1035A>G	Unverified	CCA	GGA	TAT	3	A	G
TCGA-04-1367	ATP11A	ENST00000375645	p.E204G	c.611A>G	Unverified	ACA	GAG	GAG	2	A	G

TCGA-04-1336	ATP5O	ENST00000290299	p.R64G	c.190A>G	Verified	TTG	AGA	GTA	1	A	G
TCGA-20-0991	ATRIP	ENST00000320211	p.H418R	c.1253A>G	Unverified	GTG	CAT	TTC	2	A	G
TCGA-13-0890	AURKA	ENST00000395909	p.G265G	c.795A>G	Verified	CTT	GGA	TCA	3	A	G
TCGA-29-1785	BAI3	ENST00000370598	p.T976T	c.2928A>G	Unverified	AGG	ACA	CGG	3	A	G
TCGA-29-1785	BAI3	ENST00000370598	p.T976T	c.2928A>G	Unverified	AGG	ACA	CGG	3	A	G
TCGA-13-0893	BAZ1B	ENST00000339594	p.H1033R	c.3098A>G	Verified	ATT	CAT	CTA	2	A	G
TCGA-61-1904	BCOR	ENST00000397354	p.T347A	c.1039A>G	Unverified	CCC	ACC	CAG	1	A	G
TCGA-13-0762	BCORL1	ENST00000218147	p.K913E	c.2737A>G	Verified	gcc	aag	cct	1	A	G
TCGA-13-0762	BCORL1	ENST00000218147	p.K913E	c.2737A>G	Verified	gcc	aag	cct	1	A	G
TCGA-30-1857	BDKRB1	ENST00000216629	p.I178V	c.532A>G	Unverified	TCC	ATC	CAA	1	A	G
TCGA-24-1464	BECN1	ENST00000361523	p.D405G	c.1214A>G	Verified	gaa	gac	aca	2	A	G
TCGA-25-1313	BIN3	ENST00000276416	p.E143G	c.428A>G	Unverified	GTG	GAG	AAG	2	A	G
TCGA-24-1469	BIRC3	ENST00000263464	p.S123S	c.369A>G	Verified	cac	tca	tta	3	A	G
TCGA-61-2008	BLM	ENST00000355112	p.I1279V	c.3835A>G	Verified	GTG	ATT	TCA	1	A	G
TCGA-13-0904	BMP3	ENST00000282701	p.D193G	c.578A>G	Verified	CGA	GAT	ATT	2	A	G
TCGA-23-1022	BMP3	ENST00000282701	p.G150G	c.450A>G	Verified	TCT	GGA	GGA	3	A	G
TCGA-24-1103	BMP8B	ENST00000372827	p.A264A	c.792A>G	Unverified	CGG	GCA	GTG	3	A	G
TCGA-61-1740	BOD1L	ENST00000040738	p.S124S	c.372A>G	Unverified	AAA	TCA	GGA	3	A	G
TCGA-61-2009	BUB3	ENST00000368865	p.E232G	c.695A>G	Verified	ATT	GAG	CAG	2	A	G
TCGA-23-1124	C10orf96	ENST00000333254	p.E155G	c.464A>G	Verified	ATG	GAA	CAT	2	A	G
TCGA-13-0884	C12orf11	ENST00000261191	p.N36D	c.106A>G	Verified	AAG	AAT	AGA	1	A	G
TCGA-09-2056	C19orf46	ENST00000324444	p.N107D	c.319A>G	Verified	CAG	AAC	AGC	1	A	G
TCGA-09-2056	C1orf168	ENST00000343433	p.K637E	c.1909A>G	Verified	ACC	AAG	AAG	1	A	G
TCGA-24-1563	C1orf172	ENST00000320567	p.S158G	c.472A>G	Verified	AGC	AGC	TTC	1	A	G
TCGA-23-1022	C21orf63	ENST00000300255	p.R344R	c.1032A>G	Verified	ATC	AGA	GAG	3	A	G
TCGA-24-1850	C2orf78	ENST00000342345	p.D454G	c.1361A>G	Unverified	CTT	GAC	CAA	2	A	G
TCGA-24-1850	C2orf78_EN:	ENST00000409561	p.D464G	c.1391A>G	Unverified	CTT	GAC	CAA	2	A	G
TCGA-25-1318	C3orf23	ENST00000342649	p.R185R	c.555A>G	Verified	TCG	AGA	GTG	3	A	G
TCGA-25-1318	C3orf23	ENST00000342649	p.R185R	c.555A>G	Verified	TCG	AGA	GTG	3	A	G
TCGA-24-2289	C3orf70	ENST00000335012	p.K107E	c.319A>G	Unverified	TTG	AAA	TTT	1	A	G
TCGA-29-1691	C5orf28	ENST00000500337	p.D91G	c.272A>G	Unverified	ATT	GAT	GTA	2	A	G
TCGA-29-1761	C6orf170	ENST00000398212	p.G421G	c.1263A>G	Unverified	TTA	GGA	CAA	3	A	G
TCGA-29-1696	C6orf221	ENST00000370367	p.G60G	c.180A>G	Unverified	GGC	GGA	GAA	3	A	G
TCGA-29-1696	C6orf221	ENST00000370367	p.G60G	c.180A>G	Unverified	GGC	GGA	GAA	3	A	G

TCGA-23-1022	C7orf16	ENST00000342032	p.D136G	c.407A>G	Verified	AGG	GAC	GAG	2	A	G
TCGA-13-1481	C7orf58	ENST00000310396	p.D151G	c.452A>G	Verified	TGG	GAT	CTG	2	A	G
TCGA-23-1120	C7orf72	ENST00000297001	p.D336G	c.1007A>G	Verified	GAT	GAT	GTT	2	A	G
TCGA-13-0883	CABS1	ENST00000273936	p.I106V	c.316A>G	Verified	TCC	ATA	ACA	1	A	G
TCGA-29-1785	CAMTA1	ENST00000303635	p.T563A	c.1687A>G	Unverified	CTG	ACC	GCC	1	A	G
TCGA-29-1785	CAMTA1	ENST00000303635	p.T563A	c.1687A>G	Unverified	CTG	ACC	GCC	1	A	G
TCGA-13-1481	CAPN10	ENST00000354082	p.T332T	c.996A>G	Verified	TAC	ACA	GAG	3	A	G
TCGA-29-1761	CAPS	ENST00000222125	p.T7A	c.19A>G	Unverified	GCC	ACC	ATG	1	A	G
TCGA-24-2289	CASQ1	ENST00000368078	p.D178G	c.533A>G	Unverified	GAG	GAT	GAG	2	A	G
TCGA-30-1857	CCDC27	ENST00000294600	p.K84E	c.250A>G	Unverified	TGG	AAA	CCG	1	A	G
TCGA-24-2288	CCDC39	ENST00000273654	p.R725R	c.2175A>G	Unverified	AAT	AGA	TAT	3	A	G
TCGA-04-1347	CCDC88A	ENST00000263630	p.D1409G	c.4226A>G	Verified	AAA	GAT	ATT	2	A	G
TCGA-30-1718	CCNB3	ENST00000376042	p.K1299E	c.3895A>G	Unverified	GAG	AAG	GCT	1	A	G
TCGA-30-1718	CCNB3_ENS	ENST00000376042	p.K1299E	c.3895A>G	Unverified	GAG	AAG	GCT	1	A	G
TCGA-29-1777	CCT4	ENST00000394440	p.I529V	c.1585A>G	Unverified	AGC	ATT	CTG	1	A	G
TCGA-04-1356	CD163L1	ENST00000313599	p.H991R	c.2972A>G	Verified	ATC	CAT	GGA	2	A	G
TCGA-13-1488	CD200R1L	ENST00000398214	p.H135R	c.404A>G	Verified	TTC	CAT	CGT	2	A	G
TCGA-25-1326	CD276	ENST00000318443	p.S233G	c.697A>G	Unverified	CAC	AGC	TCT	1	A	G
TCGA-29-1705	CD46	ENST00000358170	p.D33G	c.98A>G	Unverified	TCC	GAT	GCC	2	A	G
TCGA-23-1124	CDCA7	ENST00000306721	p.E330G	c.989A>G	Verified	TTG	GAG	AAC	2	A	G
TCGA-23-1029	CDH6	ENST00000265071	p.E662G	c.1985A>G	Unverified	GAC	GAA	GGT	2	A	G
TCGA-61-2009	CDK19	ENST00000368911	p.D90G	c.269A>G	Verified	agt	gac	agg	2	A	G
TCGA-13-1408	CEACAM6	ENST00000199764	p.R72R	c.216A>G	Unverified	GAA	AGA	GTG	3	A	G
TCGA-61-1740	CEP128	ENST00000281129	p.A670A	c.2010A>G	Unverified	ACT	GCA	CAG	3	A	G
TCGA-24-1846	CEP192	ENST00000325971	p.R1932R	c.5796A>G	Unverified	ATT	CGA	CTA	3	A	G
TCGA-24-1846	CEP192_ENS	ENST00000506447	p.R2528R	c.7584A>G	Unverified	ATT	CGA	CTA	3	A	G
TCGA-09-2049	CHD5	ENST00000262450	p.M1167V	c.3499A>G	Verified	ATG	ATG	CTC	1	A	G
TCGA-23-1122	CHD8	ENST00000399982	p.H803R	c.2408A>G	Verified	tca	cat	gaa	2	A	G
TCGA-24-1469	CIT	SU_CRIK	p.A666A	c.1998A>G	Verified	cag	gca	aag	3	A	G
TCGA-30-1891	CLDN11	ENST00000064724	p.I151V	c.451A>G	Unverified	ACC	ATC	GTG	1	A	G
TCGA-10-0930	CLEC14A	ENST00000342213	p.E482G	c.1445A>G	Verified	GCG	GAG	TCC	2	A	G
TCGA-10-0930	CLEC14A	ENST00000342213	p.E482G	c.1445A>G	Verified	GCG	GAG	TCC	2	A	G
TCGA-61-2012	CLEC4F	ENST00000272367	p.I390V	c.1168A>G	Verified	GAG	ATA	CAG	1	A	G
TCGA-13-0904	CLEC4F	ENST00000272367	p.E97G	c.290A>G	Verified	AGG	GAG	GCA	2	A	G

TCGA-29-1693	CNN3	ENST00000370206	p.H309R	c.926A>G	Unverified	TAT	CAT	GGC	2	A	G
TCGA-29-1701	CNTN1	ENST00000551295	p.K755E	c.2263A>G	Unverified	tgg	aaa	aaa	1	A	G
TCGA-23-1120	COASY	ENST00000393818	p.N299D	c.895A>G	Unverified	ATC	AAC	CGC	1	A	G
TCGA-29-1690	COG2	ENST00000366669	p.R453R	c.1359A>G	Unverified	GCA	CGA	TAC	3	A	G
TCGA-24-1563	COPS7B	ENST00000410024	p.I162V	c.484A>G	Verified	gac	atc	cga	1	A	G
TCGA-23-1117	CPN2	ENST00000323830	p.S321S	c.963A>G	Verified	CTC	TCA	TAC	3	A	G
TCGA-23-1117	CPN2	ENST00000323830	p.S321S	c.963A>G	Verified	CTC	TCA	TAC	3	A	G
TCGA-23-2078	CRB1	ENST00000367400	p.E710G	c.2129A>G	Verified	AGA	GAG	TAT	2	A	G
TCGA-24-1435	CREB3L3	ENST00000078445	p.T135T	c.405A>G	Verified	ACC	ACA	ACC	3	A	G
TCGA-20-1686	CRMP1	ENST00000324989	p.I139V	c.415A>G	Unverified	ATC	ATC	AAC	1	A	G
TCGA-13-0762	CROT	ENST00000331536	p.K375E	c.1123A>G	Verified	GAG	AAA	GTT	1	A	G
TCGA-13-0762	CROT	ENST00000331536	p.K375E	c.1123A>G	Verified	GAG	AAA	GTT	1	A	G
TCGA-04-1362	CSGALNACT	ENST00000332246	p.D219G	c.656A>G	Verified	AGG	GAC	AAA	2	A	G
TCGA-61-1904	CSH1	ENST00000316193	p.E145G	c.434A>G	Unverified	GAG	GAA	GGC	2	A	G
TCGA-13-0924	CSMD3	ENST00000297405	p.D2079G	c.6236A>G	Verified	tgt	gat	caa	2	A	G
TCGA-29-1776	CSN1S1	ENST00000246891	p.E35G	c.104A>G	Unverified	AGT	GAG	CCT	2	A	G
TCGA-29-1699	CSN2	ENST00000353151	p.I210V	c.628A>G	Unverified	CAG	ATC	TAC	1	A	G
TCGA-24-1849	CTH	ENST00000370938	p.H324R	c.971A>G	Unverified	CAG	CAT	GCT	2	A	G
TCGA-13-0887	CTNND2	ENST00000304623	p.R196R	c.588A>G	Verified	gcc	cga	gct	3	A	G
TCGA-61-2008	CUL2	ENST00000374751	p.S524S	c.1572A>G	Verified	cct	tca	tct	3	A	G
TCGA-29-1766	CYBB	ENST00000378588	p.Q311R	c.932A>G	Unverified	cta	cag	atg	2	A	G
TCGA-13-0923	CYP2C19	ENST00000371321	p.Q324R	c.971A>G	Verified	GTC	CAG	GAA	2	A	G
TCGA-61-1915	CYSLTR1	ENST00000373304	p.T308T	c.924A>G	Unverified	tct	aca	ttc	3	A	G
TCGA-10-0930	DCAF15	ENST00000254337	p.T447T	c.1341A>G	Unverified	CTC	ACA	CTA	3	A	G
TCGA-10-0930	DCAF15	ENST00000254337	p.T447T	c.1341A>G	Unverified	CTC	ACA	CTA	3	A	G
TCGA-24-2262	DCDC2	ENST00000378454	p.Q91R	c.272A>G	Verified	GGC	CAG	GAA	2	A	G
TCGA-29-1693	DEFB127	ENST00000382388	p.K14E	c.40A>G	Unverified	CAG	AAA	CCC	1	A	G
TCGA-61-2012	DIO3	ENST00000359323	p.S240G	c.718A>G	Unverified	CAG	AGT	GGC	1	A	G
TCGA-04-1367	DLG1	ENST00000346964	p.K760E	c.2278A>G	Verified	GAC	AAA	TTT	1	A	G
TCGA-29-1769	DNAH11	ENST00000328843	p.N979D	c.2935A>G	Unverified	TGC	AAT	AGT	1	A	G
TCGA-04-1367	DNAH3	ENST00000261383	p.M2246V	c.6736A>G	Verified	AAG	ATG	CTG	1	A	G
TCGA-24-1470	DNAH5	ENST00000265104	p.S2616G	c.7846A>G	Verified	AAG	AGT	CTG	1	A	G
TCGA-29-1703	DNAJC1	ENST00000376980	p.Q395R	c.1184A>G	Unverified	gtt	cag	aat	2	A	G
TCGA-29-1764	DNAJC6	ENST00000395325	p.R32G	c.94A>G	Unverified	ggg	agg	ctc	1	A	G

TCGA-24-2035	DNMT1	ENST00000340748	p.T523A	c.1567A>G	Verified	TCG	ACC	TAT	1	A	G
TCGA-61-2095	DNTTIP1	ENST00000372622	p.Q124R	c.371A>G	Unverified	GAG	CAG	GCT	2	A	G
TCGA-24-1103	DOCK11	ENST00000276202	p.K234E	c.700A>G	Verified	CCC	AAA	ATG	1	A	G
TCGA-23-1114	DPPA3	ENST00000345088	p.D153G	c.458A>G	Unverified	caa	gac	acc	2	A	G
TCGA-13-0916	DSC1	ENST00000257197	p.E188G	c.563A>G	Verified	ATA	GAG	AAA	2	A	G
TCGA-23-1809	DSC2	ENST00000280904	p.T362T	c.1086A>G	Unverified	GTG	ACA	TCA	3	A	G
TCGA-23-1809	DSC2_ENST(	ENST00000251081	p.T362T	c.1086A>G	Unverified	GTG	ACA	TCA	3	A	G
TCGA-13-0886	DST	ENST00000370765	p.D696G	c.2087A>G	Verified	GAA	GAT	AGC	2	A	G
TCGA-09-2051	DST_ENST0(	ENST00000370769	p.I6046V	c.18136A>G	Unverified	GCC	ATT	TCT	1	A	G
TCGA-13-0886	DST_ENST0(	ENST00000370769	p.D1022G	c.3065A>G	Verified	GAA	GAT	AGC	2	A	G
TCGA-25-1313	DYNC2H1_E	ENST00000398093	p.G117G	c.351A>G	Verified	AGA	GGA	TAT	3	A	G
TCGA-24-1474	EBF3	ENST00000368648	p.S162G	c.484A>G	Unverified	TGC	AGC	CGG	1	A	G
TCGA-24-2290	ECH1	ENST00000221418	p.S28G	c.82A>G	Verified	CTC	AGT	ATT	1	A	G
TCGA-24-2290	ECH1	ENST00000221418	p.S28G	c.82A>G	Verified	CTC	AGT	ATT	1	A	G
TCGA-24-1431	ECM1	ENST00000369047	p.P196P	c.588A>G	Verified	CTA	CCA	CAG	3	A	G
TCGA-24-0979	EDDM3B	ENST00000326783	p.K26E	c.76A>G	Verified	AGC	AAA	GAA	1	A	G
TCGA-29-1705	EDIL3	ENST00000296591	p.D440G	c.1319A>G	Unverified	aat	gac	act	2	A	G
TCGA-24-0975	EED	ENST00000263360	p.P60P	c.180A>G	Unverified	acg	cca	aat	3	A	G
TCGA-13-1501	EGFR	ENST00000275493	p.Q1164R	c.3491A>G	Verified	CAC	CAA	ATT	2	A	G
TCGA-13-1501	EGFR	ENST00000275493	p.Q1164R	c.3491A>G	Verified	CAC	CAA	ATT	2	A	G
TCGA-61-1899	EHD3	ENST00000322054	p.I338V	c.1012A>G	Unverified	GAG	ATC	TAT	1	A	G
TCGA-24-1469	EIF2B5	ENST00000273783	p.N263D	c.787A>G	Verified	GAC	AAC	TTT	1	A	G
TCGA-13-0923	EIF2C4	ENST00000373210	p.K250E	c.748A>G	Verified	ACC	AAA	GAA	1	A	G
TCGA-29-1702	EMB	ENST00000303221	p.I131V	c.391A>G	Unverified	ACC	ATC	ATT	1	A	G
TCGA-13-1509	EML5	ENST00000554922	p.P268P	c.804A>G	Verified	AAA	CCA	ATT	3	A	G
TCGA-24-1563	EMR1	ENST00000312053	p.I533V	c.1597A>G	Verified	ATC	ATC	TAC	1	A	G
TCGA-29-1777	ENSG00001	ENST00000329762	p.M292V	c.874A>G	Unverified	ATC	ATG	GTC	1	A	G
TCGA-09-1674	ENSG00001	ENST00000356920	p.E783G	c.2348A>G	Unverified	ATG	GAG	AAG	2	A	G
TCGA-10-0930	ENSG00001	ENST00000338994	p.T72T	c.216A>G	Verified	CAC	ACA	TAC	3	A	G
TCGA-10-0930	ENSG00001	ENST00000338994	p.T72T	c.216A>G	Verified	CAC	ACA	TAC	3	A	G
TCGA-30-1718	ENSG00002	ENST00000430863	p.M760V	c.2278A>G	Unverified	GAG	ATG	CTG	1	A	G
TCGA-61-1904	ENTHD1	ENST00000325157	p.H92R	c.275A>G	Unverified	CAG	CAT	TGC	2	A	G
TCGA-24-1422	EPB41L3	ENST00000341928	p.T919A	c.2755A>G	Verified	GAG	ACA	GCC	1	A	G
TCGA-10-0930	EPHA6	SU_EPHA6	p.T72T	c.216A>G	Verified	cac	aca	tac	3	A	G

TCGA-10-0930	EPHA6	SU_EPHA6	p.T72T	c.216A>G	Verified	cac	aca	tac	3	A	G
TCGA-23-1809	EPHX2	ENST00000521400	p.I511V	c.1531A>G	Unverified	TGG	ATT	CCC	1	A	G
TCGA-24-1849	ESPL1	ENST00000257934	p.D1403G	c.4208A>G	Unverified	GAA	GAC	CCT	2	A	G
TCGA-04-1367	ESPN	ENST00000377828	p.D205G	c.614A>G	Unverified	CAC	GAC	GGC	2	A	G
TCGA-29-2427	EWSR1	ENST00000397938	p.T414A	c.1240A>G	Verified	GCC	ACA	GTG	1	A	G
TCGA-13-1507	EXOC4	ENST00000253861	p.D643G	c.1928A>G	Verified	GAT	GAT	GAT	2	A	G
TCGA-04-1336	EXOC5	ENST00000413566	p.?	c.939-2A>G	Verified	ACA	AGA	ACC	3	A	G
TCGA-23-1114	F13B	ENST00000367412	p.E590G	c.1769A>G	Unverified	ATG	GAA	AAG	2	A	G
TCGA-24-2035	FAM55C	ENST00000273347	p.I412V	c.1234A>G	Verified	CCC	ATC	CGC	1	A	G
TCGA-13-0906	FANCA	ENST00000389301	p.S1094G	c.3280A>G	Verified	AGC	AGC	TTC	1	A	G
TCGA-13-1496	FANCI	ENST00000300027	p.D611G	c.1832A>G	Verified	TAT	GAT	GTT	2	A	G
TCGA-61-1910	FAT4	ENST00000330166	p.P994P	c.2982A>G	Unverified	TCA	CCA	GTG	3	A	G
TCGA-61-1910	FAT4_ENST(	ENST00000394329	p.P994P	c.2982A>G	Unverified	TCA	CCA	GTG	3	A	G
TCGA-04-1347	FGGY	ENST00000315089	p.D438G	c.1313A>G	Verified	AAT	GAT	GAC	2	A	G
TCGA-29-1690	FLG	ENST00000368799	p.D2936G	c.8807A>G	Unverified	GAA	GAC	AGA	2	A	G
TCGA-13-1481	FLG2	ENST00000388718	p.H1454R	c.4361A>G	Verified	CAA	CAT	GGA	2	A	G
TCGA-13-0906	FLG2	ENST00000388718	p.T2241T	c.6723A>G	Unverified	TCC	ACA	CAG	3	A	G
TCGA-61-2009	FLJ35880	ENST00000312481	p.Q77R	c.230A>G	Verified	AAC	CAA	CTC	2	A	G
TCGA-30-1718	FLJ43860	ENST00000599122	p.M760V	c.2278A>G	Unverified	GAG	ATG	CTG	1	A	G
TCGA-24-1845	FMO4	ENST00000367749	p.T381T	c.1143A>G	Unverified	ggc	aca	gag	3	A	G
TCGA-13-1481	FRMD6	ENST00000395718	p.D538G	c.1613A>G	Verified	CTC	GAT	GAC	2	A	G
TCGA-29-1690	FRMPD3	XM_042978.4	p.P1421P	c.4263A>G	Unverified	ggc	cca	ggc	3	A	G
TCGA-29-1690	FRMPD3_EN	ENST00000276185	p.P1372P	c.4116A>G	Unverified	GGC	CCA	GGC	3	A	G
TCGA-13-0885	FSHB	ENST00000254122	p.K72E	c.214A>G	Verified	TTC	AAG	GAA	1	A	G
TCGA-23-1114	FUNDC2	ENST00000369498	p.G181G	c.543A>G	Unverified	ttc	gga	ggc	3	A	G
TCGA-30-1855	FYCO1	ENST00000296137	p.Q1111R	c.3332A>G	Unverified	TGC	CAG	GAG	2	A	G
TCGA-04-1338	GABARAPL2	ENST00000037243	p.N113D	c.337A>G	Verified	GAG	AAC	ACT	1	A	G
TCGA-24-0979	GABRA6	ENST00000274545	p.Q259R	c.776A>G	Verified	TCC	CAG	GTG	2	A	G
TCGA-61-2113	GADD45A	ENST00000370986	p.E100G	c.299A>G	Unverified	gcg	gag	ctc	2	A	G
TCGA-13-0887	GALNTL5	ENST00000392800	p.G359G	c.1077A>G	Verified	GTA	GGA	CAT	3	A	G
TCGA-25-1326	GALR1	ENST00000299727	p.H289R	c.866A>G	Unverified	GCC	CAC	TGC	2	A	G
TCGA-29-1705	GAPVD1	ENST00000394105	p.T354A	c.1060A>G	Unverified	ATG	ACT	GGC	1	A	G
TCGA-09-2050	GCLC	ENST00000229416	p.R307R	c.921A>G	Verified	GAT	AGA	ACT	3	A	G
TCGA-24-1563	GFM1	ENST00000486715	p.I238V	c.712A>G	Verified	GAG	ATT	CCA	1	A	G

TCGA-23-1116	GJD2	ENST00000290374	p.T44A	c.130A>G	Unverified	GAG	ACG	GTG	1	A	G
TCGA-29-1698	GK5	ENST00000392993	p.R144R	c.432A>G	Unverified	TGC	CGA	GTG	3	A	G
TCGA-29-1705	GLDN	ENST00000335449	p.G172G	c.516A>G	Unverified	AAA	GGA	GCA	3	A	G
TCGA-29-1768	GLI2	ENST00000361492	p.R146G	c.436A>G	Unverified	GCC	AGG	GGC	1	A	G
TCGA-24-1844	GMPS	ENST00000496455	p.P513P	c.1539A>G	Unverified	ctg	cca	att	3	A	G
TCGA-29-1776	GNAS_ENST	ENST00000371100	p.G578G	c.1734A>G	Unverified	AGC	GGA	GAC	3	A	G
TCGA-13-0792	GOLGA1	ENST00000373555	p.E662G	c.1985A>G	Verified	AAT	GAG	CTC	2	A	G
TCGA-23-1032	GPC5	ENST00000377067	p.E365G	c.1094A>G	Verified	AAA	GAG	AAG	2	A	G
TCGA-29-1761	GPNMB	ENST00000381990	p.R455R	c.1365A>G	Unverified	GTG	AGA	CGA	3	A	G
TCGA-61-2008	GPRIN3	ENST00000333209	p.I394V	c.1180A>G	Unverified	CAC	ATT	CAG	1	A	G
TCGA-13-0923	GRIA3	NM_000828.2	p.R328R	c.984A>G	Verified	cag	cga	gta	3	A	G
TCGA-13-0923	GRIA3_ENST	ENST00000264357	p.R328R	c.984A>G	Verified	CAG	CGA	GTA	3	A	G
TCGA-13-0762	GRID1	ENST00000327946	p.P560P	c.1680A>G	Verified	GCT	CCA	TTT	3	A	G
TCGA-13-0762	GRID1	ENST00000327946	p.P560P	c.1680A>G	Verified	GCT	CCA	TTT	3	A	G
TCGA-23-1032	GRM3	ENST00000361669	p.T725T	c.2175A>G	Verified	GAA	ACA	GTC	3	A	G
TCGA-23-1118	HCFC1	NM_005334.1	p.S645G	c.1933A>G	Verified	gcc	agt	ggg	1	A	G
TCGA-13-0906	HECW1	ENST00000395891	p.E477G	c.1430A>G	Verified	GGT	GAA	GCC	2	A	G
TCGA-13-0923	HERPUD2	ENST00000396081	p.E255G	c.764A>G	Verified	AAT	GAG	AAT	2	A	G
TCGA-13-0885	HHATL	ENST00000310417	p.E501G	c.1502A>G	Verified	AAA	GAG	AAG	2	A	G
TCGA-23-1124	HHEX	ENST00000282728	p.I250V	c.748A>G	Verified	gag	att	tca	1	A	G
TCGA-04-1347	HIST1H2BG	ENST00000244601	p.K17E	c.49A>G	Verified	AAG	AAG	GCT	1	A	G
TCGA-04-1338	HKDC1	ENST00000354624	p.I545V	c.1633A>G	Verified	AAG	ATC	AGA	1	A	G
TCGA-04-1362	HOXA2	ENST00000222718	p.S358G	c.1072A>G	Verified	GAC	AGC	TTA	1	A	G
TCGA-24-2280	HOXC9	ENST00000303450	p.R243R	c.729A>G	Unverified	AAT	CGA	AGG	3	A	G
TCGA-23-1110	HRC	ENST00000252825	p.S401G	c.1201A>G	Verified	AAG	AGT	GAT	1	A	G
TCGA-29-1785	HSD17B3	ENST00000375263	p.P228P	c.684A>G	Unverified	ACC	CCA	TAT	3	A	G
TCGA-29-1785	HSD17B3	ENST00000375263	p.P228P	c.684A>G	Unverified	ACC	CCA	TAT	3	A	G
TCGA-29-1693	HSPA6	NM_002155.3	p.Q158R	c.473A>G	Unverified	cgc	cag	gcc	2	A	G
TCGA-23-1022	HSPG2	ENST00000374695	p.M804V	c.2410A>G	Verified	GCC	ATG	AAG	1	A	G
TCGA-36-1577	HUWE1	XM_497119.1	p.H2999R	c.8996A>G	Unverified	atg	cat	gag	2	A	G
TCGA-24-1843	HYDIN	ENST00000321489	p.E282G	c.845A>G	Unverified	GGT	GAA	AAG	2	A	G
TCGA-24-1843	HYDIN_ENST	ENST00000316490_v65	p.E282G	c.845A>G	Unverified	GGT	GAA	AAG	2	A	G
TCGA-24-1843	HYDIN_ENST	ENST00000393550	p.E282G	c.845A>G	Unverified	GGT	GAA	AAG	2	A	G
TCGA-24-1843	HYDIN_ENST	ENST00000538248	p.E309G	c.926A>G	Unverified	GGT	GAA	AAG	2	A	G

TCGA-13-0900	ID1	ENST00000376112	p.D100G	c.299A>G	Verified	atc	gac	tac	2	A	G
TCGA-24-2267	IFIH1	ENST00000263642	p.K351E	c.1051A>G	Verified	AAG	AAA	AAA	1	A	G
TCGA-13-0913	IGSF1	ENST00000361420	p.E242G	c.725A>G	Verified	GGA	GAA	AGC	2	A	G
TCGA-09-2049	IGSF10	ENST00000282466	p.Q431R	c.1292A>G	Verified	ATG	CAA	GAC	2	A	G
TCGA-13-0885	IL7R	ENST00000303115	p.T410A	c.1228A>G	Verified	GGG	ACT	ACA	1	A	G
TCGA-24-1846	IL9R	ENST00000244174	p.M507V	c.1519A>G	Unverified	GGC	ATG	TTG	1	A	G
TCGA-29-1761	ILDR2	ENST00000271417	p.R326G	c.976A>G	Unverified	GCC	AGA	AGG	1	A	G
TCGA-61-1906	INO80	ENST00000361937	p.T682A	c.2044A>G	Unverified	GGG	ACC	CCA	1	A	G
TCGA-61-1906	INO80	ENST00000361937	p.T682A	c.2044A>G	Unverified	GGG	ACC	CCA	1	A	G
TCGA-61-1906	INOC1	ENST00000361937	p.T682A	c.2044A>G	Unverified	ggg	acc	cca	1	A	G
TCGA-61-1906	INOC1	ENST00000361937	p.T682A	c.2044A>G	Unverified	ggg	acc	cca	1	A	G
TCGA-13-0903	IQGAP1	ENST00000268182	p.Q954R	c.2861A>G	Verified	AAA	CAG	AAG	2	A	G
TCGA-25-1313	IRAK4_ENST	ENST00000448290	p.I53V	c.157A>G	Verified	CAC	ATA	AGG	1	A	G
TCGA-13-0919	IRS1	ENST00000305123	p.D731G	c.2192A>G	Unverified	ggt	gac	tac	2	A	G
TCGA-04-1343	ITGA8	ENST00000378076	p.T162A	c.484A>G	Verified	CCG	ACA	CCA	1	A	G
TCGA-13-1497	ITPR1	ENST00000456211	p.P1841P	c.5523A>G	Verified	GCC	CCA	TCA	3	A	G
TCGA-13-0793	ITPR2	ENST00000381340	p.I405V	c.1213A>G	Verified	CCC	ATA	GAC	1	A	G
TCGA-24-1616	IVD	ENST00000487418	p.T55A	c.163A>G	Verified	CAG	ACC	ATG	1	A	G
TCGA-61-2095	JAKMIP3	ENST00000298622	p.M705V	c.2113A>G	Unverified	AAG	ATG	GTG	1	A	G
TCGA-09-1665	KAL1	ENST00000262648	p.K98E	c.292A>G	Verified	AGG	AAA	CAC	1	A	G
TCGA-13-0793	KALRN	ENST00000240874	p.N1524D	c.4570A>G	Unverified	AAG	AAC	AAG	1	A	G
TCGA-24-1422	KANK4	ENST00000371153	p.A84A	c.252A>G	Verified	CCT	GCA	GCC	3	A	G
TCGA-04-1337	KCNA4	ENST00000328224	p.T200A	c.598A>G	Unverified	GAG	ACT	TTG	1	A	G
TCGA-29-1691	KCNC4	ENST00000369787	p.K493E	c.1477A>G	Unverified	CGG	AAG	AAG	1	A	G
TCGA-29-1691	KCNC4_ENST	ENST00000438661	p.K493E	c.1477A>G	Unverified	CGG	AAG	AAG	1	A	G
TCGA-24-1849	KCNH7	ENST00000332142	p.E480G	c.1439A>G	Unverified	GAA	GAA	GTG	2	A	G
TCGA-24-1849	KCNH7_ENS	ENST00000328032	p.E473G	c.1418A>G	Unverified	GAA	GAA	GTG	2	A	G
TCGA-09-1665	KDM3B	ENST00000314358	p.H83R	c.248A>G	Unverified	GTT	CAT	GCT	2	A	G
TCGA-13-1481	KHDRBS3	ENST00000355849	p.H306R	c.917A>G	Verified	GGA	CAT	GGA	2	A	G
TCGA-29-2427	KIAA0174	ENST00000329908	p.G142G	c.426A>G	Verified	ATT	GGA	ACT	3	A	G
TCGA-23-1031	KIAA0586	ENST00000354386	p.D924G	c.2771A>G	Verified	TTT	GAT	GAA	2	A	G
TCGA-23-1123	KIAA1404	ENST00000396105	p.R1180G	c.3538A>G	Verified	GTC	AGG	GTC	1	A	G
TCGA-23-1123	KIAA1404	ENST00000396105	p.R1180G	c.3538A>G	Verified	GTC	AGG	GTC	1	A	G
TCGA-13-0923	KIAA1715	ENST00000272748	p.R251R	c.753A>G	Verified	CCC	CGA	GAA	3	A	G

TCGA-24-1103	KIAA2022	ENST00000373468	p.E1468G	c.4403A>G	Verified	atg	gag	cga	2	A	G
TCGA-24-1616	KIDINS220	ENST00000256707	p.R604R	c.1812A>G	Verified	AAT	AGA	CTG	3	A	G
TCGA-10-0930	KIF18B	ENST00000339151	p.T322A	c.964A>G	Verified	CGC	ACA	GTG	1	A	G
TCGA-10-0930	KIF18B	ENST00000339151	p.T322A	c.964A>G	Verified	CGC	ACA	GTG	1	A	G
TCGA-13-0760	KIF4A	ENST00000374403	p.P502P	c.1506A>G	Verified	agt	cca	gag	3	A	G
TCGA-23-1120	KIT	ENST00000288135	p.T22A	c.64A>G	Unverified	cag	aca	ggc	1	A	G
TCGA-23-1124	KLF12	ENST00000377669	p.K335E	c.1003A>G	Verified	CTG	AAG	GCT	1	A	G
TCGA-13-0924	KLHL25	ENST00000337975	p.I568V	c.1702A>G	Verified	TGC	ATC	ACC	1	A	G
TCGA-25-2392	KLHL32	ENST00000369261	p.H338R	c.1013A>G	Verified	AGC	CAC	CAT	2	A	G
TCGA-13-0755	KRI1	ENST00000312962	p.R334G	c.1000A>G	Verified	CGC	AGA	AAG	1	A	G
TCGA-29-1711	KRT3	ENST00000417996	p.D340G	c.1019A>G	Unverified	TAC	GAC	GCT	2	A	G
TCGA-29-1711	KRT3	ENST00000417996	p.D340G	c.1019A>G	Unverified	TAC	GAC	GCT	2	A	G
TCGA-20-1685	KRTAP13-4	ENST00000334068	p.P149P	c.447A>G	Unverified	AGA	CCA	ATC	3	A	G
TCGA-20-1687	KRTAP5-5	ENST00000399676	p.G29G	c.87A>G	Unverified	TGT	GGA	GGC	3	A	G
TCGA-29-1705	LAMA3	ENST00000313654	p.S3118G	c.9352A>G	Unverified	aag	agc	ctc	1	A	G
TCGA-29-1705	LAMA3_ENS	ENST00000269217	p.S1509G	c.4525A>G	Unverified	AAG	AGC	CTC	1	A	G
TCGA-24-1844	LARP7	ENST00000324052	p.K221E	c.661A>G	Unverified	AAG	AAA	AAG	1	A	G
TCGA-13-0905	LAS1L	ENST00000374811	p.E556G	c.1667A>G	Verified	cag	gag	gag	2	A	G
TCGA-61-1733	LAX1	ENST00000442561	p.S303G	c.907A>G	Unverified	CCC	AGT	GGA	1	A	G
TCGA-61-1733	LAX1	ENST00000442561	p.S303G	c.907A>G	Unverified	CCC	AGT	GGA	1	A	G
TCGA-25-1326	LGR6	ENST00000367278	p.D536G	c.1607A>G	Verified	GAG	GAC	TCA	2	A	G
TCGA-04-1542	LHX9	ENST00000367387	p.R40G	c.118A>G	Verified	CGC	AGA	TCC	1	A	G
TCGA-23-1118	LILRA2	ENST00000251376	p.I179V	c.535A>G	Verified	GCC	ATC	TTC	1	A	G
TCGA-20-0990	LINGO2	ENST00000379992	p.D146G	c.437A>G	Verified	CTA	GAC	TAC	2	A	G
TCGA-24-2267	LINGO4	ENST00000368820	p.A425A	c.1275A>G	Unverified	ATT	GCA	GAG	3	A	G
TCGA-20-0991	LMNB1	ENST00000261366	p.E567G	c.1700A>G	Unverified	gag	gaa	gaa	2	A	G
TCGA-29-1691	LOC152485	ENST00000379448	p.Q8R	c.23A>G	Unverified	GAG	CAG	CCC	2	A	G
TCGA-24-1843	LOC652153	ENST00000316490	p.E282G	c.845A>G	Unverified	GGT	GAA	AAG	2	A	G
TCGA-24-1422	LOC652153	ENST00000316490	p.A1197A	c.3591A>G	Verified	CCA	GCA	ATC	3	A	G
TCGA-20-1683	LONRF3	ENST00000365713	p.R551R	c.1653A>G	Unverified	acg	aga	cag	3	A	G
TCGA-20-1683	LONRF3_EN	ENST00000371628	p.R592R	c.1776A>G	Unverified	ACG	AGA	CAG	3	A	G
TCGA-30-1855	LPAR1	ENST00000374430	p.D315G	c.944A>G	Unverified	CGC	GAC	AAA	2	A	G
TCGA-23-1110	LPIN1	ENST00000256720	p.Q654R	c.1961A>G	Verified	ACG	CAG	TAC	2	A	G
TCGA-24-1463	LRCH1	ENST00000389798	p.K244E	c.730A>G	Verified	AAC	AAA	GTG	1	A	G

TCGA-09-2050	LRCH2	NM_020871.2	p.S320S	c.960A>G	Verified	cca	tca	ttg	3	A	G
TCGA-29-1762	LRCH2	NM_020871.2	p.S327S	c.981A>G	Unverified	ccc	tca	cag	3	A	G
TCGA-29-1762	LRCH2_ENST	ENST00000317135	p.S327S	c.981A>G	Unverified	CCC	TCA	CAG	3	A	G
TCGA-23-1117	LRP2	ENST00000263816	p.E3395G	c.10184A>G	Verified	TTG	GAG	GGC	2	A	G
TCGA-23-1117	LRP2	ENST00000263816	p.E3395G	c.10184A>G	Verified	TTG	GAG	GGC	2	A	G
TCGA-13-0904	LRP5	ENST00000294304	p.E1094G	c.3281A>G	Unverified	ATC	GAA	CGC	2	A	G
TCGA-13-0792	LRRC15	ENST00000347624	p.I305V	c.913A>G	Verified	CAC	ATC	TCT	1	A	G
TCGA-23-1021	LRRC37B	ENST00000341671	p.T833T	c.2499A>G	Verified	GAA	ACA	CAT	3	A	G
TCGA-04-1337	MAGEC3	ENST00000298296	p.E375G	c.1124A>G	Unverified	gga	gaa	gat	2	A	G
TCGA-04-1337	MAGEC3_EN	ENST00000544766	p.E77G	c.230A>G	Unverified	CCA	GAA	GAT	2	A	G
TCGA-13-0792	MAGI1_ENS	ENST00000402939	p.K1288E	c.3862A>G	Verified	AGG	AAA	CCC	1	A	G
TCGA-24-1464	MAML2	ENST00000524717	p.M514V	c.1540A>G	Verified	tac	atg	tac	1	A	G
TCGA-09-2049	MAP4K2	ENST00000294066	p.R792R	c.2376A>G	Verified	CAC	AGA	GAC	3	A	G
TCGA-09-2049	MAP7D1	ENST00000373151	p.E796G	c.2387A>G	Verified	CAG	GAG	AAT	2	A	G
TCGA-13-1509	MAPKAP1	ENST00000350766	p.D376G	c.1127A>G	Verified	ATA	GAC	CCT	2	A	G
TCGA-13-0920	MAPRE3	ENST00000233121	p.T8T	c.24A>G	Verified	TCC	ACA	TCT	3	A	G
TCGA-29-1691	MASP2	ENST00000400897	p.D120G	c.359A>G	Unverified	TCC	GAC	TAC	2	A	G
TCGA-23-2077	MAST1	ENST00000251472	p.D872G	c.2615A>G	Verified	GGG	GAT	GCA	2	A	G
TCGA-13-0923	MCHR2	ENST00000369212	p.Q314R	c.941A>G	Verified	TTC	CAG	AAA	2	A	G
TCGA-29-1691	MCM10_EN	ENST00000361282	p.E264G	c.791A>G	Unverified	ACA	GAA	ATG	2	A	G
TCGA-13-0792	MCM2	ENST00000265056	p.M167V	c.499A>G	Verified	GAG	ATG	ATC	1	A	G
TCGA-09-2049	MDH1B	ENST00000374412	p.G373G	c.1119A>G	Verified	TTT	GGA	GGC	3	A	G
TCGA-13-1497	MDS2	ENST00000374555	p.S49G	c.145A>G	Verified	CTG	AGC	TGC	1	A	G
TCGA-10-0930	MED12L	ENST00000474524	p.K758E	c.2272A>G	Verified	AAG	AAG	AGC	1	A	G
TCGA-10-0930	MED12L	ENST00000474524	p.K758E	c.2272A>G	Verified	AAG	AAG	AGC	1	A	G
TCGA-30-1718	MED12L	ENST00000474524	p.E1616G	c.4847A>G	Unverified	TCA	GAA	AGT	2	A	G
TCGA-09-1674	MED13L	ENST00000281928	p.K81E	c.241A>G	Unverified	tgc	aaa	gag	1	A	G
TCGA-13-0923	MED17	ENST00000251871	p.E252G	c.755A>G	Verified	tta	gag	ggg	2	A	G
TCGA-61-1725	MEGF10	ENST00000274473	p.N678D	c.2032A>G	Unverified	AAC	AAC	GGA	1	A	G
TCGA-09-2044	MET	ENST00000318493	p.E221G	c.662A>G	Verified	AAG	GAA	ACG	2	A	G
TCGA-13-1489	MGAM_ENS	ENST00000549489	p.E883G	c.2648A>G	Verified	TTG	GAG	GTG	2	A	G
TCGA-23-1122	MLL	NM_005933.1	p.E1464G	c.4391A>G	Verified	aac	gag	cgc	2	A	G
TCGA-29-1701	MLLT10_EN	ENST00000307729	p.I912V	c.2734A>G	Unverified	GGC	ATT	GTA	1	A	G
TCGA-29-1701	MLLT10_EN	ENST00000377072_v68	p.I928V	c.2782A>G	Unverified	GGC	ATT	GTA	1	A	G

TCGA-13-0760	MLST8_ENS	ENST00000564088	p.Q312R	c.935A>G	Verified	CAC	CAG	AAG	2	A	G
TCGA-24-1417	MMP16	ENST00000286614	p.D89G	c.266A>G	Unverified	gtg	gac	aga	2	A	G
TCGA-29-1711	MMRN2	ENST00000372027	p.Q271R	c.812A>G	Unverified	CGC	CAG	GCC	2	A	G
TCGA-29-1711	MMRN2	ENST00000372027	p.Q271R	c.812A>G	Unverified	CGC	CAG	GCC	2	A	G
TCGA-29-1769	MTERFD1	ENST00000287025	p.S406S	c.1218A>G	Unverified	GCA	TCA	GTA	3	A	G
TCGA-61-1737	MUC15	ENST00000455601	p.D326G	c.977A>G	Unverified	GAT	GAC	ATA	2	A	G
TCGA-20-1686	MUC17	ENST00000306151	p.E2118G	c.6353A>G	Unverified	CCT	GAG	GCT	2	A	G
TCGA-30-1718	MVP	ENST00000395353	p.T256A	c.766A>G	Unverified	GAC	ACA	GAG	1	A	G
TCGA-04-1367	MYBL1	ENST00000522677	p.E716G	c.2147A>G	Verified	tgg	gaa	aca	2	A	G
TCGA-61-1910	MYH1	ENST00000226207	p.K1001E	c.3001A>G	Unverified	AAG	AAG	GCT	1	A	G
TCGA-04-1652	MYH1	ENST00000226207	p.E1409G	c.4226A>G	Unverified	GTA	GAA	GCT	2	A	G
TCGA-04-1652	MYH1	ENST00000226207	p.E1409G	c.4226A>G	Unverified	GTA	GAA	GCT	2	A	G
TCGA-29-1784	MYH1	ENST00000226207	p.E1735G	c.5204A>G	Unverified	CTG	GAG	ACA	2	A	G
TCGA-13-0795	MYH3	ENST00000226209	p.K568E	c.1702A>G	Verified	CCC	AAG	GTG	1	A	G
TCGA-29-1777	MYH7	ENST00000355349	p.E1507G	c.4520A>G	Unverified	CAG	GAG	GAG	2	A	G
TCGA-24-2267	MYO16	ENST00000357550	p.T978A	c.2932A>G	Unverified	ATG	ACA	GCT	1	A	G
TCGA-61-1904	MYOM2	ENST00000262113	p.T40A	c.118A>G	Unverified	TCC	ACC	CAG	1	A	G
TCGA-13-0887	MYST4	ENST00000287239	p.E1373G	c.4118A>G	Verified	GAA	GAA	GGA	2	A	G
TCGA-13-1510	MYT1	ENST00000328439	p.E576G	c.1727A>G	Unverified	AAG	GAG	CTG	2	A	G
TCGA-24-1845	MYT1L	ENST00000399161	p.E637G	c.1910A>G	Unverified	AAG	GAG	CTC	2	A	G
TCGA-20-1686	N6AMT1	ENST00000303775	p.D161G	c.482A>G	Unverified	CCA	GAT	CTC	2	A	G
TCGA-20-1685	NAA16	ENST00000379406	p.R295R	c.885A>G	Unverified	AGA	AGA	TTA	3	A	G
TCGA-04-1530	NAV1	ENST00000367296	p.K1538E	c.4612A>G	Unverified	CCC	AAG	CCG	1	A	G
TCGA-61-1910	NAV3	NM_014903.3	p.E803G	c.2408A>G	Unverified	agt	gag	aaa	2	A	G
TCGA-23-1123	NCF2	ENST00000367536	p.M150V	c.448A>G	Verified	AGC	ATG	AAG	1	A	G
TCGA-23-1123	NCF2	ENST00000367536	p.M150V	c.448A>G	Verified	AGC	ATG	AAG	1	A	G
TCGA-25-1326	NCKAP1	ENST00000360982	p.D1107G	c.3320A>G	Verified	ATG	GAT	CTT	2	A	G
TCGA-29-1761	NCKAP1	ENST00000360982	p.S968S	c.2904A>G	Unverified	TTA	TCA	TCA	3	A	G
TCGA-24-1423	NDUFV3	ENST00000354250	p.T120A	c.358A>G	Verified	AAG	ACT	TTG	1	A	G
TCGA-24-1847	NEK1	ENST00000507142	p.D1103G	c.3308A>G	Unverified	CAA	GAC	AAT	2	A	G
TCGA-24-1847	NEK1_ENST(	ENST00000507142	p.D1103G	c.3308A>G	Unverified	CAA	GAC	AAT	2	A	G
TCGA-04-1331	NEK8	SU_NEK8	p.A383A	c.1149A>G	Unverified	ggg	gca	gtg	3	A	G
TCGA-13-0919	NLGN4X	ENST00000381095	p.T335A	c.1003A>G	Unverified	gcc	acc	tac	1	A	G
TCGA-25-2391	NLRP4	ENST00000301295	p.H532R	c.1595A>G	Unverified	ATT	CAC	CAG	2	A	G

TCGA-29-1769	NLRP7	ENST00000448121	p.K808E	c.2422A>G	Unverified	TGC	AAG	GAC	1	A	G
TCGA-29-1769	NLRP7_ENST	ENST00000328092	p.K836E	c.2506A>G	Unverified	TGC	AAG	GAC	1	A	G
TCGA-13-0885	NOL6	ENST00000297990	p.A643A	c.1929A>G	Verified	GAT	GCA	CTT	3	A	G
TCGA-23-1022	NPAS2	ENST00000335681	p.Q136R	c.407A>G	Verified	GAA	CAA	GAA	2	A	G
TCGA-04-1361	NPAT	ENST00000278612	p.S1184S	c.3552A>G	Verified	AAT	TCA	AAA	3	A	G
TCGA-04-1361	NPAT	ENST00000278612	p.S1184S	c.3552A>G	Verified	AAT	TCA	AAA	3	A	G
TCGA-61-1910	NPY5R	ENST00000338566	p.H115R	c.344A>G	Unverified	TGC	CAT	ATT	2	A	G
TCGA-61-2095	NRAS	ENST00000369535	p.Q61R	c.182A>G	Verified	gga	caa	gaa	2	A	G
TCGA-04-1367	NRCAM	ENST00000351718	p.T690A	c.2068A>G	Verified	ACC	ACA	GCC	1	A	G
TCGA-09-2056	NT5C1A	ENST00000235628	p.N242D	c.724A>G	Verified	GAG	AAC	AAA	1	A	G
TCGA-61-1910	NUP155	ENST00000231498	p.R563R	c.1689A>G	Unverified	GAT	AGA	GAA	3	A	G
TCGA-13-0890	NUP210L	ENST00000368559	p.T400T	c.1200A>G	Verified	ATT	ACA	TAC	3	A	G
TCGA-25-2393	NXPH1	ENST00000405863	p.K117E	c.349A>G	Verified	AAG	AAA	ATG	1	A	G
TCGA-13-1497	ODC1	ENST00000234111	p.D10G	c.29A>G	Verified	TTT	GAC	TGC	2	A	G
TCGA-61-1740	ODZ2_ENST	ENST00000518659	p.D410G	c.1229A>G	Unverified	GCA	GAT	GGG	2	A	G
TCGA-61-1740	ODZ2_ENST	ENST00000519204	p.D289G	c.866A>G	Unverified	GCA	GAT	GGG	2	A	G
TCGA-23-1021	OR10J1_ENS	ENST00000328408	p.S89G	c.265A>G	Verified	CTG	AGT	CAG	1	A	G
TCGA-29-1775	OR2AP1	ENST00000321688	p.E264G	c.791A>G	Unverified	AAA	GAA	GGG	2	A	G
TCGA-25-2392	OR2F2	ENST00000408955	p.D4G	c.11A>G	Verified	ATA	GAT	AAC	2	A	G
TCGA-23-1031	OR2M2	ENST00000359682	p.Q6R	c.17A>G	Verified	AAT	CAG	ACC	2	A	G
TCGA-24-2024	OR4K13	ENST00000315693	p.T288T	c.864A>G	Verified	TAT	ACA	TTA	3	A	G
TCGA-23-1114	OR4K14	ENST00000305045	p.G97G	c.291A>G	Unverified	GGA	GGA	TGT	3	A	G
TCGA-24-1843	OR52M1	ENST00000360213	p.D123G	c.368A>G	Unverified	TTT	GAT	CGC	2	A	G
TCGA-23-1123	OR5AK2	ENST00000326855	p.I196V	c.586A>G	Verified	AAC	ATC	ATG	1	A	G
TCGA-23-1123	OR5AK2	ENST00000326855	p.I196V	c.586A>G	Verified	AAC	ATC	ATG	1	A	G
TCGA-29-1769	OR5K3	ENST00000383695	p.R306R	c.918A>G	Unverified	AAG	AGA	AAA	3	A	G
TCGA-24-2262	OR5V1	ENST00000377154	p.D296G	c.887A>G	Verified	AAG	GAC	ATC	2	A	G
TCGA-30-1891	OR6C75	ENST00000343399	p.I27V	c.79A>G	Unverified	TTC	ATA	TTT	1	A	G
TCGA-24-2289	OSBP	ENST00000263847	p.S492G	c.1474A>G	Unverified	acc	agt	aag	1	A	G
TCGA-23-1124	OTOP2	ENST00000331427	p.T364A	c.1090A>G	Verified	CCC	ACG	CGC	1	A	G
TCGA-09-2044	PADI3	ENST00000375460	p.M180V	c.538A>G	Verified	GAC	ATG	TCT	1	A	G
TCGA-09-2051	PALLD	ENST00000261509	p.R743R	c.2229A>G	Unverified	CAG	AGA	CTC	3	A	G
TCGA-24-2267	PAPLN	ENST00000340738	p.I968V	c.2902A>G	Unverified	ATC	ATA	GGG	1	A	G
TCGA-13-1507	PARP11	ENST00000228820	p.K242E	c.724A>G	Verified	TGC	AAA	GAT	1	A	G

TCGA-23-2078	PARP4	ENST00000381989	p.K429E	c.1285A>G	Verified	AGC	AAA	CTT	1	A	G
TCGA-61-1914	PCDHGA6_E	ENST00000517434	p.Q640R	c.1919A>G	Unverified	AAG	CAG	AGC	2	A	G
TCGA-24-1844	PCDHGA8	ENST00000398604	p.A582A	c.1746A>G	Unverified	TCC	GCA	GAG	3	A	G
TCGA-29-1691	PDE6A	ENST00000255266	p.K786E	c.2356A>G	Unverified	TAC	AAG	GAA	1	A	G
TCGA-24-2024	PDGFRA	ENST00000257290	p.Q431R	c.1292A>G	Verified	GGA	CAG	ACG	2	A	G
TCGA-13-0923	PDIA4	ENST00000286091	p.I107V	c.319A>G	Unverified	AAC	ATA	TTA	1	A	G
TCGA-61-1904	PDS5B	ENST00000315596	p.K627E	c.1879A>G	Unverified	AAC	AAA	TCA	1	A	G
TCGA-29-1784	PER3	ENST00000361923	p.K998E	c.2992A>G	Unverified	ATG	AAG	AAT	1	A	G
TCGA-61-2109	PFKL	NM_001002021.1	p.M782V	c.2344A>G	Unverified	cgc	atg	cca	1	A	G
TCGA-20-1685	PGLYRP4	ENST00000359650	p.R353R	c.1059A>G	Unverified	GCC	CGA	ACC	3	A	G
TCGA-61-1740	PGM2L1	ENST00000298198	p.I246V	c.736A>G	Unverified	AAG	ATC	TGT	1	A	G
TCGA-09-2044	PHF8	ENST00000338154	p.Q446R	c.1337A>G	Verified	caa	cag	aac	2	A	G
TCGA-29-1699	PIGT	ENST00000279036	p.D208G	c.623A>G	Unverified	GCA	GAT	CGC	2	A	G
TCGA-29-1699	PIGT_ENST0	ENST00000279036	p.D208G	c.623A>G	Unverified	GCA	GAT	CGC	2	A	G
TCGA-24-1845	PIK3R5	ENST00000447110	p.S297G	c.889A>G	Unverified	GAC	AGC	TTT	1	A	G
TCGA-13-1512	PKN1	ENST00000242783	p.E327G	c.980A>G	Verified	CTG	GAG	GTA	2	A	G
TCGA-61-2095	PKNOX1	ENST00000291547	p.D433G	c.1298A>G	Unverified	AGT	GAC	TCC	2	A	G
TCGA-10-0930	PLCD4	ENST00000450993	p.K435E	c.1303A>G	Verified	GGG	AAG	AAG	1	A	G
TCGA-10-0930	PLCD4	ENST00000450993	p.K435E	c.1303A>G	Verified	GGG	AAG	AAG	1	A	G
TCGA-61-1914	PLCH1	ENST00000334686	p.H341R	c.1022A>G	Unverified	CAT	CAT	GGT	2	A	G
TCGA-61-1914	PLCH1_ENST	ENST00000340059	p.H359R	c.1076A>G	Unverified	CAT	CAT	GGT	2	A	G
TCGA-23-1029	PLCL1	NM_006226.1	p.P636P	c.1908A>G	Unverified	ttc	cca	aag	3	A	G
TCGA-23-1029	PLCL1_ENST	ENST00000428675	p.P734P	c.2202A>G	Unverified	TTC	CCA	AAG	3	A	G
TCGA-29-1699	PLCXD2	ENST00000393934	p.Q74R	c.221A>G	Unverified	gac	caa	acc	2	A	G
TCGA-24-1846	PLIN2	ENST00000276914	p.K344E	c.1030A>G	Unverified	GCC	AAG	CAC	1	A	G
TCGA-29-1761	PLK2	NM_006622	p.I157V	c.469A>G	Unverified	tac	att	ctc	1	A	G
TCGA-24-2289	PLXNA4_ENS	ENST00000321063	p.T1767A	c.5299A>G	Unverified	ATC	ACA	GAC	1	A	G
TCGA-61-2008	PLXNB1	ENST00000296440	p.I886V	c.2656A>G	Unverified	CTC	ATC	CTC	1	A	G
TCGA-29-1763	PMEL	ENST00000552882	p.T414A	c.1240A>G	Unverified	ACC	ACA	GCT	1	A	G
TCGA-29-1699	PNLIPRP2	ENST00000298771	p.A399A	c.1197A>G	Unverified	GAT	GCA	AGT	3	A	G
TCGA-29-1699	PNLIPRP2_E	ENST00000537242	p.A399A	c.1197A>G	Unverified	GAT	GCA	AGT	3	A	G
TCGA-04-1361	PPIG	ENST00000260970	p.S527G	c.1579A>G	Verified	AAG	AGT	AAT	1	A	G
TCGA-04-1361	PPIG	ENST00000260970	p.S527G	c.1579A>G	Verified	AAG	AGT	AAT	1	A	G
TCGA-09-2050	PPP1R3A	NM_002711.2	p.S87G	c.259A>G	Verified	ccg	agt	gct	1	A	G

TCGA-23-2078	PQBP1	ENST00000376563	p.D129G	c.386A>G	Unverified	cac	gac	aag	2	A	G
TCGA-24-2280	PRDM15	ENST00000269844	p.R881R	c.2643A>G	Unverified	CGG	CGA	GTG	3	A	G
TCGA-13-0920	PRPS1L1	ENST00000506618	p.S58G	c.172A>G	Verified	ATC	ATC	ACC	1	A	G
TCGA-23-1116	PRTN3	ENST00000234347	p.Q78R	c.233A>G	Unverified	CCC	CAG	CGC	2	A	G
TCGA-13-0760	PSMB6	ENST00000270586	p.E103G	c.308A>G	Verified	ATT	GAA	CTG	2	A	G
TCGA-13-0884	PTCHD1	NM_173495.1	p.T474A	c.1420A>G	Verified	tac	acc	aag	1	A	G
TCGA-23-1031	PTMA	ENST00000409115	p.S2S	c.6A>G	Unverified	ATG	TCA	GAC	3	A	G
TCGA-13-0795	PTPN5	ENST00000358540	p.K329E	c.985A>G	Verified	TAC	AAA	ACC	1	A	G
TCGA-13-0919	PTPRS	ENST00000357368	p.T996A	c.2986A>G	Unverified	CTC	ACG	CTG	1	A	G
TCGA-29-1705	PTPRS	ENST00000357368	p.M16V	c.46A>G	Unverified	CCC	ATG	GGC	1	A	G
TCGA-24-1104	PTPRT	NM_133170.2	p.T1314A	c.3940A>G	Verified	aag	acc	tcc	1	A	G
TCGA-04-1652	PTPRT	NM_133170.2	p.E548G	c.1643A>G	Unverified	aat	gaa	acc	2	A	G
TCGA-04-1652	PTPRT	NM_133170.2	p.E548G	c.1643A>G	Unverified	aat	gaa	acc	2	A	G
TCGA-24-1469	PUM1	ENST00000257075	p.S657G	c.1969A>G	Verified	CAG	AGC	AGC	1	A	G
TCGA-29-1703	QARS	ENST00000306125	p.D170G	c.509A>G	Unverified	GTG	GAC	ATG	2	A	G
TCGA-23-1123	QRICH1	ENST00000395443	p.P698P	c.2094A>G	Verified	AAT	CCA	TTG	3	A	G
TCGA-23-1123	QRICH1	ENST00000395443	p.P698P	c.2094A>G	Verified	AAT	CCA	TTG	3	A	G
TCGA-61-2095	QRICH2	ENST00000262765	p.Q197R	c.590A>G	Unverified	GTT	CAA	CAG	2	A	G
TCGA-25-2392	QSOX2	ENST00000358701	p.E541G	c.1622A>G	Unverified	CAT	GAG	GAA	2	A	G
TCGA-04-1338	RAB41	ENST00000276066	p.S197S	c.591A>G	Verified	ACT	TCA	CCT	3	A	G
TCGA-29-1769	RAD21	ENST00000297338	p.T199A	c.595A>G	Unverified	agc	acc	agc	1	A	G
TCGA-13-0906	RAPGEF1	ENST00000372190	p.K940E	c.2818A>G	Verified	cgg	aag	ctg	1	A	G
TCGA-24-2290	RBM12	ENST00000374104	p.G399G	c.1197A>G	Verified	TCT	GGA	CAA	3	A	G
TCGA-24-2290	RBM12	ENST00000374104	p.G399G	c.1197A>G	Verified	TCT	GGA	CAA	3	A	G
TCGA-23-1116	RBM38	ENST00000356208	p.E89G	c.266A>G	Unverified	GCT	GAG	AGG	2	A	G
TCGA-24-1104	RGL1	ENST00000304685	p.T92T	c.276A>G	Verified	CAC	ACA	GTC	3	A	G
TCGA-13-1509	RHBDD1	ENST00000392062	p.D262G	c.785A>G	Verified	TAT	GAC	ACG	2	A	G
TCGA-23-1021	RIF1	ENST00000444746	p.N1387D	c.4159A>G	Verified	gag	aat	aca	1	A	G
TCGA-24-1464	RIOK1	ENST00000379834	p.D526G	c.1577A>G	Verified	ACG	GAC	CCT	2	A	G
TCGA-13-1496	RLF	ENST00000372771	p.K1073E	c.3217A>G	Verified	TTG	AAA	AAC	1	A	G
TCGA-24-1469	RNASEL	ENST00000367559	p.E676G	c.2027A>G	Verified	GAA	GAA	AAG	2	A	G
TCGA-61-2113	ROS1	ENST00000368508	p.I1399V	c.4195A>G	Verified	ATC	ATC	ACA	1	A	G
TCGA-29-1701	RP11-231C1	ENST00000524087	p.H502R	c.1505A>G	Unverified	GAG	CAT	CTG	2	A	G
TCGA-29-1701	RP11-231C1	ENST00000524087_v68	p.H502R	c.1505A>G	Unverified	GAG	CAT	CTG	2	A	G

TCGA-24-1417	RPGRIP1_EN	ENST00000400017	p.M41V	c.121A>G	Verified	AGG	ATG	AAC	1	A	G
TCGA-25-2392	RPS6KA3	ENST00000379565	p.R337R	c.1011A>G	Verified	TAT	AGA	AGA	3	A	G
TCGA-24-1845	RPUSD2	ENST00000315616	p.H214R	c.641A>G	Unverified	AGG	CAT	GAG	2	A	G
TCGA-04-1356	RSF1	ENST00000308488	p.D1367G	c.4100A>G	Verified	GTG	GAC	TTA	2	A	G
TCGA-04-1343	RUNDC2A	ENST00000268271	p.N124D	c.370A>G	Verified	CTC	AAC	GAA	1	A	G
TCGA-24-1845	RUNDC2A	ENST00000268271	p.Q338R	c.1013A>G	Unverified	TAC	CAG	AAG	2	A	G
TCGA-29-1785	RUVBL2_EN	ENST00000221413	p.I348V	c.1042A>G	Unverified	CCC	ATA	GAC	1	A	G
TCGA-29-1785	RUVBL2_EN	ENST00000221413	p.I348V	c.1042A>G	Unverified	CCC	ATA	GAC	1	A	G
TCGA-13-0900	S100A7L2	ENST00000368724	p.G22G	c.66A>G	Verified	AGT	GGA	GAT	3	A	G
TCGA-23-1022	SACS	NM_014363.3	p.H1235R	c.3704A>G	Verified	ata	cat	cat	2	A	G
TCGA-25-2393	SCN2A	ENST00000375427	p.E518G	c.1553A>G	Verified	GAA	GAG	AAA	2	A	G
TCGA-23-1120	SCYL1_ENST	ENST00000270176	p.E690G	c.2069A>G	Unverified	CCA	GAG	TCC	2	A	G
TCGA-24-1435	SEC14L3	ENST00000215812	p.T246A	c.736A>G	Verified	CTG	ACT	GAC	1	A	G
TCGA-10-0930	SEC14L5	ENST00000251170	p.E441G	c.1322A>G	Unverified	AAT	GAG	AAC	2	A	G
TCGA-10-0930	SEC14L5	ENST00000251170	p.E441G	c.1322A>G	Unverified	AAT	GAG	AAC	2	A	G
TCGA-13-1488	SECISBP2	ENST00000375807	p.R256R	c.768A>G	Verified	CTA	AGA	GAA	3	A	G
TCGA-29-1764	SEL1L2	ENST00000284951	p.P665P	c.1995A>G	Unverified	GGA	CCA	CAC	3	A	G
TCGA-29-1705	SERPINB4	ENST00000341074	p.S36S	c.108A>G	Unverified	aca	tca	gca	3	A	G
TCGA-13-0920	SF3B2	ENST00000322535	p.T599T	c.1797A>G	Verified	GAG	ACA	CGA	3	A	G
TCGA-61-2012	SGK1	ENST00000237305	p.K400E	c.1198A>G	Unverified	ggc	aag	tcc	1	A	G
TCGA-13-1488	SH3BGRL	ENST00000373212	p.G33G	c.99A>G	Verified	ata	gga	ttt	3	A	G
TCGA-23-1114	SIDT1	ENST00000264852	p.I359V	c.1075A>G	Unverified	CCC	ATT	GCT	1	A	G
TCGA-29-1764	SIPA1L1	ENST00000555818	p.T1794T	c.5382A>G	Unverified	TTC	ACA	GAA	3	A	G
TCGA-29-1691	SIPA1L2	ENST00000366630	p.K1201E	c.3601A>G	Unverified	CAG	AAA	GAT	1	A	G
TCGA-61-1907	SKIV2L2	ENST00000230640	p.G724G	c.2172A>G	Unverified	AAA	GGA	GAG	3	A	G
TCGA-24-1846	SLC23A3	ENST00000295738	p.G78G	c.234A>G	Unverified	GGA	GGA	CTC	3	A	G
TCGA-24-1846	SLC23A3_EN	ENST00000455516	p.G78G	c.234A>G	Unverified	GGA	GGA	CTC	3	A	G
TCGA-23-1809	SLC25A15	ENST00000426521	p.E206G	c.617A>G	Unverified	gat	gaa	tta	2	A	G
TCGA-25-1313	SLC27A4	ENST00000300456	p.E640G	c.1919A>G	Unverified	GGC	GAG	GAG	2	A	G
TCGA-23-1021	SLC2A3	ENST00000075120	p.P8P	c.24A>G	Verified	ACC	CCA	GCT	3	A	G
TCGA-24-2288	SLC39A12	ENST00000377374	p.K474E	c.1420A>G	Unverified	GAC	AAG	AAA	1	A	G
TCGA-24-2262	SLC6A2	ENST00000379906	p.R587G	c.1759A>G	Verified	GAG	AGA	CTG	1	A	G
TCGA-24-1846	SLC6A4	ENST00000261707	p.K490E	c.1468A>G	Unverified	GTG	AAG	CTG	1	A	G
TCGA-24-2267	SNAI2	ENST00000020945	p.N134D	c.400A>G	Verified	tgc	aat	aag	1	A	G

TCGA-13-1499	SNRPA	ENST00000243563	p.T100A	c.298A>G	Verified	GGC	ACC	TTC	1	A	G
TCGA-13-0890	SORBS2	ENST00000284776	p.E939G	c.2816A>G	Verified	gga	gaa	atc	2	A	G
TCGA-13-0916	SOX6	ENST00000316399	p.T739A	c.2215A>G	Verified	ATG	ACA	TCT	1	A	G
TCGA-23-1122	SP140	ENST00000373645	p.G13G	c.39A>G	Unverified	AGT	GGA	GAC	3	A	G
TCGA-24-1103	SPAG17	ENST00000336338	p.T2206T	c.6618A>G	Verified	TCT	ACA	ATT	3	A	G
TCGA-61-2113	SPAM1	ENST00000340011	p.K290E	c.868A>G	Verified	TCC	AAA	ATA	1	A	G
TCGA-25-1326	SPATA16	ENST00000351008	p.T232A	c.694A>G	Verified	GAG	ACA	AAG	1	A	G
TCGA-13-1499	SPATA21	ENST00000335496	p.Q401R	c.1202A>G	Verified	GCC	CAG	GTG	2	A	G
TCGA-61-2113	SPHK1	ENST00000323374	p.T243A	c.727A>G	Unverified	CAC	ACG	GCT	1	A	G
TCGA-24-1847	SPRED2	ENST00000356388	p.D141G	c.422A>G	Unverified	GGC	GAT	GAT	2	A	G
TCGA-29-1777	SPTLC1	ENST00000262554	p.E19G	c.56A>G	Unverified	TAC	GAG	GCT	2	A	G
TCGA-13-1488	SSH1	ENST00000326495	p.E25G	c.74A>G	Verified	TTG	GAG	GCT	2	A	G
TCGA-61-2109	ST6GAL2	ENST00000361686	p.M445V	c.1333A>G	Unverified	ATG	ATG	TCC	1	A	G
TCGA-09-2051	STAB1	ENST00000321725	p.H430R	c.1289A>G	Unverified	CAG	CAC	ATC	2	A	G
TCGA-30-1718	STC1	ENST00000290271	p.D200G	c.599A>G	Unverified	ACA	GAC	CAC	2	A	G
TCGA-29-1703	STC2	ENST00000265087	p.K104E	c.310A>G	Unverified	ATC	AAA	GAC	1	A	G
TCGA-13-0760	STIP1	ENST00000305218	p.R77R	c.231A>G	Verified	TCA	CGA	AAA	3	A	G
TCGA-29-1695	STK31	ENST00000355870	p.E929G	c.2786A>G	Unverified	TTT	GAG	ATA	2	A	G
TCGA-61-1914	STS	ENST00000217961	p.Q571R	c.1712A>G	Unverified	tgc	cag	tgt	2	A	G
TCGA-13-0795	STX3	ENST00000337979	p.N173D	c.517A>G	Verified	GGC	AAC	CCG	1	A	G
TCGA-30-1891	SULT1A1	ENST00000314752	p.T107A	c.319A>G	Unverified	AAG	ACA	CAC	1	A	G
TCGA-04-1361	SULT1C2	ENST00000326853	p.E169G	c.506A>G	Verified	GAA	GAG	TAT	2	A	G
TCGA-04-1361	SULT1C2	ENST00000326853	p.E169G	c.506A>G	Verified	GAA	GAG	TAT	2	A	G
TCGA-09-2050	SUPT3H	ENST00000371460	p.N142D	c.424A>G	Verified	GCG	AAC	AAA	1	A	G
TCGA-13-0904	SUPT3H	ENST00000371460	p.R144R	c.432A>G	Verified	AAA	AGA	CAA	3	A	G
TCGA-61-1904	SVEP1	ENST00000401783	p.G2688G	c.8064A>G	Unverified	CCA	GGA	TAT	3	A	G
TCGA-24-1616	SYN1	ENST00000340666	p.I363V	c.1087A>G	Verified	gag	att	ttt	1	A	G
TCGA-61-2008	SYNE1	ENST00000265368	p.H6693R	c.20078A>G	Verified	TCC	CAT	CTG	2	A	G
TCGA-61-2008	SYNE1_ENST	ENST00000265368	p.H6693R	c.20078A>G	Verified	TCC	CAT	CTG	2	A	G
TCGA-24-1844	SYNE2	ENST00000358025	p.D2590G	c.7769A>G	Unverified	ACT	GAC	ATG	2	A	G
TCGA-61-2094	TACC3	ENST00000313288	p.D412G	c.1235A>G	Unverified	GAT	GAC	CCA	2	A	G
TCGA-13-1408	TAF12	ENST00000263974	p.Q123R	c.368A>G	Verified	CGC	CAG	TGG	2	A	G
TCGA-24-2035	TAF4	ENST00000252996	p.T1051A	c.3151A>G	Verified	TTC	ACG	CGA	1	A	G
TCGA-24-2267	TBC1D10A	ENST00000215790	p.H101R	c.302A>G	Verified	AAG	CAC	AAA	2	A	G

TCGA-29-1693	TBC1D24	ENST00000567020	p.K84E	c.250A>G	Unverified	GGC	AAG	CAC	1	A	G
TCGA-13-1488	TBC1D8	ENST00000409318	p.Q72R	c.215A>G	Verified	TCC	CAG	GTT	2	A	G
TCGA-23-1120	TBX20_ENST	ENST00000408931	p.I402V	c.1204A>G	Unverified	GCC	ATT	GCC	1	A	G
TCGA-29-1763	TCEB3	ENST00000374536	p.E83G	c.248A>G	Unverified	GCT	GAG	CCT	2	A	G
TCGA-29-1764	TECTA	ENST00000392793	p.K757E	c.2269A>G	Unverified	AAG	AAG	AAG	1	A	G
TCGA-13-0755	TGM6	ENST00000202625	p.I437V	c.1309A>G	Unverified	GAC	ATC	ACT	1	A	G
TCGA-23-1120	THADA	ENST00000405975	p.T1696T	c.5088A>G	Verified	cct	aca	gag	3	A	G
TCGA-13-0792	THAP10	ENST00000249861	p.R218R	c.654A>G	Verified	TCT	AGA	ACT	3	A	G
TCGA-13-0714	TIMELESS	ENST00000553532	p.H730R	c.2189A>G	Verified	CTG	CAC	CGG	2	A	G
TCGA-13-0760	TLR3	ENST00000296795	p.T262A	c.784A>G	Verified	tcc	acc	acc	1	A	G
TCGA-13-0791	TLR6	ENST00000381950	p.E371G	c.1112A>G	Verified	TTT	GAA	AAA	2	A	G
TCGA-30-1857	TM4SF4	ENST00000305354	p.I60V	c.178A>G	Unverified	ATG	ATC	TTC	1	A	G
TCGA-23-1110	TNS1	ENST00000171887	p.Q1513R	c.4538A>G	Verified	CAG	CAG	AAT	2	A	G
TCGA-23-1022	TP53	ENST00000269305	p.K164E	c.490A>G	Verified	TAC	AAG	CAG	1	A	G
TCGA-29-1701	TP53	ENST00000269305	p.?	c.994-2A>G	Unverified	CAG	ATC	CGT	1	A	G
TCGA-09-0369	TP53	ENST00000269305	p.H193R	c.578A>G	Verified	CAG	CAT	CTT	2	A	G
TCGA-09-0369	TP53	ENST00000269305	p.H193R	c.578A>G	Verified	CAG	CAT	CTT	2	A	G
TCGA-13-0762	TP53	ENST00000269305	p.H193R	c.578A>G	Verified	CAG	CAT	CTT	2	A	G
TCGA-13-0762	TP53	ENST00000269305	p.H193R	c.578A>G	Verified	CAG	CAT	CTT	2	A	G
TCGA-29-1701	TP53_ENST0	ENST00000269305	p.?	c.994-2A>G	Unverified	CAG	ATC	CGT	1	A	G
TCGA-24-1463	TPRKB	ENST00000272424	p.R30R	c.90A>G	Verified	TTG	AGA	AGA	3	A	G
TCGA-29-2427	TRIM36	ENST00000282369	p.R501R	c.1503A>G	Verified	AGC	AGA	GAA	3	A	G
TCGA-61-1737	TRIO	ENST00000344204	p.S1642S	c.4926A>G	Unverified	gcc	tca	cgg	3	A	G
TCGA-23-1123	TROAP	ENST00000257909	p.E573G	c.1718A>G	Verified	CAG	GAA	CAG	2	A	G
TCGA-23-1123	TROAP	ENST00000257909	p.E573G	c.1718A>G	Verified	CAG	GAA	CAG	2	A	G
TCGA-09-1674	TRPM1	ENST00000397795	p.K590E	c.1768A>G	Unverified	AAG	AAG	AAA	1	A	G
TCGA-13-1510	TRPM6	ENST00000360774	p.K1631E	c.4891A>G	Unverified	tcc	aaa	ttt	1	A	G
TCGA-61-1740	TRPV4	ENST00000261740	p.D425G	c.1274A>G	Unverified	CTG	GAC	ACG	2	A	G
TCGA-04-1638	TSC2	ENST00000219476	p.M260V	c.778A>G	Unverified	CTG	ATG	CGG	1	A	G
TCGA-04-1638	TSC2_ENST0	ENST00000219476	p.M260V	c.778A>G	Unverified	CTG	ATG	CGG	1	A	G
TCGA-24-1423	TTC35	ENST00000220853	p.I141V	c.421A>G	Verified	GCC	ATT	CGG	1	A	G
TCGA-13-1509	TTLL8	ENST00000266182	p.I288V	c.862A>G	Verified	TGC	ATC	TTC	1	A	G
TCGA-13-0893	TTN	NM_003319	p.K14359E	c.43075A>G	Verified	aag	aaa	agt	1	A	G
TCGA-29-1770	TTN	NM_003319	p.P15867P	c.47601A>G	Unverified	gag	cca	atc	3	A	G

TCGA-25-1313	TTN_ENST0(	ENST00000342175	p.T5539T	c.16617A>G	Unverified	ATC	ACA	CCT	3	A	G
TCGA-29-1770	TTN_ENST0(	ENST00000342175	p.P16059P	c.48177A>G	Unverified	GAG	CCA	ATC	3	A	G
TCGA-61-1733	TTN_ENST0(	ENST00000342992	p.S4474G	c.13420A>G	Unverified	AGC	AGC	ATC	1	A	G
TCGA-61-1733	TTN_ENST0(	ENST00000342992	p.S4474G	c.13420A>G	Unverified	AGC	AGC	ATC	1	A	G
TCGA-30-1855	TTN_ENST0(	ENST00000342992	p.E5139G	c.15416A>G	Unverified	CAA	GAA	CCA	2	A	G
TCGA-29-1770	TTN_ENST0(	ENST00000342992	p.P22364P	c.67092A>G	Unverified	GAG	CCA	ATC	3	A	G
TCGA-13-0893	TTN_ENST0(	ENST00000356127	p.K20854E	c.62560A>G	Verified	AAG	AAA	AGT	1	A	G
TCGA-25-1313	TTN_ENST0(	ENST00000356127	p.T11844T	c.35532A>G	Unverified	ATC	ACA	CCT	3	A	G
TCGA-29-1770	TTN_ENST0(	ENST00000356127	p.P22362P	c.67086A>G	Unverified	GAG	CCA	ATC	3	A	G
TCGA-29-1770	TTN_ENST0(	ENST00000359218	p.P15992P	c.47976A>G	Unverified	GAG	CCA	ATC	3	A	G
TCGA-09-2050	TULP4	ENST00000367097	p.K541E	c.1621A>G	Verified	CCC	AAA	CTC	1	A	G
TCGA-24-2035	TUSC3	ENST00000382020	p.?	c.799-2A>G	Verified	GTG	AGC	TAC	1	A	G
TCGA-30-1856	TYR	ENST00000263321	p.A493A	c.1479A>G	Unverified	CTG	GCA	GGG	3	A	G
TCGA-23-1124	UACA	ENST00000379983	p.D1281G	c.3842A>G	Verified	TGT	GAT	AAG	2	A	G
TCGA-23-1022	UGGT1	ENST00000259253	p.N416D	c.1246A>G	Verified	AGG	AAT	GAA	1	A	G
TCGA-25-1313	UNC45A	ENST00000418476	p.T660A	c.1978A>G	Verified	CTG	ACC	AGT	1	A	G
TCGA-30-1855	UNC5D	ENST00000287272	p.Q635R	c.1904A>G	Unverified	ACA	CAG	CAG	2	A	G
TCGA-30-1855	UNC5D_ENS	ENST00000404895	p.Q640R	c.1919A>G	Unverified	ACA	CAG	CAG	2	A	G
TCGA-61-1740	USP2	ENST00000260187	p.H501R	c.1502A>G	Unverified	CTC	CAT	CTG	2	A	G
TCGA-24-1422	USP24	XM_371254.3	p.E944G	c.2831A>G	Verified	gat	gaa	caa	2	A	G
TCGA-24-1431	USP34	ENST00000398571	p.R2950G	c.8848A>G	Verified	TTC	AGA	ATA	1	A	G
TCGA-24-1474	USP36	ENST00000312010	p.T827A	c.2479A>G	Unverified	GAG	ACG	CGC	1	A	G
TCGA-24-1849	USP36	ENST00000312010	p.D270G	c.809A>G	Unverified	TTG	GAC	GTC	2	A	G
TCGA-24-1474	USP36_ENS	ENST00000312010	p.T827A	c.2479A>G	Unverified	GAG	ACG	CGC	1	A	G
TCGA-24-1849	USP36_ENS	ENST00000312010	p.D270G	c.809A>G	Unverified	TTG	GAC	GTC	2	A	G
TCGA-61-1900	USP9X	OTTHUMT00000056248.	p.M345V	c.1033A>G	Unverified	aag	atg	aat	1	A	G
TCGA-61-1900	USP9X_ENS	ENST00000324545	p.M352V	c.1054A>G	Unverified	AAG	ATG	AAT	1	A	G
TCGA-24-1844	UTP20	ENST00000261637	p.E314G	c.941A>G	Unverified	TGT	GAA	AGT	2	A	G
TCGA-23-1110	UTRN	ENST00000367545	p.M721V	c.2161A>G	Verified	AAG	ATG	CAA	1	A	G
TCGA-24-2288	VCAN	ENST00000265077	p.S1909S	c.5727A>G	Verified	ATT	TCA	GAG	3	A	G
TCGA-09-2049	VDAC2	ENST00000332211	p.D41G	c.122A>G	Verified	CTG	GAT	GTG	2	A	G
TCGA-04-1542	VPS13B	ENST00000358544	p.H303R	c.908A>G	Verified	tgt	cat	aat	2	A	G
TCGA-09-2044	VPS16	ENST00000380445	p.D739G	c.2216A>G	Verified	GAA	GAT	TGG	2	A	G
TCGA-04-1367	VPS39	ENST00000318006	p.M125V	c.373A>G	Verified	cgg	atg	tgt	1	A	G

TCGA-24-2267	VPS8	ENST00000437079	p.R501G	c.1501A>G	Verified	TGG	AGA	GAG	1	A	G
TCGA-24-1464	WBP11	ENST00000261167	p.D263G	c.788A>G	Verified	ATG	GAT	CAA	2	A	G
TCGA-29-1701	WDFY3	ENST00000295888	p.R501G	c.1501A>G	Unverified	TTC	AGG	GAG	1	A	G
TCGA-04-1342	WDR36	ENST00000506538	p.M586V	c.1756A>G	Verified	ATC	ATG	TTG	1	A	G
TCGA-30-1891	WNK1	NM_018979	p.K1966E	c.5896A>G	Unverified	gac	aag	atc	1	A	G
TCGA-23-1032	WSB2	ENST00000315436	p.P390P	c.1170A>G	Verified	CTG	CCA	ATC	3	A	G
TCGA-13-0893	XIRP2	ENST00000295237	p.E3260G	c.9779A>G	Verified	CAA	GAG	GAA	2	A	G
TCGA-24-1417	XIRP2	ENST00000295237	p.P2292P	c.6876A>G	Verified	CCT	CCA	TCT	3	A	G
TCGA-20-0990	ZBTB38	ENST00000514251	p.N921D	c.2761A>G	Verified	TAC	AAC	TAC	1	A	G
TCGA-61-1740	ZBTB7B	ENST00000292176	p.D179G	c.536A>G	Unverified	GAA	GAC	AGT	2	A	G
TCGA-13-1507	ZC3H6	ENST00000409871	p.D154G	c.461A>G	Verified	GAT	GAC	AAC	2	A	G
TCGA-23-1118	ZC3H6	ENST00000409871	p.D696G	c.2087A>G	Unverified	GAA	GAT	GAT	2	A	G
TCGA-09-2056	ZC3H7B	ENST00000352645	p.T801A	c.2401A>G	Unverified	CAG	ACC	TAT	1	A	G
TCGA-25-2042	ZCCHC14	ENST00000268616	p.D176G	c.527A>G	Unverified	CTG	GAC	TCA	2	A	G
TCGA-23-1032	ZEB2	ENST00000303660	p.D86G	c.257A>G	Verified	gaa	gat	gaa	2	A	G
TCGA-09-2050	ZFP112	ENST00000337401	p.T828T	c.2484A>G	Verified	CAC	ACA	GGA	3	A	G
TCGA-13-1499	ZFPM2	ENST00000407775	p.E77G	c.230A>G	Verified	GCA	GAA	TCA	2	A	G
TCGA-13-1512	ZFYVE27	ENST00000356257	p.E240G	c.719A>G	Verified	GAA	GAG	CAT	2	A	G
TCGA-20-0991	ZMYND10	ENST00000231749	p.H36R	c.107A>G	Verified	CAG	CAT	GAG	2	A	G
TCGA-25-2400	ZNF142	ENST00000396788	p.D88G	c.263A>G	Unverified	TCA	GAC	ACC	2	A	G
TCGA-04-1362	ZNF229	ENST00000291187	p.G261G	c.783A>G	Verified	CCT	GGA	GAG	3	A	G
TCGA-24-2288	ZNF248	ENST00000395867	p.E461G	c.1382A>G	Unverified	GGG	GAG	AAG	2	A	G
TCGA-04-1649	ZNF280B	ENST00000360412	p.T147T	c.441A>G	Unverified	ATT	ACA	TTC	3	A	G
TCGA-61-1915	ZNF337	ENST00000376436	p.E164G	c.491A>G	Unverified	ACA	GAA	ATA	2	A	G
TCGA-04-1347	ZNF398	ENST00000475153	p.A39A	c.117A>G	Verified	ACA	GCA	GCT	3	A	G
TCGA-13-0760	ZNF425	ENST00000378061	p.H516R	c.1547A>G	Verified	CAG	CAC	CTG	2	A	G
TCGA-29-1776	ZNF462	ENST00000277225	p.H1977R	c.5930A>G	Unverified	GTG	CAC	CCA	2	A	G
TCGA-13-0760	ZNF470	ENST00000330619	p.D221G	c.662A>G	Verified	CAA	GAC	CGT	2	A	G
TCGA-09-2051	ZNF513	ENST00000323703	p.G345G	c.1035A>G	Unverified	GCT	GGA	GGG	3	A	G
TCGA-04-1638	ZNF516	ENST00000217537	p.E692G	c.2075A>G	Unverified	GTG	GAG	TTC	2	A	G
TCGA-13-0913	ZNF552	ENST00000391701	p.E386G	c.1157A>G	Unverified	TGT	GAA	AAA	2	A	G
TCGA-13-1509	ZNF577	ENST00000301399	p.E211G	c.632A>G	Verified	GGA	GAG	AAG	2	A	G
TCGA-13-0923	ZNF586	ENST00000396154	p.G80G	c.240A>G	Verified	CAG	GGA	GGT	3	A	G
TCGA-30-1857	ZNF594	ENST00000399604	p.S580G	c.1738A>G	Unverified	GGC	AGC	TCA	1	A	G

TCGA-13-0791	ZNF599	ENST00000329285	p.E567G	c.1700A>G	Verified	AAT	GAA	TGT	2	A	G
TCGA-29-1761	ZNF610	ENST00000327920	p.H446R	c.1337A>G	Unverified	CGA	CAT	CGG	2	A	G
TCGA-61-1740	ZNF626_ENS	ENST00000305570	p.K92E	c.274A>G	Unverified	ATG	AAA	GAT	1	A	G
TCGA-29-1693	ZNF630	ENST00000409324	p.G455G	c.1365A>G	Unverified	aca	gga	gaa	3	A	G
TCGA-24-2267	ZNF655	ENST00000394163	p.E409G	c.1226A>G	Verified	CAT	GAA	TGT	2	A	G
TCGA-13-0755	ZNF70	ENST00000341976	p.K287E	c.859A>G	Verified	GGG	AAA	GCC	1	A	G
TCGA-61-2109	ZNF708	ENST00000356929	p.E57G	c.170A>G	Unverified	CTG	GAG	CAA	2	A	G
TCGA-13-0793	ZNF8	ENST00000196548	p.R572G	c.1714A>G	Verified	ATC	AGA	GAA	1	A	G
TCGA-29-1691	ZNF827	ENST00000379448	p.Q8R	c.23A>G	Unverified	GAG	CAG	CCC	2	A	G
TCGA-09-1674	ZNF83	ENST00000301096	p.H66R	c.197A>G	Unverified	ACC	CAC	ATT	2	A	G
TCGA-29-1770	ZNF831	ENST00000371030	p.K1267E	c.3799A>G	Unverified	TGG	AAG	AAA	1	A	G
TCGA-23-1123	ZNFX1	ENST00000371752	p.R1180G	c.3538A>G	Verified	GTC	AGG	GTC	1	A	G
TCGA-23-1123	ZNFX1	ENST00000371752	p.R1180G	c.3538A>G	Verified	GTC	AGG	GTC	1	A	G
TCGA-13-1498	ZSCAN10	ENST00000252463	p.R143G	c.427A>G	Verified	TGG	AGG	CTT	1	A	G
TCGA-23-1022	ZSCAN20	ENST00000326544	p.T597A	c.1789A>G	Verified	GAG	ACT	GAT	1	A	G
TCGA-13-0883	ZWILCH	ENST00000307897	p.R259G	c.775A>G	Verified	CCC	AGA	GGT	1	A	G
TCGA-13-1488	ZZZ3	ENST00000370801	p.K669E	c.2005A>G	Verified	ATC	AAA	TAC	1	A	G
TCGA-29-1785	ABCB10	ENST00000344517	p.I584V	c.1750A>G	Unverified	TCT	ATT	GCT	1	A	G
TCGA-29-1785	ABCB10	ENST00000344517	p.I584V	c.1750A>G	Unverified	TCT	ATT	GCT	1	A	G
TCGA-24-1463	ACACA	ENST00000360679	p.L2117L	c.6351A>G	Verified	TTC	CTA	ATT	3	A	G
TCGA-23-1117	ACAN	ENST00000439576	p.V84V	c.252A>G	Verified	GAG	GTA	GTG	3	A	G
TCGA-23-1117	ACAN	ENST00000439576	p.V84V	c.252A>G	Verified	GAG	GTA	GTG	3	A	G
TCGA-61-2095	ACN9	ENST00000432641	p.K28R	c.83A>G	Unverified	CTC	AAA	TCC	2	A	G
TCGA-29-1711	ACSL4	ENST00000469796	p.K630K	c.1890A>G	Unverified	cag	aaa	ggg	3	A	G
TCGA-29-1711	ACSL4	ENST00000469796	p.K630K	c.1890A>G	Unverified	cag	aaa	ggg	3	A	G
TCGA-04-1347	ADAM29	ENST00000359240	p.K365E	c.1093A>G	Verified	ACT	AAA	TTT	1	A	G
TCGA-13-0900	ADAM30	ENST00000369400	p.N462D	c.1384A>G	Verified	gga	aat	gaa	1	A	G
TCGA-29-1693	ADAMTS12	ENST00000504830	p.?	c.1480-2A>G	Unverified	gaa	aac	gtc	1	A	G
TCGA-13-0884	ADAMTS16	NM_139056.1	p.?	c.764-2A>G	Verified	aaa	tac	atg	2	A	G
TCGA-13-0884	ADAMTS16_	ENST00000274181	p.?	c.764-2A>G	Verified	AAA	TAC	ATG	2	A	G
TCGA-24-2289	ADAR	ENST00000368474	p.K1039R	c.3116A>G	Unverified	GAC	AAA	ATC	2	A	G
TCGA-23-1032	AFM	ENST00000226355	p.R121G	c.361A>G	Verified	CAA	AGA	AGA	1	A	G
TCGA-13-0884	AGFG1	ENST00000310078	p.K523R	c.1568A>G	Verified	aca	aag	cca	2	A	G
TCGA-29-1766	AKR1A1	ENST00000372070	p.N322S	c.965A>G	Unverified	ttt	aat	gac	2	A	G

TCGA-24-1422	ALDH7A1	NM_001182.2	p.Y406C	c.1217A>G	Verified	ctc	tat	gtc	2	A	G
TCGA-29-1784	ANAPC5	ENST00000261819	p.E287E	c.861A>G	Unverified	GCC	GAA	AGC	3	A	G
TCGA-25-1318	ANK2	ENST00000357077	p.N453S	c.1358A>G	Unverified	CAG	AAC	GGA	2	A	G
TCGA-25-1318	ANK2	ENST00000357077	p.N453S	c.1358A>G	Unverified	CAG	AAC	GGA	2	A	G
TCGA-29-1763	ANKRD12	ENST00000262126	p.E721E	c.2163A>G	Unverified	TTA	GAA	AAA	3	A	G
TCGA-13-1408	ANKRD23	ENST00000318357	p.S11G	c.31A>G	Verified	GTA	AGT	GGA	1	A	G
TCGA-24-0975	ANKRD6	ENST00000339746	p.K224R	c.671A>G	Unverified	GCC	AAA	ATC	2	A	G
TCGA-36-1577	ANO10	ENST00000292246	p.K606R	c.1817A>G	Verified	TTA	AAG	TTT	2	A	G
TCGA-29-1691	APC	ENST00000457016	p.I1060V	c.3178A>G	Unverified	GAA	ATA	AAA	1	A	G
TCGA-13-0791	APOB	ENST00000233242	p.K2195K	c.6585A>G	Verified	TTG	AAA	ATA	3	A	G
TCGA-24-1435	ARFGEF1	ENST00000262215	p.Q591Q	c.1773A>G	Verified	gct	caa	gga	3	A	G
TCGA-13-0714	ARHGAP1	ENST00000311956	p.Y188C	c.563A>G	Unverified	TTC	TAT	GTG	2	A	G
TCGA-25-2400	ARHGAP29	ENST00000260526	p.M792V	c.2374A>G	Verified	AAT	ATG	TGT	1	A	G
TCGA-20-1685	ARHGEF12	ENST00000397843	p.E1111E	c.3333A>G	Unverified	TAT	GAA	CTG	3	A	G
TCGA-24-0979	ARHGEF2	ENST00000313695	p.N681S	c.2042A>G	Verified	ttc	aat	ggc	2	A	G
TCGA-24-1464	ARID4B	ENST00000366603	p.N1102S	c.3305A>G	Verified	agt	aat	cag	2	A	G
TCGA-24-1464	ARID4B_ENS	ENST00000264183	p.N1102S	c.3305A>G	Verified	AGT	AAT	CAG	2	A	G
TCGA-13-1509	ARL8A	ENST00000272217	p.L114L	c.342A>G	Verified	aac	cta	ctg	3	A	G
TCGA-23-1111	ASH1L	ENST00000392403	p.Q2371Q	c.7113A>G	Unverified	CGT	CAA	AAA	3	A	G
TCGA-29-1702	ASXL1	ENST00000375687	p.E185E	c.555A>G	Unverified	GTG	GAA	TCT	3	A	G
TCGA-13-0903	ATAD2B_EN	ENST00000238789	p.R355G	c.1063A>G	Verified	GCA	AGA	GCA	1	A	G
TCGA-29-1762	ATG2A	ENST00000377264	p.E695E	c.2085A>G	Unverified	CTG	GAA	CTC	3	A	G
TCGA-13-1501	ATM	ENST00000278616	p.Y2954C	c.8861A>G	Verified	CTA	TAT	GAT	2	A	G
TCGA-13-1501	ATM	ENST00000278616	p.Y2954C	c.8861A>G	Verified	CTA	TAT	GAT	2	A	G
TCGA-29-1695	ATP2B1	ENST00000261173	p.I1194V	c.3580A>G	Unverified	GGA	ATT	CAC	1	A	G
TCGA-24-2262	ATP2B2	ENST00000383800	p.N13D	c.37A>G	Verified	AAA	AAC	CAA	1	A	G
TCGA-29-1783	ATRNL1	ENST00000355044	p.E622E	c.1866A>G	Unverified	GAA	GAA	CTT	3	A	G
TCGA-29-1784	AZI2	ENST00000479665	p.N232D	c.694A>G	Unverified	TCT	AAT	TTA	1	A	G
TCGA-61-1915	B4GALT5	ENST00000371711	p.N287S	c.860A>G	Unverified	ATC	AAT	GGC	2	A	G
TCGA-29-1698	BAZ1A	ENST00000382422	p.Q1440Q	c.4320A>G	Unverified	gaa	caa	ctt	3	A	G
TCGA-13-1497	BBS2	ENST00000245157	p.N671S	c.2012A>G	Verified	GGA	AAC	CTC	2	A	G
TCGA-24-1563	BCAS3	ENST00000390652	p.Y223C	c.668A>G	Verified	TGC	TAT	CCA	2	A	G
TCGA-04-1356	BEX4	XM_043653.9	p.E360E	c.1080A>G	Unverified	gaa	gaa	tcc	3	A	G
TCGA-29-1761	BFAR	ENST00000261658	p.E31E	c.93A>G	Unverified	AGT	GAA	TTT	3	A	G

TCGA-04-1347	BLOC1S2	ENST00000370372	p.K30E	c.88A>G	Verified	GCA	AAG	GAG	1	A	G
TCGA-61-1906	BMP2	ENST00000378827	p.Q252Q	c.756A>G	Unverified	cac	caa	gat	3	A	G
TCGA-61-1906	BMP2	ENST00000378827	p.Q252Q	c.756A>G	Unverified	cac	caa	gat	3	A	G
TCGA-23-1110	BOD1L	ENST00000040738	p.K1611E	c.4831A>G	Verified	ACT	AAG	GAA	1	A	G
TCGA-13-0761	BRCA1	ENST00000357654	p.K135R	c.404A>G	Unverified	gcc	aaa	aga	2	A	G
TCGA-13-0761	BRCA1	ENST00000357654	p.K135R	c.404A>G	Unverified	gcc	aaa	aga	2	A	G
TCGA-24-1103	BRCA2	ENST00000380152	p.K1638E	c.4912A>G	Verified	GTT	AAA	GTA	1	A	G
TCGA-61-2095	BRF1	ENST00000546474	p.E466E	c.1398A>G	Unverified	TCG	GAA	GCC	3	A	G
TCGA-23-1029	BRPF3	ENST00000357641	p.K98R	c.293A>G	Unverified	GGC	AAA	AAG	2	A	G
TCGA-29-1783	BTBD1	ENST00000261721	p.M208V	c.622A>G	Unverified	ACA	ATG	GAT	1	A	G
TCGA-29-2427	BUB1	ENST00000302759	p.N534S	c.1601A>G	Verified	gga	aac	aaa	2	A	G
TCGA-13-0904	BUD31	ENST00000222969	p.K3E	c.7A>G	Verified	CCT	AAA	GTC	1	A	G
TCGA-61-1899	C10orf18	ENST00000328090	p.E2117E	c.6351A>G	Unverified	GCT	GAA	TTC	3	A	G
TCGA-09-1674	C10orf68	ENST00000302316	p.K448K	c.1344A>G	Unverified	GAT	AAA	GAA	3	A	G
TCGA-61-1895	C10orf71_EI	ENST00000323868	p.K138R	c.413A>G	Unverified	AAT	AAG	CCT	2	A	G
TCGA-61-1895	C10orf71_EI	ENST00000374144	p.K138R	c.413A>G	Unverified	AAT	AAG	CCT	2	A	G
TCGA-23-1032	C14orf135	ENST00000404681	p.N152S	c.455A>G	Verified	AGC	AAT	TCC	2	A	G
TCGA-24-1422	C1orf9	ENST00000263688	p.I1130M	c.3390A>G	Verified	CCC	ATA	GCC	3	A	G
TCGA-23-1111	C1RL	ENST00000266542	p.K72R	c.215A>G	Unverified	ATC	AAG	GCT	2	A	G
TCGA-24-1845	C2	ENST00000299367	p.Y347C	c.1040A>G	Unverified	GTC	TAT	CTC	2	A	G
TCGA-09-2050	C20orf12	ENST00000262547	p.K210R	c.629A>G	Verified	CTC	AAG	TGT	2	A	G
TCGA-09-1665	C20orf54	ENST00000217254	p.N311S	c.932A>G	Verified	GTC	AAC	GCG	2	A	G
TCGA-09-2049	C2orf16	ENST00000408964	p.Q1206Q	c.3618A>G	Unverified	ACT	CAA	GCT	3	A	G
TCGA-09-2049	C2orf16_EN:	ENST00000408964	p.Q1206Q	c.3618A>G	Unverified	ACT	CAA	GCT	3	A	G
TCGA-24-1470	C2orf78	ENST00000342345	p.V566V	c.1698A>G	Verified	AAG	GTA	GAA	3	A	G
TCGA-29-1761	C3P1	ENST00000333905	p.V156V	c.468A>G	Unverified	ATG	GTA	CCC	3	A	G
TCGA-24-2288	C7orf45	ENST00000297819	p.E158E	c.474A>G	Unverified	TCA	GAA	GCC	3	A	G
TCGA-13-0883	C7orf64	ENST00000265732	p.T328A	c.982A>G	Verified	AAT	ACA	ACG	1	A	G
TCGA-23-1114	C9orf174	ENST00000375202	p.R1578G	c.4732A>G	Unverified	GCA	AGG	ATG	1	A	G
TCGA-20-1686	CACNA2D1	ENST00000356860	p.N1030S	c.3089A>G	Unverified	CCA	AAT	CCT	2	A	G
TCGA-09-1674	CALM2	ENST00000272298	p.K95E	c.283A>G	Unverified	GAT	AAG	GAT	1	A	G
TCGA-09-2051	CALML5	ENST00000380332	p.N25S	c.74A>G	Unverified	GGA	AAC	GGC	2	A	G
TCGA-24-1464	CAMK4	ENST00000512453	p.K146E	c.436A>G	Verified	gtt	aaa	caa	1	A	G
TCGA-24-1604	CCAR1	ENST00000265872	p.K156E	c.466A>G	Verified	ACA	AAA	CTA	1	A	G

TCGA-61-2109	CCDC39	ENST00000273654	p.K212E	c.634A>G	Unverified	CAA	AAA	TTG	1	A	G
TCGA-04-1652	CCDC51	ENST00000395694	p.N93S	c.278A>G	Unverified	CTC	AAC	GAG	2	A	G
TCGA-04-1652	CCDC51	ENST00000395694	p.N93S	c.278A>G	Unverified	CTC	AAC	GAG	2	A	G
TCGA-23-1031	CCR10	ENST00000332438	p.E18E	c.54A>G	Unverified	GAT	GAA	GAG	3	A	G
TCGA-23-1031	CD163	ENST00000359156	p.K640E	c.1918A>G	Verified	GGA	AAA	GGA	1	A	G
TCGA-25-1313	CDC42BPG_	ENST00000342711	p.Y697C	c.2090A>G	Unverified	GGC	TAC	CTG	2	A	G
TCGA-61-1904	CDC42EP1	ENST00000249014	p.T235A	c.703A>G	Unverified	CCT	ACT	GCA	1	A	G
TCGA-61-1900	CDC5L	ENST00000371477	p.Q175Q	c.525A>G	Unverified	aaa	caa	ttg	3	A	G
TCGA-04-1337	CDH1	ENST00000261769	p.M316V	c.946A>G	Verified	AAT	ATG	TTC	1	A	G
TCGA-04-1336	CDH17	ENST00000027335	p.K486E	c.1456A>G	Verified	TCT	AAA	ATT	1	A	G
TCGA-20-0990	CDK10	ENST00000353379	p.N157S	c.470A>G	Verified	AGG	AAC	TTC	2	A	G
TCGA-24-1846	CDK13	ENST00000181839	p.I875V	c.2623A>G	Unverified	GTA	ATT	ACT	1	A	G
TCGA-13-1498	CEACAM7	ENST00000006724	p.Y140C	c.419A>G	Verified	TTC	TAC	GTA	2	A	G
TCGA-24-1104	CECR2	ENST00000400585	p.K387R	c.1160A>G	Verified	AAA	AAG	ACT	2	A	G
TCGA-61-2102	CELA2A	ENST00000359621	p.Y246C	c.737A>G	Verified	TAC	TAC	CAC	2	A	G
TCGA-13-1512	CEP120	ENST00000306467	p.E438E	c.1314A>G	Verified	TCA	GAA	GTA	3	A	G
TCGA-24-2262	CEP135	ENST00000257287	p.K749E	c.2245A>G	Verified	GAA	AAG	ATT	1	A	G
TCGA-29-1785	CEP63	ENST00000337090	p.M638V	c.1912A>G	Unverified	ACT	ATG	TCT	1	A	G
TCGA-29-1785	CEP63	ENST00000337090	p.M638V	c.1912A>G	Unverified	ACT	ATG	TCT	1	A	G
TCGA-04-1367	CES3	ENST00000303334	p.K326R	c.977A>G	Verified	CCC	AAG	GAA	2	A	G
TCGA-20-1686	CHD7	ENST00000423902	p.K2587K	c.7761A>G	Unverified	CCT	AAA	AAT	3	A	G
TCGA-20-1686	CHD7_ENST	ENST00000423902	p.K2587K	c.7761A>G	Unverified	CCT	AAA	AAT	3	A	G
TCGA-13-1499	CKAP2	ENST00000258607	p.N580S	c.1739A>G	Verified	CCC	AAT	ACA	2	A	G
TCGA-04-1649	CLCA4	ENST00000370563	p.N811S	c.2432A>G	Unverified	GTA	AAT	ACT	2	A	G
TCGA-13-0906	CLEC16A	ENST00000409790	p.V190V	c.570A>G	Verified	GCT	GTA	AGA	3	A	G
TCGA-24-1422	CLIC1	ENST00000375780	p.E4E	c.12A>G	Verified	GAA	GAA	CAA	3	A	G
TCGA-13-0923	CLNK	ENST00000226951	p.K45R	c.134A>G	Verified	AAC	AAG	CCT	2	A	G
TCGA-23-1032	CLTC	ENST00000269122	p.Y1289C	c.3866A>G	Verified	AAC	TAC	TAT	2	A	G
TCGA-25-2400	CMYA5	ENST00000238522	p.K3055K	c.9165A>G	Verified	GAA	AAA	TAT	3	A	G
TCGA-13-0904	CNBD1	ENST00000518476	p.E429E	c.1287A>G	Unverified	ATT	GAA	GAT	3	A	G
TCGA-04-1362	CNNM3	ENST00000305510	p.Y456C	c.1367A>G	Verified	GAC	TAC	CGA	2	A	G
TCGA-29-1763	COBL	ENST00000265136	p.E106E	c.318A>G	Unverified	CTT	GAA	ATT	3	A	G
TCGA-20-1686	COL19A1	ENST00000322773	p.I245M	c.735A>G	Unverified	GAA	ATA	TCA	3	A	G
TCGA-24-2024	COL3A1	ENST00000304636	p.K1407R	c.4220A>G	Verified	AGC	AAA	TTC	2	A	G

TCGA-09-1665	COL4A6	ENST00000334504	p.Q1367Q	c.4101A>G	Verified	GGA	CAA	ACA	3	A	G
TCGA-04-1649	COL5A2	ENST00000374866	p.N1357S	c.4070A>G	Unverified	GAC	AAT	AAA	2	A	G
TCGA-23-1031	COPS7A	ENST00000543155	p.Y156C	c.467A>G	Verified	gac	tac	agc	2	A	G
TCGA-24-1849	COX18	ENST00000295890	p.T209A	c.625A>G	Unverified	GCT	ACT	GGT	1	A	G
TCGA-29-1698	CP	ENST00000264613	p.Y260C	c.779A>G	Unverified	ATG	TAT	TCT	2	A	G
TCGA-13-0905	CPAMD8	ENST00000291440	p.Y213C	c.638A>G	Verified	GCC	TAC	ATC	2	A	G
TCGA-23-1116	CPAMD8	ENST00000291440	p.N1104S	c.3311A>G	Verified	TCC	AAT	GAG	2	A	G
TCGA-29-1783	CPT2	ENST00000371486	p.N585S	c.1754A>G	Unverified	CAC	AAT	GTC	2	A	G
TCGA-13-1505	CR1	ENST00000400960	p.I1799V	c.5395A>G	Verified	GAA	ATA	TCT	1	A	G
TCGA-23-1118	CRBN	ENST00000231948	p.T119A	c.355A>G	Verified	AGA	ACC	TTT	1	A	G
TCGA-24-2280	CS	ENST00000351328	p.N400S	c.1199A>G	Unverified	ccc	aat	gta	2	A	G
TCGA-24-1843	CSMD3	ENST00000297405	p.I3255V	c.9763A>G	Unverified	agt	att	agc	1	A	G
TCGA-24-1843	CSMD3_ENS	ENST00000343508	p.I3215V	c.9643A>G	Unverified	AGT	ATT	AGC	1	A	G
TCGA-61-1915	CTNNA3	NM_013266.1	p.T437A	c.1309A>G	Unverified	tca	aca	aat	1	A	G
TCGA-61-1915	CTNNA3_EN	ENST00000433211	p.T437A	c.1309A>G	Unverified	TCA	ACA	AAT	1	A	G
TCGA-24-1604	CTR9	ENST00000361367	p.Y185C	c.554A>G	Verified	GCT	TAC	TAT	2	A	G
TCGA-24-2267	CUL2	ENST00000374751	p.Y100C	c.299A>G	Verified	gac	tat	atg	2	A	G
TCGA-09-2049	CXorf23_EN	ENST00000379687	p.K222E	c.664A>G	Verified	CCT	AAA	GAC	1	A	G
TCGA-24-2280	CYP20A1	ENST00000356079	p.Q264Q	c.792A>G	Unverified	GAC	CAA	CAG	3	A	G
TCGA-13-1488	DCAF16	ENST00000382247	p.T198A	c.592A>G	Verified	ACT	ACT	TAT	1	A	G
TCGA-29-1784	DCBLD2	ENST00000326840	p.Y265C	c.794A>G	Unverified	TAT	TAT	GAA	2	A	G
TCGA-23-1021	DCK	ENST00000286648	p.T72A	c.214A>G	Verified	CTT	ACA	ATG	1	A	G
TCGA-30-1856	DDI2	ENST00000480945	p.N57S	c.170A>G	Unverified	GAC	AAC	CAC	2	A	G
TCGA-13-0886	DDX6	NM_004397.3	p.K356E	c.1066A>G	Verified	gct	aaa	atg	1	A	G
TCGA-61-1998	DEPDC5	ENST00000400248	p.K1079R	c.3236A>G	Unverified	CGA	AAG	GTA	2	A	G
TCGA-24-2035	DGKB	ENST00000403951	p.L462L	c.1386A>G	Verified	TAT	CTA	TTA	3	A	G
TCGA-24-1845	DHX9	ENST00000367549	p.N981D	c.2941A>G	Unverified	ACT	AAC	ACT	1	A	G
TCGA-13-0885	DHX9	ENST00000367549	p.L684L	c.2052A>G	Verified	ATT	CTA	CCC	3	A	G
TCGA-29-1762	DMD_ENST(	ENST00000357033	p.K760K	c.2280A>G	Unverified	TTA	AAA	GAA	3	A	G
TCGA-29-1762	DMD_ENST(	ENST00000378677	p.K756K	c.2268A>G	Unverified	TTA	AAA	GAA	3	A	G
TCGA-24-1464	DMD_ENST(	ENST00000378677	p.E607E	c.1821A>G	Verified	CTA	GAA	AAG	3	A	G
TCGA-29-1762	DMD_ENST(	ENST00000378677	p.K756K	c.2268A>G	Unverified	TTA	AAA	GAA	3	A	G
TCGA-04-1652	DNAH11	ENST00000328843	p.K2761K	c.8283A>G	Unverified	AAA	AAA	GAT	3	A	G
TCGA-04-1652	DNAH11	ENST00000328843	p.K2761K	c.8283A>G	Unverified	AAA	AAA	GAT	3	A	G

TCGA-04-1347	DNALI1	ENST00000296218	p.Y186C	c.557A>G	Verified	GCC	TAC	CAG	2	A	G
TCGA-20-1683	DNMBP	ENST00000324109	p.Y1284C	c.3851A>G	Unverified	AGG	TAT	CCC	2	A	G
TCGA-61-1913	DOCK2	ENST00000256935	p.K268R	c.803A>G	Unverified	CCT	AAG	GAG	2	A	G
TCGA-30-1855	DOCK4_ENS	ENST00000437633	p.N328D	c.982A>G	Unverified	TGT	AAC	ACA	1	A	G
TCGA-24-2024	DPP4	ENST00000360534	p.R310G	c.928A>G	Verified	GAA	AGA	ATT	1	A	G
TCGA-61-1738	DPYD	ENST00000370192	p.V162V	c.486A>G	Unverified	GAG	GTA	TTC	3	A	G
TCGA-61-1738	DPYD	ENST00000370192	p.V162V	c.486A>G	Unverified	GAG	GTA	TTC	3	A	G
TCGA-04-1336	DPYS	ENST00000351513	p.K153E	c.457A>G	Verified	gat	aaa	ggt	1	A	G
TCGA-61-1910	DRP2	ENST00000402866	p.K609R	c.1826A>G	Unverified	ACC	AAG	TGC	2	A	G
TCGA-04-1649	DSCAM	ENST00000400454	p.N1341S	c.4022A>G	Unverified	AGC	AAC	GGA	2	A	G
TCGA-29-1777	DST	ENST00000370765	p.S4G	c.10A>G	Unverified	AGT	AGT	AGT	1	A	G
TCGA-29-1777	DST_ENST0(	ENST00000244364	p.S4G	c.10A>G	Unverified	AGT	AGT	AGT	1	A	G
TCGA-29-1777	DST_ENST0(	ENST00000370765	p.S4G	c.10A>G	Unverified	AGT	AGT	AGT	1	A	G
TCGA-13-0760	DST_ENST0(	ENST00000370769	p.K5067E	c.15199A>G	Verified	TTT	AAA	GAA	1	A	G
TCGA-13-0760	DST_ENST0(	ENST00000370769	p.K3597K	c.10791A>G	Verified	AGC	AAA	CAA	3	A	G
TCGA-13-0893	DUSP19	ENST00000354221	p.N90S	c.269A>G	Verified	aag	aat	aag	2	A	G
TCGA-24-1844	DUSP6	ENST00000279488	p.K324K	c.972A>G	Unverified	GTC	AAA	ATG	3	A	G
TCGA-04-1338	DYRK1A	ENST00000398960	p.I283V	c.847A>G	Verified	agt	atc	att	1	A	G
TCGA-13-0923	DYSF	ENST00000258104	p.I1582V	c.4744A>G	Verified	CGT	ATC	TAC	1	A	G
TCGA-24-0975	EDC4	ENST00000358933	p.N484D	c.1450A>G	Verified	GAA	AAT	GAC	1	A	G
TCGA-04-1367	EEA1	ENST00000322349	p.K983K	c.2949A>G	Verified	CTT	AAA	ATT	3	A	G
TCGA-24-2289	EGFR	ENST00000275493	p.K716R	c.2147A>G	Unverified	ATC	AAA	GTG	2	A	G
TCGA-13-0760	EIF3K	ENST00000248342	p.I18V	c.52A>G	Verified	GGT	ATC	GAC	1	A	G
TCGA-09-2044	EIF4A1	ENST00000293831	p.N280S	c.839A>G	Verified	ATC	AAC	ACC	2	A	G
TCGA-61-1915	ENSG00001	ENST00000341450	p.M20V	c.58A>G	Unverified	CCA	ATG	TAT	1	A	G
TCGA-24-1469	ENSG00002	ENST00000429810	p.S197G	c.589A>G	Verified	GCA	AGT	GCT	1	A	G
TCGA-30-1857	ENSG00002	ENST00000454856	p.K326K	c.978A>G	Unverified	GAA	AAA	CAG	3	A	G
TCGA-13-0884	EPRS	ENST00000366923	p.K865K	c.2595A>G	Verified	TAT	AAA	GAA	3	A	G
TCGA-24-2288	EPS8	ENST00000281172	p.S353G	c.1057A>G	Unverified	CCT	AGT	GCT	1	A	G
TCGA-13-1512	ERVFRD-1	ENST00000472091	p.K27E	c.79A>G	Verified	GAA	AAA	GCT	1	A	G
TCGA-24-2280	EXOC6	ENST00000260762	p.T127A	c.379A>G	Unverified	ACA	ACT	GTA	1	A	G
TCGA-25-2392	EZH1	ENST00000428826	p.K322R	c.965A>G	Verified	cgc	aag	aat	2	A	G
TCGA-04-1655	F5	ENST00000367797	p.Y1546C	c.4637A>G	Unverified	CCC	TAT	GAT	2	A	G
TCGA-13-0760	F5	ENST00000367797	p.Y2149C	c.6446A>G	Verified	ATG	TAT	GTA	2	A	G

TCGA-61-2095	FAM178B	ENST00000393526	p.T93A	c.277A>G	Unverified	GCT	ACC	CAG	1	A	G
TCGA-24-0975	FAM190B	ENST00000224756	p.N291S	c.872A>G	Verified	TTT	AAT	AGG	2	A	G
TCGA-61-1737	FAM21B	ENST00000358474	p.L694L	c.2082A>G	Unverified	GAT	TTA	TTT	3	A	G
TCGA-61-1737	FAM21B_EN	ENST00000358474	p.L694L	c.2082A>G	Unverified	GAT	TTA	TTT	3	A	G
TCGA-13-0714	FAM83B	ENST00000306858	p.K489R	c.1466A>G	Verified	ACA	AAG	TCA	2	A	G
TCGA-24-1422	FAM83B	ENST00000306858	p.K823K	c.2469A>G	Verified	ACA	AAA	GTT	3	A	G
TCGA-29-1761	FANCA	ENST00000389301	p.K143R	c.428A>G	Unverified	AGA	AAG	AAG	2	A	G
TCGA-61-2009	FAT3_ENST(	ENST00000298047_v61	p.T764A	c.2290A>G	Verified	TTT	ACA	ATA	1	A	G
TCGA-23-1114	FAT3_ENST(	ENST00000298047_v61	p.E2556E	c.7668A>G	Unverified	ACA	GAA	AGG	3	A	G
TCGA-23-1114	FAT3_ENST(	ENST00000409404	p.E2556E	c.7668A>G	Unverified	ACA	GAA	AGG	3	A	G
TCGA-61-1733	FBN1	ENST00000316623	p.K2840R	c.8519A>G	Unverified	AAG	AAA	GAA	2	A	G
TCGA-61-1733	FBN1	ENST00000316623	p.K2840R	c.8519A>G	Unverified	AAG	AAA	GAA	2	A	G
TCGA-13-0903	FCRL3	ENST00000368184	p.E716E	c.2148A>G	Verified	GAA	GAA	GAT	3	A	G
TCGA-13-1510	FGB	ENST00000302068	p.Q180Q	c.540A>G	Unverified	CAC	CAA	TTA	3	A	G
TCGA-23-1124	FLJ42177	ENST00000355283	p.E245E	c.735A>G	Verified	ATT	GAA	AAG	3	A	G
TCGA-61-1725	FOLR3	ENST00000456237	p.Y271C	c.812A>G	Unverified	TTC	TAT	GCT	2	A	G
TCGA-30-1718	FOXM1	ENST00000342628	p.Q712Q	c.2136A>G	Unverified	CCG	CAA	AGG	3	A	G
TCGA-24-0975	FREM2	ENST00000280481	p.N358S	c.1073A>G	Unverified	TTC	AAC	CTT	2	A	G
TCGA-24-1469	FRK	ENST00000368626	p.I121V	c.361A>G	Verified	gca	atc	gga	1	A	G
TCGA-61-2012	FRMPD1	ENST00000377765	p.S359G	c.1075A>G	Verified	ATT	AGC	TTC	1	A	G
TCGA-13-1498	FRZB	ENST00000295113	p.Y259C	c.776A>G	Verified	GGC	TAT	GAA	2	A	G
TCGA-29-1761	FSIP2	ENST00000343098	p.K465E	c.1393A>G	Unverified	AGT	AAA	CCA	1	A	G
TCGA-29-1761	FSIP2_ENST(	ENST00000343098	p.K465E	c.1393A>G	Unverified	AGT	AAA	CCA	1	A	G
TCGA-29-1775	GAB2	ENST00000361507	p.K650R	c.1949A>G	Unverified	gag	aag	acc	2	A	G
TCGA-24-1470	GABBR1	ENST00000377034	p.N431S	c.1292A>G	Verified	CTG	AAT	CCT	2	A	G
TCGA-24-1616	GABRG2	ENST00000356592	p.Y452C	c.1355A>G	Verified	TCC	TAT	GCT	2	A	G
TCGA-13-0916	GALNT6	ENST00000356317	p.K70R	c.209A>G	Verified	CCC	AAG	CTC	2	A	G
TCGA-29-1701	GANAB	ENST00000356638	p.K231R	c.692A>G	Unverified	TTC	AAA	ACT	2	A	G
TCGA-61-1740	GBP5	ENST00000370459	p.Q327Q	c.981A>G	Unverified	GTG	CAA	AAG	3	A	G
TCGA-04-1649	GGA1	ENST00000343632	p.N284S	c.851A>G	Unverified	GCC	AAT	GAC	2	A	G
TCGA-23-1022	GJA8	ENST00000369235	p.K390R	c.1169A>G	Verified	GAG	AAG	GTG	2	A	G
TCGA-29-1784	GLDN	ENST00000335449	p.T547A	c.1639A>G	Unverified	TCA	ACT	ACC	1	A	G
TCGA-13-0923	GLUD2	ENST00000328078	p.L293L	c.879A>G	Unverified	att	tta	gga	3	A	G
TCGA-29-1777	GLUL	ENST00000339526	p.K333R	c.998A>G	Unverified	GAG	AAG	AAG	2	A	G

TCGA-10-0930	GNB2	ENST00000303210	p.M101V	c.301A>G	Verified	gta	atg	acc	1	A	G
TCGA-10-0930	GNB2	ENST00000303210	p.M101V	c.301A>G	Verified	gta	atg	acc	1	A	G
TCGA-24-0979	GOLGA3	ENST00000204726	p.T934A	c.2800A>G	Verified	GAA	ACA	CAC	1	A	G
TCGA-20-1683	GOSR2	ENST00000225567	p.K57R	c.170A>G	Unverified	AGC	AAG	GAG	2	A	G
TCGA-20-1683	GOSR2_ENS	ENST00000415811	p.K57R	c.170A>G	Unverified	AGC	AAG	GAG	2	A	G
TCGA-20-1683	GOSR2_ENS	ENST00000439730	p.K57R	c.170A>G	Unverified	AGC	AAG	GAG	2	A	G
TCGA-29-1695	GPI	ENST00000356487	p.Q15Q	c.45A>G	Unverified	cag	caa	tgg	3	A	G
TCGA-23-1031	GPR12	ENST00000381436	p.L82L	c.246A>G	Unverified	CTG	CTA	ATA	3	A	G
TCGA-25-2398	GPR141	ENST00000334425	p.Y265C	c.794A>G	Verified	TTT	TAT	AAC	2	A	G
TCGA-23-1124	GPR149	ENST00000389740	p.I196V	c.586A>G	Verified	TCT	ATC	GTG	1	A	G
TCGA-13-0916	GPR21	ENST00000373642	p.Y190C	c.569A>G	Verified	TCC	TAC	TTC	2	A	G
TCGA-13-0807	GPR98	ENST00000405460	p.S2368G	c.7102A>G	Verified	AGA	AGT	TCC	1	A	G
TCGA-24-2035	GPR98	ENST00000405460	p.E5087E	c.15261A>G	Unverified	ATA	GAA	GAA	3	A	G
TCGA-13-1497	GRPR	ENST00000380289	p.K235K	c.705A>G	Verified	GCT	AAA	AAT	3	A	G
TCGA-24-1435	GTF2IRD2B	ENST00000312575	p.K790E	c.2368A>G	Verified	GCA	AAA	CTG	1	A	G
TCGA-23-1114	GUCY1A2	ENST00000526355	p.Q170Q	c.510A>G	Unverified	ATT	CAA	AAA	3	A	G
TCGA-23-1122	GUCY2C	ENST00000261170	p.T618A	c.1852A>G	Verified	TCT	ACC	AAC	1	A	G
TCGA-24-2267	GUCY2C	ENST00000261170	p.S23G	c.67A>G	Verified	TTT	AGT	TCC	1	A	G
TCGA-04-1331	HACE1	ENST00000262903	p.M114V	c.340A>G	Verified	TGT	ATG	AGT	1	A	G
TCGA-29-1775	HDC	ENST00000267845	p.N588S	c.1763A>G	Unverified	TGC	AAC	AGT	2	A	G
TCGA-25-1326	HDX	ENST00000297977	p.N242D	c.724A>G	Verified	cat	aac	tta	1	A	G
TCGA-04-1651	HEATR1	ENST00000366582	p.E1926E	c.5778A>G	Unverified	ACA	GAA	GAT	3	A	G
TCGA-04-1651	HEATR1	ENST00000366582	p.E1926E	c.5778A>G	Unverified	ACA	GAA	GAT	3	A	G
TCGA-29-1763	HEATR5A	ENST00000389961	p.E1518E	c.4554A>G	Unverified	GAT	GAA	GGA	3	A	G
TCGA-29-1763	HEATR5A_EI	ENST00000389961_v65	p.E1518E	c.4554A>G	Unverified	GAT	GAA	GGA	3	A	G
TCGA-23-2078	HERC4	ENST00000395198	p.N906S	c.2717A>G	Verified	TTC	AAT	AAA	2	A	G
TCGA-09-1674	HEXA	ENST00000268097	p.N295S	c.884A>G	Unverified	CTC	AAT	AAT	2	A	G
TCGA-24-1417	HFM1	ENST00000370425	p.L1011L	c.3033A>G	Unverified	ATA	TTA	AGA	3	A	G
TCGA-29-1696	HLTF	ENST00000310053	p.N24S	c.71A>G	Unverified	gga	aat	ttt	2	A	G
TCGA-29-1696	HLTF	ENST00000310053	p.N24S	c.71A>G	Unverified	gga	aat	ttt	2	A	G
TCGA-61-1740	HPS4	ENST00000336873	p.E499E	c.1497A>G	Unverified	TGT	GAA	AGC	3	A	G
TCGA-61-1740	HPS4_ENST(	ENST00000398141	p.E512E	c.1536A>G	Unverified	TGT	GAA	AGC	3	A	G
TCGA-29-2427	HSD17B4	ENST00000256216	p.I399V	c.1195A>G	Verified	TCA	ATC	AAC	1	A	G
TCGA-61-1895	IGFN1	ENST00000335211	p.K66R	c.197A>G	Unverified	TCC	AAA	GGT	2	A	G

TCGA-61-1895	IGFN1_ENST	ENST00000335211	p.K2906R	c.8717A>G	Unverified	TCC	AAG	GAT	2	A	G
TCGA-09-2044	IL31RA	ENST00000447346	p.N663S	c.1988A>G	Verified	AAC	AAT	TTA	2	A	G
TCGA-24-2289	INSRR	ENST00000368195	p.E451E	c.1353A>G	Unverified	TTG	GAA	CAC	3	A	G
TCGA-24-2289	INSRR_ENST	ENST00000368195	p.E451E	c.1353A>G	Unverified	TTG	GAA	CAC	3	A	G
TCGA-13-0904	INTS6	ENST00000311234	p.T686A	c.2056A>G	Verified	cct	aca	act	1	A	G
TCGA-25-2392	IPP	ENST00000396478	p.M471V	c.1411A>G	Verified	CCA	ATG	GGA	1	A	G
TCGA-61-1737	IQSEC2	XM_291345.2	p.K1005R	c.3014A>G	Unverified	acc	aaa	att	2	A	G
TCGA-61-1737	IQSEC2_ENS	ENST00000396435	p.K1008R	c.3023A>G	Unverified	ACC	AAA	ATT	2	A	G
TCGA-13-0920	IRF8	ENST00000268638	p.K148R	c.443A>G	Verified	ATC	AAG	GAG	2	A	G
TCGA-61-1907	ITGA6_ENST	ENST00000409080	p.K1067R	c.3200A>G	Unverified	ATC	AAA	GAT	2	A	G
TCGA-24-0979	ITPR3	ENST00000374316	p.N1487S	c.4460A>G	Verified	GAG	AAC	AGC	2	A	G
TCGA-24-1423	JMJD1C	ENST00000402544	p.N2195S	c.6584A>G	Verified	GTG	AAC	AAA	2	A	G
TCGA-13-1488	KATNAL2	ENST00000245121	p.E131E	c.393A>G	Verified	AGT	GAA	CTT	3	A	G
TCGA-61-1914	KCNK10	ENST00000340700	p.K297R	c.890A>G	Unverified	TAT	AAG	CCC	2	A	G
TCGA-61-1914	KCNK10_EN:	ENST00000312350	p.K302R	c.905A>G	Unverified	TAT	AAG	CCC	2	A	G
TCGA-61-1914	KCNK10_EN:	ENST00000319231	p.K302R	c.905A>G	Unverified	TAT	AAG	CCC	2	A	G
TCGA-24-0975	KDM3B	ENST00000314358	p.N848S	c.2543A>G	Verified	CCC	AAT	GGG	2	A	G
TCGA-13-1498	KIAA1109	ENST00000264501	p.Y4272C	c.12815A>G	Verified	TGG	TAT	AGA	2	A	G
TCGA-24-1422	KIAA1109	ENST00000264501	p.E4578E	c.13734A>G	Verified	CAG	GAA	TTT	3	A	G
TCGA-23-1114	KIAA1529	ENST00000375206	p.R1523G	c.4567A>G	Unverified	GCA	AGG	ATG	1	A	G
TCGA-61-1737	KIDINS220	ENST00000256707	p.Y1079C	c.3236A>G	Unverified	GCG	TAC	CCC	2	A	G
TCGA-04-1542	KIF13A	ENST00000259711	p.K404K	c.1212A>G	Verified	ATA	AAA	GAA	3	A	G
TCGA-29-1776	KIF13A	ENST00000259711	p.I995M	c.2985A>G	Unverified	TCC	ATA	TTA	3	A	G
TCGA-29-1776	KIF13A_ENS	ENST00000259711	p.I995M	c.2985A>G	Unverified	TCC	ATA	TTA	3	A	G
TCGA-13-0887	KIF21A	ENST00000361961	p.K950K	c.2850A>G	Verified	GAG	AAA	CTT	3	A	G
TCGA-13-0762	KIF2A	NM_004520.1	p.I609V	c.1825A>G	Unverified	tct	att	cgg	1	A	G
TCGA-13-0762	KIF2A	NM_004520.1	p.I609V	c.1825A>G	Unverified	tct	att	cgg	1	A	G
TCGA-04-1638	KLB	ENST00000257408	p.Q980Q	c.2940A>G	Unverified	ACC	CAA	GAA	3	A	G
TCGA-23-1022	KLHL6	ENST00000341319	p.Y519C	c.1556A>G	Verified	ATC	TAT	GTC	2	A	G
TCGA-10-0930	KRIT1	ENST00000394507	p.N580D	c.1738A>G	Verified	gaa	aat	cta	1	A	G
TCGA-10-0930	KRIT1	ENST00000394507	p.N580D	c.1738A>G	Verified	gaa	aat	cta	1	A	G
TCGA-23-1809	KRT19	ENST00000361566	p.K370R	c.1109A>G	Unverified	ATC	AAG	TCG	2	A	G
TCGA-24-1423	KRT6B	ENST00000252252	p.K68R	c.203A>G	Unverified	TCC	AAG	AGG	2	A	G
TCGA-61-1914	L1TD1	ENST00000498273	p.Y849C	c.2546A>G	Unverified	GAT	TAT	GTT	2	A	G

TCGA-25-1318	LACTB2	ENST00000522447	p.Q212Q	c.636A>G	Verified	caa	caa	tac	3	A	G
TCGA-25-1318	LACTB2	ENST00000522447	p.Q212Q	c.636A>G	Verified	caa	caa	tac	3	A	G
TCGA-13-1489	LATS1_ENST	ENST00000253339	p.K662K	c.1986A>G	Verified	AAA	AAA	CAA	3	A	G
TCGA-24-1847	LCE1D	ENST00000326233	p.K15R	c.44A>G	Unverified	CCC	AAG	TGC	2	A	G
TCGA-30-1856	LHX9	ENST00000367387	p.K121R	c.362A>G	Unverified	TGC	AAG	GAG	2	A	G
TCGA-30-1856	LHX9_ENST(	ENST00000367390	p.K112R	c.335A>G	Unverified	TGC	AAG	GAG	2	A	G
TCGA-13-1496	LIG3	NM_002311.2	p.I623V	c.1867A>G	Verified	tca	atc	ttc	1	A	G
TCGA-24-2280	LINGO2	ENST00000379992	p.N258S	c.773A>G	Unverified	ACC	AAC	ACC	2	A	G
TCGA-24-2280	LINGO2_ENS	ENST00000379992	p.N258S	c.773A>G	Unverified	ACC	AAC	ACC	2	A	G
TCGA-09-2044	LIPK	ENST00000404190	p.I337V	c.1009A>G	Verified	GCA	ATA	TGG	1	A	G
TCGA-04-1651	LMAN2	ENST00000303127	p.N305S	c.914A>G	Unverified	GAC	AAC	GTG	2	A	G
TCGA-04-1651	LMAN2	ENST00000303127	p.N305S	c.914A>G	Unverified	GAC	AAC	GTG	2	A	G
TCGA-61-1998	LOC652153	ENST00000316490	p.Q1749Q	c.5247A>G	Unverified	AGC	CAA	ACC	3	A	G
TCGA-23-1029	LPHN2	ENST00000359929	p.T965A	c.2893A>G	Unverified	GGA	ACA	GAA	1	A	G
TCGA-23-1029	LPHN2_ENST	ENST00000370725	p.T978A	c.2932A>G	Unverified	GGA	ACA	GAA	1	A	G
TCGA-23-1032	LPXN	ENST00000395074	p.M226V	c.676A>G	Verified	GCA	ATG	AAC	1	A	G
TCGA-13-0887	LRBA	ENST00000357115	p.I2458V	c.7372A>G	Verified	TCA	ATA	ACT	1	A	G
TCGA-29-1768	LRCH1	ENST00000389798	p.T401A	c.1201A>G	Unverified	TTT	ACT	GAT	1	A	G
TCGA-29-1768	LRCH1_ENST	ENST00000311191	p.T401A	c.1201A>G	Unverified	TTT	ACT	GAT	1	A	G
TCGA-24-1845	LRP12	ENST00000276654	p.Y275C	c.824A>G	Unverified	AAT	TAT	CCA	2	A	G
TCGA-13-1510	LRP4	ENST00000378623	p.N1589S	c.4766A>G	Unverified	CGG	AAC	AAG	2	A	G
TCGA-61-1910	LRRC28	ENST00000301981	p.T324A	c.970A>G	Unverified	TTT	ACC	ATC	1	A	G
TCGA-04-1367	LRRC31	ENST00000316428	p.N124S	c.371A>G	Verified	TGG	AAT	GGT	2	A	G
TCGA-24-1844	LRRC66	ENST00000343457	p.S603G	c.1807A>G	Unverified	GAT	AGT	AAG	1	A	G
TCGA-30-1855	LRRIQ1_ENS	ENST00000393217	p.S1677G	c.5029A>G	Unverified	GTA	AGC	AGA	1	A	G
TCGA-24-1463	LYAR	ENST00000343470	p.N7S	c.20A>G	Unverified	TGC	AAT	GCA	2	A	G
TCGA-61-1740	LYST	ENST00000389793	p.Y1976C	c.5927A>G	Unverified	GAA	TAC	AAA	2	A	G
TCGA-61-2109	MACF1	ENST00000360115	p.N3607S	c.10820A>G	Unverified	CTT	AAC	AAA	2	A	G
TCGA-25-2391	MACF1	ENST00000360115	p.L2655L	c.7965A>G	Unverified	CTT	CTA	CCC	3	A	G
TCGA-61-2109	MACF1_ENS	ENST00000361689	p.N3105S	c.9314A>G	Unverified	CTT	AAC	AAA	2	A	G
TCGA-25-2391	MACF1_ENS	ENST00000361689	p.L2153L	c.6459A>G	Unverified	CTT	CTA	CCC	3	A	G
TCGA-61-1910	MALT1_ENS	ENST00000348428	p.K226R	c.677A>G	Unverified	TCC	AAG	TTG	2	A	G
TCGA-25-2042	MAN2B2	ENST00000285599	p.Y362C	c.1085A>G	Verified	TTC	TAC	ACG	2	A	G
TCGA-61-2109	MAP3K7	ENST00000369332	p.Y93C	c.278A>G	Unverified	ctt	tat	gga	2	A	G

TCGA-13-0714	MAPK9_ENS	ENST00000452135	p.N90S	c.269A>G	Unverified	TTA	AAT	GTG	2	A	G
TCGA-13-0923	MARS	ENST00000262027	p.Y314C	c.941A>G	Verified	gag	tat	ggt	2	A	G
TCGA-13-1510	MBD4	ENST00000249910	p.K340R	c.1019A>G	Verified	gcc	aaa	gac	2	A	G
TCGA-29-1777	MCF2_ENST	ENST00000519895	p.K526K	c.1578A>G	Unverified	TGT	AAA	CTT	3	A	G
TCGA-13-0714	MCM3AP	ENST00000397708	p.N1781S	c.5342A>G	Unverified	aaa	aac	gat	2	A	G
TCGA-24-1423	MDH2	ENST00000315758	p.K307R	c.920A>G	Verified	ggc	aaa	gtc	2	A	G
TCGA-29-1781	MDN1	ENST00000369393	p.T996A	c.2986A>G	Unverified	TTA	ACA	CAG	1	A	G
TCGA-13-0793	MECOM	ENST00000392736	p.M711V	c.2131A>G	Verified	TCT	ATG	TTC	1	A	G
TCGA-29-1693	MECOM	ENST00000392736	p.N804S	c.2411A>G	Unverified	ACC	AAT	TTA	2	A	G
TCGA-20-1687	MEP1A	ENST00000230588	p.Y113C	c.338A>G	Unverified	CCC	TAT	GAA	2	A	G
TCGA-61-1733	METTL14	ENST00000388822	p.L170L	c.510A>G	Unverified	TAC	TTA	CAA	3	A	G
TCGA-61-1733	METTL14	ENST00000388822	p.L170L	c.510A>G	Unverified	TAC	TTA	CAA	3	A	G
TCGA-61-1910	MFGE8	ENST00000268150	p.Y53C	c.158A>G	Unverified	TCG	TAC	ACC	2	A	G
TCGA-25-1313	MGAM	ENST00000389322	p.K281R	c.842A>G	Verified	TGG	AAG	ACC	2	A	G
TCGA-25-1313	MGAM_ENS	ENST00000549489	p.K281R	c.842A>G	Verified	TGG	AAG	ACC	2	A	G
TCGA-13-1481	MGAT4C	ENST00000332156	p.K305E	c.913A>G	Verified	TTT	AAA	CCA	1	A	G
TCGA-04-1542	MLIP	ENST00000274897	p.N114S	c.341A>G	Verified	GCA	AAC	AAA	2	A	G
TCGA-13-1501	MLL4	ENST00000222270	p.N700S	c.2099A>G	Unverified	ACC	AAC	CAC	2	A	G
TCGA-13-1501	MLL4	ENST00000222270	p.N700S	c.2099A>G	Unverified	ACC	AAC	CAC	2	A	G
TCGA-13-0904	MMRN1	ENST00000394980	p.V1144V	c.3432A>G	Verified	GGA	GTA	TAT	3	A	G
TCGA-09-2044	MOCS1	ENST00000373186	p.K362R	c.1085A>G	Verified	AGG	AAG	AAG	2	A	G
TCGA-13-0890	MPHOSPH1(	ENST00000244230	p.K669R	c.2006A>G	Verified	AAG	AAG	AAA	2	A	G
TCGA-24-0979	MSH5	NM_025259.3	p.Q569Q	c.1707A>G	Verified	cca	caa	gtc	3	A	G
TCGA-10-0930	MTO1	ENST00000370300	p.K245R	c.734A>G	Verified	GCC	AAA	GAG	2	A	G
TCGA-10-0930	MTO1	ENST00000370300	p.K245R	c.734A>G	Verified	GCC	AAA	GAG	2	A	G
TCGA-61-2012	MTTP	ENST00000265517	p.K454R	c.1361A>G	Verified	GCT	AAG	AAG	2	A	G
TCGA-29-1691	MUC16	ENST00000397910	p.K6942R	c.20825A>G	Unverified	ATT	AAG	AGA	2	A	G
TCGA-29-1770	MUC16	ENST00000397910	p.Q4179Q	c.12537A>G	Unverified	TCA	CAA	GGG	3	A	G
TCGA-13-1512	MUC16_ENS	ENST00000397910	p.T14068A	c.42202A>G	Verified	ACA	ACA	GCC	1	A	G
TCGA-29-1691	MUC16_ENS	ENST00000397910	p.K6942R	c.20825A>G	Unverified	ATT	AAG	AGA	2	A	G
TCGA-29-1770	MUC16_ENS	ENST00000397910	p.Q4179Q	c.12537A>G	Unverified	TCA	CAA	GGG	3	A	G
TCGA-13-1499	MUC7	ENST00000304887	p.K122K	c.366A>G	Verified	ACC	AAA	ATT	3	A	G
TCGA-24-2288	MYBPC1	ENST00000361466	p.K1127K	c.3381A>G	Unverified	TGC	AAA	GCA	3	A	G
TCGA-24-1469	MYH1	ENST00000226207	p.S1096G	c.3286A>G	Verified	CAA	AGC	AAG	1	A	G

TCGA-13-0807	MYH1	ENST00000226207	p.K386R	c.1157A>G	Verified	GAC	AAG	GCA	2	A	G
TCGA-04-1343	MYH1	ENST00000226207	p.E1494E	c.4482A>G	Verified	GAG	GAA	TCT	3	A	G
TCGA-13-0792	MYH11	ENST00000338282	p.K414E	c.1240A>G	Verified	ACA	AAA	GAA	1	A	G
TCGA-24-2035	MYLK4	ENST00000274643	p.K374R	c.1121A>G	Unverified	CAG	AAG	AAG	2	A	G
TCGA-13-0791	MYO18B	ENST00000335473	p.K2401R	c.7202A>G	Unverified	GGA	AAG	GAG	2	A	G
TCGA-13-0904	MYST3	ENST00000265713	p.N456S	c.1367A>G	Verified	GAC	AAT	CAG	2	A	G
TCGA-13-1512	NASP	ENST00000341288	p.K645R	c.1934A>G	Verified	CTA	AAG	GAA	2	A	G
TCGA-24-2267	NDUFB2	ENST00000247866	p.I99V	c.295A>G	Verified	GGT	ATC	CCT	1	A	G
TCGA-24-2289	NEFL	ENST00000380781	p.Y43C	c.128A>G	Verified	AGC	TAC	TCG	2	A	G
TCGA-24-2262	NEK6	NM_014397	p.Y259C	c.776A>G	Verified	gac	tac	ccc	2	A	G
TCGA-04-1331	NF1	ENST00000358273	p.K1444E	c.4330A>G	Verified	TCA	AAG	ATA	1	A	G
TCGA-25-2392	NFATC2IP	ENST00000320805	p.I269V	c.805A>G	Verified	AAA	ATC	CGT	1	A	G
TCGA-10-0930	NFKB1	ENST00000226574	p.L70L	c.210A>G	Verified	GGA	CTA	CCT	3	A	G
TCGA-10-0930	NFKB1	ENST00000226574	p.L70L	c.210A>G	Verified	GGA	CTA	CCT	3	A	G
TCGA-13-1507	NIPBL	ENST00000282516	p.T1918A	c.5752A>G	Verified	cca	act	cca	1	A	G
TCGA-13-0924	NIPBL	ENST00000282516	p.K1690R	c.5069A>G	Verified	gaa	aaa	gca	2	A	G
TCGA-13-0795	NKAPL	ENST00000343684	p.K190R	c.569A>G	Verified	AGA	AAG	AAT	2	A	G
TCGA-13-0900	NOTCH2	ENST00000256646	p.Y790C	c.2369A>G	Verified	ggc	tat	aac	2	A	G
TCGA-24-1470	NP_0010739	ENST00000296794	p.L542L	c.1626A>G	Verified	AAT	CTA	CAG	3	A	G
TCGA-23-1114	NPC1L1	ENST00000289547	p.N497S	c.1490A>G	Unverified	AAC	AAC	CGC	2	A	G
TCGA-24-1850	NR2C1	ENST00000333003	p.Q290Q	c.870A>G	Unverified	TCT	CAA	AAT	3	A	G
TCGA-09-2050	NRAP	ENST00000359988	p.S985G	c.2953A>G	Verified	ATT	AGT	TAT	1	A	G
TCGA-20-1685	NUDCD2	ENST00000302764	p.I71V	c.211A>G	Unverified	ACA	ATA	GCT	1	A	G
TCGA-13-0791	NUFIP2	ENST00000225388	p.N416D	c.1246A>G	Verified	TCT	AAT	GGG	1	A	G
TCGA-29-1781	NUP214	ENST00000359428	p.Q70Q	c.210A>G	Unverified	att	caa	aat	3	A	G
TCGA-09-2051	NXF1	ENST00000294172	p.T76A	c.226A>G	Unverified	tat	acc	acc	1	A	G
TCGA-04-1652	OR11A1	ENST00000377149	p.I308V	c.922A>G	Unverified	TGT	ATC	AAA	1	A	G
TCGA-04-1652	OR11A1	ENST00000377149	p.I308V	c.922A>G	Unverified	TGT	ATC	AAA	1	A	G
TCGA-29-1691	OR4A15	ENST00000314706	p.N165S	c.494A>G	Unverified	ATG	AAT	CGT	2	A	G
TCGA-25-2042	OR51E2	ENST00000396950	p.T239A	c.715A>G	Verified	GGA	ACC	TGT	1	A	G
TCGA-24-2289	OR5B3	ENST00000309403	p.K4E	c.10A>G	Unverified	AAT	AAG	ACA	1	A	G
TCGA-29-1768	OR5D14	ENST00000335605	p.V42V	c.126A>G	Unverified	GTG	GTA	GGA	3	A	G
TCGA-13-0884	OR5M3	ENST00000312240	p.T158A	c.472A>G	Verified	GCA	ACA	TTA	1	A	G
TCGA-29-1705	ORC5	ENST00000297431	p.K355K	c.1065A>G	Unverified	cca	aaa	cca	3	A	G

TCGA-29-1705	OSBPL11	ENST00000296220	p.K692R	c.2075A>G	Unverified	GAT	AAG	GCC	2	A	G
TCGA-29-1693	PAK3	ENST00000446737	p.N7D	c.19A>G	Unverified	gat	aat	gaa	1	A	G
TCGA-29-1693	PAK3_ENST(	ENST00000360648	p.N7D	c.19A>G	Unverified	GAT	AAT	GAA	1	A	G
TCGA-10-0930	PALM2	ENST00000374531	p.M283V	c.847A>G	Verified	TTT	ATG	GGC	1	A	G
TCGA-10-0930	PALM2	ENST00000374531	p.M283V	c.847A>G	Verified	TTT	ATG	GGC	1	A	G
TCGA-24-1844	PAPD4	ENST00000296783	p.I266V	c.796A>G	Unverified	CCA	ATT	GTG	1	A	G
TCGA-23-1116	PAPPA2	ENST00000367662	p.Y893C	c.2678A>G	Verified	CAG	TAT	GTG	2	A	G
TCGA-29-1763	PARD3	ENST00000374789	p.L369L	c.1107A>G	Unverified	CAA	CTA	TCC	3	A	G
TCGA-29-1769	PARP14	ENST00000310276	p.K129E	c.385A>G	Unverified	AAT	AAA	ATG	1	A	G
TCGA-29-1769	PARP14_EN!	ENST00000474629	p.K292E	c.874A>G	Unverified	AAT	AAA	ATG	1	A	G
TCGA-04-1362	PC	ENST00000393955	p.E728E	c.2184A>G	Verified	GCC	GAA	GAG	3	A	G
TCGA-23-1029	PCDH10	ENST00000264360	p.N816S	c.2447A>G	Unverified	CAC	AAC	CAG	2	A	G
TCGA-24-1846	PCDH15_EN	ENST00000395438	p.E1465E	c.4395A>G	Unverified	CTG	GAA	TTG	3	A	G
TCGA-09-2049	PCDH9	ENST00000377865	p.N1014S	c.3041A>G	Verified	TGT	AAC	TCA	2	A	G
TCGA-29-1701	PCDHA13	ENST00000289272	p.N87S	c.260A>G	Unverified	GTG	AAT	TCT	2	A	G
TCGA-24-1422	PCDHA2	ENST00000378132	p.E144E	c.432A>G	Verified	CCC	GAA	TCA	3	A	G
TCGA-13-0903	PCDHA3	ENST00000378129	p.I212M	c.636A>G	Verified	CTA	ATA	ACA	3	A	G
TCGA-25-1326	PCDHA9	ENST00000378122	p.K138R	c.413A>G	Unverified	CAA	AAG	AAT	2	A	G
TCGA-13-1408	PDSS2	ENST00000369037	p.L168L	c.504A>G	Unverified	AAT	TTA	AAT	3	A	G
TCGA-61-2012	PDZRN3	NM_015009.1	p.K935R	c.2804A>G	Unverified	acc	aag	agg	2	A	G
TCGA-25-1318	PEG3	ENST00000326441	p.K290K	c.870A>G	Verified	CTA	AAA	ACT	3	A	G
TCGA-25-1318	PEG3	ENST00000326441	p.K290K	c.870A>G	Verified	CTA	AAA	ACT	3	A	G
TCGA-23-1118	PENK	ENST00000314922	p.R216G	c.646A>G	Verified	AGA	AGA	GTA	1	A	G
TCGA-13-1408	PER2	ENST00000254657	p.K553R	c.1658A>G	Unverified	AAG	AAA	GCT	2	A	G
TCGA-24-2290	PEX5L	ENST00000467460	p.T405A	c.1213A>G	Verified	TAT	ACT	AAC	1	A	G
TCGA-24-2290	PEX5L	ENST00000467460	p.T405A	c.1213A>G	Verified	TAT	ACT	AAC	1	A	G
TCGA-13-0903	PGAP1	ENST00000354764	p.K315R	c.944A>G	Verified	TCC	AAG	AAG	2	A	G
TCGA-24-2289	PHF8	ENST00000338154	p.T578A	c.1732A>G	Unverified	agt	acg	aaa	1	A	G
TCGA-23-1029	PHLDB2	ENST00000412622	p.N382S	c.1145A>G	Unverified	AGG	AAC	TTC	2	A	G
TCGA-23-1029	PHLDB2_EN	ENST00000393923	p.N409S	c.1226A>G	Unverified	AGG	AAC	TTC	2	A	G
TCGA-23-1029	PHLDB2_EN	ENST00000431670	p.N382S	c.1145A>G	Unverified	AGG	AAC	TTC	2	A	G
TCGA-30-1714	PIK3C3	ENST00000262039	p.S710G	c.2128A>G	Unverified	cca	agt	gag	1	A	G
TCGA-24-2035	PIK3CA_ENS	ENST00000263967	p.E176E	c.528A>G	Verified	CCA	GAA	TTG	3	A	G
TCGA-13-0885	PIP	ENST00000291009	p.K96E	c.286A>G	Verified	CCA	AAA	ACC	1	A	G

TCGA-24-2267	PIR	ENST00000380420	p.K46E	c.136A>G	Verified	ttt	aaa	gga	1	A	G
TCGA-13-1481	PKDREJ	ENST00000253255	p.N1385S	c.4154A>G	Verified	TTC	AAT	AGG	2	A	G
TCGA-09-1665	PLCB1	ENST00000338037	p.Y460C	c.1379A>G	Verified	atg	tat	aaa	2	A	G
TCGA-25-1326	PLCB4	NM_000933.2	p.K1014K	c.3042A>G	Verified	gtc	aaa	gag	3	A	G
TCGA-09-2050	PLCE1	ENST00000260766	p.N912S	c.2735A>G	Verified	CTT	AAT	AAC	2	A	G
TCGA-61-1910	PLD5	ENST00000366545	p.N367S	c.1100A>G	Unverified	GGG	AAT	GAT	2	A	G
TCGA-61-1910	PLD5_ENST(	ENST00000536534	p.N459S	c.1376A>G	Unverified	GGG	AAT	GAT	2	A	G
TCGA-23-1114	PMS1	ENST00000441310	p.Y750C	c.2249A>G	Unverified	cca	tat	aga	2	A	G
TCGA-23-1110	PNMA5	ENST00000361887	p.Q141Q	c.423A>G	Verified	ccc	caa	gtt	3	A	G
TCGA-61-1913	PODN	ENST00000312553	p.N197S	c.590A>G	Unverified	CTC	AAT	TAC	2	A	G
TCGA-13-0913	POLA1	NM_016937.1	p.K988K	c.2964A>G	Verified	acg	aaa	gag	3	A	G
TCGA-13-0885	POLD2	ENST00000406581	p.Y332C	c.995A>G	Verified	CCC	TAC	CAG	2	A	G
TCGA-24-1845	POLD2	ENST00000406581	p.N406S	c.1217A>G	Unverified	GGC	AAC	ACC	2	A	G
TCGA-24-1845	POLD2_ENS'	ENST00000406581	p.N406S	c.1217A>G	Unverified	GGC	AAC	ACC	2	A	G
TCGA-61-2102	POLI	NM_007195.1	p.K271E	c.811A>G	Verified	gaa	aaa	gaa	1	A	G
TCGA-30-1857	POP1	ENST00000349693	p.M214V	c.640A>G	Unverified	CAT	ATG	GTC	1	A	G
TCGA-04-1338	POPDC3	ENST00000254765	p.I77M	c.231A>G	Verified	GAC	ATA	TTT	3	A	G
TCGA-30-1857	POTEB	ENST00000327822	p.K363K	c.1089A>G	Unverified	GAA	AAA	CAG	3	A	G
TCGA-23-1123	POTED	ENST00000299443	p.K151R	c.452A>G	Verified	GGT	AAA	GTC	2	A	G
TCGA-23-1123	POTED	ENST00000299443	p.K151R	c.452A>G	Verified	GGT	AAA	GTC	2	A	G
TCGA-23-1111	PRAMEF12	ENST00000357726	p.L355L	c.1065A>G	Unverified	GAC	TTA	GAG	3	A	G
TCGA-20-0991	PRKAA2	ENST00000371244	p.T506A	c.1516A>G	Verified	ACA	ACT	GCA	1	A	G
TCGA-20-0991	PRKAA2_EN	ENST00000371244	p.T506A	c.1516A>G	Verified	ACA	ACT	GCA	1	A	G
TCGA-09-2051	PRKCQ	ENST00000263125	p.N663S	c.1988A>G	Unverified	agc	aat	ttc	2	A	G
TCGA-13-0795	PRKDC	NM_006904	p.K2717R	c.8150A>G	Verified	gtg	aaa	ggt	2	A	G
TCGA-24-1847	PRPF40A	ENST00000354363	p.M290V	c.868A>G	Unverified	AAA	ATG	ATT	1	A	G
TCGA-24-1847	PRPF40A_EI	ENST00000359961	p.M421V	c.1261A>G	Unverified	AAA	ATG	ATT	1	A	G
TCGA-24-1847	PRPF40A_EI	ENST00000410080	p.M394V	c.1180A>G	Unverified	AAA	ATG	ATT	1	A	G
TCGA-20-0990	PRPH2	ENST00000230381	p.Y184C	c.551A>G	Verified	CGC	TAC	CTG	2	A	G
TCGA-13-0760	PSD2	ENST00000274710	p.K530R	c.1589A>G	Verified	GGC	AAG	AGG	2	A	G
TCGA-24-2288	PTGIS	ENST00000244043	p.N302S	c.905A>G	Unverified	AAG	AAT	CCT	2	A	G
TCGA-23-1120	PTPN12	ENST00000248594	p.T755A	c.2263A>G	Verified	CCA	ACA	GAA	1	A	G
TCGA-04-1338	PXDNL_ENS'	ENST00000356297	p.Y578C	c.1733A>G	Verified	AGA	TAT	GAA	2	A	G
TCGA-29-1691	Q8N9Z5_HU	ENST00000314855	p.S126G	c.376A>G	Unverified	CGA	AGT	GCA	1	A	G

TCGA-30-1857	Q96RW6_HI	ENST00000295249	p.L58L	c.174A>G	Unverified	CTG	TTA	GAA	3	A	G
TCGA-25-2400	RABGGTB	ENST00000319942	p.L329L	c.987A>G	Verified	GAG	CTA	GTG	3	A	G
TCGA-13-1489	RALGAPA1_	ENST00000307138	p.M2060V	c.6178A>G	Verified	CCA	ATG	TCT	1	A	G
TCGA-13-0795	RANBP17	ENST00000523189	p.E452E	c.1356A>G	Verified	TGT	GAA	TAT	3	A	G
TCGA-23-1022	RANBP6	ENST00000259569	p.K106R	c.317A>G	Verified	gtt	aag	tta	2	A	G
TCGA-24-1469	RBM12	ENST00000374104	p.M395V	c.1183A>G	Verified	AAT	ATG	GGA	1	A	G
TCGA-13-0924	RBM14	ENST00000310137	p.Y114C	c.341A>G	Verified	GAC	TAC	GCG	2	A	G
TCGA-61-2012	RBM15	ENST00000369784	p.K431R	c.1292A>G	Verified	GCC	AAA	TTA	2	A	G
TCGA-61-1915	RBM27	ENST00000265271	p.L416L	c.1248A>G	Unverified	CCT	TTA	CCC	3	A	G
TCGA-24-1469	RBM28	ENST00000223073	p.K688R	c.2063A>G	Verified	GGC	AAA	GTG	2	A	G
TCGA-13-0893	RC3H1	ENST00000258349	p.L106L	c.318A>G	Verified	GAA	TTA	GCA	3	A	G
TCGA-09-1665	REL	ENST00000295025	p.K135K	c.405A>G	Verified	gaa	aaa	cag	3	A	G
TCGA-23-1114	REN	ENST00000272190	p.T151A	c.451A>G	Unverified	TCA	ACA	GGG	1	A	G
TCGA-09-1674	RIF1	ENST00000444746	p.K318R	c.953A>G	Unverified	ctc	aag	ttg	2	A	G
TCGA-61-1998	RIF1	ENST00000444746	p.K925K	c.2775A>G	Unverified	aat	aaa	cag	3	A	G
TCGA-61-2095	RILP	ENST00000301336	p.K259K	c.777A>G	Unverified	CTC	AAA	GCC	3	A	G
TCGA-20-1683	RIN2	NM_018993.2	p.Y665C	c.1994A>G	Unverified	atg	tat	tcg	2	A	G
TCGA-20-1683	RIN2_ENST0	ENST00000255006	p.Y714C	c.2141A>G	Unverified	ATG	TAT	TCG	2	A	G
TCGA-29-1691	RLIM	ENST00000332687	p.K9K	c.27A>G	Unverified	gac	aaa	gga	3	A	G
TCGA-13-0791	RNF145	ENST00000274542	p.I196V	c.586A>G	Verified	ACA	ATT	GTT	1	A	G
TCGA-13-0791	RNMTL1	ENST00000304478	p.K73R	c.218A>G	Verified	GAG	AAA	CAA	2	A	G
TCGA-09-2049	ROR1	ENST00000371079	p.S864G	c.2590A>G	Verified	ACT	AGC	ACT	1	A	G
TCGA-23-1022	RORB	ENST00000376896	p.N134S	c.401A>G	Verified	CTG	AAC	AAC	2	A	G
TCGA-13-0883	ROS1	ENST00000368508	p.N1495D	c.4483A>G	Verified	GTT	AAT	GAC	1	A	G
TCGA-61-1914	RPS6KA3	ENST00000379565	p.V492V	c.1476A>G	Unverified	GTA	GTA	ACA	3	A	G
TCGA-13-0905	RRAGC	ENST00000373001	p.Y165C	c.494A>G	Verified	GCC	TAC	AAA	2	A	G
TCGA-23-1022	RRAGD	ENST00000369415	p.I226V	c.676A>G	Verified	tca	ata	ttt	1	A	G
TCGA-04-1343	RRBP1	ENST00000360807	p.E640E	c.1920A>G	Verified	CCA	GAA	CTC	3	A	G
TCGA-25-1318	RRM2B	ENST00000251810	p.S25G	c.73A>G	Verified	gaa	agt	gaa	1	A	G
TCGA-25-1318	RRM2B	ENST00000251810	p.S25G	c.73A>G	Verified	gaa	agt	gaa	1	A	G
TCGA-24-0979	RSPH1	ENST00000291536	p.E300E	c.900A>G	Verified	gaa	gaa	gaa	3	A	G
TCGA-09-2049	RXRA	ENST00000481739	p.Y397C	c.1190A>G	Verified	gtc	tat	gcg	2	A	G
TCGA-13-0906	RYR2	ENST00000366574	p.N4399S	c.13196A>G	Verified	CCA	AAT	GCT	2	A	G
TCGA-09-2051	SAMD7	ENST00000335556	p.N57S	c.170A>G	Unverified	CCA	AAC	ACA	2	A	G

TCGA-30-1857	SCAI	ENST00000373549	p.N504S	c.1511A>G	Unverified	AAC	AAT	CCT	2	A	G
TCGA-13-0714	SCCPDH	ENST00000366510	p.T67A	c.199A>G	Verified	CCA	ACA	CTG	1	A	G
TCGA-13-0886	SCEL	ENST00000349847	p.K75R	c.224A>G	Verified	AGG	AAA	GTA	2	A	G
TCGA-04-1337	SEC24B	ENST00000399100	p.Y426C	c.1277A>G	Verified	GGC	TAT	GGC	2	A	G
TCGA-29-1761	SETX	ENST00000224140	p.E1139E	c.3417A>G	Unverified	ATT	GAA	GAA	3	A	G
TCGA-29-1761	SG269_HUN	ENST00000312493	p.K397R	c.1190A>G	Unverified	GAT	AAA	GAT	2	A	G
TCGA-29-1761	SgK269	ENST00000312493	p.K397R	c.1190A>G	Unverified	GAT	AAA	GAT	2	A	G
TCGA-24-1849	SGOL2	ENST00000357799	p.E78E	c.234A>G	Unverified	ACT	GAA	AAG	3	A	G
TCGA-29-1695	SH3RF3	ENST00000309415	p.Y247C	c.740A>G	Unverified	AGC	TAT	ATC	2	A	G
TCGA-13-0924	SIGLEC6	ENST00000346477	p.Q355Q	c.1065A>G	Verified	GTG	CAA	AAC	3	A	G
TCGA-61-2094	SIN3A	ENST00000394947	p.Y558C	c.1673A>G	Unverified	agc	tat	cga	2	A	G
TCGA-13-0760	SIPA1L1	ENST00000555818	p.Y504C	c.1511A>G	Verified	AAC	TAT	TTT	2	A	G
TCGA-20-1686	SIPA1L2	ENST00000366630	p.N627S	c.1880A>G	Unverified	AAC	AAT	GAG	2	A	G
TCGA-24-2288	SKAP2	ENST00000345317	p.K350E	c.1048A>G	Unverified	CCT	AAA	GCC	1	A	G
TCGA-30-1856	SKOR1	ENST00000389002	p.N873D	c.2617A>G	Unverified	TGT	AAC	GAA	1	A	G
TCGA-30-1856	SKOR1_ENS	ENST00000380035	p.N917D	c.2749A>G	Unverified	TGT	AAC	GAA	1	A	G
TCGA-24-2290	SLC16A7	ENST00000261187	p.Y271C	c.812A>G	Verified	CCA	TAT	GCT	2	A	G
TCGA-24-2290	SLC16A7	ENST00000261187	p.Y271C	c.812A>G	Verified	CCA	TAT	GCT	2	A	G
TCGA-24-1435	SLC26A3	ENST00000340010	p.I613M	c.1839A>G	Verified	CAG	ATA	GAA	3	A	G
TCGA-24-1844	SLC31A1	ENST00000374212	p.M81V	c.241A>G	Unverified	GCA	ATG	TTC	1	A	G
TCGA-13-1489	SLC38A1	ENST00000398637	p.K306E	c.916A>G	Verified	AAA	AAA	ATG	1	A	G
TCGA-13-0920	SLC44A4	ENST00000229729	p.Y302C	c.905A>G	Verified	GCC	TAC	CAG	2	A	G
TCGA-13-0760	SLC5A3	ENST00000381151	p.M200V	c.598A>G	Verified	CTT	ATG	ATT	1	A	G
TCGA-30-1718	SLC6A5	ENST00000525748	p.N358S	c.1073A>G	Unverified	GTT	AAT	TTC	2	A	G
TCGA-23-1110	SLITRK5	ENST00000325089	p.V925V	c.2775A>G	Verified	TTT	GTA	GAA	3	A	G
TCGA-61-1915	SLX4	ENST00000294008	p.E132E	c.396A>G	Unverified	AGT	GAA	CCG	3	A	G
TCGA-24-1431	SMG5	ENST00000361813	p.E718E	c.2154A>G	Verified	TGT	GAA	CTG	3	A	G
TCGA-61-1910	SMG7	ENST00000347615	p.E404E	c.1212A>G	Unverified	CAT	GAA	GAG	3	A	G
TCGA-61-1910	SMG7_ENST	ENST00000507469	p.E404E	c.1212A>G	Unverified	CAT	GAA	GAG	3	A	G
TCGA-13-0887	SORL1	ENST00000260197	p.N466S	c.1397A>G	Verified	ATC	AAT	TGT	2	A	G
TCGA-23-1021	SORL1	ENST00000260197	p.Y728C	c.2183A>G	Verified	GGC	TAC	CGG	2	A	G
TCGA-25-2393	SP140_ENST	ENST00000392045	p.I230V	c.688A>G	Verified	CAA	ATA	GAT	1	A	G
TCGA-23-1029	SPG21	ENST00000204566	p.N203S	c.608A>G	Unverified	TTG	AAT	TGT	2	A	G
TCGA-61-1899	SPICE1	ENST00000295872	p.I529V	c.1585A>G	Unverified	CAT	ATT	TTT	1	A	G

TCGA-61-1899	SPICE1_ENS	ENST00000295872_v68	p.I529V	c.1585A>G	Unverified	CAT	ATT	TTT	1	A	G
TCGA-29-1776	SQLE	ENST00000265896	p.I561V	c.1681A>G	Unverified	GTA	ATA	TTT	1	A	G
TCGA-04-1651	SREBF1	ENST00000355815	p.I373V	c.1117A>G	Unverified	AAA	ATC	ATT	1	A	G
TCGA-04-1651	SREBF1	ENST00000355815	p.I373V	c.1117A>G	Unverified	AAA	ATC	ATT	1	A	G
TCGA-24-1616	SRGAP1	ENST00000355086	p.Y633C	c.1898A>G	Verified	AGG	TAC	CTC	2	A	G
TCGA-61-2102	SRRM2	ENST00000301740	p.R940G	c.2818A>G	Verified	AGT	AGG	GTG	1	A	G
TCGA-09-1665	ST6GALNAC	ENST00000328299	p.E117E	c.351A>G	Verified	GAA	GAA	GAT	3	A	G
TCGA-29-1699	STAT6	ENST00000300134	p.N430S	c.1289A>G	Unverified	gac	aat	gcc	2	A	G
TCGA-13-0887	STK31	ENST00000355870	p.S586G	c.1756A>G	Verified	TAT	AGT	CAA	1	A	G
TCGA-13-1481	STON1-GTF	ENST00000394754	p.V871V	c.2613A>G	Verified	TCA	GTA	ATA	3	A	G
TCGA-29-1690	STRN3	NM_014574.2	p.V195V	c.585A>G	Unverified	gat	gta	cgg	3	A	G
TCGA-29-1690	STRN3_ENS	ENST00000357479	p.V195V	c.585A>G	Unverified	GAT	GTA	CGG	3	A	G
TCGA-25-2401	STXBP3	ENST00000370008	p.I108V	c.322A>G	Verified	TAT	ATT	TAC	1	A	G
TCGA-13-0885	SULT2A1	ENST00000222002	p.T85A	c.253A>G	Verified	TAT	ACA	GCA	1	A	G
TCGA-13-0762	SUPT6H	ENST00000314616	p.N800S	c.2399A>G	Verified	GTC	AAT	GGT	2	A	G
TCGA-13-0762	SUPT6H	ENST00000314616	p.N800S	c.2399A>G	Verified	GTC	AAT	GGT	2	A	G
TCGA-29-1711	SYNE2	ENST00000358025	p.Q796Q	c.2388A>G	Unverified	ATA	CAA	AAT	3	A	G
TCGA-29-1711	SYNE2	ENST00000358025	p.Q796Q	c.2388A>G	Unverified	ATA	CAA	AAT	3	A	G
TCGA-24-1474	TAF3	ENST00000344293	p.K706R	c.2117A>G	Verified	AAA	AAG	GAG	2	A	G
TCGA-29-1690	TAOK1	ENST00000261716	p.I540V	c.1618A>G	Unverified	CAT	ATT	CAG	1	A	G
TCGA-29-1690	TAOK1_ENS	ENST00000261716	p.I540V	c.1618A>G	Unverified	CAT	ATT	CAG	1	A	G
TCGA-04-1649	TARDBP	ENST00000240185	p.T157A	c.469A>G	Unverified	GAA	ACA	CAA	1	A	G
TCGA-24-1422	TAS2R46	ENST00000533467	p.K122R	c.365A>G	Verified	TTA	AAG	AGG	2	A	G
TCGA-29-1703	TDRD6	ENST00000316081	p.I841M	c.2523A>G	Unverified	GGG	ATA	CAG	3	A	G
TCGA-24-1616	TEDDM1	ENST00000367565	p.K237R	c.710A>G	Verified	CCC	AAA	GAA	2	A	G
TCGA-61-1904	TEP1	ENST00000262715	p.I1721V	c.5161A>G	Unverified	GGA	ATC	TCT	1	A	G
TCGA-04-1530	TFRC	ENST00000360110	p.Y563C	c.1688A>G	Verified	GAT	TAT	CCT	2	A	G
TCGA-29-1702	TH1L	ENST00000344018	p.I328V	c.982A>G	Unverified	ctt	atc	cgc	1	A	G
TCGA-13-0887	THAP2	ENST00000308086	p.N4S	c.11A>G	Verified	ACC	AAT	TGC	2	A	G
TCGA-29-1784	THRA	ENST00000394121	p.L324L	c.972A>G	Unverified	ctg	cta	atg	3	A	G
TCGA-29-1784	THRA_ENST	ENST00000450525	p.L324L	c.972A>G	Unverified	CTG	CTA	ATG	3	A	G
TCGA-29-1763	THUMPD2	ENST00000260619	p.Y229C	c.686A>G	Unverified	ATT	TAC	TCT	2	A	G
TCGA-24-1844	TLK1	NM_012290	p.T235A	c.703A>G	Unverified	agt	aca	caa	1	A	G
TCGA-24-1844	TLK1_ENST0	ENST00000431350	p.T283A	c.847A>G	Unverified	AGT	ACA	CAA	1	A	G

TCGA-04-1638	TLN2	ENST00000306829	p.K2334E	c.7000A>G	Unverified	CCA	AAA	CAA	1	A	G
TCGA-04-1342	TLR4	ENST00000355622	p.N282S	c.845A>G	Verified	TGC	AAT	TTG	2	A	G
TCGA-20-0991	TMC1	ENST00000297784	p.N620S	c.1859A>G	Verified	TGC	AAT	GTT	2	A	G
TCGA-24-1470	TMEM131	ENST00000186436	p.K1272K	c.3816A>G	Verified	ACT	CAA	GGT	3	A	G
TCGA-20-1685	TMEM132B	ENST00000299308	p.E753E	c.2259A>G	Unverified	GGT	GAA	GGA	3	A	G
TCGA-23-2077	TMEM2	ENST00000377044	p.Q15Q	c.45A>G	Verified	CTC	CAA	CCT	3	A	G
TCGA-23-1021	TMEM67	ENST00000453321	p.N89S	c.266A>G	Verified	TCT	AAT	AAT	2	A	G
TCGA-24-2267	TMEM68	ENST00000334667	p.K143E	c.427A>G	Unverified	GCT	AAA	ATA	1	A	G
TCGA-61-1906	TMEM87A	ENST00000389834	p.Y319C	c.956A>G	Unverified	GGA	TAT	GGC	2	A	G
TCGA-61-1906	TMEM87A	ENST00000389834	p.Y319C	c.956A>G	Unverified	GGA	TAT	GGC	2	A	G
TCGA-13-0916	TNFAIP3	ENST00000237289	p.N776S	c.2327A>G	Verified	tgc	aac	ggc	2	A	G
TCGA-24-1604	TNRC6A	ENST00000395799	p.N528S	c.1583A>G	Unverified	TCT	AAT	TAC	2	A	G
TCGA-13-0923	TOP2B	ENST00000435706	p.Y857C	c.2570A>G	Verified	tgg	tat	att	2	A	G
TCGA-29-1764	TOX3	ENST00000219746	p.K239R	c.716A>G	Unverified	GGC	AAG	AAG	2	A	G
TCGA-29-1764	TOX3_ENST(	ENST00000407228	p.K234R	c.701A>G	Unverified	GGC	AAG	AAG	2	A	G
TCGA-04-1356	TP53	ENST00000269305	p.Y220C	c.659A>G	Verified	CCC	TAT	GAG	2	A	G
TCGA-04-1361	TP53	ENST00000269305	p.Y234C	c.701A>G	Verified	CAC	TAC	AAC	2	A	G
TCGA-04-1361	TP53	ENST00000269305	p.Y234C	c.701A>G	Verified	CAC	TAC	AAC	2	A	G
TCGA-13-0903	TP53	ENST00000269305	p.Y205C	c.614A>G	Verified	GAG	TAT	TTG	2	A	G
TCGA-24-1464	TP53	ENST00000269305	p.N239S	c.716A>G	Verified	TGT	AAC	AGT	2	A	G
TCGA-24-1616	TP53	ENST00000269305	p.Y236C	c.707A>G	Verified	AAC	TAC	ATG	2	A	G
TCGA-61-2012	TP53	ENST00000269305	p.Y220C	c.659A>G	Verified	CCC	TAT	GAG	2	A	G
TCGA-04-1356	TP53_ENST(	ENST00000269305	p.Y220C	c.659A>G	Verified	CCC	TAT	GAG	2	A	G
TCGA-61-2012	TP53_ENST(	ENST00000269305	p.Y220C	c.659A>G	Verified	CCC	TAT	GAG	2	A	G
TCGA-04-1356	TP53_ENST(	ENST00000413465	p.Y220C	c.659A>G	Verified	CCC	TAT	GAG	2	A	G
TCGA-61-2012	TP53_ENST(	ENST00000413465	p.Y220C	c.659A>G	Verified	CCC	TAT	GAG	2	A	G
TCGA-04-1356	TP53_ENST(	ENST00000545858	p.Y127C	c.380A>G	Verified	CCC	TAT	GAG	2	A	G
TCGA-61-2012	TP53_ENST(	ENST00000545858	p.Y127C	c.380A>G	Verified	CCC	TAT	GAG	2	A	G
TCGA-23-1123	TPR	ENST00000367478	p.L454L	c.1362A>G	Verified	AGT	TTA	TCT	3	A	G
TCGA-23-1123	TPR	ENST00000367478	p.L454L	c.1362A>G	Verified	AGT	TTA	TCT	3	A	G
TCGA-13-0885	TRA2B	ENST00000453386	p.R62G	c.184A>G	Verified	AGA	AGA	AGC	1	A	G
TCGA-30-1891	TRA2B	ENST00000453386	p.N180S	c.539A>G	Unverified	GCC	AAT	GGA	2	A	G
TCGA-04-1652	TRAK2	ENST00000332624	p.Q205Q	c.615A>G	Unverified	TCT	CAA	GGG	3	A	G
TCGA-04-1652	TRAK2	ENST00000332624	p.Q205Q	c.615A>G	Unverified	TCT	CAA	GGG	3	A	G

TCGA-13-0923	TRIP11	ENST00000267622	p.L390L	c.1170A>G	Verified	AGA	CTA	CAA	3	A	G
TCGA-29-1691	TRPM4	ENST00000252826	p.K492E	c.1474A>G	Unverified	CTA	AAA	GGG	1	A	G
TCGA-30-1855	TSHZ1	ENST00000322038	p.K809R	c.2426A>G	Unverified	CCC	AAG	TCC	2	A	G
TCGA-13-0904	TTN_ENST0(	ENST00000356127	p.N8026S	c.24077A>G	Verified	ATT	AAT	GAT	2	A	G
TCGA-23-1122	TUBGCP2	ENST00000252936	p.Y423C	c.1268A>G	Unverified	GAT	TAC	AAC	2	A	G
TCGA-23-2078	TUBGCP2	ENST00000252936	p.K770R	c.2309A>G	Unverified	ATG	AAA	TTA	2	A	G
TCGA-29-1705	TUBGCP6	ENST00000248846	p.I667V	c.1999A>G	Unverified	GAA	ATT	GCA	1	A	G
TCGA-13-0755	TXNIP	ENST00000369317	p.K166R	c.497A>G	Verified	GAA	AAG	AAA	2	A	G
TCGA-04-1343	TYW3	ENST00000370867	p.T76A	c.226A>G	Verified	GTT	ACA	CAC	1	A	G
TCGA-24-1845	UBAP2L	ENST00000361546	p.T708A	c.2122A>G	Unverified	TCA	ACA	TCT	1	A	G
TCGA-24-1845	UBAP2L_EN	ENST00000343815	p.T708A	c.2122A>G	Unverified	TCA	ACA	TCT	1	A	G
TCGA-24-1845	UBAP2L_EN	ENST00000456955	p.T204A	c.610A>G	Unverified	TCA	ACA	TCT	1	A	G
TCGA-61-1907	UBE2O	ENST00000319380	p.T967A	c.2899A>G	Unverified	GCT	ACC	TCA	1	A	G
TCGA-61-1907	UBE2O_ENS	ENST00000319380	p.T967A	c.2899A>G	Unverified	GCT	ACC	TCA	1	A	G
TCGA-13-0887	UBL4B	ENST00000334179	p.N64S	c.191A>G	Verified	CCC	AAT	GCC	2	A	G
TCGA-04-1651	UNC5C	ENST00000453304	p.E132E	c.396A>G	Unverified	GTG	GAA	GAA	3	A	G
TCGA-04-1651	UNC5C	ENST00000453304	p.E132E	c.396A>G	Unverified	GTG	GAA	GAA	3	A	G
TCGA-13-0903	UNC5D	ENST00000287272	p.E311E	c.933A>G	Verified	AGC	GAA	TGG	3	A	G
TCGA-20-0991	USH2A	ENST00000307340	p.N2560S	c.7679A>G	Verified	TCC	AAT	GGG	2	A	G
TCGA-61-2109	USH2A	ENST00000307340	p.N4244S	c.12731A>G	Verified	TGG	AAT	TCA	2	A	G
TCGA-24-2289	USP18	ENST00000215794	p.K37R	c.110A>G	Unverified	atg	aag	aga	2	A	G
TCGA-29-1766	USP54	NM_152586.2	p.S242G	c.724A>G	Unverified	aga	agt	ttg	1	A	G
TCGA-29-1766	USP54_ENS	ENST00000408019	p.S1154G	c.3460A>G	Unverified	AGA	AGT	TTG	1	A	G
TCGA-29-1783	VASH1	ENST00000167106	p.K145E	c.433A>G	Unverified	ATT	AAG	AAG	1	A	G
TCGA-29-1783	VASH1_ENS	ENST00000554237	p.K145E	c.433A>G	Unverified	ATT	AAG	AAG	1	A	G
TCGA-24-1616	VCAN	ENST00000265077	p.T2243A	c.6727A>G	Verified	CCA	ACA	ATT	1	A	G
TCGA-24-1849	VPS13B	ENST00000358544	p.L2557L	c.7671A>G	Unverified	ctt	cta	gag	3	A	G
TCGA-24-1849	VPS13B_ENS	ENST00000357162	p.L2532L	c.7596A>G	Unverified	CTT	CTA	GAG	3	A	G
TCGA-30-1718	WBP11	ENST00000261167	p.E300E	c.900A>G	Unverified	AAT	GAA	GAA	3	A	G
TCGA-13-1481	WDR1	ENST00000400001	p.N545S	c.1634A>G	Verified	GAC	AAT	GAA	2	A	G
TCGA-23-1124	WIF1	ENST00000286574	p.K364R	c.1091A>G	Verified	CTT	AAA	AAG	2	A	G
TCGA-13-1489	YSK4_ENST0	ENST00000375845	p.S204G	c.610A>G	Verified	CGA	AGC	ATC	1	A	G
TCGA-04-1652	ZC3H12C	ENST00000278590	p.N353S	c.1058A>G	Unverified	TCC	AAT	GAT	2	A	G
TCGA-04-1652	ZC3H12C	ENST00000278590	p.N353S	c.1058A>G	Unverified	TCC	AAT	GAT	2	A	G

TCGA-20-0991	ZC3H4	ENST00000253048	p.K991R	c.2972A>G	Verified	CCC	AAG	CAG	2	A	G
TCGA-13-0714	ZCCHC11	ENST00000257177	p.N744S	c.2231A>G	Verified	ACC	AAT	GGT	2	A	G
TCGA-30-1855	ZCCHC11	ENST00000257177	p.E151E	c.453A>G	Unverified	TCA	GAA	AAA	3	A	G
TCGA-24-1563	ZFX	ENST00000379188	p.N314S	c.941A>G	Verified	TTA	AAT	GTT	2	A	G
TCGA-29-1764	ZFYVE1	ENST00000556143	p.K209E	c.625A>G	Unverified	TTT	AAA	ACC	1	A	G
TCGA-13-1507	ZMYM6	ENST00000357182	p.N527S	c.1580A>G	Verified	CCA	AAT	TTG	2	A	G
TCGA-24-2267	ZMYM6	ENST00000357182	p.K223K	c.669A>G	Verified	TCA	AAA	TTT	3	A	G
TCGA-23-1124	ZNF16	ENST00000394909	p.Y545C	c.1634A>G	Verified	CCC	TAT	GAA	2	A	G
TCGA-13-1497	ZNF182	ENST00000396965	p.N444S	c.1331A>G	Verified	tca	aac	ctt	2	A	G
TCGA-10-0930	ZNF189	ENST00000339664	p.Q307Q	c.921A>G	Verified	cat	caa	aga	3	A	G
TCGA-10-0930	ZNF189	ENST00000339664	p.Q307Q	c.921A>G	Verified	cat	caa	aga	3	A	G
TCGA-13-0760	ZNF202	ENST00000336139	p.T387A	c.1159A>G	Verified	ACT	ACA	CCC	1	A	G
TCGA-29-1775	ZNF227	ENST00000313040	p.K157E	c.469A>G	Unverified	CCT	AAA	GGA	1	A	G
TCGA-30-1856	ZNF251_ENS	ENST00000292562_v68	p.K459R	c.1376A>G	Unverified	GAG	AAG	CCC	2	A	G
TCGA-13-1408	ZNF281	ENST00000367353	p.N384S	c.1151A>G	Unverified	TCA	AAC	CAT	2	A	G
TCGA-23-1123	ZNF285	ENST00000330997	p.K27R	c.80A>G	Verified	GAT	AAA	GCC	2	A	G
TCGA-23-1123	ZNF285	ENST00000330997	p.K27R	c.80A>G	Verified	GAT	AAA	GCC	2	A	G
TCGA-24-1422	ZNF292	ENST00000339907	p.V412V	c.1236A>G	Verified	ATA	GTA	CGA	3	A	G
TCGA-09-1665	ZNF333	ENST00000292530	p.K308E	c.922A>G	Verified	GAA	AAA	CTC	1	A	G
TCGA-24-0979	ZNF341	ENST00000342427	p.Y601C	c.1802A>G	Verified	CAT	TAT	CTC	2	A	G
TCGA-24-1469	ZNF45	ENST00000269973	p.N76S	c.227A>G	Verified	GAT	AAC	TCC	2	A	G
TCGA-13-1507	ZNF479	ENST00000331162	p.R349G	c.1045A>G	Unverified	ACT	AGA	GAG	1	A	G
TCGA-24-1844	ZNF614	ENST00000270649	p.I123V	c.367A>G	Unverified	CCT	ATA	GTG	1	A	G
TCGA-29-2427	ZNF621	ENST00000339296	p.Y207C	c.620A>G	Verified	CCC	TAT	GAA	2	A	G
TCGA-61-1899	ZNF717	ENST00000422325	p.T86A	c.256A>G	Unverified	GAA	ACC	CCA	1	A	G
TCGA-04-1343	ZNF724P	ENST00000418100	p.Y506C	c.1517A>G	Unverified	CCC	TAC	AAA	2	A	G
TCGA-29-1698	ZNF79	ENST00000342483	p.Y204C	c.611A>G	Unverified	AGT	TAC	TGT	2	A	G
TCGA-25-2401	ZNF804B	ENST00000333190	p.V932V	c.2796A>G	Verified	AGA	GTA	CAA	3	A	G
TCGA-13-0906	ZNF823	ENST00000341191	p.T381A	c.1141A>G	Verified	ATA	ACA	CAC	1	A	G
TCGA-13-0923	ZNF823	ENST00000341191	p.Y527C	c.1580A>G	Verified	CCA	TAT	GAA	2	A	G
TCGA-25-2042	ZRANB1	ENST00000359653	p.N639S	c.1916A>G	Verified	GGT	AAT	GAG	2	A	G
TCGA-29-1691	ZSCAN20	ENST00000326544	p.K985R	c.2954A>G	Unverified	GAG	AAG	CCG	2	A	G
TCGA-04-1356	ZSWIM2	ENST00000295131	p.K546R	c.1637A>G	Verified	ACC	AAA	GGC	2	A	G
TCGA-13-1512	ZZZ3	ENST00000370801	p.E491E	c.1473A>G	Verified	TTT	GAA	TCA	3	A	G

TCGA-23-1117	AAMP	ENST00000248450	p.K254*	c.760A>T	Verified	CTG	AAA	GGG	1	A	T
TCGA-23-1117	AAMP	ENST00000248450	p.K254*	c.760A>T	Verified	CTG	AAA	GGG	1	A	T
TCGA-61-2012	ABCA4	ENST00000370225	p.T1497S	c.4489A>T	Verified	CTC	ACC	ATG	1	A	T
TCGA-09-2050	ABCA5	ENST00000392676	p.S1308S	c.3924A>T	Unverified	CTT	TCA	AGA	3	A	T
TCGA-29-1761	ABCB4	ENST00000359206	p.D180V	c.539A>T	Unverified	GAT	GAC	ATC	2	A	T
TCGA-23-2077	ABCF3	ENST00000429586	p.E518V	c.1553A>T	Verified	CTC	GAG	TCT	2	A	T
TCGA-13-0792	ACOT11	ENST00000343744	p.K144*	c.430A>T	Unverified	TGC	AAG	GCC	1	A	T
TCGA-24-2267	ACSS1	ENST00000323482	p.E145V	c.434A>T	Verified	AGG	GAA	CTA	2	A	T
TCGA-61-1900	ACTRT2	ENST00000378404	p.Q53L	c.158A>T	Unverified	AAC	CAG	AAG	2	A	T
TCGA-61-1740	ACVR1	ENST00000263640	p.I171F	c.511A>T	Unverified	CTC	ATC	ACC	1	A	T
TCGA-13-0900	ADAMTS14	NM_139155.1	p.E608V	c.1823A>T	Verified	gag	gag	tgc	2	A	T
TCGA-13-0913	ADAMTS14	NM_139155.1	p.H635L	c.1904A>T	Unverified	aag	cac	agc	2	A	T
TCGA-24-1844	ADAMTS8	ENST00000257359	p.S508S	c.1524A>T	Unverified	TGC	TCA	GAA	3	A	T
TCGA-24-1844	ADAMTS8_E	ENST00000257359	p.S508S	c.1524A>T	Unverified	TGC	TCA	GAA	3	A	T
TCGA-24-1463	ADCY9	ENST00000294016	p.R1310S	c.3930A>T	Verified	AAG	AGA	CCG	3	A	T
TCGA-13-1509	AGTPBP1	ENST00000376083	p.A1003A	c.3009A>T	Verified	AAT	GCA	ACT	3	A	T
TCGA-04-1356	AIF1	ENST00000376059	p.D66V	c.197A>T	Verified	ATT	GAT	ATC	2	A	T
TCGA-04-1356	AIF1_ENST0	ENST00000376051	p.D78V	c.233A>T	Verified	CCA	GAT	ATC	2	A	T
TCGA-61-2113	AKAP7	NM_016377.2	p.S169S	c.507A>T	Verified	aac	tca	ctt	3	A	T
TCGA-23-1122	ALPK1	ENST00000458497	p.T906S	c.2716A>T	Verified	TGC	ACT	ACC	1	A	T
TCGA-13-0885	ANK2	ENST00000357077	p.N793Y	c.2377A>T	Verified	GCG	AAT	GGC	1	A	T
TCGA-61-1915	ANKRD30A	ENST00000361713	p.E420V	c.1259A>T	Unverified	GAG	GAA	GAT	2	A	T
TCGA-09-2050	ANKRD30A	ENST00000361713	p.T608T	c.1824A>T	Verified	CAA	ACA	TTG	3	A	T
TCGA-13-1497	ANKRD35	ENST00000355594	p.E655V	c.1964A>T	Verified	CGG	GAG	TTT	2	A	T
TCGA-13-0916	ANKRD44	NM_153697.1	p.D410V	c.1229A>T	Verified	aag	gac	aag	2	A	T
TCGA-13-0714	ANKRD5	ENST00000378392	p.R308S	c.924A>T	Unverified	GAG	AGA	ATC	3	A	T
TCGA-24-1845	ANTXR2	ENST00000307333	p.T313T	c.939A>T	Unverified	GCC	ACA	GAA	3	A	T
TCGA-24-1845	ANTXR2_EN	ENST00000403729	p.T313T	c.939A>T	Unverified	GCC	ACA	GAA	3	A	T
TCGA-23-1117	AOC3	ENST00000308423	p.Q434L	c.1301A>T	Verified	GAA	CAG	AAC	2	A	T
TCGA-23-1117	AOC3	ENST00000308423	p.Q434L	c.1301A>T	Verified	GAA	CAG	AAC	2	A	T
TCGA-29-1691	AOX1	ENST00000374700	p.E724V	c.2171A>T	Unverified	CTG	GAA	TAT	2	A	T
TCGA-29-1703	AP1G2	ENST00000397120	p.I468F	c.1402A>T	Unverified	GAC	ATT	TCC	1	A	T
TCGA-13-0923	APAF1	ENST00000551964	p.E146V	c.437A>T	Verified	ggt	gaa	cca	2	A	T
TCGA-29-1776	APOB	ENST00000233242	p.D4169V	c.12506A>T	Unverified	AAG	GAT	AAC	2	A	T

TCGA-61-1907	APPL2	ENST00000258530	p.E661V	c.1982A>T	Unverified	GCA	GAA	TCC	2	A	T
TCGA-24-1844	ARHGAP32	ENST00000392657	p.R296S	c.888A>T	Unverified	GAA	AGA	AAG	3	A	T
TCGA-24-1844	ARHGAP32_	ENST00000310343	p.R645S	c.1935A>T	Unverified	GAA	AGA	AAG	3	A	T
TCGA-23-1031	ARID5B	ENST00000279873	p.E498V	c.1493A>T	Verified	ATA	GAA	GGG	2	A	T
TCGA-61-2095	ARL4A	ENST00000396662	p.E109V	c.326A>T	Unverified	ATG	GAA	GAA	2	A	T
TCGA-13-0761	ART3	ENST00000349321	p.S188S	c.564A>T	Verified	TAT	TCA	GCC	3	A	T
TCGA-13-0761	ART3	ENST00000349321	p.S188S	c.564A>T	Verified	TAT	TCA	GCC	3	A	T
TCGA-24-1417	ASAP1	ENST00000357668	p.K262*	c.784A>T	Unverified	TTG	AAA	CAG	1	A	T
TCGA-23-2078	ASXL3	ENST00000269197	p.T760S	c.2278A>T	Verified	TCC	ATA	AAT	1	A	T
TCGA-13-0714	ASXL3_ENST	ENST00000269197	p.T1480S	c.4438A>T	Verified	ACC	ACG	CTG	1	A	T
TCGA-09-0369	ATG2A	ENST00000377264	p.D308V	c.923A>T	Verified	ACA	GAC	CAC	2	A	T
TCGA-09-0369	ATG2A	ENST00000377264	p.D308V	c.923A>T	Verified	ACA	GAC	CAC	2	A	T
TCGA-13-0904	ATP10D	ENST00000273859	p.D701V	c.2102A>T	Verified	GGT	GAT	GCA	2	A	T
TCGA-61-2012	ATP2B4	ENST00000357681	p.E1152V	c.3455A>T	Verified	GAA	GAG	GAG	2	A	T
TCGA-23-1122	AXDND1	ENST00000367618	p.Q155L	c.464A>T	Verified	TTA	CAG	TCA	2	A	T
TCGA-29-1769	B4GALNT2	ENST00000300404	p.T537S	c.1609A>T	Unverified	AAG	ACC	TAC	1	A	T
TCGA-61-1913	B4GALNT3	ENST00000266383	p.E607V	c.1820A>T	Unverified	GAG	GAA	GAG	2	A	T
TCGA-24-1464	BCAS3	ENST00000390652	p.D811V	c.2432A>T	Verified	TCT	GAT	CGA	2	A	T
TCGA-25-2401	BCL7B	ENST00000223368	p.T52S	c.154A>T	Verified	gtg	aca	gac	1	A	T
TCGA-13-0890	BCORL1	ENST00000218147	p.N134Y	c.400A>T	Verified	agc	aac	agc	1	A	T
TCGA-23-1124	BDKRB2	ENST00000554311	p.I361F	c.1081A>T	Verified	CCC	ATT	CAG	1	A	T
TCGA-24-2290	BMP1	ENST00000306385	p.T780S	c.2338A>T	Verified	AGC	ACC	CCC	1	A	T
TCGA-24-2290	BMP1	ENST00000306385	p.T780S	c.2338A>T	Verified	AGC	ACC	CCC	1	A	T
TCGA-23-1032	BMP15	ENST00000252677	p.Q210L	c.629A>T	Verified	TGT	CAG	CAG	2	A	T
TCGA-13-0793	BRD8	ENST00000254900	p.I364F	c.1090A>T	Verified	ATC	ATC	AAT	1	A	T
TCGA-04-1651	BSN	ENST00000296452	p.A3392A	c.10176A>T	Unverified	CCT	GCA	GTC	3	A	T
TCGA-04-1651	BSN	ENST00000296452	p.A3392A	c.10176A>T	Unverified	CCT	GCA	GTC	3	A	T
TCGA-23-1118	C10orf2	ENST00000311916	p.I104F	c.310A>T	Verified	TTC	ATT	GAC	1	A	T
TCGA-20-0990	C11orf41	ENST00000265654	p.D11V	c.32A>T	Verified	CAG	GAT	CTC	2	A	T
TCGA-30-1891	C11orf82	ENST00000430323	p.I689F	c.2065A>T	Unverified	GAC	ATT	GCA	1	A	T
TCGA-24-1604	C19orf75	ENST00000316401	p.K87*	c.259A>T	Verified	GGG	AAG	AAC	1	A	T
TCGA-61-1733	C1D	ENST00000355848	p.R111*	c.331A>T	Unverified	gac	aga	ggt	1	A	T
TCGA-61-1733	C1D	ENST00000355848	p.R111*	c.331A>T	Unverified	gac	aga	ggt	1	A	T
TCGA-13-1510	C20orf26	ENST00000245957	p.E817V	c.2450A>T	Verified	GAA	GAG	GAT	2	A	T

TCGA-23-1031	C2orf90	ENST00000434864	p.H17L	c.50A>T	Verified	AAA	CAC	CAG	2	A	T
TCGA-61-2012	C4BPA	ENST00000367070	p.E534V	c.1601A>T	Unverified	CCA	GAG	GTG	2	A	T
TCGA-13-0885	C4orf42	ENST00000357591	p.R34W	c.100A>T	Verified	TGC	AGG	GAC	1	A	T
TCGA-23-1116	C5orf45	ENST00000292586	p.P247P	c.741A>T	Unverified	CAG	CCA	AGG	3	A	T
TCGA-24-1850	C6orf146	ENST00000274673	p.H298L	c.893A>T	Unverified	CAA	CAT	ATG	2	A	T
TCGA-24-1850	C6orf72	ENST00000367419	p.S126C	c.376A>T	Unverified	TCC	AGT	TTG	1	A	T
TCGA-13-0760	C9orf129	ENST00000375419	p.A147A	c.441A>T	Verified	ACA	GCA	GCC	3	A	T
TCGA-23-1118	CAPN9	ENST00000354537	p.D580V	c.1739A>T	Verified	GCT	GAC	AAG	2	A	T
TCGA-24-1417	CARS	ENST00000397111	p.A500A	c.1500A>T	Unverified	ACA	GCA	ATT	3	A	T
TCGA-13-0920	CASS4	ENST00000360314	p.S254C	c.760A>T	Verified	GCC	AGC	GTC	1	A	T
TCGA-25-2042	CATSPER2	ENST00000396879	p.H31L	c.92A>T	Verified	GAG	CAT	TTG	2	A	T
TCGA-24-1470	CCDC11	ENST00000398545	p.S77C	c.229A>T	Verified	GAC	AGC	CTT	1	A	T
TCGA-61-1737	CCDC13	ENST00000310232	p.H82L	c.245A>T	Unverified	GAA	CAC	CTT	2	A	T
TCGA-24-1464	CCDC141	ENST00000343876	p.D452V	c.1355A>T	Verified	GAA	GAT	GCA	2	A	T
TCGA-13-0807	CCDC3	ENST00000378825	p.E195V	c.584A>T	Verified	TTT	GAG	GAG	2	A	T
TCGA-13-0884	CCDC33	ENST00000398814	p.M179L	c.535A>T	Verified	CAC	ATG	GCT	1	A	T
TCGA-24-1846	CCDC40	ENST00000397545	p.H349L	c.1046A>T	Unverified	AGT	CAC	GAC	2	A	T
TCGA-24-1846	CCDC40_EN:	ENST00000374877	p.H349L	c.1046A>T	Unverified	AGT	CAC	GAC	2	A	T
TCGA-61-1725	CCDC80	ENST00000206423	p.N556Y	c.1666A>T	Unverified	AAG	AAT	GAG	1	A	T
TCGA-25-2391	CCDC80	ENST00000206423	p.R374S	c.1122A>T	Unverified	GCA	AGA	CCT	3	A	T
TCGA-20-1683	CCDC85A	ENST00000407595	p.G551G	c.1653A>T	Unverified	AAA	GGA	CCA	3	A	T
TCGA-13-1481	CCDC87	ENST00000333861	p.I84F	c.250A>T	Verified	CTC	ATC	AAG	1	A	T
TCGA-24-1846	CCL21	ENST00000259607	p.T112S	c.334A>T	Unverified	AAG	ACT	GGC	1	A	T
TCGA-04-1655	CCT7	ENST00000398422	p.D114V	c.341A>T	Unverified	GAG	GAT	CTG	2	A	T
TCGA-04-1655	CCT7_ENST(	ENST00000258091	p.D318V	c.953A>T	Unverified	GAG	GAT	CTG	2	A	T
TCGA-09-2056	CDH9	ENST00000231021	p.D409V	c.1226A>T	Verified	TAC	GAT	CCA	2	A	T
TCGA-13-0885	CDK5RAP2	ENST00000349780	p.T607S	c.1819A>T	Verified	aag	acc	ttg	1	A	T
TCGA-24-1843	CEP350	ENST00000367607	p.Q2291L	c.6872A>T	Unverified	GAA	CAG	GGA	2	A	T
TCGA-24-1843	CEP350_EN:	ENST00000367607	p.Q2291L	c.6872A>T	Unverified	GAA	CAG	GGA	2	A	T
TCGA-10-0930	CEP89	ENST00000305768	p.H323L	c.968A>T	Verified	GTC	CAT	CGT	2	A	T
TCGA-10-0930	CEP89	ENST00000305768	p.H323L	c.968A>T	Verified	GTC	CAT	CGT	2	A	T
TCGA-13-0886	CGNL1	ENST00000281282	p.R874R	c.2622A>T	Verified	ATA	CGA	CAG	3	A	T
TCGA-25-1326	CHD1L	NM_004284.2	p.S562C	c.1684A>T	Verified	ggg	agc	aga	1	A	T
TCGA-29-1784	CHD9	NM_025134.3	p.H146L	c.437A>T	Unverified	gga	cat	tca	2	A	T

TCGA-13-0795	CHGB	ENST00000378961	p.R469S	c.1407A>T	Verified	GAC	AGA	AAT	3	A	T
TCGA-24-1422	CHST6	ENST00000390664	p.H249L	c.746A>T	Unverified	AGC	CAC	GTA	2	A	T
TCGA-61-2094	CLCA1	ENST00000234701	p.S535C	c.1603A>T	Unverified	CCC	AGT	GGA	1	A	T
TCGA-13-0919	CLCN2	ENST00000265593	p.T679S	c.2035A>T	Unverified	AAC	ACA	GAA	1	A	T
TCGA-04-1367	CLIC6	ENST00000349499	p.D499V	c.1496A>T	Verified	GCA	GAC	CTG	2	A	T
TCGA-29-1698	CLMN	ENST00000298912	p.E698V	c.2093A>T	Unverified	TTG	GAG	ACC	2	A	T
TCGA-13-0920	CLOCK	ENST00000381322	p.Q569L	c.1706A>T	Verified	CAA	CAA	TCA	2	A	T
TCGA-25-2392	CMYA5	ENST00000238522	p.A1595A	c.4785A>T	Verified	CCG	GCA	GTG	3	A	T
TCGA-29-1693	CNIH4	ENST00000465271	p.M111L	c.331A>T	Unverified	CAC	ATG	AAA	1	A	T
TCGA-61-1740	CNTN5	ENST00000528682	p.Q190L	c.569A>T	Unverified	ctg	cag	ttt	2	A	T
TCGA-61-1740	CNTN5_ENS	ENST00000524871	p.Q190L	c.569A>T	Unverified	CTG	CAG	TTT	2	A	T
TCGA-13-0923	CNTNAP2	ENST00000361727	p.T737S	c.2209A>T	Verified	TGC	ACA	GAT	1	A	T
TCGA-09-2050	COG8	ENST00000306875	p.E20V	c.59A>T	Verified	GGC	GAG	GTG	2	A	T
TCGA-13-0893	COL11A1	ENST00000358392	p.E290V	c.869A>T	Verified	TCT	GAA	AAA	2	A	T
TCGA-20-1687	COL4A1	ENST00000375820	p.E1083V	c.3248A>T	Unverified	GGA	GAA	AAA	2	A	T
TCGA-24-1563	COL4A1	ENST00000375820	p.Q1051L	c.3152A>T	Verified	GGG	CAG	GCA	2	A	T
TCGA-20-1687	COL4A1_EN	ENST00000375815	p.E726V	c.2177A>T	Unverified	GGA	GAA	AAA	2	A	T
TCGA-13-1501	COL4A6	ENST00000334504	p.G22G	c.66A>T	Verified	GCG	GGA	GAG	3	A	T
TCGA-13-1501	COL4A6	ENST00000334504	p.G22G	c.66A>T	Verified	GCG	GGA	GAG	3	A	T
TCGA-13-0913	COL5A1	ENST00000371817	p.P741P	c.2223A>T	Verified	CCT	CCA	GGA	3	A	T
TCGA-24-1104	CR1	ENST00000400960	p.S1682C	c.5044A>T	Verified	CCC	AGC	TAT	1	A	T
TCGA-23-2078	CRTC2	ENST00000368633	p.P491P	c.1473A>T	Verified	TCC	CCA	AGT	3	A	T
TCGA-24-2288	CSMD3	ENST00000297405	p.S1367C	c.4099A>T	Verified	atc	agt	gac	1	A	T
TCGA-13-0900	CSMD3	ENST00000297405	p.E689V	c.2066A>T	Verified	ttt	gaa	tgc	2	A	T
TCGA-29-1781	CTAGE1	ENST00000391403	p.R214S	c.642A>T	Unverified	AAA	AGA	ACA	3	A	T
TCGA-29-1781	CTAGE1_EN	ENST00000391403	p.R214S	c.642A>T	Unverified	AAA	AGA	ACA	3	A	T
TCGA-24-1845	CTNNA3	NM_013266.1	p.E180V	c.539A>T	Unverified	aag	gag	ctg	2	A	T
TCGA-24-1845	CTNNA3_EN	ENST00000433211	p.E180V	c.539A>T	Unverified	AAG	GAG	CTG	2	A	T
TCGA-09-1665	CTSE	ENST00000358184	p.T282T	c.846A>T	Verified	GAC	ACA	GGG	3	A	T
TCGA-13-1509	CUBN	ENST00000377833	p.S1254C	c.3760A>T	Verified	GAC	AGC	ATG	1	A	T
TCGA-04-1367	CUX1	ENST00000292535	p.D14V	c.41A>T	Verified	CTC	GAT	GCC	2	A	T
TCGA-04-1367	CUX1_ENST(	ENST00000292538	p.D25V	c.74A>T	Verified	CTC	GAT	GCC	2	A	T
TCGA-13-0923	CYP24A1	ENST00000216862	p.N237Y	c.709A>T	Verified	GTG	AAC	TTC	1	A	T
TCGA-04-1367	CYTIP	ENST00000264192	p.S248C	c.742A>T	Verified	AAG	AGC	TGG	1	A	T

TCGA-29-1775	DENND2A	ENST00000275884	p.Q545L	c.1634A>T	Unverified	AAG	CAG	GCG	2	A	T
TCGA-29-1699	DENND5A	ENST00000328194	p.T688S	c.2062A>T	Unverified	TGG	ACG	AAA	1	A	T
TCGA-61-2008	DHX34	ENST00000328771	p.Q52L	c.155A>T	Verified	cgt	cag	ggt	2	A	T
TCGA-29-1775	DNAH6	ENST00000237449	p.T497S	c.1489A>T	Unverified	GGG	ACC	CTT	1	A	T
TCGA-29-1785	DNAH7	ENST00000312428	p.Q1594L	c.4781A>T	Unverified	CTT	CAA	GTA	2	A	T
TCGA-29-1785	DNAH7	ENST00000312428	p.Q1594L	c.4781A>T	Unverified	CTT	CAA	GTA	2	A	T
TCGA-23-1122	DNAH8	NM_001371.1	p.R3878S	c.11634A>T	Verified	gca	aga	aag	3	A	T
TCGA-29-1775	DNHL1	ENST00000314395	p.T76S	c.226A>T	Unverified	GGG	ACC	CTT	1	A	T
TCGA-24-1845	DOCK7	ENST00000340370	p.D62V	c.185A>T	Unverified	GAA	GAT	TAC	2	A	T
TCGA-24-2280	DPYD	ENST00000370192	p.R78*	c.232A>T	Unverified	ATG	AGA	TGC	1	A	T
TCGA-13-1510	DROSHA	ENST00000511367	p.D929V	c.2786A>T	Unverified	GGA	GAC	AGA	2	A	T
TCGA-04-1336	DSC2	ENST00000280904	p.K270*	c.808A>T	Verified	GAC	AAA	GAT	1	A	T
TCGA-04-1367	DSCAM	ENST00000400454	p.I1332F	c.3994A>T	Verified	ACG	ATT	GAT	1	A	T
TCGA-23-1123	DSG4	ENST00000308128	p.R967S	c.2901A>T	Verified	GAG	AGA	GTA	3	A	T
TCGA-23-1123	DSG4	ENST00000308128	p.R967S	c.2901A>T	Verified	GAG	AGA	GTA	3	A	T
TCGA-04-1652	DST	ENST00000370765	p.H315L	c.944A>T	Unverified	AAT	CAT	AAA	2	A	T
TCGA-04-1652	DST	ENST00000370765	p.H315L	c.944A>T	Unverified	AAT	CAT	AAA	2	A	T
TCGA-04-1652	DST_ENST0(	ENST00000244364	p.H315L	c.944A>T	Unverified	AAT	CAT	AAA	2	A	T
TCGA-04-1652	DST_ENST0(	ENST00000244364	p.H315L	c.944A>T	Unverified	AAT	CAT	AAA	2	A	T
TCGA-04-1652	DST_ENST0(	ENST00000370765	p.H315L	c.944A>T	Unverified	AAT	CAT	AAA	2	A	T
TCGA-04-1652	DST_ENST0(	ENST00000370765	p.H315L	c.944A>T	Unverified	AAT	CAT	AAA	2	A	T
TCGA-04-1652	DST_ENST0(	ENST00000370769	p.H641L	c.1922A>T	Unverified	AAT	CAT	AAA	2	A	T
TCGA-04-1652	DST_ENST0(	ENST00000370769	p.H641L	c.1922A>T	Unverified	AAT	CAT	AAA	2	A	T
TCGA-13-0761	DYX1C1	ENST00000321149	p.E352V	c.1055A>T	Unverified	CTG	GAA	TTA	2	A	T
TCGA-13-0761	DYX1C1	ENST00000321149	p.E352V	c.1055A>T	Unverified	CTG	GAA	TTA	2	A	T
TCGA-13-0791	EEF1D	ENST00000442189	p.I377F	c.1129A>T	Verified	aag	atc	tgg	1	A	T
TCGA-61-1915	EFEMP1	ENST00000394554	p.K43*	c.127A>T	Unverified	TGC	AAA	GAT	1	A	T
TCGA-61-1914	ELAVL2	ENST00000380117	p.G300G	c.900A>T	Unverified	TTT	GGA	GCT	3	A	T
TCGA-23-2077	ELMO2	ENST00000396391	p.H284L	c.851A>T	Verified	GCC	CAT	CAG	2	A	T
TCGA-13-0920	ENSG00000Z	ENST00000377171	p.E309V	c.926A>T	Verified	TGG	GAA	TGG	2	A	T
TCGA-23-1124	ENSG00000Z	ENST00000400226	p.S539C	c.1615A>T	Verified	CTG	AGT	TGG	1	A	T
TCGA-13-1488	ENSG00000Z	ENST00000402465	p.D34V	c.101A>T	Verified	AGA	GAT	GTG	2	A	T
TCGA-04-1331	ENSG00000Z	ENST00000513104	p.D210V	c.629A>T	Unverified	AAA	GAC	TCC	2	A	T
TCGA-13-1488	ENSG00000Z	ENST00000513104	p.D210V	c.629A>T	Unverified	AAA	GAC	TCC	2	A	T

TCGA-23-1029	ENTHD1	ENST00000325157	p.K184*	c.550A>T	Unverified	GAG	AAG	AAG	1	A	T
TCGA-29-1783	ESF1	ENST00000202816	p.M743L	c.2227A>T	Unverified	CTC	ATG	AAA	1	A	T
TCGA-25-1313	EVL	ENST00000392920	p.S331C	c.991A>T	Verified	CGG	AGC	AAC	1	A	T
TCGA-24-1849	EXOC7	ENST00000357231	p.D626V	c.1877A>T	Unverified	CCA	GAC	ACA	2	A	T
TCGA-29-1761	F5	ENST00000367797	p.D552V	c.1655A>T	Unverified	TTT	GAT	GAG	2	A	T
TCGA-04-1530	FABP3	ENST00000373713	p.D18V	c.53A>T	Unverified	TTC	GAT	GAC	2	A	T
TCGA-23-1117	FAM153B	ENST00000253490	p.T104T	c.312A>T	Verified	AGC	ACA	TGG	3	A	T
TCGA-23-1117	FAM153B	ENST00000253490	p.T104T	c.312A>T	Verified	AGC	ACA	TGG	3	A	T
TCGA-13-1510	FAM46C	ENST00000369448	p.Q106L	c.317A>T	Unverified	TTT	CAG	CTG	2	A	T
TCGA-25-2392	FAM55D	ENST00000375478	p.T312S	c.934A>T	Verified	ATG	ACA	TCC	1	A	T
TCGA-23-1110	FARP1	ENST00000319562	p.E353V	c.1058A>T	Unverified	aaa	gaa	gga	2	A	T
TCGA-24-1104	FAT3_ENST(	ENST00000298047_v61	p.I2103F	c.6307A>T	Verified	CTG	ATT	TAT	1	A	T
TCGA-24-1847	FAT4	ENST00000330166	p.T4249S	c.12745A>T	Unverified	CAC	ACT	TTT	1	A	T
TCGA-24-1847	FAT4_ENST(	ENST00000394329	p.T4306S	c.12916A>T	Unverified	CAC	ACT	TTT	1	A	T
TCGA-13-0886	FBXO16	ENST00000380254	p.E148V	c.443A>T	Verified	TTT	GAG	CAG	2	A	T
TCGA-13-0755	FEN1	ENST00000305885	p.E318V	c.953A>T	Verified	tct	gag	gag	2	A	T
TCGA-13-1489	FLG2	ENST00000388718	p.E443V	c.1328A>T	Verified	TTC	GAA	CAA	2	A	T
TCGA-10-0930	FNDC5	ENST00000373471	p.M66L	c.196A>T	Verified	ACC	ATG	AAA	1	A	T
TCGA-10-0930	FNDC5	ENST00000373471	p.M66L	c.196A>T	Verified	ACC	ATG	AAA	1	A	T
TCGA-25-1313	FOXB1_ENS'	ENST00000396057	p.I172F	c.514A>T	Unverified	GCC	ATC	GAG	1	A	T
TCGA-09-1674	FOXJ2	ENST00000162391	p.E562V	c.1685A>T	Unverified	aat	gag	gag	2	A	T
TCGA-13-0916	FRG2	ENST00000378763	p.S52C	c.154A>T	Verified	TCC	AGT	GAG	1	A	T
TCGA-09-2049	GABBR1	ENST00000377034	p.G364G	c.1092A>T	Verified	GTG	GGA	CTT	3	A	T
TCGA-13-0885	GABRA5	ENST00000335625	p.D97V	c.290A>T	Verified	ATA	GAC	GTG	2	A	T
TCGA-23-1111	GABRE	NM_021990	p.T12S	c.34A>T	Unverified	cag	acc	tgg	1	A	T
TCGA-23-1111	GABRE_ENS'	ENST00000370328	p.T125S	c.373A>T	Unverified	CAG	ACC	TGG	1	A	T
TCGA-20-1683	GABRG2	ENST00000356592	p.R233S	c.699A>T	Unverified	ACA	AGA	TCC	3	A	T
TCGA-61-1910	GBP6	ENST00000370456	p.S253S	c.759A>T	Unverified	GTG	TCA	GAA	3	A	T
TCGA-13-0906	GGA3	ENST00000245541	p.K575*	c.1723A>T	Unverified	CCG	AAG	GGG	1	A	T
TCGA-24-2290	GGTLC2	ENST00000215938	p.S94S	c.282A>T	Verified	CCC	TCA	CCT	3	A	T
TCGA-24-2290	GGTLC2	ENST00000215938	p.S94S	c.282A>T	Verified	CCC	TCA	CCT	3	A	T
TCGA-13-0890	GIF	ENST00000257248	p.I153L	c.457A>T	Verified	CCG	ATA	GCC	1	A	T
TCGA-23-1120	GLI1	ENST00000228682	p.R1093S	c.3279A>T	Unverified	ctg	aga	tcc	3	A	T
TCGA-29-1761	GLT8D2	ENST00000360814	p.D125V	c.374A>T	Unverified	CCA	GAC	TCA	2	A	T

TCGA-13-0791	GNAT3	ENST00000398291	p.H322L	c.965A>T	Verified	TCC	CAC	ATG	2	A	T
TCGA-13-1497	GPLD1	ENST00000230036	p.A286A	c.858A>T	Verified	att	gca	tgt	3	A	T
TCGA-13-0923	GPR98	ENST00000405460	p.E2642V	c.7925A>T	Verified	GGA	GAG	ATT	2	A	T
TCGA-61-1910	GPR98	ENST00000405460	p.S578S	c.1734A>T	Unverified	ATA	TCA	AGG	3	A	T
TCGA-13-0906	GRIN2A	ENST00000396573	p.T1223S	c.3667A>T	Verified	ccc	acc	tat	1	A	T
TCGA-23-2078	GRIP1	ENST00000398016	p.P382P	c.1146A>T	Verified	AGC	CCA	CGT	3	A	T
TCGA-13-1497	GYG1	ENST00000345003	p.D113V	c.338A>T	Verified	GAT	GAT	CTT	2	A	T
TCGA-13-0886	GZMA	ENST00000274306	p.I53F	c.157A>T	Verified	ACC	ATC	TGT	1	A	T
TCGA-23-1120	HAO1	ENST00000378789	p.R46S	c.138A>T	Verified	TCC	AGA	TGG	3	A	T
TCGA-13-1489	HAUS1	ENST00000282058	p.M276L	c.826A>T	Verified	ATG	ATG	GAA	1	A	T
TCGA-20-0991	HAUS6	ENST00000380502	p.H888L	c.2663A>T	Verified	TTG	CAT	ACT	2	A	T
TCGA-24-1849	HCK	ENST00000538448	p.E479V	c.1436A>T	Unverified	GAG	GAG	CGG	2	A	T
TCGA-24-1849	HCK_ENST0(	ENST00000375852	p.E500V	c.1499A>T	Unverified	AGC	CAG	TAC	2	A	T
TCGA-23-1022	HCN4	ENST00000261917	p.E321V	c.962A>T	Verified	GTG	GAG	GAC	2	A	T
TCGA-04-1362	HEPHL1	ENST00000315765	p.S1011C	c.3031A>T	Verified	GAG	AGC	TTT	1	A	T
TCGA-25-2392	HEXIM1	ENST00000332499	p.N17Y	c.49A>T	Verified	AGC	AAC	TGT	1	A	T
TCGA-29-1777	HHAT	ENST00000367010	p.T489S	c.1465A>T	Unverified	CAG	ACC	TAC	1	A	T
TCGA-29-1702	HIRA	ENST00000263208	p.D869V	c.2606A>T	Unverified	GCA	GAC	TTT	2	A	T
TCGA-13-0762	HMCN1	ENST00000271588	p.E1917V	c.5750A>T	Verified	CAT	GAA	CCA	2	A	T
TCGA-13-0762	HMCN1	ENST00000271588	p.E1917V	c.5750A>T	Verified	CAT	GAA	CCA	2	A	T
TCGA-24-1104	HOXB1	ENST00000239174	p.S78C	c.232A>T	Verified	CCC	AGC	TCC	1	A	T
TCGA-13-1499	HSD3B2	ENST00000369416	p.E354V	c.1061A>T	Verified	GTG	GAG	TGG	2	A	T
TCGA-13-0913	HSPBAP1	ENST00000306103	p.P114P	c.342A>T	Verified	gga	cca	ttt	3	A	T
TCGA-24-0975	HSPH1	ENST00000320027	p.M174L	c.520A>T	Unverified	GAC	ATG	ACA	1	A	T
TCGA-13-0920	HTN3	ENST00000526767	p.M13L	c.37A>T	Verified	CTC	ATG	CTT	1	A	T
TCGA-29-1775	IDO1	ENST00000522495	p.R58S	c.174A>T	Unverified	gaa	aga	gtt	3	A	T
TCGA-29-1761	IFIH1	ENST00000263642	p.N324Y	c.970A>T	Unverified	AAG	AAT	ATC	1	A	T
TCGA-13-0904	IGDCC4	ENST00000352385	p.E156V	c.467A>T	Verified	GAG	GAG	AAC	2	A	T
TCGA-13-0913	IL7R	ENST00000303115	p.T56S	c.166A>T	Verified	CTG	ACC	TGT	1	A	T
TCGA-13-0904	IQCC	ENST00000291358	p.D304V	c.911A>T	Verified	GAC	GAT	GGA	2	A	T
TCGA-29-1762	ITGA8	ENST00000378076	p.R763*	c.2287A>T	Unverified	ATC	AGA	AGT	1	A	T
TCGA-04-1655	ITPR1	ENST00000456211	p.H594L	c.1781A>T	Unverified	AAA	CAC	ATT	2	A	T
TCGA-04-1655	ITPR1_ENST	ENST00000302640	p.H594L	c.1781A>T	Unverified	AAA	CAC	ATT	2	A	T
TCGA-04-1655	ITPR1_ENST	ENST00000357086	p.H609L	c.1826A>T	Unverified	AAA	CAC	ATT	2	A	T

TCGA-13-1498	ITPR2	ENST00000381340	p.T2444S	c.7330A>T	Unverified	ATG	ACT	TTA	1	A	T
TCGA-25-1318	IVL	ENST00000368764	p.M280L	c.838A>T	Verified	CAG	ATG	GGG	1	A	T
TCGA-25-1318	IVL	ENST00000368764	p.M280L	c.838A>T	Verified	CAG	ATG	GGG	1	A	T
TCGA-13-0761	IVNS1ABP	ENST00000367498	p.A97A	c.291A>T	Verified	AAA	GCA	GAT	3	A	T
TCGA-13-0761	IVNS1ABP	ENST00000367498	p.A97A	c.291A>T	Verified	AAA	GCA	GAT	3	A	T
TCGA-61-1725	JAK3	ENST00000458235	p.I955F	c.2863A>T	Unverified	AAC	ATC	CTC	1	A	T
TCGA-61-1725	JAK3_ENST0	ENST00000458235	p.I955F	c.2863A>T	Unverified	AAC	ATC	CTC	1	A	T
TCGA-29-1699	KCNH1	ENST00000271751	p.T205S	c.613A>T	Unverified	AAG	ACT	CCC	1	A	T
TCGA-04-1338	KCNJ15	ENST00000398930	p.K319*	c.955A>T	Verified	TCC	AAA	AAT	1	A	T
TCGA-04-1649	KIAA0020	ENST00000397885	p.H24L	c.71A>T	Unverified	TTT	CAT	AAA	2	A	T
TCGA-25-1313	KIAA0100	ENST00000005905	p.N804Y	c.2410A>T	Verified	CGG	AAC	CCC	1	A	T
TCGA-13-1512	KIAA0467_E	ENST00000372442	p.E2284V	c.6851A>T	Verified	GCT	GAG	CGG	2	A	T
TCGA-24-1422	KIAA0913_E	ENST00000398706	p.R1044W	c.3130A>T	Unverified	CAG	AGG	TCT	1	A	T
TCGA-29-1783	KIAA0913_E	ENST00000398706	p.S1261C	c.3781A>T	Unverified	AAC	AGC	AGC	1	A	T
TCGA-24-1422	KIAA0913_E	ENST00000412131	p.R1039W	c.3115A>T	Unverified	CAG	AGG	TCT	1	A	T
TCGA-29-1783	KIAA0913_E	ENST00000412131	p.S1256C	c.3766A>T	Unverified	AAC	AGC	AGC	1	A	T
TCGA-13-0903	KIAA1407	ENST00000295878	p.E246V	c.737A>T	Verified	GAG	GAA	GAG	2	A	T
TCGA-13-0714	KIF4A	ENST00000374403	p.P71P	c.213A>T	Verified	gcg	cca	ctc	3	A	T
TCGA-24-2280	KRTAP20-2	ENST00000330798	p.G57G	c.171A>T	Unverified	TAT	GGA	AGA	3	A	T
TCGA-24-1843	LAMA1	ENST00000389658	p.D2794V	c.8381A>T	Unverified	AGT	GAT	GGC	2	A	T
TCGA-29-1781	LASS3	ENST00000284382	p.K171*	c.511A>T	Unverified	CCC	AAA	CAG	1	A	T
TCGA-20-1687	LASS4	ENST00000251363	p.E24V	c.71A>T	Unverified	ACA	GAG	CTA	2	A	T
TCGA-25-2392	LCOR	ENST00000371103	p.K150*	c.448A>T	Verified	CTC	AAA	GTT	1	A	T
TCGA-13-1512	LHX4	ENST00000263726	p.R259R	c.777A>T	Verified	TTC	CGA	GAG	3	A	T
TCGA-29-1784	LILRA1	ENST00000251372	p.T119T	c.357A>T	Unverified	GTG	ACA	GGA	3	A	T
TCGA-61-1906	LIN9	ENST00000328205	p.T171T	c.513A>T	Unverified	AAA	ACA	AGA	3	A	T
TCGA-61-1906	LIN9	ENST00000328205	p.T171T	c.513A>T	Unverified	AAA	ACA	AGA	3	A	T
TCGA-04-1336	LNX2	ENST00000316334	p.E25V	c.74A>T	Verified	ttt	gaa	tgt	2	A	T
TCGA-13-1510	LOC65121	ENST00000332296	p.G253G	c.759A>T	Unverified	CAA	GGA	CGG	3	A	T
TCGA-24-1849	LPHN3	ENST00000512091	p.N380Y	c.1138A>T	Unverified	TGG	AAT	AAC	1	A	T
TCGA-24-1849	LPHN3_ENS'	ENST00000512091	p.N380Y	c.1138A>T	Unverified	TGG	AAT	AAC	1	A	T
TCGA-24-1849	LPHN3_ENS'	ENST00000514591	p.N380Y	c.1138A>T	Unverified	TGG	AAT	AAC	1	A	T
TCGA-29-1775	LRBA	ENST00000357115	p.P2497P	c.7491A>T	Unverified	AGT	CCA	TTG	3	A	T
TCGA-29-1775	LRFN2	ENST00000338305	p.E281V	c.842A>T	Unverified	CGT	GAG	GAG	2	A	T

TCGA-24-2289	LRP1B	ENST00000389484	p.D2299V	c.6896A>T	Unverified	GTG	GAC	CAG	2	A	T
TCGA-61-2094	LRP6	ENST00000261349	p.G1467G	c.4401A>T	Unverified	ACA	GGA	GCA	3	A	T
TCGA-30-1857	LRRC16B	ENST00000342740	p.S342C	c.1024A>T	Unverified	CTG	AGC	AAG	1	A	T
TCGA-04-1651	LRRIQ4	ENST00000340806	p.Q435L	c.1304A>T	Unverified	AAG	CAA	CTT	2	A	T
TCGA-04-1651	LRRIQ4	ENST00000340806	p.Q435L	c.1304A>T	Unverified	AAG	CAA	CTT	2	A	T
TCGA-24-1845	LTBR	ENST00000228918	p.D147V	c.440A>T	Unverified	TCT	GAC	TGC	2	A	T
TCGA-13-0920	LUZP4	ENST00000371920	p.Q94L	c.281A>T	Verified	tcc	caa	aaa	2	A	T
TCGA-24-2035	MACF1	ENST00000360115	p.M3554L	c.10660A>T	Verified	ATG	ATG	GAA	1	A	T
TCGA-24-2035	MACF1_ENS	ENST00000361689	p.M3052L	c.9154A>T	Verified	ATG	ATG	GAA	1	A	T
TCGA-61-2113	MAGI1_ENS	ENST00000402939	p.E1324V	c.3971A>T	Unverified	CCA	GAG	AAG	2	A	T
TCGA-04-1331	MALL	ENST00000272462	p.T98S	c.292A>T	Verified	GGG	ACC	ACT	1	A	T
TCGA-04-1367	MAN1B1	ENST00000371589	p.E117V	c.350A>T	Unverified	GAG	GAA	GAG	2	A	T
TCGA-13-0900	MAP4	ENST00000360240	p.D16V	c.47A>T	Verified	CCA	GAC	ATT	2	A	T
TCGA-23-1022	MATN2	ENST00000520016	p.E246V	c.737A>T	Verified	CTG	GAG	CAT	2	A	T
TCGA-61-1914	MBOAT1	ENST00000324607	p.R108*	c.322A>T	Unverified	CAC	AGA	TAT	1	A	T
TCGA-61-2095	MCM10	ENST00000361282	p.H374L	c.1121A>T	Verified	GAT	CAT	CCT	2	A	T
TCGA-61-2102	MDM1	ENST00000303145	p.Q667L	c.2000A>T	Verified	TTG	CAG	TTA	2	A	T
TCGA-13-0883	MED19	ENST00000337672	p.Q169L	c.506A>T	Verified	ATT	CAG	CCT	2	A	T
TCGA-24-1423	MGAM_ENS	ENST00000549489	p.Q520L	c.1559A>T	Verified	AAT	CAA	GTA	2	A	T
TCGA-24-1845	MGC42105	ENST00000326035	p.I283F	c.847A>T	Unverified	AGC	ATC	CTC	1	A	T
TCGA-25-1313	MGEA5	ENST00000361464	p.E818V	c.2453A>T	Verified	GCT	GAG	AAA	2	A	T
TCGA-13-1509	MGST2	ENST00000265498	p.N138Y	c.412A>T	Verified	CTC	AAT	ATT	1	A	T
TCGA-13-1507	MLH1	ENST00000231790	p.N187Y	c.559A>T	Verified	cac	aat	gca	1	A	T
TCGA-23-1029	MLLT10_EN	ENST00000307729	p.D379V	c.1136A>T	Unverified	ACA	GAC	TCA	2	A	T
TCGA-23-1029	MLLT10_EN	ENST00000377072_v68	p.D379V	c.1136A>T	Unverified	ACA	GAC	TCA	2	A	T
TCGA-24-1103	MLLT4	ENST00000366809	p.R1419*	c.4255A>T	Verified	CGG	AGA	GAG	1	A	T
TCGA-13-0755	MPP1	ENST00000369534	p.G83G	c.249A>T	Verified	atg	gga	atc	3	A	T
TCGA-24-1469	MPP5	ENST00000261681	p.N615Y	c.1843A>T	Verified	AAG	AAT	CCA	1	A	T
TCGA-13-1510	MRE11A	ENST00000323977	p.T199T	c.597A>T	Unverified	gta	aca	atg	3	A	T
TCGA-24-2288	MRPL54	ENST00000330133	p.G28G	c.84A>T	Unverified	TCC	GGA	AGA	3	A	T
TCGA-09-2049	MTL5	ENST00000255087	p.H443L	c.1328A>T	Verified	AGT	CAC	GAT	2	A	T
TCGA-23-1032	MTMR8	ENST00000374852	p.N143Y	c.427A>T	Verified	ccc	aac	aga	1	A	T
TCGA-23-1117	MTTP	ENST00000265517	p.Q872L	c.2615A>T	Verified	CAT	CAA	GAG	2	A	T
TCGA-23-1117	MTTP	ENST00000265517	p.Q872L	c.2615A>T	Verified	CAT	CAA	GAG	2	A	T

TCGA-24-1847	MUC17	ENST00000306151	p.Q1078L	c.3233A>T	Unverified	TCT	CAA	GCC	2	A	T
TCGA-25-1326	MUC17	ENST00000306151	p.S2182S	c.6546A>T	Unverified	CTT	TCA	ACA	3	A	T
TCGA-13-1507	MYH1	ENST00000226207	p.D754V	c.2261A>T	Verified	ATT	GAC	ATT	2	A	T
TCGA-13-0885	MYH2	ENST00000245503	p.Q1634L	c.4901A>T	Verified	ATC	CAG	CTG	2	A	T
TCGA-29-1784	MYH4	ENST00000255381	p.K1026*	c.3076A>T	Unverified	ACC	AAA	GCT	1	A	T
TCGA-36-1577	MYH4	ENST00000255381	p.A1694A	c.5082A>T	Verified	AGG	GCA	TCC	3	A	T
TCGA-04-1347	MYH8	ENST00000403437	p.R1132R	c.3396A>T	Verified	TCC	CGA	GCC	3	A	T
TCGA-61-2012	MYO15A	ENST00000205890	p.A2129A	c.6387A>T	Unverified	CTG	GCA	CAG	3	A	T
TCGA-13-0793	MYO1A	ENST00000300119	p.T676T	c.2028A>T	Verified	aag	aca	aag	3	A	T
TCGA-25-2042	MYO7B	ENST00000272666	p.H1225L	c.3674A>T	Unverified	ATG	CAC	ATC	2	A	T
TCGA-24-1604	MYOT	ENST00000239926	p.D209V	c.626A>T	Unverified	CAA	GAC	TCG	2	A	T
TCGA-29-2427	MYST4	ENST00000287239	p.Q1194L	c.3581A>T	Verified	TTC	CAG	CAT	2	A	T
TCGA-13-1481	MYT1L	ENST00000399161	p.H486L	c.1457A>T	Verified	AGC	CAT	GTC	2	A	T
TCGA-24-2267	NBAS	ENST00000281513	p.I2150F	c.6448A>T	Verified	GAC	ATT	GAG	1	A	T
TCGA-13-0906	NBPF7	ENST00000401014	p.D273V	c.818A>T	Verified	CAG	GAT	GCT	2	A	T
TCGA-23-1809	NCAM2	ENST00000400546	p.D200V	c.599A>T	Unverified	CGT	GAT	ATC	2	A	T
TCGA-23-1029	NCCRP1	ENST00000339852	p.G222G	c.666A>T	Unverified	AGA	GGA	CCC	3	A	T
TCGA-23-1118	NCOA3	ENST00000371998	p.D359V	c.1076A>T	Verified	aat	gat	cga	2	A	T
TCGA-24-2289	NDRG4	ENST00000394279	p.Q111L	c.332A>T	Unverified	TCG	CAG	TTT	2	A	T
TCGA-04-1331	NDUFAF2	ENST00000296597	p.S16S	c.48A>T	Unverified	CTG	TCA	AGG	3	A	T
TCGA-13-0893	NEUROD1	ENST00000295108	p.K39*	c.115A>T	Verified	AAG	AAG	GAG	1	A	T
TCGA-13-0762	NEUROD4	ENST00000242994	p.K74*	c.220A>T	Verified	AAG	AAA	AAG	1	A	T
TCGA-13-0762	NEUROD4	ENST00000242994	p.K74*	c.220A>T	Verified	AAG	AAA	AAG	1	A	T
TCGA-61-1740	NGLY1	ENST00000280700	p.D335V	c.1004A>T	Unverified	ACA	GAC	CAT	2	A	T
TCGA-61-1910	NICN1	ENST00000273598	p.G92G	c.276A>T	Unverified	GAG	GGA	GCC	3	A	T
TCGA-23-1111	NIPSNAP3A	ENST00000374767	p.H195L	c.584A>T	Unverified	GTT	CAT	GTT	2	A	T
TCGA-23-1031	NLRP14	ENST00000299481	p.G745G	c.2235A>T	Verified	AAT	GGA	GTA	3	A	T
TCGA-09-2051	NNT	ENST00000264663	p.D919V	c.2756A>T	Unverified	ATT	GAC	ATG	2	A	T
TCGA-29-1693	NOMO3	ENST00000399336	p.D262V	c.785A>T	Unverified	CAA	GAC	GAG	2	A	T
TCGA-24-1470	NPVF	ENST00000222674	p.T16S	c.46A>T	Verified	GCC	ACT	TCA	1	A	T
TCGA-13-1499	NQO2	ENST00000380430	p.S108C	c.322A>T	Verified	TTC	AGC	GTG	1	A	T
TCGA-04-1367	NR2F2	ENST00000394166	p.R109W	c.325A>T	Verified	cgg	agg	aac	1	A	T
TCGA-23-1110	NR6A1	ENST00000487099	p.I62F	c.184A>T	Verified	ctc	att	tgt	1	A	T
TCGA-24-2035	NRK	SU_ZC4-NRK	p.Q552L	c.1655A>T	Verified	gag	cag	aac	2	A	T

TCGA-13-0791	NRXN1	ENST00000342183	p.H146L	c.437A>T	Verified	CTG	CAT	ATA	2	A	T
TCGA-23-1120	NSMCE2	ENST00000287437	p.T188S	c.562A>T	Unverified	CAC	ACC	TAT	1	A	T
TCGA-61-2012	NUDCD1	ENST00000239690	p.T283S	c.847A>T	Verified	CAG	ACT	GAA	1	A	T
TCGA-24-1847	ODZ2_ENST	ENST00000518659	p.H981L	c.2942A>T	Unverified	CTA	CAC	TTT	2	A	T
TCGA-24-1847	ODZ2_ENST	ENST00000519204	p.H860L	c.2579A>T	Unverified	CTA	CAC	TTT	2	A	T
TCGA-04-1530	OLFM3	ENST00000370103	p.R64W	c.190A>T	Unverified	AGC	AGG	CAA	1	A	T
TCGA-23-1110	OR10J1	ENST00000423932	p.I120F	c.358A>T	Verified	GGC	ATC	ACT	1	A	T
TCGA-13-0919	OR11A1	ENST00000377149	p.T283S	c.847A>T	Verified	GTC	ACC	CCT	1	A	T
TCGA-23-1809	OR2A4	ENST00000315453	p.M257L	c.769A>T	Unverified	ATC	ATG	TAT	1	A	T
TCGA-24-1464	OR4C16	ENST00000314634	p.K86*	c.256A>T	Verified	TTG	AAG	AAG	1	A	T
TCGA-10-0930	OR4C46	ENST00000328188	p.E194V	c.581A>T	Verified	CTG	GAA	CTC	2	A	T
TCGA-10-0930	OR4C46	ENST00000328188	p.E194V	c.581A>T	Verified	CTG	GAA	CTC	2	A	T
TCGA-29-1761	OR4D9	ENST00000329328	p.T37T	c.111A>T	Unverified	ATG	ACA	ACT	3	A	T
TCGA-61-1740	OR52E8	ENST00000537935	p.R239R	c.717A>T	Unverified	GCT	CGA	CTC	3	A	T
TCGA-13-0791	OR5K2	ENST00000427338	p.T146T	c.438A>T	Verified	ACC	ACA	GGC	3	A	T
TCGA-61-1910	OR5W2	ENST00000344514	p.N294Y	c.880A>T	Unverified	AGG	AAC	AAG	1	A	T
TCGA-13-1499	PADI4	ENST00000375448	p.Q358L	c.1073A>T	Verified	ATC	CAA	GCC	2	A	T
TCGA-04-1342	PANK4	ENST00000378466	p.E395V	c.1184A>T	Unverified	CCC	GAG	CTC	2	A	T
TCGA-24-0979	PARP14	ENST00000310276	p.T912T	c.2736A>T	Verified	GGC	ACA	GTG	3	A	T
TCGA-13-0920	PASD1	ENST00000370357	p.S678S	c.2034A>T	Verified	GAC	TCA	ACC	3	A	T
TCGA-13-1507	PCDH1	ENST00000287008	p.D745V	c.2234A>T	Verified	TTT	GAC	TCT	2	A	T
TCGA-61-1738	PCDHA2	ENST00000378132	p.I104F	c.310A>T	Unverified	AGC	ATC	CAC	1	A	T
TCGA-61-1738	PCDHA2	ENST00000378132	p.I104F	c.310A>T	Unverified	AGC	ATC	CAC	1	A	T
TCGA-61-1738	PCDHA2_EN	ENST00000526136	p.I104F	c.310A>T	Unverified	AGC	ATC	CAC	1	A	T
TCGA-61-1738	PCDHA2_EN	ENST00000526136	p.I104F	c.310A>T	Unverified	AGC	ATC	CAC	1	A	T
TCGA-25-2401	PCDHB14	ENST00000239449	p.H129L	c.386A>T	Verified	GAT	CAC	TCC	2	A	T
TCGA-13-1488	PCDHGC4	ENST00000306593	p.D78V	c.233A>T	Verified	GTG	GAT	TTG	2	A	T
TCGA-24-1104	PDCD6IP	ENST00000307296	p.T586T	c.1758A>T	Verified	ttg	aca	gcc	3	A	T
TCGA-23-1120	PDE12	ENST00000311180	p.E134V	c.401A>T	Unverified	GCT	GAG	GAC	2	A	T
TCGA-25-2391	PDZD2	ENST00000282493	p.D776V	c.2327A>T	Unverified	GGG	GAT	CAA	2	A	T
TCGA-61-1910	PHIP	ENST00000275034	p.Q1263L	c.3788A>T	Unverified	GAT	CAG	ACT	2	A	T
TCGA-24-2289	PHLDB1	ENST00000361417	p.S273C	c.817A>T	Unverified	CCC	AGT	GGG	1	A	T
TCGA-24-1563	PHLDB2	ENST00000412622	p.A393A	c.1179A>T	Verified	GAG	GCA	GAT	3	A	T
TCGA-13-0792	PIGZ	ENST00000412723	p.E181V	c.542A>T	Verified	ATT	GAG	GGA	2	A	T

TCGA-13-0904	PIGZ	ENST00000412723	p.G5G	c.15A>T	Verified	TGT	GGA	TCC	3	A	T
TCGA-29-1770	PIK3CB	ENST00000477593	p.S4C	c.10A>T	Unverified	ttc	agt	ttc	1	A	T
TCGA-30-1891	PKHD1L1	ENST00000378402	p.T170S	c.508A>T	Unverified	TTC	ACT	GAT	1	A	T
TCGA-13-0762	PKP1	ENST00000367324	p.R340S	c.1020A>T	Verified	AGG	AGA	ACC	3	A	T
TCGA-13-0762	PKP1	ENST00000367324	p.R340S	c.1020A>T	Verified	AGG	AGA	ACC	3	A	T
TCGA-09-2049	PKP4	ENST00000389759	p.K790*	c.2368A>T	Verified	AAG	AAG	AAA	1	A	T
TCGA-04-1655	PLCH1	ENST00000334686	p.N866Y	c.2596A>T	Unverified	AAC	AAT	TCC	1	A	T
TCGA-04-1655	PLCH1_ENST	ENST00000340059	p.N904Y	c.2710A>T	Unverified	AAC	AAT	TCC	1	A	T
TCGA-13-1498	PLD1	ENST00000351298	p.H608L	c.1823A>T	Verified	TTT	CAC	CCG	2	A	T
TCGA-29-1693	PLD1	ENST00000351298	p.R339R	c.1017A>T	Unverified	CAT	CGA	TTT	3	A	T
TCGA-04-1651	PLEK	ENST00000234313	p.A83A	c.249A>T	Unverified	CAG	GCA	GCC	3	A	T
TCGA-04-1651	PLEK	ENST00000234313	p.A83A	c.249A>T	Unverified	CAG	GCA	GCC	3	A	T
TCGA-29-1781	PLXNA4_EN:	ENST00000321063	p.D1031V	c.3092A>T	Unverified	CAG	GAC	CTG	2	A	T
TCGA-24-1845	PLXNA4_EN:	ENST00000321063	p.A1831A	c.5493A>T	Unverified	AAC	GCA	TAC	3	A	T
TCGA-29-1781	PLXNA4_EN:	ENST00000321063	p.D1031V	c.3092A>T	Unverified	CAG	GAC	CTG	2	A	T
TCGA-24-1845	PLXNA4_EN:	ENST00000321063	p.A1831A	c.5493A>T	Unverified	AAC	GCA	TAC	3	A	T
TCGA-04-1649	PNLDC1	ENST00000275275	p.S417C	c.1249A>T	Unverified	CCC	AGT	ATC	1	A	T
TCGA-13-1488	PNMT	ENST00000269582	p.P189P	c.567A>T	Verified	AGC	CCA	GAT	3	A	T
TCGA-23-1123	PNP	ENST00000361505	p.E201V	c.602A>T	Verified	ttt	gag	act	2	A	T
TCGA-23-1123	PNP	ENST00000361505	p.E201V	c.602A>T	Verified	ttt	gag	act	2	A	T
TCGA-10-0930	PPFIA2	ENST00000549396	p.G607G	c.1821A>T	Verified	att	gga	gta	3	A	T
TCGA-10-0930	PPFIA2	ENST00000549396	p.G607G	c.1821A>T	Verified	att	gga	gta	3	A	T
TCGA-23-1032	PPP1R3A	NM_002711.2	p.D580V	c.1739A>T	Verified	gca	gat	gtg	2	A	T
TCGA-13-1510	PPP1R3A	NM_002711.2	p.G413G	c.1239A>T	Verified	atg	gga	gaa	3	A	T
TCGA-13-1510	PRAMEF1	ENST00000332296	p.G253G	c.759A>T	Unverified	CAA	GGA	CGG	3	A	T
TCGA-29-1763	PREX1	ENST00000371941	p.Q796L	c.2387A>T	Unverified	GCC	CAG	GAA	2	A	T
TCGA-29-1763	PREX1_ENST	ENST00000396220	p.Q796L	c.2387A>T	Unverified	GCC	CAG	GAA	2	A	T
TCGA-29-1784	PRKAG2	ENST00000287878	p.Q337L	c.1010A>T	Unverified	GTA	CAG	ATT	2	A	T
TCGA-29-1784	PRKAG2_EN	ENST00000433631	p.Q212L	c.635A>T	Unverified	GTA	CAG	ATT	2	A	T
TCGA-29-1691	PRPF3	ENST00000324862	p.R518R	c.1554A>T	Unverified	GCC	CGA	AAA	3	A	T
TCGA-29-1698	PSEN2	ENST00000366783	p.I255F	c.763A>T	Unverified	GTC	ATC	CTG	1	A	T
TCGA-13-1498	PTCH2	ENST00000372192	p.K158*	c.472A>T	Verified	aac	aaa	atc	1	A	T
TCGA-61-1738	PTGR2	ENST00000267568	p.T157T	c.471A>T	Unverified	AAG	ACA	ATG	3	A	T
TCGA-61-1738	PTGR2	ENST00000267568	p.T157T	c.471A>T	Unverified	AAG	ACA	ATG	3	A	T

TCGA-04-1338	PTPN3	ENST00000394831	p.H812L	c.2435A>T	Verified	GAC	CAC	GGT	2	A	T
TCGA-24-1843	PTPRZ1	ENST00000393386	p.I470L	c.1408A>T	Unverified	CGC	ATA	GGG	1	A	T
TCGA-24-1843	PTPRZ1_ENS	ENST00000393386	p.I470L	c.1408A>T	Unverified	CGC	ATA	GGG	1	A	T
TCGA-61-2095	PTRF	ENST00000357037	p.E77V	c.230A>T	Unverified	GAG	GAG	CGG	2	A	T
TCGA-23-1022	PYHIN1	ENST00000368140	p.Q397L	c.1190A>T	Verified	ATC	CAG	ATA	2	A	T
TCGA-61-1740	RAB37	ENST00000402449	p.T55S	c.163A>T	Unverified	GCC	ACT	GTG	1	A	T
TCGA-61-1740	RAB37_ENST	ENST00000402449	p.T55S	c.163A>T	Unverified	GCC	ACT	GTG	1	A	T
TCGA-61-1910	RAMP3	ENST00000242249	p.T31T	c.93A>T	Unverified	GAG	ACA	GGC	3	A	T
TCGA-13-1408	RASA3	ENST00000334062	p.R88W	c.262A>T	Unverified	cgg	agg	gat	1	A	T
TCGA-13-0893	RCSD1	ENST00000367854	p.I86F	c.256A>T	Verified	CTG	ATT	GAG	1	A	T
TCGA-25-2392	RFX6	ENST00000332958	p.Q15L	c.44A>T	Verified	GCG	CAG	CCT	2	A	T
TCGA-04-1362	RIMS2	ENST00000507740	p.H825L	c.2474A>T	Verified	CAT	CAC	AGG	2	A	T
TCGA-04-1362	RIMS2_ENST	ENST00000436393	p.H811L	c.2432A>T	Verified	CAT	CAC	AGG	2	A	T
TCGA-20-0991	RNF111	ENST00000348370	p.R904S	c.2712A>T	Verified	GAA	AGA	TGT	3	A	T
TCGA-23-1031	RPH3A	ENST00000389385	p.E72V	c.215A>T	Verified	ATG	GAG	CAG	2	A	T
TCGA-13-1498	RSPO2	ENST00000276659	p.P79P	c.237A>T	Verified	TGC	CCA	TCC	3	A	T
TCGA-13-1505	RSRC2	ENST00000331738	p.K23*	c.67A>T	Verified	AAG	AAA	AAA	1	A	T
TCGA-23-1117	RYR1	ENST00000359596	p.E5V	c.14A>T	Unverified	GCA	GAA	GGC	2	A	T
TCGA-23-1117	RYR1	ENST00000359596	p.E5V	c.14A>T	Unverified	GCA	GAA	GGC	2	A	T
TCGA-24-0975	RYR1	ENST00000359596	p.E4691V	c.14072A>T	Verified	ACG	GAG	CAG	2	A	T
TCGA-30-1714	RYR1	ENST00000359596	p.R1993S	c.5979A>T	Unverified	GCA	AGA	CGT	3	A	T
TCGA-61-1910	RYR2	ENST00000366574	p.N1945Y	c.5833A>T	Unverified	TAC	AAC	GAA	1	A	T
TCGA-13-0903	RYR2	ENST00000366574	p.D3896V	c.11687A>T	Verified	AAA	GAT	GTT	2	A	T
TCGA-24-1463	RYR2	ENST00000366574	p.E4906V	c.14717A>T	Verified	TTT	GAA	ACC	2	A	T
TCGA-13-1498	RYR2	ENST00000366574	p.G4711G	c.14133A>T	Verified	CTA	GGA	GTC	3	A	T
TCGA-29-1761	RYR3	ENST00000389232	p.R279S	c.837A>T	Unverified	ATC	AGA	TGG	3	A	T
TCGA-61-2113	SBF2	ENST00000256190	p.D705V	c.2114A>T	Verified	CCT	GAT	GAC	2	A	T
TCGA-23-1117	SCAI	ENST00000373549	p.Q44L	c.131A>T	Verified	TCA	CAA	TCT	2	A	T
TCGA-23-1117	SCAI	ENST00000373549	p.Q44L	c.131A>T	Verified	TCA	CAA	TCT	2	A	T
TCGA-13-0795	SCN1A	ENST00000375405	p.I1639F	c.4915A>T	Verified	CTG	ATC	AAA	1	A	T
TCGA-13-1509	SCN1A	ENST00000375405	p.R1514R	c.4542A>T	Verified	CCT	CGA	CCA	3	A	T
TCGA-04-1652	SCN3A	ENST00000283254	p.T399T	c.1197A>T	Unverified	AAA	ACA	TAC	3	A	T
TCGA-04-1652	SCN3A	ENST00000283254	p.T399T	c.1197A>T	Unverified	AAA	ACA	TAC	3	A	T
TCGA-04-1652	SCN3A_ENST	ENST00000409101	p.T399T	c.1197A>T	Unverified	AAA	ACA	TAC	3	A	T

TCGA-04-1652	SCN3A_ENST	ENST00000409101	p.T399T	c.1197A>T	Unverified	AAA	ACA	TAC	3	A	T
TCGA-29-1696	SCN7A	ENST00000409855	p.E291V	c.872A>T	Unverified	CGA	GAA	ACA	2	A	T
TCGA-29-1696	SCN7A	ENST00000409855	p.E291V	c.872A>T	Unverified	CGA	GAA	ACA	2	A	T
TCGA-29-1696	SCN7A_ENST	ENST00000409855	p.E291V	c.872A>T	Unverified	CGA	GAA	ACA	2	A	T
TCGA-29-1696	SCN7A_ENST	ENST00000409855	p.E291V	c.872A>T	Unverified	CGA	GAA	ACA	2	A	T
TCGA-04-1367	SCRN1	ENST00000242059	p.S241C	c.721A>T	Verified	GAC	AGC	TTA	1	A	T
TCGA-04-1362	SCRN2	ENST00000290216	p.D296V	c.887A>T	Verified	CAG	GAT	CCC	2	A	T
TCGA-24-1845	SELV	ENST00000335426	p.H318L	c.953A>T	Unverified	GTC	CAT	TCC	2	A	T
TCGA-61-1899	SEMA3C	ENST00000265361	p.Q638L	c.1913A>T	Unverified	GAC	CAA	GGA	2	A	T
TCGA-29-1764	SERPINB4	ENST00000341074	p.S380C	c.1138A>T	Unverified	aac	agc	atc	1	A	T
TCGA-29-1769	SETBP1	ENST00000282030	p.T1423S	c.4267A>T	Unverified	TCC	ACC	AAG	1	A	T
TCGA-13-0920	SF3A1	ENST00000215793	p.K102*	c.304A>T	Verified	TTC	AAG	GAA	1	A	T
TCGA-23-1022	SH3TC2	ENST00000515425	p.E1045V	c.3134A>T	Verified	GCT	GAG	GCC	2	A	T
TCGA-13-1496	SI	ENST00000264382	p.I1034F	c.3100A>T	Verified	AAG	ATT	TAT	1	A	T
TCGA-29-1770	SIGLEC8	ENST00000321424	p.T327S	c.979A>T	Unverified	TTC	ACC	TGC	1	A	T
TCGA-30-1855	SIM2	ENST00000290399	p.M198L	c.592A>T	Unverified	GAC	ATG	TCC	1	A	T
TCGA-13-0791	SLC12A1	ENST00000380993	p.D285V	c.854A>T	Verified	GTA	GAT	CTT	2	A	T
TCGA-09-0369	SLC1A7	ENST00000371494	p.I472F	c.1414A>T	Verified	GGG	ATC	ATG	1	A	T
TCGA-09-0369	SLC1A7	ENST00000371494	p.I472F	c.1414A>T	Verified	GGG	ATC	ATG	1	A	T
TCGA-25-2401	SLC20A1	ENST00000272542	p.E259V	c.776A>T	Verified	ATT	GAA	CGA	2	A	T
TCGA-61-2095	SLC22A18	ENST00000380574	p.R419W	c.1255A>T	Verified	CAG	AGG	AAG	1	A	T
TCGA-04-1530	SLC25A44	ENST00000359511	p.M243L	c.727A>T	Unverified	CCC	ATG	GAT	1	A	T
TCGA-04-1530	SLC26A7	ENST00000276609	p.G367G	c.1101A>T	Unverified	ATG	GGA	AGG	3	A	T
TCGA-25-1318	SLC37A3	ENST00000326232	p.E134V	c.401A>T	Verified	ACA	GAA	TGG	2	A	T
TCGA-25-1318	SLC37A3	ENST00000326232	p.E134V	c.401A>T	Verified	ACA	GAA	TGG	2	A	T
TCGA-09-1674	SLC38A11	ENST00000303735	p.S123S	c.369A>T	Unverified	ATT	TCA	CTG	3	A	T
TCGA-61-2012	SLC45A2	ENST00000296589	p.M342L	c.1024A>T	Verified	TTC	ATG	GGC	1	A	T
TCGA-30-1855	SLC4A10	ENST00000415876	p.Q417L	c.1250A>T	Unverified	TCC	CAG	GAG	2	A	T
TCGA-30-1855	SLC4A10_EN	ENST00000446997	p.Q447L	c.1340A>T	Unverified	TCC	CAG	GAG	2	A	T
TCGA-13-1509	SLC4A4	ENST00000340595	p.H386L	c.1157A>T	Verified	GGA	CAT	GGG	2	A	T
TCGA-10-0930	SLC9A10	ENST00000305815	p.M234L	c.700A>T	Verified	TGG	ATG	TCA	1	A	T
TCGA-10-0930	SLC9A10	ENST00000305815	p.M234L	c.700A>T	Verified	TGG	ATG	TCA	1	A	T
TCGA-13-1507	SLCO1B1	ENST00000256958	p.H451L	c.1352A>T	Verified	TCT	CAT	AGA	2	A	T
TCGA-24-1847	SLCO1C1	ENST00000266509	p.R627S	c.1881A>T	Unverified	AGT	AGA	GGA	3	A	T

TCGA-24-1847	SLCO1C1_EN	ENST00000381552	p.R627S	c.1881A>T	Unverified	AGT	AGA	GGA	3	A	T
TCGA-61-2094	SLCO2B1	ENST00000289575	p.I664F	c.1990A>T	Unverified	GTG	ATC	TGC	1	A	T
TCGA-24-2280	SLIT1	ENST00000266058	p.I495F	c.1483A>T	Unverified	TTC	ATT	CCA	1	A	T
TCGA-25-2400	SMARCC2	ENST00000267064	p.Q478L	c.1433A>T	Verified	CCC	CAA	GAG	2	A	T
TCGA-13-0885	SMARCD3	ENST00000392811	p.R282W	c.844A>T	Verified	aac	agg	ctg	1	A	T
TCGA-24-2290	SMC5	ENST00000361138	p.R309R	c.927A>T	Verified	TGT	CGA	ATT	3	A	T
TCGA-24-2290	SMC5	ENST00000361138	p.R309R	c.927A>T	Verified	TGT	CGA	ATT	3	A	T
TCGA-13-0913	SMS	ENST00000404933	p.I181F	c.541A>T	Unverified	gcc	atc	atg	1	A	T
TCGA-24-1845	SORBS2	ENST00000284776	p.R686*	c.2056A>T	Unverified	ccc	aga	agg	1	A	T
TCGA-13-0884	SOX5	ENST00000451604	p.E214V	c.641A>T	Verified	cga	gag	cag	2	A	T
TCGA-20-0991	SP1	ENST00000327443	p.A580A	c.1740A>T	Verified	ACA	GCA	GGT	3	A	T
TCGA-29-1696	SPAG6	ENST00000376624	p.S231S	c.693A>T	Unverified	CTT	TCA	GCT	3	A	T
TCGA-29-1696	SPAG6	ENST00000376624	p.S231S	c.693A>T	Unverified	CTT	TCA	GCT	3	A	T
TCGA-13-1510	SPARC	ENST00000231061	p.T177S	c.529A>T	Unverified	GTC	ACC	CTG	1	A	T
TCGA-25-2042	SPCS3	ENST00000503362	p.D79V	c.236A>T	Unverified	TTT	GAT	TGG	2	A	T
TCGA-24-1604	SPR	ENST00000234454	p.K226*	c.676A>T	Verified	CTG	AAG	GCA	1	A	T
TCGA-24-2267	STAG2	ENST00000371160	p.E721V	c.2162A>T	Verified	atc	gaa	aat	2	A	T
TCGA-23-1114	STARD8	ENST00000252336	p.E693V	c.2078A>T	Unverified	CGA	GAG	GTG	2	A	T
TCGA-23-1114	STARD8_EN!	ENST00000252336	p.E693V	c.2078A>T	Unverified	CGA	GAG	GTG	2	A	T
TCGA-23-1114	STARD8_EN!	ENST00000374599	p.E773V	c.2318A>T	Unverified	CGA	GAG	GTG	2	A	T
TCGA-29-1761	STIP1	ENST00000305218	p.E419V	c.1256A>T	Unverified	GAG	GAA	TGT	2	A	T
TCGA-23-2078	SYT14	ENST00000472886	p.D137V	c.410A>T	Verified	TAT	GAT	GGA	2	A	T
TCGA-13-0762	SYT6	ENST00000369546	p.I348F	c.1042A>T	Unverified	ATC	ATC	TTT	1	A	T
TCGA-13-0762	SYT6	ENST00000369546	p.I348F	c.1042A>T	Unverified	ATC	ATC	TTT	1	A	T
TCGA-04-1331	SYTL4	ENST00000372989	p.H476L	c.1427A>T	Verified	GAT	CAT	TGC	2	A	T
TCGA-13-1512	SZT2	ENST00000372442	p.E2284V	c.6851A>T	Verified	GCT	GAG	CGG	2	A	T
TCGA-13-0913	TANC1	ENST00000263635	p.K1418*	c.4252A>T	Verified	TGC	AAA	CAA	1	A	T
TCGA-29-1702	TARBP1	ENST00000040877	p.S912C	c.2734A>T	Unverified	GGG	AGT	GAA	1	A	T
TCGA-23-1120	TBC1D21	ENST00000300504	p.Q158L	c.473A>T	Verified	ACG	CAG	GCA	2	A	T
TCGA-13-0903	TBK1	ENST00000331710	p.R525S	c.1575A>T	Verified	AGC	AGA	TTA	3	A	T
TCGA-23-1116	TCEAL3	ENST00000372628	p.E39V	c.116A>T	Unverified	GTG	GAG	GGG	2	A	T
TCGA-24-1843	TCP11L2	ENST00000299045	p.Q14L	c.41A>T	Unverified	GAC	CAG	CCA	2	A	T
TCGA-13-1408	TECPR2	ENST00000359520	p.E628V	c.1883A>T	Unverified	GGG	GAA	GAC	2	A	T
TCGA-23-1031	TEKT3	ENST00000395930	p.E163V	c.488A>T	Verified	CAT	GAG	TTG	2	A	T

TCGA-61-1740	TEKT4	ENST00000295201	p.K123*	c.367A>T	Unverified	CAG	AAG	CAA	1	A	T
TCGA-13-0913	TEX101	ENST00000253435	p.A87A	c.261A>T	Verified	AAA	GCA	GGG	3	A	T
TCGA-09-2044	THAP9	ENST00000302236	p.E776V	c.2327A>T	Verified	TGT	GAG	CGA	2	A	T
TCGA-24-1422	TIE1	ENST00000372476	p.S190C	c.568A>T	Unverified	TCG	AGC	GGC	1	A	T
TCGA-13-1497	TJP1	ENST00000346128	p.E957V	c.2870A>T	Verified	CTG	GAG	GAG	2	A	T
TCGA-23-1021	TMEM39A	ENST00000319172	p.N31Y	c.91A>T	Verified	GGC	AAT	GGA	1	A	T
TCGA-09-2049	TMEM45A	ENST00000323523	p.T222S	c.664A>T	Verified	CTC	ACC	ATA	1	A	T
TCGA-23-1111	TMPO_ENST	ENST00000266732	p.M457L	c.1369A>T	Unverified	CTG	ATG	TCT	1	A	T
TCGA-61-1907	TNNI3K	ENST00000326637	p.H518L	c.1553A>T	Unverified	aat	cat	ccc	2	A	T
TCGA-61-1913	TNPO1	ENST00000506351	p.D543V	c.1628A>T	Unverified	tac	gat	gcc	2	A	T
TCGA-24-1844	TNR	ENST00000367674	p.P976P	c.2928A>T	Unverified	GCA	CCA	GTG	3	A	T
TCGA-04-1331	TNS3	ENST00000311160	p.S1048S	c.3144A>T	Verified	GCG	TCA	CCG	3	A	T
TCGA-61-2095	TOM1	ENST00000449058	p.K480*	c.1438A>T	Unverified	AAG	AAG	ACC	1	A	T
TCGA-09-2050	TP63	ENST00000264731	p.M107L	c.319A>T	Verified	ccc	atg	tgg	1	A	T
TCGA-13-0906	TPO	ENST00000345913	p.H805L	c.2414A>T	Verified	gcc	cac	ccc	2	A	T
TCGA-29-1775	TRHR	ENST00000518632	p.S349C	c.1045A>T	Unverified	tac	agt	gtg	1	A	T
TCGA-29-1761	TRIML2	ENST00000512729	p.D318V	c.953A>T	Unverified	TTG	GAC	ACA	2	A	T
TCGA-04-1331	TRPC7	ENST00000513104	p.D210V	c.629A>T	Unverified	AAA	GAC	TCC	2	A	T
TCGA-13-1488	TRPC7	ENST00000513104	p.D210V	c.629A>T	Unverified	AAA	GAC	TCC	2	A	T
TCGA-29-1781	TRPV3	ENST00000576742	p.I277F	c.829A>T	Unverified	GAG	ATT	GTG	1	A	T
TCGA-24-1422	TRPV4	ENST00000261740	p.N204Y	c.610A>T	Verified	AGC	AAT	GGC	1	A	T
TCGA-20-1685	TTF2	ENST00000369466	p.T694S	c.2080A>T	Unverified	ATC	ACT	ACC	1	A	T
TCGA-61-1733	TTN	NM_003319	p.I370F	c.1108A>T	Unverified	cag	atc	agg	1	A	T
TCGA-61-1733	TTN	NM_003319	p.I370F	c.1108A>T	Unverified	cag	atc	agg	1	A	T
TCGA-61-1733	TTN_ENST0(	ENST00000342175	p.I370F	c.1108A>T	Unverified	CAG	ATC	AGG	1	A	T
TCGA-61-1733	TTN_ENST0(	ENST00000342175	p.I370F	c.1108A>T	Unverified	CAG	ATC	AGG	1	A	T
TCGA-61-1733	TTN_ENST0(	ENST00000342992	p.I370F	c.1108A>T	Unverified	CAG	ATC	AGG	1	A	T
TCGA-61-1733	TTN_ENST0(	ENST00000342992	p.I370F	c.1108A>T	Unverified	CAG	ATC	AGG	1	A	T
TCGA-61-1733	TTN_ENST0(	ENST00000356127	p.I370F	c.1108A>T	Unverified	CAG	ATC	AGG	1	A	T
TCGA-61-1733	TTN_ENST0(	ENST00000356127	p.I370F	c.1108A>T	Unverified	CAG	ATC	AGG	1	A	T
TCGA-61-1733	TTN_ENST0(	ENST00000359218	p.I370F	c.1108A>T	Unverified	CAG	ATC	AGG	1	A	T
TCGA-61-1733	TTN_ENST0(	ENST00000359218	p.I370F	c.1108A>T	Unverified	CAG	ATC	AGG	1	A	T
TCGA-61-1733	TTN_ENST0(	ENST00000360870	p.I370F	c.1108A>T	Unverified	CAG	ATC	AGG	1	A	T
TCGA-61-1733	TTN_ENST0(	ENST00000360870	p.I370F	c.1108A>T	Unverified	CAG	ATC	AGG	1	A	T

TCGA-13-0886	TUBB1	ENST00000217133	p.S168C	c.502A>T	Verified	TTC	AGC	GTC	1	A	T
TCGA-36-1577	TXNDC12	ENST00000371626	p.Q155L	c.464A>T	Verified	GCT	CAG	GAA	2	A	T
TCGA-24-2262	UBE3B	ENST00000434735	p.I833F	c.2497A>T	Verified	tcc	atc	aag	1	A	T
TCGA-13-0905	UBR5	ENST00000520539	p.D2493V	c.7478A>T	Verified	tta	gat	gat	2	A	T
TCGA-29-1777	UGT2A3	ENST00000251566	p.T197S	c.589A>T	Unverified	ATG	ACA	GGA	1	A	T
TCGA-30-1857	UNC13C	ENST00000260323	p.I2068F	c.6202A>T	Unverified	GTG	ATT	GCT	1	A	T
TCGA-30-1857	UNC13C_EN	ENST00000260323	p.I2068F	c.6202A>T	Unverified	GTG	ATT	GCT	1	A	T
TCGA-13-0923	USH2A	ENST00000307340	p.I399F	c.1195A>T	Verified	AGG	ATT	CAA	1	A	T
TCGA-13-0905	UTP20	ENST00000261637	p.H240L	c.719A>T	Verified	TTT	CAC	TCC	2	A	T
TCGA-13-0792	VIPR2	ENST00000262178	p.H28L	c.83A>T	Unverified	TTT	CAT	CTG	2	A	T
TCGA-61-1740	VPS13A	ENST00000360280	p.E3131V	c.9392A>T	Unverified	ATT	GAA	GCA	2	A	T
TCGA-13-1512	VPS13C	ENST00000261517	p.R3464S	c.10392A>T	Verified	GTG	AGA	AGC	3	A	T
TCGA-04-1338	WDR88	ENST00000355868	p.S119C	c.355A>T	Verified	CTC	AGT	GGC	1	A	T
TCGA-13-1510	WDR88	ENST00000355868	p.R252W	c.754A>T	Unverified	GAC	AGG	TGC	1	A	T
TCGA-61-1906	XDH	ENST00000379416	p.A812A	c.2436A>T	Unverified	ACG	GCA	GTG	3	A	T
TCGA-61-1906	XDH	ENST00000379416	p.A812A	c.2436A>T	Unverified	ACG	GCA	GTG	3	A	T
TCGA-24-1103	XIRP1	ENST00000340369	p.D1289V	c.3866A>T	Verified	AAG	GAC	CCC	2	A	T
TCGA-24-2024	XIRP2	ENST00000295237	p.Q1158L	c.3473A>T	Verified	ATC	CAA	GGT	2	A	T
TCGA-29-1781	XPO6	ENST00000304658	p.P268P	c.804A>T	Unverified	ACC	CCA	TCC	3	A	T
TCGA-29-1781	XPO6_ENST(	ENST00000304658	p.P268P	c.804A>T	Unverified	ACC	CCA	TCC	3	A	T
TCGA-25-2393	ZC3H14	ENST00000251038	p.N57Y	c.169A>T	Verified	GGG	AAC	AAC	1	A	T
TCGA-25-2401	ZC3H7B	ENST00000352645	p.R166R	c.498A>T	Unverified	CTG	CGA	GTT	3	A	T
TCGA-25-1313	ZFP1	ENST00000393430	p.T284S	c.850A>T	Verified	CTC	ACC	ACA	1	A	T
TCGA-04-1347	ZFP90	ENST00000398253	p.E271V	c.812A>T	Verified	ACT	GAG	CAT	2	A	T
TCGA-23-1120	ZNF148	ENST00000360647	p.D465V	c.1394A>T	Verified	TAT	GAT	GAT	2	A	T
TCGA-29-1691	ZNF233	ENST00000391958	p.M624L	c.1870A>T	Unverified	GGC	ATG	TGT	1	A	T
TCGA-24-1845	ZNF236	ENST00000253159	p.Q359L	c.1076A>T	Unverified	ATC	CAG	CAG	2	A	T
TCGA-24-1845	ZNF236_ENS	ENST00000543926	p.Q359L	c.1076A>T	Unverified	ATC	CAG	CAG	2	A	T
TCGA-25-2392	ZNF238	ENST00000358704	p.S399C	c.1195A>T	Verified	CTG	AGC	ACG	1	A	T
TCGA-24-1846	ZNF295	ENST00000398499	p.T895T	c.2685A>T	Unverified	AGC	ACA	GCC	3	A	T
TCGA-13-1510	ZNF345	ENST00000420450	p.E255V	c.764A>T	Unverified	GGT	GAG	AAA	2	A	T
TCGA-13-0886	ZNF513	ENST00000323703	p.S383C	c.1147A>T	Verified	CAC	AGT	GGT	1	A	T
TCGA-13-0884	ZNF608	ENST00000306315	p.D590V	c.1769A>T	Verified	CCT	GAC	AGT	2	A	T
TCGA-25-2398	ZNF611	ENST00000319783	p.S607S	c.1821A>T	Verified	AGG	TCA	TCC	3	A	T

TCGA-23-1122	ZNF620	ENST00000314529	p.T55S	c.163A>T	Verified	TTC	ACC	ACG	1	A	T
TCGA-61-1998	ZNF644	ENST00000370440	p.T664T	c.1992A>T	Verified	CGA	ACA	TTT	3	A	T
TCGA-29-1702	ZNF850	ENST00000591344	p.H1027L	c.3080A>T	Unverified	CAA	CAT	AAA	2	A	T
TCGA-13-0913	ZSCAN1	ENST00000282326	p.A169A	c.507A>T	Verified	CCA	GCA	CTC	3	A	T
TCGA-30-1857	ZSWIM3	ENST00000255152	p.Q657L	c.1970A>T	Unverified	TCC	CAG	CCA	2	A	T
TCGA-25-2401	ABCA1	ENST00000374736	p.M433L	c.1297A>T	Verified	GAA	ATG	GAC	1	A	T
TCGA-23-2078	ACCN2	ENST00000228468	p.S251C	c.751A>T	Verified	CAT	AGT	CAG	1	A	T
TCGA-13-1501	ACCSL	ENST00000378832	p.T393S	c.1177A>T	Verified	GGT	ACC	AGT	1	A	T
TCGA-13-1501	ACCSL	ENST00000378832	p.T393S	c.1177A>T	Verified	GGT	ACC	AGT	1	A	T
TCGA-29-1761	ACOX3	ENST00000356406	p.N258I	c.773A>T	Unverified	GAT	AAT	GGT	2	A	T
TCGA-30-1891	ADAM10	ENST00000260408	p.K299M	c.896A>T	Unverified	gag	aag	ttt	2	A	T
TCGA-13-0792	ADAMTS16_	ENST00000274181	p.K1209M	c.3626A>T	Verified	CAC	AAG	TTC	2	A	T
TCGA-29-1768	AGAP2	ENST00000257897	p.K501*	c.1501A>T	Unverified	aaa	aaa	ttg	1	A	T
TCGA-29-1768	AGAP2_ENS	ENST00000547588	p.K837*	c.2509A>T	Unverified	AAA	AAA	TTG	1	A	T
TCGA-13-0760	ALB	ENST00000295897	p.Y174F	c.521A>T	Verified	TTT	TAT	GCC	2	A	T
TCGA-24-2289	ALS2CR12	ENST00000286190	p.N125I	c.374A>T	Unverified	ACC	AAC	ATC	2	A	T
TCGA-24-1849	ANKRD12	ENST00000262126	p.K945N	c.2835A>T	Unverified	AGT	AAA	TCA	3	A	T
TCGA-24-2280	ANKRD35	ENST00000355594	p.K913I	c.2738A>T	Verified	CTG	AAA	GAG	2	A	T
TCGA-13-0714	ANXA11	ENST00000372234	p.N41I	c.122A>T	Verified	GAT	AAC	GTG	2	A	T
TCGA-29-1775	AP1S2	ENST00000329235	p.K17I	c.50A>T	Unverified	caa	aaa	tgg	2	A	T
TCGA-09-2049	AQP2	ENST00000199280	p.N184I	c.551A>T	Verified	ATG	AAT	CCT	2	A	T
TCGA-04-1655	ARAP2	ENST00000303965	p.K1224N	c.3672A>T	Unverified	AAA	AAA	TAT	3	A	T
TCGA-09-1665	ARFGEF1	ENST00000262215	p.E294D	c.882A>T	Verified	aat	gaa	cag	3	A	T
TCGA-09-2049	ASB17	ENST00000284142	p.K6N	c.18A>T	Verified	act	aaa	tta	3	A	T
TCGA-13-0792	ATP11C	ENST00000327569	p.Y879F	c.2636A>T	Verified	CAG	TAC	TTC	2	A	T
TCGA-61-1906	ATP13A5	ENST00000342358	p.M332L	c.994A>T	Unverified	ACT	ATG	CCT	1	A	T
TCGA-61-1906	ATP13A5	ENST00000342358	p.M332L	c.994A>T	Unverified	ACT	ATG	CCT	1	A	T
TCGA-13-0760	ATR	NM_001184	p.N2056I	c.6167A>T	Verified	gac	aac	aaa	2	A	T
TCGA-09-2049	ATRNL1	ENST00000355044	p.S724C	c.2170A>T	Verified	AAA	AGC	TGT	1	A	T
TCGA-23-2077	BAZ1B	ENST00000339594	p.K216I	c.647A>T	Verified	CCA	AAA	TTT	2	A	T
TCGA-61-2012	BCL6	ENST00000406870	p.Y293F	c.878A>T	Verified	ccc	tac	ttc	2	A	T
TCGA-61-2113	BOD1L	ENST00000040738	p.Q1210H	c.3630A>T	Verified	ATA	CAA	AGT	3	A	T
TCGA-30-1857	BRCA1	ENST00000357654	p.K800*	c.2398A>T	Unverified	aat	aaa	tgt	1	A	T
TCGA-13-0792	BRCA2	ENST00000380152	p.E1143D	c.3429A>T	Verified	TTT	GAA	GTG	3	A	T

TCGA-13-0893	C10orf113	ENST00000377118	p.Q72H	c.216A>T	Verified	CTG	CAA	AGC	3	A	T
TCGA-13-0893	C10orf113_I	ENST00000377118	p.Q72H	c.216A>T	Verified	CTG	CAA	AGC	3	A	T
TCGA-61-1906	C10orf120	ENST00000329446	p.R238*	c.712A>T	Unverified	AAA	AGA	CGA	1	A	T
TCGA-61-1906	C10orf120	ENST00000329446	p.R238*	c.712A>T	Unverified	AAA	AGA	CGA	1	A	T
TCGA-13-0792	C11orf30	ENST00000334736	p.K222M	c.665A>T	Verified	CCA	AAG	GCC	2	A	T
TCGA-09-2044	C14orf118	ENST00000261530	p.K354N	c.1062A>T	Verified	AAT	AAA	GCG	3	A	T
TCGA-24-2280	C14orf28	ENST00000325192	p.R24W	c.70A>T	Unverified	TGT	AGG	CCT	1	A	T
TCGA-24-1845	C1orf146	ENST00000370375	p.K6I	c.17A>T	Unverified	GGA	AAA	GAA	2	A	T
TCGA-13-0916	C5orf42	ENST00000274258	p.V88V	c.264A>T	Verified	CCT	GTA	GCA	3	A	T
TCGA-24-2024	C7orf58	ENST00000310396	p.I419I	c.1257A>T	Verified	TCC	ATA	TTT	3	A	T
TCGA-10-0930	C8orf80	ENST00000341513	p.K652M	c.1955A>T	Verified	AGG	AAG	AGG	2	A	T
TCGA-10-0930	C8orf80	ENST00000341513	p.K652M	c.1955A>T	Verified	AGG	AAG	AGG	2	A	T
TCGA-13-1498	CACNA2D3	ENST00000474759	p.R897*	c.2689A>T	Verified	AAA	AGA	ATT	1	A	T
TCGA-29-1776	CC2D2A	ENST00000389652	p.L302L	c.906A>T	Unverified	ATC	CTA	GCT	3	A	T
TCGA-29-1776	CC2D2A_EN	ENST00000424120	p.L351L	c.1053A>T	Unverified	ATC	CTA	GCT	3	A	T
TCGA-13-0923	CCDC136	ENST00000397697	p.E851D	c.2553A>T	Verified	GAG	GAA	ATG	3	A	T
TCGA-29-1777	CCDC164	ENST00000288710	p.M463L	c.1387A>T	Unverified	CTT	ATG	CGC	1	A	T
TCGA-13-0906	CCDC39	ENST00000273654	p.K515N	c.1545A>T	Verified	CTG	AAA	CAT	3	A	T
TCGA-13-1512	CCDC69	ENST00000355417	p.E20D	c.60A>T	Verified	CAA	GAA	CCA	3	A	T
TCGA-23-1124	CD163	ENST00000359156	p.Q241H	c.723A>T	Verified	CAT	CAA	GGA	3	A	T
TCGA-61-2113	CD38	ENST00000226279	p.N183I	c.548A>T	Verified	AAC	AAC	CCT	2	A	T
TCGA-13-0904	CD97	ENST00000242786	p.K281I	c.842A>T	Verified	GAC	AAA	GTC	2	A	T
TCGA-13-0885	CDCP2	ENST00000371330	p.N267I	c.800A>T	Verified	GGC	AAC	TTC	2	A	T
TCGA-13-0885	CDH10	ENST00000264463	p.K53N	c.159A>T	Verified	CAA	AAA	CGT	3	A	T
TCGA-04-1338	CDH11	ENST00000268603	p.E646D	c.1938A>T	Verified	AAA	GAA	CCA	3	A	T
TCGA-23-1110	CDH16	ENST00000299752	p.E281D	c.843A>T	Verified	TTT	GAA	GTG	3	A	T
TCGA-10-0930	CDH19	ENST00000262150	p.N508I	c.1523A>T	Verified	TTT	AAT	CTA	2	A	T
TCGA-10-0930	CDH19	ENST00000262150	p.N508I	c.1523A>T	Verified	TTT	AAT	CTA	2	A	T
TCGA-61-1740	CENPF	ENST00000366955	p.T729S	c.2185A>T	Unverified	CTT	ACT	GGG	1	A	T
TCGA-25-1326	CENPJ	ENST00000381884	p.N123Y	c.367A>T	Verified	AAA	AAT	GAC	1	A	T
TCGA-24-1469	CEP63	ENST00000337090	p.V167V	c.501A>T	Verified	CAG	GTA	TCT	3	A	T
TCGA-61-1910	CFH	ENST00000367429	p.K156I	c.467A>T	Unverified	gga	aaa	att	2	A	T
TCGA-29-1693	CFHR4_ENST	ENST00000367416	p.Y176F	c.527A>T	Unverified	TGC	TAT	GAT	2	A	T
TCGA-04-1655	CHD9	NM_025134.3	p.K1350*	c.4048A>T	Unverified	aaa	aag	gaa	1	A	T

TCGA-13-0903	CHL1	ENST00000256509	p.K1132M	c.3395A>T	Verified	GAA	AAG	GAA	2	A	T
TCGA-09-2049	CHSY3	ENST00000305031	p.L851F	c.2553A>T	Verified	TGC	TTA	GGA	3	A	T
TCGA-04-1347	CLDN17	ENST00000286808	p.E48D	c.144A>T	Verified	TGG	GAA	GGG	3	A	T
TCGA-29-1785	CLLU1OS	ENST00000378487	p.T52S	c.154A>T	Unverified	CTA	ACA	AGT	1	A	T
TCGA-29-1785	CLLU1OS	ENST00000378487	p.T52S	c.154A>T	Unverified	CTA	ACA	AGT	1	A	T
TCGA-30-1855	CLNK	ENST00000226951	p.L187F	c.561A>T	Unverified	CCT	TTA	TCT	3	A	T
TCGA-30-1855	CLNK_ENST(	ENST00000226951	p.L187F	c.561A>T	Unverified	CCT	TTA	TCT	3	A	T
TCGA-29-1781	COL11A1	ENST00000358392	p.T1490S	c.4468A>T	Unverified	GGA	ACT	CAA	1	A	T
TCGA-29-1781	COL11A1_EI	ENST00000370096	p.T1478S	c.4432A>T	Unverified	GGA	ACT	CAA	1	A	T
TCGA-23-1122	COL11A2	ENST00000341947	p.K962N	c.2886A>T	Unverified	GGA	AAA	GAA	3	A	T
TCGA-04-1638	COL19A1	ENST00000322773	p.K560M	c.1679A>T	Unverified	GAA	AAG	GGA	2	A	T
TCGA-30-1855	COL22A1	ENST00000303045	p.T1478S	c.4432A>T	Unverified	GAA	ACC	AGA	1	A	T
TCGA-13-0924	CPA6	ENST00000297770	p.K255I	c.764A>T	Verified	AGA	AAA	ACA	2	A	T
TCGA-24-2288	CRTAC1	ENST00000370597	p.N444I	c.1331A>T	Unverified	AAC	AAC	TGG	2	A	T
TCGA-13-0923	CRY1	ENST00000008527	p.K228N	c.684A>T	Verified	AGA	AAA	GCT	3	A	T
TCGA-24-1423	CSF3R	ENST00000373103	p.E363D	c.1089A>T	Verified	GAG	GAA	GAC	3	A	T
TCGA-23-1122	CSMD1_ENS	ENST00000318252	p.R2921S	c.8763A>T	Verified	TGC	CAA	GCT	3	A	T
TCGA-20-1683	CSMD1_ENS	ENST00000537824	p.Q3451H	c.10353A>T	Unverified	TTT	CAA	GGT	3	A	T
TCGA-24-2035	CSMD3	ENST00000297405	p.R1885*	c.5653A>T	Verified	cca	aga	ttc	1	A	T
TCGA-61-1910	CTAGE8	ENST00000487179	p.K317I	c.950A>T	Unverified	AAG	AAA	CTG	2	A	T
TCGA-25-2392	CXCL9	ENST00000264888	p.K104N	c.312A>T	Verified	aaa	aaa	cat	3	A	T
TCGA-23-1032	DACH2	ENST00000373125	p.K526M	c.1577A>T	Verified	acc	aag	aga	2	A	T
TCGA-23-1032	DACH2_ENS	ENST00000373131	p.K513M	c.1538A>T	Verified	ACC	AAG	AGA	2	A	T
TCGA-23-1022	DEFB114	ENST00000322066	p.T22S	c.64A>T	Verified	TGT	ACC	TTG	1	A	T
TCGA-13-0887	DGKA	ENST00000331886	p.E700D	c.2100A>T	Verified	gga	gaa	ccc	3	A	T
TCGA-24-1616	DIAPH2	ENST00000324765	p.I333L	c.997A>T	Verified	ttt	ata	aat	1	A	T
TCGA-23-1032	DIAPH2	ENST00000324765	p.K946M	c.2837A>T	Verified	gaa	aag	atg	2	A	T
TCGA-24-2280	DNAH7	ENST00000312428	p.K3457I	c.10370A>T	Unverified	TCA	AAA	CTT	2	A	T
TCGA-04-1361	DNAH8	NM_001371.1	p.K4454M	c.13361A>T	Verified	tac	aag	aaa	2	A	T
TCGA-04-1361	DNAH8	NM_001371.1	p.K4454M	c.13361A>T	Verified	tac	aag	aaa	2	A	T
TCGA-04-1530	DNAH8	NM_001371.1	p.N2531I	c.7592A>T	Verified	att	aat	gag	2	A	T
TCGA-13-0755	DOCK8	ENST00000432829	p.K624*	c.1870A>T	Verified	GAA	AAA	TTG	1	A	T
TCGA-13-0924	DQX1	ENST00000393951	p.E450D	c.1350A>T	Verified	CTA	GAA	CTC	3	A	T
TCGA-24-1469	DSG2	ENST00000261590	p.K536N	c.1608A>T	Verified	GAC	AAA	CCA	3	A	T

TCGA-29-1761	DSN1	ENST00000373750	p.K190I	c.569A>T	Unverified	CAA	AAA	TGT	2	A	T
TCGA-25-1318	DST_ENST0(	ENST00000370769	p.T4615S	c.13843A>T	Verified	TTA	ACA	ATT	1	A	T
TCGA-25-1318	DST_ENST0(	ENST00000370769	p.T4615S	c.13843A>T	Verified	TTA	ACA	ATT	1	A	T
TCGA-13-0755	DUSP9	ENST00000370167	p.N326Y	c.976A>T	Verified	tct	aac	atc	1	A	T
TCGA-24-0979	DUSP9	ENST00000370167	p.N332I	c.995A>T	Verified	ttc	aac	ttc	2	A	T
TCGA-29-1770	E2F5	ENST00000416274	p.K237*	c.709A>T	Unverified	TCT	AAG	CCC	1	A	T
TCGA-29-1775	EGLN1	ENST00000366641	p.K249M	c.746A>T	Unverified	tcc	aag	gac	2	A	T
TCGA-09-2050	ELMO1	ENST00000310758	p.I121I	c.363A>T	Verified	TTT	ATA	AAC	3	A	T
TCGA-04-1655	EMCN	ENST00000296420	p.M215L	c.643A>T	Unverified	CGA	ATG	TGC	1	A	T
TCGA-61-2102	ENSG00000:	ENST00000301831	p.K34*	c.100A>T	Verified	ATT	AAA	TCT	1	A	T
TCGA-24-1850	ENSG00000:	ENST00000325845	p.K143*	c.427A>T	Unverified	GGA	AAG	AGG	1	A	T
TCGA-13-1501	ERMAP	ENST00000372514	p.Y298F	c.893A>T	Verified	GTG	TAT	GTG	2	A	T
TCGA-13-1501	ERMAP	ENST00000372514	p.Y298F	c.893A>T	Verified	GTG	TAT	GTG	2	A	T
TCGA-61-2113	ETV5	ENST00000306376	p.L46L	c.138A>T	Verified	GAG	CTA	TTT	3	A	T
TCGA-13-0920	F5	ENST00000367797	p.I2196F	c.6586A>T	Verified	CCA	ATC	ATT	1	A	T
TCGA-29-1763	FABP12	ENST00000360464	p.K10M	c.29A>T	Unverified	TGG	AAG	TCC	2	A	T
TCGA-29-1763	FABP12_EN!	ENST00000360464	p.K10M	c.29A>T	Unverified	TGG	AAG	TCC	2	A	T
TCGA-25-2392	FAM105B	ENST00000284274	p.K233N	c.699A>T	Verified	GTA	AAA	TTT	3	A	T
TCGA-24-2289	FAM161A	ENST00000307507	p.L283F	c.849A>T	Unverified	CAT	TTA	CAG	3	A	T
TCGA-29-1761	FAM161A	ENST00000307507	p.V129V	c.387A>T	Unverified	ACA	GTA	CCG	3	A	T
TCGA-29-1761	FAM161A_E	ENST00000404929	p.V238V	c.714A>T	Unverified	ACA	GTA	CCG	3	A	T
TCGA-29-1761	FAM161A_E	ENST00000405894	p.V238V	c.714A>T	Unverified	ACA	GTA	CCG	3	A	T
TCGA-25-1326	FAM194A	ENST00000295910	p.K624*	c.1870A>T	Verified	GAA	AAA	TTA	1	A	T
TCGA-29-1763	FAM60A	ENST00000337682	p.S105C	c.313A>T	Unverified	AAA	AGC	AAC	1	A	T
TCGA-09-2056	FAM71A	ENST00000294829	p.K106M	c.317A>T	Verified	ACC	AAG	AGA	2	A	T
TCGA-13-1510	FAT2	ENST00000261800	p.V3024V	c.9072A>T	Unverified	GAT	GTA	TTT	3	A	T
TCGA-13-0890	FBXO18	ENST00000379999	p.I629F	c.1885A>T	Verified	cct	att	tgg	1	A	T
TCGA-04-1331	FBXO28	ENST00000366862	p.N133Y	c.397A>T	Verified	aga	aac	cat	1	A	T
TCGA-61-1998	FLJ25006	ENST00000301037	p.K130N	c.390A>T	Verified	CAG	AAA	GCT	3	A	T
TCGA-10-0930	FRAS1_ENST	ENST00000325942	p.Y1040F	c.3119A>T	Verified	GGG	TAC	TTT	2	A	T
TCGA-10-0930	FRAS1_ENST	ENST00000325942	p.Y1040F	c.3119A>T	Verified	GGG	TAC	TTT	2	A	T
TCGA-23-1110	FSTL5	ENST00000306100	p.K533N	c.1599A>T	Verified	CAA	AAA	GTT	3	A	T
TCGA-23-2078	GAD1	ENST00000358196	p.Q115H	c.345A>T	Verified	GTG	CAA	TTC	3	A	T
TCGA-24-1103	GALNT2	ENST00000366672	p.K323M	c.968A>T	Verified	GGG	AAG	TAC	2	A	T

TCGA-23-1114	GATA3	ENST00000379328	p.N393I	c.1178A>T	Unverified	ttt	aac	ccg	2	A	T
TCGA-25-2391	GFRA1	ENST00000369236	p.N276I	c.827A>T	Unverified	GAA	AAC	TAC	2	A	T
TCGA-24-1422	GGNBP2	ENST00000304718	p.K134M	c.401A>T	Verified	AAG	AAG	CTT	2	A	T
TCGA-29-1691	GNPAT	ENST00000366647	p.K304I	c.911A>T	Unverified	CCA	AAA	GAG	2	A	T
TCGA-04-1361	GNPDA2	ENST00000295448	p.K126*	c.376A>T	Verified	ATA	AAA	GAA	1	A	T
TCGA-04-1361	GNPDA2	ENST00000295448	p.K126*	c.376A>T	Verified	ATA	AAA	GAA	1	A	T
TCGA-29-1769	GOLGB1	ENST00000340645	p.K1741M	c.5222A>T	Unverified	AAG	AAG	TTT	2	A	T
TCGA-13-0760	GOLGB1	ENST00000340645	p.E1265D	c.3795A>T	Verified	GAA	GAA	CCT	3	A	T
TCGA-24-2290	GREB1_ENST	ENST00000381486	p.N1084I	c.3251A>T	Verified	AGG	AAC	GAG	2	A	T
TCGA-24-2290	GREB1_ENST	ENST00000381486	p.N1084I	c.3251A>T	Verified	AGG	AAC	GAG	2	A	T
TCGA-30-1891	GRK6	ENST00000355472	p.N22Y	c.64A>T	Unverified	GGA	AAT	CGC	1	A	T
TCGA-61-1738	GRLF1	ENST00000317082	p.K368*	c.1102A>T	Unverified	AAA	AAG	CTC	1	A	T
TCGA-61-1738	GRLF1	ENST00000317082	p.K368*	c.1102A>T	Unverified	AAA	AAG	CTC	1	A	T
TCGA-61-1738	GRLF1_ENST	ENST00000317082_v61	p.K368*	c.1102A>T	Unverified	AAA	AAG	CTC	1	A	T
TCGA-61-1738	GRLF1_ENST	ENST00000317082_v61	p.K368*	c.1102A>T	Unverified	AAA	AAG	CTC	1	A	T
TCGA-13-0762	GSPT2	ENST00000340438	p.Q578H	c.1734A>T	Unverified	aaa	caa	gat	3	A	T
TCGA-13-0762	GSPT2	ENST00000340438	p.Q578H	c.1734A>T	Unverified	aaa	caa	gat	3	A	T
TCGA-24-2267	GSTA2	ENST00000493422	p.I35L	c.103A>T	Verified	TTT	ATA	AAA	1	A	T
TCGA-29-1702	HAUS2	ENST00000260372	p.K165N	c.495A>T	Unverified	AAG	AAA	ATG	3	A	T
TCGA-30-1855	HEATR1	ENST00000366582	p.K1656N	c.4968A>T	Unverified	AAG	AAA	AAG	3	A	T
TCGA-24-1844	HEATR5A	ENST00000389961	p.M1033L	c.3097A>T	Unverified	GTA	ATG	CAA	1	A	T
TCGA-24-1844	HEATR5A_EI	ENST00000389961_v65	p.M1033L	c.3097A>T	Unverified	GTA	ATG	CAA	1	A	T
TCGA-61-1910	HIAT1	ENST00000370152	p.T252S	c.754A>T	Unverified	ATT	ACA	GTG	1	A	T
TCGA-13-0900	HMCN1	ENST00000271588	p.T3598S	c.10792A>T	Verified	TAT	ACA	TGT	1	A	T
TCGA-09-2056	HPSE2	ENST00000370552	p.K160I	c.479A>T	Verified	GAT	AAA	CAG	2	A	T
TCGA-13-0890	HRH4	ENST00000256906	p.Y358F	c.1073A>T	Verified	TTG	TAT	CCA	2	A	T
TCGA-23-2078	HSPA5	ENST00000324460	p.I426F	c.1276A>T	Verified	ggt	att	gaa	1	A	T
TCGA-61-1738	HSPA5	ENST00000324460	p.E614D	c.1842A>T	Unverified	att	gaa	gac	3	A	T
TCGA-61-1738	HSPA5	ENST00000324460	p.E614D	c.1842A>T	Unverified	att	gaa	gac	3	A	T
TCGA-61-1907	HUWE1	XM_497119.1	p.Y3996F	c.11987A>T	Unverified	agc	tac	ttc	2	A	T
TCGA-61-1907	HUWE1_ENS	ENST00000342160	p.Y4106F	c.12317A>T	Unverified	AGC	TAC	TTC	2	A	T
TCGA-30-1718	IDH3A	ENST00000299518	p.K90*	c.268A>T	Unverified	gat	aag	aac	1	A	T
TCGA-24-1846	IL33	ENST00000381434	p.N171Y	c.511A>T	Unverified	TCA	AAT	GAA	1	A	T
TCGA-24-1469	IL6ST	ENST00000381298	p.I253I	c.759A>T	Verified	gtt	ata	ata	3	A	T

TCGA-13-0883	INSR_ENST0	ENST00000302850	p.K1047M	c.3140A>T	Verified	ATC	AAG	GGT	2	A	T
TCGA-61-2009	IQCC	ENST00000291358	p.Y33F	c.98A>T	Verified	GAG	TAT	GAG	2	A	T
TCGA-61-2008	IRX6	ENST00000290552	p.K176M	c.527A>T	Verified	ACT	AAG	GGT	2	A	T
TCGA-09-2049	ITGA4	ENST00000397033	p.K363N	c.1089A>T	Verified	GAC	AAA	TAT	3	A	T
TCGA-23-1122	ITIH1	ENST00000273283	p.T220S	c.658A>T	Verified	CAA	ACT	ATC	1	A	T
TCGA-61-1910	KCNA6	ENST00000280684	p.Y307F	c.920A>T	Unverified	CCC	TAC	TTC	2	A	T
TCGA-13-0761	KCNJ15	ENST00000398930	p.Y355F	c.1064A>T	Verified	AAG	TAC	AGG	2	A	T
TCGA-13-0761	KCNJ15	ENST00000398930	p.Y355F	c.1064A>T	Verified	AAG	TAC	AGG	2	A	T
TCGA-13-0885	KCNQ5	ENST00000370398	p.K664N	c.1992A>T	Verified	AGC	AAA	GAT	3	A	T
TCGA-29-1770	KIAA1324L	ENST00000297222	p.K669*	c.2005A>T	Unverified	ATT	AAA	GGA	1	A	T
TCGA-29-1770	KIAA1324L_	ENST00000450689	p.K909*	c.2725A>T	Unverified	ATT	AAA	GGA	1	A	T
TCGA-29-1777	KIF26B	ENST00000407071	p.K195M	c.584A>T	Unverified	TTG	AAG	CAG	2	A	T
TCGA-29-1777	KIF26B_ENS	ENST00000407071	p.K195M	c.584A>T	Unverified	TTG	AAG	CAG	2	A	T
TCGA-13-0903	LIPI	ENST00000344577	p.R335W	c.1003A>T	Verified	GAA	AGG	ATG	1	A	T
TCGA-61-1910	LPCAT1	ENST00000283415	p.K185N	c.555A>T	Unverified	AGG	AAA	ACA	3	A	T
TCGA-13-0807	LRCH2_ENS	ENST00000317135	p.K743N	c.2229A>T	Verified	GTG	AAA	GTT	3	A	T
TCGA-13-1510	LRMP	ENST00000354454	p.N309I	c.926A>T	Unverified	cta	aac	tcc	2	A	T
TCGA-09-0369	LRP2	ENST00000263816	p.N682I	c.2045A>T	Verified	GAT	AAT	GAT	2	A	T
TCGA-09-0369	LRP2	ENST00000263816	p.N682I	c.2045A>T	Verified	GAT	AAT	GAT	2	A	T
TCGA-29-1761	LRP2BP	ENST00000328559	p.N24I	c.71A>T	Unverified	AAA	AAC	CAA	2	A	T
TCGA-23-1124	LRRC1	ENST00000370888	p.N115I	c.344A>T	Verified	GGA	AAC	CCA	2	A	T
TCGA-24-1844	LRRC41	ENST00000343304	p.?	c.358-2A>T	Unverified	GAA	AGT	GTG	1	A	T
TCGA-13-1498	LRRIQ3	ENST00000395089	p.S549C	c.1645A>T	Verified	AAA	AGC	CTG	1	A	T
TCGA-13-0885	MAGEA1	ENST00000356661	p.K278I	c.833A>T	Verified	GTG	AAA	GTC	2	A	T
TCGA-13-0760	MAGED1	ENST00000375695	p.K131*	c.391A>T	Verified	cct	aag	tca	1	A	T
TCGA-23-1022	MANEA	ENST00000358812	p.N58I	c.173A>T	Verified	AAA	AAT	TTT	2	A	T
TCGA-29-1693	MAP1B	ENST00000296755	p.E1304D	c.3912A>T	Unverified	CAA	GAA	GTA	3	A	T
TCGA-25-1313	MAP2K2_EN	ENST00000262948	p.K39M	c.116A>T	Unverified	CAG	AAG	AAG	2	A	T
TCGA-61-2009	MASP2	ENST00000400897	p.S546C	c.1636A>T	Verified	AAT	AGC	AAC	1	A	T
TCGA-13-1497	MCART1	ENST00000377716	p.N187I	c.560A>T	Verified	AGC	AAT	GTC	2	A	T
TCGA-24-2024	MCC	ENST00000302475	p.K29M	c.86A>T	Verified	CTA	AAG	GGA	2	A	T
TCGA-24-2024	MCC_ENST0	ENST00000408903	p.K219M	c.656A>T	Verified	CTA	AAG	GGA	2	A	T
TCGA-13-0885	MCMBP	ENST00000360003	p.N25I	c.74A>T	Verified	GTT	AAT	CCT	2	A	T
TCGA-20-1683	MCTS1	ENST00000371317	p.L170F	c.510A>T	Unverified	tat	tta	aat	3	A	T

TCGA-20-1683	MCTS1_ENS	ENST00000371315	p.L171F	c.513A>T	Unverified	TAT	TTA	AAT	3	A	T
TCGA-24-1474	MET	ENST00000318493	p.L238F	c.714A>T	Verified	GTT	TTA	CCT	3	A	T
TCGA-13-0760	MIF	ENST00000215754	p.N98I	c.293A>T	Unverified	ATC	AAC	TAT	2	A	T
TCGA-29-1691	MLL3	ENST00000262189	p.I3488L	c.10462A>T	Unverified	AAT	ATA	CAA	1	A	T
TCGA-29-1691	MLL3_ENST(	ENST00000355193	p.I3488L	c.10462A>T	Unverified	AAT	ATA	CAA	1	A	T
TCGA-13-1501	MORC4	NM_024657.2	p.T227S	c.679A>T	Verified	aaa	aca	tct	1	A	T
TCGA-13-1501	MORC4	NM_024657.2	p.T227S	c.679A>T	Verified	aaa	aca	tct	1	A	T
TCGA-30-1718	MPDZ	NM_003829.1	p.I126F	c.376A>T	Unverified	ctt	atc	aaa	1	A	T
TCGA-30-1718	MPDZ_ENST	ENST00000541718	p.I126F	c.376A>T	Unverified	CTT	ATC	AAA	1	A	T
TCGA-24-2280	MTERFD1	ENST00000287025	p.N326I	c.977A>T	Unverified	GCA	AAT	AAA	2	A	T
TCGA-23-1022	MUC17	ENST00000306151	p.T3099S	c.9295A>T	Verified	TCA	ACT	TAT	1	A	T
TCGA-13-0887	MUM1L1	NM_152423.2	p.S412C	c.1234A>T	Verified	aaa	agt	atc	1	A	T
TCGA-23-1120	MYH15	ENST00000273353	p.K1569M	c.4706A>T	Verified	AGC	AAG	ATT	2	A	T
TCGA-29-1781	MYO18B	ENST00000335473	p.K2216M	c.6647A>T	Unverified	AGA	AAG	TCC	2	A	T
TCGA-25-1326	NAV3	NM_014903.3	p.T1360S	c.4078A>T	Verified	gat	act	ccg	1	A	T
TCGA-13-0714	NCK2	ENST00000233154	p.I6F	c.16A>T	Verified	GTT	ATT	GTG	1	A	T
TCGA-61-1895	NDST4	ENST00000264363	p.N321I	c.962A>T	Unverified	ATG	AAT	GTC	2	A	T
TCGA-29-1761	NEGR1	ENST00000357731	p.K259I	c.776A>T	Unverified	TAC	AAA	GGA	2	A	T
TCGA-29-1784	NEURL	ENST00000369780	p.K109M	c.326A>T	Unverified	CTG	AAG	ATC	2	A	T
TCGA-29-1775	NIPBL	ENST00000282516	p.K1187*	c.3559A>T	Unverified	cca	aaa	cta	1	A	T
TCGA-29-1775	NIPBL_ENST	ENST00000448238	p.K1187*	c.3559A>T	Unverified	CCA	AAA	CTA	1	A	T
TCGA-13-1481	NKIRAS2	ENST00000307641	p.K101M	c.302A>T	Verified	ctc	aag	aag	2	A	T
TCGA-23-1022	NLRP13	ENST00000342929	p.E404D	c.1212A>T	Verified	AGT	GAA	GTT	3	A	T
TCGA-61-2008	NNT	ENST00000264663	p.E725D	c.2175A>T	Verified	ATA	GAA	TAT	3	A	T
TCGA-13-1489	NOTCH4	ENST00000375023	p.Q975H	c.2925A>T	Verified	tcc	caa	ccc	3	A	T
TCGA-23-1031	NPHP3	ENST00000337331	p.K319M	c.956A>T	Verified	CTT	AAG	GAC	2	A	T
TCGA-13-0900	NPR3	ENST00000265074	p.L492F	c.1476A>T	Verified	GCT	TTA	CTA	3	A	T
TCGA-29-1703	NRG2	ENST00000361474	p.K286N	c.858A>T	Unverified	ATC	AAA	TAT	3	A	T
TCGA-29-1703	NRG2_ENST	ENST00000544729	p.K194N	c.582A>T	Unverified	ATC	AAA	TAT	3	A	T
TCGA-61-2095	NUP205	ENST00000285968	p.T1176S	c.3526A>T	Unverified	GCT	ACA	AAA	1	A	T
TCGA-24-2267	ODZ1	ENST00000371130	p.T671S	c.2011A>T	Verified	GGA	ACT	TTT	1	A	T
TCGA-13-0916	ODZ4	ENST00000278550	p.N1835I	c.5504A>T	Verified	CAC	AAC	CGA	2	A	T
TCGA-25-2393	OR2G6	ENST00000343414	p.I256I	c.768A>T	Verified	ATC	ATA	TTC	3	A	T
TCGA-24-1850	OR2T2	ENST00000342927	p.K304N	c.912A>T	Unverified	AGG	AAA	GTA	3	A	T

TCGA-23-1022	OR4N2	ENST00000315947	p.N303I	c.908A>T	Verified	TTT	AAT	AAG	2	A	T
TCGA-24-2289	OR4S2	ENST00000312422	p.K23N	c.69A>T	Unverified	GAG	AAA	GTT	3	A	T
TCGA-23-1110	OR5AN1	ENST00000313940	p.I104F	c.310A>T	Verified	TTT	ATC	TTT	1	A	T
TCGA-24-1422	OR5H1	ENST00000354565	p.N286I	c.857A>T	Verified	TTA	AAT	CCT	2	A	T
TCGA-29-1696	OR5M1_ENS	ENST00000526538	p.E11D	c.33A>T	Unverified	ACA	GAA	TTC	3	A	T
TCGA-29-1696	OR5M1_ENS	ENST00000526538	p.E11D	c.33A>T	Unverified	ACA	GAA	TTC	3	A	T
TCGA-23-1809	OR8B3	ENST00000354597	p.N195I	c.584A>T	Unverified	GTC	AAC	GAG	2	A	T
TCGA-24-1616	ORC4	ENST00000264169	p.I66F	c.196A>T	Verified	att	atc	gga	1	A	T
TCGA-24-1469	OTC	ENST00000039007	p.L9F	c.27A>T	Verified	CTG	TTA	AAC	3	A	T
TCGA-13-1499	PADI3	ENST00000375460	p.K382N	c.1146A>T	Unverified	TAC	AAA	AGA	3	A	T
TCGA-24-1470	PCLO	ENST00000333891	p.Q3737H	c.11211A>T	Verified	ACT	CAA	TCC	3	A	T
TCGA-61-1740	PCSK9	ENST00000302118	p.T162S	c.484A>T	Unverified	ATT	ACC	CCT	1	A	T
TCGA-30-1857	PEX11A	ENST00000300056	p.K57N	c.171A>T	Unverified	CGT	AAA	TGG	3	A	T
TCGA-61-1914	PICALM	ENST00000447890	p.Q616H	c.1848A>T	Unverified	CCA	CAA	ATG	3	A	T
TCGA-29-1777	PIK3C2G	ENST00000433979	p.Y516F	c.1547A>T	Unverified	TCC	TAT	CTA	2	A	T
TCGA-29-1777	PIK3C2G_EN	ENST00000433979	p.Y516F	c.1547A>T	Unverified	TCC	TAT	CTA	2	A	T
TCGA-24-0979	PIKFYVE	ENST00000264380	p.K1446*	c.4336A>T	Verified	GAA	AAA	ATG	1	A	T
TCGA-23-1114	PLAC1	ENST00000359237	p.Y94F	c.281A>T	Unverified	atc	tac	agc	2	A	T
TCGA-61-1900	PLG	ENST00000308192	p.K528*	c.1582A>T	Unverified	GAA	AAA	AAT	1	A	T
TCGA-23-1118	PNLIPRP3	ENST00000369230	p.S415C	c.1243A>T	Verified	ACA	AGT	GTT	1	A	T
TCGA-04-1361	POLQ	NM_006596.3	p.I1594I	c.4782A>T	Verified	gtt	ata	ctt	3	A	T
TCGA-04-1361	POLQ	NM_006596.3	p.I1594I	c.4782A>T	Verified	gtt	ata	ctt	3	A	T
TCGA-13-0887	POLR2B	ENST00000381227	p.T761S	c.2281A>T	Verified	ACT	ACA	CGG	1	A	T
TCGA-29-1775	PPAT	ENST00000264220	p.S145C	c.433A>T	Unverified	ACA	AGT	TCT	1	A	T
TCGA-29-1691	PPFIA2	ENST00000549396	p.R877*	c.2629A>T	Unverified	cga	aga	cta	1	A	T
TCGA-13-1496	PPP1R12B	NM_032105.1	p.M775L	c.2323A>T	Verified	gaa	atg	gac	1	A	T
TCGA-24-1469	PPP1R3A	NM_002711.2	p.L156F	c.468A>T	Verified	aag	tta	gta	3	A	T
TCGA-29-1770	PPP4R4	ENST00000304338	p.K509I	c.1526A>T	Unverified	CAG	AAA	TAT	2	A	T
TCGA-24-1431	PRB4	ENST00000279575	p.N212I	c.635A>T	Verified	GGC	AAT	CCC	2	A	T
TCGA-29-1761	PRDM11	ENST00000263765	p.K407M	c.1220A>T	Unverified	TCC	AAG	GAG	2	A	T
TCGA-29-1761	PRDM9	ENST00000296682	p.T160S	c.478A>T	Unverified	AGT	ACC	TCT	1	A	T
TCGA-23-1122	PRIC285	ENST00000467148	p.K2441M	c.7322A>T	Unverified	TAC	AAG	AGC	2	A	T
TCGA-13-0762	PRICKLE2	ENST00000295902	p.N389I	c.1166A>T	Verified	CTC	AAC	CGG	2	A	T
TCGA-13-0762	PRICKLE2	ENST00000295902	p.N389I	c.1166A>T	Verified	CTC	AAC	CGG	2	A	T

TCGA-04-1361	PRPH2	ENST00000230381	p.Q18H	c.54A>T	Verified	GCC	CAA	GGG	3	A	T
TCGA-04-1361	PRPH2	ENST00000230381	p.Q18H	c.54A>T	Verified	GCC	CAA	GGG	3	A	T
TCGA-29-1698	PRSS58	ENST00000547058	p.K226I	c.677A>T	Unverified	GCC	AAA	ATT	2	A	T
TCGA-30-1718	PSG8	ENST00000306511	p.E348D	c.1044A>T	Unverified	GGA	GAA	GTC	3	A	T
TCGA-24-1422	PTPRZ1	ENST00000393386	p.N468I	c.1403A>T	Unverified	TAC	AAT	CGC	2	A	T
TCGA-24-1422	PTPRZ1_ENS	ENST00000393386	p.N468I	c.1403A>T	Unverified	TAC	AAT	CGC	2	A	T
TCGA-61-1895	Q8N8K0_HU	ENST00000301807	p.Y1244F	c.3731A>T	Unverified	GCC	TAT	TTC	2	A	T
TCGA-61-1725	RAD17_ENS	ENST00000361732	p.I246L	c.736A>T	Unverified	TTT	ATA	ATC	1	A	T
TCGA-13-0887	RALGAPA2	ENST00000202677	p.K667N	c.2001A>T	Verified	GAT	AAA	TTA	3	A	T
TCGA-24-2289	RASGRF2	ENST00000265080	p.Y1106F	c.3317A>T	Unverified	atc	tac	agg	2	A	T
TCGA-13-1507	RBM7	ENST00000540163	p.Q167H	c.501A>T	Verified	GAT	CAA	TCA	3	A	T
TCGA-24-1845	RHEB	ENST00000262187	p.E53D	c.159A>T	Unverified	caa	gaa	tat	3	A	T
TCGA-29-1761	RIF1	ENST00000444746	p.N2083Y	c.6247A>T	Unverified	gct	aat	aaa	1	A	T
TCGA-13-0887	RIMS1	ENST00000521978	p.R1113W	c.3337A>T	Verified	GCT	AGG	AGT	1	A	T
TCGA-13-0887	RNASE11	ENST00000398008	p.S173C	c.517A>T	Verified	CAT	AGT	GTT	1	A	T
TCGA-23-1117	RNASEL	ENST00000367559	p.T222S	c.664A>T	Verified	ATT	ACG	CAT	1	A	T
TCGA-23-1117	RNASEL	ENST00000367559	p.T222S	c.664A>T	Verified	ATT	ACG	CAT	1	A	T
TCGA-23-1022	RNF111	ENST00000348370	p.K915I	c.2744A>T	Verified	AGG	AAA	CTG	2	A	T
TCGA-24-1850	RP11-419C5	ENST00000532298	p.K175*	c.523A>T	Unverified	GGA	AAG	AGG	1	A	T
TCGA-24-1850	RP11-419C5	ENST00000532298_v68	p.K175*	c.523A>T	Unverified	GGA	AAG	AGG	1	A	T
TCGA-24-1844	RPGRIP1_EN	ENST00000400017	p.R73W	c.217A>T	Unverified	AAA	AGG	CTG	1	A	T
TCGA-23-1114	RPUSD4	ENST00000298317	p.N366I	c.1097A>T	Unverified	CAA	AAT	GAG	2	A	T
TCGA-13-0792	SCAF11	ENST00000369367	p.Q101H	c.303A>T	Verified	GTT	CAA	GTA	3	A	T
TCGA-25-2391	SCAND3	ENST00000452236	p.K772I	c.2315A>T	Unverified	ATG	AAA	CCA	2	A	T
TCGA-23-1022	SCLY	ENST00000254663	p.Y269F	c.806A>T	Verified	TTT	TAT	GGT	2	A	T
TCGA-23-1120	SCN11A	ENST00000302328	p.M1508L	c.4522A>T	Unverified	ATT	ATG	TTT	1	A	T
TCGA-29-1784	SCN4A	ENST00000435607	p.K1329M	c.3986A>T	Unverified	TCC	AAG	AAG	2	A	T
TCGA-24-0979	SCN8A	ENST00000354534	p.T1888S	c.5662A>T	Verified	ACA	ACC	ACA	1	A	T
TCGA-04-1362	SCN9A	ENST00000409672	p.T465S	c.1393A>T	Verified	GAA	ACA	TCC	1	A	T
TCGA-09-0369	SCN9A	ENST00000409672	p.N1109Y	c.3325A>T	Verified	TTA	AAC	CGG	1	A	T
TCGA-09-0369	SCN9A	ENST00000409672	p.N1109Y	c.3325A>T	Verified	TTA	AAC	CGG	1	A	T
TCGA-61-2012	SELE	ENST00000333360	p.V84V	c.252A>T	Verified	TGG	GTA	GGA	3	A	T
TCGA-09-2050	SEMA3A	ENST00000265362	p.N43I	c.128A>T	Verified	TCC	AAC	AAT	2	A	T
TCGA-61-1914	SEMA3E	ENST00000307792	p.T427S	c.1279A>T	Unverified	AAA	ACA	GAT	1	A	T

TCGA-23-1021	SENP7	NM_020654.2	p.K467*	c.1399A>T	Verified	ata	aaa	gga	1	A	T
TCGA-13-0904	SERPINB5	ENST00000382771	p.M307L	c.919A>T	Verified	GGA	ATG	TCA	1	A	T
TCGA-61-1998	SgK494	SU_SgK494	p.K169N	c.507A>T	Verified	cag	aaa	gct	3	A	T
TCGA-13-0714	SLC16A13	ENST00000308027	p.Y166F	c.497A>T	Verified	CAC	TAC	GCC	2	A	T
TCGA-24-1616	SLCO1B1	ENST00000256958	p.K160I	c.479A>T	Verified	GGA	AAA	GGT	2	A	T
TCGA-20-0991	SLCO1C1	ENST00000266509	p.K481N	c.1443A>T	Verified	TGC	AAA	TGT	3	A	T
TCGA-13-0900	SLFN5	ENST00000299977	p.L488F	c.1464A>T	Verified	AGG	TTA	TGC	3	A	T
TCGA-13-0924	SLIT1	ENST00000266058	p.N1026I	c.3077A>T	Verified	GGC	AAC	TAC	2	A	T
TCGA-29-1777	SMC1B	NM_148674.1	p.I83L	c.247A>T	Unverified	att	ata	tat	1	A	T
TCGA-29-1777	SMC1B_ENS	ENST00000357450	p.I83L	c.247A>T	Unverified	ATT	ATA	TAT	1	A	T
TCGA-29-1696	SMC4	NM_005496.2	p.K153M	c.458A>T	Unverified	cac	aag	gac	2	A	T
TCGA-29-1696	SMC4	NM_005496.2	p.K153M	c.458A>T	Unverified	cac	aag	gac	2	A	T
TCGA-24-1422	SORCS3	ENST00000369701	p.N1154I	c.3461A>T	Verified	ATT	AAC	ATC	2	A	T
TCGA-04-1347	SOX5	ENST00000451604	p.Q761H	c.2283A>T	Verified	gga	caa	gcc	3	A	T
TCGA-13-1509	SP110	ENST00000258381	p.K593*	c.1777A>T	Verified	TCT	AAG	ACC	1	A	T
TCGA-24-1843	SPTLC3	ENST00000399002	p.T347S	c.1039A>T	Unverified	CCA	ACC	GGC	1	A	T
TCGA-13-0887	ST7	ENST00000265437	p.E214D	c.642A>T	Unverified	AAT	GAA	ATT	3	A	T
TCGA-24-1563	STARD13	ENST00000336934	p.V103V	c.309A>T	Verified	CTT	GTA	GAA	3	A	T
TCGA-61-1907	SURF4	ENST00000371989	p.N120I	c.359A>T	Unverified	AGG	AAC	CTG	2	A	T
TCGA-04-1542	SVEP1	ENST00000401783	p.K189N	c.567A>T	Verified	ACA	AAA	GTT	3	A	T
TCGA-24-1563	SYCP2	ENST00000357552	p.K1427*	c.4279A>T	Verified	GAA	AAA	GAT	1	A	T
TCGA-04-1336	SYNGAP1	ENST00000418600	p.K793*	c.2377A>T	Verified	GAA	AAG	CCA	1	A	T
TCGA-04-1336	SYNGAP1_EI	ENST00000293748	p.K793*	c.2377A>T	Verified	GAA	AAG	CCA	1	A	T
TCGA-29-1699	SYT11	ENST00000368324	p.S138C	c.412A>T	Unverified	ACA	AGC	CTG	1	A	T
TCGA-25-2391	TBX15	ENST00000207157	p.Y481F	c.1442A>T	Unverified	CAG	TAT	GGG	2	A	T
TCGA-24-1845	TESK1	ENST00000336395	p.Q81H	c.243A>T	Unverified	GGG	CAA	GTC	3	A	T
TCGA-13-0920	TFAP2D	ENST00000008391	p.T414S	c.1240A>T	Verified	ACT	ACT	CAC	1	A	T
TCGA-25-2392	TFCP2	ENST00000257915	p.Q68H	c.204A>T	Verified	TTT	CAA	TAT	3	A	T
TCGA-30-1857	TG	ENST00000220616	p.L104F	c.312A>T	Unverified	ATC	TTA	CTG	3	A	T
TCGA-10-0930	THOC7	ENST00000295899	p.E182D	c.546A>T	Verified	TTG	GAA	AAT	3	A	T
TCGA-10-0930	THOC7	ENST00000295899	p.E182D	c.546A>T	Verified	TTG	GAA	AAT	3	A	T
TCGA-24-1423	TMC3	ENST00000359440	p.S176C	c.526A>T	Verified	GGA	AGC	ACA	1	A	T
TCGA-09-2049	TMPRSS15	ENST00000284885	p.N457Y	c.1369A>T	Verified	GGA	AAT	TAT	1	A	T
TCGA-61-2009	TNC	ENST00000350763	p.Y779F	c.2336A>T	Verified	GAG	TAT	GAG	2	A	T

TCGA-61-1733	TP53	ENST00000269305	p.K305*	c.913A>T	Unverified	ACT	AAG	CGA	1	A	T
TCGA-61-1733	TP53	ENST00000269305	p.K305*	c.913A>T	Unverified	ACT	AAG	CGA	1	A	T
TCGA-61-1733	TP53_ENST(	ENST00000269305	p.K305*	c.913A>T	Unverified	ACT	AAG	CGA	1	A	T
TCGA-61-1733	TP53_ENST(	ENST00000269305	p.K305*	c.913A>T	Unverified	ACT	AAG	CGA	1	A	T
TCGA-09-2049	TRAF3IP1	ENST00000373327	p.K559N	c.1677A>T	Verified	CCC	AAA	CCT	3	A	T
TCGA-61-1895	TRANK1	ENST00000428977	p.Y1251F	c.3752A>T	Unverified	GCC	TAT	TTC	2	A	T
TCGA-61-1895	TRANK1_EN	ENST00000429976	p.Y1801F	c.5402A>T	Unverified	GCC	TAT	TTC	2	A	T
TCGA-23-2078	TRDN	ENST00000398178	p.N75I	c.224A>T	Unverified	AAA	AAC	TTT	2	A	T
TCGA-13-0916	TRIP11	ENST00000267622	p.L1868L	c.5604A>T	Verified	TTT	CTA	GAA	3	A	T
TCGA-29-1769	TRPV2	ENST00000338560	p.Y675F	c.2024A>T	Unverified	GGC	TAT	TGG	2	A	T
TCGA-30-1714	TSPYL1	ENST00000368608	p.T353S	c.1057A>T	Unverified	TCT	ACT	CCA	1	A	T
TCGA-29-2427	TTN	NM_003319	p.R23644*	c.70930A>T	Verified	gaa	aga	tac	1	A	T
TCGA-23-1122	TTN	NM_003319	p.K6268M	c.18803A>T	Verified	ctg	aag	tgg	2	A	T
TCGA-61-1900	TTN	NM_003319	p.Y11574F	c.34721A>T	Unverified	tac	tat	ttc	2	A	T
TCGA-13-0885	TTN	NM_003319	p.E1926D	c.5778A>T	Verified	act	gaa	acc	3	A	T
TCGA-61-1900	TTN_ENST0(	ENST00000342175	p.Y11766F	c.35297A>T	Unverified	TAC	TAT	TTC	2	A	T
TCGA-61-1900	TTN_ENST0(	ENST00000342992	p.Y18071F	c.54212A>T	Unverified	TAC	TAT	TTC	2	A	T
TCGA-29-2427	TTN_ENST0(	ENST00000356127	p.R30139*	c.90415A>T	Verified	GAA	AGA	TAC	1	A	T
TCGA-23-1122	TTN_ENST0(	ENST00000356127	p.K12765M	c.38294A>T	Verified	CTG	AAG	TGG	2	A	T
TCGA-61-1900	TTN_ENST0(	ENST00000356127	p.Y18069F	c.54206A>T	Unverified	TAC	TAT	TTC	2	A	T
TCGA-13-0885	TTN_ENST0(	ENST00000356127	p.E1972D	c.5916A>T	Verified	ACT	GAA	ACC	3	A	T
TCGA-61-1900	TTN_ENST0(	ENST00000359218	p.Y11699F	c.35096A>T	Unverified	TAC	TAT	TTC	2	A	T
TCGA-13-1509	TTN_ENST0(	ENST00000360870	p.N3541I	c.10622A>T	Verified	AAC	AAT	CGG	2	A	T
TCGA-13-0885	TTN_ENST0(	ENST00000360870	p.E1972D	c.5916A>T	Verified	ACT	GAA	ACC	3	A	T
TCGA-25-1313	TUBB2C	ENST00000340384	p.T351S	c.1051A>T	Unverified	AAA	ACG	GCT	1	A	T
TCGA-13-0885	UBR4	ENST00000375254	p.Y3544F	c.10631A>T	Verified	TGT	TAT	ATC	2	A	T
TCGA-13-0906	UGGT2	ENST00000376747	p.K719*	c.2155A>T	Verified	GAT	AAG	AGT	1	A	T
TCGA-24-1844	UGT1A9	ENST00000354728	p.K132*	c.394A>T	Unverified	aaa	aaa	tta	1	A	T
TCGA-61-2008	UGT1A9	ENST00000354728	p.K132I	c.395A>T	Verified	aaa	aaa	tta	2	A	T
TCGA-25-1318	USH2A	ENST00000307340	p.Q3102H	c.9306A>T	Verified	ACC	CAA	ATT	3	A	T
TCGA-25-1318	USH2A	ENST00000307340	p.Q3102H	c.9306A>T	Verified	ACC	CAA	ATT	3	A	T
TCGA-09-2050	USP34	ENST00000398571	p.N1819I	c.5456A>T	Verified	TTC	AAT	CTC	2	A	T
TCGA-09-2051	USP48	ENST00000308271	p.N580Y	c.1738A>T	Unverified	aat	aat	ctg	1	A	T
TCGA-24-1104	USP48	ENST00000308271	p.K940N	c.2820A>T	Verified	aga	aaa	gtt	3	A	T

TCGA-24-1470	USP7	ENST00000344836	p.K934I	c.2801A>T	Verified	GAG	AAA	GCA	2	A	T
TCGA-29-1764	VAV2	ENST00000406606	p.K333I	c.998A>T	Unverified	CTC	AAA	TAC	2	A	T
TCGA-29-1764	VAV2_ENST(	ENST00000371850	p.K338I	c.1013A>T	Unverified	CTC	AAA	TAC	2	A	T
TCGA-61-1998	VCL	ENST00000211998	p.E623D	c.1869A>T	Verified	AGG	GAA	GAG	3	A	T
TCGA-61-1900	WDR33	ENST00000322313	p.K119*	c.355A>T	Unverified	GTA	AAG	TGT	1	A	T
TCGA-61-1900	WDR33_ENS	ENST00000409658	p.K119*	c.355A>T	Unverified	GTA	AAG	TGT	1	A	T
TCGA-04-1649	WRN	ENST00000298139	p.Y1034F	c.3101A>T	Unverified	cgg	tat	aac	2	A	T
TCGA-29-1784	ZBED1	ENST00000381223	p.N546I	c.1637A>T	Unverified	AAC	AAC	ATG	2	A	T
TCGA-61-1895	ZFHX3	ENST00000268489	p.K2790I	c.8369A>T	Unverified	AGT	AAA	ACC	2	A	T
TCGA-24-1846	ZFHX4	ENST00000521891	p.K159I	c.476A>T	Unverified	AGC	AAA	CTC	2	A	T
TCGA-04-1347	ZNF323	ENST00000439158	p.Y267F	c.800A>T	Verified	CCA	TAT	GAA	2	A	T
TCGA-13-0761	ZNF449	ENST00000339249	p.K316M	c.947A>T	Verified	aaa	aag	agt	2	A	T
TCGA-13-0761	ZNF449	ENST00000339249	p.K316M	c.947A>T	Verified	aaa	aag	agt	2	A	T
TCGA-24-1843	ZNF479	ENST00000331162	p.Q276H	c.828A>T	Unverified	GGC	CAA	GCC	3	A	T
TCGA-29-1763	ZNF518A	ENST00000371192	p.R228*	c.682A>T	Unverified	CAT	AGA	CAT	1	A	T
TCGA-29-1763	ZNF518A_EI	ENST00000371192_v61	p.R228*	c.682A>T	Unverified	CAT	AGA	CAT	1	A	T
TCGA-61-2095	ZNF546	ENST00000347077	p.N595I	c.1784A>T	Unverified	TAT	AAT	CTT	2	A	T
TCGA-09-2044	ZNF765_ENS	ENST00000396408	p.N508Y	c.1522A>T	Verified	TCA	AAC	CTT	1	A	T
TCGA-24-1847	ZSCAN5B	ENST00000358992	p.T31S	c.91A>T	Unverified	GAA	ACT	CAA	1	A	T
TCGA-24-1847	ZSCAN5B_EI	ENST00000358992	p.T31S	c.91A>T	Unverified	GAA	ACT	CAA	1	A	T
TCGA-24-1847	A2M	ENST00000318602	p.L835I	c.2503C>A	Unverified	TTC	CTA	GCT	1	C	A
TCGA-29-1764	ABCA13_EN	ENST00000435803	p.P389H	c.1166C>A	Unverified	AAG	CCC	TGG	2	C	A
TCGA-13-0913	ABCA4	ENST00000370225	p.A781D	c.2342C>A	Verified	TTC	GCC	TGG	2	C	A
TCGA-61-1907	ABCA4	ENST00000370225	p.P1512P	c.4536C>A	Unverified	CCC	CCC	CAG	3	C	A
TCGA-25-1326	ABCA5	ENST00000392676	p.H994N	c.2980C>A	Verified	TAT	CAT	TTA	1	C	A
TCGA-24-1104	ABCA6	ENST00000284425	p.S1541Y	c.4622C>A	Verified	TAT	TCC	TCT	2	C	A
TCGA-09-1665	ABCG4	ENST00000307417	p.T442N	c.1325C>A	Verified	CCA	ACT	GTG	2	C	A
TCGA-13-1497	ABI3BP	ENST00000284322	p.S500R	c.1500C>A	Verified	AAA	AGC	CCT	3	C	A
TCGA-24-1847	ABLIM3	ENST00000309868	p.N363K	c.1089C>A	Unverified	GAG	AAC	CTG	3	C	A
TCGA-24-2290	ABLIM3	ENST00000309868	p.S547R	c.1641C>A	Verified	CGG	AGC	TCC	3	C	A
TCGA-24-2290	ABLIM3	ENST00000309868	p.S547R	c.1641C>A	Verified	CGG	AGC	TCC	3	C	A
TCGA-23-1021	ABP1	ENST00000360937	p.T215N	c.644C>A	Verified	CCC	ACT	GGG	2	C	A
TCGA-23-1022	ABRA	ENST00000311955	p.P63T	c.187C>A	Verified	GCT	CCT	AAA	1	C	A
TCGA-29-1691	AC013469_8	ENST00000408934	p.L92I	c.274C>A	Unverified	CCT	CTT	CAC	1	C	A

TCGA-13-0916	ACCN1	ENST00000225823	p.P383T	c.1147C>A	Verified	GCC	CCT	TTT	1	C	A
TCGA-04-1356	ACCSL	ENST00000378832	p.I335I	c.1005C>A	Verified	GAC	ATC	TAC	3	C	A
TCGA-29-1764	ACSM2A	ENST00000396104	p.L550M	c.1648C>A	Unverified	AAC	CTG	CCC	1	C	A
TCGA-24-1470	ACTL8	ENST00000375406	p.L287M	c.859C>A	Verified	TCC	CTG	CGC	1	C	A
TCGA-29-1761	ACTN2	ENST00000366578	p.P85H	c.254C>A	Unverified	CTG	CCC	AAA	2	C	A
TCGA-04-1331	ACTN4	ENST00000252699	p.V287V	c.861C>A	Unverified	GCT	GTC	AAC	3	C	A
TCGA-24-2288	ACTR6	ENST00000188312	p.P59H	c.176C>A	Unverified	CTC	CCT	TTT	2	C	A
TCGA-29-1784	ACTRT1	ENST00000371124	p.T368K	c.1103C>A	Unverified	ggg	aca	tct	2	C	A
TCGA-23-1029	ADAD1	ENST00000296513	p.L65I	c.193C>A	Unverified	TTG	CTT	TCC	1	C	A
TCGA-29-1705	ADAM12	ENST00000368679	p.P835P	c.2505C>A	Unverified	AGA	CCC	CTG	3	C	A
TCGA-29-1705	ADAM12_EN	ENST00000368679	p.P835P	c.2505C>A	Unverified	AGA	CCC	CTG	3	C	A
TCGA-04-1367	ADAM19	NM_023038.2	p.H350Q	c.1050C>A	Verified	ggc	cac	aac	3	C	A
TCGA-29-1761	ADAM28	ENST00000265769	p.P724P	c.2172C>A	Unverified	CAA	CCC	CAA	3	C	A
TCGA-29-1702	ADAM29	ENST00000359240	p.Q803K	c.2407C>A	Unverified	TCC	CAG	AGG	1	C	A
TCGA-29-1695	ADAMTS18	ENST00000282849	p.C748*	c.2244C>A	Unverified	act	tgc	aag	3	C	A
TCGA-13-0883	ADAMTSL1_	ENST00000380548	p.Q998K	c.2992C>A	Verified	CAC	CAG	AAC	1	C	A
TCGA-23-1022	ADCY3	ENST00000260600	p.Y730*	c.2190C>A	Verified	CAG	TAC	TAC	3	C	A
TCGA-13-0906	ADCY4	ENST00000310677	p.P991P	c.2973C>A	Verified	GGA	CCC	GTA	3	C	A
TCGA-25-2042	ADM	ENST00000278175	p.S49S	c.147C>A	Unverified	ATG	TCC	AGC	3	C	A
TCGA-13-1498	ADSS	ENST00000366535	p.G165G	c.495C>A	Verified	aag	ggc	att	3	C	A
TCGA-24-2288	AFAP1L1	ENST00000296721	p.T415T	c.1245C>A	Unverified	TCC	ACC	GAG	3	C	A
TCGA-09-1674	AGAP3	ENST00000397238	p.S829Y	c.2486C>A	Unverified	CTC	TCC	AGT	2	C	A
TCGA-23-2077	AGXT	ENST00000307503	p.D341E	c.1023C>A	Unverified	ATA	GAC	CAC	3	C	A
TCGA-29-1766	AHCYL1	ENST00000369799	p.L517I	c.1549C>A	Unverified	GGA	CTC	AAC	1	C	A
TCGA-04-1336	AHNAK2_EN	ENST00000333244	p.D3355E	c.10065C>A	Verified	ACT	GAC	CTC	3	C	A
TCGA-04-1361	AIFM1	ENST00000319908	p.P484P	c.1452C>A	Verified	ggc	ccc	gat	3	C	A
TCGA-04-1361	AIFM1	ENST00000319908	p.P484P	c.1452C>A	Verified	ggc	ccc	gat	3	C	A
TCGA-04-1356	AIM2	ENST00000368130	p.Q137K	c.409C>A	Verified	AAA	CAG	ATG	1	C	A
TCGA-61-1740	AIM2	ENST00000368130	p.L317M	c.949C>A	Unverified	ACA	CTG	TCA	1	C	A
TCGA-13-0920	ALB	ENST00000295897	p.A234D	c.701C>A	Verified	AGA	GCT	TTC	2	C	A
TCGA-29-1761	ALDH1L1	ENST00000393434	p.N227K	c.681C>A	Unverified	GGG	AAC	GAC	3	C	A
TCGA-23-2077	ALG10B	ENST00000308742	p.S14R	c.42C>A	Verified	TTG	AGC	TGT	3	C	A
TCGA-24-2267	ALK	ENST00000389048	p.F64L	c.192C>A	Unverified	ctc	ttc	cgt	3	C	A
TCGA-61-1740	ALKBH8	ENST00000260318	p.H173Q	c.519C>A	Unverified	AAA	CAC	AGA	3	C	A

TCGA-61-1740	ALKBH8_EN:	ENST00000428149	p.H173Q	c.519C>A	Unverified	AAA	CAC	AGA	3	C	A
TCGA-61-1740	ALKBH8_EN:	ENST00000428149	p.H173Q	c.519C>A	Unverified	AAA	CAC	AGA	3	C	A
TCGA-13-0884	ALMS1	ENST00000264448	p.A2623A	c.7869C>A	Verified	AGA	GCC	AAG	3	C	A
TCGA-13-0906	ALOXE3	NM_021628.1	p.F45L	c.135C>A	Unverified	gac	ttc	gcc	3	C	A
TCGA-13-0755	ALS2CR11	ENST00000286195	p.P289T	c.865C>A	Verified	TAT	CCA	GCA	1	C	A
TCGA-25-1313	AMOTL2	ENST00000249883	p.P74H	c.221C>A	Unverified	GAG	CCC	CAG	2	C	A
TCGA-04-1338	AMPD2	ENST00000342115	p.D125E	c.375C>A	Verified	ggt	gac	cgg	3	C	A
TCGA-04-1338	AMPD2_EN:	ENST00000393689	p.D206E	c.618C>A	Verified	GGT	GAC	CGG	3	C	A
TCGA-61-2094	ANGPT1	ENST00000297450	p.T170T	c.510C>A	Unverified	TCC	ACC	TAC	3	C	A
TCGA-29-1702	ANK1	ENST00000347528	p.S594S	c.1782C>A	Unverified	ggc	tcc	ccg	3	C	A
TCGA-29-1702	ANK1_ENST(	ENST00000265709	p.S627S	c.1881C>A	Unverified	GGC	TCC	CCG	3	C	A
TCGA-61-2095	ANKRD2	ENST00000307518	p.H333N	c.997C>A	Unverified	GAG	CAT	CCT	1	C	A
TCGA-09-2049	ANKRD24_E	ENST00000262970	p.G195G	c.585C>A	Verified	CAT	GGC	AGC	3	C	A
TCGA-61-2109	ANKRD55	ENST00000341048	p.N566K	c.1698C>A	Unverified	AAC	AAC	CTA	3	C	A
TCGA-29-1776	ANKS1B	ENST00000547776	p.P746T	c.2236C>A	Unverified	TAT	CCT	TCC	1	C	A
TCGA-20-1687	ANO5	ENST00000324559	p.F385L	c.1155C>A	Unverified	GTG	TTC	TTT	3	C	A
TCGA-29-1781	ANXA2	ENST00000332680	p.S135S	c.405C>A	Unverified	GCT	TCC	ATG	3	C	A
TCGA-23-1123	AP3B2	ENST00000261722	p.I903I	c.2709C>A	Verified	CCC	ATC	AAG	3	C	A
TCGA-23-1123	AP3B2	ENST00000261722	p.I903I	c.2709C>A	Verified	CCC	ATC	AAG	3	C	A
TCGA-24-2267	APOB	ENST00000233242	p.Q3088K	c.9262C>A	Verified	TGG	CAA	GTA	1	C	A
TCGA-24-2288	APOB	ENST00000233242	p.S1653Y	c.4958C>A	Unverified	ATA	TCT	ACC	2	C	A
TCGA-29-1781	APOB	ENST00000233242	p.G230G	c.690C>A	Unverified	AAA	GGC	ATG	3	C	A
TCGA-24-1604	APOL3_ENS'	ENST00000349314	p.S44Y	c.131C>A	Verified	GTG	TCT	GGT	2	C	A
TCGA-29-1777	AQP6	ENST00000315520	p.P83T	c.247C>A	Unverified	AAC	CCC	GCC	1	C	A
TCGA-23-1114	ARHGAP20	ENST00000260283	p.P1172T	c.3514C>A	Unverified	agc	cca	gac	1	C	A
TCGA-13-0760	ARHGAP21	NM_020824.2	p.P795Q	c.2384C>A	Verified	agt	ccg	cct	2	C	A
TCGA-13-0903	ARHGAP36	NM_144967.2	p.L472I	c.1414C>A	Verified	cta	ctt	tct	1	C	A
TCGA-20-1687	ARHGAP36	NM_144967.2	p.S510Y	c.1529C>A	Unverified	cgt	tcc	cat	2	C	A
TCGA-61-1740	ARHGEF10	NM_014629.1	p.A849D	c.2546C>A	Unverified	cat	gct	gta	2	C	A
TCGA-61-1740	ARHGEF10_	ENST00000349830	p.A1072D	c.3215C>A	Unverified	CAT	GCT	GTA	2	C	A
TCGA-20-1683	ARHGEF15	ENST00000361926	p.A74D	c.221C>A	Unverified	cct	gct	ctt	2	C	A
TCGA-13-0904	ARHGEF4	ENST00000326016	p.S160R	c.480C>A	Verified	ggg	agc	gag	3	C	A
TCGA-29-1763	ARID2	ENST00000334344	p.R1273R	c.3817C>A	Unverified	CGA	CGA	GGA	1	C	A
TCGA-23-1111	ARID3B	ENST00000346246	p.S381Y	c.1142C>A	Unverified	ATA	TCC	CCA	2	C	A

TCGA-13-0893	ARPC3	ENST00000228825	p.T36K	c.107C>A	Verified	GAG	ACA	AAA	2	C	A
TCGA-24-1844	ARR3	ENST00000307959	p.Q195K	c.583C>A	Unverified	CTA	CAA	CTC	1	C	A
TCGA-13-1408	ASB11	ENST00000480796	p.A264E	c.791C>A	Unverified	agt	gcg	ctt	2	C	A
TCGA-23-1022	ASNSD1	ENST00000260952	p.P243H	c.728C>A	Verified	GTT	CCT	TTA	2	C	A
TCGA-24-1843	ASTE1	ENST00000264992	p.F63L	c.189C>A	Unverified	AAA	TTC	TTT	3	C	A
TCGA-23-1031	ASTN1	ENST00000361833	p.D291E	c.873C>A	Verified	agt	gac	aat	3	C	A
TCGA-13-0883	ATF7IP	ENST00000261168	p.T155T	c.465C>A	Verified	tcc	acc	tct	3	C	A
TCGA-24-1423	ATP13A1	ENST00000357324	p.A500D	c.1499C>A	Verified	CTG	GCC	GTC	2	C	A
TCGA-13-0791	ATP9B	ENST00000307671	p.T472T	c.1416C>A	Verified	gga	acc	ctc	3	C	A
TCGA-23-1120	AVIL	ENST00000257861	p.L425M	c.1273C>A	Unverified	TAT	CTG	GTC	1	C	A
TCGA-13-0885	AVPR1B	ENST00000367126	p.A188E	c.563C>A	Verified	TGG	GCA	GAC	2	C	A
TCGA-29-1781	AXL	ENST00000359092	p.V178V	c.534C>A	Unverified	GCT	GTC	CCC	3	C	A
TCGA-24-0975	B3GALT1	ENST00000392690	p.P124H	c.371C>A	Verified	GAT	CCT	GTT	2	C	A
TCGA-13-0755	B4GALNT2	ENST00000300404	p.A533D	c.1598C>A	Verified	GCT	GCC	CTA	2	C	A
TCGA-09-2044	B4GALT5	ENST00000371711	p.T113N	c.338C>A	Verified	CAT	ACC	TGC	2	C	A
TCGA-23-1022	B9D2	ENST00000243578	p.S37*	c.110C>A	Verified	CTG	TCA	GGC	2	C	A
TCGA-13-1501	BAI2	ENST00000373658	p.L178I	c.532C>A	Verified	GCC	CTA	GCC	1	C	A
TCGA-13-1501	BAI2	ENST00000373658	p.L178I	c.532C>A	Verified	GCC	CTA	GCC	1	C	A
TCGA-13-1408	BAI2	ENST00000373658	p.T997N	c.2990C>A	Unverified	TGC	ACC	ATG	2	C	A
TCGA-24-1470	BAI2	ENST00000373658	p.T149N	c.446C>A	Verified	TTT	ACC	TTC	2	C	A
TCGA-23-1110	BAI3	ENST00000370598	p.Q1358K	c.4072C>A	Unverified	GAC	CAG	TTC	1	C	A
TCGA-24-2280	BAI3	ENST00000370598	p.Q142K	c.424C>A	Unverified	TTA	CAG	AAA	1	C	A
TCGA-30-1718	BAI3	ENST00000370598	p.G473G	c.1419C>A	Unverified	GAT	GGC	GGC	3	C	A
TCGA-04-1338	BAZ2B	ENST00000392783	p.S429S	c.1287C>A	Verified	AGA	TCC	AAA	3	C	A
TCGA-61-2113	BCAN	ENST00000329117	p.R896S	c.2686C>A	Unverified	GGA	CGC	TGG	1	C	A
TCGA-23-1029	BCAS1	ENST00000395961	p.P8P	c.24C>A	Unverified	GTT	CCC	CAA	3	C	A
TCGA-13-0887	BCAT2	ENST00000316273	p.P82H	c.245C>A	Verified	CAG	CCC	TTC	2	C	A
TCGA-09-2050	BCL3	ENST00000164227	p.P444Q	c.1331C>A	Unverified	CGG	CCG	GTG	2	C	A
TCGA-04-1655	BCL9L	NM_182557.1	p.A274A	c.822C>A	Unverified	cgg	gcc	aag	3	C	A
TCGA-04-1655	BCL9L_ENST	ENST00000392849	p.A274A	c.822C>A	Unverified	CGG	GCC	AAG	3	C	A
TCGA-09-2044	BDKRB1	ENST00000216629	p.H199N	c.595C>A	Verified	TGG	CAC	TTT	1	C	A
TCGA-24-1845	BEND2	ENST00000380033	p.N21K	c.63C>A	Unverified	GAT	AAC	AAT	3	C	A
TCGA-24-1844	BHMT	ENST00000274353	p.T135N	c.404C>A	Unverified	GAA	ACT	GAA	2	C	A
TCGA-04-1542	BICC1	ENST00000373886	p.P226H	c.677C>A	Verified	TCC	CCC	TCT	2	C	A

TCGA-13-1498	BMP15	ENST00000252677	p.L293I	c.877C>A	Verified	TCC	CTC	CAC	1	C	A
TCGA-24-1417	BMPER	ENST00000297161	p.V680V	c.2040C>A	Unverified	CCA	GTC	CTT	3	C	A
TCGA-23-1123	BMPR2	ENST00000374580	p.P283T	c.847C>A	Verified	tat	ccc	aat	1	C	A
TCGA-23-1123	BMPR2	ENST00000374580	p.P283T	c.847C>A	Verified	tat	ccc	aat	1	C	A
TCGA-30-1857	BNC1	ENST00000345382	p.S847Y	c.2540C>A	Unverified	AAC	TCT	GGC	2	C	A
TCGA-04-1338	BNC1	ENST00000345382	p.N393K	c.1179C>A	Unverified	TGT	AAC	ATG	3	C	A
TCGA-13-0906	BOC	ENST00000355385	p.P823T	c.2467C>A	Verified	CCA	CCC	CCA	1	C	A
TCGA-04-1337	BOC	ENST00000355385	p.T721K	c.2162C>A	Unverified	TTC	ACG	GAT	2	C	A
TCGA-09-2050	BRCA2	ENST00000380152	p.S1882*	c.5645C>A	Verified	AAA	TCA	AAA	2	C	A
TCGA-04-1331	BRCA2	ENST00000380152	p.C711*	c.2133C>A	Verified	TCA	TGC	CTG	3	C	A
TCGA-23-1021	BRF1	ENST00000546474	p.A549A	c.1647C>A	Verified	AGC	GCC	GGC	3	C	A
TCGA-10-0930	BRI3BP	ENST00000341446	p.L82M	c.244C>A	Verified	GTG	CTG	GGA	1	C	A
TCGA-10-0930	BRI3BP	ENST00000341446	p.L82M	c.244C>A	Verified	GTG	CTG	GGA	1	C	A
TCGA-23-1022	BRSK1	ENST00000309383	p.P194P	c.582C>A	Verified	TCC	CCC	CAT	3	C	A
TCGA-24-1103	BSN	ENST00000296452	p.H899Q	c.2697C>A	Verified	CCC	CAC	AAT	3	C	A
TCGA-13-0792	BTBD3	ENST00000378226	p.A303E	c.908C>A	Unverified	CTG	GCG	TTG	2	C	A
TCGA-24-1846	BTF3L4	ENST00000313334	p.L145I	c.433C>A	Unverified	GAT	CTT	GTA	1	C	A
TCGA-24-1849	BTNL9	ENST00000327705	p.S223R	c.669C>A	Unverified	CTC	AGC	AAT	3	C	A
TCGA-13-0793	C10orf12	ENST00000286067	p.S526*	c.1577C>A	Unverified	AGT	TCA	GAT	2	C	A
TCGA-24-2262	C11orf40	ENST00000307616	p.C99*	c.297C>A	Verified	CCT	TGC	TTT	3	C	A
TCGA-04-1347	C11orf42	ENST00000316375	p.P271H	c.812C>A	Verified	AAA	CCC	ACC	2	C	A
TCGA-04-1655	C13orf33	ENST00000380482	p.S191R	c.573C>A	Unverified	TTA	AGC	TTT	3	C	A
TCGA-20-1685	C15orf2	ENST00000329468	p.P307H	c.920C>A	Unverified	AAG	CCT	TTC	2	C	A
TCGA-24-2280	C15orf2	ENST00000329468	p.P623Q	c.1868C>A	Unverified	TCC	CCA	CTT	2	C	A
TCGA-24-2280	C15orf63	ENST00000406925	p.R34R	c.100C>A	Unverified	CCA	CGG	AAA	1	C	A
TCGA-20-1687	C16orf71	ENST00000299320	p.S60S	c.180C>A	Unverified	ACC	TCC	CTG	3	C	A
TCGA-13-0762	C17orf49	ENST00000439424	p.P129H	c.386C>A	Unverified	GGT	CCC	CCC	2	C	A
TCGA-13-0762	C17orf49	ENST00000439424	p.P129H	c.386C>A	Unverified	GGT	CCC	CCC	2	C	A
TCGA-13-1510	C17orf63	ENST00000452648	p.P250P	c.750C>A	Unverified	ATC	CCC	CTT	3	C	A
TCGA-24-1845	C19orf46	ENST00000324444	p.T245K	c.734C>A	Unverified	TCC	ACA	GAG	2	C	A
TCGA-24-2289	C19orf55	ENST00000396908	p.T32N	c.95C>A	Unverified	CAG	ACC	TGG	2	C	A
TCGA-13-0906	C1orf101	ENST00000366533	p.P683T	c.2047C>A	Verified	CGA	CCT	AGT	1	C	A
TCGA-13-1481	C20orf46	ENST00000381894	p.T55T	c.165C>A	Verified	cca	acc	agg	3	C	A
TCGA-23-2078	C21orf33	ENST00000291577	p.H88N	c.262C>A	Verified	ATG	CAC	GTG	1	C	A

TCGA-20-1685	C2CD3	ENST00000313663	p.T1610N	c.4829C>A	Unverified	TCC	ACC	CAG	2	C	A
TCGA-20-1685	C2CD3_ENST	ENST00000334126	p.T1610N	c.4829C>A	Unverified	TCC	ACC	CAG	2	C	A
TCGA-24-1463	C2orf89	ENST00000409520	p.D92E	c.276C>A	Verified	ACA	GAC	CCC	3	C	A
TCGA-13-1488	C3	ENST00000245907	p.T1341N	c.4022C>A	Verified	GGC	ACC	TTG	2	C	A
TCGA-24-1846	C3AR1	ENST00000307637	p.L43M	c.127C>A	Unverified	ggg	ctg	gtg	1	C	A
TCGA-13-0904	C3orf20	ENST00000253697	p.L28L	c.84C>A	Verified	AAA	CTC	CTC	3	C	A
TCGA-29-1701	C3orf79	ENST00000446603	p.L42I	c.124C>A	Unverified	TTG	CTT	TAC	1	C	A
TCGA-04-1367	C4orf50	ENST00000324058	p.Q114K	c.340C>A	Unverified	GCG	CAG	AAC	1	C	A
TCGA-04-1361	C5orf42	ENST00000274258	p.S1059Y	c.3176C>A	Verified	TCA	TCT	CAA	2	C	A
TCGA-04-1361	C5orf42	ENST00000274258	p.S1059Y	c.3176C>A	Verified	TCA	TCT	CAA	2	C	A
TCGA-61-1914	C5orf42	ENST00000274258	p.P417H	c.1250C>A	Unverified	CTT	CCT	GTA	2	C	A
TCGA-61-1914	C5orf42_ENS	ENST00000425232	p.P1536H	c.4607C>A	Unverified	CTT	CCT	GTA	2	C	A
TCGA-13-0760	C7orf52	ENST00000300303	p.A38D	c.113C>A	Verified	GAG	GCC	GAG	2	C	A
TCGA-13-0916	C7orf52	ENST00000300303	p.P285H	c.854C>A	Unverified	TTC	CCC	ATC	2	C	A
TCGA-23-1122	C7orf70	ENST00000313324	p.P150H	c.449C>A	Unverified	GAG	CCT	CGG	2	C	A
TCGA-23-1114	C8orf47	ENST00000318528	p.T21N	c.62C>A	Unverified	GTA	ACT	TCA	2	C	A
TCGA-24-2035	C9orf171	ENST00000343036	p.P266T	c.796C>A	Verified	CCG	CCC	GTG	1	C	A
TCGA-29-1691	C9orf30	ENST00000395067	p.Q209K	c.625C>A	Unverified	TTA	CAA	CTG	1	C	A
TCGA-13-0887	C9orf79	ENST00000325643	p.G317G	c.951C>A	Verified	TGG	GGC	CTC	3	C	A
TCGA-24-1847	CABS1	ENST00000273936	p.T227N	c.680C>A	Unverified	ACT	ACT	ATT	2	C	A
TCGA-13-0760	CACNA1C	ENST00000402845	p.A101E	c.302C>A	Verified	AAT	GCG	ACC	2	C	A
TCGA-13-1509	CACNA1C	ENST00000402845	p.A1843D	c.5528C>A	Verified	AAC	GCC	AAC	2	C	A
TCGA-04-1337	CACNG3	ENST00000005284	p.Y89*	c.267C>A	Unverified	GAC	TAC	GAA	3	C	A
TCGA-29-2427	CACNG6	ENST00000252729	p.C114*	c.342C>A	Verified	AAC	TGC	ACC	3	C	A
TCGA-13-0900	CADM3	ENST00000368124	p.H387Q	c.1161C>A	Verified	GGC	CAC	TAC	3	C	A
TCGA-04-1342	CAMK4	ENST00000512453	p.A329E	c.986C>A	Verified	gca	gcg	gtg	2	C	A
TCGA-24-1845	CAMTA1	ENST00000303635	p.L196I	c.586C>A	Unverified	ATC	CTC	TGC	1	C	A
TCGA-23-1029	CAPN13	ENST00000295055	p.H206Q	c.618C>A	Unverified	CTG	CAC	TCT	3	C	A
TCGA-29-1781	CAPN3	ENST00000357568	p.N770K	c.2310C>A	Unverified	ATG	AAC	ATC	3	C	A
TCGA-29-1781	CAPN3_ENST	ENST00000356316	p.N683K	c.2049C>A	Unverified	ATG	AAC	ATC	3	C	A
TCGA-24-1849	CARD14	ENST00000573882	p.L331M	c.991C>A	Unverified	CTG	CTG	CAG	1	C	A
TCGA-13-0885	CARD14	ENST00000573882	p.S463S	c.1389C>A	Verified	ACG	TCC	AGC	3	C	A
TCGA-24-1849	CARD14_EN	ENST00000309710	p.L94M	c.280C>A	Unverified	CTG	CTG	CAG	1	C	A
TCGA-04-1356	CARD18	ENST00000530950	p.T21K	c.62C>A	Verified	GGC	ACA	ATA	2	C	A

TCGA-61-1740	CARD6	ENST00000254691	p.P768H	c.2303C>A	Unverified	CAT	CCT	TTG	2	C	A
TCGA-61-1740	CASK	ENST00000378166	p.F91L	c.273C>A	Unverified	GTT	TTC	GAA	3	C	A
TCGA-29-1762	CASQ2	ENST00000261448	p.R121S	c.361C>A	Unverified	GAT	CGC	ACA	1	C	A
TCGA-24-1849	CATSPER1	ENST00000312106	p.P100T	c.298C>A	Unverified	GCT	CCC	TCT	1	C	A
TCGA-13-0904	CCBE1	ENST00000439986	p.P263T	c.787C>A	Verified	GGA	CCA	AAG	1	C	A
TCGA-04-1331	CCDC102A	ENST00000258214	p.T245K	c.734C>A	Verified	GCC	ACG	GAG	2	C	A
TCGA-24-2024	CCDC135	ENST00000360716	p.Q743K	c.2227C>A	Unverified	CGG	CAG	GTG	1	C	A
TCGA-25-2042	CCDC141	ENST00000343876	p.S271S	c.813C>A	Verified	CTT	TCC	TTA	3	C	A
TCGA-23-1022	CCDC165	ENST00000359865	p.P542H	c.1625C>A	Verified	TCC	CCC	TTG	2	C	A
TCGA-61-2094	CCDC17	ENST00000343901	p.S200Y	c.599C>A	Unverified	TAT	TCT	CCA	2	C	A
TCGA-23-1118	CCDC60	ENST00000327554	p.D201E	c.603C>A	Verified	AAG	GAC	AAG	3	C	A
TCGA-04-1343	CCDC61	ENST00000263284	p.R155S	c.463C>A	Verified	AAC	CGC	AAG	1	C	A
TCGA-23-1029	CCNA1	ENST00000255465	p.P73T	c.217C>A	Unverified	gcc	ccg	ctg	1	C	A
TCGA-24-1604	CCNL2	ENST00000400809	p.R495R	c.1483C>A	Verified	CGA	CGA	GAG	1	C	A
TCGA-04-1651	CCR3	ENST00000357422	p.P254H	c.761C>A	Unverified	aca	ccc	tac	2	C	A
TCGA-04-1651	CCR3	ENST00000357422	p.P254H	c.761C>A	Unverified	aca	ccc	tac	2	C	A
TCGA-29-1775	CCT8L1	ENST00000021776	p.T305K	c.914C>A	Unverified	CTC	ACA	CTG	2	C	A
TCGA-29-1777	CCT8L2	ENST00000359963	p.A44D	c.131C>A	Unverified	CTG	GCC	AGT	2	C	A
TCGA-13-0923	CD163L1	ENST00000313599	p.A764D	c.2291C>A	Verified	GAA	GCC	TCT	2	C	A
TCGA-09-2049	CD5	ENST00000347785	p.A306D	c.917C>A	Verified	TCA	GCC	AGG	2	C	A
TCGA-61-1914	CD5	ENST00000347785	p.R292R	c.876C>A	Unverified	GTG	CGC	CAG	3	C	A
TCGA-29-1761	CD93	ENST00000246006	p.Q559K	c.1675C>A	Unverified	CCC	CAG	GAG	1	C	A
TCGA-29-1781	CDH12	ENST00000382254	p.D696E	c.2088C>A	Unverified	CCA	GAC	TCT	3	C	A
TCGA-13-0762	CDH17	ENST00000027335	p.P701T	c.2101C>A	Verified	GGT	CCC	CAT	1	C	A
TCGA-13-0762	CDH17	ENST00000027335	p.P701T	c.2101C>A	Verified	GGT	CCC	CAT	1	C	A
TCGA-61-1900	CDH20	ENST00000262717	p.P77T	c.229C>A	Unverified	GAC	CCT	TTG	1	C	A
TCGA-13-0920	CDH8	ENST00000577390	p.P15T	c.43C>A	Verified	ACT	CCA	TTA	1	C	A
TCGA-23-1032	CDKL5	ENST00000379996	p.T990T	c.2970C>A	Verified	cca	acc	cag	3	C	A
TCGA-23-1124	CEACAM21	ENST00000401445	p.L164M	c.490C>A	Verified	GTC	CTG	ACC	1	C	A
TCGA-61-1733	CEL	ENST00000372080	p.Q749K	c.2245C>A	Unverified	GCT	CAG	ATG	1	C	A
TCGA-61-1733	CEL	ENST00000372080	p.Q749K	c.2245C>A	Unverified	GCT	CAG	ATG	1	C	A
TCGA-29-1763	CELSR1	ENST00000262738	p.F2518L	c.7554C>A	Unverified	GTG	TTC	GTG	3	C	A
TCGA-61-1738	CELSR1	ENST00000262738	p.F2518L	c.7554C>A	Unverified	GTG	TTC	GTG	3	C	A
TCGA-61-1738	CELSR1	ENST00000262738	p.F2518L	c.7554C>A	Unverified	GTG	TTC	GTG	3	C	A

TCGA-25-1313	CELSR2	ENST00000271332	p.P136Q	c.407C>A	Unverified	CAC	CCG	TGC	2	C	A
TCGA-04-1342	CENPJ	ENST00000381884	p.T515K	c.1544C>A	Unverified	TGC	ACA	GGG	2	C	A
TCGA-25-1326	CENPP	ENST00000375587	p.H62N	c.184C>A	Verified	GGA	CAT	TTA	1	C	A
TCGA-24-2288	CEP128	ENST00000281129	p.D392E	c.1176C>A	Unverified	AAA	GAC	AAG	3	C	A
TCGA-61-1907	CEP164	ENST00000278935	p.V1278V	c.3834C>A	Unverified	AGT	GTC	CTC	3	C	A
TCGA-13-1498	CEP57	ENST00000325542	p.Q428K	c.1282C>A	Verified	AAA	CAG	AAG	1	C	A
TCGA-25-1326	CEP72	ENST00000264935	p.C437*	c.1311C>A	Unverified	GGC	TGC	AGG	3	C	A
TCGA-20-1685	CFHR5	ENST00000256785	p.S454Y	c.1361C>A	Unverified	CCA	TCT	ATT	2	C	A
TCGA-25-1326	CGN	ENST00000271636	p.L475I	c.1423C>A	Unverified	GAA	CTT	CTG	1	C	A
TCGA-61-1900	CGN	ENST00000271636	p.D1015E	c.3045C>A	Unverified	CAG	GAC	CTG	3	C	A
TCGA-23-1116	CHKB	ENST00000406938	p.R151R	c.451C>A	Unverified	AGT	CGG	CCA	1	C	A
TCGA-29-2427	CHPF2	ENST00000035307	p.Q279K	c.835C>A	Verified	CAG	CAG	TAT	1	C	A
TCGA-09-2050	CHRAC1	ENST00000220913	p.I27I	c.81C>A	Verified	GTC	ATC	ATG	3	C	A
TCGA-29-1770	CHRD	ENST00000204604	p.S919Y	c.2756C>A	Unverified	CTG	TCC	TGT	2	C	A
TCGA-13-1507	CHST10	ENST00000264249	p.C92*	c.276C>A	Verified	GTC	TGC	AGG	3	C	A
TCGA-10-0930	CIDEA	ENST00000342845	p.P150T	c.448C>A	Verified	GTC	CCC	ACT	1	C	A
TCGA-10-0930	CIDEA	ENST00000342845	p.P150T	c.448C>A	Verified	GTC	CCC	ACT	1	C	A
TCGA-13-0792	CIITA	ENST00000324288	p.A691D	c.2072C>A	Unverified	ATG	GCC	AAA	2	C	A
TCGA-13-1510	CILP2	ENST00000291495	p.P1107T	c.3319C>A	Unverified	GAG	CCA	CCG	1	C	A
TCGA-13-0793	CLCN4	ENST00000380833	p.G182G	c.546C>A	Verified	tct	ggc	ata	3	C	A
TCGA-29-1783	CLCN5	ENST00000376108	p.L528M	c.1582C>A	Unverified	gaa	ctg	act	1	C	A
TCGA-29-1783	CLCN5_ENST	ENST00000376088	p.L598M	c.1792C>A	Unverified	GAA	CTG	ACT	1	C	A
TCGA-23-1122	CLDN16	ENST00000264734	p.A241D	c.722C>A	Verified	atg	gct	ggg	2	C	A
TCGA-25-2401	CLDN18_EN:	ENST00000343735	p.A91D	c.272C>A	Verified	GGT	GCC	ATT	2	C	A
TCGA-20-0990	CLDN2	ENST00000541806	p.T56K	c.167C>A	Verified	gcc	aca	cac	2	C	A
TCGA-13-0760	CLEC4F	ENST00000272367	p.H93Q	c.279C>A	Verified	CAC	CAC	TTT	3	C	A
TCGA-29-1696	CLEC5A	ENST00000546910	p.Q49K	c.145C>A	Unverified	TCA	CAG	ATT	1	C	A
TCGA-29-1696	CLEC5A	ENST00000546910	p.Q49K	c.145C>A	Unverified	TCA	CAG	ATT	1	C	A
TCGA-61-2094	CLK1	ENST00000321356	p.H233N	c.697C>A	Unverified	GAG	CAT	CAT	1	C	A
TCGA-61-1733	CLRN1	ENST00000327047	p.L230I	c.688C>A	Unverified	GAT	CTA	ATG	1	C	A
TCGA-61-1733	CLRN1	ENST00000327047	p.L230I	c.688C>A	Unverified	GAT	CTA	ATG	1	C	A
TCGA-13-0761	CLUL1	ENST00000338387	p.P428T	c.1282C>A	Verified	CTG	CCT	TCC	1	C	A
TCGA-13-0761	CLUL1	ENST00000338387	p.P428T	c.1282C>A	Verified	CTG	CCT	TCC	1	C	A
TCGA-20-1685	CNGA1	ENST00000358519	p.L461I	c.1381C>A	Unverified	AAA	CTA	AGA	1	C	A

TCGA-61-1914	CNGA2	ENST00000329903	p.A625D	c.1874C>A	Unverified	ggg	gcc	cag	2	C	A
TCGA-23-2078	CNIH4	ENST00000465271	p.N92K	c.276C>A	Verified	GGT	AAC	ATG	3	C	A
TCGA-13-0893	CNKSR3	ENST00000367213	p.S296Y	c.887C>A	Verified	GGG	TCT	TTC	2	C	A
TCGA-24-2035	CNTFR	ENST00000351266	p.R167R	c.501C>A	Verified	ATT	CGC	TAC	3	C	A
TCGA-23-1118	CNTN1	ENST00000551295	p.P892Q	c.2675C>A	Verified	cct	cca	agt	2	C	A
TCGA-04-1542	CNTNAP2	ENST00000361727	p.Q881K	c.2641C>A	Verified	GAC	CAG	TGG	1	C	A
TCGA-29-1761	CNTNAP2	ENST00000361727	p.R114R	c.340C>A	Unverified	TAC	CGG	ATG	1	C	A
TCGA-13-0906	COBL	ENST00000265136	p.L978M	c.2932C>A	Verified	TCA	CTG	GTT	1	C	A
TCGA-23-1022	COL11A2	ENST00000341947	p.P252Q	c.755C>A	Verified	CAA	CCA	TCA	2	C	A
TCGA-29-1699	COL14A1	ENST00000297848	p.P1347T	c.4039C>A	Unverified	gga	cct	gaa	1	C	A
TCGA-13-0886	COL16A1	ENST00000373672	p.T85N	c.254C>A	Verified	GTG	ACC	CAG	2	C	A
TCGA-09-1674	COL17A1	ENST00000353479	p.P652T	c.1954C>A	Unverified	GAA	CCA	GGT	1	C	A
TCGA-23-2077	COL4A4	ENST00000396625	p.I1680I	c.5040C>A	Verified	AAA	ATC	AGC	3	C	A
TCGA-29-1761	COL5A3	ENST00000264828	p.P706T	c.2116C>A	Unverified	CCT	CCG	GGC	1	C	A
TCGA-24-1845	COL6A6	ENST00000358511	p.P1607H	c.4820C>A	Unverified	GGG	CCT	CCA	2	C	A
TCGA-24-1845	COL6A6_EN:	ENST00000358511_v70	p.P1607H	c.4820C>A	Unverified	GGG	CCT	CCA	2	C	A
TCGA-29-1691	COL9A1	ENST00000357250	p.P29H	c.86C>A	Unverified	CGC	CCC	AGA	2	C	A
TCGA-29-1761	COPA	ENST00000241704	p.R233R	c.699C>A	Unverified	TGG	CGC	ATG	3	C	A
TCGA-29-1761	COPA_ENST(	ENST00000368069	p.R233R	c.699C>A	Unverified	TGG	CGC	ATG	3	C	A
TCGA-29-1762	COQ6	ENST00000334571	p.H392Q	c.1176C>A	Unverified	CAT	CAC	CTC	3	C	A
TCGA-13-0900	CORO2B	ENST00000566799	p.Y364*	c.1092C>A	Verified	TCC	TAC	CAG	3	C	A
TCGA-13-1501	CPA4	ENST00000222482	p.Q296K	c.886C>A	Verified	ATC	CAA	AAA	1	C	A
TCGA-13-1501	CPA4	ENST00000222482	p.Q296K	c.886C>A	Verified	ATC	CAA	AAA	1	C	A
TCGA-13-0904	CPE	ENST00000402744	p.P299Q	c.896C>A	Verified	CCA	CCA	TGT	2	C	A
TCGA-23-1123	CPEB1	ENST00000398591	p.P35H	c.104C>A	Verified	CGA	CCC	TGG	2	C	A
TCGA-23-1123	CPEB1	ENST00000398591	p.P35H	c.104C>A	Verified	CGA	CCC	TGG	2	C	A
TCGA-20-1683	CPEB4	ENST00000265085	p.P196T	c.586C>A	Unverified	GTC	CCT	GCT	1	C	A
TCGA-13-0714	CPS1	ENST00000233072	p.T1227T	c.3681C>A	Verified	GCT	ACC	CGG	3	C	A
TCGA-09-1674	CR1	ENST00000400960	p.L1685L	c.5055C>A	Unverified	GAC	CTC	AGA	3	C	A
TCGA-13-0920	CR1	ENST00000400960	p.P891P	c.2673C>A	Verified	TTT	CCC	TTT	3	C	A
TCGA-09-1674	CR1_ENST0(	ENST00000367049	p.L2135L	c.6405C>A	Unverified	GAC	CTC	AGA	3	C	A
TCGA-13-0886	CRYBB1	ENST00000215939	p.H149Q	c.447C>A	Verified	GAG	CAC	AAA	3	C	A
TCGA-29-1781	CSMD3	ENST00000297405	p.H2861N	c.8581C>A	Unverified	gat	cac	aat	1	C	A
TCGA-20-1686	CSMD3	ENST00000297405	p.T2293N	c.6878C>A	Unverified	ggc	acc	att	2	C	A

TCGA-29-1784	CSMD3	ENST00000297405	p.P3513H	c.10538C>A	Unverified	caa	ccc	atg	2	C	A
TCGA-24-1104	CSMD3	ENST00000297405	p.G1705G	c.5115C>A	Verified	cca	ggc	aat	3	C	A
TCGA-24-2024	CSMD3	ENST00000297405	p.I3171I	c.9513C>A	Verified	ata	atc	agt	3	C	A
TCGA-29-1781	CSMD3_ENS	ENST00000343508	p.H2821N	c.8461C>A	Unverified	GAT	CAC	AAT	1	C	A
TCGA-20-1686	CSMD3_ENS	ENST00000343508	p.T2253N	c.6758C>A	Unverified	GGC	ACC	ATT	2	C	A
TCGA-29-1784	CSMD3_ENS	ENST00000343508	p.P3473H	c.10418C>A	Unverified	CAA	CCC	ATG	2	C	A
TCGA-09-0369	CSN3	ENST00000304954	p.T60N	c.179C>A	Verified	GGA	ACC	AAT	2	C	A
TCGA-09-0369	CSN3	ENST00000304954	p.T60N	c.179C>A	Verified	GGA	ACC	AAT	2	C	A
TCGA-36-1577	CSRNP1	ENST00000273153	p.S124Y	c.371C>A	Unverified	TTC	TCT	TTG	2	C	A
TCGA-13-0887	CTBP1	ENST00000290921	p.P308H	c.923C>A	Verified	GCA	CCC	AAC	2	C	A
TCGA-23-2077	CTCFL	ENST00000243914	p.D58E	c.174C>A	Verified	cag	gac	agc	3	C	A
TCGA-13-0792	CTNND2	ENST00000304623	p.P689H	c.2066C>A	Unverified	atc	ccc	cac	2	C	A
TCGA-30-1891	CTSC	ENST00000227266	p.Y168*	c.504C>A	Unverified	CTC	TAC	AAG	3	C	A
TCGA-20-1685	CUBN	ENST00000377833	p.N3396K	c.10188C>A	Unverified	TGC	AAC	AGA	3	C	A
TCGA-13-0923	CUX1	ENST00000292535	p.P1055T	c.3163C>A	Unverified	AGC	CCT	GCC	1	C	A
TCGA-61-2095	CUX1	ENST00000292535	p.R1261R	c.3781C>A	Unverified	AAA	CGA	GCG	1	C	A
TCGA-29-1762	CXorf22	NM_152632.1	p.P537H	c.1610C>A	Unverified	ttg	cct	tcg	2	C	A
TCGA-29-1762	CXorf22_EN:	ENST00000297866	p.P537H	c.1610C>A	Unverified	TTG	CCT	TCG	2	C	A
TCGA-24-1474	CYB5D2	ENST00000301391	p.H236N	c.706C>A	Verified	CCA	CAC	AGA	1	C	A
TCGA-13-1510	CYGB	ENST00000293230	p.S75R	c.225C>A	Unverified	CGG	AGC	CCC	3	C	A
TCGA-23-1124	CYP11A1	ENST00000268053	p.H388Q	c.1164C>A	Verified	CTT	CAC	CCC	3	C	A
TCGA-61-2012	CYP11B1	ENST00000292427	p.H109N	c.325C>A	Verified	CCC	CAC	AGG	1	C	A
TCGA-13-0920	CYP1A1	ENST00000395048	p.Q507K	c.1519C>A	Verified	TTC	CAA	ATG	1	C	A
TCGA-29-1781	CYP26B1	ENST00000001146	p.R76R	c.226C>A	Unverified	TCG	CGG	AGG	1	C	A
TCGA-09-2044	CYP2A6	ENST00000301141	p.L219L	c.657C>A	Verified	CAG	CTC	TAT	3	C	A
TCGA-13-0885	CYP2J2	ENST00000371204	p.S490Y	c.1469C>A	Verified	ATT	TCC	CCA	2	C	A
TCGA-24-1463	CYP4A11	ENST00000310638	p.N427K	c.1281C>A	Verified	CCC	AAC	CCA	3	C	A
TCGA-24-1847	CYP4F22	ENST00000269703	p.H93Q	c.279C>A	Unverified	ATG	CAC	CAT	3	C	A
TCGA-04-1652	CYP7B1	ENST00000310193	p.P54H	c.161C>A	Unverified	CTT	CCT	TAT	2	C	A
TCGA-04-1652	CYP7B1	ENST00000310193	p.P54H	c.161C>A	Unverified	CTT	CCT	TAT	2	C	A
TCGA-24-1846	CYTH3	ENST00000350796	p.Q132K	c.394C>A	Unverified	ctt	caa	gcc	1	C	A
TCGA-25-2391	DAB1	ENST00000371233	p.P434H	c.1301C>A	Unverified	CAG	CCC	TCC	2	C	A
TCGA-13-0793	DAXX	ENST00000266000	p.S534*	c.1601C>A	Verified	ctg	tca	gaa	2	C	A
TCGA-10-0930	DCAF5	ENST00000341516	p.P485P	c.1455C>A	Verified	GGG	CCC	CTG	3	C	A

TCGA-10-0930	DCAF5	ENST00000341516	p.P485P	c.1455C>A	Verified	GGG	CCC	CTG	3	C	A
TCGA-29-1781	DCC	ENST00000442544	p.A431D	c.1292C>A	Unverified	TCG	GCT	CCC	2	C	A
TCGA-13-0904	DCHS1	ENST00000299441	p.P1189T	c.3565C>A	Verified	AGC	CCA	CCC	1	C	A
TCGA-13-0924	DCLRE1C	ENST00000378246	p.T102N	c.305C>A	Verified	TTC	ACC	AAC	2	C	A
TCGA-13-0924	DCLRE1C_EN	ENST00000378278	p.T217N	c.650C>A	Verified	TTC	ACC	AAC	2	C	A
TCGA-25-2393	DCLRE1C_EN	ENST00000378278	p.I87I	c.261C>A	Verified	GAA	ATC	GAG	3	C	A
TCGA-25-1313	DCPS	ENST00000263579	p.H279N	c.835C>A	Unverified	GTG	CAC	TTC	1	C	A
TCGA-24-1844	DCTN1	ENST00000361874	p.L866M	c.2596C>A	Unverified	GCT	CTG	GAG	1	C	A
TCGA-61-1895	DCTN2	ENST00000434715	p.L118M	c.352C>A	Unverified	GAG	CTG	ACA	1	C	A
TCGA-61-1895	DCTN2_ENS	ENST00000434715	p.L118M	c.352C>A	Unverified	GAG	CTG	ACA	1	C	A
TCGA-13-0886	DCUN1D3	ENST00000324344	p.Q188K	c.562C>A	Verified	TTT	CAG	TTT	1	C	A
TCGA-24-1563	DDC	ENST00000357936	p.D59E	c.177C>A	Verified	AAC	GAC	GTT	3	C	A
TCGA-24-1469	DDX21	ENST00000354185	p.P192T	c.574C>A	Verified	ttt	ccc	ata	1	C	A
TCGA-04-1655	DDX54	ENST00000306014	p.S530Y	c.1589C>A	Unverified	gag	tcc	atc	2	C	A
TCGA-29-1696	DEF6	ENST00000316637	p.P573T	c.1717C>A	Unverified	CCC	CCT	CTG	1	C	A
TCGA-29-1696	DEF6	ENST00000316637	p.P573T	c.1717C>A	Unverified	CCC	CCT	CTG	1	C	A
TCGA-13-0885	DEFB115	ENST00000400552	p.P3T	c.7C>A	Verified	CTG	CCA	GAT	1	C	A
TCGA-13-0923	DEFB118	ENST00000253381	p.T91K	c.272C>A	Verified	ACG	ACA	GAC	2	C	A
TCGA-09-2049	DEFB129	ENST00000246105	p.T129T	c.387C>A	Verified	GCC	ACC	ATC	3	C	A
TCGA-04-1336	DENND2A	ENST00000275884	p.L762M	c.2284C>A	Verified	TCC	CTG	CTT	1	C	A
TCGA-13-1489	DENND2D	ENST00000357640	p.P73H	c.218C>A	Verified	gag	cct	ata	2	C	A
TCGA-23-2078	DGKD	ENST00000264057	p.H1156Q	c.3468C>A	Unverified	gag	cac	ctc	3	C	A
TCGA-30-1857	DHTKD1	ENST00000263035	p.T917N	c.2750C>A	Unverified	AAG	ACC	TTC	2	C	A
TCGA-29-1777	DHX33	ENST00000225296	p.T430N	c.1289C>A	Unverified	atg	acc	gtg	2	C	A
TCGA-23-1031	DIABLO	ENST00000443649	p.T182N	c.545C>A	Unverified	ata	acc	gcc	2	C	A
TCGA-04-1367	DISP2	ENST00000267889	p.G1074G	c.3222C>A	Verified	GCA	GGC	GTG	3	C	A
TCGA-13-0923	DLG5	ENST00000372391	p.S1508S	c.4524C>A	Verified	AAG	TCC	CAG	3	C	A
TCGA-24-2267	DMD	ENST00000378707	p.L1104I	c.3310C>A	Verified	AAG	CTA	CTG	1	C	A
TCGA-24-2267	DMD_ENST(	ENST00000378677	p.L3560I	c.10678C>A	Verified	AAG	CTA	CTG	1	C	A
TCGA-04-1542	DMD_ENST(	ENST00000378677	p.T633T	c.1899C>A	Verified	GTG	ACC	CAG	3	C	A
TCGA-13-1488	DMRT3	ENST00000190165	p.S382R	c.1146C>A	Verified	TCG	AGC	CCC	3	C	A
TCGA-30-1857	DMRTC2	ENST00000269945	p.P316H	c.947C>A	Unverified	CCC	CCC	AAC	2	C	A
TCGA-29-1693	DMTF1	ENST00000394703	p.P603T	c.1807C>A	Unverified	GAG	CCT	CCA	1	C	A
TCGA-24-2289	DMXL2	ENST00000251076	p.H1982Q	c.5946C>A	Unverified	GAT	CAC	GAC	3	C	A

TCGA-13-1481	DNAAF1	ENST00000378553	p.P366T	c.1096C>A	Verified	CCT	CCC	GGG	1	C	A
TCGA-09-2051	DNAH1_ENS	ENST00000420323	p.L921I	c.2761C>A	Unverified	ATC	CTT	GGG	1	C	A
TCGA-04-1347	DNAH10	ENST00000339192	p.A244D	c.731C>A	Verified	GAA	GCC	AGG	2	C	A
TCGA-13-0760	DNAH10	ENST00000339192	p.H666Q	c.1998C>A	Verified	GTC	CAC	ACC	3	C	A
TCGA-24-2289	DNAH11	ENST00000328843	p.N2211K	c.6633C>A	Unverified	TTA	AAC	CCT	3	C	A
TCGA-29-1711	DNAH17	ENST00000300671	p.N1811K	c.5433C>A	Unverified	GGC	AAC	ACG	3	C	A
TCGA-29-1711	DNAH17	ENST00000300671	p.N1811K	c.5433C>A	Unverified	GGC	AAC	ACG	3	C	A
TCGA-24-2288	DNAH3	ENST00000261383	p.P59T	c.175C>A	Unverified	CTG	CCT	CCT	1	C	A
TCGA-23-1029	DNAH3	ENST00000261383	p.Y531*	c.1593C>A	Unverified	AAA	TAC	ATA	3	C	A
TCGA-24-2288	DNAH3_ENS	ENST00000261383	p.P59T	c.175C>A	Unverified	CTG	CCT	CCT	1	C	A
TCGA-23-1029	DNAH3_ENS	ENST00000261383	p.Y531*	c.1593C>A	Unverified	AAA	TAC	ATA	3	C	A
TCGA-61-1914	DNAH6	ENST00000237449	p.L631I	c.1891C>A	Unverified	AGT	CTT	GAT	1	C	A
TCGA-29-1768	DNAH7	ENST00000312428	p.T1429K	c.4286C>A	Unverified	ATA	ACA	ATG	2	C	A
TCGA-24-2267	DNAH9	ENST00000262442	p.T1700N	c.5099C>A	Unverified	GTA	ACT	GCC	2	C	A
TCGA-61-1904	DNAH9	ENST00000262442	p.F3792L	c.11376C>A	Unverified	GAG	TTC	CTC	3	C	A
TCGA-04-1367	DNAJA4	ENST00000343789	p.D9E	c.27C>A	Unverified	TAT	GAC	ATC	3	C	A
TCGA-25-2392	DNAJB11	ENST00000439351	p.T120N	c.359C>A	Verified	gga	acc	cct	2	C	A
TCGA-23-1110	DNASE2B_EI	ENST00000370665	p.A7E	c.20C>A	Verified	ATG	GCA	AGA	2	C	A
TCGA-61-1914	DNHL1	ENST00000314395	p.L210I	c.628C>A	Unverified	AGT	CTT	GAT	1	C	A
TCGA-04-1338	DNMBP	ENST00000324109	p.T246T	c.738C>A	Verified	GGG	ACC	TAT	3	C	A
TCGA-24-1417	DNTT	ENST00000371174	p.Q247K	c.739C>A	Unverified	TAT	CAA	TCC	1	C	A
TCGA-29-1777	DOCK10	ENST00000258390	p.D1939E	c.5817C>A	Unverified	TTG	GAC	CCC	3	C	A
TCGA-29-1777	DOCK10_EN	ENST00000373702	p.D448E	c.1344C>A	Unverified	TTG	GAC	CCC	3	C	A
TCGA-29-1775	DOCK3	ENST00000266037	p.L1710M	c.5128C>A	Unverified	GAC	CTG	TAC	1	C	A
TCGA-29-1775	DOCK3_ENS	ENST00000266037	p.L1710M	c.5128C>A	Unverified	GAC	CTG	TAC	1	C	A
TCGA-04-1343	DOCK3_ENS	ENST00000266037	p.T1185N	c.3554C>A	Verified	GAG	ACC	GGC	2	C	A
TCGA-30-1857	DOCK4	ENST00000342288	p.H1409N	c.4225C>A	Unverified	GTG	CTA	GAA	1	C	A
TCGA-30-1857	DOCK4_ENS	ENST00000437633	p.H1412N	c.4234C>A	Unverified	AAT	CAC	ATC	1	C	A
TCGA-25-1313	DOCK6	ENST00000319867	p.A1992D	c.5975C>A	Unverified	AAG	GCC	CTG	2	C	A
TCGA-30-1714	DOK2	ENST00000276420	p.P280T	c.838C>A	Unverified	ccg	ccg	cct	1	C	A
TCGA-23-1031	DPM1	ENST00000371588	p.R8S	c.22C>A	Verified	AGT	CGT	AGT	1	C	A
TCGA-20-1686	DPP3	ENST00000360510	p.A382D	c.1145C>A	Unverified	TTC	GCT	GGC	2	C	A
TCGA-61-1907	DPYD	ENST00000370192	p.R394R	c.1180C>A	Unverified	CCA	CGG	AAG	1	C	A
TCGA-04-1542	DRD1	ENST00000329144	p.L76M	c.226C>A	Verified	GTC	CTG	GTC	1	C	A

TCGA-24-1103	DRD3	ENST00000383673	p.A33D	c.98C>A	Verified	TAT	GCC	CTC	2	C	A
TCGA-23-1117	DSG3	ENST00000257189	p.S874Y	c.2621C>A	Verified	CCC	TCT	AAA	2	C	A
TCGA-23-1117	DSG3	ENST00000257189	p.S874Y	c.2621C>A	Verified	CCC	TCT	AAA	2	C	A
TCGA-29-1763	DSG3	ENST00000257189	p.L4L	c.12C>A	Unverified	GGG	CTC	TTC	3	C	A
TCGA-61-1895	DSG4	ENST00000308128	p.Q1005K	c.3013C>A	Unverified	ATG	CAA	ATG	1	C	A
TCGA-61-1895	DSG4_ENST(	ENST00000359747	p.Q1024K	c.3070C>A	Unverified	ATG	CAA	ATG	1	C	A
TCGA-29-1693	DST	ENST00000370765	p.V2082V	c.6246C>A	Unverified	TCA	GTC	AGG	3	C	A
TCGA-29-1693	DST_ENST0(	ENST00000370765	p.V2082V	c.6246C>A	Unverified	TCA	GTC	AGG	3	C	A
TCGA-09-2049	DST_ENST0(	ENST00000370769	p.Q2278K	c.6832C>A	Verified	GAC	CAG	TTT	1	C	A
TCGA-61-1737	DSTYK_ENST	ENST00000367162	p.Q218K	c.652C>A	Unverified	TTA	CAG	GAA	1	C	A
TCGA-30-1891	DUSP27	ENST00000361200	p.T819T	c.2457C>A	Unverified	GGG	ACC	AGC	3	C	A
TCGA-24-1423	DUSP5	ENST00000369583	p.I317I	c.951C>A	Unverified	GAG	ATC	CTG	3	C	A
TCGA-29-1691	EBF3	ENST00000368648	p.N100K	c.300C>A	Unverified	CCA	AAC	AAC	3	C	A
TCGA-29-1691	EBF3_ENST(	ENST00000355311	p.N100K	c.300C>A	Unverified	CCA	AAC	AAC	3	C	A
TCGA-13-0762	EDA	ENST00000374552	p.L62I	c.184C>A	Verified	TAC	CTA	GAG	1	C	A
TCGA-13-0762	EDA	ENST00000374552	p.L62I	c.184C>A	Verified	TAC	CTA	GAG	1	C	A
TCGA-09-1674	EDC4	ENST00000358933	p.S1042R	c.3126C>A	Unverified	CGC	AGC	ATA	3	C	A
TCGA-25-2400	EDIL3	ENST00000296591	p.L162M	c.484C>A	Unverified	cca	ctg	gga	1	C	A
TCGA-23-1123	EFCAB4B	ENST00000252322	p.L236L	c.708C>A	Verified	CAT	CTC	TAT	3	C	A
TCGA-23-1123	EFCAB4B	ENST00000252322	p.L236L	c.708C>A	Verified	CAT	CTC	TAT	3	C	A
TCGA-23-2078	EFHC2	ENST00000333807	p.N594K	c.1782C>A	Verified	gga	aac	ctt	3	C	A
TCGA-25-1326	EIF2D	ENST00000271764	p.S361Y	c.1082C>A	Verified	CCC	TCC	CCG	2	C	A
TCGA-25-2042	EIF3L	ENST00000412331	p.Q243K	c.727C>A	Unverified	CGA	CAG	TTG	1	C	A
TCGA-24-1846	EIF4G2	ENST00000339995	p.H307N	c.919C>A	Unverified	GGA	CAG	ACA	1	C	A
TCGA-13-0923	EIF5B	ENST00000289371	p.Q900K	c.2698C>A	Verified	ACT	CAG	ATT	1	C	A
TCGA-13-0904	ELOVL3	ENST00000370005	p.H252N	c.754C>A	Verified	GCC	CAC	TTC	1	C	A
TCGA-25-1326	EMILIN1	ENST00000380320	p.L762I	c.2284C>A	Unverified	GGG	CTC	CGG	1	C	A
TCGA-04-1331	EMILIN2	ENST00000254528	p.T245K	c.734C>A	Verified	GAA	ACG	GGC	2	C	A
TCGA-24-1435	ENPP2	ENST00000259486	p.P840T	c.2518C>A	Verified	GGC	CCT	CTC	1	C	A
TCGA-30-1856	ENPP2	ENST00000259486	p.A98A	c.294C>A	Unverified	ACA	GCC	CGT	3	C	A
TCGA-30-1856	ENPP2_ENST	ENST00000522826	p.A98A	c.294C>A	Unverified	ACA	GCC	CGT	3	C	A
TCGA-23-1029	ENSG00000[illegible]	ENST00000326071	p.P332Q	c.995C>A	Unverified	CCC	CCA	GCC	2	C	A
TCGA-61-1740	ENSG00000[illegible]	ENST00000398617	p.P88T	c.262C>A	Unverified	AAC	CCT	ATG	1	C	A
TCGA-24-1103	ENSG00000[illegible]	ENST00000402465	p.T26K	c.77C>A	Verified	GAC	ACA	TCC	2	C	A

TCGA-29-1711	ENSG00000	ENST00000596508	p.P108H	c.323C>A	Unverified	GCC	CCC	TGG	2	C	A
TCGA-29-1711	ENSG00000	ENST00000596508	p.P108H	c.323C>A	Unverified	GCC	CCC	TGG	2	C	A
TCGA-23-1114	EPB42	ENST00000300215	p.T105T	c.315C>A	Unverified	AGG	ACC	CAA	3	C	A
TCGA-24-1423	EPC1	ENST00000263062	p.L361M	c.1081C>A	Verified	GCA	CTG	CCA	1	C	A
TCGA-61-2109	EPHB1	ENST00000398015	p.T117N	c.350C>A	Unverified	GAG	ACT	GAC	2	C	A
TCGA-61-2109	EPHB1_ENS	ENST00000398015	p.T117N	c.350C>A	Unverified	GAG	ACT	GAC	2	C	A
TCGA-13-1489	EPHB2	NM_017449	p.G986G	c.2958C>A	Verified	gag	ggc	cag	3	C	A
TCGA-24-2288	EPHB3	ENST00000330394	p.P74H	c.221C>A	Unverified	aat	ccc	atc	2	C	A
TCGA-61-2113	EPHB4	ENST00000358173	p.G82G	c.246C>A	Unverified	CGG	GGC	GCC	3	C	A
TCGA-13-1497	EPS8	ENST00000281172	p.P9H	c.26C>A	Verified	CAT	CCC	AGT	2	C	A
TCGA-61-2009	EPX	ENST00000225371	p.Q662K	c.1984C>A	Verified	AGA	CAG	CGC	1	C	A
TCGA-09-1674	ERN1	ENST00000433197	p.A868D	c.2603C>A	Unverified	AGA	GCC	GTG	2	C	A
TCGA-09-1674	ERN1_ENST(	ENST00000433197	p.A868D	c.2603C>A	Unverified	AGA	GCC	GTG	2	C	A
TCGA-29-1693	ETFDH	ENST00000511912	p.P545T	c.1633C>A	Unverified	ATA	CCT	GTA	1	C	A
TCGA-29-1703	ETV4_ENST(	ENST00000319349	p.P173H	c.518C>A	Unverified	CAC	CCT	GGC	2	C	A
TCGA-13-0887	ETV7	ENST00000340181	p.L90I	c.268C>A	Verified	ATC	CTC	ACC	1	C	A
TCGA-04-1361	EVI5	ENST00000370331	p.P770T	c.2308C>A	Verified	gac	ccc	gca	1	C	A
TCGA-04-1361	EVI5	ENST00000370331	p.P770T	c.2308C>A	Verified	gac	ccc	gca	1	C	A
TCGA-09-2049	EVX2	ENST00000308618	p.Y263*	c.789C>A	Verified	ACC	TAC	ATG	3	C	A
TCGA-13-0920	EXOC4	ENST00000253861	p.A6D	c.17C>A	Verified	GCA	GCT	GGT	2	C	A
TCGA-04-1347	EYA3	ENST00000373871	p.T112N	c.335C>A	Verified	GCA	ACC	CAA	2	C	A
TCGA-61-1998	FADS3	ENST00000278829	p.S358R	c.1074C>A	Verified	GTC	AGC	TCT	3	C	A
TCGA-61-1740	FAHD2B	ENST00000272610	p.Q251K	c.751C>A	Unverified	AAC	CAG	ATG	1	C	A
TCGA-61-1910	FAM115A	ENST00000355951	p.S121S	c.363C>A	Unverified	GAC	TCC	CTG	3	C	A
TCGA-13-1498	FAM123A	ENST00000357816	p.G534G	c.1602C>A	Verified	CGG	GGC	TTG	3	C	A
TCGA-29-1784	FAM126A	ENST00000432176	p.C100*	c.300C>A	Unverified	GGA	TGC	ATT	3	C	A
TCGA-23-1021	FAM129A	ENST00000367511	p.P728T	c.2182C>A	Verified	GCT	CCA	GTG	1	C	A
TCGA-29-1764	FAM129A	ENST00000367511	p.P663T	c.1987C>A	Unverified	GAC	CCC	GTG	1	C	A
TCGA-30-1855	FAM135B	ENST00000395297	p.Q954K	c.2860C>A	Unverified	CAG	CAA	AGC	1	C	A
TCGA-24-2280	FAM135B	ENST00000395297	p.L1402L	c.4206C>A	Unverified	GGA	CTC	AAC	3	C	A
TCGA-61-2094	FAM135B	ENST00000395297	p.G1351G	c.4053C>A	Unverified	CTG	GGC	CCT	3	C	A
TCGA-61-2008	FAM179A	ENST00000379558	p.S356Y	c.1067C>A	Verified	AAG	TCT	GCC	2	C	A
TCGA-23-1124	FAM187B	ENST00000324675	p.H50Q	c.150C>A	Verified	GCG	CAC	TGG	3	C	A
TCGA-13-0900	FAM47B	ENST00000329357	p.L374I	c.1120C>A	Verified	GAA	CTC	ACC	1	C	A

TCGA-29-1775	FAM65A_EN	ENST00000379312	p.S35R	c.105C>A	Unverified	CCC	AGC	CTC	3	C	A
TCGA-29-2427	FAM73A	ENST00000370791	p.A32D	c.95C>A	Verified	GGA	GCC	ATG	2	C	A
TCGA-24-1422	FAM83C	ENST00000374408	p.T674T	c.2022C>A	Verified	CTA	ACC	CTG	3	C	A
TCGA-25-1326	FAM84B	ENST00000304916	p.P296T	c.886C>A	Unverified	CCC	CCT	GCG	1	C	A
TCGA-61-2095	FANCC_ENS	ENST00000289081	p.L450M	c.1348C>A	Unverified	GTG	CTG	GGC	1	C	A
TCGA-09-2049	FAP	ENST00000188790	p.T741K	c.2222C>A	Verified	TCC	ACG	AAC	2	C	A
TCGA-29-1696	FASTKD5	ENST00000380266	p.S762Y	c.2285C>A	Unverified	ACC	TCT	GCT	2	C	A
TCGA-29-1696	FASTKD5	ENST00000380266	p.S762Y	c.2285C>A	Unverified	ACC	TCT	GCT	2	C	A
TCGA-20-1686	FAT	ENST00000441802	p.P4453P	c.13359C>A	Unverified	CCG	CCC	GAA	3	C	A
TCGA-10-0930	FAT1	ENST00000441802	p.P4393H	c.13178C>A	Verified	GTT	CCT	CTG	2	C	A
TCGA-10-0930	FAT1	ENST00000441802	p.P4393H	c.13178C>A	Verified	GTT	CCT	CTG	2	C	A
TCGA-20-1686	FAT1	ENST00000441802	p.P4453P	c.13359C>A	Unverified	CCG	CCC	GAA	3	C	A
TCGA-25-1313	FAT2	ENST00000261800	p.P2951P	c.8853C>A	Verified	GAC	CCC	CTG	3	C	A
TCGA-29-1763	FAT3_ENST(	ENST00000298047_v61	p.Q4229K	c.12685C>A	Unverified	CCG	CAG	GTC	1	C	A
TCGA-23-1021	FAT3_ENST(	ENST00000298047_v61	p.A3313A	c.9939C>A	Verified	GAA	GCC	AAA	3	C	A
TCGA-24-1431	FAT3_ENST(	ENST00000298047_v61	p.Y1259*	c.3777C>A	Verified	GTC	TAC	CAG	3	C	A
TCGA-29-1763	FAT3_ENST(	ENST00000409404	p.Q4229K	c.12685C>A	Unverified	CCG	CAG	GTC	1	C	A
TCGA-13-1505	FAT4	ENST00000330166	p.I3413I	c.10239C>A	Verified	CAG	ATC	AGT	3	C	A
TCGA-13-1505	FAT4_ENST(	ENST00000394329	p.I3413I	c.10239C>A	Verified	CAG	ATC	AGT	3	C	A
TCGA-23-1117	FBLN2	ENST00000404922	p.A567E	c.1700C>A	Verified	CCT	GCG	CAT	2	C	A
TCGA-23-1117	FBLN2	ENST00000404922	p.A567E	c.1700C>A	Verified	CCT	GCG	CAT	2	C	A
TCGA-23-1117	FBLN2_ENS1	ENST00000492059	p.A1148E	c.3443C>A	Verified	CCT	GCG	CAT	2	C	A
TCGA-23-1117	FBLN2_ENS1	ENST00000492059	p.A1148E	c.3443C>A	Verified	CCT	GCG	CAT	2	C	A
TCGA-24-2289	FBXL20	ENST00000583610	p.S371Y	c.1112C>A	Unverified	gca	tcc	ctg	2	C	A
TCGA-13-0906	FBXO25	ENST00000276326	p.A347D	c.1040C>A	Unverified	gcg	gcc	gac	2	C	A
TCGA-61-2113	FBXO34	ENST00000313833	p.L317I	c.949C>A	Unverified	ttg	ctt	gca	1	C	A
TCGA-29-1769	FBXO38	ENST00000340253	p.T256K	c.767C>A	Unverified	gta	aca	ggc	2	C	A
TCGA-24-0979	FBXO39	ENST00000321535	p.N168K	c.504C>A	Verified	CTC	AAC	CTA	3	C	A
TCGA-24-1423	FBXO40	ENST00000338040	p.P678T	c.2032C>A	Verified	GAC	CCG	ATT	1	C	A
TCGA-29-1764	FBXW7_NM	ENST00000263981	p.S11R	c.33C>A	Unverified	ctg	agc	tgc	3	C	A
TCGA-04-1542	FEZ2	ENST00000405912	p.Y235*	c.705C>A	Verified	GAG	TAC	TCT	3	C	A
TCGA-25-2401	FGA	ENST00000302053	p.P565H	c.1694C>A	Verified	CAC	CCT	GGG	2	C	A
TCGA-24-1845	FGA	ENST00000302053	p.S756S	c.2268C>A	Unverified	GTC	TCC	TCC	3	C	A
TCGA-29-1785	FGA	ENST00000302053	p.D789E	c.2367C>A	Unverified	TTT	GAC	AGG	3	C	A

TCGA-29-1785	FGA	ENST00000302053	p.D789E	c.2367C>A	Unverified	TTT	GAC	AGG	3	C	A
TCGA-04-1338	FGD6	ENST00000343958	p.P855H	c.2564C>A	Verified	GAG	CCT	GAC	2	C	A
TCGA-30-1855	FGF4	ENST00000168712	p.P204T	c.610C>A	Unverified	ctc	ccc	agg	1	C	A
TCGA-30-1714	FHOD3	ENST00000257209	p.A602D	c.1805C>A	Unverified	ATA	GCC	TCT	2	C	A
TCGA-29-2427	FIGN	ENST00000333129	p.P441T	c.1321C>A	Verified	CAC	CCA	ATG	1	C	A
TCGA-30-1714	FIGN	ENST00000333129	p.A36D	c.107C>A	Unverified	CCT	GCC	CAC	2	C	A
TCGA-24-1850	FLG	ENST00000368799	p.S1592Y	c.4775C>A	Unverified	GGA	TCC	AGT	2	C	A
TCGA-13-0920	FLG2	ENST00000388718	p.Q796K	c.2386C>A	Verified	GGA	CAA	CAT	1	C	A
TCGA-25-1313	FLG2	ENST00000388718	p.H2196N	c.6586C>A	Verified	ACA	CAT	TCA	1	C	A
TCGA-29-1696	FLG2	ENST00000388718	p.S2315Y	c.6944C>A	Unverified	CAA	TCC	ACA	2	C	A
TCGA-29-1696	FLG2	ENST00000388718	p.S2315Y	c.6944C>A	Unverified	CAA	TCC	ACA	2	C	A
TCGA-61-1904	FLNC	ENST00000325888	p.S1780Y	c.5339C>A	Unverified	GAG	TCC	ATG	2	C	A
TCGA-04-1530	FLVCR2	ENST00000238667	p.I451I	c.1353C>A	Unverified	GGG	ATC	ATC	3	C	A
TCGA-23-1123	FMN2	ENST00000319653	p.Q952K	c.2854C>A	Verified	TCA	CAG	CCT	1	C	A
TCGA-23-1123	FMN2	ENST00000319653	p.Q952K	c.2854C>A	Verified	TCA	CAG	CCT	1	C	A
TCGA-23-1032	FMNL2	ENST00000288670	p.P558P	c.1674C>A	Unverified	CCG	CCC	CCT	3	C	A
TCGA-29-1762	FN1	ENST00000336916	p.D2300E	c.6900C>A	Unverified	TGT	GAC	AAC	3	C	A
TCGA-29-1762	FN1_ENST0(	ENST00000354785	p.D2422E	c.7266C>A	Unverified	TGT	GAC	AAC	3	C	A
TCGA-61-1737	FOSB	ENST00000353609	p.Q325K	c.973C>A	Unverified	GAC	CAG	CCT	1	C	A
TCGA-61-1904	FOXO1	ENST00000379561	p.S290*	c.869C>A	Unverified	GGA	TCA	CAG	2	C	A
TCGA-61-1904	FOXO1_ENS	ENST00000379561	p.S290*	c.869C>A	Unverified	GGA	TCA	CAG	2	C	A
TCGA-20-0990	FREM2	ENST00000280481	p.A2972A	c.8916C>A	Verified	AAT	GCC	AAA	3	C	A
TCGA-24-1423	FREM2	ENST00000280481	p.D750E	c.2250C>A	Verified	ACA	GAC	GAA	3	C	A
TCGA-61-2095	FRG2	ENST00000378763	p.P201T	c.601C>A	Unverified	TCT	CCA	CTT	1	C	A
TCGA-04-1338	FRMD4B	ENST00000264546	p.Q422K	c.1264C>A	Verified	CCT	CAG	GTC	1	C	A
TCGA-24-2288	FRMPD2	ENST00000374201	p.R1053S	c.3157C>A	Unverified	GAA	CGC	ACG	1	C	A
TCGA-29-1769	FRMPD3	XM_042978.4	p.G1285G	c.3855C>A	Unverified	gaa	ggc	cct	3	C	A
TCGA-29-1769	FRMPD3_EN	ENST00000276185	p.G1236G	c.3708C>A	Unverified	GAA	GGC	CCT	3	C	A
TCGA-23-1110	FRMPD4	ENST00000380682	p.P965Q	c.2894C>A	Verified	cct	cca	aag	2	C	A
TCGA-24-1844	FRY	ENST00000380250	p.L791I	c.2371C>A	Unverified	CAG	CTA	AGT	1	C	A
TCGA-23-1124	FRZB	ENST00000295113	p.A115D	c.344C>A	Verified	CGG	GCC	CGG	2	C	A
TCGA-13-0924	FSHR	ENST00000406846	p.N632K	c.1896C>A	Verified	AAA	AAC	TTT	3	C	A
TCGA-23-1022	FSIP1	ENST00000350221	p.S156Y	c.467C>A	Verified	AAG	TCT	GCA	2	C	A
TCGA-25-2392	FSTL5	ENST00000306100	p.S305Y	c.914C>A	Unverified	GGG	TCC	TTG	2	C	A

TCGA-13-0923	FUBP3	ENST00000319725	p.A417D	c.1250C>A	Verified	GTG	GCC	AGG	2	C	A
TCGA-24-2024	GAA	ENST00000390015	p.S918Y	c.2753C>A	Verified	CTC	TCC	AAC	2	C	A
TCGA-13-1498	GABRG2	ENST00000356592	p.S256S	c.768C>A	Verified	ACT	TCC	GGA	3	C	A
TCGA-23-1022	GABRG2	ENST00000356592	p.T165T	c.495C>A	Verified	ACC	ACC	CCC	3	C	A
TCGA-36-1577	GADD45GIP	ENST00000316939	p.A122E	c.365C>A	Unverified	ATC	GCA	GAG	2	C	A
TCGA-29-1691	GALE	ENST00000374497	p.L127M	c.379C>A	Unverified	AAC	CTG	GTG	1	C	A
TCGA-13-0916	GALNT13	ENST00000392825	p.L414I	c.1240C>A	Verified	TAC	CTA	GAA	1	C	A
TCGA-61-1910	GALNTL2	ENST00000339732	p.I526I	c.1578C>A	Unverified	GAC	ATC	CTG	3	C	A
TCGA-04-1338	GAS2	ENST00000454584	p.L67I	c.199C>A	Verified	ttg	ctc	tgt	1	C	A
TCGA-61-2012	GBP6	ENST00000370456	p.T98N	c.293C>A	Verified	GAC	ACC	GAA	2	C	A
TCGA-04-1651	GDF3	ENST00000329913	p.L234M	c.700C>A	Unverified	ctg	ctg	gtg	1	C	A
TCGA-04-1651	GDF3	ENST00000329913	p.L234M	c.700C>A	Unverified	ctg	ctg	gtg	1	C	A
TCGA-61-2012	GDF3	ENST00000329913	p.L2I	c.4C>A	Verified	atg	ctt	cgt	1	C	A
TCGA-29-1763	GGT7	ENST00000336431	p.L461L	c.1383C>A	Unverified	CCA	CTC	CTG	3	C	A
TCGA-24-1423	GH2_ENST0	ENST00000456543	p.T177K	c.530C>A	Unverified	ACA	ACG	ATG	2	C	A
TCGA-24-1563	GIGYF2	ENST00000373563	p.Y538*	c.1614C>A	Verified	TAT	TAC	AAA	3	C	A
TCGA-10-0930	GIMAP6	ENST00000328902	p.L212M	c.634C>A	Verified	CAA	CTG	CGA	1	C	A
TCGA-10-0930	GIMAP6	ENST00000328902	p.L212M	c.634C>A	Verified	CAA	CTG	CGA	1	C	A
TCGA-25-2400	GJA1	ENST00000282561	p.D360E	c.1080C>A	Verified	GTG	GAC	CAG	3	C	A
TCGA-13-1510	GLI1	ENST00000228682	p.D647E	c.1941C>A	Unverified	gct	gac	cgt	3	C	A
TCGA-23-1124	GLS2	ENST00000311966	p.D550E	c.1650C>A	Verified	AAG	GAC	AGG	3	C	A
TCGA-25-2401	GNA11	ENST00000078429	p.Y235*	c.705C>A	Unverified	GAA	TAC	GAC	3	C	A
TCGA-61-1907	GNB4	ENST00000232564	p.Q75K	c.223C>A	Unverified	tct	caa	gat	1	C	A
TCGA-36-1577	GOLIM4	ENST00000470487	p.A389E	c.1166C>A	Unverified	CAC	GCG	CGT	2	C	A
TCGA-61-1910	GPC5	ENST00000377067	p.S93R	c.279C>A	Unverified	TCC	AGC	TCT	3	C	A
TCGA-09-1674	GPD1	ENST00000301149	p.I199I	c.597C>A	Unverified	GAG	ATC	TGT	3	C	A
TCGA-61-1906	GPD2	ENST00000310454	p.P320H	c.959C>A	Unverified	ATG	CCT	GGT	2	C	A
TCGA-61-1906	GPD2	ENST00000310454	p.P320H	c.959C>A	Unverified	ATG	CCT	GGT	2	C	A
TCGA-09-2050	GPR119	ENST00000276218	p.Q198K	c.592C>A	Verified	cag	cag	att	1	C	A
TCGA-24-1843	GPR137C	ENST00000321662	p.T200N	c.599C>A	Unverified	TGG	ACT	GTG	2	C	A
TCGA-13-1497	GPR149	ENST00000389740	p.I314I	c.942C>A	Verified	TTG	ATC	CTA	3	C	A
TCGA-09-2049	GPR15	ENST00000284311	p.T338N	c.1013C>A	Verified	CTC	ACT	AAG	2	C	A
TCGA-23-1031	GPR176	ENST00000561100	p.P424T	c.1270C>A	Verified	CCA	CCC	CTG	1	C	A
TCGA-13-0760	GPR83	ENST00000243673	p.T403T	c.1209C>A	Verified	CCC	ACC	TCC	3	C	A

TCGA-61-1740	GPRC6A	ENST00000310357	p.P737T	c.2209C>A	Unverified	TTG	CCC	AGA	1	C	A
TCGA-25-2392	GPRIN1	ENST00000303991	p.S82S	c.246C>A	Verified	GCC	TCC	TGC	3	C	A
TCGA-04-1343	GRIA4	ENST00000282499	p.P384H	c.1151C>A	Verified	GGA	CCT	AGA	2	C	A
TCGA-24-1844	GRIK1	ENST00000389125	p.S739Y	c.2216C>A	Unverified	GAG	TCC	ACC	2	C	A
TCGA-24-1844	GRIK1_ENST	ENST00000399907	p.S754Y	c.2261C>A	Unverified	GAG	TCC	ACC	2	C	A
TCGA-61-1737	GRIN2B	ENST00000279593	p.T475N	c.1424C>A	Unverified	TTC	ACC	TAT	2	C	A
TCGA-13-1510	GRIN2B	ENST00000279593	p.S130S	c.390C>A	Unverified	GGC	TCC	TCT	3	C	A
TCGA-23-1124	GRIP1	ENST00000398016	p.S303S	c.909C>A	Verified	CTC	TCC	ATC	3	C	A
TCGA-04-1337	GRLF1_ENST	ENST00000317082_v61	p.T409T	c.1227C>A	Verified	GAT	ACC	GTC	3	C	A
TCGA-10-0930	GRM7	ENST00000389335	p.F122L	c.366C>A	Verified	ACT	TTC	GTC	3	C	A
TCGA-10-0930	GRM7	ENST00000389335	p.F122L	c.366C>A	Verified	ACT	TTC	GTC	3	C	A
TCGA-13-0795	GRM8	ENST00000339582	p.P643T	c.1927C>A	Verified	GCA	CCA	GAT	1	C	A
TCGA-29-1696	GSDMC	ENST00000276708	p.L117L	c.351C>A	Unverified	TCC	CTC	GAG	3	C	A
TCGA-29-1696	GSDMC	ENST00000276708	p.L117L	c.351C>A	Unverified	TCC	CTC	GAG	3	C	A
TCGA-23-2078	GTF2IRD1	ENST00000265755	p.P852H	c.2555C>A	Unverified	AAC	CCC	AAC	2	C	A
TCGA-29-1693	GUCA1B	ENST00000230361	p.L94M	c.280C>A	Unverified	AAG	CTG	AAG	1	C	A
TCGA-20-1686	GYG1	ENST00000345003	p.D279E	c.837C>A	Unverified	TCA	GAC	TTG	3	C	A
TCGA-25-2392	GZMH	ENST00000216338	p.P90H	c.269C>A	Verified	ATC	CCT	GTG	2	C	A
TCGA-20-1685	H3F3C	ENST00000340398	p.F78L	c.234C>A	Unverified	GAT	TTC	AAC	3	C	A
TCGA-09-1665	HACE1	ENST00000262903	p.H252N	c.754C>A	Verified	TAT	CAC	CCG	1	C	A
TCGA-61-1907	HAPLN2	ENST00000255039	p.Y66*	c.198C>A	Unverified	AGC	TAC	AAG	3	C	A
TCGA-29-1775	HAUS3	ENST00000243706	p.Q410K	c.1228C>A	Unverified	GTT	CAA	GAA	1	C	A
TCGA-09-2049	HBXIP	ENST00000256644	p.H169N	c.505C>A	Verified	GTG	CAC	AAA	1	C	A
TCGA-04-1356	HCN4	ENST00000261917	p.P618T	c.1852C>A	Verified	CAG	CCT	GGG	1	C	A
TCGA-13-0885	HCRTR2	ENST00000370862	p.T205T	c.615C>A	Verified	ACC	ACC	CTC	3	C	A
TCGA-13-1501	HEATR5B	ENST00000233099	p.S1237*	c.3710C>A	Verified	AAA	TCA	AAG	2	C	A
TCGA-13-1501	HEATR5B	ENST00000233099	p.S1237*	c.3710C>A	Verified	AAA	TCA	AAG	2	C	A
TCGA-04-1331	HEATR7B2	ENST00000399564	p.T456N	c.1367C>A	Verified	CTG	ACT	TTT	2	C	A
TCGA-29-1764	HECTD1	ENST00000399332	p.P50T	c.148C>A	Unverified	CCT	CCT	CGC	1	C	A
TCGA-61-1900	HECW1	ENST00000395891	p.Y1223*	c.3669C>A	Unverified	CCC	TAC	CGA	3	C	A
TCGA-61-1900	HECW1_ENS	ENST00000395891	p.Y1223*	c.3669C>A	Unverified	CCC	TAC	CGA	3	C	A
TCGA-24-1845	HEG1	ENST00000311127	p.L1163I	c.3487C>A	Unverified	CAG	CTC	TTG	1	C	A
TCGA-24-1470	HEG1	ENST00000311127	p.I530I	c.1590C>A	Verified	TCG	ATC	GCT	3	C	A
TCGA-24-2289	HEPH	NM_138737.1	p.P748H	c.2243C>A	Unverified	tgc	cct	gac	2	C	A

TCGA-25-2042	HFE	ENST00000357618	p.Y135*	c.405C>A	Unverified	GGC	TAC	TGG	3	C	A
TCGA-29-2427	HHATL	ENST00000310417	p.L347I	c.1039C>A	Verified	TGG	CTT	TGC	1	C	A
TCGA-20-0991	HIPK1	NM_152696	p.A78D	c.233C>A	Verified	cca	gct	cct	2	C	A
TCGA-29-1711	HIPK2	NM_022740	p.D383E	c.1149C>A	Unverified	att	gac	atg	3	C	A
TCGA-29-1711	HIPK2	NM_022740	p.D383E	c.1149C>A	Unverified	att	gac	atg	3	C	A
TCGA-29-1711	HIPK2_ENST	ENST00000406875	p.D383E	c.1149C>A	Unverified	ATT	GAC	ATG	3	C	A
TCGA-29-1711	HIPK2_ENST	ENST00000406875	p.D383E	c.1149C>A	Unverified	ATT	GAC	ATG	3	C	A
TCGA-09-1674	HIST1H1E	ENST00000304218	p.A61D	c.182C>A	Unverified	GCC	GCT	CTC	2	C	A
TCGA-29-1784	HIST1H2AM	ENST00000359611	p.A87A	c.261C>A	Unverified	CTG	GCC	ATC	3	C	A
TCGA-29-1766	HIST1H4K	ENST00000357549	p.T83N	c.248C>A	Unverified	GTC	ACC	GCC	2	C	A
TCGA-13-1489	HIST2H2AB	ENST00000331128	p.V101V	c.303C>A	Verified	GGT	GTC	ACC	3	C	A
TCGA-09-2044	HLCS	ENST00000399120	p.N156K	c.468C>A	Verified	GTC	AAC	CTC	3	C	A
TCGA-04-1638	HMCN1	ENST00000271588	p.Q2713K	c.8137C>A	Unverified	GGA	CAG	CCC	1	C	A
TCGA-24-1469	HMGCLL1	ENST00000398661	p.S187S	c.561C>A	Verified	TGT	TCC	ATT	3	C	A
TCGA-09-2056	HOXA1	ENST00000343060	p.A185A	c.555C>A	Verified	CAC	GCC	AGC	3	C	A
TCGA-25-1326	HOXC6	ENST00000243108	p.S76Y	c.227C>A	Verified	AAT	TCC	TTT	2	C	A
TCGA-04-1347	HS3ST5	ENST00000312719	p.L47M	c.139C>A	Verified	CGA	CTG	GGT	1	C	A
TCGA-29-1696	HSPA1B	ENST00000375650	p.T636N	c.1907C>A	Unverified	CCT	ACC	ATT	2	C	A
TCGA-29-1696	HSPA1B	ENST00000375650	p.T636N	c.1907C>A	Unverified	CCT	ACC	ATT	2	C	A
TCGA-23-1032	HSPA1L	ENST00000375654	p.P438P	c.1314C>A	Verified	CAA	CCC	GGG	3	C	A
TCGA-13-0885	HSPB2	ENST00000304298	p.R131R	c.391C>A	Verified	tgg	cga	gtc	1	C	A
TCGA-13-0884	HSPG2	ENST00000374695	p.Q1062K	c.3184C>A	Verified	GAG	CAA	GCA	1	C	A
TCGA-29-1705	HTATIP2	ENST00000421577	p.L7M	c.19C>A	Unverified	GCC	CTG	TCG	1	C	A
TCGA-29-1705	HTATIP2_EN	ENST00000419348	p.L41M	c.121C>A	Unverified	GCC	CTG	TCG	1	C	A
TCGA-24-0979	HTR1E	ENST00000369584	p.T49N	c.146C>A	Verified	GGC	ACC	ACC	2	C	A
TCGA-24-1844	HTR2C	ENST00000371951	p.Q22K	c.64C>A	Unverified	tgg	caa	tgt	1	C	A
TCGA-29-1784	HTR3D	ENST00000334128	p.S32Y	c.95C>A	Unverified	CCT	TCC	ATG	2	C	A
TCGA-29-1784	HTR3D_ENS	ENST00000382489	p.S167Y	c.500C>A	Unverified	CCT	TCC	ATG	2	C	A
TCGA-61-1910	HTR5A	ENST00000287907	p.L59M	c.175C>A	Unverified	AAC	CTG	CTG	1	C	A
TCGA-13-0900	HUS1	ENST00000436444	p.H109N	c.325C>A	Verified	aaa	cac	ttt	1	C	A
TCGA-29-1777	HUWE1	XM_497119.1	p.Q3121K	c.9361C>A	Unverified	ttg	cag	cgc	1	C	A
TCGA-29-1777	HUWE1_EN	ENST00000342160	p.Q3231K	c.9691C>A	Unverified	TTG	CAG	CGC	1	C	A
TCGA-25-2042	HYAL3	ENST00000336307	p.G94G	c.282C>A	Unverified	GGG	GGC	ATC	3	C	A
TCGA-23-1032	IDO2	ENST00000389060	p.N121K	c.363C>A	Verified	AGG	AAC	TTG	3	C	A

TCGA-61-2095	IGDCC3	ENST00000327987	p.P738T	c.2212C>A	Unverified	GAT	CCT	GCA	1	C	A
TCGA-20-0991	IGF1R	ENST00000268035	p.F958L	c.2874C>A	Verified	GTC	TTC	CAT	3	C	A
TCGA-13-0762	IGSF1	ENST00000361420	p.T609N	c.1826C>A	Verified	TTA	ACC	CTC	2	C	A
TCGA-13-0762	IGSF1	ENST00000361420	p.T609N	c.1826C>A	Verified	TTA	ACC	CTC	2	C	A
TCGA-13-0916	IGSF21	ENST00000251296	p.D314E	c.942C>A	Unverified	ATC	GAC	AAC	3	C	A
TCGA-24-2262	IGSF9B_ENS	ENST00000321016_v61	p.P1092T	c.3274C>A	Unverified	TAC	CCG	GGC	1	C	A
TCGA-04-1367	IKBKAP	ENST00000374647	p.D460E	c.1380C>A	Verified	gct	gac	cct	3	C	A
TCGA-23-1124	IKZF4_ENST(	ENST00000262032	p.D48E	c.144C>A	Verified	CAA	GAC	TCC	3	C	A
TCGA-04-1343	IL12RB2	ENST00000262345	p.L253M	c.757C>A	Verified	GTA	CTG	CTT	1	C	A
TCGA-30-1855	IL21R_ENST(	ENST00000395754	p.P123T	c.367C>A	Unverified	CCC	CCT	TTC	1	C	A
TCGA-13-1498	IL2RB	ENST00000216223	p.D426E	c.1278C>A	Unverified	GAT	GAC	CTG	3	C	A
TCGA-13-0760	IL3	ENST00000296870	p.A107A	c.321C>A	Verified	CTG	GCC	ACG	3	C	A
TCGA-25-2401	IL3	ENST00000296870	p.I118I	c.354C>A	Verified	CAT	ATC	AAG	3	C	A
TCGA-24-0979	IL36G	ENST00000259205	p.P113P	c.339C>A	Verified	GAG	CCC	GTG	3	C	A
TCGA-13-0714	IL8	ENST00000307407	p.C61*	c.183C>A	Verified	CAC	TGC	GCC	3	C	A
TCGA-61-2094	INO80D_EN!	ENST00000403263	p.T874T	c.2622C>A	Unverified	CCA	ACC	CAA	3	C	A
TCGA-61-1900	INPP5D_EN!	ENST00000359570	p.L676I	c.2026C>A	Unverified	GTC	CTC	TGG	1	C	A
TCGA-29-1781	INSC	ENST00000379554	p.H316N	c.946C>A	Unverified	GTC	CAC	CAG	1	C	A
TCGA-24-2267	INSR	ENST00000302850	p.N175K	c.525C>A	Verified	TTG	AAC	AAA	3	C	A
TCGA-24-2280	INSR_ENST0	ENST00000302850	p.S1094Y	c.3281C>A	Unverified	GTG	TCC	AAG	2	C	A
TCGA-30-1857	INSRR_ENST	ENST00000368195	p.P670Q	c.2009C>A	Unverified	GAT	CCG	CGC	2	C	A
TCGA-13-0885	IQGAP2	ENST00000274364	p.L635I	c.1903C>A	Verified	TGG	CTC	ACA	1	C	A
TCGA-13-0887	IRGC	ENST00000244314	p.D130E	c.390C>A	Verified	GTA	GAC	TTC	3	C	A
TCGA-30-1718	IRS4	ENST00000372129	p.D842E	c.2526C>A	Unverified	cta	gac	aaa	3	C	A
TCGA-61-1904	IRX1	ENST00000302006	p.L205I	c.613C>A	Unverified	gcg	ctc	ttc	1	C	A
TCGA-61-1907	IRX2	NM_033267.2	p.T163N	c.488C>A	Unverified	tcc	acc	tgg	2	C	A
TCGA-04-1367	ISX	ENST00000308700	p.P213T	c.637C>A	Verified	CAG	CCT	GTC	1	C	A
TCGA-09-2056	ISYNA1	ENST00000338128	p.T326N	c.977C>A	Verified	AAG	ACC	ATG	2	C	A
TCGA-61-1895	ITGA2B	ENST00000262407	p.L752M	c.2254C>A	Unverified	cag	ctg	cag	1	C	A
TCGA-13-0900	ITGA4	ENST00000397033	p.P642H	c.1925C>A	Verified	AAG	CCC	CAT	2	C	A
TCGA-13-1499	ITGA6	ENST00000264107	p.Q485K	c.1453C>A	Verified	CGC	CAG	AAA	1	C	A
TCGA-25-2392	ITGAV	ENST00000261023	p.S501Y	c.1502C>A	Verified	GTT	TCC	TGT	2	C	A
TCGA-24-2024	ITIH1	ENST00000273283	p.A79A	c.237C>A	Verified	ACT	GCC	AAT	3	C	A
TCGA-20-0990	ITIH2	ENST00000358415	p.N96K	c.288C>A	Verified	GTG	AAC	AAT	3	C	A

TCGA-13-1488	ITIH4	ENST00000266041	p.T51K	c.152C>A	Unverified	CAC	ACG	GTC	2	C	A
TCGA-61-1998	ITIH5L	ENST00000218436	p.Q461K	c.1381C>A	Verified	cta	cag	ctg	1	C	A
TCGA-24-1104	ITPR2	ENST00000381340	p.L1189I	c.3565C>A	Verified	AGG	CTA	AGT	1	C	A
TCGA-13-0904	IYD	ENST00000344419	p.D53E	c.159C>A	Verified	AGT	GAC	CTG	3	C	A
TCGA-29-1695	JAKMIP3	ENST00000298622	p.I158I	c.474C>A	Unverified	GAG	ATC	TCC	3	C	A
TCGA-23-1022	JAM3	ENST00000299106	p.L68I	c.202C>A	Verified	CTG	CTT	TTC	1	C	A
TCGA-25-1326	JARID2	ENST00000341776	p.S986Y	c.2957C>A	Verified	atc	tcc	ccg	2	C	A
TCGA-13-0905	KALRN	ENST00000240874	p.A1142E	c.3425C>A	Verified	CAG	GCG	CTT	2	C	A
TCGA-20-1685	KATNB1	ENST00000379661	p.A10D	c.29C>A	Unverified	ACA	GCC	TGG	2	C	A
TCGA-13-1497	KBTBD3	ENST00000526793	p.F54L	c.162C>A	Verified	GAT	TTC	AAA	3	C	A
TCGA-13-1488	KBTBD4	ENST00000526005	p.Q203K	c.607C>A	Verified	TCT	CAG	AAC	1	C	A
TCGA-04-1542	KCNA4	ENST00000328224	p.T470N	c.1409C>A	Verified	CAC	ACC	CTC	2	C	A
TCGA-13-0885	KCNA5	ENST00000252321	p.S395S	c.1185C>A	Verified	atg	tcc	ctg	3	C	A
TCGA-13-0793	KCNC4	ENST00000369787	p.F235L	c.705C>A	Verified	TTC	TTC	ATC	3	C	A
TCGA-23-1021	KCNE1	ENST00000399284	p.S128S	c.384C>A	Verified	CCT	TCC	CCA	3	C	A
TCGA-13-0883	KCNG1	ENST00000371571	p.F84L	c.252C>A	Verified	GAG	TTC	CCG	3	C	A
TCGA-29-1784	KCNH1	ENST00000271751	p.T866K	c.2597C>A	Unverified	AGG	ACA	AAA	2	C	A
TCGA-13-0714	KCNJ9	ENST00000368088	p.H323Q	c.969C>A	Verified	TTT	CAC	GAG	3	C	A
TCGA-24-1846	KCNU1	ENST00000399881	p.P803T	c.2407C>A	Unverified	CCC	CCA	CCC	1	C	A
TCGA-24-1846	KCNU1_ENS	ENST00000399881	p.P803T	c.2407C>A	Unverified	CCC	CCA	CCC	1	C	A
TCGA-13-1488	KDM1A	ENST00000400181	p.P152T	c.454C>A	Verified	ctt	ccc	cca	1	C	A
TCGA-13-1507	KDM3B	ENST00000314358	p.L875M	c.2623C>A	Verified	CCC	CTG	AAA	1	C	A
TCGA-24-2290	KDM5B	NM_006618.2	p.L878I	c.2632C>A	Verified	gat	ctc	tac	1	C	A
TCGA-24-2290	KDM5B	NM_006618.2	p.L878I	c.2632C>A	Verified	gat	ctc	tac	1	C	A
TCGA-24-2035	KEAP1	ENST00000393623	p.S45S	c.135C>A	Verified	CCC	TCC	CAG	3	C	A
TCGA-30-1857	KIAA0100	ENST00000005905	p.S556*	c.1667C>A	Unverified	AAG	TCA	GCT	2	C	A
TCGA-23-1110	KIAA0247	ENST00000342745	p.S120Y	c.359C>A	Verified	CTG	TCT	ATA	2	C	A
TCGA-29-1698	KIAA0556	ENST00000261588	p.T1552N	c.4655C>A	Unverified	CAC	ACC	ATC	2	C	A
TCGA-29-1698	KIAA0556_E	ENST00000261588	p.T1552N	c.4655C>A	Unverified	CAC	ACC	ATC	2	C	A
TCGA-13-1499	KIAA0748	ENST00000316577	p.T377N	c.1130C>A	Verified	CAG	ACT	TTT	2	C	A
TCGA-25-2401	KIAA1324L	ENST00000297222	p.T314N	c.941C>A	Verified	TAC	ACC	CAT	2	C	A
TCGA-13-0886	KIAA1522	ENST00000401073	p.G486G	c.1458C>A	Unverified	TCT	GGC	CGG	3	C	A
TCGA-24-1843	KIAA1755	ENST00000279024	p.D224E	c.672C>A	Unverified	CCA	GAC	AAC	3	C	A
TCGA-04-1542	KIAA1919	ENST00000368847	p.P381T	c.1141C>A	Verified	TAC	CCT	GAT	1	C	A

TCGA-61-1907	KIAA1949	ENST00000399199	p.A159A	c.477C>A	Unverified	GGA	GCC	CAA	3	C	A
TCGA-24-2288	KIAA2018	ENST00000316407	p.T560N	c.1679C>A	Unverified	AGC	ACC	ACC	2	C	A
TCGA-30-1714	KIAA2022	ENST00000373468	p.P1344P	c.4032C>A	Unverified	gaa	ccc	atg	3	C	A
TCGA-09-1674	KIF13B	ENST00000524189	p.A1198E	c.3593C>A	Unverified	GAT	GCG	ACC	2	C	A
TCGA-09-1674	KIF13B_ENS	ENST00000524189	p.A1198E	c.3593C>A	Unverified	GAT	GCG	ACC	2	C	A
TCGA-23-1123	KIF17	ENST00000247986	p.S963R	c.2889C>A	Verified	CTC	AGC	ACA	3	C	A
TCGA-23-1123	KIF17	ENST00000247986	p.S963R	c.2889C>A	Verified	CTC	AGC	ACA	3	C	A
TCGA-09-2044	KIF3C	ENST00000264712	p.P445P	c.1335C>A	Verified	CCG	CCC	CAG	3	C	A
TCGA-23-2078	KISS1	ENST00000367194	p.T16T	c.48C>A	Verified	GCC	ACC	CAC	3	C	A
TCGA-30-1891	KLF17	ENST00000372299	p.P71T	c.211C>A	Unverified	TCC	CCT	TTG	1	C	A
TCGA-13-0760	KLHL4	ENST00000373114	p.P281H	c.842C>A	Verified	cat	cct	tca	2	C	A
TCGA-23-1022	KLK3	ENST00000326003	p.H78Q	c.234C>A	Verified	cgg	cac	agc	3	C	A
TCGA-24-1845	KLK3	ENST00000326003	p.T150T	c.450C>A	Unverified	ggg	acc	acc	3	C	A
TCGA-24-1845	KLK3_ENST0	ENST00000360617	p.T150T	c.450C>A	Unverified	GGG	ACC	ACC	3	C	A
TCGA-13-0793	KLK7	ENST00000304045	p.C211*	c.633C>A	Unverified	gtg	tgc	aga	3	C	A
TCGA-24-1463	KLKB1	ENST00000264690	p.Q103K	c.307C>A	Unverified	AAG	CAA	TGT	1	C	A
TCGA-13-0920	KLKB1	ENST00000264690	p.T520T	c.1560C>A	Verified	GTA	ACC	GGA	3	C	A
TCGA-24-1435	KMO	ENST00000366559	p.L272I	c.814C>A	Verified	AAA	CTC	CTA	1	C	A
TCGA-24-1847	KMO	ENST00000366559	p.R452R	c.1356C>A	Unverified	CTC	CGC	TTG	3	C	A
TCGA-04-1367	KNTC1	ENST00000333479	p.I736I	c.2208C>A	Verified	tcc	atc	tta	3	C	A
TCGA-23-1122	KPNA5	ENST00000368564	p.Q41K	c.121C>A	Unverified	aaa	caa	aaa	1	C	A
TCGA-24-1847	KRT2	ENST00000309680	p.T504K	c.1511C>A	Unverified	GTG	ACA	AGC	2	C	A
TCGA-04-1347	KRT25	ENST00000312150	p.L198M	c.592C>A	Verified	ACC	CTG	TGC	1	C	A
TCGA-23-2078	KRT32	ENST00000225899	p.P433T	c.1297C>A	Unverified	GTG	CCA	CGC	1	C	A
TCGA-20-1687	KRT36	ENST00000328119	p.L56L	c.168C>A	Unverified	GGC	CTC	TCT	3	C	A
TCGA-04-1638	KRT6A	ENST00000330722	p.A500D	c.1499C>A	Unverified	GGT	GCC	AGT	2	C	A
TCGA-13-0916	KRT72	ENST00000293745	p.T339N	c.1016C>A	Verified	CTC	ACC	AAG	2	C	A
TCGA-29-1777	KRTAP6-1	ENST00000329122	p.T12T	c.36C>A	Unverified	GGC	ACC	CCT	3	C	A
TCGA-13-0761	L1CAM	ENST00000370060	p.T910N	c.2729C>A	Verified	TTC	ACC	TTC	2	C	A
TCGA-13-0761	L1CAM	ENST00000370060	p.T910N	c.2729C>A	Verified	TTC	ACC	TTC	2	C	A
TCGA-13-1505	L1CAM	ENST00000370060	p.A897A	c.2691C>A	Verified	CAG	GCC	TTT	3	C	A
TCGA-23-1021	LAMA3	ENST00000313654	p.N2296K	c.6888C>A	Verified	gcc	aac	gac	3	C	A
TCGA-04-1336	LAMB1	ENST00000222399	p.Q1037K	c.3109C>A	Verified	GTG	CAA	GAG	1	C	A
TCGA-09-1665	LAMB4	ENST00000205386	p.D1219E	c.3657C>A	Verified	AAA	GAC	CTC	3	C	A

TCGA-24-2035	LAMC3	ENST00000361069	p.T1292N	c.3875C>A	Verified	CGA	ACC	CTC	2	C	A
TCGA-13-0755	LASS4	ENST00000251363	p.T282T	c.846C>A	Verified	CCC	ACC	CAG	3	C	A
TCGA-13-0714	LATS1_ENST	ENST00000253339	p.P1028T	c.3082C>A	Verified	ATT	CCT	AAA	1	C	A
TCGA-13-0714	LBP	ENST00000217407	p.L349M	c.1045C>A	Verified	CTC	CTG	AAC	1	C	A
TCGA-23-1114	LCE2B	ENST00000368780	p.P96H	c.287C>A	Unverified	GAA	CCT	TCT	2	C	A
TCGA-13-0905	LCP1	ENST00000323076	p.G113G	c.339C>A	Verified	GTT	GGC	ACC	3	C	A
TCGA-23-1021	LCP2	ENST00000046794	p.A393D	c.1178C>A	Verified	AGA	GCC	GAA	2	C	A
TCGA-61-1910	LDHC	NM_002301.2	p.R297R	c.889C>A	Unverified	ggg	cgg	aat	1	C	A
TCGA-30-1857	LDLR	ENST00000558518	p.C95*	c.285C>A	Unverified	GAC	TGC	GAC	3	C	A
TCGA-23-1031	LEPRE1	ENST00000236040	p.N423K	c.1269C>A	Verified	GGG	AAC	CTT	3	C	A
TCGA-25-1313	LGALS12	ENST00000394618	p.I92I	c.276C>A	Verified	GAT	ATC	GCC	3	C	A
TCGA-23-1111	LGALS4	ENST00000307751	p.I223I	c.669C>A	Unverified	GCT	ATC	AAC	3	C	A
TCGA-09-2044	LGALS9C	ENST00000328114	p.D27E	c.81C>A	Verified	CAG	GAC	GGA	3	C	A
TCGA-24-2267	LHX8	ENST00000294638	p.P304Q	c.911C>A	Verified	TCT	CCA	CCC	2	C	A
TCGA-61-1914	LIG3	NM_002311.2	p.T709K	c.2126C>A	Unverified	cat	aca	gct	2	C	A
TCGA-61-1914	LIG3_ENST0	ENST00000378526	p.T796K	c.2387C>A	Unverified	CAT	ACA	GCT	2	C	A
TCGA-23-1110	LILRA3	ENST00000251390	p.G81G	c.243C>A	Verified	AAG	GGC	CAG	3	C	A
TCGA-29-2427	LILRA6	ENST00000396365	p.L243L	c.729C>A	Unverified	ACC	CTC	CAG	3	C	A
TCGA-13-1488	LIPJ	ENST00000371939	p.S292Y	c.875C>A	Verified	ACG	TCT	CCA	2	C	A
TCGA-29-2427	LLGL1	ENST00000316843	p.A410A	c.1230C>A	Verified	TGG	GCC	CGC	3	C	A
TCGA-13-0913	LMNA	ENST00000368300	p.N580K	c.1740C>A	Unverified	tac	aac	ctg	3	C	A
TCGA-30-1855	LOC51059	ENST00000395297	p.Q954K	c.2860C>A	Unverified	CAG	CAA	AGC	1	C	A
TCGA-24-2280	LOC51059	ENST00000395297	p.L1402L	c.4206C>A	Unverified	GGA	CTC	AAC	3	C	A
TCGA-61-2094	LOC51059	ENST00000395297	p.G1351G	c.4053C>A	Unverified	CTG	GGC	CCT	3	C	A
TCGA-24-1464	LOC51123	ENST00000520984	p.C41*	c.123C>A	Verified	ACC	TGC	ACT	3	C	A
TCGA-25-2400	LOC91807	ENST00000394809	p.G74G	c.222C>A	Unverified	CCG	GGC	CCG	3	C	A
TCGA-61-2008	LONRF1	ENST00000398246	p.Q771K	c.2311C>A	Verified	GAC	CAA	TCT	1	C	A
TCGA-20-0991	LOXL2	ENST00000389131	p.D28E	c.84C>A	Unverified	tat	gac	agc	3	C	A
TCGA-24-1843	LPA	ENST00000316300	p.L1253I	c.3757C>A	Unverified	GTC	CTT	GCG	1	C	A
TCGA-13-1481	LPA	ENST00000316300	p.S1299S	c.3897C>A	Verified	TGG	TCC	TCT	3	C	A
TCGA-29-1783	LPAR4	ENST00000435339	p.T187N	c.560C>A	Unverified	acc	acc	tgc	2	C	A
TCGA-13-0923	LPIN1	ENST00000256720	p.T627N	c.1880C>A	Verified	AAG	ACT	CTC	2	C	A
TCGA-23-1022	LRCH4	ENST00000310300	p.P535T	c.1603C>A	Verified	GTT	CCA	GAT	1	C	A
TCGA-04-1356	LRFN2	ENST00000338305	p.P234H	c.701C>A	Verified	CCA	CCC	TTG	2	C	A

TCGA-29-1705	LRFN2	ENST00000338305	p.L271L	c.813C>A	Unverified	GGC	CTC	AAG	3	C	A
TCGA-24-2035	LRIG1	AF381545.1	p.A984D	c.2951C>A	Verified	act	gcc	gct	2	C	A
TCGA-61-1900	LRIG1	AF381545.1	p.R647R	c.1941C>A	Unverified	cga	cgc	atg	3	C	A
TCGA-20-1686	LRP1B	ENST00000389484	p.T2529T	c.7587C>A	Unverified	CTC	ACC	TGT	3	C	A
TCGA-25-2392	LRP2	ENST00000263816	p.Q1959K	c.5875C>A	Unverified	CAC	CAG	CTT	1	C	A
TCGA-23-2077	LRP2	ENST00000263816	p.A1967E	c.5900C>A	Verified	ATT	GCA	GTC	2	C	A
TCGA-13-0920	LRP6	ENST00000261349	p.S1613Y	c.4838C>A	Verified	TCC	TCC	TGA	2	C	A
TCGA-23-1124	LRPAP1	ENST00000500728	p.Q244K	c.730C>A	Verified	CAC	CAG	GGC	1	C	A
TCGA-29-1764	LRRC30	ENST00000383467	p.Y294*	c.882C>A	Unverified	GGC	TAC	CTG	3	C	A
TCGA-23-1110	LRRC6	ENST00000250173	p.D241E	c.723C>A	Verified	TTA	GAC	AAC	3	C	A
TCGA-04-1338	LRRC63	ENST00000446175	p.Q169K	c.505C>A	Verified	GCT	CAA	AAA	1	C	A
TCGA-24-1103	LRRC7	ENST00000035383	p.P1496H	c.4487C>A	Verified	CAG	CCT	GGT	2	C	A
TCGA-24-1845	LRRK1	SU_LRRK1	p.N737K	c.2211C>A	Unverified	tgg	aac	ctg	3	C	A
TCGA-24-1845	LRRK1_ENST	ENST00000388948	p.N725K	c.2175C>A	Unverified	TGG	AAC	CTG	3	C	A
TCGA-25-1326	LRRK2_ENST	ENST00000298910	p.D1756E	c.5268C>A	Verified	TTA	GAC	AAT	3	C	A
TCGA-24-1431	LRRTM1	ENST00000295057	p.P516H	c.1547C>A	Verified	CAG	CCC	GCG	2	C	A
TCGA-29-1701	LRRTM4	ENST00000409088	p.R232S	c.694C>A	Unverified	CTC	CGC	TCA	1	C	A
TCGA-29-1701	LRRTM4_EN	ENST00000409093	p.R232S	c.694C>A	Unverified	CTC	CGC	TCA	1	C	A
TCGA-29-1784	LSM12	ENST00000293406	p.I55I	c.165C>A	Unverified	GAC	ATC	TTG	3	C	A
TCGA-04-1651	LY6G6F	ENST00000375832	p.G56G	c.168C>A	Unverified	GCA	GGC	TCC	3	C	A
TCGA-04-1651	LY6G6F	ENST00000375832	p.G56G	c.168C>A	Unverified	GCA	GGC	TCC	3	C	A
TCGA-25-1313	LYST	ENST00000389793	p.P594H	c.1781C>A	Verified	ATT	CCT	TTG	2	C	A
TCGA-61-1915	LZTS1	ENST00000381569	p.P104H	c.311C>A	Unverified	CCC	CCC	AAG	2	C	A
TCGA-29-1775	MAB21L2	ENST00000317605	p.T342T	c.1026C>A	Unverified	CAG	ACC	TGG	3	C	A
TCGA-24-1563	MAGEA11	NM_005366.3	p.H239Q	c.717C>A	Verified	gag	cac	ttc	3	C	A
TCGA-24-1846	MAGEA13P	XM_498392.1	p.A303D	c.908C>A	Unverified	gat	gcc	agt	2	C	A
TCGA-13-0887	MAGEB5	XM_293407.3	p.A208D	c.623C>A	Verified	aga	gcc	tat	2	C	A
TCGA-23-1114	MAGEC1	ENST00000285879	p.T279N	c.836C>A	Unverified	tcc	act	tta	2	C	A
TCGA-04-1649	MAGED1	ENST00000375695	p.P30H	c.89C>A	Unverified	cag	cct	cct	2	C	A
TCGA-61-1910	MAGEE1	ENST00000361470	p.S293R	c.879C>A	Unverified	TCA	AGC	ACC	3	C	A
TCGA-61-1910	MAGEE1_EN	ENST00000361470	p.S293R	c.879C>A	Unverified	TCA	AGC	ACC	3	C	A
TCGA-13-1496	MAGI2	ENST00000354212	p.G930G	c.2790C>A	Verified	GAG	GGC	TTC	3	C	A
TCGA-04-1331	MAP2	ENST00000360351	p.Q34K	c.100C>A	Verified	GAT	CAA	GGC	1	C	A
TCGA-30-1718	MAP3K14	NM_003954	p.H683Q	c.2049C>A	Unverified	tac	cac	cag	3	C	A

TCGA-29-1762	MAP3K2	ENST00000409947	p.T339N	c.1016C>A	Unverified	TTG	ACC	GTA	2	C	A
TCGA-29-1762	MAP3K2_EN	ENST00000409947	p.T339N	c.1016C>A	Unverified	TTG	ACC	GTA	2	C	A
TCGA-36-1577	MAP3K3	ENST00000361733	p.L416I	c.1246C>A	Unverified	TTG	CTA	AAG	1	C	A
TCGA-61-1914	MAP4K1	NM_007181	p.T266N	c.797C>A	Unverified	gcc	acc	aag	2	C	A
TCGA-61-1914	MAP4K1_EN	ENST00000396857	p.T266N	c.797C>A	Unverified	GCC	ACC	AAG	2	C	A
TCGA-29-1763	MAPK7	ENST00000308406	p.S803Y	c.2408C>A	Unverified	gac	tcc	cca	2	C	A
TCGA-13-1408	MAST1	ENST00000251472	p.A269D	c.806C>A	Unverified	GTG	GCC	TTC	2	C	A
TCGA-29-1693	MBD3L3	ENST00000333843	p.A130D	c.389C>A	Unverified	AGG	GCT	GGT	2	C	A
TCGA-23-1124	MBTPS1	ENST00000343411	p.T238N	c.713C>A	Verified	TGG	ACC	AAC	2	C	A
TCGA-09-2051	MC4R	ENST00000299766	p.N3K	c.9C>A	Unverified	GTG	AAC	TCC	3	C	A
TCGA-13-1489	MCAT	ENST00000290429	p.S217Y	c.650C>A	Verified	AAG	TCT	TTA	2	C	A
TCGA-61-1915	MCC	ENST00000302475	p.D271E	c.813C>A	Unverified	CGG	GAC	CGG	3	C	A
TCGA-61-1915	MCC_ENST0	ENST00000408903	p.D461E	c.1383C>A	Unverified	CGG	GAC	CGG	3	C	A
TCGA-25-1313	MCM2	ENST00000265056	p.A263A	c.789C>A	Verified	GCT	GCC	CTG	3	C	A
TCGA-29-1784	MCTP2	ENST00000357742	p.N557K	c.1671C>A	Unverified	TGG	AAC	AAA	3	C	A
TCGA-25-1326	MDGA2	ENST00000357362	p.P598T	c.1792C>A	Verified	AAC	CCT	TAT	1	C	A
TCGA-13-1501	MDGA2	ENST00000357362	p.G445G	c.1335C>A	Verified	TTG	GGC	ATC	3	C	A
TCGA-13-1501	MDGA2	ENST00000357362	p.G445G	c.1335C>A	Verified	TTG	GGC	ATC	3	C	A
TCGA-23-1123	MDS2	ENST00000374555	p.S49R	c.147C>A	Verified	CTG	AGC	TGC	3	C	A
TCGA-23-1123	MDS2	ENST00000374555	p.S49R	c.147C>A	Verified	CTG	AGC	TGC	3	C	A
TCGA-23-1118	ME1	ENST00000369705	p.Q488K	c.1462C>A	Verified	CAG	CAA	GTG	1	C	A
TCGA-23-1120	ME1	ENST00000369705	p.P22T	c.64C>A	Unverified	AAC	CCT	CAC	1	C	A
TCGA-29-1785	MED12L	ENST00000474524	p.P1793H	c.5378C>A	Unverified	GTG	CCT	CCT	2	C	A
TCGA-29-1785	MED12L	ENST00000474524	p.P1793H	c.5378C>A	Unverified	GTG	CCT	CCT	2	C	A
TCGA-29-1766	MED13L	ENST00000281928	p.R2075R	c.6223C>A	Unverified	agt	cgg	agc	1	C	A
TCGA-04-1649	MED24	NM_014815.2	p.R385R	c.1155C>A	Unverified	aag	cgc	aaa	3	C	A
TCGA-09-2044	MEPE	ENST00000361056	p.N356K	c.1068C>A	Verified	GGA	AAC	AGA	3	C	A
TCGA-23-1120	METTL17	ENST00000339374	p.A110D	c.329C>A	Verified	CGG	GCT	AGG	2	C	A
TCGA-20-1687	MFSD2A	ENST00000372811	p.T229K	c.686C>A	Unverified	CAT	ACA	CAT	2	C	A
TCGA-13-0924	MGA	XM_031689.7	p.S645R	c.1935C>A	Verified	gta	agc	cct	3	C	A
TCGA-13-0795	MGAM_ENS	ENST00000549489	p.R1285R	c.3853C>A	Verified	GAG	CGG	CAG	1	C	A
TCGA-20-0990	MGAM_ENS	ENST00000549489	p.P1492T	c.4474C>A	Verified	AGA	CCC	ACA	1	C	A
TCGA-04-1638	MGAT3	ENST00000341184	p.H276Q	c.828C>A	Unverified	GAC	CAC	TTC	3	C	A
TCGA-24-1431	MGC15476	ENST00000300875	p.A338A	c.1014C>A	Unverified	TCT	GCC	TCC	3	C	A

TCGA-13-0905	MGC33530	ENST00000402026	p.Y133*	c.399C>A	Verified	AAC	TAC	GGG	3	C	A
TCGA-61-1913	MIA3	ENST00000344922	p.L504I	c.1510C>A	Unverified	AAC	CTC	AAC	1	C	A
TCGA-23-1114	MLL	NM_005933.1	p.R176S	c.526C>A	Unverified	cct	cgt	ggg	1	C	A
TCGA-24-2288	MLL	NM_005933.1	p.S3202R	c.9606C>A	Unverified	tcc	agc	cag	3	C	A
TCGA-23-1114	MLL_ENST0(	ENST00000534358	p.R176S	c.526C>A	Unverified	CCT	CGT	GGG	1	C	A
TCGA-04-1347	MMP7	ENST00000260227	p.L6M	c.16C>A	Verified	gtg	ctg	tgt	1	C	A
TCGA-24-1847	MOAP1	ENST00000298894	p.F168L	c.504C>A	Unverified	GTG	TTC	TCG	3	C	A
TCGA-13-0903	MOCS3	ENST00000244051	p.P45T	c.133C>A	Verified	GTT	CCG	GTG	1	C	A
TCGA-04-1361	MOGAT2	ENST00000198801	p.P6T	c.16C>A	Verified	gcg	ccc	ttg	1	C	A
TCGA-04-1361	MOGAT2	ENST00000198801	p.P6T	c.16C>A	Verified	gcg	ccc	ttg	1	C	A
TCGA-24-2290	MOGAT3	ENST00000223114	p.T339N	c.1016C>A	Verified	CTC	ACC	TTC	2	C	A
TCGA-24-2290	MOGAT3	ENST00000223114	p.T339N	c.1016C>A	Verified	CTC	ACC	TTC	2	C	A
TCGA-29-1691	MRC2	ENST00000303375	p.H1317N	c.3949C>A	Unverified	GAG	CAC	CTG	1	C	A
TCGA-61-1740	MRGPRX1	ENST00000302797	p.S150Y	c.449C>A	Unverified	CTG	TCC	CTG	2	C	A
TCGA-24-1850	MRPL30	ENST00000338148	p.P63H	c.188C>A	Unverified	AAC	CCT	CAT	2	C	A
TCGA-04-1367	MSLNL	ENST00000293892	p.R190S	c.568C>A	Verified	ACC	CGT	GCA	1	C	A
TCGA-09-1674	MST1R	ENST00000296474	p.P1365H	c.4094C>A	Unverified	GGC	CCC	AGC	2	C	A
TCGA-09-1674	MST1R_ENS	ENST00000296474	p.P1365H	c.4094C>A	Unverified	GGC	CCC	AGC	2	C	A
TCGA-24-1604	MTFMT	ENST00000220058	p.R266S	c.796C>A	Verified	TAC	CGT	GCC	1	C	A
TCGA-09-1665	MTMR8	ENST00000374852	p.T147T	c.441C>A	Verified	tgg	acc	ata	3	C	A
TCGA-04-1336	MTNR1A	ENST00000307161	p.F196L	c.588C>A	Verified	CAC	TTC	CTC	3	C	A
TCGA-13-0886	MTUS2_ENS	ENST00000431530	p.P998Q	c.2993C>A	Unverified	GAC	CCG	CAG	2	C	A
TCGA-24-1435	MUC16	ENST00000397910	p.Q6158K	c.18472C>A	Verified	ACA	CAA	ACA	1	C	A
TCGA-24-2035	MUC16	ENST00000397910	p.S1733Y	c.5198C>A	Verified	TCC	TCC	TCT	2	C	A
TCGA-04-1652	MUC16	ENST00000397910	p.T1322T	c.3966C>A	Unverified	CCC	ACC	ACC	3	C	A
TCGA-04-1652	MUC16	ENST00000397910	p.T1322T	c.3966C>A	Unverified	CCC	ACC	ACC	3	C	A
TCGA-24-1435	MUC16_ENS	ENST00000331986	p.Q1791K	c.5371C>A	Verified	GGC	CAA	CTG	1	C	A
TCGA-04-1652	MUC16_ENS	ENST00000397910	p.T1322T	c.3966C>A	Unverified	CCC	ACC	ACC	3	C	A
TCGA-04-1652	MUC16_ENS	ENST00000397910	p.T1322T	c.3966C>A	Unverified	CCC	ACC	ACC	3	C	A
TCGA-13-0755	MUC16_ENS	ENST00000397910	p.C13862*	c.41586C>A	Verified	ATC	TGC	ACC	3	C	A
TCGA-23-1110	MUC17	ENST00000306151	p.S3984Y	c.11951C>A	Verified	ACA	TCT	TTT	2	C	A
TCGA-29-1763	MUC17	ENST00000306151	p.T2784N	c.8351C>A	Unverified	ACC	ACT	TCT	2	C	A
TCGA-30-1891	MUC17	ENST00000306151	p.T3688K	c.11063C>A	Unverified	TGG	ACG	CCT	2	C	A
TCGA-13-0885	MUC17	ENST00000306151	p.T2298T	c.6894C>A	Verified	AGC	ACC	CTT	3	C	A

TCGA-61-1740	MUC17	ENST00000306151	p.L3820L	c.11460C>A	Unverified	GTC	CTC	ATC	3	C	A
TCGA-25-1313	MUC2	ENST00000359061	p.T1571T	c.4713C>A	Unverified	GTG	ACC	CCA	3	C	A
TCGA-25-1313	MUC2_ENST	ENST00000441003	p.T1570T	c.4710C>A	Unverified	GTG	ACC	CCA	3	C	A
TCGA-61-1998	MUC5AC	ENST00000349637	p.Q117K	c.349C>A	Verified	GTC	CAG	CTA	1	C	A
TCGA-04-1342	MUC6	ENST00000421673	p.P1409Q	c.4226C>A	Unverified	TCC	CCG	CCT	2	C	A
TCGA-61-1998	MX1	ENST00000398598	p.L619I	c.1855C>A	Verified	CAG	CTC	CTG	1	C	A
TCGA-29-2427	MYBPC2_EN	ENST00000357701	p.P837T	c.2509C>A	Unverified	GAG	CCA	CCC	1	C	A
TCGA-29-1769	MYBPC2_EN	ENST00000357701	p.T118N	c.353C>A	Unverified	TAC	ACC	GTG	2	C	A
TCGA-24-2289	MYH13	ENST00000252172	p.T1377N	c.4130C>A	Unverified	AGG	ACC	AAA	2	C	A
TCGA-24-2289	MYH13_ENS	ENST00000570743	p.T1377N	c.4130C>A	Unverified	AGG	ACC	AAA	2	C	A
TCGA-13-1497	MYH2	ENST00000245503	p.Q501K	c.1501C>A	Verified	GAG	CAG	GAG	1	C	A
TCGA-10-0930	MYH8	ENST00000403437	p.Q1214K	c.3640C>A	Verified	TTG	CAG	CGG	1	C	A
TCGA-10-0930	MYH8	ENST00000403437	p.Q1214K	c.3640C>A	Verified	TTG	CAG	CGG	1	C	A
TCGA-13-1408	MYL1	ENST00000352451	p.T158T	c.474C>A	Verified	GCC	ACC	CTG	3	C	A
TCGA-09-2051	MYL4	ENST00000393450	p.T68N	c.203C>A	Verified	CCG	ACT	GGA	2	C	A
TCGA-25-2400	MYLK3	SU_caMLCK	p.G74G	c.222C>A	Unverified	ccg	ggc	ccg	3	C	A
TCGA-09-2049	MYO18B	ENST00000335473	p.P2223T	c.6667C>A	Verified	GAA	CCT	GCT	1	C	A
TCGA-61-1740	MYO1B	ENST00000339514	p.L295I	c.883C>A	Unverified	GGT	CTA	GAT	1	C	A
TCGA-61-1740	MYO1B_ENS	ENST00000392318	p.L295I	c.883C>A	Unverified	GGT	CTA	GAT	1	C	A
TCGA-61-2009	MYO5B	ENST00000285039	p.P619T	c.1855C>A	Verified	AGA	CCC	CCC	1	C	A
TCGA-24-1845	MYOCD	ENST00000343344	p.A667D	c.2000C>A	Unverified	GGG	GCC	CAG	2	C	A
TCGA-24-1845	MYOCD_ENS	ENST00000425538	p.A667D	c.2000C>A	Unverified	GGG	GCC	CAG	2	C	A
TCGA-23-1110	MYOM2	ENST00000262113	p.D1317E	c.3951C>A	Verified	CTT	GAC	CTG	3	C	A
TCGA-13-0904	MYOZ1	ENST00000359322	p.P216P	c.648C>A	Verified	CTT	CCC	AAA	3	C	A
TCGA-29-1703	MYT1L	ENST00000399161	p.R245R	c.733C>A	Unverified	GGT	CGG	AAA	1	C	A
TCGA-24-1847	N4BP3	ENST00000274605	p.P207T	c.619C>A	Unverified	AGC	CCC	TTC	1	C	A
TCGA-23-1031	NAA30	ENST00000556492	p.S190S	c.570C>A	Unverified	TCG	TCC	CTG	3	C	A
TCGA-24-1417	NALCN	ENST00000251127	p.P1236T	c.3706C>A	Verified	GAC	CCG	GTG	1	C	A
TCGA-61-1899	NALP6	ENST00000312165	p.P486Q	c.1457C>A	Unverified	CTG	CCG	GGC	2	C	A
TCGA-24-1470	NAPB	ENST00000377026	p.T260N	c.779C>A	Verified	TAC	ACT	GAA	2	C	A
TCGA-24-1604	NAV1	ENST00000367296	p.A745E	c.2234C>A	Unverified	AAG	GCA	GTG	2	C	A
TCGA-24-2024	NCAM2	ENST00000400546	p.Q616K	c.1846C>A	Verified	AAA	CAG	GAC	1	C	A
TCGA-30-1714	NCKAP1L	ENST00000293373	p.N64K	c.192C>A	Unverified	CCC	AAC	ATA	3	C	A
TCGA-30-1718	NCOA6	ENST00000374796	p.P988T	c.2962C>A	Unverified	CCT	CCT	CAA	1	C	A

TCGA-13-0900	NCOR2	NM_006312.2	p.Y471*	c.1413C>A	Verified	tac	tac	ctg	3	C	A
TCGA-09-2049	NDRG3	ENST00000349004	p.T348N	c.1043C>A	Verified	GTC	ACC	AGC	2	C	A
TCGA-24-2289	NDUFB11	ENST00000276062	p.P49T	c.145C>A	Unverified	ccc	cca	gaa	1	C	A
TCGA-25-1326	NEB	ENST00000409198	p.I2779I	c.8337C>A	Verified	CCT	ATC	AAG	3	C	A
TCGA-04-1331	NEDD4	ENST00000338963	p.S161Y	c.482C>A	Verified	GGG	TCT	TAC	2	C	A
TCGA-13-0761	NEDD4L	ENST00000382850	p.T63K	c.188C>A	Unverified	CAG	ACA	AAA	2	C	A
TCGA-13-0761	NEDD4L	ENST00000382850	p.T63K	c.188C>A	Unverified	CAG	ACA	AAA	2	C	A
TCGA-13-0883	NES	ENST00000368223	p.I278I	c.834C>A	Verified	CAG	ATC	GCT	3	C	A
TCGA-24-0975	NFATC3_ENS	ENST00000346183	p.L1075L	c.3225C>A	Unverified	GGG	CTC	TAA	3	C	A
TCGA-25-2400	NFATC4	ENST00000250373	p.P62H	c.185C>A	Verified	ATT	CCC	CGA	2	C	A
TCGA-29-1768	NFXL1	ENST00000381538	p.L198I	c.592C>A	Unverified	TTT	CTT	GTA	1	C	A
TCGA-04-1652	NHLRC1	ENST00000340650	p.A322A	c.966C>A	Unverified	ACT	GCC	TCC	3	C	A
TCGA-04-1652	NHLRC1	ENST00000340650	p.A322A	c.966C>A	Unverified	ACT	GCC	TCC	3	C	A
TCGA-13-1496	NIPBL	ENST00000282516	p.S861*	c.2582C>A	Verified	agt	tca	aaa	2	C	A
TCGA-13-0883	NISCH	ENST00000345716	p.L1232M	c.3694C>A	Verified	GAC	CTG	CAG	1	C	A
TCGA-25-2400	NKG7	ENST00000221978	p.L144I	c.430C>A	Unverified	TTG	CTC	TGT	1	C	A
TCGA-13-0760	NLK	NM_016231	p.P223T	c.667C>A	Verified	caa	cca	ctc	1	C	A
TCGA-09-2056	NLRC4	ENST00000360906	p.H56Q	c.168C>A	Verified	att	cac	atg	3	C	A
TCGA-25-2401	NLRP10	ENST00000328600	p.L83M	c.247C>A	Verified	AAC	CTG	TTG	1	C	A
TCGA-29-1763	NLRP10	ENST00000328600	p.R465S	c.1393C>A	Unverified	TTC	CGC	CAC	1	C	A
TCGA-13-0886	NLRP14	ENST00000299481	p.A398D	c.1193C>A	Verified	ACA	GCT	CTG	2	C	A
TCGA-29-1766	NLRP4_ENST	ENST00000301295	p.S589Y	c.1766C>A	Unverified	GTT	TCT	GCC	2	C	A
TCGA-24-2288	NME1-NME2	ENST00000393193	p.H162N	c.484C>A	Unverified	GAA	CAC	CTG	1	C	A
TCGA-24-2288	NME2	ENST00000514264	p.H47N	c.139C>A	Unverified	gaa	cac	ctg	1	C	A
TCGA-61-1914	NOD2	ENST00000300589	p.L145I	c.433C>A	Unverified	agg	ctc	cac	1	C	A
TCGA-29-1699	NOL4	ENST00000261592	p.T119K	c.356C>A	Unverified	GAA	ACG	GGG	2	C	A
TCGA-29-1698	NOP2	ENST00000399466	p.S655*	c.1964C>A	Unverified	GAC	TCA	GAA	2	C	A
TCGA-61-1998	NOS1	ENST00000317775	p.P1186T	c.3556C>A	Verified	TCC	CCA	GAC	1	C	A
TCGA-24-1616	NOTCH2NL	ENST00000362074	p.T39N	c.116C>A	Verified	GGG	ACT	TGT	2	C	A
TCGA-24-2024	NOTCH4	ENST00000375023	p.Q1809K	c.5425C>A	Verified	cac	caa	cgt	1	C	A
TCGA-29-1711	NOTCH4	ENST00000375023	p.C859*	c.2577C>A	Unverified	cac	tgc	ctc	3	C	A
TCGA-29-1711	NOTCH4	ENST00000375023	p.C859*	c.2577C>A	Unverified	cac	tgc	ctc	3	C	A
TCGA-29-1761	NOVA1	ENST00000539517	p.A276D	c.827C>A	Unverified	act	gct	gaa	2	C	A
TCGA-23-1122	NOX1	NM_013954.1	p.R130R	c.388C>A	Verified	agc	cga	cag	1	C	A

TCGA-29-1784	NOXRED1	ENST00000380835	p.S358Y	c.1073C>A	Unverified	CCA	TCC	CAA	2	C	A
TCGA-30-1891	NPC1L1	ENST00000289547	p.I218I	c.654C>A	Unverified	GAC	ATC	ACC	3	C	A
TCGA-29-1695	NPFFR2	ENST00000308744	p.I149I	c.447C>A	Unverified	GCA	ATC	TTC	3	C	A
TCGA-13-1498	NPR1	ENST00000368680	p.G646G	c.1938C>A	Verified	aag	ggc	atg	3	C	A
TCGA-13-1498	NR_002217_	ENST00000338780	p.D124E	c.372C>A	Verified	CCA	GAC	ACG	3	C	A
TCGA-61-2113	NR1D2	ENST00000312521	p.L555I	c.1663C>A	Unverified	CTG	CTT	CTA	1	C	A
TCGA-36-1577	NR1H3	ENST00000441012	p.R156S	c.466C>A	Unverified	CTT	CGC	AAA	1	C	A
TCGA-23-1022	NRAP	ENST00000359988	p.P1220H	c.3659C>A	Verified	ACT	CCC	AAC	2	C	A
TCGA-24-1563	NRAP	ENST00000359988	p.N456K	c.1368C>A	Verified	TAC	AAC	TAC	3	C	A
TCGA-29-1691	NRBF2	ENST00000277746	p.T242N	c.725C>A	Unverified	TCA	ACC	TGG	2	C	A
TCGA-24-2288	NRG3	ENST00000372141	p.S147S	c.441C>A	Unverified	GCC	TCC	TCC	3	C	A
TCGA-24-1423	NRXN1_ENS	ENST00000406316	p.T766T	c.2298C>A	Verified	GAC	ACC	CTC	3	C	A
TCGA-30-1714	NRXN2	ENST00000377551	p.L670I	c.2008C>A	Unverified	gac	ctc	cgg	1	C	A
TCGA-29-1775	NRXN2	ENST00000377551	p.S1020Y	c.3059C>A	Unverified	gac	tcc	cgc	2	C	A
TCGA-24-1435	NRXN2	ENST00000377551	p.P1329P	c.3987C>A	Verified	gac	ccc	aat	3	C	A
TCGA-09-1665	NTM	ENST00000374786	p.P112H	c.335C>A	Verified	GGC	CCT	TAC	2	C	A
TCGA-24-2280	NTRK1	ENST00000524377	p.D537E	c.1611C>A	Unverified	CAG	GAC	AAG	3	C	A
TCGA-24-2280	NTRK1_ENS	ENST00000392302	p.D501E	c.1503C>A	Unverified	CAG	GAC	AAG	3	C	A
TCGA-24-1845	NUP133	ENST00000261396	p.A949D	c.2846C>A	Unverified	aag	gct	cat	2	C	A
TCGA-30-1855	NUP160	ENST00000378460	p.R1116R	c.3346C>A	Unverified	CTC	CGG	GGA	1	C	A
TCGA-09-2056	NXF2B	ENST00000372750	p.Q624K	c.1870C>A	Verified	AAG	CAA	ATC	1	C	A
TCGA-29-1761	O5AK3_HUN	ENST00000326876	p.L130I	c.388C>A	Unverified	CCC	CTT	CGC	1	C	A
TCGA-23-1031	OBSCN	SU_OBSCN.1	p.A5650A	c.16950C>A	Unverified	gag	gcc	cgc	3	C	A
TCGA-29-1691	OC90	XM_070277.6	p.F69L	c.207C>A	Unverified	cac	ttc	acc	3	C	A
TCGA-24-1846	OC90_ENST(	ENST00000262283	p.T394T	c.1182C>A	Unverified	ACA	ACC	ATC	3	C	A
TCGA-29-1691	OC90_ENST(	ENST00000262283	p.F259L	c.777C>A	Unverified	CAC	TTC	ACC	3	C	A
TCGA-13-0923	ODZ2	ENST00000388903	p.S1584Y	c.4751C>A	Verified	TAT	GCT	AAT	2	C	A
TCGA-29-1784	ODZ2_ENST(	ENST00000518659	p.S1975Y	c.5924C>A	Unverified	ATG	TCC	ACA	2	C	A
TCGA-29-1784	ODZ2_ENST(	ENST00000519204	p.S1854Y	c.5561C>A	Unverified	ATG	TCC	ACA	2	C	A
TCGA-61-2008	OGT	ENST00000373701	p.T444K	c.1331C>A	Verified	cgc	acg	gct	2	C	A
TCGA-61-2008	OGT_ENST0	ENST00000373719	p.T454K	c.1361C>A	Verified	CGC	ACG	GCT	2	C	A
TCGA-61-1900	OLFM4	ENST00000219022	p.T354N	c.1061C>A	Unverified	CTG	ACC	ACC	2	C	A
TCGA-29-1784	OLFM4	ENST00000219022	p.T448T	c.1344C>A	Unverified	AAC	ACC	AGA	3	C	A
TCGA-24-2280	OLIG3	ENST00000367734	p.Q33K	c.97C>A	Unverified	CAC	CAG	GAG	1	C	A

TCGA-04-1361	OMD	ENST00000375550	p.P232H	c.695C>A	Verified	TTG	CCT	TCT	2	C	A
TCGA-04-1361	OMD	ENST00000375550	p.P232H	c.695C>A	Verified	TTG	CCT	TCT	2	C	A
TCGA-61-1907	OPCML	ENST00000331898	p.T96N	c.287C>A	Unverified	CCA	ACC	CAG	2	C	A
TCGA-61-1907	OPCML_ENS	ENST00000524381	p.T89N	c.266C>A	Unverified	CCA	ACC	CAG	2	C	A
TCGA-29-1702	OPN1LW	ENST00000369951	p.Y284*	c.852C>A	Unverified	CCC	TAC	ACC	3	C	A
TCGA-29-1702	OPN1LW_EN	ENST00000369951	p.Y284*	c.852C>A	Unverified	CCC	TAC	ACC	3	C	A
TCGA-24-1604	OR10A6	ENST00000309838	p.T181N	c.542C>A	Verified	GAA	ACC	CCA	2	C	A
TCGA-36-1577	OR10H2	ENST00000305899	p.P263P	c.789C>A	Verified	AAG	CCC	AAA	3	C	A
TCGA-61-2008	OR10J3	ENST00000332217	p.A77D	c.230C>A	Verified	GTG	GCC	ATC	2	C	A
TCGA-04-1347	OR10X1	ENST00000368150	p.T54N	c.161C>A	Verified	CTC	ACC	CTT	2	C	A
TCGA-61-1900	OR14I1	ENST00000342623	p.C150*	c.450C>A	Unverified	AGC	TGC	TTT	3	C	A
TCGA-09-1674	OR1L4	ENST00000259466	p.L116M	c.346C>A	Unverified	CTG	CTG	GCC	1	C	A
TCGA-24-1843	OR2G6	ENST00000343414	p.Q20K	c.58C>A	Unverified	GAT	CAG	CCT	1	C	A
TCGA-30-1718	OR2K2	ENST00000302681	p.G41G	c.123C>A	Unverified	TTG	GGC	AAC	3	C	A
TCGA-30-1718	OR2K2_ENS	ENST00000374428	p.G70G	c.210C>A	Unverified	TTG	GGC	AAC	3	C	A
TCGA-23-1809	OR2L3	ENST00000359959	p.T279N	c.836C>A	Unverified	ACC	ACC	CTC	2	C	A
TCGA-09-2049	OR2M3	ENST00000456743	p.T133N	c.398C>A	Verified	TAC	ACC	AAT	2	C	A
TCGA-61-1900	OR2T3	ENST00000359594	p.L32I	c.94C>A	Unverified	CTC	CTC	TAC	1	C	A
TCGA-13-0903	OR2T34	ENST00000328782	p.L60I	c.178C>A	Verified	CGC	CTC	CAC	1	C	A
TCGA-24-2290	OR2W1	ENST00000377175	p.R122S	c.364C>A	Verified	GAT	CGT	TTT	1	C	A
TCGA-24-2290	OR2W1	ENST00000377175	p.R122S	c.364C>A	Verified	GAT	CGT	TTT	1	C	A
TCGA-13-0890	OR2W5	ENST00000366492	p.T110T	c.330C>A	Verified	TCC	ACC	GAG	3	C	A
TCGA-29-1781	OR4F6	ENST00000328882	p.A78D	c.233C>A	Unverified	ACA	GCT	CCC	2	C	A
TCGA-30-1855	OR4K1	ENST00000285600	p.P284H	c.851C>A	Unverified	AAC	CCC	ATC	2	C	A
TCGA-29-1775	OR4N2	ENST00000315947	p.T134N	c.401C>A	Unverified	CCT	ACT	GTC	2	C	A
TCGA-24-1469	OR4N4	ENST00000328795	p.S133*	c.398C>A	Verified	TGT	TCA	ACT	2	C	A
TCGA-24-2290	OR4N4	ENST00000328795	p.I31I	c.93C>A	Verified	CTG	ATC	TTA	3	C	A
TCGA-24-2290	OR4N4	ENST00000328795	p.I31I	c.93C>A	Verified	CTG	ATC	TTA	3	C	A
TCGA-13-0884	OR4N5	ENST00000333629	p.P284H	c.851C>A	Verified	AAC	CCT	GTT	2	C	A
TCGA-61-1906	OR51B6	ENST00000380219	p.H242N	c.724C>A	Unverified	TCT	CAT	ATC	1	C	A
TCGA-61-1906	OR51B6	ENST00000380219	p.H242N	c.724C>A	Unverified	TCT	CAT	ATC	1	C	A
TCGA-24-2280	OR52A4	ENST00000380369	p.T146N	c.437C>A	Unverified	GTC	ACT	TAT	2	C	A
TCGA-24-1843	OR52E8	ENST00000537935	p.L165M	c.493C>A	Unverified	CCA	CTG	GTG	1	C	A
TCGA-24-1431	OR56A1	ENST00000316650	p.P8T	c.22C>A	Verified	TCA	CCC	AGC	1	C	A

TCGA-29-1763	OR5B17	ENST00000357377	p.D50E	c.150C>A	Unverified	CTG	GAC	TCT	3	C	A
TCGA-13-1505	OR5D13	ENST00000361760	p.A3E	c.8C>A	Verified	ATG	GCA	TCT	2	C	A
TCGA-13-0807	OR5D14	ENST00000335605	p.P81T	c.241C>A	Verified	ACT	CCC	AAG	1	C	A
TCGA-13-1497	OR5H15	ENST00000356526	p.L255I	c.763C>A	Verified	CCC	CTT	CTC	1	C	A
TCGA-29-1763	OR5K4	ENST00000354924	p.L104L	c.312C>A	Unverified	TTT	CTC	TGT	3	C	A
TCGA-09-2050	OR5P2	ENST00000329434	p.P75H	c.224C>A	Verified	ACA	CCC	AAC	2	C	A
TCGA-61-1998	OR5T2	ENST00000313264	p.T357N	c.1070C>A	Verified	CAT	ACT	AAA	2	C	A
TCGA-13-1497	OR6A2	ENST00000332601	p.P215Q	c.644C>A	Verified	GGG	CCA	CTC	2	C	A
TCGA-24-2280	OR6Q1	ENST00000302622	p.T109T	c.327C>A	Unverified	TTC	ACC	TTT	3	C	A
TCGA-13-0893	OR6V1	ENST00000418316	p.T244K	c.731C>A	Verified	CTC	ACA	CTG	2	C	A
TCGA-29-1761	OR7E5P	ENST00000360245	p.P46T	c.136C>A	Unverified	GTC	CCC	AAG	1	C	A
TCGA-13-0887	OR7G1	ENST00000293614	p.V281V	c.843C>A	Verified	GTG	GTC	CCT	3	C	A
TCGA-24-1843	OR8A1	ENST00000284287	p.I168I	c.504C>A	Unverified	GCC	ATC	GGA	3	C	A
TCGA-61-1914	OR8B12	ENST00000306842	p.H226Q	c.678C>A	Unverified	CTA	CAC	AAC	3	C	A
TCGA-13-0919	OR8B2	ENST00000375013	p.V37V	c.111C>A	Unverified	ATT	GTC	ACC	3	C	A
TCGA-09-2044	OR8H1	ENST00000313022	p.T223T	c.669C>A	Verified	TCT	ACC	ATC	3	C	A
TCGA-29-1699	OR8I2	ENST00000302124	p.Q234K	c.700C>A	Unverified	AGG	CAG	AAG	1	C	A
TCGA-23-2077	OR8K3	ENST00000312711	p.I20I	c.60C>A	Verified	GAT	ATC	GCT	3	C	A
TCGA-13-0884	OR9Q2	ENST00000311591	p.T105N	c.314C>A	Verified	TTC	ACC	TTC	2	C	A
TCGA-13-0916	OSR1	ENST00000272223	p.T261N	c.782C>A	Verified	AAA	ACC	TCC	2	C	A
TCGA-24-1846	OTOL1	ENST00000327928	p.Q440K	c.1318C>A	Unverified	GAC	CAA	GTC	1	C	A
TCGA-30-1857	OXER1	ENST00000378661	p.S53Y	c.158C>A	Unverified	CTC	TCC	TCC	2	C	A
TCGA-29-2427	P2RX2	ENST00000343948	p.G446G	c.1338C>A	Unverified	TCA	GGC	CAG	3	C	A
TCGA-61-1906	P2RY1	ENST00000305097	p.I118I	c.354C>A	Unverified	TGG	ATC	TTC	3	C	A
TCGA-61-1906	P2RY1	ENST00000305097	p.I118I	c.354C>A	Unverified	TGG	ATC	TTC	3	C	A
TCGA-24-1469	P2RY10	ENST00000544091	p.Q233K	c.697C>A	Verified	ttc	caa	ggg	1	C	A
TCGA-24-1844	PABPC5	ENST00000312600	p.A158D	c.473C>A	Unverified	gct	gcc	aat	2	C	A
TCGA-29-1776	PAG1	ENST00000220597	p.A376E	c.1127C>A	Unverified	CCA	GCA	GGG	2	C	A
TCGA-13-1488	PALM2	ENST00000374531	p.S129Y	c.386C>A	Verified	TTC	TCC	AGT	2	C	A
TCGA-13-1488	PALM2-AKA	ENST00000374530	p.S127Y	c.380C>A	Verified	TTC	TCC	AGT	2	C	A
TCGA-10-0930	PARP9	ENST00000360356	p.I33I	c.99C>A	Verified	CAG	ATC	TTT	3	C	A
TCGA-10-0930	PARP9	ENST00000360356	p.I33I	c.99C>A	Verified	CAG	ATC	TTT	3	C	A
TCGA-61-1900	PASD1	ENST00000370357	p.L532M	c.1594C>A	Unverified	AAG	CTG	CAG	1	C	A
TCGA-61-1900	PASD1_ENS	ENST00000370357	p.L532M	c.1594C>A	Unverified	AAG	CTG	CAG	1	C	A

TCGA-61-2102	PASK	ENST00000234040	p.T1161N	c.3482C>A	Verified	TAT	ACT	TTT	2	C	A
TCGA-29-1763	PCCA	ENST00000376285	p.R390S	c.1168C>A	Unverified	att	cgc	atc	1	C	A
TCGA-04-1342	PCDH17	ENST00000377918	p.T837N	c.2510C>A	Unverified	TTC	ACC	GGA	2	C	A
TCGA-24-1470	PCDH20	ENST00000358674	p.R572R	c.1714C>A	Verified	GAC	CGA	GAA	1	C	A
TCGA-09-2056	PCDHB2	ENST00000194155	p.T152N	c.455C>A	Verified	GGA	ACT	ACT	2	C	A
TCGA-23-1114	PCDHB7	ENST00000231137	p.Q10K	c.28C>A	Unverified	GTG	CAG	AAA	1	C	A
TCGA-29-1777	PCDHG_clus	ENST00000394576	p.N127K	c.381C>A	Unverified	ATT	AAC	GAT	3	C	A
TCGA-61-1737	PCDHGA12	ENST00000252085	p.N545K	c.1635C>A	Unverified	AGC	AAC	GTG	3	C	A
TCGA-61-1737	PCDHGA12_	ENST00000252085	p.N545K	c.1635C>A	Unverified	AGC	AAC	GTG	3	C	A
TCGA-29-1777	PCDHGA2	ENST00000305759	p.N127K	c.381C>A	Unverified	ATT	AAC	GAT	3	C	A
TCGA-13-1498	PCDHGA5	ENST00000518069	p.L632M	c.1894C>A	Verified	GCC	CTG	CTG	1	C	A
TCGA-04-1361	PCDHGC5	ENST00000252087	p.P307T	c.919C>A	Verified	GGT	CCC	ATA	1	C	A
TCGA-04-1361	PCDHGC5	ENST00000252087	p.P307T	c.919C>A	Verified	GGT	CCC	ATA	1	C	A
TCGA-04-1361	PCDHGC5_E	ENST00000252087	p.P307T	c.919C>A	Verified	GGT	CCC	ATA	1	C	A
TCGA-04-1361	PCDHGC5_E	ENST00000252087	p.P307T	c.919C>A	Verified	GGT	CCC	ATA	1	C	A
TCGA-13-1488	PCLO	ENST00000333891	p.S770*	c.2309C>A	Verified	TCA	TCA	TCA	2	C	A
TCGA-13-0760	PCSK9	ENST00000302118	p.P404Q	c.1211C>A	Verified	GAG	CCG	GAG	2	C	A
TCGA-04-1361	PDCD8	ENST00000287295	p.P488P	c.1464C>A	Verified	ggc	ccc	gat	3	C	A
TCGA-04-1361	PDCD8	ENST00000287295	p.P488P	c.1464C>A	Verified	ggc	ccc	gat	3	C	A
TCGA-24-2280	PDE11A	ENST00000286063	p.Q360K	c.1078C>A	Unverified	ATG	CAG	ATG	1	C	A
TCGA-61-1907	PDE11A	ENST00000286063	p.H199N	c.595C>A	Unverified	AAG	CAT	AAT	1	C	A
TCGA-24-1469	PDE11A	ENST00000286063	p.A918D	c.2753C>A	Verified	ACT	GCC	TCA	2	C	A
TCGA-04-1655	PDE11A	ENST00000286063	p.V407V	c.1221C>A	Unverified	ATT	GTC	AAG	3	C	A
TCGA-04-1655	PDE11A_EN:	ENST00000358450	p.V157V	c.471C>A	Unverified	ATT	GTC	AAG	3	C	A
TCGA-04-1362	PDE2A	ENST00000334456	p.Q307K	c.919C>A	Verified	ATC	CAG	CTG	1	C	A
TCGA-09-1665	PDE8B	ENST00000264917	p.G147G	c.441C>A	Unverified	GAT	GGC	TTC	3	C	A
TCGA-24-1844	PDK3	ENST00000379162	p.D287E	c.861C>A	Unverified	AGT	GAC	CTA	3	C	A
TCGA-24-1844	PDK3_ENST(	ENST00000441463	p.D287E	c.861C>A	Unverified	AGT	GAC	CTA	3	C	A
TCGA-29-1768	PDLIM3	ENST00000284770	p.H238N	c.712C>A	Unverified	CTC	CAC	GAC	1	C	A
TCGA-29-1768	PDLIM3_EN:	ENST00000284771	p.H190N	c.568C>A	Unverified	CTC	CAC	GAC	1	C	A
TCGA-24-2035	PDLIM4	ENST00000253754	p.T114K	c.341C>A	Unverified	CCA	ACA	ACC	2	C	A
TCGA-13-0916	PDZRN4	ENST00000539469	p.G157G	c.471C>A	Verified	CTG	GGC	CTG	3	C	A
TCGA-13-1498	PELP1	ENST00000456564	p.P544T	c.1630C>A	Verified	GCC	CCC	AAG	1	C	A
TCGA-13-0761	PEX16	ENST00000241041	p.L135L	c.405C>A	Verified	GGC	CTC	CAG	3	C	A

TCGA-13-0761	PEX16	ENST00000241041	p.L135L	c.405C>A	Verified	GGC	CTC	CAG	3	C	A
TCGA-10-0930	PGA3	ENST00000325558	p.P67P	c.201C>A	Verified	CAG	CCC	CTG	3	C	A
TCGA-10-0930	PGA3	ENST00000325558	p.P67P	c.201C>A	Verified	CAG	CCC	CTG	3	C	A
TCGA-30-1856	PGAP1	ENST00000354764	p.Q387K	c.1159C>A	Unverified	TGT	CAG	AGC	1	C	A
TCGA-61-1900	PGC	ENST00000373025	p.Q246K	c.736C>A	Unverified	ACC	CAG	GAA	1	C	A
TCGA-23-1031	PGLYRP2	ENST00000340880	p.P567Q	c.1700C>A	Verified	CCC	CCA	AGG	2	C	A
TCGA-04-1331	PGLYRP3	ENST00000290722	p.S229Y	c.686C>A	Verified	CAG	TCC	TTT	2	C	A
TCGA-24-1845	PHF3	ENST00000262043	p.S341Y	c.1022C>A	Unverified	gga	tct	tct	2	C	A
TCGA-24-1844	PHKA1	ENST00000373542	p.P1026T	c.3076C>A	Unverified	TCA	CCT	GGA	1	C	A
TCGA-24-2280	PHLDB2	ENST00000412622	p.P885P	c.2655C>A	Unverified	CTG	CCC	CTA	3	C	A
TCGA-10-0930	PHOX2B	ENST00000226382	p.S7Y	c.20C>A	Verified	TAT	TCT	TAC	2	C	A
TCGA-10-0930	PHOX2B	ENST00000226382	p.S7Y	c.20C>A	Verified	TAT	TCT	TAC	2	C	A
TCGA-29-1701	PI15	ENST00000260113	p.S30*	c.89C>A	Unverified	GAC	TCA	TCC	2	C	A
TCGA-13-1512	PIK3CA	NM_006218.1	p.I800I	c.2400C>A	Verified	atc	atc	ttt	3	C	A
TCGA-09-1674	PIK3CB	ENST00000477593	p.A875D	c.2624C>A	Unverified	gca	gcc	ttc	2	C	A
TCGA-61-1737	PIK3CD	ENST00000377346	p.N464K	c.1392C>A	Unverified	CCC	AAC	ACG	3	C	A
TCGA-13-1509	PIK3CG	ENST00000440650	p.L379I	c.1135C>A	Verified	GAC	CTC	ACA	1	C	A
TCGA-29-1762	PIK3R1	ENST00000521381	p.S565R	c.1695C>A	Unverified	aac	agc	att	3	C	A
TCGA-29-1762	PIK3R1_ENS	ENST00000320694	p.S265R	c.795C>A	Unverified	AAC	AGC	ATT	3	C	A
TCGA-29-1762	PIK3R1_ENS	ENST00000336483	p.S295R	c.885C>A	Unverified	AAC	AGC	ATT	3	C	A
TCGA-23-1021	PIKFYVE	ENST00000264380	p.P919H	c.2756C>A	Verified	CCT	CCT	GAG	2	C	A
TCGA-23-1029	PIKFYVE	ENST00000264380	p.P747H	c.2240C>A	Unverified	CGA	CCC	ACC	2	C	A
TCGA-04-1651	PINK1	ENST00000321556	p.R312R	c.934C>A	Unverified	ggc	cgg	acg	1	C	A
TCGA-04-1651	PINK1	ENST00000321556	p.R312R	c.934C>A	Unverified	ggc	cgg	acg	1	C	A
TCGA-04-1362	PKD1L1	ENST00000289672	p.P780T	c.2338C>A	Verified	GCC	CCT	GTC	1	C	A
TCGA-13-0923	PKD1L1	ENST00000289672	p.S2504*	c.7511C>A	Verified	CCC	TCA	TCC	2	C	A
TCGA-13-0791	PKD2L1	ENST00000318222	p.L391M	c.1171C>A	Verified	GAC	CTG	GTG	1	C	A
TCGA-29-1693	PKD2L1	ENST00000318222	p.V307V	c.921C>A	Unverified	TCA	GTC	TAC	3	C	A
TCGA-29-1695	PKD2L2	ENST00000290431	p.Y19*	c.57C>A	Unverified	CAT	TAC	AGA	3	C	A
TCGA-24-1417	PLA2G4C	ENST00000413144	p.T123T	c.369C>A	Verified	AAA	ACC	ATC	3	C	A
TCGA-25-1313	PLA2G4F	ENST00000382396	p.S308R	c.924C>A	Verified	ATG	AGC	TCC	3	C	A
TCGA-61-1904	PLEK	ENST00000234313	p.V161V	c.483C>A	Unverified	TGC	GTC	ATT	3	C	A
TCGA-29-1785	PLEKHA7	ENST00000355661	p.S363Y	c.1088C>A	Unverified	AGG	TCT	CCG	2	C	A
TCGA-29-1785	PLEKHA7	ENST00000355661	p.S363Y	c.1088C>A	Unverified	AGG	TCT	CCG	2	C	A

TCGA-24-2288	PLEKHG1	ENST00000358517	p.P804T	c.2410C>A	Unverified	ACT	CCC	GAT	1	C	A
TCGA-23-1029	PLEKHH2	ENST00000282406	p.S1102*	c.3305C>A	Unverified	CCC	TCA	AGG	2	C	A
TCGA-23-1124	PLEKHO2	ENST00000323544	p.P402P	c.1206C>A	Verified	CAT	CCC	TTG	3	C	A
TCGA-29-1702	PLG	ENST00000308192	p.D328E	c.984C>A	Unverified	CCT	GAC	GGA	3	C	A
TCGA-61-1900	PLK4	ENST00000270861	p.S718Y	c.2153C>A	Unverified	AAT	TCT	CCT	2	C	A
TCGA-24-1844	PLXNA2	ENST00000367033	p.L1681I	c.5041C>A	Unverified	CGG	CTA	CTG	1	C	A
TCGA-24-1422	PNMA1	ENST00000316836	p.A313D	c.938C>A	Unverified	GGG	GCT	GGG	2	C	A
TCGA-13-0916	POGK	ENST00000367875	p.Y511*	c.1533C>A	Unverified	GTC	TAC	AAG	3	C	A
TCGA-29-1777	POLD2	ENST00000406581	p.L118L	c.354C>A	Unverified	CTG	CTC	CCC	3	C	A
TCGA-29-1777	POLD2_ENST	ENST00000406581	p.L118L	c.354C>A	Unverified	CTG	CTC	CCC	3	C	A
TCGA-24-1104	POLE	ENST00000320574	p.T2245N	c.6734C>A	Verified	CTC	ACC	ATC	2	C	A
TCGA-13-0884	POLI	NM_007195.1	p.T65T	c.195C>A	Verified	gtt	acc	tgc	3	C	A
TCGA-13-1481	POTEA	ENST00000354247	p.A135D	c.404C>A	Verified	ACA	GCT	CTG	2	C	A
TCGA-13-1488	POTEJ	ENST00000409602	p.Q297K	c.889C>A	Unverified	TCT	CAA	GAT	1	C	A
TCGA-13-1488	POTEJ_ENST	ENST00000409602_v68	p.Q297K	c.889C>A	Unverified	TCT	CAA	GAT	1	C	A
TCGA-24-0975	POU2F2	ENST00000389341	p.S433R	c.1299C>A	Unverified	ccc	agc	cct	3	C	A
TCGA-61-1910	PPAPDC3	ENST00000372264	p.L267L	c.801C>A	Unverified	ATG	CTC	ATC	3	C	A
TCGA-61-1725	PPARGC1B	ENST00000309241	p.L848I	c.2542C>A	Unverified	CAG	CTC	TGT	1	C	A
TCGA-24-2288	PPARGC1B	ENST00000309241	p.D51E	c.153C>A	Unverified	AGC	GAC	TTT	3	C	A
TCGA-13-0904	PPEF2	ENST00000286719	p.C377*	c.1131C>A	Verified	ccc	tgc	agc	3	C	A
TCGA-23-1117	PPP1R10	ENST00000376511	p.S327R	c.981C>A	Verified	acg	agc	aca	3	C	A
TCGA-23-1117	PPP1R10	ENST00000376511	p.S327R	c.981C>A	Verified	acg	agc	aca	3	C	A
TCGA-61-1910	PPP1R3A	NM_002711.2	p.A67D	c.200C>A	Unverified	ttt	gct	gat	2	C	A
TCGA-61-1725	PPP2R5E	ENST00000337537	p.D78E	c.234C>A	Unverified	atg	gac	acg	3	C	A
TCGA-23-1029	PRAM1	ENST00000423345	p.P110H	c.329C>A	Unverified	CCG	CCT	GAG	2	C	A
TCGA-24-2280	PRAMEF10	ENST00000235347	p.A240D	c.719C>A	Unverified	TTA	GCC	TTC	2	C	A
TCGA-04-1336	PRAMEF12	ENST00000357726	p.L331M	c.991C>A	Verified	ACA	CTG	ACC	1	C	A
TCGA-23-1114	PRDM5	ENST00000264808	p.R77R	c.229C>A	Unverified	CCA	CGG	CAC	1	C	A
TCGA-23-1117	PRICKLE2	ENST00000295902	p.L624I	c.1870C>A	Verified	CAG	CTC	AGC	1	C	A
TCGA-23-1117	PRICKLE2	ENST00000295902	p.L624I	c.1870C>A	Verified	CAG	CTC	AGC	1	C	A
TCGA-29-1763	PRKD1	ENST00000331968	p.T833T	c.2499C>A	Unverified	AAG	ACC	TTG	3	C	A
TCGA-29-1763	PRKD1_ENST	ENST00000331968	p.T833T	c.2499C>A	Unverified	AAG	ACC	TTG	3	C	A
TCGA-29-1781	PRLHR	ENST00000369169	p.P49T	c.145C>A	Unverified	ACG	CCC	TTC	1	C	A
TCGA-24-1422	PRLHR	ENST00000369169	p.A288D	c.863C>A	Unverified	TTC	GCC	GTC	2	C	A

TCGA-24-1103	PRLR	ENST00000382002	p.L117I	c.349C>A	Verified	GAA	CTT	TAT	1	C	A
TCGA-13-0913	PRODH2	ENST00000301175	p.N207K	c.621C>A	Verified	GGG	AAC	CTC	3	C	A
TCGA-13-0755	PROM1	ENST00000447510	p.N272K	c.816C>A	Verified	GAG	AAC	ATG	3	C	A
TCGA-13-1499	PROM1	ENST00000447510	p.H681Q	c.2043C>A	Verified	ATT	CAC	CAG	3	C	A
TCGA-13-1408	PRR16	ENST00000379551	p.P84Q	c.251C>A	Verified	CCC	CCA	GCA	2	C	A
TCGA-29-1691	PRR21	ENST00000408934	p.L92I	c.274C>A	Unverified	CCT	CTT	CAC	1	C	A
TCGA-24-1850	PRR23C	ENST00000413199	p.F219L	c.657C>A	Unverified	GAA	TTC	CAT	3	C	A
TCGA-61-2008	PRRC2B	ENST00000357304	p.A54D	c.161C>A	Verified	GCA	GCC	CGG	2	C	A
TCGA-23-1029	PRUNE	ENST00000271620	p.C274*	c.822C>A	Unverified	TTC	TGC	CAG	3	C	A
TCGA-29-1691	PSD3_ENST(	ENST00000327040	p.D450E	c.1350C>A	Unverified	TTG	GAC	AAC	3	C	A
TCGA-04-1336	PSKH2	ENST00000276616	p.T252K	c.755C>A	Verified	ATC	ACA	TAT	2	C	A
TCGA-24-1843	PSMF1	ENST00000335877	p.P245P	c.735C>A	Unverified	GAC	CCC	TTT	3	C	A
TCGA-04-1652	PSTK	ENST00000368887	p.R16R	c.46C>A	Unverified	CCG	CGG	AAA	1	C	A
TCGA-04-1652	PSTK	ENST00000368887	p.R16R	c.46C>A	Unverified	CCG	CGG	AAA	1	C	A
TCGA-24-1845	PTCHD3	ENST00000438700	p.R409R	c.1225C>A	Unverified	ATA	CGA	AAC	1	C	A
TCGA-13-0887	PTCRA	ENST00000304672	p.P54T	c.160C>A	Verified	GCA	CCC	CCT	1	C	A
TCGA-24-1431	PTDSS1	ENST00000517309	p.H411Q	c.1233C>A	Verified	GGT	CAC	CGA	3	C	A
TCGA-13-1496	PTGDS	ENST00000371625	p.Y128*	c.384C>A	Verified	CAG	TAC	GCG	3	C	A
TCGA-13-0913	PTGFR	ENST00000370756	p.H143N	c.427C>A	Verified	TTT	CAT	TCT	1	C	A
TCGA-13-0913	PTGFR_ENS⁻	ENST00000370758	p.H143N	c.427C>A	Verified	TTT	CAT	TCT	1	C	A
TCGA-29-1763	PTH2R	ENST00000272847	p.H510Q	c.1530C>A	Unverified	TTC	CAC	GAG	3	C	A
TCGA-09-2044	PTPN13	ENST00000436978	p.T2422N	c.7265C>A	Verified	GGG	ACC	CTG	2	C	A
TCGA-24-1422	PTPN21	ENST00000328736	p.P419H	c.1256C>A	Verified	AAC	CCT	AGC	2	C	A
TCGA-04-1338	PTPN3	ENST00000394831	p.D811E	c.2433C>A	Unverified	CCT	GAC	CAC	3	C	A
TCGA-24-1616	PTPRC	ENST00000442510	p.P160Q	c.479C>A	Verified	gac	cca	gtt	2	C	A
TCGA-13-0920	PTPRG	ENST00000474889	p.Q856K	c.2566C>A	Verified	GTC	CAG	CGC	1	C	A
TCGA-29-1763	PTPRH	ENST00000376350	p.P210Q	c.629C>A	Unverified	AAC	CCA	GTG	2	C	A
TCGA-23-1110	PTPRK	ENST00000368226	p.T1248K	c.3743C>A	Verified	gtc	aca	caa	2	C	A
TCGA-13-0761	PTPRT	NM_133170.2	p.T1271N	c.3812C>A	Verified	gtc	acc	cag	2	C	A
TCGA-13-0761	PTPRT	NM_133170.2	p.T1271N	c.3812C>A	Verified	gtc	acc	cag	2	C	A
TCGA-04-1356	PTPRU	ENST00000345512	p.H928Q	c.2784C>A	Verified	CGG	CAC	CGA	3	C	A
TCGA-61-1737	PTPRZ1	ENST00000393386	p.Q387K	c.1159C>A	Unverified	CTT	CAG	ATA	1	C	A
TCGA-61-1910	PTPRZ1	ENST00000393386	p.S548Y	c.1643C>A	Unverified	AGA	TCT	CCA	2	C	A
TCGA-23-2078	PTPRZ1	ENST00000393386	p.N1746K	c.5238C>A	Verified	TCC	AAC	CAC	3	C	A

TCGA-61-1737	PTPRZ1_ENS	ENST00000393386	p.Q387K	c.1159C>A	Unverified	CTT	CAG	ATA	1	C	A
TCGA-61-1910	PTPRZ1_ENS	ENST00000393386	p.S548Y	c.1643C>A	Unverified	AGA	TCT	CCA	2	C	A
TCGA-13-0916	PXN	ENST00000424649	p.S502Y	c.1505C>A	Verified	TGT	TCT	GGC	2	C	A
TCGA-24-1850	PZP	ENST00000261336	p.A1067E	c.3200C>A	Unverified	GAA	GCA	CAC	2	C	A
TCGA-24-1850	PZP_ENST0C	ENST00000381997	p.A853E	c.2558C>A	Unverified	GAA	GCA	CAC	2	C	A
TCGA-13-1509	Q6YL47_HU	ENST00000322367	p.A1300D	c.3899C>A	Verified	AAC	GCC	AAC	2	C	A
TCGA-61-1900	Q8NH47_HU	ENST00000319748	p.I162I	c.486C>A	Unverified	TGC	ATC	TTT	3	C	A
TCGA-13-0807	Q9C0K3_HU	ENST00000252071	p.D141E	c.423C>A	Verified	ATA	GAC	GTT	3	C	A
TCGA-13-1488	Q9Y6V0-3	ENST00000333891	p.S716*	c.2147C>A	Verified	TCA	TCA	TCA	2	C	A
TCGA-13-1408	QSER1	ENST00000399302	p.S1482Y	c.4445C>A	Unverified	CCT	TCC	GTG	2	C	A
TCGA-13-1481	QSOX2	ENST00000358701	p.F473L	c.1419C>A	Verified	ACC	TTC	TTT	3	C	A
TCGA-29-1770	RAB34	ENST00000301043	p.L68I	c.202C>A	Unverified	TGC	CTC	ATT	1	C	A
TCGA-29-1770	RAB34_ENST	ENST00000453384	p.L126I	c.376C>A	Unverified	TGC	CTC	ATT	1	C	A
TCGA-29-1763	RANBP6	ENST00000259569	p.I566I	c.1698C>A	Unverified	act	atc	gag	3	C	A
TCGA-61-1998	RAPGEF6_EI	ENST00000509018	p.A1588E	c.4763C>A	Verified	GAT	GCA	GAT	2	C	A
TCGA-23-1032	RAPH1	ENST00000319170	p.A593A	c.1779C>A	Verified	AAG	GCC	AGA	3	C	A
TCGA-13-1489	RB1	ENST00000267163	p.Q702K	c.2104C>A	Verified	GAC	CAA	ATT	1	C	A
TCGA-04-1362	RBBP4	ENST00000373493	p.A82D	c.245C>A	Verified	ATA	GCC	AGT	2	C	A
TCGA-24-2290	RBFA	ENST00000306735	p.D132E	c.396C>A	Verified	CCA	GAC	TTC	3	C	A
TCGA-24-2290	RBFA	ENST00000306735	p.D132E	c.396C>A	Verified	CCA	GAC	TTC	3	C	A
TCGA-29-1775	RBMS3_ENS	ENST00000383767	p.I78I	c.234C>A	Unverified	CTA	ATC	AAG	3	C	A
TCGA-20-1687	RFFL	ENST00000394597	p.P32T	c.94C>A	Unverified	AAC	CCT	GGG	1	C	A
TCGA-29-1691	RGAG1	ENST00000465301	p.P900Q	c.2699C>A	Unverified	TCA	CCA	CTA	2	C	A
TCGA-61-1899	RGS1	NM_002922.3	p.Q80K	c.238C>A	Unverified	aac	caa	act	1	C	A
TCGA-61-1899	RGS1_ENST(	ENST00000367459	p.Q93K	c.277C>A	Unverified	AAC	CAA	ACT	1	C	A
TCGA-24-1847	RGS9	ENST00000262406	p.A462D	c.1385C>A	Unverified	GCA	GCC	AAC	2	C	A
TCGA-13-0923	RHOC	ENST00000339083	p.D59E	c.177C>A	Verified	tgg	gac	aca	3	C	A
TCGA-13-1489	RIMS1	ENST00000521978	p.H1085N	c.3253C>A	Verified	CTT	CAT	CAT	1	C	A
TCGA-29-1703	RIMS2_ENST	ENST00000507740	p.L32I	c.94C>A	Unverified	GAG	CTT	TTT	1	C	A
TCGA-61-1906	RLBP1	ENST00000268125	p.Y124*	c.372C>A	Unverified	CAG	TAC	CCT	3	C	A
TCGA-61-1906	RLBP1	ENST00000268125	p.Y124*	c.372C>A	Unverified	CAG	TAC	CCT	3	C	A
TCGA-61-2095	RNASE1	ENST00000397967	p.S141R	c.423C>A	Unverified	GGG	AGC	CCA	3	C	A
TCGA-24-2280	RNASE13	ENST00000382951	p.A77D	c.230C>A	Unverified	CAT	GCC	CCT	2	C	A
TCGA-61-2095	RNF213	ENST00000336301	p.P1950T	c.5848C>A	Unverified	AAG	CCC	AGT	1	C	A

TCGA-61-2095	RNF213_ENS	ENST00000411702	p.P3926T	c.11776C>A	Unverified	AAG	CCC	AGT	1	C	A
TCGA-04-1356	RNF31	ENST00000324103	p.L398I	c.1192C>A	Verified	CCC	CTT	CAG	1	C	A
TCGA-25-2393	RNF43	ENST00000407977	p.P118T	c.352C>A	Verified	CGC	CCC	TGC	1	C	A
TCGA-61-1740	ROBO2	ENST00000461745	p.F357L	c.1071C>A	Unverified	CTT	TTC	CCA	3	C	A
TCGA-61-1740	ROBO2_ENS	ENST00000487694	p.F373L	c.1119C>A	Unverified	CTT	TTC	CCA	3	C	A
TCGA-13-0755	ROBO4	ENST00000306534	p.A495D	c.1484C>A	Verified	CGA	GCT	AGG	2	C	A
TCGA-13-1497	ROS1	ENST00000368508	p.S370Y	c.1109C>A	Verified	ATT	TCT	TCT	2	C	A
TCGA-04-1347	ROS1	ENST00000368508	p.G559G	c.1677C>A	Verified	CCA	GGC	CGC	3	C	A
TCGA-29-1696	RP11-166B2	ENST00000399147	p.L126I	c.376C>A	Unverified	ACT	CTA	CGG	1	C	A
TCGA-29-1696	RP11-166B2	ENST00000399147	p.L126I	c.376C>A	Unverified	ACT	CTA	CGG	1	C	A
TCGA-29-1696	RP11-166B2	ENST00000399147_v68	p.L126I	c.376C>A	Unverified	ACT	CTA	CGG	1	C	A
TCGA-29-1696	RP11-166B2	ENST00000399147_v68	p.L126I	c.376C>A	Unverified	ACT	CTA	CGG	1	C	A
TCGA-29-1690	RP11-231C1	ENST00000524087	p.P567P	c.1701C>A	Unverified	ACT	CCC	CTT	3	C	A
TCGA-29-1690	RP11-231C1	ENST00000524087_v68	p.P567P	c.1701C>A	Unverified	ACT	CCC	CTT	3	C	A
TCGA-13-0885	RP1L1	ENST00000382483	p.T24N	c.71C>A	Verified	CGC	ACC	CCC	2	C	A
TCGA-13-0807	RP1L1	ENST00000382483	p.L1576L	c.4728C>A	Verified	GAG	CTC	CAG	3	C	A
TCGA-23-1029	RP1L1	ENST00000382483	p.D2371E	c.7113C>A	Unverified	TAT	GAC	CTA	3	C	A
TCGA-23-2078	RP1L1	ENST00000382483	p.C16*	c.48C>A	Unverified	GAG	TGC	TTC	3	C	A
TCGA-61-1900	RPGRIP1_EN	ENST00000400017	p.G883G	c.2649C>A	Unverified	CCT	GGC	TCG	3	C	A
TCGA-23-1031	RPP21	ENST00000442966	p.L109L	c.327C>A	Verified	TTA	CTC	TGG	3	C	A
TCGA-04-1655	RPS10	ENST00000326199	p.N42K	c.126C>A	Unverified	CCC	AAC	CTT	3	C	A
TCGA-23-1032	RPS9	ENST00000391752	p.R172S	c.514C>A	Unverified	GGC	CGC	GTG	1	C	A
TCGA-04-1347	RPSAP12	ENST00000361461	p.P263H	c.788C>A	Verified	TTC	CCT	ACT	2	C	A
TCGA-29-1691	RPTN	ENST00000316073	p.A219D	c.656C>A	Unverified	CAG	GCT	AAA	2	C	A
TCGA-13-1505	RQCD1	ENST00000273064	p.N88K	c.264C>A	Verified	TCT	AAC	AGA	3	C	A
TCGA-09-2051	RRP15	ENST00000366932	p.L140M	c.418C>A	Unverified	AGG	CTG	GAG	1	C	A
TCGA-61-2095	RRP8	ENST00000254605	p.I21I	c.63C>A	Unverified	GTA	ATC	TCA	3	C	A
TCGA-23-1118	RTN1	ENST00000267484	p.T296N	c.887C>A	Verified	ACT	ACC	CAA	2	C	A
TCGA-23-1122	RTN4R	ENST00000043402	p.A215D	c.644C>A	Unverified	GTG	GCC	CAT	2	C	A
TCGA-13-0920	RTP3	ENST00000296142	p.P177Q	c.530C>A	Verified	ACC	CCA	AGA	2	C	A
TCGA-13-1512	RUNX2	ENST00000465038	p.S323S	c.969C>A	Verified	tct	tcc	aca	3	C	A
TCGA-23-1021	RWDD1	ENST00000466444	p.P230T	c.688C>A	Verified	GAT	CCA	GAC	1	C	A
TCGA-23-1021	RWDD1	ENST00000466444	p.P230Q	c.689C>A	Verified	GAT	CCA	GAC	2	C	A
TCGA-29-1775	RXRG	ENST00000359842	p.Q407K	c.1219C>A	Unverified	aag	cag	aag	1	C	A

TCGA-29-1784	RYR1	ENST00000359596	p.P263T	c.787C>A	Unverified	GAG	CCA	CTG	1	C	A
TCGA-13-0893	RYR2	ENST00000366574	p.H3082N	c.9244C>A	Verified	ACT	CAC	ACC	1	C	A
TCGA-13-0760	RYR3	ENST00000389232	p.P2885H	c.8654C>A	Verified	AAG	CCC	CTT	2	C	A
TCGA-13-0883	S100A6	ENST00000368720	p.S20S	c.60C>A	Verified	TAC	TCC	GGC	3	C	A
TCGA-25-1313	S1PR1	ENST00000305352	p.S44R	c.132C>A	Verified	AAC	AGC	ATT	3	C	A
TCGA-25-2042	SAG	ENST00000409110	p.P206T	c.616C>A	Verified	AAG	CCC	CTG	1	C	A
TCGA-10-0930	SALL1	ENST00000251020	p.P577H	c.1730C>A	Verified	GCC	CCC	ATC	2	C	A
TCGA-10-0930	SALL1	ENST00000251020	p.P577H	c.1730C>A	Verified	GCC	CCC	ATC	2	C	A
TCGA-29-1701	SALL4	ENST00000217086	p.P212Q	c.635C>A	Unverified	ATC	CCG	TGG	2	C	A
TCGA-09-2044	SAP30	ENST00000296504	p.I100I	c.300C>A	Verified	aag	atc	gag	3	C	A
TCGA-29-1698	SCAF1	ENST00000360565	p.P233P	c.699C>A	Unverified	CAC	CCC	ACC	3	C	A
TCGA-20-1683	SCN3A	ENST00000283254	p.Q1473K	c.4417C>A	Unverified	AAC	CAG	CAG	1	C	A
TCGA-20-1683	SCN3A_ENST	ENST00000409101	p.Q1424K	c.4270C>A	Unverified	AAC	CAG	CAG	1	C	A
TCGA-23-1122	SCN8A	ENST00000354534	p.P1739T	c.5215C>A	Verified	AAC	CCC	TCA	1	C	A
TCGA-13-1507	SCNM1	ENST00000368905	p.P184T	c.550C>A	Verified	AGC	CCC	ACA	1	C	A
TCGA-25-2393	SCNN1B	ENST00000343070	p.T486T	c.1458C>A	Verified	ATC	ACC	CTG	3	C	A
TCGA-09-2056	SCRT2	ENST00000246104	p.F240L	c.720C>A	Unverified	CCG	TTC	GGC	3	C	A
TCGA-29-1776	SDK1	ENST00000404826	p.T2043K	c.6128C>A	Unverified	AGC	ACA	GGA	2	C	A
TCGA-29-1764	SEC14L5	ENST00000251170	p.Q321K	c.961C>A	Unverified	TAC	CAG	GAC	1	C	A
TCGA-29-1768	SEC16B	ENST00000308284	p.I673I	c.2019C>A	Unverified	CTC	ATC	AAG	3	C	A
TCGA-23-1809	SEC31A_ENS	ENST00000395310	p.P869T	c.2605C>A	Unverified	GTA	CCA	CCT	1	C	A
TCGA-23-1809	SEC31A_ENS	ENST00000432794	p.P869T	c.2605C>A	Unverified	GTA	CCA	CCT	1	C	A
TCGA-10-0930	SEPX1	ENST00000361871	p.S98R	c.294C>A	Verified	TTC	AGC	AGC	3	C	A
TCGA-10-0930	SEPX1	ENST00000361871	p.S98R	c.294C>A	Verified	TTC	AGC	AGC	3	C	A
TCGA-13-0792	SERPINA4	ENST00000557004	p.H147Q	c.441C>A	Verified	AGC	CAC	AAC	3	C	A
TCGA-61-1998	SERPINA4	ENST00000557004	p.I101I	c.303C>A	Verified	CAG	ATC	CTT	3	C	A
TCGA-29-1762	SERPINE3_E	ENST00000521255	p.P141T	c.421C>A	Unverified	GAA	CCA	GCC	1	C	A
TCGA-29-1764	SERTAD4	ENST00000367012	p.S112Y	c.335C>A	Unverified	ATG	TCC	TTA	2	C	A
TCGA-61-1900	SETBP1	ENST00000282030	p.P37T	c.109C>A	Unverified	GAA	CCT	TTG	1	C	A
TCGA-61-1900	SETBP1_ENS	ENST00000426838	p.P37T	c.109C>A	Unverified	GAA	CCT	TTG	1	C	A
TCGA-24-1470	SETX	ENST00000224140	p.R128S	c.382C>A	Verified	GAA	CGT	GTT	1	C	A
TCGA-24-2280	SEZ6	ENST00000317338	p.P740T	c.2218C>A	Unverified	TAC	CCT	GGC	1	C	A
TCGA-13-1497	SEZ6L	ENST00000248933	p.Q548K	c.1642C>A	Verified	GAC	CAG	GCC	1	C	A
TCGA-13-1507	SHC4	ENST00000332408	p.P497T	c.1489C>A	Verified	GCA	CCA	GAA	1	C	A

TCGA-24-1104	SHF	ENST00000290894	p.P270Q	c.809C>A	Verified	GGA	CCG	GAG	2	C	A
TCGA-04-1338	SHQ1	ENST00000325599	p.R269S	c.805C>A	Verified	TGT	CGT	CAA	1	C	A
TCGA-13-0904	SHQ1	ENST00000325599	p.H198N	c.592C>A	Verified	GAT	CAT	TAT	1	C	A
TCGA-09-2051	SI	ENST00000264382	p.P1202T	c.3604C>A	Unverified	ACT	CCA	GAA	1	C	A
TCGA-04-1342	SIDT2	ENST00000324225	p.Q86K	c.256C>A	Unverified	CGC	CAG	AAG	1	C	A
TCGA-30-1718	SIGLEC12	ENST00000291707	p.A195A	c.585C>A	Unverified	GGG	GCC	GAT	3	C	A
TCGA-23-1029	SIGLEC16	ENST00000456956	p.P370Q	c.1109C>A	Unverified	CCC	CCA	GCC	2	C	A
TCGA-23-1022	SIGLEC8	ENST00000321424	p.L375M	c.1123C>A	Verified	TTC	CTG	TCC	1	C	A
TCGA-04-1338	SIRPD	ENST00000381623	p.T102T	c.306C>A	Verified	TCC	ACC	CGC	3	C	A
TCGA-29-1785	SIRT7	ENST00000328666	p.A258A	c.774C>A	Unverified	GCT	GCC	AGC	3	C	A
TCGA-29-1785	SIRT7	ENST00000328666	p.A258A	c.774C>A	Unverified	GCT	GCC	AGC	3	C	A
TCGA-23-1117	SLAMF9	ENST00000368093	p.S157Y	c.470C>A	Verified	TGC	TCT	GTG	2	C	A
TCGA-23-1117	SLAMF9	ENST00000368093	p.S157Y	c.470C>A	Verified	TGC	TCT	GTG	2	C	A
TCGA-24-1417	SLC11A1	ENST00000233202	p.G498G	c.1494C>A	Verified	TTC	GGC	CTT	3	C	A
TCGA-23-1021	SLC12A8_EN	ENST00000393469	p.A135A	c.405C>A	Verified	GGT	GCC	ATG	3	C	A
TCGA-23-1032	SLC16A14	ENST00000295190	p.I369I	c.1107C>A	Verified	GTG	ATC	CTG	3	C	A
TCGA-24-2280	SLC17A1	ENST00000244527	p.S332R	c.996C>A	Verified	CTC	AGC	GTA	3	C	A
TCGA-24-1845	SLC17A2	ENST00000360488	p.T133T	c.399C>A	Unverified	CTC	ACC	CTC	3	C	A
TCGA-13-0885	SLC17A8	ENST00000323346	p.Q579K	c.1735C>A	Verified	TAC	CAG	AAT	1	C	A
TCGA-13-0904	SLC22A15_E	ENST00000369503	p.S108Y	c.323C>A	Verified	AGA	TCC	TAC	2	C	A
TCGA-23-1117	SLC24A2	ENST00000341998	p.P488T	c.1462C>A	Verified	TTA	CCT	GAC	1	C	A
TCGA-23-1117	SLC24A2	ENST00000341998	p.P488T	c.1462C>A	Verified	TTA	CCT	GAC	1	C	A
TCGA-09-2050	SLC25A23	ENST00000301454	p.F260L	c.780C>A	Verified	AAG	TTC	ATG	3	C	A
TCGA-23-1117	SLC25A32	ENST00000297578	p.F101L	c.303C>A	Verified	TTT	TTC	TTT	3	C	A
TCGA-23-1117	SLC25A32	ENST00000297578	p.F101L	c.303C>A	Verified	TTT	TTC	TTT	3	C	A
TCGA-29-1701	SLC25A41	ENST00000321510	p.T219K	c.656C>A	Unverified	CGG	ACG	GGC	2	C	A
TCGA-24-1417	SLC26A3	ENST00000340010	p.P532Q	c.1595C>A	Unverified	GAG	CCA	GAA	2	C	A
TCGA-61-1910	SLC28A1	ENST00000286749	p.A312D	c.935C>A	Unverified	GTG	GCT	GGA	2	C	A
TCGA-24-2024	SLC29A2	ENST00000357440	p.S443Y	c.1328C>A	Verified	CTT	TCC	TGT	2	C	A
TCGA-13-0900	SLC2A11	ENST00000398356	p.S104Y	c.311C>A	Verified	AAG	TCC	CTC	2	C	A
TCGA-04-1347	SLC2A13	ENST00000380863	p.Y436*	c.1308C>A	Verified	TGC	TAC	AAG	3	C	A
TCGA-25-2400	SLC34A3	ENST00000361134	p.D52E	c.156C>A	Unverified	AAG	GAC	ACA	3	C	A
TCGA-25-1326	SLC35F3	ENST00000366618	p.L282I	c.844C>A	Verified	ATC	CTC	GCC	1	C	A
TCGA-13-0903	SLC35F4	ENST00000339762	p.S51*	c.152C>A	Verified	AAA	TCA	CTG	2	C	A

TCGA-61-1913	SLC37A4	ENST00000330775	p.S293S	c.879C>A	Unverified	CTG	TCC	AAC	3	C	A
TCGA-61-1740	SLC38A4	ENST00000266579	p.A462E	c.1385C>A	Unverified	ATT	GCA	CTT	2	C	A
TCGA-29-1785	SLC40A1	ENST00000261024	p.P424T	c.1270C>A	Unverified	ATA	CCT	GAA	1	C	A
TCGA-29-1785	SLC40A1	ENST00000261024	p.P424T	c.1270C>A	Unverified	ATA	CCT	GAA	1	C	A
TCGA-24-0979	SLC47A2	ENST00000325411	p.G289G	c.867C>A	Unverified	TGG	GGC	CCC	3	C	A
TCGA-24-1845	SLC4A1	ENST00000262418	p.L738L	c.2214C>A	Unverified	GCC	CTC	ACT	3	C	A
TCGA-29-1781	SLC4A10	ENST00000415876	p.R237S	c.709C>A	Unverified	GTT	CGT	TCC	1	C	A
TCGA-29-1781	SLC4A10_EN	ENST00000446997	p.R237S	c.709C>A	Unverified	GTT	CGT	TCC	1	C	A
TCGA-23-1124	SLC4A5	ENST00000394019	p.P30T	c.88C>A	Verified	CCT	CCT	ATC	1	C	A
TCGA-24-1474	SLC4A7	ENST00000295736	p.A357E	c.1070C>A	Verified	GAA	GCA	GCG	2	C	A
TCGA-13-0893	SLC5A9	ENST00000236495	p.T282T	c.846C>A	Verified	AAC	ACC	ACC	3	C	A
TCGA-13-1488	SLC6A12	ENST00000397296	p.A73D	c.218C>A	Verified	GGA	GCC	TTC	2	C	A
TCGA-29-1696	SLC7A14	ENST00000231706	p.L599I	c.1795C>A	Unverified	ATC	CTT	CTG	1	C	A
TCGA-29-1696	SLC7A14	ENST00000231706	p.L599I	c.1795C>A	Unverified	ATC	CTT	CTG	1	C	A
TCGA-13-0887	SLC7A14	ENST00000231706	p.S24S	c.72C>A	Verified	CAC	TCC	AGG	3	C	A
TCGA-61-2009	SLCO2B1	ENST00000289575	p.C553*	c.1659C>A	Verified	TCC	TGC	GAC	3	C	A
TCGA-13-0920	SLITRK3	ENST00000241274	p.T94T	c.282C>A	Verified	TAT	ACC	AAC	3	C	A
TCGA-24-1474	SLITRK4	ENST00000381779	p.A833D	c.2498C>A	Unverified	aca	gct	ttg	2	C	A
TCGA-13-0760	SLITRK6	ENST00000400286	p.L95I	c.283C>A	Verified	CAC	CTT	GGA	1	C	A
TCGA-04-1342	SMAD7	ENST00000262158	p.C160*	c.480C>A	Unverified	CTG	TGC	AAA	3	C	A
TCGA-13-0761	SMARCA1	NM_003069.2	p.I195I	c.585C>A	Verified	ttg	atc	tct	3	C	A
TCGA-13-0761	SMARCA1	NM_003069.2	p.I195I	c.585C>A	Verified	ttg	atc	tct	3	C	A
TCGA-23-1123	SMARCA4	ENST00000429416	p.T814K	c.2441C>A	Verified	tca	acg	ctg	2	C	A
TCGA-23-1123	SMARCA4	ENST00000429416	p.T814K	c.2441C>A	Verified	tca	acg	ctg	2	C	A
TCGA-13-1501	SMARCA4	ENST00000429416	p.L476L	c.1428C>A	Verified	tac	ctc	aat	3	C	A
TCGA-13-1501	SMARCA4	ENST00000429416	p.L476L	c.1428C>A	Verified	tac	ctc	aat	3	C	A
TCGA-29-1711	SMC1A	ENST00000322213	p.A1143A	c.3429C>A	Unverified	ttt	gcc	atc	3	C	A
TCGA-29-1711	SMC1A	ENST00000322213	p.A1143A	c.3429C>A	Unverified	ttt	gcc	atc	3	C	A
TCGA-04-1649	SMOC2	ENST00000354536	p.T209N	c.626C>A	Unverified	TGG	ACT	GAA	2	C	A
TCGA-23-1120	SMYD4	ENST00000305513	p.P797H	c.2390C>A	Unverified	CCA	CCC	ACC	2	C	A
TCGA-29-1762	SNRK	ENST00000454177	p.A603D	c.1808C>A	Unverified	AAT	GCT	GGT	2	C	A
TCGA-29-1762	SNRK_ENST(	ENST00000454177	p.A603D	c.1808C>A	Unverified	AAT	GCT	GGT	2	C	A
TCGA-13-0904	SNRNP200	ENST00000323853	p.T790T	c.2370C>A	Verified	ATG	ACC	AGG	3	C	A
TCGA-23-1123	SNX8	ENST00000222990	p.R236R	c.708C>A	Verified	CTT	CGC	GAC	3	C	A

TCGA-23-1123	SNX8	ENST00000222990	p.R236R	c.708C>A	Verified	CTT	CGC	GAC	3	C	A
TCGA-09-1674	SORL1	ENST00000260197	p.Q479K	c.1435C>A	Unverified	GCT	CAG	CGC	1	C	A
TCGA-13-0893	SOS1	ENST00000426016	p.P1184H	c.3551C>A	Verified	cct	cct	agg	2	C	A
TCGA-13-1481	SPAG1	ENST00000388798	p.S581*	c.1742C>A	Verified	CTG	TCA	CCT	2	C	A
TCGA-29-1775	SPAG17	ENST00000336338	p.T2013N	c.6038C>A	Unverified	CCA	ACC	CAG	2	C	A
TCGA-09-1665	SPAG8	ENST00000340291	p.S299R	c.897C>A	Verified	CCA	AGC	ATG	3	C	A
TCGA-23-2077	SPHKAP	XM_051221.5	p.P1617T	c.4849C>A	Unverified	ccg	cct	acg	1	C	A
TCGA-24-1845	SPIRE1	ENST00000309836	p.L420I	c.1258C>A	Unverified	AAG	CTC	TGC	1	C	A
TCGA-24-1845	SPIRE1_ENST	ENST00000409402	p.L593I	c.1777C>A	Unverified	AAG	CTC	TGC	1	C	A
TCGA-61-2102	SPN	ENST00000360121	p.P389H	c.1166C>A	Unverified	GCT	CCT	GAT	2	C	A
TCGA-24-0979	SPOCD1	ENST00000360482	p.I59I	c.177C>A	Unverified	AAG	ATC	CCC	3	C	A
TCGA-23-1124	SPP1	ENST00000395080	p.Q229K	c.685C>A	Verified	agt	cag	ctg	1	C	A
TCGA-04-1649	SPRY1	ENST00000339241	p.T193N	c.578C>A	Unverified	tgc	act	gct	2	C	A
TCGA-25-2042	SPRYD4	ENST00000338146	p.L162M	c.484C>A	Unverified	AAG	CTG	AGC	1	C	A
TCGA-09-2051	SPTBN5	ENST00000320955	p.C3366*	c.10098C>A	Unverified	GAG	TGC	CGC	3	C	A
TCGA-04-1655	SRCAP	ENST00000262518	p.T3133N	c.9398C>A	Unverified	GAG	ACT	GAG	2	C	A
TCGA-29-1702	SRP72	ENST00000342756	p.L521I	c.1561C>A	Unverified	GCT	CTT	GAA	1	C	A
TCGA-13-1488	SRP9P1	ENST00000318333	p.F65L	c.195C>A	Verified	AAA	TTC	CAC	3	C	A
TCGA-09-2051	SRSF1	ENST00000258962	p.H140Q	c.420C>A	Unverified	GAT	CAC	ATG	3	C	A
TCGA-29-1762	SSFA2	ENST00000320370	p.H505N	c.1513C>A	Unverified	CAG	CAT	TCC	1	C	A
TCGA-25-1326	ST8SIA5	ENST00000315087	p.L130I	c.388C>A	Verified	AAG	CTC	AAG	1	C	A
TCGA-24-1474	STAB2	ENST00000388887	p.P1929T	c.5785C>A	Verified	CTG	CCC	TTC	1	C	A
TCGA-24-1563	STAG2	ENST00000371160	p.R305R	c.913C>A	Verified	att	cga	gct	1	C	A
TCGA-61-1998	STAG3	ENST00000426455	p.R232S	c.694C>A	Verified	ttc	cgt	cac	1	C	A
TCGA-13-0890	STAG3	ENST00000426455	p.A441A	c.1323C>A	Verified	ctg	gcc	tct	3	C	A
TCGA-24-1474	STAMBPL1	ENST00000371924	p.A318E	c.953C>A	Verified	TCT	GCG	GGA	2	C	A
TCGA-24-1422	STARD4	ENST00000296632	p.S81R	c.243C>A	Verified	gac	agc	ttg	3	C	A
TCGA-23-1123	STARD5	ENST00000302824	p.T178T	c.534C>A	Verified	CAT	ACC	GAC	3	C	A
TCGA-23-1123	STARD5	ENST00000302824	p.T178T	c.534C>A	Verified	CAT	ACC	GAC	3	C	A
TCGA-13-0904	STARD8	ENST00000252336	p.N456K	c.1368C>A	Verified	GGG	AAC	TCC	3	C	A
TCGA-13-0904	STARD8_ENS	ENST00000252336	p.N456K	c.1368C>A	Verified	GGG	AAC	TCC	3	C	A
TCGA-24-1849	STEAP1	ENST00000297205	p.P41H	c.122C>A	Unverified	AGA	CCT	GTG	2	C	A
TCGA-13-0886	STK10	ENST00000176763	p.G513G	c.1539C>A	Verified	ATG	GGC	TCT	3	C	A
TCGA-13-0913	STK38L	ENST00000389032	p.L359I	c.1075C>A	Verified	ATT	CTC	AGA	1	C	A

TCGA-04-1649	STRC_ENST(	ENST00000450892	p.T1178N	c.3533C>A	Unverified	CCC	ACC	AAC	2	C	A
TCGA-25-1318	STX3	ENST00000337979	p.P69T	c.205C>A	Verified	ATT	CCA	GAG	1	C	A
TCGA-25-1318	STX3	ENST00000337979	p.P69T	c.205C>A	Verified	ATT	CCA	GAG	1	C	A
TCGA-24-2262	SULF1	ENST00000260128	p.S676R	c.2028C>A	Unverified	TGT	AGC	TGC	3	C	A
TCGA-24-2288	SULT1C2	ENST00000326853	p.L43L	c.129C>A	Unverified	CTC	CTC	ATC	3	C	A
TCGA-29-1762	SULT1C4	ENST00000272452	p.L112M	c.334C>A	Unverified	ATC	CTG	AAA	1	C	A
TCGA-23-2078	SURF1	ENST00000371974	p.Y274*	c.822C>A	Unverified	cag	tac	atc	3	C	A
TCGA-24-2035	SUSD3	ENST00000375472	p.T42N	c.125C>A	Verified	gca	acc	ttc	2	C	A
TCGA-23-1118	SUV420H1	NM_017635.3	p.P670T	c.2008C>A	Verified	gct	cct	tca	1	C	A
TCGA-23-1124	SWAP70	ENST00000318950	p.S197Y	c.590C>A	Verified	GTG	TCT	ATG	2	C	A
TCGA-24-1104	SYCE1	ENST00000368517	p.T188N	c.563C>A	Verified	TCC	ACC	CTT	2	C	A
TCGA-61-1899	SYCP2L	ENST00000283141	p.L249I	c.745C>A	Unverified	GAA	CTT	GTC	1	C	A
TCGA-29-2427	SYNE1	ENST00000265368	p.T6205K	c.18614C>A	Verified	CTA	ACA	GCC	2	C	A
TCGA-61-1737	SYNE1	ENST00000265368	p.A4125D	c.12374C>A	Unverified	CAG	GCC	CAG	2	C	A
TCGA-04-1338	SYNE1	ENST00000265368	p.G5491G	c.16473C>A	Verified	AAT	GGC	CAA	3	C	A
TCGA-29-2427	SYNE1_ENST	ENST00000265368	p.T6205K	c.18614C>A	Verified	CTA	ACA	GCC	2	C	A
TCGA-61-1737	SYNE1_ENST	ENST00000265368	p.A4125D	c.12374C>A	Unverified	CAG	GCC	CAG	2	C	A
TCGA-04-1338	SYNE1_ENST	ENST00000265368	p.G5491G	c.16473C>A	Verified	AAT	GGC	CAA	3	C	A
TCGA-61-1737	SYNE1_ENST	ENST00000423061	p.A4054D	c.12161C>A	Unverified	CAG	GCC	CAG	2	C	A
TCGA-29-1691	SYNE2	ENST00000358025	p.P5531H	c.16592C>A	Unverified	AAT	CCT	GAC	2	C	A
TCGA-61-1915	SYT11	ENST00000368324	p.T68N	c.203C>A	Unverified	GAG	ACC	CTC	2	C	A
TCGA-04-1347	SYTL1	ENST00000318074	p.N349K	c.1047C>A	Verified	CGC	AAC	ATC	3	C	A
TCGA-25-1326	TAAR6	ENST00000275198	p.Q62K	c.184C>A	Verified	AAG	CAG	CTG	1	C	A
TCGA-24-1422	TAC3	ENST00000300108	p.P41T	c.121C>A	Verified	GAT	CCA	GAT	1	C	A
TCGA-23-1022	TAF6	ENST00000344095	p.L201M	c.601C>A	Verified	TTG	CTG	GAG	1	C	A
TCGA-24-1843	TAS1R2	ENST00000375371	p.L34L	c.102C>A	Unverified	TAC	CTC	CTG	3	C	A
TCGA-29-1691	TAS2R1	ENST00000382492	p.L247I	c.739C>A	Unverified	TTT	CTC	TCT	1	C	A
TCGA-24-1463	TAS2R9	ENST00000240691	p.T209N	c.626C>A	Unverified	CAC	ACC	AAG	2	C	A
TCGA-24-1563	TBC1D2	ENST00000375066	p.A720D	c.2159C>A	Verified	GCG	GCC	ATT	2	C	A
TCGA-24-2289	TBC1D5	ENST00000253692	p.D794E	c.2382C>A	Unverified	CTG	GAC	ATC	3	C	A
TCGA-61-1914	TBC1D8	ENST00000409318	p.L531I	c.1591C>A	Unverified	TGG	CTT	CTC	1	C	A
TCGA-61-1914	TBC1D8_EN!	ENST00000376840	p.L516I	c.1546C>A	Unverified	TGG	CTT	CTC	1	C	A
TCGA-29-1777	TBC1D8B	ENST00000357242	p.S920*	c.2759C>A	Unverified	gct	tca	aaa	2	C	A
TCGA-29-1775	TBPL2	ENST00000247219	p.P160T	c.478C>A	Unverified	CAC	CCT	GGT	1	C	A

TCGA-20-1685	TBPL2	ENST00000247219	p.H119Q	c.357C>A	Unverified	AAA	CAC	GAA	3	C	A
TCGA-30-1714	TBRG4	ENST00000258770	p.L527M	c.1579C>A	Unverified	GTG	CTG	CTG	1	C	A
TCGA-13-0883	TBX22	ENST00000373296	p.L201I	c.601C>A	Verified	aaa	ctc	acc	1	C	A
TCGA-24-1431	TCEB3	ENST00000374536	p.L139I	c.415C>A	Verified	GAG	CTC	GAG	1	C	A
TCGA-30-1855	TCF12	ENST00000438423	p.L471L	c.1413C>A	Unverified	AGC	CTC	AAT	3	C	A
TCGA-30-1855	TCF12_ENST	ENST00000343827	p.L277L	c.831C>A	Unverified	AGC	CTC	AAT	3	C	A
TCGA-30-1855	TCF12_ENST	ENST00000438423	p.L471L	c.1413C>A	Unverified	AGC	CTC	AAT	3	C	A
TCGA-30-1855	TCF12_ENST	ENST00000452095	p.L467L	c.1401C>A	Unverified	AGC	CTC	AAT	3	C	A
TCGA-61-2109	TCF20	ENST00000359486	p.P372T	c.1114C>A	Unverified	TCT	CCA	GCT	1	C	A
TCGA-61-1910	TCL1A	ENST00000554012	p.T65T	c.195C>A	Unverified	CCC	ACC	CAG	3	C	A
TCGA-29-1776	TCP1	ENST00000321394	p.H113N	c.337C>A	Unverified	ATT	CAT	CCC	1	C	A
TCGA-20-1687	TDGF1	ENST00000296145	p.C97*	c.291C>A	Unverified	GCC	TGC	CCT	3	C	A
TCGA-13-0920	TDP2	ENST00000378198	p.S69R	c.207C>A	Verified	gag	agc	gcc	3	C	A
TCGA-29-1711	TEAD2	ENST00000311227	p.I115I	c.345C>A	Unverified	GAA	ATC	CAG	3	C	A
TCGA-29-1711	TEAD2	ENST00000311227	p.I115I	c.345C>A	Unverified	GAA	ATC	CAG	3	C	A
TCGA-29-1766	TECTA	ENST00000392793	p.I233I	c.699C>A	Unverified	AAT	ATC	CAG	3	C	A
TCGA-09-2049	TEKT4	ENST00000295201	p.I212I	c.636C>A	Verified	AAC	ATC	GAC	3	C	A
TCGA-23-1031	TEP1	ENST00000262715	p.L1222M	c.3664C>A	Unverified	TAT	CTG	CGT	1	C	A
TCGA-29-1768	TET2	ENST00000380013	p.P732Q	c.2195C>A	Unverified	CAA	CCA	TCC	2	C	A
TCGA-29-1768	TET2_ENST0	ENST00000305737	p.P732Q	c.2195C>A	Unverified	CAA	CCA	TCC	2	C	A
TCGA-61-1725	TFR2	ENST00000223051	p.Y594*	c.1782C>A	Unverified	GCC	TAC	CCA	3	C	A
TCGA-24-1604	TG	ENST00000220616	p.Q1095K	c.3283C>A	Verified	TCC	CAA	GAA	1	C	A
TCGA-04-1338	TGFBR3	ENST00000212355	p.H94N	c.280C>A	Verified	GTC	CAC	ATC	1	C	A
TCGA-25-1313	THAP8	ENST00000392182	p.Q181K	c.541C>A	Unverified	CTG	CAA	CGC	1	C	A
TCGA-13-0890	THNSL2	ENST00000324166	p.R413R	c.1237C>A	Verified	CCC	CGG	TGC	1	C	A
TCGA-13-0760	THOP1	ENST00000307741	p.L182M	c.544C>A	Verified	AAC	CTG	AAC	1	C	A
TCGA-23-1123	THSD7B	ENST00000272643	p.S659Y	c.1976C>A	Verified	CAT	TCC	TGT	2	C	A
TCGA-23-1123	THSD7B	ENST00000272643	p.S659Y	c.1976C>A	Verified	CAT	TCC	TGT	2	C	A
TCGA-24-1604	THSD7B	ENST00000272643	p.S1485Y	c.4454C>A	Verified	TTC	TCC	TAC	2	C	A
TCGA-23-1031	TIAM2	ENST00000318981	p.S402S	c.1206C>A	Verified	AGG	TCC	AAG	3	C	A
TCGA-24-1604	TIE1	ENST00000372476	p.V1041V	c.3123C>A	Verified	GGA	GTC	CTT	3	C	A
TCGA-13-1510	TIMELESS	ENST00000553532	p.R277R	c.829C>A	Verified	TCT	CGA	TTT	1	C	A
TCGA-23-1124	TLE6	ENST00000452088	p.P331H	c.992C>A	Verified	AAA	CCT	CTG	2	C	A
TCGA-13-0791	TLR5	ENST00000366881	p.Y108*	c.324C>A	Verified	ATA	TAC	TTC	3	C	A

TCGA-23-1110	TLR5	ENST00000366881	p.T468T	c.1404C>A	Verified	CAA	ACC	CCT	3	C	A
TCGA-24-1103	TMC2	ENST00000358864	p.P560P	c.1680C>A	Verified	GTC	CCC	CGA	3	C	A
TCGA-13-0760	TMED1	ENST00000214869	p.V83V	c.249C>A	Verified	TTG	GTC	AGC	3	C	A
TCGA-09-2050	TMED3	ENST00000299705	p.T108N	c.323C>A	Verified	TCC	ACC	TTC	2	C	A
TCGA-24-2262	TMEM105	ENST00000332900	p.S59S	c.177C>A	Verified	GGG	TCC	CCA	3	C	A
TCGA-61-1733	TMEM132B	ENST00000299308	p.F412L	c.1236C>A	Unverified	ATC	TTC	GTC	3	C	A
TCGA-61-1733	TMEM132B	ENST00000299308	p.F412L	c.1236C>A	Unverified	ATC	TTC	GTC	3	C	A
TCGA-13-1498	TMEM38B	ENST00000374692	p.A43E	c.128C>A	Verified	TTG	GCA	TGG	2	C	A
TCGA-23-1110	TMEM53	ENST00000372237	p.R134S	c.400C>A	Verified	TGC	CGC	CTG	1	C	A
TCGA-61-1725	TMEM55A	ENST00000285419	p.G212G	c.636C>A	Unverified	GTT	GGC	ACC	3	C	A
TCGA-04-1361	TMEM59L	ENST00000262817	p.P214T	c.640C>A	Verified	CAC	CCT	GAA	1	C	A
TCGA-04-1361	TMEM59L	ENST00000262817	p.P214T	c.640C>A	Verified	CAC	CCT	GAA	1	C	A
TCGA-04-1651	TMEM62	ENST00000260403	p.S182*	c.545C>A	Unverified	TAT	TCG	TTC	2	C	A
TCGA-04-1651	TMEM62	ENST00000260403	p.S182*	c.545C>A	Unverified	TAT	TCG	TTC	2	C	A
TCGA-25-2042	TMEM70	ENST00000312184	p.S40Y	c.119C>A	Unverified	GCG	TCC	TCC	2	C	A
TCGA-13-0893	TMEM74	ENST00000297459	p.T286K	c.857C>A	Verified	agc	acg	aat	2	C	A
TCGA-61-1900	TMEM9B	ENST00000534025	p.H156Q	c.468C>A	Unverified	GCA	CAC	GAT	3	C	A
TCGA-29-1783	TMPRSS11A	ENST00000334830	p.Q105K	c.313C>A	Unverified	AAC	CAA	GTA	1	C	A
TCGA-24-1422	TMPRSS15	ENST00000284885	p.T42K	c.125C>A	Verified	CTG	ACA	ATC	2	C	A
TCGA-13-0760	TMPRSS3	ENST00000291532	p.A387E	c.1160C>A	Verified	TGC	GCG	GGC	2	C	A
TCGA-29-1763	TNFRSF8	ENST00000263932	p.T294T	c.882C>A	Unverified	GCC	ACC	AAC	3	C	A
TCGA-13-1510	TNIK	ENST00000436636	p.P869T	c.2605C>A	Verified	GCT	CCA	GGC	1	C	A
TCGA-13-0884	TNPO3	ENST00000265388	p.R160R	c.478C>A	Verified	tta	cga	att	1	C	A
TCGA-24-1844	TNS3	ENST00000311160	p.P483H	c.1448C>A	Unverified	ATG	CCC	CAC	2	C	A
TCGA-61-2008	TOPORS	ENST00000360538	p.T185K	c.554C>A	Verified	CTG	ACA	AGG	2	C	A
TCGA-61-2012	TOX2_ENST(	ENST00000348077	p.A30E	c.89C>A	Verified	CTT	GCA	AGA	2	C	A
TCGA-04-1347	TPO	ENST00000345913	p.D585E	c.1755C>A	Verified	cgg	gac	cac	3	C	A
TCGA-61-1740	TRAK1_ENS	ENST00000396175	p.P819T	c.2455C>A	Unverified	GGG	CCC	CCG	1	C	A
TCGA-29-1777	TRAPPC8	ENST00000283351	p.P474Q	c.1421C>A	Unverified	CAA	CCA	GGA	2	C	A
TCGA-09-2044	TREX2	ENST00000393862	p.S38S	c.114C>A	Unverified	cgc	tcc	tcc	3	C	A
TCGA-13-0793	TRIM31	ENST00000376734	p.Q5K	c.13C>A	Unverified	ggg	cag	ttt	1	C	A
TCGA-25-2042	TRIM5	ENST00000380034	p.Y138*	c.414C>A	Unverified	GAG	TAC	CAA	3	C	A
TCGA-24-2289	TRIM58	ENST00000366481	p.L439I	c.1315C>A	Unverified	CAA	CTC	TTC	1	C	A
TCGA-24-2289	TRIM71	ENST00000383763	p.S504S	c.1512C>A	Unverified	GCC	TCC	TTC	3	C	A

TCGA-24-1845	TRIP10	ENST00000313285	p.D467E	c.1401C>A	Unverified	cag	gac	acc	3	C	A
TCGA-24-1845	TRIP10_ENS	ENST00000313244	p.D523E	c.1569C>A	Unverified	CAG	GAC	ACC	3	C	A
TCGA-24-0979	TRO	ENST00000319167	p.L394M	c.1180C>A	Verified	tat	ctg	gct	1	C	A
TCGA-24-2289	TRPC6	ENST00000344327	p.L368I	c.1102C>A	Unverified	AAA	CTT	GCC	1	C	A
TCGA-29-1766	TRPM3_ENS	ENST00000377110	p.L39I	c.115C>A	Unverified	CCC	CTA	AAC	1	C	A
TCGA-25-2398	TRPV5	ENST00000265310	p.R302R	c.904C>A	Verified	AAA	CGA	GAG	1	C	A
TCGA-13-0886	TRRAP	ENST00000355540	p.L774L	c.2322C>A	Unverified	GAT	CTC	TTG	3	C	A
TCGA-13-0886	TRRAP_ENS	ENST00000359863	p.L774L	c.2322C>A	Unverified	GAT	CTC	TTG	3	C	A
TCGA-61-1900	TSC1	ENST00000298552	p.P670P	c.2010C>A	Unverified	tta	ccc	agc	3	C	A
TCGA-23-1122	TSPEAR	ENST00000323084	p.S631R	c.1893C>A	Unverified	CAC	AGC	CTC	3	C	A
TCGA-23-1021	TSTD2	ENST00000341170	p.L307I	c.919C>A	Verified	ATC	CTT	CTT	1	C	A
TCGA-29-1764	TTC39B	NM_152574.1	p.A310E	c.929C>A	Unverified	gtt	gcg	gaa	2	C	A
TCGA-29-1764	TTC39B_ENS	ENST00000512701	p.A376E	c.1127C>A	Unverified	GTT	GCG	GAA	2	C	A
TCGA-29-1777	TTC7A	ENST00000319190	p.V698V	c.2094C>A	Unverified	TCG	GTC	CTG	3	C	A
TCGA-24-1469	TTC7B	ENST00000328459	p.P209H	c.626C>A	Verified	GCT	CCC	CAC	2	C	A
TCGA-04-1338	TTN	NM_003319	p.R16184S	c.48550C>A	Verified	agc	cgc	tta	1	C	A
TCGA-24-1846	TTN	NM_003319	p.T18292K	c.54875C>A	Unverified	ggt	aca	aag	2	C	A
TCGA-61-1738	TTN	NM_003319	p.S15027*	c.45080C>A	Unverified	gat	tca	aca	2	C	A
TCGA-61-1738	TTN	NM_003319	p.S15027*	c.45080C>A	Unverified	gat	tca	aca	2	C	A
TCGA-29-1775	TTN	NM_003319	p.L10325L	c.30975C>A	Unverified	cac	ctc	gac	3	C	A
TCGA-13-1408	TTN_ENST0(	ENST00000342175	p.P19284T	c.57850C>A	Unverified	GGT	CCT	CCT	1	C	A
TCGA-24-1846	TTN_ENST0(	ENST00000342175	p.T18484K	c.55451C>A	Unverified	GGT	ACA	AAG	2	C	A
TCGA-61-1738	TTN_ENST0(	ENST00000342175	p.S15219*	c.45656C>A	Unverified	GAT	TCA	ACA	2	C	A
TCGA-61-1738	TTN_ENST0(	ENST00000342175	p.S15219*	c.45656C>A	Unverified	GAT	TCA	ACA	2	C	A
TCGA-29-1775	TTN_ENST0(	ENST00000342175	p.L10517L	c.31551C>A	Unverified	CAC	CTC	GAC	3	C	A
TCGA-30-1718	TTN_ENST0(	ENST00000342992	p.L7272I	c.21814C>A	Unverified	GTT	CTC	AAA	1	C	A
TCGA-24-1846	TTN_ENST0(	ENST00000342992	p.T24789K	c.74366C>A	Unverified	GGT	ACA	AAG	2	C	A
TCGA-61-1738	TTN_ENST0(	ENST00000342992	p.S21524*	c.64571C>A	Unverified	GAT	TCA	ACA	2	C	A
TCGA-61-1738	TTN_ENST0(	ENST00000342992	p.S21524*	c.64571C>A	Unverified	GAT	TCA	ACA	2	C	A
TCGA-29-1775	TTN_ENST0(	ENST00000342992	p.L16822L	c.50466C>A	Unverified	CAC	CTC	GAC	3	C	A
TCGA-04-1338	TTN_ENST0(	ENST00000356127	p.R22679S	c.68035C>A	Verified	AGC	CGC	TTA	1	C	A
TCGA-13-1408	TTN_ENST0(	ENST00000356127	p.P25587T	c.76759C>A	Unverified	GGT	CCT	CCT	1	C	A
TCGA-24-1846	TTN_ENST0(	ENST00000356127	p.T24787K	c.74360C>A	Unverified	GGT	ACA	AAG	2	C	A
TCGA-61-1738	TTN_ENST0(	ENST00000356127	p.S21522*	c.64565C>A	Unverified	GAT	TCA	ACA	2	C	A

TCGA-61-1738	TTN_ENST0(	ENST00000356127	p.S21522*	c.64565C>A	Unverified	GAT	TCA	ACA	2	C	A
TCGA-23-1022	TTN_ENST0(	ENST00000356127	p.G16021G	c.48063C>A	Verified	ATT	GGC	CTC	3	C	A
TCGA-29-1775	TTN_ENST0(	ENST00000356127	p.L16820L	c.50460C>A	Unverified	CAC	CTC	GAC	3	C	A
TCGA-24-1846	TTN_ENST0(	ENST00000359218	p.T18417K	c.55250C>A	Unverified	GGT	ACA	AAG	2	C	A
TCGA-61-1738	TTN_ENST0(	ENST00000359218	p.S15152*	c.45455C>A	Unverified	GAT	TCA	ACA	2	C	A
TCGA-61-1738	TTN_ENST0(	ENST00000359218	p.S15152*	c.45455C>A	Unverified	GAT	TCA	ACA	2	C	A
TCGA-29-1775	TTN_ENST0(	ENST00000359218	p.L10450L	c.31350C>A	Unverified	CAC	CTC	GAC	3	C	A
TCGA-09-1665	TTN_ENST0(	ENST00000360870	p.A4869D	c.14606C>A	Verified	GAA	GCT	TCT	2	C	A
TCGA-29-1770	TUBAL3	ENST00000380419	p.S230Y	c.689C>A	Unverified	CCC	TCT	CAT	2	C	A
TCGA-61-1740	TUBB3	ENST00000315491	p.F378L	c.1134C>A	Unverified	CTG	TTC	AAG	3	C	A
TCGA-30-1718	TULP1	ENST00000229771	p.N490K	c.1470C>A	Unverified	AAG	AAC	TTC	3	C	A
TCGA-04-1338	TULP2	ENST00000221399	p.N474K	c.1422C>A	Verified	AAG	AAC	TTC	3	C	A
TCGA-30-1855	TULP4	ENST00000367097	p.I348I	c.1044C>A	Unverified	TCC	ATC	TGC	3	C	A
TCGA-23-1124	UBA7	ENST00000333486	p.N527K	c.1581C>A	Verified	gat	aac	ttt	3	C	A
TCGA-13-0760	UHRF1BP1	ENST00000192788	p.L1093I	c.3277C>A	Verified	CGA	CTC	CGA	1	C	A
TCGA-13-0893	UIMC1	ENST00000377227	p.S27Y	c.80C>A	Verified	AGT	TCT	GTC	2	C	A
TCGA-24-1103	UMODL1	ENST00000400424	p.H693N	c.2077C>A	Verified	TTG	CAC	CTG	1	C	A
TCGA-24-2280	UNC13C	ENST00000260323	p.L597M	c.1789C>A	Unverified	ACC	CTG	TAT	1	C	A
TCGA-20-1685	UNC13C	ENST00000260323	p.Y57*	c.171C>A	Unverified	TCT	TAC	ACT	3	C	A
TCGA-20-1685	UNC13C_EN	ENST00000260323	p.Y57*	c.171C>A	Unverified	TCT	TAC	ACT	3	C	A
TCGA-13-0904	UNC45B	ENST00000394570	p.R261R	c.781C>A	Verified	CAT	CGA	GGG	1	C	A
TCGA-13-0904	UNC45B	ENST00000394570	p.T847T	c.2541C>A	Verified	ACA	ACC	CAG	3	C	A
TCGA-13-0885	UNC5D	ENST00000287272	p.A240D	c.719C>A	Unverified	TCG	GCC	ACT	2	C	A
TCGA-61-1910	UNC5D	ENST00000287272	p.T504N	c.1511C>A	Unverified	AGG	ACT	TTT	2	C	A
TCGA-61-1910	UNC5D_ENS	ENST00000404895	p.T509N	c.1526C>A	Unverified	AGG	ACT	TTT	2	C	A
TCGA-20-0990	USH2A	ENST00000307340	p.P139T	c.415C>A	Verified	TCT	CCT	CCT	1	C	A
TCGA-61-1900	USH2A	ENST00000307340	p.T1143K	c.3428C>A	Unverified	AAG	ACA	AAA	2	C	A
TCGA-61-1915	USH2A	ENST00000307340	p.T911T	c.2733C>A	Unverified	GGG	ACC	ATT	3	C	A
TCGA-13-0906	USP49	ENST00000373006	p.Q448K	c.1342C>A	Unverified	GGG	CAG	CTG	1	C	A
TCGA-20-1687	USP6_ENST(	ENST00000250066	p.L1163L	c.3489C>A	Unverified	TCA	CTC	AGC	3	C	A
TCGA-29-1769	UTP20	ENST00000261637	p.T1129N	c.3386C>A	Unverified	GCA	ACC	GTA	2	C	A
TCGA-29-1693	UTRN	ENST00000367545	p.T1081N	c.3242C>A	Unverified	GAG	ACT	AAT	2	C	A
TCGA-23-1123	VAX1	ENST00000277905	p.S178Y	c.533C>A	Verified	GAC	TCC	GGG	2	C	A
TCGA-23-1123	VAX1	ENST00000277905	p.S178Y	c.533C>A	Verified	GAC	TCC	GGG	2	C	A

TCGA-61-1906	VCL	ENST00000211998	p.T1077N	c.3230C>A	Unverified	CGG	ACC	AAC	2	C	A
TCGA-61-1906	VCL	ENST00000211998	p.T1077N	c.3230C>A	Unverified	CGG	ACC	AAC	2	C	A
TCGA-29-1691	VDAC3	ENST00000022615	p.S205Y	c.614C>A	Unverified	ACA	TCC	ATA	2	C	A
TCGA-13-0807	VN1R4	ENST00000311170	p.I107I	c.321C>A	Verified	GTG	ATC	ACG	3	C	A
TCGA-09-2050	VPS13B	ENST00000358544	p.A3982D	c.11945C>A	Verified	gtg	gct	gca	2	C	A
TCGA-13-1488	VPS13B	ENST00000358544	p.S899Y	c.2696C>A	Verified	cct	tct	gac	2	C	A
TCGA-24-2280	VPS13C	ENST00000261517	p.T3017N	c.9050C>A	Unverified	GGT	ACC	AGA	2	C	A
TCGA-23-1111	VPS37D	ENST00000324941	p.A146D	c.437C>A	Unverified	GAG	GCC	TTC	2	C	A
TCGA-23-2077	VSIG2	ENST00000326621	p.L13M	c.37C>A	Unverified	GCC	CTG	CTA	1	C	A
TCGA-04-1338	VWCE	ENST00000335613	p.A174A	c.522C>A	Unverified	TCT	GCC	GAC	3	C	A
TCGA-23-1111	VWF	ENST00000261405	p.S2137Y	c.6410C>A	Unverified	AGC	TCC	CAC	2	C	A
TCGA-61-2094	WAPAL	ENST00000298767	p.N506K	c.1518C>A	Unverified	ACT	AAC	AAT	3	C	A
TCGA-29-1762	WARS	ENST00000392882	p.C305*	c.915C>A	Unverified	CAG	TGC	CTT	3	C	A
TCGA-61-1910	WDR31	ENST00000374193	p.L3I	c.7C>A	Unverified	CTG	CTA	CTC	1	C	A
TCGA-30-1718	WDR37	ENST00000358220	p.P235T	c.703C>A	Unverified	ACA	CCC	CAG	1	C	A
TCGA-13-1496	WDR45	ENST00000356463	p.S210*	c.629C>A	Verified	gcc	tca	gcc	2	C	A
TCGA-09-2050	WDR49	ENST00000308378	p.P293H	c.878C>A	Verified	TTA	CCT	CCA	2	C	A
TCGA-24-1474	WDR66	ENST00000288912	p.Y293*	c.879C>A	Verified	CAA	TAC	CAC	3	C	A
TCGA-09-1674	WDR69	ENST00000309931	p.I234I	c.702C>A	Unverified	AGA	ATC	ATC	3	C	A
TCGA-13-1497	WDR78	ENST00000371026	p.D118E	c.354C>A	Verified	TTT	GAC	ATA	3	C	A
TCGA-30-1714	WDR91	ENST00000354475	p.Q168K	c.502C>A	Unverified	TTT	CAG	TGC	1	C	A
TCGA-24-2262	WFIKKN2	ENST00000311378	p.C448*	c.1344C>A	Unverified	GCC	TGC	AAG	3	C	A
TCGA-29-1781	WIPF1	ENST00000392546	p.P140H	c.419C>A	Unverified	AAA	CCC	TTT	2	C	A
TCGA-61-2095	WIPF1	ENST00000392546	p.D371E	c.1113C>A	Unverified	AGG	GAC	CCG	3	C	A
TCGA-13-1498	WNK1	NM_018979	p.G803G	c.2409C>A	Verified	caa	ggc	gaa	3	C	A
TCGA-13-1496	WNK3	ENST00000354646	p.L1121L	c.3363C>A	Verified	TTG	CTC	TAT	3	C	A
TCGA-24-2024	WNT6	ENST00000233948	p.L130M	c.388C>A	Verified	CTG	CTG	CAG	1	C	A
TCGA-24-1422	WNT7B	ENST00000339464	p.T319N	c.956C>A	Unverified	TAC	ACC	AAG	2	C	A
TCGA-24-2267	XAGE5	ENST00000351072	p.S73*	c.218C>A	Verified	cag	tca	aag	2	C	A
TCGA-25-2400	XIRP2	ENST00000295237	p.L1917I	c.5749C>A	Verified	GAG	CTT	CTC	1	C	A
TCGA-04-1649	XIRP2	ENST00000295237	p.T1702K	c.5105C>A	Unverified	GAC	ACA	ATT	2	C	A
TCGA-23-1032	XIRP2_ENST	ENST00000409728	p.P597T	c.1789C>A	Verified	TGG	CCA	CCT	1	C	A
TCGA-30-1891	XKRX	NM_212559.1	p.V353V	c.1059C>A	Unverified	ctc	gtc	gac	3	C	A
TCGA-04-1361	XPC	ENST00000285021	p.D121E	c.363C>A	Verified	GAA	GAC	AGC	3	C	A

TCGA-04-1361	XPC	ENST00000285021	p.D121E	c.363C>A	Verified	GAA	GAC	AGC	3	C	A
TCGA-29-1763	YARS	ENST00000373477	p.A291D	c.872C>A	Unverified	ACA	GCT	TAC	2	C	A
TCGA-30-1891	YOD1	ENST00000315927	p.P278T	c.832C>A	Unverified	GAT	CCA	GAT	1	C	A
TCGA-13-0885	ZAN	ENST00000348028	p.H1086N	c.3256C>A	Verified	AAC	CAC	TGC	1	C	A
TCGA-13-1488	ZBED4	ENST00000216268	p.Q691K	c.2071C>A	Verified	CCT	CAG	TAC	1	C	A
TCGA-24-1417	ZBTB16_ENS	ENST00000335953	p.D270E	c.810C>A	Unverified	GGG	GAC	AAG	3	C	A
TCGA-23-1022	ZBTB20	ENST00000357258	p.P623T	c.1867C>A	Verified	ACA	CCC	CCA	1	C	A
TCGA-24-2289	ZBTB33	ENST00000326624	p.T207N	c.620C>A	Unverified	gag	act	ttg	2	C	A
TCGA-09-2051	ZBTB37	ENST00000367702	p.S85Y	c.254C>A	Unverified	CTT	TCT	TTC	2	C	A
TCGA-61-1725	ZBTB37	ENST00000367702	p.A51D	c.152C>A	Unverified	CTG	GCT	GCC	2	C	A
TCGA-61-1725	ZBTB37_ENS	ENST00000427304	p.A51D	c.152C>A	Unverified	CTG	GCT	GCC	2	C	A
TCGA-24-1103	ZBTB38	ENST00000514251	p.T1108N	c.3323C>A	Verified	TCC	ACC	AAA	2	C	A
TCGA-13-0760	ZBTB5	ENST00000307750	p.T121K	c.362C>A	Verified	ACG	ACA	AGG	2	C	A
TCGA-04-1542	ZC3HAV1	ENST00000242351	p.N163K	c.489C>A	Verified	TGT	AAC	CAG	3	C	A
TCGA-23-1021	ZCCHC6	ENST00000375963	p.G900G	c.2700C>A	Verified	TTA	GGC	GAA	3	C	A
TCGA-13-0792	ZDHHC24	ENST00000310442	p.V153V	c.459C>A	Unverified	CAC	GTC	TCT	3	C	A
TCGA-09-1665	ZEB2	ENST00000303660	p.T915N	c.2744C>A	Verified	cag	acc	agt	2	C	A
TCGA-30-1855	ZFHX4	ENST00000521891	p.A2146D	c.6437C>A	Unverified	AGG	GCT	TAT	2	C	A
TCGA-61-2012	ZFHX4	ENST00000521891	p.S720*	c.2159C>A	Unverified	CAG	TCG	GAC	2	C	A
TCGA-61-2012	ZFP64	ENST00000216923	p.P342H	c.1025C>A	Verified	CAT	CCT	GAG	2	C	A
TCGA-61-1913	ZFPM2	ENST00000407775	p.P776T	c.2326C>A	Unverified	GAA	CCC	ACA	1	C	A
TCGA-24-2035	ZFYVE26	ENST00000347230	p.R1428R	c.4282C>A	Unverified	TCC	CGG	GCC	1	C	A
TCGA-25-1313	ZGPAT	ENST00000328969	p.G25G	c.75C>A	Unverified	TTG	GGC	GCC	3	C	A
TCGA-13-1481	ZMIZ1	ENST00000334512	p.C763*	c.2289C>A	Verified	cag	tgc	ttt	3	C	A
TCGA-13-0904	ZNF133	ENST00000377671	p.R524R	c.1570C>A	Verified	TGC	CGA	GAG	1	C	A
TCGA-10-0930	ZNF197	ENST00000344387	p.T28N	c.83C>A	Verified	gga	acc	agc	2	C	A
TCGA-10-0930	ZNF197	ENST00000344387	p.T28N	c.83C>A	Verified	gga	acc	agc	2	C	A
TCGA-24-1103	ZNF197	ENST00000344387	p.F575L	c.1725C>A	Verified	gtt	ttc	att	3	C	A
TCGA-13-0760	ZNF277	ENST00000361822	p.Q130K	c.388C>A	Verified	GAA	CAA	GAG	1	C	A
TCGA-13-1481	ZNF32	ENST00000395797	p.Q74K	c.220C>A	Verified	AGA	CAA	AGG	1	C	A
TCGA-04-1362	ZNF354A	ENST00000335815	p.T377N	c.1130C>A	Verified	CAC	ACT	GGA	2	C	A
TCGA-23-1116	ZNF365	ENST00000395254	p.L194M	c.580C>A	Unverified	GAA	CTG	TTG	1	C	A
TCGA-23-1116	ZNF365_ENS	ENST00000410046	p.L194M	c.580C>A	Unverified	GAA	CTG	TTG	1	C	A
TCGA-30-1714	ZNF366	ENST00000318442	p.Y626*	c.1878C>A	Unverified	TGC	TAC	GAG	3	C	A

TCGA-61-1910	ZNF383	ENST00000352998	p.Q60K	c.178C>A	Unverified	GAA	CAA	GGG	1	C	A
TCGA-23-1021	ZNF449	ENST00000339249	p.Q211K	c.631C>A	Verified	tta	cag	gat	1	C	A
TCGA-61-1738	ZNF462	ENST00000277225	p.D2403E	c.7209C>A	Unverified	TCT	GAC	CAC	3	C	A
TCGA-61-1738	ZNF462	ENST00000277225	p.D2403E	c.7209C>A	Unverified	TCT	GAC	CAC	3	C	A
TCGA-13-0760	ZNF488	ENST00000395702	p.L192M	c.574C>A	Verified	GAG	CTG	TCT	1	C	A
TCGA-24-2289	ZNF521	NM_015461.1	p.N989K	c.2967C>A	Unverified	gga	aac	tgc	3	C	A
TCGA-29-1784	ZNF536	ENST00000355537	p.A46A	c.138C>A	Unverified	CAT	GCC	TTC	3	C	A
TCGA-13-0760	ZNF568	ENST00000333987	p.S166*	c.497C>A	Verified	AGT	TCA	GAC	2	C	A
TCGA-13-0793	ZNF592	ENST00000299927	p.T1024N	c.3071C>A	Verified	CAC	ACC	CCC	2	C	A
TCGA-29-1766	ZNF626_ENS	ENST00000305570	p.L85I	c.253C>A	Unverified	GAC	CTT	TGG	1	C	A
TCGA-25-2391	ZNF629	ENST00000262525	p.N302K	c.906C>A	Unverified	CAG	AAC	CAC	3	C	A
TCGA-61-1740	ZNF644	ENST00000370440	p.H985N	c.2953C>A	Unverified	GGG	CAT	CTT	1	C	A
TCGA-23-1029	ZNF676	ENST00000397121	p.T459T	c.1377C>A	Unverified	TTC	ACC	TGG	3	C	A
TCGA-13-1488	ZNF677	ENST00000333952	p.A439D	c.1316C>A	Verified	AGG	GCT	TTT	2	C	A
TCGA-09-2049	ZNF680	ENST00000309683	p.A19D	c.56C>A	Verified	GTG	GCC	ATA	2	C	A
TCGA-24-1464	ZNF706	ENST00000311212	p.C41*	c.123C>A	Verified	ACC	TGC	ACT	3	C	A
TCGA-13-0920	ZNF711	NM_021998.2	p.L587I	c.1759C>A	Verified	gag	ctt	caa	1	C	A
TCGA-13-0807	ZNF737	ENST00000344519_v61	p.L243I	c.727C>A	Verified	ACA	CAT	AAG	1	C	A
TCGA-61-2102	ZNF761	ENST00000334095	p.S146*	c.437C>A	Verified	TCT	TCA	TTA	2	C	A
TCGA-13-0791	ZNF799	ENST00000357979	p.A299D	c.896C>A	Verified	AAA	GCC	TTC	2	C	A
TCGA-24-1844	ZNF804A	ENST00000302277	p.P1077T	c.3229C>A	Unverified	CCC	CCT	AGC	1	C	A
TCGA-13-0916	ZNF804A	ENST00000302277	p.A217E	c.650C>A	Verified	TTT	GCA	TTT	2	C	A
TCGA-25-2400	ZNF804A	ENST00000302277	p.I212I	c.636C>A	Verified	AAA	ATC	GGC	3	C	A
TCGA-13-0923	ZNF804B	ENST00000333190	p.T685T	c.2055C>A	Verified	ATG	ACC	AGC	3	C	A
TCGA-24-2280	ZNF831	ENST00000371030	p.P1529T	c.4585C>A	Unverified	TCC	CCA	GAC	1	C	A
TCGA-25-2042	ZNRF3	ENST00000406323	p.P805H	c.2414C>A	Unverified	TTC	CCT	AGT	2	C	A
TCGA-13-0795	ZPLD1	ENST00000306176	p.P275T	c.823C>A	Verified	GAC	CCT	CAG	1	C	A
TCGA-24-1850	ZSCAN1	ENST00000282326	p.L220I	c.658C>A	Unverified	GAC	CTT	CTC	1	C	A
TCGA-23-1117	ZSCAN20	ENST00000326544	p.T109N	c.326C>A	Verified	CAG	ACC	TGG	2	C	A
TCGA-23-1117	ZSCAN20	ENST00000326544	p.T109N	c.326C>A	Verified	CAG	ACC	TGG	2	C	A
TCGA-25-1326	AARS	ENST00000261772	p.R962R	c.2886C>G	Unverified	CTG	CGC	CTC	3	C	G
TCGA-25-2042	ABCA12	ENST00000272895	p.L2455V	c.7363C>G	Verified	GCT	CTC	TGT	1	C	G
TCGA-09-2056	ABCA3	ENST00000301732	p.I703M	c.2109C>G	Verified	GCC	ATC	TGG	3	C	G
TCGA-29-1711	ABCA5	ENST00000392676	p.H1394D	c.4180C>G	Unverified	GAA	CAT	TTT	1	C	G

TCGA-29-1711	ABCA5	ENST00000392676	p.H1394D	c.4180C>G	Unverified	GAA	CAT	TTT	1	C	G
TCGA-13-0900	ABCB4	ENST00000359206	p.T685S	c.2054C>G	Verified	GAA	ACC	GAT	2	C	G
TCGA-20-1685	ABCB5	ENST00000258738	p.Q500E	c.1498C>G	Unverified	ATT	CAA	GCT	1	C	G
TCGA-20-1685	ABCB5_ENS[illegible]	ENST00000404938	p.Q945E	c.2833C>G	Unverified	ATT	CAA	GCT	1	C	G
TCGA-36-1577	ABCC2	ENST00000370449	p.S789C	c.2366C>G	Verified	CTG	TCT	GCA	2	C	G
TCGA-09-2044	ABCF3	ENST00000429586	p.T533T	c.1599C>G	Verified	TCT	ACC	ATG	3	C	G
TCGA-13-0903	ABI3BP	ENST00000284322	p.D203E	c.609C>G	Verified	AAA	GAC	AAT	3	C	G
TCGA-13-1496	AC013469_[illegible]	ENST00000408934	p.G17G	c.51C>G	Verified	CAC	GGC	TTT	3	C	G
TCGA-29-1766	AC022601.1	ENST00000554864	p.T179R	c.536C>G	Unverified	CCC	ACG	GCA	2	C	G
TCGA-29-1766	AC022601.1[illegible]	ENST00000554864_v68	p.T179R	c.536C>G	Unverified	CCC	ACG	GCA	2	C	G
TCGA-10-0930	ACACB	ENST00000377848	p.T1128R	c.3383C>G	Verified	AAA	ACA	GTG	2	C	G
TCGA-10-0930	ACACB	ENST00000377848	p.T1128R	c.3383C>G	Verified	AAA	ACA	GTG	2	C	G
TCGA-29-1769	ACACB	ENST00000377848	p.P1260R	c.3779C>G	Unverified	TGC	CCC	GAG	2	C	G
TCGA-24-2280	ACADSB	ENST00000358776	p.T155R	c.464C>G	Unverified	GGA	ACA	GAA	2	C	G
TCGA-13-1510	ACD	ENST00000219251	p.L95V	c.283C>G	Unverified	GTC	CTA	CGG	1	C	G
TCGA-13-1501	ACLY	ENST00000352035	p.A1048G	c.3143C>G	Verified	gaa	gct	gat	2	C	G
TCGA-13-1501	ACLY	ENST00000352035	p.A1048G	c.3143C>G	Verified	gaa	gct	gat	2	C	G
TCGA-23-1022	ACOT9	ENST00000336430	p.A205G	c.614C>G	Verified	gtg	gct	cgt	2	C	G
TCGA-29-2427	ACRC	ENST00000373695	p.P405A	c.1213C>G	Verified	GCA	CCT	GTG	1	C	G
TCGA-13-0884	ACSM5	ENST00000331849	p.I509M	c.1527C>G	Verified	CCC	ATC	AGG	3	C	G
TCGA-25-2391	ACTG1	ENST00000331925	p.L180V	c.538C>G	Unverified	GAC	CTG	GCT	1	C	G
TCGA-13-0919	ACTR1A	ENST00000369905	p.T340R	c.1019C>G	Unverified	TCC	ACG	TGG	2	C	G
TCGA-25-1313	ADAM30	ENST00000369400	p.A437G	c.1310C>G	Verified	ggt	gcc	aac	2	C	G
TCGA-25-2042	ADAMTS1	ENST00000284984	p.L185V	c.553C>G	Unverified	CAC	CTC	CTG	1	C	G
TCGA-25-1313	ADAMTS12	ENST00000504830	p.N494K	c.1482C>G	Verified	gaa	aac	gtc	3	C	G
TCGA-04-1362	ADAMTS3	ENST00000286657	p.P736A	c.2206C>G	Verified	CCC	CCT	GGG	1	C	G
TCGA-29-1699	ADAMTS3	ENST00000286657	p.T981T	c.2943C>G	Unverified	GTG	ACC	TGC	3	C	G
TCGA-20-0991	ADAMTS4	ENST00000367996	p.H486Q	c.1458C>G	Verified	AAA	CAC	TCG	3	C	G
TCGA-61-1737	ADAMTS4	ENST00000367996	p.S385R	c.1155C>G	Unverified	TTG	AGC	ACC	3	C	G
TCGA-61-1737	ADAMTS4_[illegible]	ENST00000367996	p.S385R	c.1155C>G	Unverified	TTG	AGC	ACC	3	C	G
TCGA-04-1649	ADAMTS9	ENST00000498707	p.S290S	c.870C>G	Unverified	tta	tcc	tat	3	C	G
TCGA-13-0755	ADAMTSL3	ENST00000286744	p.R938G	c.2812C>G	Unverified	CGA	CGA	TTC	1	C	G
TCGA-13-0893	ADAMTSL3	ENST00000286744	p.I195M	c.585C>G	Verified	TGT	ATC	AGT	3	C	G
TCGA-61-1737	ADAMTSL4	ENST00000369038	p.P154A	c.460C>G	Unverified	AAG	CCA	GGA	1	C	G

TCGA-13-0905	ADC	ENST00000373443	p.P75R	c.224C>G	Verified	AGC	CCA	GGT	2	C	G
TCGA-23-1114	ADCY2	ENST00000338316	p.L315V	c.943C>G	Unverified	ATG	CTG	AAT	1	C	G
TCGA-04-1651	ADCY9	ENST00000294016	p.G402G	c.1206C>G	Unverified	GTG	GGC	TTC	3	C	G
TCGA-04-1651	ADCY9	ENST00000294016	p.G402G	c.1206C>G	Unverified	GTG	GGC	TTC	3	C	G
TCGA-13-1488	ADRA1A	ENST00000380586	p.H256Q	c.768C>G	Verified	ACG	CAC	TTC	3	C	G
TCGA-24-1103	ADSS	ENST00000366535	p.T361T	c.1083C>G	Verified	ctt	acc	aag	3	C	G
TCGA-23-1110	AHNAK	ENST00000378024	p.S5125C	c.15374C>G	Verified	CTT	TCT	TTG	2	C	G
TCGA-13-0924	AHNAK	ENST00000378024	p.I2702M	c.8106C>G	Verified	AAT	ATC	AAA	3	C	G
TCGA-24-1417	AHNAK	ENST00000378024	p.V2131V	c.6393C>G	Unverified	AAA	GTC	AAA	3	C	G
TCGA-24-2280	AIG1	ENST00000357847	p.L42V	c.124C>G	Unverified	TTC	CTG	ACG	1	C	G
TCGA-13-1501	AK3	ENST00000381809	p.Q179E	c.535C>G	Verified	GAC	CAA	ACA	1	C	G
TCGA-13-1501	AK3	ENST00000381809	p.Q179E	c.535C>G	Verified	GAC	CAA	ACA	1	C	G
TCGA-61-1904	AKAP13	ENST00000361243	p.P733A	c.2197C>G	Unverified	tgt	cct	ttc	1	C	G
TCGA-61-1904	AKAP13_ENS	ENST00000394518	p.P733A	c.2197C>G	Unverified	TGT	CCT	TTC	1	C	G
TCGA-23-1022	AKAP6	ENST00000280979	p.S2179C	c.6536C>G	Verified	GAA	TCT	GCA	2	C	G
TCGA-04-1362	AKAP7	NM_016377.2	p.P269P	c.807C>G	Verified	gag	ccc	gat	3	C	G
TCGA-29-1769	AKAP7	NM_016377.2	p.S307R	c.921C>G	Unverified	ggg	agc	tct	3	C	G
TCGA-29-1769	AKAP7_ENST	ENST00000474850	p.S85R	c.255C>G	Unverified	GGG	AGC	TCT	3	C	G
TCGA-23-1123	AKNA	ENST00000307564	p.P226R	c.677C>G	Verified	GGC	CCC	CAG	2	C	G
TCGA-23-1123	AKNA	ENST00000307564	p.P226R	c.677C>G	Verified	GGC	CCC	CAG	2	C	G
TCGA-13-0885	ALDH2	ENST00000261733	p.L189L	c.567C>G	Verified	CCG	CTC	CTG	3	C	G
TCGA-13-1497	ALG11	ENST00000521508	p.Q90E	c.268C>G	Verified	CTG	CAG	AAA	1	C	G
TCGA-24-1103	ALG8	ENST00000299626	p.R41G	c.121C>G	Verified	CAC	CGA	AAC	1	C	G
TCGA-29-1695	ALMS1	ENST00000264448	p.L2264V	c.6790C>G	Unverified	ACA	CTT	TTG	1	C	G
TCGA-24-1604	ALOX5	ENST00000374391	p.T138R	c.413C>G	Verified	gaa	aca	cgg	2	C	G
TCGA-13-0916	ALPK2	NM_052947	p.S504C	c.1511C>G	Verified	ctg	tcc	cag	2	C	G
TCGA-13-0916	ALPK2_ENST	ENST00000361673	p.S1143C	c.3428C>G	Verified	CTG	TCC	CAG	2	C	G
TCGA-30-1714	ALPP	ENST00000392027	p.D450E	c.1350C>G	Unverified	CTG	GAC	GAA	3	C	G
TCGA-29-1784	AMFR	ENST00000290649	p.L354V	c.1060C>G	Unverified	AAA	CTG	CCC	1	C	G
TCGA-13-0906	ANK3	ENST00000280772	p.L1171V	c.3511C>G	Verified	GCC	CTA	ACT	1	C	G
TCGA-13-1489	ANK3	ENST00000280772	p.H3008Q	c.9024C>G	Verified	CAG	CAC	TTT	3	C	G
TCGA-13-0793	ANKAR	ENST00000313581	p.Q1254E	c.3760C>G	Verified	TTT	CAA	ATG	1	C	G
TCGA-24-1103	ANKAR	ENST00000313581	p.L1109V	c.3325C>G	Verified	TTT	CTT	GAA	1	C	G
TCGA-13-0792	ANKHD1	ENST00000360839	p.N1477K	c.4431C>G	Verified	gaa	aac	aaa	3	C	G

TCGA-13-0792	ANKHD1-EIF	ENST00000297183	p.N1477K	c.4431C>G	Verified	GAA	AAC	AAA	3	C	G
TCGA-04-1367	ANKRD11	ENST00000301030	p.S1518C	c.4553C>G	Verified	AAG	TCC	AGG	2	C	G
TCGA-61-1907	ANKRD12	ENST00000262126	p.Q455E	c.1363C>G	Unverified	ATG	CAA	ACC	1	C	G
TCGA-13-0900	ANKRD17	NM_032217.3	p.S2255R	c.6765C>G	Verified	ttt	agc	aca	3	C	G
TCGA-13-1510	ANKRD27	ENST00000306065	p.L619V	c.1855C>G	Unverified	CAC	CTG	TCC	1	C	G
TCGA-61-2113	ANKS4B	ENST00000311620	p.P299R	c.896C>G	Verified	CTG	CCC	AGT	2	C	G
TCGA-23-1110	ANKS6	ENST00000353234	p.A693G	c.2078C>G	Verified	CCA	GCA	CCG	2	C	G
TCGA-24-2024	AP2A1	ENST00000359032	p.N103K	c.309C>G	Verified	GTG	AAC	TCG	3	C	G
TCGA-29-1776	APC	ENST00000457016	p.P2808A	c.8422C>G	Unverified	ACT	CCA	GTG	1	C	G
TCGA-23-1022	APC	ENST00000457016	p.S2005*	c.6014C>G	Verified	GCA	TCA	GGC	2	C	G
TCGA-30-1855	APC	ENST00000457016	p.A928A	c.2784C>G	Unverified	GCT	GCC	CAT	3	C	G
TCGA-23-1029	APCS	ENST00000255040	p.D180E	c.540C>G	Unverified	TGG	GAC	TCT	3	C	G
TCGA-04-1356	APLP1	ENST00000221891	p.L603L	c.1809C>G	Verified	CTG	CTC	CTG	3	C	G
TCGA-29-1764	APOB	ENST00000233242	p.T2596S	c.7787C>G	Unverified	GGG	ACC	ATG	2	C	G
TCGA-13-1496	APOB	ENST00000233242	p.I4266M	c.12798C>G	Verified	GTA	ATC	TCG	3	C	G
TCGA-25-1313	APOLD1	ENST00000356591	p.F141L	c.423C>G	Verified	TTT	TTC	TGC	3	C	G
TCGA-23-1124	ARFIP1	ENST00000353617	p.Q294E	c.880C>G	Verified	TCA	CAG	CAT	1	C	G
TCGA-04-1367	ARHGAP21	NM_020824.2	p.S1224S	c.3672C>G	Verified	aaa	tcc	ttc	3	C	G
TCGA-13-1509	ARHGAP21	NM_020824.2	p.N1061K	c.3183C>G	Verified	tac	aac	aat	3	C	G
TCGA-23-1022	ARHGAP30	ENST00000368013	p.Q777E	c.2329C>G	Verified	GAT	CAA	GTT	1	C	G
TCGA-23-1117	ARHGEF15	ENST00000361926	p.P54A	c.160C>G	Verified	gca	cca	acc	1	C	G
TCGA-23-1117	ARHGEF15	ENST00000361926	p.P54A	c.160C>G	Verified	gca	cca	acc	1	C	G
TCGA-24-2288	ARHGEF19	ENST00000270747	p.R185G	c.553C>G	Unverified	acc	cga	gtg	1	C	G
TCGA-13-0900	ARHGEF40	ENST00000298694	p.P655A	c.1963C>G	Verified	AAG	CCA	GAG	1	C	G
TCGA-10-0930	ARID1A	ENST00000324856	p.S664*	c.1991C>G	Verified	aca	tca	ggg	2	C	G
TCGA-10-0930	ARID1A	ENST00000324856	p.S664*	c.1991C>G	Verified	aca	tca	ggg	2	C	G
TCGA-13-1505	ARL15_ENST	ENST00000504924	p.L11V	c.31C>G	Unverified	TTT	CTG	TAC	1	C	G
TCGA-24-2267	ARL4D	ENST00000320033	p.P17R	c.50C>G	Verified	ttg	ccc	cac	2	C	G
TCGA-13-0919	ARMCX2	ENST00000330154	p.T358T	c.1074C>G	Verified	gag	acc	cag	3	C	G
TCGA-61-2012	ARMCX3	ENST00000341189	p.A96G	c.287C>G	Verified	agg	gcc	agg	2	C	G
TCGA-23-1114	ARSJ	ENST00000315366	p.I144M	c.432C>G	Unverified	TCT	ATC	ATA	3	C	G
TCGA-29-1784	ASAP3	ENST00000336689	p.N730K	c.2190C>G	Unverified	AGC	AAC	AAG	3	C	G
TCGA-04-1638	ASL	ENST00000304874	p.A390G	c.1169C>G	Unverified	GAG	GCC	TCC	2	C	G
TCGA-23-1022	ASNS	ENST00000175506	p.Y488*	c.1464C>G	Verified	GAA	TAC	GTT	3	C	G

TCGA-23-1022	ASNS_ENST(	ENST00000394309	p.Y488*	c.1464C>G	Verified	GAA	TAC	GTT	3	C	G
TCGA-29-1776	ASPSCR1_EN	ENST00000306729	p.V564V	c.1692C>G	Unverified	GGT	GTC	TAC	3	C	G
TCGA-24-2262	ASRGL1	ENST00000301776	p.P135A	c.403C>G	Unverified	ATT	CCT	GGA	1	C	G
TCGA-13-0885	ASTN1	ENST00000361833	p.V186V	c.558C>G	Verified	cgg	gtc	ccg	3	C	G
TCGA-29-1761	ASXL3_ENST	ENST00000269197	p.I1102M	c.3306C>G	Unverified	CAC	ATC	AAA	3	C	G
TCGA-23-1124	ATAD2B_EN	ENST00000238789	p.S1202C	c.3605C>G	Verified	TTA	TCT	ATG	2	C	G
TCGA-13-0807	ATF7	ENST00000328463	p.L109V	c.325C>G	Verified	TCT	CTG	CCT	1	C	G
TCGA-24-1849	ATG4D	ENST00000309469	p.C330W	c.990C>G	Unverified	CTG	TGC	CTG	3	C	G
TCGA-61-1914	ATP11C	ENST00000327569	p.R19G	c.55C>G	Unverified	AAA	CGA	GTT	1	C	G
TCGA-24-1474	ATP11C	ENST00000327569	p.S570W	c.1709C>G	Verified	GAC	TCG	GCA	2	C	G
TCGA-61-1914	ATP11C_ENS	ENST00000361648	p.R19G	c.55C>G	Unverified	AAA	CGA	GTT	1	C	G
TCGA-04-1530	ATP13A2	ENST00000326735	p.P606A	c.1816C>G	Unverified	CCC	CCA	CTT	1	C	G
TCGA-61-1895	ATP8B1	ENST00000283684	p.P610A	c.1828C>G	Unverified	ACC	CCA	GAA	1	C	G
TCGA-61-2102	ATP9B	ENST00000307671	p.Q42E	c.124C>G	Verified	tac	cag	ctg	1	C	G
TCGA-04-1367	ATXN10	ENST00000252934	p.S127C	c.380C>G	Verified	GAA	TCT	CTG	2	C	G
TCGA-13-0893	ATXN2L	ENST00000336783	p.P783A	c.2347C>G	Verified	ACG	CCC	TAT	1	C	G
TCGA-29-1696	ATXN7L2	ENST00000369870	p.P73R	c.218C>G	Unverified	TGC	CCT	GCC	2	C	G
TCGA-29-1696	ATXN7L2	ENST00000369870	p.P73R	c.218C>G	Unverified	TGC	CCT	GCC	2	C	G
TCGA-24-1847	ATXN7L3	ENST00000389384	p.G46G	c.138C>G	Unverified	TGT	GGC	TAC	3	C	G
TCGA-13-0761	AURKC	ENST00000448930	p.H52D	c.154C>G	Unverified	GAG	CAC	CAG	1	C	G
TCGA-13-0761	AURKC	ENST00000448930	p.H52D	c.154C>G	Unverified	GAG	CAC	CAG	1	C	G
TCGA-23-1032	AZI1	ENST00000374782	p.L919V	c.2755C>G	Unverified	CAG	CTT	GGG	1	C	G
TCGA-23-2078	B3GALT4	ENST00000451237	p.L16V	c.46C>G	Unverified	CTG	CTG	GTG	1	C	G
TCGA-04-1367	B3GAT2	ENST00000230053	p.L277V	c.829C>G	Verified	TTT	CTC	AAA	1	C	G
TCGA-13-0905	B3GAT3	ENST00000265471	p.N162K	c.486C>G	Unverified	CGG	AAC	AAG	3	C	G
TCGA-23-1117	BACE1	ENST00000313005	p.A419G	c.1256C>G	Verified	AGC	GCT	TGC	2	C	G
TCGA-23-1117	BACE1	ENST00000313005	p.A419G	c.1256C>G	Verified	AGC	GCT	TGC	2	C	G
TCGA-13-0760	BAI3	ENST00000370598	p.H577D	c.1729C>G	Verified	CAG	CAT	TCA	1	C	G
TCGA-30-1714	BARHL2	ENST00000370445	p.P225R	c.674C>G	Unverified	AGT	CCC	CCT	2	C	G
TCGA-13-0755	BARHL2	ENST00000370445	p.Y304*	c.912C>G	Verified	AAC	TAC	TCG	3	C	G
TCGA-30-1856	BAZ2B	ENST00000392783	p.L95V	c.283C>G	Unverified	ACA	CTT	GGT	1	C	G
TCGA-13-0762	BBS10	ENST00000393262	p.P458R	c.1373C>G	Verified	GCA	CCA	GAT	2	C	G
TCGA-13-0762	BBS10	ENST00000393262	p.P458R	c.1373C>G	Verified	GCA	CCA	GAT	2	C	G
TCGA-24-1847	BCAN	ENST00000329117	p.A369G	c.1106C>G	Unverified	CCA	GCC	TCC	2	C	G

TCGA-30-1891	BCAN	ENST00000329117	p.S419C	c.1256C>G	Unverified	AGC	TCC	ACT	2	C	G
TCGA-20-0990	BCAN	ENST00000329117	p.Y104*	c.312C>G	Verified	GCC	TAC	CGG	3	C	G
TCGA-24-1847	BCAN_ENST	ENST00000361588	p.A369G	c.1106C>G	Unverified	CCA	GCC	TCC	2	C	G
TCGA-04-1347	BCAR3	ENST00000260502	p.Q197E	c.589C>G	Verified	gct	cag	cac	1	C	G
TCGA-24-1604	BCCIP	ENST00000299130	p.L150V	c.448C>G	Verified	GTT	CTA	CGC	1	C	G
TCGA-61-1740	BCL9L	NM_182557.1	p.P694P	c.2082C>G	Unverified	cgc	ccc	ctg	3	C	G
TCGA-61-1740	BCL9L_ENST	ENST00000392849	p.P694P	c.2082C>G	Unverified	CGC	CCC	CTG	3	C	G
TCGA-29-1768	BCORL1	ENST00000218147	p.D100E	c.300C>G	Unverified	atg	gac	tac	3	C	G
TCGA-25-1318	BEND2	ENST00000380033	p.A383G	c.1148C>G	Verified	CCA	GCC	TCT	2	C	G
TCGA-25-1318	BEND2	ENST00000380033	p.A383G	c.1148C>G	Verified	CCA	GCC	TCT	2	C	G
TCGA-10-0930	BHLHB9	ENST00000457056	p.S235C	c.704C>G	Verified	AGA	TCC	CAG	2	C	G
TCGA-10-0930	BHLHB9	ENST00000457056	p.S235C	c.704C>G	Verified	AGA	TCC	CAG	2	C	G
TCGA-24-1469	BHLHE40	ENST00000256495	p.P324A	c.970C>G	Verified	CTG	CCC	TTC	1	C	G
TCGA-23-1124	BHMT	ENST00000274353	p.T339S	c.1016C>G	Verified	CAC	ACC	AAA	2	C	G
TCGA-13-0886	BIN1	ENST00000316724	p.V281V	c.843C>G	Unverified	acg	gtc	aag	3	C	G
TCGA-13-0884	BIRC3	ENST00000263464	p.S248C	c.743C>G	Verified	gtt	tct	aat	2	C	G
TCGA-23-1110	BIRC3	ENST00000263464	p.T536R	c.1607C>G	Verified	ccc	aca	gaa	2	C	G
TCGA-23-1031	BIRC6	ENST00000421745	p.Q1771E	c.5311C>G	Verified	CTT	CAA	CCT	1	C	G
TCGA-23-1124	BMP2K_ENS	ENST00000335016	p.T360S	c.1079C>G	Verified	GAT	ACC	ATT	2	C	G
TCGA-23-1022	BMPER	ENST00000297161	p.L93V	c.277C>G	Verified	GCC	CTG	GCC	1	C	G
TCGA-61-1733	BMS1	ENST00000374518	p.S122*	c.365C>G	Unverified	GTG	TCA	GGT	2	C	G
TCGA-61-1733	BMS1	ENST00000374518	p.S122*	c.365C>G	Unverified	GTG	TCA	GGT	2	C	G
TCGA-13-0920	BNC2	ENST00000380672	p.P382R	c.1145C>G	Verified	ACA	CCC	AAT	2	C	G
TCGA-09-2050	BPIL1	ENST00000170150	p.F99L	c.297C>G	Verified	ACT	TTC	AAG	3	C	G
TCGA-29-1781	BPTF	ENST00000306378	p.Q2286E	c.6856C>G	Unverified	CCC	CAG	TCC	1	C	G
TCGA-29-1781	BPTF_ENST0	ENST00000335221	p.Q2412E	c.7234C>G	Unverified	CCC	CAG	TCC	1	C	G
TCGA-04-1347	BRAF	ENST00000288602	p.S637*	c.1910C>G	Verified	cag	tca	gat	2	C	G
TCGA-04-1331	BRPF1	ENST00000383829	p.H541Q	c.1623C>G	Verified	CTG	CAC	AGC	3	C	G
TCGA-13-0885	BRS3	ENST00000370648	p.S231C	c.692C>G	Verified	ctc	tct	att	2	C	G
TCGA-13-0807	BRWD3	ENST00000373275	p.S1500*	c.4499C>G	Verified	GTT	TCA	GAT	2	C	G
TCGA-61-1915	BSN	ENST00000296452	p.P1410R	c.4229C>G	Unverified	AGT	CCT	TCA	2	C	G
TCGA-61-1907	BSND	ENST00000371265	p.L141V	c.421C>G	Unverified	AAG	CTG	GGA	1	C	G
TCGA-10-0930	BTBD1	ENST00000261721	p.H174D	c.520C>G	Verified	AAA	CAT	CTT	1	C	G
TCGA-10-0930	BTBD1	ENST00000261721	p.H174D	c.520C>G	Verified	AAA	CAT	CTT	1	C	G

TCGA-04-1347	BTBD3	ENST00000378226	p.T218S	c.653C>G	Verified	GAG	ACC	AGC	2	C	G
TCGA-29-1784	BTN3A3	ENST00000244519	p.L532V	c.1594C>G	Unverified	TCC	CTG	GAG	1	C	G
TCGA-25-1313	BZRAP1	ENST00000343736	p.S1768S	c.5304C>G	Unverified	CAC	TCC	ATG	3	C	G
TCGA-30-1856	C10orf18	ENST00000328090	p.S2014C	c.6041C>G	Unverified	ATC	TCC	ATT	2	C	G
TCGA-24-1422	C10orf46	ENST00000369151	p.P70A	c.208C>G	Unverified	GGC	CCC	AAG	1	C	G
TCGA-25-2392	C11orf65	ENST00000393084	p.A59G	c.176C>G	Unverified	GAG	GCA	GAG	2	C	G
TCGA-09-2049	C12orf35	ENST00000312561	p.A586G	c.1757C>G	Verified	CTC	GCT	TTG	2	C	G
TCGA-09-2051	C12orf35	ENST00000312561	p.A389G	c.1166C>G	Unverified	GTT	GCA	AAA	2	C	G
TCGA-25-2398	C13orf23	ENST00000352251	p.P797R	c.2390C>G	Unverified	ACC	CCA	AGC	2	C	G
TCGA-25-1326	C13orf23	ENST00000352251	p.P148P	c.444C>G	Verified	TAT	CCC	AAA	3	C	G
TCGA-24-1422	C14orf181	ENST00000328595	p.P41R	c.122C>G	Unverified	TCC	CCA	CGA	2	C	G
TCGA-24-1435	C14orf39	ENST00000321731	p.Q561E	c.1681C>G	Verified	GGA	CAG	GGT	1	C	G
TCGA-24-1104	C14orf93	ENST00000397379	p.L246V	c.736C>G	Verified	AAG	CTG	CCA	1	C	G
TCGA-24-1423	C15orf29	ENST00000256544	p.L222V	c.664C>G	Verified	CCT	CTA	GTA	1	C	G
TCGA-09-2056	C16orf62	ENST00000251143	p.S98C	c.293C>G	Verified	AGC	TCC	AGA	2	C	G
TCGA-25-1326	C17orf63	ENST00000452648	p.P452A	c.1354C>G	Verified	CAA	CCC	CTG	1	C	G
TCGA-24-1845	C18orf1	ENST00000361205	p.D43E	c.129C>G	Unverified	agt	gac	gaa	3	C	G
TCGA-24-1470	C18orf54	ENST00000300091	p.P228A	c.682C>G	Verified	AGT	CCT	TGC	1	C	G
TCGA-29-1763	C19orf2	ENST00000542441	p.R406G	c.1216C>G	Unverified	cct	cgc	aaa	1	C	G
TCGA-13-1510	C1orf112	ENST00000286031	p.A684G	c.2051C>G	Unverified	GAA	GCT	ATC	2	C	G
TCGA-23-2077	C1orf141	ENST00000371007	p.P273A	c.817C>G	Verified	ATG	CCT	ACA	1	C	G
TCGA-13-1481	C1orf183	ENST00000357260	p.P93R	c.278C>G	Verified	tcc	ccc	tcc	2	C	G
TCGA-13-1498	C1orf226	ENST00000426197	p.L284V	c.850C>G	Verified	AGC	CTG	GCT	1	C	G
TCGA-25-1313	C1orf56	ENST00000368926	p.T31T	c.93C>G	Unverified	CCG	ACC	GAA	3	C	G
TCGA-13-0900	C1orf95	ENST00000366788	p.Y48*	c.144C>G	Unverified	CCC	TAC	ATG	3	C	G
TCGA-13-1505	C1QBP	ENST00000225698	p.A125A	c.375C>G	Verified	GTT	GCC	GGG	3	C	G
TCGA-61-2113	C21orf124	ENST00000291565	p.P286R	c.857C>G	Unverified	AGC	CCC	ATG	2	C	G
TCGA-24-2035	C21orf91	ENST00000284881	p.Q134E	c.400C>G	Verified	CCA	CAG	TTT	1	C	G
TCGA-25-2393	C22orf42	ENST00000382097	p.A219G	c.656C>G	Verified	ATG	GCA	AAG	2	C	G
TCGA-20-1685	C2orf29	ENST00000289382	p.D248E	c.744C>G	Unverified	CCA	GAC	CCG	3	C	G
TCGA-09-2056	C2orf53	ENST00000335524	p.P349A	c.1045C>G	Unverified	GAC	CCA	GTC	1	C	G
TCGA-24-2035	C2orf63	ENST00000407122	p.T156S	c.467C>G	Verified	TGT	ACT	TTC	2	C	G
TCGA-24-2035	C2orf63_EN:	ENST00000407122	p.T156S	c.467C>G	Verified	TGT	ACT	TTC	2	C	G
TCGA-24-2280	C3orf32	ENST00000341795	p.P16R	c.47C>G	Unverified	tgg	cct	cag	2	C	G

TCGA-29-1775	C3orf32	ENST00000341795	p.S179S	c.537C>G	Unverified	cca	tcc	tgc	3	C	G
TCGA-24-2267	C3orf37	ENST00000389735	p.P346R	c.1037C>G	Verified	GAA	CCT	GTG	2	C	G
TCGA-25-1326	C3orf45	ENST00000316436	p.S150R	c.450C>G	Verified	CTC	AGC	CAG	3	C	G
TCGA-29-1775	C3P1	ENST00000333905	p.A113G	c.338C>G	Unverified	AGT	GCG	TCA	2	C	G
TCGA-29-1781	C4orf27	ENST00000393381	p.A88G	c.263C>G	Unverified	CTT	GCT	GGA	2	C	G
TCGA-29-1776	C4orf50	ENST00000324058	p.S260R	c.780C>G	Unverified	CAA	AGC	ATT	3	C	G
TCGA-29-1776	C4orf50_EN:	ENST00000531445	p.S734R	c.2202C>G	Unverified	CAA	AGC	ATT	3	C	G
TCGA-29-1785	C5	ENST00000223642	p.Q785E	c.2353C>G	Unverified	AAA	CAG	TTG	1	C	G
TCGA-29-1785	C5	ENST00000223642	p.Q785E	c.2353C>G	Unverified	AAA	CAG	TTG	1	C	G
TCGA-29-2427	C5	ENST00000223642	p.A1252A	c.3756C>G	Verified	ACT	GCC	TAT	3	C	G
TCGA-24-2280	C5orf34	ENST00000306862	p.T87S	c.260C>G	Unverified	GAA	ACC	ATC	2	C	G
TCGA-24-2267	C5orf48	ENST00000357147	p.P103A	c.307C>G	Verified	GGC	CCA	CCA	1	C	G
TCGA-20-1685	C6orf114	ENST00000379278	p.T22T	c.66C>G	Unverified	CAG	ACC	CAG	3	C	G
TCGA-25-2042	C6orf138	ENST00000339488	p.L245V	c.733C>G	Unverified	ATC	CTG	ACC	1	C	G
TCGA-13-1481	C6orf222	ENST00000437635	p.R202G	c.604C>G	Verified	GCT	CGC	AGG	1	C	G
TCGA-24-1463	C7orf26	ENST00000344417	p.L285V	c.853C>G	Verified	CAC	CTC	TTG	1	C	G
TCGA-24-1103	C7orf47	ENST00000292330	p.P228R	c.683C>G	Verified	CCC	CCT	CTC	2	C	G
TCGA-09-2050	C8orf31	ENST00000395172	p.P50P	c.150C>G	Verified	TCT	CCC	TTG	3	C	G
TCGA-09-2051	C8orf73	ENST00000398882	p.Q272E	c.814C>G	Unverified	ACA	CAG	CTG	1	C	G
TCGA-04-1342	C9orf16	ENST00000372994	p.P83P	c.249C>G	Unverified	AGC	CCC	TAG	3	C	G
TCGA-29-1761	C9orf43	ENST00000374165	p.T190T	c.570C>G	Unverified	CCA	ACC	CCA	3	C	G
TCGA-20-1686	C9orf79	ENST00000325643	p.P664R	c.1991C>G	Unverified	AGG	CCC	CAG	2	C	G
TCGA-61-2012	C9orf89	ENST00000375464	p.L95V	c.283C>G	Unverified	CGC	CTG	CCC	1	C	G
TCGA-13-1488	CACHD1	ENST00000290039	p.V660V	c.1980C>G	Verified	CAC	GTC	ACA	3	C	G
TCGA-61-1913	CACNA1E	ENST00000367570	p.T49S	c.146C>G	Unverified	CGG	ACT	ATG	2	C	G
TCGA-61-1913	CACNA1E_EI	ENST00000367573	p.T49S	c.146C>G	Unverified	CGG	ACT	ATG	2	C	G
TCGA-13-0906	CACNA1F	ENST00000376265	p.D314E	c.942C>G	Unverified	ttt	gac	aac	3	C	G
TCGA-29-1761	CACNA1G	ENST00000359106	p.P2315A	c.6943C>G	Unverified	GAC	CCC	CCC	1	C	G
TCGA-29-1761	CACNA1G_E	ENST00000354983	p.P2281A	c.6841C>G	Unverified	GAC	CCC	CCC	1	C	G
TCGA-29-1761	CACNA1G_E	ENST00000359106	p.P2315A	c.6943C>G	Unverified	GAC	CCC	CCC	1	C	G
TCGA-24-2280	CACNA1G_E	ENST00000359106	p.A1585G	c.4754C>G	Unverified	GCT	GCG	TCA	2	C	G
TCGA-25-1318	CACNA1G_E	ENST00000359106	p.S1263C	c.3788C>G	Verified	CAG	TCC	AGG	2	C	G
TCGA-25-1318	CACNA1G_E	ENST00000359106	p.S1263C	c.3788C>G	Verified	CAG	TCC	AGG	2	C	G
TCGA-24-1850	CACNA1S	ENST00000362061	p.R1229G	c.3685C>G	Unverified	GCC	CGC	ATC	1	C	G

TCGA-61-2113	CAMSAP1	ENST00000389532	p.S835R	c.2505C>G	Unverified	ACC	AGC	AGC	3	C	G
TCGA-61-2009	CAPN13	ENST00000295055	p.F130L	c.390C>G	Verified	CGG	TTC	TGG	3	C	G
TCGA-61-1914	CARM1	ENST00000327064	p.I210M	c.630C>G	Unverified	AAA	ATC	TAC	3	C	G
TCGA-61-1914	CASC1	ENST00000320267	p.Q190E	c.568C>G	Unverified	AAA	CAA	GCT	1	C	G
TCGA-61-1914	CASC1_ENST	ENST00000354189	p.Q254E	c.760C>G	Unverified	AAA	CAA	GCT	1	C	G
TCGA-13-0883	CASKIN1	ENST00000343516	p.N188K	c.564C>G	Unverified	CCC	AAC	GGC	3	C	G
TCGA-13-0883	CASKIN1_EN	ENST00000382453	p.N17K	c.51C>G	Unverified	CCC	AAC	GGC	3	C	G
TCGA-09-2056	CASP8AP2	ENST00000237177	p.S903C	c.2708C>G	Verified	CAG	TCT	GAT	2	C	G
TCGA-61-2113	CATSPER3	ENST00000282611	p.S49R	c.147C>G	Verified	ATG	AGC	CGT	3	C	G
TCGA-24-1423	CBLB	ENST00000264122	p.D759E	c.2277C>G	Verified	CCT	GAC	TTA	3	C	G
TCGA-24-1469	CCDC138	ENST00000295124	p.H369D	c.1105C>G	Unverified	CAT	CAT	CTT	1	C	G
TCGA-24-1104	CCDC15	ENST00000344762	p.G529G	c.1587C>G	Verified	CAA	GGC	CAG	3	C	G
TCGA-24-1846	CCDC157	ENST00000338306	p.L242L	c.726C>G	Unverified	GCC	CTC	AGG	3	C	G
TCGA-24-1846	CCDC157_EI	ENST00000405659	p.L293L	c.879C>G	Unverified	GCC	CTC	AGG	3	C	G
TCGA-24-1847	CCDC165	ENST00000359865	p.T694S	c.2081C>G	Unverified	AAC	ACC	CTC	2	C	G
TCGA-24-1423	CCDC19	ENST00000368099	p.S105R	c.315C>G	Verified	ATC	AGC	CCT	3	C	G
TCGA-24-1844	CCDC41	ENST00000339839	p.P112A	c.334C>G	Unverified	ACT	CCA	CAA	1	C	G
TCGA-20-0990	CCDC65	ENST00000320516	p.P482A	c.1444C>G	Verified	CAT	CCA	ACC	1	C	G
TCGA-23-1117	CCDC69	ENST00000355417	p.R55G	c.163C>G	Verified	GAG	CGG	CAC	1	C	G
TCGA-23-1117	CCDC69	ENST00000355417	p.R55G	c.163C>G	Verified	GAG	CGG	CAC	1	C	G
TCGA-13-0924	CCDC88B	ENST00000356786	p.D1279E	c.3837C>G	Verified	CTG	GAC	CAG	3	C	G
TCGA-23-1124	CCDC88C	ENST00000389857	p.L803V	c.2407C>G	Verified	GAC	CTG	GAG	1	C	G
TCGA-10-0930	CCR8	ENST00000326306	p.N232K	c.696C>G	Verified	cac	aac	aag	3	C	G
TCGA-10-0930	CCR8	ENST00000326306	p.N232K	c.696C>G	Verified	cac	aac	aag	3	C	G
TCGA-13-0923	CCT3	ENST00000295688	p.L245V	c.733C>G	Verified	TCT	CTG	GAA	1	C	G
TCGA-61-2113	CCT8L2	ENST00000359963	p.S262C	c.785C>G	Unverified	CTT	TCT	AGT	2	C	G
TCGA-09-2050	CD22	ENST00000085219	p.N771K	c.2313C>G	Verified	ATG	AAC	ATA	3	C	G
TCGA-24-1469	CD300LB	ENST00000392621	p.C5W	c.15C>G	Verified	AGG	TGC	AAG	3	C	G
TCGA-29-1691	CDC16	ENST00000360383	p.L287V	c.859C>G	Unverified	TAT	CTT	TCT	1	C	G
TCGA-13-0884	CDC25B	NM_021874.2	p.A420G	c.1259C>G	Verified	aag	gcc	ttc	2	C	G
TCGA-61-1740	CDC5L	ENST00000371477	p.L682V	c.2044C>G	Unverified	aat	ctg	gct	1	C	G
TCGA-23-1118	CDCP1	ENST00000296129	p.P716A	c.2146C>G	Verified	ATG	CCG	AGG	1	C	G
TCGA-61-1899	CDH10	ENST00000264463	p.Q538E	c.1612C>G	Unverified	GTA	CAG	GAT	1	C	G
TCGA-61-2009	CDH11	ENST00000268603	p.P473R	c.1418C>G	Verified	GTC	CCA	GTG	2	C	G

TCGA-23-1124	CDH16	ENST00000299752	p.P395R	c.1184C>G	Verified	GAC	CCC	ACT	2	C	G
TCGA-23-1029	CDH2	ENST00000269141	p.F894L	c.2682C>G	Unverified	CGG	TTC	AAG	3	C	G
TCGA-04-1367	CDHR3	ENST00000317716	p.Y440*	c.1320C>G	Verified	CCT	TAC	TAT	3	C	G
TCGA-29-1696	CDK12	NM_016507	p.A1174G	c.3521C>G	Unverified	atg	gcc	cca	2	C	G
TCGA-29-1696	CDK12	NM_016507	p.A1174G	c.3521C>G	Unverified	atg	gcc	cca	2	C	G
TCGA-13-1510	CDK17	ENST00000261211	p.R312G	c.934C>G	Verified	CAT	CGA	GAC	1	C	G
TCGA-04-1362	CDKL4	SU_CDKL4	p.I103M	c.309C>G	Verified	gtg	atc	aaa	3	C	G
TCGA-61-2102	CDKN2C	ENST00000262662	p.A41G	c.122C>G	Verified	act	gcg	ctg	2	C	G
TCGA-13-0924	CDRT1_ENST	ENST00000395906	p.H494Q	c.1482C>G	Verified	GGT	CAC	CAG	3	C	G
TCGA-13-1496	CDS1	ENST00000295887	p.L235V	c.703C>G	Verified	AAT	CTG	TTT	1	C	G
TCGA-13-0760	CELA2B	ENST00000375910	p.H56Q	c.168C>G	Verified	TAC	CAC	ACC	3	C	G
TCGA-24-1604	CENPF	ENST00000366955	p.Q1989E	c.5965C>G	Verified	ACA	CAA	GAG	1	C	G
TCGA-13-0904	CEP192	ENST00000325971	p.T636R	c.1907C>G	Verified	AGC	ACA	GTT	2	C	G
TCGA-13-1499	CEP250	ENST00000397527	p.T2299T	c.6897C>G	Verified	AGT	ACC	TTG	3	C	G
TCGA-13-0913	CEP290	ENST00000552810	p.A1920G	c.5759C>G	Verified	CAA	GCC	AAA	2	C	G
TCGA-13-0887	CEP350	ENST00000367607	p.P2207R	c.6620C>G	Verified	TCT	CCA	GTT	2	C	G
TCGA-23-1116	CHCHD3	ENST00000262570	p.Q82E	c.244C>G	Unverified	GAT	CAG	AAA	1	C	G
TCGA-61-1900	CHD3	ENST00000330494	p.P1825P	c.5475C>G	Unverified	CAC	CCC	GCC	3	C	G
TCGA-13-0906	CHD4_ENST	ENST00000309577	p.R645G	c.1933C>G	Unverified	TGG	CGG	GAC	1	C	G
TCGA-24-1843	CHD9	NM_025134.3	p.I742M	c.2226C>G	Unverified	agg	atc	cag	3	C	G
TCGA-09-0369	CHEK2	ENST00000328354	p.R519G	c.1555C>G	Unverified	agt	cga	aag	1	C	G
TCGA-09-0369	CHEK2	ENST00000328354	p.R519G	c.1555C>G	Unverified	agt	cga	aag	1	C	G
TCGA-13-0760	CHEK2	ENST00000328354	p.R519G	c.1555C>G	Unverified	agt	cga	aag	1	C	G
TCGA-13-0762	CHEK2	ENST00000328354	p.R519G	c.1555C>G	Unverified	agt	cga	aag	1	C	G
TCGA-13-0762	CHEK2	ENST00000328354	p.R519G	c.1555C>G	Unverified	agt	cga	aag	1	C	G
TCGA-04-1652	CHIA	ENST00000430615	p.G227G	c.681C>G	Unverified	GTT	GGC	TAT	3	C	G
TCGA-04-1652	CHIA	ENST00000430615	p.G227G	c.681C>G	Unverified	GTT	GGC	TAT	3	C	G
TCGA-04-1652	CHIA_ENSTC	ENST00000369740	p.G335G	c.1005C>G	Unverified	GTT	GGC	TAT	3	C	G
TCGA-04-1652	CHIA_ENSTC	ENST00000369740	p.G335G	c.1005C>G	Unverified	GTT	GGC	TAT	3	C	G
TCGA-36-1577	CHML	ENST00000366553	p.S514C	c.1541C>G	Verified	TCA	TCT	TCT	2	C	G
TCGA-24-1469	CHRM2	ENST00000320658	p.T15R	c.44C>G	Verified	CTT	ACA	AGT	2	C	G
TCGA-23-1114	CHST14	ENST00000306243	p.R231G	c.691C>G	Unverified	ATC	CGA	GAG	1	C	G
TCGA-09-2056	CHST2	ENST00000309575	p.F51L	c.153C>G	Verified	GTG	TTC	CGT	3	C	G
TCGA-13-0900	CIDEC	ENST00000336832	p.S172C	c.515C>G	Unverified	CTT	TCC	TAT	2	C	G

TCGA-24-1431	CILP2	ENST00000291495	p.D202E	c.606C>G	Verified	CTT	GAC	ACC	3	C	G
TCGA-13-0900	CIT	SU_CRIK	p.D772E	c.2316C>G	Verified	ttg	gac	aat	3	C	G
TCGA-24-1845	CIT	SU_CRIK	p.I1765M	c.5295C>G	Unverified	tgt	atc	cac	3	C	G
TCGA-24-1845	CIT_ENST00	ENST00000261833	p.I1737M	c.5211C>G	Unverified	TGT	ATC	CAC	3	C	G
TCGA-24-1845	CIT_ENST00	ENST00000392521	p.I1779M	c.5337C>G	Unverified	TGT	ATC	CAC	3	C	G
TCGA-61-1740	CLASRP	ENST00000221455	p.P574R	c.1721C>G	Unverified	ACG	CCT	CAG	2	C	G
TCGA-24-1849	CLCN1	ENST00000343257	p.P408A	c.1222C>G	Unverified	CCA	CCA	GGA	1	C	G
TCGA-23-1031	CLDN6	ENST00000396925	p.P135A	c.403C>G	Unverified	ATC	CCC	GTG	1	C	G
TCGA-25-1326	CLDND1	ENST00000341181	p.L198L	c.594C>G	Verified	CTA	CTC	CAC	3	C	G
TCGA-25-2398	CLEC14A	ENST00000342213	p.D265E	c.795C>G	Unverified	GAC	GAC	TTG	3	C	G
TCGA-13-0762	CLEC4C	ENST00000360345	p.S49R	c.147C>G	Verified	TAT	AGC	AAA	3	C	G
TCGA-13-0762	CLEC4C	ENST00000360345	p.S49R	c.147C>G	Verified	TAT	AGC	AAA	3	C	G
TCGA-09-0369	CLGN	ENST00000325617	p.P182R	c.545C>G	Verified	GGA	CCA	GAT	2	C	G
TCGA-09-0369	CLGN	ENST00000325617	p.P182R	c.545C>G	Verified	GGA	CCA	GAT	2	C	G
TCGA-13-1499	CLSPN	ENST00000318121	p.L456V	c.1366C>G	Verified	gcc	ctg	gag	1	C	G
TCGA-61-2008	CLUL1	ENST00000338387	p.L434L	c.1302C>G	Verified	ACA	CTC	AAG	3	C	G
TCGA-13-0884	CLVS1	ENST00000325897	p.Q55E	c.163C>G	Verified	CAG	CAA	GTC	1	C	G
TCGA-13-0923	CMKLR1	ENST00000312143	p.A110A	c.330C>G	Verified	ACA	GCC	ATG	3	C	G
TCGA-24-2289	CMTM4	ENST00000330687	p.L132V	c.394C>G	Unverified	ttc	ctt	ttc	1	C	G
TCGA-13-0900	CNGA1	ENST00000358519	p.S116*	c.347C>G	Verified	AAG	TCA	GAT	2	C	G
TCGA-29-1777	CNNM3	ENST00000305510	p.G694G	c.2082C>G	Unverified	CCC	GGC	GTC	3	C	G
TCGA-04-1356	CNOT1	ENST00000317147	p.T1510S	c.4529C>G	Verified	AAG	ACT	GCA	2	C	G
TCGA-61-1907	CNPY3	ENST00000372836	p.D81E	c.243C>G	Unverified	CTG	GAC	CAG	3	C	G
TCGA-24-1464	CNTN5	ENST00000528682	p.L539V	c.1615C>G	Verified	atc	cta	aat	1	C	G
TCGA-61-2094	CNTNAP1	ENST00000264638	p.L399V	c.1195C>G	Unverified	CTC	CTT	TTC	1	C	G
TCGA-13-1510	CNTNAP4	ENST00000339740	p.S690C	c.2069C>G	Unverified	GGT	TCT	TCG	2	C	G
TCGA-13-1510	CNTNAP4_E	ENST00000307431	p.S714C	c.2141C>G	Unverified	GGT	TCT	TCG	2	C	G
TCGA-61-1998	COBL	ENST00000265136	p.R353R	c.1059C>G	Unverified	AAC	CGC	ACT	3	C	G
TCGA-24-2267	COBLL1	ENST00000342193	p.S1077C	c.3230C>G	Verified	GTG	TCT	GAT	2	C	G
TCGA-13-1481	COG1	ENST00000299886	p.D384E	c.1152C>G	Verified	CGG	GAC	GCC	3	C	G
TCGA-24-1563	COL12A1	ENST00000322507	p.S554*	c.1661C>G	Verified	TCA	TCA	GAT	2	C	G
TCGA-10-0930	COL12A1	ENST00000322507	p.N1849K	c.5547C>G	Verified	AGG	AAC	CTG	3	C	G
TCGA-10-0930	COL12A1	ENST00000322507	p.N1849K	c.5547C>G	Verified	AGG	AAC	CTG	3	C	G
TCGA-20-1686	COL4A4	ENST00000396625	p.P1025R	c.3074C>G	Unverified	GGG	CCT	CCT	2	C	G

TCGA-24-1845	COL5A1	ENST00000371817	p.L1576V	c.4726C>G	Unverified	CCC	CTG	CCA	1	C	G
TCGA-13-0793	COPB1	ENST00000249923	p.T468T	c.1404C>G	Verified	AGT	ACC	AAG	3	C	G
TCGA-23-1118	CORIN	ENST00000273857	p.P467R	c.1400C>G	Verified	TTG	CCC	TAC	2	C	G
TCGA-61-2012	CORO2B	ENST00000566799	p.C335W	c.1005C>G	Verified	GCC	TGC	GAG	3	C	G
TCGA-13-0886	CPA2	ENST00000222481	p.P398P	c.1194C>G	Verified	CTG	CCC	ACA	3	C	G
TCGA-04-1347	CPNE6	ENST00000397016	p.S477C	c.1430C>G	Verified	TTC	TCT	GAC	2	C	G
TCGA-04-1655	CPVL	ENST00000396276	p.T321R	c.962C>G	Unverified	TGC	ACG	GAA	2	C	G
TCGA-23-1031	CPVL	ENST00000396276	p.T396R	c.1187C>G	Verified	CTG	ACA	GAG	2	C	G
TCGA-29-1705	CRADD	ENST00000332896	p.L30L	c.90C>G	Unverified	tac	ctc	tac	3	C	G
TCGA-20-1686	CRB1	ENST00000367400	p.R1286G	c.3856C>G	Unverified	TGC	CGG	CCA	1	C	G
TCGA-13-0916	CRB1	ENST00000367400	p.A595G	c.1784C>G	Verified	AAA	GCT	CCT	2	C	G
TCGA-61-1900	CREB3L2	ENST00000330387	p.L57L	c.171C>G	Unverified	ttc	ctc	tca	3	C	G
TCGA-29-1705	CREBBP	ENST00000262367	p.N390K	c.1170C>G	Unverified	AAA	AAC	GTT	3	C	G
TCGA-61-1900	CSF2RB	ENST00000403662	p.R799G	c.2395C>G	Unverified	GAA	CGC	CCG	1	C	G
TCGA-29-1696	CSMD1_ENS	ENST00000537824	p.L544L	c.1632C>G	Unverified	TTC	CTC	CAT	3	C	G
TCGA-29-1696	CSMD1_ENS	ENST00000537824	p.L544L	c.1632C>G	Unverified	TTC	CTC	CAT	3	C	G
TCGA-30-1718	CSMD2	ENST00000241312	p.L1520V	c.4558C>G	Unverified	CAG	CTC	CCA	1	C	G
TCGA-30-1718	CSMD2_ENS	ENST00000373381	p.L1560V	c.4678C>G	Unverified	CAG	CTC	CCA	1	C	G
TCGA-29-1701	CSMD3	ENST00000297405	p.T643R	c.1928C>G	Unverified	caa	acg	gac	2	C	G
TCGA-29-2427	CSMD3	ENST00000297405	p.I2122M	c.6366C>G	Verified	gtg	atc	ctc	3	C	G
TCGA-29-1701	CSMD3_ENS	ENST00000343508	p.T603R	c.1808C>G	Unverified	CAA	ACG	GAC	2	C	G
TCGA-25-2393	CSN3	ENST00000304954	p.P52A	c.154C>G	Verified	GTG	CCA	AAT	1	C	G
TCGA-61-1900	CSRNP1	ENST00000273153	p.L583V	c.1747C>G	Unverified	TCT	CTA	ATG	1	C	G
TCGA-29-1695	CSTF2T	ENST00000331173	p.P225R	c.674C>G	Unverified	GGA	CCT	AAT	2	C	G
TCGA-61-1900	CTBP2	ENST00000309035	p.A97G	c.290C>G	Unverified	CAG	GCA	GTG	2	C	G
TCGA-24-1563	CUX2	ENST00000261726	p.Q386E	c.1156C>G	Verified	CCC	CAG	GGC	1	C	G
TCGA-04-1638	CXCL16	ENST00000293778	p.H192D	c.574C>G	Unverified	ATT	CAC	ACT	1	C	G
TCGA-04-1638	CXCL16_ENS	ENST00000293778	p.H192D	c.574C>G	Unverified	ATT	CAC	ACT	1	C	G
TCGA-29-1691	CXCR5	ENST00000292174	p.T203T	c.609C>G	Unverified	tgc	acc	ttc	3	C	G
TCGA-23-1021	CXXC1	ENST00000285106	p.F219L	c.657C>G	Verified	TAC	TTC	CCT	3	C	G
TCGA-61-1910	CYC1	ENST00000318911	p.P247A	c.739C>G	Unverified	CCT	CCC	ATC	1	C	G
TCGA-13-1488	CYFIP2_ENST	ENST00000442283	p.T896S	c.2687C>G	Verified	TTC	ACC	CAA	2	C	G
TCGA-29-1777	CYP11B1	ENST00000292427	p.P14R	c.41C>G	Unverified	GTG	CCC	TGG	2	C	G
TCGA-24-1846	CYP11B2	ENST00000323110	p.Q54E	c.160C>G	Unverified	CTG	CAG	ATC	1	C	G

TCGA-29-1764	CYP4A11	ENST00000310638	p.D69E	c.207C>G	Unverified	CAG	GAC	CAG	3	C	G
TCGA-20-1683	CYP4F2	ENST00000221700	p.I271M	c.813C>G	Unverified	GTC	ATC	CAG	3	C	G
TCGA-23-2078	CYTH4	ENST00000248901	p.L253V	c.757C>G	Verified	GAC	CTC	ACT	1	C	G
TCGA-25-2401	DAAM1	ENST00000351081	p.A832G	c.2495C>G	Verified	aat	gca	tat	2	C	G
TCGA-24-1849	DAB2IP	ENST00000309989	p.P409A	c.1225C>G	Unverified	TCG	CCC	TCA	1	C	G
TCGA-24-1849	DAB2IP_ENS	ENST00000259371	p.P505A	c.1513C>G	Unverified	TCG	CCC	TCA	1	C	G
TCGA-24-2289	DARS2	ENST00000361951	p.L169V	c.505C>G	Unverified	GCT	CTT	CGG	1	C	G
TCGA-29-1776	DBC1	ENST00000373969	p.S689C	c.2066C>G	Unverified	TCC	TCT	TCG	2	C	G
TCGA-13-0791	DCC	ENST00000442544	p.Q460E	c.1378C>G	Verified	ATT	CAA	ACT	1	C	G
TCGA-13-0761	DCTN1	ENST00000361874	p.L705V	c.2113C>G	Verified	GAA	CTG	CTG	1	C	G
TCGA-13-0761	DCTN1	ENST00000361874	p.L705V	c.2113C>G	Verified	GAA	CTG	CTG	1	C	G
TCGA-09-2049	DCTN1	ENST00000361874	p.S892*	c.2675C>G	Verified	CAG	TCA	TGC	2	C	G
TCGA-13-1497	DCTN1	ENST00000361874	p.S651R	c.1953C>G	Verified	CTC	AGC	TTT	3	C	G
TCGA-30-1718	DDI2	ENST00000480945	p.A226G	c.677C>G	Unverified	GAG	GCT	CCG	2	C	G
TCGA-61-1906	DDX11_ENS	ENST00000407793	p.P922A	c.2764C>G	Unverified	TGC	CCG	CTG	1	C	G
TCGA-61-1906	DDX11_ENS	ENST00000407793	p.P922A	c.2764C>G	Unverified	TGC	CCG	CTG	1	C	G
TCGA-23-1110	DDX58	ENST00000379883	p.L391V	c.1171C>G	Verified	tat	cta	gat	1	C	G
TCGA-13-0886	DFNB31	ENST00000362057	p.R423G	c.1267C>G	Verified	ACA	CGA	GTG	1	C	G
TCGA-09-2049	DFNB31	ENST00000362057	p.S160W	c.479C>G	Verified	GGC	TCG	GAG	2	C	G
TCGA-24-1843	DGKH	ENST00000337343	p.I935M	c.2805C>G	Unverified	att	atc	aaa	3	C	G
TCGA-24-2024	DIDO1	ENST00000395343	p.P2059A	c.6175C>G	Unverified	GGC	CCC	GAG	1	C	G
TCGA-23-1110	DIP2C	ENST00000280886	p.P480P	c.1440C>G	Verified	CCG	CCC	CGA	3	C	G
TCGA-23-1110	DKK2	ENST00000285311	p.R112G	c.334C>G	Verified	cac	cga	gat	1	C	G
TCGA-13-0920	DLAT	ENST00000280346	p.P187R	c.560C>G	Unverified	gca	cct	acc	2	C	G
TCGA-25-1326	DLG1	ENST00000346964	p.P464R	c.1391C>G	Verified	GAA	CCT	AGA	2	C	G
TCGA-13-1481	DLGAP3	ENST00000373347	p.I883M	c.2649C>G	Unverified	TCC	ATC	GAG	3	C	G
TCGA-24-2289	DLGAP5	ENST00000247191	p.L466V	c.1396C>G	Unverified	GAT	CTT	ATT	1	C	G
TCGA-25-2401	DLX5	ENST00000222598	p.S40C	c.119C>G	Verified	GAG	TCT	TCA	2	C	G
TCGA-13-1509	DMRT2_ENS	ENST00000302441	p.P295A	c.883C>G	Verified	CCA	CCA	AGC	1	C	G
TCGA-04-1347	DMXL1	ENST00000311085	p.S1218C	c.3653C>G	Verified	GTT	TCT	TTA	2	C	G
TCGA-24-1431	DNA2L	ENST00000358410	p.Q203E	c.607C>G	Unverified	AGT	CAA	GAT	1	C	G
TCGA-25-2398	DNAH17	ENST00000300671	p.A3207G	c.9620C>G	Unverified	GAG	GCC	TGC	2	C	G
TCGA-04-1343	DNAH2	ENST00000389173	p.L2373V	c.7117C>G	Verified	AAG	CTC	CCT	1	C	G
TCGA-13-0885	DNAH2	ENST00000389173	p.Q294E	c.880C>G	Verified	AAG	CAG	CTG	1	C	G

TCGA-13-1507	DNAH2	ENST00000389173	p.L640L	c.1920C>G	Verified	ATT	CTC	TTT	3	C	G
TCGA-24-1846	DNAH5	ENST00000265104	p.S1618*	c.4853C>G	Unverified	AAC	TCA	ACA	2	C	G
TCGA-13-0920	DNAJB7	ENST00000307221	p.H110Q	c.330C>G	Verified	ttt	cac	ttc	3	C	G
TCGA-25-1326	DNAJC7	NM_003315.1	p.L208V	c.622C>G	Verified	ggt	ctt	tgc	1	C	G
TCGA-25-2393	DNHD1	ENST00000254579	p.L4528V	c.13582C>G	Verified	GCT	CTC	TGG	1	C	G
TCGA-29-1781	DNM2	ENST00000359692	p.S503R	c.1509C>G	Unverified	AGG	AGC	ACG	3	C	G
TCGA-29-1781	DNM2_ENST	ENST00000389253	p.S503R	c.1509C>G	Unverified	AGG	AGC	ACG	3	C	G
TCGA-04-1530	DNMT3B	ENST00000328111	p.A241A	c.723C>G	Unverified	ccc	gcc	atg	3	C	G
TCGA-29-1784	DOCK4_ENS	ENST00000437633	p.I862M	c.2586C>G	Unverified	CTT	ATC	AAG	3	C	G
TCGA-29-1705	DPP10	ENST00000310323	p.P545P	c.1635C>G	Unverified	CTT	CCC	AAA	3	C	G
TCGA-29-1705	DPP10_ENST	ENST00000410059	p.P552P	c.1656C>G	Unverified	CTT	CCC	AAA	3	C	G
TCGA-61-2094	DPY19L2	ENST00000324472	p.L42V	c.124C>G	Unverified	GCC	CTA	GGC	1	C	G
TCGA-24-1604	DPY19L3	ENST00000342179	p.L532V	c.1594C>G	Verified	ATA	CTG	CTG	1	C	G
TCGA-25-2392	DPY19L3	ENST00000342179	p.L561V	c.1681C>G	Verified	GAG	CTG	ATG	1	C	G
TCGA-61-2095	DPYSL3	ENST00000398514	p.Q340E	c.1018C>G	Unverified	GCC	CAG	AAA	1	C	G
TCGA-20-0990	DPYSL5	ENST00000288699	p.H433Q	c.1299C>G	Verified	TGC	CAC	GGC	3	C	G
TCGA-29-1766	DTNA_ENST	ENST00000269190	p.T179R	c.536C>G	Unverified	CCC	ACG	GCA	2	C	G
TCGA-29-1766	DTNA_ENST	ENST00000315456	p.T179R	c.536C>G	Unverified	CCC	ACG	GCA	2	C	G
TCGA-29-1766	DTNA_ENST	ENST00000444659	p.T179R	c.536C>G	Unverified	CCC	ACG	GCA	2	C	G
TCGA-13-0919	DUOX1	ENST00000389037	p.R76G	c.226C>G	Unverified	CCC	CGA	GAC	1	C	G
TCGA-04-1367	DUOX1	ENST00000389037	p.L1117L	c.3351C>G	Verified	TTC	CTC	AAC	3	C	G
TCGA-13-0923	DYNC1H1	ENST00000360184	p.L3649V	c.10945C>G	Verified	GTG	CTG	AAC	1	C	G
TCGA-61-1906	ECM2	ENST00000344604	p.A530A	c.1590C>G	Unverified	tta	gcc	tgg	3	C	G
TCGA-61-1906	ECM2	ENST00000344604	p.A530A	c.1590C>G	Unverified	tta	gcc	tgg	3	C	G
TCGA-61-1906	ECM2_ENST	ENST00000444490	p.A508A	c.1524C>G	Unverified	TTA	GCC	TGG	3	C	G
TCGA-61-1906	ECM2_ENST	ENST00000444490	p.A508A	c.1524C>G	Unverified	TTA	GCC	TGG	3	C	G
TCGA-29-1775	ECT2	ENST00000232458	p.P565A	c.1693C>G	Unverified	cga	cca	gta	1	C	G
TCGA-20-1685	EEA1	ENST00000322349	p.A502G	c.1505C>G	Unverified	ACG	GCA	AAA	2	C	G
TCGA-23-1032	EFCAB6	ENST00000262726	p.S853C	c.2558C>G	Verified	TTG	TCT	AAG	2	C	G
TCGA-13-0919	EGFR	ENST00000275493	p.P772R	c.2315C>G	Unverified	AAC	CCC	CAC	2	C	G
TCGA-24-1844	EGR1_ENST(	ENST00000239938	p.H25D	c.73C>G	Unverified	CCT	CAC	TCG	1	C	G
TCGA-25-1313	EGR2	ENST00000242480	p.L219L	c.657C>G	Verified	GGT	CTC	TTC	3	C	G
TCGA-24-1463	EIF2AK2	ENST00000233057	p.A2G	c.5C>G	Verified	atg	gct	ggt	2	C	G
TCGA-13-1501	EIF2B3	ENST00000360403	p.V340V	c.1020C>G	Verified	CCA	GTC	CAT	3	C	G

TCGA-13-1501	EIF2B3	ENST00000360403	p.V340V	c.1020C>G	Verified	CCA	GTC	CAT	3	C	G
TCGA-24-1845	EIF3L	ENST00000412331	p.L457V	c.1369C>G	Unverified	CAG	CTT	TCA	1	C	G
TCGA-04-1361	EIF3L	ENST00000412331	p.S217C	c.650C>G	Verified	CGT	TCC	AAT	2	C	G
TCGA-04-1361	EIF3L	ENST00000412331	p.S217C	c.650C>G	Verified	CGT	TCC	AAT	2	C	G
TCGA-24-2280	EIF4G3	ENST00000264211	p.Q1166E	c.3496C>G	Unverified	TCT	CAA	GAA	1	C	G
TCGA-24-0979	ELF1	NM_172373.2	p.P225A	c.673C>G	Verified	tgt	cct	aaa	1	C	G
TCGA-13-0795	ELK3	ENST00000228741	p.P95A	c.283C>G	Verified	gat	cct	cac	1	C	G
TCGA-61-1740	ELMOD3	ENST00000315658	p.S7C	c.20C>G	Unverified	TGC	TCT	TTC	2	C	G
TCGA-13-0795	ELMOD3	ENST00000315658	p.S166R	c.498C>G	Verified	GAT	AGC	CAA	3	C	G
TCGA-61-1740	ELMOD3_EN	ENST00000409890	p.S7C	c.20C>G	Unverified	TGC	TCT	TTC	2	C	G
TCGA-29-1698	EMID2	ENST00000397927	p.G265G	c.795C>G	Unverified	CCA	GGC	CCC	3	C	G
TCGA-29-1691	EMP2	ENST00000359543	p.R166R	c.498C>G	Unverified	AAG	CGC	AAA	3	C	G
TCGA-13-0795	ENAM	ENST00000396073	p.Q942E	c.2824C>G	Verified	AGC	CAA	AAC	1	C	G
TCGA-04-1331	ENSG00000:	ENST00000315797	p.S54C	c.161C>G	Verified	TCC	TCC	TCT	2	C	G
TCGA-04-1331	ENSG00000:	ENST00000398226	p.G189G	c.567C>G	Verified	TGA	GGC	TAA	3	C	G
TCGA-29-1768	EPB41_ENST	ENST00000343067	p.L82V	c.244C>G	Unverified	CGA	CTA	TTC	1	C	G
TCGA-23-1031	EPB42	ENST00000300215	p.A502A	c.1506C>G	Verified	ACT	GCC	AGT	3	C	G
TCGA-24-2280	EPS8L3	ENST00000369805	p.P163R	c.488C>G	Unverified	CAG	CCA	GGC	2	C	G
TCGA-13-1408	ERCC2	ENST00000391945	p.A503G	c.1508C>G	Unverified	GTG	GCC	ATC	2	C	G
TCGA-61-1740	ERMAP	ENST00000372514	p.A432G	c.1295C>G	Unverified	CAT	GCT	AAT	2	C	G
TCGA-23-1124	ERVFRD-1	ENST00000472091	p.V256V	c.768C>G	Verified	CAA	GTC	TTA	3	C	G
TCGA-09-2051	ESPL1	ENST00000257934	p.S1820S	c.5460C>G	Unverified	GCC	TCC	CGC	3	C	G
TCGA-04-1336	ETAA1	ENST00000272342	p.Q622E	c.1864C>G	Verified	ACT	CAG	CAA	1	C	G
TCGA-24-2288	ETS1	ENST00000319397	p.P190R	c.569C>G	Unverified	cat	ccc	atc	2	C	G
TCGA-61-1740	EVPL	ENST00000301607	p.T327T	c.981C>G	Unverified	GAG	ACC	CAG	3	C	G
TCGA-29-1766	EXOC3	ENST00000315013	p.N313K	c.939C>G	Unverified	CTG	AAC	ATG	3	C	G
TCGA-13-0923	EYA3	ENST00000373871	p.L480V	c.1438C>G	Verified	GTT	CTG	ATC	1	C	G
TCGA-20-1683	EYA4	ENST00000367895	p.A36G	c.107C>G	Unverified	CTA	GCA	AGT	2	C	G
TCGA-20-1683	EYA4_ENST(	ENST00000355167	p.A36G	c.107C>G	Unverified	CTA	GCA	AGT	2	C	G
TCGA-24-0975	FAAH2	ENST00000374900	p.S408S	c.1224C>G	Verified	CCT	TCC	ATT	3	C	G
TCGA-09-2056	FADS2	ENST00000278840	p.P77R	c.230C>G	Verified	CAC	CCT	GAC	2	C	G
TCGA-24-1563	FAM111A	ENST00000528737	p.T366S	c.1097C>G	Verified	GCA	ACT	ACG	2	C	G
TCGA-25-2401	FAM116A	ENST00000311128	p.P202A	c.604C>G	Unverified	GAA	CCT	TAT	1	C	G
TCGA-61-1907	FAM120C	ENST00000375180	p.S1059C	c.3176C>G	Unverified	TTA	TCC	AGA	2	C	G

TCGA-23-2078	FAM123C	ENST00000321420	p.P639R	c.1916C>G	Unverified	TGG	CCC	TGC	2	C	G
TCGA-23-1021	FAM129A	ENST00000367511	p.P728R	c.2183C>G	Verified	GCT	CCA	GTG	2	C	G
TCGA-24-1103	FAM135B	ENST00000395297	p.T1259T	c.3777C>G	Verified	AGC	ACC	CTG	3	C	G
TCGA-13-1501	FAM161A	ENST00000307507	p.L184L	c.552C>G	Verified	TTT	CTC	CCC	3	C	G
TCGA-13-1501	FAM161A	ENST00000307507	p.L184L	c.552C>G	Verified	TTT	CTC	CCC	3	C	G
TCGA-04-1331	FAM166A	ENST00000344774	p.P140A	c.418C>G	Unverified	TGC	CCA	CCA	1	C	G
TCGA-20-1683	FAM171B	ENST00000304698	p.P250R	c.749C>G	Unverified	ACT	CCT	CTT	2	C	G
TCGA-04-1362	FAM183A	ENST00000335282	p.P52P	c.156C>G	Verified	AAG	CCC	ATG	3	C	G
TCGA-24-1435	FAM35A	ENST00000358313	p.A644G	c.1931C>G	Verified	GGA	GCA	GCC	2	C	G
TCGA-13-0807	FAM47B	ENST00000329357	p.S453C	c.1358C>G	Verified	GAC	TCT	CTT	2	C	G
TCGA-04-1362	FAM47C	ENST00000358047	p.P644A	c.1930C>G	Unverified	cgc	ccg	gag	1	C	G
TCGA-13-0761	FAM53B	ENST00000337318	p.P108A	c.322C>G	Verified	GCA	CCC	CCT	1	C	G
TCGA-13-0761	FAM53B	ENST00000337318	p.P108A	c.322C>G	Verified	GCA	CCC	CCT	1	C	G
TCGA-61-1915	FAM57B	ENST00000380495	p.T169R	c.506C>G	Unverified	AGC	ACG	CCC	2	C	G
TCGA-13-0760	FAM81A	ENST00000288228	p.L29V	c.85C>G	Verified	AGC	CTC	GTG	1	C	G
TCGA-13-1505	FAM90A1	ENST00000307435	p.P7P	c.21C>G	Verified	GAC	CCC	AAA	3	C	G
TCGA-04-1361	FAM91A1	ENST00000334705	p.P547A	c.1639C>G	Verified	CCA	CCA	TCC	1	C	G
TCGA-04-1361	FAM91A1	ENST00000334705	p.P547A	c.1639C>G	Verified	CCA	CCA	TCC	1	C	G
TCGA-61-1733	FAM98C	ENST00000252530	p.P325P	c.975C>G	Unverified	CCT	CCC	ATG	3	C	G
TCGA-61-1733	FAM98C	ENST00000252530	p.P325P	c.975C>G	Unverified	CCT	CCC	ATG	3	C	G
TCGA-24-1845	FARSA	ENST00000314606	p.G470G	c.1410C>G	Unverified	TAT	GGC	ATC	3	C	G
TCGA-61-1913	FAT	ENST00000441802	p.L334L	c.1002C>G	Unverified	AAT	CTC	ACA	3	C	G
TCGA-61-1913	FAT1	ENST00000441802	p.L334L	c.1002C>G	Unverified	AAT	CTC	ACA	3	C	G
TCGA-61-2109	FAT1	ENST00000441802	p.S1553R	c.4659C>G	Unverified	GTC	AGC	GAC	3	C	G
TCGA-20-0991	FAT2	ENST00000261800	p.P2732A	c.8194C>G	Verified	ACA	CCT	GAG	1	C	G
TCGA-09-2049	FAT3_ENST(	ENST00000298047_v61	p.A2778G	c.8333C>G	Verified	CCA	GCT	TTC	2	C	G
TCGA-04-1655	FAT4	ENST00000330166	p.P2870A	c.8608C>G	Unverified	GCT	CCA	AGA	1	C	G
TCGA-29-1703	FAT4	ENST00000330166	p.L4738V	c.14212C>G	Unverified	AGG	CTC	AAC	1	C	G
TCGA-04-1655	FAT4_ENST(	ENST00000394329	p.P2870A	c.8608C>G	Unverified	GCT	CCA	AGA	1	C	G
TCGA-29-1703	FAT4_ENST(	ENST00000394329	p.L4795V	c.14383C>G	Unverified	AGG	CTC	AAC	1	C	G
TCGA-13-1481	FBLN5	ENST00000342058	p.L325V	c.973C>G	Verified	TAT	CTG	AGG	1	C	G
TCGA-13-0900	FBLN5	ENST00000342058	p.S100*	c.299C>G	Verified	CTC	TCA	GCT	2	C	G
TCGA-24-1845	FBXL19	NM_019085.1	p.L171V	c.511C>G	Unverified	tcg	ctg	agt	1	C	G
TCGA-24-1845	FBXL19_ENS	ENST00000380310	p.L341V	c.1021C>G	Unverified	TCG	CTG	AGT	1	C	G

TCGA-24-1422	FBXO15	ENST00000269500	p.S204C	c.611C>G	Verified	ata	tct	acc	2	C	G
TCGA-23-1114	FBXO38	ENST00000340253	p.I681M	c.2043C>G	Unverified	cag	atc	aaa	3	C	G
TCGA-23-1114	FBXO46	ENST00000317683	p.R192G	c.574C>G	Unverified	cca	cga	ccg	1	C	G
TCGA-04-1530	FBXW2	NM_012164.2	p.L261V	c.781C>G	Verified	tgc	ctg	aac	1	C	G
TCGA-23-1122	FCGRT	ENST00000221466	p.S60R	c.180C>G	Verified	CTG	AGC	TAC	3	C	G
TCGA-24-2024	FETUB	ENST00000265029	p.F82L	c.246C>G	Verified	CTG	TTC	TAT	3	C	G
TCGA-04-1331	FEZ1	ENST00000278919	p.P17P	c.51C>G	Verified	cga	ccc	tcc	3	C	G
TCGA-13-0885	FGD1	ENST00000375135	p.L908V	c.2722C>G	Verified	gaa	cta	cag	1	C	G
TCGA-23-1809	FGFR2	ENST00000358487	p.P253R	c.758C>G	Unverified	TCG	CCT	CAC	2	C	G
TCGA-23-1809	FGFR2_ENST	ENST00000351936	p.P253R	c.758C>G	Unverified	TCG	CCT	CAC	2	C	G
TCGA-23-1809	FGFR2_ENST	ENST00000357555	p.P164R	c.491C>G	Unverified	TCG	CCT	CAC	2	C	G
TCGA-23-1809	FGFR2_ENST	ENST00000457416	p.P253R	c.758C>G	Unverified	TCG	CCT	CAC	2	C	G
TCGA-23-1123	FH	ENST00000366560	p.T442R	c.1325C>G	Verified	aat	aca	gaa	2	C	G
TCGA-23-1123	FH	ENST00000366560	p.T442R	c.1325C>G	Verified	aat	aca	gaa	2	C	G
TCGA-13-0904	FHDC1	ENST00000260008	p.R210G	c.628C>G	Verified	TTG	CGA	GAA	1	C	G
TCGA-13-1505	FHOD1	ENST00000258201	p.P50A	c.148C>G	Unverified	CTG	CCC	TTG	1	C	G
TCGA-24-1845	FIP1L1_ENST	ENST00000337488	p.L152V	c.454C>G	Unverified	CCA	CTC	TTA	1	C	G
TCGA-04-1347	FKBP8	ENST00000222308	p.A222G	c.665C>G	Verified	ctg	gcc	aac	2	C	G
TCGA-30-1891	FKBP8	ENST00000222308	p.V118V	c.354C>G	Unverified	ccg	gtc	aag	3	C	G
TCGA-61-1913	FLG2	ENST00000388718	p.S1491C	c.4472C>G	Unverified	GGG	TCC	AGT	2	C	G
TCGA-13-0886	FLJ44216	ENST00000345702	p.T584T	c.1752C>G	Verified	CAG	ACC	CTG	3	C	G
TCGA-29-1764	FN1	ENST00000336916	p.Q792E	c.2374C>G	Unverified	TAT	CAG	ATA	1	C	G
TCGA-13-0905	FN1	ENST00000336916	p.T2132S	c.6395C>G	Verified	CTC	ACC	AGA	2	C	G
TCGA-29-1764	FN1_ENST0(	ENST00000354785	p.Q792E	c.2374C>G	Unverified	TAT	CAG	ATA	1	C	G
TCGA-61-1900	FNDC3A	ENST00000492622	p.L20V	c.58C>G	Unverified	TCT	CTT	TTG	1	C	G
TCGA-25-2401	FOXB1	ENST00000396057	p.P91R	c.272C>G	Verified	CAC	CCA	AGC	2	C	G
TCGA-25-2401	FOXJ2	ENST00000162391	p.P335A	c.1003C>G	Verified	cct	cca	ctg	1	C	G
TCGA-25-2042	FOXK1	ENST00000328914	p.T600R	c.1799C>G	Unverified	CAC	ACG	GTC	2	C	G
TCGA-13-0904	FPR2	ENST00000340023	p.L162L	c.486C>G	Verified	TTC	CTC	TTT	3	C	G
TCGA-24-2288	FREM1	ENST00000422223	p.P295A	c.883C>G	Unverified	ATT	CCA	AAG	1	C	G
TCGA-13-0885	FREM1	ENST00000422223	p.N1830K	c.5490C>G	Verified	CTG	AAC	TCC	3	C	G
TCGA-61-1900	FRMD4B	ENST00000264546	p.A462G	c.1385C>G	Unverified	GAA	GCT	GCA	2	C	G
TCGA-61-1900	FRMD4B_EN	ENST00000398540	p.A516G	c.1547C>G	Unverified	GAA	GCT	GCA	2	C	G
TCGA-13-1497	FRMPD3	XM_042978.4	p.R717G	c.2149C>G	Verified	tct	cgt	gac	1	C	G

TCGA-29-1762	FRMPD3	XM_042978.4	p.P1428P	c.4284C>G	Unverified	agc	ccc	atc	3	C	G
TCGA-29-1762	FRMPD3_EN	ENST00000276185	p.P1379P	c.4137C>G	Unverified	AGC	CCC	ATC	3	C	G
TCGA-13-0906	FRY	ENST00000380250	p.R2486G	c.7456C>G	Verified	AGA	CGT	TCT	1	C	G
TCGA-30-1718	FRY	ENST00000380250	p.L856L	c.2568C>G	Unverified	TGC	CTC	TTC	3	C	G
TCGA-61-1910	FST	ENST00000256759	p.A29G	c.86C>G	Unverified	CAG	GCT	GGG	2	C	G
TCGA-23-1114	FTSJ3	ENST00000582115	p.G8G	c.24C>G	Unverified	GTT	GGC	AAG	3	C	G
TCGA-23-2078	G3BP1	ENST00000394123	p.P316R	c.947C>G	Verified	GGA	CCC	AGA	2	C	G
TCGA-13-0792	G6PD	ENST00000291567	p.F501L	c.1503C>G	Verified	GGT	TTC	CAG	3	C	G
TCGA-09-2056	GAB2	ENST00000361507	p.S503R	c.1509C>G	Verified	ccc	agc	aga	3	C	G
TCGA-13-0792	GAB2	ENST00000361507	p.S634S	c.1902C>G	Verified	tca	tcc	gtc	3	C	G
TCGA-24-1844	GABBR1	ENST00000377034	p.S546R	c.1638C>G	Unverified	GGC	AGC	TAC	3	C	G
TCGA-24-0979	GADD45GIP	ENST00000316939	p.L163L	c.489C>G	Verified	GAG	CTC	CTG	3	C	G
TCGA-61-2095	GAL3ST4	ENST00000360039	p.D203E	c.609C>G	Unverified	GGG	GAC	CAC	3	C	G
TCGA-25-1326	GALNT12	ENST00000375011	p.L153V	c.457C>G	Verified	CTC	CTT	CGG	1	C	G
TCGA-09-2051	GALNT6	ENST00000356317	p.P401P	c.1203C>G	Unverified	ATC	CCC	TGC	3	C	G
TCGA-61-1737	GARNL3	ENST00000373387	p.L1001V	c.3001C>G	Unverified	CAG	CTC	ACG	1	C	G
TCGA-61-2113	GAS2L1_ENS	ENST00000333679	p.T496T	c.1488C>G	Unverified	GGC	ACC	ACA	3	C	G
TCGA-29-1761	GBX2	ENST00000306318	p.L259V	c.775C>G	Unverified	CAG	CTG	CTG	1	C	G
TCGA-24-1849	GCC1	ENST00000321407	p.S613C	c.1838C>G	Unverified	GCC	TCT	GGG	2	C	G
TCGA-09-2051	GCC2	ENST00000309863	p.Q1607E	c.4819C>G	Unverified	AAT	CAA	GAG	1	C	G
TCGA-13-1488	GCNT4	ENST00000322348	p.S288C	c.863C>G	Verified	ATC	TCC	AAG	2	C	G
TCGA-13-0890	GDF2	ENST00000249598	p.A321G	c.962C>G	Verified	agc	gcc	ggg	2	C	G
TCGA-13-0924	GDF2	ENST00000249598	p.V75V	c.225C>G	Verified	ggg	gtc	cct	3	C	G
TCGA-61-1725	GDPD3	ENST00000406256	p.I234M	c.702C>G	Unverified	ATC	ATC	AAC	3	C	G
TCGA-24-2288	GEN1	ENST00000317402	p.S102C	c.305C>G	Unverified	TGG	TCT	CAG	2	C	G
TCGA-24-1844	GFM1	ENST00000486715	p.L360V	c.1078C>G	Unverified	AAA	CTG	GAG	1	C	G
TCGA-24-1844	GFM1_ENST	ENST00000312756	p.L94V	c.280C>G	Unverified	AAA	CTG	GAG	1	C	G
TCGA-23-1114	GFRA1	ENST00000369236	p.S229C	c.686C>G	Unverified	TGC	TCC	TAT	2	C	G
TCGA-23-1114	GFRA1	ENST00000369236	p.P144P	c.432C>G	Unverified	ATT	CCC	AAA	3	C	G
TCGA-10-0930	GFRA3	ENST00000274721	p.V195V	c.585C>G	Verified	CAC	GTC	TGC	3	C	G
TCGA-10-0930	GFRA3	ENST00000274721	p.V195V	c.585C>G	Verified	CAC	GTC	TGC	3	C	G
TCGA-23-1022	GIMAP5	ENST00000358647	p.P51P	c.153C>G	Verified	CAG	CCC	GTG	3	C	G
TCGA-13-0893	GLI3	ENST00000395925	p.Q670E	c.2008C>G	Verified	ACT	CAG	GGA	1	C	G
TCGA-04-1362	GLS	ENST00000320717	p.S586C	c.1757C>G	Verified	GAT	TCT	AGA	2	C	G

TCGA-13-0913	GNAS	ENST00000371085	p.Y163*	c.489C>G	Verified	tgc	tac	gaa	3	C	G
TCGA-23-1123	GNAS	ENST00000371085	p.Y163*	c.489C>G	Verified	tgc	tac	gaa	3	C	G
TCGA-23-1123	GNAS	ENST00000371085	p.Y163*	c.489C>G	Verified	tgc	tac	gaa	3	C	G
TCGA-25-1326	GNAS	ENST00000371085	p.F273L	c.819C>G	Unverified	ctc	ttc	aag	3	C	G
TCGA-13-0913	GNAS_ENST	ENST00000371100	p.Y806*	c.2418C>G	Verified	TGC	TAC	GAA	3	C	G
TCGA-23-1123	GNAS_ENST	ENST00000371100	p.Y806*	c.2418C>G	Verified	TGC	TAC	GAA	3	C	G
TCGA-23-1123	GNAS_ENST	ENST00000371100	p.Y806*	c.2418C>G	Verified	TGC	TAC	GAA	3	C	G
TCGA-25-1326	GNAS_ENST	ENST00000371100	p.F916L	c.2748C>G	Unverified	CTC	TTC	AAG	3	C	G
TCGA-29-1701	GOLGA8J_EI	ENST00000567927	p.D571E	c.1713C>G	Unverified	GCA	GAC	AAG	3	C	G
TCGA-24-1604	GOLGB1	ENST00000340645	p.Q1239E	c.3715C>G	Verified	ATT	CAA	GTA	1	C	G
TCGA-23-1110	GON4L	ENST00000361040	p.H303D	c.907C>G	Verified	GAA	CAC	GTG	1	C	G
TCGA-13-1510	GON4L_ENS	ENST00000437809	p.S2103*	c.6308C>G	Unverified	ACC	TCA	GAG	2	C	G
TCGA-29-1695	GORASP2	ENST00000234160	p.P366R	c.1097C>G	Unverified	CTC	CCG	TCA	2	C	G
TCGA-13-0792	GP2	ENST00000381362	p.P51A	c.151C>G	Verified	GCT	CCT	GGC	1	C	G
TCGA-24-1103	GPBAR1	ENST00000479077	p.L246L	c.738C>G	Verified	CTC	CTC	TCA	3	C	G
TCGA-24-1849	GPC5	ENST00000377067	p.Y394*	c.1182C>G	Unverified	CTG	TAC	AGG	3	C	G
TCGA-13-0916	GPI	ENST00000356487	p.S367C	c.1100C>G	Verified	aaa	tct	gga	2	C	G
TCGA-13-0887	GPR101	ENST00000298110	p.D77E	c.231C>G	Verified	acc	gac	ctg	3	C	G
TCGA-13-0904	GPR112	ENST00000394143	p.P538R	c.1613C>G	Verified	tta	ccc	aga	2	C	G
TCGA-61-1740	GPR116	ENST00000265417	p.P1226A	c.3676C>G	Unverified	ACA	CCA	CTC	1	C	G
TCGA-23-1110	GPR124	ENST00000412232	p.P869P	c.2607C>G	Verified	GCA	CCC	CCT	3	C	G
TCGA-20-1683	GPR128	ENST00000273352	p.P115R	c.344C>G	Unverified	AAT	CCA	ATG	2	C	G
TCGA-23-1124	GPR146	ENST00000397095	p.T247R	c.740C>G	Verified	TGG	ACG	CCA	2	C	G
TCGA-61-1914	GPR148	ENST00000309926	p.L183V	c.547C>G	Unverified	TTC	CTT	ATT	1	C	G
TCGA-25-2042	GPR155	ENST00000392551	p.T416S	c.1247C>G	Verified	ACA	ACT	AAT	2	C	G
TCGA-24-2280	GPR161	ENST00000367835	p.T38S	c.113C>G	Unverified	ATC	ACC	ATT	2	C	G
TCGA-13-0900	GPR19	ENST00000332427	p.P405A	c.1213C>G	Verified	TGG	CCC	ATT	1	C	G
TCGA-24-0975	GPR34	ENST00000378142	p.T356S	c.1067C>G	Verified	agc	act	tca	2	C	G
TCGA-24-1850	GPR98	ENST00000405460	p.D1534E	c.4602C>G	Unverified	AAT	GAC	GAG	3	C	G
TCGA-24-1846	GPRASP2	ENST00000543253	p.T52S	c.155C>G	Unverified	aaa	act	gag	2	C	G
TCGA-10-0930	GPX2	ENST00000389614	p.R171R	c.513C>G	Verified	AGC	CGC	ACC	3	C	G
TCGA-10-0930	GPX2	ENST00000389614	p.R171R	c.513C>G	Verified	AGC	CGC	ACC	3	C	G
TCGA-25-1313	GRAMD1C	ENST00000358160	p.L325V	c.973C>G	Verified	GAT	CTT	CAT	1	C	G
TCGA-61-2095	GRB10	ENST00000398812	p.A86A	c.258C>G	Unverified	CAT	GCC	CGC	3	C	G

TCGA-13-0904	GRIK2	ENST00000318991	p.G410G	c.1230C>G	Verified	AGT	GGC	CTG	3	C	G
TCGA-13-0904	GRIK2_ENST	ENST00000421544	p.G410G	c.1230C>G	Verified	AGT	GGC	CTG	3	C	G
TCGA-24-2024	GRLF1_ENST	ENST00000317082_v61	p.S985*	c.2954C>G	Verified	GAT	TCA	GAA	2	C	G
TCGA-29-1783	GRM1	ENST00000282753	p.P369A	c.1105C>G	Unverified	TTC	CCT	GAG	1	C	G
TCGA-29-1783	GRM1_ENST	ENST00000392299	p.P369A	c.1105C>G	Unverified	TTC	CCT	GAG	1	C	G
TCGA-13-0924	GSDMC	ENST00000276708	p.L499L	c.1497C>G	Verified	ACT	CTC	TCG	3	C	G
TCGA-13-0714	GSK3A	ENST00000222330	p.I122M	c.366C>G	Verified	GAC	ATC	AAA	3	C	G
TCGA-61-2109	GSK3A	ENST00000222330	p.H400Q	c.1200C>G	Unverified	GCG	CAC	AGC	3	C	G
TCGA-13-0755	GSS	ENST00000216951	p.Q64E	c.190C>G	Verified	GAG	CAA	GCC	1	C	G
TCGA-13-0923	GSS	ENST00000216951	p.P138A	c.412C>G	Verified	TCC	CCA	GCC	1	C	G
TCGA-23-1124	GTF2IRD2	ENST00000405086	p.T796S	c.2387C>G	Verified	GAG	ACT	CAT	2	C	G
TCGA-04-1530	GTF3C3	ENST00000263956	p.D601E	c.1803C>G	Verified	AAT	GAC	CAA	3	C	G
TCGA-24-2288	GUCY1B3	ENST00000264424	p.A443A	c.1329C>G	Unverified	GGA	GCC	ATG	3	C	G
TCGA-24-2280	GYS1	ENST00000323798	p.L349L	c.1047C>G	Unverified	CGG	CTC	AAC	3	C	G
TCGA-61-1733	GYS2	ENST00000261195	p.I552M	c.1656C>G	Unverified	TAC	ATC	GTT	3	C	G
TCGA-61-1733	GYS2	ENST00000261195	p.I552M	c.1656C>G	Unverified	TAC	ATC	GTT	3	C	G
TCGA-29-1769	HABP4	ENST00000375249	p.G194G	c.582C>G	Unverified	CGC	GGC	AGA	3	C	G
TCGA-13-1498	HAP1	ENST00000347901	p.A522G	c.1565C>G	Verified	CCG	GCT	GAG	2	C	G
TCGA-24-0979	HDAC5	ENST00000393622	p.Q583E	c.1747C>G	Verified	ACA	CAG	GAA	1	C	G
TCGA-13-1497	HEATR5B	ENST00000233099	p.P1922R	c.5765C>G	Verified	ACT	CCT	TAT	2	C	G
TCGA-04-1367	HECTD1	ENST00000399332	p.C1893W	c.5679C>G	Verified	GGT	TGC	TGG	3	C	G
TCGA-13-0904	HECW1	ENST00000395891	p.R859G	c.2575C>G	Verified	CAG	CGT	CCG	1	C	G
TCGA-23-1022	HELQ	ENST00000295488	p.L1046L	c.3138C>G	Verified	GTG	CTC	GTA	3	C	G
TCGA-13-0883	HEPACAM2	ENST00000341723	p.L192V	c.574C>G	Verified	ACC	CTT	CAT	1	C	G
TCGA-20-0991	HERPUD2	ENST00000396081	p.P92P	c.276C>G	Verified	CCT	CCC	AGT	3	C	G
TCGA-61-1998	HEXA	ENST00000268097	p.T217S	c.650C>G	Verified	TTC	ACT	TTT	2	C	G
TCGA-13-1481	HIPK1	NM_152696	p.Y70*	c.210C>G	Verified	gct	tac	gac	3	C	G
TCGA-13-0884	HIPK2	NM_022740	p.S604S	c.1812C>G	Verified	att	tcc	tta	3	C	G
TCGA-25-1326	HIST1H1A	ENST00000244573	p.P169A	c.505C>G	Verified	AAT	CCA	AAA	1	C	G
TCGA-13-1501	HIST1H1A	ENST00000244573	p.T95R	c.284C>G	Verified	GGA	ACG	TTG	2	C	G
TCGA-13-1501	HIST1H1A	ENST00000244573	p.T95R	c.284C>G	Verified	GGA	ACG	TTG	2	C	G
TCGA-24-1616	HIST1H1C	ENST00000343677	p.T4S	c.11C>G	Verified	gag	act	gct	2	C	G
TCGA-23-1117	HIST1H2BD	ENST00000377777	p.A118A	c.354C>G	Verified	AAG	GCC	GTC	3	C	G
TCGA-23-1117	HIST1H2BD	ENST00000377777	p.A118A	c.354C>G	Verified	AAG	GCC	GTC	3	C	G

TCGA-20-1683	HIST1H2BI	ENST00000377733	p.P2R	c.5C>G	Unverified	ATG	CCT	GAA	2	C	G
TCGA-61-1740	HIST1H2BJ	ENST00000541790	p.A111A	c.333C>G	Unverified	CAC	GCC	GTG	3	C	G
TCGA-25-1313	HIST1H3C	ENST00000540144	p.R3G	c.7C>G	Verified	GCT	CGT	ACG	1	C	G
TCGA-13-1481	HIST1H3H	ENST00000369163	p.R9G	c.25C>G	Verified	GCT	CGC	AAG	1	C	G
TCGA-23-1031	HIST1H3J	ENST00000359303	p.R9R	c.27C>G	Verified	GCT	CGC	AAG	3	C	G
TCGA-13-0795	HIST1H4H	ENST00000377727	p.S2C	c.5C>G	Verified	ATG	TCT	GGC	2	C	G
TCGA-04-1362	HIVEP2	ENST00000012134	p.L1693V	c.5077C>G	Verified	GCT	CTT	CTG	1	C	G
TCGA-24-2024	HK1	ENST00000404387	p.L87V	c.259C>G	Verified	GCC	CTG	GAT	1	C	G
TCGA-24-2035	HKDC1	ENST00000354624	p.T811R	c.2432C>G	Verified	AGC	ACG	TGT	2	C	G
TCGA-61-1910	HLA-DPA1	ENST00000419277	p.P186P	c.558C>G	Unverified	GTG	CCC	TCA	3	C	G
TCGA-09-2051	HLX	ENST00000366903	p.Q270E	c.808C>G	Unverified	CCG	CAG	ACG	1	C	G
TCGA-09-2049	HMBOX1	ENST00000397358	p.R268G	c.802C>G	Verified	CGA	CGA	GGG	1	C	G
TCGA-61-1904	HMCN1	ENST00000271588	p.Q1161E	c.3481C>G	Unverified	ACT	CAG	GCT	1	C	G
TCGA-13-0762	HMCN1	ENST00000271588	p.S1798*	c.5393C>G	Verified	GTG	TCA	AAC	2	C	G
TCGA-13-0762	HMCN1	ENST00000271588	p.S1798*	c.5393C>G	Verified	GTG	TCA	AAC	2	C	G
TCGA-61-1725	HMCN1	ENST00000271588	p.P1919R	c.5756C>G	Unverified	CCA	CCT	AGT	2	C	G
TCGA-61-1914	HNF4A	ENST00000316099	p.P51R	c.152C>G	Unverified	gcg	ccc	aac	2	C	G
TCGA-61-1914	HNF4A_ENS	ENST00000443598	p.P51R	c.152C>G	Unverified	GCG	CCC	AAC	2	C	G
TCGA-24-1103	HNRNPA2B1	ENST00000356674	p.G270G	c.810C>G	Verified	GGG	GGC	TAC	3	C	G
TCGA-25-1313	HPN	ENST00000392226	p.I332M	c.996C>G	Verified	CAG	ATC	AAG	3	C	G
TCGA-61-1738	HR	ENST00000381418	p.H1055Q	c.3165C>G	Unverified	TGG	CAC	GTG	3	C	G
TCGA-61-1738	HR	ENST00000381418	p.H1055Q	c.3165C>G	Unverified	TGG	CAC	GTG	3	C	G
TCGA-29-1781	HSD3B7	ENST00000297679	p.H155Q	c.465C>G	Unverified	GTG	CAC	AGG	3	C	G
TCGA-29-1703	HSDL2	ENST00000398805	p.L321V	c.961C>G	Unverified	TCT	CTC	AGT	1	C	G
TCGA-29-1784	HSPA1L	ENST00000375654	p.P178R	c.533C>G	Unverified	GAG	CCC	ACG	2	C	G
TCGA-29-1693	HSPB8	ENST00000281938	p.R18G	c.52C>G	Unverified	CGC	CGA	GAC	1	C	G
TCGA-29-1693	HSPB8_ENS	ENST00000281938	p.R18G	c.52C>G	Unverified	CGC	CGA	GAC	1	C	G
TCGA-23-1021	HTR2A	ENST00000378688	p.Q398E	c.1192C>G	Verified	TGT	CAG	TAC	1	C	G
TCGA-13-0913	HTR4	ENST00000360693	p.P290R	c.869C>G	Verified	GTC	CCT	GGG	2	C	G
TCGA-09-2050	HUWE1	XM_497119.1	p.S1766*	c.5297C>G	Verified	ggc	tca	gga	2	C	G
TCGA-09-2051	HYAL4	ENST00000223026	p.L79V	c.235C>G	Unverified	CCA	CTG	GCC	1	C	G
TCGA-04-1338	IBTK	ENST00000306270	p.D769E	c.2307C>G	Verified	TGT	GAC	GTG	3	C	G
TCGA-61-1725	ICOS	ENST00000316386	p.T188R	c.563C>G	Unverified	AAC	ACA	GCC	2	C	G
TCGA-30-1855	IDS	ENST00000340855	p.P371A	c.1111C>G	Unverified	ctt	ccg	gag	1	C	G

TCGA-04-1649	IFI44L	ENST00000342282	p.D127E	c.381C>G	Unverified	CTA	GAC	GAC	3	C	G
TCGA-04-1649	IFI44L_ENST	ENST00000370751	p.D166E	c.498C>G	Unverified	CTA	GAC	GAC	3	C	G
TCGA-13-0807	IFNA1	ENST00000276927	p.R148G	c.442C>G	Verified	TTC	CGA	AGA	1	C	G
TCGA-04-1652	IFNA21	ENST00000380225	p.P49A	c.145C>G	Unverified	TCT	CCT	TTC	1	C	G
TCGA-04-1652	IFNA21	ENST00000380225	p.P49A	c.145C>G	Unverified	TCT	CCT	TTC	1	C	G
TCGA-23-1123	IFNA5	ENST00000259555	p.T110S	c.329C>G	Verified	TAC	ACT	GAA	2	C	G
TCGA-23-1123	IFNA5	ENST00000259555	p.T110S	c.329C>G	Verified	TAC	ACT	GAA	2	C	G
TCGA-13-1497	IFT52	ENST00000373039	p.P319A	c.955C>G	Verified	GAA	CCA	CTC	1	C	G
TCGA-24-1435	IFT81	ENST00000361948	p.S307C	c.920C>G	Verified	CAT	TCT	GAT	2	C	G
TCGA-13-0890	IGF2R	ENST00000356956	p.R1949G	c.5845C>G	Verified	TAC	CGG	ACA	1	C	G
TCGA-24-1845	IGSF1	ENST00000361420	p.S1227R	c.3681C>G	Unverified	TGC	AGC	TAC	3	C	G
TCGA-04-1347	IGSF10	ENST00000282466	p.P1082R	c.3245C>G	Verified	TTT	CCA	AGT	2	C	G
TCGA-13-1498	IKZF4_ENST(	ENST00000262032	p.I392M	c.1176C>G	Verified	TGC	ATC	TCA	3	C	G
TCGA-23-1032	IL28A	ENST00000331982	p.S67W	c.200C>G	Verified	GAG	TCG	CTT	2	C	G
TCGA-13-0923	IL4I1	ENST00000341114	p.R124G	c.370C>G	Verified	TAC	CGG	GAC	1	C	G
TCGA-24-2288	ILF3	ENST00000318511	p.T238S	c.713C>G	Unverified	TGC	ACT	CGC	2	C	G
TCGA-24-2267	IMPAD1	ENST00000262644	p.A269G	c.806C>G	Verified	GGT	GCT	GGT	2	C	G
TCGA-09-2051	INPP4A	ENST00000074304	p.R456G	c.1366C>G	Unverified	ACA	CGG	CAG	1	C	G
TCGA-25-2398	INPP5D	NM_005541.2	p.F28L	c.84C>G	Verified	agc	ttc	ctc	3	C	G
TCGA-30-1855	INPP5F	ENST00000361976	p.I235M	c.705C>G	Unverified	atg	atc	caa	3	C	G
TCGA-61-1740	INPPL1	ENST00000298229	p.A785A	c.2355C>G	Unverified	GAT	GCC	CAG	3	C	G
TCGA-20-1685	INSR	ENST00000302850	p.S636S	c.1908C>G	Unverified	TCA	TCC	CAG	3	C	G
TCGA-20-1685	INSR_ENST0	ENST00000302850	p.S636S	c.1908C>G	Unverified	TCA	TCC	CAG	3	C	G
TCGA-23-1118	INSRR	ENST00000368195	p.P160R	c.479C>G	Verified	CAG	CCA	GCA	2	C	G
TCGA-04-1343	INTS4	ENST00000534064	p.V184V	c.552C>G	Verified	AGT	GTC	ACA	3	C	G
TCGA-13-0906	INVS	ENST00000262457	p.I447M	c.1341C>G	Verified	AAG	ATC	AAT	3	C	G
TCGA-29-1691	IPP	ENST00000396478	p.A10G	c.29C>G	Unverified	GCT	GCT	GAT	2	C	G
TCGA-04-1336	IQCC	ENST00000291358	p.R38G	c.112C>G	Verified	GTA	CGA	GAG	1	C	G
TCGA-25-2392	IQCD	ENST00000299732	p.S133C	c.398C>G	Unverified	CTT	TCC	ATA	2	C	G
TCGA-29-1699	IQCH	ENST00000335894	p.P537R	c.1610C>G	Unverified	ACA	CCT	GAA	2	C	G
TCGA-61-1737	IQGAP2	ENST00000274364	p.L1535V	c.4603C>G	Unverified	CAA	CTC	AAT	1	C	G
TCGA-13-1512	IQGAP3	ENST00000361170	p.L1130V	c.3388C>G	Verified	TTC	CTT	TTA	1	C	G
TCGA-13-1489	IQGAP3	ENST00000361170	p.A487A	c.1461C>G	Verified	GAT	GCC	CTG	3	C	G
TCGA-61-1900	IRAK4	ENST00000448290	p.P148A	c.442C>G	Unverified	CCA	CCT	GAC	1	C	G

TCGA-61-1900	IRAK4_ENST	ENST00000448290	p.P148A	c.442C>G	Unverified	CCA	CCT	GAC	1	C	G
TCGA-09-2051	IRX4	NM_016358.1	p.G143G	c.429C>G	Unverified	agc	ggc	acg	3	C	G
TCGA-61-2113	ISLR2	ENST00000361742	p.P560R	c.1679C>G	Unverified	CGG	CCG	GGT	2	C	G
TCGA-61-2009	ITGA3	ENST00000007722	p.P364R	c.1091C>G	Verified	GGC	CCC	AGT	2	C	G
TCGA-13-0920	ITGA5	ENST00000293379	p.S769R	c.2307C>G	Verified	CTC	AGC	AAG	3	C	G
TCGA-29-1691	ITGAL	ENST00000356798	p.P994R	c.2981C>G	Unverified	GAG	CCT	CCC	2	C	G
TCGA-04-1338	ITIH5	ENST00000256861	p.T410R	c.1229C>G	Verified	CCC	ACG	GTC	2	C	G
TCGA-13-0714	ITPKB	NM_002221	p.T435S	c.1304C>G	Verified	cag	acc	ctg	2	C	G
TCGA-23-1116	ITPKC	ENST00000263370	p.L449V	c.1345C>G	Unverified	CCG	CTG	CGA	1	C	G
TCGA-61-1740	ITPR2	ENST00000381340	p.A591G	c.1772C>G	Unverified	TTG	GCA	GAA	2	C	G
TCGA-23-1809	ITPR3	ENST00000374316	p.L651L	c.1953C>G	Unverified	GAG	CTC	ATC	3	C	G
TCGA-23-1809	ITPR3_ENST	ENST00000374316	p.L651L	c.1953C>G	Unverified	GAG	CTC	ATC	3	C	G
TCGA-09-1665	ITSN1	ENST00000381318	p.L1487L	c.4461C>G	Verified	TTC	CTC	CTG	3	C	G
TCGA-20-1687	ITSN2	ENST00000355123	p.P133A	c.397C>G	Unverified	CCT	CCA	GCT	1	C	G
TCGA-29-1766	ITSN2	ENST00000355123	p.L1426V	c.4276C>G	Unverified	TCT	CTC	ACC	1	C	G
TCGA-23-1122	ITSN2	ENST00000355123	p.S330C	c.989C>G	Verified	CCA	TCT	TTC	2	C	G
TCGA-20-1687	ITSN2_ENST	ENST00000406921	p.P133A	c.397C>G	Unverified	CCT	CCA	GCT	1	C	G
TCGA-29-1775	IVL	ENST00000368764	p.Q505E	c.1513C>G	Unverified	GAA	CAG	CAG	1	C	G
TCGA-23-1117	IZUMO1	ENST00000332955	p.A243A	c.729C>G	Verified	CCA	GCC	ACG	3	C	G
TCGA-23-1117	IZUMO1	ENST00000332955	p.A243A	c.729C>G	Verified	CCA	GCC	ACG	3	C	G
TCGA-29-1775	JAKMIP2	ENST00000265272	p.Q400E	c.1198C>G	Unverified	CAA	CAG	AAC	1	C	G
TCGA-23-1114	JPH2	ENST00000372980	p.P576P	c.1728C>G	Unverified	CCG	CCC	GAG	3	C	G
TCGA-04-1337	KATNA1	ENST00000335647	p.I313M	c.939C>G	Unverified	TCC	ATC	TGT	3	C	G
TCGA-61-2095	KAZALD1	ENST00000370200	p.L106V	c.316C>G	Unverified	CAG	CTT	GAG	1	C	G
TCGA-09-2050	KCNA4	ENST00000328224	p.R68G	c.202C>G	Verified	TCA	CGC	GGG	1	C	G
TCGA-13-0761	KCND1	ENST00000218176	p.P639P	c.1917C>G	Verified	ttc	ccc	gag	3	C	G
TCGA-13-0761	KCND1	ENST00000218176	p.P639P	c.1917C>G	Verified	ttc	ccc	gag	3	C	G
TCGA-04-1342	KCND2	ENST00000331113	p.V294V	c.882C>G	Unverified	CGA	GTC	TTC	3	C	G
TCGA-61-1907	KCNH7	ENST00000332142	p.A745A	c.2235C>G	Unverified	AAA	GCC	TTT	3	C	G
TCGA-24-2280	KCNH8	ENST00000328405	p.R166G	c.496C>G	Unverified	AGA	CGG	AGT	1	C	G
TCGA-13-0807	KCNK16	ENST00000373229	p.G286G	c.858C>G	Verified	AGA	GGC	TCT	3	C	G
TCGA-25-1326	KCNMB1	ENST00000274629	p.T30T	c.90C>G	Verified	ATC	ACC	TAC	3	C	G
TCGA-24-1847	KCNMB3	ENST00000314235	p.P27A	c.79C>G	Unverified	TTT	CCT	GCC	1	C	G
TCGA-24-1847	KCNMB3_EN	ENST00000349697	p.P25A	c.73C>G	Unverified	TTT	CCT	GCC	1	C	G

TCGA-24-1847	KCNMB3_EN	ENST00000392685	p.P23A	c.67C>G	Unverified	TTT	CCT	GCC	1	C	G
TCGA-24-1847	KCNMB3_EN	ENST00000497599	p.P25A	c.73C>G	Unverified	TTT	CCT	GCC	1	C	G
TCGA-13-0905	KCNQ3	ENST00000388996	p.P608R	c.1823C>G	Verified	AGA	CCA	TCC	2	C	G
TCGA-61-1740	KCNQ3	ENST00000388996	p.L261L	c.783C>G	Unverified	GAA	CTC	ATC	3	C	G
TCGA-61-1914	KCTD19	ENST00000304372	p.S44*	c.131C>G	Unverified	GCT	TCA	GCC	2	C	G
TCGA-13-1498	KDM3A	ENST00000409556	p.P876P	c.2628C>G	Verified	ACA	CCC	GTA	3	C	G
TCGA-24-1474	KEAP1	ENST00000393623	p.N189K	c.567C>G	Unverified	GCC	AAC	TTC	3	C	G
TCGA-13-0883	KHDRBS2	ENST00000281156	p.T229T	c.687C>G	Verified	GTA	ACC	CGT	3	C	G
TCGA-25-2391	KIAA0195	ENST00000314256	p.S880S	c.2640C>G	Unverified	ATC	TCC	CTC	3	C	G
TCGA-25-2042	KIAA0415_E	ENST00000450194	p.T578R	c.1733C>G	Unverified	CAC	ACG	GTC	2	C	G
TCGA-13-0760	KIAA0564	ENST00000379310	p.S1450C	c.4349C>G	Verified	aca	tct	ggt	2	C	G
TCGA-09-2051	KIAA0889	ENST00000237536	p.R276G	c.826C>G	Unverified	CTG	CGC	AAA	1	C	G
TCGA-61-2012	KIAA0947_E	ENST00000296564	p.P1693R	c.5078C>G	Verified	TCT	CCT	CCA	2	C	G
TCGA-29-1761	KIAA1267	ENST00000262419	p.A440G	c.1319C>G	Unverified	GCT	GCA	GAC	2	C	G
TCGA-24-1463	KIAA1383	ENST00000418460	p.P512A	c.1534C>G	Verified	CCT	CCA	CAT	1	C	G
TCGA-25-2398	KIAA1462	ENST00000375377	p.P713R	c.2138C>G	Unverified	GGG	CCG	AGT	2	C	G
TCGA-23-1032	KIAA1797	ENST00000380249	p.P738A	c.2212C>G	Verified	ATT	CCT	GAA	1	C	G
TCGA-04-1367	KIAA2018	ENST00000316407	p.P185A	c.553C>G	Verified	GTG	CCA	GTA	1	C	G
TCGA-13-0760	KIAA2026_E	ENST00000399933_v61	p.A306G	c.917C>G	Verified	CCA	GCT	ATT	2	C	G
TCGA-29-1696	KIF20B	ENST00000260753	p.Q1344E	c.4030C>G	Unverified	GAA	CAG	GAA	1	C	G
TCGA-29-1696	KIF20B	ENST00000260753	p.Q1344E	c.4030C>G	Unverified	GAA	CAG	GAA	1	C	G
TCGA-13-0887	KIF21B	ENST00000332129	p.S1019S	c.3057C>G	Verified	ACA	TCC	GTG	3	C	G
TCGA-13-0906	KIF5A	ENST00000455537	p.T938S	c.2813C>G	Verified	GGC	ACC	CGG	2	C	G
TCGA-13-1488	KLHDC7A	ENST00000400664	p.A25G	c.74C>G	Verified	TCA	GCC	GCT	2	C	G
TCGA-29-1698	KLHDC7A	ENST00000400664	p.P392R	c.1175C>G	Unverified	GAC	CCG	GGC	2	C	G
TCGA-13-1501	KLHDC8A	ENST00000367155	p.A117A	c.351C>G	Verified	GCC	GCC	ATG	3	C	G
TCGA-13-1501	KLHDC8A	ENST00000367155	p.A117A	c.351C>G	Verified	GCC	GCC	ATG	3	C	G
TCGA-24-1843	KLHL11	ENST00000319121	p.L448V	c.1342C>G	Unverified	GGA	CTA	ACA	1	C	G
TCGA-61-2109	KLHL24	ENST00000242810	p.Y593*	c.1779C>G	Unverified	AGA	TAC	AAT	3	C	G
TCGA-61-2008	KLHL34	ENST00000379499	p.R553G	c.1657C>G	Unverified	ACT	CGG	TTG	1	C	G
TCGA-04-1331	KLHL7	ENST00000322231	p.A535G	c.1604C>G	Unverified	GTT	GCC	AAC	2	C	G
TCGA-23-2078	KLHL9	ENST00000359039	p.S288*	c.863C>G	Verified	CAG	TCA	GAT	2	C	G
TCGA-61-1915	KLRK1	ENST00000240618	p.Q85E	c.253C>G	Unverified	AAC	CAA	GAA	1	C	G
TCGA-23-1122	KPTN	ENST00000338134	p.S345W	c.1034C>G	Verified	GAG	TCG	GGG	2	C	G

TCGA-04-1338	KRT38	ENST00000246646	p.S198S	c.594C>G	Unverified	CGC	TCC	CTG	3	C	G
TCGA-04-1336	KRT6A	ENST00000330722	p.S31C	c.92C>G	Verified	CGC	TCT	GGC	2	C	G
TCGA-24-1846	KRT7	ENST00000331817	p.L361V	c.1081C>G	Unverified	GCC	CTG	CAG	1	C	G
TCGA-61-1913	KRTCAP3	ENST00000288873	p.L86V	c.256C>G	Unverified	AAC	CTT	CTT	1	C	G
TCGA-09-1674	KSR2	SU_KSR2	p.L491V	c.1471C>G	Unverified	ctt	ctg	atc	1	C	G
TCGA-09-1674	KSR2_ENST(	ENST00000302438	p.L156V	c.466C>G	Unverified	CTT	CTG	ATC	1	C	G
TCGA-13-0905	L1CAM	ENST00000370060	p.V841V	c.2523C>G	Verified	CAG	GTC	AAG	3	C	G
TCGA-23-1029	LAMA1	ENST00000389658	p.H881Q	c.2643C>G	Unverified	GCC	CAC	TGT	3	C	G
TCGA-61-1907	LAMA3	ENST00000313654	p.P750A	c.2248C>G	Unverified	gat	ccg	ctg	1	C	G
TCGA-13-0913	LAMA3	ENST00000313654	p.P1093P	c.3279C>G	Verified	ctg	ccc	cag	3	C	G
TCGA-24-1846	LAMB1	ENST00000222399	p.A1475G	c.4424C>G	Unverified	GAG	GCA	AAA	2	C	G
TCGA-13-1509	LAMC1	ENST00000258341	p.P327R	c.980C>G	Verified	CGG	CCG	TGG	2	C	G
TCGA-24-1850	LAMC1	ENST00000258341	p.D169E	c.507C>G	Unverified	GAA	GAC	GGG	3	C	G
TCGA-13-0884	LAMC2	ENST00000264144	p.P1182A	c.3544C>G	Verified	CCC	CCA	GGC	1	C	G
TCGA-13-0903	LARGE	ENST00000397394	p.D470E	c.1410C>G	Verified	GCA	GAC	AGC	3	C	G
TCGA-61-1907	LARS2	ENST00000265537	p.I736M	c.2208C>G	Unverified	GTC	ATC	TCT	3	C	G
TCGA-29-1762	LAT2	ENST00000398475	p.A22G	c.65C>G	Unverified	GCA	GCC	AGT	2	C	G
TCGA-23-2078	LBR	ENST00000272163	p.A94G	c.281C>G	Unverified	agt	gcc	cgc	2	C	G
TCGA-13-0913	LBR	ENST00000272163	p.L429L	c.1287C>G	Verified	ctt	ctc	tat	3	C	G
TCGA-30-1857	LCT	ENST00000264162	p.S101R	c.303C>G	Unverified	GGA	AGC	ACC	3	C	G
TCGA-61-1998	LECT1	ENST00000377962	p.P305R	c.914C>G	Verified	TAC	CCA	TGG	2	C	G
TCGA-29-1769	LEPR_ENST(	ENST00000349533	p.S905*	c.2714C>G	Unverified	GCA	TCA	GTG	2	C	G
TCGA-61-1906	LETM2	ENST00000297720	p.A49G	c.146C>G	Unverified	CAA	GCC	ACA	2	C	G
TCGA-61-1906	LETM2	ENST00000297720	p.A49G	c.146C>G	Unverified	CAA	GCC	ACA	2	C	G
TCGA-61-1906	LETM2_ENST	ENST00000379957	p.A96G	c.287C>G	Unverified	CAA	GCC	ACA	2	C	G
TCGA-61-1906	LETM2_ENST	ENST00000379957	p.A96G	c.287C>G	Unverified	CAA	GCC	ACA	2	C	G
TCGA-29-1777	LGALS2	ENST00000215886	p.Q69E	c.205C>G	Unverified	GAA	CAA	CGG	1	C	G
TCGA-61-2113	LGI1	ENST00000371418	p.P204R	c.611C>G	Verified	CCC	CCA	GAA	2	C	G
TCGA-23-1120	LIMCH1	ENST00000313860	p.T932R	c.2795C>G	Verified	GGA	ACA	AAC	2	C	G
TCGA-25-1326	LIMK2	ENST00000331728	p.I325M	c.975C>G	Verified	CAG	ATC	TTC	3	C	G
TCGA-29-1762	LIPH	ENST00000296252	p.L39V	c.115C>G	Unverified	GGA	CTA	AAT	1	C	G
TCGA-61-1910	LLGL2	ENST00000392550	p.S704W	c.2111C>G	Unverified	cgc	tcg	gca	2	C	G
TCGA-61-1910	LLGL2_ENST	ENST00000167462	p.S704W	c.2111C>G	Unverified	CGC	TCG	GCA	2	C	G
TCGA-13-1507	LMCD1	ENST00000157600	p.L326V	c.976C>G	Verified	GAT	CTG	GCC	1	C	G

TCGA-24-1847	LMOD3	ENST00000420581	p.P49P	c.147C>G	Unverified	CTT	CCC	GTG	3	C	G
TCGA-24-1847	LMOD3_ENS	ENST00000420581	p.P49P	c.147C>G	Unverified	CTT	CCC	GTG	3	C	G
TCGA-24-1103	LOC51059	ENST00000395297	p.T1259T	c.3777C>G	Verified	AGC	ACC	CTG	3	C	G
TCGA-23-1022	LOC652153	ENST00000316490	p.P2147R	c.6440C>G	Verified	AGT	CCC	AGT	2	C	G
TCGA-29-1766	LOC728378	ENST00000357462	p.Y245*	c.735C>G	Unverified	CAC	TAC	GCT	3	C	G
TCGA-29-1783	LOC81691	ENST00000261377	p.G76G	c.228C>G	Unverified	CTG	GGC	AAA	3	C	G
TCGA-24-1850	LPHN2	ENST00000359929	p.R66G	c.196C>G	Unverified	GGT	CGG	ACG	1	C	G
TCGA-24-1850	LPHN2_ENS	ENST00000370725	p.R66G	c.196C>G	Unverified	GGT	CGG	ACG	1	C	G
TCGA-24-1469	LPHN3	ENST00000512091	p.L860V	c.2578C>G	Unverified	GTA	CTG	ATG	1	C	G
TCGA-13-0885	LPHN3	ENST00000512091	p.T659R	c.1976C>G	Unverified	ACT	ACG	AGT	2	C	G
TCGA-24-1616	LPHN3	ENST00000512091	p.T783R	c.2348C>G	Unverified	ATT	ACG	GCA	2	C	G
TCGA-24-1469	LPHN3_ENS	ENST00000512091	p.L860V	c.2578C>G	Unverified	GTA	CTG	ATG	1	C	G
TCGA-13-0885	LPHN3_ENS	ENST00000512091	p.T659R	c.1976C>G	Unverified	ACT	ACG	AGT	2	C	G
TCGA-24-1616	LPHN3_ENS	ENST00000512091	p.T783R	c.2348C>G	Unverified	ATT	ACG	GCA	2	C	G
TCGA-24-1469	LPHN3_ENS	ENST00000514591	p.L860V	c.2578C>G	Unverified	GTA	CTG	ATG	1	C	G
TCGA-13-0885	LPHN3_ENS	ENST00000514591	p.T659R	c.1976C>G	Unverified	ACT	ACG	AGT	2	C	G
TCGA-24-1616	LPHN3_ENS	ENST00000514591	p.T783R	c.2348C>G	Unverified	ATT	ACG	GCA	2	C	G
TCGA-61-1900	LPIN1	ENST00000256720	p.S60S	c.180C>G	Unverified	CGC	TCC	CGA	3	C	G
TCGA-29-1776	LPPR4	ENST00000370185	p.Q635E	c.1903C>G	Unverified	ATA	CAG	ATC	1	C	G
TCGA-29-1761	LRBA	ENST00000357115	p.Q2860E	c.8578C>G	Unverified	TAC	CAA	ACC	1	C	G
TCGA-10-0930	LRIF1	ENST00000369763	p.Q716E	c.2146C>G	Verified	GAA	CAA	AGT	1	C	G
TCGA-10-0930	LRIF1	ENST00000369763	p.Q716E	c.2146C>G	Verified	GAA	CAA	AGT	1	C	G
TCGA-13-0885	LRIF1	ENST00000369763	p.T432T	c.1296C>G	Verified	AAT	ACC	CAG	3	C	G
TCGA-61-1906	LRIT1	ENST00000372105	p.P387A	c.1159C>G	Unverified	ATT	CCC	AAA	1	C	G
TCGA-61-1906	LRIT1	ENST00000372105	p.P387A	c.1159C>G	Unverified	ATT	CCC	AAA	1	C	G
TCGA-29-1770	LRMP	ENST00000354454	p.S354*	c.1061C>G	Unverified	ggg	tca	aag	2	C	G
TCGA-13-1408	LRP1	ENST00000243077	p.N2048K	c.6144C>G	Verified	GTC	AAC	GTC	3	C	G
TCGA-13-0906	LRP10	ENST00000359591	p.R235G	c.703C>G	Unverified	GTG	CGC	TTC	1	C	G
TCGA-23-1114	LRP1B	ENST00000389484	p.H3241D	c.9721C>G	Unverified	GCC	CAT	AAA	1	C	G
TCGA-04-1367	LRP5	ENST00000294304	p.L458V	c.1372C>G	Unverified	GAC	CTG	GAC	1	C	G
TCGA-61-1895	LRP6	ENST00000261349	p.R985G	c.2953C>G	Unverified	TCA	CGA	CAA	1	C	G
TCGA-29-1705	LRPPRC	ENST00000260665	p.P636A	c.1906C>G	Unverified	GTT	CCT	GAA	1	C	G
TCGA-13-0904	LRRC16B	ENST00000342740	p.L849L	c.2547C>G	Verified	GAG	CTC	TAC	3	C	G
TCGA-24-1844	LRRC20	ENST00000373224	p.N126K	c.378C>G	Unverified	ATC	AAC	CTG	3	C	G

TCGA-29-1764	LRRC37B	ENST00000341671	p.P252A	c.754C>G	Unverified	CCT	CCT	GAG	1	C	G
TCGA-13-1507	LRRC4C	ENST00000278198	p.Q87E	c.259C>G	Verified	AAC	CAA	ATC	1	C	G
TCGA-24-1849	LRRC59	ENST00000225972	p.I283M	c.849C>G	Unverified	ACC	ATC	TAT	3	C	G
TCGA-61-2009	LRRC7	ENST00000035383	p.R72G	c.214C>G	Verified	CTA	CGA	AAA	1	C	G
TCGA-09-2051	LRRC8A	ENST00000372599	p.R389G	c.1165C>G	Unverified	AAG	CGC	TTC	1	C	G
TCGA-04-1651	LRRN2	ENST00000367175	p.R427G	c.1279C>G	Unverified	CCA	CGA	AGC	1	C	G
TCGA-04-1651	LRRN2	ENST00000367175	p.R427G	c.1279C>G	Unverified	CCA	CGA	AGC	1	C	G
TCGA-61-2109	LRRTM4	ENST00000409088	p.R227G	c.679C>G	Unverified	CCA	CGT	CTC	1	C	G
TCGA-13-1509	LTBP4_ENST	ENST00000308370	p.L578L	c.1734C>G	Verified	CGG	CTC	AGC	3	C	G
TCGA-29-1691	LTN1	ENST00000361371	p.S295C	c.884C>G	Unverified	GCA	TCC	AAA	2	C	G
TCGA-61-2009	LUZP2	ENST00000336930	p.A175G	c.524C>G	Verified	AAG	GCG	CAG	2	C	G
TCGA-29-1764	LY75	ENST00000263636	p.P919A	c.2755C>G	Unverified	AAA	CCA	ACA	1	C	G
TCGA-04-1331	LZTS2	ENST00000370220	p.S191C	c.572C>G	Verified	TCC	TCC	TCT	2	C	G
TCGA-25-2393	MACF1	ENST00000360115	p.L306V	c.916C>G	Verified	GCC	CTA	GAA	1	C	G
TCGA-29-1781	MACF1	ENST00000360115	p.H4811D	c.14431C>G	Unverified	CAC	CAT	GTC	1	C	G
TCGA-13-1507	MACF1	ENST00000360115	p.A4559G	c.13676C>G	Verified	GCA	GCT	CTC	2	C	G
TCGA-29-1781	MACF1_ENS	ENST00000361689	p.H4309D	c.12925C>G	Unverified	CAC	CAT	GTC	1	C	G
TCGA-30-1855	MACF1_ENS	ENST00000361689	p.Q437E	c.1309C>G	Unverified	ATC	CAG	AAT	1	C	G
TCGA-13-1507	MACF1_ENS	ENST00000361689	p.A4057G	c.12170C>G	Verified	GCA	GCT	CTC	2	C	G
TCGA-29-1763	MAGEA11_E	ENST00000355220	p.S153C	c.458C>G	Unverified	TCC	TCT	ACT	2	C	G
TCGA-04-1530	MAGEB1	ENST00000378981	p.S40S	c.120C>G	Unverified	ccc	tcc	tcc	3	C	G
TCGA-61-1740	MAGEC2	ENST00000247452	p.T126S	c.377C>G	Unverified	GGC	ACC	TGT	2	C	G
TCGA-13-0916	MAGI3	ENST00000369615	p.L897V	c.2689C>G	Verified	AAA	CTG	AAA	1	C	G
TCGA-24-0979	MAML3	ENST00000509479	p.P421R	c.1262C>G	Verified	ACT	CCA	AAC	2	C	G
TCGA-24-2288	MAMLD1	NM_005491.1	p.L315V	c.943C>G	Unverified	tta	ctg	tca	1	C	G
TCGA-10-0930	MAN2A2	ENST00000360468	p.L310V	c.928C>G	Verified	ATG	CTG	ATT	1	C	G
TCGA-10-0930	MAN2A2	ENST00000360468	p.L310V	c.928C>G	Verified	ATG	CTG	ATT	1	C	G
TCGA-23-1123	MAN2B1	ENST00000221363	p.I100M	c.300C>G	Verified	TAC	ATC	CTG	3	C	G
TCGA-23-1123	MAN2B1	ENST00000221363	p.I100M	c.300C>G	Verified	TAC	ATC	CTG	3	C	G
TCGA-24-1103	MAP2	ENST00000360351	p.H27D	c.79C>G	Verified	TCA	CAT	CCA	1	C	G
TCGA-24-1469	MAP2	ENST00000360351	p.S626S	c.1878C>G	Verified	GAA	TCC	CAG	3	C	G
TCGA-13-1509	MAP3K2	ENST00000409947	p.T464S	c.1391C>G	Verified	TAC	ACC	CGT	2	C	G
TCGA-30-1857	MAP3K6_EN	ENST00000374040	p.P948R	c.2843C>G	Unverified	CCA	CCC	AGC	2	C	G
TCGA-30-1857	MAP3K6_EN	ENST00000493901	p.P956R	c.2867C>G	Unverified	CCA	CCC	AGC	2	C	G

TCGA-61-1740	MAPK1	ENST00000215832	p.L157L	c.471C>G	Unverified	CTG	CTC	AAC	3	C	G
TCGA-10-0930	MARCO	ENST00000327097	p.G151G	c.453C>G	Verified	CAA	GGC	GCC	3	C	G
TCGA-10-0930	MARCO	ENST00000327097	p.G151G	c.453C>G	Verified	CAA	GGC	GCC	3	C	G
TCGA-25-1326	MARK1	ENST00000366917	p.S403S	c.1209C>G	Verified	CAG	TCC	CCT	3	C	G
TCGA-61-2008	MARS2	ENST00000282276	p.F297L	c.891C>G	Verified	GAG	TTC	AAA	3	C	G
TCGA-25-2392	MAST2	NM_015112	p.L91V	c.271C>G	Verified	caa	ctg	agt	1	C	G
TCGA-25-2392	MAST2_ENS	ENST00000361297	p.L91V	c.271C>G	Verified	CAA	CTG	AGT	1	C	G
TCGA-13-0920	MATR3	ENST00000394805	p.S240W	c.719C>G	Verified	ACC	TCG	CAT	2	C	G
TCGA-29-1775	MATR3	ENST00000394805	p.D20E	c.60C>G	Unverified	CGT	GAC	CTG	3	C	G
TCGA-29-1775	MATR3_ENS	ENST00000337359	p.D20E	c.60C>G	Unverified	CGT	GAC	CTG	3	C	G
TCGA-13-0913	MB21D2	ENST00000392452	p.T434S	c.1301C>G	Verified	AGC	ACC	ACC	2	C	G
TCGA-13-0755	MBNL1	ENST00000282486	p.S28*	c.83C>G	Verified	TGC	TCA	CGG	2	C	G
TCGA-13-0755	MBNL1_ENS	ENST00000545754	p.S28*	c.83C>G	Verified	TGC	TCA	CGG	2	C	G
TCGA-23-1032	MCF2L_ENS	ENST00000375601	p.S7C	c.20C>G	Unverified	GCA	TCC	CGG	2	C	G
TCGA-13-0916	MDC1_ENST	ENST00000376406	p.P1655R	c.4964C>G	Verified	GAA	CCA	GCA	2	C	G
TCGA-24-0975	ME3	ENST00000393324	p.T451R	c.1352C>G	Unverified	TGC	ACG	GCT	2	C	G
TCGA-10-0930	MED12	ENST00000374080	p.H1508Q	c.4524C>G	Verified	GTG	CAC	CAG	3	C	G
TCGA-10-0930	MED12	ENST00000374080	p.H1508Q	c.4524C>G	Verified	GTG	CAC	CAG	3	C	G
TCGA-10-0930	MED12	ENST00000374080	p.H1508Q	c.4524C>G	Verified	GTG	CAC	CAG	3	C	G
TCGA-10-0930	MED12	ENST00000374080	p.H1508Q	c.4524C>G	Verified	GTG	CAC	CAG	3	C	G
TCGA-10-0930	MED12_ENS	ENST00000374080	p.H1508Q	c.4524C>G	Verified	GTG	CAC	CAG	3	C	G
TCGA-10-0930	MED12_ENS	ENST00000374080	p.H1508Q	c.4524C>G	Verified	GTG	CAC	CAG	3	C	G
TCGA-13-0791	MEF2D	ENST00000348159	p.T80S	c.239C>G	Verified	CGC	ACC	AAC	2	C	G
TCGA-61-1915	MEST	ENST00000223215	p.T169S	c.506C>G	Unverified	CTT	ACC	ATA	2	C	G
TCGA-29-1762	METTL21A	ENST00000411432	p.H161D	c.481C>G	Unverified	GAA	CAT	CTC	1	C	G
TCGA-23-1031	MFSD6	ENST00000392328	p.P218R	c.653C>G	Verified	GCT	CCA	AAC	2	C	G
TCGA-24-2289	MGA	XM_031689.7	p.V1228V	c.3684C>G	Unverified	cca	gtc	tac	3	C	G
TCGA-13-1496	MIA3	ENST00000344922	p.F388L	c.1164C>G	Verified	ATC	TTC	TCT	3	C	G
TCGA-24-1103	MKI67	ENST00000368654	p.L2956V	c.8866C>G	Verified	CCT	CTA	AAA	1	C	G
TCGA-25-1326	MKL1	ENST00000355630	p.L39V	c.115C>G	Verified	GAG	CTG	GTC	1	C	G
TCGA-61-1737	MLL	NM_005933.1	p.L1043V	c.3127C>G	Unverified	agt	ctt	aaa	1	C	G
TCGA-61-1737	MLL_ENST0	ENST00000534358	p.L1043V	c.3127C>G	Unverified	AGT	CTT	AAA	1	C	G
TCGA-13-1497	MLL3	ENST00000262189	p.Q3499E	c.10495C>G	Verified	ACC	CAA	ACT	1	C	G
TCGA-23-1110	MLL3	ENST00000262189	p.P2101A	c.6301C>G	Verified	ACC	CCA	CAT	1	C	G

TCGA-13-0903	MME	ENST00000360490	p.V199V	c.597C>G	Verified	AAA	GTC	CTT	3	C	G
TCGA-25-1313	MORC3	ENST00000400485	p.Q533E	c.1597C>G	Verified	CCT	CAG	TCT	1	C	G
TCGA-25-1313	MOSPD3	ENST00000393950	p.T235T	c.705C>G	Verified	CGG	ACC	TGA	3	C	G
TCGA-24-1616	MPEG1	ENST00000361050	p.A499G	c.1496C>G	Verified	CCA	GCC	GGC	2	C	G
TCGA-29-1764	MPG	ENST00000219431	p.L209L	c.627C>G	Unverified	GTC	CTC	AAG	3	C	G
TCGA-24-0975	MPP2	ENST00000269095	p.Y464*	c.1392C>G	Unverified	CCT	TAC	GTG	3	C	G
TCGA-23-1032	MPP3	ENST00000398389	p.D7E	c.21C>G	Unverified	GAG	GAC	TCT	3	C	G
TCGA-04-1347	MPP4_ENST	ENST00000409474	p.P136A	c.406C>G	Verified	GAA	CCC	CTT	1	C	G
TCGA-24-1435	MPZ	ENST00000360451	p.A206G	c.617C>G	Verified	AGT	GCT	ATG	2	C	G
TCGA-13-0885	MPZL1	ENST00000359523	p.A188G	c.563C>G	Verified	CTG	GCT	GTC	2	C	G
TCGA-61-1913	MRPL10	ENST00000351111	p.P56R	c.167C>G	Unverified	CCC	CCG	AAA	2	C	G
TCGA-30-1891	MRPL39	ENST00000307301	p.R295G	c.883C>G	Unverified	ATA	CGA	AGA	1	C	G
TCGA-13-1488	MRPL42	ENST00000393126	p.H88D	c.262C>G	Verified	ACA	CAT	GAT	1	C	G
TCGA-29-1775	MRPS23	ENST00000313608	p.A124G	c.371C>G	Unverified	AAG	GCT	TTA	2	C	G
TCGA-13-0905	MRRF	ENST00000344641	p.N217K	c.651C>G	Verified	ATG	AAC	AAG	3	C	G
TCGA-24-1849	MSH4	ENST00000263187	p.Q377E	c.1129C>G	Unverified	ctt	caa	gat	1	C	G
TCGA-24-1464	MSH6	ENST00000234420	p.Y1044*	c.3132C>G	Verified	AAT	TAC	AAG	3	C	G
TCGA-09-2056	MT4	ENST00000219162	p.S55*	c.164C>G	Verified	GGC	TCA	GAC	2	C	G
TCGA-20-1683	MTA2	ENST00000278823	p.P384R	c.1151C>G	Unverified	CCA	CCT	AAC	2	C	G
TCGA-09-2051	MTMR9	ENST00000221086	p.R379R	c.1137C>G	Unverified	cag	cgc	tgt	3	C	G
TCGA-29-1691	MTOR	ENST00000361445	p.A754G	c.2261C>G	Unverified	agt	gcc	cgc	2	C	G
TCGA-61-1740	MTRR	ENST00000264668	p.P578A	c.1732C>G	Unverified	GCC	CCG	TTT	1	C	G
TCGA-23-1031	MUC16	ENST00000397910	p.A4167G	c.12500C>G	Verified	CAT	GCT	ACA	2	C	G
TCGA-24-2035	MUC17	ENST00000306151	p.P1849A	c.5545C>G	Verified	TCT	CCT	ACA	1	C	G
TCGA-13-0762	MUC17	ENST00000306151	p.T1449S	c.4346C>G	Unverified	TCA	ACT	CCT	2	C	G
TCGA-13-0762	MUC17	ENST00000306151	p.T1449S	c.4346C>G	Unverified	TCA	ACT	CCT	2	C	G
TCGA-61-1733	MUC17	ENST00000306151	p.T3065T	c.9195C>G	Unverified	AGC	ACC	CTT	3	C	G
TCGA-61-1733	MUC17	ENST00000306151	p.T3065T	c.9195C>G	Unverified	AGC	ACC	CTT	3	C	G
TCGA-23-1031	MUC4_ENST	ENST00000405167	p.S733S	c.2199C>G	Verified	CTT	TCC	AAA	3	C	G
TCGA-61-1910	MUC5AC	ENST00000349637	p.A1898A	c.5694C>G	Unverified	ACG	GCC	ACG	3	C	G
TCGA-61-1910	MUC5B	ENST00000529681	p.A1895A	c.5685C>G	Unverified	ACG	GCC	ACG	3	C	G
TCGA-04-1337	MUS81	ENST00000308110	p.Y497*	c.1491C>G	Unverified	CGA	TAC	AGC	3	C	G
TCGA-23-1110	MXRA5	ENST00000381114	p.P877A	c.2629C>G	Verified	GAA	CCT	GAA	1	C	G
TCGA-24-2289	MXRA5	ENST00000381114	p.I60M	c.180C>G	Unverified	AGA	ATC	AAT	3	C	G

TCGA-23-1122	MXRA5_ENS	ENST00000381114	p.L2531V	c.7591C>G	Unverified	ATC	CTG	CAG	1	C	G
TCGA-13-0885	MYCBP2	NM_015057.1	p.L934V	c.2800C>G	Verified	cag	cta	gga	1	C	G
TCGA-30-1891	MYH13	ENST00000252172	p.L954V	c.2860C>G	Unverified	TCT	CTC	AAG	1	C	G
TCGA-30-1891	MYH13_ENS	ENST00000570743	p.L954V	c.2860C>G	Unverified	TCT	CTC	AAG	1	C	G
TCGA-13-0791	MYH4	ENST00000255381	p.Q1708E	c.5122C>G	Verified	GAG	CAA	GAG	1	C	G
TCGA-09-2056	MYLK	NM_053025	p.P62A	c.184C>G	Verified	gag	ccc	cag	1	C	G
TCGA-04-1338	MYO18A_EN	ENST00000527372	p.D1722E	c.5166C>G	Verified	GAT	GAC	ATC	3	C	G
TCGA-23-1122	MYO18B	ENST00000335473	p.L1840V	c.5518C>G	Verified	CAG	CTG	GAG	1	C	G
TCGA-13-0883	MYO1G	ENST00000258787	p.L78L	c.234C>G	Unverified	CAT	CTC	TAT	3	C	G
TCGA-24-2024	MYO3B	NM_138995	p.H1049D	c.3145C>G	Verified	tac	cat	gtt	1	C	G
TCGA-13-0714	MYO3B	NM_138995	p.A70G	c.209C>G	Verified	gag	gca	gaa	2	C	G
TCGA-13-0903	MYO5A	ENST00000399231	p.A109G	c.326C>G	Verified	GTA	GCT	ATA	2	C	G
TCGA-23-1122	MYO6	ENST00000369977	p.L1280V	c.3838C>G	Unverified	ATG	CTG	CAG	1	C	G
TCGA-23-1114	MYOC	ENST00000037502	p.N469K	c.1407C>G	Unverified	aag	aac	cgc	3	C	G
TCGA-24-1850	MYOCD	ENST00000343344	p.T621S	c.1862C>G	Unverified	CAA	ACC	AAT	2	C	G
TCGA-24-1850	MYOCD_ENS	ENST00000425538	p.T621S	c.1862C>G	Unverified	CAA	ACC	AAT	2	C	G
TCGA-10-0930	MYOM1	ENST00000356443	p.P1521A	c.4561C>G	Verified	GAG	CCC	ACC	1	C	G
TCGA-10-0930	MYOM1	ENST00000356443	p.P1521A	c.4561C>G	Verified	GAG	CCC	ACC	1	C	G
TCGA-24-1470	MYST1	ENST00000219797	p.R86G	c.256C>G	Verified	GGC	CGA	GAG	1	C	G
TCGA-13-1481	NACA	ENST00000356769	p.I128M	c.384C>G	Verified	AAG	ATC	GAA	3	C	G
TCGA-09-2056	NAF1	ENST00000274054	p.S216C	c.647C>G	Verified	GAA	TCT	ATG	2	C	G
TCGA-10-0930	NAF1	ENST00000274054	p.F373L	c.1119C>G	Verified	GAA	TTC	ACA	3	C	G
TCGA-10-0930	NAF1	ENST00000274054	p.F373L	c.1119C>G	Verified	GAA	TTC	ACA	3	C	G
TCGA-23-1111	NALCN	ENST00000251127	p.T1415R	c.4244C>G	Unverified	GCA	ACA	GAC	2	C	G
TCGA-13-1512	NAPRT1	ENST00000340490	p.A444A	c.1332C>G	Verified	CCA	GCC	CAG	3	C	G
TCGA-09-2056	NAV3	NM_014903.3	p.S1168R	c.3504C>G	Verified	agc	agc	aag	3	C	G
TCGA-20-1683	NBAS	ENST00000281513	p.R2106G	c.6316C>G	Unverified	CCC	CGC	ATT	1	C	G
TCGA-20-1683	NBAS	ENST00000281513	p.L1467V	c.4399C>G	Unverified	GAT	CTA	GAG	1	C	G
TCGA-20-1687	NBAS	ENST00000281513	p.P71A	c.211C>G	Unverified	AGC	CCG	GCA	1	C	G
TCGA-25-2400	NBEAL2	ENST00000292309	p.P474R	c.1421C>G	Unverified	GGT	CCA	GCT	2	C	G
TCGA-61-1907	NCAN	ENST00000252575	p.A582G	c.1745C>G	Unverified	AGG	GCC	CCT	2	C	G
TCGA-61-1907	NCAN_ENST	ENST00000252575	p.A582G	c.1745C>G	Unverified	AGG	GCC	CCT	2	C	G
TCGA-24-1849	NCEH1	ENST00000273512	p.P18R	c.53C>G	Unverified	TCG	CCC	TTT	2	C	G
TCGA-24-1849	NCEH1_ENS	ENST00000538775	p.P18R	c.53C>G	Unverified	TCG	CCC	TTT	2	C	G

TCGA-23-1111	NCF2	ENST00000367536	p.L392V	c.1174C>G	Unverified	AAG	CTG	AGC	1	C	G
TCGA-10-0930	NCKAP1L	ENST00000293373	p.S1016C	c.3047C>G	Verified	CCT	TCT	TCC	2	C	G
TCGA-10-0930	NCKAP1L	ENST00000293373	p.S1016C	c.3047C>G	Verified	CCT	TCT	TCC	2	C	G
TCGA-13-0890	NCOA3	ENST00000371998	p.P1198R	c.3593C>G	Verified	aac	cct	act	2	C	G
TCGA-09-2051	NDST1	ENST00000261797	p.A264A	c.792C>G	Unverified	CAC	GCC	ACT	3	C	G
TCGA-61-1740	NDUFV1	ENST00000322776	p.H136Q	c.408C>G	Unverified	CCT	CAC	AAG	3	C	G
TCGA-25-2392	NEK10	SU_NEK10	p.F50L	c.150C>G	Verified	aac	ttc	gat	3	C	G
TCGA-24-1423	NES	ENST00000368223	p.P906A	c.2716C>G	Verified	TCT	CCA	GAG	1	C	G
TCGA-29-1691	NFE2L3	ENST00000056233	p.H459D	c.1375C>G	Unverified	CAT	CAT	GAC	1	C	G
TCGA-29-1703	NFU1	ENST00000410022	p.I217M	c.651C>G	Unverified	TCA	ATC	ATT	3	C	G
TCGA-20-1687	NHLRC2	ENST00000369301	p.G374G	c.1122C>G	Unverified	TCT	GGC	AAA	3	C	G
TCGA-25-2391	NID2	ENST00000216286	p.A1169G	c.3506C>G	Unverified	CGT	GCT	GGT	2	C	G
TCGA-13-0905	NISCH	ENST00000345716	p.L1345L	c.4035C>G	Verified	GCC	CTC	AGC	3	C	G
TCGA-24-2262	NLRC3	ENST00000301749	p.L962V	c.2884C>G	Verified	CAG	CTC	AAC	1	C	G
TCGA-04-1542	NLRP3	ENST00000391828	p.R327G	c.979C>G	Verified	gag	cgg	gga	1	C	G
TCGA-24-1470	NLRP3	ENST00000391828	p.I59M	c.177C>G	Verified	atg	atc	gac	3	C	G
TCGA-13-0762	NMBR	ENST00000258042	p.L49L	c.147C>G	Unverified	TCC	CTC	TAC	3	C	G
TCGA-13-0762	NMBR	ENST00000258042	p.L49L	c.147C>G	Unverified	TCC	CTC	TAC	3	C	G
TCGA-23-1021	NMT2	ENST00000378165	p.I348M	c.1044C>G	Verified	GAT	ATC	AAA	3	C	G
TCGA-23-1022	NNMT	ENST00000299964	p.S29C	c.86C>G	Verified	GGT	TCT	AGG	2	C	G
TCGA-25-1318	NOS2	ENST00000313735	p.Y451*	c.1353C>G	Verified	GAA	TAC	CGG	3	C	G
TCGA-25-1318	NOS2	ENST00000313735	p.Y451*	c.1353C>G	Verified	GAA	TAC	CGG	3	C	G
TCGA-23-1116	NOS3	ENST00000297494	p.P970P	c.2910C>G	Unverified	GGC	CCC	CTG	3	C	G
TCGA-13-0761	NOTCH1	ENST00000277541	p.P2417A	c.7249C>G	Unverified	CAG	CCG	CAC	1	C	G
TCGA-13-0761	NOTCH1	ENST00000277541	p.P2417A	c.7249C>G	Unverified	CAG	CCG	CAC	1	C	G
TCGA-13-0761	NOTCH1_EN	ENST00000277541	p.P2417A	c.7249C>G	Unverified	CAG	CCG	CAC	1	C	G
TCGA-13-0761	NOTCH1_EN	ENST00000277541	p.P2417A	c.7249C>G	Unverified	CAG	CCG	CAC	1	C	G
TCGA-23-1124	NOV	ENST00000259526	p.P175A	c.523C>G	Verified	ggc	cca	gat	1	C	G
TCGA-24-2035	NPAT	ENST00000278612	p.Q410E	c.1228C>G	Verified	AGA	CAA	GAA	1	C	G
TCGA-61-2102	NPLOC4	ENST00000331134	p.S80W	c.239C>G	Verified	CCC	TCG	AGC	2	C	G
TCGA-61-1915	NPR2	ENST00000342694	p.A530G	c.1589C>G	Unverified	ACA	GCC	CAT	2	C	G
TCGA-61-1915	NPR2_ENST(	ENST00000342694	p.A530G	c.1589C>G	Unverified	ACA	GCC	CAT	2	C	G
TCGA-09-2044	NPRL3	ENST00000399953	p.S353C	c.1058C>G	Unverified	TTC	TCC	CAC	2	C	G
TCGA-61-1914	NRIP1	ENST00000400199	p.L1029V	c.3085C>G	Unverified	ggg	ctt	ttg	1	C	G

TCGA-24-1422	NSD1	ENST00000439151	p.P2420R	c.7259C>G	Verified	CCA	CCT	CCT	2	C	G
TCGA-61-2113	NT5DC3	NM_016575.1	p.R444G	c.1330C>G	Verified	ccc	cgg	agg	1	C	G
TCGA-13-1481	NTNG1	ENST00000370074	p.Y286*	c.858C>G	Verified	TTT	TAC	GCG	3	C	G
TCGA-13-1497	NUAK1	ENST00000261402	p.V556V	c.1668C>G	Verified	GGT	GTC	CCT	3	C	G
TCGA-25-1313	NUAK2	ENST00000367157	p.L374L	c.1122C>G	Verified	TCG	CTC	AAG	3	C	G
TCGA-61-1998	NUP153	ENST00000262077	p.T695S	c.2084C>G	Verified	CAG	ACT	GGA	2	C	G
TCGA-23-1114	NUP155	ENST00000231498	p.L1123V	c.3367C>G	Unverified	ATT	CTT	AGT	1	C	G
TCGA-09-2056	NUP205	ENST00000285968	p.A348G	c.1043C>G	Verified	ACA	GCT	CTG	2	C	G
TCGA-61-1915	NUP210	ENST00000254508	p.A614G	c.1841C>G	Unverified	GAG	GCC	CAG	2	C	G
TCGA-29-1693	NUP62	ENST00000352066	p.L458V	c.1372C>G	Unverified	CAC	CTG	AAC	1	C	G
TCGA-23-1124	NWD1	ENST00000339803	p.T106S	c.317C>G	Verified	AAG	ACC	CAC	2	C	G
TCGA-61-1733	OAS2	ENST00000342315	p.V452V	c.1356C>G	Unverified	GAA	GTC	AGC	3	C	G
TCGA-61-1733	OAS2	ENST00000342315	p.V452V	c.1356C>G	Unverified	GAA	GTC	AGC	3	C	G
TCGA-29-1691	OAS3	ENST00000228928	p.L215V	c.643C>G	Unverified	TGC	CTA	CAG	1	C	G
TCGA-13-0890	OBSCN	SU_OBSCN.1	p.Q1192E	c.3574C>G	Verified	gcc	cag	ccc	1	C	G
TCGA-13-1507	OBSCN	SU_OBSCN.1	p.S1518R	c.4554C>G	Verified	ggg	agc	cag	3	C	G
TCGA-24-1846	OBSCN	SU_OBSCN.1	p.C3181W	c.9543C>G	Unverified	gtg	tgc	tgg	3	C	G
TCGA-29-1777	OBSCN	SU_OBSCN.1	p.Y3900*	c.11700C>G	Unverified	aga	tac	agc	3	C	G
TCGA-24-1846	OBSCN_ENS	ENST00000284548	p.C3127W	c.9381C>G	Unverified	GTG	TGC	TGG	3	C	G
TCGA-29-1777	OBSCN_ENS	ENST00000284548	p.Y3846*	c.11538C>G	Unverified	AGA	TAC	AGC	3	C	G
TCGA-13-0890	OBSCN_ENS	ENST00000359599	p.Q1192E	c.3574C>G	Verified	GCC	CAG	CCC	1	C	G
TCGA-13-1507	OBSCN_ENS	ENST00000359599	p.S1518R	c.4554C>G	Verified	GGG	AGC	CAG	3	C	G
TCGA-24-1846	OBSCN_ENS	ENST00000359599	p.C3410W	c.10230C>G	Unverified	GTG	TGC	TGG	3	C	G
TCGA-29-1777	OBSCN_ENS	ENST00000359599	p.Y4129*	c.12387C>G	Unverified	AGA	TAC	AGC	3	C	G
TCGA-24-1846	OBSCN_ENS	ENST00000570156	p.C3311W	c.9933C>G	Unverified	GTG	TGC	TGG	3	C	G
TCGA-29-1777	OBSCN_ENS	ENST00000570156	p.Y4030*	c.12090C>G	Unverified	AGA	TAC	AGC	3	C	G
TCGA-25-2398	OCA2	ENST00000354638	p.T482S	c.1445C>G	Verified	GCC	ACT	GCC	2	C	G
TCGA-29-2427	ODAM	ENST00000396094	p.Q137E	c.409C>G	Verified	CCT	CAA	GAG	1	C	G
TCGA-24-0979	ODZ2	ENST00000388903	p.N2124K	c.6372C>G	Verified	CAG	TAC	TTC	3	C	G
TCGA-61-1740	ODZ4	ENST00000278550	p.A2096G	c.6287C>G	Unverified	CAG	GCT	GTG	2	C	G
TCGA-61-1740	ODZ4_ENST	ENST00000278550	p.A2096G	c.6287C>G	Unverified	CAG	GCT	GTG	2	C	G
TCGA-29-1777	OLFM1	ENST00000252854	p.D94E	c.282C>G	Unverified	TTG	GAC	AGG	3	C	G
TCGA-13-1510	OLFM2	ENST00000264833	p.T337T	c.1011C>G	Unverified	TAC	ACC	ACC	3	C	G
TCGA-61-1907	OLFM3	ENST00000370103	p.Q152E	c.454C>G	Unverified	ACC	CAG	TTC	1	C	G

TCGA-29-1763	OLFM3	ENST00000370103	p.S5S	c.15C>G	Unverified	ACG	TCC	AAC	3	C	G
TCGA-61-1907	OLFM3_ENS	ENST00000338858	p.Q172E	c.514C>G	Unverified	ACC	CAG	TTC	1	C	G
TCGA-24-2280	OMD	ENST00000375550	p.Q374E	c.1120C>G	Unverified	ATA	CAA	CTA	1	C	G
TCGA-30-1718	OPN3	ENST00000366554	p.S294W	c.881C>G	Unverified	GTT	TCG	TAC	2	C	G
TCGA-09-2051	OPRD1	ENST00000234961	p.S344R	c.1032C>G	Unverified	AGC	AGC	TTC	3	C	G
TCGA-61-2009	OR1L8	ENST00000304865	p.L56V	c.166C>G	Verified	CAT	CTT	CAG	1	C	G
TCGA-24-1846	OR2Y1	ENST00000307832	p.R90R	c.270C>G	Unverified	GAC	CGC	ACC	3	C	G
TCGA-29-1690	OR2Z1	ENST00000324060	p.L66L	c.198C>G	Unverified	CAG	CTC	TCC	3	C	G
TCGA-29-1763	OR3A2_ENS	ENST00000408891	p.P191A	c.571C>G	Unverified	CTC	CCA	CAG	1	C	G
TCGA-24-1469	OR3A4	ENST00000323164	p.L169V	c.505C>G	Verified	CCT	CTT	AAC	1	C	G
TCGA-30-1857	OR4D1	ENST00000268912	p.C308W	c.924C>G	Unverified	ATT	TGC	AGG	3	C	G
TCGA-04-1361	OR4E2	ENST00000408935	p.L301L	c.903C>G	Verified	CAG	CTC	AGG	3	C	G
TCGA-04-1361	OR4E2	ENST00000408935	p.L301L	c.903C>G	Verified	CAG	CTC	AGG	3	C	G
TCGA-24-1422	OR52A4	ENST00000380369	p.C101W	c.303C>G	Verified	GCT	TGC	CTC	3	C	G
TCGA-23-1124	OR52H1	ENST00000322653	p.S72S	c.216C>G	Verified	CTC	TCC	ATG	3	C	G
TCGA-24-1474	OR5AP2	ENST00000302981	p.I180M	c.540C>G	Verified	AGG	ATC	AAC	3	C	G
TCGA-24-1103	OR5B21	ENST00000360374	p.L183V	c.547C>G	Verified	CTC	CTG	GCT	1	C	G
TCGA-29-1775	OR5L2	ENST00000378397	p.L288V	c.862C>G	Unverified	CCC	CTG	ATC	1	C	G
TCGA-23-1117	OR5M1_ENS	ENST00000526538	p.V194V	c.582C>G	Verified	CGT	GTC	AAA	3	C	G
TCGA-23-1117	OR5M1_ENS	ENST00000526538	p.V194V	c.582C>G	Verified	CGT	GTC	AAA	3	C	G
TCGA-13-1501	OR6S1	ENST00000320704	p.N67K	c.201C>G	Verified	GGT	AAC	CTG	3	C	G
TCGA-13-1501	OR6S1	ENST00000320704	p.N67K	c.201C>G	Verified	GGT	AAC	CTG	3	C	G
TCGA-23-1022	OR7D2	ENST00000344248	p.L110V	c.328C>G	Verified	ATT	CTG	GAC	1	C	G
TCGA-25-1326	OR8D1	ENST00000357821	p.H193Q	c.579C>G	Verified	ACA	CAC	CTC	3	C	G
TCGA-23-1029	OR8D4	ENST00000321355	p.H229Q	c.687C>G	Unverified	ATC	CAC	TCT	3	C	G
TCGA-04-1338	OR9G4	ENST00000302957	p.D286E	c.858C>G	Verified	AGG	GAC	AAA	3	C	G
TCGA-61-2009	ORC1	ENST00000371568	p.I317M	c.951C>G	Verified	ata	atc	ctg	3	C	G
TCGA-13-1488	OSCP1	ENST00000356637	p.I362M	c.1086C>G	Verified	CGA	ATC	ATG	3	C	G
TCGA-13-0714	OSMR	ENST00000274276	p.T88S	c.263C>G	Verified	ACC	ACT	GTG	2	C	G
TCGA-13-0890	OVGP1	ENST00000369732	p.L24V	c.70C>G	Verified	AAA	CTC	GTG	1	C	G
TCGA-29-1693	OVOL2	ENST00000278780	p.T110R	c.329C>G	Unverified	ACC	ACA	GGC	2	C	G
TCGA-13-0792	P2RY10	ENST00000544091	p.T18S	c.53C>G	Verified	agt	acc	agc	2	C	G
TCGA-61-1907	PABPC4	ENST00000372857	p.A477G	c.1430C>G	Unverified	TTG	GCT	ATG	2	C	G
TCGA-04-1542	PACS1	ENST00000320580	p.R709G	c.2125C>G	Verified	GGG	CGG	GTG	1	C	G

TCGA-13-0913	PACS1	ENST00000320580	p.S59C	c.176C>G	Verified	TCG	TCC	ACC	2	C	G
TCGA-04-1347	PAK7	ENST00000378429	p.Q423E	c.1267C>G	Verified	cag	cag	ccc	1	C	G
TCGA-24-1435	PAMR1	ENST00000278360	p.H149Q	c.447C>G	Verified	GCT	CAC	TGT	3	C	G
TCGA-04-1331	PAPD7	ENST00000230859	p.S271C	c.812C>G	Verified	AGG	TCC	TAT	2	C	G
TCGA-04-1356	PAPOLG	ENST00000238714	p.L167V	c.499C>G	Verified	GAT	CTA	GTC	1	C	G
TCGA-25-1313	PAPPA	ENST00000328252	p.L1543V	c.4627C>G	Verified	GGT	CTC	AAG	1	C	G
TCGA-04-1342	PAPPA	ENST00000328252	p.T1015R	c.3044C>G	Verified	TAC	ACG	CCC	2	C	G
TCGA-29-1784	PAPPA2	ENST00000367662	p.S256C	c.767C>G	Unverified	AAC	TCC	CAA	2	C	G
TCGA-29-1784	PAPPA2_EN	ENST00000367661	p.S256C	c.767C>G	Unverified	AAC	TCC	CAA	2	C	G
TCGA-24-2280	PAPSS2	ENST00000361175	p.T425S	c.1274C>G	Unverified	GAC	ACT	CGC	2	C	G
TCGA-23-2077	PAQR5	ENST00000395407	p.I304M	c.912C>G	Verified	ATC	ATC	TTC	3	C	G
TCGA-13-0923	PARD3B	ENST00000358768	p.H1080Q	c.3240C>G	Verified	CAG	CAC	TAC	3	C	G
TCGA-13-0885	PARM1	ENST00000307428	p.P104A	c.310C>G	Verified	GAC	CCC	TCA	1	C	G
TCGA-29-1777	PARP10	ENST00000313028	p.P312A	c.934C>G	Unverified	GGT	CCC	ATG	1	C	G
TCGA-61-1740	PARP4	ENST00000381989	p.T951R	c.2852C>G	Unverified	AAC	ACA	GAC	2	C	G
TCGA-29-1705	PASK	ENST00000234040	p.L351L	c.1053C>G	Unverified	ACC	CTC	CTG	3	C	G
TCGA-29-1705	PASK_ENST(	ENST00000234040	p.L351L	c.1053C>G	Unverified	ACC	CTC	CTG	3	C	G
TCGA-23-1118	PATL1	ENST00000300146	p.P323P	c.969C>G	Verified	CCA	CCC	TCC	3	C	G
TCGA-23-1022	PAX4	ENST00000341640	p.L223L	c.669C>G	Verified	AAG	CTC	AAG	3	C	G
TCGA-61-1740	PBRM1	ENST00000337303	p.Y963*	c.2889C>G	Unverified	GAT	TAC	GTC	3	C	G
TCGA-61-1740	PBRM1_ENS	ENST00000296302	p.Y963*	c.2889C>G	Unverified	GAT	TAC	GTC	3	C	G
TCGA-61-1740	PBRM1_ENS	ENST00000356770	p.Y931*	c.2793C>G	Unverified	GAT	TAC	GTC	3	C	G
TCGA-61-2113	PCDHA10	ENST00000506939	p.N450K	c.1350C>G	Unverified	GAC	AAC	GCG	3	C	G
TCGA-61-2113	PCDHA10_E	ENST00000505235	p.N450K	c.1350C>G	Unverified	GAC	AAC	GCG	3	C	G
TCGA-24-1463	PCDHB12	ENST00000239450	p.F17L	c.51C>G	Unverified	CTT	TTC	TTT	3	C	G
TCGA-04-1342	PCDHB14	ENST00000239449	p.H512Q	c.1536C>G	Unverified	GGC	CAC	CTG	3	C	G
TCGA-30-1718	PCDHB8	ENST00000239444	p.G584G	c.1752C>G	Unverified	CCG	GGC	TAC	3	C	G
TCGA-61-1725	PCDHGA3_E	ENST00000253812	p.Q98E	c.292C>G	Unverified	GCT	CAG	ATC	1	C	G
TCGA-61-1915	PCGF3	ENST00000362003	p.L24V	c.70C>G	Unverified	tac	ctc	atc	1	C	G
TCGA-13-1512	PCSK2	ENST00000262545	p.N362K	c.1086C>G	Verified	AGG	AAC	CCC	3	C	G
TCGA-24-1422	PDCD2L	ENST00000246535	p.R161G	c.481C>G	Verified	GCT	CGG	CTC	1	C	G
TCGA-09-1674	PDE1C	ENST00000396184	p.R330G	c.988C>G	Unverified	TTT	CGA	ACC	1	C	G
TCGA-09-1674	PDE1C_ENST	ENST00000396191	p.R330G	c.988C>G	Unverified	TTT	CGA	ACC	1	C	G
TCGA-09-1674	PDE1C_ENST	ENST00000396193	p.R390G	c.1168C>G	Unverified	TTT	CGA	ACC	1	C	G

TCGA-61-1740	PDE4A	ENST00000344979	p.Q72E	c.214C>G	Unverified	AAA	CAG	CAA	1	C	G
TCGA-61-1740	PDE4A_ENST	ENST00000293683	p.Q285E	c.853C>G	Unverified	AAA	CAG	CAA	1	C	G
TCGA-61-1740	PDE4A_ENST	ENST00000352831	p.Q311E	c.931C>G	Unverified	AAA	CAG	CAA	1	C	G
TCGA-61-1740	PDE4A_ENST	ENST00000440014	p.Q250E	c.748C>G	Unverified	AAA	CAG	CAA	1	C	G
TCGA-24-2289	PDE6A	ENST00000255266	p.D366E	c.1098C>G	Unverified	GAG	GAC	TTT	3	C	G
TCGA-24-1469	PDGFRB	ENST00000261799	p.R849G	c.2545C>G	Verified	gct	cga	gac	1	C	G
TCGA-04-1542	PDIA4	ENST00000286091	p.Q575E	c.1723C>G	Verified	GGC	CAA	AAG	1	C	G
TCGA-61-2109	PDLIM7	ENST00000355841	p.T424S	c.1271C>G	Unverified	GAC	ACC	TGC	2	C	G
TCGA-30-1714	PDS5B	ENST00000315596	p.L1297V	c.3889C>G	Unverified	CCT	CTT	GGT	1	C	G
TCGA-61-2113	PDXK	ENST00000291565	p.P286R	c.857C>G	Unverified	agc	ccc	atg	2	C	G
TCGA-13-1501	PER1	ENST00000317276	p.P403A	c.1207C>G	Unverified	CGA	CCC	CTC	1	C	G
TCGA-13-1501	PER1	ENST00000317276	p.P403A	c.1207C>G	Unverified	CGA	CCC	CTC	1	C	G
TCGA-23-1022	PEX3	ENST00000367591	p.T177R	c.530C>G	Verified	CTG	ACA	GAA	2	C	G
TCGA-29-1702	PFDN5	ENST00000551018	p.T95R	c.284C>G	Unverified	aag	aca	gct	2	C	G
TCGA-24-1845	PFDN6_ENST	ENST00000463584	p.L89V	c.265C>G	Unverified	TTT	CTG	AGG	1	C	G
TCGA-24-0975	PFKM	ENST00000359794	p.V295V	c.885C>G	Verified	act	gtc	ttg	3	C	G
TCGA-13-0923	PHB2	ENST00000535923	p.Q258E	c.772C>G	Verified	GCC	CAG	AAT	1	C	G
TCGA-13-1507	PHEX	ENST00000379374	p.Q383E	c.1147C>G	Verified	TTT	CAG	TAT	1	C	G
TCGA-09-2056	PI4KB	ENST00000368875	p.P540A	c.1618C>G	Verified	GAT	CCT	TCT	1	C	G
TCGA-23-1022	PIK3C2A	ENST00000265970	p.H400Q	c.1200C>G	Verified	AAT	CAC	CGC	3	C	G
TCGA-23-1117	PIKFYVE	ENST00000264380	p.L1530V	c.4588C>G	Verified	AGA	CTG	AGA	1	C	G
TCGA-23-1117	PIKFYVE	ENST00000264380	p.L1530V	c.4588C>G	Verified	AGA	CTG	AGA	1	C	G
TCGA-13-0920	PIP5K1A	ENST00000349792	p.P196A	c.586C>G	Verified	ctt	cca	gga	1	C	G
TCGA-29-1785	PIWIL1	ENST00000245255	p.R439G	c.1315C>G	Unverified	CTT	CGA	GAC	1	C	G
TCGA-29-1785	PIWIL1	ENST00000245255	p.R439G	c.1315C>G	Unverified	CTT	CGA	GAC	1	C	G
TCGA-25-1326	PIWIL3	ENST00000332271	p.L859V	c.2575C>G	Verified	AAG	CTG	GCT	1	C	G
TCGA-24-1469	PIWIL3	ENST00000332271	p.A33G	c.98C>G	Verified	TCA	GCT	ACA	2	C	G
TCGA-13-1509	PKD1L1	ENST00000289672	p.A1968G	c.5903C>G	Verified	TAC	GCG	TGT	2	C	G
TCGA-13-0924	PKHD1	ENST00000371117	p.P875A	c.2623C>G	Verified	AAT	CCT	GCT	1	C	G
TCGA-29-1785	PLA2G15	ENST00000219345	p.R9G	c.25C>G	Unverified	TAC	CGT	GTG	1	C	G
TCGA-29-1785	PLA2G15	ENST00000219345	p.R9G	c.25C>G	Unverified	TAC	CGT	GTG	1	C	G
TCGA-29-1699	PLA2G3	ENST00000215885	p.N40K	c.120C>G	Unverified	GGC	AAC	CCA	3	C	G
TCGA-29-1699	PLA2G3_EN!	ENST00000215885	p.N40K	c.120C>G	Unverified	GGC	AAC	CCA	3	C	G
TCGA-24-1469	PLA2G4A	ENST00000367466	p.H442Q	c.1326C>G	Unverified	TCA	CAC	GAA	3	C	G

TCGA-13-0923	PLAU	ENST00000372764	p.P216R	c.647C>G	Verified	AGC	CCT	TGC	2	C	G
TCGA-24-1844	PLCB1_ENST	ENST00000378641	p.T1152S	c.3455C>G	Unverified	GAA	ACT	TGC	2	C	G
TCGA-25-2393	PLCB2	ENST00000260402	p.H311Q	c.933C>G	Verified	CTC	CAC	CAC	3	C	G
TCGA-29-1766	PLCD3	NM_133373.2	p.P464P	c.1392C>G	Unverified	aat	ccc	gag	3	C	G
TCGA-29-1766	PLCD3_ENST	ENST00000322765	p.P464P	c.1392C>G	Unverified	AAT	CCC	GAG	3	C	G
TCGA-61-1740	PLD1	ENST00000351298	p.R700G	c.2098C>G	Unverified	CAG	CGC	TGG	1	C	G
TCGA-23-1117	PLEKHA5	ENST00000299275	p.Q1039E	c.3115C>G	Verified	CCT	CAA	GAT	1	C	G
TCGA-23-1117	PLEKHA5	ENST00000299275	p.Q1039E	c.3115C>G	Verified	CCT	CAA	GAT	1	C	G
TCGA-23-1117	PLEKHA5_EN	ENST00000429027	p.Q1200E	c.3598C>G	Verified	CCT	CAA	GAT	1	C	G
TCGA-23-1117	PLEKHA5_EN	ENST00000429027	p.Q1200E	c.3598C>G	Verified	CCT	CAA	GAT	1	C	G
TCGA-25-2392	PLEKHA6	ENST00000272203	p.F82L	c.246C>G	Verified	tgg	ttc	gtc	3	C	G
TCGA-24-1422	PLEKHB2	ENST00000234115	p.D74E	c.222C>G	Verified	AAA	GAC	TGC	3	C	G
TCGA-24-1849	PLEKHB2	ENST00000234115	p.N196K	c.588C>G	Unverified	GAC	AAC	GAC	3	C	G
TCGA-13-1505	PLEKHH3	ENST00000293349	p.P64A	c.190C>G	Verified	CAG	CCA	GTG	1	C	G
TCGA-13-0905	PLEKHM2	ENST00000420314	p.P871R	c.2612C>G	Verified	CCC	CCC	GAG	2	C	G
TCGA-61-1740	PLSCR1	ENST00000342435	p.P23A	c.67C>G	Unverified	CCT	CCT	CAG	1	C	G
TCGA-13-0906	PLXNB2	XM_371474.2	p.P1033R	c.3098C>G	Verified	gag	ccg	cta	2	C	G
TCGA-24-1422	PMPCB	ENST00000249269	p.L25V	c.73C>G	Verified	AGT	CTT	CTA	1	C	G
TCGA-23-2077	PNLIPRP3	ENST00000369230	p.A211G	c.632C>G	Unverified	GAT	GCC	AAC	2	C	G
TCGA-23-1809	PODXL	ENST00000378555	p.I74M	c.222C>G	Unverified	GAA	ATC	TTG	3	C	G
TCGA-23-1124	POGZ	ENST00000271715	p.L235V	c.703C>G	Verified	ACT	CTT	ACC	1	C	G
TCGA-13-0883	POLD1	ENST00000440232	p.P1102A	c.3304C>G	Unverified	CCC	CCT	GGA	1	C	G
TCGA-20-0990	POLD2	ENST00000406581	p.A51G	c.152C>G	Verified	TAT	GCC	CAC	2	C	G
TCGA-09-1665	POLM	ENST00000242248	p.L217V	c.649C>G	Unverified	ctg	ctg	gag	1	C	G
TCGA-29-1761	POLR2A	ENST00000322644	p.G1570G	c.4710C>G	Unverified	CCG	GGC	TCC	3	C	G
TCGA-09-2051	POLR2I	ENST00000221859	p.Y7*	c.21C>G	Unverified	ACT	TAC	GAG	3	C	G
TCGA-13-1501	POMGNT1	ENST00000371984	p.H358D	c.1072C>G	Verified	CAG	CAT	ACT	1	C	G
TCGA-13-1501	POMGNT1	ENST00000371984	p.H358D	c.1072C>G	Verified	CAG	CAT	ACT	1	C	G
TCGA-04-1338	PON3	ENST00000265627	p.L205V	c.613C>G	Verified	GTT	CTT	TTC	1	C	G
TCGA-29-1766	POTEF	ENST00000409914	p.Y245*	c.735C>G	Unverified	CAC	TAC	GCT	3	C	G
TCGA-29-1785	PPL	ENST00000345988	p.L1209L	c.3627C>G	Unverified	CAG	CTC	CGG	3	C	G
TCGA-29-1785	PPL	ENST00000345988	p.L1209L	c.3627C>G	Unverified	CAG	CTC	CGG	3	C	G
TCGA-61-2109	PPP1R3A	NM_002711.2	p.G433G	c.1299C>G	Verified	act	ggc	agc	3	C	G
TCGA-13-0906	PPP1R9A	XM_374491.1	p.F467L	c.1401C>G	Verified	gtt	ttc	aac	3	C	G

TCGA-23-1031	PPP3CA	ENST00000394854	p.A280A	c.840C>G	Verified	cga	gcc	cac	3	C	G
TCGA-61-1914	PPP6R3	ENST00000265636	p.T613R	c.1838C>G	Unverified	GAA	ACA	ACC	2	C	G
TCGA-61-1914	PPP6R3_ENS	ENST00000393801	p.T693R	c.2078C>G	Unverified	GAA	ACA	ACC	2	C	G
TCGA-61-1737	PPPDE1	ENST00000302550	p.A173G	c.518C>G	Unverified	GAT	GCT	GCC	2	C	G
TCGA-23-1124	PRAMEF12	ENST00000357726	p.P32R	c.95C>G	Verified	CTG	CCC	AGG	2	C	G
TCGA-13-0890	PRC1	ENST00000394249	p.P468R	c.1403C>G	Verified	GCT	CCT	CGA	2	C	G
TCGA-24-1844	PRDM15	ENST00000269844	p.P655A	c.1963C>G	Unverified	GAA	CCC	ACA	1	C	G
TCGA-61-2094	PRDM16	ENST00000270722	p.L737V	c.2209C>G	Unverified	TCC	CTT	TAC	1	C	G
TCGA-24-2289	PRDM9	ENST00000296682	p.H686Q	c.2058C>G	Unverified	ACA	CAC	ACA	3	C	G
TCGA-61-1906	PRICKLE2	ENST00000295902	p.P627A	c.1879C>G	Unverified	AAC	CCC	ATT	1	C	G
TCGA-61-1906	PRICKLE2	ENST00000295902	p.P627A	c.1879C>G	Unverified	AAC	CCC	ATT	1	C	G
TCGA-29-1775	PRICKLE2	ENST00000295902	p.A719G	c.2156C>G	Unverified	AGA	GCC	AGG	2	C	G
TCGA-25-2392	PRKAG3	ENST00000233944	p.L331V	c.991C>G	Verified	ttc	ctg	cac	1	C	G
TCGA-61-2102	PRKDC	NM_006904	p.H1133D	c.3397C>G	Verified	gat	cac	cta	1	C	G
TCGA-29-2427	PRMT8	ENST00000382622	p.I250M	c.750C>G	Verified	TGC	ATC	CGG	3	C	G
TCGA-25-2392	PROX1	ENST00000498508	p.T522R	c.1565C>G	Verified	acc	acg	agt	2	C	G
TCGA-23-1117	PRPF40A_EN	ENST00000359961	p.P86A	c.256C>G	Verified	ATG	CCG	CAG	1	C	G
TCGA-23-1117	PRPF40A_EN	ENST00000359961	p.P86A	c.256C>G	Verified	ATG	CCG	CAG	1	C	G
TCGA-13-1496	PRR21	ENST00000408934	p.G17G	c.51C>G	Verified	CAC	GGC	TTT	3	C	G
TCGA-61-1914	PRRC2A	ENST00000376033	p.H226D	c.676C>G	Unverified	CTT	CAT	CAT	1	C	G
TCGA-13-1481	PRRC2A	ENST00000376033	p.A407A	c.1221C>G	Unverified	CCT	GCC	CCA	3	C	G
TCGA-04-1367	PRSS21	ENST00000005995	p.P241R	c.722C>G	Verified	GGA	CCC	TTG	2	C	G
TCGA-23-1022	PSD4	ENST00000245796	p.H537Q	c.1611C>G	Verified	ATT	CAC	CTG	3	C	G
TCGA-61-2113	PSG1_ENST(	ENST00000449000	p.P158A	c.472C>G	Verified	AAC	CCC	AGG	1	C	G
TCGA-29-1761	PSMA3	ENST00000216455	p.G5G	c.15C>G	Unverified	ATC	GGC	ACT	3	C	G
TCGA-24-1422	PSMD13	ENST00000532097	p.Q164E	c.490C>G	Verified	TAT	CAA	ACA	1	C	G
TCGA-24-1422	PSMD13_EN	ENST00000431206	p.Q166E	c.496C>G	Verified	TAT	CAA	ACA	1	C	G
TCGA-24-1847	PSME4	NM_014614.1	p.P110A	c.328C>G	Unverified	cct	cca	gaa	1	C	G
TCGA-24-1847	PSME4_ENS	ENST00000404125	p.P224A	c.670C>G	Unverified	CCT	CCA	GAA	1	C	G
TCGA-04-1338	PSORS1C1	ENST00000259881	p.L14V	c.40C>G	Verified	GCT	CTC	GGC	1	C	G
TCGA-24-2288	PSORS1C2	ENST00000259845	p.P58P	c.174C>G	Unverified	GCA	CCC	CCT	3	C	G
TCGA-61-2113	PTAR1_ENST	ENST00000340434	p.T359R	c.1076C>G	Verified	CGG	ACG	CCA	2	C	G
TCGA-29-1703	PTCD2	ENST00000380639	p.R38G	c.112C>G	Unverified	TGC	CGC	TGC	1	C	G
TCGA-13-0760	PTPN13	ENST00000436978	p.I1175M	c.3525C>G	Verified	GTT	ATC	TCT	3	C	G

TCGA-24-1423	PTPN18	ENST00000175756	p.I74M	c.222C>G	Verified	gta	atc	ctc	3	C	G
TCGA-13-0904	PTPN21	ENST00000328736	p.A969A	c.2907C>G	Verified	ATT	GCC	ACA	3	C	G
TCGA-23-1124	PTPRF	ENST00000359947	p.N896K	c.2688C>G	Verified	AAG	AAC	CGG	3	C	G
TCGA-20-0990	PTPRG	ENST00000474889	p.S545R	c.1635C>G	Verified	GCC	AGC	AAG	3	C	G
TCGA-23-1111	PTPRG	ENST00000474889	p.S77R	c.231C>G	Unverified	GTC	AGC	TGT	3	C	G
TCGA-24-1850	PTPRK	ENST00000368226	p.P684R	c.2051C>G	Unverified	gcc	ccg	ttc	2	C	G
TCGA-24-1850	PTPRK_ENST	ENST00000368213	p.P684R	c.2051C>G	Unverified	GCC	CCG	TTC	2	C	G
TCGA-29-1769	PXDN	ENST00000252804	p.P348R	c.1043C>G	Unverified	CAG	CCA	CAG	2	C	G
TCGA-09-2049	PXDN	ENST00000252804	p.S250S	c.750C>G	Verified	ACC	TCC	GAG	3	C	G
TCGA-04-1347	PZP	ENST00000261336	p.I93M	c.279C>G	Verified	AGG	ATC	TCA	3	C	G
TCGA-13-1488	Q5I0X0_HUI	ENST00000322446	p.A155G	c.464C>G	Verified	GCA	GCT	CTT	2	C	G
TCGA-61-1914	Q8N8C9_HU	ENST00000316673	p.P29R	c.86C>G	Unverified	GCG	CCC	AAC	2	C	G
TCGA-25-2401	Q96K91_HU	ENST00000288841	p.Y549*	c.1647C>G	Unverified	CAG	TAC	AAG	3	C	G
TCGA-13-0906	RAB2B	ENST00000397762	p.T152R	c.455C>G	Verified	aaa	aca	gcc	2	C	G
TCGA-23-1122	RABEP1	ENST00000262477	p.A445G	c.1334C>G	Unverified	GGA	GCA	GAT	2	C	G
TCGA-25-2042	RABGAP1	NM_012197.2	p.L28L	c.84C>G	Verified	cag	ctc	tcc	3	C	G
TCGA-13-0887	RAGE	ENST00000361847	p.S364C	c.1091C>G	Verified	tac	tcc	agc	2	C	G
TCGA-61-2113	RAI1	ENST00000353383	p.L1418V	c.4252C>G	Unverified	AAA	CTG	TCT	1	C	G
TCGA-61-1737	RAPGEF6_EI	ENST00000509018	p.H1526Q	c.4578C>G	Unverified	ACA	CAC	CTA	3	C	G
TCGA-09-2044	RAPSN	ENST00000298854	p.D303E	c.909C>G	Verified	CTG	GAC	AAG	3	C	G
TCGA-61-1740	RASA1	ENST00000274376	p.L902V	c.2704C>G	Unverified	ttt	ctt	cga	1	C	G
TCGA-61-1740	RASA1_ENST	ENST00000456692	p.L725V	c.2173C>G	Unverified	TTT	CTT	CGA	1	C	G
TCGA-23-1021	RASA2	ENST00000286364	p.T136S	c.407C>G	Verified	GAA	ACT	TGG	2	C	G
TCGA-29-1691	RASGRF1	ENST00000419573	p.N972K	c.2916C>G	Unverified	acc	aac	gat	3	C	G
TCGA-04-1542	RASSF2	ENST00000379400	p.Q288E	c.862C>G	Verified	att	cag	aag	1	C	G
TCGA-13-1507	RBM33	ENST00000401878	p.S851*	c.2552C>G	Verified	CCA	TCA	CCC	2	C	G
TCGA-04-1542	RBM6	ENST00000266022	p.P933A	c.2797C>G	Verified	CAG	CCC	CAG	1	C	G
TCGA-13-1510	RBM6	ENST00000266022	p.G702G	c.2106C>G	Unverified	TAT	GGC	TTT	3	C	G
TCGA-23-1110	RDH13	ENST00000396247	p.Y216*	c.648C>G	Verified	AAG	TAC	TTC	3	C	G
TCGA-24-1422	RDH8	ENST00000171214	p.H27D	c.79C>G	Verified	GCC	CAT	GAC	1	C	G
TCGA-23-1122	REEP4	ENST00000306306	p.V207V	c.621C>G	Unverified	GCA	GTC	CCC	3	C	G
TCGA-24-2288	REG4	ENST00000354219	p.L67V	c.199C>G	Unverified	CAC	CTG	GCA	1	C	G
TCGA-13-1507	RELB	ENST00000221452	p.V372V	c.1116C>G	Verified	att	gtc	gag	3	C	G
TCGA-13-0904	RELN	ENST00000343529	p.D3348E	c.10044C>G	Unverified	AGT	GAC	CTG	3	C	G

TCGA-29-1711	RELN	ENST00000343529	p.D1216E	c.3648C>G	Unverified	GAT	GAC	ATC	3	C	G
TCGA-29-1711	RELN	ENST00000343529	p.D1216E	c.3648C>G	Unverified	GAT	GAC	ATC	3	C	G
TCGA-24-1423	RFC1	ENST00000349703	p.L209V	c.625C>G	Verified	cag	ctt	gat	1	C	G
TCGA-04-1356	RFX3	ENST00000358730	p.S178C	c.533C>G	Verified	AGC	TCT	CTT	2	C	G
TCGA-13-0920	RGS12	ENST00000344733	p.S506C	c.1517C>G	Unverified	gcc	tct	cct	2	C	G
TCGA-29-1703	RGS22	ENST00000360863	p.H1107Q	c.3321C>G	Unverified	GAA	CAC	CGG	3	C	G
TCGA-29-1703	RGS22_ENS	ENST00000360863	p.H1107Q	c.3321C>G	Unverified	GAA	CAC	CGG	3	C	G
TCGA-24-1104	RGS8	ENST00000258302	p.L119V	c.355C>G	Verified	AAA	CTG	GTC	1	C	G
TCGA-29-1785	RHOBTB3	ENST00000379982	p.S215C	c.644C>G	Unverified	AAC	TCC	TTT	2	C	G
TCGA-29-1785	RHOBTB3	ENST00000379982	p.S215C	c.644C>G	Unverified	AAC	TCC	TTT	2	C	G
TCGA-04-1361	RIMS1	ENST00000521978	p.T69R	c.206C>G	Verified	AAA	ACA	CCA	2	C	G
TCGA-04-1361	RIMS1	ENST00000521978	p.T69R	c.206C>G	Verified	AAA	ACA	CCA	2	C	G
TCGA-61-1915	RIN2	NM_018993.2	p.L325V	c.973C>G	Unverified	cgg	ctg	gcc	1	C	G
TCGA-61-1915	RIN2_ENST0	ENST00000255006	p.L374V	c.1120C>G	Unverified	CGG	CTG	GCC	1	C	G
TCGA-61-1899	RIPK2	ENST00000220751	p.P358A	c.1072C>G	Unverified	TCT	CCT	GAA	1	C	G
TCGA-13-0920	RIPK4	ENST00000332512	p.I624M	c.1872C>G	Verified	CTC	ATC	GAC	3	C	G
TCGA-13-0923	RMND5B	ENST00000313386	p.H200D	c.598C>G	Verified	ctg	cac	ttc	1	C	G
TCGA-29-1691	RNF121	ENST00000361756	p.T131T	c.393C>G	Unverified	aca	acc	cca	3	C	G
TCGA-61-1913	RNF157	XM_290732.4	p.Q855E	c.2563C>G	Unverified	aca	caa	gat	1	C	G
TCGA-04-1347	RNF213	ENST00000336301	p.F144L	c.432C>G	Verified	CTT	TTC	AAG	3	C	G
TCGA-29-1783	ROCK2	ENST00000315872	p.L484V	c.1450C>G	Unverified	GAG	CTA	GAA	1	C	G
TCGA-29-1783	ROCK2_ENS	ENST00000315872	p.L484V	c.1450C>G	Unverified	GAG	CTA	GAA	1	C	G
TCGA-13-0913	RPE65	ENST00000262340	p.H314D	c.940C>G	Verified	CAT	CAC	ATC	1	C	G
TCGA-29-1783	RPL30	ENST00000287038	p.Y31*	c.93C>G	Unverified	GGG	TAC	AAG	3	C	G
TCGA-23-1029	RPL8	ENST00000394920	p.Q253E	c.757C>G	Unverified	GTG	CAG	GAG	1	C	G
TCGA-61-1907	RPP21	ENST00000442966	p.R86G	c.256C>G	Unverified	CAG	CGC	TGG	1	C	G
TCGA-13-1488	RPS6KA5	ENST00000261991	p.S263C	c.788C>G	Verified	AAT	TCC	CAA	2	C	G
TCGA-13-0919	RRAS	ENST00000246792	p.Y66*	c.198C>G	Unverified	tcc	tac	acg	3	C	G
TCGA-04-1343	RREB1	ENST00000379933	p.P1539A	c.4615C>G	Verified	AGG	CCA	TAC	1	C	G
TCGA-61-1740	RRP7A	ENST00000323013	p.S222R	c.666C>G	Unverified	GCC	AGC	TTG	3	C	G
TCGA-23-2078	RS1	ENST00000379984	p.Y166*	c.498C>G	Verified	tac	tac	aag	3	C	G
TCGA-20-1687	RSBN1L	ENST00000334955	p.P766A	c.2296C>G	Unverified	GAA	CCT	GTG	1	C	G
TCGA-09-2051	RSPH3	ENST00000252655	p.P42P	c.126C>G	Unverified	CTT	CCC	GGG	3	C	G
TCGA-25-2042	RTN2	ENST00000245923	p.R223G	c.667C>G	Unverified	TCC	CGA	TCG	1	C	G

TCGA-23-2078	RTN4	ENST00000357376	p.S642C	c.1925C>G	Verified	ATT	TCT	AAG	2	C	G
TCGA-13-0920	RUFY1	ENST00000437570	p.L348V	c.1042C>G	Verified	CAG	CTG	GAA	1	C	G
TCGA-13-1501	RUFY1	ENST00000437570	p.T227S	c.680C>G	Verified	AAG	ACT	AAC	2	C	G
TCGA-13-1501	RUFY1	ENST00000437570	p.T227S	c.680C>G	Verified	AAG	ACT	AAC	2	C	G
TCGA-24-2035	RUSC1	ENST00000292254	p.L18V	c.52C>G	Verified	GGT	CTT	CTG	1	C	G
TCGA-23-1022	RYR1	ENST00000359596	p.S2948W	c.8843C>G	Verified	GAC	TCG	TCT	2	C	G
TCGA-23-1022	RYR2	ENST00000366574	p.A2936G	c.8807C>G	Verified	GAA	GCC	CAT	2	C	G
TCGA-25-1318	SACS	NM_014363.3	p.L2288V	c.6862C>G	Verified	agt	ctc	att	1	C	G
TCGA-25-1318	SACS	NM_014363.3	p.L2288V	c.6862C>G	Verified	agt	ctc	att	1	C	G
TCGA-61-2094	SAFB	ENST00000292123	p.R913G	c.2737C>G	Unverified	act	cgc	cgc	1	C	G
TCGA-29-1761	SAFB2	ENST00000252542	p.P365R	c.1094C>G	Unverified	GTC	CCT	CCG	2	C	G
TCGA-24-1850	SAMD9	ENST00000379958	p.S1185*	c.3554C>G	Unverified	AAG	TCA	AAA	2	C	G
TCGA-13-0903	SATB1	ENST00000338745	p.V267V	c.801C>G	Verified	cat	gtc	aat	3	C	G
TCGA-13-1498	SCAF11	ENST00000369367	p.T602R	c.1805C>G	Verified	AAA	ACA	GAG	2	C	G
TCGA-04-1652	SCAF8	ENST00000367178	p.P664R	c.1991C>G	Unverified	ATG	CCG	GTT	2	C	G
TCGA-04-1652	SCAF8	ENST00000367178	p.P664R	c.1991C>G	Unverified	ATG	CCG	GTT	2	C	G
TCGA-61-1907	SCN11A	ENST00000302328	p.F1146L	c.3438C>G	Unverified	TCC	TTC	CGG	3	C	G
TCGA-61-2012	SCN1A	ENST00000375405	p.S1907C	c.5720C>G	Verified	GTA	TCT	GCT	2	C	G
TCGA-09-2050	SCN3A	ENST00000283254	p.A1768G	c.5303C>G	Verified	ATC	GCG	GTC	2	C	G
TCGA-13-0904	SCN9A	ENST00000409672	p.N365K	c.1095C>G	Verified	GAA	AAC	CTT	3	C	G
TCGA-24-1845	SCRN2	ENST00000290216	p.T335S	c.1004C>G	Unverified	CCC	ACT	TTT	2	C	G
TCGA-04-1530	SCYL1	ENST00000270176	p.H495Q	c.1485C>G	Unverified	ACC	CAC	AAC	3	C	G
TCGA-04-1530	SCYL1_ENST	ENST00000270176	p.H495Q	c.1485C>G	Unverified	ACC	CAC	AAC	3	C	G
TCGA-61-1915	SDCBP2	ENST00000381808	p.D65E	c.195C>G	Unverified	GGG	GAC	CAG	3	C	G
TCGA-61-1915	SDCBP2_EN:	ENST00000360779	p.D150E	c.450C>G	Unverified	GGG	GAC	CAG	3	C	G
TCGA-61-1915	SDCBP2_EN:	ENST00000381812	p.D150E	c.450C>G	Unverified	GGG	GAC	CAG	3	C	G
TCGA-13-0755	SEC14L3	ENST00000215812	p.C301W	c.903C>G	Verified	GGC	TGC	GTT	3	C	G
TCGA-13-0905	SEC22A	ENST00000309934	p.L132V	c.394C>G	Verified	TCT	CTT	TCA	1	C	G
TCGA-23-1120	SEC23IP	ENST00000369075	p.Q127E	c.379C>G	Verified	TCC	CAA	GAT	1	C	G
TCGA-24-2024	SELE	ENST00000333360	p.T514S	c.1541C>G	Verified	GGC	ACT	GTG	2	C	G
TCGA-24-1422	SEMA4C	ENST00000305476	p.Y641*	c.1923C>G	Unverified	GGC	TAC	CTT	3	C	G
TCGA-13-0890	SENP6	ENST00000447266	p.T297S	c.890C>G	Verified	CAC	ACT	TAT	2	C	G
TCGA-29-1691	SERPINB2	ENST00000299502	p.S226W	c.677C>G	Unverified	aac	tcg	gct	2	C	G
TCGA-04-1651	SERPINC1	ENST00000367698	p.T433S	c.1298C>G	Unverified	gtg	act	ttc	2	C	G

TCGA-04-1651	SERPINC1	ENST00000367698	p.T433S	c.1298C>G	Unverified	gtg	act	ttc	2	C	G
TCGA-13-0913	SERPINH1	ENST00000533603	p.L80L	c.240C>G	Verified	ggg	ctc	gtg	3	C	G
TCGA-13-1507	SETDB1	ENST00000271640	p.S504C	c.1511C>G	Verified	CAT	TCC	TCC	2	C	G
TCGA-10-0930	SH2B3	ENST00000341259	p.S360C	c.1079C>G	Verified	CTG	TCC	TGC	2	C	G
TCGA-10-0930	SH2B3	ENST00000341259	p.S360C	c.1079C>G	Verified	CTG	TCC	TGC	2	C	G
TCGA-25-1318	SIAH1	ENST00000568007	p.T13T	c.39C>G	Verified	GGT	ACC	TCG	3	C	G
TCGA-25-1318	SIAH1	ENST00000568007	p.T13T	c.39C>G	Verified	GGT	ACC	TCG	3	C	G
TCGA-61-1904	SIDT2	ENST00000324225	p.Q161E	c.481C>G	Unverified	GAG	CAG	TTC	1	C	G
TCGA-04-1367	SIGLEC11	ENST00000447370	p.P80R	c.239C>G	Verified	AGC	CCA	AAG	2	C	G
TCGA-24-2024	SIGLEC12	ENST00000291707	p.L420V	c.1258C>G	Verified	ACC	CTG	AGC	1	C	G
TCGA-04-1655	SIK1	ENST00000270162	p.L781V	c.2341C>G	Unverified	GTC	CTG	GTG	1	C	G
TCGA-61-1900	SIK3	SU_QSK	p.Q848E	c.2542C>G	Unverified	cag	cag	cct	1	C	G
TCGA-23-1021	SIPA1L1	ENST00000555818	p.S364C	c.1091C>G	Verified	GCT	TCC	GCA	2	C	G
TCGA-61-1998	SIPA1L2	ENST00000366630	p.V226V	c.678C>G	Verified	ATG	GTC	CCT	3	C	G
TCGA-61-1998	SLC10A2	ENST00000245312	p.Y253*	c.759C>G	Verified	TGG	TAC	AGG	3	C	G
TCGA-13-0923	SLC16A4	ENST00000369779	p.S307C	c.920C>G	Verified	TGG	TCT	TTT	2	C	G
TCGA-13-0904	SLC18A3	ENST00000374115	p.P515A	c.1543C>G	Verified	CCG	CCT	GGC	1	C	G
TCGA-25-1313	SLC1A7	ENST00000371494	p.A449A	c.1347C>G	Verified	ATT	GCC	GTT	3	C	G
TCGA-23-1116	SLC22A17	ENST00000206544	p.F354L	c.1062C>G	Unverified	gtc	ttc	ctg	3	C	G
TCGA-24-1844	SLC23A3	ENST00000295738	p.S97C	c.290C>G	Unverified	ATG	TCT	ACC	2	C	G
TCGA-24-1844	SLC23A3_EN	ENST00000455516	p.S97C	c.290C>G	Unverified	ATG	TCT	ACC	2	C	G
TCGA-23-2077	SLC25A10	ENST00000350690	p.V123V	c.369C>G	Unverified	TTG	GTC	AAC	3	C	G
TCGA-23-1029	SLC35D3	ENST00000331858	p.A351G	c.1052C>G	Unverified	GAG	GCA	GCA	2	C	G
TCGA-29-1775	SLC36A4	ENST00000326402	p.P490R	c.1469C>G	Unverified	AGT	CCT	TTT	2	C	G
TCGA-61-1740	SLC38A7	ENST00000219320	p.P231A	c.691C>G	Unverified	ACC	CCA	GGG	1	C	G
TCGA-23-2078	SLC43A3	ENST00000395123	p.A411G	c.1232C>G	Unverified	GCG	GCC	TTC	2	C	G
TCGA-29-1691	SLC44A1	ENST00000374720	p.S447C	c.1340C>G	Unverified	GGA	TCT	TTC	2	C	G
TCGA-24-1422	SLC45A4	ENST00000024061	p.P271A	c.811C>G	Unverified	GAG	CCC	GAG	1	C	G
TCGA-24-2024	SLC4A9_ENS	ENST00000506757	p.P441R	c.1322C>G	Verified	CAG	CCC	CTC	2	C	G
TCGA-13-0906	SLC5A1	ENST00000266088	p.S446*	c.1337C>G	Unverified	CAG	TCA	GCA	2	C	G
TCGA-61-1899	SLC5A6	ENST00000310574	p.L268L	c.804C>G	Unverified	ATG	CTC	TCC	3	C	G
TCGA-30-1714	SLC5A8	ENST00000536262	p.T498S	c.1493C>G	Unverified	TTT	ACT	ACT	2	C	G
TCGA-10-0930	SLC6A3	ENST00000270349	p.R445G	c.1333C>G	Verified	CAC	CGT	GAG	1	C	G
TCGA-10-0930	SLC6A3	ENST00000270349	p.R445G	c.1333C>G	Verified	CAC	CGT	GAG	1	C	G

TCGA-24-1435	SLCO1C1	ENST00000266509	p.L442V	c.1324C>G	Verified	TCC	CTG	TTT	1	C	G
TCGA-23-1110	SLCO2A1	ENST00000310926	p.A595A	c.1785C>G	Unverified	TGC	GCC	TAC	3	C	G
TCGA-20-1686	SLCO3A1	ENST00000318445	p.L391V	c.1171C>G	Unverified	CTG	CTT	GGG	1	C	G
TCGA-10-0930	SLIT1	ENST00000266058	p.Q163E	c.487C>G	Verified	TTA	CAG	CTG	1	C	G
TCGA-10-0930	SLIT1	ENST00000266058	p.Q163E	c.487C>G	Verified	TTA	CAG	CTG	1	C	G
TCGA-20-1686	SLIT2	ENST00000504154	p.L832V	c.2494C>G	Unverified	TCT	CTA	CAT	1	C	G
TCGA-13-0762	SLIT2	ENST00000504154	p.P1387R	c.4160C>G	Verified	TTG	CCC	ATC	2	C	G
TCGA-13-0762	SLIT2	ENST00000504154	p.P1387R	c.4160C>G	Verified	TTG	CCC	ATC	2	C	G
TCGA-61-1910	SLX4	ENST00000294008	p.A1409A	c.4227C>G	Unverified	CAG	GCC	AAC	3	C	G
TCGA-29-1768	SMARCA1	NM_003069.2	p.F620L	c.1860C>G	Unverified	gta	ttc	cgt	3	C	G
TCGA-04-1530	SMARCAL1	ENST00000357276	p.P199R	c.596C>G	Verified	tcc	cct	tcg	2	C	G
TCGA-25-1318	SMC1A	ENST00000322213	p.T443T	c.1329C>G	Verified	atc	acc	act	3	C	G
TCGA-25-1318	SMC1A	ENST00000322213	p.T443T	c.1329C>G	Verified	atc	acc	act	3	C	G
TCGA-13-0900	SMC1B	NM_148674.1	p.H1143Q	c.3429C>G	Verified	gtg	cac	agt	3	C	G
TCGA-09-1665	SMOC2	ENST00000354536	p.A92A	c.276C>G	Verified	GTG	GCC	GAA	3	C	G
TCGA-24-1470	SMYD1	ENST00000419482	p.L62L	c.186C>G	Verified	AAG	CTC	CAT	3	C	G
TCGA-13-1488	SNAP47	ENST00000366759	p.S370*	c.1109C>G	Verified	TGC	TCA	GTC	2	C	G
TCGA-23-1122	SNPH	ENST00000381873	p.A375G	c.1124C>G	Unverified	GAG	GCC	CCA	2	C	G
TCGA-61-1740	SNTA1	ENST00000217381	p.L113V	c.337C>G	Unverified	ATT	CTC	ATT	1	C	G
TCGA-61-1907	SNUPN	ENST00000308588	p.H312D	c.934C>G	Unverified	GAG	CAC	AAG	1	C	G
TCGA-13-0795	SNW1	ENST00000261531	p.Q194E	c.580C>G	Verified	aaa	cag	agg	1	C	G
TCGA-29-1784	SNX1	ENST00000559844	p.L351V	c.1051C>G	Unverified	agt	cta	gcc	1	C	G
TCGA-09-1665	SNX27	ENST00000368843	p.N206K	c.618C>G	Verified	CAG	AAC	CTG	3	C	G
TCGA-61-1907	SOCS5	ENST00000306503	p.L108V	c.322C>G	Unverified	aga	ctt	gca	1	C	G
TCGA-29-1769	SORCS3	ENST00000369701	p.I213M	c.639C>G	Unverified	CTT	ATC	CTG	3	C	G
TCGA-04-1530	SORT1	ENST00000256637	p.A762G	c.2285C>G	Unverified	CTG	GCC	ATC	2	C	G
TCGA-04-1362	SOS1	ENST00000426016	p.L1231V	c.3691C>G	Verified	cca	cta	cat	1	C	G
TCGA-25-2400	SOS1	ENST00000426016	p.P1183R	c.3548C>G	Verified	att	cct	cct	2	C	G
TCGA-30-1856	SOSTDC1	ENST00000307068	p.P12A	c.34C>G	Unverified	CTT	CCC	CTT	1	C	G
TCGA-23-1120	SOX13	ENST00000367204	p.P497R	c.1490C>G	Unverified	CGG	CCC	AAG	2	C	G
TCGA-61-2095	SOX13	ENST00000367204	p.N105K	c.315C>G	Unverified	TTC	AAC	CGA	3	C	G
TCGA-24-2267	SOX5	ENST00000451604	p.P19R	c.56C>G	Verified	cga	cca	gcc	2	C	G
TCGA-13-0760	SP110	ENST00000258381	p.P174A	c.520C>G	Verified	TCG	CCC	AGC	1	C	G
TCGA-25-2401	SP4	ENST00000222584	p.Q415E	c.1243C>G	Verified	CAA	CAG	ATC	1	C	G

TCGA-25-1326	SPAG4	ENST00000374273	p.P129R	c.386C>G	Unverified	CCC	CCG	CGG	2	C	G
TCGA-24-1435	SPANXN3	ENST00000370503	p.S134C	c.401C>G	Verified	GGA	TCT	TCA	2	C	G
TCGA-13-0900	SPECC1	ENST00000261503	p.T144R	c.431C>G	Verified	CCT	ACG	AAA	2	C	G
TCGA-13-1489	SPOCD1	ENST00000360482	p.P579A	c.1735C>G	Verified	GGC	CCA	ATG	1	C	G
TCGA-25-2401	SPRED1	ENST00000299084	p.V246V	c.738C>G	Verified	ATT	GTC	AGA	3	C	G
TCGA-30-1718	SPRYD5_ENS	ENST00000327733	p.D29E	c.87C>G	Unverified	ATA	GAC	TGT	3	C	G
TCGA-61-2102	SPTAN1	ENST00000372731	p.L1157V	c.3469C>G	Verified	GAC	CTG	GAG	1	C	G
TCGA-13-0904	SPTB	ENST00000389722	p.L728V	c.2182C>G	Verified	CAG	CTG	AAG	1	C	G
TCGA-61-1733	SRCAP	ENST00000262518	p.P1878R	c.5633C>G	Unverified	CCC	CCA	CCA	2	C	G
TCGA-61-1733	SRCAP	ENST00000262518	p.P1878R	c.5633C>G	Unverified	CCC	CCA	CCA	2	C	G
TCGA-24-2280	SRCAP	ENST00000262518	p.F2032L	c.6096C>G	Unverified	CAG	TTC	CCT	3	C	G
TCGA-25-1326	SRCAP	ENST00000262518	p.V990V	c.2970C>G	Verified	CCA	GTC	AAG	3	C	G
TCGA-29-1784	SREBF2	ENST00000361204	p.A373G	c.1118C>G	Unverified	AAG	GCC	ATT	2	C	G
TCGA-13-0885	SREK1IP1	ENST00000513458	p.T47R	c.140C>G	Verified	AGT	ACA	AGT	2	C	G
TCGA-20-0990	SRGAP1	ENST00000355086	p.S938C	c.2813C>G	Verified	AGG	TCC	ACG	2	C	G
TCGA-61-2095	SSH2	ENST00000269033	p.H212D	c.634C>G	Unverified	AGC	CAT	ATC	1	C	G
TCGA-24-1435	ST3GAL3	ENST00000361746	p.P117A	c.349C>G	Verified	CAC	CCT	CTA	1	C	G
TCGA-30-1857	STK10	ENST00000176763	p.T341S	c.1022C>G	Unverified	CAT	ACT	CAG	2	C	G
TCGA-61-1910	STK16_ENST	ENST00000409638	p.L247V	c.739C>G	Unverified	GCC	CTT	GCT	1	C	G
TCGA-25-1326	STK24	ENST00000376547	p.A322G	c.965C>G	Verified	GAC	GCG	GAA	2	C	G
TCGA-13-0755	STK31	ENST00000355870	p.S183C	c.548C>G	Verified	ACC	TCT	GAA	2	C	G
TCGA-13-0714	STK32C	ENST00000298630	p.Y256*	c.768C>G	Unverified	CCG	TAC	ATG	3	C	G
TCGA-25-2392	STRADA	ENST00000336174	p.H231D	c.691C>G	Verified	GTC	CAC	GAT	1	C	G
TCGA-61-1740	STX12	ENST00000373943	p.T275R	c.824C>G	Unverified	AAA	ACG	AAG	2	C	G
TCGA-09-2056	SULF1	ENST00000260128	p.F168L	c.504C>G	Verified	CGC	TTC	TAT	3	C	G
TCGA-36-1577	SULF2	ENST00000359930	p.N640K	c.1920C>G	Unverified	cag	aac	aaa	3	C	G
TCGA-20-1687	SUPT7L	ENST00000337768	p.L158L	c.474C>G	Unverified	CTC	CTC	TAC	3	C	G
TCGA-04-1338	SVEP1	ENST00000401783	p.A807G	c.2420C>G	Verified	GCA	GCT	CGT	2	C	G
TCGA-29-1785	SVEP1	ENST00000401783	p.A608G	c.1823C>G	Unverified	CCA	GCT	TTC	2	C	G
TCGA-29-1785	SVEP1	ENST00000401783	p.A608G	c.1823C>G	Unverified	CCA	GCT	TTC	2	C	G
TCGA-24-2289	SVIL	ENST00000355867	p.L124V	c.370C>G	Unverified	ACT	CTG	GAT	1	C	G
TCGA-13-0924	SVIL	ENST00000355867	p.V1426V	c.4278C>G	Verified	AGC	GTC	AAC	3	C	G
TCGA-30-1718	SYNE1	ENST00000265368	p.Q3961E	c.11881C>G	Unverified	ACC	CAG	CAA	1	C	G
TCGA-30-1718	SYNE1_ENST	ENST00000265368	p.Q3961E	c.11881C>G	Unverified	ACC	CAG	CAA	1	C	G

TCGA-13-1497	SYNJ1	NM_003895.2	p.V355V	c.1065C>G	Verified	caa	gtc	cag	3	C	G
TCGA-04-1367	SYNPO2L	ENST00000372873	p.R544G	c.1630C>G	Verified	AGC	CGT	GCG	1	C	G
TCGA-24-1470	SYT13	ENST00000020926	p.H252Q	c.756C>G	Verified	CGT	CAC	AGC	3	C	G
TCGA-04-1651	SYT3	ENST00000338916	p.S196C	c.587C>G	Unverified	GGC	TCT	GGG	2	C	G
TCGA-04-1651	SYT3	ENST00000338916	p.S196C	c.587C>G	Unverified	GGC	TCT	GGG	2	C	G
TCGA-24-1616	SYT6	ENST00000369546	p.P314R	c.941C>G	Verified	GAT	CCC	TAT	2	C	G
TCGA-24-1616	SYT6	ENST00000369546	p.S410C	c.1229C>G	Verified	CAC	TCC	TTG	2	C	G
TCGA-24-2288	TACC2	ENST00000369005	p.P1934A	c.5800C>G	Unverified	ACT	CCC	TCC	1	C	G
TCGA-13-0920	TADA1	ENST00000367874	p.L151V	c.451C>G	Verified	CAG	CTT	GAA	1	C	G
TCGA-24-0975	TAF1	ENST00000373790	p.S319C	c.956C>G	Unverified	gag	tcc	aaa	2	C	G
TCGA-13-0890	TAF1	ENST00000373790	p.D1416E	c.4248C>G	Verified	aag	gac	tac	3	C	G
TCGA-24-1846	TAF1L	ENST00000242310	p.L1148V	c.3442C>G	Unverified	gaa	cta	cgg	1	C	G
TCGA-13-0923	TAF4	ENST00000252996	p.R459G	c.1375C>G	Verified	GTC	CGA	AGT	1	C	G
TCGA-10-0930	TAF5	ENST00000369839	p.L515V	c.1543C>G	Verified	AGT	CTT	ATA	1	C	G
TCGA-10-0930	TAF5	ENST00000369839	p.L515V	c.1543C>G	Verified	AGT	CTT	ATA	1	C	G
TCGA-13-0900	TAGAP	ENST00000367066	p.R203G	c.607C>G	Verified	CCC	CGG	CCC	1	C	G
TCGA-24-2288	TANC1	ENST00000263635	p.L1826V	c.5476C>G	Unverified	CAT	CTG	TAC	1	C	G
TCGA-25-1318	TARS	ENST00000265112	p.H464D	c.1390C>G	Verified	GCT	CAC	ATA	1	C	G
TCGA-25-1318	TARS	ENST00000265112	p.H464D	c.1390C>G	Verified	GCT	CAC	ATA	1	C	G
TCGA-04-1367	TAS2R50	ENST00000506868	p.L12V	c.34C>G	Verified	ATT	CTA	ATA	1	C	G
TCGA-61-2102	TBC1D4	ENST00000377636	p.L994V	c.2980C>G	Verified	AAC	CTC	CTG	1	C	G
TCGA-24-2024	TBCA	ENST00000380377	p.P65R	c.194C>G	Verified	ATC	CCA	GAT	2	C	G
TCGA-04-1331	TBL1XR1	ENST00000430069	p.T94R	c.281C>G	Verified	caa	aca	aga	2	C	G
TCGA-13-1481	TBXAS1	ENST00000263552	p.I179M	c.537C>G	Verified	GAC	ATC	CAG	3	C	G
TCGA-29-1761	TCEB1	ENST00000284811	p.S24C	c.71C>G	Unverified	TCA	TCT	GAT	2	C	G
TCGA-24-2280	TCERG1	ENST00000296702	p.P988A	c.2962C>G	Unverified	GAT	CCT	CGA	1	C	G
TCGA-13-0884	TCF20	ENST00000359486	p.T1831S	c.5492C>G	Verified	ACC	ACT	TCA	2	C	G
TCGA-13-1507	TCF20	ENST00000359486	p.A1060A	c.3180C>G	Verified	CTG	GCC	TCT	3	C	G
TCGA-24-2267	TCF20	ENST00000359486	p.G522G	c.1566C>G	Verified	GGA	GGC	TGC	3	C	G
TCGA-13-1488	TCTEX1D2	ENST00000325318	p.F140L	c.420C>G	Verified	TGT	TTC	TAC	3	C	G
TCGA-23-1122	TDRD6	ENST00000316081	p.A1042A	c.3126C>G	Verified	CTT	GCC	AAG	3	C	G
TCGA-13-0893	TESK1	ENST00000336395	p.A160G	c.479C>G	Verified	ATT	GCC	CGA	2	C	G
TCGA-24-1104	TESK2	NM_007170	p.R469R	c.1407C>G	Verified	cca	cgc	cta	3	C	G
TCGA-36-1577	TEX10	ENST00000374902	p.R703G	c.2107C>G	Verified	CTT	CGA	GGA	1	C	G

TCGA-61-2094	TEX15	ENST00000256246	p.S1842C	c.5525C>G	Unverified	GAC	TCT	CCT	2	C	G
TCGA-29-1695	TFAP2D	ENST00000008391	p.T400R	c.1199C>G	Unverified	CAA	ACA	GTT	2	C	G
TCGA-61-1900	TFB2M	ENST00000366514	p.Q353E	c.1057C>G	Unverified	ATG	CAA	ATA	1	C	G
TCGA-24-1423	TFRC	ENST00000360110	p.T658R	c.1973C>G	Verified	ACA	ACA	GAT	2	C	G
TCGA-13-1499	TGM3	ENST00000381458	p.T464R	c.1391C>G	Verified	AAC	ACG	CCA	2	C	G
TCGA-13-0916	TGM6	ENST00000202625	p.V529V	c.1587C>G	Verified	AGG	GTC	AAC	3	C	G
TCGA-23-1110	TGS1	ENST00000260129	p.A726G	c.2177C>G	Verified	ATT	GCC	CTT	2	C	G
TCGA-25-1326	THEMIS	ENST00000368248	p.L184V	c.550C>G	Verified	ACT	CTA	AAG	1	C	G
TCGA-23-2077	THOC2	OTTHUMT00000058153.	p.T896R	c.2687C>G	Verified	atc	aca	aaa	2	C	G
TCGA-09-1674	THOC6	ENST00000326266	p.S338C	c.1013C>G	Unverified	TTC	TCC	CTG	2	C	G
TCGA-30-1856	THSD7B	ENST00000272643	p.T1302S	c.3905C>G	Unverified	GTG	ACC	CCC	2	C	G
TCGA-30-1856	THSD7B_EN	ENST00000413152	p.T1271S	c.3812C>G	Unverified	GTG	ACC	CCC	2	C	G
TCGA-13-1408	TIAM1	ENST00000286827	p.F515L	c.1545C>G	Verified	GTC	TTC	TGC	3	C	G
TCGA-61-1913	TIMM50	ENST00000314349	p.R102R	c.306C>G	Unverified	CAG	CGC	AAG	3	C	G
TCGA-23-1124	TIMMDC1	ENST00000494664	p.D73E	c.219C>G	Verified	AAG	GAC	CTT	3	C	G
TCGA-61-2094	TINAG	ENST00000259782	p.H39D	c.115C>G	Unverified	AAT	CAC	ACC	1	C	G
TCGA-09-2049	TJP1	ENST00000346128	p.Q1724E	c.5170C>G	Verified	TGG	CAA	AAC	1	C	G
TCGA-13-0886	TKTL1	ENST00000369915	p.A491G	c.1472C>G	Unverified	TTA	GCA	GCT	2	C	G
TCGA-30-1855	TLL1	ENST00000061240	p.P347A	c.1039C>G	Unverified	TGT	CCA	GCA	1	C	G
TCGA-24-1845	TLN2	ENST00000306829	p.P2264A	c.6790C>G	Unverified	ACC	CCA	GAA	1	C	G
TCGA-04-1331	TMEM132A	ENST00000005286	p.P717R	c.2150C>G	Unverified	CTG	CCA	GCT	2	C	G
TCGA-24-2280	TMEM146	ENST00000381624	p.A622G	c.1865C>G	Unverified	TCG	GCC	ATG	2	C	G
TCGA-29-1776	TMEM63B	ENST00000259746	p.P530R	c.1589C>G	Unverified	CTA	CCC	TCG	2	C	G
TCGA-29-1777	TMEM74	ENST00000297459	p.P50A	c.148C>G	Unverified	acc	cca	aga	1	C	G
TCGA-24-2289	TMEM87B	ENST00000283206	p.L329V	c.985C>G	Unverified	CTT	CTA	TAC	1	C	G
TCGA-20-0991	TMOD3	ENST00000308580	p.Q161E	c.481C>G	Verified	GAC	CAA	GAA	1	C	G
TCGA-29-1762	TMPRSS4	ENST00000437212	p.N255K	c.765C>G	Unverified	TTC	AAC	TGG	3	C	G
TCGA-13-1510	TNFAIP3	ENST00000237289	p.L421V	c.1261C>G	Unverified	aag	ctg	aac	1	C	G
TCGA-04-1337	TNFRSF1A	ENST00000162749	p.F144L	c.432C>G	Unverified	tgc	ttc	aat	3	C	G
TCGA-24-1422	TNFSF9	ENST00000245817	p.L124V	c.370C>G	Unverified	ggc	ctg	agc	1	C	G
TCGA-04-1356	TNIP1	ENST00000315050	p.A102A	c.306C>G	Verified	ACA	GCC	CCT	3	C	G
TCGA-24-2288	TNNI3K	ENST00000326637	p.Q291E	c.871C>G	Unverified	atc	caa	ata	1	C	G
TCGA-23-2078	TNR	ENST00000367674	p.T113S	c.338C>G	Verified	GTC	ACC	TTT	2	C	G
TCGA-25-2401	TNRC18_EN	ENST00000430969	p.Y1494*	c.4482C>G	Unverified	CAG	TAC	AAG	3	C	G

TCGA-24-2288	TOP1	ENST00000361337	p.G583G	c.1749C>G	Unverified	gag	ggc	ttg	3	C	G
TCGA-13-0919	TOP2A	ENST00000423485	p.Q355E	c.1063C>G	Verified	cat	cag	gtg	1	C	G
TCGA-13-0919	TOP2A	ENST00000423485	p.G307G	c.921C>G	Verified	aaa	ggc	ttt	3	C	G
TCGA-61-2008	TOR1AIP2	ENST00000367612	p.A138A	c.414C>G	Verified	GTG	GCC	CTC	3	C	G
TCGA-29-1699	TP53	ENST00000269305	p.R175G	c.523C>G	Unverified	AGG	CGC	TGC	1	C	G
TCGA-61-2094	TP53	ENST00000269305	p.L130V	c.388C>G	Verified	GCC	CTC	AAC	1	C	G
TCGA-13-0913	TP53	ENST00000269305	p.P278R	c.833C>G	Verified	TGT	CCT	GGG	2	C	G
TCGA-13-1481	TP53	ENST00000269305	p.P177R	c.530C>G	Verified	TGC	CCC	CAC	2	C	G
TCGA-30-1714	TP53	ENST00000269305	p.P151R	c.452C>G	Unverified	ACA	CCC	CCG	2	C	G
TCGA-29-1699	TP53_ENST(	ENST00000269305	p.R175G	c.523C>G	Unverified	AGG	CGC	TGC	1	C	G
TCGA-13-1481	TP53_ENST(	ENST00000269305	p.P177R	c.530C>G	Verified	TGC	CCC	CAC	2	C	G
TCGA-30-1714	TP53_ENST(	ENST00000269305	p.P151R	c.452C>G	Unverified	ACA	CCC	CCG	2	C	G
TCGA-29-1699	TP53_ENST(	ENST00000413465	p.R175G	c.523C>G	Unverified	AGG	CGC	TGC	1	C	G
TCGA-13-1481	TP53_ENST(	ENST00000413465	p.P177R	c.530C>G	Verified	TGC	CCC	CAC	2	C	G
TCGA-30-1714	TP53_ENST(	ENST00000413465	p.P151R	c.452C>G	Unverified	ACA	CCC	CCG	2	C	G
TCGA-29-1699	TP53_ENST(	ENST00000414315	p.R43G	c.127C>G	Unverified	AGG	CGC	TGC	1	C	G
TCGA-13-1481	TP53_ENST(	ENST00000414315	p.P45R	c.134C>G	Verified	TGC	CCC	CAC	2	C	G
TCGA-30-1714	TP53_ENST(	ENST00000414315	p.P19R	c.56C>G	Unverified	ACA	CCC	CCG	2	C	G
TCGA-29-1699	TP53_ENST(	ENST00000545858	p.R82G	c.244C>G	Unverified	AGG	CGC	TGC	1	C	G
TCGA-13-1481	TP53_ENST(	ENST00000545858	p.P84R	c.251C>G	Verified	TGC	CCC	CAC	2	C	G
TCGA-30-1714	TP53_ENST(	ENST00000545858	p.P58R	c.173C>G	Unverified	ACA	CCC	CCG	2	C	G
TCGA-13-1505	TPH2	ENST00000333850	p.T316R	c.947C>G	Verified	GAC	ACA	TGC	2	C	G
TCGA-25-1326	TPO	ENST00000345913	p.L11V	c.31C>G	Verified	acg	ctg	gtt	1	C	G
TCGA-24-1850	TPPP	ENST00000360578	p.A148G	c.443C>G	Unverified	AAG	GCG	CCC	2	C	G
TCGA-24-1435	TRAD	ENST00000291478	p.P801A	c.2401C>G	Verified	CGG	CCA	AAG	1	C	G
TCGA-24-1435	TRAIP	ENST00000331456	p.P47A	c.139C>G	Verified	tgc	cca	cag	1	C	G
TCGA-25-1313	TRAPPC8	ENST00000283351	p.L1102V	c.3304C>G	Verified	ATG	CTA	GTC	1	C	G
TCGA-24-1422	TRAPPC8	ENST00000283351	p.A1160G	c.3479C>G	Verified	AAG	GCA	ATA	2	C	G
TCGA-25-2392	TRIB3	ENST00000217233	p.P9A	c.25C>G	Unverified	GCT	CCT	GCG	1	C	G
TCGA-30-1855	TRIM33	NM_015906	p.L822V	c.2464C>G	Unverified	aac	ctg	cat	1	C	G
TCGA-13-0755	TRIM46	ENST00000334634	p.Q589E	c.1765C>G	Verified	acc	cag	ggc	1	C	G
TCGA-24-1431	TRIM54	NM_032546.2	p.S51C	c.152C>G	Unverified	ggc	tcc	acc	2	C	G
TCGA-04-1331	TRIO	ENST00000344204	p.R400R	c.1200C>G	Verified	aac	cgc	atc	3	C	G
TCGA-25-2392	TRIO	ENST00000344204	p.N1671K	c.5013C>G	Verified	tgc	aac	agc	3	C	G

TCGA-09-2056	TRIOBP_ENS	ENST00000406386	p.S1153C	c.3458C>G	Unverified	GAC	TCT	CTG	2	C	G
TCGA-24-1422	TRIP12	ENST00000283943	p.Q1281E	c.3841C>G	Verified	AAG	CAG	TGG	1	C	G
TCGA-23-2078	TRO	ENST00000319167	p.T323S	c.968C>G	Unverified	gcc	act	cag	2	C	G
TCGA-24-1103	TRPM5	ENST00000155858	p.G860G	c.2580C>G	Verified	CTG	GGC	CCC	3	C	G
TCGA-13-1510	TRPM7	ENST00000313478	p.S1427C	c.4280C>G	Verified	TGC	TCT	AAA	2	C	G
TCGA-61-1910	TRRAP	ENST00000355540	p.P2528R	c.7583C>G	Unverified	GAG	CCC	CGG	2	C	G
TCGA-61-1910	TRRAP_ENST	ENST00000359863	p.P2546R	c.7637C>G	Unverified	GAG	CCC	CGG	2	C	G
TCGA-23-1031	TSC22D2	ENST00000361875	p.S198C	c.593C>G	Verified	AGA	TCC	GGG	2	C	G
TCGA-29-1691	TSHZ3	NM_020856.1	p.P791A	c.2371C>G	Unverified	cac	ccc	gtc	1	C	G
TCGA-29-1691	TSHZ3_ENST	ENST00000240587	p.P974A	c.2920C>G	Unverified	CAC	CCC	GTC	1	C	G
TCGA-36-1577	TSPYL1	ENST00000368608	p.Y303*	c.909C>G	Unverified	AGG	TAC	ATA	3	C	G
TCGA-13-0913	TTBK2	ENST00000267890	p.Q752E	c.2254C>G	Verified	tct	caa	gac	1	C	G
TCGA-61-2095	TTC16	ENST00000373289	p.L555V	c.1663C>G	Unverified	GAG	CTG	AAG	1	C	G
TCGA-13-0760	TTC17	ENST00000039989	p.Y871*	c.2613C>G	Verified	CGT	TAC	CAA	3	C	G
TCGA-29-1703	TTC18	ENST00000355577	p.P134P	c.402C>G	Unverified	TCA	CCC	TTA	3	C	G
TCGA-61-1737	TTC21A	ENST00000431162	p.P602A	c.1804C>G	Unverified	TTG	CCA	GCT	1	C	G
TCGA-23-1124	TTC3	ENST00000354749	p.H1121D	c.3361C>G	Verified	GAA	CAT	GGT	1	C	G
TCGA-13-1497	TTF1	ENST00000334270	p.S240W	c.719C>G	Verified	GGA	TCG	CAA	2	C	G
TCGA-13-1497	TTK	ENST00000369798	p.S214*	c.641C>G	Verified	GAA	TCA	TTT	2	C	G
TCGA-25-1326	TTK	ENST00000369798	p.T637R	c.1910C>G	Verified	CAC	ACA	ATC	2	C	G
TCGA-04-1343	TTN	NM_003319	p.L2929V	c.8785C>G	Verified	atg	ctg	aaa	1	C	G
TCGA-09-2056	TTN	NM_003319	p.H769D	c.2305C>G	Verified	cct	cac	ttt	1	C	G
TCGA-13-0807	TTN	NM_003319	p.Q25512E	c.76534C>G	Verified	gat	cag	tat	1	C	G
TCGA-13-0755	TTN	NM_003319	p.T10524R	c.31571C>G	Verified	gtt	aca	gaa	2	C	G
TCGA-24-1463	TTN	NM_003319	p.V25109V	c.75327C>G	Verified	ggt	gtc	cga	3	C	G
TCGA-61-1900	TTN	NM_003319	p.Y23013*	c.69039C>G	Unverified	cgg	tac	gat	3	C	G
TCGA-61-1900	TTN_ENST0(	ENST00000342175	p.Y23205*	c.69615C>G	Unverified	CGG	TAC	GAT	3	C	G
TCGA-61-1900	TTN_ENST0(	ENST00000342992	p.Y29510*	c.88530C>G	Unverified	CGG	TAC	GAT	3	C	G
TCGA-04-1343	TTN_ENST0(	ENST00000356127	p.L2975V	c.8923C>G	Verified	ATG	CTG	AAA	1	C	G
TCGA-09-2056	TTN_ENST0(	ENST00000356127	p.H815D	c.2443C>G	Verified	CCT	CAC	TTT	1	C	G
TCGA-13-0807	TTN_ENST0(	ENST00000356127	p.Q32007E	c.96019C>G	Verified	GAT	CAG	TAT	1	C	G
TCGA-13-0755	TTN_ENST0(	ENST00000356127	p.T17019R	c.51056C>G	Verified	GTT	ACA	GAA	2	C	G
TCGA-24-1463	TTN_ENST0(	ENST00000356127	p.V31604V	c.94812C>G	Verified	GGC	GTC	CGA	3	C	G
TCGA-61-1900	TTN_ENST0(	ENST00000356127	p.Y29508*	c.88524C>G	Unverified	CGG	TAC	GAT	3	C	G

TCGA-61-1900	TTN_ENST0(	ENST00000359218	p.Y23138*	c.69414C>G	Unverified	CGG	TAC	GAT	3	C	G
TCGA-04-1343	TTN_ENST0(	ENST00000360870	p.L2975V	c.8923C>G	Verified	ATG	CTG	AAA	1	C	G
TCGA-09-2056	TTN_ENST0(	ENST00000360870	p.H815D	c.2443C>G	Verified	CCT	CAC	TTT	1	C	G
TCGA-13-1481	TUBB8	ENST00000328974	p.P346R	c.1037C>G	Verified	CTC	CCC	AAC	2	C	G
TCGA-29-1768	TUBGCP4	ENST00000564079	p.H121D	c.361C>G	Unverified	TCA	CAT	GTC	1	C	G
TCGA-24-1431	TXNRD2	ENST00000400521	p.Y194*	c.582C>G	Verified	AGA	TAC	CCC	3	C	G
TCGA-23-1031	TYRO3	NM_006293	p.A265G	c.794C>G	Verified	ctg	gct	gtt	2	C	G
TCGA-13-1497	TYRO3	NM_006293	p.P765P	c.2295C>G	Verified	gac	ccc	aag	3	C	G
TCGA-24-2288	TYW1	ENST00000359626	p.R406G	c.1216C>G	Unverified	CAT	CGC	TGC	1	C	G
TCGA-61-2012	UBA1	ENST00000377351	p.V589V	c.1767C>G	Verified	tgt	gtc	tac	3	C	G
TCGA-25-1313	UBAC2	ENST00000376440	p.L102V	c.304C>G	Verified	GCT	CTG	TTT	1	C	G
TCGA-04-1338	UBAP2	ENST00000379238	p.A744G	c.2231C>G	Verified	ACG	GCA	GCG	2	C	G
TCGA-13-0807	UBASH3A	ENST00000319294	p.N638K	c.1914C>G	Verified	GTG	AAC	CCA	3	C	G
TCGA-61-2095	UBE2O	ENST00000319380	p.L1258L	c.3774C>G	Unverified	CCC	CTC	TTC	3	C	G
TCGA-25-1313	UBE2Q2	ENST00000267938	p.P371R	c.1112C>G	Verified	CCT	CCA	AAG	2	C	G
TCGA-13-1499	UBR4	ENST00000375254	p.R1349G	c.4045C>G	Verified	GCT	CGT	GTT	1	C	G
TCGA-13-0887	UGT1A10	ENST00000344644	p.H37Q	c.111C>G	Verified	AGT	CAC	TGG	3	C	G
TCGA-61-2012	UGT1A4	ENST00000373409	p.P40P	c.120C>G	Unverified	AGC	CCC	TGG	3	C	G
TCGA-23-1110	UGT1A8	ENST00000373450	p.S78*	c.233C>G	Verified	TAC	TCA	ACC	2	C	G
TCGA-29-1764	UGT2A1	ENST00000503640	p.Q109E	c.325C>G	Unverified	TAT	CAG	GAG	1	C	G
TCGA-13-1488	UGT2A3	ENST00000251566	p.I129M	c.387C>G	Verified	TTT	ATC	TAC	3	C	G
TCGA-23-1022	UMOD	ENST00000302509	p.A556G	c.1667C>G	Verified	TTT	GCT	GGA	2	C	G
TCGA-24-1422	UMPS	ENST00000232607	p.P234A	c.700C>G	Unverified	ctg	ccc	agg	1	C	G
TCGA-29-1702	UNG	ENST00000336865	p.T129T	c.387C>G	Unverified	tgg	acc	cag	3	C	G
TCGA-29-1702	UNG_ENST0	ENST00000242576	p.T138T	c.414C>G	Unverified	TGG	ACC	CAG	3	C	G
TCGA-24-1104	UPB1	ENST00000326010	p.D309E	c.927C>G	Verified	CAG	GAC	TTT	3	C	G
TCGA-09-2056	UPF2	ENST00000356352	p.A587G	c.1760C>G	Verified	AAG	GCA	GCA	2	C	G
TCGA-04-1530	UQCRC1	ENST00000203407	p.T343T	c.1029C>G	Unverified	CAG	ACC	TTC	3	C	G
TCGA-29-1703	USP21	ENST00000368002	p.?	c.1384+5C>G	Unverified	CTC	CAT	CTG	1	C	G
TCGA-23-1120	USP24	XM_371254.3	p.L1006V	c.3016C>G	Verified	cag	ctc	gcc	1	C	G
TCGA-13-0885	USP25	ENST00000285679	p.L881L	c.2643C>G	Verified	tat	ctc	ata	3	C	G
TCGA-24-1847	USP29	ENST00000254181	p.P324R	c.971C>G	Unverified	att	ccc	ttt	2	C	G
TCGA-23-1124	USP33	ENST00000370793	p.P758R	c.2273C>G	Verified	ggc	cct	att	2	C	G
TCGA-04-1338	USP34	ENST00000398571	p.F2924L	c.8772C>G	Verified	CAG	TTC	AAG	3	C	G

TCGA-29-1696	USP53	ENST00000274030	p.S416C	c.1247C>G	Unverified	TCA	TCT	AAT	2	C	G
TCGA-29-1696	USP53	ENST00000274030	p.S416C	c.1247C>G	Unverified	TCA	TCT	AAT	2	C	G
TCGA-23-1124	USP7	ENST00000344836	p.A27G	c.80C>G	Verified	GAA	GCG	GGA	2	C	G
TCGA-23-1123	UTP14A	ENST00000394422	p.?	c.27-3C>G	Verified	gag	agc	ctt	3	C	G
TCGA-23-1123	UTP14A	ENST00000394422	p.?	c.27-3C>G	Verified	gag	agc	ctt	3	C	G
TCGA-24-1474	UTP6	ENST00000261708	p.A388G	c.1163C>G	Verified	GAA	GCT	CTG	2	C	G
TCGA-61-1998	VASP	ENST00000245932	p.R86R	c.258C>G	Unverified	GCT	CGC	CAG	3	C	G
TCGA-23-1114	VCL	ENST00000211998	p.Q689E	c.2065C>G	Unverified	AAT	CAA	GCT	1	C	G
TCGA-29-1699	VCL	ENST00000211998	p.P847R	c.2540C>G	Unverified	CCA	CCA	GAC	2	C	G
TCGA-13-0807	VN1R4	ENST00000311170	p.F23L	c.69C>G	Verified	AGC	TTC	TCT	3	C	G
TCGA-09-2051	VNN1	ENST00000367928	p.R459G	c.1375C>G	Unverified	GGA	CGC	TTG	1	C	G
TCGA-23-1118	VNN1	ENST00000367928	p.S486*	c.1457C>G	Verified	GCA	TCA	AAT	2	C	G
TCGA-04-1347	VPS11	ENST00000300793	p.A196G	c.587C>G	Verified	CAA	GCA	GGA	2	C	G
TCGA-13-0893	VPS13C	ENST00000261517	p.L935V	c.2803C>G	Verified	ATT	CTA	GTA	1	C	G
TCGA-61-1910	VPS13C	ENST00000261517	p.H2269D	c.6805C>G	Unverified	GAA	CAT	TCA	1	C	G
TCGA-61-1910	VPS13C_ENS	ENST00000395896	p.H2269D	c.6805C>G	Unverified	GAA	CAT	TCA	1	C	G
TCGA-29-1769	VPS13D	ENST00000358136	p.P3102P	c.9306C>G	Unverified	CGG	CCC	AAA	3	C	G
TCGA-24-1417	VPS33B	ENST00000333371	p.L399L	c.1197C>G	Verified	CGC	CTC	ATG	3	C	G
TCGA-09-2049	VPS52	ENST00000445902	p.A204G	c.611C>G	Verified	GAT	GCC	AAG	2	C	G
TCGA-13-0760	VPS52	ENST00000445902	p.T526R	c.1577C>G	Verified	CAG	ACA	ATT	2	C	G
TCGA-24-1464	VPS52	ENST00000445902	p.T597R	c.1790C>G	Verified	CGG	ACA	CAG	2	C	G
TCGA-13-0755	VSIG1	ENST00000217957	p.R89G	c.265C>G	Verified	gat	cga	att	1	C	G
TCGA-04-1361	VTI1A	ENST00000393077	p.L207V	c.619C>G	Verified	ATC	CTG	ATG	1	C	G
TCGA-04-1361	VTI1A	ENST00000393077	p.L207V	c.619C>G	Verified	ATC	CTG	ATG	1	C	G
TCGA-61-2113	VTN	ENST00000226218	p.P240P	c.720C>G	Verified	TAC	CCC	CGA	3	C	G
TCGA-61-1915	VWA3B	ENST00000477737	p.L15V	c.43C>G	Unverified	CAG	CTG	CAG	1	C	G
TCGA-25-2042	VWA5A	ENST00000392748	p.L230L	c.690C>G	Verified	GAA	CTC	CTG	3	C	G
TCGA-13-0923	VWF	ENST00000261405	p.T2255S	c.6764C>G	Unverified	TGC	ACT	CAG	2	C	G
TCGA-24-1423	WASF2	ENST00000430629	p.P268A	c.802C>G	Verified	ttg	cct	cct	1	C	G
TCGA-23-1022	WBP2NL	ENST00000329620	p.L83V	c.247C>G	Verified	AAC	CTC	ACT	1	C	G
TCGA-23-1809	WDR74	ENST00000529106	p.R7R	c.21C>G	Unverified	GCA	CGC	TGG	3	C	G
TCGA-23-1809	WDR74_ENS	ENST00000525239	p.R7R	c.21C>G	Unverified	GCA	CGC	TGG	3	C	G
TCGA-25-2042	WDR75	ENST00000314761	p.S743C	c.2228C>G	Verified	CCA	TCT	GCT	2	C	G
TCGA-13-0885	WNK3	ENST00000354646	p.Q625E	c.1873C>G	Verified	TAC	CAG	CAA	1	C	G

TCGA-24-0979	WNK4	ENST00000246914	p.S1231R	c.3693C>G	Verified	GCA	AGC	AAG	3	C	G
TCGA-61-2109	WNT7B	ENST00000339464	p.A154G	c.461C>G	Unverified	TCG	GCC	GAC	2	C	G
TCGA-25-2042	WRN	ENST00000298139	p.L211V	c.631C>G	Verified	aaa	ctg	tat	1	C	G
TCGA-25-1326	WWC3	NM_015691.2	p.P403P	c.1209C>G	Verified	aag	ccc	gac	3	C	G
TCGA-04-1652	XAB2	ENST00000358368	p.F388L	c.1164C>G	Unverified	CCC	TTC	AAG	3	C	G
TCGA-04-1652	XAB2	ENST00000358368	p.F388L	c.1164C>G	Unverified	CCC	TTC	AAG	3	C	G
TCGA-13-0903	XDH	ENST00000379416	p.T1033S	c.3098C>G	Verified	CTG	ACC	CAC	2	C	G
TCGA-13-1497	XPNPEP1	ENST00000369680	p.R473G	c.1417C>G	Verified	GCC	CGT	TCA	1	C	G
TCGA-13-1497	XYLT1	ENST00000261381	p.P849A	c.2545C>G	Verified	CAG	CCC	ATC	1	C	G
TCGA-23-1114	YARS2	ENST00000324868	p.S245C	c.734C>G	Unverified	GGA	TCT	GAT	2	C	G
TCGA-13-1507	YBX1	ENST00000321358	p.P149R	c.446C>G	Verified	TAT	CCA	CGT	2	C	G
TCGA-30-1891	YOD1	ENST00000315927	p.A53G	c.158C>G	Unverified	AAG	GCC	AAG	2	C	G
TCGA-24-1844	YTHDC1	ENST00000344157	p.S393C	c.1178C>G	Unverified	AGA	TCT	GCA	2	C	G
TCGA-13-0755	YY1AP1	ENST00000295566	p.T314S	c.941C>G	Verified	CTA	ACC	TGC	2	C	G
TCGA-29-1761	ZBTB12	ENST00000375527	p.D99E	c.297C>G	Unverified	AGG	GAC	ATC	3	C	G
TCGA-04-1342	ZBTB17	ENST00000375743	p.A107A	c.321C>G	Unverified	ACG	GCC	TGC	3	C	G
TCGA-24-2288	ZBTB47	ENST00000232974	p.L591V	c.1771C>G	Unverified	CAG	CTG	CGG	1	C	G
TCGA-30-1857	ZBTB5	ENST00000307750	p.L158L	c.474C>G	Unverified	GCC	CTC	AAT	3	C	G
TCGA-29-1770	ZC3H12C	ENST00000278590	p.S520S	c.1560C>G	Unverified	CCT	TCC	TTA	3	C	G
TCGA-25-2401	ZC3H18	ENST00000301011	p.V359V	c.1077C>G	Unverified	GAT	GTC	AGA	3	C	G
TCGA-23-1031	ZC3H7A	ENST00000355758	p.N465K	c.1395C>G	Verified	GCT	AAC	ATA	3	C	G
TCGA-13-1481	ZDHHC14	ENST00000359775	p.Q336E	c.1006C>G	Verified	ccg	cag	cca	1	C	G
TCGA-29-1761	ZEB2	ENST00000303660	p.A191G	c.572C>G	Unverified	gag	gcc	aat	2	C	G
TCGA-04-1649	ZFAT_ENST0	ENST00000377838	p.T933S	c.2798C>G	Unverified	AGC	ACT	GAG	2	C	G
TCGA-25-2400	ZFP28	ENST00000301318	p.I152M	c.456C>G	Verified	GTG	ATC	TCC	3	C	G
TCGA-23-1031	ZFPM2	ENST00000407775	p.S423R	c.1269C>G	Verified	CAG	AGC	CAA	3	C	G
TCGA-13-0762	ZIC1	ENST00000282928	p.S399C	c.1196C>G	Verified	AGC	TCT	GGC	2	C	G
TCGA-13-0762	ZIC1	ENST00000282928	p.S399C	c.1196C>G	Verified	AGC	TCT	GGC	2	C	G
TCGA-13-0920	ZKSCAN1	ENST00000324306	p.Q257E	c.769C>G	Verified	AGG	CAG	GAG	1	C	G
TCGA-13-1408	ZMIZ2	ENST00000309315	p.P403A	c.1207C>G	Verified	AAG	CCC	AAC	1	C	G
TCGA-61-1998	ZMIZ2	ENST00000309315	p.Q53E	c.157C>G	Verified	ACA	CAG	AGC	1	C	G
TCGA-24-1431	ZNF121	ENST00000320451	p.A96A	c.288C>G	Verified	GAA	GCC	TTT	3	C	G
TCGA-04-1338	ZNF136	ENST00000343979	p.L40V	c.118C>G	Verified	AAT	CTG	GCC	1	C	G
TCGA-61-1900	ZNF16	ENST00000394909	p.P48R	c.143C>G	Unverified	ACC	CCC	TGC	2	C	G

TCGA-61-2094	ZNF167	ENST00000273320	p.L454V	c.1360C>G	Unverified	CAT	CTC	ATT	1	C	G
TCGA-24-1563	ZNF177	ENST00000343499	p.H254Q	c.762C>G	Verified	GTG	CAC	AAG	3	C	G
TCGA-09-1674	ZNF197	ENST00000344387	p.T393S	c.1178C>G	Unverified	cac	act	ggt	2	C	G
TCGA-09-1674	ZNF213	ENST00000574902	p.L226V	c.676C>G	Unverified	GAT	CTC	TTC	1	C	G
TCGA-61-2109	ZNF215	ENST00000278319	p.L188V	c.562C>G	Unverified	AAC	CTG	TAC	1	C	G
TCGA-04-1356	ZNF224	ENST00000336976	p.H162Q	c.486C>G	Verified	TCC	CAC	TTT	3	C	G
TCGA-24-1846	ZNF24	ENST00000399061	p.Q90E	c.268C>G	Unverified	GAA	CAA	ATC	1	C	G
TCGA-23-1809	ZNF24	ENST00000399061	p.S142C	c.425C>G	Unverified	GTT	TCT	CTC	2	C	G
TCGA-30-1856	ZNF257	ENST00000322487	p.L300V	c.898C>G	Unverified	GCA	CTA	ACC	1	C	G
TCGA-20-1685	ZNF280D	ENST00000267807	p.Q496E	c.1486C>G	Unverified	ACT	CAA	CAC	1	C	G
TCGA-29-1777	ZNF333	ENST00000292530	p.T72R	c.215C>G	Unverified	GCC	ACA	GGT	2	C	G
TCGA-09-2049	ZNF385B	ENST00000410066	p.P128R	c.383C>G	Verified	TTT	CCA	AAT	2	C	G
TCGA-29-1702	ZNF439	ENST00000304030	p.S57C	c.170C>G	Unverified	ACC	TCT	ATA	2	C	G
TCGA-04-1343	ZNF44	ENST00000426973	p.P514A	c.1540C>G	Verified	AAA	CCT	TAT	1	C	G
TCGA-30-1855	ZNF493	ENST00000355504	p.H635D	c.1903C>G	Unverified	ATT	CAT	ATT	1	C	G
TCGA-30-1855	ZNF493_ENS	ENST00000392288	p.H763D	c.2287C>G	Unverified	ATT	CAT	ATT	1	C	G
TCGA-13-1481	ZNF498	ENST00000394152	p.R450G	c.1348C>G	Verified	CAC	CGG	AGG	1	C	G
TCGA-13-0760	ZNF536	ENST00000355537	p.P56A	c.166C>G	Verified	AAC	CCC	GAG	1	C	G
TCGA-13-0906	ZNF546	ENST00000347077	p.S70C	c.209C>G	Verified	CTC	TCC	CAA	2	C	G
TCGA-09-1665	ZNF563	ENST00000293725	p.S323R	c.969C>G	Unverified	GGA	AGC	TTT	3	C	G
TCGA-29-1761	ZNF574	ENST00000359044	p.R493G	c.1477C>G	Unverified	GAG	CGG	CGC	1	C	G
TCGA-04-1337	ZNF582	ENST00000301310	p.T331S	c.992C>G	Verified	CAG	ACT	GTT	2	C	G
TCGA-13-1507	ZNF608	ENST00000306315	p.L732V	c.2194C>G	Verified	AAA	CTG	AAA	1	C	G
TCGA-29-1762	ZNF638	ENST00000264447	p.T1568S	c.4703C>G	Unverified	GAA	ACT	CTC	2	C	G
TCGA-29-1693	ZNF644	ENST00000370440	p.S671*	c.2012C>G	Unverified	CAA	TCA	AGT	2	C	G
TCGA-25-2400	ZNF646	ENST00000300850	p.A386G	c.1157C>G	Unverified	CAT	GCT	GGG	2	C	G
TCGA-29-2427	ZNF70	ENST00000341976	p.L284V	c.850C>G	Verified	GAT	CTC	TGT	1	C	G
TCGA-04-1651	ZNF700	ENST00000254321	p.A544A	c.1632C>G	Unverified	AAA	GCC	TTC	3	C	G
TCGA-04-1651	ZNF700	ENST00000254321	p.A544A	c.1632C>G	Unverified	AAA	GCC	TTC	3	C	G
TCGA-24-1435	ZNF729	ENST00000357491	p.T763S	c.2288C>G	Unverified	CAT	ACT	AGG	2	C	G
TCGA-29-1764	ZNF777	ENST00000247930	p.P132A	c.394C>G	Unverified	TCC	CCT	GAA	1	C	G
TCGA-24-1850	ZNF862	ENST00000223210	p.I778M	c.2334C>G	Unverified	GAG	ATC	ATC	3	C	G
TCGA-24-1463	ZNRF3	ENST00000406323	p.T275T	c.825C>G	Verified	AGG	ACC	AAC	3	C	G
TCGA-13-1509	ZSCAN21	ENST00000292450	p.T321T	c.963C>G	Verified	CTC	ACC	CTC	3	C	G

TCGA-13-0760	ZZEF1	ENST00000381638	p.L2607L	c.7821C>G	Verified	TAC	CTC	TTC	3	C	G
TCGA-04-1638	A2M	ENST00000318602	p.V476V	c.1428C>T	Unverified	ACA	GTC	CAG	3	C	T
TCGA-30-1718	A4GNT	ENST00000236709	p.T306I	c.917C>T	Unverified	AAC	ACA	CTG	2	C	T
TCGA-24-1103	AACS	ENST00000316519	p.P413S	c.1237C>T	Verified	TCC	CCA	CTG	1	C	T
TCGA-61-2113	AARS2	ENST00000244571	p.P700P	c.2100C>T	Unverified	GTG	CCC	CTG	3	C	T
TCGA-24-1616	AASDH	ENST00000205214	p.R442*	c.1324C>T	Verified	GGA	CGA	AAA	1	C	T
TCGA-23-1122	ABCA10	ENST00000269081	p.L930F	c.2788C>T	Verified	GAT	CTT	GGT	1	C	T
TCGA-23-1032	ABCA12	ENST00000272895	p.H1573Y	c.4717C>T	Verified	TAT	CAC	CTC	1	C	T
TCGA-61-1737	ABCA12	ENST00000272895	p.Y1997Y	c.5991C>T	Unverified	GGC	TAC	TCT	3	C	T
TCGA-29-1761	ABCA13_EN	ENST00000435803	p.T1377M	c.4130C>T	Unverified	GAC	ACG	TTA	2	C	T
TCGA-29-1702	ABCA13_EN	ENST00000435803	p.R4043R	c.12129C>T	Unverified	GAC	CGC	GTG	3	C	T
TCGA-04-1337	ABCA3	ENST00000301732	p.H1069Y	c.3205C>T	Unverified	CCT	CAC	GCC	1	C	T
TCGA-13-0755	ABCA3	ENST00000301732	p.C1494C	c.4482C>T	Verified	GCC	TGC	GTG	3	C	T
TCGA-13-1507	ABCA4	ENST00000370225	p.S281L	c.842C>T	Verified	ATG	TCA	CCA	2	C	T
TCGA-25-2393	ABCA6	ENST00000284425	p.A1534V	c.4601C>T	Verified	CAG	GCT	GCA	2	C	T
TCGA-23-1122	ABCA9	ENST00000340001	p.P898S	c.2692C>T	Verified	TCT	CCA	AAT	1	C	T
TCGA-61-1904	ABCB4	ENST00000359206	p.T560M	c.1679C>T	Unverified	GCC	ACG	TCA	2	C	T
TCGA-04-1342	ABCB9	ENST00000392439	p.R700W	c.2098C>T	Unverified	CAC	CGG	CTG	1	C	T
TCGA-20-0991	ABCC10	ENST00000244533	p.R957W	c.2869C>T	Verified	CTC	CGG	GCA	1	C	T
TCGA-23-1022	ABCC10	ENST00000244533	p.Q311*	c.931C>T	Verified	CTT	CAG	GCA	1	C	T
TCGA-30-1714	ABCC11	ENST00000356608	p.T70T	c.210C>T	Unverified	AGA	ACC	ATG	3	C	T
TCGA-29-1693	ABCC5	ENST00000334444	p.S944S	c.2832C>T	Unverified	GCT	TCC	TCC	3	C	T
TCGA-23-1122	ABCC6	ENST00000205557	p.A624V	c.1871C>T	Verified	GCT	GCC	GGG	2	C	T
TCGA-13-0883	ABCC9	ENST00000261200	p.S51S	c.153C>T	Verified	GGG	AGC	CAA	3	C	T
TCGA-24-1844	ABCC9	ENST00000261200	p.V1319V	c.3957C>T	Unverified	TGT	GTC	AGA	3	C	T
TCGA-24-1844	ABCC9_ENS	ENST00000261201	p.V1319V	c.3957C>T	Unverified	TGT	GTC	AGA	3	C	T
TCGA-23-1120	ABCF1	ENST00000326195	p.P843S	c.2527C>T	Verified	CGG	CCC	CGA	1	C	T
TCGA-29-1703	ABCF2	ENST00000287844	p.A170V	c.509C>T	Unverified	CGG	GCC	ATG	2	C	T
TCGA-29-1695	ABCF2	ENST00000287844	p.N622N	c.1866C>T	Unverified	CAC	AAC	GTG	3	C	T
TCGA-13-1499	ABCG4	ENST00000307417	p.A608V	c.1823C>T	Verified	CGA	GCG	CTG	2	C	T
TCGA-23-1029	ABCG8	ENST00000272286	p.Y37Y	c.111C>T	Unverified	CTG	TAC	TTC	3	C	T
TCGA-20-1683	ABHD16B	ENST00000369916	p.V227V	c.681C>T	Unverified	GTG	GTC	GAG	3	C	T
TCGA-36-1577	ABHD6	ENST00000295962	p.P269L	c.806C>T	Verified	GTT	CCG	ACG	2	C	T
TCGA-29-1691	AC003101.1	ENST00000412403	p.V133V	c.399C>T	Unverified	TAC	GTC	CAA	3	C	T

TCGA-29-1691	AC003101.1_	ENST00000412403_v68	p.V133V	c.399C>T	Unverified	TAC	GTC	CAA	3	C	T
TCGA-29-1703	AC011537.1	ENST00000412610	p.R149C	c.445C>T	Unverified	TGC	CGC	CTG	1	C	T
TCGA-29-1703	AC011537.1_	ENST00000412610_v68	p.R149C	c.445C>T	Unverified	TGC	CGC	CTG	1	C	T
TCGA-61-1910	AC012100.1	ENST00000261854	p.P272L	c.815C>T	Unverified	ATA	CCA	TAT	2	C	T
TCGA-29-1783	AC013469_8	ENST00000408934	p.R53W	c.157C>T	Unverified	CCA	CGG	CTC	1	C	T
TCGA-23-1124	AC027369_8	ENST00000402751	p.L450L	c.1348C>T	Verified	TCC	CTG	ATA	1	C	T
TCGA-23-1120	ACACB	ENST00000377848	p.T1404I	c.4211C>T	Verified	CGC	ACC	TCC	2	C	T
TCGA-13-0919	ACAD10	ENST00000313698	p.A990V	c.2969C>T	Unverified	GCA	GCC	TTG	2	C	T
TCGA-04-1362	ACAD10	ENST00000313698	p.R939R	c.2817C>T	Verified	GCC	CGC	GTG	3	C	T
TCGA-24-1470	ACADVL	ENST00000356839	p.I258I	c.774C>T	Verified	GAC	ATC	TTC	3	C	T
TCGA-29-1698	ACAP1	ENST00000158762	p.H381H	c.1143C>T	Unverified	GGA	CAC	CTG	3	C	T
TCGA-09-0369	ACBD4	ENST00000398322	p.R67*	c.199C>T	Verified	GGA	CGA	TAT	1	C	T
TCGA-09-0369	ACBD4	ENST00000398322	p.R67*	c.199C>T	Verified	GGA	CGA	TAT	1	C	T
TCGA-23-1122	ACCN3	ENST00000349064	p.P259S	c.775C>T	Verified	TCC	CCG	GGC	1	C	T
TCGA-29-1768	ACER1	ENST00000301452	p.S10S	c.30C>T	Unverified	AGC	TCC	GAG	3	C	T
TCGA-29-1770	ACIN1	ENST00000262710	p.L529L	c.1587C>T	Unverified	CCC	CTC	CCT	3	C	T
TCGA-29-1691	ACOT12	ENST00000307624	p.F108F	c.324C>T	Unverified	GCT	TTC	TCC	3	C	T
TCGA-24-1850	ACP1_ENST(	ENST00000272067	p.N51N	c.153C>T	Unverified	TGG	AAC	GTG	3	C	T
TCGA-23-1124	ACP6	ENST00000369238	p.Y110Y	c.330C>T	Verified	CAA	TAC	CAT	3	C	T
TCGA-61-1904	ACRBP	ENST00000229243	p.Q293*	c.877C>T	Unverified	GCC	CAG	GAA	1	C	T
TCGA-24-1616	ACRC	ENST00000373695	p.D206D	c.618C>T	Unverified	TCC	GAC	GAC	3	C	T
TCGA-24-2262	ACSBG1	ENST00000258873	p.I540I	c.1620C>T	Unverified	GCC	ATC	GAC	3	C	T
TCGA-29-1783	ACSBG1	ENST00000258873	p.G552G	c.1656C>T	Unverified	GCT	GGC	CGC	3	C	T
TCGA-09-2049	ACSBG2	ENST00000252669	p.S338S	c.1014C>T	Verified	AAG	TCC	ATG	3	C	T
TCGA-20-1686	ACSL4	ENST00000469796	p.P418L	c.1253C>T	Unverified	gca	cct	ctt	2	C	T
TCGA-23-1809	ACTL7A	ENST00000333999	p.A41A	c.123C>T	Unverified	CGG	GCC	GTG	3	C	T
TCGA-24-2267	ACTL8	ENST00000375406	p.Q134*	c.400C>T	Verified	CTG	CAG	ATG	1	C	T
TCGA-04-1331	ACTRT1	ENST00000371124	p.R231C	c.691C>T	Verified	cta	cgc	aag	1	C	T
TCGA-25-2400	ADA	ENST00000372874	p.R253W	c.757C>T	Verified	ctg	cgg	cag	1	C	T
TCGA-29-1784	ADAM11	ENST00000200557	p.S145F	c.434C>T	Unverified	CAC	TCC	TTC	2	C	T
TCGA-29-1784	ADAM11_EI	ENST00000200557	p.S145F	c.434C>T	Unverified	CAC	TCC	TTC	2	C	T
TCGA-24-1417	ADAM15	ENST00000356955	p.L402F	c.1204C>T	Verified	gcc	ctc	ctg	1	C	T
TCGA-13-0920	ADAM19	NM_023038.2	p.P685S	c.2053C>T	Verified	aca	ccg	ggc	1	C	T
TCGA-23-1029	ADAM30	ENST00000369400	p.A252V	c.755C>T	Unverified	aag	gct	ctt	2	C	T

TCGA-61-1895	ADAM9	ENST00000487273	p.P790L	c.2369C>T	Unverified	agg	cca	cct	2	C	T
TCGA-61-2012	ADAMTS13	ENST00000371929	p.P557S	c.1669C>T	Verified	AGC	CCA	CGG	1	C	T
TCGA-24-1844	ADAMTS19	NM_133638.1	p.L605L	c.1813C>T	Unverified	aag	cta	gac	1	C	T
TCGA-04-1652	ADAMTS5	ENST00000284987	p.S566S	c.1698C>T	Unverified	TCA	AGC	CAT	3	C	T
TCGA-04-1652	ADAMTS5	ENST00000284987	p.S566S	c.1698C>T	Unverified	TCA	AGC	CAT	3	C	T
TCGA-13-0906	ADAMTS5	ENST00000284987	p.R614R	c.1842C>T	Verified	TAC	CGC	TCC	3	C	T
TCGA-29-1695	ADAMTS5	ENST00000284987	p.A458A	c.1374C>T	Unverified	TCA	GCC	ACC	3	C	T
TCGA-29-1695	ADAMTS5	ENST00000284987	p.C721C	c.2163C>T	Unverified	AAG	TGC	GGA	3	C	T
TCGA-20-1686	ADAMTS7	ENST00000388820	p.D512D	c.1536C>T	Unverified	GTG	GAC	GGC	3	C	T
TCGA-61-1904	ADAMTS8	ENST00000257359	p.C294C	c.882C>T	Unverified	TTC	TGC	AAC	3	C	T
TCGA-61-1904	ADAMTS8_E	ENST00000257359	p.C294C	c.882C>T	Unverified	TTC	TGC	AAC	3	C	T
TCGA-61-1904	ADAMTS8_E	ENST00000414575	p.C323C	c.969C>T	Unverified	GAG	TGC	AAG	3	C	T
TCGA-24-2267	ADAMTSL1	NM_139238.1	p.C49C	c.147C>T	Verified	acc	tgc	ggg	3	C	T
TCGA-24-2267	ADAMTSL1_	ENST00000380548	p.C49C	c.147C>T	Verified	ACC	TGC	GGG	3	C	T
TCGA-04-1343	ADAMTSL3	ENST00000286744	p.R958W	c.2872C>T	Verified	AAA	CGG	CTT	1	C	T
TCGA-20-1686	ADAMTSL4	ENST00000369038	p.R1060C	c.3178C>T	Unverified	TGC	CGC	TCT	1	C	T
TCGA-24-1850	ADARB2	ENST00000381312	p.T692M	c.2075C>T	Unverified	GAC	ACG	CCC	2	C	T
TCGA-30-1856	ADC	ENST00000373443	p.P374S	c.1120C>T	Unverified	CTG	CCG	CAA	1	C	T
TCGA-29-1776	ADCK3	NM_020247	p.D278D	c.834C>T	Unverified	gaa	gac	gcc	3	C	T
TCGA-29-1776	ADCK3_ENS	ENST00000366779	p.D557D	c.1671C>T	Unverified	GAA	GAC	GCC	3	C	T
TCGA-13-1510	ADCY3	ENST00000260600	p.P25S	c.73C>T	Unverified	CTG	CCC	TCC	1	C	T
TCGA-29-1762	ADCY3	ENST00000260600	p.T976I	c.2927C>T	Unverified	AAA	ACC	ATT	2	C	T
TCGA-23-2078	ADCY4	ENST00000310677	p.P847L	c.2540C>T	Unverified	CTC	CCT	GCA	2	C	T
TCGA-23-1114	ADCY9	ENST00000294016	p.N1280N	c.3840C>T	Unverified	GCC	AAC	CTG	3	C	T
TCGA-25-2392	ADD2	ENST00000355733	p.R420*	c.1258C>T	Verified	CTG	CGA	CAG	1	C	T
TCGA-13-0920	ADH1A	ENST00000209668	p.V84V	c.252C>T	Verified	aca	gtc	aaa	3	C	T
TCGA-24-1474	ADH1B	ENST00000305046	p.S194F	c.581C>T	Verified	GGC	TCT	ACC	2	C	T
TCGA-61-1738	ADIPOR1	ENST00000340990	p.G278G	c.834C>T	Unverified	AGT	GGC	GTC	3	C	T
TCGA-61-1738	ADIPOR1	ENST00000340990	p.G278G	c.834C>T	Unverified	AGT	GGC	GTC	3	C	T
TCGA-13-0791	ADNP2	ENST00000262198	p.I916I	c.2748C>T	Verified	TGC	ATC	CAC	3	C	T
TCGA-24-1847	ADNP2	ENST00000262198	p.L390L	c.1170C>T	Unverified	GTC	CTC	CCC	3	C	T
TCGA-23-1022	ADORA2B	ENST00000304222	p.L310F	c.928C>T	Verified	ctt	ctc	tgc	1	C	T
TCGA-29-1763	AEN	ENST00000332810	p.A201V	c.602C>T	Unverified	CAG	GCG	CTC	2	C	T
TCGA-13-0885	AFF3	ENST00000356421	p.D1117D	c.3351C>T	Verified	ATC	GAC	TAT	3	C	T

TCGA-24-2280	AFF4	ENST00000265343	p.S965F	c.2894C>T	Unverified	AAA	TCC	CCA	2	C	T
TCGA-23-1123	AFP	ENST00000395792	p.I255I	c.765C>T	Verified	GAA	ATC	CAG	3	C	T
TCGA-23-1123	AFP	ENST00000395792	p.I255I	c.765C>T	Verified	GAA	ATC	CAG	3	C	T
TCGA-13-1496	AGBL5	ENST00000360131	p.S664F	c.1991C>T	Verified	AAT	TCC	CCC	2	C	T
TCGA-13-0900	AHCYL1	ENST00000369799	p.R365W	c.1093C>T	Verified	ATC	CGG	CAA	1	C	T
TCGA-13-0795	AHCYL2	ENST00000325006	p.Q150*	c.448C>T	Verified	TCT	CAG	TCA	1	C	T
TCGA-24-2280	AHNAK	ENST00000378024	p.P3575S	c.10723C>T	Unverified	GGG	CCA	GAG	1	C	T
TCGA-24-0979	AHNAK	ENST00000378024	p.P2656L	c.7967C>T	Verified	GGC	CCA	GAT	2	C	T
TCGA-61-2094	AHNAK2_EN	ENST00000333244	p.S3955F	c.11864C>T	Unverified	CTG	TCC	CTG	2	C	T
TCGA-09-0369	AIM1	ENST00000369066	p.L613F	c.1837C>T	Verified	GAG	CTC	GCG	1	C	T
TCGA-09-0369	AIM1	ENST00000369066	p.L613F	c.1837C>T	Verified	GAG	CTC	GCG	1	C	T
TCGA-29-1763	AKAP13	ENST00000361243	p.T1888I	c.5663C>T	Unverified	gaa	acc	gct	2	C	T
TCGA-29-1763	AKAP13_EN	ENST00000394518	p.T1884I	c.5651C>T	Unverified	GAA	ACC	GCT	2	C	T
TCGA-30-1714	AKD1_ENST	ENST00000424296	p.T1810I	c.5429C>T	Unverified	GCA	ACT	TCT	2	C	T
TCGA-24-1470	AKR1C1	ENST00000380872	p.L257L	c.769C>T	Verified	GCC	CTG	CGC	1	C	T
TCGA-29-1711	AKR1C4	ENST00000380448	p.T127M	c.380C>T	Unverified	GAG	ACG	CCA	2	C	T
TCGA-29-1711	AKR1C4	ENST00000380448	p.T127M	c.380C>T	Unverified	GAG	ACG	CCA	2	C	T
TCGA-20-0990	AKR1D1	ENST00000242375	p.I21I	c.63C>T	Unverified	ATC	ATC	GGA	3	C	T
TCGA-04-1338	ALB	ENST00000295897	p.H312H	c.936C>T	Verified	TCC	CAC	TGC	3	C	T
TCGA-13-0923	ALDH3A2	ENST00000581518	p.P441L	c.1322C>T	Verified	tat	cct	ccc	2	C	T
TCGA-13-0923	ALG8	ENST00000299626	p.H160Y	c.478C>T	Verified	GAC	CAT	ATT	1	C	T
TCGA-04-1336	ALOX15B	ENST00000380183	p.A363V	c.1088C>T	Verified	aat	gcc	gag	2	C	T
TCGA-04-1331	ALOXE3	NM_021628.1	p.I148I	c.444C>T	Unverified	aag	atc	tat	3	C	T
TCGA-13-1481	ALPK1	ENST00000458497	p.A848A	c.2544C>T	Verified	GAT	GCC	TCC	3	C	T
TCGA-09-1665	ALPK2	NM_052947	p.A1230V	c.3689C>T	Verified	act	gct	gaa	2	C	T
TCGA-25-2398	ALPK2	NM_052947	p.A650A	c.1950C>T	Verified	ttg	gcc	ccc	3	C	T
TCGA-09-1665	ALPK2_ENST	ENST00000361673	p.A1869V	c.5606C>T	Verified	ACT	GCT	GAA	2	C	T
TCGA-25-2398	ALPK2_ENST	ENST00000361673	p.A1289A	c.3867C>T	Verified	TTG	GCC	CCC	3	C	T
TCGA-24-1469	ALPP	ENST00000392027	p.P4S	c.10C>T	Verified	GGG	CCC	TGC	1	C	T
TCGA-23-2077	AMDHD1	ENST00000266736	p.A302A	c.906C>T	Unverified	TCT	GCC	ATC	3	C	T
TCGA-29-1781	AMPD1	NM_000036.1	p.A412V	c.1235C>T	Unverified	ggt	gcg	gac	2	C	T
TCGA-24-2289	AMPD1	NM_000036.1	p.F637F	c.1911C>T	Unverified	gat	ttc	ctt	3	C	T
TCGA-29-1781	AMPD1_ENS	ENST00000520113	p.A445V	c.1334C>T	Unverified	GGT	GCG	GAC	2	C	T
TCGA-09-1665	AMPH	ENST00000356264	p.P249S	c.745C>T	Verified	GCG	CCC	AGT	1	C	T

TCGA-04-1337	ANGPTL2	ENST00000373425	p.V224V	c.672C>T	Unverified	CGG	GTC	TAC	3	C	T
TCGA-24-2289	ANGPTL5	ENST00000334289	p.C324C	c.972C>T	Unverified	AGC	TGC	AGT	3	C	T
TCGA-61-1738	ANK2	ENST00000357077	p.R1062C	c.3184C>T	Unverified	AGC	CGC	ATT	1	C	T
TCGA-61-1738	ANK2	ENST00000357077	p.R1062C	c.3184C>T	Unverified	AGC	CGC	ATT	1	C	T
TCGA-61-1914	ANK2	ENST00000357077	p.L1044F	c.3130C>T	Unverified	AAA	CTT	CAC	1	C	T
TCGA-61-2113	ANK2	ENST00000357077	p.H242Y	c.724C>T	Verified	GCA	CAT	TAC	1	C	T
TCGA-24-2289	ANK2	ENST00000357077	p.A702V	c.2105C>T	Unverified	CTT	GCA	GCC	2	C	T
TCGA-61-1725	ANKHD1	ENST00000360839	p.G2172G	c.6516C>T	Unverified	caa	ggc	cca	3	C	T
TCGA-13-0755	ANKHD1-EIF	ENST00000297183	p.C2579C	c.7737C>T	Verified	CAT	TGC	CCG	3	C	T
TCGA-61-1725	ANKHD1-EIF	ENST00000297183	p.G2172G	c.6516C>T	Unverified	CAA	GGC	CCA	3	C	T
TCGA-13-0807	ANKRD17	NM_032217.3	p.N2366N	c.7098C>T	Verified	gct	aac	ttt	3	C	T
TCGA-25-2400	ANKRD30A	ENST00000361713	p.I327I	c.981C>T	Verified	AAG	ATC	GCA	3	C	T
TCGA-13-0885	ANO2	ENST00000327087	p.D312D	c.936C>T	Verified	CAT	GAC	GGT	3	C	T
TCGA-04-1652	ANO2	ENST00000327087	p.N769N	c.2307C>T	Unverified	AAC	AAC	GTC	3	C	T
TCGA-04-1652	ANO2	ENST00000327087	p.N769N	c.2307C>T	Unverified	AAC	AAC	GTC	3	C	T
TCGA-04-1652	ANO2_ENST	ENST00000327087_v68	p.N769N	c.2307C>T	Unverified	AAC	AAC	GTC	3	C	T
TCGA-04-1652	ANO2_ENST	ENST00000327087_v68	p.N769N	c.2307C>T	Unverified	AAC	AAC	GTC	3	C	T
TCGA-13-1496	ANO4	ENST00000392979	p.T573I	c.1718C>T	Verified	TCC	ACA	TTT	2	C	T
TCGA-29-1775	ANO7	ENST00000274979	p.D111D	c.333C>T	Unverified	GCA	GAC	TTC	3	C	T
TCGA-10-0930	ANO8	ENST00000159087	p.D783D	c.2349C>T	Verified	AGC	GAC	GCC	3	C	T
TCGA-10-0930	ANO8	ENST00000159087	p.D783D	c.2349C>T	Verified	AGC	GAC	GCC	3	C	T
TCGA-13-0884	ANTXR2	ENST00000307333	p.P269L	c.806C>T	Verified	AAA	CCA	GTA	2	C	T
TCGA-24-1845	ANTXR2	ENST00000307333	p.L24L	c.72C>T	Unverified	GTG	CTC	AGC	3	C	T
TCGA-24-1845	ANTXR2_EN	ENST00000403729	p.L24L	c.72C>T	Unverified	GTG	CTC	AGC	3	C	T
TCGA-09-0369	AOC2	ENST00000253799	p.Q632*	c.1894C>T	Verified	ACC	CAG	AGA	1	C	T
TCGA-09-0369	AOC2	ENST00000253799	p.Q632*	c.1894C>T	Verified	ACC	CAG	AGA	1	C	T
TCGA-23-1123	AOC2	ENST00000253799	p.S311L	c.932C>T	Verified	TTC	TCG	CCC	2	C	T
TCGA-23-1123	AOC2	ENST00000253799	p.S311L	c.932C>T	Verified	TTC	TCG	CCC	2	C	T
TCGA-29-1702	AOC3	ENST00000308423	p.R329*	c.985C>T	Unverified	AGT	CGA	GTG	1	C	T
TCGA-13-1496	AP001011.2	ENST00000261598	p.H430Y	c.1288C>T	Verified	TAT	CAT	CCA	1	C	T
TCGA-13-1496	AP001011.3	ENST00000320876	p.H430Y	c.1288C>T	Verified	TAT	CAT	CCA	1	C	T
TCGA-09-1674	AP1G2	ENST00000397120	p.R40C	c.118C>T	Unverified	TTC	CGC	GAC	1	C	T
TCGA-13-0904	AP2A1	ENST00000359032	p.S179L	c.536C>T	Verified	GCC	TCG	CCT	2	C	T
TCGA-13-1510	AP3B2	ENST00000261722	p.C954C	c.2862C>T	Unverified	CTG	TGC	ACC	3	C	T

TCGA-29-1784	AP4E1	ENST00000261842	p.Q57*	c.169C>T	Unverified	ATC	CAG	CAG	1	C	T
TCGA-61-1725	APBA2	ENST00000561069	p.A57V	c.170C>T	Unverified	CCC	GCG	CCA	2	C	T
TCGA-29-1761	APOB	ENST00000233242	p.I2218I	c.6654C>T	Unverified	CAT	ATC	CGT	3	C	T
TCGA-61-1895	APOB	ENST00000233242	p.S242S	c.726C>T	Unverified	AGC	AGC	CAG	3	C	T
TCGA-13-0890	APOBEC3B	ENST00000333467	p.A295V	c.884C>T	Verified	cgt	gcg	ttc	2	C	T
TCGA-24-1470	APOBEC3D_	ENST00000216099	p.A301A	c.903C>T	Unverified	GTG	GCC	GAG	3	C	T
TCGA-13-1498	APTX	ENST00000309615	p.R43C	c.127C>T	Verified	GGG	CGT	GGC	1	C	T
TCGA-25-2391	AQR	ENST00000156471	p.I226I	c.678C>T	Unverified	CTC	ATC	CAG	3	C	T
TCGA-09-2056	ARAF	ENST00000377045	p.P561P	c.1683C>T	Verified	ttc	ccc	cag	3	C	T
TCGA-20-0990	ARHGAP19_	ENST00000358531	p.R18W	c.52C>T	Verified	GGC	CGG	AGT	1	C	T
TCGA-24-1435	ARHGAP30	ENST00000368013	p.V286V	c.858C>T	Verified	AAG	GTC	AGG	3	C	T
TCGA-25-1313	ARHGAP31	ENST00000264245	p.L310L	c.928C>T	Verified	AAC	CTG	GGA	1	C	T
TCGA-29-1699	ARHGAP36	NM_144967.2	p.R483W	c.1447C>T	Unverified	gcc	cgg	gct	1	C	T
TCGA-13-1408	ARHGAP6	ENST00000337414	p.R580W	c.1738C>T	Unverified	GCC	CGG	GCT	1	C	T
TCGA-09-1674	ARHGAP8	ENST00000389774	p.P144L	c.431C>T	Unverified	CAC	CCC	ACC	2	C	T
TCGA-25-2391	ARHGEF10L	ENST00000361221	p.G631G	c.1893C>T	Unverified	TCA	GGC	GCC	3	C	T
TCGA-04-1347	ARHGEF11	ENST00000368194	p.A515A	c.1545C>T	Verified	ttc	gcc	ctc	3	C	T
TCGA-25-1318	ARHGEF12	ENST00000397843	p.L1267L	c.3799C>T	Unverified	CAG	CTA	GGT	1	C	T
TCGA-25-1318	ARHGEF12	ENST00000397843	p.L1267L	c.3799C>T	Unverified	CAG	CTA	GGT	1	C	T
TCGA-29-1702	ARHGEF15	ENST00000361926	p.S69S	c.207C>T	Unverified	gca	tcc	ctc	3	C	T
TCGA-61-1740	ARHGEF2	ENST00000313695	p.I370I	c.1110C>T	Unverified	ctc	atc	agc	3	C	T
TCGA-29-1695	ARHGEF26	ENST00000356448	p.V703V	c.2109C>T	Unverified	AAC	GTC	AAT	3	C	T
TCGA-29-1695	ARHGEF26_	ENST00000356448	p.V703V	c.2109C>T	Unverified	AAC	GTC	AAT	3	C	T
TCGA-24-2280	ARHGEF4	ENST00000326016	p.H526H	c.1578C>T	Unverified	gac	cac	cag	3	C	T
TCGA-13-0760	ARID1A	ENST00000324856	p.Q1708*	c.5122C>T	Verified	agt	cag	ctc	1	C	T
TCGA-61-1998	ARID2	ENST00000334344	p.P1144S	c.3430C>T	Unverified	GTG	CCC	ATT	1	C	T
TCGA-04-1343	ARID2	ENST00000334344	p.S742F	c.2225C>T	Verified	AGT	TCT	GTT	2	C	T
TCGA-13-0920	ARMC10	ENST00000323716	p.H271Y	c.811C>T	Verified	AGC	CAC	GTA	1	C	T
TCGA-09-2050	ARMCX1	ENST00000372829	p.D32D	c.96C>T	Verified	aga	gac	gag	3	C	T
TCGA-13-1496	ARMCX1	ENST00000372829	p.N277N	c.831C>T	Verified	gtg	aac	gca	3	C	T
TCGA-25-2393	ARPP21	ENST00000187397	p.P528L	c.1583C>T	Unverified	TCC	CCG	CAG	2	C	T
TCGA-29-1701	ARPP21	ENST00000187397	p.T651M	c.1952C>T	Unverified	ACC	ACG	CAA	2	C	T
TCGA-04-1651	ARSB	ENST00000264914	p.L502L	c.1504C>T	Unverified	CGC	CTA	CAG	1	C	T
TCGA-04-1651	ARSB	ENST00000264914	p.L502L	c.1504C>T	Unverified	CGC	CTA	CAG	1	C	T

TCGA-29-1703	ARSI	ENST00000328668	p.S378S	c.1134C>T	Unverified	ATC	AGC	GAG	3	C	T
TCGA-13-1501	ART5	ENST00000397068	p.R80*	c.238C>T	Verified	CGT	CGA	GGG	1	C	T
TCGA-13-1501	ART5	ENST00000397068	p.R80*	c.238C>T	Verified	CGT	CGA	GGG	1	C	T
TCGA-13-0913	ASCC3	ENST00000369162	p.A348V	c.1043C>T	Verified	ATT	GCC	AGA	2	C	T
TCGA-25-1313	ASF1B	ENST00000263382	p.P130S	c.388C>T	Unverified	AAG	CCA	GAT	1	C	T
TCGA-04-1542	ASNA1	ENST00000357332	p.R189W	c.565C>T	Verified	GGC	CGG	CTT	1	C	T
TCGA-24-2267	ASPHD2	ENST00000382701	p.T22T	c.66C>T	Verified	GCC	ACC	GGC	3	C	T
TCGA-20-1686	ASPM	ENST00000367409	p.A1719V	c.5156C>T	Unverified	GCT	GCA	CAA	2	C	T
TCGA-20-1686	ASPN	ENST00000375544	p.R84*	c.250C>T	Unverified	TCA	CGA	GTT	1	C	T
TCGA-29-1690	ASXL1	ENST00000375687	p.A1312V	c.3935C>T	Unverified	GCT	GCA	ACC	2	C	T
TCGA-29-1769	ASXL3_ENST	ENST00000269197	p.T93M	c.278C>T	Unverified	GGC	ACG	TTG	2	C	T
TCGA-61-1733	ASXL3_ENST	ENST00000269197	p.T563I	c.1688C>T	Unverified	GAA	ACT	GCA	2	C	T
TCGA-61-1733	ASXL3_ENST	ENST00000269197	p.T563I	c.1688C>T	Unverified	GAA	ACT	GCA	2	C	T
TCGA-29-1711	ATG7	ENST00000354449	p.A57V	c.170C>T	Unverified	TCT	GCT	GGG	2	C	T
TCGA-29-1711	ATG7	ENST00000354449	p.A57V	c.170C>T	Unverified	TCT	GCT	GGG	2	C	T
TCGA-29-1693	ATM	ENST00000278616	p.R451C	c.1351C>T	Unverified	GAA	CGT	ACA	1	C	T
TCGA-29-1693	ATM_ENST0	ENST00000278616	p.R451C	c.1351C>T	Unverified	GAA	CGT	ACA	1	C	T
TCGA-10-0930	ATN1	ENST00000396684	p.A1071V	c.3212C>T	Verified	CAT	GCA	GCC	2	C	T
TCGA-10-0930	ATN1	ENST00000396684	p.A1071V	c.3212C>T	Verified	CAT	GCA	GCC	2	C	T
TCGA-23-1809	ATP10B	ENST00000327245	p.S667F	c.2000C>T	Unverified	GCA	TCT	GTG	2	C	T
TCGA-04-1336	ATP10B	ENST00000327245	p.A711A	c.2133C>T	Verified	CTG	GCC	AGG	3	C	T
TCGA-30-1718	ATP12A	ENST00000381946	p.A345A	c.1035C>T	Unverified	GTG	GCC	AAT	3	C	T
TCGA-04-1361	ATP13A1	ENST00000357324	p.Q1086*	c.3256C>T	Verified	GAG	CAG	TTC	1	C	T
TCGA-04-1361	ATP13A1	ENST00000357324	p.Q1086*	c.3256C>T	Verified	GAG	CAG	TTC	1	C	T
TCGA-24-1103	ATP1A1	ENST00000295598	p.R385W	c.1153C>T	Verified	AAC	CGG	ATG	1	C	T
TCGA-61-1740	ATP1A4	ENST00000368081	p.A938V	c.2813C>T	Unverified	TGG	GCG	GAT	2	C	T
TCGA-24-1431	ATP1B1	ENST00000367816	p.T67I	c.200C>T	Verified	CCC	ACA	TAT	2	C	T
TCGA-29-1703	ATP2A1	ENST00000357084	p.D707D	c.2121C>T	Unverified	AAT	GAC	GCC	3	C	T
TCGA-23-1031	ATP2B2	ENST00000383800	p.R59C	c.175C>T	Verified	CGG	CGC	CTC	1	C	T
TCGA-25-2393	ATP2B3	ENST00000359149	p.S163S	c.489C>T	Unverified	CTG	TCC	GTC	3	C	T
TCGA-25-2393	ATP2B3_ENS	ENST00000349466	p.S163S	c.489C>T	Unverified	CTG	TCC	GTC	3	C	T
TCGA-25-2393	ATP2B3_ENS	ENST00000370186	p.S163S	c.489C>T	Unverified	CTG	TCC	GTC	3	C	T
TCGA-30-1856	ATP4A	ENST00000262623	p.R95W	c.283C>T	Unverified	CCA	CGG	GGC	1	C	T
TCGA-24-1849	ATP5C1	ENST00000356708	p.R4C	c.10C>T	Unverified	TCT	CGC	GCG	1	C	T

TCGA-29-1764	ATP5E	ENST00000395659	p.S16F	c.47C>T	Unverified	TAC	TCC	CAG	2	C	T
TCGA-23-1110	ATP7B	ENST00000242839	p.R1411W	c.4231C>T	Verified	TGG	CGG	GAC	1	C	T
TCGA-24-1616	ATP8A2	ENST00000381655	p.P944S	c.2830C>T	Verified	TTT	CCC	CAG	1	C	T
TCGA-04-1337	ATP8B3	ENST00000539485	p.H1250Y	c.3748C>T	Unverified	CCT	CAT	GTA	1	C	T
TCGA-13-0795	ATP9A	ENST00000338821	p.S184L	c.551C>T	Verified	GGG	TCA	TGC	2	C	T
TCGA-61-2008	ATRNL1	ENST00000355044	p.L603L	c.1809C>T	Verified	GTA	CTC	CTT	3	C	T
TCGA-13-0883	ATXN7L2	ENST00000369870	p.H103Y	c.307C>T	Verified	AGA	CAT	GGG	1	C	T
TCGA-29-1770	AUP1	ENST00000377526	p.V315V	c.945C>T	Unverified	GGT	GTC	ATC	3	C	T
TCGA-24-2262	AUTS2	ENST00000342771	p.P596P	c.1788C>T	Verified	CCA	CCC	ACT	3	C	T
TCGA-29-1777	AVPI1	ENST00000370626	p.R130C	c.388C>T	Unverified	ATC	CGC	CGG	1	C	T
TCGA-13-0890	AWAT1	XM_088691.4	p.T165M	c.494C>T	Verified	att	acg	atc	2	C	T
TCGA-23-1111	AXDND1	ENST00000367618	p.P10L	c.29C>T	Unverified	ACC	CCG	CTA	2	C	T
TCGA-29-1775	AZGP1	ENST00000292401	p.A46V	c.137C>T	Unverified	CCC	GCG	TTT	2	C	T
TCGA-04-1652	AZI1	ENST00000374782	p.P133P	c.399C>T	Unverified	CAG	CCC	CGG	3	C	T
TCGA-04-1652	AZI1	ENST00000374782	p.P133P	c.399C>T	Unverified	CAG	CCC	CGG	3	C	T
TCGA-04-1652	AZI1_ENST0	ENST00000450824	p.P133P	c.399C>T	Unverified	CAG	CCC	CGG	3	C	T
TCGA-04-1652	AZI1_ENST0	ENST00000450824	p.P133P	c.399C>T	Unverified	CAG	CCC	CGG	3	C	T
TCGA-23-1031	AZI2	ENST00000479665	p.S83S	c.249C>T	Verified	AGT	TCC	GTG	3	C	T
TCGA-24-1469	B3GALTL	ENST00000343307	p.H323Y	c.967C>T	Verified	GGT	CAT	TGT	1	C	T
TCGA-13-0923	B3GAT3	ENST00000265471	p.V190V	c.570C>T	Unverified	GTC	GTC	TAC	3	C	T
TCGA-29-1775	B4GALNT2	ENST00000300404	p.Q493*	c.1477C>T	Unverified	CTG	CAA	CGA	1	C	T
TCGA-23-1124	B7	ENST00000323702	p.P300S	c.898C>T	Verified	GTG	CCG	GTC	1	C	T
TCGA-24-1843	BACH2	ENST00000257749	p.Y528Y	c.1584C>T	Unverified	TCC	TAC	GCG	3	C	T
TCGA-24-1843	BACH2_ENS	ENST00000257749	p.Y528Y	c.1584C>T	Unverified	TCC	TAC	GCG	3	C	T
TCGA-13-0760	BAG3	ENST00000369085	p.T285M	c.854C>T	Verified	AGC	ACG	CCA	2	C	T
TCGA-23-1114	BAHCC1	ENST00000436173	p.F1829F	c.5487C>T	Unverified	AGC	TTC	GCC	3	C	T
TCGA-20-0991	BAI1	NM_001702.1	p.R865C	c.2593C>T	Verified	ctg	cgc	aca	1	C	T
TCGA-29-1701	BAI2	ENST00000373658	p.T507M	c.1520C>T	Unverified	GGC	ACG	CAG	2	C	T
TCGA-23-1809	BAIAP3	ENST00000324385	p.P81P	c.243C>T	Unverified	AAA	CCC	GGG	3	C	T
TCGA-24-1470	BAK1	ENST00000374467	p.Y38Y	c.114C>T	Verified	agc	tac	gtt	3	C	T
TCGA-13-0884	BANK1	ENST00000322953	p.T94I	c.281C>T	Verified	CTA	ACT	CCA	2	C	T
TCGA-13-0714	BAP1	ENST00000460680	p.R227C	c.679C>T	Verified	ATC	CGC	TTC	1	C	T
TCGA-29-1766	BASP1	ENST00000322611	p.G71G	c.213C>T	Unverified	GAG	GGC	GAG	3	C	T
TCGA-29-1695	BAT2D1	ENST00000392078	p.R2825W	c.8473C>T	Unverified	CAA	CGG	TTC	1	C	T

TCGA-61-1899	BAT2D1	ENST00000392078	p.P659L	c.1976C>T	Unverified	CGG	CCG	GCT	2	C	T
TCGA-29-1695	BAT2D1_EN	ENST00000392078	p.R2825W	c.8473C>T	Unverified	CAA	CGG	TTC	1	C	T
TCGA-61-1899	BAT2D1_EN	ENST00000392078	p.P659L	c.1976C>T	Unverified	CGG	CCG	GCT	2	C	T
TCGA-61-1915	BBS2	ENST00000245157	p.D636D	c.1908C>T	Unverified	AGG	GAC	ATG	3	C	T
TCGA-23-1122	BBX	ENST00000415149	p.P705L	c.2114C>T	Verified	AGT	CCT	GTT	2	C	T
TCGA-13-0885	BCL3	ENST00000164227	p.R153W	c.457C>T	Verified	CAC	CGG	CTG	1	C	T
TCGA-04-1542	BCL9L	NM_182557.1	p.R680W	c.2038C>T	Verified	ctg	cgg	cac	1	C	T
TCGA-23-1022	BCOR	ENST00000397354	p.H1333H	c.3999C>T	Verified	AAA	CAC	TTG	3	C	T
TCGA-29-1770	BCORL1	ENST00000218147	p.P901L	c.2702C>T	Unverified	gac	cct	gtt	2	C	T
TCGA-24-1431	BDKRB1	ENST00000216629	p.A250V	c.749C>T	Verified	ACA	GCG	CTG	2	C	T
TCGA-29-1770	BDKRB2	ENST00000554311	p.L159L	c.475C>T	Unverified	GCC	CTG	GTG	1	C	T
TCGA-25-1326	BEST2	ENST00000042931	p.Q238*	c.712C>T	Verified	ACG	CAG	GTG	1	C	T
TCGA-04-1338	BIRC2	ENST00000227758	p.S109S	c.327C>T	Verified	cct	agc	tgt	3	C	T
TCGA-25-1318	BIRC6	ENST00000421745	p.T4672M	c.14015C>T	Unverified	AAC	ACG	TGG	2	C	T
TCGA-25-1318	BIRC6	ENST00000421745	p.T4672M	c.14015C>T	Unverified	AAC	ACG	TGG	2	C	T
TCGA-04-1356	BMP10	ENST00000295379	p.P396S	c.1186C>T	Verified	GAG	CCC	ATC	1	C	T
TCGA-61-2113	BMP3	ENST00000282701	p.A188A	c.564C>T	Verified	ATG	GCC	AAA	3	C	T
TCGA-29-1701	BMP7	ENST00000395863	p.H384H	c.1152C>T	Unverified	gtc	cac	ttc	3	C	T
TCGA-04-1542	BNC1	ENST00000345382	p.R107C	c.319C>T	Verified	GTT	CGC	CTA	1	C	T
TCGA-13-0795	BNIP1	ENST00000231668	p.V9V	c.27C>T	Verified	CAC	GTC	CGG	3	C	T
TCGA-04-1343	BOD1	ENST00000311086	p.A81V	c.242C>T	Verified	CCA	GCT	TAC	2	C	T
TCGA-24-1847	BOD1L	ENST00000040738	p.Q137*	c.409C>T	Unverified	TCT	CAG	GTT	1	C	T
TCGA-13-0885	BPI	ENST00000262865	p.V22V	c.66C>T	Verified	CTG	GTC	GCC	3	C	T
TCGA-24-1431	BPI	ENST00000262865	p.S396S	c.1188C>T	Verified	GGT	TCC	ATG	3	C	T
TCGA-23-1120	BPIL2	ENST00000397452	p.R408C	c.1222C>T	Verified	TTC	CGC	CTT	1	C	T
TCGA-24-1474	BPIL3	ENST00000349552	p.A295V	c.884C>T	Verified	CTG	GCT	CGC	2	C	T
TCGA-23-1122	BRPF3	ENST00000357641	p.G997G	c.2991C>T	Verified	ACA	GGC	ATG	3	C	T
TCGA-61-1899	BRWD1	ENST00000342449	p.S145F	c.434C>T	Unverified	GGT	TCC	CCA	2	C	T
TCGA-61-1899	BRWD1_ENS	ENST00000333229	p.S145F	c.434C>T	Unverified	GGT	TCC	CCA	2	C	T
TCGA-04-1342	BSN	ENST00000296452	p.P533L	c.1598C>T	Unverified	CCG	CCG	CCC	2	C	T
TCGA-61-2094	BSND	ENST00000371265	p.P193S	c.577C>T	Unverified	GCC	CCC	TTG	1	C	T
TCGA-20-0990	BTBD11	ENST00000280758	p.A775V	c.2324C>T	Verified	CTG	GCG	GAG	2	C	T
TCGA-24-2288	BTBD7	ENST00000334746	p.R963R	c.2889C>T	Unverified	AGA	CGC	ACC	3	C	T
TCGA-29-1701	BTBD9	ENST00000314100	p.R31W	c.91C>T	Unverified	GGG	CGG	GCA	1	C	T

TCGA-29-1701	BTBD9_ENS	ENST00000403056	p.R99W	c.295C>T	Unverified	GGG	CGG	GCA	1	C	T
TCGA-13-1489	BTK	ENST00000308731	p.S592F	c.1775C>T	Verified	TAC	TCC	CTG	2	C	T
TCGA-61-1907	BTK	ENST00000308731	p.Y134Y	c.402C>T	Unverified	CGG	TAC	AAC	3	C	T
TCGA-23-1111	BTN2A3	ENST00000297020	p.F337F	c.1011C>T	Unverified	AGA	TTC	GAC	3	C	T
TCGA-09-2056	BTNL9	ENST00000327705	p.A457V	c.1370C>T	Verified	GAG	GCC	GGA	2	C	T
TCGA-13-0887	BTRC	ENST00000370187	p.P66S	c.196C>T	Verified	ccc	cct	agg	1	C	T
TCGA-23-1117	C10orf68	ENST00000302316	p.Q150*	c.448C>T	Verified	GTG	CAA	TTA	1	C	T
TCGA-23-1117	C10orf68	ENST00000302316	p.Q150*	c.448C>T	Verified	GTG	CAA	TTA	1	C	T
TCGA-13-1507	C11orf63	ENST00000227349	p.H762H	c.2286C>T	Verified	AGA	CAC	GAA	3	C	T
TCGA-61-1733	C11orf63	ENST00000227349	p.L163L	c.489C>T	Unverified	CCC	CTC	TAC	3	C	T
TCGA-61-1733	C11orf63	ENST00000227349	p.L163L	c.489C>T	Unverified	CCC	CTC	TAC	3	C	T
TCGA-30-1856	C11orf67	ENST00000526415	p.G68G	c.204C>T	Unverified	ATT	GGC	CGA	3	C	T
TCGA-30-1891	C11orf70	ENST00000260246	p.P98L	c.293C>T	Unverified	GAT	CCA	TTT	2	C	T
TCGA-09-2044	C11orf82	ENST00000430323	p.S60F	c.179C>T	Verified	CTT	TCC	TTA	2	C	T
TCGA-24-1463	C12orf42	ENST00000548883	p.A281V	c.842C>T	Unverified	ATG	GCG	CCG	2	C	T
TCGA-13-0887	C12orf42	ENST00000548883	p.A213A	c.639C>T	Verified	TCT	GCC	GCC	3	C	T
TCGA-04-1331	C12orf49	ENST00000261318	p.G123G	c.369C>T	Verified	AAC	GGC	TGC	3	C	T
TCGA-13-0916	C12orf50	ENST00000298699	p.Q53*	c.157C>T	Verified	ATT	CAG	GAA	1	C	T
TCGA-61-1904	C12orf51_EI	ENST00000377560_v62	p.S1388S	c.4164C>T	Unverified	AGC	AGC	TCC	3	C	T
TCGA-13-0885	C12orf59	ENST00000298530	p.S12S	c.36C>T	Verified	TCA	TCC	TGT	3	C	T
TCGA-13-0904	C12orf66	ENST00000398055	p.T92T	c.276C>T	Verified	CGC	ACC	ATC	3	C	T
TCGA-61-1899	C14orf37	ENST00000267485	p.S20S	c.60C>T	Unverified	TTC	AGC	GTT	3	C	T
TCGA-13-0883	C14orf43	ENST00000286523	p.R322C	c.964C>T	Verified	CTG	CGC	AAG	1	C	T
TCGA-24-2289	C15orf2	ENST00000329468	p.A1084V	c.3251C>T	Unverified	TCT	GCT	GCC	2	C	T
TCGA-13-0923	C15orf32	ENST00000333334	p.H68Y	c.202C>T	Verified	TTG	CAC	CCA	1	C	T
TCGA-29-1785	C15orf32	ENST00000333334	p.P104L	c.311C>T	Unverified	ACA	CCA	TCT	2	C	T
TCGA-29-1785	C15orf32	ENST00000333334	p.P104L	c.311C>T	Unverified	ACA	CCA	TCT	2	C	T
TCGA-24-1435	C15orf44	ENST00000395644	p.S22S	c.66C>T	Verified	GGG	TCC	GAG	3	C	T
TCGA-13-0762	C16orf89	ENST00000350219	p.S58S	c.174C>T	Verified	TGG	TCC	TCC	3	C	T
TCGA-13-0762	C16orf89	ENST00000350219	p.S58S	c.174C>T	Verified	TGG	TCC	TCC	3	C	T
TCGA-23-1031	C17orf39	ENST00000268719	p.Y230Y	c.690C>T	Unverified	GAC	TAC	GTC	3	C	T
TCGA-24-2262	C19orf51	ENST00000391720	p.P68S	c.202C>T	Unverified	TCC	CCG	GCG	1	C	T
TCGA-23-1031	C19orf55	ENST00000396908	p.H184H	c.552C>T	Verified	CTC	CAC	ACA	3	C	T
TCGA-13-0893	C1orf162	ENST00000343534	p.A121V	c.362C>T	Verified	TAT	GCC	AGC	2	C	T

TCGA-24-1845	C1orf173	ENST00000326665	p.L16F	c.46C>T	Unverified	AGC	CTT	ATG	1	C	T
TCGA-30-1856	C1orf174	ENST00000361605	p.P142L	c.425C>T	Unverified	GTG	CCA	AAA	2	C	T
TCGA-25-1313	C1orf201	ENST00000003583	p.P142L	c.425C>T	Verified	CCT	CCC	CCA	2	C	T
TCGA-04-1338	C1orf38	ENST00000373925	p.T101I	c.302C>T	Verified	ACA	ACT	CAG	2	C	T
TCGA-20-0991	C1orf85	ENST00000362007	p.P104P	c.312C>T	Verified	GAG	CCC	GAT	3	C	T
TCGA-29-1691	C1QTNF9	ENST00000382071	p.D46D	c.138C>T	Unverified	CGA	GAC	GGA	3	C	T
TCGA-29-1691	C1QTNF9B	ENST00000382140	p.D46D	c.138C>T	Unverified	CGA	GAC	GGA	3	C	T
TCGA-29-1770	C20orf112	ENST00000359676	p.S105F	c.314C>T	Unverified	GCC	TCC	TAC	2	C	T
TCGA-24-1850	C22orf15	ENST00000336186	p.G16G	c.48C>T	Unverified	AGA	GGC	AGA	3	C	T
TCGA-13-0791	C2orf3	ENST00000321027	p.Q229*	c.685C>T	Verified	CAA	CAG	CAA	1	C	T
TCGA-25-1313	C3orf15	ENST00000273390	p.A207V	c.620C>T	Verified	TCT	GCA	GAA	2	C	T
TCGA-25-2391	C3orf25	ENST00000505956	p.P507L	c.1520C>T	Unverified	TGG	CCG	GGT	2	C	T
TCGA-23-1022	C3orf25	ENST00000505956	p.P24P	c.72C>T	Verified	ACT	CCC	ATC	3	C	T
TCGA-24-1423	C3orf54	ENST00000333323	p.R236W	c.706C>T	Unverified	CGC	CGG	CTG	1	C	T
TCGA-13-0807	C4orf27	ENST00000393381	p.T207T	c.621C>T	Verified	AGA	ACC	GTG	3	C	T
TCGA-24-1469	C5AR1	ENST00000355085	p.T29I	c.86C>T	Verified	AAA	ACT	TCT	2	C	T
TCGA-30-1857	C6orf105	ENST00000414691	p.F197F	c.591C>T	Unverified	TTC	TTC	TCT	3	C	T
TCGA-23-1123	C6orf146	ENST00000274673	p.P152S	c.454C>T	Verified	TGG	CCC	TAT	1	C	T
TCGA-23-1123	C6orf146	ENST00000274673	p.P152S	c.454C>T	Verified	TGG	CCC	TAT	1	C	T
TCGA-24-2262	C6orf222	ENST00000437635	p.R483W	c.1447C>T	Unverified	CAT	CGG	CCC	1	C	T
TCGA-30-1714	C6orf224	ENST00000321784	p.T209I	c.626C>T	Unverified	GCA	ACT	TCT	2	C	T
TCGA-13-0893	C7_ENST000	ENST00000313164	p.Y719Y	c.2157C>T	Verified	CCC	TAC	GAA	3	C	T
TCGA-13-0887	C7orf57	ENST00000348904	p.S39F	c.116C>T	Verified	GCG	TCC	CAG	2	C	T
TCGA-23-1114	C7orf62	ENST00000297203	p.V5V	c.15C>T	Unverified	TCG	GTC	CAT	3	C	T
TCGA-13-0762	C8orf4	ENST00000315792	p.H23Y	c.67C>T	Verified	ATC	CAT	GGC	1	C	T
TCGA-13-0762	C8orf4	ENST00000315792	p.H23Y	c.67C>T	Verified	ATC	CAT	GGC	1	C	T
TCGA-25-1326	C9orf106	ENST00000316786	p.L170L	c.510C>T	Unverified	GCC	CTC	AGA	3	C	T
TCGA-29-1698	C9orf114	ENST00000361256	p.P80L	c.239C>T	Unverified	CTG	CCG	GGC	2	C	T
TCGA-23-1114	C9orf125	ENST00000374848	p.A75V	c.224C>T	Unverified	GAG	GCT	GCC	2	C	T
TCGA-61-1998	C9orf125	ENST00000374848	p.T5T	c.15C>T	Unverified	TCA	ACC	TCT	3	C	T
TCGA-09-1674	C9orf131	ENST00000312292	p.S719S	c.2157C>T	Unverified	CCC	AGC	CCT	3	C	T
TCGA-23-1124	C9orf24	ENST00000297623	p.R129W	c.385C>T	Verified	CCT	CGG	CTG	1	C	T
TCGA-13-1512	C9orf41	ENST00000376834	p.S160F	c.479C>T	Verified	AAA	TCC	ACG	2	C	T
TCGA-30-1714	C9orf72	ENST00000380003	p.G390G	c.1170C>T	Unverified	CCT	GGC	TTA	3	C	T

TCGA-23-1123	C9orf79	ENST00000325643	p.H1207H	c.3621C>T	Verified	GCC	CAC	AGG	3	C	T
TCGA-23-1123	C9orf79	ENST00000325643	p.H1207H	c.3621C>T	Verified	GCC	CAC	AGG	3	C	T
TCGA-13-1497	C9orf84	ENST00000394779	p.A421V	c.1262C>T	Verified	GGA	GCA	GCA	2	C	T
TCGA-24-1846	CAB39_ENST	ENST00000258418	p.Q289*	c.865C>T	Unverified	ACG	CAG	CCC	1	C	T
TCGA-29-1785	CABP4	ENST00000325656	p.P242S	c.724C>T	Unverified	GAG	CCG	CTG	1	C	T
TCGA-29-1785	CABP4	ENST00000325656	p.P242S	c.724C>T	Unverified	GAG	CCG	CTG	1	C	T
TCGA-61-2102	CACNA1C	ENST00000402845	p.N1547N	c.4641C>T	Verified	ATC	AAC	CTC	3	C	T
TCGA-61-1904	CACNA1D	ENST00000288139	p.R2093C	c.6277C>T	Unverified	GGA	CGC	TAT	1	C	T
TCGA-24-0979	CACNA1H_E	ENST00000358590	p.I359I	c.1077C>T	Unverified	GCC	ATC	AAC	3	C	T
TCGA-13-1498	CACNA1S	ENST00000362061	p.L202L	c.604C>T	Verified	CTG	CTG	GTC	1	C	T
TCGA-29-1699	CACNA1S	ENST00000362061	p.S525S	c.1575C>T	Unverified	ATC	TCC	GTG	3	C	T
TCGA-23-1110	CACNG7	ENST00000391767	p.S244S	c.732C>T	Verified	CCC	TCC	GAC	3	C	T
TCGA-09-1665	CADM3	ENST00000368124	p.T229I	c.686C>T	Verified	GTG	ACA	TTC	2	C	T
TCGA-13-0762	CADPS2	ENST00000449022	p.N265N	c.795C>T	Verified	GAT	AAC	GCA	3	C	T
TCGA-13-0762	CADPS2	ENST00000449022	p.N265N	c.795C>T	Verified	GAT	AAC	GCA	3	C	T
TCGA-24-2024	CAGE1_ENST	ENST00000338150	p.Q456*	c.1366C>T	Verified	CTA	CAA	CAA	1	C	T
TCGA-13-1496	CALCR	ENST00000394441	p.R45*	c.133C>T	Verified	GGA	CGA	AAG	1	C	T
TCGA-24-2035	CAMSAP1	ENST00000389532	p.H934Y	c.2800C>T	Verified	GAG	CAC	TTT	1	C	T
TCGA-24-1563	CAMTA1	ENST00000303635	p.S44S	c.132C>T	Verified	AAC	AGC	AAT	3	C	T
TCGA-25-1326	CAPN11	ENST00000398776	p.Q450*	c.1348C>T	Verified	ATG	CAG	AAG	1	C	T
TCGA-30-1855	CAPN9	ENST00000354537	p.P484S	c.1450C>T	Unverified	AAG	CCA	ACT	1	C	T
TCGA-30-1855	CAPN9_ENST	ENST00000271971	p.P510S	c.1528C>T	Unverified	AAG	CCA	ACT	1	C	T
TCGA-29-2427	CARD11	ENST00000396946	p.I573I	c.1719C>T	Verified	TCC	ATC	GTC	3	C	T
TCGA-09-1674	CARD14	ENST00000573882	p.P506L	c.1517C>T	Unverified	ATC	CCG	GAG	2	C	T
TCGA-61-2012	CARD14	ENST00000573882	p.I586I	c.1758C>T	Verified	GTC	ATC	GGC	3	C	T
TCGA-09-1674	CARD14_EN	ENST00000309710	p.P269L	c.806C>T	Unverified	ATC	CCG	GAG	2	C	T
TCGA-61-1907	CARKD	ENST00000309957	p.A193A	c.579C>T	Unverified	CCG	GCC	CTC	3	C	T
TCGA-61-2009	CARTPT	ENST00000296777	p.S42F	c.125C>T	Verified	TAC	TCT	GCC	2	C	T
TCGA-23-1032	CASKIN1	ENST00000343516	p.S366L	c.1097C>T	Unverified	TCA	TCG	GGA	2	C	T
TCGA-23-1032	CASKIN1_EN	ENST00000382453	p.S195L	c.584C>T	Unverified	TCA	TCG	GGA	2	C	T
TCGA-29-1699	CASP8	ENST00000264275	p.P451L	c.1352C>T	Unverified	TGT	CCT	CGA	2	C	T
TCGA-29-1699	CASP8_ENST	ENST00000264275	p.P451L	c.1352C>T	Unverified	TGT	CCT	CGA	2	C	T
TCGA-29-1699	CASP8_ENST	ENST00000358485	p.P493L	c.1478C>T	Unverified	TGT	CCT	CGA	2	C	T
TCGA-09-1665	CASR	ENST00000296154	p.R1041W	c.3121C>T	Verified	CAG	CGG	CCA	1	C	T

TCGA-24-0975	CASZ1	ENST00000377022	p.F1280F	c.3840C>T	Unverified	TAC	TTC	ACC	3	C	T
TCGA-61-1906	CATSPER1	ENST00000312106	p.N4N	c.12C>T	Unverified	CAA	AAC	TCA	3	C	T
TCGA-61-1906	CATSPER1	ENST00000312106	p.N4N	c.12C>T	Unverified	CAA	AAC	TCA	3	C	T
TCGA-23-1031	CATSPERG	ENST00000312265	p.S768F	c.2303C>T	Verified	CCG	TCT	CTG	2	C	T
TCGA-61-2012	CBLL1	ENST00000440859	p.Q279*	c.835C>T	Verified	AGT	CAG	GAA	1	C	T
TCGA-23-1022	CBLL1	ENST00000440859	p.L30L	c.90C>T	Verified	AAG	CTC	ATC	3	C	T
TCGA-13-0905	CC2D1B	ENST00000371586	p.R534W	c.1600C>T	Verified	GTG	CGG	GAG	1	C	T
TCGA-61-2102	CCBL1	ENST00000302586	p.R174C	c.520C>T	Verified	TCA	CGC	ACC	1	C	T
TCGA-13-0893	CCDC102B	ENST00000360242	p.L426F	c.1276C>T	Verified	AAC	CTT	CAA	1	C	T
TCGA-23-1110	CCDC113	ENST00000219299	p.S6F	c.17C>T	Unverified	GAG	TCC	GAG	2	C	T
TCGA-24-1844	CCDC129	ENST00000407970	p.I643I	c.1929C>T	Unverified	TGT	ATC	CCC	3	C	T
TCGA-24-2290	CCDC136	ENST00000397697	p.R354W	c.1060C>T	Verified	CTT	CGG	TTT	1	C	T
TCGA-24-2290	CCDC136	ENST00000397697	p.R354W	c.1060C>T	Verified	CTT	CGG	TTT	1	C	T
TCGA-61-1907	CCDC137	ENST00000329214	p.T93I	c.278C>T	Unverified	GTG	ACC	TTC	2	C	T
TCGA-25-2392	CCDC141	ENST00000343876	p.P875S	c.2623C>T	Verified	AAG	CCA	TAA	1	C	T
TCGA-04-1542	CCDC144B	ENST00000360524	p.C240C	c.720C>T	Verified	GGA	TGC	GAA	3	C	T
TCGA-61-1910	CCDC15	ENST00000344762	p.Q707*	c.2119C>T	Unverified	GAC	CAA	GAC	1	C	T
TCGA-61-1910	CCDC15_EN!	ENST00000344762	p.Q707*	c.2119C>T	Unverified	GAC	CAA	GAC	1	C	T
TCGA-23-1120	CCDC155	ENST00000447857	p.A43V	c.128C>T	Verified	GAA	GCT	TGT	2	C	T
TCGA-24-2024	CCDC159	ENST00000427879	p.F259F	c.777C>T	Verified	AAG	TTC	CTG	3	C	T
TCGA-13-1496	CCDC164	ENST00000288710	p.L157L	c.471C>T	Verified	ATG	CTC	AAT	3	C	T
TCGA-13-0913	CCDC165	ENST00000359865	p.S1269F	c.3806C>T	Verified	GCC	TCC	CCA	2	C	T
TCGA-13-0884	CCDC8	ENST00000307522	p.S233S	c.699C>T	Verified	GAG	AGC	GCA	3	C	T
TCGA-13-0885	CCDC8	ENST00000307522	p.T63T	c.189C>T	Verified	AGC	ACC	CCG	3	C	T
TCGA-13-0755	CCDC85A	ENST00000407595	p.R131C	c.391C>T	Verified	GGT	CGC	TAC	1	C	T
TCGA-04-1367	CCDC88C	ENST00000389857	p.R1425C	c.4273C>T	Verified	GAA	CGC	TTA	1	C	T
TCGA-25-2042	CCDC94	ENST00000262962	p.Y77Y	c.231C>T	Unverified	TTT	TAC	ATC	3	C	T
TCGA-20-0991	CCNB3	ENST00000376042	p.S361F	c.1082C>T	Verified	GAT	TCC	CTT	2	C	T
TCGA-20-0991	CCNB3_ENS1	ENST00000376042	p.S361F	c.1082C>T	Verified	GAT	TCC	CTT	2	C	T
TCGA-29-1785	CCNYL2_EN!	ENST00000426433	p.Q19*	c.55C>T	Unverified	CAC	CAG	CAC	1	C	T
TCGA-29-1785	CCNYL2_EN!	ENST00000426433	p.Q19*	c.55C>T	Unverified	CAC	CAG	CAC	1	C	T
TCGA-29-1703	CCR2	ENST00000292301	p.N234N	c.702C>T	Unverified	CGA	AAC	GAG	3	C	T
TCGA-29-1703	CCR2_ENST(	ENST00000445132	p.N234N	c.702C>T	Unverified	CGA	AAC	GAG	3	C	T
TCGA-20-1687	CCT8L2	ENST00000359963	p.T258M	c.773C>T	Unverified	GCA	ACG	GCC	2	C	T

TCGA-61-1914	CD163L1	ENST00000313599	p.P199L	c.596C>T	Unverified	TGT	CCA	TCT	2	C	T
TCGA-13-0923	CD1C	ENST00000368170	p.L12F	c.34C>T	Verified	CTT	CTT	CTC	1	C	T
TCGA-61-1737	CD2	ENST00000369478	p.P338S	c.1012C>T	Unverified	CCT	CCC	CAT	1	C	T
TCGA-09-2050	CD22	ENST00000085219	p.S320L	c.959C>T	Verified	AGG	TCG	GAA	2	C	T
TCGA-13-1509	CD2AP	ENST00000359314	p.R504C	c.1510C>T	Verified	GGC	CGT	TTC	1	C	T
TCGA-13-1488	CD3D	ENST00000300692	p.D54D	c.162C>T	Verified	TCA	GAC	ATT	3	C	T
TCGA-61-2094	CD48	ENST00000368046	p.T24T	c.72C>T	Unverified	GTG	ACC	AGC	3	C	T
TCGA-29-1695	CD68	ENST00000250092	p.T161M	c.482C>T	Unverified	TAC	ACG	TGG	2	C	T
TCGA-25-1318	CD79A	ENST00000221972	p.F52F	c.156C>T	Verified	CAC	TTC	CAA	3	C	T
TCGA-25-1318	CD79A	ENST00000221972	p.F52F	c.156C>T	Verified	CAC	TTC	CAA	3	C	T
TCGA-13-0923	CD84	ENST00000368054	p.R84W	c.250C>T	Verified	GAA	CGG	ATA	1	C	T
TCGA-20-1683	CD97	ENST00000242786	p.G606G	c.1818C>T	Unverified	GGC	GGC	CAG	3	C	T
TCGA-61-1904	CDC20B	ENST00000296733	p.S87F	c.260C>T	Unverified	TCC	TCT	GAT	2	C	T
TCGA-13-1507	CDCA4	ENST00000392590	p.R67W	c.199C>T	Verified	GTC	CGG	CAG	1	C	T
TCGA-29-1766	CDH11	ENST00000268603	p.P504S	c.1510C>T	Unverified	AAG	CCA	CTT	1	C	T
TCGA-30-1855	CDH18	ENST00000382275	p.T757M	c.2270C>T	Unverified	GCA	ACG	ACA	2	C	T
TCGA-30-1855	CDH18_ENS	ENST00000507958	p.T757M	c.2270C>T	Unverified	GCA	ACG	ACA	2	C	T
TCGA-13-0923	CDH23	ENST00000398860	p.P2316S	c.6946C>T	Verified	ACC	CCT	GGG	1	C	T
TCGA-61-2113	CDH23	ENST00000398860	p.I920I	c.2760C>T	Unverified	GCC	ATC	GAC	3	C	T
TCGA-23-1032	CDH4	ENST00000360469	p.R194W	c.580C>T	Verified	ATC	CGG	TCC	1	C	T
TCGA-23-1114	CDH4	ENST00000360469	p.Y581Y	c.1743C>T	Unverified	GTC	TAC	GAG	3	C	T
TCGA-23-1021	CDH7	ENST00000323011	p.A742V	c.2225C>T	Verified	GTT	GCT	GAA	2	C	T
TCGA-30-1857	CDH7	ENST00000323011	p.P357L	c.1070C>T	Unverified	GGT	CCG	TTC	2	C	T
TCGA-61-1738	CDH7	ENST00000323011	p.D588D	c.1764C>T	Unverified	GCT	GAC	GGC	3	C	T
TCGA-61-1738	CDH7	ENST00000323011	p.D588D	c.1764C>T	Unverified	GCT	GAC	GGC	3	C	T
TCGA-30-1857	CDH7_ENST	ENST00000323011	p.P357L	c.1070C>T	Unverified	GGT	CCG	TTC	2	C	T
TCGA-61-1738	CDH7_ENST	ENST00000323011	p.D588D	c.1764C>T	Unverified	GCT	GAC	GGC	3	C	T
TCGA-61-1738	CDH7_ENST	ENST00000323011	p.D588D	c.1764C>T	Unverified	GCT	GAC	GGC	3	C	T
TCGA-29-1691	CDH8	ENST00000577390	p.P79L	c.236C>T	Unverified	GAA	CCG	ATT	2	C	T
TCGA-24-1616	CDH8	ENST00000577390	p.Y775Y	c.2325C>T	Verified	GAC	TAC	CTC	3	C	T
TCGA-29-1711	CDK12	NM_016507	p.P653L	c.1958C>T	Unverified	aaa	cct	gtg	2	C	T
TCGA-29-1711	CDK12	NM_016507	p.P653L	c.1958C>T	Unverified	aaa	cct	gtg	2	C	T
TCGA-10-0930	CDK5R1	ENST00000313401	p.P174P	c.522C>T	Verified	gac	ccc	gtg	3	C	T
TCGA-10-0930	CDK5R1	ENST00000313401	p.P174P	c.522C>T	Verified	gac	ccc	gtg	3	C	T

TCGA-20-0990	CDK5RAP1	ENST00000346416	p.R169W	c.505C>T	Verified	agg	cgg	ccc	1	C	T
TCGA-29-1690	CDK5RAP2	ENST00000349780	p.S613S	c.1839C>T	Unverified	atc	agc	gaa	3	C	T
TCGA-29-1703	CDK6	ENST00000265734	p.Y196Y	c.588C>T	Unverified	agc	tac	gcc	3	C	T
TCGA-09-1665	CDKL1	ENST00000395834	p.L222L	c.666C>T	Verified	GAT	CTC	ATT	3	C	T
TCGA-20-1687	CDRT1_ENST	ENST00000395906	p.S235F	c.704C>T	Unverified	ATA	TCC	GAA	2	C	T
TCGA-24-1464	CDX1	ENST00000231656	p.T162T	c.486C>T	Verified	TAC	ACC	GAC	3	C	T
TCGA-04-1337	CEACAM18_	ENST00000451626	p.R27*	c.79C>T	Verified	AGA	CGA	GAC	1	C	T
TCGA-25-2401	CEACAM8	ENST00000244336	p.L202L	c.604C>T	Verified	ACT	CTA	CTC	1	C	T
TCGA-29-1776	CECR1	ENST00000399837	p.P193S	c.577C>T	Unverified	CAC	CCG	GAG	1	C	T
TCGA-24-1846	CECR2	ENST00000400585	p.A360V	c.1079C>T	Unverified	AGG	GCA	TGG	2	C	T
TCGA-20-1687	CECR2	ENST00000400585	p.H785H	c.2355C>T	Unverified	GGT	CAC	GTG	3	C	T
TCGA-24-1846	CECR2_ENST	ENST00000262608	p.A361V	c.1082C>T	Unverified	ATG	TCT	GAT	2	C	T
TCGA-20-1687	CECR2_ENST	ENST00000262608	p.H786H	c.2358C>T	Unverified	TCA	GCC	CCC	3	C	T
TCGA-04-1336	CELA2A	ENST00000359621	p.S195S	c.585C>T	Verified	AGC	AGC	GTG	3	C	T
TCGA-25-2392	CELF4	ENST00000361795	p.A308V	c.923C>T	Verified	CTG	GCG	GCC	2	C	T
TCGA-25-2393	CELSR2	ENST00000271332	p.A687V	c.2060C>T	Verified	ACC	GCC	TCC	2	C	T
TCGA-61-1725	CENPE	ENST00000265148.1	p.R290*	c.868C>T	Unverified	aca	cga	att	1	C	T
TCGA-13-0883	CENPF	ENST00000366955	p.Q541*	c.1621C>T	Verified	CTT	CAA	GAA	1	C	T
TCGA-13-1509	CENPF	ENST00000366955	p.L1300L	c.3898C>T	Verified	AAG	CTG	AAT	1	C	T
TCGA-30-1891	CENPJ	ENST00000381884	p.D49D	c.147C>T	Unverified	GTG	GAC	ATT	3	C	T
TCGA-23-1809	CEP170	ENST00000366542	p.R956*	c.2866C>T	Unverified	AAG	CGA	AAG	1	C	T
TCGA-13-0755	CEP170	ENST00000366542	p.T565I	c.1694C>T	Verified	ACT	ACA	TCT	2	C	T
TCGA-30-1714	CEP290	ENST00000552810	p.R2112W	c.6334C>T	Unverified	CAG	CGG	AAA	1	C	T
TCGA-24-1423	CEP72	ENST00000264935	p.H150Y	c.448C>T	Verified	CTG	CAT	TTT	1	C	T
TCGA-61-2012	CEP78	ENST00000277082	p.L613L	c.1839C>T	Verified	CCT	CTC	GAC	3	C	T
TCGA-20-0990	CERCAM	ENST00000372842	p.L455L	c.1365C>T	Unverified	CTG	CTC	GCT	3	C	T
TCGA-25-2398	CETN1	ENST00000327228	p.R164W	c.490C>T	Verified	CTT	CGG	ATC	1	C	T
TCGA-29-1695	CFB	ENST00000425368	p.H737H	c.2211C>T	Unverified	GCT	CAC	GCC	3	C	T
TCGA-29-1763	CHD3	ENST00000330494	p.S1902S	c.5706C>T	Unverified	CGC	AGC	ATC	3	C	T
TCGA-04-1651	CHD6	ENST00000373233	p.Q1861*	c.5581C>T	Unverified	agt	cag	aac	1	C	T
TCGA-04-1651	CHD6	ENST00000373233	p.Q1861*	c.5581C>T	Unverified	agt	cag	aac	1	C	T
TCGA-09-2051	CHD9	NM_025134.3	p.Q230*	c.688C>T	Unverified	cag	caa	ttt	1	C	T
TCGA-09-0369	CHEK2	ENST00000328354	p.P536L	c.1607C>T	Unverified	cgc	cca	gct	2	C	T
TCGA-09-0369	CHEK2	ENST00000328354	p.P536L	c.1607C>T	Unverified	cgc	cca	gct	2	C	T

TCGA-13-0755	CHEK2	ENST00000328354	p.P536L	c.1607C>T	Unverified	cgc	cca	gct	2	C	T
TCGA-13-0760	CHEK2	ENST00000328354	p.P536L	c.1607C>T	Unverified	cgc	cca	gct	2	C	T
TCGA-24-1850	CHEK2	ENST00000328354	p.T45M	c.134C>T	Unverified	agc	acg	atg	2	C	T
TCGA-09-0369	CHEK2	ENST00000328354	p.P522P	c.1566C>T	Unverified	cgg	ccc	cgt	3	C	T
TCGA-09-0369	CHEK2	ENST00000328354	p.P522P	c.1566C>T	Unverified	cgg	ccc	cgt	3	C	T
TCGA-13-0755	CHEK2	ENST00000328354	p.P522P	c.1566C>T	Unverified	cgg	ccc	cgt	3	C	T
TCGA-13-0760	CHEK2	ENST00000328354	p.P522P	c.1566C>T	Unverified	cgg	ccc	cgt	3	C	T
TCGA-13-0762	CHEK2	ENST00000328354	p.P522P	c.1566C>T	Unverified	cgg	ccc	cgt	3	C	T
TCGA-13-0762	CHEK2	ENST00000328354	p.P522P	c.1566C>T	Unverified	cgg	ccc	cgt	3	C	T
TCGA-20-1683	CHGB	ENST00000378961	p.Y526Y	c.1578C>T	Unverified	TAC	TAC	GAC	3	C	T
TCGA-23-2078	CHKB	ENST00000406938	p.G112G	c.336C>T	Unverified	CAG	GGC	GTG	3	C	T
TCGA-13-0761	CHMP6	ENST00000325167	p.A195V	c.584C>T	Verified	CAG	GCG	GAG	2	C	T
TCGA-13-0761	CHMP6	ENST00000325167	p.A195V	c.584C>T	Verified	CAG	GCG	GAG	2	C	T
TCGA-13-0920	CHRNA2	ENST00000407991	p.R61W	c.181C>T	Verified	GAC	CGG	CTC	1	C	T
TCGA-09-2049	CHRNA3	ENST00000326828	p.S423F	c.1268C>T	Verified	AGC	TCT	AGT	2	C	T
TCGA-24-2267	CHST2	ENST00000309575	p.A391V	c.1172C>T	Verified	CAC	GCT	CTG	2	C	T
TCGA-04-1362	CHUK	ENST00000370397	p.S263S	c.789C>T	Verified	AAT	AGC	CTT	3	C	T
TCGA-61-1998	CIC	ENST00000160740	p.S1389F	c.4166C>T	Unverified	GGC	TCT	TAC	2	C	T
TCGA-24-1846	CIDEB	ENST00000258807	p.D85D	c.255C>T	Unverified	GTG	GAC	AGT	3	C	T
TCGA-29-2427	CLASP1	ENST00000263710	p.A344V	c.1031C>T	Verified	AAT	GCT	CTA	2	C	T
TCGA-23-1116	CLCN1	ENST00000343257	p.R47W	c.139C>T	Unverified	CTC	CGG	AAG	1	C	T
TCGA-04-1651	CLCN7	ENST00000382745	p.S232S	c.696C>T	Unverified	GTG	TCC	GGT	3	C	T
TCGA-04-1651	CLCN7	ENST00000382745	p.S232S	c.696C>T	Unverified	GTG	TCC	GGT	3	C	T
TCGA-13-0791	CLCNKA	ENST00000331433	p.L94F	c.280C>T	Unverified	TAT	CTT	TCC	1	C	T
TCGA-29-1701	CLCNKB	ENST00000375679	p.L444L	c.1332C>T	Unverified	ACT	CTC	TCT	3	C	T
TCGA-61-1740	CLEC17A_EN	ENST00000417570	p.P100S	c.298C>T	Unverified	AGA	CCT	CCA	1	C	T
TCGA-29-1693	CLEC18B	ENST00000339953	p.T151M	c.452C>T	Unverified	TAC	ACG	CAG	2	C	T
TCGA-30-1857	CLEC18B	ENST00000339953	p.A79A	c.237C>T	Unverified	CTG	GCC	CAA	3	C	T
TCGA-04-1362	CLGN	ENST00000325617	p.S579F	c.1736C>T	Verified	AAG	TCT	GGG	2	C	T
TCGA-13-1489	CLIC5	ENST00000339561	p.N220N	c.660C>T	Verified	AAG	AAC	GCC	3	C	T
TCGA-24-2035	CLINT1	ENST00000523094	p.T240M	c.719C>T	Verified	GTG	ACG	ACA	2	C	T
TCGA-24-1843	CLIP1	ENST00000358808	p.S147L	c.440C>T	Unverified	ACT	TCA	CCG	2	C	T
TCGA-04-1338	CLIP4	ENST00000320081	p.A254V	c.761C>T	Verified	GAT	GCG	GTG	2	C	T
TCGA-13-0923	CLK4	ENST00000316308	p.R2W	c.4C>T	Verified	ATG	CGG	CAT	1	C	T

TCGA-13-0885	CLN5	ENST00000377453	p.F206F	c.618C>T	Verified	CCT	TTC	TGG	3	C	T
TCGA-13-0923	CLPP	ENST00000245816	p.I177I	c.531C>T	Unverified	ATG	ATC	CAC	3	C	T
TCGA-29-1698	CLPTM1L	ENST00000320895	p.S261S	c.783C>T	Unverified	TAC	TCC	CTG	3	C	T
TCGA-30-1857	CLSTN2	ENST00000458420	p.T170M	c.509C>T	Unverified	GTG	ACG	GAG	2	C	T
TCGA-04-1362	CLU	NM_001831.2	p.R250W	c.748C>T	Verified	acc	cgg	gag	1	C	T
TCGA-04-1338	CLU	NM_001831.2	p.P286L	c.857C>T	Verified	tct	ccg	tac	2	C	T
TCGA-13-0760	CLUAP1	ENST00000576634	p.D257D	c.771C>T	Verified	TAT	GAC	ACT	3	C	T
TCGA-13-1497	CMYA5	ENST00000238522	p.T2592I	c.7775C>T	Verified	GCT	ACA	TCA	2	C	T
TCGA-29-1703	CNBD1	ENST00000518476	p.R380*	c.1138C>T	Unverified	CTA	CGA	TCA	1	C	T
TCGA-29-1703	CNBD1_ENS	ENST00000518476	p.R380*	c.1138C>T	Unverified	CTA	CGA	TCA	1	C	T
TCGA-23-1122	CNDP2	ENST00000324262	p.A465V	c.1394C>T	Verified	GCC	GCG	TAC	2	C	T
TCGA-24-2288	CNGA1	ENST00000358519	p.R643*	c.1927C>T	Unverified	GCC	CGA	ATC	1	C	T
TCGA-13-0913	CNGA3	ENST00000272602	p.T654I	c.1961C>T	Verified	GCC	ACC	CAG	2	C	T
TCGA-23-1111	CNGA3	ENST00000272602	p.T565M	c.1694C>T	Unverified	AGG	ACG	GCC	2	C	T
TCGA-29-1761	CNGA3	ENST00000272602	p.P683P	c.2049C>T	Unverified	GTT	CCC	GGG	3	C	T
TCGA-61-2095	CNGA3	ENST00000272602	p.V382V	c.1146C>T	Verified	GTG	GTC	GTA	3	C	T
TCGA-61-1910	CNGA4	ENST00000379936	p.I487I	c.1461C>T	Unverified	GAG	ATC	GCC	3	C	T
TCGA-61-1907	CNGB1	ENST00000251102	p.N653N	c.1959C>T	Unverified	ACC	AAC	CTG	3	C	T
TCGA-23-1032	CNIH4	ENST00000465271	p.S28F	c.83C>T	Unverified	TTG	TCT	GAT	2	C	T
TCGA-61-1740	CNKSR1	ENST00000361530	p.L585L	c.1753C>T	Unverified	CTC	CTA	GGC	1	C	T
TCGA-23-1114	CNN3	ENST00000370206	p.G326G	c.978C>T	Unverified	CAA	GGC	ATT	3	C	T
TCGA-13-1510	CNPY1	ENST00000321736	p.L63F	c.187C>T	Unverified	TCA	CTT	ATC	1	C	T
TCGA-13-0900	CNPY3	ENST00000372836	p.N153N	c.459C>T	Verified	TGG	AAC	GAG	3	C	T
TCGA-29-1693	CNR1	ENST00000369499	p.I348I	c.1044C>T	Unverified	CTG	ATC	CTG	3	C	T
TCGA-13-1509	CNTN4	ENST00000397459	p.L134F	c.400C>T	Verified	AAC	CTC	AGA	1	C	T
TCGA-61-1899	CNTNAP2	ENST00000361727	p.N379N	c.1137C>T	Unverified	TTC	AAC	GCT	3	C	T
TCGA-04-1655	CNTNAP5	ENST00000431078	p.R1302W	c.3904C>T	Unverified	AAA	CGG	GAA	1	C	T
TCGA-61-1740	COG7	ENST00000307149	p.F324F	c.972C>T	Unverified	CAC	TTC	GCC	3	C	T
TCGA-04-1331	COL15A1	ENST00000375001	p.S37F	c.110C>T	Verified	GCT	TCC	CAG	2	C	T
TCGA-04-1652	COL16A1	ENST00000373672	p.P764P	c.2292C>T	Unverified	CAA	CCC	GGT	3	C	T
TCGA-04-1652	COL16A1	ENST00000373672	p.P764P	c.2292C>T	Unverified	CAA	CCC	GGT	3	C	T
TCGA-23-1116	COL22A1	ENST00000303045	p.R216C	c.646C>T	Unverified	CGC	CGT	CTT	1	C	T
TCGA-13-1496	COL22A1	ENST00000303045	p.G508G	c.1524C>T	Verified	GTT	GGC	GCT	3	C	T
TCGA-20-0991	COL22A1	ENST00000303045	p.G178G	c.534C>T	Verified	GTG	GGC	GAG	3	C	T

TCGA-30-1857	COL23A1	ENST00000390654	p.G424G	c.1272C>T	Unverified	ATG	GGC	CTC	3	C	T
TCGA-25-1313	COL25A1	ENST00000399132	p.R364W	c.1090C>T	Verified	GAA	CGG	GGG	1	C	T
TCGA-13-1505	COL27A1	ENST00000356083	p.R1611W	c.4831C>T	Verified	GGG	CGG	CCC	1	C	T
TCGA-61-2113	COL27A1	ENST00000356083	p.G775G	c.2325C>T	Verified	CGA	GGC	CTG	3	C	T
TCGA-23-1124	COL2A1	ENST00000337299	p.R265W	c.793C>T	Verified	GGA	CGG	ACT	1	C	T
TCGA-13-0884	COL4A1	ENST00000375820	p.P1367S	c.4099C>T	Verified	GGC	CCC	CCA	1	C	T
TCGA-13-0791	COL4A2	ENST00000360467	p.P164S	c.490C>T	Verified	GGA	CCA	AAA	1	C	T
TCGA-25-2393	COL4A4	ENST00000396625	p.P1174L	c.3521C>T	Verified	TCA	CCT	GGC	2	C	T
TCGA-29-1784	COL4A6	ENST00000334504	p.P1554P	c.4662C>T	Unverified	ATG	CCC	GTC	3	C	T
TCGA-24-2288	COL5A2	ENST00000374866	p.P659S	c.1975C>T	Unverified	CCG	CCG	GGT	1	C	T
TCGA-29-1699	COL5A2	ENST00000374866	p.T434M	c.1301C>T	Unverified	CCA	ACG	GGC	2	C	T
TCGA-29-1691	COL5A3	ENST00000264828	p.P299L	c.896C>T	Unverified	CTG	CCT	CCG	2	C	T
TCGA-30-1855	COL5A3	ENST00000264828	p.A125V	c.374C>T	Unverified	CCA	GCG	CTG	2	C	T
TCGA-20-0990	COL6A2	ENST00000300527	p.R933C	c.2797C>T	Unverified	CCG	CGT	GGC	1	C	T
TCGA-24-1843	COL6A3	ENST00000295550	p.R1727W	c.5179C>T	Unverified	CTG	CGG	GTA	1	C	T
TCGA-30-1714	COL6A3	ENST00000295550	p.R1742W	c.5224C>T	Unverified	CAG	CGG	GTC	1	C	T
TCGA-29-1781	COL6A3	ENST00000295550	p.S454F	c.1361C>T	Unverified	TCA	TCT	GCA	2	C	T
TCGA-29-1775	COL6A3	ENST00000295550	p.P8P	c.24C>T	Unverified	TTG	CCC	TTA	3	C	T
TCGA-29-1781	COL6A3_EN!	ENST00000392004	p.S248F	c.743C>T	Unverified	TCA	TCT	GCA	2	C	T
TCGA-29-1775	COL6A3_EN!	ENST00000392004	p.P8P	c.24C>T	Unverified	TTG	CCC	TTA	3	C	T
TCGA-61-2009	COL6A6	ENST00000358511	p.T932M	c.2795C>T	Verified	GCC	ACG	GCA	2	C	T
TCGA-13-0883	COL6A6	ENST00000358511	p.A1867A	c.5601C>T	Verified	ATC	GCC	ACA	3	C	T
TCGA-24-2288	COL7A1	ENST00000328333	p.G1863G	c.5589C>T	Unverified	TCA	GGC	GCC	3	C	T
TCGA-04-1331	COL9A1	ENST00000357250	p.R352C	c.1054C>T	Verified	TCG	CGT	GGA	1	C	T
TCGA-24-1604	COL9A2	ENST00000372748	p.G340G	c.1020C>T	Verified	CCA	GGC	CAG	3	C	T
TCGA-24-1846	COLQ	ENST00000383788	p.D395D	c.1185C>T	Unverified	GGT	GAC	GAC	3	C	T
TCGA-24-1846	COLQ_ENST	ENST00000383781	p.D385D	c.1155C>T	Unverified	GGT	GAC	GAC	3	C	T
TCGA-24-1464	COMMD10	ENST00000274458	p.V110V	c.330C>T	Verified	TTT	GTC	AAT	3	C	T
TCGA-20-0991	COPA	ENST00000241704	p.P594L	c.1781C>T	Verified	GAT	CCC	ACT	2	C	T
TCGA-24-1845	CORO2A	ENST00000343933	p.S28F	c.83C>T	Unverified	gac	tcc	gtg	2	C	T
TCGA-29-1699	CORO2B	ENST00000566799	p.R474C	c.1420C>T	Unverified	TTG	CGC	AAC	1	C	T
TCGA-24-1104	COX6B1_EN	ENST00000392201	p.Q6*	c.16C>T	Verified	CCT	CAG	ATA	1	C	T
TCGA-25-2392	CPA1	ENST00000011292	p.L210L	c.630C>T	Verified	ATT	CTC	GAC	3	C	T
TCGA-61-1895	CPA2	ENST00000222481	p.R394C	c.1180C>T	Unverified	GCC	CGT	CAG	1	C	T

TCGA-13-0793	CPA5	ENST00000393213	p.P73S	c.217C>T	Verified	GGC	CCA	GCC	1	C	T
TCGA-24-2280	CPA5	ENST00000393213	p.R335*	c.1003C>T	Unverified	GGC	CGA	TTG	1	C	T
TCGA-23-1123	CPB1	ENST00000282957	p.R152C	c.454C>T	Verified	GGA	CGC	GCT	1	C	T
TCGA-23-1123	CPB1	ENST00000282957	p.R152C	c.454C>T	Verified	GGA	CGC	GCT	1	C	T
TCGA-13-0714	CPN2	ENST00000323830	p.P49L	c.146C>T	Verified	CCG	CCA	TAT	2	C	T
TCGA-29-1777	CPNE6	ENST00000397016	p.P334P	c.1002C>T	Unverified	CAG	CCC	AAC	3	C	T
TCGA-04-1336	CPXM1	ENST00000380605	p.H180Y	c.538C>T	Verified	GGG	CAC	CCC	1	C	T
TCGA-24-1846	CPXM2	ENST00000241305	p.S662S	c.1986C>T	Unverified	ATC	TCC	GTA	3	C	T
TCGA-09-1674	CPZ	ENST00000360986	p.T181I	c.542C>T	Unverified	CCC	ACC	TTC	2	C	T
TCGA-04-1362	CRB1	ENST00000367400	p.H699Y	c.2095C>T	Verified	TGC	CAC	AGG	1	C	T
TCGA-09-2051	CRB1	ENST00000367400	p.T289M	c.866C>T	Unverified	TGC	ACG	GGT	2	C	T
TCGA-20-0991	CRCT1	ENST00000368790	p.C75C	c.225C>T	Unverified	GGT	TGC	TGC	3	C	T
TCGA-61-1915	CREB3L2	ENST00000330387	p.S150F	c.449C>T	Unverified	atc	tcc	acc	2	C	T
TCGA-20-1687	CRISPLD2	ENST00000262424	p.P426L	c.1277C>T	Unverified	GCT	CCG	GTG	2	C	T
TCGA-29-1761	CRLS1	ENST00000378863	p.Q297*	c.889C>T	Unverified	GTT	CAG	GTG	1	C	T
TCGA-24-1470	CRMP1	ENST00000324989	p.A375A	c.1125C>T	Verified	GCA	GCC	GAC	3	C	T
TCGA-29-1785	CROCC	ENST00000375541	p.R918W	c.2752C>T	Unverified	CAA	CGG	CAG	1	C	T
TCGA-29-1785	CROCC	ENST00000375541	p.R918W	c.2752C>T	Unverified	CAA	CGG	CAG	1	C	T
TCGA-61-1998	CRTC2	ENST00000368633	p.R200*	c.598C>T	Verified	AGC	CGA	CGT	1	C	T
TCGA-29-1695	CRTC3	ENST00000268184	p.R386W	c.1156C>T	Unverified	CGT	CGG	CAG	1	C	T
TCGA-04-1331	CSAD	ENST00000308926	p.R379W	c.1135C>T	Verified	GAG	CGG	CGC	1	C	T
TCGA-29-1770	CSF1R	ENST00000286301	p.Q835*	c.2503C>T	Unverified	GTT	CAG	AGC	1	C	T
TCGA-29-1770	CSF1R_ENST	ENST00000286301	p.Q835*	c.2503C>T	Unverified	GTT	CAG	AGC	1	C	T
TCGA-29-1785	CSF2RA	ENST00000381529	p.L27L	c.79C>T	Unverified	gat	ctg	cga	1	C	T
TCGA-29-1785	CSF2RA	ENST00000381529	p.L27L	c.79C>T	Unverified	gat	ctg	cga	1	C	T
TCGA-29-1785	CSF2RA_ENS	ENST00000355432	p.L27L	c.79C>T	Unverified	GAT	CTG	CGA	1	C	T
TCGA-29-1785	CSF2RA_ENS	ENST00000355432	p.L27L	c.79C>T	Unverified	GAT	CTG	CGA	1	C	T
TCGA-29-1785	CSF2RA_ENS	ENST00000381509	p.L27L	c.79C>T	Unverified	GAT	CTG	CGA	1	C	T
TCGA-29-1785	CSF2RA_ENS	ENST00000381509	p.L27L	c.79C>T	Unverified	GAT	CTG	CGA	1	C	T
TCGA-04-1649	CSH2_ENST(	ENST00000392886	p.R159C	c.475C>T	Unverified	AGC	CGC	CGG	1	C	T
TCGA-04-1638	CSMD1_ENS	ENST00000318252	p.S2904S	c.8712C>T	Unverified	TTC	TTC	CAG	3	C	T
TCGA-29-1762	CSMD1_ENS	ENST00000318252	p.T2236T	c.6708C>T	Unverified	GAG	TTC	ACT	3	C	T
TCGA-04-1638	CSMD1_ENS	ENST00000537824	p.S3175S	c.9525C>T	Unverified	AAG	TCC	GAA	3	C	T
TCGA-04-1652	CSMD1_ENS	ENST00000537824	p.N2867N	c.8601C>T	Unverified	GCC	AAC	GCC	3	C	T

TCGA-04-1652	CSMD1_ENS	ENST00000537824	p.N2867N	c.8601C>T	Unverified	GCC	AAC	GCC	3	C	T
TCGA-29-1762	CSMD1_ENS	ENST00000537824	p.T2507T	c.7521C>T	Unverified	TTT	ACC	GGG	3	C	T
TCGA-20-1686	CSMD2	ENST00000241312	p.Y49Y	c.147C>T	Unverified	AAT	TAC	GCC	3	C	T
TCGA-20-1686	CSMD2_ENS	ENST00000373381	p.Y89Y	c.267C>T	Unverified	AAT	TAC	GCC	3	C	T
TCGA-29-1785	CSMD3	ENST00000297405	p.P141S	c.421C>T	Unverified	ctg	cca	cct	1	C	T
TCGA-29-1785	CSMD3	ENST00000297405	p.P141S	c.421C>T	Unverified	ctg	cca	cct	1	C	T
TCGA-29-1785	CSMD3_ENS	ENST00000343508	p.P101S	c.301C>T	Unverified	CTG	CCA	CCT	1	C	T
TCGA-29-1785	CSMD3_ENS	ENST00000343508	p.P101S	c.301C>T	Unverified	CTG	CCA	CCT	1	C	T
TCGA-13-1481	CSPG4	ENST00000308508	p.F1906F	c.5718C>T	Verified	gcc	ttc	gtg	3	C	T
TCGA-13-1488	CTAGE4	ENST00000486333	p.S733F	c.2198C>T	Unverified	GCT	TCT	CGA	2	C	T
TCGA-29-1703	CTC1	ENST00000315684	p.P516L	c.1547C>T	Unverified	GCT	CCG	CCA	2	C	T
TCGA-04-1649	CTDSP1	ENST00000273062	p.D248D	c.744C>T	Unverified	GTG	GAC	GAC	3	C	T
TCGA-20-0990	CTNNA3	NM_013266.1	p.R297*	c.889C>T	Verified	ata	cga	cca	1	C	T
TCGA-61-1740	CTNNA3	NM_013266.1	p.T422I	c.1265C>T	Unverified	cac	acc	agc	2	C	T
TCGA-04-1652	CTNNA3	NM_013266.1	p.S434S	c.1302C>T	Unverified	tgt	tcc	atg	3	C	T
TCGA-04-1652	CTNNA3	NM_013266.1	p.S434S	c.1302C>T	Unverified	tgt	tcc	atg	3	C	T
TCGA-61-1740	CTNNA3_EN	ENST00000433211	p.T422I	c.1265C>T	Unverified	CAC	ACC	AGC	2	C	T
TCGA-04-1652	CTNNA3_EN	ENST00000433211	p.S434S	c.1302C>T	Unverified	TGT	TCC	ATG	3	C	T
TCGA-04-1652	CTNNA3_EN	ENST00000433211	p.S434S	c.1302C>T	Unverified	TGT	TCC	ATG	3	C	T
TCGA-20-0990	CTSL1	ENST00000343150	p.H36Y	c.106C>T	Verified	ATG	CAC	AAC	1	C	T
TCGA-24-2288	CUL9	ENST00000252050	p.R1730C	c.5188C>T	Verified	GAC	CGT	TTC	1	C	T
TCGA-04-1367	CUX2	ENST00000261726	p.R1088W	c.3262C>T	Verified	TCC	CGG	CCC	1	C	T
TCGA-25-2391	CUX2	ENST00000261726	p.P1256S	c.3766C>T	Unverified	GAC	CCC	ACC	1	C	T
TCGA-13-0923	CUZD1	ENST00000368904	p.D94D	c.282C>T	Verified	TTT	GAC	GGA	3	C	T
TCGA-61-1740	CXCR2	ENST00000318507	p.A255V	c.764C>T	Unverified	ttt	gct	gtc	2	C	T
TCGA-13-1509	CXorf57	ENST00000372548	p.P315S	c.943C>T	Verified	GTC	CCC	GTG	1	C	T
TCGA-23-1110	CXorf67	NM_203407.1	p.T494I	c.1481C>T	Verified	agc	aca	gct	2	C	T
TCGA-24-2262	CYB5R1	ENST00000367249	p.R196W	c.586C>T	Verified	ATC	CGG	GCC	1	C	T
TCGA-13-0886	CYP11B2	ENST00000323110	p.D335D	c.1005C>T	Unverified	CCC	GAC	GTG	3	C	T
TCGA-29-1776	CYP26B1	ENST00000001146	p.R469W	c.1405C>T	Unverified	ACA	CGG	ACC	1	C	T
TCGA-20-0990	CYP2A13	ENST00000330436	p.P75S	c.223C>T	Verified	GGG	CCC	CGG	1	C	T
TCGA-13-1509	CYP2B6	ENST00000324071	p.R378*	c.1132C>T	Verified	TTC	CGA	GGG	1	C	T
TCGA-04-1649	CYP2D6	ENST00000359033	p.S166L	c.497C>T	Unverified	GAG	TCG	GGC	2	C	T
TCGA-04-1649	CYP2D6_EN!	ENST00000360608	p.S217L	c.650C>T	Unverified	GAG	TCG	GGC	2	C	T

TCGA-13-0807	CYP2R1	ENST00000334636	p.R138*	c.412C>T	Verified	AGA	CGA	TTA	1	C	T
TCGA-61-1737	CYP4A22	ENST00000371891	p.L48F	c.142C>T	Unverified	GCC	CTC	CAG	1	C	T
TCGA-61-1998	CYP4A22	ENST00000371891	p.P157S	c.469C>T	Verified	AAG	CCA	TAC	1	C	T
TCGA-23-1122	CYP4F2	ENST00000221700	p.R274W	c.820C>T	Verified	GAG	CGG	CGC	1	C	T
TCGA-13-1497	CYP4F3	ENST00000221307	p.H264H	c.792C>T	Verified	GTG	CAC	GAC	3	C	T
TCGA-29-1781	CYP7A1	ENST00000301645	p.Y401Y	c.1203C>T	Unverified	ATC	TAC	CCA	3	C	T
TCGA-09-2044	CYP7B1	ENST00000310193	p.T426I	c.1277C>T	Verified	AAA	ACC	ACC	2	C	T
TCGA-23-1021	CYTH4	ENST00000248901	p.L140L	c.420C>T	Verified	AAC	CTC	GTC	3	C	T
TCGA-13-0923	DAB1	ENST00000371233	p.G304G	c.912C>T	Unverified	ATG	GGC	GCT	3	C	T
TCGA-13-1501	DAGLB	ENST00000297056	p.P88L	c.263C>T	Verified	AAC	CCT	GGA	2	C	T
TCGA-13-1501	DAGLB	ENST00000297056	p.P88L	c.263C>T	Verified	AAC	CCT	GGA	2	C	T
TCGA-13-0905	DAGLB	ENST00000297056	p.A458A	c.1374C>T	Verified	AGA	GCC	GCC	3	C	T
TCGA-25-1326	DAP3	ENST00000368336	p.S215S	c.645C>T	Verified	gaa	agc	act	3	C	T
TCGA-24-1843	DARC_ENST0	ENST00000368121	p.T172I	c.515C>T	Unverified	CTC	ACT	GTG	2	C	T
TCGA-29-1776	DBC1	ENST00000373969	p.R198C	c.592C>T	Unverified	ACA	CGC	ACT	1	C	T
TCGA-24-1847	DBH	ENST00000393056	p.L197F	c.589C>T	Unverified	CAG	CTC	CTG	1	C	T
TCGA-61-2095	DBH	ENST00000393056	p.R79W	c.235C>T	Unverified	GTG	CGG	AGG	1	C	T
TCGA-29-1691	DBN1_ENST	ENST00000292385	p.R22R	c.66C>T	Unverified	GGA	CGC	GAG	3	C	T
TCGA-29-1691	DCAF12L2	ENST00000538699	p.N107N	c.321C>T	Unverified	ctg	aac	gcc	3	C	T
TCGA-29-1702	DCAF4L2	ENST00000319675	p.L37L	c.111C>T	Unverified	TTC	CTC	AGA	3	C	T
TCGA-24-1845	DCAF4L2	ENST00000319675	p.N361N	c.1083C>T	Unverified	GAG	AAC	GAC	3	C	T
TCGA-04-1649	DCC	ENST00000442544	p.R130W	c.388C>T	Unverified	AGT	CGG	ACA	1	C	T
TCGA-13-0792	DCC	ENST00000442544	p.D620D	c.1860C>T	Verified	TCT	GAC	GTG	3	C	T
TCGA-20-1686	DCC	ENST00000442544	p.A812A	c.2436C>T	Unverified	AGT	GCC	ACC	3	C	T
TCGA-13-1498	DCLK1	ENST00000255448	p.A560V	c.1679C>T	Verified	ATT	GCA	GAG	2	C	T
TCGA-13-0760	DCLK3	ENST00000416516	p.R592W	c.1774C>T	Verified	agc	cgg	ttg	1	C	T
TCGA-20-1683	DCLRE1C	ENST00000378246	p.P539P	c.1617C>T	Unverified	GTT	CCC	TCA	3	C	T
TCGA-20-1683	DCLRE1C_EN	ENST00000378278	p.P654P	c.1962C>T	Unverified	GTT	CCC	TCA	3	C	T
TCGA-13-1496	DCST2	ENST00000368424	p.T134T	c.402C>T	Verified	CAG	ACC	GCC	3	C	T
TCGA-61-1913	DDAH1	ENST00000284031	p.S276L	c.827C>T	Unverified	TGC	TCA	GTT	2	C	T
TCGA-04-1338	DDB1	ENST00000301764	p.P721L	c.2162C>T	Verified	TCT	CCA	AGG	2	C	T
TCGA-13-0920	DDR2	ENST00000367922	p.G39G	c.117C>T	Verified	GGA	GGC	CAG	3	C	T
TCGA-61-2009	DDX24	ENST00000330836	p.T667T	c.2001C>T	Verified	cgt	acc	tcg	3	C	T
TCGA-13-0884	DDX42	ENST00000578681	p.R858W	c.2572C>T	Verified	cat	cgg	cac	1	C	T

TCGA-24-1417	DDX50	ENST00000373585	p.S527S	c.1581C>T	Verified	AAA	AGC	ATG	3	C	T
TCGA-13-1488	DEFB118	ENST00000253381	p.Q37*	c.109C>T	Verified	AAA	CAA	TGC	1	C	T
TCGA-29-1690	DEFB118	ENST00000253381	p.R64*	c.190C>T	Unverified	AGG	CGA	GTT	1	C	T
TCGA-13-1488	DEFB125	ENST00000382410	p.S137F	c.410C>T	Verified	CCA	TCT	GAG	2	C	T
TCGA-13-1488	DENND3	ENST00000262585	p.H461Y	c.1381C>T	Verified	TCG	CAC	CTG	1	C	T
TCGA-25-2400	DENND4A	ENST00000431932	p.T155M	c.464C>T	Verified	AAT	ACG	TTG	2	C	T
TCGA-24-1845	DENND5B	ENST00000389082	p.R1216C	c.3646C>T	Unverified	GAT	CGC	CTG	1	C	T
TCGA-23-1032	DEPDC5	ENST00000400248	p.Q107*	c.319C>T	Verified	GAT	CAG	TAT	1	C	T
TCGA-04-1361	DEPDC5	ENST00000400248	p.S442F	c.1325C>T	Verified	ACA	TCT	CTC	2	C	T
TCGA-04-1361	DEPDC5	ENST00000400248	p.S442F	c.1325C>T	Verified	ACA	TCT	CTC	2	C	T
TCGA-13-0885	DERL3	ENST00000318109	p.D187D	c.561C>T	Verified	GAG	GAC	GTC	3	C	T
TCGA-04-1361	DFNA5	ENST00000342947	p.R108R	c.324C>T	Verified	AGC	CGC	GTA	3	C	T
TCGA-04-1361	DFNA5	ENST00000342947	p.R108R	c.324C>T	Verified	AGC	CGC	GTA	3	C	T
TCGA-24-2035	DFNB31	ENST00000362057	p.L816L	c.2446C>T	Unverified	ACT	CTG	GTC	1	C	T
TCGA-29-1775	DGKB	ENST00000403951	p.T696T	c.2088C>T	Unverified	ACC	ACC	GTC	3	C	T
TCGA-10-0930	DHCR7	ENST00000407721	p.T135I	c.404C>T	Verified	GTG	ACT	CCT	2	C	T
TCGA-10-0930	DHCR7	ENST00000407721	p.T135I	c.404C>T	Verified	GTG	ACT	CCT	2	C	T
TCGA-13-0795	DHRS4	ENST00000313250	p.A47V	c.140C>T	Verified	TTC	GCC	ATC	2	C	T
TCGA-04-1362	DIAPH3	ENST00000400324	p.P940S	c.2818C>T	Verified	TTT	CCC	CCT	1	C	T
TCGA-13-1512	DIDO1	ENST00000395343	p.A333V	c.998C>T	Verified	ACG	GCA	GAT	2	C	T
TCGA-13-1512	DIDO1	ENST00000395343	p.S1812L	c.5435C>T	Unverified	TTC	TCG	GGG	2	C	T
TCGA-30-1856	DIS3	ENST00000377767	p.D554D	c.1662C>T	Unverified	TGT	GAC	GTG	3	C	T
TCGA-25-2401	DISP2	ENST00000267889	p.R1337W	c.4009C>T	Verified	AAG	CGG	GAC	1	C	T
TCGA-13-1510	DKFZP434P1	ENST00000305663	p.F34F	c.102C>T	Unverified	GGC	TTC	CTT	3	C	T
TCGA-24-1844	DKK2	ENST00000285311	p.R230C	c.688C>T	Unverified	cag	cgt	tgc	1	C	T
TCGA-20-1686	DLGAP1	ENST00000315677	p.H216H	c.648C>T	Unverified	TAC	CAC	GCC	3	C	T
TCGA-09-0369	DLGAP2_EN	ENST00000356067	p.N289N	c.867C>T	Verified	CGC	AGC	TCC	3	C	T
TCGA-09-0369	DLGAP2_EN	ENST00000356067	p.N289N	c.867C>T	Verified	CGC	AGC	TCC	3	C	T
TCGA-13-1408	DLGAP3	ENST00000373347	p.H238Y	c.712C>T	Verified	CGG	CAC	GGC	1	C	T
TCGA-13-0883	DLK1	ENST00000341267	p.G161G	c.483C>T	Verified	CCT	GGC	TTC	3	C	T
TCGA-13-0913	DLK1	ENST00000341267	p.D380D	c.1140C>T	Verified	GGC	GAC	GAG	3	C	T
TCGA-23-1122	DMBT1_ENS	ENST00000368915	p.L1723L	c.5169C>T	Verified	TGG	CTC	TCC	3	C	T
TCGA-24-2280	DMP1	ENST00000339673	p.D112D	c.336C>T	Unverified	gat	gac	acc	3	C	T
TCGA-61-1900	DNAH1	ENST00000273600	p.A438V	c.1313C>T	Unverified	CTT	GCC	AGA	2	C	T

TCGA-61-1900	DNAH1_ENS	ENST00000420323	p.A438V	c.1313C>T	Unverified	CTT	GCC	AGA	2	C	T
TCGA-24-1469	DNAH17	ENST00000300671	p.I2196I	c.6588C>T	Verified	TGG	ATC	ATC	3	C	T
TCGA-29-1690	DNAH2	ENST00000389173	p.R330C	c.988C>T	Unverified	TCT	CGT	CAA	1	C	T
TCGA-29-1762	DNAH2	ENST00000389173	p.A25V	c.74C>T	Unverified	CGG	GCC	ACT	2	C	T
TCGA-20-0990	DNAH5	ENST00000265104	p.R3197W	c.9589C>T	Verified	GTG	CGG	ACC	1	C	T
TCGA-61-1914	DNAH5	ENST00000265104	p.N119N	c.357C>T	Unverified	GGA	AAC	GAT	3	C	T
TCGA-25-1318	DNAH9	ENST00000262442	p.P935L	c.2804C>T	Verified	TAT	CCG	TCT	2	C	T
TCGA-25-1318	DNAH9	ENST00000262442	p.P935L	c.2804C>T	Verified	TAT	CCG	TCT	2	C	T
TCGA-61-1733	DNAH9	ENST00000262442	p.A2449V	c.7346C>T	Unverified	CAG	GCG	TGT	2	C	T
TCGA-61-1733	DNAH9	ENST00000262442	p.A2449V	c.7346C>T	Unverified	CAG	GCG	TGT	2	C	T
TCGA-24-1435	DNAH9	ENST00000262442	p.N2504N	c.7512C>T	Verified	AAA	AAC	GTG	3	C	T
TCGA-13-1496	DNAI2	ENST00000311014	p.H363Y	c.1087C>T	Verified	GGC	CAT	CAT	1	C	T
TCGA-04-1347	DNAI2	ENST00000311014	p.Y199Y	c.597C>T	Verified	TCA	TAC	ATC	3	C	T
TCGA-13-0762	DNAJC18	ENST00000302060	p.D17D	c.51C>T	Verified	ATT	GAC	GCA	3	C	T
TCGA-13-0762	DNAJC18	ENST00000302060	p.D17D	c.51C>T	Verified	ATT	GAC	GCA	3	C	T
TCGA-09-2049	DNAJC22	ENST00000549441	p.L178L	c.534C>T	Verified	ccg	ctc	agt	3	C	T
TCGA-24-1847	DNAJC22	ENST00000549441	p.P333P	c.999C>T	Unverified	caa	ccc	agg	3	C	T
TCGA-23-1809	DNAJC5	ENST00000360864	p.T117M	c.350C>T	Unverified	ctc	acg	tgc	2	C	T
TCGA-30-1718	DNAJC5	ENST00000360864	p.N25N	c.75C>T	Unverified	aag	aac	gca	3	C	T
TCGA-29-1703	DNAJC6	ENST00000395325	p.R766C	c.2296C>T	Unverified	gaa	cgt	ggg	1	C	T
TCGA-29-1691	DNALI1	ENST00000296218	p.A7V	c.20C>T	Unverified	AAG	GCC	CAC	2	C	T
TCGA-24-1846	DNHD1	ENST00000254579	p.S4675F	c.14024C>T	Unverified	CCT	TCC	AGC	2	C	T
TCGA-13-1497	DNMT3A	ENST00000321117	p.L737F	c.2209C>T	Verified	cgc	ctc	ctg	1	C	T
TCGA-23-1022	DOC2A	ENST00000350119	p.A65V	c.194C>T	Verified	CCT	GCA	GCC	2	C	T
TCGA-23-1110	DOCK1	NM_001380.2	p.R279*	c.835C>T	Verified	ttg	cga	gcc	1	C	T
TCGA-24-1846	DOCK10	ENST00000258390	p.S1984L	c.5951C>T	Unverified	CTG	TCG	GGC	2	C	T
TCGA-24-1846	DOCK10_EN	ENST00000373702	p.S493L	c.1478C>T	Unverified	CTG	TCG	GGC	2	C	T
TCGA-20-0990	DOCK3_ENS	ENST00000266037	p.R202C	c.604C>T	Verified	ATG	CGC	CCA	1	C	T
TCGA-04-1337	DOCK6	ENST00000319867	p.P1926S	c.5776C>T	Verified	GAC	CCA	CCA	1	C	T
TCGA-61-1899	DOCK6	ENST00000319867	p.P482L	c.1445C>T	Unverified	CGC	CCG	TCG	2	C	T
TCGA-24-1846	DOCK6	ENST00000319867	p.H1882H	c.5646C>T	Unverified	GAC	CAC	GCC	3	C	T
TCGA-20-0990	DOCK8	ENST00000432829	p.R1965C	c.5893C>T	Verified	AAG	CGT	CTC	1	C	T
TCGA-24-1849	DOK7_ENST	ENST00000389653	p.P540P	c.1620C>T	Unverified	ATC	CCC	GTC	3	C	T
TCGA-29-1696	DOPEY2	ENST00000399151	p.L367F	c.1099C>T	Unverified	GTC	CTC	ATC	1	C	T

TCGA-29-1696	DOPEY2	ENST00000399151	p.L367F	c.1099C>T	Unverified	GTC	CTC	ATC	1	C	T
TCGA-24-1846	DPP4	ENST00000360534	p.V233V	c.699C>T	Unverified	GAA	GTC	CCA	3	C	T
TCGA-04-1652	DPP6	ENST00000404039	p.T268T	c.804C>T	Unverified	TAC	ACC	GGC	3	C	T
TCGA-04-1652	DPP6	ENST00000404039	p.T268T	c.804C>T	Unverified	TAC	ACC	GGC	3	C	T
TCGA-04-1652	DPP6_ENST(	ENST00000332007	p.T270T	c.810C>T	Unverified	TAC	ACC	GGC	3	C	T
TCGA-04-1652	DPP6_ENST(	ENST00000332007	p.T270T	c.810C>T	Unverified	TAC	ACC	GGC	3	C	T
TCGA-04-1652	DPP6_ENST(	ENST00000377770	p.T332T	c.996C>T	Unverified	TAC	ACC	GGC	3	C	T
TCGA-04-1652	DPP6_ENST(	ENST00000377770	p.T332T	c.996C>T	Unverified	TAC	ACC	GGC	3	C	T
TCGA-61-2113	DPP7	ENST00000371579	p.A193V	c.578C>T	Unverified	GTG	GCA	GGC	2	C	T
TCGA-13-0760	DQX1	ENST00000393951	p.P116L	c.347C>T	Verified	TCC	CCC	ATC	2	C	T
TCGA-29-1699	DRP2	ENST00000402866	p.T506M	c.1517C>T	Unverified	GGC	ACG	GAA	2	C	T
TCGA-04-1356	DRP2	ENST00000402866	p.G156G	c.468C>T	Verified	CGG	GGC	CCC	3	C	T
TCGA-24-2267	DSC1	ENST00000257197	p.S643F	c.1928C>T	Verified	TAT	TCT	GTG	2	C	T
TCGA-04-1655	DSCAM	ENST00000400454	p.G381G	c.1143C>T	Unverified	GGG	GGC	GCA	3	C	T
TCGA-24-2280	DSCAML1	ENST00000321322	p.T2092I	c.6275C>T	Unverified	AGC	ACA	TCG	2	C	T
TCGA-04-1331	DSEL	ENST00000310045	p.T46I	c.137C>T	Verified	TTC	ACA	GAT	2	C	T
TCGA-23-1031	DSG3	ENST00000257189	p.S512S	c.1536C>T	Verified	GTC	TCC	GCT	3	C	T
TCGA-25-2391	DSP	ENST00000379802	p.V860V	c.2580C>T	Unverified	AAA	GTC	ACA	3	C	T
TCGA-29-1703	DST	ENST00000370765	p.L2508L	c.7522C>T	Unverified	GGC	CTG	GTT	1	C	T
TCGA-29-1703	DST_ENST0(	ENST00000370765	p.L2508L	c.7522C>T	Unverified	GGC	CTG	GTT	1	C	T
TCGA-04-1652	DTX3L	ENST00000296161	p.L553L	c.1657C>T	Unverified	GAA	CTG	GAC	1	C	T
TCGA-04-1652	DTX3L	ENST00000296161	p.L553L	c.1657C>T	Unverified	GAA	CTG	GAC	1	C	T
TCGA-29-1775	DTX3L	ENST00000296161	p.P10L	c.29C>T	Unverified	TCC	CCG	CTC	2	C	T
TCGA-23-1110	DUOX1	ENST00000389037	p.I962I	c.2886C>T	Verified	ATG	ATC	TGT	3	C	T
TCGA-04-1651	DUPD1	ENST00000338487	p.D167D	c.501C>T	Unverified	AAG	GAC	ATG	3	C	T
TCGA-04-1651	DUPD1	ENST00000338487	p.D167D	c.501C>T	Unverified	AAG	GAC	ATG	3	C	T
TCGA-24-1423	DUPD1	ENST00000338487	p.D133D	c.399C>T	Verified	ATC	GAC	AGA	3	C	T
TCGA-61-1907	DUSP27	ENST00000361200	p.G228G	c.684C>T	Unverified	ATG	GGC	ATC	3	C	T
TCGA-24-1469	DYNC2H1_E	ENST00000398093	p.F222F	c.666C>T	Verified	AAC	TTC	ATG	3	C	T
TCGA-24-1850	DYRK2	ENST00000344096	p.R386C	c.1156C>T	Unverified	TCG	CGT	TTT	1	C	T
TCGA-24-1849	DYSF	ENST00000258104	p.R253W	c.757C>T	Unverified	ACG	CGG	ATC	1	C	T
TCGA-24-1849	DYSF_ENST(	ENST00000410020	p.R285W	c.853C>T	Unverified	ACG	CGG	ATC	1	C	T
TCGA-13-0913	DYTN	ENST00000452335	p.A558V	c.1673C>T	Verified	GGA	GCT	CAG	2	C	T
TCGA-13-0793	EBP	ENST00000495186	p.A54V	c.161C>T	Verified	gct	gcg	gtt	2	C	T

TCGA-25-2398	ECE2	ENST00000359140	p.T266M	c.797C>T	Unverified	TCC	ACG	AGG	2	C	T
TCGA-25-2398	ECE2_ENSTC	ENST00000402825	p.T413M	c.1238C>T	Unverified	TCC	ACG	AGG	2	C	T
TCGA-09-2051	EDC4	ENST00000358933	p.R860*	c.2578C>T	Unverified	CAC	CGA	CCA	1	C	T
TCGA-25-2401	EFHB_ENSTC	ENST00000295824	p.A778V	c.2333C>T	Verified	ATT	GCA	GAG	2	C	T
TCGA-25-2400	EFNB1	ENST00000204961	p.R266C	c.796C>T	Unverified	cta	cgc	aag	1	C	T
TCGA-13-0923	EFNB2	ENST00000245323	p.P261L	c.782C>T	Verified	TCG	CCG	CAG	2	C	T
TCGA-29-1775	EFTUD2	ENST00000426333	p.R220C	c.658C>T	Unverified	TTG	CGC	ATC	1	C	T
TCGA-24-1844	EGFL4	ENST00000251268	p.P1589S	c.4765C>T	Unverified	ATG	CCG	GTG	1	C	T
TCGA-13-0887	EGFLAM	ENST00000322350	p.C810C	c.2430C>T	Verified	GAC	TGC	CCC	3	C	T
TCGA-23-2078	EGLN2	ENST00000303961	p.R187W	c.559C>T	Verified	atg	cgg	tac	1	C	T
TCGA-61-2012	EHD4	ENST00000220325	p.A505V	c.1514C>T	Verified	TTC	GCG	CTG	2	C	T
TCGA-10-0930	EHD4	ENST00000220325	p.N133N	c.399C>T	Verified	GGA	AAC	GCT	3	C	T
TCGA-10-0930	EHD4	ENST00000220325	p.N133N	c.399C>T	Verified	GGA	AAC	GCT	3	C	T
TCGA-24-1431	EHMT1	ENST00000298728	p.T616T	c.1848C>T	Unverified	GAC	ACC	ACC	3	C	T
TCGA-13-0887	EHMT2	ENST00000375537	p.S119F	c.356C>T	Verified	TCT	TCC	CCC	2	C	T
TCGA-29-1699	EIF2B1	ENST00000424014	p.D274D	c.822C>T	Unverified	GTC	GAC	TAC	3	C	T
TCGA-09-2051	EIF3B	ENST00000397011	p.G536G	c.1608C>T	Unverified	AAA	GGC	ACC	3	C	T
TCGA-13-0755	EIF4EBP3	ENST00000310331	p.A54V	c.161C>T	Verified	ATT	GCC	CGG	2	C	T
TCGA-23-2077	ELF3	ENST00000359651	p.P30S	c.88C>T	Unverified	GTT	CCC	CCT	1	C	T
TCGA-13-1505	ELF4	ENST00000335997	p.P360S	c.1078C>T	Verified	CAG	CCA	TCT	1	C	T
TCGA-25-1326	ELK3	ENST00000228741	p.C112C	c.336C>T	Unverified	gac	tgc	aag	3	C	T
TCGA-04-1336	ELL3	ENST00000319359	p.R320C	c.958C>T	Verified	GCC	CGT	GTT	1	C	T
TCGA-13-0795	ELMO1	ENST00000310758	p.I280I	c.840C>T	Verified	GTC	ATC	CGA	3	C	T
TCGA-29-1770	ELMO3	ENST00000393997	p.A64A	c.192C>T	Unverified	ATT	GCC	ATC	3	C	T
TCGA-30-1714	ELOVL2	ENST00000354666	p.T218M	c.653C>T	Unverified	ATC	ACG	CAC	2	C	T
TCGA-13-0904	EMID2	ENST00000397927	p.F27F	c.81C>T	Unverified	CCC	TTC	TCG	3	C	T
TCGA-23-1116	EMX2	ENST00000369201	p.A59V	c.176C>T	Unverified	GCC	GCC	GGT	2	C	T
TCGA-24-1469	ENO1	ENST00000234590	p.F178F	c.534C>T	Verified	aac	ttc	agg	3	C	T
TCGA-23-1022	ENPP1	ENST00000360971	p.R59C	c.175C>T	Verified	GGT	CGC	TGT	1	C	T
TCGA-25-2400	ENPP7	ENST00000328313	p.Y388Y	c.1164C>T	Unverified	GTG	TAC	GAG	3	C	T
TCGA-13-0792	ENSG00001	ENST00000314191	p.A2031V	c.6092C>T	Unverified	TTG	GCA	GAC	2	C	T
TCGA-04-1652	ENSG00001	ENST00000324457	p.R67C	c.199C>T	Unverified	TCC	CGC	GTG	1	C	T
TCGA-04-1652	ENSG00001	ENST00000324457	p.R67C	c.199C>T	Unverified	TCC	CGC	GTG	1	C	T
TCGA-29-1785	ENSG00001	ENST00000456556	p.A1627V	c.4880C>T	Unverified	CAA	GCT	GAG	2	C	T

TCGA-29-1785	ENSG00001	ENST00000456556	p.A1627V	c.4880C>T	Unverified	CAA	GCT	GAG	2	C	T
TCGA-29-1693	ENSG00001	ENST00000330252	p.T101M	c.302C>T	Unverified	ACC	ACG	TGG	2	C	T
TCGA-13-0887	ENSG00001	ENST00000334646	p.P165L	c.494C>T	Verified	CTG	CCC	TTC	2	C	T
TCGA-24-1470	ENSG00001	ENST00000341569	p.T135M	c.404C>T	Verified	GTT	ACG	TTG	2	C	T
TCGA-20-1685	ENSG00002	ENST00000400226	p.A246V	c.737C>T	Unverified	CAG	GCG	CTG	2	C	T
TCGA-29-1711	ENSG00002	ENST00000402510	p.T1498T	c.4494C>T	Unverified	TTC	ACC	CAG	3	C	T
TCGA-29-1711	ENSG00002	ENST00000402510	p.T1498T	c.4494C>T	Unverified	TTC	ACC	CAG	3	C	T
TCGA-20-1687	ENSG00002	ENST00000395906	p.S235F	c.704C>T	Unverified	ATA	TCC	GAA	2	C	T
TCGA-13-0920	ENTHD1	ENST00000325157	p.N27N	c.81C>T	Verified	TCT	AAC	GAC	3	C	T
TCGA-09-2050	EP400	ENST00000389561	p.A1150A	c.3450C>T	Verified	TGG	GCC	GAA	3	C	T
TCGA-24-1464	EPB41L1	ENST00000338074	p.H37H	c.111C>T	Verified	GGC	CAC	GGC	3	C	T
TCGA-61-2009	EPB41L1	ENST00000338074	p.G581G	c.1743C>T	Verified	ACA	GGC	GAT	3	C	T
TCGA-25-1313	EPB41L3	ENST00000341928	p.T752M	c.2255C>T	Verified	GTA	ACG	AAT	2	C	T
TCGA-61-1740	EPDR1	ENST00000559325	p.S65F	c.194C>T	Unverified	GCG	TCC	GGA	2	C	T
TCGA-29-1785	EPG5	ENST00000282041	p.P247L	c.740C>T	Unverified	CTC	CCG	TCT	2	C	T
TCGA-29-1785	EPG5	ENST00000282041	p.P247L	c.740C>T	Unverified	CTC	CCG	TCT	2	C	T
TCGA-13-1497	EPHA1	ENST00000275815	p.R966C	c.2896C>T	Verified	AAG	CGC	ATT	1	C	T
TCGA-30-1718	EPHA10	CGP_EPHA10.1	p.T516T	c.1548C>T	Unverified	gtc	acc	gtc	3	C	T
TCGA-29-1699	EPHA3	ENST00000336596	p.V543V	c.1629C>T	Unverified	gtg	gtc	atg	3	C	T
TCGA-13-0920	EPHA5	ENST00000273854	p.C610C	c.1830C>T	Verified	CTG	TGC	GCT	3	C	T
TCGA-61-2095	EPHA6	SU_EPHA6	p.P730S	c.2188C>T	Unverified	cat	cca	gtg	1	C	T
TCGA-04-1655	EPHB1	ENST00000398015	p.T387M	c.1160C>T	Unverified	CTG	ACG	GAG	2	C	T
TCGA-04-1655	EPHB1_ENST	ENST00000398015	p.T387M	c.1160C>T	Unverified	CTG	ACG	GAG	2	C	T
TCGA-29-1766	EPHB2	NM_017449	p.R79W	c.235C>T	Unverified	atc	cgg	cgc	1	C	T
TCGA-29-1766	EPHB2_ENST	ENST00000374632	p.R79W	c.235C>T	Unverified	ATC	CGG	CGC	1	C	T
TCGA-61-2008	EPOR	ENST00000222139	p.T466I	c.1397C>T	Verified	TCA	ACT	GAC	2	C	T
TCGA-61-1904	EPSTI1	ENST00000313640	p.D26D	c.78C>T	Unverified	CAG	GAC	CCT	3	C	T
TCGA-61-1910	EPSTI1	ENST00000313640	p.L301L	c.903C>T	Unverified	GGC	CTC	GAG	3	C	T
TCGA-24-1463	EPX	ENST00000225371	p.R645*	c.1933C>T	Verified	GCC	CGA	GAC	1	C	T
TCGA-13-0884	ERC1	ENST00000397203	p.Q827*	c.2479C>T	Verified	cag	cag	cta	1	C	T
TCGA-09-2044	ERCC4	ENST00000311895	p.S780S	c.2340C>T	Verified	atc	tcc	agc	3	C	T
TCGA-13-0905	ERCC4	ENST00000311895	p.D457D	c.1371C>T	Verified	gaa	gac	agt	3	C	T
TCGA-30-1891	ERCC5	ENST00000355739	p.S1078F	c.3233C>T	Unverified	GAT	TCT	AAA	2	C	T
TCGA-13-0885	ERCC5	ENST00000355739	p.A1165A	c.3495C>T	Verified	ACC	GCC	AGA	3	C	T

TCGA-24-1104	ERCC6	ENST00000355832	p.Q524*	c.1570C>T	Verified	TGC	CAG	CAG	1	C	T
TCGA-04-1336	ERG	ENST00000442448	p.S305L	c.914C>T	Verified	CTG	TCG	GAC	2	C	T
TCGA-23-2078	ERMN	ENST00000397283	p.T148I	c.443C>T	Verified	ATT	ACT	GAG	2	C	T
TCGA-04-1542	ESRRG	ENST00000408911	p.S81L	c.242C>T	Verified	gac	tcg	cca	2	C	T
TCGA-61-1906	ESRRG	ENST00000408911	p.P112L	c.335C>T	Unverified	gat	ccc	cag	2	C	T
TCGA-61-1906	ESRRG	ENST00000408911	p.P112L	c.335C>T	Unverified	gat	ccc	cag	2	C	T
TCGA-13-0916	ESX1	ENST00000372588	p.A364V	c.1091C>T	Verified	atg	gcg	cct	2	C	T
TCGA-61-1910	ETV3L	ENST00000454449	p.Y202Y	c.606C>T	Unverified	GTC	TAC	CGA	3	C	T
TCGA-61-1910	ETV3L_ENST	ENST00000454449	p.Y202Y	c.606C>T	Unverified	GTC	TAC	CGA	3	C	T
TCGA-13-1408	EVC2	ENST00000344408	p.A785V	c.2354C>T	Unverified	ATG	GCT	GCA	2	C	T
TCGA-13-1496	EVC2	ENST00000344408	p.A814V	c.2441C>T	Verified	GAC	GCT	CCT	2	C	T
TCGA-13-0919	EVI2B	NM_006495.2	p.T87I	c.260C>T	Verified	tat	acc	tct	2	C	T
TCGA-25-1326	EVI2B	NM_006495.2	p.S150L	c.449C>T	Verified	caa	tca	tca	2	C	T
TCGA-61-1915	EXOC8	ENST00000360394	p.F541F	c.1623C>T	Unverified	ACC	TTC	ATC	3	C	T
TCGA-61-2012	EXTL1	ENST00000374280	p.L30F	c.88C>T	Unverified	CTT	CTC	CGC	1	C	T
TCGA-13-1512	F11R	ENST00000289779	p.L14F	c.40C>T	Verified	TGC	CTC	TTC	1	C	T
TCGA-24-1104	F2	ENST00000311907	p.R198C	c.592C>T	Verified	CCA	CGC	TCC	1	C	T
TCGA-04-1367	F8	ENST00000360256	p.R1715*	c.5143C>T	Verified	ACA	CGA	CAC	1	C	T
TCGA-04-1367	F8_ENST000	ENST00000360256	p.R1715*	c.5143C>T	Verified	ACA	CGA	CAC	1	C	T
TCGA-04-1331	FAAH2	ENST00000374900	p.R148C	c.442C>T	Verified	AAC	CGT	CGT	1	C	T
TCGA-20-0990	FAHD2A	ENST00000233379	p.A163A	c.489C>T	Verified	CTG	GCC	GTG	3	C	T
TCGA-23-1021	FAIM2	ENST00000320634	p.C223C	c.669C>T	Verified	tcc	tgc	cag	3	C	T
TCGA-23-1116	FAM102A	ENST00000373095	p.T317T	c.951C>T	Unverified	CCG	ACC	TGG	3	C	T
TCGA-23-1021	FAM113A	ENST00000360652	p.P201P	c.603C>T	Verified	CAG	CCC	CTG	3	C	T
TCGA-29-1703	FAM120C	ENST00000375180	p.L754F	c.2260C>T	Unverified	ATG	CTC	AAT	1	C	T
TCGA-09-0369	FAM123B_E	ENST00000330258	p.G877G	c.2631C>T	Verified	CCT	GGC	CTG	3	C	T
TCGA-09-0369	FAM123B_E	ENST00000330258	p.G877G	c.2631C>T	Verified	CCT	GGC	CTG	3	C	T
TCGA-29-1693	FAM134B	ENST00000306320	p.V122V	c.366C>T	Unverified	TCC	GTC	ATG	3	C	T
TCGA-13-0760	FAM135B	ENST00000395297	p.I398I	c.1194C>T	Verified	GAC	ATC	GAC	3	C	T
TCGA-61-2095	FAM161A_E	ENST00000405894	p.L15L	c.45C>T	Unverified	AGT	CTC	CAG	3	C	T
TCGA-25-1326	FAM164A	ENST00000263849	p.L219F	c.655C>T	Verified	AAA	CTT	CAG	1	C	T
TCGA-13-1496	FAM184A	ENST00000338891	p.T723M	c.2168C>T	Verified	TTT	ACG	CAA	2	C	T
TCGA-23-1022	FAM187B	ENST00000324675	p.L92F	c.274C>T	Verified	GGC	CTC	TAC	1	C	T
TCGA-29-1699	FAM187B	ENST00000324675	p.F296F	c.888C>T	Unverified	CAG	TTC	AAA	3	C	T

TCGA-24-1469	FAM193A	ENST00000382839	p.S858S	c.2574C>T	Verified	AGC	TCC	GAA	3	C	T
TCGA-20-1686	FAM21C	ENST00000374362	p.S637F	c.1910C>T	Unverified	GCA	TCT	GAC	2	C	T
TCGA-61-1725	FAM47A	ENST00000346193	p.R500W	c.1498C>T	Unverified	cgt	cgg	acg	1	C	T
TCGA-61-2095	FAM50A	ENST00000393600	p.I252I	c.756C>T	Unverified	tac	atc	aag	3	C	T
TCGA-09-1674	FAM5B	ENST00000361539	p.R524C	c.1570C>T	Unverified	AGC	CGC	ATT	1	C	T
TCGA-04-1367	FAM63A	ENST00000361936	p.P374L	c.1121C>T	Verified	GGG	CCT	GGA	2	C	T
TCGA-23-1029	FAM65A	ENST00000042381	p.S688S	c.2064C>T	Unverified	CTC	TCC	AGC	3	C	T
TCGA-13-1507	FAM65C	ENST00000327979	p.P750S	c.2248C>T	Verified	CTC	CCC	GGA	1	C	T
TCGA-24-2290	FAM71D	ENST00000311864	p.S172S	c.516C>T	Verified	ATC	TCC	CTC	3	C	T
TCGA-24-2290	FAM71D	ENST00000311864	p.S172S	c.516C>T	Verified	ATC	TCC	CTC	3	C	T
TCGA-30-1855	FAM75D1	ENST00000344803	p.L143L	c.427C>T	Unverified	CAA	CTG	CTG	1	C	T
TCGA-30-1855	FAM75D4	ENST00000344803	p.L143L	c.427C>T	Unverified	CAA	CTG	CTG	1	C	T
TCGA-04-1367	FAM83A	ENST00000518448	p.A109V	c.326C>T	Verified	gtg	gcc	tca	2	C	T
TCGA-04-1331	FAM83D	ENST00000217429	p.S503S	c.1509C>T	Unverified	TCT	TCC	AGT	3	C	T
TCGA-61-1904	FAM83E	ENST00000263266	p.P57S	c.169C>T	Unverified	TGG	CCC	TTC	1	C	T
TCGA-61-1907	FAM90A20	ENST00000360191	p.V441V	c.1323C>T	Unverified	CGT	GTC	CCA	3	C	T
TCGA-13-1499	FANCE	ENST00000229769	p.H236Y	c.706C>T	Verified	GAA	CAT	AAG	1	C	T
TCGA-13-0904	FANCG	ENST00000378643	p.Y367Y	c.1101C>T	Verified	cat	tac	ttg	3	C	T
TCGA-24-2267	FANCL	ENST00000233741	p.T367I	c.1100C>T	Verified	ATT	ACC	TTA	2	C	T
TCGA-24-1469	FAP	ENST00000188790	p.P245L	c.734C>T	Verified	TAT	CCT	AGA	2	C	T
TCGA-25-1326	FARP2	ENST00000264042	p.L578L	c.1732C>T	Verified	ACG	CTG	CTC	1	C	T
TCGA-04-1347	FAT1	ENST00000441802	p.A250V	c.749C>T	Verified	CAG	GCC	AAT	2	C	T
TCGA-24-1470	FAT1	ENST00000441802	p.S172S	c.516C>T	Verified	GTC	AGC	GCC	3	C	T
TCGA-61-1725	FAT2	ENST00000261800	p.Q2016*	c.6046C>T	Unverified	GTC	CAG	TCA	1	C	T
TCGA-13-1488	FAT3_ENST(	ENST00000298047_v61	p.A100V	c.299C>T	Unverified	ATT	GCA	GAT	2	C	T
TCGA-29-1699	FAT3_ENST(	ENST00000298047_v61	p.C2360C	c.7080C>T	Unverified	CAC	TGC	ACT	3	C	T
TCGA-13-1488	FAT3_ENST(	ENST00000409404	p.A100V	c.299C>T	Unverified	ATT	GCA	GAT	2	C	T
TCGA-29-1699	FAT3_ENST(	ENST00000409404	p.C2360C	c.7080C>T	Unverified	CAC	TGC	ACT	3	C	T
TCGA-13-1498	FAT4	ENST00000330166	p.P4292L	c.12875C>T	Verified	ATT	CCA	CCC	2	C	T
TCGA-61-2102	FAT4	ENST00000330166	p.S1342S	c.4026C>T	Verified	GAT	TCC	GAT	3	C	T
TCGA-13-1498	FAT4_ENST(	ENST00000394329	p.P4349L	c.13046C>T	Verified	ATT	CCA	CCC	2	C	T
TCGA-61-2102	FAT4_ENST(	ENST00000394329	p.S1342S	c.4026C>T	Verified	GAT	TCC	GAT	3	C	T
TCGA-13-1498	FBN1	ENST00000316623	p.D2567D	c.7701C>T	Verified	GAA	GAC	GTG	3	C	T
TCGA-24-2280	FBN1	ENST00000316623	p.S2613S	c.7839C>T	Unverified	CTC	AGC	GCT	3	C	T

TCGA-09-2056	FBN2	ENST00000262464	p.T1748I	c.5243C>T	Verified	ACC	ACT	TGT	2	C	T
TCGA-61-2009	FBN2	ENST00000262464	p.I633I	c.1899C>T	Verified	TGC	ATC	AAT	3	C	T
TCGA-13-1499	FBN3	ENST00000270509	p.P221S	c.661C>T	Verified	CTT	CCA	TGT	1	C	T
TCGA-13-0760	FBN3	ENST00000270509	p.T404T	c.1212C>T	Verified	GCT	ACC	CTG	3	C	T
TCGA-13-1499	FBN3	ENST00000270509	p.C846C	c.2538C>T	Verified	CCC	TGC	GAA	3	C	T
TCGA-29-1762	FBXL13	ENST00000436908	p.R513*	c.1537C>T	Unverified	TTA	CGA	AAT	1	C	T
TCGA-24-1423	FBXL19	NM_019085.1	p.R408C	c.1222C>T	Verified	ctg	cgt	ctg	1	C	T
TCGA-29-1699	FBXL4	ENST00000369244	p.S618S	c.1854C>T	Unverified	aag	agc	ttt	3	C	T
TCGA-13-1497	FBXL7	ENST00000504595	p.R152W	c.454C>T	Verified	ccg	cgg	ctc	1	C	T
TCGA-23-1123	FBXL7	ENST00000504595	p.T160M	c.479C>T	Verified	ctg	acg	ggc	2	C	T
TCGA-23-1123	FBXL7	ENST00000504595	p.T160M	c.479C>T	Verified	ctg	acg	ggc	2	C	T
TCGA-24-1847	FBXO18	ENST00000379999	p.Q215*	c.643C>T	Unverified	agg	caa	gaa	1	C	T
TCGA-23-1110	FBXO46	ENST00000317683	p.F503F	c.1509C>T	Unverified	cac	ttc	aag	3	C	T
TCGA-24-1469	FBXO7	ENST00000266087	p.T217I	c.650C>T	Verified	GGC	ACC	GAA	2	C	T
TCGA-04-1342	FCAR	ENST00000355524	p.L238L	c.714C>T	Verified	GTC	CTC	GTG	3	C	T
TCGA-24-0975	FCGBP	ENST00000221347	p.D4257D	c.12771C>T	Unverified	TGG	GAC	GAA	3	C	T
TCGA-61-1904	FCGBP	ENST00000221347	p.Y198Y	c.594C>T	Unverified	CCC	TAC	AAT	3	C	T
TCGA-13-1489	FCRL1	ENST00000368176	p.A60A	c.180C>T	Verified	CGG	GCC	TTG	3	C	T
TCGA-24-2280	FCRL5	ENST00000361835	p.Y447Y	c.1341C>T	Verified	TAC	TAC	TGC	3	C	T
TCGA-13-1501	FER1L5	ENST00000505256	p.Y1394Y	c.4182C>T	Verified	CTC	TAC	AGA	3	C	T
TCGA-13-1501	FER1L5	ENST00000505256	p.Y1394Y	c.4182C>T	Verified	CTC	TAC	AGA	3	C	T
TCGA-04-1530	FER1L6	ENST00000399018	p.G810G	c.2430C>T	Verified	GCT	GGC	ACC	3	C	T
TCGA-61-1740	FERD3L	ENST00000275461	p.R82R	c.246C>T	Unverified	GAG	CGC	GGA	3	C	T
TCGA-24-1464	FERMT3	ENST00000279227	p.R542C	c.1624C>T	Verified	CTG	CGC	TTC	1	C	T
TCGA-13-0760	FFAR3	ENST00000327809	p.N32N	c.96C>T	Unverified	CTC	AAC	CTG	3	C	T
TCGA-23-1123	FGD4	ENST00000427716	p.H197Y	c.589C>T	Verified	CAC	CAT	GAG	1	C	T
TCGA-23-1123	FGD4	ENST00000427716	p.H197Y	c.589C>T	Verified	CAC	CAT	GAG	1	C	T
TCGA-25-2393	FGD4	ENST00000427716	p.R224*	c.670C>T	Verified	AAC	CGA	CTT	1	C	T
TCGA-29-1705	FGF8	ENST00000320185	p.T194M	c.581C>T	Unverified	AAG	ACG	CGG	2	C	T
TCGA-13-1510	FGFRL1	ENST00000398484	p.A94A	c.282C>T	Unverified	GAT	GCC	GGC	3	C	T
TCGA-29-1775	FGGY	ENST00000315089	p.I190I	c.570C>T	Unverified	GGG	ATC	AGC	3	C	T
TCGA-29-1775	FGGY_ENST(	ENST00000303721	p.I302I	c.906C>T	Unverified	GGG	ATC	AGC	3	C	T
TCGA-24-1464	FHL2	ENST00000409177	p.L75L	c.223C>T	Verified	TCA	CTG	GTG	1	C	T
TCGA-13-1496	FHOD3	ENST00000257209	p.A1324V	c.3971C>T	Verified	GAC	GCG	GCT	2	C	T

TCGA-29-1690	FLG	ENST00000368799	p.D2781D	c.8343C>T	Unverified	CAA	GAC	GGT	3	C	T
TCGA-61-1904	FLG	ENST00000368799	p.S3845S	c.11535C>T	Unverified	CAG	TCC	GGC	3	C	T
TCGA-20-0991	FLI1	ENST00000429175	p.Y332Y	c.996C>T	Verified	aat	tac	gac	3	C	T
TCGA-09-2049	FLJ13236	ENST00000395069	p.L178L	c.534C>T	Verified	CCG	CTC	AGT	3	C	T
TCGA-24-1847	FLJ13236	ENST00000395069	p.P333P	c.999C>T	Unverified	CAA	CCC	AGG	3	C	T
TCGA-61-1740	FLJ16165	ENST00000331256	p.S67L	c.200C>T	Unverified	CCG	TCG	GGG	2	C	T
TCGA-10-0930	FLJ90650	ENST00000357872	p.V43V	c.129C>T	Verified	CGC	GTC	CCA	3	C	T
TCGA-10-0930	FLJ90650	ENST00000357872	p.V43V	c.129C>T	Verified	CGC	GTC	CCA	3	C	T
TCGA-29-1696	FLNB	ENST00000295956	p.F1443F	c.4329C>T	Unverified	TCC	TTC	ACG	3	C	T
TCGA-29-1696	FLNB	ENST00000295956	p.F1443F	c.4329C>T	Unverified	TCC	TTC	ACG	3	C	T
TCGA-29-1696	FLNB_ENST(	ENST00000490882	p.F1443F	c.4329C>T	Unverified	TCC	TTC	ACG	3	C	T
TCGA-29-1696	FLNB_ENST(	ENST00000490882	p.F1443F	c.4329C>T	Unverified	TCC	TTC	ACG	3	C	T
TCGA-29-1783	FLNC	ENST00000325888	p.R269*	c.805C>T	Unverified	GTT	CGA	TCC	1	C	T
TCGA-13-0920	FLRT2	ENST00000330753	p.I609I	c.1827C>T	Verified	CAG	ATC	GTC	3	C	T
TCGA-04-1331	FLYWCH2	ENST00000293981	p.N128N	c.384C>T	Unverified	GAG	AAC	TTT	3	C	T
TCGA-23-1124	FMNL2	ENST00000288670	p.S160S	c.480C>T	Verified	GAG	AGC	TCG	3	C	T
TCGA-20-1686	FNDC1	ENST00000297267	p.Y198Y	c.594C>T	Unverified	GGC	TAC	GGG	3	C	T
TCGA-24-1103	FNDC8	ENST00000158009	p.P300P	c.900C>T	Verified	GAA	CCC	CGG	3	C	T
TCGA-25-2400	FOXA3	ENST00000302177	p.R212C	c.634C>T	Unverified	aaa	cgc	ttc	1	C	T
TCGA-29-1785	FOXH1	ENST00000377317	p.S5S	c.15C>T	Unverified	tgc	agc	ggc	3	C	T
TCGA-29-1785	FOXH1	ENST00000377317	p.S5S	c.15C>T	Unverified	tgc	agc	ggc	3	C	T
TCGA-20-1683	FOXJ3	ENST00000372573	p.P387L	c.1160C>T	Unverified	cga	ccg	cat	2	C	T
TCGA-04-1652	FREM1	ENST00000422223	p.R1654C	c.4960C>T	Unverified	TCC	CGC	GTG	1	C	T
TCGA-04-1652	FREM1	ENST00000422223	p.R1654C	c.4960C>T	Unverified	TCC	CGC	GTG	1	C	T
TCGA-13-1505	FREM2	ENST00000280481	p.S402F	c.1205C>T	Verified	GAC	TCT	GAC	2	C	T
TCGA-13-0904	FRG2B	ENST00000425520	p.R214W	c.640C>T	Unverified	CTC	CGG	GGG	1	C	T
TCGA-13-0904	FRG2C	ENST00000308062	p.R215W	c.643C>T	Verified	CTC	CGG	GGG	1	C	T
TCGA-13-0884	FRMPD1	ENST00000377765	p.N915N	c.2745C>T	Verified	ACA	AAC	CCA	3	C	T
TCGA-24-1422	FRMPD4	ENST00000380682	p.A728V	c.2183C>T	Verified	gcc	gcg	gag	2	C	T
TCGA-61-1907	FRRS1	ENST00000287474	p.L459F	c.1375C>T	Unverified	GTT	CTT	CAG	1	C	T
TCGA-13-0906	FRS3	ENST00000259748	p.R121C	c.361C>T	Verified	ACC	CGC	AAT	1	C	T
TCGA-61-1740	FRY	ENST00000380250	p.Y556Y	c.1668C>T	Unverified	TAT	TAC	TCT	3	C	T
TCGA-13-0913	FSCN1	ENST00000382361	p.F394F	c.1182C>T	Verified	GGC	TTC	ATC	3	C	T
TCGA-24-1474	FUBP3	ENST00000319725	p.Y558Y	c.1674C>T	Unverified	TTC	TAC	GGA	3	C	T

TCGA-25-2393	FUT1	ENST00000310160	p.A343V	c.1028C>T	Verified	gag	gcg	gcc	2	C	T
TCGA-29-1703	FUT10	ENST00000327671	p.T236I	c.707C>T	Unverified	ATG	ACT	TAC	2	C	T
TCGA-24-1616	FYB	ENST00000351578	p.P584L	c.1751C>T	Verified	GCC	CCT	TCA	2	C	T
TCGA-29-1777	FZD1	ENST00000287934	p.P313L	c.938C>T	Unverified	ggg	ccc	gag	2	C	T
TCGA-13-0792	G6PD	ENST00000291567	p.P512S	c.1534C>T	Verified	AAC	CCC	CAC	1	C	T
TCGA-24-1469	G6PD	ENST00000291567	p.S180F	c.539C>T	Verified	AGC	TCT	GAC	2	C	T
TCGA-29-2427	GABRA6	ENST00000274545	p.P401L	c.1202C>T	Verified	ACA	CCT	GTC	2	C	T
TCGA-61-2095	GAL3ST3	ENST00000312006	p.H72Y	c.214C>T	Unverified	ACT	CAC	AAG	1	C	T
TCGA-61-1738	GALK2	ENST00000327171	p.R330*	c.988C>T	Unverified	GCG	CGA	GTG	1	C	T
TCGA-61-1738	GALK2	ENST00000327171	p.R330*	c.988C>T	Unverified	GCG	CGA	GTG	1	C	T
TCGA-61-1738	GALK2_ENST	ENST00000560031	p.R341*	c.1021C>T	Unverified	GCG	CGA	GTG	1	C	T
TCGA-61-1738	GALK2_ENST	ENST00000560031	p.R341*	c.1021C>T	Unverified	GCG	CGA	GTG	1	C	T
TCGA-30-1856	GALNT14	ENST00000349752	p.I228I	c.684C>T	Unverified	GTG	ATC	GAT	3	C	T
TCGA-25-1326	GALNT2	ENST00000366672	p.D80D	c.240C>T	Verified	CCA	GAC	TTT	3	C	T
TCGA-20-0991	GALNTL1	ENST00000337827	p.F412F	c.1236C>T	Verified	TCC	TTC	CGC	3	C	T
TCGA-13-0755	GANAB	ENST00000356638	p.C47C	c.141C>T	Verified	TTC	TGC	AAG	3	C	T
TCGA-25-1326	GATA3	ENST00000379328	p.S199F	c.596C>T	Verified	tcg	tcc	cac	2	C	T
TCGA-29-1696	GATC	ENST00000551765	p.A35V	c.104C>T	Unverified	GCT	GCG	GTG	2	C	T
TCGA-29-1696	GATC	ENST00000551765	p.A35V	c.104C>T	Unverified	GCT	GCG	GTG	2	C	T
TCGA-13-1505	GBE1	ENST00000429644	p.D571D	c.1713C>T	Verified	ACT	GAC	GAC	3	C	T
TCGA-24-1563	GBE1	ENST00000429644	p.N58N	c.174C>T	Verified	AAG	AAC	ATT	3	C	T
TCGA-09-0369	GCHFR	ENST00000260447	p.P53L	c.158C>T	Verified	CCT	CCC	CGC	2	C	T
TCGA-09-0369	GCHFR	ENST00000260447	p.P53L	c.158C>T	Verified	CCT	CCC	CGC	2	C	T
TCGA-24-1850	GCK	ENST00000345378	p.R44C	c.130C>T	Unverified	gac	cgc	ggc	1	C	T
TCGA-24-1850	GCK_ENST0(	ENST00000403799	p.R43C	c.127C>T	Unverified	GAC	CGC	GGC	1	C	T
TCGA-24-1464	GCN1L1	ENST00000300648	p.P1020P	c.3060C>T	Verified	TCC	CCC	AAC	3	C	T
TCGA-61-2009	GDAP2	ENST00000369443	p.P429P	c.1287C>T	Verified	CAT	CCC	ACA	3	C	T
TCGA-30-1857	GDF5OS	ENST00000374375	p.Y48Y	c.144C>T	Unverified	AGG	TAC	TCA	3	C	T
TCGA-30-1857	GDF5OS_EN	ENST00000374375_v68	p.Y48Y	c.144C>T	Unverified	AGG	TAC	TCA	3	C	T
TCGA-04-1342	GDF6	ENST00000287020	p.D73D	c.219C>T	Verified	cag	gac	gaa	3	C	T
TCGA-13-0916	GDPD2	ENST00000374382	p.A165V	c.494C>T	Verified	GCA	GCC	GCT	2	C	T
TCGA-13-0761	GEMIN4	ENST00000319004	p.I402I	c.1206C>T	Verified	GAT	ATC	ACA	3	C	T
TCGA-13-0761	GEMIN4	ENST00000319004	p.I402I	c.1206C>T	Verified	GAT	ATC	ACA	3	C	T
TCGA-13-0923	GEMIN4	ENST00000319004	p.H285H	c.855C>T	Unverified	TAC	CAC	CAG	3	C	T

TCGA-24-1843	GFM1	ENST00000486715	p.R164C	c.490C>T	Unverified	AAG	CGC	TAC	1	C	T
TCGA-24-1422	GGT7	ENST00000336431	p.L439L	c.1317C>T	Verified	ATG	CTC	AGC	3	C	T
TCGA-23-2077	GIGYF2	ENST00000373563	p.A793V	c.2378C>T	Verified	GAG	GCT	CTG	2	C	T
TCGA-09-2056	GIMAP4	ENST00000255945	p.T39T	c.117C>T	Verified	AAA	ACC	GGA	3	C	T
TCGA-24-2262	GJA8	ENST00000369235	p.A96V	c.287C>T	Verified	CAC	GCG	GTG	2	C	T
TCGA-29-1690	GJD4	ENST00000321660	p.L87F	c.259C>T	Unverified	CTC	CTC	CCC	1	C	T
TCGA-23-1022	GLDN	ENST00000335449	p.S223S	c.669C>T	Verified	GTG	TCC	AAC	3	C	T
TCGA-23-1120	GLG1	ENST00000205061	p.L880F	c.2638C>T	Unverified	ACC	CTC	ATG	1	C	T
TCGA-61-1914	GLG1	ENST00000205061	p.T783T	c.2349C>T	Unverified	ACG	ACC	GTG	3	C	T
TCGA-09-0369	GLI2	ENST00000361492	p.T648T	c.1944C>T	Unverified	AAG	ACC	GAG	3	C	T
TCGA-09-0369	GLI2	ENST00000361492	p.T648T	c.1944C>T	Unverified	AAG	ACC	GAG	3	C	T
TCGA-61-1737	GLI2	ENST00000361492	p.D1435D	c.4305C>T	Unverified	CAG	GAC	GCA	3	C	T
TCGA-29-1766	GLRA4	XM_497139.1	p.L119F	c.355C>T	Unverified	gac	ctc	gat	1	C	T
TCGA-29-1766	GLRA4_ENS	ENST00000372617	p.L119F	c.355C>T	Unverified	GAC	CTC	GAT	1	C	T
TCGA-23-1111	GLT25D1	ENST00000252599	p.D91D	c.273C>T	Unverified	ACG	GAC	CAC	3	C	T
TCGA-61-1733	GLT8D2	ENST00000360814	p.A179V	c.536C>T	Unverified	GCG	GCG	GCT	2	C	T
TCGA-61-1733	GLT8D2	ENST00000360814	p.A179V	c.536C>T	Unverified	GCG	GCG	GCT	2	C	T
TCGA-13-0924	GMPS	ENST00000496455	p.Q626*	c.1876C>T	Verified	ctt	caa	aag	1	C	T
TCGA-20-1687	GNB1	ENST00000378609	p.C114C	c.342C>T	Unverified	gcc	tgc	ggt	3	C	T
TCGA-25-2042	GNL1	ENST00000376621	p.P467S	c.1399C>T	Verified	GAC	CCC	TCA	1	C	T
TCGA-04-1367	GNL2	ENST00000373062	p.F427F	c.1281C>T	Verified	GCT	TTC	CGG	3	C	T
TCGA-09-1674	GNPTAB	ENST00000299314	p.R46*	c.136C>T	Unverified	AGC	CGA	GAT	1	C	T
TCGA-24-1616	GNPTAB	ENST00000299314	p.Q1075*	c.3223C>T	Verified	ACT	CAG	GAA	1	C	T
TCGA-61-2008	GOLGA5	ENST00000163416	p.L492F	c.1474C>T	Verified	CAG	CTC	AGA	1	C	T
TCGA-24-2024	GOLGB1	ENST00000340645	p.S2612F	c.7835C>T	Verified	ATA	TCC	CAG	2	C	T
TCGA-61-2012	GON4L	ENST00000361040	p.A784V	c.2351C>T	Verified	ACT	GCG	AAT	2	C	T
TCGA-24-1435	GORASP1	ENST00000319283	p.R106C	c.316C>T	Verified	TTC	CGC	AGG	1	C	T
TCGA-61-2109	GOSR1	ENST00000225724	p.S200L	c.599C>T	Unverified	AAG	TCA	ATT	2	C	T
TCGA-29-1777	GP2	ENST00000381362	p.L491L	c.1471C>T	Unverified	GAC	CTA	GCC	1	C	T
TCGA-13-0714	GPATCH2	ENST00000366935	p.P482S	c.1444C>T	Verified	ACG	CCT	GGG	1	C	T
TCGA-09-2050	GPATCH4	ENST00000368232	p.T4T	c.12C>T	Verified	GTC	ACC	CCA	3	C	T
TCGA-13-0886	GPBP1L1	ENST00000290795	p.S126S	c.378C>T	Verified	CAC	TCC	CGG	3	C	T
TCGA-23-2078	GPCPD1	ENST00000379019	p.R629R	c.1887C>T	Unverified	GAA	CGC	CTG	3	C	T
TCGA-24-1616	GPD2	ENST00000310454	p.A708V	c.2123C>T	Verified	ACT	GCA	GAA	2	C	T

TCGA-61-1899	GPR1	ENST00000407325	p.R149*	c.445C>T	Unverified	CAT	CGA	ACC	1	C	T
TCGA-09-1674	GPR110	ENST00000371253	p.R426W	c.1276C>T	Unverified	TCT	CGG	AAA	1	C	T
TCGA-25-1313	GPR110	ENST00000371253	p.L382F	c.1144C>T	Verified	ATC	CTT	AAT	1	C	T
TCGA-04-1336	GPR112	ENST00000394143	p.S549L	c.1646C>T	Verified	atg	tcg	aaa	2	C	T
TCGA-13-1505	GPR113	ENST00000333478	p.A611A	c.1833C>T	Verified	CAC	GCC	GCC	3	C	T
TCGA-29-1690	GPR113	ENST00000333478	p.H515H	c.1545C>T	Unverified	CCT	CAC	TGT	3	C	T
TCGA-29-1770	GPR113	ENST00000333478	p.S425S	c.1275C>T	Unverified	TAC	AGC	ATC	3	C	T
TCGA-29-1690	GPR113_EN	ENST00000311519	p.H714H	c.2142C>T	Unverified	CCT	CAC	TGT	3	C	T
TCGA-29-1770	GPR113_EN	ENST00000311519	p.S624S	c.1872C>T	Unverified	TAC	AGC	ATC	3	C	T
TCGA-24-2289	GPR116	ENST00000265417	p.Q786*	c.2356C>T	Unverified	ACC	CAA	GTA	1	C	T
TCGA-13-0760	GPR119	ENST00000276218	p.L305F	c.913C>T	Verified	ttc	ctc	ctc	1	C	T
TCGA-25-2392	GPR124	ENST00000412232	p.P384P	c.1152C>T	Verified	TAT	CCC	TTC	3	C	T
TCGA-23-1022	GPR128	ENST00000273352	p.P780L	c.2339C>T	Verified	TCT	CCG	AGT	2	C	T
TCGA-23-1118	GPR139	ENST00000570682	p.P256S	c.766C>T	Verified	GCG	CCC	ATC	1	C	T
TCGA-23-1029	GPR142	ENST00000335666	p.F375F	c.1125C>T	Unverified	GTC	TTC	GTC	3	C	T
TCGA-29-1769	GPR155	ENST00000392551	p.T584I	c.1751C>T	Unverified	TTT	ACA	AGC	2	C	T
TCGA-04-1651	GPR158	ENST00000376351	p.R928C	c.2782C>T	Unverified	GAA	CGC	ACT	1	C	T
TCGA-04-1651	GPR158	ENST00000376351	p.R928C	c.2782C>T	Unverified	GAA	CGC	ACT	1	C	T
TCGA-13-0923	GPR173	ENST00000332582	p.V74V	c.222C>T	Unverified	GCC	GTC	TGC	3	C	T
TCGA-61-1998	GPR25	ENST00000304244	p.T326T	c.978C>T	Verified	CGC	ACC	GGC	3	C	T
TCGA-25-1326	GPR27	ENST00000304411	p.L292L	c.876C>T	Verified	CTG	CTC	TTC	3	C	T
TCGA-24-1849	GPR31	ENST00000366834	p.L145L	c.435C>T	Unverified	GCC	CTC	ACC	3	C	T
TCGA-61-1906	GPR34	ENST00000378142	p.P34L	c.101C>T	Unverified	ccg	cca	caa	2	C	T
TCGA-61-1906	GPR34	ENST00000378142	p.P34L	c.101C>T	Unverified	ccg	cca	caa	2	C	T
TCGA-61-1899	GPR37	ENST00000303921	p.N585N	c.1755C>T	Unverified	GAC	AAC	GAG	3	C	T
TCGA-29-2427	GPR63	ENST00000369268	p.N117N	c.351C>T	Verified	ATT	AAC	ATC	3	C	T
TCGA-24-2262	GPR78	ENST00000382487	p.R235C	c.703C>T	Unverified	CGG	CGC	CGC	1	C	T
TCGA-24-2280	GPRC5A	ENST00000014914	p.S196F	c.587C>T	Unverified	TCC	TCC	TTC	2	C	T
TCGA-29-1691	GPRIN1	ENST00000303991	p.C98C	c.294C>T	Unverified	ACC	TGC	TTC	3	C	T
TCGA-13-0900	GPT2	ENST00000340124	p.D97D	c.291C>T	Verified	GGG	GAC	GCC	3	C	T
TCGA-24-1849	GPX6	ENST00000361902	p.V207V	c.621C>T	Unverified	ACA	GTC	AAG	3	C	T
TCGA-04-1331	GRAMD1B	ENST00000322282	p.P415S	c.1243C>T	Verified	CAT	CCA	TGG	1	C	T
TCGA-61-2113	GRAMD1B	ENST00000322282	p.P208S	c.622C>T	Unverified	AAG	CCT	CTG	1	C	T
TCGA-04-1649	GRAMD4	ENST00000361034	p.L312F	c.934C>T	Unverified	AAC	CTT	TTC	1	C	T

TCGA-13-0793	GRB2	ENST00000392564	p.V185V	c.555C>T	Verified	cat	gtc	atg	3	C	T
TCGA-29-1698	GREB1_ENS1	ENST00000381486	p.I1349I	c.4047C>T	Unverified	CAG	ATC	GGG	3	C	T
TCGA-13-1505	GRIA1	ENST00000285900	p.N425N	c.1275C>T	Verified	AAG	AAC	GCC	3	C	T
TCGA-13-0884	GRID1	ENST00000327946	p.G479G	c.1437C>T	Verified	CTG	GGC	TTT	3	C	T
TCGA-24-2290	GRIK3	ENST00000373091	p.T118T	c.354C>T	Verified	TGC	ACC	AAT	3	C	T
TCGA-24-2290	GRIK3	ENST00000373091	p.T118T	c.354C>T	Verified	TGC	ACC	AAT	3	C	T
TCGA-61-2102	GRIK3	ENST00000373091	p.P131P	c.393C>T	Verified	GTG	CCC	CAC	3	C	T
TCGA-04-1530	GRIK3	ENST00000373091	p.Y745Y	c.2235C>T	Unverified	GAG	TAC	GTC	3	C	T
TCGA-09-1665	GRIK4	ENST00000527524	p.I207I	c.621C>T	Verified	GAG	ATC	CGG	3	C	T
TCGA-04-1342	GRIP2	ENST00000273083	p.R951W	c.2851C>T	Unverified	ATG	CGG	CAT	1	C	T
TCGA-25-1318	GRK1	NM_002929.2	p.A14V	c.41C>T	Verified	tct	gcc	ttc	2	C	T
TCGA-25-1318	GRK1	NM_002929.2	p.A14V	c.41C>T	Verified	tct	gcc	ttc	2	C	T
TCGA-13-1505	GRLF1_ENS1	ENST00000317082_v61	p.R1145*	c.3433C>T	Unverified	GAG	CGA	GGG	1	C	T
TCGA-25-2398	GRLF1_ENS1	ENST00000317082_v61	p.Q586*	c.1756C>T	Verified	AAT	CAG	AAA	1	C	T
TCGA-13-0905	GRM6	ENST00000231188	p.P573S	c.1717C>T	Verified	CGC	CCC	ACA	1	C	T
TCGA-24-0979	GRM7	ENST00000389335	p.T377M	c.1130C>T	Verified	TTG	ACG	ATT	2	C	T
TCGA-29-1783	GRM8	ENST00000339582	p.S219L	c.656C>T	Unverified	GTT	TCG	ACA	2	C	T
TCGA-29-1783	GRM8_ENS1	ENST00000405249	p.S219L	c.656C>T	Unverified	GTT	TCG	ACA	2	C	T
TCGA-25-2401	GSG1L	ENST00000380897	p.S71F	c.212C>T	Verified	GGC	TCC	TTT	2	C	T
TCGA-61-2113	GSK3A	ENST00000222330	p.S482S	c.1446C>T	Verified	AAC	TCC	TCC	3	C	T
TCGA-24-2024	GSPT2	ENST00000340438	p.D16D	c.48C>T	Verified	tgg	gac	cag	3	C	T
TCGA-30-1718	GTF2B	ENST00000370500	p.R185*	c.553C>T	Unverified	TCA	CGA	ATT	1	C	T
TCGA-24-2280	GTF2IRD1	ENST00000265755	p.C15C	c.45C>T	Unverified	GGC	TGC	GGA	3	C	T
TCGA-20-1687	GTF2IRD2B	ENST00000312575	p.T876M	c.2627C>T	Unverified	AAC	ACG	GTC	2	C	T
TCGA-61-1998	GTPBP4	ENST00000360803	p.I280I	c.840C>T	Verified	TTC	ATC	AAC	3	C	T
TCGA-24-1847	GTPBP5	ENST00000370823	p.G281G	c.843C>T	Unverified	CCC	GGC	ATC	3	C	T
TCGA-13-0916	GUCY1A2	ENST00000526355	p.Y572Y	c.1716C>T	Verified	GCC	TAC	TGT	3	C	T
TCGA-09-1665	GUCY2C	ENST00000261170	p.T682I	c.2045C>T	Verified	TAC	ACT	TTG	2	C	T
TCGA-23-1116	GYS1	ENST00000323798	p.A215A	c.645C>T	Verified	TGT	GCC	GGT	3	C	T
TCGA-61-1913	GYS1	ENST00000323798	p.F22F	c.66C>T	Unverified	GAA	TTC	GAC	3	C	T
TCGA-13-1507	H2BFM	XM_210048.3	p.R88C	c.262C>T	Verified	aac	cgc	cgt	1	C	T
TCGA-13-1498	HAAO	ENST00000294973	p.S270F	c.809C>T	Verified	GGC	TCT	GTG	2	C	T
TCGA-13-1498	HAL	ENST00000261208	p.V579V	c.1737C>T	Verified	AAG	GTC	TAT	3	C	T
TCGA-13-1489	HAO1	ENST00000378789	p.R4W	c.10C>T	Verified	CCC	CGG	CTA	1	C	T

TCGA-13-0913	HAPLN3	ENST00000359595	p.N174N	c.522C>T	Unverified	CCC	AAC	GGG	3	C	T
TCGA-13-1507	HCAR1	ENST00000356987	p.H121H	c.363C>T	Verified	CCC	CAC	CAC	3	C	T
TCGA-29-1764	HCFC1	NM_005334.1	p.T637T	c.1911C>T	Unverified	acc	acc	atc	3	C	T
TCGA-09-2049	HCFC1_ENST	ENST00000310441	p.R37C	c.109C>T	Verified	CAC	CGC	GCC	1	C	T
TCGA-29-1764	HCFC1_ENST	ENST00000310441	p.T734T	c.2202C>T	Unverified	ACC	ACC	ATC	3	C	T
TCGA-13-0920	HCFC2	ENST00000229330	p.T458M	c.1373C>T	Verified	CTG	ACG	GAT	2	C	T
TCGA-29-1701	HCN3	ENST00000368358	p.G313G	c.939C>T	Unverified	ATT	GGC	TAT	3	C	T
TCGA-61-1913	HCN4	ENST00000261917	p.H512H	c.1536C>T	Unverified	GGC	CAC	GCC	3	C	T
TCGA-13-0904	HDAC6	ENST00000334136	p.L147L	c.439C>T	Verified	agc	cta	gaa	1	C	T
TCGA-24-2288	HDC	ENST00000267845	p.A343A	c.1029C>T	Unverified	GTG	GCC	ACC	3	C	T
TCGA-29-1691	HDX	ENST00000297977	p.L278L	c.832C>T	Unverified	att	ctg	gga	1	C	T
TCGA-61-1895	HECTD1	ENST00000399332	p.R1055*	c.3163C>T	Unverified	GTT	CGA	AAA	1	C	T
TCGA-13-1498	HECW2	ENST00000260983	p.D354D	c.1062C>T	Verified	gat	gac	gag	3	C	T
TCGA-13-1499	HECW2	ENST00000260983	p.R867R	c.2601C>T	Verified	atc	cgc	aga	3	C	T
TCGA-24-1469	HEPH	NM_138737.1	p.A306V	c.917C>T	Verified	aca	gca	ttt	2	C	T
TCGA-13-1497	HEPH	NM_138737.1	p.A525A	c.1575C>T	Verified	cat	gcc	ggt	3	C	T
TCGA-04-1347	HEPHL1	ENST00000315765	p.A7V	c.20C>T	Verified	CCA	GCT	GGC	2	C	T
TCGA-29-1761	HEPN1	ENST00000408930	p.F66F	c.198C>T	Unverified	CTG	TTC	AAT	3	C	T
TCGA-13-0760	HERC1	ENST00000443617	p.H3625Y	c.10873C>T	Verified	GCA	CAT	AAG	1	C	T
TCGA-23-2078	HERC1	ENST00000443617	p.I2785I	c.8355C>T	Verified	AAT	ATC	ACT	3	C	T
TCGA-09-2051	HERC2	NM_004667.3	p.R1725W	c.5173C>T	Unverified	aat	cgg	atg	1	C	T
TCGA-24-1469	HERC2	NM_004667.3	p.L2162L	c.6486C>T	Verified	ggg	ctc	atc	3	C	T
TCGA-61-2095	HERC2	NM_004667.3	p.V1436V	c.4308C>T	Unverified	gag	gtc	ggt	3	C	T
TCGA-24-0979	HERC5	NM_016323.1	p.R885W	c.2653C>T	Verified	ttt	cgg	aga	1	C	T
TCGA-04-1356	HERC6_ENST	ENST00000264346	p.A249V	c.746C>T	Verified	ACT	GCG	GTG	2	C	T
TCGA-61-1733	HERPUD2	ENST00000396081	p.Q316*	c.946C>T	Unverified	CAC	CAA	GCT	1	C	T
TCGA-61-1733	HERPUD2	ENST00000396081	p.Q316*	c.946C>T	Unverified	CAC	CAA	GCT	1	C	T
TCGA-23-1022	HEXA	ENST00000268097	p.Y435Y	c.1305C>T	Verified	TTC	TAC	ATA	3	C	T
TCGA-25-2042	HEYL	ENST00000372852	p.S10S	c.30C>T	Verified	GGC	TCC	GAC	3	C	T
TCGA-30-1718	HGS	ENST00000329138	p.A301V	c.902C>T	Unverified	TCA	GCG	CCC	2	C	T
TCGA-29-1703	HHATL	ENST00000310417	p.G167G	c.501C>T	Unverified	ACA	GGC	ACT	3	C	T
TCGA-23-1122	HIC2	ENST00000407464	p.T577M	c.1730C>T	Unverified	CTC	ACG	GAG	2	C	T
TCGA-61-1740	HIGD2BP	ENST00000311755	p.R41C	c.121C>T	Unverified	CTT	CGC	AAG	1	C	T
TCGA-13-1489	HIPK2	NM_022740	p.T772I	c.2315C>T	Verified	ttg	acc	ggt	2	C	T

TCGA-24-2280	HIST1H1A	ENST00000244573	p.A71V	c.212C>T	Unverified	GCG	GCC	GCA	2	C	T
TCGA-13-0714	HIST1H1C	ENST00000343677	p.A61V	c.182C>T	Verified	gct	gct	ctg	2	C	T
TCGA-23-1111	HIST1H2AG	ENST00000359193	p.L35F	c.103C>T	Unverified	CTG	CTC	CGC	1	C	T
TCGA-61-1733	HIST1H2BC	ENST00000396984	p.V119V	c.357C>T	Unverified	GCC	GTC	ACC	3	C	T
TCGA-61-1733	HIST1H2BC	ENST00000396984	p.V119V	c.357C>T	Unverified	GCC	GTC	ACC	3	C	T
TCGA-13-1488	HIST1H2BH	ENST00000356350	p.P11L	c.32C>T	Verified	GCC	CCG	AAG	2	C	T
TCGA-29-1766	HIST1H2BK	ENST00000356950	p.A118A	c.354C>T	Unverified	AAG	GCC	GTC	3	C	T
TCGA-29-1766	HIST1H2BK_	ENST00000396891	p.A118A	c.354C>T	Unverified	AAG	GCC	GTC	3	C	T
TCGA-13-1499	HIST1H4H	ENST00000377727	p.A16V	c.47C>T	Verified	GGA	GCT	AAG	2	C	T
TCGA-09-2044	HIST2H2AC	ENST00000331380	p.Q25*	c.73C>T	Verified	CTC	CAG	TTC	1	C	T
TCGA-13-1509	HIST2H3PS2	ENST00000369176	p.R9C	c.25C>T	Verified	GCC	CGC	AAG	1	C	T
TCGA-29-1693	HIVEP1	ENST00000379388	p.G1350G	c.4050C>T	Unverified	GCT	GGC	TTG	3	C	T
TCGA-20-0991	HIVEP3	ENST00000372583	p.S1504L	c.4511C>T	Verified	TCC	TCA	GAT	2	C	T
TCGA-29-1775	HK2	ENST00000290573	p.R849C	c.2545C>T	Unverified	aac	cgt	ggg	1	C	T
TCGA-23-1031	HKR1	ENST00000324411	p.P147S	c.439C>T	Verified	AAT	CCT	CTC	1	C	T
TCGA-61-1910	HLA-DQB2	ENST00000411527	p.L225F	c.673C>T	Unverified	GGA	CTC	CTG	1	C	T
TCGA-09-2051	HMCN1	ENST00000271588	p.P997L	c.2990C>T	Unverified	ATT	CCA	GTA	2	C	T
TCGA-04-1347	HMCN1	ENST00000271588	p.C5470C	c.16410C>T	Verified	ACA	TGC	CAA	3	C	T
TCGA-13-1499	HMCN1	ENST00000271588	p.H1910H	c.5730C>T	Verified	CAG	CAC	ATT	3	C	T
TCGA-24-2289	HMGCL	ENST00000374490	p.L281L	c.841C>T	Unverified	GAC	CTG	GTC	1	C	T
TCGA-13-0920	HMGCS2	ENST00000369406	p.F420F	c.1260C>T	Verified	AGT	TTC	TTT	3	C	T
TCGA-13-0795	HMOX1	ENST00000216117	p.D92D	c.276C>T	Verified	CAG	GAC	CTG	3	C	T
TCGA-61-1740	HNF1A	ENST00000257555	p.S337S	c.1011C>T	Unverified	AGC	AGC	GGC	3	C	T
TCGA-09-1674	HNRNPA0	ENST00000314940	p.G236G	c.708C>T	Unverified	GGA	GGC	GGC	3	C	T
TCGA-23-1809	HNRNPA3	ENST00000392524	p.G278G	c.834C>T	Unverified	TAT	GGC	GGT	3	C	T
TCGA-61-1900	HNRNPL	ENST00000221419	p.H587H	c.1761C>T	Unverified	CAG	CAC	GCC	3	C	T
TCGA-61-1900	HNRPL	ENST00000388749	p.H454H	c.1362C>T	Unverified	CAG	CAC	GCC	3	C	T
TCGA-29-1766	HOMER1	ENST00000334082	p.T9I	c.26C>T	Unverified	AGC	ACT	CGA	2	C	T
TCGA-24-1844	HOOK2	ENST00000397668	p.Q281*	c.841C>T	Unverified	AAC	CAG	GCG	1	C	T
TCGA-13-0760	HOOK2	ENST00000397668	p.A274V	c.821C>T	Verified	GTT	GCG	GAG	2	C	T
TCGA-61-1737	HOOK2	ENST00000397668	p.D592D	c.1776C>T	Unverified	GCG	GAC	TTG	3	C	T
TCGA-61-2102	HOXA10	ENST00000283921	p.G23G	c.69C>T	Unverified	GAG	AGC	CCC	3	C	T
TCGA-61-1914	HOXA2	ENST00000222718	p.I135I	c.405C>T	Unverified	GAA	ATC	GCC	3	C	T
TCGA-24-1423	HPCA	ENST00000373467	p.D60D	c.180C>T	Verified	GGT	GAC	GCC	3	C	T

TCGA-13-0924	HPS3	ENST00000296051	p.P656L	c.1967C>T	Verified	AAT	CCT	TTA	2	C	T
TCGA-13-1481	HR	ENST00000381418	p.A588A	c.1764C>T	Verified	CCA	GCC	GTG	3	C	T
TCGA-29-1763	HRNR	ENST00000368801	p.S358S	c.1074C>T	Unverified	CAG	TCC	TCT	3	C	T
TCGA-61-1915	HS3ST4	ENST00000331351	p.L323L	c.967C>T	Unverified	GGG	CTG	ATC	1	C	T
TCGA-61-1915	HS3ST4_ENS	ENST00000331351	p.L323L	c.967C>T	Unverified	GGG	CTG	ATC	1	C	T
TCGA-24-1464	HSD17B2	ENST00000199936	p.P340S	c.1018C>T	Verified	ACG	CCA	GGG	1	C	T
TCGA-61-1906	HSPG2	ENST00000374695	p.Y3867Y	c.11601C>T	Unverified	AGC	TAC	GTG	3	C	T
TCGA-61-1906	HSPG2	ENST00000374695	p.Y3867Y	c.11601C>T	Unverified	AGC	TAC	GTG	3	C	T
TCGA-24-1431	HTR1A	ENST00000323865	p.A60V	c.179C>T	Verified	GCT	GCC	ATC	2	C	T
TCGA-24-1846	HTR1E	ENST00000369584	p.Y213Y	c.639C>T	Unverified	CTT	TAC	CAG	3	C	T
TCGA-13-0923	HTR2C	ENST00000371951	p.P188L	c.563C>T	Verified	gtt	cct	atc	2	C	T
TCGA-13-0916	HTR7	ENST00000336152	p.V206V	c.618C>T	Verified	TCC	GTC	TGG	3	C	T
TCGA-29-1691	HTT	ENST00000355072	p.P2849L	c.8546C>T	Unverified	GGG	CCG	GAA	2	C	T
TCGA-13-1505	HTT	ENST00000355072	p.S1950S	c.5850C>T	Unverified	GCC	AGC	GGC	3	C	T
TCGA-13-1505	HUNK	ENST00000270112	p.S454F	c.1361C>T	Unverified	ttc	tcc	aag	2	C	T
TCGA-25-2400	IBTK	ENST00000306270	p.N656N	c.1968C>T	Verified	AAA	AAC	CCA	3	C	T
TCGA-61-2008	ICAM2	ENST00000449662	p.Q72*	c.214C>T	Verified	GAA	CAG	GCT	1	C	T
TCGA-24-1845	IFI44L	ENST00000342282	p.R7C	c.19C>T	Unverified	AGC	CGT	CAG	1	C	T
TCGA-24-1845	IFI44L_ENST	ENST00000370751	p.R46C	c.136C>T	Unverified	AGC	CGT	CAG	1	C	T
TCGA-23-1022	IFLTD1	ENST00000282881	p.A210V	c.629C>T	Verified	GCA	GCA	TCT	2	C	T
TCGA-24-1616	IFNA17	ENST00000413767	p.P62P	c.186C>T	Unverified	CTT	CCC	CAG	3	C	T
TCGA-24-2267	IFNA4	ENST00000421715	p.L119L	c.355C>T	Verified	GAC	CTG	GAA	1	C	T
TCGA-20-1683	IFNGR2	ENST00000290219	p.N42N	c.126C>T	Unverified	tac	aac	gca	3	C	T
TCGA-61-2094	IFNW1	ENST00000380229	p.L185L	c.553C>T	Unverified	AGA	CTG	AGA	1	C	T
TCGA-13-0795	IFT140	ENST00000426508	p.R158W	c.472C>T	Verified	TTC	CGG	CTC	1	C	T
TCGA-09-2049	IFT140	ENST00000426508	p.D972D	c.2916C>T	Unverified	ATG	GAC	GCC	3	C	T
TCGA-13-0890	IFT172	ENST00000260570	p.T35I	c.104C>T	Verified	TGC	ACA	GTG	2	C	T
TCGA-09-1665	IGF2R	ENST00000356956	p.F2246F	c.6738C>T	Verified	TTC	TTC	CAC	3	C	T
TCGA-23-1116	IGSF1	ENST00000361420	p.L956L	c.2868C>T	Unverified	TAT	CTC	AGT	3	C	T
TCGA-23-1032	IGSF10	ENST00000282466	p.T1381I	c.4142C>T	Verified	ACC	ACA	GCC	2	C	T
TCGA-29-1691	IGSF21	ENST00000251296	p.R288C	c.862C>T	Unverified	CTG	CGC	CAC	1	C	T
TCGA-30-1714	IGSF21	ENST00000251296	p.A201V	c.602C>T	Unverified	GCT	GCG	AGC	2	C	T
TCGA-13-1496	IGSF21	ENST00000251296	p.D393D	c.1179C>T	Verified	TTC	GAC	GGG	3	C	T
TCGA-24-1616	IGSF3	ENST00000369486	p.R21W	c.61C>T	Verified	CAG	CGG	CAG	1	C	T

TCGA-13-1481	IGSF9	ENST00000361509	p.R645W	c.1933C>T	Verified	GGC	CGG	CAA	1	C	T
TCGA-20-1687	IGSF9	ENST00000361509	p.T258I	c.773C>T	Unverified	CTC	ACC	TAC	2	C	T
TCGA-29-1705	IKBKE	ENST00000367120	p.Q655*	c.1963C>T	Unverified	CTT	CAG	GAC	1	C	T
TCGA-24-1616	IKBKE	ENST00000367120	p.A371V	c.1112C>T	Verified	ATC	GCC	CAC	2	C	T
TCGA-13-0924	IKBKE	ENST00000367120	p.P400P	c.1200C>T	Verified	GTC	CCC	AAG	3	C	T
TCGA-29-1784	IL1RAPL2	ENST00000372582	p.P604S	c.1810C>T	Unverified	ctc	cct	gaa	1	C	T
TCGA-04-1338	IL20RB	ENST00000329582	p.T146T	c.438C>T	Verified	ATC	ACC	AAA	3	C	T
TCGA-29-1696	IL25	ENST00000329715	p.H107H	c.321C>T	Unverified	TAC	CAC	GCC	3	C	T
TCGA-29-1696	IL25	ENST00000329715	p.H107H	c.321C>T	Unverified	TAC	CAC	GCC	3	C	T
TCGA-29-1691	IL8	ENST00000307407	p.T39I	c.116C>T	Unverified	AAG	ACA	TAC	2	C	T
TCGA-13-0913	ILF3	ENST00000318511	p.S503S	c.1509C>T	Verified	GTC	TCC	ACC	3	C	T
TCGA-04-1338	IMMT	ENST00000410111	p.S150F	c.449C>T	Unverified	ATT	TCT	GCA	2	C	T
TCGA-23-1031	IMPG1	ENST00000369950	p.P482S	c.1444C>T	Verified	ATC	CCC	ACC	1	C	T
TCGA-24-0979	INADL	ENST00000371158	p.H66Y	c.196C>T	Verified	AAC	CAT	ATA	1	C	T
TCGA-24-1844	INADL	ENST00000371158	p.R1261C	c.3781C>T	Unverified	TCA	CGC	ATG	1	C	T
TCGA-25-2398	INO80	ENST00000361937	p.D1379D	c.4137C>T	Verified	AGT	GAC	ATG	3	C	T
TCGA-25-2398	INOC1	ENST00000361937	p.D1379D	c.4137C>T	Verified	agt	gac	atg	3	C	T
TCGA-13-0762	INSC	ENST00000379554	p.R3W	c.7C>T	Verified	AGA	CGG	CCC	1	C	T
TCGA-13-0762	INSC	ENST00000379554	p.R3W	c.7C>T	Verified	AGA	CGG	CCC	1	C	T
TCGA-04-1367	INSC	ENST00000379554	p.P5L	c.14C>T	Verified	CCC	CCT	GGC	2	C	T
TCGA-61-2008	INSR	ENST00000302850	p.S787S	c.2361C>T	Verified	ACC	AGC	GTG	3	C	T
TCGA-24-2035	INTS3	ENST00000318967	p.F285F	c.855C>T	Verified	CAG	TTC	ACA	3	C	T
TCGA-20-0991	INTS4	ENST00000534064	p.N421N	c.1263C>T	Verified	TTC	AAC	GAT	3	C	T
TCGA-09-2049	INTS5	ENST00000330574	p.S240S	c.720C>T	Verified	ATT	TCC	CGG	3	C	T
TCGA-29-1690	IPO13	ENST00000372343	p.H756Y	c.2266C>T	Unverified	GCT	CAT	GAG	1	C	T
TCGA-24-1843	IPO13	ENST00000372343	p.T430M	c.1289C>T	Unverified	GAC	ACG	CTC	2	C	T
TCGA-61-1904	IPO4	ENST00000354464	p.T784T	c.2352C>T	Unverified	GGG	ACC	CTC	3	C	T
TCGA-13-0900	IQCF1	ENST00000310914	p.R141C	c.421C>T	Verified	ATC	CGC	AGA	1	C	T
TCGA-61-2008	IQCH	ENST00000335894	p.T966T	c.2898C>T	Verified	ATG	ACC	TTT	3	C	T
TCGA-04-1367	IQGAP2	ENST00000274364	p.Q115*	c.343C>T	Verified	GTC	CAG	TGG	1	C	T
TCGA-13-1497	IQGAP3	ENST00000361170	p.L237L	c.709C>T	Verified	CTT	CTG	GAG	1	C	T
TCGA-61-1915	IQUB	ENST00000324698	p.A565V	c.1694C>T	Unverified	ATT	GCG	ACA	2	C	T
TCGA-13-0919	IRF2BPL	ENST00000238647	p.P196L	c.587C>T	Unverified	GGC	CCA	AAC	2	C	T
TCGA-24-2024	IRF3	ENST00000309877	p.P103S	c.307C>T	Verified	GAC	CCA	CAT	1	C	T

TCGA-61-1740	IRF6	ENST00000367021	p.P104S	c.310C>T	Unverified	GTG	CCC	ATG	1	C	T
TCGA-24-1464	IRX1	ENST00000302006	p.A249A	c.747C>T	Verified	aag	gcc	gag	3	C	T
TCGA-24-2289	IRX1	ENST00000302006	p.F123F	c.369C>T	Unverified	cag	ttc	caa	3	C	T
TCGA-24-1843	ITGA2	NM_002203.2	p.Q693*	c.2077C>T	Unverified	aat	caa	gtg	1	C	T
TCGA-24-1843	ITGA2_ENST	ENST00000296585	p.Q693*	c.2077C>T	Unverified	AAT	CAA	GTG	1	C	T
TCGA-25-1313	ITGB3	ENST00000262017	p.D510D	c.1530C>T	Unverified	cag	gac	gaa	3	C	T
TCGA-29-1777	ITGBL1	ENST00000376180	p.T268I	c.803C>T	Unverified	GAC	ACC	TGT	2	C	T
TCGA-13-0791	ITIH1	ENST00000273283	p.T685T	c.2055C>T	Verified	GAC	ACC	CTG	3	C	T
TCGA-61-1910	ITIH1	ENST00000273283	p.I21I	c.63C>T	Unverified	CTC	ATC	CTG	3	C	T
TCGA-24-1422	ITIH3	ENST00000398670	p.L14F	c.40C>T	Verified	CTG	CTC	TCC	1	C	T
TCGA-24-2288	ITIH5	ENST00000256861	p.P481L	c.1442C>T	Unverified	ACC	CCG	CTC	2	C	T
TCGA-23-2078	ITK	ENST00000422843	p.P159L	c.476C>T	Verified	CCT	CCT	ACT	2	C	T
TCGA-25-1313	ITPK1	ENST00000267615	p.S223L	c.668C>T	Unverified	ACA	TCA	GAC	2	C	T
TCGA-24-1470	ITPKB	NM_002221	p.R48C	c.142C>T	Verified	acg	cgt	ggc	1	C	T
TCGA-25-2392	JAK1	ENST00000342505	p.T478T	c.1434C>T	Verified	ATG	ACC	GTC	3	C	T
TCGA-61-1740	JAK2	ENST00000381652	p.S1115S	c.3345C>T	Unverified	CCC	TCC	TTT	3	C	T
TCGA-13-0900	JAKMIP2	ENST00000265272	p.Q778*	c.2332C>T	Unverified	TTA	CAG	CAA	1	C	T
TCGA-13-0900	JAKMIP2	ENST00000265272	p.R204W	c.610C>T	Unverified	GAG	CGG	GAT	1	C	T
TCGA-09-2051	JMJD1C	ENST00000402544	p.S100S	c.300C>T	Unverified	GAT	AGC	AGT	3	C	T
TCGA-29-1691	JPH4	ENST00000356300	p.P453P	c.1359C>T	Unverified	ACC	CCC	TCA	3	C	T
TCGA-13-0885	KANK4	ENST00000371153	p.N669N	c.2007C>T	Verified	GTT	AAC	GGT	3	C	T
TCGA-13-0791	KBTBD12	ENST00000405109	p.C557C	c.1671C>T	Verified	GTC	TGC	GGG	3	C	T
TCGA-04-1652	KBTBD5	ENST00000287777	p.R402C	c.1204C>T	Unverified	CCC	CGC	TGC	1	C	T
TCGA-04-1652	KBTBD5	ENST00000287777	p.R402C	c.1204C>T	Unverified	CCC	CGC	TGC	1	C	T
TCGA-13-0903	KCMF1	ENST00000409785	p.S169L	c.506C>T	Verified	AGA	TCA	AAC	2	C	T
TCGA-29-1783	KCND3	ENST00000369697	p.R37W	c.109C>T	Unverified	AAG	CGG	CAG	1	C	T
TCGA-24-1463	KCNH1	ENST00000271751	p.N941N	c.2823C>T	Verified	TTA	AAC	GCC	3	C	T
TCGA-04-1338	KCNJ12	ENST00000331718	p.S343L	c.1028C>T	Verified	TAC	TCG	CAC	2	C	T
TCGA-13-0761	KCNJ12	ENST00000331718	p.S343L	c.1028C>T	Verified	TAC	TCG	CAC	2	C	T
TCGA-13-0761	KCNJ12	ENST00000331718	p.S343L	c.1028C>T	Verified	TAC	TCG	CAC	2	C	T
TCGA-13-1497	KCNJ16	ENST00000392670	p.Y46Y	c.138C>T	Verified	GTC	TAC	TTC	3	C	T
TCGA-29-1785	KCNJ3	ENST00000295101	p.I167I	c.501C>T	Unverified	TCC	ATC	CTG	3	C	T
TCGA-29-1785	KCNJ3	ENST00000295101	p.I167I	c.501C>T	Unverified	TCC	ATC	CTG	3	C	T
TCGA-23-1114	KCNK13	ENST00000282146	p.I156I	c.468C>T	Unverified	TAC	ATC	ATG	3	C	T

TCGA-04-1331	KCNK17	ENST00000373231	p.F135F	c.405C>T	Unverified	atc	ttc	ttt	3	C	T
TCGA-23-2078	KCNMB1	ENST00000274629	p.D119D	c.357C>T	Verified	GCC	GAC	GTG	3	C	T
TCGA-13-1496	KCNS2	ENST00000287042	p.Y377Y	c.1131C>T	Verified	GGG	TAC	GGG	3	C	T
TCGA-13-0795	KCNT2	ENST00000294725	p.P751P	c.2253C>T	Verified	AAT	CCC	ATA	3	C	T
TCGA-23-1110	KCNU1	ENST00000399881	p.A424V	c.1271C>T	Verified	CAT	GCT	GAA	2	C	T
TCGA-29-1784	KCNV1	ENST00000524391	p.H328Y	c.982C>T	Unverified	aga	cat	tcc	1	C	T
TCGA-29-1691	KCNV1	ENST00000524391	p.A209V	c.626C>T	Unverified	gct	gcc	cgt	2	C	T
TCGA-23-1029	KCTD17	ENST00000352736	p.P275P	c.825C>T	Unverified	GCA	CCC	GGA	3	C	T
TCGA-61-1740	KCTD20	ENST00000373731	p.H4Y	c.10C>T	Unverified	GTT	CAC	CGT	1	C	T
TCGA-23-2077	KDELC2	ENST00000323468	p.T376T	c.1128C>T	Verified	GGG	ACC	GTG	3	C	T
TCGA-13-0760	KDM2A	ENST00000529006	p.R1117C	c.3349C>T	Verified	cgg	cgc	att	1	C	T
TCGA-09-2049	KDM5A	NM_005056.1	p.L1229F	c.3685C>T	Verified	tca	ctc	ctg	1	C	T
TCGA-30-1718	KDM5A	NM_005056.1	p.L1453F	c.4357C>T	Unverified	gaa	ctt	atg	1	C	T
TCGA-30-1718	KDM5A_ENS	ENST00000399788	p.L1448F	c.4342C>T	Unverified	GAA	CTT	ATG	1	C	T
TCGA-09-2056	KDM5C	ENST00000375401	p.P1429L	c.4286C>T	Verified	CCC	CCA	GAC	2	C	T
TCGA-24-2289	KDM6B	XM_043272.9	p.T1276T	c.3828C>T	Unverified	acc	acc	att	3	C	T
TCGA-24-1846	KDR	ENST00000263923	p.H1144Y	c.3430C>T	Unverified	tgg	cac	ggg	1	C	T
TCGA-23-1021	KEL	ENST00000355265	p.A491V	c.1472C>T	Verified	CTG	GCC	CGA	2	C	T
TCGA-24-1469	KIAA0090	ENST00000477853	p.S287F	c.860C>T	Verified	GCT	TCC	CGG	2	C	T
TCGA-13-0791	KIAA0182	ENST00000253458	p.A1156V	c.3467C>T	Verified	gag	gcc	cgg	2	C	T
TCGA-24-1604	KIAA0196	ENST00000318410	p.R391C	c.1171C>T	Verified	AAA	CGC	CTT	1	C	T
TCGA-13-1499	KIAA0317	ENST00000356357	p.H101Y	c.301C>T	Verified	GTT	CAC	ATC	1	C	T
TCGA-25-2393	KIAA0355	ENST00000299505	p.P813S	c.2437C>T	Verified	GGA	CCT	AGA	1	C	T
TCGA-24-1847	KIAA0430	ENST00000396368	p.H1530H	c.4590C>T	Unverified	GGC	CAC	AGC	3	C	T
TCGA-20-1687	KIAA0467_E	ENST00000372442	p.Q2299*	c.6895C>T	Unverified	AGC	CAG	CGA	1	C	T
TCGA-29-1785	KIAA0556	ENST00000261588	p.R107*	c.319C>T	Unverified	GGA	CGA	AGA	1	C	T
TCGA-29-1785	KIAA0556	ENST00000261588	p.R107*	c.319C>T	Unverified	GGA	CGA	AGA	1	C	T
TCGA-04-1638	KIAA0556	ENST00000261588	p.T917M	c.2750C>T	Unverified	AAC	ACG	GAG	2	C	T
TCGA-29-1785	KIAA0556_E	ENST00000261588	p.R107*	c.319C>T	Unverified	GGA	CGA	AGA	1	C	T
TCGA-29-1785	KIAA0556_E	ENST00000261588	p.R107*	c.319C>T	Unverified	GGA	CGA	AGA	1	C	T
TCGA-04-1638	KIAA0556_E	ENST00000261588	p.T917M	c.2750C>T	Unverified	AAC	ACG	GAG	2	C	T
TCGA-61-2095	KIAA0701	ENST00000356828	p.H78Y	c.232C>T	Unverified	ACA	CAT	CCC	1	C	T
TCGA-25-2401	KIAA0889	ENST00000237536	p.H551H	c.1653C>T	Verified	GAG	CAC	GAG	3	C	T
TCGA-24-1849	KIAA1024	ENST00000305428	p.P58S	c.172C>T	Unverified	GAT	CCC	AAT	1	C	T

TCGA-29-1711	KIAA1210	XM_172801.3	p.T1322T	c.3966C>T	Unverified	ttc	acc	cag	3	C	T
TCGA-29-1711	KIAA1210	XM_172801.3	p.T1322T	c.3966C>T	Unverified	ttc	acc	cag	3	C	T
TCGA-13-1488	KIAA1217	ENST00000376454	p.S302F	c.905C>T	Verified	GGA	TCT	ACT	2	C	T
TCGA-24-1104	KIAA1217	ENST00000376454	p.P300P	c.900C>T	Verified	CGC	CCC	GGA	3	C	T
TCGA-24-1616	KIAA1409	ENST00000256339	p.S1937L	c.5810C>T	Verified	GCC	TCG	TCT	2	C	T
TCGA-20-0990	KIAA1522	ENST00000401073	p.R479W	c.1435C>T	Unverified	ATT	CGG	AGC	1	C	T
TCGA-24-1563	KIAA1614	ENST00000367588	p.T597I	c.1790C>T	Verified	GAA	ACA	CAC	2	C	T
TCGA-25-2392	KIAA1683	ENST00000359737	p.A707V	c.2120C>T	Verified	CTG	GCC	CAT	2	C	T
TCGA-13-1488	KIAA1755	ENST00000279024	p.G295G	c.885C>T	Verified	GCA	GGC	ACA	3	C	T
TCGA-61-2012	KIAA1755	ENST00000279024	p.T182T	c.546C>T	Unverified	ATA	ACC	AGC	3	C	T
TCGA-24-1850	KIAA1797	ENST00000380249	p.G1636G	c.4908C>T	Unverified	ACG	GGC	GTT	3	C	T
TCGA-24-2289	KIAA1804	ENST00000366624	p.R575*	c.1723C>T	Unverified	TTT	CGA	CAA	1	C	T
TCGA-29-1701	KIAA1958	ENST00000337530	p.R528R	c.1584C>T	Unverified	TCT	CGC	AAC	3	C	T
TCGA-09-1674	KIAA1967	ENST00000389279	p.P37S	c.109C>T	Unverified	CCT	CCT	GTG	1	C	T
TCGA-20-1686	KIAA2013	ENST00000376572	p.T393M	c.1178C>T	Unverified	TCG	ACG	CTC	2	C	T
TCGA-24-2289	KIAA2018	ENST00000316407	p.P1069L	c.3206C>T	Unverified	CTC	CCT	GAT	2	C	T
TCGA-29-1703	KIF13A_ENS	ENST00000378814	p.T1746I	c.5237C>T	Unverified	GGC	ACA	ACC	2	C	T
TCGA-04-1638	KIF13B	ENST00000524189	p.R641W	c.1921C>T	Unverified	CTC	CGG	AGA	1	C	T
TCGA-04-1638	KIF13B_ENS	ENST00000524189	p.R641W	c.1921C>T	Unverified	CTC	CGG	AGA	1	C	T
TCGA-29-2427	KIF14	ENST00000367350	p.P1552S	c.4654C>T	Verified	GTG	CCT	TCT	1	C	T
TCGA-61-1733	KIF18B	ENST00000339151	p.P835P	c.2505C>T	Unverified	AGT	CCC	CTG	3	C	T
TCGA-61-1733	KIF18B	ENST00000339151	p.P835P	c.2505C>T	Unverified	AGT	CCC	CTG	3	C	T
TCGA-13-1510	KIF21A	ENST00000361961	p.S1261F	c.3782C>T	Unverified	CCT	TCT	TCC	2	C	T
TCGA-29-1691	KIF21B	ENST00000332129	p.A1303V	c.3908C>T	Unverified	GAT	GCC	ACA	2	C	T
TCGA-30-1714	KIF21B	ENST00000332129	p.P1246L	c.3737C>T	Unverified	CGG	CCC	CGC	2	C	T
TCGA-13-1488	KIF26B	ENST00000407071	p.L415L	c.1245C>T	Verified	CCG	CTC	TTT	3	C	T
TCGA-29-1769	KIF27	ENST00000297814	p.R717C	c.2149C>T	Unverified	GAA	CGC	ATA	1	C	T
TCGA-24-2280	KIF3A	ENST00000378746	p.A556V	c.1667C>T	Unverified	GCT	GCA	AAG	2	C	T
TCGA-24-1847	KIF5A	ENST00000455537	p.T58I	c.173C>T	Unverified	ACG	ACT	CAA	2	C	T
TCGA-13-1498	KIRREL	ENST00000392272	p.T293T	c.879C>T	Verified	CTC	ACC	ATC	3	C	T
TCGA-24-2267	KIRREL3	ENST00000278934	p.S466S	c.1398C>T	Verified	GGC	TCC	GAC	3	C	T
TCGA-09-1665	KIT	ENST00000288135	p.P31L	c.92C>T	Verified	agt	cca	ggg	2	C	T
TCGA-09-2056	KIT	ENST00000288135	p.N326N	c.978C>T	Verified	gta	aac	gat	3	C	T
TCGA-13-0923	KITLG	ENST00000228280	p.H67Y	c.199C>T	Verified	agt	cat	tgt	1	C	T

TCGA-13-0900	KLC3	ENST00000391946	p.T282M	c.845C>T	Verified	CAG	ACG	CTG	2	C	T
TCGA-09-1674	KLF13	ENST00000307145	p.A246V	c.737C>T	Unverified	CAC	GCG	CGC	2	C	T
TCGA-25-2392	KLF3	ENST00000261438	p.P205L	c.614C>T	Verified	GAA	CCA	CAG	2	C	T
TCGA-13-1510	KLHDC1	ENST00000359332	p.H225H	c.675C>T	Unverified	TTG	CAC	TAT	3	C	T
TCGA-61-1904	KLHDC7A	ENST00000400664	p.R558C	c.1672C>T	Unverified	AGC	CGC	GTC	1	C	T
TCGA-04-1342	KLHL1	ENST00000377844	p.L57L	c.171C>T	Unverified	CTG	CTC	AAA	3	C	T
TCGA-13-0916	KLHL14	ENST00000359358	p.R452C	c.1354C>T	Verified	TGG	CGC	TAT	1	C	T
TCGA-13-1505	KLHL25	ENST00000337975	p.L39F	c.115C>T	Verified	ACG	CTT	CGC	1	C	T
TCGA-13-0714	KLHL29	ENST00000288548	p.S141S	c.423C>T	Verified	AGG	TCC	GTG	3	C	T
TCGA-24-1563	KLHL32	ENST00000369261	p.A588V	c.1763C>T	Verified	TTT	GCT	TCC	2	C	T
TCGA-61-1737	KLK1	ENST00000301420	p.P234L	c.701C>T	Unverified	gtc	cct	tgt	2	C	T
TCGA-13-1507	KLK12	ENST00000250351	p.C161C	c.483C>T	Verified	cag	tgc	ctc	3	C	T
TCGA-61-1998	KLK2	ENST00000325321	p.P237S	c.709C>T	Verified	ctg	cct	gaa	1	C	T
TCGA-04-1649	KLRAQ1	ENST00000281394	p.I550I	c.1650C>T	Unverified	CGC	ATC	CTT	3	C	T
TCGA-13-1488	KLRC3	ENST00000381903	p.N149N	c.447C>T	Verified	AAG	AAC	TCT	3	C	T
TCGA-23-1114	KNTC1	ENST00000333479	p.T13T	c.39C>T	Unverified	gat	acc	gga	3	C	T
TCGA-61-1738	KPNA5	ENST00000368564	p.A21A	c.63C>T	Unverified	aaa	gcc	cta	3	C	T
TCGA-61-1738	KPNA5	ENST00000368564	p.A21A	c.63C>T	Unverified	aaa	gcc	cta	3	C	T
TCGA-24-2267	KPTN	ENST00000338134	p.L190L	c.570C>T	Unverified	AAC	CTC	TTC	3	C	T
TCGA-25-2398	KREMEN1	ENST00000327813	p.S421F	c.1262C>T	Verified	aaa	tcc	cat	2	C	T
TCGA-24-2262	KRT17	ENST00000311208	p.L99L	c.295C>T	Verified	TAC	CTG	GAC	1	C	T
TCGA-29-1766	KRT17	ENST00000311208	p.I300I	c.900C>T	Unverified	GAG	ATC	TCG	3	C	T
TCGA-24-1469	KRT2	ENST00000309680	p.G566G	c.1698C>T	Verified	TCT	GGC	GGT	3	C	T
TCGA-29-1701	KRT31	ENST00000251645	p.S55S	c.165C>T	Unverified	GGT	AGC	GAG	3	C	T
TCGA-29-1701	KRT31_ENST	ENST00000251645	p.S55S	c.165C>T	Unverified	GGT	AGC	GAG	3	C	T
TCGA-13-0919	KRT6A	ENST00000330722	p.A403A	c.1209C>T	Verified	TGC	GCC	AAC	3	C	T
TCGA-13-0904	KRT6C	ENST00000252250	p.L434L	c.1300C>T	Verified	GCC	CTG	CAG	1	C	T
TCGA-25-2401	KRT73	ENST00000305748	p.A82V	c.245C>T	Verified	TTT	GCT	GGC	2	C	T
TCGA-13-0760	KRT74	ENST00000305620	p.S430S	c.1290C>T	Verified	ATG	AGC	CTG	3	C	T
TCGA-13-0919	KRT9	ENST00000246662	p.R261W	c.781C>T	Verified	CTG	CGG	CAG	1	C	T
TCGA-09-2049	KRTAP12-4	ENST00000391618	p.T4I	c.11C>T	Verified	CAC	ACC	AGC	2	C	T
TCGA-29-1699	KRTAP27-1	ENST00000382835	p.F151F	c.453C>T	Unverified	AAT	TTC	GAA	3	C	T
TCGA-29-1703	KRTAP4-5	ENST00000343246	p.R37C	c.109C>T	Unverified	TGC	CGC	CCC	1	C	T
TCGA-20-1687	KRTAP5-4	ENST00000399682	p.G106G	c.318C>T	Unverified	GGG	GGC	TCC	3	C	T

TCGA-10-0930	KY	ENST00000423778	p.R479C	c.1435C>T	Verified	GGG	CGC	TGC	1	C	T
TCGA-10-0930	KY	ENST00000423778	p.R479C	c.1435C>T	Verified	GGG	CGC	TGC	1	C	T
TCGA-20-0990	L1CAM	ENST00000370060	p.S1062F	c.3185C>T	Verified	GCT	TCC	CTT	2	C	T
TCGA-29-1777	LAG3	ENST00000203629	p.N250N	c.750C>T	Unverified	TTC	AAC	GTC	3	C	T
TCGA-61-1904	LAMA1	ENST00000389658	p.P424L	c.1271C>T	Unverified	CAG	CCA	GGT	2	C	T
TCGA-24-2262	LAMA1	ENST00000389658	p.P1085P	c.3255C>T	Verified	TTT	CCC	GAC	3	C	T
TCGA-13-0923	LAMA4	ENST00000389463	p.Q331*	c.991C>T	Verified	ATA	CAA	ATC	1	C	T
TCGA-13-1498	LAMA4	ENST00000389463	p.H1451H	c.4353C>T	Verified	TGC	CAC	CTT	3	C	T
TCGA-23-1021	LAMB1	ENST00000222399	p.A1437V	c.4310C>T	Verified	GTT	GCA	CAC	2	C	T
TCGA-30-1856	LARGE	ENST00000397394	p.I554I	c.1662C>T	Unverified	CAC	ATC	AGC	3	C	T
TCGA-13-1488	LARS2	ENST00000265537	p.I314I	c.942C>T	Verified	GCC	ATC	TCG	3	C	T
TCGA-23-2077	LCE2B	ENST00000368780	p.C110C	c.330C>T	Verified	TGC	TGC	TGA	3	C	T
TCGA-23-1110	LCE5A	ENST00000334269	p.P33L	c.98C>T	Verified	TGT	CCC	CCA	2	C	T
TCGA-09-2049	LCK	ENST00000336890	p.V434V	c.1302C>T	Verified	ATT	GTC	ACC	3	C	T
TCGA-20-0991	LCT	ENST00000264162	p.S1387F	c.4160C>T	Verified	GCT	TCT	GCT	2	C	T
TCGA-10-0930	LDB3	ENST00000361373	p.S241S	c.723C>T	Verified	GAC	AGC	GCC	3	C	T
TCGA-10-0930	LDB3	ENST00000361373	p.S241S	c.723C>T	Verified	GAC	AGC	GCC	3	C	T
TCGA-23-1029	LDHAL6A	ENST00000280706	p.V49V	c.147C>T	Unverified	CTT	GTC	CTT	3	C	T
TCGA-13-1507	LDLR	ENST00000558518	p.P848L	c.2543C>T	Verified	TAC	CCC	TCG	2	C	T
TCGA-29-1783	LDLRAP1	ENST00000374338	p.R151W	c.451C>T	Unverified	AAG	CGG	AAG	1	C	T
TCGA-20-1683	LEPR	ENST00000371060	p.T85I	c.254C>T	Unverified	AAA	ACA	ACT	2	C	T
TCGA-61-1733	LEPR	ENST00000371060	p.S60L	c.179C>T	Unverified	AAT	TCG	AAT	2	C	T
TCGA-61-1733	LEPR	ENST00000371060	p.S60L	c.179C>T	Unverified	AAT	TCG	AAT	2	C	T
TCGA-20-1683	LEPR_ENST0	ENST00000344610	p.T85I	c.254C>T	Unverified	AAA	ACA	ACT	2	C	T
TCGA-61-1733	LEPR_ENST0	ENST00000344610	p.S60L	c.179C>T	Unverified	AAT	TCG	AAT	2	C	T
TCGA-61-1733	LEPR_ENST0	ENST00000344610	p.S60L	c.179C>T	Unverified	AAT	TCG	AAT	2	C	T
TCGA-20-1683	LEPR_ENST0	ENST00000349533	p.T85I	c.254C>T	Unverified	AAA	ACA	ACT	2	C	T
TCGA-61-1733	LEPR_ENST0	ENST00000349533	p.S60L	c.179C>T	Unverified	AAT	TCG	AAT	2	C	T
TCGA-61-1733	LEPR_ENST0	ENST00000349533	p.S60L	c.179C>T	Unverified	AAT	TCG	AAT	2	C	T
TCGA-61-1998	LGALS13	ENST00000221797	p.D79D	c.237C>T	Verified	ACA	GAC	TAC	3	C	T
TCGA-23-1021	LHX1	ENST00000254457	p.H85H	c.255C>T	Verified	TTT	CAC	CTG	3	C	T
TCGA-29-1775	LHX8	ENST00000294638	p.H355Y	c.1063C>T	Unverified	AGT	CAT	ACC	1	C	T
TCGA-25-2401	LIG3	NM_002311.2	p.A128V	c.383C>T	Verified	ggc	gcc	tca	2	C	T
TCGA-24-2288	LIG4	ENST00000405925	p.P492L	c.1475C>T	Unverified	ccc	cct	cct	2	C	T

TCGA-09-2044	LILRA1	ENST00000251372	p.P113S	c.337C>T	Verified	GAC	CCC	CTG	1	C	T
TCGA-24-1844	LILRA4	ENST00000291759	p.Y376Y	c.1128C>T	Unverified	AAG	TAC	CAG	3	C	T
TCGA-23-1111	LILRB1	ENST00000396331	p.R179R	c.537C>T	Unverified	TCC	CGC	GCC	3	C	T
TCGA-29-1691	LILRB2	ENST00000314446	p.L243F	c.727C>T	Unverified	ACC	CTC	CAG	1	C	T
TCGA-13-0760	LIMA1	ENST00000341247	p.R9W	c.25C>T	Verified	AGA	CGG	CAA	1	C	T
TCGA-25-2392	LIMK2	ENST00000331728	p.L244L	c.730C>T	Verified	CGC	CTG	GAC	1	C	T
TCGA-23-2078	LIN28B	ENST00000345080	p.P146L	c.437C>T	Unverified	CCT	CCT	CAG	2	C	T
TCGA-61-1900	LIN7A	ENST00000552864	p.R204*	c.610C>T	Unverified	CTA	CGA	ACA	1	C	T
TCGA-13-0792	LIPK	ENST00000404190	p.Y168Y	c.504C>T	Verified	TAC	TAC	GTG	3	C	T
TCGA-13-0795	LL0XNC01-2	ENST00000218249	p.T70M	c.209C>T	Verified	gat	acg	tcg	2	C	T
TCGA-61-1915	LL0XNC01-2	ENST00000218249	p.Y11Y	c.33C>T	Unverified	gcc	tac	gac	3	C	T
TCGA-29-1701	LLPH	ENST00000266604	p.P23S	c.67C>T	Unverified	GCC	CCA	AAG	1	C	T
TCGA-13-0884	LMBRD1	ENST00000370577	p.S461F	c.1382C>T	Verified	CTT	TCT	GTG	2	C	T
TCGA-29-1695	LMX1A	ENST00000367893	p.R197C	c.589C>T	Unverified	AAA	CGT	CCG	1	C	T
TCGA-13-0884	LNX2	ENST00000316334	p.P227S	c.679C>T	Verified	tta	cca	gaa	1	C	T
TCGA-04-1331	LOC114984	ENST00000396958	p.N128N	c.384C>T	Unverified	GAG	AAC	TTT	3	C	T
TCGA-13-1488	LOC168850	ENST00000393313	p.H310Y	c.928C>T	Verified	GTT	CAT	AGA	1	C	T
TCGA-24-1844	LOC223075	ENST00000319386	p.I495I	c.1485C>T	Unverified	TGT	ATC	CCC	3	C	T
TCGA-24-1435	LOC257106	ENST00000368016	p.V286V	c.858C>T	Verified	AAG	GTC	AGG	3	C	T
TCGA-29-1691	LOC340578	ENST00000360028	p.N107N	c.321C>T	Unverified	CTG	AAC	GCC	3	C	T
TCGA-23-1111	LOC389900	XM_372261.2	p.D416D	c.1248C>T	Unverified	ctg	gac	cat	3	C	T
TCGA-13-0760	LOC51059	ENST00000395297	p.I398I	c.1194C>T	Verified	GAC	ATC	GAC	3	C	T
TCGA-61-1737	LOC65121	ENST00000332296	p.H324Y	c.970C>T	Unverified	AAG	CAT	CTG	1	C	T
TCGA-13-0883	LOC652153	ENST00000316490	p.T2472M	c.7415C>T	Unverified	CGC	ACG	GAG	2	C	T
TCGA-13-0883	LOC652737	ENST00000309900	p.T151M	c.452C>T	Unverified	CGC	ACG	GAG	2	C	T
TCGA-13-0887	LOC91807	ENST00000394809	p.F673F	c.2019C>T	Verified	AAC	TTC	GGC	3	C	T
TCGA-24-1844	LONP1	ENST00000360614	p.T190M	c.569C>T	Unverified	GGG	ACG	TTT	2	C	T
TCGA-29-1695	LONP1	ENST00000360614	p.A341A	c.1023C>T	Unverified	GGG	GCC	GAG	3	C	T
TCGA-24-1474	LOXL4	ENST00000260702	p.I694I	c.2082C>T	Verified	tat	atc	ttc	3	C	T
TCGA-13-0755	LPA	ENST00000316300	p.T331M	c.992C>T	Verified	CTG	ACG	CAA	2	C	T
TCGA-24-1844	LPA	ENST00000316300	p.T1349M	c.4046C>T	Unverified	CTG	ACG	CAA	2	C	T
TCGA-29-1690	LPA	ENST00000316300	p.T1739M	c.5216C>T	Unverified	GGG	ACG	CCA	2	C	T
TCGA-30-1714	LPA	ENST00000316300	p.A912V	c.2735C>T	Unverified	GTC	GCG	CCT	2	C	T
TCGA-24-2035	LPAR6	ENST00000345941	p.S92F	c.275C>T	Verified	ATT	TCT	GTG	2	C	T

TCGA-13-1507	LPCAT1	ENST00000283415	p.A507V	c.1520C>T	Verified	CCT	GCG	CCA	2	C	T
TCGA-20-0990	LPIN3	ENST00000373257	p.N788N	c.2364C>T	Verified	GTC	AAC	CCC	3	C	T
TCGA-24-1616	LPPR4	ENST00000370185	p.Y661Y	c.1983C>T	Verified	ACT	TAC	GAG	3	C	T
TCGA-61-1913	LRCH2	NM_020871.2	p.N279N	c.837C>T	Unverified	gat	aac	aat	3	C	T
TCGA-61-1913	LRCH2_ENS	ENST00000317135	p.N279N	c.837C>T	Unverified	GAT	AAC	AAT	3	C	T
TCGA-24-1463	LRFN2	ENST00000338305	p.T788M	c.2363C>T	Unverified	AGC	ACG	GTC	2	C	T
TCGA-29-1695	LRFN3	ENST00000246529	p.C515C	c.1545C>T	Unverified	GGC	TGC	GCC	3	C	T
TCGA-61-2012	LRFN5	ENST00000298119	p.T691M	c.2072C>T	Verified	CCC	ACG	TCT	2	C	T
TCGA-09-2051	LRGUK	ENST00000285928	p.I328I	c.984C>T	Unverified	CCC	ATC	CTT	3	C	T
TCGA-13-0760	LRP1	ENST00000243077	p.R1423C	c.4267C>T	Verified	GGG	CGC	CGC	1	C	T
TCGA-30-1857	LRP1	ENST00000243077	p.R2135*	c.6403C>T	Unverified	CTG	CGA	ACC	1	C	T
TCGA-61-1740	LRP1	ENST00000243077	p.C3439C	c.10317C>T	Unverified	AAC	TGC	GGA	3	C	T
TCGA-09-2051	LRP5	ENST00000294304	p.D736D	c.2208C>T	Unverified	GCC	GAC	ACT	3	C	T
TCGA-29-1781	LRRC1	ENST00000370888	p.R497W	c.1489C>T	Unverified	TTA	CGG	AAT	1	C	T
TCGA-24-1464	LRRC17	ENST00000339431	p.N298N	c.894C>T	Verified	TTA	AAC	CTC	3	C	T
TCGA-09-2051	LRRC36	ENST00000329956	p.P325S	c.973C>T	Unverified	TTA	CCT	TCA	1	C	T
TCGA-23-1022	LRRC39	ENST00000370137	p.F275F	c.825C>T	Verified	AAC	TTC	AGA	3	C	T
TCGA-24-2290	LRRC41	ENST00000343304	p.H766Y	c.2296C>T	Verified	GGT	CAC	CTG	1	C	T
TCGA-24-2290	LRRC41	ENST00000343304	p.H766Y	c.2296C>T	Verified	GGT	CAC	CTG	1	C	T
TCGA-13-0886	LRRC8C	ENST00000370454	p.P746L	c.2237C>T	Verified	TCA	CCG	AAA	2	C	T
TCGA-13-0795	LRRFIP2	ENST00000336686	p.D390D	c.1170C>T	Verified	TTA	GAC	AAT	3	C	T
TCGA-09-2056	LRRIQ1	ENST00000256007	p.R329*	c.985C>T	Verified	ATA	CGA	CAA	1	C	T
TCGA-23-1032	LRRN3	ENST00000308478	p.I241I	c.723C>T	Verified	AGC	ATC	TCT	3	C	T
TCGA-61-1907	LRRTM4	ENST00000409088	p.R181W	c.541C>T	Unverified	TGT	CGG	AAT	1	C	T
TCGA-61-1907	LRRTM4_EN	ENST00000409093	p.R181W	c.541C>T	Unverified	TGT	CGG	AAT	1	C	T
TCGA-13-0923	LRTM2	ENST00000299194	p.Y335Y	c.1005C>T	Unverified	ATC	TAC	GCC	3	C	T
TCGA-13-1481	LSMD1	ENST00000333775	p.T36I	c.107C>T	Verified	GGA	ACT	GCA	2	C	T
TCGA-61-2109	LTBP1	NM_206943.1	p.C943C	c.2829C>T	Unverified	att	tgc	cca	3	C	T
TCGA-23-1116	LTBR	ENST00000228918	p.H349Y	c.1045C>T	Unverified	ATT	CAT	GTC	1	C	T
TCGA-13-1497	LTN1	ENST00000361371	p.T96I	c.287C>T	Verified	GAC	ACA	GAA	2	C	T
TCGA-29-1690	LUC7L3	ENST00000505658	p.R261W	c.781C>T	Unverified	GAA	CGG	GAG	1	C	T
TCGA-29-1701	LY6G6F	ENST00000375832	p.P296P	c.888C>T	Unverified	AAG	CCC	AGG	3	C	T
TCGA-13-0903	LYST	ENST00000389793	p.P837S	c.2509C>T	Verified	GTT	CCA	GAT	1	C	T
TCGA-61-1904	LZTR1	ENST00000215739	p.D185D	c.555C>T	Unverified	AGT	GAC	AAG	3	C	T

TCGA-13-0884	MADD	ENST00000311027	p.A1421V	c.4262C>T	Verified	CTG	GCG	AAC	2	C	T
TCGA-24-0979	MALT1	ENST00000345724	p.F746F	c.2238C>T	Verified	CCA	TTC	CAT	3	C	T
TCGA-25-1326	MALT1	ENST00000345724	p.L348L	c.1044C>T	Verified	AAG	CTC	AAA	3	C	T
TCGA-24-1469	MAML2	ENST00000524717	p.L1148F	c.3442C>T	Unverified	aat	ctt	gat	1	C	T
TCGA-04-1336	MAMSTR	ENST00000356751	p.S297F	c.890C>T	Verified	TTA	TCT	GAC	2	C	T
TCGA-23-1122	MAN1A2	ENST00000356554	p.D59D	c.177C>T	Unverified	CCA	GAC	TCT	3	C	T
TCGA-24-1849	MAN2A1	ENST00000261483	p.S551L	c.1652C>T	Unverified	TCA	TCA	CTT	2	C	T
TCGA-13-1498	MAP1A	ENST00000300231	p.S2229L	c.6686C>T	Verified	TCC	TCA	CTG	2	C	T
TCGA-24-2024	MAP3K12	ENST00000267079	p.S805L	c.2414C>T	Verified	CCT	TCA	GAG	2	C	T
TCGA-09-1665	MAP3K5	ENST00000359015	p.P834S	c.2500C>T	Verified	AAC	CCC	TGT	1	C	T
TCGA-24-2288	MAP4K1_EN	ENST00000396857	p.P811L	c.2432C>T	Unverified	GAT	CCT	ACT	2	C	T
TCGA-61-1904	MAP4K4	NM_145686	p.P976S	c.2926C>T	Unverified	gat	cct	acc	1	C	T
TCGA-13-0885	MAP7D2	ENST00000379651	p.P722S	c.2164C>T	Verified	AGT	CCT	GGC	1	C	T
TCGA-61-1998	MAPK8	ENST00000374182	p.L198F	c.592C>T	Verified	atc	ctt	ggc	1	C	T
TCGA-30-1891	MAPRE3	ENST00000233121	p.I251I	c.753C>T	Unverified	GGC	ATC	ATT	3	C	T
TCGA-24-0975	MAPT	ENST00000262410	p.D704D	c.2112C>T	Unverified	ACA	GAC	CAC	3	C	T
TCGA-24-1470	MARCKSL1	ENST00000329421	p.Q126*	c.376C>T	Verified	GAG	CAG	GAG	1	C	T
TCGA-23-1118	MARS	ENST00000262027	p.A657V	c.1970C>T	Verified	aga	gct	ggg	2	C	T
TCGA-30-1714	MAST1	ENST00000251472	p.R117R	c.351C>T	Unverified	GAG	CGC	CTT	3	C	T
TCGA-30-1714	MAST1_ENS	ENST00000251472	p.R117R	c.351C>T	Unverified	GAG	CGC	CTT	3	C	T
TCGA-13-0807	MAST2	NM_015112	p.P730L	c.2189C>T	Verified	act	ccg	gag	2	C	T
TCGA-13-0807	MAST2_ENS	ENST00000361297	p.P730L	c.2189C>T	Verified	ACT	CCG	GAG	2	C	T
TCGA-20-0991	MAST4_ENS	ENST00000403625	p.R199C	c.595C>T	Unverified	ATG	CGC	AGC	1	C	T
TCGA-04-1651	MASTL	ENST00000375946	p.A237V	c.710C>T	Unverified	TCA	GCC	TGT	2	C	T
TCGA-04-1651	MASTL	ENST00000375946	p.A237V	c.710C>T	Unverified	TCA	GCC	TGT	2	C	T
TCGA-24-1850	MAT2A	ENST00000306434	p.R177C	c.529C>T	Unverified	TTA	CGC	CCT	1	C	T
TCGA-24-1474	MATN4	ENST00000372756	p.R182C	c.544C>T	Unverified	CTG	CGC	GCC	1	C	T
TCGA-23-2078	MATN4	ENST00000372756	p.F48F	c.144C>T	Unverified	CCT	TTC	GAG	3	C	T
TCGA-29-1696	MAVS	ENST00000428216	p.D53D	c.159C>T	Unverified	CGG	GAC	ACC	3	C	T
TCGA-29-1696	MAVS	ENST00000428216	p.D53D	c.159C>T	Unverified	CGG	GAC	ACC	3	C	T
TCGA-23-1114	MBD5	ENST00000407073	p.P1080L	c.3239C>T	Unverified	GGC	CCA	GGT	2	C	T
TCGA-13-1507	MC2R	ENST00000399821	p.C293C	c.879C>T	Verified	TTC	TGC	AGC	3	C	T
TCGA-61-1914	MC3R	NM_019888.2	p.R344C	c.1030C>T	Unverified	ttg	cgc	aac	1	C	T
TCGA-20-1687	MC3R	NM_019888.2	p.V75V	c.225C>T	Unverified	cag	gtc	ttc	3	C	T

TCGA-24-2024	MC3R	NM_019888.2	p.N103N	c.309C>T	Verified	agg	aac	ggc	3	C	T
TCGA-09-2049	MC5R	ENST00000324750	p.T171M	c.512C>T	Verified	TGC	ACG	GGC	2	C	T
TCGA-10-0930	MC5R	ENST00000324750	p.I219I	c.657C>T	Verified	CGG	ATC	GCG	3	C	T
TCGA-10-0930	MC5R	ENST00000324750	p.I219I	c.657C>T	Verified	CGG	ATC	GCG	3	C	T
TCGA-25-1318	MCCC1	ENST00000265594	p.L515L	c.1545C>T	Verified	GGT	CTC	ATC	3	C	T
TCGA-25-1318	MCCC1	ENST00000265594	p.L515L	c.1545C>T	Verified	GGT	CTC	ATC	3	C	T
TCGA-13-0906	MCM3	ENST00000229854	p.A509V	c.1526C>T	Verified	aga	gca	cct	2	C	T
TCGA-24-2024	MDN1	ENST00000369393	p.Q5005*	c.15013C>T	Verified	TTC	CAG	CCC	1	C	T
TCGA-29-1766	MDN1	ENST00000369393	p.H91Y	c.271C>T	Unverified	CTG	CAT	GAA	1	C	T
TCGA-13-0903	MECR	ENST00000263702	p.P292P	c.876C>T	Verified	CAG	CCC	GTC	3	C	T
TCGA-25-2393	MED12	ENST00000374080	p.P1734S	c.5200C>T	Unverified	CGG	CCC	CGC	1	C	T
TCGA-13-0923	MED13L	ENST00000281928	p.S1604F	c.4811C>T	Verified	tct	tct	tca	2	C	T
TCGA-24-1850	MED22	ENST00000344469	p.D121D	c.363C>T	Unverified	CGA	GAC	GAG	3	C	T
TCGA-04-1338	MED24	NM_014815.2	p.S601F	c.1802C>T	Verified	gag	tcc	atc	2	C	T
TCGA-29-1785	MED25	ENST00000312865	p.I114I	c.342C>T	Unverified	CTC	ATC	GCG	3	C	T
TCGA-29-1785	MED25	ENST00000312865	p.I114I	c.342C>T	Unverified	CTC	ATC	GCG	3	C	T
TCGA-29-1785	MED25_ENS	ENST00000312881	p.I114I	c.342C>T	Unverified	CTC	ATC	GCG	3	C	T
TCGA-29-1785	MED25_ENS	ENST00000312881	p.I114I	c.342C>T	Unverified	CTC	ATC	GCG	3	C	T
TCGA-24-1843	MED29	ENST00000315588	p.P56S	c.166C>T	Unverified	CCG	CCA	GCA	1	C	T
TCGA-24-1844	MEGF8	ENST00000251268_v61	p.P2048S	c.6142C>T	Unverified	ATG	CCG	GTG	1	C	T
TCGA-24-1844	MEGF8_ENS	ENST00000334370	p.P1981S	c.5941C>T	Unverified	ATG	CCG	GTG	1	C	T
TCGA-23-1118	MELK	ENST00000298048	p.H514Y	c.1540C>T	Verified	GCA	CAT	ATG	1	C	T
TCGA-13-0755	MELK	ENST00000298048	p.P346L	c.1037C>T	Verified	ACC	CCA	TTC	2	C	T
TCGA-29-1769	MEPCE	ENST00000310512	p.R170C	c.508C>T	Unverified	AGG	CGC	AGG	1	C	T
TCGA-04-1362	MEST	ENST00000223215	p.Q123*	c.367C>T	Verified	GAG	CAG	GCC	1	C	T
TCGA-13-0761	MEST	ENST00000223215	p.L157F	c.469C>T	Verified	CTT	CTC	TAC	1	C	T
TCGA-13-0761	MEST	ENST00000223215	p.L157F	c.469C>T	Verified	CTT	CTC	TAC	1	C	T
TCGA-29-1703	METTL11A	ENST00000372480	p.I163I	c.489C>T	Unverified	ATC	ATC	GTC	3	C	T
TCGA-29-1699	METTL13	ENST00000361735	p.L666L	c.1996C>T	Unverified	CTC	CTA	GAA	1	C	T
TCGA-04-1342	METTL16	NM_024086.2	p.A442A	c.1326C>T	Verified	gct	gcc	gct	3	C	T
TCGA-13-1488	METTL19	ENST00000285635	p.P15L	c.44C>T	Verified	ACA	CCC	AAT	2	C	T
TCGA-04-1347	MFN2	ENST00000235329	p.R564W	c.1690C>T	Verified	CGT	CGG	GCC	1	C	T
TCGA-23-1124	MGAT1	ENST00000333055	p.R187C	c.559C>T	Verified	GCG	CGC	CAC	1	C	T
TCGA-25-2400	MGAT1	ENST00000333055	p.P227L	c.680C>T	Unverified	TAT	CCG	CTG	2	C	T

TCGA-29-1776	MGAT1	ENST00000333055	p.D60D	c.180C>T	Unverified	CAA	GAC	GCC	3	C	T
TCGA-29-2427	MGAT4C	ENST00000332156	p.F73F	c.219C>T	Verified	ACT	TTC	AAG	3	C	T
TCGA-09-2050	MGAT5	ENST00000281923	p.Q12*	c.34C>T	Verified	TCT	CAG	AAG	1	C	T
TCGA-23-1124	MGC42105	ENST00000326035	p.F337F	c.1011C>T	Verified	CCT	TTC	CAA	3	C	T
TCGA-61-2102	MGMT	NM_002412.1	p.I76I	c.228C>T	Verified	gct	atc	gaa	3	C	T
TCGA-13-0760	MGP	ENST00000539261	p.A91V	c.272C>T	Verified	GCT	GCC	TAT	2	C	T
TCGA-25-2398	MGRN1	ENST00000262370	p.S233F	c.698C>T	Verified	TTC	TCT	GTG	2	C	T
TCGA-24-1846	MIA3	ENST00000344922	p.H880Y	c.2638C>T	Unverified	GAC	CAT	GAG	1	C	T
TCGA-25-1313	MIA3	ENST00000344922	p.G1883G	c.5649C>T	Verified	TCA	GGC	TCT	3	C	T
TCGA-23-2077	MICAL2	ENST00000256194	p.I489I	c.1467C>T	Unverified	TAT	ATC	ACT	3	C	T
TCGA-13-1501	MID2	NM_012216.2	p.R85C	c.253C>T	Verified	gat	cgc	ttc	1	C	T
TCGA-13-1501	MID2	NM_012216.2	p.R85C	c.253C>T	Verified	gat	cgc	ttc	1	C	T
TCGA-30-1714	MIDN	ENST00000300952	p.S442S	c.1326C>T	Unverified	GCC	TCC	GGC	3	C	T
TCGA-29-1698	MIER2	ENST00000264819	p.Q43*	c.127C>T	Unverified	CAT	CAG	TTC	1	C	T
TCGA-13-1507	MIIP	ENST00000235332	p.H280H	c.840C>T	Verified	GCG	CAC	GTC	3	C	T
TCGA-24-1846	MINK1_ENS	ENST00000347992	p.L1220L	c.3658C>T	Unverified	GTG	CTG	CAG	1	C	T
TCGA-24-1846	MINK1_ENS	ENST00000355280	p.L1249L	c.3745C>T	Unverified	GTG	CTG	CAG	1	C	T
TCGA-13-0795	MKI67	ENST00000368654	p.T1655I	c.4964C>T	Verified	CAG	ACA	TCA	2	C	T
TCGA-23-1032	MKRN3	ENST00000314520	p.P76S	c.226C>T	Verified	GCC	CCA	GCC	1	C	T
TCGA-61-1900	MKRN3	ENST00000314520	p.A75A	c.225C>T	Unverified	CCT	GCC	CCA	3	C	T
TCGA-25-2400	MKS1	ENST00000393119	p.Q548*	c.1642C>T	Verified	CCG	CAG	GAC	1	C	T
TCGA-04-1336	MLIP	ENST00000274897	p.S404L	c.1211C>T	Verified	CTC	TCA	CAT	2	C	T
TCGA-24-2280	MLIP	ENST00000274897	p.S418S	c.1254C>T	Verified	CTG	TCC	TTC	3	C	T
TCGA-24-1847	MLL4	ENST00000222270	p.R152W	c.454C>T	Unverified	GGT	CGG	GGT	1	C	T
TCGA-24-1431	MLNR	ENST00000218721	p.P280L	c.839C>T	Unverified	CGG	CCG	CTG	2	C	T
TCGA-29-1702	MMEL1	ENST00000288709	p.I579I	c.1737C>T	Unverified	GGG	ATC	CTC	3	C	T
TCGA-13-0904	MMP15	ENST00000219271	p.P474S	c.1420C>T	Unverified	gag	ccc	aca	1	C	T
TCGA-29-1761	MMP16	ENST00000286614	p.P336P	c.1008C>T	Unverified	tat	ccc	gga	3	C	T
TCGA-09-0369	MMP2	ENST00000219070	p.A366A	c.1098C>T	Unverified	AGC	GCC	GGC	3	C	T
TCGA-09-0369	MMP2	ENST00000219070	p.A366A	c.1098C>T	Unverified	AGC	GCC	GGC	3	C	T
TCGA-20-0991	MMP21	ENST00000368808	p.T297M	c.890C>T	Verified	agg	acg	gga	2	C	T
TCGA-29-1775	MMP27	ENST00000260229	p.A482V	c.1445C>T	Unverified	AAA	GCA	CAT	2	C	T
TCGA-24-1845	MMP27	ENST00000260229	p.A232A	c.696C>T	Unverified	ACA	GCC	TTG	3	C	T
TCGA-23-1116	MMP8	ENST00000236826	p.D378D	c.1134C>T	Unverified	att	gac	gca	3	C	T

TCGA-13-0924	MOG	ENST00000376917	p.R233*	c.697C>T	Verified	CAT	CGA	AGA	1	C	T
TCGA-24-1474	MORC2	ENST00000215862	p.R674W	c.2020C>T	Verified	AGT	CGG	AAG	1	C	T
TCGA-04-1542	MOV10	ENST00000357443	p.S631L	c.1892C>T	Verified	GTC	TCG	GCC	2	C	T
TCGA-61-1737	MPL	ENST00000372470	p.P70L	c.209C>T	Unverified	tac	ccg	cgg	2	C	T
TCGA-13-1481	MPO	ENST00000225275	p.L463L	c.1387C>T	Verified	TAC	CTG	CCC	1	C	T
TCGA-29-1691	MPP3	ENST00000398389	p.T513M	c.1538C>T	Unverified	AAA	ACG	CCA	2	C	T
TCGA-29-2427	MPP4_ENST	ENST00000409474	p.P320L	c.959C>T	Verified	CAG	CCG	TAC	2	C	T
TCGA-25-2391	MRPL38	ENST00000309352	p.A208V	c.623C>T	Unverified	GAG	GCA	GAA	2	C	T
TCGA-13-1481	MRPS5	ENST00000272418	p.D407D	c.1221C>T	Verified	CTG	GAC	TGG	3	C	T
TCGA-13-0760	MS4A4E	ENST00000427611	p.P20L	c.59C>T	Verified	AAC	CCA	AAG	2	C	T
TCGA-29-1775	MSH2	ENST00000233146	p.A917V	c.2750C>T	Unverified	ata	gca	aag	2	C	T
TCGA-13-0885	MSL3	ENST00000312196	p.L253F	c.757C>T	Verified	GAC	CTT	TGT	1	C	T
TCGA-04-1361	MST4	ENST00000394334	p.L381L	c.1143C>T	Verified	gaa	ctc	gag	3	C	T
TCGA-04-1361	MST4	ENST00000394334	p.L381L	c.1143C>T	Verified	gaa	ctc	gag	3	C	T
TCGA-29-1693	MT-ATP6	ENST00000361899	p.L222L	c.664C>T	Unverified	TAC	CTG	CAC	1	C	T
TCGA-61-1895	MT-ATP6	ENST00000361899	p.L5L	c.13C>T	Unverified	AAT	CTG	TTC	1	C	T
TCGA-61-1900	MT-ATP6	ENST00000361899	p.S99F	c.296C>T	Unverified	CTA	TCT	ATA	2	C	T
TCGA-29-1693	MT-ATP6_EI	ENST00000361899_v68	p.L222L	c.664C>T	Unverified	TAC	CTG	CAC	1	C	T
TCGA-61-1895	MT-ATP6_EI	ENST00000361899_v68	p.L5L	c.13C>T	Unverified	AAT	CTG	TTC	1	C	T
TCGA-61-1900	MT-ATP6_EI	ENST00000361899_v68	p.S99F	c.296C>T	Unverified	CTA	TCT	ATA	2	C	T
TCGA-61-1913	MT-ATP8	ENST00000361851	p.L22L	c.66C>T	Unverified	TTC	CTC	ATC	3	C	T
TCGA-04-1342	MTF1	ENST00000373036	p.G178G	c.534C>T	Unverified	TGT	GGC	AAA	3	C	T
TCGA-61-1738	MTHFD2_EN	ENST00000394053	p.R54W	c.160C>T	Unverified	GTG	CGG	CAG	1	C	T
TCGA-61-1738	MTHFD2_EN	ENST00000394053	p.R54W	c.160C>T	Unverified	GTG	CGG	CAG	1	C	T
TCGA-23-2078	MTM1	ENST00000370396	p.P226S	c.676C>T	Verified	att	cca	gtg	1	C	T
TCGA-13-0791	MTMR1	ENST00000370390	p.T301M	c.902C>T	Verified	gca	acg	att	2	C	T
TCGA-61-1907	MTNR1B	ENST00000257068	p.D104D	c.312C>T	Unverified	TAT	GAC	GGC	3	C	T
TCGA-61-1733	MTO1	ENST00000370300	p.R328C	c.982C>T	Unverified	AAC	CGT	CTA	1	C	T
TCGA-61-1733	MTO1	ENST00000370300	p.R328C	c.982C>T	Unverified	AAC	CGT	CTA	1	C	T
TCGA-23-1021	MTUS1	ENST00000262102	p.Y243Y	c.729C>T	Verified	ACT	TAC	ACA	3	C	T
TCGA-29-1703	MUC16	ENST00000397910	p.T5962T	c.17886C>T	Unverified	CTC	ACC	TCT	3	C	T
TCGA-29-1703	MUC16_ENS	ENST00000331986	p.T1595T	c.4785C>T	Unverified	TTG	GAC	ACA	3	C	T
TCGA-13-0920	MUC16_ENS	ENST00000397910	p.I14083I	c.42249C>T	Verified	ACC	ATC	TCC	3	C	T
TCGA-29-1703	MUC16_ENS	ENST00000397910	p.T5962T	c.17886C>T	Unverified	CTC	ACC	TCT	3	C	T

TCGA-04-1652	MUC20	ENST00000447234	p.A521V	c.1562C>T	Unverified	GGA	GCT	CTG	2	C	T
TCGA-04-1652	MUC20	ENST00000447234	p.A521V	c.1562C>T	Unverified	GGA	GCT	CTG	2	C	T
TCGA-24-2289	MUC5AC	ENST00000349637	p.T4770M	c.14309C>T	Unverified	GGG	ACG	ACC	2	C	T
TCGA-13-0714	MUC6	ENST00000421673	p.S1676S	c.5028C>T	Unverified	ACC	AGC	GGG	3	C	T
TCGA-23-1122	MVD	ENST00000301012	p.Q76*	c.226C>T	Unverified	GGG	CAG	CCG	1	C	T
TCGA-23-1809	MVK	ENST00000228510	p.S150L	c.449C>T	Unverified	TAC	TCG	GTG	2	C	T
TCGA-24-1103	MX2	ENST00000330714	p.S147S	c.441C>T	Verified	GGC	AGC	GGA	3	C	T
TCGA-24-1470	MXRA5	ENST00000381114	p.P820S	c.2458C>T	Verified	CCA	CCT	TTT	1	C	T
TCGA-61-1895	MXRA5	ENST00000381114	p.A2044V	c.6131C>T	Unverified	GCG	GCA	CTG	2	C	T
TCGA-61-1895	MXRA5_ENS	ENST00000381114	p.A2044V	c.6131C>T	Unverified	GCG	GCA	CTG	2	C	T
TCGA-13-1505	MYCBPAP	NM_032133.2	p.R210C	c.628C>T	Unverified	cag	cgt	aac	1	C	T
TCGA-09-0369	MYCN	ENST00000281043	p.P358L	c.1073C>T	Verified	cgt	ccg	ctc	2	C	T
TCGA-09-0369	MYCN	ENST00000281043	p.P358L	c.1073C>T	Verified	cgt	ccg	ctc	2	C	T
TCGA-24-1563	MYH1	ENST00000226207	p.R1638C	c.4912C>T	Verified	AAC	CGC	ATG	1	C	T
TCGA-29-1785	MYH11	ENST00000338282	p.R1895C	c.5683C>T	Unverified	CAG	CGC	ATC	1	C	T
TCGA-29-1785	MYH11	ENST00000338282	p.R1895C	c.5683C>T	Unverified	CAG	CGC	ATC	1	C	T
TCGA-29-1785	MYH11_ENS	ENST00000396324	p.R1902C	c.5704C>T	Unverified	CAG	CGC	ATC	1	C	T
TCGA-29-1785	MYH11_ENS	ENST00000396324	p.R1902C	c.5704C>T	Unverified	CAG	CGC	ATC	1	C	T
TCGA-30-1856	MYH15	ENST00000273353	p.L892F	c.2674C>T	Unverified	TCC	CTC	ACT	1	C	T
TCGA-29-1763	MYH4	ENST00000255381	p.F233F	c.699C>T	Unverified	GCC	TTC	GGC	3	C	T
TCGA-13-0913	MYH7	ENST00000355349	p.R143W	c.427C>T	Verified	TAC	CGG	GGC	1	C	T
TCGA-61-1910	MYH9	ENST00000216181	p.T1009I	c.3026C>T	Unverified	CTC	ACA	GAA	2	C	T
TCGA-04-1331	MYLK	NM_053025	p.T690M	c.2069C>T	Unverified	gac	acg	ggc	2	C	T
TCGA-13-0887	MYLK3	SU_caMLCK	p.F752F	c.2256C>T	Verified	aac	ttc	ggc	3	C	T
TCGA-09-2044	MYO10	ENST00000274203	p.A1743V	c.5228C>T	Verified	TTT	GCT	TTG	2	C	T
TCGA-61-2008	MYO16	ENST00000357550	p.S1689S	c.5067C>T	Unverified	AAA	TCC	GCG	3	C	T
TCGA-13-0904	MYO18B	ENST00000335473	p.L736L	c.2208C>T	Unverified	CAG	CTC	CAG	3	C	T
TCGA-29-1783	MYO1A	ENST00000300119	p.R352C	c.1054C>T	Unverified	agc	cgc	ctc	1	C	T
TCGA-24-1604	MYO1F	ENST00000338257	p.Y242Y	c.726C>T	Verified	ACC	TAC	CAG	3	C	T
TCGA-04-1542	MYO3A	ENST00000265944	p.H1400Y	c.4198C>T	Verified	AAA	CAT	GAG	1	C	T
TCGA-13-1496	MYO7A	ENST00000409709	p.R302C	c.904C>T	Unverified	ATC	CGC	TCC	1	C	T
TCGA-24-2267	MYOCD	ENST00000343344	p.S185L	c.554C>T	Verified	GCC	TCA	GAT	2	C	T
TCGA-24-2267	MYOF	ENST00000359263	p.H927H	c.2781C>T	Verified	GGT	CAC	ACG	3	C	T
TCGA-29-1696	MYOM1	ENST00000356443	p.T1141I	c.3422C>T	Unverified	GGA	ACC	AAA	2	C	T

TCGA-29-1696	MYOM1	ENST00000356443	p.T1141I	c.3422C>T	Unverified	GGA	ACC	AAA	2	C	T
TCGA-61-2095	MYST4	ENST00000287239	p.Q2022*	c.6064C>T	Unverified	ATG	CAG	ATG	1	C	T
TCGA-04-1638	MYT1L	ENST00000399161	p.G209G	c.627C>T	Unverified	CTC	GGC	AAA	3	C	T
TCGA-20-0990	N4BP2L1	ENST00000380139	p.T136T	c.408C>T	Verified	CAG	ACC	GAA	3	C	T
TCGA-61-1998	NAA30	ENST00000556492	p.L191L	c.571C>T	Unverified	TCC	CTG	ACC	1	C	T
TCGA-29-1690	NAP1L3	ENST00000373079	p.P308L	c.923C>T	Unverified	gac	cct	aaa	2	C	T
TCGA-29-1691	NASP	ENST00000341288	p.A737V	c.2210C>T	Unverified	AAA	GCC	AAA	2	C	T
TCGA-24-1844	NAT2	ENST00000286479	p.Q143*	c.427C>T	Unverified	GAT	CAG	CCT	1	C	T
TCGA-23-1124	NAV3	NM_014903.3	p.R1937C	c.5809C>T	Verified	aga	cgt	ctc	1	C	T
TCGA-13-0807	NBEA	ENST00000310336	p.R1325W	c.3973C>T	Verified	CCA	CGG	ACT	1	C	T
TCGA-13-1496	NBL1	ENST00000375136	p.T41T	c.123C>T	Verified	ATC	ACC	CAG	3	C	T
TCGA-13-1498	NBL1	ENST00000375136	p.T71T	c.213C>T	Unverified	AAC	ACC	TTC	3	C	T
TCGA-23-1122	NBPF14	ENST00000369219	p.L909F	c.2725C>T	Verified	AGT	CTC	CAC	1	C	T
TCGA-04-1347	NCAM2	ENST00000400546	p.P302S	c.904C>T	Verified	CAG	CCT	CAC	1	C	T
TCGA-61-1899	NCAM2	ENST00000400546	p.T79T	c.237C>T	Unverified	TTA	ACC	ATC	3	C	T
TCGA-25-1313	NCAPD2	ENST00000315579	p.C816C	c.2448C>T	Verified	GTG	TGC	CAT	3	C	T
TCGA-29-1775	NCAPD3	ENST00000534548	p.D1031D	c.3093C>T	Unverified	cca	gac	att	3	C	T
TCGA-29-2427	NCAPG2	NM_017760.4	p.F289F	c.867C>T	Verified	gac	ttc	atg	3	C	T
TCGA-13-0793	NCOA1	ENST00000406961	p.P356L	c.1067C>T	Verified	CAA	CCT	TTC	2	C	T
TCGA-61-1895	NCOA3	ENST00000371998	p.D718D	c.2154C>T	Unverified	ggg	gac	gga	3	C	T
TCGA-13-1497	NCOA6	ENST00000374796	p.R1323W	c.3967C>T	Verified	AAA	CGG	GCA	1	C	T
TCGA-13-1510	NDST1	ENST00000261797	p.R80C	c.238C>T	Unverified	TCC	CGC	ACA	1	C	T
TCGA-20-1686	NDUFAF1	ENST00000260361	p.R217W	c.649C>T	Unverified	GGT	CGG	CCT	1	C	T
TCGA-23-1809	NEB	ENST00000409198	p.L2634L	c.7900C>T	Unverified	TGC	CTG	CCC	1	C	T
TCGA-23-1809	NEB_ENST0(	ENST00000397345	p.L2634L	c.7900C>T	Unverified	TGC	CTG	CCC	1	C	T
TCGA-23-1809	NEB_ENST0(	ENST00000427231	p.L2634L	c.7900C>T	Unverified	TGC	CTG	CCC	1	C	T
TCGA-61-1915	NEBL_ENST(	ENST00000417816	p.N40N	c.120C>T	Unverified	CTC	AAC	ATG	3	C	T
TCGA-13-1481	NECAB2	ENST00000305202	p.L365L	c.1093C>T	Verified	ACA	CTG	AGC	1	C	T
TCGA-30-1714	NEK11	ENST00000429253	p.R53*	c.157C>T	Unverified	AAA	CGA	GGA	1	C	T
TCGA-04-1652	NES	ENST00000368223	p.R766W	c.2296C>T	Unverified	CGA	CGG	AGG	1	C	T
TCGA-04-1652	NES	ENST00000368223	p.R766W	c.2296C>T	Unverified	CGA	CGG	AGG	1	C	T
TCGA-13-0900	NEU1	ENST00000375631	p.R294C	c.880C>T	Verified	CTC	CGC	AGC	1	C	T
TCGA-24-2035	NEU3	ENST00000294064	p.H437Y	c.1309C>T	Verified	ACA	CAC	CGG	1	C	T
TCGA-20-0991	NEUROD4	ENST00000242994	p.D105D	c.315C>T	Verified	AAT	GAC	GCC	3	C	T

TCGA-04-1367	NF1	ENST00000358273	p.Q112*	c.334C>T	Verified	AAA	CAG	TTG	1	C	T
TCGA-24-2024	NFASC	ENST00000360049	p.R844C	c.2530C>T	Verified	AAC	CGC	GTC	1	C	T
TCGA-04-1356	NFATC1	ENST00000329101	p.H554H	c.1662C>T	Unverified	gtt	cac	gtc	3	C	T
TCGA-24-1850	NFATC2	ENST00000396009	p.T512I	c.1535C>T	Unverified	GCA	ACC	ATC	2	C	T
TCGA-09-1674	NFE2L1	ENST00000362042	p.F625F	c.1875C>T	Unverified	CCT	TTC	ACC	3	C	T
TCGA-29-1761	NFKB1	ENST00000226574	p.A111V	c.332C>T	Unverified	CAT	GCC	CAC	2	C	T
TCGA-24-2290	NFRKB	ENST00000524794	p.A1056A	c.3168C>T	Verified	AGT	GCC	CCT	3	C	T
TCGA-24-2290	NFRKB	ENST00000524794	p.A1056A	c.3168C>T	Verified	AGT	GCC	CCT	3	C	T
TCGA-13-1498	NFS1	ENST00000374092	p.Q235*	c.703C>T	Verified	GCC	CAG	GCT	1	C	T
TCGA-24-1845	NIP7	ENST00000254940	p.P158S	c.472C>T	Unverified	GAC	CCC	ATG	1	C	T
TCGA-25-2042	NIT2	ENST00000394140	p.H221Y	c.661C>T	Verified	GGA	CAC	AGC	1	C	T
TCGA-24-2267	NKAP	ENST00000371410	p.A86V	c.257C>T	Verified	TCT	GCG	CCC	2	C	T
TCGA-24-1423	NLGN4X	ENST00000381095	p.D617D	c.1851C>T	Unverified	cca	gac	atg	3	C	T
TCGA-13-0893	NLRP12	ENST00000324134	p.N505N	c.1515C>T	Verified	ATC	AAC	TGT	3	C	T
TCGA-29-1781	NLRP2_ENST	ENST00000543010	p.L813L	c.2437C>T	Unverified	TCC	CTG	ACG	1	C	T
TCGA-29-1770	NLRP3	ENST00000391828	p.P294L	c.881C>T	Unverified	aaa	ccc	tcc	2	C	T
TCGA-24-1563	NLRP5	ENST00000390649	p.R562C	c.1684C>T	Verified	CTC	CGT	GCT	1	C	T
TCGA-04-1342	NLRP5	ENST00000390649	p.T281M	c.842C>T	Verified	CGC	ACG	GTG	2	C	T
TCGA-61-1914	NLRP5_ENST	ENST00000390649	p.I1061I	c.3183C>T	Unverified	GTG	ATC	TCG	3	C	T
TCGA-24-1844	NLRP7	ENST00000448121	p.L551L	c.1651C>T	Unverified	TTG	CTG	CAA	1	C	T
TCGA-24-1844	NLRP7_ENST	ENST00000328092	p.L551L	c.1651C>T	Unverified	TTG	CTG	CAA	1	C	T
TCGA-23-1032	NMBR	ENST00000258042	p.S102L	c.305C>T	Unverified	GCC	TCG	CGC	2	C	T
TCGA-61-1907	NMT2	ENST00000378165	p.R148C	c.442C>T	Unverified	GTA	CGC	CAA	1	C	T
TCGA-04-1652	NMUR2	ENST00000255262	p.T123M	c.368C>T	Unverified	AAG	ACG	GCC	2	C	T
TCGA-04-1652	NMUR2	ENST00000255262	p.T123M	c.368C>T	Unverified	AAG	ACG	GCC	2	C	T
TCGA-24-1850	NNT	ENST00000264663	p.G804G	c.2412C>T	Unverified	ACT	GGC	ATC	3	C	T
TCGA-29-1695	NOBOX	ENST00000223140	p.R324W	c.970C>T	Unverified	CGC	CGG	GCC	1	C	T
TCGA-04-1638	NOBOX	ENST00000223140	p.S54S	c.162C>T	Unverified	AGC	TCC	TTC	3	C	T
TCGA-29-1695	NOBOX_ENS	ENST00000467773	p.R356W	c.1066C>T	Unverified	CGC	CGG	GCC	1	C	T
TCGA-04-1638	NOBOX_ENS	ENST00000467773	p.S54S	c.162C>T	Unverified	AGC	TCC	TTC	3	C	T
TCGA-04-1542	NOD2	ENST00000300589	p.Q571*	c.1711C>T	Verified	ctc	cag	gca	1	C	T
TCGA-29-1711	NODAL	ENST00000287139	p.P182L	c.545C>T	Unverified	TGG	CCG	CGG	2	C	T
TCGA-29-1711	NODAL	ENST00000287139	p.P182L	c.545C>T	Unverified	TGG	CCG	CGG	2	C	T
TCGA-25-2391	NOG	ENST00000332822	p.S195S	c.585C>T	Unverified	CCG	TCC	AAG	3	C	T

TCGA-13-0916	NOLC1	ENST00000370007	p.R262W	c.784C>T	Verified	ACC	CGG	AAG	1	C	T
TCGA-24-2280	NOMO1	ENST00000287667	p.A599V	c.1796C>T	Unverified	CAC	GCC	ATC	2	C	T
TCGA-04-1331	NOP56	ENST00000329276	p.Q215*	c.643C>T	Unverified	GCC	CAG	TTT	1	C	T
TCGA-61-2012	NOTCH1	ENST00000277541	p.R2327W	c.6979C>T	Unverified	CTG	CGG	GGG	1	C	T
TCGA-29-2427	NOTCH1	ENST00000277541	p.D1807D	c.5421C>T	Unverified	ATG	GAC	GAC	3	C	T
TCGA-61-2012	NOTCH1_EN	ENST00000277541	p.R2327W	c.6979C>T	Unverified	CTG	CGG	GGG	1	C	T
TCGA-29-2427	NOTCH1_EN	ENST00000277541	p.D1807D	c.5421C>T	Unverified	ATG	GAC	GAC	3	C	T
TCGA-24-1849	NOTCH2	ENST00000256646	p.R5R	c.15C>T	Unverified	ctg	cgc	ccc	3	C	T
TCGA-04-1347	NOTCH4	ENST00000375023	p.R132C	c.394C>T	Verified	ggc	cgc	tgc	1	C	T
TCGA-61-1915	NOTCH4	ENST00000375023	p.P1173S	c.3517C>T	Unverified	aaa	ccc	gga	1	C	T
TCGA-09-2044	NPBWR1	ENST00000331251	p.T323I	c.968C>T	Verified	ATA	ACT	TGC	2	C	T
TCGA-29-1768	NPC1	ENST00000269228	p.S1197S	c.3591C>T	Unverified	AGC	TCC	GTG	3	C	T
TCGA-23-1114	NPC1	ENST00000269228	p.S809S	c.2427C>T	Unverified	ACA	AGC	GTC	3	C	T
TCGA-10-0930	NPC1L1	ENST00000289547	p.P1149P	c.3447C>T	Verified	GTG	CCC	ACC	3	C	T
TCGA-10-0930	NPC1L1	ENST00000289547	p.P1149P	c.3447C>T	Verified	GTG	CCC	ACC	3	C	T
TCGA-29-1784	NPHP4	ENST00000378156	p.S209F	c.626C>T	Unverified	GTG	TCT	GGT	2	C	T
TCGA-13-1509	NPHS1	ENST00000378910	p.R695W	c.2083C>T	Verified	CCC	CGG	CAT	1	C	T
TCGA-29-1785	NPHS1	ENST00000378910	p.Y1193Y	c.3579C>T	Unverified	CTC	TAC	GAT	3	C	T
TCGA-29-1785	NPHS1	ENST00000378910	p.Y1193Y	c.3579C>T	Unverified	CTC	TAC	GAT	3	C	T
TCGA-13-0795	NR2C1	ENST00000333003	p.N317N	c.951C>T	Verified	ACC	AAC	GGT	3	C	T
TCGA-61-1904	NR3C2	ENST00000358102	p.R28C	c.82C>T	Unverified	GAG	CGT	TCT	1	C	T
TCGA-13-1488	NRK	SU_ZC4-NRK	p.S108S	c.324C>T	Verified	gtg	tcc	ttc	3	C	T
TCGA-23-2078	NRXN1_ENS	ENST00000406316	p.T1035I	c.3104C>T	Verified	GAA	ACA	TAC	2	C	T
TCGA-24-1847	NRXN3_ENS	ENST00000428277	p.S42S	c.126C>T	Unverified	TCC	TCC	TCC	3	C	T
TCGA-04-1338	NT5E	ENST00000257770	p.S438S	c.1314C>T	Verified	CAT	AGC	GTG	3	C	T
TCGA-25-1313	NTRK1	ENST00000524377	p.R649W	c.1945C>T	Verified	CAC	CGG	GAC	1	C	T
TCGA-24-2280	NTRK1	ENST00000524377	p.T264M	c.791C>T	Unverified	GTG	ACG	TGC	2	C	T
TCGA-13-1498	NTRK1	ENST00000524377	p.V634V	c.1902C>T	Verified	CAG	GTC	GCT	3	C	T
TCGA-24-2280	NTRK1_ENST	ENST00000392302	p.T234M	c.701C>T	Unverified	GTG	ACG	TGC	2	C	T
TCGA-61-1733	NTRK2	ENST00000376214	p.N355N	c.1065C>T	Unverified	ATG	AAC	AAT	3	C	T
TCGA-61-1733	NTRK2	ENST00000376214	p.N355N	c.1065C>T	Unverified	ATG	AAC	AAT	3	C	T
TCGA-61-1733	NTRK2_ENST	ENST00000304053	p.N355N	c.1065C>T	Unverified	ATG	AAC	AAT	3	C	T
TCGA-61-1733	NTRK2_ENST	ENST00000304053	p.N355N	c.1065C>T	Unverified	ATG	AAC	AAT	3	C	T
TCGA-61-1733	NTRK2_ENST	ENST00000376214	p.N355N	c.1065C>T	Unverified	ATG	AAC	AAT	3	C	T

TCGA-61-1733	NTRK2_ENST	ENST00000376214	p.N355N	c.1065C>T	Unverified	ATG	AAC	AAT	3	C	T
TCGA-24-1845	NTRK3	ENST00000394480	p.P304L	c.911C>T	Unverified	TAT	CCC	CCA	2	C	T
TCGA-24-1845	NTRK3_ENST	ENST00000317501	p.P304L	c.911C>T	Unverified	TAT	CCC	CCA	2	C	T
TCGA-24-1845	NTRK3_ENST	ENST00000360948	p.P304L	c.911C>T	Unverified	TAT	CCC	CCA	2	C	T
TCGA-24-1844	NUDT11	ENST00000375992	p.S27S	c.81C>T	Unverified	cgg	agc	gaa	3	C	T
TCGA-61-1914	NUDT16	ENST00000359850	p.G132G	c.396C>T	Unverified	ATT	GGC	TCT	3	C	T
TCGA-24-2289	NUMA1	ENST00000393695	p.A601V	c.1802C>T	Unverified	gat	gcg	gct	2	C	T
TCGA-23-1022	NUMBL	ENST00000252891	p.R94W	c.280C>T	Verified	TCC	CGG	GGA	1	C	T
TCGA-24-1850	NUP155	ENST00000231498	p.A646A	c.1938C>T	Unverified	CAG	GCC	ACA	3	C	T
TCGA-13-1481	NUP160	ENST00000378460	p.P768S	c.2302C>T	Verified	GCT	CCC	CTA	1	C	T
TCGA-23-1123	NUP160	ENST00000378460	p.R43W	c.127C>T	Verified	GAA	CGG	AGC	1	C	T
TCGA-23-1123	NUP160	ENST00000378460	p.R43W	c.127C>T	Verified	GAA	CGG	AGC	1	C	T
TCGA-04-1342	NUP160	ENST00000378460	p.R53R	c.159C>T	Verified	GAG	CGC	GAA	3	C	T
TCGA-13-1512	NUP210	ENST00000254508	p.D1221D	c.3663C>T	Verified	CTG	GAC	CTC	3	C	T
TCGA-29-1693	NXF2	ENST00000372757	p.T101M	c.302C>T	Unverified	ACC	ACG	TGG	2	C	T
TCGA-24-1845	NXF2B	ENST00000372750	p.P108S	c.322C>T	Unverified	CCT	CCG	GAG	1	C	T
TCGA-13-1498	O52L2_HUM	ENST00000316540	p.Q315*	c.943C>T	Verified	ACC	CAG	AAG	1	C	T
TCGA-13-0890	O95431_HU	ENST00000238638	p.T128I	c.383C>T	Verified	CGA	ACC	AGC	2	C	T
TCGA-24-1845	OASL	ENST00000257570	p.Q435*	c.1303C>T	Unverified	ATC	CAG	GTC	1	C	T
TCGA-61-1738	OASL	ENST00000257570	p.I178I	c.534C>T	Unverified	CTG	ATC	AAG	3	C	T
TCGA-61-1738	OASL	ENST00000257570	p.I178I	c.534C>T	Unverified	CTG	ATC	AAG	3	C	T
TCGA-23-1029	OBFC1	ENST00000369764	p.D156D	c.468C>T	Unverified	GTG	GAC	GAC	3	C	T
TCGA-24-1845	OCA2	ENST00000354638	p.Q309*	c.925C>T	Unverified	CTG	CAG	CAG	1	C	T
TCGA-29-1691	ODC1	ENST00000234111	p.R165C	c.493C>T	Unverified	TGT	CGT	CTC	1	C	T
TCGA-13-0913	ODF2L	ENST00000370567	p.T444M	c.1331C>T	Verified	TTG	ACG	GCG	2	C	T
TCGA-13-0904	ODZ1	ENST00000371130	p.A2258V	c.6773C>T	Verified	GTC	GCG	AGT	2	C	T
TCGA-29-1699	ODZ2	ENST00000388903	p.T2539M	c.7616C>T	Unverified	AGC	ACC	GGG	2	C	T
TCGA-29-1699	ODZ2_ENST	ENST00000518659	p.T2706M	c.8117C>T	Unverified	GGC	ACG	GCC	2	C	T
TCGA-29-1699	ODZ2_ENST	ENST00000519204	p.T2585M	c.7754C>T	Unverified	GGC	ACG	GCC	2	C	T
TCGA-61-1737	OFCC1	ENST00000331403	p.T109M	c.326C>T	Unverified	GCG	ACG	GAG	2	C	T
TCGA-29-1784	OFCC1	ENST00000331403	p.Y89Y	c.267C>T	Unverified	GAA	TAC	GGC	3	C	T
TCGA-09-2051	OGDHL	ENST00000374103	p.N482N	c.1446C>T	Unverified	AGA	AAC	ACT	3	C	T
TCGA-61-1910	OGN	ENST00000375561	p.D110D	c.330C>T	Unverified	GTT	GAC	ATT	3	C	T
TCGA-29-1766	OLFML1	ENST00000329293	p.T53M	c.158C>T	Unverified	GCA	ACG	AGG	2	C	T

TCGA-61-2009	OMG	ENST00000247271	p.P253L	c.758C>T	Verified	ACT	CCA	TGT	2	C	T
TCGA-61-2012	ONECUT2	ENST00000262095	p.R438C	c.1312C>T	Verified	CAA	CGC	CGA	1	C	T
TCGA-20-1683	OPA1	ENST00000361510	p.R873W	c.2617C>T	Unverified	GTC	CGG	AAG	1	C	T
TCGA-24-0975	OPLAH	ENST00000360660	p.H29H	c.87C>T	Unverified	GGG	CAC	GTG	3	C	T
TCGA-09-0369	OR10A7	ENST00000326258	p.T232I	c.695C>T	Verified	GCC	ACT	GGC	2	C	T
TCGA-09-0369	OR10A7	ENST00000326258	p.T232I	c.695C>T	Verified	GCC	ACT	GGC	2	C	T
TCGA-24-2288	OR10C1	ENST00000444197	p.T307M	c.920C>T	Unverified	AAA	ACG	GTG	2	C	T
TCGA-24-2024	OR10G4	ENST00000320891	p.A297V	c.890C>T	Verified	AAA	GCT	GTG	2	C	T
TCGA-13-0885	OR10J3	ENST00000332217	p.S268F	c.803C>T	Verified	AGT	TCC	CTG	2	C	T
TCGA-24-1464	OR10Q1	ENST00000316770	p.R142R	c.426C>T	Verified	ACC	CGC	GAG	3	C	T
TCGA-29-1763	OR10R2	ENST00000368152	p.T295M	c.884C>T	Unverified	GTG	ACG	GTG	2	C	T
TCGA-24-2289	OR10W1	ENST00000395079	p.G55G	c.165C>T	Unverified	CTG	GGC	AGC	3	C	T
TCGA-23-1124	OR11H4	ENST00000315409	p.S85S	c.255C>T	Verified	GTG	TCC	TCC	3	C	T
TCGA-13-0755	OR12D3	ENST00000377143	p.A110V	c.329C>T	Verified	GAG	GCC	ATT	2	C	T
TCGA-25-1326	OR1K1	ENST00000277309	p.P132S	c.394C>T	Verified	CTC	CCC	TAT	1	C	T
TCGA-13-1507	OR1Q1	ENST00000297913	p.G233G	c.699C>T	Verified	AAG	GGC	AGG	3	C	T
TCGA-09-2049	OR1S2	ENST00000302592	p.H189H	c.567C>T	Verified	CCA	CAC	TTC	3	C	T
TCGA-24-1422	OR2A5	ENST00000408906	p.I181I	c.543C>T	Verified	GAA	ATC	CTG	3	C	T
TCGA-29-1784	OR2F2	ENST00000408955	p.P79S	c.235C>T	Unverified	GTC	CCC	CAG	1	C	T
TCGA-13-1497	OR2G6	ENST00000343414	p.T193I	c.578C>T	Verified	ACG	ACT	TTC	2	C	T
TCGA-25-2393	OR2G6	ENST00000343414	p.Y132Y	c.396C>T	Unverified	CGC	TAC	ATA	3	C	T
TCGA-29-1766	OR2J2	ENST00000377167	p.T241I	c.722C>T	Unverified	AGG	ACA	TGT	2	C	T
TCGA-61-1899	OR2M4	ENST00000306687	p.L15L	c.43C>T	Unverified	CTG	CTG	GGA	1	C	T
TCGA-30-1714	OR2M4	ENST00000306687	p.T267M	c.800C>T	Unverified	CAT	ACG	CCA	2	C	T
TCGA-29-1690	OR2T2	ENST00000342927	p.I222I	c.666C>T	Unverified	CAC	ATC	CTC	3	C	T
TCGA-13-0916	OR2T6	ENST00000355728	p.G88G	c.264C>T	Verified	ATG	GGC	GAG	3	C	T
TCGA-24-1422	OR4A47	ENST00000446524	p.P181L	c.542C>T	Verified	TAT	CCC	TTA	2	C	T
TCGA-23-1114	OR4M2	ENST00000332663	p.P282S	c.844C>T	Unverified	TTC	CCT	TTA	1	C	T
TCGA-29-1695	OR4X1	ENST00000320048	p.R120C	c.358C>T	Unverified	GAC	CGC	TAT	1	C	T
TCGA-29-1699	OR52A5	ENST00000307388	p.Q301*	c.901C>T	Unverified	AAG	CAA	ATT	1	C	T
TCGA-24-1850	OR52K2	ENST00000325719	p.T298T	c.894C>T	Unverified	AAG	ACC	AAG	3	C	T
TCGA-29-1761	OR52N1	ENST00000317078	p.T7T	c.21C>T	Unverified	GGC	ACC	AGC	3	C	T
TCGA-29-1761	OR5B2	ENST00000302581	p.S169F	c.506C>T	Unverified	AAA	TCC	AAT	2	C	T
TCGA-61-1740	OR5F1	ENST00000278409	p.T110T	c.330C>T	Unverified	ACA	ACC	GAA	3	C	T

TCGA-13-1496	OR5H2	ENST00000355273	p.L53F	c.157C>T	Verified	GCT	CTT	ATC	1	C	T
TCGA-13-0920	OR5I1	ENST00000301532	p.T59T	c.177C>T	Unverified	CAA	ACC	CCC	3	C	T
TCGA-13-1499	OR5M1_ENS	ENST00000526538	p.L107L	c.319C>T	Verified	GCC	CTG	GTG	1	C	T
TCGA-25-1313	OR5T1	ENST00000313033	p.F322F	c.966C>T	Verified	TGG	TTC	ATA	3	C	T
TCGA-25-2398	OR6A2	ENST00000332601	p.R266W	c.796C>T	Verified	GCT	CGG	CCA	1	C	T
TCGA-24-2280	OR6B3	ENST00000319423	p.R91C	c.271C>T	Unverified	AAA	CGC	ATC	1	C	T
TCGA-29-1702	OR6K6	ENST00000368144	p.F96F	c.288C>T	Unverified	TCC	TTC	CTG	3	C	T
TCGA-09-2050	OR6X1	ENST00000327930	p.F166F	c.498C>T	Verified	CCA	TTC	TGT	3	C	T
TCGA-29-1764	OR7D4	ENST00000308682	p.Y278Y	c.834C>T	Unverified	ATG	TAC	GCC	3	C	T
TCGA-61-2094	OR7E24	ENST00000456448	p.S82F	c.245C>T	Unverified	CTC	TCC	AAC	2	C	T
TCGA-29-1695	OR8H1	ENST00000313022	p.T15M	c.44C>T	Unverified	CTT	ACG	GGA	2	C	T
TCGA-30-1718	OR8H3	ENST00000313472	p.N172N	c.516C>T	Unverified	TCA	AAC	ATA	3	C	T
TCGA-29-1770	OR8I2	ENST00000302124	p.V280V	c.840C>T	Unverified	ATT	GTC	ATT	3	C	T
TCGA-61-1740	OR8J3	ENST00000301529	p.G72G	c.216C>T	Unverified	CTT	GGC	AAC	3	C	T
TCGA-13-0714	OR9Q1	ENST00000335397	p.A230V	c.689C>T	Verified	CCT	GCT	GGA	2	C	T
TCGA-29-1776	ORC2	ENST00000234296	p.T450I	c.1349C>T	Unverified	act	aca	tac	2	C	T
TCGA-25-2392	OSBPL1A	ENST00000319481	p.C872C	c.2616C>T	Verified	GAC	TGC	AGG	3	C	T
TCGA-24-2267	OTOF	ENST00000272371	p.R1680C	c.5038C>T	Unverified	TGC	CGC	CTG	1	C	T
TCGA-25-2392	OTOF	ENST00000272371	p.P476S	c.1426C>T	Unverified	GAG	CCC	CTG	1	C	T
TCGA-61-1904	OTOF	ENST00000272371	p.P218S	c.652C>T	Unverified	GAT	CCC	GAC	1	C	T
TCGA-13-1497	OTOF	ENST00000272371	p.S1456F	c.4367C>T	Verified	GGC	TCC	CTC	2	C	T
TCGA-24-2267	OTOF_ENST	ENST00000339598	p.R913C	c.2737C>T	Unverified	TGC	CGC	CTG	1	C	T
TCGA-13-1497	OTOF_ENST	ENST00000339598	p.S689F	c.2066C>T	Verified	GGC	TCC	CTC	2	C	T
TCGA-04-1362	OTOP2	ENST00000331427	p.R548C	c.1642C>T	Verified	TAC	CGC	ATG	1	C	T
TCGA-13-1497	OTOP3	ENST00000328801	p.L534F	c.1600C>T	Verified	TTC	CTC	ATC	1	C	T
TCGA-04-1655	OTUD7B	ENST00000369135	p.R233C	c.697C>T	Unverified	AGG	CGC	TGG	1	C	T
TCGA-61-2009	OVCH1	ENST00000318184	p.A477V	c.1430C>T	Verified	GAT	GCT	GTT	2	C	T
TCGA-61-1738	P2RX1	ENST00000225538	p.N153N	c.459C>T	Unverified	TTC	AAC	GAC	3	C	T
TCGA-61-1738	P2RX1	ENST00000225538	p.N153N	c.459C>T	Unverified	TTC	AAC	GAC	3	C	T
TCGA-29-1702	P2RY1	ENST00000305097	p.A327V	c.980C>T	Unverified	TTG	GCG	GGA	2	C	T
TCGA-04-1638	P2RY11	ENST00000321826	p.R103C	c.307C>T	Unverified	TGC	CGC	CTG	1	C	T
TCGA-29-1693	P2RY11	ENST00000321826	p.A313A	c.939C>T	Unverified	CTG	GCC	TTC	3	C	T
TCGA-09-2044	PACS1	ENST00000320580	p.R203W	c.607C>T	Verified	AAT	CGG	ACC	1	C	T
TCGA-30-1856	PADI2	NM_007365.1	p.R126W	c.376C>T	Unverified	gac	cgg	gat	1	C	T

TCGA-61-1914	PAH	ENST00000553106	p.I174I	c.522C>T	Unverified	CCC	ATC	CCT	3	C	T
TCGA-29-1698	PAK6	ENST00000441369	p.S628S	c.1884C>T	Unverified	gtc	tcc	cca	3	C	T
TCGA-13-1498	PAK7	ENST00000378429	p.S260S	c.780C>T	Verified	ccc	agc	ctg	3	C	T
TCGA-09-2056	PALB2	ENST00000261584	p.Q370*	c.1108C>T	Verified	CTT	CAG	GAA	1	C	T
TCGA-23-1124	PALLD	ENST00000261509	p.H823H	c.2469C>T	Verified	GAT	CAC	TAC	3	C	T
TCGA-24-1850	PALM2	ENST00000374531	p.R70W	c.208C>T	Unverified	AGG	CGG	CAG	1	C	T
TCGA-24-1850	PALM2-AKA	ENST00000374530	p.R68W	c.202C>T	Unverified	AGG	CGG	CAG	1	C	T
TCGA-13-0760	PAMR1	ENST00000278360	p.R699C	c.2095C>T	Verified	CCA	CGC	TGG	1	C	T
TCGA-24-1846	PAPOLA	ENST00000216277	p.N557N	c.1671C>T	Unverified	AGA	AAC	AGT	3	C	T
TCGA-29-1761	PAPPA2	ENST00000367662	p.D1547D	c.4641C>T	Unverified	CAC	GAC	GTG	3	C	T
TCGA-29-1784	PAPPA2	ENST00000367662	p.P847P	c.2541C>T	Unverified	ATC	CCC	ATT	3	C	T
TCGA-09-1665	PAQR5	ENST00000395407	p.S98S	c.294C>T	Verified	GTG	TCC	AGC	3	C	T
TCGA-13-1505	PARD6A	ENST00000219255	p.R262C	c.784C>T	Verified	GGG	CGT	TTG	1	C	T
TCGA-24-2024	PARP1	ENST00000366794	p.T556T	c.1668C>T	Verified	GCC	ACC	CTT	3	C	T
TCGA-24-1422	PARP12	ENST00000263549	p.R582W	c.1744C>T	Verified	TGG	CGG	GTC	1	C	T
TCGA-61-1914	PARP9	ENST00000360356	p.P843S	c.2527C>T	Unverified	CAT	CCT	TGG	1	C	T
TCGA-23-1117	PASD1	ENST00000370357	p.A194V	c.581C>T	Verified	GAA	GCT	GTA	2	C	T
TCGA-23-1117	PASD1	ENST00000370357	p.A194V	c.581C>T	Verified	GAA	GCT	GTA	2	C	T
TCGA-25-1318	PASD1	ENST00000370357	p.T675I	c.2024C>T	Verified	ATA	ACT	TCA	2	C	T
TCGA-25-1318	PASD1	ENST00000370357	p.T675I	c.2024C>T	Verified	ATA	ACT	TCA	2	C	T
TCGA-13-1481	PCDH12	ENST00000231484	p.S992S	c.2976C>T	Verified	GGC	AGC	AGG	3	C	T
TCGA-24-1470	PCDH15	ENST00000361849	p.L429L	c.1285C>T	Verified	GCT	CTG	GAC	1	C	T
TCGA-61-1906	PCDH15	ENST00000361849	p.R962C	c.2884C>T	Unverified	AGT	CGT	GTG	1	C	T
TCGA-61-1906	PCDH15	ENST00000361849	p.R962C	c.2884C>T	Unverified	AGT	CGT	GTG	1	C	T
TCGA-61-1906	PCDH15_EN	ENST00000395438	p.R962C	c.2884C>T	Unverified	AGT	CGT	GTG	1	C	T
TCGA-61-1906	PCDH15_EN	ENST00000395438	p.R962C	c.2884C>T	Unverified	AGT	CGT	GTG	1	C	T
TCGA-61-1906	PCDH15_EN	ENST00000414778	p.R967C	c.2899C>T	Unverified	AGT	CGT	GTG	1	C	T
TCGA-61-1906	PCDH15_EN	ENST00000414778	p.R967C	c.2899C>T	Unverified	AGT	CGT	GTG	1	C	T
TCGA-61-1906	PCDH15_EN	ENST00000417177	p.R967C	c.2899C>T	Unverified	AGT	CGT	GTG	1	C	T
TCGA-61-1906	PCDH15_EN	ENST00000417177	p.R967C	c.2899C>T	Unverified	AGT	CGT	GTG	1	C	T
TCGA-29-1784	PCDH19	ENST00000373034	p.P566S	c.1696C>T	Unverified	GCC	CCA	CCT	1	C	T
TCGA-29-1784	PCDH19_NM	NM_020766.1	p.P67S	c.199C>T	Unverified	gcc	cca	cct	1	C	T
TCGA-13-1489	PCDH9	ENST00000377865	p.P1145S	c.3433C>T	Verified	ATG	CCT	CCT	1	C	T
TCGA-24-2035	PCDHA10	ENST00000506939	p.S614S	c.1842C>T	Verified	CAA	TCC	GGT	3	C	T

TCGA-23-2078	PCDHA13	ENST00000289272	p.S280L	c.839C>T	Unverified	TAC	TCA	TTT	2	C	T
TCGA-24-1843	PCDHA13	ENST00000289272	p.D481D	c.1443C>T	Unverified	CAG	GAC	GCG	3	C	T
TCGA-24-2035	PCDHA13	ENST00000289272	p.S879S	c.2637C>T	Verified	CAA	TCC	GGT	3	C	T
TCGA-09-2050	PCDHA2	ENST00000378132	p.S411S	c.1233C>T	Verified	GAC	AGC	GCC	3	C	T
TCGA-61-1998	PCDHA4	ENST00000356878	p.P134S	c.400C>T	Unverified	TTC	CCA	GCA	1	C	T
TCGA-29-1766	PCDHA4	ENST00000356878	p.F455F	c.1365C>T	Unverified	GCG	TTC	GCG	3	C	T
TCGA-29-1766	PCDHA4_EN	ENST00000530339	p.F455F	c.1365C>T	Unverified	GCG	TTC	GCG	3	C	T
TCGA-24-2035	PCDHAC1	ENST00000253807	p.S892S	c.2676C>T	Verified	CAA	TCC	GGT	3	C	T
TCGA-24-2035	PCDHAC2	ENST00000289269	p.S936S	c.2808C>T	Verified	CAA	TCC	GGT	3	C	T
TCGA-24-1470	PCDHB10	ENST00000239446	p.R792*	c.2374C>T	Verified	TTC	CGA	AAT	1	C	T
TCGA-24-2262	PCDHB11	ENST00000354757	p.N555N	c.1665C>T	Unverified	GCC	AAC	GAC	3	C	T
TCGA-04-1652	PCDHB12	ENST00000239450	p.R289C	c.865C>T	Unverified	ATT	CGC	AAG	1	C	T
TCGA-04-1652	PCDHB12	ENST00000239450	p.R289C	c.865C>T	Unverified	ATT	CGC	AAG	1	C	T
TCGA-24-1435	PCDHB12	ENST00000239450	p.A677A	c.2031C>T	Unverified	GCG	GCC	CCG	3	C	T
TCGA-23-1114	PCDHB13	ENST00000341948	p.R530C	c.1588C>T	Unverified	TTC	CGC	GTG	1	C	T
TCGA-13-0916	PCDHB13	ENST00000341948	p.P221L	c.662C>T	Verified	TCT	CCG	CCC	2	C	T
TCGA-30-1855	PCDHB13	ENST00000341948	p.T626T	c.1878C>T	Unverified	CGC	ACC	GCC	3	C	T
TCGA-13-0884	PCDHB16	ENST00000361016	p.N169N	c.507C>T	Verified	CAA	AAC	TAT	3	C	T
TCGA-24-2267	PCDHB2	ENST00000194155	p.A678V	c.2033C>T	Verified	GAG	GCG	GCA	2	C	T
TCGA-13-0792	PCDHB5	ENST00000231134	p.R789W	c.2365C>T	Verified	TTC	CGG	AAT	1	C	T
TCGA-23-1031	PCDHGA12	ENST00000252085	p.S763L	c.2288C>T	Unverified	GAC	TCG	CGG	2	C	T
TCGA-23-1031	PCDHGA12_	ENST00000252085	p.S763L	c.2288C>T	Unverified	GAC	TCG	CGG	2	C	T
TCGA-04-1343	PCDHGA5	ENST00000518069	p.R805*	c.2413C>T	Verified	CGG	CGA	GTT	1	C	T
TCGA-24-1470	PCNT	ENST00000359568	p.Q2821*	c.8461C>T	Verified	CAG	CAG	CTT	1	C	T
TCGA-04-1337	PCSK1	ENST00000311106	p.L741L	c.2221C>T	Verified	CGG	CTG	CTT	1	C	T
TCGA-13-1501	PCSK2	ENST00000262545	p.R419W	c.1255C>T	Verified	AAA	CGG	AAC	1	C	T
TCGA-13-1501	PCSK2	ENST00000262545	p.R419W	c.1255C>T	Verified	AAA	CGG	AAC	1	C	T
TCGA-20-0991	PCSK2	ENST00000262545	p.T340I	c.1019C>T	Verified	AGG	ACT	GCC	2	C	T
TCGA-13-1512	PDCD11	ENST00000369797	p.P665S	c.1993C>T	Verified	ctc	ccc	aca	1	C	T
TCGA-04-1542	PDE11A	ENST00000286063	p.A341V	c.1022C>T	Verified	CAA	GCG	ATA	2	C	T
TCGA-04-1652	PDE1C	ENST00000396184	p.R112W	c.334C>T	Unverified	ACG	CGG	CAG	1	C	T
TCGA-04-1652	PDE1C	ENST00000396184	p.R112W	c.334C>T	Unverified	ACG	CGG	CAG	1	C	T
TCGA-29-1768	PDE1C	ENST00000396184	p.H132H	c.396C>T	Unverified	GTT	CAC	GCA	3	C	T
TCGA-04-1652	PDE1C_ENS	ENST00000396191	p.R112W	c.334C>T	Unverified	ACG	CGG	CAG	1	C	T

TCGA-04-1652	PDE1C_ENS	ENST00000396191	p.R112W	c.334C>T	Unverified	ACG	CGG	CAG	1	C	T
TCGA-29-1768	PDE1C_ENS	ENST00000396191	p.H132H	c.396C>T	Unverified	GTT	CAC	GCA	3	C	T
TCGA-04-1652	PDE1C_ENS	ENST00000396193	p.R172W	c.514C>T	Unverified	ACG	CGG	CAG	1	C	T
TCGA-04-1652	PDE1C_ENS	ENST00000396193	p.R172W	c.514C>T	Unverified	ACG	CGG	CAG	1	C	T
TCGA-29-1768	PDE1C_ENS	ENST00000396193	p.H192H	c.576C>T	Unverified	GTT	CAC	GCA	3	C	T
TCGA-24-1844	PDE2A	ENST00000334456	p.R905C	c.2713C>T	Unverified	ATC	CGC	GGC	1	C	T
TCGA-13-0919	PDE4C	ENST00000355502	p.P648P	c.1944C>T	Verified	AAC	CCC	GAG	3	C	T
TCGA-24-1422	PDE6B	ENST00000255622	p.?	c.2269-4_2271de	Unverified	ATT	CCT	ATG	1	C	T
TCGA-29-1695	PDGFRA	ENST00000257290	p.H920H	c.2760C>T	Unverified	GAC	CAC	GCT	3	C	T
TCGA-13-1498	PDHB	ENST00000302746	p.V283V	c.849C>T	Verified	AGT	GTC	ATG	3	C	T
TCGA-23-2077	PDIA3	ENST00000300289	p.A19V	c.56C>T	Unverified	GCG	GCC	CGC	2	C	T
TCGA-04-1343	PDXP	ENST00000215904	p.C221C	c.663C>T	Verified	GAG	TGC	ATC	3	C	T
TCGA-20-1685	PDZD8	ENST00000334464	p.A759V	c.2276C>T	Unverified	GAA	GCC	CCC	2	C	T
TCGA-13-1498	PDZRN3	NM_015009.1	p.T612T	c.1836C>T	Verified	gac	acc	ttg	3	C	T
TCGA-04-1367	PEG3	ENST00000326441	p.R193W	c.577C>T	Verified	CCG	CGG	GAT	1	C	T
TCGA-61-2012	PEPD	ENST00000244137	p.G149G	c.447C>T	Verified	CGT	GGC	GTC	3	C	T
TCGA-23-1111	PER1	ENST00000317276	p.R230*	c.688C>T	Unverified	GGC	CGA	ATC	1	C	T
TCGA-30-1891	PER1	ENST00000317276	p.Q846*	c.2536C>T	Unverified	CAC	CAG	AAC	1	C	T
TCGA-13-1489	PEX16	ENST00000241041	p.P228L	c.683C>T	Verified	CGG	CCG	CTG	2	C	T
TCGA-09-1665	PEX5	ENST00000266564	p.L507L	c.1521C>T	Verified	GCC	CTC	AGC	3	C	T
TCGA-23-1114	PEX5	ENST00000266564	p.T417T	c.1251C>T	Unverified	GAA	ACC	CTA	3	C	T
TCGA-13-0885	PEX6	ENST00000304611	p.P697L	c.2090C>T	Verified	GCC	CCC	AAG	2	C	T
TCGA-04-1356	PFKFB3	ENST00000379775	p.Q228*	c.682C>T	Unverified	GTG	CAG	GAC	1	C	T
TCGA-24-1469	PGBD1	ENST00000259883	p.I695I	c.2085C>T	Verified	ATT	ATC	AGT	3	C	T
TCGA-23-2077	PGBD5	ENST00000321327	p.Q35*	c.103C>T	Verified	CTG	CAG	CCA	1	C	T
TCGA-09-2044	PGLYRP4	ENST00000359650	p.R253C	c.757C>T	Verified	TGC	CGC	CTG	1	C	T
TCGA-13-1497	PGLYRP4	ENST00000359650	p.T74M	c.221C>T	Verified	ACC	ACG	CCA	2	C	T
TCGA-24-1463	PGM2L1	ENST00000298198	p.F352F	c.1056C>T	Verified	GTT	TTC	ACA	3	C	T
TCGA-29-1762	PHACTR1_EI	ENST00000432934	p.L248L	c.742C>T	Unverified	CTA	CTG	CCC	1	C	T
TCGA-20-0991	PHACTR2	ENST00000305766	p.H198Y	c.592C>T	Verified	TCT	CAC	CTG	1	C	T
TCGA-09-1674	PHACTR3	ENST00000371015	p.R396W	c.1186C>T	Unverified	CCA	CGG	AAA	1	C	T
TCGA-25-1326	PHC1	ENST00000251757	p.T320I	c.959C>T	Unverified	CAA	ACA	GTG	2	C	T
TCGA-29-1691	PHF14	NM_001007157.1	p.P180L	c.539C>T	Unverified	gag	cca	aaa	2	C	T
TCGA-24-0979	PHF2	ENST00000359246	p.T312T	c.936C>T	Verified	CAG	ACC	CTC	3	C	T

TCGA-61-1899	PHF20	ENST00000374012	p.P342S	c.1024C>T	Unverified	GAT	CCT	GAC	1	C	T
TCGA-13-1481	PHKA1	ENST00000373542	p.R802W	c.2404C>T	Verified	GAA	CGG	AGT	1	C	T
TCGA-23-1022	PIAS1	ENST00000249636	p.D159D	c.477C>T	Verified	tca	gac	aac	3	C	T
TCGA-24-1849	PIGO	ENST00000378617	p.Q389*	c.1165C>T	Unverified	ctt	caa	gct	1	C	T
TCGA-13-0755	PIH1D2	ENST00000280350	p.T307M	c.920C>T	Verified	TCC	ACG	CTA	2	C	T
TCGA-25-1313	PIK3C2G	ENST00000433979	p.Y1017Y	c.3051C>T	Verified	TTC	TAC	TCC	3	C	T
TCGA-13-0890	PIK3CA	NM_006218.1	p.H510H	c.1530C>T	Verified	tcc	cac	gca	3	C	T
TCGA-24-1844	PIK3R2	ENST00000222254	p.D400D	c.1200C>T	Unverified	GTG	GAC	CTC	3	C	T
TCGA-13-0923	PIKFYVE	ENST00000264380	p.R1984*	c.5950C>T	Verified	GTT	CGA	GAC	1	C	T
TCGA-13-0887	PITPNM3	ENST00000262483	p.R624W	c.1870C>T	Verified	AAG	CGG	ACT	1	C	T
TCGA-61-2012	PKP2	ENST00000070846	p.H213Y	c.637C>T	Verified	CGC	CAC	TTT	1	C	T
TCGA-23-1111	PKP4	ENST00000389759	p.R53*	c.157C>T	Unverified	ACC	CGA	GAA	1	C	T
TCGA-25-2392	PLA2G12B	ENST00000373032	p.G104G	c.312C>T	Verified	TTG	GGC	ATT	3	C	T
TCGA-04-1338	PLA2G15	ENST00000219345	p.Y72Y	c.216C>T	Verified	AGC	TAC	TTC	3	C	T
TCGA-24-2289	PLA2G4C	ENST00000413144	p.Y486Y	c.1458C>T	Unverified	ACA	TAC	GAC	3	C	T
TCGA-61-1733	PLA2R1	ENST00000283243	p.H240H	c.720C>T	Unverified	TCA	CAC	ATT	3	C	T
TCGA-61-1733	PLA2R1	ENST00000283243	p.H240H	c.720C>T	Unverified	TCA	CAC	ATT	3	C	T
TCGA-13-0884	PLAGL2	ENST00000246229	p.A80V	c.239C>T	Verified	ttt	gct	tcc	2	C	T
TCGA-29-1785	PLBD2	ENST00000280800	p.A437V	c.1310C>T	Unverified	AAT	GCC	AGT	2	C	T
TCGA-29-1785	PLBD2	ENST00000280800	p.A437V	c.1310C>T	Unverified	AAT	GCC	AGT	2	C	T
TCGA-61-1738	PLCB3	ENST00000279230	p.H865H	c.2595C>T	Unverified	AAG	CAC	GTC	3	C	T
TCGA-61-1738	PLCB3	ENST00000279230	p.H865H	c.2595C>T	Unverified	AAG	CAC	GTC	3	C	T
TCGA-24-2267	PLCE1	ENST00000260766	p.D257D	c.771C>T	Verified	TGT	GAC	ACC	3	C	T
TCGA-61-1907	PLCH1	ENST00000334686	p.R573*	c.1717C>T	Unverified	CGC	CGA	AGG	1	C	T
TCGA-29-1785	PLCH1	ENST00000334686	p.T1620M	c.4859C>T	Unverified	TGC	ACG	GCT	2	C	T
TCGA-29-1785	PLCH1	ENST00000334686	p.T1620M	c.4859C>T	Unverified	TGC	ACG	GCT	2	C	T
TCGA-61-1907	PLCH1_ENST	ENST00000340059	p.R591*	c.1771C>T	Unverified	CGC	CGA	AGG	1	C	T
TCGA-29-1785	PLCH1_ENST	ENST00000340059	p.T1658M	c.4973C>T	Unverified	TGC	ACG	GCT	2	C	T
TCGA-29-1785	PLCH1_ENST	ENST00000340059	p.T1658M	c.4973C>T	Unverified	TGC	ACG	GCT	2	C	T
TCGA-24-1849	PLCL1	NM_006226.1	p.T248I	c.743C>T	Unverified	gtc	acc	cat	2	C	T
TCGA-24-1849	PLCL1_ENST	ENST00000428675	p.T346I	c.1037C>T	Unverified	GTC	ACC	CAT	2	C	T
TCGA-09-2050	PLD2_ENST(	ENST00000263088	p.D13D	c.39C>T	Verified	GGG	GAC	GAA	3	C	T
TCGA-13-0906	PLD3	ENST00000359274	p.F411F	c.1233C>T	Verified	ATT	TTC	CTG	3	C	T
TCGA-23-1116	PLEC	ENST00000322810	p.P3956S	c.11866C>T	Unverified	CTT	CCC	CTG	1	C	T

TCGA-24-1849	PLEC	ENST00000322810	p.A2242V	c.6725C>T	Unverified	GCG	GCG	CAG	2	C	T
TCGA-24-1849	PLEC_ENST0	ENST00000345136	p.A2105V	c.6314C>T	Unverified	GCG	GCG	CAG	2	C	T
TCGA-24-1849	PLEC_ENST0	ENST00000436759	p.A2132V	c.6395C>T	Unverified	GCG	GCG	CAG	2	C	T
TCGA-24-1616	PLEKHG1	ENST00000358517	p.P150S	c.448C>T	Verified	CTT	CCC	CTG	1	C	T
TCGA-25-2400	PLEKHG4	ENST00000360461	p.L190L	c.568C>T	Unverified	CTG	CTG	GCC	1	C	T
TCGA-25-1313	PLEKHG4	ENST00000360461	p.S11F	c.32C>T	Unverified	GAG	TCC	CCA	2	C	T
TCGA-24-2290	PLOD1	ENST00000196061	p.L311L	c.933C>T	Verified	CGG	CTC	CAC	3	C	T
TCGA-24-2290	PLOD1	ENST00000196061	p.L311L	c.933C>T	Verified	CGG	CTC	CAC	3	C	T
TCGA-29-1761	PLOD3	ENST00000223127	p.Q349*	c.1045C>T	Unverified	CCG	CAG	CTC	1	C	T
TCGA-36-1577	PLVAP	ENST00000252590	p.R107R	c.321C>T	Unverified	CGC	CGC	GAC	3	C	T
TCGA-61-2012	PLXNA4_EN	ENST00000321063	p.T1777I	c.5330C>T	Verified	CAG	ACC	TTC	2	C	T
TCGA-24-1850	PLXNC1	ENST00000258526	p.S691L	c.2072C>T	Unverified	GCA	TCG	AAC	2	C	T
TCGA-24-2288	PMPCA	ENST00000371717	p.P174L	c.521C>T	Unverified	CAG	CCC	CGG	2	C	T
TCGA-13-1488	PNMA1	ENST00000316836	p.A210A	c.630C>T	Verified	CCC	GCC	GCT	3	C	T
TCGA-10-0930	PNPLA6	ENST00000221249	p.D809D	c.2427C>T	Verified	ACG	GAC	GCC	3	C	T
TCGA-10-0930	PNPLA6	ENST00000221249	p.D809D	c.2427C>T	Verified	ACG	GAC	GCC	3	C	T
TCGA-24-1417	POGK	ENST00000367875	p.P542L	c.1625C>T	Verified	AAG	CCA	CCC	2	C	T
TCGA-24-2290	POLE	ENST00000320574	p.V1394V	c.4182C>T	Verified	ATG	GTC	TAC	3	C	T
TCGA-24-2290	POLE	ENST00000320574	p.V1394V	c.4182C>T	Verified	ATG	GTC	TAC	3	C	T
TCGA-23-1124	POLM	ENST00000242248	p.S64S	c.192C>T	Verified	agc	tcc	gaa	3	C	T
TCGA-24-1844	POLR1A	ENST00000263857	p.R1113C	c.3337C>T	Unverified	GGC	CGC	AGC	1	C	T
TCGA-24-2024	POLR1A	ENST00000263857	p.S1677S	c.5031C>T	Verified	ACC	AGC	TTC	3	C	T
TCGA-30-1718	POLR2A_EN	ENST00000412468	p.V112V	c.336C>T	Unverified	AAC	GTC	CTC	3	C	T
TCGA-29-1783	POLR3B	ENST00000228347	p.P1006P	c.3018C>T	Unverified	GGC	CCC	GTG	3	C	T
TCGA-24-1474	POP5	ENST00000357500	p.P18L	c.53C>T	Unverified	GAC	CCC	CGC	2	C	T
TCGA-20-0991	POSTN	ENST00000379747	p.N445N	c.1335C>T	Verified	TAC	AAC	GGG	3	C	T
TCGA-13-1488	POTEF	ENST00000409914	p.Q759*	c.2275C>T	Unverified	GCC	CAG	AGC	1	C	T
TCGA-24-2035	POU2F2	ENST00000389341	p.R280W	c.838C>T	Verified	ggc	cgg	aga	1	C	T
TCGA-04-1638	PPAN-P2RY1	ENST00000393796	p.R523C	c.1567C>T	Unverified	TGC	CGC	CTG	1	C	T
TCGA-29-1693	PPAN-P2RY1	ENST00000393796	p.A733A	c.2199C>T	Unverified	CTG	GCC	TTC	3	C	T
TCGA-13-0795	PPARD	ENST00000360694	p.H287Y	c.859C>T	Verified	gtg	cac	gag	1	C	T
TCGA-04-1652	PPARGC1A	ENST00000264867	p.S359S	c.1077C>T	Unverified	ctc	agc	aag	3	C	T
TCGA-04-1652	PPARGC1A	ENST00000264867	p.S359S	c.1077C>T	Unverified	ctc	agc	aag	3	C	T
TCGA-61-2012	PPARGC1B	ENST00000309241	p.D120D	c.360C>T	Unverified	GGA	GAC	GCT	3	C	T

TCGA-23-1123	PPFIBP2	ENST00000299492	p.D449D	c.1347C>T	Verified	cct	gac	gcc	3	C	T
TCGA-23-1123	PPFIBP2	ENST00000299492	p.D449D	c.1347C>T	Verified	cct	gac	gcc	3	C	T
TCGA-13-0791	PPIL1	ENST00000373699	p.A3V	c.8C>T	Verified	GCG	GCA	ATT	2	C	T
TCGA-61-2009	PPOX	ENST00000352210	p.N476N	c.1428C>T	Verified	CCT	AAC	AGC	3	C	T
TCGA-04-1649	PPP1R21	ENST00000294952	p.I550I	c.1650C>T	Unverified	CGC	ATC	CTT	3	C	T
TCGA-61-1910	PPP1R7	ENST00000407025	p.N218N	c.654C>T	Unverified	aaa	aac	aaa	3	C	T
TCGA-20-1683	PPP2R2B	ENST00000336640	p.R329C	c.985C>T	Unverified	ctc	cgc	agc	1	C	T
TCGA-20-1683	PPP2R2B_EN	ENST00000394409	p.R384C	c.1150C>T	Unverified	CTC	CGC	AGC	1	C	T
TCGA-20-1683	PPP2R2B_EN	ENST00000394413	p.R326C	c.976C>T	Unverified	CTC	CGC	AGC	1	C	T
TCGA-20-1683	PPP2R2B_EN	ENST00000508545	p.R315C	c.943C>T	Unverified	CTC	CGC	AGC	1	C	T
TCGA-23-1124	PPP3CC	ENST00000240139	p.P305S	c.913C>T	Verified	gcc	ccc	aat	1	C	T
TCGA-25-1313	PPP6C	ENST00000373547	p.L149L	c.447C>T	Verified	atg	ctc	aca	3	C	T
TCGA-24-1431	PPP6R2	ENST00000359139	p.P420L	c.1259C>T	Unverified	CCT	CCG	CAT	2	C	T
TCGA-61-1738	PQLC1	ENST00000397778	p.T181T	c.543C>T	Unverified	CTG	ACC	GAA	3	C	T
TCGA-61-1738	PQLC1	ENST00000397778	p.T181T	c.543C>T	Unverified	CTG	ACC	GAA	3	C	T
TCGA-61-1737	PRAMEF1	ENST00000332296	p.H324Y	c.970C>T	Unverified	AAG	CAT	CTG	1	C	T
TCGA-25-2042	PRDM16	ENST00000270722	p.G651G	c.1953C>T	Verified	GGG	GGC	GGC	3	C	T
TCGA-29-1762	PRDM9	ENST00000296682	p.R619W	c.1855C>T	Unverified	AGC	CGG	CAG	1	C	T
TCGA-23-1122	PRELP	ENST00000343110	p.A168V	c.503C>T	Verified	TCG	GCC	CTG	2	C	T
TCGA-04-1343	PREP	ENST00000369110	p.P482P	c.1446C>T	Verified	ACA	CCC	AAC	3	C	T
TCGA-20-1686	PREX1	ENST00000371941	p.A222V	c.665C>T	Unverified	CCC	GCG	GTC	2	C	T
TCGA-20-1686	PREX1_ENST	ENST00000396220	p.A222V	c.665C>T	Unverified	CCC	GCG	GTC	2	C	T
TCGA-04-1638	PRIC285	ENST00000467148	p.D1028D	c.3084C>T	Unverified	GGA	GAC	GCA	3	C	T
TCGA-24-1844	PRKCA	ENST00000413366	p.R632*	c.1894C>T	Unverified	ACA	CGA	GGA	1	C	T
TCGA-24-1844	PRKCB	ENST00000303531	p.L575L	c.1723C>T	Unverified	GGG	CTG	ATG	1	C	T
TCGA-24-1844	PRKCB_ENST	ENST00000303531	p.L575L	c.1723C>T	Unverified	GGG	CTG	ATG	1	C	T
TCGA-24-1844	PRKCB_ENST	ENST00000321728	p.L575L	c.1723C>T	Unverified	GGG	CTG	ATG	1	C	T
TCGA-04-1638	PRKCE	ENST00000306156	p.V208V	c.624C>T	Unverified	GTG	GTC	CAC	3	C	T
TCGA-23-1031	PRKCSH	ENST00000252455	p.L25L	c.75C>T	Unverified	TCC	CTC	ACC	3	C	T
TCGA-36-1577	PRKD1	ENST00000331968	p.P270L	c.809C>T	Verified	GTG	CCG	CAC	2	C	T
TCGA-36-1577	PRKD1_ENST	ENST00000331968	p.P270L	c.809C>T	Verified	GTG	CCG	CAC	2	C	T
TCGA-24-1604	PRMT2	ENST00000397637	p.G74G	c.222C>T	Verified	GCG	GGC	TGC	3	C	T
TCGA-13-1488	PRODH2	ENST00000301175	p.R525W	c.1573C>T	Verified	TGG	CGG	CGG	1	C	T
TCGA-61-1740	PROKR1	ENST00000303786	p.I305I	c.915C>T	Unverified	ACC	ATC	GTG	3	C	T

TCGA-25-1313	PROKR2	ENST00000217270	p.R85C	c.253C>T	Verified	TTG	CGC	AAC	1	C	T
TCGA-30-1891	PROKR2	ENST00000217270	p.S98S	c.294C>T	Unverified	ATC	TCC	GAC	3	C	T
TCGA-13-1509	PRPF31	ENST00000321030	p.I279I	c.837C>T	Verified	GAC	ATC	GTG	3	C	T
TCGA-04-1542	PRPF4B_ENS	ENST00000337659	p.R183*	c.547C>T	Verified	AAA	CGA	AGT	1	C	T
TCGA-04-1542	PRPS1L1_EN	ENST00000506618	p.R49C	c.145C>T	Verified	ATG	CTC	TCT	1	C	T
TCGA-29-1783	PRR21	ENST00000408934	p.R53W	c.157C>T	Unverified	CCA	CGG	CTC	1	C	T
TCGA-09-1674	PRR5-ARHG.	NM_181334.1	p.P149L	c.446C>T	Unverified	cac	ccc	acc	2	C	T
TCGA-61-1910	PRRC2A	ENST00000376033	p.R904C	c.2710C>T	Unverified	GCC	CGC	GGA	1	C	T
TCGA-13-0913	PRRC2A	ENST00000376033	p.R1831R	c.5493C>T	Verified	CAG	CGC	CTG	3	C	T
TCGA-30-1718	PRSS23	ENST00000280258	p.T99M	c.296C>T	Unverified	GAG	ACG	CAG	2	C	T
TCGA-13-0762	PRSS3	ENST00000379405	p.S200F	c.599C>T	Unverified	GAC	TCT	GGT	2	C	T
TCGA-13-0762	PRSS3	ENST00000379405	p.S200F	c.599C>T	Unverified	GAC	TCT	GGT	2	C	T
TCGA-04-1361	PSAP	ENST00000394936	p.P294S	c.880C>T	Verified	ATC	CCT	GCC	1	C	T
TCGA-04-1361	PSAP	ENST00000394936	p.P294S	c.880C>T	Verified	ATC	CCT	GCC	1	C	T
TCGA-13-1496	PSD4	ENST00000245796	p.R594C	c.1780C>T	Verified	TCA	CGC	CTC	1	C	T
TCGA-29-1763	PSD4	ENST00000245796	p.D82D	c.246C>T	Unverified	CAG	GAC	GGG	3	C	T
TCGA-09-0369	PSENEN	ENST00000222266	p.R82W	c.244C>T	Verified	tac	cgg	ccc	1	C	T
TCGA-09-0369	PSENEN	ENST00000222266	p.R82W	c.244C>T	Verified	tac	cgg	ccc	1	C	T
TCGA-24-1844	PSG3	ENST00000327495	p.L4F	c.10C>T	Unverified	CCC	CTC	TCA	1	C	T
TCGA-23-1120	PSMB6	ENST00000270586	p.L210L	c.628C>T	Verified	CGC	CTG	GCA	1	C	T
TCGA-13-0760	PSMC3	ENST00000298852	p.Q184*	c.550C>T	Verified	GAG	CAA	TAC	1	C	T
TCGA-24-2289	PSMC5	ENST00000310144	p.A238V	c.713C>T	Unverified	ATG	GCA	CGG	2	C	T
TCGA-30-1718	PSME4	NM_014614.1	p.S861F	c.2582C>T	Unverified	tta	tct	aca	2	C	T
TCGA-30-1718	PSME4_ENS	ENST00000404125	p.S975F	c.2924C>T	Unverified	TTA	TCT	ACA	2	C	T
TCGA-61-1915	PSMF1	ENST00000335877	p.R180W	c.538C>T	Unverified	AGT	CGG	CAG	1	C	T
TCGA-25-2400	PSPN	ENST00000245810	p.A32A	c.96C>T	Unverified	GTG	GCC	GAT	3	C	T
TCGA-20-1683	PTBP2	ENST00000426398	p.P57S	c.169C>T	Unverified	GCT	CCT	TCT	1	C	T
TCGA-29-1781	PTCH1	ENST00000331920	p.P1441S	c.4321C>T	Unverified	AGG	CCC	CGG	1	C	T
TCGA-29-1781	PTCH1_ENS	ENST00000331920	p.P1441S	c.4321C>T	Unverified	AGG	CCC	CGG	1	C	T
TCGA-29-1781	PTCH1_ENS	ENST00000375274	p.P1440S	c.4318C>T	Unverified	AGG	CCC	CGG	1	C	T
TCGA-29-1781	PTCH1_ENS	ENST00000375274	p.P1440S	c.4318C>T	Unverified	AGG	CCC	CGG	1	C	T
TCGA-23-2078	PTCHD2	XM_052561.7	p.R335C	c.1003C>T	Verified	cag	cgt	ttc	1	C	T
TCGA-24-1843	PTCHD2	XM_052561.7	p.R631C	c.1891C>T	Unverified	gac	cgc	tgg	1	C	T
TCGA-61-1740	PTEN	ENST00000371953	p.H123Y	c.367C>T	Unverified	att	cac	tgt	1	C	T

TCGA-25-2392	PTGFR	ENST00000370756	p.A253V	c.758C>T	Verified	CTG	GCG	ATA	2	C	T
TCGA-25-2392	PTGFR_ENST	ENST00000370758	p.A253V	c.758C>T	Verified	CTG	GCG	ATA	2	C	T
TCGA-29-2427	PTGIR	ENST00000291294	p.F146F	c.438C>T	Unverified	GCC	TTC	TGC	3	C	T
TCGA-29-1698	PTGS1_ENST	ENST00000362012	p.P582L	c.1745C>T	Unverified	GTG	CCG	GAT	2	C	T
TCGA-24-2035	PTK2	NM_005607	p.R448*	c.1342C>T	Verified	gga	cga	tgt	1	C	T
TCGA-61-1907	PTK6	ENST00000217185	p.Y251Y	c.753C>T	Unverified	ctg	tac	gcc	3	C	T
TCGA-23-1022	PTPN13	ENST00000436978	p.T673I	c.2018C>T	Verified	CAT	ACT	CTG	2	C	T
TCGA-13-0807	PTPN18	ENST00000175756	p.R331C	c.991C>T	Verified	ccc	cgc	cca	1	C	T
TCGA-61-1915	PTPN23	ENST00000265562	p.F510F	c.1530C>T	Unverified	TCC	TTC	ACC	3	C	T
TCGA-13-1499	PTPN3	ENST00000394831	p.R371C	c.1111C>T	Verified	TCT	CGT	TCC	1	C	T
TCGA-24-2280	PTPRD	ENST00000381196	p.P974S	c.2920C>T	Unverified	GTT	CCA	GCT	1	C	T
TCGA-25-1326	PTPRG	ENST00000474889	p.P570L	c.1709C>T	Unverified	TCA	CCA	ACC	2	C	T
TCGA-04-1655	PTPRN	ENST00000295718	p.V443V	c.1329C>T	Unverified	cct	gtc	ctg	3	C	T
TCGA-24-1849	PTPRN2	NM_002847.2	p.A617V	c.1850C>T	Unverified	atc	gcg	ctc	2	C	T
TCGA-61-1899	PTPRN2	NM_002847.2	p.A467A	c.1401C>T	Unverified	ggg	gcc	gct	3	C	T
TCGA-24-2280	PTPRS	ENST00000357368	p.R188*	c.562C>T	Unverified	CTG	CGA	TCA	1	C	T
TCGA-13-1507	PTPRT	NM_133170.2	p.A149V	c.446C>T	Verified	aag	gca	gag	2	C	T
TCGA-61-2113	PURA	ENST00000331327	p.L159F	c.475C>T	Unverified	GAT	CTC	AAG	1	C	T
TCGA-29-1766	PWP2	ENST00000291576	p.T791I	c.2372C>T	Unverified	GTC	ACC	TCC	2	C	T
TCGA-61-2094	PXDN	ENST00000252804	p.Y118Y	c.354C>T	Unverified	CTG	TAC	AAG	3	C	T
TCGA-61-1910	PYGL	ENST00000216392	p.D51D	c.153C>T	Unverified	CGC	GAC	TAC	3	C	T
TCGA-20-1686	PYGM	ENST00000164139	p.R570W	c.1708C>T	Unverified	AAG	CGG	ATT	1	C	T
TCGA-09-0369	PZP	ENST00000261336	p.T89I	c.266C>T	Verified	TTC	ACT	CTC	2	C	T
TCGA-09-0369	PZP	ENST00000261336	p.T89I	c.266C>T	Verified	TTC	ACT	CTC	2	C	T
TCGA-04-1362	PZP	ENST00000261336	p.V130V	c.390C>T	Verified	TTT	GTC	CAG	3	C	T
TCGA-29-1784	Q5TBE2_HU	ENST00000315260	p.Q79*	c.235C>T	Unverified	ATT	CAA	GAT	1	C	T
TCGA-61-2102	Q6YL47_HU	ENST00000322367	p.N1004N	c.3012C>T	Verified	ATC	AAC	CTC	3	C	T
TCGA-23-1111	Q7Z5Z2_HU	ENST00000340697	p.S20L	c.59C>T	Unverified	TCT	TCG	ACA	2	C	T
TCGA-13-0887	Q8N0W1_H	ENST00000330155	p.P165L	c.494C>T	Verified	CTG	CCC	TTC	2	C	T
TCGA-29-1691	Q8NFX8_HU	ENST00000330652	p.P100L	c.299C>T	Unverified	TTG	CCC	ACA	2	C	T
TCGA-10-0930	Q8NGM6_H	ENST00000316506	p.H99H	c.297C>T	Verified	GTC	CAC	GCT	3	C	T
TCGA-10-0930	Q8NGM6_H	ENST00000316506	p.H99H	c.297C>T	Verified	GTC	CAC	GCT	3	C	T
TCGA-24-1847	Q8NH77_HU	ENST00000316517	p.F115F	c.345C>T	Unverified	ATC	TTC	CTC	3	C	T
TCGA-23-2078	RAB11FIP1	ENST00000330843	p.V897V	c.2691C>T	Verified	GAA	GTC	CCC	3	C	T

TCGA-29-1770	RAB11FIP5	ENST00000258098	p.P433L	c.1298C>T	Unverified	AAG	CCA	GTC	2	C	T
TCGA-04-1342	RAB28	ENST00000288723	p.R146W	c.436C>T	Verified	TTA	CGG	TTT	1	C	T
TCGA-24-2267	RAB3D	ENST00000222120	p.D156D	c.468C>T	Verified	gac	gac	ctt	3	C	T
TCGA-24-1463	RAB3GAP1	ENST00000264158	p.L963L	c.2889C>T	Verified	GTT	CTC	ACC	3	C	T
TCGA-20-1683	RAB3IL1	ENST00000394836	p.R316W	c.946C>T	Unverified	ATC	CGG	CTC	1	C	T
TCGA-13-0795	RAB40AL	ENST00000218249	p.T70M	c.209C>T	Verified	GAT	ACG	TCG	2	C	T
TCGA-61-1915	RAB40AL	ENST00000218249	p.Y11Y	c.33C>T	Unverified	GCC	TAC	GAC	3	C	T
TCGA-29-1761	RAB4B	NM_016154.2	p.A171A	c.513C>T	Unverified	gag	gcc	tcc	3	C	T
TCGA-24-1431	RAB9A	ENST00000464506	p.A138V	c.413C>T	Verified	gaa	gcc	caa	2	C	T
TCGA-23-1110	RABEP2	ENST00000358201	p.R322W	c.964C>T	Verified	AGA	CGG	AGC	1	C	T
TCGA-13-0885	RABGAP1	NM_012197.2	p.L144L	c.432C>T	Verified	atc	ctc	ttc	3	C	T
TCGA-24-1843	RABIF	ENST00000367262	p.L65L	c.195C>T	Unverified	ctc	ctc	cag	3	C	T
TCGA-20-1685	RAD51AP2	ENST00000399080	p.P1122L	c.3365C>T	Unverified	CGA	CCG	CTT	2	C	T
TCGA-29-1691	RAD54L	ENST00000442598	p.P269S	c.805C>T	Unverified	TCT	CCC	ATC	1	C	T
TCGA-24-1847	RALGAPA2	ENST00000202677	p.A1650V	c.4949C>T	Unverified	ATC	GCA	GTG	2	C	T
TCGA-24-1847	RALGAPA2_	ENST00000202677	p.A1650V	c.4949C>T	Unverified	ATC	GCA	GTG	2	C	T
TCGA-61-1914	RAMP3	ENST00000242249	p.P114L	c.341C>T	Unverified	GAC	CCC	CCA	2	C	T
TCGA-04-1638	RANBP2	ENST00000283195	p.P512L	c.1535C>T	Unverified	CAG	CCG	TTA	2	C	T
TCGA-04-1638	RANBP2_EN	ENST00000283195	p.P512L	c.1535C>T	Unverified	CAG	CCG	TTA	2	C	T
TCGA-24-2280	RAPGEF2	ENST00000264431	p.T923I	c.2768C>T	Unverified	AGG	ACT	CGG	2	C	T
TCGA-24-1850	RAPGEFL1	ENST00000264644	p.R92C	c.274C>T	Unverified	ttc	cgc	cgg	1	C	T
TCGA-23-1122	RAPSN	ENST00000298854	p.L247L	c.741C>T	Unverified	GCG	CTC	TGC	3	C	T
TCGA-29-1785	RARRES3	ENST00000255688	p.I92I	c.276C>T	Unverified	GTG	ATC	ATC	3	C	T
TCGA-29-1785	RARRES3	ENST00000255688	p.I92I	c.276C>T	Unverified	GTG	ATC	ATC	3	C	T
TCGA-24-1845	RASGRF1	ENST00000419573	p.L182F	c.544C>T	Unverified	ctg	ctc	aag	1	C	T
TCGA-24-2280	RASGRF1	ENST00000419573	p.P877S	c.2629C>T	Unverified	ttc	cca	ctc	1	C	T
TCGA-30-1856	RASGRF1	ENST00000419573	p.R161W	c.481C>T	Unverified	ctt	cgg	cag	1	C	T
TCGA-24-1563	RASGRP1	ENST00000310803	p.S314L	c.941C>T	Verified	AGT	TCG	CAT	2	C	T
TCGA-61-2095	RASL11B	ENST00000248706	p.P211L	c.632C>T	Unverified	ACA	CCC	GAG	2	C	T
TCGA-29-1761	RASSF2	ENST00000379400	p.R245*	c.733C>T	Unverified	gcc	cga	atc	1	C	T
TCGA-29-1691	RB1	ENST00000267163	p.Q575*	c.1723C>T	Unverified	AAA	CAA	TCA	1	C	T
TCGA-09-1665	RB1	ENST00000267163	p.A264V	c.791C>T	Verified	ATA	GCA	AAA	2	C	T
TCGA-29-1691	RB1_ENST0(	ENST00000267163	p.Q575*	c.1723C>T	Unverified	AAA	CAA	TCA	1	C	T
TCGA-04-1530	RBBP6	ENST00000319715	p.P602S	c.1804C>T	Unverified	cct	cca	ggg	1	C	T

TCGA-61-1733	RBBP6	ENST00000319715	p.N1394N	c.4182C>T	Unverified	aaa	aac	tct	3	C	T
TCGA-61-1733	RBBP6	ENST00000319715	p.N1394N	c.4182C>T	Unverified	aaa	aac	tct	3	C	T
TCGA-25-2398	RBL2	ENST00000262133	p.T742M	c.2225C>T	Unverified	CAA	ACG	GTA	2	C	T
TCGA-24-2289	RBM22	ENST00000199814	p.Q13*	c.37C>T	Unverified	agg	cag	aac	1	C	T
TCGA-24-2262	RBM41	ENST00000372479	p.G351G	c.1053C>T	Verified	AGG	GGC	CAG	3	C	T
TCGA-20-0991	RBM45	ENST00000286070	p.I146I	c.438C>T	Unverified	GAT	ATC	GAG	3	C	T
TCGA-24-1845	RC3H2	ENST00000373670	p.L321L	c.961C>T	Unverified	AAG	CTA	CAG	1	C	T
TCGA-24-1845	RC3H2_ENST	ENST00000335387	p.L321L	c.961C>T	Unverified	AAG	CTA	CAG	1	C	T
TCGA-61-2113	RCBTB2	ENST00000344532	p.H317Y	c.949C>T	Verified	tgt	cac	tcc	1	C	T
TCGA-09-1665	RCBTB2	ENST00000344532	p.V228V	c.684C>T	Verified	tat	gtc	tgg	3	C	T
TCGA-13-0887	RCN3	ENST00000270645	p.I106I	c.318C>T	Verified	TGG	ATC	GCG	3	C	T
TCGA-61-1740	RCOR2	ENST00000301459	p.P217S	c.649C>T	Unverified	GAG	CCC	GAT	1	C	T
TCGA-61-1895	RECK	ENST00000377966	p.Q645*	c.1933C>T	Unverified	GGG	CAG	AAT	1	C	T
TCGA-13-1498	REG4	ENST00000354219	p.R157*	c.469C>T	Verified	TAC	CGA	CCA	1	C	T
TCGA-25-1313	RET	ENST00000355710	p.N437N	c.1311C>T	Unverified	atc	aac	gtc	3	C	T
TCGA-23-1110	RFTN1	ENST00000334133	p.R448*	c.1342C>T	Verified	TGG	CGA	TTC	1	C	T
TCGA-29-1783	RFTN2	ENST00000295049	p.Y36Y	c.108C>T	Unverified	GCT	TAC	GAA	3	C	T
TCGA-29-1770	RFX3_ENST(	ENST00000382004	p.L720L	c.2160C>T	Unverified	AGC	CTC	CTG	3	C	T
TCGA-61-1910	RFX5	ENST00000368870	p.A371A	c.1113C>T	Unverified	AGG	GCC	GGG	3	C	T
TCGA-29-1781	RFX6	ENST00000332958	p.L306L	c.918C>T	Unverified	CTG	CTC	GAA	3	C	T
TCGA-09-2049	RGAG4	XM_291322.3	p.S185S	c.555C>T	Verified	cgc	agc	acc	3	C	T
TCGA-13-0903	RGPD3	ENST00000409886	p.S496S	c.1488C>T	Verified	ACC	AGC	CAC	3	C	T
TCGA-61-1914	RGR	ENST00000359452	p.F138F	c.414C>T	Unverified	CTC	TTC	GTG	3	C	T
TCGA-29-1763	RGS16	ENST00000367558	p.A187A	c.561C>T	Unverified	GCC	GCC	TCT	3	C	T
TCGA-24-1435	RGS22	ENST00000360863	p.T12I	c.35C>T	Verified	CCA	ACT	ATT	2	C	T
TCGA-23-1031	RGS3	NM_144488.2	p.I26I	c.78C>T	Verified	agg	atc	acg	3	C	T
TCGA-23-1032	RGS4	ENST00000367909	p.R166C	c.496C>T	Verified	tac	cgc	cgc	1	C	T
TCGA-04-1652	RGS7BP	ENST00000334025	p.S40S	c.120C>T	Unverified	GGC	TCC	GAG	3	C	T
TCGA-04-1652	RGS7BP	ENST00000334025	p.S40S	c.120C>T	Unverified	GGC	TCC	GAG	3	C	T
TCGA-13-0760	RHBDF1	ENST00000262316	p.Y654Y	c.1962C>T	Verified	TTC	TAC	CGC	3	C	T
TCGA-13-0923	RHPN2	ENST00000254260	p.S548L	c.1643C>T	Verified	GCC	TCG	GTG	2	C	T
TCGA-09-2049	RICTOR	ENST00000357387	p.S1373F	c.4118C>T	Verified	GAG	TCC	AGA	2	C	T
TCGA-13-1505	RILP	ENST00000301336	p.A397A	c.1191C>T	Verified	TCA	GCC	GCC	3	C	T
TCGA-04-1649	RIMBP2	ENST00000261655	p.P320L	c.959C>T	Unverified	CCC	CCG	GCG	2	C	T

TCGA-61-1904	RIMKLA	ENST00000372570	p.R318W	c.952C>T	Unverified	ATC	CGG	GAT	1	C	T
TCGA-61-1998	RIMS1	ENST00000521978	p.L1140L	c.3418C>T	Unverified	TCC	CTA	GAT	1	C	T
TCGA-13-1497	RIMS4	ENST00000372851	p.R36W	c.106C>T	Verified	AGC	CGG	AGG	1	C	T
TCGA-23-1022	RIMS4	ENST00000372851	p.L223L	c.667C>T	Verified	GAG	CTG	GAC	1	C	T
TCGA-23-2077	RIMS4	ENST00000372851	p.R108C	c.322C>T	Unverified	GGC	CGC	CAG	1	C	T
TCGA-29-1763	RIN2	NM_018993.2	p.Q820*	c.2458C>T	Unverified	tgt	cag	atc	1	C	T
TCGA-29-1763	RIN2_ENST0	ENST00000255006	p.Q869*	c.2605C>T	Unverified	TGT	CAG	ATC	1	C	T
TCGA-24-2035	RLIM	ENST00000332687	p.S501L	c.1502C>T	Verified	agc	tca	tca	2	C	T
TCGA-23-1029	RLN1	ENST00000223862	p.R41R	c.123C>T	Unverified	GTT	CGC	GCG	3	C	T
TCGA-09-2044	RMI2	ENST00000312499	p.L125F	c.373C>T	Verified	GAC	CTT	TCT	1	C	T
TCGA-24-1470	RNASEH1	ENST00000315212	p.Y163Y	c.489C>T	Verified	GTT	TAC	TGG	3	C	T
TCGA-29-1702	RNF111	ENST00000348370	p.R949C	c.2845C>T	Unverified	AGA	CGT	CTT	1	C	T
TCGA-29-1702	RNF111_EN:	ENST00000434298	p.R966C	c.2896C>T	Unverified	AGA	CGT	CTT	1	C	T
TCGA-04-1347	RNF114	ENST00000244061	p.R76*	c.226C>T	Verified	GTC	CGA	GCC	1	C	T
TCGA-20-0991	RNF150	XM_371709.3	p.I75I	c.225C>T	Verified	gac	atc	gtg	3	C	T
TCGA-04-1338	RNF25	ENST00000295704	p.P305L	c.914C>T	Verified	ttg	cca	cct	2	C	T
TCGA-24-1843	ROBO1	ENST00000464233	p.P303S	c.907C>T	Unverified	CTG	CCC	AAA	1	C	T
TCGA-24-1850	ROBO1	ENST00000464233	p.R1420*	c.4258C>T	Unverified	GCA	CGA	CGG	1	C	T
TCGA-24-1843	ROBO1_ENS	ENST00000464233	p.P303S	c.907C>T	Unverified	CTG	CCC	AAA	1	C	T
TCGA-24-1850	ROBO1_ENS	ENST00000464233	p.R1420*	c.4258C>T	Unverified	GCA	CGA	CGG	1	C	T
TCGA-24-1843	ROBO1_ENS	ENST00000464233	p.P303S	c.907C>T	Unverified	CTG	CCC	AAA	1	C	T
TCGA-24-1850	ROBO1_ENS	ENST00000464233	p.R1420*	c.4258C>T	Unverified	GCA	CGA	CGG	1	C	T
TCGA-24-1843	ROBO1_ENS	ENST00000495273	p.P264S	c.790C>T	Unverified	CTG	CCC	AAA	1	C	T
TCGA-24-1850	ROBO1_ENS	ENST00000495273	p.R1375*	c.4123C>T	Unverified	GCA	CGA	CGG	1	C	T
TCGA-24-1844	ROBO2	ENST00000461745	p.P551L	c.1652C>T	Unverified	CTT	CCA	GCA	2	C	T
TCGA-24-1844	ROBO2_ENS	ENST00000487694	p.P567L	c.1700C>T	Unverified	CTT	CCA	GCA	2	C	T
TCGA-29-1777	ROCK2	ENST00000315872	p.A612V	c.1835C>T	Unverified	ACT	GCC	AAG	2	C	T
TCGA-29-1777	ROCK2_ENS1	ENST00000315872	p.A612V	c.1835C>T	Unverified	ACT	GCC	AAG	2	C	T
TCGA-13-0884	ROR2	ENST00000375708	p.P516L	c.1547C>T	Verified	ggg	ccc	ctg	2	C	T
TCGA-09-1665	ROS1	ENST00000368508	p.Q177*	c.529C>T	Unverified	CTG	CAG	CTC	1	C	T
TCGA-24-2262	RP1L1	ENST00000382483	p.R1622W	c.4864C>T	Unverified	GAG	CGG	ACC	1	C	T
TCGA-61-1910	RP2	ENST00000218340	p.D287D	c.861C>T	Unverified	CCT	GAC	TTC	3	C	T
TCGA-61-1733	RPL22	ENST00000234875	p.R97C	c.289C>T	Unverified	CTA	CGT	GAC	1	C	T
TCGA-61-1733	RPL22	ENST00000234875	p.R97C	c.289C>T	Unverified	CTA	CGT	GAC	1	C	T

TCGA-29-1698	RPL22L1	ENST00000295830	p.P3S	c.7C>T	Unverified	GCG	CCG	CAG	1	C	T
TCGA-23-1117	RPN2	ENST00000237530	p.V567V	c.1701C>T	Verified	AAT	GTC	TCC	3	C	T
TCGA-23-1117	RPN2	ENST00000237530	p.V567V	c.1701C>T	Verified	AAT	GTC	TCC	3	C	T
TCGA-30-1856	RPS6KC1	ENST00000366960	p.A743V	c.2228C>T	Unverified	caa	gca	cat	2	C	T
TCGA-61-2102	RPS8	ENST00000396651	p.P120P	c.360C>T	Verified	CTG	CCC	CTG	3	C	T
TCGA-13-1510	RRBP1	ENST00000360807	p.D5D	c.15C>T	Unverified	TAC	GAC	ACT	3	C	T
TCGA-10-0930	RREB1	ENST00000379933	p.S1140F	c.3419C>T	Verified	GCC	TCT	CCC	2	C	T
TCGA-10-0930	RREB1	ENST00000379933	p.S1140F	c.3419C>T	Verified	GCC	TCT	CCC	2	C	T
TCGA-20-1683	RREB1	ENST00000379933	p.A684A	c.2052C>T	Unverified	AAG	GCC	GCG	3	C	T
TCGA-20-1683	RREB1_ENS	ENST00000379938	p.A684A	c.2052C>T	Unverified	AAG	GCC	GCG	3	C	T
TCGA-13-0884	RRP12	ENST00000370992	p.T436M	c.1307C>T	Verified	GCT	ACG	CAG	2	C	T
TCGA-13-0886	RSAD2	ENST00000382040	p.P320L	c.959C>T	Unverified	GAC	CCT	TCC	2	C	T
TCGA-23-1114	RSBN1L	ENST00000334955	p.S832L	c.2495C>T	Unverified	AGT	TCA	GCA	2	C	T
TCGA-25-2401	RSF1	ENST00000308488	p.L126L	c.378C>T	Verified	TAC	CTC	TGT	3	C	T
TCGA-61-2102	RSL24D1	ENST00000260443	p.N130N	c.390C>T	Verified	CAA	AAC	ATC	3	C	T
TCGA-04-1638	RSPH6A	ENST00000221538	p.P410L	c.1229C>T	Unverified	AAG	CCG	CCG	2	C	T
TCGA-24-1474	RTP1	ENST00000312295	p.A220V	c.659C>T	Verified	CGG	GCG	CCC	2	C	T
TCGA-30-1891	RUNDC3A	ENST00000426726	p.R147C	c.439C>T	Unverified	CTG	CGT	GAC	1	C	T
TCGA-04-1655	RUNX1T1	ENST00000360348	p.R496*	c.1486C>T	Unverified	GCC	CGA	TAC	1	C	T
TCGA-04-1655	RUNX1T1_E	ENST00000265814	p.R533*	c.1597C>T	Unverified	GCC	CGA	TAC	1	C	T
TCGA-04-1655	RUNX1T1_E	ENST00000436581	p.R544*	c.1630C>T	Unverified	GCC	CGA	TAC	1	C	T
TCGA-61-1740	RUVBL2_EN	ENST00000221413	p.S310F	c.929C>T	Unverified	TTC	TCC	TTC	2	C	T
TCGA-23-1124	RXRB	ENST00000374680	p.R252C	c.754C>T	Verified	aag	cgc	cag	1	C	T
TCGA-23-1022	RXRB	ENST00000374680	p.S410L	c.1229C>T	Verified	cat	tca	gca	2	C	T
TCGA-61-2012	RYK	NM_002958	p.R340C	c.1018C>T	Verified	ggg	cgt	att	1	C	T
TCGA-04-1655	RYR1	ENST00000359596	p.G3299G	c.9897C>T	Unverified	GCC	GGC	GCC	3	C	T
TCGA-23-1021	RYR1	ENST00000359596	p.I4902I	c.14706C>T	Verified	GAG	ATC	GAG	3	C	T
TCGA-25-2401	RYR2	ENST00000366574	p.A2498V	c.7493C>T	Verified	CGG	GCG	GCT	2	C	T
TCGA-23-1031	RYR2	ENST00000366574	p.I4094I	c.12282C>T	Verified	GAC	ATC	GGC	3	C	T
TCGA-61-1998	RYR3	ENST00000389232	p.I666I	c.1998C>T	Verified	ATT	ATC	GAC	3	C	T
TCGA-24-1469	SACS	NM_014363.3	p.Q1481*	c.4441C>T	Verified	tgt	cag	tta	1	C	T
TCGA-13-0904	SAMD3	ENST00000368134	p.A277V	c.830C>T	Verified	GAA	GCT	GTT	2	C	T
TCGA-13-1497	SAMSN1	ENST00000400566	p.L296L	c.886C>T	Verified	TTA	CTA	TCA	1	C	T
TCGA-29-1766	SAP130	ENST00000259235	p.T410T	c.1230C>T	Unverified	GTT	ACC	ATG	3	C	T

TCGA-29-1766	SAP130_ENS	ENST00000357702	p.T410T	c.1230C>T	Unverified	GTT	ACC	ATG	3	C	T
TCGA-61-1998	SAP30BP	ENST00000584667	p.Y151Y	c.453C>T	Verified	AAC	TAC	ATT	3	C	T
TCGA-61-1907	SASH1	ENST00000367467	p.A879V	c.2636C>T	Unverified	AAG	GCC	CAG	2	C	T
TCGA-04-1542	SASS6	ENST00000287482	p.A378V	c.1133C>T	Verified	TCT	GCA	GAA	2	C	T
TCGA-29-1691	SATB2	ENST00000417098	p.P606L	c.1817C>T	Unverified	CCT	CCG	ACT	2	C	T
TCGA-61-1910	SATB2	ENST00000417098	p.S707S	c.2121C>T	Unverified	GGC	TCC	GAG	3	C	T
TCGA-61-1915	SATL1	ENST00000509231	p.P484S	c.1450C>T	Unverified	gaa	ccg	ggg	1	C	T
TCGA-13-0924	SC5DL	ENST00000392789	p.V120V	c.360C>T	Verified	CTT	GTC	GTT	3	C	T
TCGA-24-1417	SCAMP3	ENST00000302631	p.R329*	c.985C>T	Unverified	GTG	CGA	ACC	1	C	T
TCGA-13-0886	SCAND3	ENST00000452236	p.A3V	c.8C>T	Verified	GAA	GCA	GTC	2	C	T
TCGA-20-1683	SCARF1	ENST00000263071	p.P687S	c.2059C>T	Unverified	GGC	CCT	GTG	1	C	T
TCGA-24-1844	SCD5	ENST00000319540	p.N259N	c.777C>T	Unverified	CAG	AAC	CCA	3	C	T
TCGA-13-0916	SCEL	ENST00000349847	p.H361Y	c.1081C>T	Verified	GGA	CAT	GAA	1	C	T
TCGA-24-0975	SCEL	ENST00000349847	p.Q318*	c.952C>T	Unverified	AAT	CAA	AGG	1	C	T
TCGA-13-0885	SCFD2	ENST00000401642	p.P157L	c.470C>T	Verified	GTC	CCG	TTA	2	C	T
TCGA-23-1022	SCGB2A2	ENST00000227918	p.A10V	c.29C>T	Verified	CTG	GCG	GCC	2	C	T
TCGA-24-2289	SCGB3A2	ENST00000296694	p.P38L	c.113C>T	Unverified	TTA	CCT	CTG	2	C	T
TCGA-04-1342	SCN10A	ENST00000449082	p.R302*	c.904C>T	Verified	AAG	CGA	GGC	1	C	T
TCGA-13-1509	SCN10A	ENST00000449082	p.R498W	c.1492C>T	Verified	CGC	CGG	GCT	1	C	T
TCGA-10-0930	SCN10A	ENST00000449082	p.S1622F	c.4865C>T	Verified	TAC	TCT	ATC	2	C	T
TCGA-10-0930	SCN10A	ENST00000449082	p.S1622F	c.4865C>T	Verified	TAC	TCT	ATC	2	C	T
TCGA-04-1638	SCN10A	ENST00000449082	p.I75I	c.225C>T	Unverified	CTG	ATC	GGG	3	C	T
TCGA-36-1577	SCN1A	ENST00000375405	p.Q459*	c.1375C>T	Unverified	GCT	CAG	CAG	1	C	T
TCGA-13-1489	SCN1A	ENST00000375405	p.F590F	c.1770C>T	Verified	GAC	TTC	GCA	3	C	T
TCGA-13-1509	SCN2A	ENST00000375427	p.P360L	c.1079C>T	Verified	AAC	CCC	AAC	2	C	T
TCGA-13-1512	SCN3A	ENST00000283254	p.R1621*	c.4861C>T	Verified	TTC	CGA	GTG	1	C	T
TCGA-24-1616	SCN3B	ENST00000299333	p.R132W	c.394C>T	Verified	CAT	CGG	CCC	1	C	T
TCGA-29-1768	SCN5A	ENST00000333535	p.T1131T	c.3393C>T	Unverified	GAG	ACC	CCA	3	C	T
TCGA-30-1891	SCN5A	ENST00000333535	p.S1937S	c.5811C>T	Unverified	CTC	TCC	GAA	3	C	T
TCGA-29-1768	SCN5A_ENS	ENST00000333535_v68	p.T1131T	c.3393C>T	Unverified	GAG	ACC	CCA	3	C	T
TCGA-29-1768	SCN5A_ENS	ENST00000413689	p.T1131T	c.3393C>T	Unverified	GAG	ACC	CCA	3	C	T
TCGA-29-1781	SCN7A	ENST00000409855	p.A1156V	c.3467C>T	Unverified	GTT	GCT	GTT	2	C	T
TCGA-29-1781	SCN7A_ENS	ENST00000409855	p.A1156V	c.3467C>T	Unverified	GTT	GCT	GTT	2	C	T
TCGA-61-2095	SCP2	ENST00000371514	p.P356S	c.1066C>T	Unverified	CAC	CCA	CTA	1	C	T

TCGA-61-1913	SDK2	ENST00000316893	p.P1766P	c.5298C>T	Unverified	AGC	CCC	GTG	3	C	T
TCGA-09-2049	SEC16A	ENST00000313050	p.A685V	c.2054C>T	Unverified	GAA	GCT	GTG	2	C	T
TCGA-09-1674	SEC16B	ENST00000308284	p.P968L	c.2903C>T	Unverified	CTG	CCG	GAT	2	C	T
TCGA-04-1356	SEL1L2	ENST00000284951	p.T267M	c.800C>T	Verified	CTA	ACG	GAA	2	C	T
TCGA-29-1703	SELP	ENST00000367790	p.R559R	c.1677C>T	Unverified	GGA	CGC	TGG	3	C	T
TCGA-20-1683	SEMA3A	ENST00000265362	p.T696I	c.2087C>T	Unverified	ATG	ACA	CCT	2	C	T
TCGA-20-1686	SEMA5A	ENST00000382496	p.P1053L	c.3158C>T	Unverified	AAC	CCA	CAT	2	C	T
TCGA-29-1691	SEMA5B	ENST00000357599	p.Q446*	c.1336C>T	Unverified	GCG	CAG	CGC	1	C	T
TCGA-29-1691	SEMA5B_EN	ENST00000451055	p.Q500*	c.1498C>T	Unverified	GCG	CAG	CGC	1	C	T
TCGA-20-1685	SENP7	NM_020654.2	p.L302L	c.904C>T	Unverified	aag	cta	tcc	1	C	T
TCGA-25-2398	SENP7	NM_020654.2	p.L797L	c.2391C>T	Verified	tat	ctc	gca	3	C	T
TCGA-20-1685	SENP7_ENST	ENST00000394095	p.L368L	c.1102C>T	Unverified	AAA	CTA	TCC	1	C	T
TCGA-61-1900	SERINC3	ENST00000255175	p.L333L	c.997C>T	Unverified	tta	ctg	gat	1	C	T
TCGA-24-1850	SERPINA1	ENST00000440909	p.F57F	c.171C>T	Unverified	gag	ttc	gcc	3	C	T
TCGA-61-1740	SERPINA2	XM_372532.2	p.S327F	c.980C>T	Unverified	gcc	tct	tcc	2	C	T
TCGA-20-1683	SERPINB2	ENST00000299502	p.H356H	c.1068C>T	Unverified	ttc	cac	caa	3	C	T
TCGA-13-0920	SERPINE1	ENST00000223095	p.A119V	c.356C>T	Verified	gac	gcg	atc	2	C	T
TCGA-04-1347	SESN2	ENST00000253063	p.Y375Y	c.1125C>T	Verified	acc	tac	aat	3	C	T
TCGA-13-0791	SETD1A	ENST00000262519	p.D581D	c.1743C>T	Unverified	GGA	GAC	GAC	3	C	T
TCGA-24-1470	SEZ6L	ENST00000248933	p.L824L	c.2470C>T	Verified	GTG	CTG	CTG	1	C	T
TCGA-61-1740	SEZ6L	ENST00000248933	p.D376D	c.1128C>T	Unverified	CAG	GAC	GAC	3	C	T
TCGA-25-1313	SF3B2	ENST00000322535	p.P559S	c.1675C>T	Verified	CGG	CCT	AAG	1	C	T
TCGA-13-1501	SF3B4	ENST00000271628	p.P304S	c.910C>T	Verified	CAT	CCA	GGG	1	C	T
TCGA-13-1501	SF3B4	ENST00000271628	p.P304S	c.910C>T	Verified	CAT	CCA	GGG	1	C	T
TCGA-13-0755	SFSWAP	ENST00000261674	p.V936V	c.2808C>T	Verified	AAA	GTC	AGA	3	C	T
TCGA-13-0904	SG223_HUN	ENST00000330777	p.T755T	c.2265C>T	Verified	ACC	ACC	GGC	3	C	T
TCGA-29-1695	SGEF	ENST00000356448	p.V703V	c.2109C>T	Unverified	AAC	GTC	AAT	3	C	T
TCGA-13-0904	SgK223	SU_SgK223	p.T757T	c.2271C>T	Verified	acc	acc	ggc	3	C	T
TCGA-25-2401	SGMS1	ENST00000361781	p.D251D	c.753C>T	Verified	gga	gac	tgg	3	C	T
TCGA-24-1845	SGPP1	ENST00000247225	p.P351S	c.1051C>T	Unverified	TTA	CCT	TTA	1	C	T
TCGA-24-2035	SGSH	ENST00000326317	p.P329S	c.985C>T	Verified	ATC	CCG	TAC	1	C	T
TCGA-10-0930	SGSM1	ENST00000400358	p.Q92*	c.274C>T	Verified	GTG	CAA	GAC	1	C	T
TCGA-10-0930	SGSM1	ENST00000400358	p.Q92*	c.274C>T	Verified	GTG	CAA	GAC	1	C	T
TCGA-61-1738	SGSM2	ENST00000268989	p.I683I	c.2049C>T	Unverified	AGC	ATC	GAC	3	C	T

TCGA-61-1738	SGSM2	ENST00000268989	p.I683I	c.2049C>T	Unverified	AGC	ATC	GAC	3	C	T
TCGA-13-0792	SH2B1	ENST00000337120	p.S627F	c.1880C>T	Verified	CCA	TCC	TCC	2	C	T
TCGA-09-1674	SH3BGR	ENST00000333634	p.S239F	c.716C>T	Unverified	GAT	TCC	TAG	2	C	T
TCGA-30-1891	SH3BP5	ENST00000383791	p.Q319*	c.955C>T	Unverified	ACC	CAG	TCC	1	C	T
TCGA-61-2094	SH3BP5L	ENST00000366472	p.T330T	c.990C>T	Unverified	GAC	ACC	GAT	3	C	T
TCGA-13-0807	SH3GL1	ENST00000269886	p.R290*	c.868C>T	Verified	TTC	CGA	TCT	1	C	T
TCGA-29-1702	SH3GL3	ENST00000324537	p.T97M	c.290C>T	Unverified	CAG	ACG	GAA	2	C	T
TCGA-24-1431	SH3PXD2A	ENST00000355946	p.T778M	c.2333C>T	Unverified	cag	acg	gcc	2	C	T
TCGA-13-1510	SH3RF3	ENST00000309415	p.H256H	c.768C>T	Unverified	CCA	CAC	GCC	3	C	T
TCGA-24-1470	SH3RF3	ENST00000309415	p.Y205Y	c.615C>T	Verified	AGC	TAC	GAG	3	C	T
TCGA-61-1740	SHANK1	ENST00000293441	p.P555L	c.1664C>T	Unverified	GTA	CCC	GGA	2	C	T
TCGA-29-1702	SHANK2	ENST00000294018	p.N685N	c.2055C>T	Unverified	GAG	AAC	AAG	3	C	T
TCGA-29-1702	SHANK2_EN	ENST00000338508	p.N1281N	c.3843C>T	Unverified	GAG	AAC	AAG	3	C	T
TCGA-23-1022	SHARPIN	ENST00000398712	p.A362A	c.1086C>T	Verified	AAT	GCC	CCA	3	C	T
TCGA-23-1022	SHC1_ENST(	ENST00000448116	p.A87V	c.260C>T	Verified	AGG	GCA	GCT	2	C	T
TCGA-61-1904	SHC3	ENST00000375835	p.I212I	c.636C>T	Unverified	AGC	ATC	TTG	3	C	T
TCGA-13-0755	SHH	ENST00000297261	p.D229D	c.687C>T	Unverified	GAC	GAC	CAG	3	C	T
TCGA-61-2012	SHMT2	ENST00000328923	p.R359W	c.1075C>T	Verified	GCT	CGG	GCC	1	C	T
TCGA-61-2012	SHOC2	ENST00000369452	p.P231S	c.691C>T	Verified	CTA	CCT	GCT	1	C	T
TCGA-04-1542	SHOX2_ENS	ENST00000425436	p.A151V	c.452C>T	Verified	GAT	GCG	AAA	2	C	T
TCGA-24-0975	SI	ENST00000264382	p.R250C	c.748C>T	Unverified	TTT	CGT	CAT	1	C	T
TCGA-04-1652	SIDT2	ENST00000324225	p.P600L	c.1799C>T	Unverified	CAC	CCG	GAC	2	C	T
TCGA-04-1652	SIDT2	ENST00000324225	p.P600L	c.1799C>T	Unverified	CAC	CCG	GAC	2	C	T
TCGA-13-0904	SIGLEC1	ENST00000344754	p.N1429N	c.4287C>T	Unverified	CAA	AAC	TTG	3	C	T
TCGA-23-1809	SIGLEC1	ENST00000344754	p.D1083D	c.3249C>T	Unverified	TTC	GAC	GCT	3	C	T
TCGA-13-0762	SIGLEC11	ENST00000447370	p.Q613*	c.1837C>T	Unverified	CAC	CAG	CAT	1	C	T
TCGA-13-0762	SIGLEC11	ENST00000447370	p.Q613*	c.1837C>T	Unverified	CAC	CAG	CAT	1	C	T
TCGA-24-1469	SIGLEC12	ENST00000291707	p.T89I	c.266C>T	Verified	GAG	ACT	CGG	2	C	T
TCGA-61-2095	SIGLEC12	ENST00000291707	p.T306M	c.917C>T	Unverified	CCC	ACG	ATC	2	C	T
TCGA-13-0793	SIK3	SU_QSK	p.R879R	c.2637C>T	Verified	ggg	cgc	ggc	3	C	T
TCGA-13-0900	SIN3A	ENST00000394947	p.H990Y	c.2968C>T	Verified	att	cat	gcc	1	C	T
TCGA-29-1691	SIPA1L1	ENST00000555818	p.T912I	c.2735C>T	Unverified	GAC	ACC	AGC	2	C	T
TCGA-30-1857	SIRT1	ENST00000212015	p.S159S	c.477C>T	Unverified	cat	tcc	tgt	3	C	T
TCGA-61-1900	SIX1	ENST00000247182	p.L203L	c.609C>T	Unverified	CAA	CTC	TCT	3	C	T

TCGA-13-0890	SIX5	ENST00000317578	p.L240L	c.718C>T	Verified	GGC	CTG	TCG	1	C	T
TCGA-29-1691	SKA1	ENST00000285116	p.G18G	c.54C>T	Unverified	ATT	GGC	AAT	3	C	T
TCGA-20-1683	SKAP2	ENST00000345317	p.R126C	c.376C>T	Unverified	AAA	CGC	AGA	1	C	T
TCGA-24-2262	SKIL	ENST00000259119	p.A634V	c.1901C>T	Verified	TTG	GCA	GAA	2	C	T
TCGA-61-1740	SLC11A1	ENST00000233202	p.P21S	c.61C>T	Unverified	AGC	CCG	ACC	1	C	T
TCGA-13-1481	SLC11A1	ENST00000233202	p.R273R	c.819C>T	Verified	GCC	CGC	CGA	3	C	T
TCGA-29-1693	SLC12A1	ENST00000380993	p.P494P	c.1482C>T	Unverified	GGC	CCC	CTC	3	C	T
TCGA-29-1693	SLC12A1_EN	ENST00000396577	p.P494P	c.1482C>T	Unverified	GGC	CCC	CTC	3	C	T
TCGA-61-1910	SLC12A2	ENST00000262461	p.R878C	c.2632C>T	Unverified	GGT	CGT	ATG	1	C	T
TCGA-23-1029	SLC12A5	ENST00000243964	p.F859F	c.2577C>T	Unverified	ATC	TTC	ACT	3	C	T
TCGA-61-1740	SLC12A5	ENST00000243964	p.G703G	c.2109C>T	Unverified	AAG	GGC	CTG	3	C	T
TCGA-24-2288	SLC12A6	ENST00000290209	p.A1011V	c.3032C>T	Unverified	AAA	GCG	AAG	2	C	T
TCGA-24-1104	SLC13A1	ENST00000194130	p.A153V	c.458C>T	Verified	GAG	GCT	GTA	2	C	T
TCGA-61-1910	SLC14A2	ENST00000255226	p.A372A	c.1116C>T	Unverified	CTG	GCC	CTC	3	C	T
TCGA-29-1775	SLC15A1	ENST00000376503	p.C25C	c.75C>T	Unverified	TTT	TGC	GAA	3	C	T
TCGA-13-0893	SLC16A4	ENST00000369779	p.T98I	c.293C>T	Verified	GTT	ACT	GGT	2	C	T
TCGA-09-1665	SLC17A6	ENST00000263160	p.T162T	c.486C>T	Verified	CTT	ACC	TCT	3	C	T
TCGA-13-0884	SLC1A6	ENST00000221742	p.A354A	c.1062C>T	Verified	CAT	GCC	GGC	3	C	T
TCGA-13-0755	SLC20A2	ENST00000342228	p.N31N	c.93C>T	Verified	GCC	AAC	TCC	3	C	T
TCGA-10-0930	SLC22A11	ENST00000301891	p.D109D	c.327C>T	Verified	GCT	GAC	ACG	3	C	T
TCGA-10-0930	SLC22A11	ENST00000301891	p.D109D	c.327C>T	Verified	GCT	GAC	ACG	3	C	T
TCGA-13-1507	SLC22A3	ENST00000275300	p.L556F	c.1666C>T	Verified	CAC	CTT	TGA	1	C	T
TCGA-61-1900	SLC22A6	ENST00000377871	p.L91L	c.273C>T	Unverified	TTT	CTC	AAT	3	C	T
TCGA-61-1738	SLC23A1	ENST00000353963	p.V203V	c.609C>T	Unverified	TCT	GTC	TTC	3	C	T
TCGA-61-1738	SLC23A1	ENST00000353963	p.V203V	c.609C>T	Unverified	TCT	GTC	TTC	3	C	T
TCGA-24-1417	SLC24A4	ENST00000298877	p.I461I	c.1383C>T	Unverified	ATT	ATC	GGA	3	C	T
TCGA-61-1904	SLC24A4	ENST00000298877	p.Y230Y	c.690C>T	Unverified	AAG	TAC	AAT	3	C	T
TCGA-13-0886	SLC26A11	ENST00000361193	p.S523S	c.1569C>T	Unverified	TGC	AGC	ATC	3	C	T
TCGA-29-1703	SLC26A9	ENST00000367135	p.N366N	c.1098C>T	Unverified	TCG	AAC	CAG	3	C	T
TCGA-29-1703	SLC26A9_EN	ENST00000367134	p.N366N	c.1098C>T	Unverified	TCG	AAC	CAG	3	C	T
TCGA-30-1855	SLC28A1	ENST00000286749	p.L132L	c.394C>T	Unverified	CTT	CTG	GGG	1	C	T
TCGA-30-1855	SLC28A1_EN	ENST00000338602	p.L132L	c.394C>T	Unverified	CTT	CTG	GGG	1	C	T
TCGA-13-1509	SLC28A2	ENST00000347644	p.A359V	c.1076C>T	Verified	GAT	GCA	TCA	2	C	T
TCGA-24-1104	SLC28A3	ENST00000376238	p.Y488Y	c.1464C>T	Verified	TCC	TAC	ATC	3	C	T

TCGA-13-0906	SLC29A1	ENST00000371708	p.S46F	c.137C>T	Unverified	ATG	TCC	CAG	2	C	T
TCGA-20-0990	SLC2A10	ENST00000359271	p.T411T	c.1233C>T	Verified	TGG	ACC	GCA	3	C	T
TCGA-29-1711	SLC2A2	ENST00000314251	p.P463L	c.1388C>T	Unverified	GGA	CCT	TAT	2	C	T
TCGA-29-1711	SLC2A2	ENST00000314251	p.P463L	c.1388C>T	Unverified	GGA	CCT	TAT	2	C	T
TCGA-04-1542	SLC30A9	ENST00000264451	p.G96G	c.288C>T	Verified	ATA	GGC	ACA	3	C	T
TCGA-13-1507	SLC33A1	ENST00000392845	p.Y298Y	c.894C>T	Verified	ACT	TAC	AAG	3	C	T
TCGA-24-1422	SLC33A1	ENST00000392845	p.N282N	c.846C>T	Verified	GAA	AAC	GAA	3	C	T
TCGA-24-2267	SLC35E1	ENST00000248069	p.F102F	c.306C>T	Verified	GCT	TTC	CTG	3	C	T
TCGA-30-1855	SLC36A2	ENST00000335244	p.N186N	c.558C>T	Unverified	AAC	AAC	TGC	3	C	T
TCGA-29-1769	SLC36A3	ENST00000335230	p.S335F	c.1004C>T	Unverified	TAC	TCT	ATC	2	C	T
TCGA-24-0979	SLC38A4	ENST00000266579	p.A382V	c.1145C>T	Verified	CTT	GCC	GCC	2	C	T
TCGA-04-1347	SLC38A5	ENST00000376876	p.A118V	c.353C>T	Verified	AGG	GCA	TTC	2	C	T
TCGA-13-1507	SLC38A5	ENST00000376876	p.I404I	c.1212C>T	Verified	GTT	ATC	GGG	3	C	T
TCGA-09-2051	SLC38A7	ENST00000219320	p.S207S	c.621C>T	Unverified	CTG	AGC	GTC	3	C	T
TCGA-13-0884	SLC39A11	ENST00000255559	p.A89V	c.266C>T	Verified	GGA	GCG	GCT	2	C	T
TCGA-24-0979	SLC39A2	ENST00000298681	p.H202Y	c.604C>T	Verified	GCT	CAT	AAG	1	C	T
TCGA-09-2050	SLC39A2	ENST00000298681	p.I27I	c.81C>T	Verified	CCC	ATC	TGC	3	C	T
TCGA-13-1510	SLC39A4	ENST00000301305	p.L619L	c.1857C>T	Unverified	TGG	CTC	CTC	3	C	T
TCGA-29-1691	SLC43A3	ENST00000395123	p.Q265*	c.793C>T	Unverified	AAG	CAG	GAA	1	C	T
TCGA-61-2113	SLC45A1	ENST00000289877	p.F608F	c.1824C>T	Unverified	TAC	TTC	ATC	3	C	T
TCGA-13-0923	SLC45A2	ENST00000296589	p.L226L	c.678C>T	Verified	GTG	CTC	ACT	3	C	T
TCGA-20-0991	SLC45A4	ENST00000024061	p.V507V	c.1521C>T	Verified	GGG	GTC	AAG	3	C	T
TCGA-20-1687	SLC45A4	ENST00000024061	p.A684A	c.2052C>T	Unverified	TTG	GCC	GGC	3	C	T
TCGA-23-1120	SLC4A1	ENST00000262418	p.T728T	c.2184C>T	Verified	ACC	ACC	GTG	3	C	T
TCGA-24-1846	SLC4A4	ENST00000340595	p.R397W	c.1189C>T	Unverified	GGA	CGG	TTC	1	C	T
TCGA-24-1846	SLC4A4_ENS	ENST00000425175	p.R441W	c.1321C>T	Unverified	GGA	CGG	TTC	1	C	T
TCGA-23-1124	SLC4A5	ENST00000394019	p.R217C	c.649C>T	Verified	CAC	CGC	CAC	1	C	T
TCGA-13-0885	SLC5A9	ENST00000236495	p.L531L	c.1591C>T	Verified	ATC	CTG	GAG	1	C	T
TCGA-61-2009	SLC5A9	ENST00000236495	p.R61*	c.181C>T	Verified	AGT	CGA	GGG	1	C	T
TCGA-09-2044	SLC6A1	ENST00000287766	p.F122F	c.366C>T	Verified	ATG	TTC	AAG	3	C	T
TCGA-13-0885	SLC6A16	ENST00000335875	p.A318V	c.953C>T	Verified	GGG	GCA	AAA	2	C	T
TCGA-29-1764	SLC6A18	ENST00000324642	p.I331I	c.993C>T	Unverified	CTC	ATC	AAC	3	C	T
TCGA-04-1649	SLC6A4	ENST00000261707	p.R234C	c.700C>T	Unverified	ACG	CGC	CAC	1	C	T
TCGA-24-2035	SLC6A9	ENST00000372310	p.C550C	c.1650C>T	Unverified	CTC	TGC	ATC	3	C	T

TCGA-29-2427	SLC8A3	ENST00000381269	p.D471D	c.1413C>T	Verified	GAT	GAC	GAC	3	C	T
TCGA-13-0887	SLC9A1	ENST00000263980	p.G702G	c.2106C>T	Verified	ATC	GGC	TCA	3	C	T
TCGA-20-1685	SLC9A6	ENST00000370698	p.A287V	c.860C>T	Unverified	aca	gcg	atg	2	C	T
TCGA-13-1507	SLC9A9	ENST00000316549	p.T128I	c.383C>T	Verified	atg	aca	ttt	2	C	T
TCGA-25-2042	SLCO1B3	ENST00000381545	p.C430C	c.1290C>T	Unverified	ATC	TGC	GAA	3	C	T
TCGA-24-1104	SLCO4A1	ENST00000370507	p.A325A	c.975C>T	Verified	ACC	GCC	GTT	3	C	T
TCGA-61-2012	SLFN13	ENST00000285013	p.Q368*	c.1102C>T	Verified	TCT	CAG	TTG	1	C	T
TCGA-24-2288	SLITRK1	ENST00000377084	p.I236I	c.708C>T	Unverified	CTG	ATC	GGC	3	C	T
TCGA-24-2262	SLITRK2	ENST00000447897	p.R22C	c.64C>T	Verified	agt	cgc	aaa	1	C	T
TCGA-04-1542	SLITRK3	ENST00000241274	p.P785L	c.2354C>T	Verified	CCA	CCG	GGA	2	C	T
TCGA-09-2056	SLITRK3	ENST00000241274	p.T832M	c.2495C>T	Verified	GTG	ACG	GTG	2	C	T
TCGA-29-1770	SLITRK5	ENST00000325089	p.P649S	c.1945C>T	Unverified	GCC	CCC	GCG	1	C	T
TCGA-24-1474	SMARCA1	NM_003069.2	p.R253*	c.757C>T	Verified	aaa	cga	tgg	1	C	T
TCGA-24-1474	SMARCA4	ENST00000429416	p.L1108L	c.3324C>T	Verified	tcc	ctc	atg	3	C	T
TCGA-24-1604	SMARCA4	ENST00000429416	p.S613S	c.1839C>T	Verified	atg	agc	gac	3	C	T
TCGA-13-0762	SMARCB1	ENST00000263121	p.P351S	c.1051C>T	Unverified	tgc	cca	ctg	1	C	T
TCGA-13-0762	SMARCB1	ENST00000263121	p.P351S	c.1051C>T	Unverified	tgc	cca	ctg	1	C	T
TCGA-13-0893	SMG5	ENST00000361813	p.R634R	c.1902C>T	Verified	GGA	CGC	TCC	3	C	T
TCGA-13-0793	SMG6	ENST00000263073	p.Q283*	c.847C>T	Unverified	CGA	CAG	GAT	1	C	T
TCGA-25-1318	SMG6	ENST00000263073	p.Q283*	c.847C>T	Unverified	CGA	CAG	GAT	1	C	T
TCGA-25-1318	SMG6	ENST00000263073	p.Q283*	c.847C>T	Unverified	CGA	CAG	GAT	1	C	T
TCGA-04-1336	SMG8	ENST00000300917	p.P370S	c.1108C>T	Verified	GTG	CCT	GCA	1	C	T
TCGA-29-1698	SMOC1	ENST00000361956	p.R96C	c.286C>T	Unverified	TGT	CGC	CTG	1	C	T
TCGA-13-0886	SMOC1	ENST00000361956	p.S22F	c.65C>T	Unverified	CTG	TCC	CCT	2	C	T
TCGA-24-2289	SMTN	ENST00000333137	p.N483N	c.1449C>T	Unverified	GGC	AAC	CAG	3	C	T
TCGA-29-1785	SMU1	ENST00000397149	p.G151G	c.453C>T	Unverified	GCT	GGC	GAA	3	C	T
TCGA-29-1785	SMU1	ENST00000397149	p.G151G	c.453C>T	Unverified	GCT	GGC	GAA	3	C	T
TCGA-23-1123	SMYD1	ENST00000419482	p.A107V	c.320C>T	Verified	CTG	GCG	GCG	2	C	T
TCGA-23-1123	SMYD1	ENST00000419482	p.A107V	c.320C>T	Verified	CTG	GCG	GCG	2	C	T
TCGA-24-2289	SNCAIP	ENST00000261368	p.P388L	c.1163C>T	Unverified	ACT	CCA	GCA	2	C	T
TCGA-24-2289	SNCAIP_ENS	ENST00000379533	p.P435L	c.1304C>T	Unverified	ACT	CCA	GCA	2	C	T
TCGA-24-2289	SNPH	ENST00000381873	p.Q173*	c.517C>T	Unverified	ATC	CAG	AAC	1	C	T
TCGA-04-1342	SNRNP200	ENST00000323853	p.R739W	c.2215C>T	Verified	ATC	CGG	GAC	1	C	T
TCGA-24-1422	SNRNP200	ENST00000323853	p.T239I	c.716C>T	Verified	TGC	ACC	CTC	2	C	T

TCGA-23-1114	SNRPB	ENST00000438552	p.S6S	c.18C>T	Unverified	aag	agc	agc	3	C	T
TCGA-09-1665	SNTG1	ENST00000518864	p.I209I	c.627C>T	Verified	CTG	ATC	CCT	3	C	T
TCGA-13-0760	SNX17	ENST00000233575	p.L266L	c.796C>T	Verified	acg	ctg	cgg	1	C	T
TCGA-61-2095	SOCS5	ENST00000306503	p.S195S	c.585C>T	Unverified	tac	agc	aag	3	C	T
TCGA-04-1362	SON	ENST00000356577	p.S1782F	c.5345C>T	Verified	GAG	TCT	TCT	2	C	T
TCGA-29-1784	SORBS2	ENST00000284776	p.P111L	c.332C>T	Unverified	aag	ccg	gat	2	C	T
TCGA-29-1784	SORBS2_EN!	ENST00000393528	p.P157L	c.470C>T	Unverified	AAG	CCG	GAT	2	C	T
TCGA-29-1784	SORBS2_EN!	ENST00000437304	p.P290L	c.869C>T	Unverified	AAG	CCG	GAT	2	C	T
TCGA-29-1784	SORBS2_EN!	ENST00000448662	p.P180L	c.539C>T	Unverified	AAG	CCG	GAT	2	C	T
TCGA-29-1691	SORCS3	ENST00000369701	p.S233L	c.698C>T	Unverified	AGG	TCG	ACA	2	C	T
TCGA-04-1347	SORL1	ENST00000260197	p.D1434D	c.4302C>T	Verified	ATG	GAC	ACC	3	C	T
TCGA-29-1690	SOS1	ENST00000426016	p.P1276L	c.3827C>T	Unverified	tca	cca	cca	2	C	T
TCGA-24-1422	SOX5	ENST00000451604	p.S370F	c.1109C>T	Verified	gta	tct	cct	2	C	T
TCGA-23-1123	SOX6	ENST00000316399	p.R592C	c.1774C>T	Verified	GCC	CGC	GGC	1	C	T
TCGA-23-1123	SOX6	ENST00000316399	p.R592C	c.1774C>T	Verified	GCC	CGC	GGC	1	C	T
TCGA-61-2113	SP4	ENST00000222584	p.R665*	c.1993C>T	Verified	TTA	CGA	GCA	1	C	T
TCGA-23-1032	SPAG1	ENST00000388798	p.L900L	c.2698C>T	Verified	CAG	CTG	TTT	1	C	T
TCGA-24-1843	SPAM1	ENST00000340011	p.S430F	c.1289C>T	Unverified	TTT	TCT	GAA	2	C	T
TCGA-24-1843	SPAM1_ENS	ENST00000439500	p.S430F	c.1289C>T	Unverified	TTT	TCT	GAA	2	C	T
TCGA-13-0760	SPDEF	ENST00000374037	p.S3S	c.9C>T	Verified	ggc	agc	gcc	3	C	T
TCGA-24-1463	SPECC1	ENST00000261503	p.S131L	c.392C>T	Verified	AAA	TCA	GTG	2	C	T
TCGA-13-1509	SPEF2_ENST	ENST00000356031	p.S1319L	c.3956C>T	Verified	AAA	TCA	CCA	2	C	T
TCGA-24-1469	SPEG	SU_SPEG	p.T159M	c.476C>T	Verified	ccc	acg	ggg	2	C	T
TCGA-04-1338	SPG11	ENST00000261866	p.L920L	c.2758C>T	Verified	CTT	CTG	ACT	1	C	T
TCGA-23-1117	SPG11	ENST00000261866	p.L113L	c.337C>T	Verified	GAA	CTG	CTT	1	C	T
TCGA-23-1117	SPG11	ENST00000261866	p.L113L	c.337C>T	Verified	GAA	CTG	CTT	1	C	T
TCGA-61-1915	SPG11	ENST00000261866	p.V1908V	c.5724C>T	Unverified	GAT	GTC	GCC	3	C	T
TCGA-09-2049	SPI1	NM_003120.1	p.S247S	c.741C>T	Unverified	ttc	agc	ggc	3	C	T
TCGA-61-2109	SPIN2A	ENST00000374908	p.V251V	c.753C>T	Unverified	TAT	GTC	TAC	3	C	T
TCGA-29-1783	SPIN3_ENST	ENST00000374919	p.P170L	c.509C>T	Unverified	GAT	CCT	GTA	2	C	T
TCGA-20-0990	SPIRE1	ENST00000309836	p.T323I	c.968C>T	Unverified	TCC	ACA	AGG	2	C	T
TCGA-61-2113	SPOCK2	ENST00000317376	p.T259T	c.777C>T	Unverified	GAC	ACC	AGT	3	C	T
TCGA-61-1910	SPPL2A	ENST00000261854	p.P272L	c.815C>T	Unverified	ata	cca	tat	2	C	T
TCGA-29-1761	SPRYD5	NM_032681.1	p.H38Y	c.112C>T	Unverified	cat	cac	ttg	1	C	T

TCGA-29-1761	SPRYD5_ENS	ENST00000327733	p.H197Y	c.589C>T	Unverified	CAT	CAC	TTG	1	C	T
TCGA-61-2012	SPTAN1	ENST00000372731	p.S43F	c.128C>T	Verified	GAT	TCC	TAT	2	C	T
TCGA-13-0893	SPTBN1	ENST00000356805	p.L962F	c.2884C>T	Verified	CAC	CTC	GAG	1	C	T
TCGA-13-1512	SPTBN4	ENST00000352632	p.S2105S	c.6315C>T	Verified	TTC	AGC	TCT	3	C	T
TCGA-25-1313	SPTBN4	ENST00000352632	p.S235S	c.705C>T	Verified	AAG	TCC	AAT	3	C	T
TCGA-23-2078	SPTBN5	ENST00000320955	p.C842C	c.2526C>T	Unverified	TCC	TGC	CAC	3	C	T
TCGA-13-1497	SRCAP	ENST00000262518	p.S956F	c.2867C>T	Verified	GTC	TCT	CGA	2	C	T
TCGA-25-1326	SRPK2	ENST00000357311	p.V146V	c.438C>T	Unverified	gtg	gtc	cag	3	C	T
TCGA-24-1474	SRRT	ENST00000347433	p.S366S	c.1098C>T	Verified	GGC	AGC	GTG	3	C	T
TCGA-10-0930	SS18L1	ENST00000331758	p.R293W	c.877C>T	Verified	GAC	CGG	TCC	1	C	T
TCGA-10-0930	SS18L1	ENST00000331758	p.R293W	c.877C>T	Verified	GAC	CGG	TCC	1	C	T
TCGA-13-0760	SSH1	ENST00000326495	p.A401V	c.1202C>T	Verified	TCG	GCC	TCC	2	C	T
TCGA-23-1032	SSH3	ENST00000308127	p.S636F	c.1907C>T	Unverified	ATT	TCC	TCT	2	C	T
TCGA-09-2044	SSRP1	ENST00000278412	p.N498N	c.1494C>T	Verified	agc	aac	gcc	3	C	T
TCGA-61-1725	ST18	ENST00000276480	p.R557C	c.1669C>T	Unverified	GGC	CGT	GCC	1	C	T
TCGA-61-2009	ST3GAL3	ENST00000361746	p.R190W	c.568C>T	Verified	ATC	CGG	GAG	1	C	T
TCGA-13-1509	ST6GAL2	ENST00000361686	p.S360L	c.1079C>T	Verified	AGT	TCA	CTG	2	C	T
TCGA-13-0893	ST6GAL2	ENST00000361686	p.A64A	c.192C>T	Verified	GGC	GCC	GCA	3	C	T
TCGA-61-1914	ST6GALNAC	ENST00000156626	p.T163M	c.488C>T	Unverified	AAG	ACG	ACC	2	C	T
TCGA-09-2051	ST8SIA1	ENST00000396037	p.S120L	c.359C>T	Verified	AAT	TCA	ACT	2	C	T
TCGA-29-1690	STAB1	ENST00000321725	p.P1458P	c.4374C>T	Unverified	GAC	CCC	TGC	3	C	T
TCGA-23-1022	STAB2	ENST00000388887	p.L583F	c.1747C>T	Verified	AAG	CTT	CTG	1	C	T
TCGA-61-1906	STAB2	ENST00000388887	p.R69*	c.205C>T	Unverified	GTT	CGA	GAT	1	C	T
TCGA-61-1906	STAB2	ENST00000388887	p.R69*	c.205C>T	Unverified	GTT	CGA	GAT	1	C	T
TCGA-24-1616	STAB2	ENST00000388887	p.A1874A	c.5622C>T	Verified	GTG	GCC	TAC	3	C	T
TCGA-20-0990	STAG2	ENST00000371160	p.R184W	c.550C>T	Verified	gta	cgg	caa	1	C	T
TCGA-61-1738	STAG3	ENST00000426455	p.S1016F	c.3047C>T	Unverified	ttt	tcc	ccc	2	C	T
TCGA-61-1738	STAG3	ENST00000426455	p.S1016F	c.3047C>T	Unverified	ttt	tcc	ccc	2	C	T
TCGA-61-1740	STAM	ENST00000377524	p.L342L	c.1026C>T	Unverified	cct	ctc	att	3	C	T
TCGA-23-1021	STAT4	ENST00000358470	p.H401Y	c.1201C>T	Verified	cga	cat	ttg	1	C	T
TCGA-20-0991	STAT6	ENST00000300134	p.V7V	c.21C>T	Verified	ctg	gtc	tcc	3	C	T
TCGA-13-0913	STAU1	ENST00000371856	p.A359V	c.1076C>T	Verified	cag	gcg	cag	2	C	T
TCGA-13-0887	STK33	ENST00000447869	p.T140M	c.419C>T	Verified	GAA	ACG	AAG	2	C	T
TCGA-29-1763	STRA6	ENST00000395105	p.A130V	c.389C>T	Unverified	CTC	GCC	TCA	2	C	T

TCGA-29-1711	STRC_ENST(	ENST00000450892	p.T259I	c.776C>T	Unverified	GTC	ACT	CAC	2	C	T
TCGA-29-1711	STRC_ENST(	ENST00000450892	p.T259I	c.776C>T	Unverified	GTC	ACT	CAC	2	C	T
TCGA-29-1776	STX16	ENST00000371132	p.Q243*	c.727C>T	Unverified	GAA	CAG	GGT	1	C	T
TCGA-61-1915	STX5	ENST00000294179	p.Q210*	c.628C>T	Unverified	GAG	CAG	TTC	1	C	T
TCGA-29-1693	STXBP3	ENST00000370008	p.F110F	c.330C>T	Unverified	TAC	TTC	ACT	3	C	T
TCGA-29-1775	SUB1	ENST00000265073	p.S4L	c.11C>T	Unverified	AAA	TCA	AAG	2	C	T
TCGA-20-1683	SULF2	ENST00000359930	p.Y604Y	c.1812C>T	Unverified	tgc	tac	atc	3	C	T
TCGA-09-0369	SUMF2	ENST00000275607	p.P297L	c.890C>T	Verified	CGG	CCG	CCA	2	C	T
TCGA-09-0369	SUMF2	ENST00000275607	p.P297L	c.890C>T	Verified	CGG	CCG	CCA	2	C	T
TCGA-24-2289	SUN5	ENST00000356173	p.Y193Y	c.579C>T	Unverified	GAT	TAC	ATC	3	C	T
TCGA-13-1497	SUPT5H	ENST00000359191	p.R416W	c.1246C>T	Verified	GAG	CGG	GAG	1	C	T
TCGA-24-1850	SURF5	ENST00000343730	p.D121D	c.363C>T	Unverified	CGA	GAC	GAG	3	C	T
TCGA-13-0905	SUSD5	ENST00000309558	p.C86C	c.258C>T	Verified	GTG	TGC	ACC	3	C	T
TCGA-25-1326	SV2A	ENST00000369146	p.R419C	c.1255C>T	Verified	CAG	CGC	TGG	1	C	T
TCGA-25-2401	SV2B	ENST00000394232	p.G264G	c.792C>T	Verified	TGG	GGC	TTC	3	C	T
TCGA-61-1904	SVEP1	ENST00000401783	p.T2519T	c.7557C>T	Unverified	CAG	ACC	GTT	3	C	T
TCGA-13-0885	SWAP70	ENST00000318950	p.A480V	c.1439C>T	Verified	GAG	GCG	GAG	2	C	T
TCGA-13-0885	SWT1	ENST00000367501	p.N216N	c.648C>T	Verified	TAT	AAC	TCC	3	C	T
TCGA-61-2008	SYCP2	ENST00000357552	p.S1161L	c.3482C>T	Verified	AAG	TCA	CCC	2	C	T
TCGA-61-1904	SYMPK	ENST00000245934	p.T193T	c.579C>T	Unverified	GTC	ACC	CTG	3	C	T
TCGA-25-2392	SYNC	ENST00000373484	p.A51V	c.152C>T	Verified	GAG	GCC	CGG	2	C	T
TCGA-29-1699	SYNCRIP	ENST00000369622	p.R577W	c.1729C>T	Unverified	AAG	CGG	CGC	1	C	T
TCGA-13-0900	SYNE2	ENST00000358025	p.L2522F	c.7564C>T	Verified	GTC	CTC	AGA	1	C	T
TCGA-23-1111	SYNE2	ENST00000358025	p.S285S	c.855C>T	Unverified	TAT	TCC	AAA	3	C	T
TCGA-61-1915	SYNGAP1	ENST00000418600	p.A585V	c.1754C>T	Unverified	ATC	GCA	GAC	2	C	T
TCGA-61-1915	SYNGAP1_EI	ENST00000293748	p.A585V	c.1754C>T	Unverified	ATC	GCA	GAC	2	C	T
TCGA-61-1900	SYNJ1	NM_003895.2	p.A691V	c.2072C>T	Unverified	ttt	gct	gca	2	C	T
TCGA-61-1900	SYNJ1_ENST	ENST00000433931	p.A730V	c.2189C>T	Unverified	TTT	GCT	GCA	2	C	T
TCGA-61-2012	SYNM	ENST00000328642	p.D292D	c.876C>T	Verified	CGG	GAC	TAT	3	C	T
TCGA-13-1507	SYNPO2	ENST00000307142	p.S1022L	c.3065C>T	Verified	GCT	TCG	TCA	2	C	T
TCGA-29-1695	SYPL2	ENST00000369872	p.A115A	c.345C>T	Unverified	CCC	GCC	GAG	3	C	T
TCGA-29-1695	SYPL2	ENST00000369872	p.F48F	c.144C>T	Unverified	ATT	TTC	GCC	3	C	T
TCGA-25-2392	SYPL2	ENST00000369872	p.C96C	c.288C>T	Verified	CTC	TGC	GAT	3	C	T
TCGA-29-1762	SYT10	ENST00000228567	p.Q159*	c.475C>T	Unverified	GTG	CAA	AGA	1	C	T

TCGA-29-1693	SYTL2	ENST00000354566	p.P751P	c.2253C>T	Unverified	CAA	CCC	ATT	3	C	T
TCGA-29-1696	SYTL2_ENST	ENST00000316356	p.L46L	c.136C>T	Unverified	CAG	CTG	AAG	1	C	T
TCGA-29-1696	SYTL2_ENST	ENST00000316356	p.L46L	c.136C>T	Unverified	CAG	CTG	AAG	1	C	T
TCGA-13-1498	SYVN1	ENST00000377190	p.R508C	c.1522C>T	Verified	CTG	CGT	AAC	1	C	T
TCGA-20-1687	SZT2	ENST00000372442	p.Q2299*	c.6895C>T	Unverified	AGC	CAG	CGA	1	C	T
TCGA-20-1687	SZT2_ENST0	ENST00000562955	p.Q3141*	c.9421C>T	Unverified	AGC	CAG	CGA	1	C	T
TCGA-24-2290	TAAR8	ENST00000275200	p.S39S	c.117C>T	Verified	TTT	AGC	TTT	3	C	T
TCGA-24-2290	TAAR8	ENST00000275200	p.S39S	c.117C>T	Verified	TTT	AGC	TTT	3	C	T
TCGA-13-0920	TACC2	ENST00000369005	p.S408F	c.1223C>T	Verified	CCT	TCC	CTG	2	C	T
TCGA-13-1512	TACC3	ENST00000313288	p.A148V	c.443C>T	Verified	GGT	GCC	CTG	2	C	T
TCGA-30-1718	TADA2B	ENST00000310074	p.Y369Y	c.1107C>T	Unverified	CGC	TAC	GTG	3	C	T
TCGA-09-2050	TAF1C	ENST00000378541	p.A655V	c.1964C>T	Verified	ACA	GCA	CCC	2	C	T
TCGA-25-2042	TAF3	ENST00000344293	p.D565D	c.1695C>T	Unverified	AAA	GAC	AAG	3	C	T
TCGA-24-1604	TAF5L	ENST00000366675	p.P254L	c.761C>T	Verified	CCT	CCC	TCC	2	C	T
TCGA-13-0886	TAF6L	ENST00000294168	p.P423S	c.1267C>T	Verified	CCG	CCA	GGG	1	C	T
TCGA-23-1122	TAF8	ENST00000372977	p.L309L	c.927C>T	Verified	TCC	CTC	TCC	3	C	T
TCGA-61-1895	TAL2_ENST0	ENST00000334077	p.A69V	c.206C>T	Unverified	GTG	GCT	GCT	2	C	T
TCGA-30-1891	TANC1	ENST00000263635	p.Q1555*	c.4663C>T	Unverified	GTT	CAG	ATC	1	C	T
TCGA-29-1766	TANC2	ENST00000389520	p.R1066*	c.3196C>T	Unverified	CGC	CGA	GGA	1	C	T
TCGA-29-1766	TANC2_ENST	ENST00000389520_v65	p.R1066*	c.3196C>T	Unverified	CGC	CGA	GGA	1	C	T
TCGA-61-1900	TAOK2	ENST00000279394	p.D376D	c.1128C>T	Unverified	TCA	GAC	AAC	3	C	T
TCGA-61-1900	TAOK2_ENST	ENST00000308893	p.D376D	c.1128C>T	Unverified	TCA	GAC	AAC	3	C	T
TCGA-09-2049	TAPBP	ENST00000434618	p.R379C	c.1135C>T	Verified	GCA	CGC	TAT	1	C	T
TCGA-13-1481	TAPBP	ENST00000434618	p.H354Y	c.1060C>T	Unverified	CGC	CAC	CAT	1	C	T
TCGA-61-1998	TAPBP	ENST00000434618	p.A192V	c.575C>T	Unverified	GCC	GCC	TCA	2	C	T
TCGA-13-0885	TARDBP	ENST00000240185	p.A329V	c.986C>T	Verified	GCA	GCA	CTA	2	C	T
TCGA-23-1117	TARDBP	ENST00000240185	p.T153M	c.458C>T	Verified	TTT	ACG	GAA	2	C	T
TCGA-23-1117	TARDBP	ENST00000240185	p.T153M	c.458C>T	Verified	TTT	ACG	GAA	2	C	T
TCGA-13-0886	TARS2	ENST00000369064	p.H367Y	c.1099C>T	Verified	GGG	CAC	TGG	1	C	T
TCGA-20-1683	TAS2R38	ENST00000547270	p.L107L	c.321C>T	Unverified	AAC	CTC	TGG	3	C	T
TCGA-61-2009	TAT	ENST00000355962	p.P3S	c.7C>T	Verified	GAC	CCA	TAC	1	C	T
TCGA-04-1542	TBC1D1	ENST00000261439	p.Q137*	c.409C>T	Verified	GAT	CAA	ACA	1	C	T
TCGA-24-1422	TBC1D1	ENST00000261439	p.F260F	c.780C>T	Unverified	GGC	TTC	TTC	3	C	T
TCGA-04-1338	TBC1D7	ENST00000379300	p.R110C	c.328C>T	Verified	CTC	CGC	ATG	1	C	T

TCGA-23-1809	TBC1D8B	ENST00000357242	p.R370C	c.1108C>T	Unverified	ttt	cgc	ttc	1	C	T
TCGA-23-1809	TBC1D8B_EI	ENST00000310452	p.R370C	c.1108C>T	Unverified	TTT	CGC	TTC	1	C	T
TCGA-24-2267	TBL3	ENST00000568546	p.H38Y	c.112C>T	Unverified	CAG	CAC	CTC	1	C	T
TCGA-04-1343	TBR1	ENST00000389554	p.N154N	c.462C>T	Verified	ACC	AAC	GGA	3	C	T
TCGA-04-1347	TBX1	ENST00000332710	p.P197L	c.590C>T	Verified	CAC	CCG	GAC	2	C	T
TCGA-30-1714	TBX15	ENST00000207157	p.S349L	c.1046C>T	Unverified	CCC	TCG	TTG	2	C	T
TCGA-23-1116	TBX2	ENST00000240328	p.A648A	c.1944C>T	Unverified	AAG	GCC	GCT	3	C	T
TCGA-04-1337	TBX4	ENST00000240335	p.P380S	c.1138C>T	Unverified	agc	ccc	tcc	1	C	T
TCGA-25-1313	TBX5	ENST00000310346	p.R375W	c.1123C>T	Verified	CAG	CGG	CAA	1	C	T
TCGA-13-1499	TCEAL8	ENST00000360000	p.S43S	c.129C>T	Verified	gta	agc	cag	3	C	T
TCGA-24-1616	TCL1B	ENST00000340722	p.P92P	c.276C>T	Verified	TAC	CCC	GGG	3	C	T
TCGA-29-1711	TCP11L2	ENST00000299045	p.S16S	c.48C>T	Unverified	CCA	AGC	GAT	3	C	T
TCGA-29-1711	TCP11L2	ENST00000299045	p.S16S	c.48C>T	Unverified	CCA	AGC	GAT	3	C	T
TCGA-29-1784	TCTE1	ENST00000371505	p.A66V	c.197C>T	Unverified	ATT	GCT	GAG	2	C	T
TCGA-04-1649	TDRD1	ENST00000251864	p.R827W	c.2479C>T	Unverified	ATA	CGG	TGC	1	C	T
TCGA-24-1849	TDRD3	ENST00000377894	p.S37L	c.110C>T	Unverified	CTC	TCA	GGC	2	C	T
TCGA-61-2008	TEKT5	ENST00000283025	p.P479L	c.1436C>T	Verified	ACC	CCG	CGC	2	C	T
TCGA-24-1604	TEP1	ENST00000262715	p.Q1309*	c.3925C>T	Verified	GAG	CAG	AGC	1	C	T
TCGA-13-0924	TESK2	NM_007170	p.H110Y	c.328C>T	Verified	tcc	cat	ccc	1	C	T
TCGA-13-0919	TEX14	ENST00000349033	p.P1370L	c.4109C>T	Unverified	CAG	CCA	CCT	2	C	T
TCGA-13-0919	TEX14_ENST	ENST00000240361	p.P1416L	c.4247C>T	Unverified	CAG	CCA	CCT	2	C	T
TCGA-13-0920	TEX15	ENST00000256246	p.L1383F	c.4147C>T	Verified	GAG	CTT	CTA	1	C	T
TCGA-29-1770	TFAP2B	ENST00000393655	p.T368M	c.1103C>T	Unverified	TTT	ACG	GAT	2	C	T
TCGA-23-2077	TFAP2D	ENST00000008391	p.P389L	c.1166C>T	Verified	ACT	CCG	GCA	2	C	T
TCGA-61-1900	TFCP2L1	ENST00000263707	p.N360N	c.1080C>T	Unverified	TTC	AAC	GCC	3	C	T
TCGA-24-1843	TFRC	ENST00000360110	p.Y689Y	c.2067C>T	Unverified	CCC	TAC	GTA	3	C	T
TCGA-29-1695	TG	ENST00000220616	p.A2097A	c.6291C>T	Unverified	CTG	GCC	CTC	3	C	T
TCGA-29-1761	TG	ENST00000220616	p.T199T	c.597C>T	Unverified	AAC	ACC	ACA	3	C	T
TCGA-24-2280	TGIF2	ENST00000373872	p.F180F	c.540C>T	Unverified	CTC	TTC	AAC	3	C	T
TCGA-13-0893	TGM6	ENST00000202625	p.F462F	c.1386C>T	Verified	CTG	TTC	GGC	3	C	T
TCGA-61-1910	TGM6	ENST00000202625	p.G244G	c.732C>T	Unverified	GGC	GGC	GGC	3	C	T
TCGA-13-0906	THADA	ENST00000405975	p.L278F	c.832C>T	Unverified	ctg	ctt	cgt	1	C	T
TCGA-13-0906	THADA	ENST00000405975	p.R279C	c.835C>T	Unverified	ctt	cgt	tca	1	C	T
TCGA-23-1032	THBS2	ENST00000366787	p.T99M	c.296C>T	Verified	GGC	ACG	CTG	2	C	T

TCGA-61-1914	THBS2	ENST00000366787	p.D863D	c.2589C>T	Unverified	GAG	GAC	ATA	3	C	T
TCGA-13-0714	THSD7B	ENST00000272643	p.T327I	c.980C>T	Verified	TTG	ACT	GTT	2	C	T
TCGA-24-2035	TIAL1	ENST00000369093	p.T126I	c.377C>T	Verified	ATT	ACA	ACA	2	C	T
TCGA-23-1022	TIAM1	ENST00000286827	p.S170F	c.509C>T	Verified	CGC	TCC	AAA	2	C	T
TCGA-25-2391	TIAM2	ENST00000318981	p.R300W	c.898C>T	Unverified	CTC	CGG	GAA	1	C	T
TCGA-13-1499	TIAM2	ENST00000318981	p.A37V	c.110C>T	Verified	CAT	GCA	AAA	2	C	T
TCGA-20-0990	TIAM2	ENST00000318981	p.Y1194Y	c.3582C>T	Verified	TAT	TAC	GCG	3	C	T
TCGA-13-0885	TIGD6	ENST00000296736	p.D438D	c.1314C>T	Verified	CAG	GAC	ATG	3	C	T
TCGA-13-1510	TIMM44	ENST00000270538	p.D373D	c.1119C>T	Unverified	ATT	GAC	AAC	3	C	T
TCGA-24-1845	TIMMDC1	ENST00000494664	p.T80M	c.239C>T	Unverified	AAG	ACG	GCA	2	C	T
TCGA-25-1313	TINAG	ENST00000259782	p.R299C	c.895C>T	Verified	AAA	CGT	GGA	1	C	T
TCGA-30-1714	TIPARP	ENST00000295924	p.T6T	c.18C>T	Unverified	ACC	ACC	GAA	3	C	T
TCGA-29-1701	TJP3	ENST00000262968	p.Q634*	c.1900C>T	Unverified	CGA	CAG	GGC	1	C	T
TCGA-61-1913	TLE3	ENST00000317509	p.P345S	c.1033C>T	Unverified	CCT	CCG	GGC	1	C	T
TCGA-61-1913	TLE3_ENST0	ENST00000558939	p.P345S	c.1033C>T	Unverified	CCT	CCG	GGC	1	C	T
TCGA-13-1499	TLL1	ENST00000061240	p.A181A	c.543C>T	Verified	CAG	GCC	ATG	3	C	T
TCGA-29-1761	TLN2	ENST00000306829	p.A1149A	c.3447C>T	Unverified	GCG	GCC	GCC	3	C	T
TCGA-24-1843	TLR1	ENST00000308979	p.S506L	c.1517C>T	Unverified	CCA	TCG	GCT	2	C	T
TCGA-24-1843	TM4SF1	ENST00000305366	p.L125L	c.375C>T	Unverified	TCC	CTC	GGC	3	C	T
TCGA-61-2102	TM7SF4	ENST00000297581	p.P24L	c.71C>T	Verified	AGC	CCC	GGA	2	C	T
TCGA-13-1497	TM9SF1	ENST00000261789	p.R221C	c.661C>T	Verified	CGC	CGT	GGT	1	C	T
TCGA-24-1422	TM9SF2	ENST00000376387	p.A572V	c.1715C>T	Verified	GAA	GCA	ACT	2	C	T
TCGA-23-1029	TM9SF4	ENST00000217315	p.L587F	c.1759C>T	Unverified	CTC	CTC	TAC	1	C	T
TCGA-29-1761	TMCO7_ENS	ENST00000261778	p.I223I	c.669C>T	Unverified	GAT	ATC	GCA	3	C	T
TCGA-61-2109	TMEM109	ENST00000227525	p.L25F	c.73C>T	Unverified	GCC	CTT	ATC	1	C	T
TCGA-29-1691	TMEM119	ENST00000392806	p.P64L	c.191C>T	Unverified	AGC	CCC	ACA	2	C	T
TCGA-23-1117	TMEM119	ENST00000392806	p.G192G	c.576C>T	Verified	CTG	GGC	GGT	3	C	T
TCGA-23-1117	TMEM119	ENST00000392806	p.G192G	c.576C>T	Verified	CTG	GGC	GGT	3	C	T
TCGA-20-0991	TMEM132B	ENST00000299308	p.A425V	c.1274C>T	Verified	CTT	GCC	ATG	2	C	T
TCGA-20-0990	TMEM132B	ENST00000299308	p.S562S	c.1686C>T	Verified	TGC	TCC	CTG	3	C	T
TCGA-13-0791	TMEM132D	ENST00000422113	p.P219L	c.656C>T	Verified	CAG	CCG	GAG	2	C	T
TCGA-23-1117	TMEM132D	ENST00000422113	p.I267I	c.801C>T	Verified	AGG	ATC	GGG	3	C	T
TCGA-23-1117	TMEM132D	ENST00000422113	p.I267I	c.801C>T	Verified	AGG	ATC	GGG	3	C	T
TCGA-24-1849	TMEM132E	ENST00000321639	p.F787F	c.2361C>T	Unverified	GGC	TTC	CTG	3	C	T

TCGA-29-1761	TMEM174	ENST00000296776	p.R7C	c.19C>T	Unverified	GGC	CGC	TTG	1	C	T
TCGA-23-1032	TMEM176B	ENST00000326442	p.T2M	c.5C>T	Verified	ATG	ACG	CAA	2	C	T
TCGA-09-1674	TMEM177	ENST00000272521	p.L171L	c.511C>T	Unverified	GCC	CTG	CTG	1	C	T
TCGA-24-1431	TMEM181	ENST00000367090	p.A142V	c.425C>T	Verified	CTG	GCG	CCC	2	C	T
TCGA-61-1915	TMEM189	ENST00000371652	p.A68V	c.203C>T	Unverified	CTG	GCC	CGC	2	C	T
TCGA-61-1915	TMEM189-U	ENST00000341698	p.A68V	c.203C>T	Unverified	CTG	GCC	CGC	2	C	T
TCGA-09-2051	TMEM204	ENST00000253934	p.A9V	c.26C>T	Unverified	GCC	GCG	GCC	2	C	T
TCGA-13-0890	TMEM27	ENST00000380342	p.P221S	c.661C>T	Verified	acc	cct	ctc	1	C	T
TCGA-04-1530	TMEM39A	ENST00000319172	p.V431V	c.1293C>T	Unverified	AGT	GTC	GTC	3	C	T
TCGA-29-2427	TMEM53	ENST00000372237	p.H252H	c.756C>T	Verified	GCA	CAC	GTC	3	C	T
TCGA-13-0760	TMEM63A	ENST00000366835	p.Q794*	c.2380C>T	Verified	GCG	CAG	AGC	1	C	T
TCGA-13-1498	TMEM67	ENST00000453321	p.V695V	c.2085C>T	Verified	ACT	GTC	CTC	3	C	T
TCGA-30-1855	TMEM69	ENST00000372025	p.P150S	c.448C>T	Unverified	CTA	CCA	GAA	1	C	T
TCGA-29-1702	TMEM71	ENST00000356838	p.C48C	c.144C>T	Unverified	GAA	TGC	GGC	3	C	T
TCGA-24-0979	TMPRSS4	ENST00000437212	p.R175C	c.523C>T	Unverified	CTT	CGC	ATG	1	C	T
TCGA-29-1696	TNC	ENST00000350763	p.T635M	c.1904C>T	Unverified	GTG	ACG	GAA	2	C	T
TCGA-29-1696	TNC	ENST00000350763	p.T635M	c.1904C>T	Unverified	GTG	ACG	GAA	2	C	T
TCGA-23-1022	TNF	ENST00000449264	p.L151F	c.451C>T	Verified	gtg	ctc	ctc	1	C	T
TCGA-61-1915	TNFRSF11A	ENST00000269485	p.S164F	c.491C>T	Unverified	ttt	tcc	tcc	2	C	T
TCGA-13-0904	TNIP3	ENST00000057513	p.R215*	c.643C>T	Verified	GAA	CGA	TCG	1	C	T
TCGA-29-1770	TNKS1BP1	ENST00000358252	p.P1702L	c.5105C>T	Unverified	TTA	CCT	CCC	2	C	T
TCGA-04-1338	TNNI3K	ENST00000326637	p.F636F	c.1908C>T	Verified	gtg	ttc	acg	3	C	T
TCGA-29-1769	TNRC6A	ENST00000395799	p.Q1469*	c.4405C>T	Unverified	CAG	CAG	ACT	1	C	T
TCGA-29-1777	TNS1	ENST00000171887	p.G1281G	c.3843C>T	Unverified	CAA	GGC	AAC	3	C	T
TCGA-04-1331	TP53	ENST00000269305	p.R306*	c.916C>T	Verified	AAG	CGA	GCA	1	C	T
TCGA-04-1336	TP53	ENST00000269305	p.R248W	c.742C>T	Verified	AAC	CGG	AGG	1	C	T
TCGA-13-0884	TP53	ENST00000269305	p.R342*	c.1024C>T	Verified	TTC	CGA	GAG	1	C	T
TCGA-13-0887	TP53	ENST00000269305	p.R213*	c.637C>T	Verified	TTT	CGA	CAT	1	C	T
TCGA-13-0916	TP53	ENST00000269305	p.R196*	c.586C>T	Verified	ATC	CGA	GTG	1	C	T
TCGA-13-0920	TP53	ENST00000269305	p.Q100*	c.298C>T	Verified	TCC	CAG	AAA	1	C	T
TCGA-13-1488	TP53	ENST00000269305	p.R306*	c.916C>T	Verified	AAG	CGA	GCA	1	C	T
TCGA-13-1489	TP53	ENST00000269305	p.R342*	c.1024C>T	Verified	TTC	CGA	GAG	1	C	T
TCGA-13-1499	TP53	ENST00000269305	p.R273C	c.817C>T	Verified	GTG	CGT	GTT	1	C	T
TCGA-13-1510	TP53	ENST00000269305	p.R213*	c.637C>T	Verified	TTT	CGA	CAT	1	C	T

TCGA-20-0990	TP53	ENST00000269305	p.Q144*	c.430C>T	Verified	GTG	CAG	CTG	1	C	T
TCGA-23-1118	TP53	ENST00000269305	p.P151S	c.451C>T	Verified	ACA	CCC	CCG	1	C	T
TCGA-24-1417	TP53	ENST00000269305	p.R248W	c.742C>T	Verified	AAC	CGG	AGG	1	C	T
TCGA-24-1422	TP53	ENST00000269305	p.R248W	c.742C>T	Verified	AAC	CGG	AGG	1	C	T
TCGA-24-1431	TP53	ENST00000269305	p.Q192*	c.574C>T	Verified	CCT	CAG	CAT	1	C	T
TCGA-24-1474	TP53	ENST00000269305	p.Q192*	c.574C>T	Verified	CCT	CAG	CAT	1	C	T
TCGA-24-2280	TP53	ENST00000269305	p.Q192*	c.574C>T	Verified	CCT	CAG	CAT	1	C	T
TCGA-24-2289	TP53	ENST00000269305	p.R196*	c.586C>T	Verified	ATC	CGA	GTG	1	C	T
TCGA-25-2392	TP53	ENST00000269305	p.R273C	c.817C>T	Verified	GTG	CGT	GTT	1	C	T
TCGA-29-1775	TP53	ENST00000269305	p.Q104*	c.310C>T	Unverified	TAC	CAG	GGC	1	C	T
TCGA-04-1367	TP53	ENST00000269305	p.S241F	c.722C>T	Verified	AGT	TCC	TGC	2	C	T
TCGA-13-0714	TP53	ENST00000269305	p.S127F	c.380C>T	Verified	TAC	TCC	CCT	2	C	T
TCGA-20-0991	TP53	ENST00000269305	p.S241F	c.722C>T	Verified	AGT	TCC	TGC	2	C	T
TCGA-23-1021	TP53	ENST00000269305	p.A159V	c.476C>T	Verified	CGC	GCC	ATG	2	C	T
TCGA-24-2288	TP53	ENST00000269305	p.P250L	c.749C>T	Verified	AGG	CCC	ATC	2	C	T
TCGA-13-1510	TP53_ENST(	ENST00000269305	p.R213*	c.637C>T	Verified	TTT	CGA	CAT	1	C	T
TCGA-24-1417	TP53_ENST(	ENST00000269305	p.R248W	c.742C>T	Verified	AAC	CGG	AGG	1	C	T
TCGA-24-1431	TP53_ENST(	ENST00000269305	p.Q192*	c.574C>T	Verified	CCT	CAG	CAT	1	C	T
TCGA-24-2280	TP53_ENST(	ENST00000269305	p.Q192*	c.574C>T	Verified	CCT	CAG	CAT	1	C	T
TCGA-29-1775	TP53_ENST(	ENST00000269305	p.Q104*	c.310C>T	Unverified	TAC	CAG	GGC	1	C	T
TCGA-13-1510	TP53_ENST(	ENST00000413465	p.R213*	c.637C>T	Verified	TTT	CGA	CAT	1	C	T
TCGA-24-1417	TP53_ENST(	ENST00000413465	p.R248W	c.742C>T	Verified	AAC	CGG	AGG	1	C	T
TCGA-24-1431	TP53_ENST(	ENST00000413465	p.Q192*	c.574C>T	Verified	CCT	CAG	CAT	1	C	T
TCGA-24-2280	TP53_ENST(	ENST00000413465	p.Q192*	c.574C>T	Verified	CCT	CAG	CAT	1	C	T
TCGA-29-1775	TP53_ENST(	ENST00000413465	p.Q104*	c.310C>T	Unverified	TAC	CAG	GGC	1	C	T
TCGA-13-1510	TP53_ENST(	ENST00000414315	p.R81*	c.241C>T	Verified	TTT	CGA	CAT	1	C	T
TCGA-24-1431	TP53_ENST(	ENST00000414315	p.Q60*	c.178C>T	Verified	CCT	CAG	CAT	1	C	T
TCGA-24-2280	TP53_ENST(	ENST00000414315	p.Q60*	c.178C>T	Verified	CCT	CAG	CAT	1	C	T
TCGA-13-1510	TP53_ENST(	ENST00000545858	p.R120*	c.358C>T	Verified	TTT	CGA	CAT	1	C	T
TCGA-24-1431	TP53_ENST(	ENST00000545858	p.Q99*	c.295C>T	Verified	CCT	CAG	CAT	1	C	T
TCGA-24-2280	TP53_ENST(	ENST00000545858	p.Q99*	c.295C>T	Verified	CCT	CAG	CAT	1	C	T
TCGA-04-1655	TPBG	ENST00000369750	p.H238H	c.714C>T	Unverified	AGG	CAC	CTG	3	C	T
TCGA-23-1032	TPCN1	ENST00000335509	p.H662Y	c.1984C>T	Unverified	TCC	CAC	TGG	1	C	T
TCGA-29-1769	TPCN2	ENST00000294309	p.Q83*	c.247C>T	Unverified	TGC	CAA	CGG	1	C	T

TCGA-29-1701	TPI1	ENST00000396705	p.H186H	c.558C>T	Unverified	gta	cac	gag	3	C	T
TCGA-29-1701	TPI1_ENST0	ENST00000229270	p.H223H	c.669C>T	Unverified	GTA	CAC	GAG	3	C	T
TCGA-29-1777	TPRG1L	ENST00000378344	p.L237L	c.709C>T	Unverified	GTG	CTG	ATC	1	C	T
TCGA-09-0369	TRAF2	ENST00000536468	p.L268F	c.802C>T	Verified	gag	ctc	ctg	1	C	T
TCGA-09-0369	TRAF2	ENST00000536468	p.L268F	c.802C>T	Verified	gag	ctc	ctg	1	C	T
TCGA-04-1362	TRAPPC8	ENST00000283351	p.L913L	c.2737C>T	Verified	CCA	CTG	TTG	1	C	T
TCGA-61-2095	TRAPPC8	ENST00000283351	p.S237L	c.710C>T	Verified	GCA	TCA	GAT	2	C	T
TCGA-61-2102	TRAPPC8	ENST00000283351	p.S1319L	c.3956C>T	Verified	GAA	TCA	TTT	2	C	T
TCGA-29-1705	TRDMT1	ENST00000377799	p.P173S	c.517C>T	Unverified	gag	cca	tta	1	C	T
TCGA-24-1469	TREML2	ENST00000373108	p.P62L	c.185C>T	Verified	GCC	CCA	GCC	2	C	T
TCGA-30-1855	TRHDE	ENST00000261180	p.H271Y	c.811C>T	Unverified	ACA	CAT	GCC	1	C	T
TCGA-20-0991	TRHDE	ENST00000261180	p.D500D	c.1500C>T	Verified	CTG	GAC	GGT	3	C	T
TCGA-20-1687	TRIM10	ENST00000449742	p.C31C	c.93C>T	Unverified	GAC	TGC	GGC	3	C	T
TCGA-20-1687	TRIM10_ENS	ENST00000449742	p.C31C	c.93C>T	Unverified	GAC	TGC	GGC	3	C	T
TCGA-04-1347	TRIM13	ENST00000420995	p.Q195*	c.583C>T	Verified	gat	caa	aag	1	C	T
TCGA-24-2262	TRIM3	ENST00000525074	p.G249G	c.747C>T	Verified	atc	ggc	agt	3	C	T
TCGA-29-1769	TRIM32	ENST00000450136	p.R238R	c.714C>T	Unverified	TCT	CGC	TGT	3	C	T
TCGA-24-1604	TRIM33	NM_015906	p.P603S	c.1807C>T	Verified	ata	ccc	agg	1	C	T
TCGA-25-1326	TRIM47	ENST00000254816	p.C377C	c.1131C>T	Verified	gcc	tgc	gtc	3	C	T
TCGA-29-1695	TRIM60	ENST00000512596	p.A398V	c.1193C>T	Unverified	gtt	gcg	tca	2	C	T
TCGA-29-1693	TRIM7_ENST	ENST00000393319	p.H12Y	c.34C>T	Unverified	CTC	CAT	GTT	1	C	T
TCGA-13-0793	TRIM71	ENST00000383763	p.G491G	c.1473C>T	Verified	ACA	GGC	GAT	3	C	T
TCGA-61-1907	TRIML1	ENST00000332517	p.Q438*	c.1312C>T	Unverified	TTC	CAA	GAG	1	C	T
TCGA-29-1764	TRIOBP_ENS	ENST00000406386	p.R727W	c.2179C>T	Unverified	CGA	CGG	GAC	1	C	T
TCGA-24-1470	TRIP11	ENST00000267622	p.L546F	c.1636C>T	Verified	CAA	CTT	GAA	1	C	T
TCGA-24-1469	TRIP12	ENST00000283943	p.G1610G	c.4830C>T	Verified	CTC	GGC	AGC	3	C	T
TCGA-25-2398	TRMT1	ENST00000357720	p.T459I	c.1376C>T	Verified	AAC	ACA	CCA	2	C	T
TCGA-61-1733	TRMT2A	ENST00000252136	p.L105F	c.313C>T	Unverified	AAA	CTC	TTT	1	C	T
TCGA-61-1733	TRMT2A	ENST00000252136	p.L105F	c.313C>T	Unverified	AAA	CTC	TTT	1	C	T
TCGA-24-1604	TRMT2B	ENST00000545398	p.Y280Y	c.840C>T	Verified	ctt	tac	ttc	3	C	T
TCGA-24-1845	TRPA1	NM_007332.1	p.N687N	c.2061C>T	Unverified	ctc	aac	gca	3	C	T
TCGA-13-1499	TRPC1	ENST00000273482	p.P491S	c.1471C>T	Verified	GGT	CCA	TTA	1	C	T
TCGA-29-1761	TRPM3	ENST00000377106	p.D587D	c.1761C>T	Unverified	CAG	GAC	GAA	3	C	T
TCGA-29-1761	TRPM3_ENS	ENST00000377110	p.D715D	c.2145C>T	Unverified	CAG	GAC	GAA	3	C	T

TCGA-29-1761	TRPM3_ENS	ENST00000423814	p.D719D	c.2157C>T	Unverified	CAG	GAC	GAA	3	C	T
TCGA-23-1114	TRPM4	ENST00000252826	p.R337C	c.1009C>T	Unverified	AGG	CGT	TTC	1	C	T
TCGA-04-1651	TRPM8	ENST00000324695	p.D544D	c.1632C>T	Unverified	CGG	GAC	GAG	3	C	T
TCGA-04-1651	TRPM8	ENST00000324695	p.D544D	c.1632C>T	Unverified	CGG	GAC	GAG	3	C	T
TCGA-23-1124	TRPV3	ENST00000576742	p.A560V	c.1679C>T	Verified	TGG	GCG	AAC	2	C	T
TCGA-24-2267	TRPV3	ENST00000576742	p.Y544Y	c.1632C>T	Verified	GCC	TAC	AAA	3	C	T
TCGA-61-1907	TSHB	ENST00000256592	p.S137F	c.410C>T	Unverified	TTT	TCT	GTC	2	C	T
TCGA-25-2391	TSNARE1	ENST00000307180	p.N267N	c.801C>T	Unverified	GCC	AAC	GTC	3	C	T
TCGA-25-2392	TSPAN14	ENST00000372156	p.D121D	c.363C>T	Verified	AGG	GAC	CGG	3	C	T
TCGA-24-1847	TSPAN16	ENST00000316737	p.A41V	c.122C>T	Unverified	GGG	GCC	TCT	2	C	T
TCGA-25-1326	TSPAN16	ENST00000316737	p.L47L	c.141C>T	Unverified	GTC	CTC	GGG	3	C	T
TCGA-04-1347	TSSK1B	ENST00000390666	p.G189G	c.567C>T	Verified	CAG	GGC	ATT	3	C	T
TCGA-23-1123	TTC15	ENST00000382110	p.R543C	c.1627C>T	Verified	TCA	CGT	CTG	1	C	T
TCGA-23-1123	TTC15	ENST00000382110	p.R543C	c.1627C>T	Verified	TCA	CGT	CTG	1	C	T
TCGA-29-1775	TTC23L	ENST00000505624	p.A232V	c.695C>T	Unverified	CTA	GCT	CTG	2	C	T
TCGA-23-1031	TTC26	ENST00000464848	p.V150V	c.450C>T	Verified	GAT	GTC	ACA	3	C	T
TCGA-29-1690	TTC9	ENST00000256367	p.R169W	c.505C>T	Unverified	TAC	CGG	TCT	1	C	T
TCGA-24-1849	TTLL12	ENST00000216129	p.P388P	c.1164C>T	Unverified	CTG	CCC	CGA	3	C	T
TCGA-24-1474	TTLL2	ENST00000239587	p.T323M	c.968C>T	Verified	TGG	ACG	CTC	2	C	T
TCGA-13-0760	TTLL5	ENST00000298832	p.P926S	c.2776C>T	Verified	ATC	CCC	AGC	1	C	T
TCGA-29-1768	TTLL8	ENST00000266182	p.R61W	c.181C>T	Unverified	GCC	CGG	GTC	1	C	T
TCGA-29-1768	TTLL8_ENST	ENST00000433387	p.R97W	c.289C>T	Unverified	GCC	CGG	GTC	1	C	T
TCGA-23-1032	TTLL9	ENST00000375938	p.C416C	c.1248C>T	Verified	GGC	TGC	GTC	3	C	T
TCGA-04-1331	TTN	NM_003319	p.P10772S	c.32314C>T	Verified	act	cca	gtt	1	C	T
TCGA-13-0792	TTN	NM_003319	p.R8634C	c.25900C>T	Verified	ggt	cgc	cct	1	C	T
TCGA-29-1775	TTN	NM_003319	p.R14835W	c.44503C>T	Unverified	ttc	cgg	gtg	1	C	T
TCGA-30-1856	TTN	NM_003319	p.R6018W	c.18052C>T	Unverified	gga	cgg	aag	1	C	T
TCGA-13-1505	TTN	NM_003319	p.R25529C	c.76585C>T	Verified	aag	cgc	atc	1	C	T
TCGA-04-1542	TTN	NM_003319	p.P26166L	c.78497C>T	Verified	tca	cca	cca	2	C	T
TCGA-24-1469	TTN	NM_003319	p.S5398L	c.16193C>T	Verified	cat	tca	atg	2	C	T
TCGA-13-0760	TTN	NM_003319	p.D6342D	c.19026C>T	Verified	gat	gac	gct	3	C	T
TCGA-20-1687	TTN	NM_003319	p.R7163R	c.21489C>T	Unverified	tac	cgc	gtg	3	C	T
TCGA-25-1318	TTN	NM_003319	p.N24809N	c.74427C>T	Verified	aga	aac	atc	3	C	T
TCGA-25-1318	TTN	NM_003319	p.N24809N	c.74427C>T	Verified	aga	aac	atc	3	C	T

TCGA-29-1761	TTN	NM_003319	p.D5481D	c.16443C>T	Unverified	caa	gac	gaa	3	C	T
TCGA-29-1775	TTN_ENST0(	ENST00000342175	p.R15027W	c.45079C>T	Unverified	TTC	CGG	GTG	1	C	T
TCGA-30-1856	TTN_ENST0(	ENST00000342175	p.R6210W	c.18628C>T	Unverified	GGA	CGG	AAG	1	C	T
TCGA-20-1687	TTN_ENST0(	ENST00000342175	p.R7355R	c.22065C>T	Unverified	TAC	CGC	GTG	3	C	T
TCGA-29-1761	TTN_ENST0(	ENST00000342175	p.D5673D	c.17019C>T	Unverified	CAA	GAC	GAA	3	C	T
TCGA-29-1775	TTN_ENST0(	ENST00000342992	p.R21332W	c.63994C>T	Unverified	TTC	CGG	GTG	1	C	T
TCGA-30-1856	TTN_ENST0(	ENST00000342992	p.R12515W	c.37543C>T	Unverified	GGA	CGG	AAG	1	C	T
TCGA-20-1687	TTN_ENST0(	ENST00000342992	p.R13660R	c.40980C>T	Unverified	TAC	CGC	GTG	3	C	T
TCGA-29-1761	TTN_ENST0(	ENST00000342992	p.D11978D	c.35934C>T	Unverified	CAA	GAC	GAA	3	C	T
TCGA-04-1331	TTN_ENST0(	ENST00000356127	p.P17267S	c.51799C>T	Verified	ACT	CCA	GTT	1	C	T
TCGA-13-0792	TTN_ENST0(	ENST00000356127	p.R15131C	c.45391C>T	Verified	GGT	CGC	CCT	1	C	T
TCGA-29-1775	TTN_ENST0(	ENST00000356127	p.R21330W	c.63988C>T	Unverified	TTC	CGG	GTG	1	C	T
TCGA-30-1856	TTN_ENST0(	ENST00000356127	p.R12515W	c.37543C>T	Unverified	GGA	CGG	AAG	1	C	T
TCGA-13-1505	TTN_ENST0(	ENST00000356127	p.R32024C	c.96070C>T	Verified	AAG	CGC	ATC	1	C	T
TCGA-04-1542	TTN_ENST0(	ENST00000356127	p.P32661L	c.97982C>T	Verified	TCA	CCA	CCA	2	C	T
TCGA-09-2050	TTN_ENST0(	ENST00000356127	p.T3676M	c.11027C>T	Verified	GTT	ACG	TGG	2	C	T
TCGA-13-0920	TTN_ENST0(	ENST00000356127	p.P16144L	c.48431C>T	Verified	TTC	CCG	ACA	2	C	T
TCGA-23-1032	TTN_ENST0(	ENST00000356127	p.A6451V	c.19352C>T	Verified	ACA	GCG	TTG	2	C	T
TCGA-24-1469	TTN_ENST0(	ENST00000356127	p.S11895L	c.35684C>T	Verified	CAT	TCA	ATG	2	C	T
TCGA-13-0760	TTN_ENST0(	ENST00000356127	p.D12839D	c.38517C>T	Verified	GAT	GAC	GCT	3	C	T
TCGA-20-1687	TTN_ENST0(	ENST00000356127	p.R13660R	c.40980C>T	Unverified	TAC	CGC	GTG	3	C	T
TCGA-24-1464	TTN_ENST0(	ENST00000356127	p.F7526F	c.22578C>T	Verified	TGG	TTC	AAA	3	C	T
TCGA-25-1318	TTN_ENST0(	ENST00000356127	p.N31304N	c.93912C>T	Verified	AGA	AAC	ATC	3	C	T
TCGA-25-1318	TTN_ENST0(	ENST00000356127	p.N31304N	c.93912C>T	Verified	AGA	AAC	ATC	3	C	T
TCGA-29-1761	TTN_ENST0(	ENST00000356127	p.D11978D	c.35934C>T	Unverified	CAA	GAC	GAA	3	C	T
TCGA-29-1775	TTN_ENST0(	ENST00000359218	p.R14960W	c.44878C>T	Unverified	TTC	CGG	GTG	1	C	T
TCGA-30-1856	TTN_ENST0(	ENST00000359218	p.R6143W	c.18427C>T	Unverified	GGA	CGG	AAG	1	C	T
TCGA-20-1687	TTN_ENST0(	ENST00000359218	p.R7288R	c.21864C>T	Unverified	TAC	CGC	GTG	3	C	T
TCGA-29-1761	TTN_ENST0(	ENST00000359218	p.D5606D	c.16818C>T	Unverified	CAA	GAC	GAA	3	C	T
TCGA-61-2094	TTPAL	ENST00000262605	p.D77D	c.231C>T	Unverified	CTC	GAC	GAT	3	C	T
TCGA-13-0913	TTYH2	ENST00000269346	p.H359H	c.1077C>T	Verified	CTT	CAC	CAG	3	C	T
TCGA-29-1785	TUB	ENST00000305253	p.R419C	c.1255C>T	Unverified	TTA	CGT	CAG	1	C	T
TCGA-29-1785	TUB	ENST00000305253	p.R419C	c.1255C>T	Unverified	TTA	CGT	CAG	1	C	T
TCGA-04-1652	TUBA3C	ENST00000400113	p.T94T	c.282C>T	Unverified	ATC	ACC	GGG	3	C	T

TCGA-04-1652	TUBA3C	ENST00000400113	p.T94T	c.282C>T	Unverified	ATC	ACC	GGG	3	C	T
TCGA-04-1343	TUBB	ENST00000327892	p.G140G	c.420C>T	Verified	CTG	GGC	GGG	3	C	T
TCGA-24-2024	TUBGCP6	ENST00000248846	p.N1112N	c.3336C>T	Verified	TCC	AAC	GCC	3	C	T
TCGA-13-1505	TXNDC3	ENST00000199447	p.A302V	c.905C>T	Unverified	GAT	GCT	TTC	2	C	T
TCGA-25-2042	TXNRD2	ENST00000400521	p.G83G	c.249C>T	Unverified	CTC	GGC	GGC	3	C	T
TCGA-13-1510	TYK2	ENST00000525621	p.P466P	c.1398C>T	Unverified	CGG	CCC	GAG	3	C	T
TCGA-20-1683	TYSND1	ENST00000287078	p.L526L	c.1576C>T	Unverified	GCC	CTG	CAG	1	C	T
TCGA-24-2035	UBASH3A	ENST00000319294	p.A546V	c.1637C>T	Verified	CCC	GCG	TTT	2	C	T
TCGA-13-0893	UBQLN3	ENST00000311659	p.R218W	c.652C>T	Verified	ATG	CGG	CAG	1	C	T
TCGA-24-2267	UBQLN3	ENST00000311659	p.T439I	c.1316C>T	Verified	AGC	ACA	AAC	2	C	T
TCGA-23-1032	UGT1A7	ENST00000373426	p.R254*	c.760C>T	Verified	ttg	cga	act	1	C	T
TCGA-23-1124	UGT1A8	ENST00000373450	p.H53Y	c.157C>T	Verified	GGG	CAT	GAG	1	C	T
TCGA-04-1342	UGT2B17	ENST00000317746	p.T31I	c.92C>T	Unverified	CCC	ACA	GAA	2	C	T
TCGA-29-1761	UGT2B4	ENST00000305107	p.L498L	c.1492C>T	Unverified	CTG	CTG	GCC	1	C	T
TCGA-61-2095	UHRF1BP1L	ENST00000279907	p.H78Y	c.232C>T	Unverified	ACA	CAT	CCC	1	C	T
TCGA-13-1512	UMODL1	ENST00000400424	p.R231W	c.691C>T	Verified	CCA	CGG	AAG	1	C	T
TCGA-29-1703	UNC13A	ENST00000428389	p.S883L	c.2648C>T	Unverified	GTG	TCG	GGT	2	C	T
TCGA-30-1857	UNC13A	ENST00000428389	p.D1431D	c.4293C>T	Unverified	CAG	GAC	GCG	3	C	T
TCGA-29-1703	UNC13A_EN	ENST00000519716	p.S795L	c.2384C>T	Unverified	GTG	TCG	GGT	2	C	T
TCGA-30-1857	UNC13A_EN	ENST00000519716	p.D1343D	c.4029C>T	Unverified	CAG	GAC	GCG	3	C	T
TCGA-09-2044	UNC13B	ENST00000378495	p.H281Y	c.841C>T	Verified	TAT	CAC	GAA	1	C	T
TCGA-61-1904	UNC13C	ENST00000260323	p.R375*	c.1123C>T	Unverified	CCT	CGA	CCC	1	C	T
TCGA-61-1904	UNC13C_EN	ENST00000260323	p.R375*	c.1123C>T	Unverified	CCT	CGA	CCC	1	C	T
TCGA-04-1347	UNC45A	ENST00000418476	p.T417M	c.1250C>T	Verified	CAG	ACG	GTG	2	C	T
TCGA-24-2267	UNC45A	ENST00000418476	p.I283I	c.849C>T	Unverified	GCC	ATC	ATT	3	C	T
TCGA-04-1338	UNC5B	ENST00000335350	p.R271W	c.811C>T	Verified	ACC	CGG	ACC	1	C	T
TCGA-13-0792	UNC5C	ENST00000453304	p.T506I	c.1517C>T	Verified	ATG	ACC	CAG	2	C	T
TCGA-30-1718	UPB1	ENST00000326010	p.L133F	c.397C>T	Unverified	AAG	CTT	CCT	1	C	T
TCGA-13-0885	UPB1	ENST00000326010	p.N206N	c.618C>T	Verified	TTC	AAC	GAG	3	C	T
TCGA-13-0760	UPF1	ENST00000262803	p.D298D	c.894C>T	Verified	GAG	GAC	GCC	3	C	T
TCGA-13-1505	UPF3B	ENST00000276201	p.R368W	c.1102C>T	Verified	aag	cgg	caa	1	C	T
TCGA-23-1031	UPP1	ENST00000331803	p.R285C	c.853C>T	Unverified	CCT	CGC	AAT	1	C	T
TCGA-24-1563	UQCR10	ENST00000332801	p.A3A	c.9C>T	Verified	GCG	GCC	GCG	3	C	T
TCGA-24-1464	URP2	ENST00000345728	p.R538C	c.1612C>T	Verified	CTG	CGC	TTC	1	C	T

TCGA-13-0886	USF1	ENST00000368020	p.S145L	c.434C>T	Verified	GGC	TCA	GAG	2	C	T
TCGA-23-1021	USH2A	ENST00000307340	p.R189C	c.565C>T	Verified	TAT	CGC	ACA	1	C	T
TCGA-23-1117	USH2A	ENST00000307340	p.R737*	c.2209C>T	Verified	CTC	CGA	AGC	1	C	T
TCGA-23-1117	USH2A	ENST00000307340	p.R737*	c.2209C>T	Verified	CTC	CGA	AGC	1	C	T
TCGA-29-1698	USH2A	ENST00000307340	p.R5116C	c.15346C>T	Unverified	ATC	CGC	AGC	1	C	T
TCGA-13-1497	USP19	XM_496642.1	p.P362P	c.1086C>T	Verified	gag	ccc	gag	3	C	T
TCGA-25-2401	USP20_ENS	ENST00000372429	p.P500S	c.1498C>T	Verified	AAG	CCA	GGC	1	C	T
TCGA-24-1435	USP21	ENST00000368002	p.A125V	c.374C>T	Verified	ATT	GCC	TTG	2	C	T
TCGA-13-0755	USP21	ENST00000368002	p.F222F	c.666C>T	Verified	TGC	TTC	CTG	3	C	T
TCGA-29-1776	USP32	ENST00000300896	p.R985*	c.2953C>T	Unverified	gta	cga	ctc	1	C	T
TCGA-24-1844	USP34	ENST00000398571	p.H3506Y	c.10516C>T	Unverified	GGC	CAT	TCC	1	C	T
TCGA-25-1313	USP34	ENST00000398571	p.P1272S	c.3814C>T	Verified	TTT	CCT	GGA	1	C	T
TCGA-61-1914	USP34	ENST00000398571	p.Q80*	c.238C>T	Unverified	GAC	CAG	CTT	1	C	T
TCGA-23-1124	USP34	ENST00000398571	p.S1433S	c.4299C>T	Verified	AAG	AGC	GCC	3	C	T
TCGA-24-2289	USP4	ENST00000265560	p.P480S	c.1438C>T	Unverified	ctg	cca	ctg	1	C	T
TCGA-13-0904	USP49	ENST00000373006	p.L496F	c.1486C>T	Verified	TTG	CTC	ACT	1	C	T
TCGA-24-1469	USP54_ENS	ENST00000408019	p.A147V	c.440C>T	Verified	ACT	GCC	CAA	2	C	T
TCGA-61-1725	USP7	ENST00000344836	p.H501Y	c.1501C>T	Unverified	GGT	CAC	GAT	1	C	T
TCGA-09-2049	USP7	ENST00000344836	p.T190I	c.569C>T	Verified	GTT	ACC	TTT	2	C	T
TCGA-61-2009	UTP14C	ENST00000521776	p.S462F	c.1385C>T	Verified	CTA	TCT	CAG	2	C	T
TCGA-25-2393	UTP14C	ENST00000521776	p.S456S	c.1368C>T	Verified	CTG	TCC	GAA	3	C	T
TCGA-29-1703	UTRN	ENST00000367545	p.H416H	c.1248C>T	Unverified	CTG	CAC	GAT	3	C	T
TCGA-13-1498	VARS	ENST00000375663	p.P1232L	c.3695C>T	Verified	GTG	CCG	CTC	2	C	T
TCGA-09-0369	VAV2	ENST00000406606	p.A111V	c.332C>T	Verified	TCC	GCG	GTG	2	C	T
TCGA-09-0369	VAV2	ENST00000406606	p.A111V	c.332C>T	Verified	TCC	GCG	GTG	2	C	T
TCGA-61-1740	VEPH1	ENST00000392832	p.F791F	c.2373C>T	Unverified	ATC	TTC	ACA	3	C	T
TCGA-13-0762	VIL1	ENST00000248444	p.R495C	c.1483C>T	Verified	GGA	CGC	ATG	1	C	T
TCGA-13-0762	VIL1	ENST00000248444	p.R495C	c.1483C>T	Verified	GGA	CGC	ATG	1	C	T
TCGA-29-1766	VIL1	ENST00000248444	p.I174I	c.522C>T	Unverified	CTT	ATC	ATC	3	C	T
TCGA-24-2280	VLDLR	ENST00000382100	p.I451I	c.1353C>T	Unverified	GAC	ATC	AGG	3	C	T
TCGA-09-2049	VPRBP	ENST00000273612	p.H25Y	c.73C>T	Verified	GAA	CAT	GGC	1	C	T
TCGA-24-1849	VPRBP	ENST00000273612	p.I778I	c.2334C>T	Unverified	CAG	ATC	ATC	3	C	T
TCGA-24-1422	VPS13B	ENST00000358544	p.R3530W	c.10588C>T	Verified	gct	cgg	tta	1	C	T
TCGA-30-1857	VPS13B	ENST00000358544	p.Q323*	c.967C>T	Unverified	atg	caa	tat	1	C	T

TCGA-29-1703	VPS13B	ENST00000358544	p.D3332D	c.9996C>T	Unverified	att	gac	atc	3	C	T
TCGA-30-1857	VPS13B_ENS	ENST00000357162	p.Q323*	c.967C>T	Unverified	ATG	CAA	TAT	1	C	T
TCGA-29-1703	VPS13B_ENS	ENST00000357162	p.D3307D	c.9921C>T	Unverified	ATT	GAC	ATC	3	C	T
TCGA-61-1914	VPS13C	ENST00000261517	p.V1033V	c.3099C>T	Unverified	CTT	GTC	GCT	3	C	T
TCGA-61-1914	VPS13C_ENS	ENST00000395896	p.V1033V	c.3099C>T	Unverified	CTT	GTC	GCT	3	C	T
TCGA-61-2095	VPS13D	ENST00000358136	p.F4044F	c.12132C>T	Verified	GAG	TTC	ATC	3	C	T
TCGA-29-1698	VPS53	ENST00000291074	p.H108H	c.324C>T	Unverified	GAT	CAC	GCC	3	C	T
TCGA-29-1698	VPS53_ENST	ENST00000437048	p.H137H	c.411C>T	Unverified	GAT	CAC	GCC	3	C	T
TCGA-25-2400	VRTN	ENST00000256362	p.R105W	c.313C>T	Unverified	CTG	CGG	GCC	1	C	T
TCGA-13-0760	VSIG2	ENST00000326621	p.F261F	c.783C>T	Verified	GCG	TTC	TGC	3	C	T
TCGA-29-1769	VSIG7	ENST00000338912	p.S94S	c.282C>T	Unverified	ACA	TCC	ATG	3	C	T
TCGA-04-1356	VWF	ENST00000261405	p.A1344A	c.4032C>T	Verified	ATT	GCC	AGC	3	C	T
TCGA-13-0904	VWF	ENST00000261405	p.T286T	c.858C>T	Verified	TGG	ACC	GAC	3	C	T
TCGA-23-1117	VWF	ENST00000261405	p.D533D	c.1599C>T	Verified	GGC	GAC	GAC	3	C	T
TCGA-23-1117	VWF	ENST00000261405	p.D533D	c.1599C>T	Verified	GGC	GAC	GAC	3	C	T
TCGA-29-1761	VWF	ENST00000261405	p.S2285S	c.6855C>T	Unverified	CTC	AGC	GGG	3	C	T
TCGA-25-2400	WAS	ENST00000376701	p.P417S	c.1249C>T	Verified	cct	cct	gct	1	C	T
TCGA-09-2051	WBSCR17	ENST00000333538	p.S171S	c.513C>T	Unverified	CGG	TCC	GTG	3	C	T
TCGA-24-2289	WDHD1	ENST00000360586	p.H667Y	c.1999C>T	Unverified	GAG	CAC	TGC	1	C	T
TCGA-61-1740	WDR13	ENST00000376729	p.D464D	c.1392C>T	Unverified	TGC	GAC	GAG	3	C	T
TCGA-61-1740	WDR13_ENS	ENST00000218056	p.D464D	c.1392C>T	Unverified	TGC	GAC	GAG	3	C	T
TCGA-23-1032	WDR16	ENST00000352665	p.N394N	c.1182C>T	Verified	TGG	AAC	GAC	3	C	T
TCGA-29-1763	WDR6	ENST00000315574	p.R1106C	c.3316C>T	Unverified	CAC	CGT	TGT	1	C	T
TCGA-24-2289	WDR6	ENST00000315574	p.T226I	c.677C>T	Unverified	GCT	ACA	GCT	2	C	T
TCGA-13-0755	WDR61	ENST00000267973	p.L182F	c.544C>T	Verified	AAA	CTT	CTG	1	C	T
TCGA-20-1683	WDR65	ENST00000528956	p.L659L	c.1975C>T	Unverified	CTG	CTA	CGT	1	C	T
TCGA-29-1775	WFDC12	ENST00000372785	p.C34C	c.102C>T	Unverified	GTT	TGC	CCA	3	C	T
TCGA-13-0913	WFDC5	ENST00000372789	p.D47D	c.141C>T	Verified	CCT	GAC	CAG	3	C	T
TCGA-13-0913	WFIKKN2	ENST00000311378	p.L230F	c.688C>T	Verified	TTC	CTC	TGT	1	C	T
TCGA-09-2050	WIPF3	ENST00000409290	p.A48V	c.143C>T	Verified	AGT	GCG	CTG	2	C	T
TCGA-13-0760	WNK1	NM_018979	p.T870I	c.2609C>T	Verified	att	act	cag	2	C	T
TCGA-23-1117	WNK1	NM_018979	p.A1351V	c.4052C>T	Verified	gca	gca	gcc	2	C	T
TCGA-23-1117	WNK1	NM_018979	p.A1351V	c.4052C>T	Verified	gca	gca	gcc	2	C	T
TCGA-13-0913	WNK1	NM_018979	p.Y422Y	c.1266C>T	Verified	cct	tac	tcg	3	C	T

TCGA-30-1891	WNK3	ENST00000354646	p.T489M	c.1466C>T	Unverified	GTG	ACG	CCA	2	C	T
TCGA-20-1687	WNT2B	ENST00000369684	p.R293C	c.877C>T	Unverified	CGC	CGT	GCC	1	C	T
TCGA-13-1507	WNT4	ENST00000290167	p.R107W	c.319C>T	Verified	ACT	CGG	GAG	1	C	T
TCGA-13-0760	WNT9A	ENST00000272164	p.R186W	c.556C>T	Unverified	AGA	CGG	TCA	1	C	T
TCGA-30-1718	WSCD1	ENST00000317744	p.A424V	c.1271C>T	Unverified	TCA	GCC	ATC	2	C	T
TCGA-23-1122	WWC2	ENST00000403733	p.R795*	c.2383C>T	Verified	CAC	CGA	AGG	1	C	T
TCGA-24-1469	WWC2	ENST00000403733	p.S227F	c.680C>T	Verified	AAA	TCT	ATC	2	C	T
TCGA-04-1362	WWC3	NM_015691.2	p.A53A	c.159C>T	Verified	ctg	gcc	cag	3	C	T
TCGA-23-1124	XCR1	ENST00000309285	p.P240P	c.720C>T	Verified	GGT	CCC	TAC	3	C	T
TCGA-29-1762	XIRP1	ENST00000340369	p.L1219L	c.3657C>T	Unverified	GGC	CTC	CAA	3	C	T
TCGA-61-1907	XIRP2	ENST00000295237	p.F736F	c.2208C>T	Unverified	TGT	TTC	GAA	3	C	T
TCGA-13-0795	XKR7	ENST00000217299	p.T497T	c.1491C>T	Verified	CCC	ACC	CCA	3	C	T
TCGA-23-1123	XPO1	ENST00000401558	p.L255L	c.763C>T	Verified	ttc	ctg	aat	1	C	T
TCGA-23-1123	XPO1	ENST00000401558	p.L255L	c.763C>T	Verified	ttc	ctg	aat	1	C	T
TCGA-23-1031	XPO5	ENST00000265351	p.P1136P	c.3408C>T	Unverified	AAC	CCC	TCC	3	C	T
TCGA-13-0793	XPO7	NM_015024.2	p.R812C	c.2434C>T	Verified	aat	cgc	atc	1	C	T
TCGA-29-1761	XRCC6	ENST00000360079	p.T302I	c.905C>T	Unverified	CGG	ACC	TTT	2	C	T
TCGA-13-1496	XRCC6BP1	ENST00000300145	p.C88C	c.264C>T	Verified	TCT	TGC	GAA	3	C	T
TCGA-13-1507	YBX1	ENST00000321358	p.P316P	c.948C>T	Verified	GCT	CCC	GAG	3	C	T
TCGA-23-1124	YES1	ENST00000584307	p.Q495*	c.1483C>T	Verified	CCT	CAG	GGC	1	C	T
TCGA-29-1691	YLPM1	ENST00000238571	p.S1800S	c.5400C>T	Unverified	GAA	AGC	GAA	3	C	T
TCGA-29-1691	YLPM1_ENS	ENST00000325680	p.S2035S	c.6105C>T	Unverified	GAA	AGC	GAA	3	C	T
TCGA-23-1117	YTHDF2	ENST00000373812	p.R355W	c.1063C>T	Verified	CCT	CGG	AAC	1	C	T
TCGA-23-1117	YTHDF2	ENST00000373812	p.R355W	c.1063C>T	Verified	CCT	CGG	AAC	1	C	T
TCGA-30-1891	YWHAE	ENST00000264335	p.L44F	c.130C>T	Unverified	AAC	CTC	CTA	1	C	T
TCGA-61-1904	ZAN	ENST00000348028	p.L1782L	c.5344C>T	Unverified	GCC	CTG	TGC	1	C	T
TCGA-61-1904	ZAN_ENST0(	ENST00000542585	p.L1782L	c.5344C>T	Unverified	GCC	CTG	TGC	1	C	T
TCGA-61-1904	ZAN_ENST0(	ENST00000546213	p.L359L	c.1075C>T	Unverified	GCC	CTG	TGC	1	C	T
TCGA-04-1343	ZBTB22	ENST00000418724	p.Q50*	c.148C>T	Verified	CAG	CAG	CGT	1	C	T
TCGA-29-1763	ZBTB24	ENST00000230122	p.F415F	c.1245C>T	Unverified	AAA	TTC	ATG	3	C	T
TCGA-24-2280	ZBTB25	ENST00000394715	p.R94C	c.280C>T	Unverified	AGT	CGT	TTG	1	C	T
TCGA-09-2050	ZBTB4	ENST00000380599	p.D639D	c.1917C>T	Verified	GAG	GAC	GAA	3	C	T
TCGA-04-1347	ZBTB43	ENST00000373457	p.Y454Y	c.1362C>T	Verified	TCC	TAC	GAA	3	C	T
TCGA-29-1775	ZC3H4	ENST00000253048	p.S76S	c.228C>T	Unverified	ACC	TCC	GGA	3	C	T

TCGA-23-1123	ZC4H2	ENST00000374839	p.S40L	c.119C>T	Verified	gag	tca	gag	2	C	T
TCGA-23-1123	ZC4H2	ENST00000374839	p.S40L	c.119C>T	Verified	gag	tca	gag	2	C	T
TCGA-13-0893	ZCCHC16	ENST00000340433	p.P146L	c.437C>T	Verified	AAT	CCT	CTG	2	C	T
TCGA-09-1665	ZCCHC9	ENST00000380199	p.P258L	c.773C>T	Verified	AAA	CCG	CAA	2	C	T
TCGA-09-2049	ZDHHC19	ENST00000296326	p.A293V	c.878C>T	Verified	CCA	GCC	CCA	2	C	T
TCGA-13-1497	ZDHHC19	ENST00000296326	p.P16L	c.47C>T	Verified	CAT	CCC	CTG	2	C	T
TCGA-29-1702	ZEB1	ENST00000320985	p.C772C	c.2316C>T	Unverified	TCT	TGC	GCA	3	C	T
TCGA-24-1846	ZFAT	ENST00000398946	p.S664L	c.1991C>T	Unverified	AGG	TCA	AAC	2	C	T
TCGA-24-1846	ZFAT_ENSTC	ENST00000377838	p.S676L	c.2027C>T	Unverified	AGG	TCA	AAC	2	C	T
TCGA-23-1809	ZFHX3	ENST00000268489	p.R1396C	c.4186C>T	Unverified	GAT	CGC	CAT	1	C	T
TCGA-13-1488	ZFHX4	ENST00000521891	p.G3000G	c.9000C>T	Verified	CAA	GGC	GGA	3	C	T
TCGA-24-1843	ZFP14	ENST00000270001	p.C77C	c.231C>T	Unverified	TAC	TGC	CCT	3	C	T
TCGA-24-1850	ZFP36L1	ENST00000336440	p.I10I	c.30C>T	Unverified	ACC	ATC	TTC	3	C	T
TCGA-30-1857	ZFYVE26	ENST00000347230	p.L1027F	c.3079C>T	Unverified	GTT	CTT	CAG	1	C	T
TCGA-23-2077	ZFYVE26	ENST00000347230	p.A539V	c.1616C>T	Verified	CCA	GCG	AAT	2	C	T
TCGA-13-0923	ZIC3	ENST00000287538	p.D385D	c.1155C>T	Verified	tcg	gac	aag	3	C	T
TCGA-04-1367	ZIM2	ENST00000221722	p.R68W	c.202C>T	Verified	CCG	CGG	GAT	1	C	T
TCGA-13-1488	ZIM2	ENST00000221722	p.R505*	c.1513C>T	Verified	GGC	CGA	CCC	1	C	T
TCGA-24-1850	ZKSCAN4	ENST00000377294	p.Q311*	c.931C>T	Unverified	AAG	CAG	AAA	1	C	T
TCGA-23-1123	ZMAT1_ENS	ENST00000372782	p.L134F	c.400C>T	Verified	CCA	CTT	ATT	1	C	T
TCGA-23-1123	ZMAT1_ENS	ENST00000372782	p.L134F	c.400C>T	Verified	CCA	CTT	ATT	1	C	T
TCGA-29-1695	ZMIZ1	ENST00000334512	p.T356M	c.1067C>T	Unverified	atg	acg	ccc	2	C	T
TCGA-24-0975	ZMIZ2	ENST00000309315	p.P293P	c.879C>T	Unverified	GCG	CCC	AGC	3	C	T
TCGA-61-1915	ZMYM4	ENST00000314607	p.S390L	c.1169C>T	Unverified	TGT	TCA	AGT	2	C	T
TCGA-13-0887	ZMYND8	ENST00000461685	p.A497A	c.1491C>T	Verified	cca	gcc	tcc	3	C	T
TCGA-13-1510	ZNF121	ENST00000320451	p.Q100*	c.298C>T	Unverified	GAT	CAG	TCA	1	C	T
TCGA-24-2035	ZNF135	ENST00000313434	p.R514*	c.1540C>T	Verified	CAG	CGA	ATC	1	C	T
TCGA-23-1124	ZNF14	ENST00000344099	p.H297Y	c.889C>T	Verified	AGG	CAT	AAA	1	C	T
TCGA-24-1104	ZNF16	ENST00000394909	p.Q102*	c.304C>T	Verified	TCC	CAG	GTA	1	C	T
TCGA-13-1499	ZNF167	ENST00000273320	p.R394W	c.1180C>T	Verified	AAT	CGG	AGT	1	C	T
TCGA-23-1123	ZNF182	ENST00000396965	p.T257M	c.770C>T	Verified	cat	acg	gga	2	C	T
TCGA-23-1123	ZNF182	ENST00000396965	p.T257M	c.770C>T	Verified	cat	acg	gga	2	C	T
TCGA-04-1338	ZNF185	NM_007150.1	p.I76I	c.228C>T	Verified	ttt	atc	gcg	3	C	T
TCGA-24-1431	ZNF213	ENST00000574902	p.L252L	c.754C>T	Unverified	TCC	CTG	CTG	1	C	T

TCGA-29-1701	ZNF217	ENST00000371471	p.P450P	c.1350C>T	Unverified	CTT	CCC	GAA	3	C	T
TCGA-29-1701	ZNF217_ENS	ENST00000371471	p.P450P	c.1350C>T	Unverified	CTT	CCC	GAA	3	C	T
TCGA-25-2400	ZNF238	ENST00000358704	p.R482C	c.1444C>T	Unverified	GAG	CGC	AGG	1	C	T
TCGA-23-1124	ZNF263	ENST00000219069	p.L62L	c.186C>T	Verified	CGG	CTC	CAA	3	C	T
TCGA-25-1313	ZNF292	ENST00000339907	p.P1119L	c.3356C>T	Verified	CTT	CCT	TTA	2	C	T
TCGA-61-1737	ZNF3	ENST00000299667	p.S185S	c.555C>T	Unverified	AAT	TCC	AAC	3	C	T
TCGA-13-0905	ZNF300	ENST00000394226	p.S475F	c.1424C>T	Verified	TTC	TCC	CGC	2	C	T
TCGA-25-2398	ZNF319	ENST00000299237	p.D500D	c.1500C>T	Unverified	TCA	GAC	CTG	3	C	T
TCGA-20-1686	ZNF324B	ENST00000336614	p.P396L	c.1187C>T	Unverified	AAG	CCC	TTC	2	C	T
TCGA-61-1904	ZNF33A	ENST00000307441	p.P383L	c.1148C>T	Unverified	AAA	CCT	TTT	2	C	T
TCGA-61-1899	ZNF341	ENST00000342427	p.R73W	c.217C>T	Unverified	AAG	CGG	GAA	1	C	T
TCGA-24-1422	ZNF385	ENST00000394313	p.R179W	c.535C>T	Verified	AAG	CGG	CTG	1	C	T
TCGA-24-1422	ZNF385A	ENST00000551109	p.R179W	c.535C>T	Verified	AAG	CGG	CTG	1	C	T
TCGA-24-1844	ZNF407	ENST00000299687	p.R321*	c.961C>T	Unverified	TTA	CGA	AAT	1	C	T
TCGA-24-1844	ZNF407_ENS	ENST00000582337	p.R321*	c.961C>T	Unverified	TTA	CGA	AAT	1	C	T
TCGA-61-1899	ZNF423	ENST00000561648	p.L82L	c.244C>T	Unverified	GAC	CTG	ACG	1	C	T
TCGA-61-2102	ZNF423	ENST00000561648	p.S21S	c.63C>T	Verified	TCC	TCC	GTG	3	C	T
TCGA-61-1899	ZNF423_ENS	ENST00000262383	p.L82L	c.244C>T	Unverified	GAC	CTG	ACG	1	C	T
TCGA-13-0760	ZNF498	ENST00000394152	p.G146G	c.438C>T	Verified	AGA	GGC	GCT	3	C	T
TCGA-23-1032	ZNF518A_EN	ENST00000371192_v61	p.P261S	c.781C>T	Verified	TTT	CCC	TTC	1	C	T
TCGA-30-1718	ZNF521	NM_015461.1	p.R163C	c.487C>T	Unverified	gat	cgc	cac	1	C	T
TCGA-24-2262	ZNF521	NM_015461.1	p.L726L	c.2178C>T	Verified	acc	ctc	tgc	3	C	T
TCGA-61-2012	ZNF527	ENST00000356178	p.S287L	c.860C>T	Verified	TAC	TCA	TTC	2	C	T
TCGA-24-2280	ZNF546	ENST00000347077	p.D78D	c.234C>T	Unverified	CTG	GAC	GCT	3	C	T
TCGA-24-2262	ZNF564	ENST00000339282	p.R157*	c.469C>T	Verified	TTT	CGA	AGA	1	C	T
TCGA-20-0991	ZNF570	ENST00000330173	p.P497L	c.1490C>T	Verified	AGA	CCC	TAT	2	C	T
TCGA-13-1496	ZNF586	ENST00000396154	p.P400L	c.1199C>T	Verified	AGG	CCT	TAT	2	C	T
TCGA-29-1762	ZNF592	ENST00000299927	p.Q903*	c.2707C>T	Unverified	GTG	CAG	AAG	1	C	T
TCGA-13-1507	ZNF592	ENST00000299927	p.H1031H	c.3093C>T	Verified	AAA	CAC	ATC	3	C	T
TCGA-13-0795	ZNF594	ENST00000399604	p.C598C	c.1794C>T	Verified	GAA	TGC	AAA	3	C	T
TCGA-24-2289	ZNF597	ENST00000301744	p.R229*	c.685C>T	Unverified	TCC	CGA	CAC	1	C	T
TCGA-24-1463	ZNF606	ENST00000341164	p.H694Y	c.2080C>T	Verified	TCT	CAC	CTC	1	C	T
TCGA-10-0930	ZNF610	ENST00000327920	p.I31I	c.93C>T	Verified	GCC	ATC	GAA	3	C	T
TCGA-10-0930	ZNF610	ENST00000327920	p.I31I	c.93C>T	Verified	GCC	ATC	GAA	3	C	T

TCGA-29-1777	ZNF619	ENST00000314686	p.G71G	c.213C>T	Unverified	AAT	GGC	GTG	3	C	T
TCGA-04-1655	ZNF627	ENST00000361113	p.H65Y	c.193C>T	Unverified	AGT	CAT	ATT	1	C	T
TCGA-30-1856	ZNF645	ENST00000323684	p.A283V	c.848C>T	Unverified	tat	gcg	cca	2	C	T
TCGA-24-2024	ZNF654	ENST00000309495	p.A18V	c.53C>T	Verified	CAG	GCT	CAT	2	C	T
TCGA-24-1103	ZNF682	ENST00000397165	p.A321V	c.962C>T	Verified	AAA	GCC	TTT	2	C	T
TCGA-04-1342	ZNF721	ENST00000338977	p.Q310*	c.928C>T	Verified	AGA	CAG	TCC	1	C	T
TCGA-13-1501	ZNF746	ENST00000340622	p.R31C	c.91C>T	Verified	GCT	CGC	CTG	1	C	T
TCGA-13-1501	ZNF746	ENST00000340622	p.R31C	c.91C>T	Verified	GCT	CGC	CTG	1	C	T
TCGA-13-0885	ZNF764	ENST00000252797	p.Y178Y	c.534C>T	Verified	TGC	TAC	GTG	3	C	T
TCGA-13-0760	ZNF785	ENST00000395216	p.T279M	c.836C>T	Verified	CAC	ACG	GGC	2	C	T
TCGA-30-1891	ZNF79	ENST00000342483	p.R105*	c.313C>T	Unverified	CTG	CGA	AGT	1	C	T
TCGA-61-2113	ZNF79	ENST00000342483	p.Y389Y	c.1167C>T	Unverified	CCC	TAC	AAG	3	C	T
TCGA-13-0923	ZNF80	ENST00000308095	p.F92F	c.276C>T	Unverified	GAC	TTC	GTT	3	C	T
TCGA-13-1488	ZNF800	ENST00000393312	p.H310Y	c.928C>T	Verified	GTT	CAT	AGA	1	C	T
TCGA-09-1674	ZNF804B	ENST00000333190	p.P536S	c.1606C>T	Unverified	AAA	CCA	AAG	1	C	T
TCGA-61-1733	ZNF816	ENST00000357666	p.H168H	c.504C>T	Unverified	CTC	CAC	ATG	3	C	T
TCGA-61-1733	ZNF816	ENST00000357666	p.H168H	c.504C>T	Unverified	CTC	CAC	ATG	3	C	T
TCGA-13-0791	ZNF841_ENS	ENST00000359973	p.G291G	c.873C>T	Verified	TGT	GGC	AAA	3	C	T
TCGA-13-0760	ZNF860	ENST00000360311	p.A78V	c.233C>T	Verified	ACA	GCG	CAA	2	C	T
TCGA-24-1470	ZNF98	ENST00000357774	p.S474F	c.1421C>T	Verified	CAG	TCC	TCA	2	C	T
TCGA-13-1496	ZSCAN10	ENST00000252463	p.R534C	c.1600C>T	Unverified	GTG	CGC	CGG	1	C	T
TCGA-29-1701	ZSCAN5B	ENST00000358992	p.N453N	c.1359C>T	Unverified	GGG	AAC	CTG	3	C	T
TCGA-29-1701	ZSCAN5B_EI	ENST00000358992	p.N453N	c.1359C>T	Unverified	GGG	AAC	CTG	3	C	T
TCGA-29-1781	ZWILCH	ENST00000307897	p.P416S	c.1246C>T	Unverified	ATT	CCA	GTT	1	C	T
TCGA-20-1687	ZZEF1	ENST00000381638	p.T561M	c.1682C>T	Unverified	CTT	ACG	GAA	2	C	T
TCGA-29-1761	ZZZ3	ENST00000370801	p.A34V	c.101C>T	Unverified	ATT	GCG	CAT	2	C	T
TCGA-23-1029	A1CF	ENST00000374001	p.R26H	c.77G>A	Unverified	CAG	CGC	ACA	2	G	A
TCGA-25-2398	A2ML1	ENST00000299698	p.A307A	c.921G>A	Verified	TAT	GCG	TAC	3	G	A
TCGA-61-1899	ABCA1	ENST00000374736	p.R1897Q	c.5690G>A	Unverified	AGG	CGG	GAA	2	G	A
TCGA-61-2109	ABCA1	ENST00000374736	p.W685*	c.2055G>A	Unverified	AGC	TGG	TTC	3	G	A
TCGA-13-1499	ABCA3	ENST00000301732	p.G429S	c.1285G>A	Verified	AAA	GGC	ATG	1	G	A
TCGA-61-1740	ABCA3	ENST00000301732	p.W179*	c.537G>A	Unverified	GGC	TGG	CAC	3	G	A
TCGA-20-1683	ABCA4	ENST00000370225	p.R1368H	c.4103G>A	Unverified	ATC	CGC	AGC	2	G	A
TCGA-29-1783	ABCA4	ENST00000370225	p.G1573E	c.4718G>A	Unverified	ACG	GGG	GAA	2	G	A

TCGA-04-1361	ABCB4	ENST00000359206	p.L1205L	c.3615G>A	Verified	GCT	CTG	GAT	3	G	A
TCGA-04-1361	ABCB4	ENST00000359206	p.L1205L	c.3615G>A	Verified	GCT	CTG	GAT	3	G	A
TCGA-13-0883	ABCB5	ENST00000258738	p.R440H	c.1319G>A	Verified	ATA	CGT	ACT	2	G	A
TCGA-04-1331	ABCB8	ENST00000358849	p.G112E	c.335G>A	Unverified	GTG	GGG	TCT	2	G	A
TCGA-23-1032	ABCC1	ENST00000399410	p.L1043L	c.3129G>A	Unverified	ATC	TTG	GCT	3	G	A
TCGA-24-2267	ABCC11	ENST00000356608	p.S835S	c.2505G>A	Verified	GGC	TCG	GGG	3	G	A
TCGA-24-1470	ABCC9	ENST00000261200	p.R1546H	c.4637G>A	Verified	GTT	CGC	GCA	2	G	A
TCGA-25-1326	ABCC9	ENST00000261200	p.A825A	c.2475G>A	Verified	CGA	GCG	CTG	3	G	A
TCGA-13-0905	ABCG1	ENST00000361802	p.R213Q	c.638G>A	Verified	ACG	CGG	ACC	2	G	A
TCGA-24-1470	ABCG1	ENST00000361802	p.L440L	c.1320G>A	Verified	CTG	CTG	TAC	3	G	A
TCGA-29-1769	ABL2	NM_005158	p.L1111L	c.3333G>A	Unverified	agc	ctg	cag	3	G	A
TCGA-29-1769	ABL2_ENST(	ENST00000502732	p.L1147L	c.3441G>A	Unverified	AGC	CTG	CAG	3	G	A
TCGA-29-1769	ABL2_ENST(	ENST00000512653	p.L1132L	c.3396G>A	Unverified	AGC	CTG	CAG	3	G	A
TCGA-13-0903	ABO	ENST00000319878	p.?	c.203+1G>A	Unverified	CCA	AGG	ATG	2	G	A
TCGA-13-0886	ABR	ENST00000302538	p.E35K	c.103G>A	Unverified	AAT	GAG	GAG	1	G	A
TCGA-61-1915	AC012100.1	ENST00000261854	p.?	c.585-1G>A	Unverified	GAA	TTG	GAA	3	G	A
TCGA-13-0890	ACACB	ENST00000377848	p.S1199S	c.3597G>A	Verified	CTG	TCG	GAC	3	G	A
TCGA-30-1857	ACAD11	ENST00000264990	p.G443R	c.1327G>A	Unverified	AGC	GGA	CTC	1	G	A
TCGA-24-1469	ACADM	ENST00000370841	p.G195R	c.583G>A	Verified	AAC	GGA	GGA	1	G	A
TCGA-29-1699	ACADM	ENST00000370841	p.?	c.850-1G>A	Unverified	GTA	GTA	GCT	1	G	A
TCGA-24-1616	ACADSB	ENST00000358776	p.V268I	c.802G>A	Verified	AAT	GTC	AAG	1	G	A
TCGA-24-2280	ACCN1_ENS1	ENST00000359872	p.V50M	c.148G>A	Unverified	TTC	GTG	GGC	1	G	A
TCGA-24-1604	ACE	ENST00000290866	p.R508Q	c.1523G>A	Verified	ACC	CGA	AAC	2	G	A
TCGA-36-1577	ACE	ENST00000290866	p.G1099D	c.3296G>A	Verified	CAG	GGC	CTC	2	G	A
TCGA-29-1698	ACRBP	ENST00000229243	p.E463E	c.1389G>A	Unverified	ACT	GAG	TTC	3	G	A
TCGA-04-1361	ACSL1	ENST00000281455	p.W611*	c.1832G>A	Verified	TCC	TGG	GCC	2	G	A
TCGA-04-1361	ACSL1	ENST00000281455	p.W611*	c.1832G>A	Verified	TCC	TGG	GCC	2	G	A
TCGA-61-1733	ACTL7A	ENST00000333999	p.T47T	c.141G>A	Unverified	CAT	ACG	AGT	3	G	A
TCGA-61-1733	ACTL7A	ENST00000333999	p.T47T	c.141G>A	Unverified	CAT	ACG	AGT	3	G	A
TCGA-24-1464	ACTL8	ENST00000375406	p.R365R	c.1095G>A	Verified	ATG	AGG	ATG	3	G	A
TCGA-13-0919	ACVR1C	ENST00000243349	p.R462H	c.1385G>A	Verified	ATG	CGT	GAG	2	G	A
TCGA-13-0924	ADAM12	ENST00000368679	p.G37R	c.109G>A	Verified	CAA	GGA	AGA	1	G	A
TCGA-30-1718	ADAM12	ENST00000368679	p.R176Q	c.527G>A	Unverified	GTC	CGG	GGA	2	G	A
TCGA-30-1718	ADAM12_EI	ENST00000368676	p.R176Q	c.527G>A	Unverified	GTC	CGG	GGA	2	G	A

TCGA-30-1718	ADAM12_EN	ENST00000368679	p.R176Q	c.527G>A	Unverified	GTC	CGG	GGA	2	G	A
TCGA-29-1763	ADAM19	NM_023038.2	p.D186N	c.556G>A	Unverified	agg	gac	tgg	1	G	A
TCGA-23-1809	ADAM19	NM_023038.2	p.R793Q	c.2378G>A	Unverified	ccc	cgg	ccc	2	G	A
TCGA-29-1763	ADAM19_EN	ENST00000257527	p.D185N	c.553G>A	Unverified	AGG	GAC	TGG	1	G	A
TCGA-23-1809	ADAM19_EN	ENST00000257527	p.R792Q	c.2375G>A	Unverified	CCC	CGG	CCC	2	G	A
TCGA-24-2289	ADAM2	ENST00000265708	p.P30P	c.90G>A	Unverified	gtt	ccg	gag	3	G	A
TCGA-13-0906	ADAM22	ENST00000265727	p.A3A	c.9G>A	Unverified	CAG	GCG	GCA	3	G	A
TCGA-13-0906	ADAM22_EN	ENST00000398209	p.A3A	c.9G>A	Unverified	CAG	GCG	GCA	3	G	A
TCGA-13-0923	ADAM23	ENST00000264377	p.G578S	c.1732G>A	Verified	tct	ggt	cag	1	G	A
TCGA-24-1845	ADAM30	ENST00000369400	p.V159I	c.475G>A	Unverified	gtc	gtc	tat	1	G	A
TCGA-29-1691	ADAM30	ENST00000369400	p.G394R	c.1180G>A	Unverified	cca	gga	cta	1	G	A
TCGA-13-0884	ADAM32	ENST00000379907	p.P390P	c.1170G>A	Verified	TCT	CCG	AAA	3	G	A
TCGA-23-1114	ADAM7	ENST00000175238	p.V358M	c.1072G>A	Unverified	TGC	GTG	ATG	1	G	A
TCGA-24-2288	ADAMTS1	ENST00000284984	p.G174E	c.521G>A	Unverified	CCA	GGG	GAG	2	G	A
TCGA-13-1408	ADAMTS12	ENST00000504830	p.T144T	c.432G>A	Verified	ggc	acg	gtt	3	G	A
TCGA-20-1686	ADAMTS12	ENST00000504830	p.V295V	c.885G>A	Unverified	gtt	gtg	gtt	3	G	A
TCGA-09-0369	ADAMTS14	NM_139155.1	p.G377D	c.1130G>A	Verified	act	ggc	atg	2	G	A
TCGA-09-0369	ADAMTS14	NM_139155.1	p.G377D	c.1130G>A	Verified	act	ggc	atg	2	G	A
TCGA-09-1674	ADAMTS19	NM_133638.1	p.R1057H	c.3170G>A	Unverified	ata	cgt	cat	2	G	A
TCGA-23-1022	ADAMTS19	NM_133638.1	p.A1042A	c.3126G>A	Verified	gag	gcg	gga	3	G	A
TCGA-24-1563	ADAMTS20	ENST00000389420	p.S1031N	c.3092G>A	Verified	TGG	AGC	GAG	2	G	A
TCGA-24-2288	ADAMTS7	ENST00000388820	p.R585H	c.1754G>A	Unverified	TTC	CGC	CTC	2	G	A
TCGA-29-1699	ADAMTSL3	ENST00000286744	p.D976N	c.2926G>A	Unverified	CCC	GAC	ATC	1	G	A
TCGA-29-1705	ADARB1	ENST00000360697	p.A289A	c.867G>A	Unverified	GCT	GCG	CAG	3	G	A
TCGA-29-1705	ADARB1_EN	ENST00000389863	p.A289A	c.867G>A	Unverified	GCT	GCG	CAG	3	G	A
TCGA-61-1913	ADCK4	ENST00000324464	p.A139T	c.415G>A	Unverified	AAT	GCC	GAG	1	G	A
TCGA-61-1913	ADCK4_ENS	ENST00000324464	p.A139T	c.415G>A	Unverified	AAT	GCC	GAG	1	G	A
TCGA-24-1423	ADCY2	ENST00000338316	p.R238H	c.713G>A	Unverified	AAA	CGT	CAA	2	G	A
TCGA-24-2280	ADD2	ENST00000355733	p.L191L	c.573G>A	Unverified	ATT	CTG	GGA	3	G	A
TCGA-23-1123	ADNP	ENST00000349014	p.V690I	c.2068G>A	Verified	GGC	GTT	GGA	1	G	A
TCGA-23-1123	ADNP	ENST00000349014	p.V690I	c.2068G>A	Verified	GGC	GTT	GGA	1	G	A
TCGA-13-0900	ADORA3_EN	ENST00000369716	p.G125G	c.375G>A	Verified	CCT	GGG	TGC	3	G	A
TCGA-25-1313	ADRA2B	ENST00000409345	p.R161H	c.482G>A	Unverified	GGG	CGC	CCC	2	G	A
TCGA-29-1761	AEBP1	ENST00000223357	p.G974R	c.2920G>A	Unverified	ATC	GGG	GCC	1	G	A

TCGA-09-2049	AFTPH	ENST00000238856	p.A895T	c.2683G>A	Verified	CCA	GCC	ACG	1	G	A
TCGA-61-1895	AGAP1	ENST00000304032	p.R188K	c.563G>A	Unverified	CCG	AGG	GTC	2	G	A
TCGA-29-1702	AGAP6	ENST00000374056	p.E28K	c.82G>A	Unverified	TCT	GAA	TCT	1	G	A
TCGA-24-1103	AGTPBP1	ENST00000376083	p.G20R	c.58G>A	Verified	GTA	GGA	CTC	1	G	A
TCGA-29-2427	AHCTF1	ENST00000391829	p.E85K	c.253G>A	Verified	AAA	GAA	TTC	1	G	A
TCGA-13-0906	AHNAK	ENST00000378024	p.V431M	c.1291G>A	Verified	AAT	GTG	CCC	1	G	A
TCGA-29-1763	AHNAK	ENST00000378024	p.K2978K	c.8934G>A	Unverified	GTG	AAG	GGC	3	G	A
TCGA-24-1846	AIFM2	ENST00000307864	p.S258N	c.773G>A	Unverified	GAG	AGC	AGA	2	G	A
TCGA-24-1103	AIFM3	ENST00000399167	p.S194N	c.581G>A	Verified	AGT	AGC	ACC	2	G	A
TCGA-30-1856	AIPL1	ENST00000381129	p.A275T	c.823G>A	Unverified	CAC	GCA	GAG	1	G	A
TCGA-13-1408	AKAP2	ENST00000374525	p.A499T	c.1495G>A	Verified	ACA	GCC	TCT	1	G	A
TCGA-09-2044	AKAP6	ENST00000280979	p.L441L	c.1323G>A	Verified	GTA	TTG	CAG	3	G	A
TCGA-61-2012	AKAP9	NM_147171.1	p.V2886V	c.8658G>A	Verified	gca	gtg	ata	3	G	A
TCGA-61-2012	AKAP9_ENS	ENST00000356239	p.V2874V	c.8622G>A	Verified	GCA	GTG	ATA	3	G	A
TCGA-61-2095	AKNA	ENST00000307564	p.G248D	c.743G>A	Verified	GAT	GGC	AGA	2	G	A
TCGA-04-1361	AKNAD1	ENST00000370001	p.P174P	c.522G>A	Verified	AAC	CCG	AAA	3	G	A
TCGA-04-1361	AKNAD1	ENST00000370001	p.P174P	c.522G>A	Verified	AAC	CCG	AAA	3	G	A
TCGA-13-1501	AKR1B15	ENST00000418096	p.E197K	c.589G>A	Verified	GAC	GAG	GGG	1	G	A
TCGA-13-1501	AKR1B15	ENST00000418096	p.E197K	c.589G>A	Verified	GAC	GAG	GGG	1	G	A
TCGA-24-2289	AKR1C1	ENST00000380872	p.A44T	c.130G>A	Unverified	GAA	GCT	GGC	1	G	A
TCGA-30-1855	AKT1	ENST00000349310	p.S122S	c.366G>A	Unverified	CGG	TCG	GGC	3	G	A
TCGA-23-1110	ALAS2	NM_000032.1	p.E342E	c.1026G>A	Verified	gag	gag	ttg	3	G	A
TCGA-24-1103	ALDH1A1	ENST00000297785	p.E107E	c.321G>A	Verified	atg	gag	tca	3	G	A
TCGA-13-0885	ALDH1A1	ENST00000297785	p.G376G	c.1128G>A	Verified	tgg	ggg	aat	3	G	A
TCGA-61-1725	ALDH1B1	ENST00000377698	p.R281K	c.842G>A	Unverified	aag	aga	gtc	2	G	A
TCGA-23-1111	ALDH1L1	ENST00000393434	p.A467T	c.1399G>A	Unverified	GCC	GCA	GCC	1	G	A
TCGA-09-1674	ALDH1L1	ENST00000393434	p.E257K	c.769G>A	Unverified	CCC	GAG	GGA	1	G	A
TCGA-61-2113	ALDH1L1	ENST00000393434	p.G628E	c.1883G>A	Unverified	CCA	GGA	TCT	2	G	A
TCGA-20-1683	ALDH3A1	ENST00000225740	p.G438D	c.1313G>A	Unverified	gaa	ggc	ctg	2	G	A
TCGA-09-2051	ALDH6A1	ENST00000553458	p.R535H	c.1604G>A	Unverified	GGC	CGT	TAG	2	G	A
TCGA-20-1686	ALDOA	ENST00000338110	p.G341E	c.1022G>A	Unverified	caa	gga	aag	2	G	A
TCGA-09-1674	ALG1	ENST00000262374	p.G145S	c.433G>A	Unverified	GTG	GGC	TGC	1	G	A
TCGA-04-1655	ALG1	ENST00000262374	p.G407D	c.1220G>A	Unverified	AAT	GGC	CTG	2	G	A
TCGA-24-2035	ALG10B	ENST00000308742	p.M249I	c.747G>A	Verified	AGT	ATG	CTT	3	G	A

TCGA-13-1489	ALG9	ENST00000531154	p.G123E	c.368G>A	Verified	CAT	GGA	CCT	2	G	A
TCGA-61-1740	ALK	ENST00000389048	p.?	c.3515+1G>A	Unverified	atc	agc	aaa	2	G	A
TCGA-25-1313	ALK	ENST00000389048	p.P1112P	c.3336G>A	Unverified	gtg	ccg	cgg	3	G	A
TCGA-04-1342	ALKBH1	ENST00000216489	p.D142N	c.424G>A	Verified	CAA	GAT	CTG	1	G	A
TCGA-23-1114	ALMS1	ENST00000264448	p.D844N	c.2530G>A	Unverified	GCT	GAC	GGA	1	G	A
TCGA-24-1849	ALOX15B	ENST00000380183	p.L402L	c.1206G>A	Unverified	ctg	ctg	atc	3	G	A
TCGA-04-1652	ALOX5	ENST00000374391	p.E147K	c.439G>A	Unverified	atg	gag	tgg	1	G	A
TCGA-04-1652	ALOX5	ENST00000374391	p.E147K	c.439G>A	Unverified	atg	gag	tgg	1	G	A
TCGA-04-1336	ALPK1	ENST00000458497	p.G487R	c.1459G>A	Verified	ACA	GGA	GTC	1	G	A
TCGA-29-1695	ALPK1	ENST00000458497	p.T305T	c.915G>A	Unverified	GGC	ACG	TGT	3	G	A
TCGA-30-1857	ALX1	ENST00000316824	p.E112E	c.336G>A	Unverified	CAA	GAG	AAG	3	G	A
TCGA-30-1855	AMAC1	ENST00000297307	p.D158N	c.472G>A	Unverified	TAC	GAC	TGG	1	G	A
TCGA-61-2012	AMIGO1	ENST00000369862	p.L265L	c.795G>A	Verified	AAC	CTG	AGT	3	G	A
TCGA-25-1318	AMIGO2	ENST00000266581	p.L140L	c.420G>A	Verified	GAG	TTG	AAG	3	G	A
TCGA-25-1318	AMIGO2	ENST00000266581	p.L140L	c.420G>A	Verified	GAG	TTG	AAG	3	G	A
TCGA-24-1847	ANGEL1	ENST00000251089	p.G432R	c.1294G>A	Unverified	TGC	GGG	GAC	1	G	A
TCGA-13-1497	ANGPT4	ENST00000381922	p.R379H	c.1136G>A	Verified	CTG	CGT	GTG	2	G	A
TCGA-61-1913	ANK1	ENST00000347528	p.C1357Y	c.4070G>A	Unverified	ctc	tgc	cac	2	G	A
TCGA-61-1913	ANK1_ENST(	ENST00000265709	p.C1398Y	c.4193G>A	Unverified	CTC	TGC	CAC	2	G	A
TCGA-09-2050	ANK3	ENST00000280772	p.V373V	c.1119G>A	Verified	CAC	GTG	GCT	3	G	A
TCGA-13-0893	ANKHD1	ENST00000360839	p.A2383T	c.7147G>A	Verified	gtt	gca	caa	1	G	A
TCGA-23-1118	ANKHD1	ENST00000360839	p.K1462K	c.4386G>A	Verified	aaa	aag	aaa	3	G	A
TCGA-13-0893	ANKHD1-EIF	ENST00000297183	p.A2383T	c.7147G>A	Verified	GTT	GCA	CAA	1	G	A
TCGA-23-1118	ANKHD1-EIF	ENST00000297183	p.K1462K	c.4386G>A	Verified	AAA	AAG	AAA	3	G	A
TCGA-29-1777	ANKRD11	ENST00000301030	p.K59K	c.177G>A	Unverified	CGG	AAG	CTG	3	G	A
TCGA-04-1342	ANKRD12	ENST00000262126	p.A138T	c.412G>A	Verified	ATG	GCA	CTT	1	G	A
TCGA-29-1690	ANKRD13C	ENST00000370944	p.E424K	c.1270G>A	Unverified	GAA	GAA	TAT	1	G	A
TCGA-04-1530	ANKRD17	NM_032217.3	p.G2330S	c.6988G>A	Unverified	tat	ggt	tct	1	G	A
TCGA-29-1703	ANKRD26	ENST00000376087	p.Q64Q	c.192G>A	Unverified	CAG	CAG	ATC	3	G	A
TCGA-30-1891	ANKRD28_E	ENST00000383777	p.L475L	c.1425G>A	Unverified	CCA	CTG	CAC	3	G	A
TCGA-13-1481	ANKRD33	ENST00000301190	p.S334S	c.1002G>A	Unverified	CCT	TCG	CTG	3	G	A
TCGA-04-1652	ANKRD34B	ENST00000338682	p.A245T	c.733G>A	Unverified	CAC	GCT	CCA	1	G	A
TCGA-04-1652	ANKRD34B	ENST00000338682	p.A245T	c.733G>A	Unverified	CAC	GCT	CCA	1	G	A
TCGA-13-0883	ANKRD35	ENST00000355594	p.E914K	c.2740G>A	Verified	AAA	GAG	AAG	1	G	A

TCGA-13-1507	ANKRD44	NM_153697.1	p.G711R	c.2131G>A	Verified	aga	ggg	agg	1	G	A
TCGA-13-0903	ANKRD44	NM_153697.1	p.L783L	c.2349G>A	Verified	cca	ctg	cac	3	G	A
TCGA-30-1891	ANKRD57	ENST00000356454	p.A363T	c.1087G>A	Unverified	GGG	GCC	TAC	1	G	A
TCGA-25-2393	ANO1	ENST00000355303	p.M378I	c.1134G>A	Verified	ACC	ATG	TGC	3	G	A
TCGA-13-0791	ANO4	ENST00000392979	p.G581R	c.1741G>A	Verified	CTC	GGA	AGA	1	G	A
TCGA-04-1367	ANP32C	ENST00000330241	p.E192E	c.576G>A	Unverified	GAG	GAG	GAA	3	G	A
TCGA-24-1469	ANTXR1	ENST00000303714	p.A192T	c.574G>A	Verified	ATT	GCG	GAC	1	G	A
TCGA-29-1696	AP001011.2	ENST00000261598	p.G910D	c.2729G>A	Unverified	CCT	GGC	TTA	2	G	A
TCGA-29-1696	AP001011.2	ENST00000261598	p.G910D	c.2729G>A	Unverified	CCT	GGC	TTA	2	G	A
TCGA-29-1696	AP001011.3	ENST00000320876	p.G910D	c.2729G>A	Unverified	CCT	GGC	TTA	2	G	A
TCGA-29-1696	AP001011.3	ENST00000320876	p.G910D	c.2729G>A	Unverified	CCT	GGC	TTA	2	G	A
TCGA-61-1915	AP1G2	ENST00000397120	p.D41N	c.121G>A	Unverified	CGC	GAC	GGG	1	G	A
TCGA-09-0369	AP1M1	ENST00000291439	p.V25M	c.73G>A	Verified	GAC	GTG	GAC	1	G	A
TCGA-09-0369	AP1M1	ENST00000291439	p.V25M	c.73G>A	Verified	GAC	GTG	GAC	1	G	A
TCGA-23-1120	AP2A2	ENST00000448903	p.R515K	c.1544G>A	Unverified	CCG	AGA	TCC	2	G	A
TCGA-09-1665	APAF1	ENST00000551964	p.D479N	c.1435G>A	Verified	gaa	gac	tgt	1	G	A
TCGA-23-1022	APC	ENST00000457016	p.G1120E	c.3359G>A	Verified	CAT	GGA	ATT	2	G	A
TCGA-04-1367	APCS	ENST00000255040	p.L133L	c.399G>A	Verified	CCT	TTG	GTG	3	G	A
TCGA-13-1507	APEX1	ENST00000555414	p.R193H	c.578G>A	Verified	ttt	cgc	aag	2	G	A
TCGA-36-1577	APOB	ENST00000233242	p.G1227S	c.3679G>A	Unverified	GTG	GGT	TCC	1	G	A
TCGA-61-1914	APOBEC1	ENST00000229304	p.E41K	c.121G>A	Unverified	tac	gaa	atc	1	G	A
TCGA-29-1703	APOBR	ENST00000328423	p.V993M	c.2977G>A	Unverified	CGC	GTG	CAC	1	G	A
TCGA-23-1110	ARAP2	ENST00000303965	p.L45L	c.135G>A	Verified	AGC	CTG	CTG	3	G	A
TCGA-25-1326	ARFGEF1	ENST00000262215	p.V1305I	c.3913G>A	Verified	att	gtc	acc	1	G	A
TCGA-23-1021	ARHGAP21	NM_020824.2	p.T1551T	c.4653G>A	Verified	agc	acg	tct	3	G	A
TCGA-61-1910	ARHGAP26	ENST00000274498	p.R171R	c.513G>A	Unverified	GTC	CGG	CAG	3	G	A
TCGA-20-1686	ARHGAP31	ENST00000264245	p.V1075M	c.3223G>A	Unverified	AGC	GTG	CAG	1	G	A
TCGA-29-1769	ARHGAP31	ENST00000264245	p.E1070K	c.3208G>A	Unverified	AAG	GAG	AGT	1	G	A
TCGA-13-1488	ARHGAP33	ENST00000314737	p.G668S	c.2002G>A	Verified	ccc	ggc	cgg	1	G	A
TCGA-24-1435	ARHGAP5	ENST00000345122	p.V474I	c.1420G>A	Verified	GAG	GTA	TAT	1	G	A
TCGA-13-1499	ARHGEF1	ENST00000337665	p.A76T	c.226G>A	Verified	atg	gcc	ctc	1	G	A
TCGA-29-1770	ARHGEF1	ENST00000337665	p.T812T	c.2436G>A	Unverified	gag	acg	tct	3	G	A
TCGA-29-1701	ARHGEF10	NM_014629.1	p.V819I	c.2455G>A	Unverified	ggc	gtc	cta	1	G	A
TCGA-24-2289	ARHGEF10_	ENST00000349830	p.E115K	c.343G>A	Unverified	GAG	GAG	AAT	1	G	A

TCGA-29-1701	ARHGEF10_	ENST00000349830	p.V1042I	c.3124G>A	Unverified	GGC	GTC	CTA	1	G	A
TCGA-04-1361	ARHGEF10L	ENST00000361221	p.A420T	c.1258G>A	Verified	AGT	GCC	ATG	1	G	A
TCGA-04-1361	ARHGEF10L	ENST00000361221	p.A420T	c.1258G>A	Verified	AGT	GCC	ATG	1	G	A
TCGA-13-0924	ARHGEF11	ENST00000368194	p.D1350N	c.4048G>A	Verified	gaa	gat	gct	1	G	A
TCGA-24-1850	ARHGEF11	ENST00000368194	p.V1132I	c.3394G>A	Unverified	ccc	gtc	cat	1	G	A
TCGA-13-0887	ARHGEF16	NM_014448.2	p.E77E	c.231G>A	Verified	gag	gag	cac	3	G	A
TCGA-29-1695	ARHGEF26	ENST00000356448	p.E229K	c.685G>A	Unverified	CTC	GAG	AAT	1	G	A
TCGA-29-1695	ARHGEF26_	ENST00000356448	p.E229K	c.685G>A	Unverified	CTC	GAG	AAT	1	G	A
TCGA-13-0919	ARHGEF37	ENST00000333677	p.W671*	c.2013G>A	Unverified	GGC	TGG	AGT	3	G	A
TCGA-23-1032	ARID4B	ENST00000366603	p.W935*	c.2805G>A	Verified	cag	tgg	cct	3	G	A
TCGA-23-1032	ARID4B_ENS	ENST00000264183	p.W935*	c.2805G>A	Verified	CAG	TGG	CCT	3	G	A
TCGA-25-1326	ARIH1	ENST00000379887	p.?	c.681+1G>A	Unverified	GGT	CAG	ACT	3	G	A
TCGA-20-1685	ARL5B	ENST00000377275	p.G49E	c.146G>A	Unverified	ata	gga	agc	2	G	A
TCGA-61-1998	ARMC6	ENST00000269932	p.G420S	c.1258G>A	Unverified	CAC	GGC	CAG	1	G	A
TCGA-24-1604	ARMC9	ENST00000349938	p.D117N	c.349G>A	Verified	CCG	GAC	AAA	1	G	A
TCGA-13-1505	ARMCX2	ENST00000330154	p.A330T	c.988G>A	Verified	cag	gct	ttc	1	G	A
TCGA-25-2398	ARMCX2	ENST00000330154	p.V145V	c.435G>A	Verified	ggg	gtg	aca	3	G	A
TCGA-20-1687	ARNT2	ENST00000303329	p.T217T	c.651G>A	Unverified	GGG	ACG	GTC	3	G	A
TCGA-30-1718	ARPC1B	ENST00000252725	p.V45M	c.133G>A	Unverified	AAG	GTG	CAC	1	G	A
TCGA-13-0714	ARPP21	ENST00000187397	p.Q751Q	c.2253G>A	Verified	AAC	CAG	GCA	3	G	A
TCGA-24-1844	ARRDC3	ENST00000265138	p.R251H	c.752G>A	Unverified	TTG	CGT	GGG	2	G	A
TCGA-24-1844	ARSI	ENST00000328668	p.V415M	c.1243G>A	Unverified	GCC	GTG	CAG	1	G	A
TCGA-29-1781	ART1	ENST00000250693	p.R30Q	c.89G>A	Unverified	CGA	CGA	GAC	2	G	A
TCGA-29-1783	ASAH2_ENST	ENST00000395526	p.D159N	c.475G>A	Unverified	ATC	GAC	ATA	1	G	A
TCGA-24-2280	ASB2	ENST00000315988	p.A214T	c.640G>A	Unverified	GGA	GCC	AAG	1	G	A
TCGA-24-1463	ASPM	ENST00000367409	p.R2111K	c.6332G>A	Verified	AGA	AGA	CAT	2	G	A
TCGA-04-1347	ASPM	ENST00000367409	p.K2575K	c.7725G>A	Verified	CAA	AAG	CTT	3	G	A
TCGA-24-1616	ASTN1	ENST00000361833	p.Q1174Q	c.3522G>A	Verified	cag	cag	acc	3	G	A
TCGA-13-0761	ASTN2	ENST00000361209	p.A489T	c.1465G>A	Verified	TAT	GCC	CCT	1	G	A
TCGA-13-0761	ASTN2	ENST00000361209	p.A489T	c.1465G>A	Verified	TAT	GCC	CCT	1	G	A
TCGA-29-1764	ASXL2	ENST00000272341	p.L132L	c.396G>A	Unverified	AAA	TTG	ACA	3	G	A
TCGA-29-1764	ASXL2_ENST	ENST00000435504	p.L392L	c.1176G>A	Unverified	AAA	TTG	ACA	3	G	A
TCGA-29-1695	ATG4D	ENST00000309469	p.A282T	c.844G>A	Unverified	AAG	GCG	GAT	1	G	A
TCGA-29-1785	ATP10B	ENST00000327245	p.K972K	c.2916G>A	Unverified	CGC	AAG	CTC	3	G	A

TCGA-29-1785	ATP10B	ENST00000327245	p.K972K	c.2916G>A	Unverified	CGC	AAG	CTC	3	G	A
TCGA-24-2267	ATP11C	ENST00000327569	p.?	c.246+1G>A	Verified	GTA	CAG	GTC	3	G	A
TCGA-61-1906	ATP1A4	ENST00000368081	p.?	c.1855-1G>A	Unverified	AAG	GTG	ATC	1	G	A
TCGA-61-1906	ATP1A4	ENST00000368081	p.?	c.1855-1G>A	Unverified	AAG	GTG	ATC	1	G	A
TCGA-13-0890	ATP2A1	ENST00000357084	p.E381K	c.1141G>A	Verified	AAT	GAG	TTC	1	G	A
TCGA-13-1505	ATP2A1	ENST00000357084	p.A547A	c.1641G>A	Verified	ATG	GCG	GTG	3	G	A
TCGA-24-1844	ATP2B2	ENST00000383800	p.S425S	c.1275G>A	Unverified	TAT	TCG	GTG	3	G	A
TCGA-24-1844	ATP2B2_EN!	ENST00000360273	p.S470S	c.1410G>A	Unverified	TAT	TCG	GTG	3	G	A
TCGA-23-1117	ATP2B3_EN!	ENST00000349466	p.V1216M	c.3646G>A	Verified	AGC	GTG	GAG	1	G	A
TCGA-23-1117	ATP2B3_EN!	ENST00000349466	p.V1216M	c.3646G>A	Verified	AGC	GTG	GAG	1	G	A
TCGA-29-1695	ATP2C2	ENST00000262429	p.G819R	c.2455G>A	Unverified	AGC	GGG	ACC	1	G	A
TCGA-29-1783	ATP5A1	ENST00000398752	p.M105I	c.315G>A	Unverified	GGT	ATG	TCC	3	G	A
TCGA-29-1784	ATP5J2	ENST00000292475	p.K59K	c.177G>A	Unverified	GTG	AAG	AAG	3	G	A
TCGA-13-0755	ATP5S	ENST00000311459	p.L214L	c.642G>A	Verified	CAA	TTG	AAG	3	G	A
TCGA-61-1915	ATP6V0D2	ENST00000285393	p.A5A	c.15G>A	Unverified	GGT	GCG	GAG	3	G	A
TCGA-25-2392	ATP6V1A	ENST00000273398	p.Q494Q	c.1482G>A	Verified	GTA	CAG	CTT	3	G	A
TCGA-09-0369	ATP6V1D	ENST00000554087	p.E242K	c.724G>A	Verified	gac	gag	gat	1	G	A
TCGA-09-0369	ATP6V1D	ENST00000554087	p.E242K	c.724G>A	Verified	gac	gag	gat	1	G	A
TCGA-23-1116	ATP6V1E2	ENST00000306448	p.R135H	c.404G>A	Verified	GTA	CGC	TGC	2	G	A
TCGA-29-1764	ATP8A2	ENST00000381655	p.G827R	c.2479G>A	Unverified	GTC	GGG	ATG	1	G	A
TCGA-13-1512	ATP8A2	ENST00000381655	p.R1139Q	c.3416G>A	Unverified	GGC	CGG	AAG	2	G	A
TCGA-24-2262	ATP9A	ENST00000338821	p.V800M	c.2398G>A	Verified	GGC	GTG	GGA	1	G	A
TCGA-04-1530	ATP9A	ENST00000338821	p.R556Q	c.1667G>A	Unverified	GTG	CGG	GAT	2	G	A
TCGA-13-1489	ATXN3L	ENST00000380622	p.R237H	c.710G>A	Verified	agc	cgc	caa	2	G	A
TCGA-61-2094	AUTS2	ENST00000342771	p.A996T	c.2986G>A	Unverified	GAG	GCC	CCG	1	G	A
TCGA-61-1904	AVP	ENST00000380293	p.P26P	c.78G>A	Unverified	TGC	CCG	AGG	3	G	A
TCGA-24-2035	AXIN1	ENST00000262320	p.Q706Q	c.2118G>A	Unverified	acc	cag	ctg	3	G	A
TCGA-61-1915	B3GALNT1	ENST00000392779	p.V91M	c.271G>A	Unverified	GAT	GTG	AAA	1	G	A
TCGA-29-1702	B3GNT1	ENST00000311181	p.V330M	c.988G>A	Unverified	TAC	GTG	GCA	1	G	A
TCGA-24-2289	B3GNT4	ENST00000324189	p.D180N	c.538G>A	Unverified	GAT	GAC	ATC	1	G	A
TCGA-61-2009	B3GNT4	ENST00000324189	p.R133Q	c.398G>A	Verified	GAG	CGA	CGT	2	G	A
TCGA-61-1740	BAG1	ENST00000472232	p.G219R	c.655G>A	Unverified	TTG	GAG	GAG	1	G	A
TCGA-61-1998	BAI2	ENST00000373658	p.V687M	c.2059G>A	Unverified	CAG	GTG	TCC	1	G	A
TCGA-25-2400	BAI2	ENST00000373658	p.L1406L	c.4218G>A	Unverified	GGC	CTG	GGG	3	G	A

TCGA-61-1907	BAIAP2L2	ENST00000381669	p.E180K	c.538G>A	Unverified	GAA	GAG	AAG	1	G	A
TCGA-13-0795	BATF2	ENST00000301887	p.Q102Q	c.306G>A	Verified	GAC	CAG	GCT	3	G	A
TCGA-25-2393	BCORL1	ENST00000218147	p.E692K	c.2074G>A	Verified	ccc	gag	atc	1	G	A
TCGA-13-1481	BDKRB2	ENST00000554311	p.G388E	c.1163G>A	Verified	GCA	GGG	AGC	2	G	A
TCGA-13-1496	BDKRB2	ENST00000554311	p.R196Q	c.587G>A	Verified	TTC	CGG	ACC	2	G	A
TCGA-13-0920	BEND2	ENST00000380033	p.V49I	c.145G>A	Verified	TAT	GTC	ACA	1	G	A
TCGA-13-1510	BEND3	ENST00000369042	p.D507N	c.1519G>A	Unverified	GAC	GAC	ATC	1	G	A
TCGA-04-1362	BEND3	ENST00000369042	p.L219L	c.657G>A	Verified	GAC	CTG	CTC	3	G	A
TCGA-24-0979	BEX2	ENST00000372677	p.A8A	c.24G>A	Verified	cga	gcg	tta	3	G	A
TCGA-13-0885	BHLHB9	ENST00000457056	p.A60T	c.178G>A	Verified	AAG	GCA	GTG	1	G	A
TCGA-09-2044	BIN1	ENST00000316724	p.D93N	c.277G>A	Verified	ccc	gat	tgg	1	G	A
TCGA-13-0887	BIN2	ENST00000267012	p.E92K	c.274G>A	Verified	AGC	GAG	TGG	1	G	A
TCGA-23-1120	BMPR2	ENST00000374580	p.L758L	c.2274G>A	Verified	agt	ttg	cct	3	G	A
TCGA-09-1665	BNC1	ENST00000345382	p.G391R	c.1171G>A	Verified	GAA	GGG	TGT	1	G	A
TCGA-04-1356	BOC	ENST00000355385	p.G50R	c.148G>A	Verified	CCC	GGA	GGC	1	G	A
TCGA-61-2094	BPIL1	ENST00000170150	p.G395D	c.1184G>A	Unverified	GTG	GGC	TTC	2	G	A
TCGA-29-1783	BRIP1	ENST00000259008	p.A395T	c.1183G>A	Unverified	GAA	GCT	CAT	1	G	A
TCGA-29-1783	BRIP1_ENST	ENST00000259008	p.A395T	c.1183G>A	Unverified	GAA	GCT	CAT	1	G	A
TCGA-61-1738	BRPF3	ENST00000357641	p.R257H	c.770G>A	Unverified	TGC	CGC	TGC	2	G	A
TCGA-61-1738	BRPF3	ENST00000357641	p.R257H	c.770G>A	Unverified	TGC	CGC	TGC	2	G	A
TCGA-04-1342	BRSK1	ENST00000309383	p.G327D	c.980G>A	Verified	CTG	GGC	TGC	2	G	A
TCGA-24-1844	BRWD3	ENST00000373275	p.R1287K	c.3860G>A	Unverified	GGA	AGA	AAG	2	G	A
TCGA-24-2024	BSN	ENST00000296452	p.R3591H	c.10772G>A	Verified	GCC	CGC	TCT	2	G	A
TCGA-13-1509	BSN	ENST00000296452	p.T2676T	c.8028G>A	Verified	CAG	ACG	GAG	3	G	A
TCGA-04-1347	BSND	ENST00000371265	p.Q95Q	c.285G>A	Verified	CCC	CAG	CCG	3	G	A
TCGA-29-1761	BTBD11	ENST00000280758	p.A784T	c.2350G>A	Unverified	TGC	GCC	AGC	1	G	A
TCGA-29-1763	BTBD7	ENST00000334746	p.E890E	c.2670G>A	Unverified	CCA	GAG	CTT	3	G	A
TCGA-24-1843	BTNL2	ENST00000374993	p.V224I	c.670G>A	Unverified	CCC	GTC	CTC	1	G	A
TCGA-29-1766	BUB1	ENST00000302759	p.?	c.1616+1G>A	Unverified	tat	gga	tta	2	G	A
TCGA-61-1906	BZW1	ENST00000353957	p.A67T	c.199G>A	Unverified	TAT	GCA	GAA	1	G	A
TCGA-61-1906	BZW1	ENST00000353957	p.A67T	c.199G>A	Unverified	TAT	GCA	GAA	1	G	A
TCGA-61-1906	BZW1_ENST	ENST00000452790	p.A99T	c.295G>A	Unverified	TAT	GCA	GAA	1	G	A
TCGA-61-1906	BZW1_ENST	ENST00000452790	p.A99T	c.295G>A	Unverified	TAT	GCA	GAA	1	G	A
TCGA-04-1638	C10orf58	ENST00000372188	p.R155H	c.464G>A	Unverified	ATC	CGT	CTG	2	G	A

TCGA-29-1777	C10orf71_EI	ENST00000323868	p.E28K	c.82G>A	Unverified	AGG	GAG	GTG	1	G	A
TCGA-29-1777	C10orf71_EI	ENST00000374144	p.E28K	c.82G>A	Unverified	AGG	GAG	GTG	1	G	A
TCGA-24-1422	C10orf71_EI	ENST00000374144	p.Q123Q	c.369G>A	Verified	GTC	CAG	AGG	3	G	A
TCGA-61-2012	C11orf41	ENST00000265654	p.R1106Q	c.3317G>A	Unverified	CGG	CGG	TTT	2	G	A
TCGA-24-1422	C11orf57	ENST00000280352	p.R274H	c.821G>A	Verified	AAA	CGC	ACA	2	G	A
TCGA-61-2109	C12orf29	ENST00000356891	p.A166T	c.496G>A	Unverified	ATT	GCC	CTG	1	G	A
TCGA-24-1850	C12orf32	ENST00000303648	p.Q143Q	c.429G>A	Unverified	GTG	CAG	GCA	3	G	A
TCGA-20-0991	C12orf34	ENST00000358906	p.E29K	c.85G>A	Verified	TGC	GAG	GCG	1	G	A
TCGA-09-2051	C12orf51_EI	ENST00000377560_v62	p.W278*	c.834G>A	Unverified	ATC	TGG	TCT	3	G	A
TCGA-24-1474	C12orf76	ENST00000309050	p.A126A	c.378G>A	Unverified	TCG	GCG	TCG	3	G	A
TCGA-61-2095	C15orf2	ENST00000329468	p.A80T	c.238G>A	Unverified	GCC	GCC	CCT	1	G	A
TCGA-13-0905	C15orf38	ENST00000357484	p.S17N	c.50G>A	Unverified	CAG	AGC	GTC	2	G	A
TCGA-25-2391	C15orf40_EI	ENST00000451195	p.R3R	c.9G>A	Unverified	CTG	CGG	CTC	3	G	A
TCGA-04-1331	C15orf42	ENST00000268138	p.V803I	c.2407G>A	Verified	GGT	GTC	CTT	1	G	A
TCGA-13-1510	C15orf42	ENST00000268138	p.E1184K	c.3550G>A	Unverified	GGA	GAA	GGT	1	G	A
TCGA-13-0920	C16orf58	ENST00000327237	p.G262E	c.785G>A	Verified	CTT	GGA	TGT	2	G	A
TCGA-24-2024	C17orf101	ENST00000329197	p.G83G	c.249G>A	Verified	GCA	GGG	AGA	3	G	A
TCGA-24-1431	C17orf39	ENST00000268719	p.M233I	c.699G>A	Unverified	TTC	ATG	AGG	3	G	A
TCGA-24-1845	C17orf58	ENST00000334461	p.R14Q	c.41G>A	Unverified	TTC	CGA	GTC	2	G	A
TCGA-24-1845	C17orf58_EI	ENST00000449250	p.R14Q	c.41G>A	Unverified	TTC	CGA	GTC	2	G	A
TCGA-30-1714	C17orf97	ENST00000360127	p.E350K	c.1048G>A	Unverified	CCC	GAG	GCC	1	G	A
TCGA-30-1714	C17orf97_EI	ENST00000360127	p.E350K	c.1048G>A	Unverified	CCC	GAG	GCC	1	G	A
TCGA-29-1703	C19orf28	ENST00000398558	p.G536R	c.1606G>A	Unverified	TGG	GGG	ACC	1	G	A
TCGA-24-1469	C19orf45	ENST00000361270	p.L378L	c.1134G>A	Unverified	CAC	TTG	CAG	3	G	A
TCGA-24-1469	C19orf45	ENST00000361270	p.Q371Q	c.1113G>A	Unverified	GTG	CAG	AAA	3	G	A
TCGA-24-1464	C19orf48	ENST00000391812	p.G24G	c.72G>A	Verified	CCA	GGG	CCC	3	G	A
TCGA-30-1857	C1orf112	ENST00000286031	p.E603K	c.1807G>A	Unverified	ATC	GAA	GTT	1	G	A
TCGA-09-1674	C1orf114	ENST00000367805	p.S404N	c.1211G>A	Unverified	AAC	AGT	AGA	2	G	A
TCGA-29-1690	C1orf172	ENST00000320567	p.R310H	c.929G>A	Unverified	AGC	CGT	GCT	2	G	A
TCGA-20-1687	C1orf192	ENST00000367974	p.M70I	c.210G>A	Unverified	CAA	ATG	CCT	3	G	A
TCGA-04-1342	C1orf85	ENST00000362007	p.V81I	c.241G>A	Verified	GTG	GTA	ATG	1	G	A
TCGA-23-2078	C1orf9	ENST00000263688	p.E52K	c.154G>A	Verified	AAT	GAA	GAT	1	G	A
TCGA-23-1022	C1QTNF1	ENST00000311661	p.S61N	c.182G>A	Verified	AAG	AGC	CAC	2	G	A
TCGA-13-0885	C20orf185	ENST00000375494	p.A362T	c.1084G>A	Verified	CCA	GCC	AAC	1	G	A

TCGA-13-0885	C20orf185	ENST00000375494	p.M125I	c.375G>A	Verified	GGC	ATG	CAT	3	G	A
TCGA-04-1361	C20orf70	ENST00000253362	p.V60I	c.178G>A	Verified	AAG	GTC	GAC	1	G	A
TCGA-04-1361	C20orf70	ENST00000253362	p.V60I	c.178G>A	Verified	AAG	GTC	GAC	1	G	A
TCGA-61-1914	C21orf2	ENST00000339818	p.R133H	c.398G>A	Unverified	TCC	CGT	GCA	2	G	A
TCGA-13-0762	C22orf32	ENST00000331479	p.R52H	c.155G>A	Verified	ACC	CGC	AGC	2	G	A
TCGA-13-0762	C22orf32	ENST00000331479	p.R52H	c.155G>A	Verified	ACC	CGC	AGC	2	G	A
TCGA-61-1998	C2CD2	ENST00000380486	p.E381E	c.1143G>A	Unverified	GCA	GAG	TTC	3	G	A
TCGA-61-1899	C2CD2	ENST00000380486	p.P559P	c.1677G>A	Unverified	GCC	CCG	CCA	3	G	A
TCGA-25-1313	C2orf67	ENST00000281772	p.R762Q	c.2285G>A	Verified	CGA	CGG	AGA	2	G	A
TCGA-13-0791	C2orf85	ENST00000343216	p.G503S	c.1507G>A	Verified	CAA	GGC	TAT	1	G	A
TCGA-61-2094	C3orf20	ENST00000253697	p.E652K	c.1954G>A	Unverified	CGC	GAG	GGG	1	G	A
TCGA-24-1422	C3orf25	ENST00000505956	p.D288N	c.862G>A	Verified	AGA	GAT	TCC	1	G	A
TCGA-25-1326	C3orf64	ENST00000295571	p.C79Y	c.236G>A	Verified	TAC	TGC	TGG	2	G	A
TCGA-61-1738	C4orf21	ENST00000309071	p.W559*	c.1677G>A	Unverified	AGT	TGG	GTA	3	G	A
TCGA-61-1738	C4orf21	ENST00000309071	p.W559*	c.1677G>A	Unverified	AGT	TGG	GTA	3	G	A
TCGA-61-1738	C4orf21_ENS	ENST00000505019	p.W559*	c.1677G>A	Unverified	AGT	TGG	GTA	3	G	A
TCGA-61-1738	C4orf21_ENS	ENST00000505019	p.W559*	c.1677G>A	Unverified	AGT	TGG	GTA	3	G	A
TCGA-24-1846	C5	ENST00000223642	p.?	c.3486+1G>A	Unverified	CTG	GTG	AAA	3	G	A
TCGA-13-0900	C6	ENST00000263413	p.T702T	c.2106G>A	Verified	CGG	ACG	GAG	3	G	A
TCGA-29-1691	C6orf138	ENST00000339488	p.E67K	c.199G>A	Unverified	CCC	GAG	GGC	1	G	A
TCGA-24-1847	C6orf138	ENST00000339488	p.Q206Q	c.618G>A	Unverified	CTC	CAG	GAG	3	G	A
TCGA-20-0991	C6orf174	ENST00000368268	p.E657E	c.1971G>A	Verified	ACG	GAG	CTG	3	G	A
TCGA-30-1714	C6orf222	ENST00000437635	p.E137K	c.409G>A	Unverified	CTG	GAA	GCA	1	G	A
TCGA-36-1577	C6orf27	ENST00000375688	p.R835Q	c.2504G>A	Verified	GAC	CGG	CAC	2	G	A
TCGA-29-1781	C7orf45	ENST00000297819	p.R131H	c.392G>A	Unverified	AGG	CGC	CAG	2	G	A
TCGA-13-0916	C8A	ENST00000361249	p.R59Q	c.176G>A	Verified	TAC	CGA	CAC	2	G	A
TCGA-13-0761	C8orf80	ENST00000341513	p.G679S	c.2035G>A	Unverified	ACG	GGC	AAA	1	G	A
TCGA-13-0761	C8orf80	ENST00000341513	p.G679S	c.2035G>A	Unverified	ACG	GGC	AAA	1	G	A
TCGA-61-1740	C8orf80	ENST00000341513	p.G793R	c.2377G>A	Unverified	CCC	GGG	ACA	1	G	A
TCGA-61-1740	C8orf80_ENS	ENST00000413272	p.G793R	c.2377G>A	Unverified	CCC	GGG	ACA	1	G	A
TCGA-04-1342	C9orf114	ENST00000361256	p.E111K	c.331G>A	Unverified	GAT	GAG	ATC	1	G	A
TCGA-29-1769	C9orf119	ENST00000320188	p.V206M	c.616G>A	Unverified	GAT	GTG	GGG	1	G	A
TCGA-13-1507	C9orf135	ENST00000377197	p.K40K	c.120G>A	Verified	CAC	AAG	AAA	3	G	A
TCGA-13-0793	C9orf171	ENST00000343036	p.R161H	c.482G>A	Verified	AAC	CGC	GGG	2	G	A

TCGA-23-1022	C9orf48	ENST00000403891	p.C756Y	c.2267G>A	Verified	AGC	TGC	TCT	2	G	A
TCGA-29-1702	C9orf66	ENST00000382387	p.G241E	c.722G>A	Unverified	CCC	GGG	GCC	2	G	A
TCGA-30-1718	C9orf79	ENST00000325643	p.A235T	c.703G>A	Unverified	CCT	GCA	ACC	1	G	A
TCGA-61-2102	CA10	ENST00000285273	p.M98I	c.294G>A	Verified	ACC	ATG	TAC	3	G	A
TCGA-04-1362	CA9	ENST00000378357	p.A276T	c.826G>A	Verified	TTG	GCC	GCC	1	G	A
TCGA-13-0755	CA9	ENST00000378357	p.L65L	c.195G>A	Verified	GAT	CTG	CCC	3	G	A
TCGA-24-1563	CABIN1	ENST00000398319	p.G1281G	c.3843G>A	Verified	TCT	GGG	GTT	3	G	A
TCGA-04-1530	CABLES1	ENST00000256925	p.A541T	c.1621G>A	Unverified	ATG	GCC	TTC	1	G	A
TCGA-29-1775	CABP1_ENST	ENST00000288616	p.Q49Q	c.147G>A	Unverified	GCC	CAG	AAC	3	G	A
TCGA-24-1474	CACHD1	ENST00000290039	p.M1090I	c.3270G>A	Verified	CGT	ATG	TCC	3	G	A
TCGA-23-1123	CACNA1A_E	ENST00000357018	p.V1953M	c.5857G>A	Verified	ACC	GTG	GGG	1	G	A
TCGA-23-1123	CACNA1A_E	ENST00000357018	p.V1953M	c.5857G>A	Verified	ACC	GTG	GGG	1	G	A
TCGA-30-1718	CACNA1B	ENST00000371372	p.Q1581Q	c.4743G>A	Unverified	CGC	CAG	GGC	3	G	A
TCGA-10-0930	CACNA1D	ENST00000288139	p.A714T	c.2140G>A	Verified	CAA	GCA	CTT	1	G	A
TCGA-10-0930	CACNA1D	ENST00000288139	p.A714T	c.2140G>A	Verified	CAA	GCA	CTT	1	G	A
TCGA-09-2051	CACNA1E	ENST00000367570	p.V1019M	c.3055G>A	Unverified	CAC	GTG	GTG	1	G	A
TCGA-24-1847	CACNA1F	ENST00000376265	p.A890T	c.2668G>A	Unverified	gcc	gct	gag	1	G	A
TCGA-25-2398	CACNA1F	ENST00000376265	p.G1701R	c.5101G>A	Unverified	agg	ggg	act	1	G	A
TCGA-30-1856	CACNA1I	ENST00000404898	p.E288K	c.862G>A	Unverified	AAG	GAG	CAG	1	G	A
TCGA-30-1856	CACNA1I_EN	ENST00000402142	p.E288K	c.862G>A	Unverified	AAG	GAG	CAG	1	G	A
TCGA-04-1356	CACNA1S	ENST00000362061	p.E747K	c.2239G>A	Verified	GAG	GAA	GAT	1	G	A
TCGA-23-2078	CACNA1S	ENST00000362061	p.A157T	c.469G>A	Unverified	GGA	GCC	GGC	1	G	A
TCGA-13-0916	CACNA2D1	ENST00000356860	p.G772E	c.2315G>A	Verified	AGT	GGA	CCT	2	G	A
TCGA-29-1703	CACNA2D3	ENST00000474759	p.A402T	c.1204G>A	Unverified	GCT	GCG	TTT	1	G	A
TCGA-61-1740	CACNG2	ENST00000300105	p.A159T	c.475G>A	Unverified	TCT	GCC	AAT	1	G	A
TCGA-13-1501	CACNG6	ENST00000252729	p.W248*	c.744G>A	Verified	CCC	TGG	GGG	3	G	A
TCGA-13-1501	CACNG6	ENST00000252729	p.W248*	c.744G>A	Verified	CCC	TGG	GGG	3	G	A
TCGA-24-1850	CADM4	ENST00000222374	p.P279P	c.837G>A	Unverified	CTG	CCG	GGT	3	G	A
TCGA-04-1649	CALCRL	ENST00000392370	p.R417H	c.1250G>A	Unverified	CTT	CGT	AGT	2	G	A
TCGA-13-0760	CAMK2B	ENST00000395749	p.R275H	c.824G>A	Verified	caa	cgc	tcc	2	G	A
TCGA-29-1699	CAMK2D	ENST00000342666	p.M443I	c.1329G>A	Unverified	gga	atg	cca	3	G	A
TCGA-23-1123	CAMKV	ENST00000477224	p.R92H	c.275G>A	Verified	ACC	CGC	AAG	2	G	A
TCGA-23-1123	CAMKV	ENST00000477224	p.R92H	c.275G>A	Verified	ACC	CGC	AAG	2	G	A
TCGA-23-1123	CAMKV_ENS	ENST00000477224	p.R92H	c.275G>A	Verified	ACC	CGC	AAG	2	G	A

TCGA-23-1123	CAMKV_ENS	ENST00000477224	p.R92H	c.275G>A	Verified	ACC	CGC	AAG	2	G	A
TCGA-24-1604	CAMKV_ENS	ENST00000477224	p.E19E	c.57G>A	Verified	TCG	GAG	GTG	3	G	A
TCGA-23-1123	CAMTA1	ENST00000303635	p.V274M	c.820G>A	Verified	AGC	GTG	CAT	1	G	A
TCGA-23-1123	CAMTA1	ENST00000303635	p.V274M	c.820G>A	Verified	AGC	GTG	CAT	1	G	A
TCGA-29-1699	CAP2	ENST00000229922	p.M53I	c.159G>A	Unverified	CTG	ATG	GAC	3	G	A
TCGA-13-0916	CAPN1	ENST00000279247	p.L112L	c.336G>A	Verified	GCA	CTG	GGG	3	G	A
TCGA-61-1914	CAPN6	ENST00000324068	p.D365N	c.1093G>A	Unverified	gat	gat	ccc	1	G	A
TCGA-04-1331	CAPRIN2	ENST00000251071	p.G80G	c.240G>A	Verified	GAG	GGG	AAT	3	G	A
TCGA-24-1843	CAPS2	ENST00000328705	p.R162Q	c.485G>A	Unverified	CTC	CGA	ATC	2	G	A
TCGA-24-1843	CAPS2_ENST	ENST00000409445	p.R394Q	c.1181G>A	Unverified	CTC	CGA	ATC	2	G	A
TCGA-24-2280	CARD11	ENST00000396946	p.G924G	c.2772G>A	Unverified	TCG	GGG	AGT	3	G	A
TCGA-29-1777	CARM1	ENST00000327064	p.G386S	c.1156G>A	Unverified	CAC	GGC	CTG	1	G	A
TCGA-61-1914	CARM1	ENST00000327064	p.D341N	c.1021G>A	Unverified	GTG	GAC	ACA	1	G	A
TCGA-13-0793	CASP8AP2	ENST00000237177	p.L198L	c.594G>A	Verified	AAT	CTG	GAA	3	G	A
TCGA-13-1497	CASR	ENST00000296154	p.R66H	c.197G>A	Verified	TTC	CGT	GGG	2	G	A
TCGA-25-1313	CASR	ENST00000296154	p.R678H	c.2033G>A	Verified	TGC	CGC	CTG	2	G	A
TCGA-61-2102	CAST	ENST00000309190	p.R288H	c.863G>A	Verified	CTC	CGC	TCA	2	G	A
TCGA-13-0795	CAT	ENST00000241052	p.Q53Q	c.159G>A	Verified	gtt	cag	gat	3	G	A
TCGA-13-1489	CBY1	ENST00000216029	p.L24L	c.72G>A	Verified	AAC	CTG	CAT	3	G	A
TCGA-29-1776	CC2D2A	ENST00000389652	p.P603P	c.1809G>A	Unverified	GTC	CCG	GAG	3	G	A
TCGA-29-1776	CC2D2A_EN	ENST00000424120	p.P652P	c.1956G>A	Unverified	GTC	CCG	GAG	3	G	A
TCGA-24-1850	CCDC104	ENST00000349456	p.E76K	c.226G>A	Unverified	AAT	GAA	GAT	1	G	A
TCGA-13-1497	CCDC105	ENST00000292574	p.R462H	c.1385G>A	Verified	GTG	CGC	CTG	2	G	A
TCGA-61-2102	CCDC126	ENST00000307471	p.S136S	c.408G>A	Verified	GTC	TCG	GGC	3	G	A
TCGA-23-1114	CCDC129	ENST00000407970	p.Q63Q	c.189G>A	Unverified	ATT	CAG	CAG	3	G	A
TCGA-09-2051	CCDC136	ENST00000397697	p.E483E	c.1449G>A	Unverified	CAG	GAG	ATG	3	G	A
TCGA-24-2290	CCDC157_EI	ENST00000405659	p.G33R	c.97G>A	Verified	GCC	GGG	CCT	1	G	A
TCGA-24-2290	CCDC157_EI	ENST00000405659	p.G33R	c.97G>A	Verified	GCC	GGG	CCT	1	G	A
TCGA-25-1318	CCDC158	ENST00000388914	p.E668K	c.2002G>A	Verified	AGT	GAA	TTA	1	G	A
TCGA-25-1318	CCDC158	ENST00000388914	p.E668K	c.2002G>A	Verified	AGT	GAA	TTA	1	G	A
TCGA-24-1470	CCDC165	ENST00000359865	p.K1010K	c.3030G>A	Verified	TCC	AAG	CTG	3	G	A
TCGA-61-2102	CCDC165	ENST00000359865	p.S1018S	c.3054G>A	Verified	TGC	TCG	GCC	3	G	A
TCGA-04-1655	CCDC33	ENST00000398814	p.R186Q	c.557G>A	Unverified	CTC	CGG	GGA	2	G	A
TCGA-23-1117	CCDC33	ENST00000398814	p.L497L	c.1491G>A	Verified	GAG	CTG	GAT	3	G	A

TCGA-23-1117	CCDC33	ENST00000398814	p.L497L	c.1491G>A	Verified	GAG	CTG	GAT	3	G	A
TCGA-04-1655	CCDC33_ENS	ENST00000321288	p.R389Q	c.1166G>A	Unverified	CTC	CGG	GGA	2	G	A
TCGA-24-1104	CCDC60	ENST00000327554	p.R131H	c.392G>A	Verified	CGT	CGC	CCA	2	G	A
TCGA-13-0760	CCDC62	ENST00000341952	p.D576N	c.1726G>A	Verified	CAA	GAT	TCC	1	G	A
TCGA-20-0991	CCDC62	ENST00000341952	p.E228K	c.682G>A	Verified	AAT	GAA	AAG	1	G	A
TCGA-13-0762	CCDC64	ENST00000397558	p.L170L	c.510G>A	Verified	GAG	CTG	GAG	3	G	A
TCGA-13-0762	CCDC64	ENST00000397558	p.L170L	c.510G>A	Verified	GAG	CTG	GAG	3	G	A
TCGA-13-0900	CCDC67	ENST00000298050	p.K149K	c.447G>A	Unverified	GCA	AAG	TCA	3	G	A
TCGA-24-1847	CCDC70	ENST00000242819	p.G85D	c.254G>A	Unverified	CTG	GGT	TTT	2	G	A
TCGA-25-2393	CCDC82	ENST00000278520	p.V17M	c.49G>A	Verified	CAC	GTG	CCT	1	G	A
TCGA-23-1029	CCDC86	ENST00000227520	p.Q349Q	c.1047G>A	Unverified	CTG	CAG	AAG	3	G	A
TCGA-61-2109	CCDC88C	ENST00000389857	p.R1569Q	c.4706G>A	Unverified	TCG	CGG	CCA	2	G	A
TCGA-24-1616	CCNA1	ENST00000255465	p.C452Y	c.1355G>A	Verified	ctg	tgt	gtg	2	G	A
TCGA-13-1501	CCNB3	ENST00000376042	p.R108Q	c.323G>A	Verified	AAG	CGG	AAT	2	G	A
TCGA-13-1501	CCNB3	ENST00000376042	p.R108Q	c.323G>A	Verified	AAG	CGG	AAT	2	G	A
TCGA-04-1652	CCNB3	ENST00000376042	p.L649L	c.1947G>A	Unverified	ACC	TTG	CAG	3	G	A
TCGA-04-1652	CCNB3	ENST00000376042	p.L649L	c.1947G>A	Unverified	ACC	TTG	CAG	3	G	A
TCGA-13-1501	CCNB3_ENST	ENST00000376042	p.R108Q	c.323G>A	Verified	AAG	CGG	AAT	2	G	A
TCGA-13-1501	CCNB3_ENST	ENST00000376042	p.R108Q	c.323G>A	Verified	AAG	CGG	AAT	2	G	A
TCGA-04-1652	CCNB3_ENST	ENST00000376042	p.L649L	c.1947G>A	Unverified	ACC	TTG	CAG	3	G	A
TCGA-04-1652	CCNB3_ENST	ENST00000376042	p.L649L	c.1947G>A	Unverified	ACC	TTG	CAG	3	G	A
TCGA-25-1326	CCNDBP1	ENST00000300213	p.E50E	c.150G>A	Verified	CGA	GAG	ATG	3	G	A
TCGA-61-1906	CCNDBP1	ENST00000300213	p.T68T	c.204G>A	Unverified	GCC	ACG	ACT	3	G	A
TCGA-61-1906	CCNDBP1	ENST00000300213	p.T68T	c.204G>A	Unverified	GCC	ACG	ACT	3	G	A
TCGA-61-1906	CCNL1	ENST00000295926	p.K486K	c.1458G>A	Unverified	gcc	aag	aaa	3	G	A
TCGA-61-1906	CCNL1	ENST00000295926	p.K486K	c.1458G>A	Unverified	gcc	aag	aaa	3	G	A
TCGA-24-2262	CCNL2	ENST00000400809	p.V45V	c.135G>A	Verified	GGG	GTG	CTC	3	G	A
TCGA-13-1501	CCNT2	ENST00000264157	p.S613N	c.1838G>A	Verified	TCT	AGC	TCC	2	G	A
TCGA-13-1501	CCNT2	ENST00000264157	p.S613N	c.1838G>A	Verified	TCT	AGC	TCC	2	G	A
TCGA-61-1899	CCR3	ENST00000357422	p.A290T	c.868G>A	Unverified	atc	gcc	tac	1	G	A
TCGA-13-0795	CCR6	ENST00000400926	p.D10N	c.28G>A	Verified	agc	gat	gtt	1	G	A
TCGA-29-1762	CCR8	ENST00000326306	p.R328K	c.983G>A	Unverified	gga	aga	caa	2	G	A
TCGA-04-1347	CCT4	ENST00000394440	p.V397V	c.1191G>A	Verified	CTG	GTG	ATT	3	G	A
TCGA-29-1763	CCT5	ENST00000280326	p.G216S	c.646G>A	Unverified	GTG	GGC	GGC	1	G	A

TCGA-13-0793	CD163	ENST00000359156	p.G1014D	c.3041G>A	Verified	TGG	GGC	CAT	2	G	A
TCGA-13-0916	CD163	ENST00000359156	p.G873E	c.2618G>A	Verified	AAA	GGG	AAA	2	G	A
TCGA-24-2288	CD209	ENST00000315599	p.A382T	c.1144G>A	Unverified	GCA	GCC	TCC	1	G	A
TCGA-24-2288	CD209_ENS	ENST00000315599	p.A382T	c.1144G>A	Unverified	GCA	GCC	TCC	1	G	A
TCGA-13-0923	CD244	ENST00000368034	p.V269I	c.805G>A	Verified	GAT	GTC	AAG	1	G	A
TCGA-13-1505	CD82	ENST00000227155	p.V62M	c.184G>A	Verified	GGC	GTG	GGG	1	G	A
TCGA-24-1431	CD83	ENST00000379153	p.Q71Q	c.213G>A	Unverified	GGA	CAG	CAC	3	G	A
TCGA-23-1110	CD8A	ENST00000283635	p.D104N	c.310G>A	Verified	AGC	GAC	TTC	1	G	A
TCGA-04-1651	CD97	ENST00000242786	p.R187H	c.560G>A	Unverified	TGC	CGC	TGC	2	G	A
TCGA-04-1651	CD97	ENST00000242786	p.R187H	c.560G>A	Unverified	TGC	CGC	TGC	2	G	A
TCGA-29-1702	CDC20	ENST00000310955	p.R471H	c.1412G>A	Unverified	TGG	CGC	TGT	2	G	A
TCGA-29-1777	CDC23	ENST00000394886	p.A517T	c.1549G>A	Unverified	CTG	GCC	CAG	1	G	A
TCGA-04-1655	CDH10	ENST00000264463	p.A727T	c.2179G>A	Unverified	ACC	GCA	CCC	1	G	A
TCGA-13-1499	CDH10	ENST00000264463	p.T258T	c.774G>A	Verified	ATC	ACG	CTG	3	G	A
TCGA-09-0369	CDH10	ENST00000264463	p.T694T	c.2082G>A	Verified	GAA	ACG	TTA	3	G	A
TCGA-09-0369	CDH10	ENST00000264463	p.T694T	c.2082G>A	Verified	GAA	ACG	TTA	3	G	A
TCGA-04-1338	CDH18	ENST00000382275	p.A195T	c.583G>A	Verified	AGC	GCT	CGG	1	G	A
TCGA-13-1498	CDH18	ENST00000382275	p.V33V	c.99G>A	Verified	AAG	GTG	ATG	3	G	A
TCGA-13-0760	CDH26	ENST00000348616	p.G583E	c.1748G>A	Verified	CAG	GGA	CTT	2	G	A
TCGA-09-2050	CDH5	ENST00000341529	p.A31T	c.91G>A	Verified	CCT	GCC	CAA	1	G	A
TCGA-04-1652	CDH9	ENST00000231021	p.E681E	c.2043G>A	Unverified	CCA	GAG	GCA	3	G	A
TCGA-04-1652	CDH9	ENST00000231021	p.E681E	c.2043G>A	Unverified	CCA	GAG	GCA	3	G	A
TCGA-13-0916	CDHR2	ENST00000261944	p.D387N	c.1159G>A	Verified	ATC	GAT	GAC	1	G	A
TCGA-13-0906	CDK1_ENST(	ENST00000395284	p.?	c.37+1G>A	Verified	GAA	GGT	ACC	1	G	A
TCGA-04-1343	CDK15	ENST00000410091	p.V210M	c.628G>A	Verified	gtc	gtg	acc	1	G	A
TCGA-24-1423	CDK17	ENST00000261211	p.M85I	c.255G>A	Verified	TTC	ATG	GCA	3	G	A
TCGA-24-2290	CDKN2C	ENST00000262662	p.D64N	c.190G>A	Verified	ccc	gat	ttg	1	G	A
TCGA-24-2290	CDKN2C	ENST00000262662	p.D64N	c.190G>A	Verified	ccc	gat	ttg	1	G	A
TCGA-29-1705	CDRT15L2	ENST00000399044	p.G58D	c.173G>A	Unverified	GCT	GGC	CAG	2	G	A
TCGA-13-0791	CEACAM6	ENST00000199764	p.P186P	c.558G>A	Verified	CTC	CCG	GTC	3	G	A
TCGA-24-1604	CELA3B	ENST00000337107	p.V252V	c.756G>A	Verified	ACG	GTG	TTC	3	G	A
TCGA-23-1029	CELF1	ENST00000358597	p.G353E	c.1058G>A	Unverified	ACC	GGG	AGC	2	G	A
TCGA-04-1338	CELF3	ENST00000290583	p.A150A	c.450G>A	Verified	CAG	GCG	GCC	3	G	A
TCGA-24-1845	CELSR2	ENST00000271332	p.A1595T	c.4783G>A	Unverified	GAC	GCG	TTC	1	G	A

TCGA-20-1685	CELSR2	ENST00000271332	p.K795K	c.2385G>A	Unverified	CAG	AAG	TCC	3	G	A
TCGA-09-0369	CELSR3	ENST00000164024	p.V749I	c.2245G>A	Verified	GAC	GTT	AAT	1	G	A
TCGA-09-0369	CELSR3	ENST00000164024	p.V749I	c.2245G>A	Verified	GAC	GTT	AAT	1	G	A
TCGA-13-1489	CELSR3	ENST00000164024	p.G706S	c.2116G>A	Verified	ACT	GGC	TGG	1	G	A
TCGA-04-1337	CELSR3	ENST00000164024	p.S2382N	c.7145G>A	Unverified	AGC	AGC	AGC	2	G	A
TCGA-29-1763	CELSR3	ENST00000164024	p.R3201Q	c.9602G>A	Unverified	TCT	CGG	GAG	2	G	A
TCGA-24-2288	CELSR3	ENST00000164024	p.G292G	c.876G>A	Unverified	CCC	GGG	CCG	3	G	A
TCGA-29-1699	CEP250	ENST00000397527	p.R2376H	c.7127G>A	Unverified	ACC	CGC	TCA	2	G	A
TCGA-61-2094	CERKL	ENST00000410087	p.V510I	c.1528G>A	Unverified	GAG	GTC	CAT	1	G	A
TCGA-23-1029	CFH	ENST00000367429	p.P1166P	c.3498G>A	Unverified	cat	ccg	tgt	3	G	A
TCGA-61-1738	CFHR1	ENST00000320493	p.Q238Q	c.714G>A	Unverified	TGC	CAG	AAC	3	G	A
TCGA-61-1738	CFHR1	ENST00000320493	p.Q238Q	c.714G>A	Unverified	TGC	CAG	AAC	3	G	A
TCGA-04-1337	CFP	ENST00000396992	p.E4K	c.10G>A	Unverified	aca	gag	gga	1	G	A
TCGA-13-0920	CGN	ENST00000271636	p.E1137K	c.3409G>A	Verified	GAG	GAG	CAG	1	G	A
TCGA-13-0760	CGN	ENST00000271636	p.E1051E	c.3153G>A	Verified	CTT	GAG	TCC	3	G	A
TCGA-29-1695	CGNL1	ENST00000281282	p.A592T	c.1774G>A	Unverified	CGA	GCA	GCT	1	G	A
TCGA-13-1512	CHAD	ENST00000258969	p.R312Q	c.935G>A	Verified	CTT	CGG	CGG	2	G	A
TCGA-29-1764	CHAT	ENST00000337653	p.G130G	c.390G>A	Unverified	TCT	GGG	CTG	3	G	A
TCGA-24-1845	CHCHD1	ENST00000372833	p.L21L	c.63G>A	Unverified	GTG	CTG	AAG	3	G	A
TCGA-24-1422	CHD4_ENST	ENST00000309577	p.G616E	c.1847G>A	Verified	TAT	GGG	ATA	2	G	A
TCGA-13-0755	CHD6	ENST00000373233	p.V1289M	c.3865G>A	Verified	ggc	gtg	ttc	1	G	A
TCGA-61-1737	CHD7	ENST00000423902	p.R809H	c.2426G>A	Unverified	AGT	CGT	TCA	2	G	A
TCGA-61-1737	CHD7_ENST	ENST00000423902	p.R809H	c.2426G>A	Unverified	AGT	CGT	TCA	2	G	A
TCGA-04-1338	CHD8	ENST00000399982	p.C958Y	c.2873G>A	Verified	aat	tgc	aag	2	G	A
TCGA-13-0760	CHD8	ENST00000399982	p.R1092H	c.3275G>A	Verified	ttg	cgc	aag	2	G	A
TCGA-09-0369	CHEK2	ENST00000328354	p.R519Q	c.1556G>A	Unverified	agt	cga	aag	2	G	A
TCGA-09-0369	CHEK2	ENST00000328354	p.R519Q	c.1556G>A	Unverified	agt	cga	aag	2	G	A
TCGA-13-0714	CHEK2	ENST00000328354	p.R519Q	c.1556G>A	Unverified	agt	cga	aag	2	G	A
TCGA-13-0760	CHEK2	ENST00000328354	p.R519Q	c.1556G>A	Unverified	agt	cga	aag	2	G	A
TCGA-13-0762	CHEK2	ENST00000328354	p.R519Q	c.1556G>A	Unverified	agt	cga	aag	2	G	A
TCGA-13-0762	CHEK2	ENST00000328354	p.R519Q	c.1556G>A	Unverified	agt	cga	aag	2	G	A
TCGA-13-0762	CHEK2	ENST00000328354	p.R535H	c.1604G>A	Unverified	aag	cgc	cca	2	G	A
TCGA-13-0762	CHEK2	ENST00000328354	p.R535H	c.1604G>A	Unverified	aag	cgc	cca	2	G	A
TCGA-24-1103	CHIA	ENST00000430615	p.E197E	c.591G>A	Verified	TAC	GAG	ATC	3	G	A

TCGA-13-0792	CHMP4A	ENST00000347519	p.E254K	c.760G>A	Verified	GAT	GAA	GAA	1	G	A
TCGA-13-0791	CHMP5	ENST00000223500	p.K62K	c.186G>A	Verified	GTC	AAG	CAG	3	G	A
TCGA-13-0905	CHP	ENST00000334660	p.A156T	c.466G>A	Verified	ATC	GCA	GAC	1	G	A
TCGA-24-1431	CHRD	ENST00000204604	p.G513R	c.1537G>A	Verified	GAC	GGA	GAG	1	G	A
TCGA-13-0791	CHRM2	ENST00000320658	p.R52H	c.155G>A	Verified	AAC	CGC	CAC	2	G	A
TCGA-13-0791	CHRM3	ENST00000255380	p.V443I	c.1327G>A	Verified	GAC	GTC	AAC	1	G	A
TCGA-61-2095	CHRNA9	ENST00000310169	p.G300D	c.899G>A	Unverified	ATA	GGT	AAA	2	G	A
TCGA-29-1701	CHRNB2	ENST00000368476	p.E30K	c.88G>A	Unverified	GAG	GAG	CGG	1	G	A
TCGA-61-1915	CHRNB2	ENST00000368476	p.R365Q	c.1094G>A	Unverified	CGG	CGA	CGC	2	G	A
TCGA-13-0905	CHST2	ENST00000309575	p.A372A	c.1116G>A	Verified	GCC	GCG	GGC	3	G	A
TCGA-13-0807	CIB3	ENST00000269878	p.M172I	c.516G>A	Verified	aac	atg	atc	3	G	A
TCGA-24-2267	CIB4	ENST00000288861	p.V85M	c.253G>A	Verified	gat	gtg	ctg	1	G	A
TCGA-61-2009	CILP	ENST00000261883	p.R73Q	c.218G>A	Verified	GAG	CGG	CTG	2	G	A
TCGA-23-1031	CIT	SU_CRIK	p.G1440S	c.4318G>A	Verified	tgc	ggc	ttg	1	G	A
TCGA-61-2102	CIT	SU_CRIK	p.G1935S	c.5803G>A	Verified	tcc	ggc	act	1	G	A
TCGA-04-1343	CIT	SU_CRIK	p.R713H	c.2138G>A	Verified	gag	cgt	aga	2	G	A
TCGA-20-1686	CIT	SU_CRIK	p.L2L	c.6G>A	Unverified	atg	ttg	aag	3	G	A
TCGA-25-1313	CIT_ENST00	ENST00000261833	p.P1871P	c.5613G>A	Unverified	AAC	CCG	CGC	3	G	A
TCGA-20-1686	CIT_ENST00	ENST00000261833	p.L2L	c.6G>A	Unverified	ATG	TTG	AAG	3	G	A
TCGA-20-1686	CIT_ENST00	ENST00000392521	p.L2L	c.6G>A	Unverified	ATG	TTG	AAG	3	G	A
TCGA-61-1738	CLASP1	ENST00000263710	p.E408K	c.1222G>A	Unverified	GCT	GAA	GCC	1	G	A
TCGA-61-1738	CLASP1	ENST00000263710	p.E408K	c.1222G>A	Unverified	GCT	GAA	GCC	1	G	A
TCGA-24-1850	CLCC1	ENST00000369970	p.V96I	c.286G>A	Unverified	CCT	GTT	TTT	1	G	A
TCGA-24-1850	CLCC1_ENST	ENST00000369971	p.V96I	c.286G>A	Unverified	CCT	GTT	TTT	1	G	A
TCGA-29-1769	CLCN1	ENST00000343257	p.V448V	c.1344G>A	Unverified	GCT	GTG	TGG	3	G	A
TCGA-30-1718	CLCN3	ENST00000347613	p.R95Q	c.284G>A	Unverified	GTG	CGA	GAA	2	G	A
TCGA-24-1846	CLCN6	ENST00000346436	p.R362H	c.1085G>A	Unverified	TAC	CGT	ATG	2	G	A
TCGA-61-1733	CLDN6	ENST00000396925	p.V42M	c.124G>A	Unverified	GTG	GTG	GCC	1	G	A
TCGA-61-1733	CLDN6	ENST00000396925	p.V42M	c.124G>A	Unverified	GTG	GTG	GCC	1	G	A
TCGA-29-1690	CLEC18A	ENST00000288040	p.P38P	c.114G>A	Unverified	GCT	CCG	ATG	3	G	A
TCGA-23-2077	CLEC18B	ENST00000339953	p.R165Q	c.494G>A	Unverified	GGG	CGG	CAC	2	G	A
TCGA-23-1021	CLIP3	ENST00000360535	p.A425T	c.1273G>A	Verified	GTC	GCG	GGC	1	G	A
TCGA-61-1915	CLN3	ENST00000359984	p.A103A	c.309G>A	Unverified	CTG	GCG	GAC	3	G	A
TCGA-29-1711	CLRN1	ENST00000327047	p.G68E	c.203G>A	Unverified	CAC	GGA	GAG	2	G	A

TCGA-29-1711	CLRN1	ENST00000327047	p.G68E	c.203G>A	Unverified	CAC	GGA	GAG	2	G	A
TCGA-09-1674	CMYA5	ENST00000238522	p.P1948P	c.5844G>A	Unverified	CTG	CCG	CAT	3	G	A
TCGA-09-1674	CMYA5_ENS	ENST00000446378	p.P1948P	c.5844G>A	Unverified	CTG	CCG	CAT	3	G	A
TCGA-13-0714	CNGA3	ENST00000272602	p.V529M	c.1585G>A	Verified	GCC	GTG	GTG	1	G	A
TCGA-61-1915	CNGB1	ENST00000251102	p.L1142L	c.3426G>A	Unverified	GCG	CTG	GAG	3	G	A
TCGA-24-1563	CNGB3	ENST00000320005	p.A305T	c.913G>A	Verified	GTC	GCA	TCA	1	G	A
TCGA-23-1116	CNKSR1	ENST00000361530	p.W486*	c.1457G>A	Unverified	ATG	TGG	GTG	2	G	A
TCGA-24-2280	CNTFR	ENST00000351266	p.A299T	c.895G>A	Unverified	CAC	GCT	ACG	1	G	A
TCGA-29-1703	CNTN4	ENST00000397459	p.S198S	c.594G>A	Unverified	CAC	TCG	CTA	3	G	A
TCGA-29-1703	CNTN4_ENS	ENST00000418658	p.S526S	c.1578G>A	Unverified	CAC	TCG	CTA	3	G	A
TCGA-09-1674	CNTN6	ENST00000446702	p.V894I	c.2680G>A	Unverified	CCA	GTC	AAT	1	G	A
TCGA-13-0884	CNTNAP1	ENST00000264638	p.W623*	c.1869G>A	Verified	GCG	TGG	ACA	3	G	A
TCGA-30-1856	CNTNAP2	ENST00000361727	p.A380T	c.1138G>A	Unverified	AAC	GCT	ACA	1	G	A
TCGA-61-2008	CNTNAP2	ENST00000361727	p.A667T	c.1999G>A	Verified	AGC	GCC	TCC	1	G	A
TCGA-61-1998	CNTNAP4	ENST00000339740	p.R374Q	c.1121G>A	Verified	TTT	CGA	ACT	2	G	A
TCGA-61-1895	CNTNAP4_E	ENST00000307431	p.V1009M	c.3025G>A	Unverified	TCC	GTG	ATA	1	G	A
TCGA-61-1895	CNTNAP4_E	ENST00000478060	p.V937M	c.2809G>A	Unverified	TCC	GTG	ATA	1	G	A
TCGA-13-0791	CNTNAP5	ENST00000431078	p.V1168I	c.3502G>A	Verified	TCC	GTC	CAG	1	G	A
TCGA-25-2042	COL11A1	ENST00000358392	p.E342E	c.1026G>A	Verified	AAT	GAG	CCA	3	G	A
TCGA-13-1509	COL13A1	ENST00000398969	p.K326K	c.978G>A	Verified	ATG	AAG	GGT	3	G	A
TCGA-29-1784	COL14A1	ENST00000297848	p.R844Q	c.2531G>A	Unverified	aac	cgg	ttg	2	G	A
TCGA-61-1737	COL14A1	ENST00000297848	p.V837V	c.2511G>A	Unverified	cgg	gtg	tcc	3	G	A
TCGA-24-1469	COL15A1	ENST00000375001	p.G434G	c.1302G>A	Verified	CCC	GGG	GAG	3	G	A
TCGA-23-1022	COL16A1	ENST00000373672	p.A622T	c.1864G>A	Verified	CCA	GCA	GGC	1	G	A
TCGA-30-1718	COL17A1	ENST00000353479	p.P1004P	c.3012G>A	Unverified	CCT	CCG	GGC	3	G	A
TCGA-23-1022	COL1A2	ENST00000297268	p.G937S	c.2809G>A	Verified	CCA	GGT	CGC	1	G	A
TCGA-24-2262	COL1A2	ENST00000297268	p.A843T	c.2527G>A	Verified	TTC	GCT	GGT	1	G	A
TCGA-61-2012	COL20A1	ENST00000358894	p.S49S	c.147G>A	Unverified	GAG	TCG	GAG	3	G	A
TCGA-10-0930	COL21A1	ENST00000244728	p.D176N	c.526G>A	Verified	GAA	GAT	GCC	1	G	A
TCGA-10-0930	COL21A1	ENST00000244728	p.D176N	c.526G>A	Verified	GAA	GAT	GCC	1	G	A
TCGA-24-1563	COL21A1	ENST00000244728	p.D227N	c.679G>A	Verified	CGT	GAT	GAA	1	G	A
TCGA-04-1651	COL2A1	ENST00000337299	p.V1100I	c.3298G>A	Unverified	CCC	GTC	GGT	1	G	A
TCGA-04-1651	COL2A1	ENST00000337299	p.V1100I	c.3298G>A	Unverified	CCC	GTC	GGT	1	G	A
TCGA-04-1651	COL2A1_EN:	ENST00000380518	p.V1169I	c.3505G>A	Unverified	CCC	GTC	GGT	1	G	A

TCGA-04-1651	COL2A1_EN!	ENST00000380518	p.V1169I	c.3505G>A	Unverified	CCC	GTC	GGT	1	G	A
TCGA-04-1542	COL3A1	ENST00000304636	p.G294E	c.881G>A	Verified	AAT	GGA	GCT	2	G	A
TCGA-10-0930	COL4A5	ENST00000361603	p.R1674Q	c.5021G>A	Verified	aca	cga	att	2	G	A
TCGA-10-0930	COL4A5	ENST00000361603	p.R1674Q	c.5021G>A	Verified	aca	cga	att	2	G	A
TCGA-24-2035	COL4A6	ENST00000334504	p.V1478V	c.4434G>A	Verified	CAG	GTG	CCC	3	G	A
TCGA-13-1512	COL5A1	ENST00000371817	p.D1657N	c.4969G>A	Verified	GTC	GAT	CCT	1	G	A
TCGA-13-0887	COL5A2	ENST00000374866	p.R1453Q	c.4358G>A	Verified	AAG	CGG	AAT	2	G	A
TCGA-29-1763	COL5A2	ENST00000374866	p.G795D	c.2384G>A	Unverified	GAT	GGT	GCA	2	G	A
TCGA-24-1463	COL5A3	ENST00000264828	p.T1730T	c.5190G>A	Verified	CAG	ACG	AAC	3	G	A
TCGA-29-1699	COL6A5	ENST00000265379	p.D2190N	c.6568G>A	Unverified	AAT	GAT	GGT	1	G	A
TCGA-61-1914	COL6A6	ENST00000358511	p.D964N	c.2890G>A	Unverified	AGC	GAC	AAG	1	G	A
TCGA-61-1914	COL6A6_EN!	ENST00000358511_v70	p.D964N	c.2890G>A	Unverified	AGC	GAC	AAG	1	G	A
TCGA-24-2290	COL7A1	ENST00000328333	p.L883L	c.2649G>A	Verified	TCG	CTG	AGG	3	G	A
TCGA-24-2290	COL7A1	ENST00000328333	p.L883L	c.2649G>A	Verified	TCG	CTG	AGG	3	G	A
TCGA-24-1846	COL8A1	ENST00000273342	p.R709K	c.2126G>A	Unverified	CTC	AGG	CCC	2	G	A
TCGA-23-2077	COL9A2	ENST00000372748	p.D141N	c.421G>A	Verified	GGG	GAC	CCT	1	G	A
TCGA-23-1022	COPB2	ENST00000333188	p.G855R	c.2563G>A	Verified	GAT	GGG	AAA	1	G	A
TCGA-23-1114	COPS4	ENST00000264389	p.G135E	c.404G>A	Unverified	aca	gga	caa	2	G	A
TCGA-13-1499	CORO2B	ENST00000566799	p.R113Q	c.338G>A	Verified	GTG	CGG	ATC	2	G	A
TCGA-04-1367	COX15	ENST00000016171	p.E115K	c.343G>A	Verified	caa	gag	gaa	1	G	A
TCGA-24-1474	CPAMD8	ENST00000291440	p.R1270H	c.3809G>A	Unverified	GCT	CGC	AGC	2	G	A
TCGA-20-0990	CPNE3	ENST00000198765	p.G295R	c.883G>A	Verified	GTG	GGA	GTG	1	G	A
TCGA-29-1761	CPO	ENST00000272852	p.L11L	c.33G>A	Unverified	CTT	TTG	GGG	3	G	A
TCGA-61-1737	CPSF2	ENST00000298875	p.Q133Q	c.399G>A	Unverified	TCT	CAG	ATT	3	G	A
TCGA-61-2113	CPT1A	ENST00000265641	p.S371S	c.1113G>A	Unverified	ACC	TCG	GAG	3	G	A
TCGA-24-1470	CPT1B	ENST00000395650	p.D429N	c.1285G>A	Verified	TAT	GAC	CCC	1	G	A
TCGA-24-1474	CPZ	ENST00000360986	p.R203H	c.608G>A	Unverified	TCC	CGC	TGC	2	G	A
TCGA-23-1809	CRB1	ENST00000367400	p.D730N	c.2188G>A	Unverified	CTT	GAT	GAG	1	G	A
TCGA-13-0919	CREG2	ENST00000324768	p.R279K	c.836G>A	Verified	TCA	AGG	GAG	2	G	A
TCGA-24-2290	CRHR2	ENST00000471646	p.L316L	c.948G>A	Verified	CTC	CTG	CCC	3	G	A
TCGA-24-2290	CRHR2	ENST00000471646	p.L316L	c.948G>A	Verified	CTC	CTG	CCC	3	G	A
TCGA-29-1690	CRIPAK	ENST00000324803	p.A44T	c.130G>A	Unverified	CCC	GCC	TGC	1	G	A
TCGA-09-1674	CRISP3	ENST00000263045	p.V43V	c.129G>A	Unverified	ATT	GTG	AAT	3	G	A
TCGA-13-0795	CRNKL1	ENST00000377340	p.R553K	c.1658G>A	Verified	GAG	AGG	ACA	2	G	A

TCGA-23-1124	CRTAC1	ENST00000370597	p.E534K	c.1600G>A	Verified	GAT	GAG	GAC	1	G	A
TCGA-29-1691	CSAD	ENST00000308926	p.V345M	c.1033G>A	Unverified	GAT	GTG	GCT	1	G	A
TCGA-29-1691	CSAD_ENST(	ENST00000267085	p.V372M	c.1114G>A	Unverified	GAT	GTG	GCT	1	G	A
TCGA-04-1338	CSF1	ENST00000369802	p.V135I	c.403G>A	Verified	TGC	GTC	CGA	1	G	A
TCGA-29-1784	CSF2RA	ENST00000381529	p.D181N	c.541G>A	Unverified	ctg	gat	aac	1	G	A
TCGA-13-0885	CSF2RA	ENST00000381529	p.R146H	c.437G>A	Verified	ccc	cgt	gac	2	G	A
TCGA-29-1784	CSF2RA_ENS	ENST00000355432	p.D181N	c.541G>A	Unverified	CTG	GAT	AAC	1	G	A
TCGA-29-1784	CSF2RA_ENS	ENST00000381509	p.D181N	c.541G>A	Unverified	CTG	GAT	AAC	1	G	A
TCGA-25-2042	CSF2RB	ENST00000403662	p.W514*	c.1541G>A	Unverified	CCG	TGG	GGC	2	G	A
TCGA-23-1124	CSF3	ENST00000331769	p.A10T	c.28G>A	Verified	CCA	GCC	CTG	1	G	A
TCGA-29-1763	CSMD1_ENS	ENST00000537824	p.G1928R	c.5782G>A	Unverified	ATC	GGA	GAT	1	G	A
TCGA-04-1342	CSMD2	ENST00000241312	p.T3195T	c.9585G>A	Verified	GAG	ACG	CCA	3	G	A
TCGA-23-2077	CSMD2	ENST00000241312	p.E1334E	c.4002G>A	Verified	GTG	GAG	AGC	3	G	A
TCGA-13-0793	CSMD3	ENST00000297405	p.E392K	c.1174G>A	Verified	tcc	gag	gaa	1	G	A
TCGA-13-0884	CSMD3	ENST00000297405	p.G863E	c.2588G>A	Verified	gaa	gga	ttt	2	G	A
TCGA-29-1770	CSMD3	ENST00000297405	p.R3391H	c.10172G>A	Unverified	aca	cgc	acc	2	G	A
TCGA-29-1770	CSMD3_ENS	ENST00000343508	p.R3351H	c.10052G>A	Unverified	ACA	CGC	ACC	2	G	A
TCGA-04-1530	CSNK1A1L	ENST00000379800	p.R106K	c.317G>A	Unverified	TCA	AGA	AGG	2	G	A
TCGA-30-1718	CSPG4	ENST00000308508	p.R1557H	c.4670G>A	Unverified	ttc	cgc	ttc	2	G	A
TCGA-25-1313	CSRNP3	ENST00000342316	p.R97H	c.290G>A	Verified	AGC	CGC	CAT	2	G	A
TCGA-61-1998	CSTF2T	ENST00000331173	p.M140I	c.420G>A	Verified	CAG	ATG	TTT	3	G	A
TCGA-23-1114	CTNNBIP1	ENST00000377263	p.V68V	c.204G>A	Unverified	gtg	gtg	atg	3	G	A
TCGA-25-2392	CTNNBL1	ENST00000361383	p.R522Q	c.1565G>A	Verified	atg	cga	gga	2	G	A
TCGA-25-2400	CTPS2	ENST00000443824	p.E349K	c.1045G>A	Verified	acc	gag	gac	1	G	A
TCGA-09-2049	CTSG	ENST00000216336	p.T140T	c.420G>A	Verified	GGG	ACG	CTG	3	G	A
TCGA-04-1655	CTTNBP2	ENST00000160373	p.V584V	c.1752G>A	Unverified	ACT	GTG	GCT	3	G	A
TCGA-09-2050	CUBN	ENST00000377833	p.S3124N	c.9371G>A	Verified	AGC	AGC	AAT	2	G	A
TCGA-13-0893	CUL2	ENST00000374751	p.?	c.715-1G>A	Verified	aag	gtt	cta	1	G	A
TCGA-13-0883	CUL7	ENST00000265348	p.V535M	c.1603G>A	Verified	gcc	gtg	ccc	1	G	A
TCGA-23-1111	CUL9	ENST00000252050	p.W361*	c.1082G>A	Unverified	GGG	TGG	GTC	2	G	A
TCGA-24-1423	CUX1	ENST00000292535	p.A769T	c.2305G>A	Verified	TCC	GCA	GCT	1	G	A
TCGA-23-1114	CUZD1	ENST00000368904	p.M48I	c.144G>A	Unverified	GCC	ATG	ATC	3	G	A
TCGA-30-1857	CXCR2	ENST00000318507	p.G79S	c.235G>A	Unverified	gtc	ggc	cgc	1	G	A
TCGA-29-1781	CXorf59	ENST00000378660	p.V425M	c.1273G>A	Unverified	AGG	GTG	TTT	1	G	A

TCGA-24-1422	CYCS	ENST00000305786	p.K87K	c.261G>A	Verified	att	aag	aag	3	G	A
TCGA-23-1124	CYFIP1	ENST00000313077	p.M824I	c.2472G>A	Verified	GCC	ATG	TTC	3	G	A
TCGA-09-2051	CYHR1	ENST00000306145	p.A115T	c.343G>A	Unverified	GTG	GCT	GGT	1	G	A
TCGA-20-1686	CYLC1	ENST00000329312	p.R95R	c.285G>A	Unverified	GCC	AGG	GAA	3	G	A
TCGA-20-1686	CYLC1_ENST	ENST00000329312	p.R95R	c.285G>A	Unverified	GCC	AGG	GAA	3	G	A
TCGA-61-1737	CYP11B1	ENST00000292427	p.R123H	c.368G>A	Unverified	CAT	CGT	GGG	2	G	A
TCGA-29-1695	CYP11B2	ENST00000323110	p.E74E	c.222G>A	Unverified	CAG	GAG	CTG	3	G	A
TCGA-61-1998	CYP2A13	ENST00000330436	p.G5E	c.14G>A	Verified	TCA	GGG	CTG	2	G	A
TCGA-24-1469	CYP2A6	ENST00000301141	p.M6I	c.18G>A	Verified	GGG	ATG	CTT	3	G	A
TCGA-20-1683	CYP2B7P1	ENST00000330446	p.E149K	c.445G>A	Unverified	GAC	GAG	GCT	1	G	A
TCGA-10-0930	CYP2C9	ENST00000260682	p.L313L	c.939G>A	Verified	CTC	CTG	CTG	3	G	A
TCGA-10-0930	CYP2C9	ENST00000260682	p.L313L	c.939G>A	Verified	CTC	CTG	CTG	3	G	A
TCGA-13-0885	CYP3A7	ENST00000336374	p.R162R	c.486G>A	Verified	AGG	CGG	GAA	3	G	A
TCGA-61-1907	CYP4A11	ENST00000310638	p.C347Y	c.1040G>A	Unverified	AGG	TGC	CGG	2	G	A
TCGA-13-0903	CYP4F3	ENST00000221307	p.L80L	c.240G>A	Verified	AGC	CTG	GCA	3	G	A
TCGA-24-1604	CYP7B1	ENST00000310193	p.E272K	c.814G>A	Verified	CTG	GAG	AAA	1	G	A
TCGA-24-1849	CYP7B1	ENST00000310193	p.D384N	c.1150G>A	Unverified	GGG	GAC	TAC	1	G	A
TCGA-30-1856	CYP7B1	ENST00000310193	p.V200I	c.598G>A	Unverified	AAA	GTT	ATT	1	G	A
TCGA-24-2262	DAAM2	ENST00000538976	p.R452Q	c.1355G>A	Verified	ttc	cgg	aaa	2	G	A
TCGA-23-1120	DACH1	ENST00000305425	p.A398T	c.1192G>A	Unverified	CCA	GCA	TCT	1	G	A
TCGA-61-1904	DACT1	ENST00000335867	p.D675N	c.2023G>A	Unverified	act	gac	tac	1	G	A
TCGA-13-0714	DAG1	ENST00000308775	p.V656M	c.1966G>A	Verified	ATC	GTG	GTG	1	G	A
TCGA-29-1691	DAGLA	ENST00000257215	p.E738E	c.2214G>A	Unverified	TCG	GAG	GGG	3	G	A
TCGA-20-1687	DCAF12L2	ENST00000538699	p.S390N	c.1169G>A	Unverified	tcc	agc	ctg	2	G	A
TCGA-29-1696	DCAF12L2	ENST00000538699	p.R337H	c.1010G>A	Unverified	cag	cgc	cag	2	G	A
TCGA-29-1696	DCAF12L2	ENST00000538699	p.R337H	c.1010G>A	Unverified	cag	cgc	cag	2	G	A
TCGA-29-1775	DCAF4L2	ENST00000319675	p.R20K	c.59G>A	Unverified	GTC	AGA	GTG	2	G	A
TCGA-13-0920	DCAF8L1	ENST00000441525	p.V396I	c.1186G>A	Verified	tgc	gtt	gtg	1	G	A
TCGA-13-0791	DCAF8L1	ENST00000441525	p.K429K	c.1287G>A	Verified	gtt	aag	aga	3	G	A
TCGA-23-1117	DCLK2	ENST00000296550	p.E594K	c.1780G>A	Verified	AGT	GAG	AAC	1	G	A
TCGA-23-1117	DCLK2	ENST00000296550	p.E594K	c.1780G>A	Verified	AGT	GAG	AAC	1	G	A
TCGA-61-1915	DCLK3	ENST00000416516	p.G339S	c.1015G>A	Unverified	agc	ggt	cgg	1	G	A
TCGA-23-1809	DCTD	ENST00000438320	p.A23T	c.67G>A	Unverified	GTG	GCC	TTC	1	G	A
TCGA-24-2035	DCTN2	ENST00000434715	p.A279T	c.835G>A	Verified	CTT	GCA	GTT	1	G	A

TCGA-24-2024	DCTN6	ENST00000221114	p.G46R	c.136G>A	Verified	GCC	GGG	CCA	1	G	A
TCGA-29-1698	DCX	ENST00000338081	p.D156N	c.466G>A	Unverified	tct	gac	cgt	1	G	A
TCGA-24-1845	DCX	ENST00000338081	p.G304E	c.911G>A	Unverified	acc	ggg	gtt	2	G	A
TCGA-29-1698	DCX_ENST0(	ENST00000356915	p.D75N	c.223G>A	Unverified	TCT	GAC	CGT	1	G	A
TCGA-24-1845	DCX_ENST0(	ENST00000356915	p.G223E	c.668G>A	Unverified	ACC	GGG	GTT	2	G	A
TCGA-24-1103	DDC	ENST00000357936	p.V184V	c.552G>A	Verified	CTG	GTG	GCT	3	G	A
TCGA-25-2393	DDO	ENST00000368924	p.V259I	c.775G>A	Verified	CAT	GTA	ACC	1	G	A
TCGA-23-1116	DDX1	ENST00000381341	p.E420K	c.1258G>A	Verified	tcc	gag	aag	1	G	A
TCGA-23-1120	DDX21	ENST00000354185	p.G235G	c.705G>A	Verified	act	ggg	aag	3	G	A
TCGA-13-1507	DDX39A	ENST00000242776	p.R174H	c.521G>A	Unverified	ggc	cgc	atc	2	G	A
TCGA-13-0923	DDX39B	ENST00000396172	p.G26R	c.76G>A	Verified	GAT	GGG	GCT	1	G	A
TCGA-29-1785	DDX4	ENST00000505374	p.V444I	c.1330G>A	Unverified	TTA	GTT	TTG	1	G	A
TCGA-29-1785	DDX4	ENST00000505374	p.V444I	c.1330G>A	Unverified	TTA	GTT	TTG	1	G	A
TCGA-29-1785	DDX4_ENST(	ENST00000505374	p.V444I	c.1330G>A	Unverified	TTA	GTT	TTG	1	G	A
TCGA-29-1785	DDX4_ENST(	ENST00000505374	p.V444I	c.1330G>A	Unverified	TTA	GTT	TTG	1	G	A
TCGA-29-1785	DDX4_ENST(	ENST00000511853	p.V295I	c.883G>A	Unverified	TTA	GTT	TTG	1	G	A
TCGA-29-1785	DDX4_ENST(	ENST00000511853	p.V295I	c.883G>A	Unverified	TTA	GTT	TTG	1	G	A
TCGA-13-0923	DDX41_ENST	ENST00000507955	p.E122K	c.364G>A	Unverified	GCC	GAG	GGC	1	G	A
TCGA-13-1498	DEFB118	ENST00000253381	p.V119I	c.355G>A	Verified	AAT	GTT	CAC	1	G	A
TCGA-13-0714	DENND1A	ENST00000373624	p.Q585Q	c.1755G>A	Verified	CTG	CAG	CCA	3	G	A
TCGA-30-1855	DENND3	ENST00000262585	p.A787A	c.2361G>A	Unverified	GTG	GCG	TCC	3	G	A
TCGA-29-1705	DENND5A	ENST00000328194	p.R607Q	c.1820G>A	Unverified	TCC	CGA	GTT	2	G	A
TCGA-24-2289	DEPTOR	NM_022783.1	p.C190Y	c.569G>A	Unverified	ctt	tgc	cac	2	G	A
TCGA-23-1117	DGKA	ENST00000331886	p.A314A	c.942G>A	Verified	caa	gcg	gtg	3	G	A
TCGA-23-1117	DGKA	ENST00000331886	p.A314A	c.942G>A	Verified	caa	gcg	gtg	3	G	A
TCGA-23-1124	DGKB	ENST00000403951	p.V214M	c.640G>A	Verified	ACC	GTG	TCT	1	G	A
TCGA-29-1784	DGKB	ENST00000403951	p.T163T	c.489G>A	Unverified	GAC	ACG	GAT	3	G	A
TCGA-24-1846	DGKG	ENST00000265022	p.R370Q	c.1109G>A	Unverified	TGC	CGG	ATG	2	G	A
TCGA-29-1784	DHCR24	ENST00000371269	p.M387I	c.1161G>A	Unverified	GAC	ATG	CTG	3	G	A
TCGA-24-2262	DHRS13	ENST00000378895	p.E288K	c.862G>A	Unverified	GTG	GAA	GAG	1	G	A
TCGA-20-0991	DHRS2	ENST00000250383	p.R30K	c.89G>A	Verified	GAC	AGG	AAG	2	G	A
TCGA-13-0807	DHRS7	ENST00000216500	p.M312I	c.936G>A	Verified	AAG	ATG	GGG	3	G	A
TCGA-24-1470	DHRSX	ENST00000334651	p.E89K	c.265G>A	Unverified	AAA	GAA	GAA	1	G	A
TCGA-24-1422	DHX16_ENST	ENST00000376442	p.A343T	c.1027G>A	Unverified	GGG	GCC	CGA	1	G	A

TCGA-10-0930	DHX30	ENST00000445061	p.L658L	c.1974G>A	Verified	gat	ctg	gtt	3	G	A
TCGA-10-0930	DHX30	ENST00000445061	p.L658L	c.1974G>A	Verified	gat	ctg	gtt	3	G	A
TCGA-61-2095	DHX37	ENST00000308736	p.E530K	c.1588G>A	Unverified	gct	gag	gtg	1	G	A
TCGA-29-1764	DIABLO	ENST00000443649	p.L153L	c.459G>A	Unverified	aag	ctg	gaa	3	G	A
TCGA-24-1463	DIDO1	ENST00000395343	p.D416N	c.1246G>A	Verified	AAT	GAC	TGT	1	G	A
TCGA-13-0924	DIDO1	ENST00000395343	p.S660N	c.1979G>A	Verified	ATG	AGT	GCT	2	G	A
TCGA-24-1469	DIP2A	ENST00000417564	p.G230S	c.688G>A	Verified	ACG	GGC	CTC	1	G	A
TCGA-61-1904	DIP2C	ENST00000280886	p.E1248K	c.3742G>A	Unverified	ACA	GAG	TCC	1	G	A
TCGA-29-1691	DIP2C	ENST00000280886	p.L683L	c.2049G>A	Unverified	GGA	CTG	ACC	3	G	A
TCGA-24-1422	DIRAS1	ENST00000323469	p.V47M	c.139G>A	Unverified	cag	gtg	atc	1	G	A
TCGA-13-1501	DIXDC1	ENST00000389821	p.G328D	c.983G>A	Verified	CCA	GGT	GTC	2	G	A
TCGA-13-1501	DIXDC1	ENST00000389821	p.G328D	c.983G>A	Verified	CCA	GGT	GTC	2	G	A
TCGA-13-0900	DLEC1	ENST00000308059	p.S1345S	c.4035G>A	Verified	TCA	TCG	GAA	3	G	A
TCGA-13-0792	DLG1	ENST00000346964	p.S347N	c.1040G>A	Verified	AAT	AGC	ATC	2	G	A
TCGA-24-1846	DLG3	ENST00000374360	p.E680K	c.2038G>A	Unverified	cga	gaa	caa	1	G	A
TCGA-24-1846	DLG3_ENST(	ENST00000374355	p.E375K	c.1123G>A	Unverified	CGA	GAA	CAA	1	G	A
TCGA-23-1031	DLG4	ENST00000293813	p.R559Q	c.1676G>A	Unverified	GGT	CGA	GAA	2	G	A
TCGA-29-1762	DLGAP2_EN	ENST00000356067	p.G414R	c.1240G>A	Unverified	GTG	GGA	CAC	1	G	A
TCGA-30-1714	DLGAP4_EN	ENST00000340491	p.G153R	c.457G>A	Unverified	ATC	GGG	GTT	1	G	A
TCGA-13-1488	DMBX1	NM_147192.2	p.P291P	c.873G>A	Verified	ggt	ccg	gcc	3	G	A
TCGA-13-1497	DMC1	ENST00000216024	p.V7V	c.21G>A	Verified	GTT	GTG	GCG	3	G	A
TCGA-29-1695	DMKN	ENST00000339686	p.S276N	c.827G>A	Unverified	GGC	AGC	AGC	2	G	A
TCGA-13-0760	DNAAF1	ENST00000378553	p.K362K	c.1086G>A	Verified	GGC	AAG	GAG	3	G	A
TCGA-13-0913	DNAH1_ENS	ENST00000420323	p.V3811M	c.11431G>A	Verified	AAG	GTG	ATG	1	G	A
TCGA-29-1691	DNAH10_EN	ENST00000409039	p.G2937R	c.8809G>A	Unverified	GTG	GGG	GAC	1	G	A
TCGA-13-0916	DNAH10_sa	ENST00000280576	p.E1834K	c.5500G>A	Unverified	GTT	GAA	GCT	1	G	A
TCGA-29-1691	DNAH10_sa	ENST00000280576	p.G1529R	c.4585G>A	Unverified	GTG	GGG	GAC	1	G	A
TCGA-24-1847	DNAH11	ENST00000328843	p.V2590M	c.7768G>A	Unverified	GAA	GTG	GAC	1	G	A
TCGA-30-1857	DNAH17	ENST00000300671	p.R3647H	c.10940G>A	Unverified	TAC	CGC	CCG	2	G	A
TCGA-29-1761	DNAH17	ENST00000300671	p.P4396P	c.13188G>A	Unverified	ACC	CCG	GCC	3	G	A
TCGA-13-0920	DNAH2	ENST00000389173	p.E833K	c.2497G>A	Verified	ATG	GAG	GAT	1	G	A
TCGA-29-1785	DNAH3	ENST00000261383	p.A3533T	c.10597G>A	Unverified	ATG	GCA	GGC	1	G	A
TCGA-29-1785	DNAH3	ENST00000261383	p.A3533T	c.10597G>A	Unverified	ATG	GCA	GGC	1	G	A
TCGA-29-1785	DNAH3_ENS	ENST00000261383	p.A3533T	c.10597G>A	Unverified	ATG	GCA	GGC	1	G	A

TCGA-29-1785	DNAH3_ENS	ENST00000261383	p.A3533T	c.10597G>A	Unverified	ATG	GCA	GGC	1	G	A
TCGA-13-0904	DNAH5	ENST00000265104	p.V3245M	c.9733G>A	Verified	GAA	GTG	ACA	1	G	A
TCGA-61-1740	DNAH7	ENST00000312428	p.C3027Y	c.9080G>A	Unverified	AAT	TGC	ATC	2	G	A
TCGA-09-2051	DNAH9	ENST00000262442	p.E389K	c.1165G>A	Unverified	GAA	GAA	AGT	1	G	A
TCGA-29-1699	DNAJC6	ENST00000395325	p.D681N	c.2041G>A	Unverified	gcc	gac	ctt	1	G	A
TCGA-29-1763	DNALI1	ENST00000296218	p.L170L	c.510G>A	Unverified	GGG	CTG	CTG	3	G	A
TCGA-24-1849	DNASE1L3	ENST00000394549	p.L216L	c.648G>A	Unverified	TGG	CTG	ATC	3	G	A
TCGA-24-1422	DNER	ENST00000341772	p.R684H	c.2051G>A	Verified	TGC	CGC	AGC	2	G	A
TCGA-04-1361	DNMT3A	ENST00000321117	p.A910T	c.2728G>A	Verified	ttt	gcg	tgt	1	G	A
TCGA-04-1361	DNMT3A	ENST00000321117	p.A910T	c.2728G>A	Verified	ttt	gcg	tgt	1	G	A
TCGA-29-1691	DOCK10	ENST00000258390	p.R2115H	c.6344G>A	Unverified	GAG	CGC	CTC	2	G	A
TCGA-29-1691	DOCK10_EN	ENST00000373702	p.R624H	c.1871G>A	Unverified	GAG	CGC	CTC	2	G	A
TCGA-29-1783	DOCK11	ENST00000276202	p.E343K	c.1027G>A	Unverified	TCA	GAA	GTT	1	G	A
TCGA-24-1844	DOCK2	ENST00000256935	p.D1146N	c.3436G>A	Unverified	GGC	GAC	GAG	1	G	A
TCGA-13-0807	DOCK2	ENST00000256935	p.R854Q	c.2561G>A	Verified	TGC	CGG	GAC	2	G	A
TCGA-23-1123	DOCK3_ENS	ENST00000266037	p.V1944I	c.5830G>A	Verified	GCC	GTC	CTG	1	G	A
TCGA-23-1123	DOCK3_ENS	ENST00000266037	p.V1944I	c.5830G>A	Verified	GCC	GTC	CTG	1	G	A
TCGA-13-0903	DOCK6	ENST00000319867	p.R1967Q	c.5900G>A	Verified	TTC	CGG	CAT	2	G	A
TCGA-04-1649	DOK3	ENST00000357198	p.T24T	c.72G>A	Unverified	GGG	ACG	GGA	3	G	A
TCGA-23-1122	DOK5	ENST00000262593	p.K66K	c.198G>A	Verified	gtg	aag	aac	3	G	A
TCGA-29-1699	DOLK	ENST00000372586	p.V114M	c.340G>A	Unverified	GTG	GTG	GCA	1	G	A
TCGA-61-1913	DOPEY1	ENST00000349129	p.V1009I	c.3025G>A	Unverified	AGG	GTT	TCA	1	G	A
TCGA-29-1702	DPM3	ENST00000368399	p.Q105Q	c.315G>A	Unverified	CTG	CAG	AGC	3	G	A
TCGA-24-1463	DPP10	ENST00000310323	p.V408M	c.1222G>A	Verified	ACC	GTG	CGG	1	G	A
TCGA-13-1501	DPP4	ENST00000360534	p.A306T	c.916G>A	Verified	TGG	GCA	ACA	1	G	A
TCGA-13-1501	DPP4	ENST00000360534	p.A306T	c.916G>A	Verified	TGG	GCA	ACA	1	G	A
TCGA-13-1497	DPPA2	ENST00000478945	p.K200K	c.600G>A	Verified	CCT	AAG	GCT	3	G	A
TCGA-13-0793	DPY19L3	ENST00000342179	p.G335E	c.1004G>A	Verified	ACT	GGA	AGC	2	G	A
TCGA-13-1481	DPY19L3	ENST00000342179	p.E686E	c.2058G>A	Verified	GAA	GAG	ATC	3	G	A
TCGA-29-1761	DPY19L4	ENST00000414645	p.E118K	c.352G>A	Unverified	TAC	GAA	CTG	1	G	A
TCGA-13-0792	DPYSL3	ENST00000398514	p.L459L	c.1377G>A	Verified	AAC	CTG	CAC	3	G	A
TCGA-25-1313	DPYSL5	ENST00000288699	p.?	c.600+1G>A	Verified	GCC	GAG	GGT	3	G	A
TCGA-29-1763	DRD5	ENST00000304374	p.A22A	c.66G>A	Unverified	CTG	GCG	CAG	3	G	A
TCGA-13-0884	DSCAM	ENST00000400454	p.E281K	c.841G>A	Verified	ATT	GAG	AAC	1	G	A

TCGA-61-1915	DSCR4	ENST00000328264	p.A86T	c.256G>A	Unverified	TGG	GCA	AAA	1	G	A
TCGA-25-2391	DSG4	ENST00000308128	p.D247N	c.739G>A	Unverified	GCA	GAT	GGA	1	G	A
TCGA-04-1638	DSG4	ENST00000308128	p.A601A	c.1803G>A	Unverified	GCC	GCG	GGC	3	G	A
TCGA-04-1638	DSG4_ENST(	ENST00000359747	p.A601A	c.1803G>A	Unverified	GCC	GCG	GGC	3	G	A
TCGA-13-0760	DST	ENST00000370765	p.R689H	c.2066G>A	Verified	TCT	CGT	TTT	2	G	A
TCGA-61-1914	DST	ENST00000370765	p.A1654A	c.4962G>A	Unverified	CTG	GCG	AAA	3	G	A
TCGA-61-1895	DST_ENST0(	ENST00000244364	p.R5140Q	c.15419G>A	Unverified	CCC	CGA	GCA	2	G	A
TCGA-61-1914	DST_ENST0(	ENST00000370765	p.A1654A	c.4962G>A	Unverified	CTG	GCG	AAA	3	G	A
TCGA-13-0885	DST_ENST0(	ENST00000370769	p.A6644T	c.19930G>A	Verified	TTT	GCC	AAT	1	G	A
TCGA-24-1469	DST_ENST0(	ENST00000370769	p.E3190K	c.9568G>A	Verified	AAT	GAG	GCA	1	G	A
TCGA-13-0760	DST_ENST0(	ENST00000370769	p.R1015H	c.3044G>A	Verified	TCT	CGT	TTT	2	G	A
TCGA-61-1895	DST_ENST0(	ENST00000370769	p.R7514Q	c.22541G>A	Unverified	CCC	CGA	GCA	2	G	A
TCGA-24-0979	DTD1	ENST00000377452	p.E100E	c.300G>A	Verified	ACG	GAG	CAG	3	G	A
TCGA-23-1032	DTNB	ENST00000406818	p.R390H	c.1169G>A	Verified	GCA	CGT	GTT	2	G	A
TCGA-24-1847	DTX3	ENST00000548804	p.E113E	c.339G>A	Unverified	GGG	GAG	CAC	3	G	A
TCGA-13-0906	DUOX2	ENST00000389039	p.E748K	c.2242G>A	Verified	GCT	GAG	ATG	1	G	A
TCGA-04-1343	DUSP1	ENST00000239223	p.V206I	c.616G>A	Verified	AAC	GTC	TCA	1	G	A
TCGA-23-1124	DUSP27	ENST00000361200	p.R964K	c.2891G>A	Verified	ACC	AGA	TCG	2	G	A
TCGA-23-1809	DUSP27	ENST00000361200	p.R1043H	c.3128G>A	Unverified	TTC	CGC	CGG	2	G	A
TCGA-29-1783	DYNC1H1	ENST00000360184	p.A2951T	c.8851G>A	Unverified	GTC	GCC	TGG	1	G	A
TCGA-13-0760	DYNC1I1	ENST00000324972	p.G115S	c.343G>A	Verified	TCA	GGC	GAT	1	G	A
TCGA-24-1435	DYNC2LI1	ENST00000260605	p.K44K	c.132G>A	Verified	GGA	AAG	ACT	3	G	A
TCGA-09-2050	E2F2	ENST00000361729	p.V175M	c.523G>A	Verified	aac	gtg	ctg	1	G	A
TCGA-20-1683	E2F7	ENST00000322886	p.V192M	c.574G>A	Unverified	AAT	GTG	CTG	1	G	A
TCGA-20-1683	E2F7_ENST0	ENST00000322886	p.V192M	c.574G>A	Unverified	AAT	GTG	CTG	1	G	A
TCGA-29-1691	EBP	ENST00000495186	p.V44M	c.130G>A	Unverified	gtc	gtg	acc	1	G	A
TCGA-20-0990	ECE1	ENST00000374893	p.R754H	c.2261G>A	Verified	TTC	CGC	TGC	2	G	A
TCGA-24-1616	ECE2	ENST00000359140	p.A533T	c.1597G>A	Verified	AAT	GCC	TAC	1	G	A
TCGA-24-2290	ECE2	ENST00000359140	p.R438Q	c.1313G>A	Verified	ATC	CGG	ACC	2	G	A
TCGA-24-2290	ECE2	ENST00000359140	p.R438Q	c.1313G>A	Verified	ATC	CGG	ACC	2	G	A
TCGA-61-1910	EDA	ENST00000374552	p.V307I	c.919G>A	Unverified	CAG	GTA	GAA	1	G	A
TCGA-61-1910	EDA_ENST0(	ENST00000374552	p.V307I	c.919G>A	Unverified	CAG	GTA	GAA	1	G	A
TCGA-24-2035	EDC4	ENST00000358933	p.E76K	c.226G>A	Unverified	CAA	GAG	AAG	1	G	A
TCGA-24-2290	EDC4	ENST00000358933	p.R974H	c.2921G>A	Verified	CAG	CGT	CTG	2	G	A

TCGA-24-2290	EDC4	ENST00000358933	p.R974H	c.2921G>A	Verified	CAG	CGT	CTG	2	G	A
TCGA-09-2051	EDEM3	ENST00000318130	p.A807T	c.2419G>A	Unverified	CTA	GCA	GAT	1	G	A
TCGA-29-1761	EDNRB	ENST00000334286	p.V325I	c.973G>A	Unverified	ACC	GTC	TTT	1	G	A
TCGA-29-1761	EDNRB_ENS	ENST00000377211	p.V415I	c.1243G>A	Unverified	ACC	GTC	TTT	1	G	A
TCGA-24-2289	EED	ENST00000263360	p.A11A	c.33G>A	Unverified	ccg	gcg	gga	3	G	A
TCGA-30-1718	EEF1A1	ENST00000316292	p.W58*	c.173G>A	Unverified	gcc	tgg	gtc	2	G	A
TCGA-61-2102	EFEMP2	ENST00000307998	p.S383S	c.1149G>A	Verified	AAC	TCG	CAG	3	G	A
TCGA-29-1763	EFHA2	ENST00000318063	p.G131S	c.391G>A	Unverified	ATT	GGC	AGA	1	G	A
TCGA-10-0930	EFHB	ENST00000295824	p.A162T	c.484G>A	Verified	CCT	GCT	TTC	1	G	A
TCGA-10-0930	EFHB	ENST00000295824	p.A162T	c.484G>A	Verified	CCT	GCT	TTC	1	G	A
TCGA-10-0930	EFHB_ENST(	ENST00000295824	p.A162T	c.484G>A	Verified	CCT	GCT	TTC	1	G	A
TCGA-10-0930	EFHB_ENST(	ENST00000295824	p.A162T	c.484G>A	Verified	CCT	GCT	TTC	1	G	A
TCGA-04-1338	EFHC2	ENST00000333807	p.R133H	c.398G>A	Verified	cgg	cgt	cat	2	G	A
TCGA-23-1114	EFNB1	ENST00000204961	p.R156H	c.467G>A	Unverified	aca	cgc	acc	2	G	A
TCGA-13-0905	EFR3A	ENST00000254624	p.L782L	c.2346G>A	Verified	ATA	TTG	GAA	3	G	A
TCGA-25-1313	EGFL7	ENST00000371698	p.K270K	c.810G>A	Verified	TGC	AAG	AAA	3	G	A
TCGA-61-1906	EGFLAM	ENST00000322350	p.G332R	c.994G>A	Unverified	AAG	GGG	AAG	1	G	A
TCGA-61-1906	EGFLAM	ENST00000322350	p.G332R	c.994G>A	Unverified	AAG	GGG	AAG	1	G	A
TCGA-61-1906	EGFLAM_EN	ENST00000354891	p.G332R	c.994G>A	Unverified	AAG	GGG	AAG	1	G	A
TCGA-61-1906	EGFLAM_EN	ENST00000354891	p.G332R	c.994G>A	Unverified	AAG	GGG	AAG	1	G	A
TCGA-09-2050	EGFR	ENST00000275493	p.G331G	c.993G>A	Unverified	GAA	GGG	CCT	3	G	A
TCGA-09-2050	EGFR_ENST(	ENST00000344576	p.G331G	c.993G>A	Unverified	GAA	GGG	CCT	3	G	A
TCGA-09-1674	EGLN2	ENST00000303961	p.V336M	c.1006G>A	Unverified	ccc	gtg	gta	1	G	A
TCGA-10-0930	EGR1	ENST00000239938	p.R320H	c.959G>A	Verified	AGC	CGC	ATG	2	G	A
TCGA-10-0930	EGR1	ENST00000239938	p.R320H	c.959G>A	Verified	AGC	CGC	ATG	2	G	A
TCGA-29-1762	EHHADH	ENST00000231887	p.G526G	c.1578G>A	Unverified	GCT	GGG	TTG	3	G	A
TCGA-09-1674	EIF3B	ENST00000397011	p.R315H	c.944G>A	Unverified	GAC	CGC	ACT	2	G	A
TCGA-29-1763	EIF4A3	ENST00000269349	p.E261K	c.781G>A	Unverified	agg	gaa	gag	1	G	A
TCGA-61-1998	ELMO1	ENST00000310758	p.A5T	c.13G>A	Verified	CCC	GCG	GAC	1	G	A
TCGA-04-1336	ELN	ENST00000252034	p.Q539Q	c.1617G>A	Verified	GCC	CAG	CTC	3	G	A
TCGA-29-1763	ELOVL7	ENST00000508821	p.V143V	c.429G>A	Unverified	CAA	GTG	ACT	3	G	A
TCGA-23-1809	ELP3	ENST00000256398	p.R462H	c.1385G>A	Unverified	TTC	CGT	TTC	2	G	A
TCGA-23-1809	EMILIN1	ENST00000380320	p.R726Q	c.2177G>A	Unverified	GGG	CGA	TTG	2	G	A
TCGA-25-2392	EMILIN1	ENST00000380320	p.G267D	c.800G>A	Unverified	GGC	GGC	AGC	2	G	A

TCGA-24-1431	EML5	ENST00000554922	p.E1166E	c.3498G>A	Verified	GTG	GAG	GTA	3	G	A
TCGA-13-0807	EMR3	ENST00000253673	p.D190N	c.568G>A	Verified	AAC	GAT	AGT	1	G	A
TCGA-23-2077	EMR3	ENST00000253673	p.G363R	c.1087G>A	Verified	GTG	GGG	CTG	1	G	A
TCGA-61-2094	ENDOU	ENST00000229003	p.R344Q	c.1031G>A	Unverified	GTC	CGG	ACA	2	G	A
TCGA-25-1318	ENO3	ENST00000323997	p.S401S	c.1203G>A	Verified	CGC	TCG	GAG	3	G	A
TCGA-25-1318	ENO3	ENST00000323997	p.S401S	c.1203G>A	Verified	CGC	TCG	GAG	3	G	A
TCGA-04-1362	ENPP3	ENST00000357639	p.W250*	c.749G>A	Verified	TGG	TGG	CAT	2	G	A
TCGA-61-1904	ENSG000000	ENST00000354191	p.S116N	c.347G>A	Unverified	TGT	AGC	CCC	2	G	A
TCGA-61-1899	ENSG000001	ENST00000324457	p.V132M	c.394G>A	Unverified	ACC	GTG	GAA	1	G	A
TCGA-13-1509	ENSG000001	ENST00000429274	p.R115Q	c.344G>A	Verified	ATG	CGG	GAG	2	G	A
TCGA-29-1690	ENSG000001	ENST00000308911	p.S172S	c.516G>A	Unverified	CCC	TCG	CCC	3	G	A
TCGA-23-1114	ENSG000001	ENST00000356069	p.R243H	c.728G>A	Unverified	ACA	CGT	TCC	2	G	A
TCGA-29-1703	ENSG000001	ENST00000331172	p.A190T	c.568G>A	Unverified	TTT	GCT	GGT	1	G	A
TCGA-23-1809	ENSG000001	ENST00000543388	p.R161H	c.482G>A	Unverified	CAT	CGT	TGA	2	G	A
TCGA-04-1638	ENSG000001	ENST00000358133	p.T113T	c.339G>A	Unverified	GAC	ACG	GGA	3	G	A
TCGA-13-0791	ENSG000002	ENST00000430863	p.R471H	c.1412G>A	Verified	AGT	CGC	CAG	2	G	A
TCGA-25-2400	ENSG000002	ENST00000260257	p.E40E	c.120G>A	Unverified	GCC	GAG	TTG	3	G	A
TCGA-25-2392	ENTHD1	ENST00000325157	p.R4K	c.11G>A	Verified	TTC	AGG	AGA	2	G	A
TCGA-23-1116	ENTHD1	ENST00000325157	p.A2A	c.6G>A	Unverified	ATG	GCG	TTC	3	G	A
TCGA-04-1343	EOMES	ENST00000295743	p.G370R	c.1108G>A	Verified	TTC	GGG	AAA	1	G	A
TCGA-61-1737	EOMES	ENST00000295743	p.G256R	c.766G>A	Unverified	CTG	GGG	GGC	1	G	A
TCGA-29-1703	EPB41L3	ENST00000341928	p.A653T	c.1957G>A	Unverified	TAC	GCT	CTC	1	G	A
TCGA-04-1356	EPHA1_ENST	ENST00000275815	p.V567I	c.1699G>A	Verified	CTC	GTT	TTC	1	G	A
TCGA-29-1693	EPHA10	CGP_EPHA10.1	p.A811A	c.2433G>A	Unverified	cca	gcg	cta	3	G	A
TCGA-09-2049	EPHA7	NM_004440	p.D108N	c.322G>A	Verified	agg	gat	tgt	1	G	A
TCGA-61-1904	EPHA8	ENST00000166244	p.V344V	c.1032G>A	Unverified	tca	gtg	act	3	G	A
TCGA-61-1904	EPHA8_ENST	ENST00000374644	p.V344V	c.1032G>A	Unverified	TCA	GTG	ACT	3	G	A
TCGA-61-1906	EPHB1	ENST00000398015	p.G81R	c.241G>A	Unverified	CGG	GGG	GCC	1	G	A
TCGA-61-1906	EPHB1	ENST00000398015	p.G81R	c.241G>A	Unverified	CGG	GGG	GCC	1	G	A
TCGA-61-2008	EPHB1	ENST00000398015	p.A176T	c.526G>A	Verified	CTC	GCT	TTT	1	G	A
TCGA-61-1906	EPHB1_ENST	ENST00000398015	p.G81R	c.241G>A	Unverified	CGG	GGG	GCC	1	G	A
TCGA-61-1906	EPHB1_ENST	ENST00000398015	p.G81R	c.241G>A	Unverified	CGG	GGG	GCC	1	G	A
TCGA-61-2008	EPHB1_ENST	ENST00000398015	p.A176T	c.526G>A	Verified	CTC	GCT	TTT	1	G	A
TCGA-23-1122	EPHX1	ENST00000366837	p.G344S	c.1030G>A	Verified	GGA	GGC	CTG	1	G	A

TCGA-61-1998	EPN3	ENST00000268933	p.P330P	c.990G>A	Verified	AGG	CCG	AAC	3	G	A
TCGA-29-1705	EPPK1	ENST00000525985	p.R1409Q	c.4226G>A	Unverified	GCG	CGG	GAC	2	G	A
TCGA-24-0975	EPPK1	ENST00000525985	p.V2231V	c.6693G>A	Unverified	CTG	GTG	CCC	3	G	A
TCGA-24-1850	EPS15L1	ENST00000248070	p.P28P	c.84G>A	Unverified	GAT	CCG	GCA	3	G	A
TCGA-13-0795	ERAP1	ENST00000296754	p.V647I	c.1939G>A	Verified	CTC	GTC	AGC	1	G	A
TCGA-09-1665	ERBB2	ENST00000269571	p.R896H	c.2687G>A	Unverified	CTC	CGC	CGG	2	G	A
TCGA-09-1665	ERBB2IP	NM_018695.1	p.V594I	c.1780G>A	Verified	gat	gtt	ttt	1	G	A
TCGA-29-1764	ERBB3	ENST00000267101	p.E1219K	c.3655G>A	Unverified	GAG	GAG	CTG	1	G	A
TCGA-13-1489	ERBB3_ENST	ENST00000267101	p.V438I	c.1312G>A	Verified	AAT	GTC	ACA	1	G	A
TCGA-29-1764	ERBB3_ENST	ENST00000267101	p.E1219K	c.3655G>A	Unverified	GAG	GAG	CTG	1	G	A
TCGA-29-1698	ERCC6	ENST00000355832	p.W581*	c.1742G>A	Unverified	CAG	TGG	GTG	2	G	A
TCGA-24-1849	ERGIC1	ENST00000393784	p.D59N	c.175G>A	Unverified	GTC	GAT	GAC	1	G	A
TCGA-25-2392	ERLEC1	ENST00000185150	p.E302E	c.906G>A	Verified	GAA	GAG	AGA	3	G	A
TCGA-13-0885	ERLEC1	ENST00000185150	p.?	c.750-1G>A	Verified	TAT	AGG	TTC	3	G	A
TCGA-24-1849	ESPL1	ENST00000257934	p.L905L	c.2715G>A	Unverified	GTC	CTG	CAG	3	G	A
TCGA-25-1326	ESYT1	ENST00000394048	p.K671K	c.2013G>A	Verified	GTG	AAG	GGC	3	G	A
TCGA-09-0369	ETNK2	ENST00000367201	p.A187T	c.559G>A	Verified	CAC	GCC	AAC	1	G	A
TCGA-09-0369	ETNK2	ENST00000367201	p.A187T	c.559G>A	Verified	CAC	GCC	AAC	1	G	A
TCGA-20-1685	ETNK2	ENST00000367201	p.V291M	c.871G>A	Unverified	GGC	GTG	AAT	1	G	A
TCGA-20-1685	ETNK2_ENST	ENST00000367202	p.V291M	c.871G>A	Unverified	GGC	GTG	AAT	1	G	A
TCGA-30-1856	ETV1	ENST00000430479	p.V476M	c.1426G>A	Unverified	tac	gtg	tat	1	G	A
TCGA-30-1856	ETV1_ENST0	ENST00000403527	p.V436M	c.1306G>A	Unverified	TAC	GTG	TAT	1	G	A
TCGA-04-1651	EWSR1	ENST00000397938	p.D359N	c.1075G>A	Unverified	TCT	GAC	AAC	1	G	A
TCGA-04-1651	EWSR1	ENST00000397938	p.D359N	c.1075G>A	Unverified	TCT	GAC	AAC	1	G	A
TCGA-29-1705	EXOC4	ENST00000253861	p.V974I	c.2920G>A	Unverified	ACC	GTT	TAG	1	G	A
TCGA-13-1489	EXOC4	ENST00000253861	p.S579N	c.1736G>A	Verified	CAG	AGC	ACA	2	G	A
TCGA-29-1691	EXOSC10	ENST00000376936	p.K803K	c.2409G>A	Unverified	TCC	AAG	AAG	3	G	A
TCGA-20-0991	EXT1	ENST00000378204	p.A151A	c.453G>A	Verified	cag	gcg	tgc	3	G	A
TCGA-13-0760	EXT1	ENST00000378204	p.T568T	c.1704G>A	Verified	gac	acg	gtg	3	G	A
TCGA-29-1775	EXTL3	ENST00000220562	p.G176S	c.526G>A	Unverified	CGG	GGC	TGC	1	G	A
TCGA-13-0883	EYA1	ENST00000388742	p.G39D	c.116G>A	Verified	aat	ggc	acc	2	G	A
TCGA-24-1469	EZH1	ENST00000428826	p.E721K	c.2161G>A	Verified	ggc	gaa	gag	1	G	A
TCGA-09-1674	F11	ENST00000403665	p.G342E	c.1025G>A	Unverified	GAA	GGG	AAG	2	G	A
TCGA-61-1899	F2R	ENST00000319211	p.V178I	c.532G>A	Unverified	TTC	GTC	ACT	1	G	A

TCGA-29-1761	F8	ENST00000360256	p.A1741T	c.5221G>A	Unverified	AGG	GCT	CAG	1	G	A
TCGA-29-1761	F8_ENST000	ENST00000360256	p.A1741T	c.5221G>A	Unverified	AGG	GCT	CAG	1	G	A
TCGA-24-1843	FAM102B_E	ENST00000370035	p.A322A	c.966G>A	Unverified	AGT	GCG	GAA	3	G	A
TCGA-04-1542	FAM105A	ENST00000274217	p.D293N	c.877G>A	Verified	GGC	GAC	ACA	1	G	A
TCGA-13-0885	FAM111B	ENST00000343597	p.S331N	c.992G>A	Verified	CTA	AGC	CAT	2	G	A
TCGA-25-1313	FAM111B	ENST00000343597	p.W562*	c.1685G>A	Verified	CTA	TGG	CGA	2	G	A
TCGA-04-1649	FAM115A	ENST00000355951	p.A829T	c.2485G>A	Unverified	ACC	GCA	CTG	1	G	A
TCGA-61-1733	FAM126A	ENST00000432176	p.P466P	c.1398G>A	Unverified	AAC	CCG	TCA	3	G	A
TCGA-61-1733	FAM126A	ENST00000432176	p.P466P	c.1398G>A	Unverified	AAC	CCG	TCA	3	G	A
TCGA-61-1904	FAM131B	ENST00000409346	p.G119S	c.355G>A	Unverified	GAT	GGC	GAG	1	G	A
TCGA-25-2042	FAM131B	ENST00000409346	p.G92E	c.275G>A	Unverified	TGG	GGG	AAG	2	G	A
TCGA-61-1904	FAM131B_E	ENST00000443739	p.G147S	c.439G>A	Unverified	GAT	GGC	GAG	1	G	A
TCGA-25-2042	FAM131B_E	ENST00000443739	p.G120E	c.359G>A	Unverified	TGG	GGG	AAG	2	G	A
TCGA-24-0979	FAM134C	ENST00000309428	p.D213N	c.637G>A	Verified	TGG	GAT	CGA	1	G	A
TCGA-29-1703	FAM135A	ENST00000370479	p.V1025I	c.3073G>A	Unverified	AGT	GTA	GAA	1	G	A
TCGA-61-1910	FAM135A	ENST00000370479	p.R250Q	c.749G>A	Unverified	CTA	CGA	ACA	2	G	A
TCGA-29-1703	FAM135A_E	ENST00000418814	p.V1238I	c.3712G>A	Unverified	AGT	GTA	GAA	1	G	A
TCGA-13-0762	FAM13A	ENST00000264344	p.R648Q	c.1943G>A	Verified	AGG	CGG	CGA	2	G	A
TCGA-13-0762	FAM13A	ENST00000264344	p.R648Q	c.1943G>A	Verified	AGG	CGG	CGA	2	G	A
TCGA-61-1910	FAM13A	ENST00000264344	p.M646I	c.1938G>A	Unverified	TTC	ATG	AGG	3	G	A
TCGA-61-1910	FAM13A_EN	ENST00000508369	p.M320I	c.960G>A	Unverified	TTC	ATG	AGG	3	G	A
TCGA-24-2267	FAM13C	ENST00000373868	p.R144Q	c.431G>A	Verified	GTG	CGA	CTT	2	G	A
TCGA-29-1702	FAM149A	ENST00000503432	p.T362T	c.1086G>A	Unverified	caa	acg	tca	3	G	A
TCGA-23-1111	FAM155B	ENST00000252338	p.E305K	c.913G>A	Unverified	cag	gaa	tgc	1	G	A
TCGA-24-1470	FAM171B	ENST00000304698	p.E787K	c.2359G>A	Verified	GTT	GAA	GAT	1	G	A
TCGA-61-1738	FAM171B	ENST00000304698	p.T110T	c.330G>A	Unverified	TAC	ACG	AAG	3	G	A
TCGA-61-1738	FAM171B	ENST00000304698	p.T110T	c.330G>A	Unverified	TAC	ACG	AAG	3	G	A
TCGA-24-1845	FAM183B	ENST00000409072	p.E22K	c.64G>A	Unverified	CGG	GAG	CTG	1	G	A
TCGA-24-1845	FAM183B_E	ENST00000409072_v68	p.E22K	c.64G>A	Unverified	CGG	GAG	CTG	1	G	A
TCGA-13-0793	FAM188A	ENST00000277632	p.L208L	c.624G>A	Verified	ccc	ttg	ata	3	G	A
TCGA-61-1725	FAM18B1	ENST00000307767	p.M159I	c.477G>A	Unverified	ATC	ATG	GGT	3	G	A
TCGA-25-2400	FAM193A	ENST00000382839	p.R126H	c.377G>A	Unverified	CTG	CGC	AGA	2	G	A
TCGA-24-1474	FAM200A	ENST00000449309	p.R245Q	c.734G>A	Unverified	CAT	CGA	GAA	2	G	A
TCGA-13-1501	FAM20B	ENST00000263733	p.D37N	c.109G>A	Verified	GAG	GAC	CAG	1	G	A

TCGA-13-1501	FAM20B	ENST00000263733	p.D37N	c.109G>A	Verified	GAG	GAC	CAG	1	G	A
TCGA-30-1855	FAM21A	ENST00000282633	p.K1326K	c.3978G>A	Unverified	CAC	AAG	GTG	3	G	A
TCGA-24-1849	FAM39B	ENST00000359512	p.R433H	c.1298G>A	Unverified	AGG	CGC	AAG	2	G	A
TCGA-23-2078	FAM45A	ENST00000361432	p.C230Y	c.689G>A	Unverified	ATG	TGC	ACA	2	G	A
TCGA-24-2290	FAM47A	ENST00000346193	p.S683S	c.2049G>A	Verified	cca	tcg	aat	3	G	A
TCGA-24-2290	FAM47A	ENST00000346193	p.S683S	c.2049G>A	Verified	cca	tcg	aat	3	G	A
TCGA-61-2113	FAM47A	ENST00000346193	p.P225P	c.675G>A	Verified	cgc	ccg	gag	3	G	A
TCGA-24-1847	FAM47A	ENST00000346193	p.P532P	c.1596G>A	Unverified	ggt	ccg	gag	3	G	A
TCGA-13-0905	FAM47B	ENST00000329357	p.R291H	c.872G>A	Verified	ACT	CGC	GTA	2	G	A
TCGA-04-1331	FAM47C	ENST00000358047	p.G820R	c.2458G>A	Verified	acc	gga	gcg	1	G	A
TCGA-61-2094	FAM47C	ENST00000358047	p.P728P	c.2184G>A	Unverified	tgc	ccg	gag	3	G	A
TCGA-10-0930	FAM50A	ENST00000393600	p.M144I	c.432G>A	Verified	gag	atg	gaa	3	G	A
TCGA-10-0930	FAM50A	ENST00000393600	p.M144I	c.432G>A	Verified	gag	atg	gaa	3	G	A
TCGA-24-0975	FAM53C	ENST00000239906	p.V352M	c.1054G>A	Unverified	CAG	GTG	CTG	1	G	A
TCGA-23-1123	FAM55B	ENST00000389586	p.S197N	c.590G>A	Verified	CCC	AGT	GAA	2	G	A
TCGA-23-1123	FAM55B	ENST00000389586	p.S197N	c.590G>A	Verified	CCC	AGT	GAA	2	G	A
TCGA-13-0760	FAM5B	ENST00000361539	p.V756M	c.2266G>A	Verified	GAG	GTG	GGC	1	G	A
TCGA-24-1470	FAM5C	ENST00000367462	p.V207I	c.619G>A	Verified	aag	gta	aca	1	G	A
TCGA-61-1737	FAM5C	ENST00000367462	p.E496K	c.1486G>A	Unverified	ctc	gag	atg	1	G	A
TCGA-24-2267	FAM63B	ENST00000559228	p.G239E	c.716G>A	Unverified	CCG	GGA	CAA	2	G	A
TCGA-30-1714	FAM71A	ENST00000294829	p.P44P	c.132G>A	Unverified	GCA	CCG	ATA	3	G	A
TCGA-13-0755	FAM75A3	ENST00000356699	p.R684K	c.2051G>A	Verified	TCC	AGG	GAT	2	G	A
TCGA-24-1616	FAM75D4	ENST00000344803	p.R1406R	c.4218G>A	Verified	ATT	AGG	AAA	3	G	A
TCGA-13-0762	FAM78A	ENST00000372271	p.V166I	c.496G>A	Verified	AGC	GTC	ACA	1	G	A
TCGA-13-0762	FAM78A	ENST00000372271	p.V166I	c.496G>A	Verified	AGC	GTC	ACA	1	G	A
TCGA-09-1674	FAM78B	ENST00000354422	p.V136I	c.406G>A	Unverified	TCC	GTC	AGC	1	G	A
TCGA-04-1338	FAM82B	ENST00000406452	p.K165K	c.495G>A	Verified	CAT	AAG	TGG	3	G	A
TCGA-24-1850	FAM86A	ENST00000427587	p.R285H	c.854G>A	Unverified	GTC	CGC	AAC	2	G	A
TCGA-13-0923	FANCI	ENST00000300027	p.R959Q	c.2876G>A	Verified	AGC	CGG	GAG	2	G	A
TCGA-61-1900	FASN	ENST00000306749	p.P1779P	c.5337G>A	Unverified	CAC	CCG	CTC	3	G	A
TCGA-24-1469	FASTKD1	ENST00000453153	p.E709K	c.2125G>A	Verified	GCA	GAG	GTA	1	G	A
TCGA-04-1367	FASTKD1	ENST00000453153	p.S720S	c.2160G>A	Verified	GCC	TCG	GTT	3	G	A
TCGA-24-1422	FASTKD2	ENST00000236980	p.M348I	c.1044G>A	Unverified	CAC	ATG	TTT	3	G	A
TCGA-13-0791	FAT1	ENST00000441802	p.V1373I	c.4117G>A	Verified	CCC	GTT	GCT	1	G	A

TCGA-23-1022	FAT1	ENST00000441802	p.R2354R	c.7062G>A	Verified	TCC	CGG	CAG	3	G	A
TCGA-13-0795	FAT3	ENST00000298047	p.A581T	c.1741G>A	Verified	AAA	GTG	GCT	1	G	A
TCGA-13-0913	FAT3_ENST(	ENST00000298047_v61	p.D573N	c.1717G>A	Unverified	AAC	GAC	AAC	1	G	A
TCGA-23-2077	FAT3_ENST(	ENST00000298047_v61	p.R3941H	c.11822G>A	Verified	CGG	CGC	CGG	2	G	A
TCGA-13-0913	FAT3_ENST(	ENST00000409404	p.D573N	c.1717G>A	Unverified	AAC	GAC	AAC	1	G	A
TCGA-23-2077	FAT3_ENST(	ENST00000409404	p.R3941H	c.11822G>A	Verified	CGG	CGC	CGG	2	G	A
TCGA-24-1469	FAT4	ENST00000330166	p.D1868N	c.5602G>A	Verified	GAT	GAT	GAC	1	G	A
TCGA-24-1469	FAT4_ENST(	ENST00000394329	p.D1868N	c.5602G>A	Verified	GAT	GAT	GAC	1	G	A
TCGA-13-0755	FBLN1	ENST00000327858	p.C50Y	c.149G>A	Verified	GAC	TGC	TCG	2	G	A
TCGA-23-1029	FBLN5	ENST00000342058	p.R331H	c.992G>A	Unverified	AAC	CGC	TGT	2	G	A
TCGA-20-0990	FBN2	ENST00000262464	p.G2185D	c.6554G>A	Verified	AAT	GGT	CAA	2	G	A
TCGA-13-0885	FBN2	ENST00000262464	p.M1315I	c.3945G>A	Verified	TTC	ATG	GCT	3	G	A
TCGA-13-0755	FBXL14	ENST00000339235	p.T334T	c.1002G>A	Verified	CGC	ACG	CTC	3	G	A
TCGA-61-1725	FBXO24	ENST00000241071	p.V250M	c.748G>A	Unverified	atc	gtg	ctg	1	G	A
TCGA-61-1725	FBXO24_EN:	ENST00000427939	p.V288M	c.862G>A	Unverified	ATC	GTG	CTG	1	G	A
TCGA-13-0919	FBXO30	ENST00000237281	p.R654H	c.1961G>A	Verified	tct	cgt	ggc	2	G	A
TCGA-13-1488	FBXO38	ENST00000340253	p.D1127N	c.3379G>A	Verified	gac	gat	gaa	1	G	A
TCGA-23-2078	FBXO38	ENST00000340253	p.D1126N	c.3376G>A	Unverified	gaa	gac	gat	1	G	A
TCGA-24-1474	FBXO44	ENST00000376770	p.D102N	c.304G>A	Verified	ggc	gat	gag	1	G	A
TCGA-04-1337	FBXW7	ENST00000281708	p.R465H	c.1394G>A	Unverified	GTG	CGT	TGT	2	G	A
TCGA-04-1337	FBXW7_ENS	ENST00000281708	p.R465H	c.1394G>A	Unverified	GTG	CGT	TGT	2	G	A
TCGA-04-1337	FBXW7_ENS	ENST00000534231	p.R226H	c.677G>A	Unverified	GTG	CGT	TGT	2	G	A
TCGA-04-1337	FBXW7_NM	ENST00000263981	p.R385H	c.1154G>A	Unverified	gtg	cgt	tgt	2	G	A
TCGA-04-1336	FCGBP	ENST00000221347	p.V5330M	c.15988G>A	Verified	TCC	GTG	AGT	1	G	A
TCGA-25-2393	FCGBP	ENST00000221347	p.V4311I	c.12931G>A	Verified	GAC	GTC	TGC	1	G	A
TCGA-29-1766	FCGBP	ENST00000221347	p.D4269N	c.12805G>A	Unverified	GAG	GAC	CGG	1	G	A
TCGA-13-0923	FCGR2A	ENST00000367972	p.A224A	c.672G>A	Verified	ATT	GCG	ACT	3	G	A
TCGA-13-1498	FCHSD2	NM_014824.1	p.T493T	c.1479G>A	Verified	cac	acg	tcc	3	G	A
TCGA-13-0919	FCRL1	ENST00000368176	p.A166T	c.496G>A	Verified	ACA	GCA	GAG	1	G	A
TCGA-23-1809	FCRL4	ENST00000271532	p.R97H	c.290G>A	Unverified	GTG	CGC	TTG	2	G	A
TCGA-25-2400	FDXACB1	ENST00000428306	p.L24L	c.72G>A	Unverified	ACC	CTG	GAT	3	G	A
TCGA-25-2391	FER1L5	ENST00000505256	p.S1958S	c.5874G>A	Unverified	CAG	TCG	GAA	3	G	A
TCGA-24-2262	FER1L6	ENST00000399018	p.R1751H	c.5252G>A	Verified	GTG	CGT	GGC	2	G	A
TCGA-24-1103	FER1L6	ENST00000399018	p.R1591R	c.4773G>A	Verified	CCA	AGG	CGA	3	G	A

TCGA-04-1342	FEZF1	ENST00000442488	p.T311T	c.933G>A	Verified	CAC	ACG	CAG	3	G	A
TCGA-13-0913	FGD1	ENST00000375135	p.D617N	c.1849G>A	Verified	aac	gac	cgc	1	G	A
TCGA-23-1809	FGD1	ENST00000375135	p.R866H	c.2597G>A	Unverified	cag	cgc	agc	2	G	A
TCGA-04-1338	FGD3	NM_033086.1	p.Q163Q	c.489G>A	Verified	gcc	cag	gag	3	G	A
TCGA-24-1470	FGF23	ENST00000237837	p.A197T	c.589G>A	Verified	cgg	gcc	cgg	1	G	A
TCGA-25-2392	FGFR4	ENST00000292410	p.R610H	c.1829G>A	Unverified	GGC	CGC	CTG	2	G	A
TCGA-25-2392	FGFR4_ENST	ENST00000292408	p.R650H	c.1949G>A	Unverified	GGC	CGC	CTG	2	G	A
TCGA-30-1718	FGR	ENST00000374005	p.R314Q	c.941G>A	Unverified	ctg	cgg	cac	2	G	A
TCGA-29-1770	FILIP1L	ENST00000331335	p.R321H	c.962G>A	Unverified	AAT	CGC	CAG	2	G	A
TCGA-29-1770	FILIP1L_ENS	ENST00000354552	p.R321H	c.962G>A	Unverified	AAT	CGC	CAG	2	G	A
TCGA-04-1530	FKBP9	ENST00000242209	p.R219H	c.656G>A	Verified	aag	cgc	atc	2	G	A
TCGA-23-1809	FLCN	ENST00000285071	p.V207M	c.619G>A	Unverified	aag	gtg	ttt	1	G	A
TCGA-23-1809	FLCN_ENST(	ENST00000389169	p.V207M	c.619G>A	Unverified	AAG	GTG	TTT	1	G	A
TCGA-29-1691	FLG	ENST00000368799	p.A1206T	c.3616G>A	Unverified	GAT	GCC	TCC	1	G	A
TCGA-13-0924	FLG	ENST00000368799	p.R2018K	c.6053G>A	Verified	TCC	AGA	CAC	2	G	A
TCGA-23-2078	FLG	ENST00000368799	p.S2897N	c.8690G>A	Unverified	GAC	AGT	GAG	2	G	A
TCGA-24-1844	FLG	ENST00000368799	p.R1036H	c.3107G>A	Unverified	TCC	CGC	CAC	2	G	A
TCGA-61-1733	FLG	ENST00000368799	p.G994D	c.2981G>A	Unverified	GCC	GGT	CAC	2	G	A
TCGA-61-1733	FLG	ENST00000368799	p.G994D	c.2981G>A	Unverified	GCC	GGT	CAC	2	G	A
TCGA-29-1696	FLG2	ENST00000388718	p.G1513D	c.4538G>A	Unverified	GGG	GGC	TCG	2	G	A
TCGA-29-1696	FLG2	ENST00000388718	p.G1513D	c.4538G>A	Unverified	GGG	GGC	TCG	2	G	A
TCGA-29-1761	FLG2	ENST00000388718	p.G736E	c.2207G>A	Unverified	CAT	GGA	TCA	2	G	A
TCGA-04-1336	FLI1	ENST00000429175	p.L283L	c.849G>A	Verified	cag	ctg	tgg	3	G	A
TCGA-29-1699	FLJ35880	ENST00000312481	p.D229N	c.685G>A	Unverified	AAT	GAT	GGT	1	G	A
TCGA-24-1423	FLJ35880	ENST00000312481	p.K191K	c.573G>A	Verified	GCG	AAG	TTC	3	G	A
TCGA-13-1498	FLRT2	ENST00000330753	p.Q382Q	c.1146G>A	Verified	ACT	CAG	CCT	3	G	A
TCGA-61-1737	FLT4_ENST0	ENST00000261937	p.S1344N	c.4031G>A	Unverified	CCA	AGC	GAG	2	G	A
TCGA-04-1530	FMN1	ENST00000414268	p.G1336R	c.4006G>A	Unverified	TTT	GGG	ATG	1	G	A
TCGA-09-1674	FMN2	ENST00000319653	p.S735S	c.2205G>A	Unverified	GCT	TCG	TTT	3	G	A
TCGA-61-2113	FMO5	ENST00000254090	p.D287N	c.859G>A	Verified	AAT	GAT	GAC	1	G	A
TCGA-13-1505	FMR1	ENST00000370475	p.R442Q	c.1325G>A	Verified	ttg	cga	cag	2	G	A
TCGA-13-0791	FNDC1	ENST00000297267	p.A664T	c.1990G>A	Verified	TTC	GCC	CAG	1	G	A
TCGA-13-1497	FNDC1	ENST00000297267	p.T1569T	c.4707G>A	Verified	GAG	ACG	TCA	3	G	A
TCGA-25-1326	FNIP1	ENST00000510461	p.R13K	c.38G>A	Unverified	AAG	AGG	ACC	2	G	A

TCGA-13-0905	FNTB	ENST00000246166	p.G369S	c.1105G>A	Verified	AGC	GGC	CTG	1	G	A
TCGA-24-1850	FOLH1	ENST00000256999	p.R527Q	c.1580G>A	Unverified	CAA	CGA	CTT	2	G	A
TCGA-13-0807	FOLH1B	ENST00000257034	p.L154L	c.462G>A	Verified	ACT	CTG	AGA	3	G	A
TCGA-10-0930	FOLR1	ENST00000393676	p.E191K	c.571G>A	Verified	AAT	GAA	ATC	1	G	A
TCGA-10-0930	FOLR1	ENST00000393676	p.E191K	c.571G>A	Verified	AAT	GAA	ATC	1	G	A
TCGA-29-1784	FOLR1	ENST00000393676	p.A64T	c.190G>A	Unverified	AAT	GCC	TGC	1	G	A
TCGA-23-1021	FOSL2	ENST00000264716	p.E150K	c.448G>A	Verified	ACA	GAG	AAG	1	G	A
TCGA-24-1103	FOXJ2	ENST00000162391	p.G279R	c.835G>A	Verified	ctg	ggg	gac	1	G	A
TCGA-25-2392	FOXJ3	ENST00000372573	p.L202L	c.606G>A	Verified	act	ctg	gca	3	G	A
TCGA-09-1665	FOXP2	ENST00000408937	p.S660N	c.1979G>A	Verified	TCC	AGT	GGC	2	G	A
TCGA-04-1530	FOXP3	ENST00000376207	p.V120V	c.360G>A	Unverified	cag	gtg	cac	3	G	A
TCGA-29-1711	FPR3	ENST00000339223	p.E311K	c.931G>A	Unverified	CAA	GAA	AGA	1	G	A
TCGA-29-1711	FPR3	ENST00000339223	p.E311K	c.931G>A	Unverified	CAA	GAA	AGA	1	G	A
TCGA-61-2012	FRAS1_ENST	ENST00000264895	p.G2391S	c.7171G>A	Verified	GAC	GGC	AGT	1	G	A
TCGA-24-1469	FRAS1_ENST	ENST00000325942	p.D44N	c.130G>A	Verified	CCC	GAT	TCA	1	G	A
TCGA-13-1497	FRAS1_ENST	ENST00000325942	p.Q1639Q	c.4917G>A	Verified	CCA	CAG	CAT	3	G	A
TCGA-61-1899	FREM1	ENST00000422223	p.V1719M	c.5155G>A	Unverified	ACC	GTG	GAA	1	G	A
TCGA-13-0893	FREM2	ENST00000280481	p.V968I	c.2902G>A	Verified	AAT	GTT	ATT	1	G	A
TCGA-25-2042	FREM2	ENST00000280481	p.V484M	c.1450G>A	Verified	GTG	GTG	GCT	1	G	A
TCGA-13-0916	FRMD5	ENST00000417257	p.M509I	c.1527G>A	Verified	ACC	ATG	GGA	3	G	A
TCGA-10-0930	FRMPD3	XM_042978.4	p.V67I	c.199G>A	Verified	acc	gtt	cac	1	G	A
TCGA-10-0930	FRMPD3	XM_042978.4	p.V67I	c.199G>A	Verified	acc	gtt	cac	1	G	A
TCGA-29-1763	FSD1	ENST00000221856	p.V288M	c.862G>A	Unverified	TCC	GTG	GAG	1	G	A
TCGA-61-1738	FTSJ3	ENST00000582115	p.V801I	c.2401G>A	Unverified	TAC	GTT	GTA	1	G	A
TCGA-61-1738	FTSJ3	ENST00000582115	p.V801I	c.2401G>A	Unverified	TAC	GTT	GTA	1	G	A
TCGA-36-1577	FURIN	ENST00000268171	p.G653E	c.1958G>A	Unverified	cag	ggg	ccg	2	G	A
TCGA-24-1845	FXR2	ENST00000250113	p.V94V	c.282G>A	Unverified	CGG	GTG	CGG	3	G	A
TCGA-13-0887	FZD2	ENST00000315323	p.S515S	c.1545G>A	Verified	atg	tcg	ccc	3	G	A
TCGA-25-1313	G2E3	ENST00000206595	p.D535N	c.1603G>A	Verified	agt	gat	aaa	1	G	A
TCGA-13-0920	G6PC	ENST00000253801	p.V304I	c.910G>A	Verified	CTC	GTC	CTC	1	G	A
TCGA-23-2078	G6PC3	ENST00000269097	p.L185L	c.555G>A	Unverified	TGG	CTG	ATG	3	G	A
TCGA-20-1686	G6PD	ENST00000291567	p.M405I	c.1215G>A	Unverified	ATG	ATG	ACC	3	G	A
TCGA-20-1686	G6PD_ENST	ENST00000393562	p.M435I	c.1305G>A	Unverified	ATG	ATG	ACC	3	G	A
TCGA-24-1435	GAB2	ENST00000361507	p.D520N	c.1558G>A	Verified	cct	gat	cgg	1	G	A

TCGA-13-1505	GAB3	ENST00000369575	p.R27H	c.80G>A	Verified	tgg	cgc	aag	2	G	A
TCGA-13-0919	GABRA1	ENST00000393943	p.R94H	c.281G>A	Verified	TTC	CGT	CAA	2	G	A
TCGA-13-1497	GABRA3	ENST00000370314	p.A420T	c.1258G>A	Verified	ggc	gct	gct	1	G	A
TCGA-30-1718	GABRA3	ENST00000370314	p.V399M	c.1195G>A	Unverified	atc	gtg	ggg	1	G	A
TCGA-20-1685	GABRB3	ENST00000311550	p.S371S	c.1113G>A	Unverified	ACA	TCG	CTG	3	G	A
TCGA-20-1685	GABRB3_EN	ENST00000299267	p.S371S	c.1113G>A	Unverified	ACA	TCG	CTG	3	G	A
TCGA-20-1685	GABRB3_EN	ENST00000541819	p.S427S	c.1281G>A	Unverified	ACA	TCG	CTG	3	G	A
TCGA-24-1474	GABRE	NM_021990	p.R215H	c.644G>A	Verified	tct	cgt	aag	2	G	A
TCGA-09-2056	GAL3ST4	ENST00000360039	p.A162A	c.486G>A	Verified	CCA	GCG	GCT	3	G	A
TCGA-23-1118	GALNT1	ENST00000269195	p.V400I	c.1198G>A	Verified	AGA	GTT	GGT	1	G	A
TCGA-24-1604	GALNT1	ENST00000269195	p.D244N	c.730G>A	Unverified	GAT	GAT	ACT	1	G	A
TCGA-24-2280	GALNT1	ENST00000269195	p.L25L	c.75G>A	Unverified	CTG	CTG	CTT	3	G	A
TCGA-20-1685	GALNT13	ENST00000392825	p.L222L	c.666G>A	Unverified	TTG	CTG	GCA	3	G	A
TCGA-30-1714	GALNT5	ENST00000259056	p.G146R	c.436G>A	Unverified	gac	ggg	aga	1	G	A
TCGA-25-2400	GALNT6	ENST00000356317	p.D602N	c.1804G>A	Verified	CAG	GAC	AAA	1	G	A
TCGA-23-1117	GALNTL5	ENST00000392800	p.G401S	c.1201G>A	Verified	CCT	GGT	CTG	1	G	A
TCGA-23-1117	GALNTL5	ENST00000392800	p.G401S	c.1201G>A	Verified	CCT	GGT	CTG	1	G	A
TCGA-23-1022	GAN	ENST00000248272	p.R286Q	c.857G>A	Verified	TCA	CGG	AAA	2	G	A
TCGA-09-1674	GAPVD1	ENST00000394105	p.V535I	c.1603G>A	Unverified	GAG	GTC	CTA	1	G	A
TCGA-24-1844	GAS2L3	ENST00000266754	p.D681N	c.2041G>A	Unverified	GAT	GAC	CAT	1	G	A
TCGA-23-1021	GATA3	ENST00000379328	p.S175S	c.525G>A	Verified	ggc	tcg	gcc	3	G	A
TCGA-29-1785	GATAD2B	ENST00000368655	p.M445I	c.1335G>A	Unverified	TGT	ATG	ACC	3	G	A
TCGA-29-1785	GATAD2B	ENST00000368655	p.M445I	c.1335G>A	Unverified	TGT	ATG	ACC	3	G	A
TCGA-24-1616	GCC1	ENST00000321407	p.R535Q	c.1604G>A	Verified	CTG	CGG	CTC	2	G	A
TCGA-61-1910	GCDH	ENST00000222214	p.A260T	c.778G>A	Unverified	TCA	GCC	ACA	1	G	A
TCGA-61-1910	GCDH_ENST	ENST00000457854	p.A260T	c.778G>A	Unverified	TCA	GCC	ACA	1	G	A
TCGA-13-0761	GCLC	ENST00000229416	p.R575K	c.1724G>A	Unverified	GCC	AGA	TGG	2	G	A
TCGA-13-0761	GCLC	ENST00000229416	p.R575K	c.1724G>A	Unverified	GCC	AGA	TGG	2	G	A
TCGA-25-2042	GCM2	ENST00000379491	p.P433P	c.1299G>A	Verified	CCT	CCG	GTG	3	G	A
TCGA-24-2288	GDF11	ENST00000257868	p.K90K	c.270G>A	Unverified	ctc	aag	gag	3	G	A
TCGA-30-1857	GDF5	ENST00000374369	p.E371E	c.1113G>A	Unverified	tat	gag	tac	3	G	A
TCGA-23-1029	GDNF	ENST00000326524	p.M83I	c.249G>A	Unverified	CAA	ATG	GCA	3	G	A
TCGA-23-1029	GDNF_ENST	ENST00000427982	p.M100I	c.300G>A	Unverified	CAA	ATG	GCA	3	G	A
TCGA-29-2427	GFI1	ENST00000294702	p.R82Q	c.245G>A	Unverified	GAA	CGG	AGC	2	G	A

TCGA-25-2400	GFOD1	ENST00000379287	p.E136K	c.406G>A	Verified	ATC	GAG	GAG	1	G	A
TCGA-61-1733	GFRA3	ENST00000274721	p.L87L	c.261G>A	Unverified	TGC	CTG	GAG	3	G	A
TCGA-61-1733	GFRA3	ENST00000274721	p.L87L	c.261G>A	Unverified	TGC	CTG	GAG	3	G	A
TCGA-04-1367	GGA1	ENST00000343632	p.M362I	c.1086G>A	Verified	CTC	ATG	TCT	3	G	A
TCGA-29-1766	GGT1	ENST00000400380	p.A110T	c.328G>A	Unverified	GTG	GCC	CCC	1	G	A
TCGA-13-0913	GGT5	ENST00000327365	p.V323I	c.967G>A	Verified	CTT	GTA	GAG	1	G	A
TCGA-29-1770	GHSR_ENST	ENST00000427970	p.R270H	c.809G>A	Unverified	CAG	CGC	GCG	2	G	A
TCGA-24-1846	GIGYF1	ENST00000275732	p.G218R	c.652G>A	Unverified	GCA	GGG	CCC	1	G	A
TCGA-29-1783	GIGYF2	ENST00000373563	p.E801K	c.2401G>A	Unverified	CAA	GAA	ATT	1	G	A
TCGA-29-1783	GIGYF2_ENS	ENST00000409451	p.E822K	c.2464G>A	Unverified	CAA	GAA	ATT	1	G	A
TCGA-09-1674	GIMAP1	ENST00000307194	p.A114A	c.342G>A	Unverified	CAC	GCG	CTG	3	G	A
TCGA-61-2095	GIT1	ENST00000225394	p.K331K	c.993G>A	Unverified	caa	aag	ctg	3	G	A
TCGA-30-1718	GJA8	ENST00000369235	p.K303K	c.909G>A	Unverified	GAG	AAG	ATC	3	G	A
TCGA-30-1714	GJB4	ENST00000339480	p.R32H	c.95G>A	Unverified	TTT	CGT	GTG	2	G	A
TCGA-24-1435	GJB5	ENST00000338513	p.D247N	c.739G>A	Verified	GGT	GAC	CTC	1	G	A
TCGA-09-1665	GLI1	ENST00000228682	p.S112S	c.336G>A	Verified	aac	tcg	cga	3	G	A
TCGA-23-1117	GLI2	ENST00000361492	p.G1519D	c.4556G>A	Verified	GAT	GGC	GAT	2	G	A
TCGA-23-1117	GLI2	ENST00000361492	p.G1519D	c.4556G>A	Verified	GAT	GGC	GAT	2	G	A
TCGA-09-2050	GLO1	ENST00000373365	p.C20Y	c.59G>A	Verified	TGC	TGC	TCC	2	G	A
TCGA-25-1313	GLP2R	ENST00000262441	p.L434L	c.1302G>A	Verified	GCC	TTG	CAG	3	G	A
TCGA-09-2044	GLRA3	ENST00000274093	p.A335T	c.1003G>A	Verified	TAT	GCA	GCT	1	G	A
TCGA-61-1913	GLT1D1	ENST00000281703	p.G236E	c.707G>A	Unverified	AAC	GGA	AGG	2	G	A
TCGA-13-0886	GMCL1	ENST00000282570	p.E322K	c.964G>A	Verified	ACT	GAA	CAA	1	G	A
TCGA-23-1021	GMIP	ENST00000203556	p.A607A	c.1821G>A	Verified	CGA	GCG	TTG	3	G	A
TCGA-09-0369	GNA12	ENST00000275364	p.R235H	c.704G>A	Verified	cag	cgc	cag	2	G	A
TCGA-09-0369	GNA12	ENST00000275364	p.R235H	c.704G>A	Verified	cag	cgc	cag	2	G	A
TCGA-13-0885	GNAI2	ENST00000313601	p.A114T	c.340G>A	Verified	ACC	GCC	GAG	1	G	A
TCGA-04-1338	GNAQ	ENST00000286548	p.E249K	c.745G>A	Verified	atg	gag	gaa	1	G	A
TCGA-29-1784	GNL2	ENST00000373062	p.E480K	c.1438G>A	Unverified	TTG	GAA	GTT	1	G	A
TCGA-23-1124	GOLGA2	ENST00000421699	p.D489N	c.1465G>A	Verified	CAA	GAC	AAT	1	G	A
TCGA-04-1336	GOLGA8C	ENST00000446144	p.V383V	c.1149G>A	Unverified	CCT	GTG	CAA	3	G	A
TCGA-13-0903	GOLGB1	ENST00000340645	p.A2789T	c.8365G>A	Verified	ACC	GCC	TTT	1	G	A
TCGA-04-1347	GOLPH3	ENST00000265070	p.A295A	c.885G>A	Verified	GCG	GCG	TTC	3	G	A
TCGA-25-1313	GON4L	ENST00000361040	p.D1237N	c.3709G>A	Verified	GCT	GAT	GGG	1	G	A

TCGA-24-2288	GON4L_ENS	ENST00000437809	p.D1620N	c.4858G>A	Unverified	CGA	GAT	CCA	1	G	A
TCGA-10-0930	GON4L_ENS	ENST00000437809	p.L1808L	c.5424G>A	Verified	GCC	CTG	CCT	3	G	A
TCGA-10-0930	GON4L_ENS	ENST00000437809	p.L1808L	c.5424G>A	Verified	GCC	CTG	CCT	3	G	A
TCGA-61-1900	GPAA1	ENST00000355091	p.R484Q	c.1451G>A	Unverified	CAC	CGG	GTG	2	G	A
TCGA-13-1408	GPATCH8	ENST00000335500	p.D321N	c.961G>A	Verified	AAG	GAC	CAT	1	G	A
TCGA-24-2035	GPBP1	ENST00000506184	p.G93R	c.277G>A	Unverified	GGT	GGA	AGT	1	G	A
TCGA-29-1769	GPBP1L1	ENST00000290795	p.A371T	c.1111G>A	Unverified	CTT	GCC	CTC	1	G	A
TCGA-09-1665	GPCPD1	ENST00000379019	p.D246N	c.736G>A	Unverified	GGT	GAT	GCC	1	G	A
TCGA-29-1690	GPR1	ENST00000407325	p.V59I	c.175G>A	Unverified	ATC	GTC	ATT	1	G	A
TCGA-23-1114	GPR1	ENST00000407325	p.S297N	c.890G>A	Unverified	AAT	AGT	TGC	2	G	A
TCGA-25-2400	GPR109B	ENST00000528880	p.G350S	c.1048G>A	Verified	TCC	GGT	GAG	1	G	A
TCGA-61-1914	GPR111	ENST00000398742	p.G387D	c.1160G>A	Unverified	CTG	GGC	ATT	2	G	A
TCGA-61-1914	GPR111_EN	ENST00000296862	p.G455D	c.1364G>A	Unverified	CTG	GGC	ATT	2	G	A
TCGA-24-1104	GPR112	ENST00000394143	p.G948E	c.2843G>A	Verified	gag	gga	att	2	G	A
TCGA-30-1857	GPR113	ENST00000333478	p.G484S	c.1450G>A	Unverified	CCT	GGC	CTG	1	G	A
TCGA-30-1857	GPR113_EN	ENST00000311519	p.G683S	c.2047G>A	Unverified	CCT	GGC	CTG	1	G	A
TCGA-61-1998	GPR115	ENST00000327753	p.W427*	c.1281G>A	Verified	GTG	TGG	TCC	3	G	A
TCGA-13-0760	GPR132	ENST00000329797	p.V145V	c.435G>A	Verified	GTG	GTG	TAC	3	G	A
TCGA-25-2398	GPR142	ENST00000335666	p.W66*	c.197G>A	Verified	AGC	TGG	GAC	2	G	A
TCGA-24-2290	GPR149	ENST00000389740	p.R542H	c.1625G>A	Verified	CAG	CGT	TCC	2	G	A
TCGA-24-2290	GPR149	ENST00000389740	p.R542H	c.1625G>A	Verified	CAG	CGT	TCC	2	G	A
TCGA-24-1844	GPR149	ENST00000389740	p.S84S	c.252G>A	Unverified	CTG	TCG	GTG	3	G	A
TCGA-13-0762	GPR174	ENST00000276077	p.G223E	c.668G>A	Unverified	ctt	gga	gag	2	G	A
TCGA-13-0762	GPR174	ENST00000276077	p.G223E	c.668G>A	Unverified	ctt	gga	gag	2	G	A
TCGA-24-1470	GPR22	ENST00000304402	p.V19V	c.57G>A	Verified	ACA	GTG	CGA	3	G	A
TCGA-29-1761	GPR32	ENST00000270590	p.A175A	c.525G>A	Unverified	TCT	GCG	CAC	3	G	A
TCGA-29-1783	GPR34	ENST00000378142	p.R152H	c.455G>A	Unverified	gat	cgc	tat	2	G	A
TCGA-30-1855	GPR39	ENST00000329321	p.S337S	c.1011G>A	Unverified	agc	tcg	gtc	3	G	A
TCGA-09-0369	GPR62	ENST00000322241	p.A315T	c.943G>A	Verified	CGG	GCC	TGC	1	G	A
TCGA-09-0369	GPR62	ENST00000322241	p.A315T	c.943G>A	Verified	CGG	GCC	TGC	1	G	A
TCGA-29-1769	GPR83	ENST00000243673	p.L6L	c.18G>A	Unverified	CTC	TTG	CTG	3	G	A
TCGA-23-1124	GPR98	ENST00000405460	p.R2424K	c.7271G>A	Verified	TGG	AGA	ACT	2	G	A
TCGA-13-1507	GPRC5C	ENST00000392627	p.A66T	c.196G>A	Verified	GGG	GCC	TGG	1	G	A
TCGA-29-1775	GPRIN3	ENST00000333209	p.R761Q	c.2282G>A	Unverified	TTC	CGA	CGC	2	G	A

TCGA-13-0807	GPS2	ENST00000389167	p.R272H	c.815G>A	Verified	CTG	CGC	CCC	2	G	A
TCGA-24-1846	GREB1_ENS1	ENST00000381486	p.G1031E	c.3092G>A	Unverified	AGT	GGG	ATG	2	G	A
TCGA-61-2095	GRHL3	ENST00000236255	p.V266I	c.796G>A	Verified	CCC	GTC	ACC	1	G	A
TCGA-29-2427	GRIA1	ENST00000285900	p.G898G	c.2694G>A	Verified	TCA	GGG	ATG	3	G	A
TCGA-23-1111	GRIA1	ENST00000285900	p.L638L	c.1914G>A	Unverified	TTC	CTG	ACC	3	G	A
TCGA-23-1111	GRIA1_ENST	ENST00000544403	p.L638L	c.1914G>A	Unverified	TTC	CTG	ACC	3	G	A
TCGA-61-1733	GRID2	ENST00000282020	p.D518N	c.1552G>A	Unverified	GCC	GAC	ATA	1	G	A
TCGA-61-1733	GRID2	ENST00000282020	p.D518N	c.1552G>A	Unverified	GCC	GAC	ATA	1	G	A
TCGA-20-1685	GRIK4	ENST00000527524	p.R198Q	c.593G>A	Unverified	ACC	CGG	GAC	2	G	A
TCGA-13-1498	GRIK5	ENST00000262895	p.R41H	c.122G>A	Unverified	GAG	CGT	CTG	2	G	A
TCGA-24-2262	GRIK5	ENST00000262895	p.R62Q	c.185G>A	Unverified	GCC	CGA	GTG	2	G	A
TCGA-13-0755	GRIN2A	ENST00000396573	p.G532E	c.1595G>A	Verified	acg	gga	atc	2	G	A
TCGA-24-2280	GRIN2D	ENST00000263269	p.E743K	c.2227G>A	Unverified	GAG	GAA	GCG	1	G	A
TCGA-24-2280	GRIN2D	ENST00000263269	p.K720K	c.2160G>A	Unverified	GAG	AAG	AAC	3	G	A
TCGA-04-1337	GRIPAP1_EN	ENST00000376441	p.R90H	c.269G>A	Unverified	TTC	CGT	TTG	2	G	A
TCGA-04-1337	GRIPAP1_EN	ENST00000376441	p.?	c.2433+1G>A	Unverified	CAC	AAG	GAT	3	G	A
TCGA-30-1718	GRM4	ENST00000374181	p.A180T	c.538G>A	Unverified	TAC	GCC	TCC	1	G	A
TCGA-30-1718	GRM4_ENST	ENST00000374177	p.A111T	c.331G>A	Unverified	TAC	GCC	TCC	1	G	A
TCGA-30-1718	GRM4_ENST	ENST00000374181	p.A180T	c.538G>A	Unverified	TAC	GCC	TCC	1	G	A
TCGA-36-1577	GRM5	ENST00000305432	p.A855T	c.2563G>A	Unverified	TCC	GCA	GCC	1	G	A
TCGA-23-2078	GRM7	ENST00000389335	p.R516Q	c.1547G>A	Verified	GTC	CGA	GAG	2	G	A
TCGA-23-1032	GRWD1	ENST00000253237	p.V446I	c.1336G>A	Verified	AGC	GTC	TGA	1	G	A
TCGA-29-1775	GSDMC	ENST00000276708	p.L477L	c.1431G>A	Unverified	GAG	CTG	GAT	3	G	A
TCGA-30-1714	GSN	ENST00000373823	p.G584S	c.1750G>A	Unverified	GAA	GGC	AGC	1	G	A
TCGA-29-1783	GSN	ENST00000373823	p.G143G	c.429G>A	Unverified	AAA	GGG	CGG	3	G	A
TCGA-30-1714	GSN_ENST0	ENST00000373818	p.G635S	c.1903G>A	Unverified	GAA	GGC	AGC	1	G	A
TCGA-29-1783	GSN_ENST0	ENST00000373818	p.G194G	c.582G>A	Unverified	AAA	GGG	CGG	3	G	A
TCGA-13-1505	GSTT1	ENST00000248935	p.T172T	c.516G>A	Verified	ATC	ACG	GAG	3	G	A
TCGA-04-1655	GTF2I	ENST00000324896	p.G588R	c.1762G>A	Unverified	CTT	GGA	CTC	1	G	A
TCGA-09-0369	GTF2IRD1	ENST00000265755	p.V375M	c.1123G>A	Verified	CCC	GTG	CCC	1	G	A
TCGA-09-0369	GTF2IRD1	ENST00000265755	p.V375M	c.1123G>A	Verified	CCC	GTG	CCC	1	G	A
TCGA-13-1488	GTF2IRD2B	ENST00000312575	p.S500S	c.1500G>A	Unverified	GGC	TCG	TCA	3	G	A
TCGA-13-0903	GTF3C1	ENST00000356183	p.G733E	c.2198G>A	Verified	CAA	GGG	GAG	2	G	A
TCGA-29-1705	GTF3C4	ENST00000372146	p.E667K	c.1999G>A	Unverified	GAA	GAG	AAA	1	G	A

TCGA-29-1784	GTSF1L	ENST00000373003	p.E72K	c.214G>A	Unverified	GAA	GAG	GAC	1	G	A
TCGA-23-1111	GUCY1A3	ENST00000296518	p.V233M	c.697G>A	Unverified	GAA	GTG	TCG	1	G	A
TCGA-13-0920	GYLTL1B	ENST00000325468	p.L393L	c.1179G>A	Verified	GCC	CTG	GCA	3	G	A
TCGA-30-1856	GYS2	ENST00000261195	p.R580H	c.1739G>A	Unverified	CGC	CGC	CAA	2	G	A
TCGA-29-1703	GZF1	ENST00000338121	p.R545H	c.1634G>A	Unverified	GAG	CGT	CCC	2	G	A
TCGA-13-0714	HAPLN1	ENST00000274341	p.R260H	c.779G>A	Verified	GGC	CGT	TTT	2	G	A
TCGA-13-0885	HAS1	ENST00000222115	p.C334Y	c.1001G>A	Verified	CAC	TGT	ACT	2	G	A
TCGA-61-1904	HAS1	ENST00000222115	p.R564Q	c.1691G>A	Unverified	GTG	CGG	AGG	2	G	A
TCGA-24-1563	HAS2	ENST00000303924	p.W148*	c.443G>A	Verified	ATC	TGG	AAG	2	G	A
TCGA-61-2102	HCAR2	ENST00000328880	p.L6L	c.18G>A	Verified	CAT	CTG	CAG	3	G	A
TCGA-61-2102	HCAR3	ENST00000528880	p.L6L	c.18G>A	Verified	CAT	CTG	CAG	3	G	A
TCGA-13-0885	HCK	ENST00000538448	p.E389K	c.1165G>A	Verified	AAC	GAG	TAC	1	G	A
TCGA-61-1904	HCN3	ENST00000368358	p.R296H	c.887G>A	Unverified	GGC	CGC	CAG	2	G	A
TCGA-24-1435	HCN4	ENST00000261917	p.V534M	c.1600G>A	Verified	CAG	GTG	GAG	1	G	A
TCGA-23-1809	HDAC10	ENST00000216271	p.G553S	c.1657G>A	Unverified	acc	ggt	ggt	1	G	A
TCGA-29-1775	HDAC6	ENST00000334136	p.G758S	c.2272G>A	Unverified	gag	ggt	tat	1	G	A
TCGA-36-1577	HDAC7	NM_015401.1	p.A808T	c.2422G>A	Unverified	ccg	gcc	cca	1	G	A
TCGA-09-2049	HEATR5B	ENST00000233099	p.A1671T	c.5011G>A	Verified	AGA	GCT	GCT	1	G	A
TCGA-24-1104	HEATR5B	ENST00000233099	p.A1951A	c.5853G>A	Verified	TTA	GCG	GTT	3	G	A
TCGA-10-0930	HECW1	ENST00000395891	p.R1124H	c.3371G>A	Verified	CTT	CGC	CAG	2	G	A
TCGA-10-0930	HECW1	ENST00000395891	p.R1124H	c.3371G>A	Verified	CTT	CGC	CAG	2	G	A
TCGA-61-2113	HECW1	ENST00000395891	p.L1391L	c.4173G>A	Verified	AGT	TTG	CAG	3	G	A
TCGA-30-1856	HECW1	ENST00000395891	p.T492T	c.1476G>A	Unverified	GCA	ACG	ACC	3	G	A
TCGA-30-1856	HECW1_ENS	ENST00000395891	p.T492T	c.1476G>A	Unverified	GCA	ACG	ACC	3	G	A
TCGA-23-1022	HECW2	ENST00000260983	p.S60N	c.179G>A	Verified	tcc	agc	tta	2	G	A
TCGA-24-1604	HEG1	ENST00000311127	p.E191E	c.573G>A	Verified	CTG	GAG	ATA	3	G	A
TCGA-04-1652	HEPH	NM_138737.1	p.L374L	c.1122G>A	Unverified	gac	ctg	ctc	3	G	A
TCGA-04-1652	HEPH	NM_138737.1	p.L374L	c.1122G>A	Unverified	gac	ctg	ctc	3	G	A
TCGA-29-1775	HERC3	ENST00000402738	p.S956S	c.2868G>A	Unverified	tac	tcg	gcc	3	G	A
TCGA-04-1367	HERPUD2	ENST00000396081	p.R285Q	c.854G>A	Verified	TCA	CGA	GCT	2	G	A
TCGA-30-1857	HFM1	ENST00000370425	p.?	c.3884-1G>A	Unverified	TCA	GGA	TTT	2	G	A
TCGA-61-2008	HGF	ENST00000222390	p.W329*	c.987G>A	Verified	CGT	TGG	GAT	3	G	A
TCGA-24-1417	HGS	ENST00000329138	p.K51K	c.153G>A	Verified	AAG	AAG	AAA	3	G	A
TCGA-24-1463	HHAT	ENST00000367010	p.D235N	c.703G>A	Verified	CAT	GAC	TCC	1	G	A

TCGA-13-1507	HHAT	ENST00000367010	p.R348K	c.1043G>A	Verified	ATC	AGG	TAT	2	G	A
TCGA-20-0991	HHATL	ENST00000310417	p.G167D	c.500G>A	Verified	ACA	GGC	ACT	2	G	A
TCGA-29-1762	HIATL1	ENST00000375344	p.V351M	c.1051G>A	Unverified	ACC	GTG	GCT	1	G	A
TCGA-13-1488	HIATL1	ENST00000375344	p.R109R	c.327G>A	Verified	GGG	AGG	AAG	3	G	A
TCGA-29-1691	HIP1	ENST00000336926	p.L279L	c.837G>A	Unverified	AAC	CTG	CAG	3	G	A
TCGA-61-1914	HIST1H1C	ENST00000343677	p.V40V	c.120G>A	Unverified	ccg	gtg	tca	3	G	A
TCGA-25-1326	HIST1H1E	ENST00000304218	p.K217K	c.651G>A	Unverified	GCC	AAG	AAA	3	G	A
TCGA-29-1705	HIST1H2AD	ENST00000341023	p.K37K	c.111G>A	Unverified	CGC	AAG	GGC	3	G	A
TCGA-24-1469	HIST1H2BO_	ENST00000303806	p.E36K	c.106G>A	Unverified	AAA	GAG	AGT	1	G	A
TCGA-24-1422	HIST1H3G	ENST00000305910	p.V47V	c.141G>A	Unverified	ACC	GTG	GCT	3	G	A
TCGA-23-2078	HIST1H4E	ENST00000360441	p.R79H	c.236G>A	Unverified	AAA	CGC	AAG	2	G	A
TCGA-04-1651	HIST1H4K	ENST00000357549	p.A90T	c.268G>A	Unverified	TAC	GCG	CTC	1	G	A
TCGA-04-1651	HIST1H4K	ENST00000357549	p.A90T	c.268G>A	Unverified	TAC	GCG	CTC	1	G	A
TCGA-13-0916	HIST1H4K	ENST00000357549	p.V66V	c.198G>A	Unverified	AAC	GTG	ATC	3	G	A
TCGA-23-1021	HIST1H4L	ENST00000355981	p.D25N	c.73G>A	Verified	CGC	GAC	AAC	1	G	A
TCGA-23-1031	HIST3H2A	ENST00000366695	p.L97L	c.291G>A	Verified	AAG	CTG	CTG	3	G	A
TCGA-13-1488	HIST4H4	ENST00000358064	p.R40Q	c.119G>A	Verified	GCC	CGA	CGT	2	G	A
TCGA-24-1470	HIVEP1	ENST00000379388	p.G916R	c.2746G>A	Verified	ACT	GGA	CAG	1	G	A
TCGA-24-1104	HIVEP2	ENST00000012134	p.S157N	c.470G>A	Verified	TCA	AGT	CTG	2	G	A
TCGA-20-0990	HIVEP3	ENST00000372583	p.V475M	c.1423G>A	Verified	GCC	GTG	GTG	1	G	A
TCGA-13-1509	HK3	ENST00000292432	p.G12E	c.35G>A	Verified	CAG	GGG	GAA	2	G	A
TCGA-61-1737	HKDC1	ENST00000354624	p.L874L	c.2622G>A	Unverified	ATA	TTG	CAG	3	G	A
TCGA-09-2050	HLA-DRA	ENST00000395388	p.M98I	c.294G>A	Verified	ATC	ATG	ACA	3	G	A
TCGA-13-0755	HLCS	ENST00000399120	p.S83N	c.248G>A	Verified	GGG	AGT	GAG	2	G	A
TCGA-29-1702	HMBS	ENST00000278715	p.R116Q	c.347G>A	Unverified	AAG	CGG	GAA	2	G	A
TCGA-13-0885	HMCN1	ENST00000271588	p.D360N	c.1078G>A	Verified	ATA	GAT	CTT	1	G	A
TCGA-29-1693	HMCN1	ENST00000271588	p.R5172H	c.15515G>A	Unverified	TAT	CGC	TGT	2	G	A
TCGA-29-1781	HMCN1	ENST00000271588	p.R3795Q	c.11384G>A	Unverified	AGG	CGA	ATA	2	G	A
TCGA-61-2102	HMCN1	ENST00000271588	p.R2533H	c.7598G>A	Verified	GGC	CGT	TTT	2	G	A
TCGA-29-1701	HMGCR	ENST00000511206	p.E446K	c.1336G>A	Unverified	aat	gaa	gaa	1	G	A
TCGA-24-2024	HMGCS2	ENST00000369406	p.W179*	c.537G>A	Verified	AAC	TGG	ATG	3	G	A
TCGA-24-1849	HMMR	ENST00000358715	p.E203K	c.607G>A	Unverified	CTC	GAA	GAG	1	G	A
TCGA-13-0884	HMP19	ENST00000303177	p.R131H	c.392G>A	Unverified	AGC	CGC	TTC	2	G	A
TCGA-24-0979	HNF4A	ENST00000316099	p.L393L	c.1179G>A	Verified	cac	ctg	atg	3	G	A

TCGA-10-0930	HNRNPA1	ENST00000546500	p.E28K	c.82G>A	Verified	GAT	GAG	AGC	1	G	A
TCGA-10-0930	HNRNPA1	ENST00000546500	p.E28K	c.82G>A	Verified	GAT	GAG	AGC	1	G	A
TCGA-25-2401	HOXA1	ENST00000343060	p.G133E	c.398G>A	Verified	TCT	GGA	AAT	2	G	A
TCGA-13-1497	HOXA6	ENST00000222728	p.V6V	c.18G>A	Verified	TTT	GTG	AAT	3	G	A
TCGA-13-0923	HOXB4	ENST00000332503	p.A135T	c.403G>A	Verified	TCC	GCG	TGC	1	G	A
TCGA-04-1367	HOXD10	ENST00000249501	p.V213I	c.637G>A	Unverified	AAA	GTC	TCC	1	G	A
TCGA-13-0884	HOXD10	ENST00000249501	p.E250K	c.748G>A	Verified	GAG	GAA	ATC	1	G	A
TCGA-61-1733	HOXD10	ENST00000249501	p.V148I	c.442G>A	Unverified	CCC	GTC	CCT	1	G	A
TCGA-61-1733	HOXD10	ENST00000249501	p.V148I	c.442G>A	Unverified	CCC	GTC	CCT	1	G	A
TCGA-09-2050	HPS5	ENST00000349215	p.A1002T	c.3004G>A	Verified	gag	gcc	ttc	1	G	A
TCGA-04-1347	HPSE	ENST00000311412	p.V268I	c.802G>A	Verified	GAT	GTT	GGT	1	G	A
TCGA-29-1690	HRNR	ENST00000368801	p.G2277S	c.6829G>A	Unverified	CAC	GGT	CAG	1	G	A
TCGA-29-1775	HS2ST1	ENST00000370550	p.R188R	c.564G>A	Unverified	TTA	CGG	AGA	3	G	A
TCGA-29-1775	HS2ST1_ENS	ENST00000370548	p.R162R	c.486G>A	Unverified	TTA	CGG	AGA	3	G	A
TCGA-04-1342	HSD17B7	ENST00000254521	p.P245P	c.735G>A	Verified	ATG	CCG	GCA	3	G	A
TCGA-61-1910	HSF2BP	ENST00000291560	p.S167S	c.501G>A	Unverified	AAG	TCG	TTA	3	G	A
TCGA-29-1698	HSPB9	ENST00000355067	p.K153K	c.459G>A	Unverified	tct	aag	gct	3	G	A
TCGA-24-2288	HSPG2	ENST00000374695	p.R3732K	c.11195G>A	Unverified	GGA	AGG	CCC	2	G	A
TCGA-23-1111	HTR1A	ENST00000323865	p.A280T	c.838G>A	Unverified	GGC	GCG	GTG	1	G	A
TCGA-29-1702	HTR1A	ENST00000323865	p.A131T	c.391G>A	Unverified	ATC	GCG	CTG	1	G	A
TCGA-29-1766	HTR2C	ENST00000371951	p.V208M	c.622G>A	Unverified	tgc	gtg	ctc	1	G	A
TCGA-24-1844	HTR3C	ENST00000318351	p.G139S	c.415G>A	Unverified	TCC	GGT	CTC	1	G	A
TCGA-61-2012	HTR3C	ENST00000318351	p.V128M	c.382G>A	Verified	ATC	GTG	GAA	1	G	A
TCGA-04-1337	HTR5A	ENST00000287907	p.V239I	c.715G>A	Verified	AGC	GTC	TCA	1	G	A
TCGA-23-1116	HTRA3	ENST00000307358	p.A331T	c.991G>A	Unverified	TTT	GCC	ATC	1	G	A
TCGA-30-1718	HTT	ENST00000355072	p.L1641L	c.4923G>A	Unverified	ATT	TTG	GCC	3	G	A
TCGA-13-0884	HTT	ENST00000355072	p.S556S	c.1668G>A	Verified	TCG	TCG	CCC	3	G	A
TCGA-29-1691	HUS1	ENST00000436444	p.S85S	c.255G>A	Unverified	aca	tcg	gaa	3	G	A
TCGA-04-1356	HUWE1	XM_497119.1	p.S2047N	c.6140G>A	Unverified	atc	agt	act	2	G	A
TCGA-09-2050	HYAL3	ENST00000336307	p.C19Y	c.56G>A	Unverified	GGT	TGT	GGC	2	G	A
TCGA-25-1318	HYDIN	ENST00000321489	p.M994I	c.2982G>A	Unverified	AGC	ATG	GAG	3	G	A
TCGA-25-1318	HYDIN	ENST00000321489	p.M994I	c.2982G>A	Unverified	AGC	ATG	GAG	3	G	A
TCGA-24-1844	HYDIN_ENST	ENST00000316490_v65	p.A3778T	c.11332G>A	Unverified	CCT	GCT	CAC	1	G	A
TCGA-61-1914	HYDIN_ENST	ENST00000316490_v65	p.E3130K	c.9388G>A	Unverified	ATT	GAG	CAC	1	G	A

TCGA-61-1738	HYDIN_ENST	ENST00000316490_v65	p.R3964H	c.11891G>A	Unverified	CAG	CGC	AAC	2	G	A
TCGA-61-1738	HYDIN_ENST	ENST00000316490_v65	p.R3964H	c.11891G>A	Unverified	CAG	CGC	AAC	2	G	A
TCGA-29-1770	HYDIN_ENST	ENST00000316490_v65	p.Q1050Q	c.3150G>A	Unverified	ATC	CAG	CTC	3	G	A
TCGA-23-1021	HYI	ENST00000372430	p.D91N	c.271G>A	Verified	CTG	GAC	ACG	1	G	A
TCGA-20-0991	IER5	ENST00000367577	p.K29K	c.87G>A	Unverified	ATC	AAG	CTG	3	G	A
TCGA-04-1649	IFLTD1	ENST00000282881	p.A213T	c.637G>A	Unverified	GAA	GCA	AAG	1	G	A
TCGA-04-1649	IFLTD1_ENS	ENST00000458174	p.A234T	c.700G>A	Unverified	GAA	GCA	AAG	1	G	A
TCGA-24-1850	IFNAR1	ENST00000270139	p.E522K	c.1564G>A	Unverified	GAT	GAA	GAT	1	G	A
TCGA-13-0887	IFT57	ENST00000264538	p.E184K	c.550G>A	Verified	gca	gaa	tta	1	G	A
TCGA-61-1740	IFT80	ENST00000326448	p.L580L	c.1740G>A	Unverified	TCC	CTG	GTT	3	G	A
TCGA-25-2042	IGDCC3	ENST00000327987	p.R377R	c.1131G>A	Verified	GTC	AGG	CTC	3	G	A
TCGA-20-1686	IGF2BP1	ENST00000290341	p.V13M	c.37G>A	Unverified	agc	gtg	acc	1	G	A
TCGA-24-1469	IGF2BP3	ENST00000258729	p.E341E	c.1023G>A	Verified	GAG	GAG	GAG	3	G	A
TCGA-24-1845	IGHMBP2	ENST00000255078	p.G589S	c.1765G>A	Unverified	GTT	GGT	TTT	1	G	A
TCGA-04-1343	IGSF21	ENST00000251296	p.A459T	c.1375G>A	Verified	CTC	GCC	CTG	1	G	A
TCGA-23-1118	IGSF22	ENST00000319338	p.?	c.2695+1G>A	Verified	CCA	GGG	CTC	1	G	A
TCGA-24-2280	IGSF3	ENST00000369486	p.V1036M	c.3106G>A	Unverified	AGC	GTG	GGC	1	G	A
TCGA-23-1032	IL12RB2	ENST00000262345	p.G370R	c.1108G>A	Unverified	GGA	GGG	AAA	1	G	A
TCGA-61-1740	IL16	NM_172217.1	p.V218M	c.652G>A	Unverified	atc	gtg	ctg	1	G	A
TCGA-61-1740	IL16_ENST0(	ENST00000302987	p.V218M	c.652G>A	Unverified	ATC	GTG	CTG	1	G	A
TCGA-29-1777	IL17RA	ENST00000319363	p.?	c.598+1G>A	Unverified	TCA	GGC	AGC	1	G	A
TCGA-09-1665	IL17RC	ENST00000295981	p.E269K	c.805G>A	Verified	CTC	GAA	GTC	1	G	A
TCGA-29-1770	IL1R2	ENST00000393414	p.A284T	c.850G>A	Unverified	AGC	GCC	TAC	1	G	A
TCGA-13-0762	IL1RAPL1	ENST00000378993	p.K440K	c.1320G>A	Verified	GAA	AAG	CAT	3	G	A
TCGA-13-0762	IL1RAPL1	ENST00000378993	p.K440K	c.1320G>A	Verified	GAA	AAG	CAT	3	G	A
TCGA-23-1116	IL20RA	ENST00000316649	p.W482*	c.1445G>A	Unverified	GAC	TGG	GAT	2	G	A
TCGA-25-2398	IL21R	ENST00000337929	p.G422E	c.1265G>A	Verified	GCA	GGG	ACC	2	G	A
TCGA-24-1417	IL21R	ENST00000337929	p.W253*	c.759G>A	Verified	TTC	TGG	AGC	3	G	A
TCGA-61-1899	IL28RA	ENST00000327535	p.E378K	c.1132G>A	Unverified	AGC	GAA	GGC	1	G	A
TCGA-24-2290	IL2RA	ENST00000379959	p.R176K	c.527G>A	Verified	ACA	AGG	TGG	2	G	A
TCGA-24-2290	IL2RA	ENST00000379959	p.R176K	c.527G>A	Verified	ACA	AGG	TGG	2	G	A
TCGA-25-2392	IL36A	ENST00000259211	p.G80R	c.238G>A	Verified	GTC	GGG	GAC	1	G	A
TCGA-61-2012	IL36B	ENST00000259213	p.K97K	c.291G>A	Verified	GGG	AAG	GAC	3	G	A
TCGA-04-1347	IL37	ENST00000263326	p.L98L	c.294G>A	Verified	TCC	TTG	AGC	3	G	A

TCGA-13-0913	IL4R	ENST00000395762	p.E577K	c.1729G>A	Verified	cag	gag	ttt	1	G	A
TCGA-25-2400	IL5RA	ENST00000446632	p.R253H	c.758G>A	Verified	ACT	CGT	CTC	2	G	A
TCGA-09-1665	IL6R	ENST00000368485	p.S443S	c.1329G>A	Verified	ACC	TCG	AGC	3	G	A
TCGA-23-1032	ILF3	ENST00000318511	p.Q423Q	c.1269G>A	Verified	TCC	CAG	ACT	3	G	A
TCGA-09-0369	ILKAP	ENST00000254654	p.T220T	c.660G>A	Verified	GCC	ACG	TGT	3	G	A
TCGA-09-0369	ILKAP	ENST00000254654	p.T220T	c.660G>A	Verified	GCC	ACG	TGT	3	G	A
TCGA-13-1510	IMPDH1	ENST00000338791	p.D341N	c.1021G>A	Unverified	gat	gac	aaa	1	G	A
TCGA-24-1850	IMPDH1	ENST00000338791	p.T479T	c.1437G>A	Unverified	act	acg	gag	3	G	A
TCGA-61-2012	IMPG1	ENST00000369950	p.K248K	c.744G>A	Verified	TTC	AAG	GCA	3	G	A
TCGA-23-1111	INPP5D_ENS	ENST00000359570	p.E636K	c.1906G>A	Unverified	GAG	GAA	GAA	1	G	A
TCGA-23-1031	INPP5D_ENS	ENST00000359570	p.E69K	c.205G>A	Unverified	TCC	GAA	GGC	1	G	A
TCGA-24-2289	INPP5F	ENST00000361976	p.E597K	c.1789G>A	Unverified	cag	gaa	cta	1	G	A
TCGA-04-1343	INPPL1	ENST00000298229	p.R342Q	c.1025G>A	Verified	GGG	CGG	CTG	2	G	A
TCGA-09-2049	INSRR	ENST00000368195	p.V698I	c.2092G>A	Verified	CAG	GTT	CTG	1	G	A
TCGA-20-0990	INSRR	ENST00000368195	p.S353N	c.1058G>A	Verified	GGA	AGC	CTC	2	G	A
TCGA-29-1785	INSRR_ENST	ENST00000368195	p.A681T	c.2041G>A	Unverified	GAG	GCC	GAG	1	G	A
TCGA-29-1785	INSRR_ENST	ENST00000368195	p.A681T	c.2041G>A	Unverified	GAG	GCC	GAG	1	G	A
TCGA-23-1809	INTU	ENST00000335251	p.D504N	c.1510G>A	Unverified	GAG	GAT	TAC	1	G	A
TCGA-13-1489	INVS	ENST00000262457	p.K279K	c.837G>A	Verified	AAT	AAG	TCT	3	G	A
TCGA-24-0975	IPCEF1	ENST00000367220	p.A11T	c.31G>A	Unverified	AGT	GCT	CTT	1	G	A
TCGA-61-2109	IPO13	ENST00000372343	p.V548M	c.1642G>A	Unverified	TCT	GTG	TCC	1	G	A
TCGA-09-1674	IPO9	ENST00000361565	p.V124M	c.370G>A	Unverified	AAA	GTG	CGC	1	G	A
TCGA-13-1499	IPO9	ENST00000361565	p.E192K	c.574G>A	Verified	CCA	GAG	ATG	1	G	A
TCGA-13-0887	IPPK	ENST00000287996	p.R267Q	c.800G>A	Verified	ACA	CGG	GTG	2	G	A
TCGA-25-2391	IQCE	ENST00000402050	p.E409K	c.1225G>A	Unverified	CAG	GAG	CAG	1	G	A
TCGA-24-1431	IQGAP2	ENST00000274364	p.D1260N	c.3778G>A	Verified	GTT	GAC	CCC	1	G	A
TCGA-04-1638	IQGAP3	ENST00000361170	p.G1316G	c.3948G>A	Unverified	CTT	GGG	GAG	3	G	A
TCGA-61-1907	IQGAP3	ENST00000361170	p.L811L	c.2433G>A	Unverified	TAC	CTG	AGG	3	G	A
TCGA-24-1474	IQSEC1	ENST00000273221	p.V593I	c.1777G>A	Unverified	TGC	GTC	GTG	1	G	A
TCGA-61-1737	IQSEC1	ENST00000273221	p.R549H	c.1646G>A	Unverified	GAG	CGT	GGC	2	G	A
TCGA-24-1435	IQSEC2	XM_291345.2	p.A401A	c.1203G>A	Verified	ccc	gcg	tac	3	G	A
TCGA-13-1512	IRAK1	ENST00000369980	p.E587K	c.1759G>A	Verified	GTG	GAG	AGT	1	G	A
TCGA-04-1649	IREB2	ENST00000258886	p.D463N	c.1387G>A	Unverified	AGC	GAT	TTC	1	G	A
TCGA-61-1914	IRF2BP2	ENST00000366609	p.P414P	c.1242G>A	Unverified	ACA	CCG	CCT	3	G	A

TCGA-13-0762	IRF3	ENST00000309877	p.E137K	c.409G>A	Verified	CAG	GAA	GAC	1	G	A
TCGA-13-0762	IRF3	ENST00000309877	p.E137K	c.409G>A	Verified	CAG	GAA	GAC	1	G	A
TCGA-23-1021	IRF4	ENST00000380956	p.V383V	c.1149G>A	Verified	CAG	GTG	ACT	3	G	A
TCGA-29-1690	IRF5	NM_002200.3	p.?	c.543-1G>A	Unverified	aag	tgg	ccg	3	G	A
TCGA-04-1638	IRS4	ENST00000372129	p.G787S	c.2359G>A	Unverified	gcc	ggt	gca	1	G	A
TCGA-23-1021	IRS4	ENST00000372129	p.A788T	c.2362G>A	Verified	ggt	gca	att	1	G	A
TCGA-24-1844	ISG20L2	ENST00000368219	p.G39D	c.116G>A	Unverified	AGA	GGC	TTT	2	G	A
TCGA-13-1507	ISX	ENST00000308700	p.S187S	c.561G>A	Verified	CCA	TCG	GCT	3	G	A
TCGA-25-2401	ITGA1	ENST00000282588	p.R266Q	c.797G>A	Verified	gcc	cgg	ggt	2	G	A
TCGA-04-1367	ITGA10	ENST00000369304	p.R521H	c.1562G>A	Verified	GGA	CGT	GTT	2	G	A
TCGA-13-0791	ITGA2B	ENST00000262407	p.G472R	c.1414G>A	Verified	tac	ggg	gcc	1	G	A
TCGA-61-1914	ITGA2B	ENST00000262407	p.D632N	c.1894G>A	Unverified	ctg	gac	tgt	1	G	A
TCGA-13-0900	ITGA2B	ENST00000262407	p.?	c.2187+1G>A	Unverified	gcc	cag	ata	3	G	A
TCGA-24-1850	ITGA8	ENST00000378076	p.T307T	c.921G>A	Unverified	ATG	ACG	TTT	3	G	A
TCGA-29-1699	ITGAD	ENST00000389202	p.R859H	c.2576G>A	Unverified	AGC	CGC	TGC	2	G	A
TCGA-13-0900	ITGAL	ENST00000356798	p.E506K	c.1516G>A	Verified	GAA	GAA	GTC	1	G	A
TCGA-13-0903	ITGAL	ENST00000356798	p.V58M	c.172G>A	Verified	ATC	GTG	GGA	1	G	A
TCGA-09-0369	ITGAX	ENST00000268296	p.T339T	c.1017G>A	Verified	ggt	acg	gag	3	G	A
TCGA-09-0369	ITGAX	ENST00000268296	p.T339T	c.1017G>A	Verified	ggt	acg	gag	3	G	A
TCGA-24-2290	ITGB2	ENST00000397852	p.V439V	c.1317G>A	Verified	ACC	GTG	CAG	3	G	A
TCGA-24-2290	ITGB2	ENST00000397852	p.V439V	c.1317G>A	Verified	ACC	GTG	CAG	3	G	A
TCGA-13-0923	ITGB7	ENST00000422257	p.V185I	c.553G>A	Verified	gaa	gtc	acc	1	G	A
TCGA-04-1542	ITGBL1	ENST00000376180	p.K114K	c.342G>A	Verified	GGC	AAG	TGC	3	G	A
TCGA-24-1843	ITIH5L	ENST00000218436	p.T401T	c.1203G>A	Unverified	ccc	acg	gcc	3	G	A
TCGA-24-1103	ITK	ENST00000422843	p.P79P	c.237G>A	Verified	TAC	CCG	TTT	3	G	A
TCGA-13-0887	ITPKB	NM_002221	p.A306T	c.916G>A	Verified	gag	gcc	ccc	1	G	A
TCGA-24-1604	ITPR1	ENST00000456211	p.G2303S	c.6907G>A	Unverified	CTG	GGC	GCT	1	G	A
TCGA-29-1781	ITPR3	ENST00000374316	p.E410K	c.1228G>A	Unverified	GAG	GAG	CGG	1	G	A
TCGA-29-1781	ITPR3_ENST	ENST00000374316	p.E410K	c.1228G>A	Unverified	GAG	GAG	CGG	1	G	A
TCGA-13-1481	ITSN2	ENST00000355123	p.A1410A	c.4230G>A	Unverified	CAG	GCG	CAC	3	G	A
TCGA-24-2267	IVL	ENST00000368764	p.P176P	c.528G>A	Verified	CAC	CCG	GAG	3	G	A
TCGA-61-1904	IZUMO1	ENST00000332955	p.R232H	c.695G>A	Unverified	TAC	CGC	TGC	2	G	A
TCGA-20-0991	JAG1	ENST00000254958	p.A959A	c.2877G>A	Verified	TGT	GCG	AAC	3	G	A
TCGA-13-0919	JAGN1	ENST00000307768	p.P77P	c.231G>A	Verified	TAC	CCG	TAT	3	G	A

TCGA-20-0991	JAKMIP1	ENST00000282924	p.E296K	c.886G>A	Verified	gct	gaa	ctg	1	G	A
TCGA-23-1124	JHDM1D	ENST00000397560	p.D686N	c.2056G>A	Verified	GGT	GAT	GAA	1	G	A
TCGA-20-1683	KALRN	ENST00000240874	p.G1549E	c.4646G>A	Unverified	TCT	GGG	CGC	2	G	A
TCGA-20-1683	KALRN_ENST	ENST00000360013	p.G1549E	c.4646G>A	Unverified	TCT	GGG	CGC	2	G	A
TCGA-13-1505	KANK1	ENST00000382293	p.G471D	c.1412G>A	Verified	ATC	GGT	TGT	2	G	A
TCGA-13-1489	KARS	ENST00000302445	p.A498A	c.1494G>A	Verified	AAT	GCG	TAT	3	G	A
TCGA-23-1029	KBTBD5	ENST00000287777	p.E222K	c.664G>A	Unverified	TTC	GAG	AGC	1	G	A
TCGA-23-1111	KBTBD6	ENST00000379485	p.R421H	c.1262G>A	Unverified	GAT	CGC	TTG	2	G	A
TCGA-13-0919	KCNA1	ENST00000382545	p.M175I	c.525G>A	Verified	GTC	ATG	GTC	3	G	A
TCGA-13-0791	KCNA3	ENST00000369769	p.A267T	c.799G>A	Verified	CCC	GCC	TCG	1	G	A
TCGA-13-0885	KCNA3	ENST00000369769	p.G468S	c.1402G>A	Verified	GCC	GGT	GTC	1	G	A
TCGA-23-1117	KCNA3	ENST00000369769	p.D293N	c.877G>A	Verified	TCC	GAT	CCC	1	G	A
TCGA-23-1117	KCNA3	ENST00000369769	p.D293N	c.877G>A	Verified	TCC	GAT	CCC	1	G	A
TCGA-23-1114	KCNA4	ENST00000328224	p.G138E	c.413G>A	Unverified	GAG	GGA	AGG	2	G	A
TCGA-09-1674	KCNA5	ENST00000252321	p.G384R	c.1150G>A	Unverified	ggt	gga	gga	1	G	A
TCGA-23-1110	KCNA5	ENST00000252321	p.M210I	c.630G>A	Unverified	gcc	atg	gag	3	G	A
TCGA-61-1899	KCNAB1	ENST00000302490	p.W142*	c.425G>A	Unverified	GGC	TGG	AGG	2	G	A
TCGA-13-1507	KCNAB1	ENST00000302490	p.M71I	c.213G>A	Verified	GGC	ATG	AAA	3	G	A
TCGA-24-1604	KCNAB1	ENST00000302490	p.L301L	c.903G>A	Verified	TCA	CTG	AAG	3	G	A
TCGA-61-1899	KCNAB1_EN	ENST00000471742	p.W149*	c.446G>A	Unverified	GGC	TGG	AGG	2	G	A
TCGA-61-1899	KCNAB1_EN	ENST00000490337	p.W160*	c.479G>A	Unverified	GGC	TGG	AGG	2	G	A
TCGA-61-1733	KCNAB2	ENST00000164247	p.R48Q	c.143G>A	Unverified	CTG	CGG	GTC	2	G	A
TCGA-61-1733	KCNAB2	ENST00000164247	p.R48Q	c.143G>A	Unverified	CTG	CGG	GTC	2	G	A
TCGA-29-1763	KCNC1	ENST00000379472	p.T410T	c.1230G>A	Unverified	CAG	ACG	TGG	3	G	A
TCGA-29-1763	KCNC1_ENST	ENST00000265969_v68	p.T410T	c.1230G>A	Unverified	CAG	ACG	TGG	3	G	A
TCGA-61-2095	KCNE4	ENST00000281830	p.E35K	c.103G>A	Unverified	AAC	GAG	TAC	1	G	A
TCGA-23-1022	KCNH1	ENST00000271751	p.P733P	c.2199G>A	Verified	CCC	CCG	GAC	3	G	A
TCGA-04-1347	KCNH2	ENST00000262186	p.D383N	c.1147G>A	Verified	GCC	GAC	GTG	1	G	A
TCGA-24-1850	KCNH5	ENST00000322893	p.V752M	c.2254G>A	Unverified	AGC	GTG	GTG	1	G	A
TCGA-29-1695	KCNH7	ENST00000332142	p.?	c.3131+1G>A	Unverified	AAC	AGG	CTT	2	G	A
TCGA-30-1718	KCNJ12	ENST00000331718	p.R43H	c.128G>A	Unverified	TGC	CGC	AAC	2	G	A
TCGA-23-1122	KCNK10	ENST00000340700	p.P428P	c.1284G>A	Verified	GGG	CCG	GAG	3	G	A
TCGA-29-1769	KCNK17	ENST00000373231	p.A89T	c.265G>A	Unverified	gga	gcc	agc	1	G	A
TCGA-29-1769	KCNK17_EN	ENST00000453413	p.A89T	c.265G>A	Unverified	GGA	GCC	AGC	1	G	A

TCGA-24-1474	KCNN4	ENST00000262888	p.E45K	c.133G>A	Verified	GCA	GAG	ATG	1	G	A
TCGA-24-1616	KCNQ5	ENST00000370398	p.V276I	c.826G>A	Verified	TTG	GTT	CTT	1	G	A
TCGA-30-1856	KCNQ5	ENST00000370398	p.A227T	c.679G>A	Unverified	TTT	GCC	ACG	1	G	A
TCGA-13-1481	KCNT2	ENST00000294725	p.?	c.1294+1G>A	Verified	GCT	GAT	CAT	1	G	A
TCGA-61-2009	KCTD14	ENST00000353172	p.V39I	c.115G>A	Verified	AAC	GTC	GGG	1	G	A
TCGA-25-2391	KCTD16	ENST00000512467	p.R191Q	c.572G>A	Unverified	CCC	CGG	ATT	2	G	A
TCGA-24-1616	KCTD7	ENST00000275532	p.R177H	c.530G>A	Verified	CTG	CGT	GCG	2	G	A
TCGA-61-1915	KDM2A	ENST00000529006	p.D1070N	c.3208G>A	Unverified	ctc	gac	ctc	1	G	A
TCGA-20-1686	KDM4C	ENST00000381309	p.S678S	c.2034G>A	Unverified	ACA	TCG	GAG	3	G	A
TCGA-20-1686	KDM4C_ENS	ENST00000381306	p.S678S	c.2034G>A	Unverified	ACA	TCG	GAG	3	G	A
TCGA-04-1331	KDM5C	ENST00000375401	p.G912E	c.2735G>A	Verified	CTG	GGG	GTG	2	G	A
TCGA-13-0900	KEL	ENST00000355265	p.D226N	c.676G>A	Verified	ATA	GAC	CAG	1	G	A
TCGA-13-1501	KHDRBS1	ENST00000327300	p.E384K	c.1150G>A	Verified	tac	gaa	ggc	1	G	A
TCGA-13-1501	KHDRBS1	ENST00000327300	p.E384K	c.1150G>A	Verified	tac	gaa	ggc	1	G	A
TCGA-13-0924	KHDRBS3	ENST00000355849	p.D285N	c.853G>A	Verified	TAT	GAT	TCC	1	G	A
TCGA-29-1781	KIAA0141	ENST00000194118	p.S453N	c.1358G>A	Unverified	TCC	AGC	CCC	2	G	A
TCGA-29-1703	KIAA0146	ENST00000297423	p.G374E	c.1121G>A	Unverified	AGT	GGA	AGT	2	G	A
TCGA-13-0893	KIAA0195	ENST00000314256	p.V373I	c.1117G>A	Verified	GCT	GTC	TCC	1	G	A
TCGA-13-1408	KIAA0226	ENST00000296343	p.?	c.2127-1G>A	Unverified	CCA	ACG	AGG	3	G	A
TCGA-13-1408	KIAA0226_E	ENST00000273582	p.?	c.1992-1G>A	Unverified	CCA	ACG	AGG	3	G	A
TCGA-61-1910	KIAA0317	ENST00000356357	p.R667Q	c.2000G>A	Unverified	TAT	CGG	CTG	2	G	A
TCGA-13-1510	KIAA0408	ENST00000483725	p.R434K	c.1301G>A	Unverified	ACA	AGG	AAT	2	G	A
TCGA-09-0369	KIAA0467_E	ENST00000372442	p.V704I	c.2110G>A	Verified	CCA	GTT	GGA	1	G	A
TCGA-09-0369	KIAA0467_E	ENST00000372442	p.V704I	c.2110G>A	Verified	CCA	GTT	GGA	1	G	A
TCGA-29-2427	KIAA0467_E	ENST00000372442	p.R995R	c.2985G>A	Verified	TTG	CGG	ACT	3	G	A
TCGA-24-2289	KIAA0746	ENST00000399878	p.A360T	c.1078G>A	Unverified	CAG	GCA	TTG	1	G	A
TCGA-24-2262	KIAA0889	ENST00000237536	p.E520K	c.1558G>A	Unverified	GTC	GAA	GAG	1	G	A
TCGA-23-1116	KIAA0913_E	ENST00000412131	p.V885M	c.2653G>A	Unverified	GAG	GTG	GCT	1	G	A
TCGA-20-1683	KIAA0930	ENST00000251993	p.K117K	c.351G>A	Unverified	CAG	AAG	CTG	3	G	A
TCGA-09-2044	KIAA0947	ENST00000296564	p.D376N	c.1126G>A	Verified	ACA	GAT	TCC	1	G	A
TCGA-23-1120	KIAA1109	ENST00000264501	p.M387I	c.1161G>A	Verified	CGA	ATG	AAT	3	G	A
TCGA-04-1343	KIAA1210	XM_172801.3	p.G389D	c.1166G>A	Verified	tat	ggc	ctg	2	G	A
TCGA-30-1855	KIAA1211	ENST00000264229	p.R1199R	c.3597G>A	Unverified	AAG	AGG	TTT	3	G	A
TCGA-13-1488	KIAA1279	ENST00000361983	p.L620L	c.1860G>A	Verified	GCC	CTG	ACT	3	G	A

TCGA-09-1665	KIAA1324L	ENST00000297222	p.R137Q	c.410G>A	Verified	TGC	CGG	GAG	2	G	A
TCGA-23-1031	KIAA1324L	ENST00000297222	p.S478N	c.1433G>A	Verified	ATC	AGT	TTA	2	G	A
TCGA-24-1845	KIAA1370	ENST00000261844	p.G303D	c.908G>A	Unverified	CCA	GGC	ACT	2	G	A
TCGA-13-1496	KIAA1377	ENST00000263468	p.R120H	c.359G>A	Verified	CAG	CGT	GCC	2	G	A
TCGA-24-1850	KIAA1462	ENST00000375377	p.E239K	c.715G>A	Unverified	CCC	GAG	AGC	1	G	A
TCGA-23-1123	KIAA1462	ENST00000375377	p.P1101P	c.3303G>A	Verified	CTG	CCG	GGC	3	G	A
TCGA-23-1123	KIAA1462	ENST00000375377	p.P1101P	c.3303G>A	Verified	CTG	CCG	GGC	3	G	A
TCGA-61-2109	KIAA1509	ENST00000331194	p.R93Q	c.278G>A	Unverified	TCG	CGG	CCA	2	G	A
TCGA-25-1326	KIAA1755	ENST00000279024	p.E113K	c.337G>A	Verified	CAG	GAG	GAG	1	G	A
TCGA-23-1122	KIAA1797	ENST00000380249	p.G1369D	c.4106G>A	Verified	ATT	GGC	TTC	2	G	A
TCGA-24-1850	KIAA2018	ENST00000316407	p.R1991H	c.5972G>A	Unverified	ATT	CGT	CAG	2	G	A
TCGA-61-2095	KIAA2018	ENST00000316407	p.A1258A	c.3774G>A	Unverified	TGT	GCG	GGT	3	G	A
TCGA-24-1464	KIAA2022	ENST00000373468	p.G22G	c.66G>A	Verified	aat	ggg	gtc	3	G	A
TCGA-29-1702	KIF18B	ENST00000339151	p.R509Q	c.1526G>A	Unverified	GCA	CGG	GAA	2	G	A
TCGA-20-1685	KIF19	ENST00000389916	p.V926M	c.2776G>A	Unverified	CCA	GTG	TGC	1	G	A
TCGA-13-1498	KIF21A	ENST00000361961	p.C1383Y	c.4148G>A	Verified	TAC	TGT	AAT	2	G	A
TCGA-13-1509	KIF26B	ENST00000407071	p.R2024H	c.6071G>A	Verified	CAC	CGC	CAG	2	G	A
TCGA-23-1116	KIF3C	ENST00000264712	p.K103K	c.309G>A	Unverified	GGC	AAG	ACC	3	G	A
TCGA-13-1488	KIF4B	ENST00000435029	p.R233H	c.698G>A	Verified	TTT	CGC	TCC	2	G	A
TCGA-24-1464	KIF5B	ENST00000302418	p.E75K	c.223G>A	Verified	CTT	GAA	GGA	1	G	A
TCGA-61-1899	KIFC1	ENST00000428849	p.E645K	c.1933G>A	Unverified	GAA	GAG	AAC	1	G	A
TCGA-04-1336	KIFC2	ENST00000301332	p.S133N	c.398G>A	Verified	CGA	AGC	CCC	2	G	A
TCGA-25-1313	KIFC3	ENST00000379655	p.R451H	c.1352G>A	Verified	GCT	CGT	GTC	2	G	A
TCGA-30-1855	KIR3DL1_EN	ENST00000391728	p.R166H	c.497G>A	Unverified	TCA	CGC	CTC	2	G	A
TCGA-30-1855	KIR3DL1_EN	ENST00000402254	p.R166H	c.497G>A	Unverified	TCA	CGC	CTC	2	G	A
TCGA-24-0975	KIRREL2	ENST00000360202	p.C652Y	c.1955G>A	Unverified	CCC	TGC	AGA	2	G	A
TCGA-24-2267	KIT	ENST00000288135	p.G961S	c.2881G>A	Verified	gtc	ggc	agc	1	G	A
TCGA-29-1702	KIT	ENST00000288135	p.R49H	c.146G>A	Unverified	gtc	cgc	gtg	2	G	A
TCGA-29-1762	KLB	ENST00000257408	p.C319Y	c.956G>A	Unverified	AAA	TGT	CAA	2	G	A
TCGA-23-1122	KLF11	ENST00000305883	p.C21Y	c.62G>A	Verified	ATA	TGT	GAG	2	G	A
TCGA-09-2049	KLF8	ENST00000468660	p.A61T	c.181G>A	Verified	CCA	GCA	CTG	1	G	A
TCGA-23-1032	KLHDC2	ENST00000298307	p.G162G	c.486G>A	Verified	GGA	GGG	TAT	3	G	A
TCGA-29-1775	KLHDC7A	ENST00000400664	p.V764I	c.2290G>A	Unverified	ACC	GTC	CTG	1	G	A
TCGA-23-1032	KLHL11	ENST00000319121	p.R686K	c.2057G>A	Unverified	ATC	AGA	CAG	2	G	A

TCGA-13-0885	KLHL15	ENST00000328046	p.W570*	c.1709G>A	Verified	aag	tgg	aag	2	G	A
TCGA-13-1509	KLHL26	ENST00000300976	p.R278H	c.833G>A	Verified	TGC	CGC	CAG	2	G	A
TCGA-29-1768	KLHL31	ENST00000370905	p.G365E	c.1094G>A	Unverified	GAT	GGA	TTT	2	G	A
TCGA-29-1695	KLHL6	ENST00000341319	p.V78V	c.234G>A	Unverified	TGT	GTG	GAC	3	G	A
TCGA-09-1674	KLRAQ1	ENST00000281394	p.E366K	c.1096G>A	Unverified	GAA	GAA	GAA	1	G	A
TCGA-13-0761	KNTC1	ENST00000333479	p.V515V	c.1545G>A	Verified	gag	gtg	cta	3	G	A
TCGA-13-0761	KNTC1	ENST00000333479	p.V515V	c.1545G>A	Verified	gag	gtg	cta	3	G	A
TCGA-61-1899	KPNA3	ENST00000261667	p.R218Q	c.653G>A	Unverified	ctt	cgg	aac	2	G	A
TCGA-61-2109	KPRP	ENST00000368773	p.R496H	c.1487G>A	Unverified	TGG	CGC	AGC	2	G	A
TCGA-24-1469	KRT14	ENST00000167586	p.Q348Q	c.1044G>A	Verified	TCC	CAG	CTC	3	G	A
TCGA-30-1855	KRT2	ENST00000309680	p.V174V	c.522G>A	Unverified	AAT	GTG	AAG	3	G	A
TCGA-29-1698	KRT3	ENST00000417996	p.G127G	c.381G>A	Unverified	GGA	GGG	GCT	3	G	A
TCGA-29-1766	KRT4	ENST00000293774	p.T364T	c.1092G>A	Unverified	GAC	ACG	TCC	3	G	A
TCGA-04-1331	KRT6A	ENST00000330722	p.R260H	c.779G>A	Verified	AAG	CGC	ACA	2	G	A
TCGA-61-1738	KRT76	ENST00000332411	p.G153G	c.459G>A	Unverified	CCT	GGG	GGA	3	G	A
TCGA-61-1738	KRT76	ENST00000332411	p.G153G	c.459G>A	Unverified	CCT	GGG	GGA	3	G	A
TCGA-20-0990	KRTAP26-1	ENST00000360542	p.R172H	c.515G>A	Verified	TAT	CGT	CCT	2	G	A
TCGA-09-1674	KSR2	SU_KSR2	p.P320P	c.960G>A	Unverified	ccc	ccg	ggg	3	G	A
TCGA-09-1665	KTN1	ENST00000395309	p.M554I	c.1662G>A	Unverified	TTA	ATG	GAA	3	G	A
TCGA-04-1342	L1CAM	ENST00000370060	p.W1078*	c.3234G>A	Verified	CAG	TGG	GAC	3	G	A
TCGA-04-1356	L1CAM	ENST00000370060	p.T851T	c.2553G>A	Unverified	GTG	ACG	TAC	3	G	A
TCGA-29-1775	LAD1	ENST00000391967	p.D510N	c.1528G>A	Unverified	TCT	GAC	TCC	1	G	A
TCGA-13-0885	LAMA1	ENST00000389658	p.R1490K	c.4469G>A	Verified	GAA	AGG	TGC	2	G	A
TCGA-29-1711	LAMA1	ENST00000389658	p.R729H	c.2186G>A	Unverified	TAC	CGC	GTG	2	G	A
TCGA-29-1711	LAMA1	ENST00000389658	p.R729H	c.2186G>A	Unverified	TAC	CGC	GTG	2	G	A
TCGA-13-1509	LAMA2	ENST00000421865	p.E1738K	c.5212G>A	Verified	AAG	GAA	ATT	1	G	A
TCGA-13-1497	LAMA3	ENST00000313654	p.R1579Q	c.4736G>A	Verified	gac	cgg	ctg	2	G	A
TCGA-23-1032	LAMA3	ENST00000313654	p.R200Q	c.599G>A	Verified	ggg	cgg	gag	2	G	A
TCGA-24-0979	LAMA3	ENST00000313654	p.C1465Y	c.4394G>A	Verified	caa	tgt	cac	2	G	A
TCGA-04-1638	LAMA5	ENST00000252999	p.G3685R	c.11053G>A	Unverified	CAC	GGG	GCA	1	G	A
TCGA-04-1342	LAMB2	ENST00000305544	p.E1264K	c.3790G>A	Unverified	GAG	GAG	CTG	1	G	A
TCGA-09-0369	LAMC3	ENST00000361069	p.G814R	c.2440G>A	Verified	AGC	GGG	AAC	1	G	A
TCGA-09-0369	LAMC3	ENST00000361069	p.G814R	c.2440G>A	Verified	AGC	GGG	AAC	1	G	A
TCGA-04-1338	LARP1	ENST00000377643	p.E412K	c.1234G>A	Verified	ATT	GAA	TAC	1	G	A

TCGA-24-1469	LARP4B	ENST00000316157	p.R307Q	c.920G>A	Verified	GCA	CGG	ATA	2	G	A
TCGA-25-2392	LARP7	ENST00000324052	p.G420R	c.1258G>A	Verified	AGT	GGA	GTA	1	G	A
TCGA-20-0991	LARP7	ENST00000324052	p.R435R	c.1305G>A	Verified	AAC	AGG	GAA	3	G	A
TCGA-09-0369	LASS3	ENST00000284382	p.D239N	c.715G>A	Verified	CAC	GAT	GTG	1	G	A
TCGA-09-0369	LASS3	ENST00000284382	p.D239N	c.715G>A	Verified	CAC	GAT	GTG	1	G	A
TCGA-13-0760	LCE3E	ENST00000368789	p.G76D	c.227G>A	Verified	AGT	GGT	CAG	2	G	A
TCGA-24-1422	LCTL	ENST00000341509	p.D305N	c.913G>A	Verified	AAG	GAC	TAC	1	G	A
TCGA-20-0991	LDB3	ENST00000361373	p.R533K	c.1598G>A	Unverified	GCC	AGG	GGG	2	G	A
TCGA-29-1698	LDB3_ENST(	ENST00000372056	p.E254K	c.760G>A	Unverified	ATC	GAG	GTG	1	G	A
TCGA-29-1698	LDB3_ENST(	ENST00000429277	p.E254K	c.760G>A	Unverified	ATC	GAG	GTG	1	G	A
TCGA-04-1342	LEPRE1	ENST00000236040	p.D623N	c.1867G>A	Verified	TTC	GAT	GGC	1	G	A
TCGA-61-1900	LEPREL4	ENST00000355468	p.G218R	c.652G>A	Unverified	AGC	GGG	GAT	1	G	A
TCGA-24-1845	LHB	ENST00000221421	p.P93P	c.279G>A	Unverified	TGC	CCG	CGT	3	G	A
TCGA-24-1435	LHCGR	ENST00000294954	p.D405N	c.1213G>A	Verified	GCA	GAC	TTT	1	G	A
TCGA-29-1770	LHFPL3	ENST00000535008	p.W60*	c.180G>A	Unverified	TAC	TGG	ATA	3	G	A
TCGA-23-1031	LHFPL4	ENST00000287585	p.T163T	c.489G>A	Unverified	AAG	ACG	GGG	3	G	A
TCGA-10-0930	LILRB4	ENST00000391736	p.A173T	c.517G>A	Verified	GGA	GCT	CAG	1	G	A
TCGA-10-0930	LILRB4	ENST00000391736	p.A173T	c.517G>A	Verified	GGA	GCT	CAG	1	G	A
TCGA-61-1899	LILRB4	ENST00000391736	p.V333M	c.997G>A	Unverified	GCC	GTG	AAG	1	G	A
TCGA-20-0991	LIPG	ENST00000261292	p.A83T	c.247G>A	Verified	ACA	GCT	AAA	1	G	A
TCGA-09-2051	LIPI	ENST00000344577	p.G253E	c.758G>A	Unverified	GGA	GGA	AAT	2	G	A
TCGA-24-1417	LIX1	ENST00000274382	p.R186Q	c.557G>A	Verified	TCC	CGA	CAG	2	G	A
TCGA-09-2051	LL0XNC01-2	ENST00000218249	p.K188K	c.564G>A	Unverified	agc	aag	gta	3	G	A
TCGA-23-1032	LMF1	ENST00000262301	p.E274K	c.820G>A	Unverified	ATC	GAG	CTC	1	G	A
TCGA-61-1895	LMO7	ENST00000341547	p.R522H	c.1565G>A	Unverified	GAC	CGT	GTA	2	G	A
TCGA-61-1895	LMO7_ENST	ENST00000357063	p.R856H	c.2567G>A	Unverified	GAC	CGT	GTA	2	G	A
TCGA-61-1895	LMO7_ENST	ENST00000465261	p.R571H	c.1712G>A	Unverified	GAC	CGT	GTA	2	G	A
TCGA-13-1509	LMOD1	ENST00000367288	p.E311K	c.931G>A	Verified	GAG	GAG	GAG	1	G	A
TCGA-24-2035	LMOD3	ENST00000420581	p.A560A	c.1680G>A	Verified	CTG	GCG	TAA	3	G	A
TCGA-23-1114	LOC223075	ENST00000319386	p.Q63Q	c.189G>A	Unverified	ATT	CAG	CAG	3	G	A
TCGA-20-1687	LOC340578	ENST00000360028	p.S390N	c.1169G>A	Unverified	TCC	AGC	CTG	2	G	A
TCGA-29-1696	LOC340578	ENST00000360028	p.R337H	c.1010G>A	Unverified	CAG	CGC	CAG	2	G	A
TCGA-29-1696	LOC340578	ENST00000360028	p.R337H	c.1010G>A	Unverified	CAG	CGC	CAG	2	G	A
TCGA-23-1117	LOC51058	ENST00000397034	p.E46K	c.136G>A	Verified	CCT	GAG	GAA	1	G	A

TCGA-23-1117	LOC51058	ENST00000397034	p.E46K	c.136G>A	Verified	CCT	GAG	GAA	1	G	A
TCGA-13-1408	LOC645864	ENST00000329538	p.V297I	c.889G>A	Verified	ACC	GTT	GCC	1	G	A
TCGA-61-1913	LOC645864	ENST00000329538	p.R268H	c.803G>A	Unverified	CCA	CGC	TAC	2	G	A
TCGA-29-1705	LOC65121	ENST00000332296	p.P464P	c.1392G>A	Unverified	TCA	CCG	TCT	3	G	A
TCGA-24-1844	LOC652153	ENST00000316490	p.A3730T	c.11188G>A	Unverified	CCT	GCT	CAC	1	G	A
TCGA-61-1914	LOC652153	ENST00000316490	p.E3082K	c.9244G>A	Unverified	ATT	GAG	CAC	1	G	A
TCGA-13-0762	LOC652153	ENST00000316490	p.G3848E	c.11543G>A	Unverified	TCG	GGA	ATC	2	G	A
TCGA-13-0762	LOC652153	ENST00000316490	p.G3848E	c.11543G>A	Unverified	TCG	GGA	ATC	2	G	A
TCGA-61-1738	LOC652153	ENST00000316490	p.R3916H	c.11747G>A	Unverified	CAG	CGC	AAC	2	G	A
TCGA-61-1738	LOC652153	ENST00000316490	p.R3916H	c.11747G>A	Unverified	CAG	CGC	AAC	2	G	A
TCGA-25-1318	LOC652153	ENST00000316490	p.M994I	c.2982G>A	Unverified	AGC	ATG	GAG	3	G	A
TCGA-25-1318	LOC652153	ENST00000316490	p.M994I	c.2982G>A	Unverified	AGC	ATG	GAG	3	G	A
TCGA-13-0714	LOC728194	ENST00000404077	p.A679T	c.2035G>A	Unverified	AGC	GCG	GTC	1	G	A
TCGA-29-1690	LOC728194	ENST00000404077	p.R92H	c.275G>A	Unverified	GTT	CGT	GGG	2	G	A
TCGA-04-1343	LOXL2	ENST00000389131	p.G155S	c.463G>A	Verified	gtc	ggt	gtg	1	G	A
TCGA-30-1857	LOXL2	ENST00000389131	p.A519A	c.1557G>A	Unverified	ctg	gcg	cac	3	G	A
TCGA-04-1638	LOXL3	ENST00000264094	p.A458T	c.1372G>A	Unverified	gtg	gcc	tgt	1	G	A
TCGA-24-1423	LOXL4	ENST00000260702	p.G316R	c.946G>A	Unverified	tcc	ggg	gcc	1	G	A
TCGA-29-2427	LPA	ENST00000316300	p.R1614Q	c.4841G>A	Verified	GTC	CGG	CAG	2	G	A
TCGA-23-1022	LPAR4	ENST00000435339	p.R195H	c.584G>A	Verified	aaa	cgt	gtc	2	G	A
TCGA-36-1577	LRBA	ENST00000357115	p.R2732K	c.8195G>A	Unverified	TTG	AGG	ACC	2	G	A
TCGA-61-1904	LRCH4	ENST00000310300	p.G456S	c.1366G>A	Unverified	AAC	GGC	TCG	1	G	A
TCGA-10-0930	LRG1	ENST00000306390	p.V275M	c.823G>A	Verified	AGC	GTG	CCC	1	G	A
TCGA-10-0930	LRG1	ENST00000306390	p.V275M	c.823G>A	Verified	AGC	GTG	CCC	1	G	A
TCGA-25-2392	LRMP	ENST00000354454	p.R345K	c.1034G>A	Verified	aga	agg	tca	2	G	A
TCGA-24-1422	LRP1	ENST00000243077	p.A2633T	c.7897G>A	Verified	GAC	GCC	TCA	1	G	A
TCGA-29-1785	LRP1	ENST00000243077	p.W2348*	c.7044G>A	Unverified	TTC	TGG	ACC	3	G	A
TCGA-29-1785	LRP1	ENST00000243077	p.W2348*	c.7044G>A	Unverified	TTC	TGG	ACC	3	G	A
TCGA-36-1577	LRP10	ENST00000359591	p.G110S	c.328G>A	Unverified	GGG	GGC	AAC	1	G	A
TCGA-04-1343	LRP1B	ENST00000389484	p.G2937R	c.8809G>A	Verified	AGT	GGA	TGT	1	G	A
TCGA-24-1435	LRP1B	ENST00000389484	p.C2158Y	c.6473G>A	Verified	CTC	TGT	CTT	2	G	A
TCGA-25-2400	LRP1B	ENST00000389484	p.C3572Y	c.10715G>A	Unverified	AAA	TGT	GAT	2	G	A
TCGA-04-1331	LRP2	ENST00000263816	p.V263I	c.787G>A	Verified	GAT	GTT	CAT	1	G	A
TCGA-04-1343	LRP2	ENST00000263816	p.R3043H	c.9128G>A	Verified	GGG	CGC	TGC	2	G	A

TCGA-61-1738	LRP4	ENST00000378623	p.D1229N	c.3685G>A	Unverified	GCC	GAT	GCC	1	G	A
TCGA-61-1738	LRP4	ENST00000378623	p.D1229N	c.3685G>A	Unverified	GCC	GAT	GCC	1	G	A
TCGA-24-2262	LRPPRC	ENST00000260665	p.L771L	c.2313G>A	Verified	ATT	CTG	AAG	3	G	A
TCGA-23-1021	LRRC15	ENST00000347624	p.R98Q	c.293G>A	Verified	TTC	CGA	AAC	2	G	A
TCGA-25-2392	LRRC18	ENST00000374160	p.G112G	c.336G>A	Unverified	AAC	GGG	CTG	3	G	A
TCGA-04-1652	LRRC2	ENST00000296144	p.R235Q	c.704G>A	Unverified	CTG	CGG	ATG	2	G	A
TCGA-04-1652	LRRC2	ENST00000296144	p.R235Q	c.704G>A	Unverified	CTG	CGG	ATG	2	G	A
TCGA-10-0930	LRRC2	ENST00000296144	p.R335Q	c.1004G>A	Verified	GAA	CGG	GAT	2	G	A
TCGA-10-0930	LRRC2	ENST00000296144	p.R335Q	c.1004G>A	Verified	GAA	CGG	GAT	2	G	A
TCGA-61-2102	LRRC39	ENST00000370137	p.L65L	c.195G>A	Verified	ATT	TTG	AAG	3	G	A
TCGA-04-1331	LRRC41	ENST00000343304	p.E178K	c.532G>A	Verified	ACC	GAG	CTG	1	G	A
TCGA-20-1685	LRRC43	ENST00000425921	p.D43N	c.127G>A	Unverified	CTG	GAC	CTG	1	G	A
TCGA-29-1762	LRRC43	ENST00000425921	p.S88N	c.263G>A	Unverified	GAC	AGC	CTG	2	G	A
TCGA-20-1685	LRRC43_ENS	ENST00000339777	p.D228N	c.682G>A	Unverified	CTG	GAC	CTG	1	G	A
TCGA-29-1762	LRRC43_ENS	ENST00000339777	p.S273N	c.818G>A	Unverified	GAC	AGC	CTG	2	G	A
TCGA-61-1895	LRRC48	ENST00000448396	p.V109M	c.325G>A	Unverified	CTG	GTG	AAC	1	G	A
TCGA-61-1895	LRRC48_ENS	ENST00000411504	p.V109M	c.325G>A	Unverified	CTG	GTG	AAC	1	G	A
TCGA-30-1891	LRRC4C	ENST00000278198	p.R295R	c.885G>A	Unverified	GAG	CGG	ATA	3	G	A
TCGA-24-1845	LRRC58	ENST00000295628	p.L188L	c.564G>A	Unverified	AAT	CTG	CCT	3	G	A
TCGA-10-0930	LRRC71	ENST00000337428	p.D266N	c.796G>A	Verified	GGT	GAC	GAG	1	G	A
TCGA-10-0930	LRRC71	ENST00000337428	p.D266N	c.796G>A	Verified	GGT	GAC	GAG	1	G	A
TCGA-23-1809	LRRFIP1_EN	ENST00000308482	p.R625H	c.1874G>A	Unverified	AAG	CGT	CTG	2	G	A
TCGA-23-1029	LRRK1	SU_LRRK1	p.E1446E	c.4338G>A	Unverified	cca	gag	atc	3	G	A
TCGA-23-1029	LRRK1_ENST	ENST00000388948	p.E1434E	c.4302G>A	Unverified	CCA	GAG	ATC	3	G	A
TCGA-29-1701	LRRK2	SU_LRRK2	p.R1948H	c.5843G>A	Unverified	att	cgt	ccc	2	G	A
TCGA-61-1904	LRRK2	SU_LRRK2	p.K1959K	c.5877G>A	Unverified	tcc	aag	ggt	3	G	A
TCGA-29-1701	LRRK2_ENST	ENST00000298910	p.R1941H	c.5822G>A	Unverified	ATT	CGT	CCC	2	G	A
TCGA-61-1904	LRRK2_ENST	ENST00000298910	p.K1952K	c.5856G>A	Unverified	TCC	AAG	GGT	3	G	A
TCGA-29-1761	LRRN3	ENST00000308478	p.M283I	c.849G>A	Unverified	AAT	ATG	CTA	3	G	A
TCGA-29-1781	LRRTM2	ENST00000274711	p.W238*	c.714G>A	Unverified	CAA	TGG	AAC	3	G	A
TCGA-30-1891	LTB4R2	ENST00000336557	p.L44L	c.132G>A	Unverified	ACA	CTG	CTG	3	G	A
TCGA-23-1022	LTBP1	NM_206943.1	p.D1245N	c.3733G>A	Verified	gaa	gat	att	1	G	A
TCGA-25-2401	LTBP1	NM_206943.1	p.G1556E	c.4667G>A	Verified	tat	gga	gag	2	G	A
TCGA-61-1907	LTBP2	ENST00000261978	p.G1106R	c.3316G>A	Unverified	TCC	GGA	GTC	1	G	A

TCGA-29-1770	LTBP4_ENST	ENST00000308370	p.E1255K	c.3763G>A	Unverified	GAC	GAA	TGT	1	G	A
TCGA-61-1900	LTN1	ENST00000361371	p.A834T	c.2500G>A	Unverified	TCA	GCG	AAA	1	G	A
TCGA-20-1686	LTN1	ENST00000361371	p.L1652L	c.4956G>A	Unverified	CAA	CTG	CCT	3	G	A
TCGA-61-1740	LUZP1	ENST00000374623	p.A858A	c.2574G>A	Unverified	GAA	GCG	GAG	3	G	A
TCGA-20-0991	MACC1	ENST00000332878	p.V696I	c.2086G>A	Verified	TAT	GTT	ATA	1	G	A
TCGA-61-2094	MACF1	ENST00000360115	p.Q5227Q	c.15681G>A	Unverified	CCA	CAG	CTG	3	G	A
TCGA-61-2094	MACF1_ENS	ENST00000361689	p.Q4725Q	c.14175G>A	Unverified	CCA	CAG	CTG	3	G	A
TCGA-24-1417	MADD	ENST00000311027	p.G887R	c.2659G>A	Verified	TTT	GGG	CTA	1	G	A
TCGA-61-1913	MAGEB17_E	ENST00000400004	p.R268H	c.803G>A	Unverified	CCA	CGC	TAC	2	G	A
TCGA-20-0990	MAGEB6	ENST00000379034	p.R46H	c.137G>A	Verified	tct	cgc	gct	2	G	A
TCGA-24-1847	MAGEC1	ENST00000285879	p.K1092K	c.3276G>A	Unverified	cta	aag	aat	3	G	A
TCGA-09-2044	MAGED1	ENST00000375695	p.Q341Q	c.1023G>A	Verified	act	cag	aac	3	G	A
TCGA-25-2398	MAGI2	ENST00000354212	p.R564Q	c.1691G>A	Verified	GAT	CGG	CCG	2	G	A
TCGA-61-1900	MAGI2	ENST00000354212	p.R1099R	c.3297G>A	Unverified	TAC	AGG	CAA	3	G	A
TCGA-13-0885	MAML1	ENST00000292599	p.R190H	c.569G>A	Verified	aag	cgt	ctg	2	G	A
TCGA-24-2267	MAP1A	ENST00000300231	p.R178H	c.533G>A	Unverified	TAT	CGT	GTG	2	G	A
TCGA-29-1695	MAP1S	ENST00000324096	p.P221P	c.663G>A	Unverified	CCA	CCG	TCC	3	G	A
TCGA-29-1699	MAP2	ENST00000360351	p.G74E	c.221G>A	Unverified	AAT	GGG	ATC	2	G	A
TCGA-13-1510	MAP2	ENST00000360351	p.E78E	c.234G>A	Verified	GGA	GAG	CTG	3	G	A
TCGA-29-1699	MAP2_ENST	ENST00000199940	p.G74E	c.221G>A	Unverified	AAT	GGG	ATC	2	G	A
TCGA-20-1686	MAP3K12	ENST00000267079	p.E39K	c.115G>A	Unverified	CCC	GAG	AAG	1	G	A
TCGA-20-1686	MAP3K12_E	ENST00000547035	p.E39K	c.115G>A	Unverified	CCC	GAG	AAG	1	G	A
TCGA-29-1761	MAP4K1	NM_007181	p.E751K	c.2251G>A	Unverified	acc	gaa	gcg	1	G	A
TCGA-29-1761	MAP4K1_EN	ENST00000396857	p.E751K	c.2251G>A	Unverified	ACC	GAA	GCG	1	G	A
TCGA-04-1331	MAP4K3	ENST00000263881	p.D196N	c.586G>A	Verified	tgt	gat	ctc	1	G	A
TCGA-13-0905	MAP7D1	ENST00000373151	p.T302T	c.906G>A	Verified	ATG	ACG	CCC	3	G	A
TCGA-61-1904	MAPK8IP3	NM_015133.2	p.S1138N	c.3413G>A	Unverified	gtc	agc	aag	2	G	A
TCGA-61-1904	MAPK8IP3_I	ENST00000250894	p.S1132N	c.3395G>A	Unverified	GTC	AGC	AAG	2	G	A
TCGA-29-1711	MARCH1_EN	ENST00000503008	p.?	c.242+1G>A	Unverified	TGC	AGA	ATC	2	G	A
TCGA-29-1711	MARCH1_EN	ENST00000503008	p.?	c.242+1G>A	Unverified	TGC	AGA	ATC	2	G	A
TCGA-13-0714	MARK3	ENST00000216288	p.E120K	c.358G>A	Unverified	TTC	GAA	GTC	1	G	A
TCGA-09-1665	MARK4	ENST00000300843	p.R410Q	c.1229G>A	Unverified	CAG	CGG	AGT	2	G	A
TCGA-29-1691	MARVELD3	ENST00000299952	p.R380H	c.1139G>A	Unverified	CTG	CGT	AGC	2	G	A
TCGA-30-1857	MAST2_ENS	ENST00000361297	p.R1773K	c.5318G>A	Unverified	CTC	AGG	AGG	2	G	A

TCGA-61-2102	MAST3	SU_MAST3	p.A895T	c.2683G>A	Verified	cca	gcc	cct	1	G	A
TCGA-04-1542	MBD1	ENST00000382948	p.R404H	c.1211G>A	Verified	tac	cgt	cgt	2	G	A
TCGA-30-1855	MBD1	ENST00000382948	p.Q103Q	c.309G>A	Unverified	ccc	cag	agt	3	G	A
TCGA-30-1855	MBD1_ENST	ENST00000269471	p.Q103Q	c.309G>A	Unverified	CCC	CAG	AGT	3	G	A
TCGA-30-1855	MBD1_ENST	ENST00000339998	p.Q103Q	c.309G>A	Unverified	CCC	CAG	AGT	3	G	A
TCGA-30-1855	MBD1_ENST	ENST00000457839	p.Q103Q	c.309G>A	Unverified	CCC	CAG	AGT	3	G	A
TCGA-61-1910	MBD5	ENST00000407073	p.L1434L	c.4302G>A	Unverified	GGT	TTG	GAA	3	G	A
TCGA-29-1699	MBTPS1	ENST00000343411	p.R399Q	c.1196G>A	Unverified	GTG	CGG	GGT	2	G	A
TCGA-25-2391	MC3R	NM_019888.2	p.V255I	c.763G>A	Unverified	cac	gtc	aag	1	G	A
TCGA-29-1711	MCF2L_ENS	ENST00000375601	p.C680Y	c.2039G>A	Unverified	GAC	TGC	CCA	2	G	A
TCGA-29-1711	MCF2L_ENS	ENST00000375601	p.C680Y	c.2039G>A	Unverified	GAC	TGC	CCA	2	G	A
TCGA-29-1711	MCF2L_ENS	ENST00000375604	p.C733Y	c.2198G>A	Unverified	GAC	TGC	CCA	2	G	A
TCGA-29-1711	MCF2L_ENS	ENST00000375604	p.C733Y	c.2198G>A	Unverified	GAC	TGC	CCA	2	G	A
TCGA-29-1711	MCF2L_ENS	ENST00000375608	p.C706Y	c.2117G>A	Unverified	GAC	TGC	CCA	2	G	A
TCGA-29-1711	MCF2L_ENS	ENST00000375608	p.C706Y	c.2117G>A	Unverified	GAC	TGC	CCA	2	G	A
TCGA-61-2113	MCM3AP	ENST00000397708	p.E1284K	c.3850G>A	Unverified	gca	gag	tgc	1	G	A
TCGA-25-2393	MCM4	ENST00000523944	p.D511N	c.1531G>A	Verified	ggc	gac	cct	1	G	A
TCGA-13-0793	MDGA2	ENST00000357362	p.R156Q	c.467G>A	Verified	GAG	CGG	ATG	2	G	A
TCGA-29-1703	MDH1B	ENST00000374412	p.R72H	c.215G>A	Unverified	GAT	CGT	GGA	2	G	A
TCGA-09-2049	MDM4	ENST00000367182	p.E285E	c.855G>A	Verified	CTG	GAG	GAC	3	G	A
TCGA-24-2290	MED23	ENST00000368068	p.V1360M	c.4078G>A	Verified	CAG	GTG	CCC	1	G	A
TCGA-24-2290	MED23	ENST00000368068	p.V1360M	c.4078G>A	Verified	CAG	GTG	CCC	1	G	A
TCGA-04-1361	MEFV	ENST00000219596	p.R348H	c.1043G>A	Verified	AGC	CGC	TCA	2	G	A
TCGA-04-1361	MEFV	ENST00000219596	p.R348H	c.1043G>A	Verified	AGC	CGC	TCA	2	G	A
TCGA-23-1110	MEGF8	ENST00000251268_v61	p.V218V	c.654G>A	Verified	AAC	GTG	AGT	3	G	A
TCGA-13-0884	MEIS2	ENST00000561208	p.D78N	c.232G>A	Verified	AAG	GAC	GCG	1	G	A
TCGA-13-0906	MEIS2	ENST00000561208	p.V113I	c.337G>A	Verified	GAC	GTC	TGC	1	G	A
TCGA-13-0761	MET	ENST00000318493	p.D1398N	c.4192G>A	Verified	GTG	GAC	ACA	1	G	A
TCGA-13-0761	MET	ENST00000318493	p.D1398N	c.4192G>A	Verified	GTG	GAC	ACA	1	G	A
TCGA-24-1469	METTL17	ENST00000339374	p.K362K	c.1086G>A	Verified	AAC	AAG	AAA	3	G	A
TCGA-29-1705	MFF	ENST00000353339	p.R149K	c.446G>A	Unverified	GGC	AGA	CTA	2	G	A
TCGA-24-2024	MFSD8	ENST00000296468	p.A159T	c.475G>A	Verified	GGT	GCT	ACT	1	G	A
TCGA-25-2400	MGA	XM_031689.7	p.R2484Q	c.7451G>A	Verified	cgg	cgg	cgt	2	G	A
TCGA-24-1604	MGAM	ENST00000389322	p.V263M	c.787G>A	Verified	AAC	GTG	TAT	1	G	A

TCGA-24-1604	MGAM_ENS	ENST00000549489	p.V263M	c.787G>A	Verified	AAC	GTG	TAT	1	G	A
TCGA-13-0904	MGAT5B	ENST00000301618	p.R68H	c.203G>A	Verified	TCC	CGC	GGC	2	G	A
TCGA-13-1498	MGC33530	ENST00000402026	p.E160K	c.478G>A	Verified	TTC	GAA	GCC	1	G	A
TCGA-61-1914	MGEA5	ENST00000361464	p.R306Q	c.917G>A	Unverified	CCA	CGG	TTA	2	G	A
TCGA-29-1691	MIOX	ENST00000216075	p.G105D	c.314G>A	Unverified	GAG	GGC	ATC	2	G	A
TCGA-24-1844	MIPOL1	ENST00000327441	p.M202I	c.606G>A	Unverified	CAT	ATG	GAA	3	G	A
TCGA-09-1674	MITD1	ENST00000289359	p.G5R	c.13G>A	Unverified	TCC	GGG	CTG	1	G	A
TCGA-23-1111	MKI67	ENST00000368654	p.D1868N	c.5602G>A	Unverified	GCG	GAC	ACC	1	G	A
TCGA-29-1761	MKS1	ENST00000393119	p.G255R	c.763G>A	Unverified	GAG	GGG	GAG	1	G	A
TCGA-29-1761	MKS1_ENST	ENST00000337050	p.G255R	c.763G>A	Unverified	GAG	GGG	GAG	1	G	A
TCGA-61-1899	MLL3	ENST00000262189	p.G315S	c.943G>A	Unverified	GCC	GGC	ACC	1	G	A
TCGA-61-1899	MLL3_ENST(	ENST00000355193	p.G315S	c.943G>A	Unverified	GCC	GGC	ACC	1	G	A
TCGA-25-2401	MLL4	ENST00000222270	p.V1428V	c.4284G>A	Unverified	GTG	GTG	GGC	3	G	A
TCGA-61-1907	MLLT10_EN:	ENST00000307729	p.L950L	c.2850G>A	Unverified	ACA	CTG	ACT	3	G	A
TCGA-61-1907	MLLT10_EN:	ENST00000377072_v68	p.L966L	c.2898G>A	Unverified	ACA	CTG	ACT	3	G	A
TCGA-20-0990	MMP13	ENST00000260302	p.D30N	c.88G>A	Verified	gat	gat	ttg	1	G	A
TCGA-23-1123	MMP13	ENST00000260302	p.V462I	c.1384G>A	Verified	cgc	gtc	atg	1	G	A
TCGA-23-1123	MMP13	ENST00000260302	p.V462I	c.1384G>A	Verified	cgc	gtc	atg	1	G	A
TCGA-30-1856	MMP24	ENST00000246186	p.R149H	c.446G>A	Unverified	AGC	CGT	AGG	2	G	A
TCGA-24-0975	MMP27	ENST00000260229	p.G205E	c.614G>A	Verified	GAT	GGA	GCA	2	G	A
TCGA-04-1347	MMP8	ENST00000236826	p.E38K	c.112G>A	Verified	ctg	gaa	aag	1	G	A
TCGA-29-1784	MMP9	ENST00000372330	p.E237E	c.711G>A	Unverified	TTC	GAG	GGC	3	G	A
TCGA-23-1032	MN1	ENST00000302326	p.P1096P	c.3288G>A	Unverified	GGA	CCG	AAG	3	G	A
TCGA-23-1022	MNDA	ENST00000368141	p.M404I	c.1212G>A	Verified	CCA	ATG	AAT	3	G	A
TCGA-04-1361	MOGAT2	ENST00000198801	p.A194T	c.580G>A	Verified	gat	gcc	agg	1	G	A
TCGA-04-1361	MOGAT2	ENST00000198801	p.A194T	c.580G>A	Verified	gat	gcc	agg	1	G	A
TCGA-61-1895	MORC2_ENS	ENST00000397641	p.R55Q	c.164G>A	Unverified	AGA	CGA	GAG	2	G	A
TCGA-04-1530	MORN1	ENST00000378531	p.V131M	c.391G>A	Verified	CAA	GTG	TAC	1	G	A
TCGA-23-1124	MOSPD1	ENST00000370783	p.D30N	c.88G>A	Verified	gat	gat	cag	1	G	A
TCGA-13-1489	MOV10	ENST00000357443	p.S224S	c.672G>A	Verified	GAG	TCG	GGT	3	G	A
TCGA-61-1914	MPP2	ENST00000269095	p.L162L	c.486G>A	Unverified	GAG	CTG	GTG	3	G	A
TCGA-13-0923	MPZ	ENST00000360451	p.G165R	c.493G>A	Verified	TAC	GGG	GTC	1	G	A
TCGA-13-0923	MRC1L1	ENST00000331429	p.G76R	c.226G>A	Verified	CTG	GGA	GTG	1	G	A
TCGA-23-1809	MRGPRD	ENST00000309106	p.V274I	c.820G>A	Unverified	CCC	GTC	ATC	1	G	A

TCGA-61-2012	MRPS15	ENST00000373116	p.R171H	c.512G>A	Verified	CTC	CGT	AAC	2	G	A
TCGA-25-2391	MRPS18B	ENST00000259873	p.R149Q	c.446G>A	Unverified	AAG	CGG	TTG	2	G	A
TCGA-09-1674	MRTO4	ENST00000330263	p.V112M	c.334G>A	Unverified	GAG	GTG	AAT	1	G	A
TCGA-23-1116	MRTO4	ENST00000330263	p.E185K	c.553G>A	Unverified	CCA	GAG	CAG	1	G	A
TCGA-23-1117	MRVI1	ENST00000436272	p.A153A	c.459G>A	Verified	CTG	GCG	CTG	3	G	A
TCGA-23-1117	MRVI1	ENST00000436272	p.A153A	c.459G>A	Verified	CTG	GCG	CTG	3	G	A
TCGA-29-1761	MS4A4E	ENST00000427611	p.A70A	c.210G>A	Unverified	GTT	GCG	TAC	3	G	A
TCGA-25-1313	MS4A5	ENST00000300190	p.A128T	c.382G>A	Verified	GGA	GCA	ATA	1	G	A
TCGA-13-1510	MS4A6A	ENST00000323961	p.G99S	c.295G>A	Unverified	TCT	GGC	TCT	1	G	A
TCGA-24-1469	MSGN1	ENST00000281047	p.A87T	c.259G>A	Verified	GGG	GCC	AGT	1	G	A
TCGA-23-1117	MSH2	ENST00000233146	p.Q409Q	c.1227G>A	Verified	tat	cag	ggt	3	G	A
TCGA-23-1117	MSH2	ENST00000233146	p.Q409Q	c.1227G>A	Verified	tat	cag	ggt	3	G	A
TCGA-13-1498	MSR1	ENST00000262101	p.?	c.1223-1G>A	Verified	CAA	GGT	ACT	2	G	A
TCGA-13-0884	MST4	ENST00000394334	p.E382K	c.1144G>A	Verified	ctc	gag	aaa	1	G	A
TCGA-13-0791	MTF1	ENST00000373036	p.G255R	c.763G>A	Verified	ACA	GGG	GAA	1	G	A
TCGA-61-1998	MTFMT	ENST00000220058	p.D183N	c.547G>A	Unverified	TTT	GAT	GTA	1	G	A
TCGA-24-1845	MTMR4	ENST00000323456	p.R526Q	c.1577G>A	Unverified	AAG	CGG	ACC	2	G	A
TCGA-04-1347	MTMR6	ENST00000381801	p.G301S	c.901G>A	Verified	TCC	GGT	TTG	1	G	A
TCGA-04-1338	MTOR	ENST00000361445	p.E875K	c.2623G>A	Unverified	act	gag	cag	1	G	A
TCGA-61-1733	MTOR	ENST00000361445	p.R281H	c.842G>A	Unverified	gag	cgt	ctg	2	G	A
TCGA-61-1733	MTOR	ENST00000361445	p.R281H	c.842G>A	Unverified	gag	cgt	ctg	2	G	A
TCGA-29-1785	MTPAP	ENST00000263063	p.L169L	c.507G>A	Unverified	CAG	TTG	CCA	3	G	A
TCGA-29-1785	MTPAP	ENST00000263063	p.L169L	c.507G>A	Unverified	CAG	TTG	CCA	3	G	A
TCGA-29-1785	MTPAP_ENS	ENST00000358107	p.L299L	c.897G>A	Unverified	CAG	TTG	CCA	3	G	A
TCGA-29-1785	MTPAP_ENS	ENST00000358107	p.L299L	c.897G>A	Unverified	CAG	TTG	CCA	3	G	A
TCGA-10-0930	MTUS2_ENS	ENST00000431530	p.A951T	c.2851G>A	Verified	GAT	GCT	CAG	1	G	A
TCGA-10-0930	MTUS2_ENS	ENST00000431530	p.A951T	c.2851G>A	Verified	GAT	GCT	CAG	1	G	A
TCGA-23-2077	MTX2	ENST00000249442	p.V89I	c.265G>A	Verified	ATA	GTC	CAA	1	G	A
TCGA-13-0913	MUC16	ENST00000397910	p.E2683K	c.8047G>A	Verified	CCA	GAG	ACT	1	G	A
TCGA-24-2288	MUC16	ENST00000397910	p.E3142K	c.9424G>A	Unverified	CCA	GAA	ACA	1	G	A
TCGA-04-1367	MUC17	ENST00000306151	p.V2859M	c.8575G>A	Verified	GTC	GTG	CCA	1	G	A
TCGA-13-1481	MUC17	ENST00000306151	p.G5G	c.15G>A	Verified	CCA	GGG	ACC	3	G	A
TCGA-13-0904	MUC17	ENST00000306151	p.V2546V	c.7638G>A	Verified	CCT	GTG	GTC	3	G	A
TCGA-25-2398	MUC21	ENST00000376296	p.E161K	c.481G>A	Unverified	AGT	GAG	GCC	1	G	A

TCGA-25-2398	MUC21	ENST00000376296	p.S103N	c.308G>A	Verified	ATC	AGC	ATA	2	G	A
TCGA-25-2398	MUC21	ENST00000376296	p.K2K	c.6G>A	Unverified	ATG	AAG	ATG	3	G	A
TCGA-24-1846	MUC4	ENST00000346145	p.G86R	c.256G>A	Unverified	TAT	GGG	GCA	1	G	A
TCGA-24-1846	MUC4_ENST	ENST00000405167	p.G4194R	c.12580G>A	Unverified	TAT	GGG	GCA	1	G	A
TCGA-24-1846	MUC4_ENST	ENST00000463781	p.G4322R	c.12964G>A	Unverified	TAT	GGG	GCA	1	G	A
TCGA-29-1691	MUC5AC	ENST00000349637	p.A2513T	c.7537G>A	Unverified	AAA	GCC	ACT	1	G	A
TCGA-29-1691	MUC5B	ENST00000529681	p.A2510T	c.7528G>A	Unverified	AAA	GCC	ACT	1	G	A
TCGA-29-1702	MUC7	ENST00000304887	p.A274T	c.820G>A	Unverified	TCC	GCC	TCA	1	G	A
TCGA-24-1846	MURC	ENST00000307584	p.R272H	c.815G>A	Unverified	ATG	CGC	AGC	2	G	A
TCGA-30-1718	MUSK	ENST00000374448	p.A706T	c.2116G>A	Unverified	ATT	GCC	AGG	1	G	A
TCGA-29-1702	MVK	ENST00000228510	p.V49V	c.147G>A	Unverified	AAA	GTG	GAC	3	G	A
TCGA-13-1505	MYADM	ENST00000391768	p.E164K	c.490G>A	Verified	GGC	GAG	ATC	1	G	A
TCGA-30-1718	MYADM	ENST00000391768	p.A152T	c.454G>A	Unverified	TAC	GCC	ACC	1	G	A
TCGA-61-2008	MYBBP1A	ENST00000254718	p.G1226G	c.3678G>A	Verified	AAC	GGG	ACG	3	G	A
TCGA-24-2024	MYBPC3	ENST00000399249	p.D1149N	c.3445G>A	Verified	AGT	GAC	AGA	1	G	A
TCGA-13-0924	MYH15	ENST00000273353	p.V1447I	c.4339G>A	Verified	AAG	GTC	CGC	1	G	A
TCGA-13-1496	MYH15	ENST00000273353	p.V315I	c.943G>A	Verified	CTG	GTA	TCT	1	G	A
TCGA-09-2050	MYH2	ENST00000245503	p.R445H	c.1334G>A	Verified	GCC	CGC	ATC	2	G	A
TCGA-24-1474	MYH2	ENST00000245503	p.R1388H	c.4163G>A	Verified	CAG	CGC	ACA	2	G	A
TCGA-13-1498	MYH3	ENST00000226209	p.V587M	c.1759G>A	Verified	ACC	GTG	GAC	1	G	A
TCGA-13-0920	MYH7	ENST00000355349	p.R1359H	c.4076G>A	Verified	CAG	CGC	GTC	2	G	A
TCGA-24-1850	MYH7	ENST00000355349	p.R237R	c.711G>A	Unverified	GTC	CGG	AAC	3	G	A
TCGA-13-1507	MYL10	ENST00000223167	p.R32Q	c.95G>A	Verified	GCT	CGG	AAA	2	G	A
TCGA-13-1496	MYL2	ENST00000228841	p.K4K	c.12G>A	Verified	CCT	AAG	AAA	3	G	A
TCGA-61-2094	MYLIP	ENST00000356840	p.E164K	c.490G>A	Unverified	TTG	GAG	GGG	1	G	A
TCGA-30-1856	MYLK2	ENST00000375994	p.A544A	c.1632G>A	Unverified	ctg	gcg	gag	3	G	A
TCGA-13-0883	MYO10	ENST00000274203	p.R1024Q	c.3071G>A	Verified	ATC	CGG	ACC	2	G	A
TCGA-24-1843	MYO16	ENST00000357550	p.D62N	c.184G>A	Unverified	ACG	GAC	ATG	1	G	A
TCGA-23-1021	MYO18A_EN	ENST00000527372	p.R466Q	c.1397G>A	Verified	TGT	CGG	CGG	2	G	A
TCGA-24-2289	MYO18A_EN	ENST00000527372	p.T349T	c.1047G>A	Unverified	GAG	ACG	GAG	3	G	A
TCGA-29-1776	MYO3A	ENST00000265944	p.E1345K	c.4033G>A	Unverified	GAG	GAA	GAA	1	G	A
TCGA-24-1846	MYO5A	ENST00000399231	p.E977K	c.2929G>A	Unverified	GAG	GAA	GCG	1	G	A
TCGA-13-0755	MYO5C	ENST00000261839	p.G68S	c.202G>A	Verified	GTG	GGC	GAG	1	G	A
TCGA-13-1498	MYO5C	ENST00000261839	p.D71N	c.211G>A	Verified	AAT	GAC	CTC	1	G	A

TCGA-04-1652	MYO6	ENST00000369977	p.R928H	c.2783G>A	Unverified	AGG	CGT	ATT	2	G	A
TCGA-04-1652	MYO6	ENST00000369977	p.R928H	c.2783G>A	Unverified	AGG	CGT	ATT	2	G	A
TCGA-13-0906	MYO9A	ENST00000356056	p.R2283H	c.6848G>A	Verified	GGG	CGT	ATT	2	G	A
TCGA-29-1770	MYO9A	ENST00000356056	p.M1965I	c.5895G>A	Unverified	TAT	ATG	AAT	3	G	A
TCGA-25-1326	MYOM1	ENST00000356443	p.G953E	c.2858G>A	Verified	CTT	GGA	TGG	2	G	A
TCGA-29-1691	MYRIP	ENST00000302541	p.D12N	c.34G>A	Unverified	ACT	GAT	GAT	1	G	A
TCGA-09-2049	MYST3	ENST00000265713	p.V585M	c.1753G>A	Verified	AAT	GTG	AGT	1	G	A
TCGA-20-1687	MYST4	ENST00000287239	p.D875N	c.2623G>A	Unverified	ATT	GAT	TTC	1	G	A
TCGA-13-0890	MYT1	ENST00000328439	p.T503T	c.1509G>A	Verified	AAC	ACG	CAC	3	G	A
TCGA-24-1845	MYT1L	ENST00000399161	p.E343K	c.1027G>A	Unverified	AGT	GAG	ACC	1	G	A
TCGA-24-1469	MZF1	ENST00000215057	p.E125E	c.375G>A	Verified	CGG	GAG	CCG	3	G	A
TCGA-61-1738	N4BP2	ENST00000261435	p.V275I	c.823G>A	Unverified	tgc	gtt	gag	1	G	A
TCGA-61-1738	N4BP2	ENST00000261435	p.V275I	c.823G>A	Unverified	tgc	gtt	gag	1	G	A
TCGA-61-1914	NAA11	ENST00000286794	p.G182D	c.545G>A	Unverified	CAG	GGC	AGC	2	G	A
TCGA-13-0887	NAA25	ENST00000261745	p.L174L	c.522G>A	Verified	TTT	CTG	CCC	3	G	A
TCGA-61-1738	NAALAD2	ENST00000534061	p.V643I	c.1927G>A	Unverified	CAA	GTT	GAT	1	G	A
TCGA-61-1738	NAALAD2	ENST00000534061	p.V643I	c.1927G>A	Unverified	CAA	GTT	GAT	1	G	A
TCGA-13-0920	NAB2	ENST00000300131	p.G232D	c.695G>A	Verified	GGG	GGT	GGT	2	G	A
TCGA-61-2113	NADK	ENST00000341991	p.R237Q	c.710G>A	Unverified	CTC	CGG	AGT	2	G	A
TCGA-04-1343	NADKD1	ENST00000397338	p.G65G	c.195G>A	Verified	GAA	GGG	ACT	3	G	A
TCGA-23-1114	NAGLU	ENST00000225927	p.A330T	c.988G>A	Unverified	GCA	GCC	ACC	1	G	A
TCGA-13-1496	NAGLU	ENST00000225927	p.P488P	c.1464G>A	Unverified	CAC	CCG	GAC	3	G	A
TCGA-29-1775	NAGPA	ENST00000312251	p.G289D	c.866G>A	Unverified	GGT	GGC	TCT	2	G	A
TCGA-04-1347	NALCN	ENST00000251127	p.R1498H	c.4493G>A	Verified	CTG	CGT	GGG	2	G	A
TCGA-04-1347	NAP1L4	ENST00000380542	p.G249R	c.745G>A	Verified	GAC	GGG	TGT	1	G	A
TCGA-30-1855	NAV2	ENST00000360655	p.G956S	c.2866G>A	Unverified	AGC	GGC	ATC	1	G	A
TCGA-29-1776	NAV2	ENST00000360655	p.R800K	c.2399G>A	Unverified	GGG	AGG	CCC	2	G	A
TCGA-23-1111	NAV3	NM_014903.3	p.V15I	c.43G>A	Unverified	gct	gtt	ggg	1	G	A
TCGA-61-1913	NAV3	NM_014903.3	p.S545N	c.1634G>A	Unverified	ggc	agc	aca	2	G	A
TCGA-29-1777	NBAS	ENST00000281513	p.G1471E	c.4412G>A	Unverified	CAA	GGG	TGT	2	G	A
TCGA-09-1674	NBAS	ENST00000281513	p.K1790K	c.5370G>A	Unverified	AAG	AAG	TTT	3	G	A
TCGA-24-1470	NBEAL2	ENST00000292309	p.A2057T	c.6169G>A	Verified	CCG	GCC	GCG	1	G	A
TCGA-23-1809	NBN	ENST00000265433	p.D272N	c.814G>A	Unverified	gtt	gat	aca	1	G	A
TCGA-23-1120	NBPF3	ENST00000318249	p.V534M	c.1600G>A	Unverified	GAC	GTG	GAT	1	G	A

TCGA-10-0930	NCAN	ENST00000252575	p.R231H	c.692G>A	Verified	GAC	CGT	AGC	2	G	A
TCGA-10-0930	NCAN	ENST00000252575	p.R231H	c.692G>A	Verified	GAC	CGT	AGC	2	G	A
TCGA-29-1781	NCAPG2	NM_017760.4	p.V355I	c.1063G>A	Unverified	ttt	gtt	gaa	1	G	A
TCGA-24-1423	NCF2	ENST00000367536	p.D316N	c.946G>A	Verified	TCC	GAC	ATC	1	G	A
TCGA-30-1855	NCKAP5	ENST00000409261	p.E463K	c.1387G>A	Unverified	AAG	GAA	CCC	1	G	A
TCGA-61-1913	NCOA1	ENST00000406961	p.L827L	c.2481G>A	Unverified	CAG	TTG	CCA	3	G	A
TCGA-61-1913	NCOA1_ENS	ENST00000405141	p.L827L	c.2481G>A	Unverified	CAG	TTG	CCA	3	G	A
TCGA-24-1844	NCOR2_ENS	ENST00000405201	p.M2337I	c.7011G>A	Unverified	AAC	ATG	GGG	3	G	A
TCGA-24-1474	NCR1	ENST00000291890	p.G167R	c.499G>A	Verified	CGC	GGA	TAC	1	G	A
TCGA-24-1422	NDC80	ENST00000261597	p.E533E	c.1599G>A	Verified	CTT	GAG	TCC	3	G	A
TCGA-29-1764	NDE1	ENST00000396354	p.V84V	c.252G>A	Unverified	GAA	GTG	CAG	3	G	A
TCGA-13-0886	NDFIP2	ENST00000218652	p.A136A	c.408G>A	Unverified	GCT	GCG	TCT	3	G	A
TCGA-29-1699	NDN	ENST00000331837	p.A74A	c.222G>A	Unverified	GCT	GCG	GAG	3	G	A
TCGA-24-1103	NDUFV2	ENST00000318388	p.K129K	c.387G>A	Verified	GGA	AAG	TAT	3	G	A
TCGA-24-1843	NEB	ENST00000409198	p.A4311T	c.12931G>A	Unverified	GCC	GCC	AAG	1	G	A
TCGA-13-0887	NEB	ENST00000409198	p.G6569E	c.19706G>A	Verified	GAC	GGG	GGT	2	G	A
TCGA-24-1843	NEB_ENST0(	ENST00000397345	p.A6012T	c.18034G>A	Unverified	GCC	GCC	AAG	1	G	A
TCGA-24-1843	NEB_ENST0(	ENST00000427231	p.A6012T	c.18034G>A	Unverified	GCC	GCC	AAG	1	G	A
TCGA-04-1649	NECAB1	ENST00000417640	p.D39N	c.115G>A	Unverified	GCA	GAC	AAA	1	G	A
TCGA-04-1542	NECAB2	ENST00000305202	p.A356A	c.1068G>A	Verified	AAG	GCG	TTC	3	G	A
TCGA-61-2109	NEFM	ENST00000221166	p.R371R	c.1113G>A	Unverified	CTT	CGG	GGC	3	G	A
TCGA-13-0890	NEK1_ENST(	ENST00000507142	p.R484H	c.1451G>A	Verified	GTT	CGT	CCA	2	G	A
TCGA-29-1777	NELL2	ENST00000429094	p.E750K	c.2248G>A	Unverified	AAT	GAG	TGC	1	G	A
TCGA-29-1777	NELL2_ENST	ENST00000437801	p.E800K	c.2398G>A	Unverified	AAT	GAG	TGC	1	G	A
TCGA-13-0885	NES	ENST00000368223	p.G1475D	c.4424G>A	Verified	AGG	GGT	GCA	2	G	A
TCGA-29-1699	NEU2	ENST00000233840	p.R152Q	c.455G>A	Unverified	TAC	CGG	GAG	2	G	A
TCGA-24-1431	NEURL4	ENST00000399464	p.R366H	c.1097G>A	Verified	ATC	CGT	ATC	2	G	A
TCGA-24-1431	NEURL4_EN	ENST00000315614	p.R366H	c.1097G>A	Verified	ATC	CGT	ATC	2	G	A
TCGA-04-1652	NEUROD6	ENST00000297142	p.E103K	c.307G>A	Unverified	CGC	GAG	AGG	1	G	A
TCGA-04-1652	NEUROD6	ENST00000297142	p.E103K	c.307G>A	Unverified	CGC	GAG	AGG	1	G	A
TCGA-23-2078	NF1	ENST00000358273	p.A1858T	c.5572G>A	Verified	ATC	GCA	TTA	1	G	A
TCGA-13-1488	NFAT5_ENS	ENST00000432919	p.G39E	c.116G>A	Verified	GCT	GGA	CTA	2	G	A
TCGA-13-0903	NFATC2	ENST00000396009	p.S107S	c.321G>A	Verified	GCC	TCG	GGC	3	G	A
TCGA-25-2042	NFKBIZ	ENST00000326172	p.R572K	c.1715G>A	Verified	CAG	AGA	AAT	2	G	A

TCGA-04-1338	NFX1	ENST00000379540	p.C520Y	c.1559G>A	Verified	CAG	TGC	CAG	2	G	A
TCGA-04-1337	NFXL1	ENST00000381538	p.R815H	c.2444G>A	Verified	GTA	CGT	GAA	2	G	A
TCGA-24-1464	NHLRC2	ENST00000369301	p.M698I	c.2094G>A	Verified	ATG	ATG	AAG	3	G	A
TCGA-61-1998	NHP2	ENST00000274606	p.D113N	c.337G>A	Unverified	ACG	GAC	CTG	1	G	A
TCGA-61-1998	NHP2	ENST00000274606	p.E134K	c.400G>A	Unverified	GAG	GAG	TAC	1	G	A
TCGA-29-1770	NHP2L1	ENST00000355257	p.V55M	c.163G>A	Unverified	atc	gtg	atg	1	G	A
TCGA-29-1690	NICN1	ENST00000273598	p.S98S	c.294G>A	Unverified	GTA	TCG	CTG	3	G	A
TCGA-24-2262	NINJ2	ENST00000305108	p.K97K	c.291G>A	Verified	CTG	AAG	GCG	3	G	A
TCGA-09-1674	NINL	ENST00000278886	p.V671V	c.2013G>A	Unverified	AGC	GTG	CTG	3	G	A
TCGA-61-1910	NIPBL	ENST00000282516	p.R2793Q	c.8378G>A	Unverified	tta	cga	tcc	2	G	A
TCGA-23-1122	NKIRAS1	ENST00000388759	p.V144I	c.430G>A	Unverified	aaa	gta	aga	1	G	A
TCGA-13-1408	NKTR	ENST00000232978	p.A1151T	c.3451G>A	Verified	AAT	GCA	CGG	1	G	A
TCGA-13-1481	NKTR	ENST00000232978	p.R430H	c.1289G>A	Verified	AAA	CGC	AGA	2	G	A
TCGA-25-2393	NLRC5	ENST00000262510	p.G1005S	c.3013G>A	Verified	CTC	GGT	CAC	1	G	A
TCGA-10-0930	NLRC5	ENST00000262510	p.L1238L	c.3714G>A	Verified	TGG	TTG	CTG	3	G	A
TCGA-10-0930	NLRC5	ENST00000262510	p.L1238L	c.3714G>A	Verified	TGG	TTG	CTG	3	G	A
TCGA-09-2056	NLRP1	ENST00000572272	p.A436A	c.1308G>A	Verified	CCG	GCG	GAT	3	G	A
TCGA-24-2290	NLRP10	ENST00000328600	p.E48K	c.142G>A	Verified	GGG	GAG	TTG	1	G	A
TCGA-24-2290	NLRP10	ENST00000328600	p.E48K	c.142G>A	Verified	GGG	GAG	TTG	1	G	A
TCGA-23-1022	NLRP12	ENST00000324134	p.E127K	c.379G>A	Verified	CAG	GAA	ACC	1	G	A
TCGA-23-1021	NLRP13	ENST00000342929	p.K686K	c.2058G>A	Verified	AAT	AAG	CTA	3	G	A
TCGA-29-1695	NLRP3	ENST00000391828	p.D282N	c.844G>A	Unverified	ccc	gac	cca	1	G	A
TCGA-29-1690	NLRP4_ENST	ENST00000301295	p.G638R	c.1912G>A	Unverified	AGC	GGG	CAC	1	G	A
TCGA-29-1764	NLRP4_ENST	ENST00000301295	p.V376I	c.1126G>A	Unverified	TTC	GTC	TTT	1	G	A
TCGA-23-1116	NLRP5	ENST00000390649	p.V407I	c.1219G>A	Unverified	ATC	GTC	ACC	1	G	A
TCGA-29-1699	NLRP8_ENST	ENST00000291971	p.R91H	c.272G>A	Unverified	GAG	CGT	TTC	2	G	A
TCGA-24-1563	NLRP9	ENST00000332836	p.S7S	c.21G>A	Verified	TTT	TCG	GAT	3	G	A
TCGA-23-1032	NM_001080	ENST00000271263	p.T79T	c.237G>A	Unverified	CAC	ACG	GGC	3	G	A
TCGA-61-1914	NME4	ENST00000219479	p.?	c.225+1G>A	Unverified	CTG	CAG	GCA	3	G	A
TCGA-25-2392	NOC3L	ENST00000371361	p.R614H	c.1841G>A	Verified	AAG	CGC	AGA	2	G	A
TCGA-29-1693	NOL11	ENST00000253247	p.E524E	c.1572G>A	Unverified	ATG	GAG	TCA	3	G	A
TCGA-23-1116	NOSIP	ENST00000391853	p.G243E	c.728G>A	Unverified	TCT	GGG	GCT	2	G	A
TCGA-04-1638	NOTCH4	ENST00000375023	p.A1175T	c.3523G>A	Unverified	gga	gcc	aag	1	G	A
TCGA-09-1674	NOTCH4	ENST00000375023	p.?	c.2438+1G>A	Unverified	gac	agc	ccc	2	G	A

TCGA-13-0903	NOTCH4	ENST00000375023	p.M252I	c.756G>A	Verified	ctg	atg	cca	3	G	A
TCGA-61-1740	NOVA2	ENST00000263257	p.L417L	c.1251G>A	Unverified	AAC	CTG	GTG	3	G	A
TCGA-61-1904	NOX5	ENST00000260364	p.V13M	c.37G>A	Unverified	TGG	GTG	ACT	1	G	A
TCGA-29-1769	NPAS2	ENST00000335681	p.L599L	c.1797G>A	Unverified	CAC	CTG	CTC	3	G	A
TCGA-25-1326	NPHP4	ENST00000378156	p.G932S	c.2794G>A	Unverified	TTG	GGC	CGG	1	G	A
TCGA-13-0792	NPHS1	ENST00000378910	p.R800H	c.2399G>A	Verified	GGG	CGC	CTG	2	G	A
TCGA-61-1899	NPHS2	ENST00000367615	p.E173E	c.519G>A	Unverified	CTG	GAG	ATA	3	G	A
TCGA-30-1718	NPNT	ENST00000379987	p.W294*	c.881G>A	Unverified	ACT	TGG	TGG	2	G	A
TCGA-09-1665	NPR1	ENST00000368680	p.G541S	c.1621G>A	Verified	tac	ggc	tcc	1	G	A
TCGA-25-2398	NPR1	ENST00000368680	p.K675K	c.2025G>A	Verified	ctc	aag	atc	3	G	A
TCGA-20-1686	NQO2	ENST00000380430	p.Q123Q	c.369G>A	Unverified	TGC	CAG	GGC	3	G	A
TCGA-24-1850	NR_003034_	ENST00000338231	p.R5R	c.15G>A	Unverified	AAC	AGG	GTG	3	G	A
TCGA-24-1845	NR1D1	ENST00000246672	p.L4L	c.12G>A	Unverified	acc	ctg	gac	3	G	A
TCGA-25-1326	NR1H3	ENST00000441012	p.R226Q	c.677G>A	Verified	AAC	CGG	CGC	2	G	A
TCGA-04-1343	NR4A2	ENST00000339562	p.R278H	c.833G>A	Verified	gtg	cgc	acc	2	G	A
TCGA-24-1422	NRCAM	ENST00000351718	p.L954L	c.2862G>A	Verified	ACT	TTG	GAA	3	G	A
TCGA-29-1764	NRCAM	ENST00000351718	p.K674K	c.2022G>A	Unverified	CAC	AAG	CCA	3	G	A
TCGA-29-1764	NRCAM_EN!	ENST00000379028	p.K690K	c.2070G>A	Unverified	CAC	AAG	CCA	3	G	A
TCGA-24-1417	NRXN1	ENST00000342183	p.K155K	c.465G>A	Unverified	GTT	AAG	TTT	3	G	A
TCGA-24-1417	NRXN1_ENS	ENST00000406316	p.K1190K	c.3570G>A	Unverified	GTT	AAG	TTT	3	G	A
TCGA-24-2267	NRXN2	ENST00000377551	p.D1081N	c.3241G>A	Verified	gcc	gac	gcc	1	G	A
TCGA-61-2113	NRXN2	ENST00000377551	p.G678G	c.2034G>A	Unverified	cag	ggg	gct	3	G	A
TCGA-30-1718	NRXN2	ENST00000377551	p.Q890Q	c.2670G>A	Unverified	ggc	cag	ccc	3	G	A
TCGA-13-1497	NSRP1	ENST00000247026	p.E441K	c.1321G>A	Verified	CGA	GAG	GTA	1	G	A
TCGA-13-0755	NT5E	ENST00000257770	p.V300I	c.898G>A	Verified	AAC	GTC	ATC	1	G	A
TCGA-09-2056	NTHL1_ENS⁻	ENST00000219066	p.L282L	c.846G>A	Unverified	CTC	TTG	GTG	3	G	A
TCGA-04-1331	NTNG1	ENST00000370074	p.M87I	c.261G>A	Verified	TAC	ATG	TGC	3	G	A
TCGA-24-1469	NTPCR	ENST00000366628	p.V185M	c.553G>A	Verified	TGC	GTG	CAG	1	G	A
TCGA-09-2050	NUMA1	ENST00000393695	p.E1275K	c.3823G>A	Verified	gca	gag	aca	1	G	A
TCGA-29-1698	NUMA1	ENST00000393695	p.A1431T	c.4291G>A	Unverified	aag	gcc	agc	1	G	A
TCGA-61-1740	NUP133	ENST00000261396	p.R476R	c.1428G>A	Unverified	tca	agg	gaa	3	G	A
TCGA-23-1029	NUP155	ENST00000231498	p.G1190G	c.3570G>A	Unverified	TAT	GGG	GAA	3	G	A
TCGA-23-1117	NUP54	ENST00000264883	p.M343I	c.1029G>A	Verified	cag	atg	act	3	G	A
TCGA-23-1117	NUP54	ENST00000264883	p.M343I	c.1029G>A	Verified	cag	atg	act	3	G	A

TCGA-04-1638	NUP98	ENST00000324932	p.G366S	c.1096G>A	Unverified	ttt	ggc	aat	1	G	A
TCGA-13-1497	NUPL2	ENST00000258742	p.G346S	c.1036G>A	Verified	ttt	ggt	agt	1	G	A
TCGA-23-1022	NXN	ENST00000336868	p.R213Q	c.638G>A	Verified	ACC	CGG	GTC	2	G	A
TCGA-24-1469	NXT1	ENST00000254998	p.E18K	c.52G>A	Verified	GCT	GAG	GAG	1	G	A
TCGA-04-1361	O5AK3_HUN	ENST00000326876	p.M258I	c.774G>A	Verified	TAC	ATG	TAC	3	G	A
TCGA-04-1361	O5AK3_HUN	ENST00000326876	p.M258I	c.774G>A	Verified	TAC	ATG	TAC	3	G	A
TCGA-13-1481	OAS1	ENST00000445409	p.E175K	c.523G>A	Verified	ATC	GAG	GAG	1	G	A
TCGA-24-0975	OBSCN	SU_OBSCN.1	p.A2858T	c.8572G>A	Unverified	gat	gcc	ggg	1	G	A
TCGA-30-1856	OBSCN	SU_OBSCN.1	p.L3510L	c.10530G>A	Unverified	ggt	ctg	agg	3	G	A
TCGA-30-1856	OBSCN_ENS	ENST00000284548	p.L3456L	c.10368G>A	Unverified	GGT	CTG	AGG	3	G	A
TCGA-24-0975	OBSCN_ENS	ENST00000359599	p.A3087T	c.9259G>A	Unverified	GAT	GCC	GGG	1	G	A
TCGA-30-1856	OBSCN_ENS	ENST00000359599	p.L3739L	c.11217G>A	Unverified	GGT	CTG	AGG	3	G	A
TCGA-30-1856	OBSCN_ENS	ENST00000570156	p.L3640L	c.10920G>A	Unverified	GGT	CTG	AGG	3	G	A
TCGA-61-1738	OBSL1_ENST	ENST00000404537	p.A693T	c.2077G>A	Unverified	GGT	GCC	CTG	1	G	A
TCGA-61-1738	OBSL1_ENST	ENST00000404537	p.A693T	c.2077G>A	Unverified	GGT	GCC	CTG	1	G	A
TCGA-24-1845	OBSL1_ENST	ENST00000404537	p.P469P	c.1407G>A	Unverified	CTG	CCG	GTC	3	G	A
TCGA-24-2024	ODF3	ENST00000325113	p.T42T	c.126G>A	Verified	CAC	ACG	CCC	3	G	A
TCGA-23-1022	ODZ4	ENST00000278550	p.G1775D	c.5324G>A	Verified	AAC	GGC	ATG	2	G	A
TCGA-29-1769	OGDH	ENST00000222673	p.G662D	c.1985G>A	Unverified	ttt	ggc	tcg	2	G	A
TCGA-13-0762	OLFML2A	ENST00000373580	p.M122I	c.366G>A	Unverified	TCC	ATG	GTG	3	G	A
TCGA-13-0762	OLFML2A	ENST00000373580	p.M122I	c.366G>A	Unverified	TCC	ATG	GTG	3	G	A
TCGA-24-1846	OPN1SW	ENST00000249389	p.E110K	c.328G>A	Unverified	TTG	GAG	GGC	1	G	A
TCGA-23-2077	OR10A7	ENST00000326258	p.V301I	c.901G>A	Verified	GCT	GTC	AAG	1	G	A
TCGA-13-0920	OR10AG1	ENST00000312345	p.A194A	c.582G>A	Unverified	GTA	GCG	GTG	3	G	A
TCGA-24-1849	OR10G9	ENST00000375024	p.L45L	c.135G>A	Unverified	CTG	CTG	GTG	3	G	A
TCGA-24-1846	OR10H4	ENST00000322107	p.S138N	c.413G>A	Unverified	ATG	AGC	CCC	2	G	A
TCGA-10-0930	OR10H4	ENST00000322107	p.T162T	c.486G>A	Verified	ACA	ACG	ATA	3	G	A
TCGA-10-0930	OR10H4	ENST00000322107	p.T162T	c.486G>A	Verified	ACA	ACG	ATA	3	G	A
TCGA-23-1118	OR10H5	ENST00000308940	p.G41D	c.122G>A	Verified	CTG	GGC	AAC	2	G	A
TCGA-24-1474	OR10J3	ENST00000332217	p.R22Q	c.65G>A	Unverified	AGG	CGG	CAG	2	G	A
TCGA-24-1850	OR10Q1	ENST00000316770	p.A280T	c.838G>A	Unverified	ATC	GCG	TTG	1	G	A
TCGA-24-1469	OR10Q1	ENST00000316770	p.R241R	c.723G>A	Verified	CGC	CGG	GCC	3	G	A
TCGA-09-2056	OR10T2	ENST00000334438	p.V84I	c.250G>A	Verified	CTG	GTC	CAC	1	G	A
TCGA-23-1021	OR11H12	ENST00000550708	p.W36*	c.107G>A	Verified	GAG	TGG	ACA	2	G	A

TCGA-61-2113	OR13F1	ENST00000334726	p.A264T	c.790G>A	Verified	TCC	GCT	GTA	1	G	A
TCGA-61-1907	OR13G1	ENST00000359688	p.E108K	c.322G>A	Unverified	GCT	GAG	ATG	1	G	A
TCGA-29-1783	OR13H1	ENST00000338616	p.V275M	c.823G>A	Unverified	tca	gtg	ttt	1	G	A
TCGA-24-1417	OR14J1	ENST00000377160	p.C314Y	c.941G>A	Unverified	ATG	TGC	TTA	2	G	A
TCGA-24-1846	OR1E1	ENST00000322608	p.G16D	c.47G>A	Unverified	CTG	GGC	CTG	2	G	A
TCGA-13-0893	OR1L6	ENST00000373684	p.G290G	c.870G>A	Unverified	TAT	GGG	AGT	3	G	A
TCGA-24-2280	OR1S2	ENST00000302592	p.V122I	c.364G>A	Unverified	GTC	GTC	ACT	1	G	A
TCGA-61-1895	OR2AG1	ENST00000307401	p.V77I	c.229G>A	Unverified	GTT	GTC	ACT	1	G	A
TCGA-24-1103	OR2AP1	ENST00000321688	p.V210V	c.630G>A	Verified	CTG	GTG	CTA	3	G	A
TCGA-23-1111	OR2J2	ENST00000377167	p.A147A	c.441G>A	Unverified	GTT	GCG	GCT	3	G	A
TCGA-04-1652	OR2L1P	ENST00000355281	p.A111A	c.333G>A	Unverified	GAA	GCG	CTG	3	G	A
TCGA-04-1652	OR2L1P	ENST00000355281	p.A111A	c.333G>A	Unverified	GAA	GCG	CTG	3	G	A
TCGA-13-0883	OR2M2	ENST00000359682	p.S21N	c.62G>A	Verified	CAC	AGC	CCA	2	G	A
TCGA-04-1331	OR2M2	ENST00000359682	p.M144I	c.432G>A	Verified	CTT	ATG	GCT	3	G	A
TCGA-61-1904	OR2T33	ENST00000318021	p.R184H	c.551G>A	Unverified	GTG	CGT	TTG	2	G	A
TCGA-24-0979	OR2W3	ENST00000360358	p.C169Y	c.506G>A	Verified	CGC	TGT	GGG	2	G	A
TCGA-29-1762	OR2W5	ENST00000366492	p.D300N	c.898G>A	Unverified	GGG	GAC	CAT	1	G	A
TCGA-04-1342	OR3A1	ENST00000323404	p.M214I	c.642G>A	Unverified	CCC	ATG	GCT	3	G	A
TCGA-29-1769	OR4C16	ENST00000314634	p.R120H	c.359G>A	Unverified	GAC	CGC	TAT	2	G	A
TCGA-24-1849	OR4D1	ENST00000268912	p.A270T	c.808G>A	Unverified	AAG	GCT	GTG	1	G	A
TCGA-23-1809	OR4N2	ENST00000315947	p.G170D	c.509G>A	Unverified	TGT	GGC	CCA	2	G	A
TCGA-61-1899	OR4N2	ENST00000315947	p.R290H	c.869G>A	Unverified	CTT	CGC	AAC	2	G	A
TCGA-13-1509	OR51A4	ENST00000380373	p.C181Y	c.542G>A	Verified	TAC	TGT	CTC	2	G	A
TCGA-24-1474	OR51B2	ENST00000328813	p.V266M	c.796G>A	Verified	AAT	GTG	CCA	1	G	A
TCGA-30-1714	OR51B6	ENST00000380219	p.V199I	c.595G>A	Unverified	GTT	GTA	GTT	1	G	A
TCGA-09-2056	OR51F2	ENST00000322110	p.A316T	c.946G>A	Verified	AAG	GCC	ATT	1	G	A
TCGA-13-0886	OR51G1	ENST00000321961	p.A221T	c.661G>A	Unverified	TAC	GCC	CTC	1	G	A
TCGA-23-1114	OR52R1	ENST00000380382	p.R323H	c.968G>A	Unverified	ACA	CGT	TCC	2	G	A
TCGA-24-1435	OR5D16	ENST00000378396	p.V282M	c.844G>A	Verified	ACC	GTG	GTG	1	G	A
TCGA-29-1761	OR5D16	ENST00000378396	p.V49V	c.147G>A	Unverified	ATA	GTG	ATC	3	G	A
TCGA-23-1110	OR5H2	ENST00000355273	p.G21E	c.62G>A	Verified	ACA	GGA	CTT	2	G	A
TCGA-04-1362	OR5I1	ENST00000301532	p.E198K	c.592G>A	Verified	AAT	GAG	TGG	1	G	A
TCGA-04-1347	OR5K2	ENST00000427338	p.V208I	c.622G>A	Verified	CAA	GTC	TTT	1	G	A
TCGA-61-1899	OR5M3	ENST00000312240	p.D294N	c.880G>A	Unverified	AAG	GAT	GTG	1	G	A

TCGA-23-2077	OR5V1	ENST00000377154	p.R261Q	c.782G>A	Verified	GTA	CGG	CCC	2	G	A
TCGA-23-1117	OR6X1	ENST00000327930	p.L79L	c.237G>A	Verified	AAA	CTG	CTA	3	G	A
TCGA-23-1117	OR6X1	ENST00000327930	p.L79L	c.237G>A	Verified	AAA	CTG	CTA	3	G	A
TCGA-13-0807	OR7C2	ENST00000248072	p.V225I	c.673G>A	Verified	TCC	GTC	CTA	1	G	A
TCGA-20-1687	OR7C2	ENST00000248072	p.T118T	c.354G>A	Unverified	ATG	ACG	GCC	3	G	A
TCGA-13-0916	OR7C2	ENST00000248072	p.T133T	c.399G>A	Verified	TAC	ACG	GTC	3	G	A
TCGA-13-1501	OR7D4	ENST00000308682	p.E173K	c.517G>A	Verified	ACT	GAG	ATT	1	G	A
TCGA-13-1501	OR7D4	ENST00000308682	p.E173K	c.517G>A	Verified	ACT	GAG	ATT	1	G	A
TCGA-09-2050	OR7D4	ENST00000308682	p.M81I	c.243G>A	Verified	AAG	ATG	CTA	3	G	A
TCGA-13-0893	OR8B3	ENST00000354597	p.R122H	c.365G>A	Verified	GAT	CGC	TAT	2	G	A
TCGA-23-1021	OR8U1	ENST00000302270	p.G44D	c.131G>A	Verified	TTG	GGT	TTG	2	G	A
TCGA-29-1761	ORC1	ENST00000371568	p.R844Q	c.2531G>A	Unverified	ctt	cgg	gtg	2	G	A
TCGA-24-1423	OSBPL1A	ENST00000319481	p.A660T	c.1978G>A	Verified	CAT	GCT	GAA	1	G	A
TCGA-23-2078	OSCP1	ENST00000356637	p.G250R	c.748G>A	Unverified	TAT	GGA	GAC	1	G	A
TCGA-13-0792	OSCP1	ENST00000356637	p.R252Q	c.755G>A	Verified	GAC	CGA	GTC	2	G	A
TCGA-23-2078	OSR2	ENST00000297565	p.E128E	c.384G>A	Unverified	CAA	GAG	GAT	3	G	A
TCGA-04-1338	OTOA	ENST00000388958	p.A705T	c.2113G>A	Verified	AGG	GCT	TGG	1	G	A
TCGA-20-1686	OTOF	ENST00000272371	p.A1063A	c.3189G>A	Unverified	GAG	GCG	TAC	3	G	A
TCGA-20-1686	OTOF_ENST	ENST00000339598	p.A316A	c.948G>A	Unverified	GAG	GCG	TAC	3	G	A
TCGA-20-1686	OTOF_ENST	ENST00000402415	p.A373A	c.1119G>A	Unverified	GAG	GCG	TAC	3	G	A
TCGA-04-1649	OTOL1	ENST00000327928	p.R319Q	c.956G>A	Unverified	GTC	CGA	GGC	2	G	A
TCGA-13-0755	OTOP1	ENST00000296358	p.R370Q	c.1109G>A	Verified	ATC	CGG	ATT	2	G	A
TCGA-04-1655	OTOP1	ENST00000296358	p.S345S	c.1035G>A	Unverified	GAG	TCG	GCA	3	G	A
TCGA-24-1435	OTOR	ENST00000246081	p.D54N	c.160G>A	Verified	ccg	gac	tgt	1	G	A
TCGA-23-2077	OVGP1	ENST00000369732	p.G259R	c.775G>A	Verified	ATG	GGG	ATC	1	G	A
TCGA-23-2078	OXGR1	ENST00000298440	p.V252I	c.754G>A	Verified	TAC	GTA	TGT	1	G	A
TCGA-23-1110	P2RY1	ENST00000305097	p.T13T	c.39G>A	Unverified	GGG	ACG	GAC	3	G	A
TCGA-13-0887	PABPN1	ENST00000216727	p.G283S	c.847G>A	Verified	AGT	GGT	TTT	1	G	A
TCGA-23-1122	PADI1	ENST00000375471	p.V418I	c.1252G>A	Unverified	CCC	GTC	ACG	1	G	A
TCGA-29-1770	PADI4	ENST00000375448	p.L151L	c.453G>A	Unverified	CTG	CTG	GTG	3	G	A
TCGA-23-1116	PADI6	ENST00000358481	p.P467P	c.1401G>A	Unverified	GCG	CCG	GTG	3	G	A
TCGA-04-1336	PAK2	ENST00000327134	p.E8K	c.22G>A	Verified	CTG	GAA	GAT	1	G	A
TCGA-13-0760	PAK4	ENST00000358301	p.L566L	c.1698G>A	Verified	gag	ctg	ctg	3	G	A
TCGA-13-1408	PALM2-AKA	ENST00000374530	p.A641T	c.1921G>A	Verified	ACA	GCC	TCT	1	G	A

TCGA-61-1900	PAPD7	ENST00000230859	p.D142N	c.424G>A	Unverified	AGG	GAC	CTG	1	G	A
TCGA-25-1318	PAPPA2	ENST00000367662	p.E1596K	c.4786G>A	Verified	TGT	GAG	CCA	1	G	A
TCGA-25-1318	PAPPA2	ENST00000367662	p.E1596K	c.4786G>A	Verified	TGT	GAG	CCA	1	G	A
TCGA-30-1855	PAPSS1	ENST00000265174	p.R139H	c.416G>A	Unverified	GAT	CGC	AAC	2	G	A
TCGA-04-1530	PARP1	ENST00000366794	p.M615I	c.1845G>A	Verified	TTC	ATG	AAA	3	G	A
TCGA-61-2012	PARP10	ENST00000313028	p.V53I	c.157G>A	Unverified	GGC	GTC	CTC	1	G	A
TCGA-24-0979	PARP15	ENST00000310366	p.S343N	c.1028G>A	Verified	AGA	AGT	TGT	2	G	A
TCGA-13-1488	PASK	ENST00000234040	p.R298K	c.893G>A	Verified	CAG	AGG	TCT	2	G	A
TCGA-13-0714	PAX3	ENST00000392069	p.R236H	c.707G>A	Verified	GAG	CGT	GCT	2	G	A
TCGA-61-1915	PAXIP1	NM_007349.2	p.V127M	c.379G>A	Unverified	att	gtg	act	1	G	A
TCGA-20-1683	PAXIP1	NM_007349.2	p.P381P	c.1143G>A	Unverified	cac	ccg	gtt	3	G	A
TCGA-61-1915	PAXIP1_ENS	ENST00000404141	p.V161M	c.481G>A	Unverified	ATT	GTG	ACT	1	G	A
TCGA-20-1683	PAXIP1_ENS	ENST00000404141	p.P415P	c.1245G>A	Unverified	CAC	CCG	GTT	3	G	A
TCGA-13-0886	PCDH1	ENST00000287008	p.L238L	c.714G>A	Verified	AAC	CTG	GAC	3	G	A
TCGA-13-0760	PCDH17	ENST00000377918	p.D703N	c.2107G>A	Verified	TGG	GAC	ATG	1	G	A
TCGA-29-1703	PCDH19	ENST00000373034	p.R980H	c.2939G>A	Unverified	ATC	CGT	TCC	2	G	A
TCGA-29-1703	PCDH19_NM	NM_020766.1	p.R433H	c.1298G>A	Unverified	atc	cgt	tcc	2	G	A
TCGA-24-1469	PCDH9	ENST00000377865	p.G1075D	c.3224G>A	Unverified	GTG	GGT	AGT	2	G	A
TCGA-61-1906	PCDH9	ENST00000377865	p.K762K	c.2286G>A	Unverified	CCT	AAG	TCT	3	G	A
TCGA-61-1906	PCDH9	ENST00000377865	p.K762K	c.2286G>A	Unverified	CCT	AAG	TCT	3	G	A
TCGA-23-1122	PCDHA10	ENST00000506939	p.A453A	c.1359G>A	Verified	CCT	GCG	TTC	3	G	A
TCGA-13-1499	PCDHA10_E	ENST00000505235	p.A704T	c.2110G>A	Verified	TGC	GCG	GTG	1	G	A
TCGA-23-1122	PCDHA10_E	ENST00000505235	p.A453A	c.1359G>A	Verified	CCT	GCG	TTC	3	G	A
TCGA-09-0369	PCDHA2	ENST00000378132	p.V624M	c.1870G>A	Verified	CGC	GTG	GGG	1	G	A
TCGA-09-0369	PCDHA2	ENST00000378132	p.V624M	c.1870G>A	Verified	CGC	GTG	GGG	1	G	A
TCGA-29-1702	PCDHA4	ENST00000356878	p.R244K	c.731G>A	Unverified	GAC	AGA	ACC	2	G	A
TCGA-24-2280	PCDHA4	ENST00000356878	p.L435L	c.1305G>A	Unverified	TCG	CTG	TGG	3	G	A
TCGA-29-1702	PCDHA4_EN	ENST00000530339	p.R244K	c.731G>A	Unverified	GAC	AGA	ACC	2	G	A
TCGA-24-1469	PCDHA5	ENST00000378126	p.V397V	c.1191G>A	Verified	CTG	GTG	TCC	3	G	A
TCGA-13-0791	PCDHA7	ENST00000378125	p.P469P	c.1407G>A	Verified	AAC	CCG	CCG	3	G	A
TCGA-13-0793	PCDHAC1	ENST00000253807	p.A19A	c.57G>A	Unverified	GCA	GCG	GGA	3	G	A
TCGA-24-1849	PCDHB10	ENST00000239446	p.D51N	c.151G>A	Unverified	AAG	GAT	CTG	1	G	A
TCGA-61-1738	PCDHB10	ENST00000239446	p.A515T	c.1543G>A	Unverified	TTC	GCC	CTC	1	G	A
TCGA-61-1738	PCDHB10	ENST00000239446	p.A515T	c.1543G>A	Unverified	TTC	GCC	CTC	1	G	A

TCGA-09-0369	PCDHB11	ENST00000354757	p.V364M	c.1090G>A	Verified	ACC	GTG	GTT	1	G	A
TCGA-09-0369	PCDHB11	ENST00000354757	p.V364M	c.1090G>A	Verified	ACC	GTG	GTT	1	G	A
TCGA-13-1509	PCDHB18	ENST00000274705	p.E246K	c.736G>A	Verified	GGC	GAA	GTC	1	G	A
TCGA-24-1845	PCDHB18	ENST00000274705	p.D319N	c.955G>A	Unverified	CGA	GAC	CAA	1	G	A
TCGA-61-1895	PCDHB3	ENST00000231130	p.A681T	c.2041G>A	Unverified	CAG	GCC	CAG	1	G	A
TCGA-13-0913	PCDHB5	ENST00000231134	p.R202Q	c.605G>A	Verified	GAC	CGG	GAG	2	G	A
TCGA-23-1116	PCDHB5	ENST00000231134	p.G510D	c.1529G>A	Unverified	AAC	GGC	CAC	2	G	A
TCGA-24-1422	PCDHB6	ENST00000231136	p.L548L	c.1644G>A	Unverified	TTG	CTG	GTG	3	G	A
TCGA-23-1022	PCDHB7	ENST00000231137	p.A579A	c.1737G>A	Verified	CGG	GCG	GCC	3	G	A
TCGA-23-1124	PCDHGA1	ENST00000378105	p.A637A	c.1911G>A	Verified	GAC	GCG	CTC	3	G	A
TCGA-24-1844	PCDHGA6_E	ENST00000517434	p.E463K	c.1387G>A	Unverified	CTT	GAA	AAC	1	G	A
TCGA-04-1652	PCDHGB2_E	ENST00000522605	p.S360S	c.1080G>A	Unverified	GAT	TCG	CCA	3	G	A
TCGA-04-1652	PCDHGB2_E	ENST00000522605	p.S360S	c.1080G>A	Unverified	GAT	TCG	CCA	3	G	A
TCGA-61-1998	PCDHGB7	ENST00000398594	p.V333I	c.997G>A	Verified	AAA	GTA	ATT	1	G	A
TCGA-29-1702	PCK1	ENST00000319441	p.T384T	c.1152G>A	Unverified	ATC	ACG	TCC	3	G	A
TCGA-04-1331	PCLO	ENST00000333891	p.A4321T	c.12961G>A	Verified	GAG	GCT	GAA	1	G	A
TCGA-13-1488	PCLO	ENST00000333891	p.Q568Q	c.1704G>A	Verified	CTG	CAG	CCA	3	G	A
TCGA-24-1849	PCNXL2	ENST00000258229	p.R2012Q	c.6035G>A	Unverified	GAG	CGG	GCC	2	G	A
TCGA-25-2398	PDE1B	ENST00000243052	p.V478M	c.1432G>A	Verified	GAT	GTG	GTC	1	G	A
TCGA-23-1122	PDE4A	ENST00000344979	p.E162K	c.484G>A	Unverified	CAG	GAG	CGG	1	G	A
TCGA-10-0930	PDE8A	ENST00000310298	p.L121L	c.363G>A	Verified	TTC	CTG	GAC	3	G	A
TCGA-10-0930	PDE8A	ENST00000310298	p.L121L	c.363G>A	Verified	TTC	CTG	GAC	3	G	A
TCGA-24-1844	PDGFD	ENST00000393158	p.D261N	c.781G>A	Unverified	CTG	GAT	AGG	1	G	A
TCGA-24-1844	PDGFD_ENS	ENST00000393158	p.D261N	c.781G>A	Unverified	CTG	GAT	AGG	1	G	A
TCGA-24-2280	PDGFRA	ENST00000257290	p.G185E	c.554G>A	Unverified	GTA	GGG	CCC	2	G	A
TCGA-23-2078	PDHA1	ENST00000422285	p.L24L	c.72G>A	Verified	gtg	ctg	gta	3	G	A
TCGA-29-1784	PDIA5	ENST00000316218	p.K167K	c.501G>A	Unverified	CTG	AAG	AAG	3	G	A
TCGA-36-1577	PDK3	ENST00000379162	p.V193M	c.577G>A	Verified	AAC	GTG	GCG	1	G	A
TCGA-61-1895	PDLIM1	ENST00000329399	p.E208K	c.622G>A	Unverified	AAT	GAG	CCC	1	G	A
TCGA-13-0760	PDLIM4	ENST00000253754	p.R299R	c.897G>A	Verified	GAG	CGG	CTC	3	G	A
TCGA-13-0905	PDZD2	ENST00000282493	p.C2022Y	c.6065G>A	Verified	AAG	TGT	AGA	2	G	A
TCGA-30-1856	PECR	ENST00000265322	p.G42E	c.125G>A	Unverified	CTG	GGG	AGT	2	G	A
TCGA-13-1488	PEG3	ENST00000326441	p.D1460N	c.4378G>A	Verified	GAA	GAC	CCA	1	G	A
TCGA-04-1337	PEG3	ENST00000326441	p.R598H	c.1793G>A	Unverified	GAA	CGT	GAA	2	G	A

TCGA-24-0975	PELO	ENST00000274311	p.W27*	c.81G>A	Unverified	ATG	TGG	CAC	3	G	A
TCGA-13-1512	PEX1	ENST00000248633	p.V223V	c.669G>A	Verified	ACT	GTG	GGA	3	G	A
TCGA-23-1029	PEX5L	ENST00000467460	p.G204E	c.611G>A	Unverified	ACT	GGA	TCA	2	G	A
TCGA-10-0930	PFAS	ENST00000314666	p.P133P	c.399G>A	Verified	CCC	CCG	TCA	3	G	A
TCGA-10-0930	PFAS	ENST00000314666	p.P133P	c.399G>A	Verified	CCC	CCG	TCA	3	G	A
TCGA-13-0792	PFKP	ENST00000381125	p.M758I	c.2274G>A	Unverified	CTC	ATG	AAA	3	G	A
TCGA-61-1740	PFN4	ENST00000313213	p.G37S	c.109G>A	Unverified	CCA	GGT	TTC	1	G	A
TCGA-23-1118	PGLYRP3	ENST00000290722	p.A17T	c.49G>A	Verified	CAG	GCT	TGG	1	G	A
TCGA-29-1763	PGM5	ENST00000396396	p.R560R	c.1680G>A	Unverified	GGC	CGG	AGG	3	G	A
TCGA-24-1474	PHACTR2	ENST00000305766	p.R395Q	c.1184G>A	Verified	CGC	CGG	AGG	2	G	A
TCGA-23-2078	PHACTR3	ENST00000371015	p.D168N	c.502G>A	Unverified	CTG	GAC	AAG	1	G	A
TCGA-13-1510	PHACTR3	ENST00000371015	p.R49H	c.146G>A	Verified	GCG	CGT	CCT	2	G	A
TCGA-13-0792	PHC1	ENST00000251757	p.A101T	c.301G>A	Verified	CAG	GCC	TCG	1	G	A
TCGA-29-1691	PHF12	ENST00000332830	p.C842Y	c.2525G>A	Unverified	GCC	TGC	ATA	2	G	A
TCGA-13-1509	PHF21B	ENST00000313237	p.A280T	c.838G>A	Verified	ATC	GCC	TTC	1	G	A
TCGA-13-0886	PHF8	ENST00000338154	p.E411K	c.1231G>A	Verified	aaa	gaa	gct	1	G	A
TCGA-61-1725	PHIP	ENST00000275034	p.K1657K	c.4971G>A	Unverified	CAC	AAG	AAA	3	G	A
TCGA-29-1762	PHLDB2	ENST00000412622	p.R1080Q	c.3239G>A	Unverified	TTG	CGG	AGC	2	G	A
TCGA-29-1762	PHLDB2_EN	ENST00000393923	p.R1107Q	c.3320G>A	Unverified	TTG	CGG	AGC	2	G	A
TCGA-29-1762	PHLDB2_EN	ENST00000431670	p.R1123Q	c.3368G>A	Unverified	TTG	CGG	AGC	2	G	A
TCGA-25-1313	PI4KA	NM_058004.1	p.S1526N	c.4577G>A	Unverified	gtt	agt	gat	2	G	A
TCGA-29-1691	PI4KB	ENST00000368875	p.G38E	c.113G>A	Unverified	AAT	GGG	GGG	2	G	A
TCGA-24-1616	PICALM	ENST00000447890	p.V634I	c.1900G>A	Verified	CCT	GTC	ATG	1	G	A
TCGA-29-1769	PIGF	ENST00000281382	p.W138*	c.414G>A	Unverified	gca	tgg	cta	3	G	A
TCGA-23-2078	PIGG	ENST00000310340	p.M837I	c.2511G>A	Unverified	GTG	ATG	CAT	3	G	A
TCGA-25-2393	PIGK	ENST00000370812	p.S278N	c.833G>A	Verified	AAA	AGT	CTG	2	G	A
TCGA-13-1488	PIGO	ENST00000378617	p.R854H	c.2561G>A	Verified	gag	cgc	atc	2	G	A
TCGA-61-2109	PIGO	ENST00000378617	p.R745Q	c.2234G>A	Unverified	cct	cgg	gct	2	G	A
TCGA-61-1737	PIGX	ENST00000314118	p.V67M	c.199G>A	Unverified	GCA	GTG	ATG	1	G	A
TCGA-24-0979	PIK3AP1	ENST00000339364	p.G618S	c.1852G>A	Verified	CTG	GGC	ATT	1	G	A
TCGA-13-1510	PIK3C2A	ENST00000265970	p.V600I	c.1798G>A	Unverified	TCA	GTA	AAG	1	G	A
TCGA-29-1775	PIK3CG	ENST00000440650	p.K455K	c.1365G>A	Unverified	TCC	AAG	GGC	3	G	A
TCGA-61-2109	PIK3R4	ENST00000356763	p.G609D	c.1826G>A	Unverified	gtt	ggc	tgg	2	G	A
TCGA-24-1847	PIP	ENST00000291009	p.A57T	c.169G>A	Unverified	CTT	GCA	GTT	1	G	A

TCGA-24-1616	PIRT	ENST00000580256	p.M3I	c.9G>A	Verified	ACG	ATG	GAG	3	G	A
TCGA-13-1507	PITPNA	ENST00000313486	p.V136M	c.406G>A	Verified	CAC	GTG	GAA	1	G	A
TCGA-13-0791	PITPNM2	ENST00000320201	p.T1092T	c.3276G>A	Verified	CAC	ACG	TTT	3	G	A
TCGA-24-1563	PITRM1	ENST00000380989	p.E107E	c.321G>A	Unverified	CTT	GAG	CAT	3	G	A
TCGA-23-1116	PKD1L1	ENST00000289672	p.R1907H	c.5720G>A	Unverified	GGT	CGC	GTG	2	G	A
TCGA-23-2077	PKD1L2_ENS	ENST00000525539	p.C1355Y	c.4064G>A	Verified	CTG	TGT	GTG	2	G	A
TCGA-20-1686	PKD2L1	ENST00000318222	p.M121I	c.363G>A	Unverified	GGA	ATG	ACA	3	G	A
TCGA-24-1469	PKHD1	ENST00000371117	p.R1804H	c.5411G>A	Verified	AAG	CGT	GAG	2	G	A
TCGA-24-1850	PKHD1	ENST00000371117	p.A1826A	c.5478G>A	Unverified	ATG	GCG	ACA	3	G	A
TCGA-24-1850	PKHD1_ENS	ENST00000340994	p.A1826A	c.5478G>A	Unverified	ATG	GCG	ACA	3	G	A
TCGA-23-1122	PKHD1L1	ENST00000378402	p.V1277M	c.3829G>A	Verified	GCG	GTG	TAT	1	G	A
TCGA-13-1507	PKHD1L1	ENST00000378402	p.C1884Y	c.5651G>A	Verified	TAC	TGC	CGC	2	G	A
TCGA-04-1356	PKHD1L1	ENST00000378402	p.Q4100Q	c.12300G>A	Verified	GGA	CAG	CCA	3	G	A
TCGA-23-1021	PKN1	ENST00000242783	p.E764K	c.2290G>A	Verified	AAG	GAG	GGG	1	G	A
TCGA-09-2050	PKNOX2	ENST00000298282	p.Q388Q	c.1164G>A	Verified	CAG	CAG	GGC	3	G	A
TCGA-13-0900	PKP1	ENST00000367324	p.S494N	c.1481G>A	Verified	TTC	AGC	AAC	2	G	A
TCGA-13-0755	PKP4	ENST00000389759	p.V743M	c.2227G>A	Verified	TGC	GTG	TGC	1	G	A
TCGA-13-0905	PKP4	ENST00000389759	p.A703T	c.2107G>A	Verified	TCC	GCG	GGG	1	G	A
TCGA-23-1123	PKP4	ENST00000389759	p.S336S	c.1008G>A	Verified	TCG	TCG	TCC	3	G	A
TCGA-23-1123	PKP4	ENST00000389759	p.S336S	c.1008G>A	Verified	TCG	TCG	TCC	3	G	A
TCGA-30-1714	PLA2G4C	ENST00000413144	p.V511I	c.1531G>A	Unverified	AAT	GTC	AGG	1	G	A
TCGA-23-1022	PLA2G6	ENST00000332509	p.C705Y	c.2114G>A	Verified	ACC	TGT	GTG	2	G	A
TCGA-61-2009	PLA2R1	ENST00000283243	p.?	c.2401+1G>A	Verified	AGA	GAT	GTG	1	G	A
TCGA-09-2056	PLB1	ENST00000327757	p.E321K	c.961G>A	Verified	GAT	GAG	CCA	1	G	A
TCGA-25-1313	PLCB1	ENST00000338037	p.E539K	c.1615G>A	Verified	gaa	gaa	atg	1	G	A
TCGA-13-0883	PLCB2	ENST00000260402	p.G193D	c.578G>A	Verified	AAA	GGC	AAA	2	G	A
TCGA-23-1124	PLCB3	ENST00000279230	p.R707Q	c.2120G>A	Verified	CGG	CGG	CCG	2	G	A
TCGA-24-0979	PLCXD3	ENST00000377801	p.G167R	c.499G>A	Unverified	tat	gga	aat	1	G	A
TCGA-04-1342	PLEC	ENST00000322810	p.R800Q	c.2399G>A	Unverified	ATG	CGG	GAG	2	G	A
TCGA-09-1674	PLEKHA4	ENST00000263265	p.R190H	c.569G>A	Unverified	GGG	CGC	ATC	2	G	A
TCGA-04-1331	PLEKHF1	ENST00000436066	p.G138S	c.412G>A	Unverified	ACG	GGC	CGC	1	G	A
TCGA-09-2049	PLEKHF2	ENST00000315367	p.D240N	c.718G>A	Verified	TCT	GAT	GAT	1	G	A
TCGA-61-1998	PLEKHM3	ENST00000427836	p.W228*	c.684G>A	Verified	TAC	TGG	CAA	3	G	A
TCGA-29-1762	PLG	ENST00000308192	p.W334*	c.1001G>A	Unverified	CCA	TGG	TGC	2	G	A

TCGA-29-1703	PLK1	ENST00000300093	p.R262Q	c.785G>A	Unverified	CTC	CGG	ATC	2	G	A
TCGA-04-1542	PLOD1	ENST00000196061	p.V218M	c.652G>A	Verified	GTC	GTG	CTC	1	G	A
TCGA-29-1777	PLXDC2	ENST00000377252	p.L97L	c.291G>A	Unverified	CTG	CTG	GAT	3	G	A
TCGA-61-1738	PLXNA3	ENST00000369682	p.A580A	c.1740G>A	Unverified	gcg	gcg	gag	3	G	A
TCGA-61-1738	PLXNA3	ENST00000369682	p.A580A	c.1740G>A	Unverified	gcg	gcg	gag	3	G	A
TCGA-29-1691	PLXNA4_EN:	ENST00000321063	p.V658I	c.1972G>A	Unverified	AGC	GTC	CAC	1	G	A
TCGA-61-1725	PLXNA4_EN:	ENST00000321063	p.G1174G	c.3522G>A	Unverified	GCT	GGG	GGC	3	G	A
TCGA-29-1691	PLXNA4_EN:	ENST00000321063	p.V658I	c.1972G>A	Unverified	AGC	GTC	CAC	1	G	A
TCGA-61-1725	PLXNA4_EN:	ENST00000321063	p.G1174G	c.3522G>A	Unverified	GCT	GGG	GGC	3	G	A
TCGA-23-1022	PLXNC1	ENST00000258526	p.V430I	c.1288G>A	Verified	CTC	GTT	CCT	1	G	A
TCGA-10-0930	PMFBP1	ENST00000237353	p.K248K	c.744G>A	Verified	CAA	AAG	GTC	3	G	A
TCGA-10-0930	PMFBP1	ENST00000237353	p.K248K	c.744G>A	Verified	CAA	AAG	GTC	3	G	A
TCGA-24-1845	PMS2P3	NM_005395.2	p.G59R	c.175G>A	Unverified	cat	gga	gtg	1	G	A
TCGA-24-1431	PNISR	ENST00000369239	p.A234T	c.700G>A	Unverified	CCC	GCT	TGG	1	G	A
TCGA-29-1770	PNMAL2_EN	ENST00000599531	p.A393A	c.1179G>A	Unverified	GAG	GCG	GGC	3	G	A
TCGA-20-1687	PODN	ENST00000312553	p.R428H	c.1283G>A	Unverified	CCT	CGC	CGC	2	G	A
TCGA-24-2290	PODXL2	ENST00000342480	p.G339R	c.1015G>A	Verified	CCT	GGA	GAC	1	G	A
TCGA-24-2290	PODXL2	ENST00000342480	p.G339R	c.1015G>A	Verified	CCT	GGA	GAC	1	G	A
TCGA-23-1122	POGZ	ENST00000271715	p.G192D	c.575G>A	Verified	CCA	GGT	ACC	2	G	A
TCGA-61-2012	POLG	ENST00000268124	p.V825M	c.2473G>A	Verified	gct	gtg	atc	1	G	A
TCGA-23-2078	POLM	ENST00000242248	p.R175H	c.524G>A	Verified	ggc	cgc	ctc	2	G	A
TCGA-61-2012	POLR1A	ENST00000263857	p.Q1526Q	c.4578G>A	Verified	TGC	CAG	GTG	3	G	A
TCGA-24-1604	POLR1B	ENST00000263331	p.L619L	c.1857G>A	Verified	AGA	CTG	GTA	3	G	A
TCGA-61-1740	POLR2B	ENST00000381227	p.R425Q	c.1274G>A	Unverified	GAT	CGA	GGA	2	G	A
TCGA-61-1904	POLR3B	ENST00000228347	p.G690R	c.2068G>A	Unverified	ATG	GGG	AAA	1	G	A
TCGA-61-1904	POM121	ENST00000257622	p.G234S	c.700G>A	Unverified	cct	ggc	agc	1	G	A
TCGA-61-1904	POM121_EN	ENST00000395270	p.G234S	c.700G>A	Unverified	CCT	GGC	AGC	1	G	A
TCGA-04-1649	POM121L12	ENST00000408890	p.A142A	c.426G>A	Unverified	ATC	GCG	CCC	3	G	A
TCGA-13-1501	POP1	ENST00000349693	p.L812L	c.2436G>A	Verified	TTA	CTG	AAG	3	G	A
TCGA-13-1501	POP1	ENST00000349693	p.L812L	c.2436G>A	Verified	TTA	CTG	AAG	3	G	A
TCGA-25-2400	POPDC3	ENST00000254765	p.R261R	c.783G>A	Verified	ATT	CGG	CTA	3	G	A
TCGA-29-1695	POT1	ENST00000357628	p.A6T	c.16G>A	Unverified	CCA	GCA	ACA	1	G	A
TCGA-29-1695	POT1_ENST(	ENST00000357628	p.A6T	c.16G>A	Unverified	CCA	GCA	ACA	1	G	A
TCGA-20-1686	POTED	ENST00000299443	p.K171K	c.513G>A	Unverified	GAC	AAG	GAA	3	G	A

TCGA-04-1652	POU2F3	ENST00000260264	p.P93P	c.279G>A	Unverified	CAT	CCG	CTC	3	G	A
TCGA-04-1652	POU2F3	ENST00000260264	p.P93P	c.279G>A	Unverified	CAT	CCG	CTC	3	G	A
TCGA-29-1695	PPARGC1A	ENST00000264867	p.G682S	c.2044G>A	Unverified	gtc	ggt	aaa	1	G	A
TCGA-13-0900	PPFIA1	ENST00000253925	p.R137Q	c.410G>A	Verified	gag	cgg	tct	2	G	A
TCGA-24-1435	PPFIA4	XM_046751.7	p.M1120I	c.3360G>A	Verified	cgc	atg	ctg	3	G	A
TCGA-24-1463	PPIAL4G	ENST00000419275	p.V136V	c.408G>A	Unverified	CGT	GTG	AAT	3	G	A
TCGA-04-1356	PPL	ENST00000345988	p.L767L	c.2301G>A	Verified	AAG	CTG	AAG	3	G	A
TCGA-09-1674	PPP1R21	ENST00000294952	p.E366K	c.1096G>A	Unverified	GAA	GAA	GAA	1	G	A
TCGA-13-0714	PPP1R3A	NM_002711.2	p.V1102I	c.3304G>A	Verified	tac	gtt	ttg	1	G	A
TCGA-13-0906	PPP2R5B	ENST00000164133	p.K331K	c.993G>A	Verified	cag	aag	gag	3	G	A
TCGA-09-1674	PPPDE1	ENST00000302550	p.G58R	c.172G>A	Unverified	CCA	GGA	AAT	1	G	A
TCGA-61-1733	PQLC2	ENST00000375155	p.W23*	c.68G>A	Unverified	ATA	TGG	GAT	2	G	A
TCGA-61-1733	PQLC2	ENST00000375155	p.W23*	c.68G>A	Unverified	ATA	TGG	GAT	2	G	A
TCGA-29-1705	PRAMEF1	ENST00000332296	p.P464P	c.1392G>A	Unverified	TCA	CCG	TCT	3	G	A
TCGA-29-1705	PRAMEF2	ENST00000240189	p.P464P	c.1392G>A	Unverified	TCA	CCG	TCT	3	G	A
TCGA-29-1766	PRAMEF7	ENST00000330881	p.R444R	c.1332G>A	Unverified	TTA	AGG	CAG	3	G	A
TCGA-29-1769	PRB2	ENST00000389362	p.G223E	c.668G>A	Unverified	CAA	GGA	CCA	2	G	A
TCGA-29-1769	PRB2_ENST(	ENST00000389362	p.G244E	c.731G>A	Unverified	CAA	GGA	CCA	2	G	A
TCGA-25-1313	PRDM14	ENST00000276594	p.V328I	c.982G>A	Verified	TAT	GTC	AAC	1	G	A
TCGA-24-1844	PRDM9	ENST00000296682	p.A41T	c.121G>A	Unverified	TGG	GCA	GAG	1	G	A
TCGA-61-1998	PRDM9	ENST00000296682	p.D266N	c.796G>A	Unverified	TCT	GAT	CTG	1	G	A
TCGA-20-1686	PRG2_ENST(	ENST00000311862	p.R126Q	c.377G>A	Unverified	TGC	CGG	AGG	2	G	A
TCGA-09-1674	PRKAB2	ENST00000254101	p.M193I	c.579G>A	Unverified	gaa	atg	tat	3	G	A
TCGA-13-0893	PRKCA	ENST00000413366	p.R161Q	c.482G>A	Verified	GGG	CGG	ATT	2	G	A
TCGA-04-1338	PRKCG	ENST00000263431	p.D254N	c.760G>A	Verified	AAC	GAC	TTC	1	G	A
TCGA-24-1604	PRKCH	NM_006255	p.V390M	c.1168G>A	Verified	gac	gtg	att	1	G	A
TCGA-61-1907	PRKRIR	ENST00000260045	p.G376R	c.1126G>A	Unverified	ATG	GGA	GTA	1	G	A
TCGA-23-1116	PRMT1	ENST00000391849	p.T291T	c.873G>A	Unverified	CAG	ACG	GTG	3	G	A
TCGA-23-2077	PROM2	ENST00000317668	p.E661K	c.1981G>A	Unverified	GAG	GAG	GCC	1	G	A
TCGA-29-1769	PROZ	ENST00000375547	p.E51K	c.151G>A	Unverified	TTC	GAG	GGA	1	G	A
TCGA-13-0919	PRPF4	ENST00000374198	p.V140I	c.418G>A	Verified	GTT	GTC	GGT	1	G	A
TCGA-13-0920	PRPH2	ENST00000230381	p.V209I	c.625G>A	Verified	GGC	GTC	CCT	1	G	A
TCGA-09-2044	PRRT2	ENST00000358758	p.E46E	c.138G>A	Unverified	CCA	GAG	GCC	3	G	A
TCGA-04-1356	PRSS36	ENST00000268281	p.W357*	c.1070G>A	Unverified	AGC	TGG	GTC	2	G	A

TCGA-24-2280	PRSS36	ENST00000268281	p.R314R	c.942G>A	Unverified	CCC	AGG	GAG	3	G	A
TCGA-24-1431	PRSS54	ENST00000219301	p.R14Q	c.41G>A	Verified	ATG	CGA	GGG	2	G	A
TCGA-13-0904	PRTFDC1	ENST00000320152	p.G125R	c.373G>A	Verified	ATC	GGA	GGC	1	G	A
TCGA-61-1725	PSAP	ENST00000394936	p.G480R	c.1438G>A	Unverified	ATT	GGA	GCC	1	G	A
TCGA-25-2398	PSD_ENST0(	ENST00000020673	p.A166T	c.496G>A	Verified	TCG	GCC	CTA	1	G	A
TCGA-29-1785	PSD2	ENST00000274710	p.A55A	c.165G>A	Unverified	CCA	GCG	GAC	3	G	A
TCGA-29-1785	PSD2	ENST00000274710	p.A55A	c.165G>A	Unverified	CCA	GCG	GAC	3	G	A
TCGA-13-0793	PSD3	ENST00000286485	p.G375S	c.1123G>A	Verified	ATC	GGC	TCT	1	G	A
TCGA-24-1469	PSG3	ENST00000327495	p.D161N	c.481G>A	Verified	GAG	GAC	ATG	1	G	A
TCGA-30-1857	PSG3	ENST00000327495	p.G17E	c.50G>A	Unverified	AAG	GGG	CTC	2	G	A
TCGA-61-1737	PSG5	ENST00000342951	p.K310K	c.930G>A	Unverified	GGC	AAG	GAA	3	G	A
TCGA-24-0979	PSMA2	ENST00000223321	p.V87V	c.261G>A	Verified	CTT	GTG	CAC	3	G	A
TCGA-29-1768	PSMC1	ENST00000261303	p.?	c.58-1G>A	Unverified	AAG	GAC	AAG	1	G	A
TCGA-29-1690	PSMC5	ENST00000310144	p.V186V	c.558G>A	Unverified	GGA	GTG	CTG	3	G	A
TCGA-61-1900	PSMD7	ENST00000219313	p.E136K	c.406G>A	Unverified	ACA	GAA	GCG	1	G	A
TCGA-30-1856	PSME2	ENST00000216802	p.V153I	c.457G>A	Unverified	GCC	GTC	AAG	1	G	A
TCGA-25-1326	PTCD1	ENST00000292478	p.K580K	c.1740G>A	Unverified	AAG	AAG	TCC	3	G	A
TCGA-13-0807	PTCH1	ENST00000331920	p.V474I	c.1420G>A	Verified	GGC	GTC	CTG	1	G	A
TCGA-13-0807	PTCH1_ENST	ENST00000331920	p.V474I	c.1420G>A	Verified	GGC	GTC	CTG	1	G	A
TCGA-61-1914	PTCHD2	XM_052561.7	p.D359N	c.1075G>A	Unverified	gcc	gac	ttc	1	G	A
TCGA-24-1845	PTCHD3	ENST00000438700	p.G437G	c.1311G>A	Unverified	ATT	GGG	GTG	3	G	A
TCGA-04-1361	PTGS2	ENST00000367468	p.G283S	c.847G>A	Verified	CCT	GGT	CTG	1	G	A
TCGA-04-1361	PTGS2	ENST00000367468	p.G283S	c.847G>A	Verified	CCT	GGT	CTG	1	G	A
TCGA-29-1769	PTGS2	ENST00000367468	p.K559K	c.1677G>A	Unverified	GTG	AAG	GGC	3	G	A
TCGA-29-1769	PTGS2_ENST	ENST00000367468	p.K559K	c.1677G>A	Unverified	GTG	AAG	GGC	3	G	A
TCGA-23-1809	PTPN3	ENST00000394831	p.D215N	c.643G>A	Unverified	CTC	GAC	TTC	1	G	A
TCGA-61-1899	PTPRD	ENST00000381196	p.?	c.541+6G>A	Unverified	TCA	GAA	TCT	1	G	A
TCGA-61-1899	PTPRD_ENST	ENST00000346816	p.?	c.541+6G>A	Unverified	TCA	GTT	TCT	1	G	A
TCGA-61-1899	PTPRD_ENST	ENST00000355233	p.?	c.541+6G>A	Unverified	TCA	GAA	TCT	1	G	A
TCGA-61-1899	PTPRD_ENST	ENST00000381196	p.?	c.541+6G>A	Unverified	TCA	GAA	TCT	1	G	A
TCGA-29-1696	PTPRD_ENST	ENST00000381196	p.M764I	c.2292G>A	Unverified	CCC	ATG	CTG	3	G	A
TCGA-29-1696	PTPRD_ENST	ENST00000381196	p.M764I	c.2292G>A	Unverified	CCC	ATG	CTG	3	G	A
TCGA-13-1496	PTPRJ	ENST00000418331	p.G338S	c.1012G>A	Verified	CCT	GGC	ACC	1	G	A
TCGA-29-2427	PTPRN2	NM_002847.2	p.V558M	c.1672G>A	Verified	aaa	gtg	agc	1	G	A

TCGA-04-1649	PTPRN2	NM_002847.2	p.P822P	c.2466G>A	Unverified	gga	ccg	ctg	3	G	A
TCGA-29-1693	PTPRO	ENST00000281171	p.S276S	c.828G>A	Unverified	ccc	tcg	ggc	3	G	A
TCGA-61-1907	PTPRR	NM_002849.2	p.R414H	c.1241G>A	Unverified	ccg	cgt	cat	2	G	A
TCGA-29-1764	PTPRZ1	ENST00000393386	p.V805V	c.2415G>A	Unverified	GAT	GTG	TCA	3	G	A
TCGA-04-1362	PTPRZ1_ENS	ENST00000393386	p.D2178N	c.6532G>A	Verified	GAT	GAT	TAT	1	G	A
TCGA-29-1764	PTPRZ1_ENS	ENST00000393386	p.V805V	c.2415G>A	Unverified	GAT	GTG	TCA	3	G	A
TCGA-61-1895	Q6ZU04_HU	ENST00000342355	p.L322L	c.966G>A	Unverified	TGG	CTG	ATC	3	G	A
TCGA-61-1907	Q8N164_HU	ENST00000313544	p.V103I	c.307G>A	Unverified	TCA	GTT	TTT	1	G	A
TCGA-24-0979	Q8N8C9_HU	ENST00000316673	p.L371L	c.1113G>A	Verified	CAC	CTG	ATG	3	G	A
TCGA-04-1347	Q96HZ0_HU	ENST00000292140	p.A467A	c.1401G>A	Verified	ATG	GCG	GAG	3	G	A
TCGA-13-1488	Q9Y6V0-3	ENST00000333891	p.Q514Q	c.1542G>A	Verified	CTG	CAG	CCA	3	G	A
TCGA-29-1776	QRICH2	ENST00000262765	p.P1196P	c.3588G>A	Unverified	GCC	CCG	CAC	3	G	A
TCGA-24-1563	QSOX1	ENST00000367602	p.A559T	c.1675G>A	Verified	GTG	GCA	GCC	1	G	A
TCGA-29-2427	RAB10	ENST00000264710	p.D125N	c.373G>A	Verified	TGT	GAT	ATG	1	G	A
TCGA-29-1766	RAB11FIP1	ENST00000330843	p.P1273P	c.3819G>A	Unverified	ATC	CCG	ACT	3	G	A
TCGA-25-2391	RAB1B	ENST00000311481	p.A152T	c.454G>A	Unverified	agc	gcc	aag	1	G	A
TCGA-25-1313	RAB36	ENST00000263116	p.W90*	c.270G>A	Verified	aag	tgg	tac	3	G	A
TCGA-04-1343	RAB3C	ENST00000282878	p.E115K	c.343G>A	Verified	aat	gaa	gaa	1	G	A
TCGA-13-0761	RAB3D	ENST00000222120	p.G198D	c.593G>A	Verified	tca	ggc	agc	2	G	A
TCGA-13-0761	RAB3D	ENST00000222120	p.G198D	c.593G>A	Verified	tca	ggc	agc	2	G	A
TCGA-13-0883	RAB3GAP2	ENST00000358951	p.R1032H	c.3095G>A	Unverified	GCA	CGT	TTT	2	G	A
TCGA-09-2051	RAB40AL	ENST00000218249	p.K188K	c.564G>A	Unverified	AGC	AAG	GTA	3	G	A
TCGA-04-1338	RABEP1	ENST00000262477	p.V830V	c.2490G>A	Verified	CAG	GTG	CAG	3	G	A
TCGA-61-1725	RABGGTA	ENST00000399409	p.G118S	c.352G>A	Unverified	CTA	GGC	CGC	1	G	A
TCGA-61-1737	RAD54L	ENST00000442598	p.R319Q	c.956G>A	Unverified	AGC	CGG	CGG	2	G	A
TCGA-61-2094	RAET1L	ENST00000367341	p.G54S	c.160G>A	Unverified	CAA	GGC	CAG	1	G	A
TCGA-24-1470	RALGAPA1	ENST00000389698	p.G1700R	c.5098G>A	Verified	ACT	GGA	ATC	1	G	A
TCGA-13-1510	RANBP10	ENST00000317506	p.G156S	c.466G>A	Unverified	act	ggc	cag	1	G	A
TCGA-24-2288	RANBP3	ENST00000340578	p.G422D	c.1265G>A	Unverified	aga	ggc	cgg	2	G	A
TCGA-23-1124	RAP1GAP_E	ENST00000374757	p.V42V	c.126G>A	Verified	CAT	GTG	TGG	3	G	A
TCGA-29-1783	RAPGEF2	ENST00000264431	p.G890R	c.2668G>A	Unverified	GAC	GGG	CTG	1	G	A
TCGA-61-2009	RAPGEF5	NM_012294.2	p.R277Q	c.830G>A	Verified	aag	cga	gtg	2	G	A
TCGA-04-1651	RAPGEF5	NM_012294.2	p.Q405Q	c.1215G>A	Unverified	aac	cag	ttt	3	G	A
TCGA-04-1651	RAPGEF5	NM_012294.2	p.Q405Q	c.1215G>A	Unverified	aac	cag	ttt	3	G	A

TCGA-61-2009	RAPGEF5_EI	ENST00000344041	p.R563Q	c.1688G>A	Verified	AAG	CGA	GTG	2	G	A
TCGA-04-1651	RAPGEF5_EI	ENST00000344041	p.Q691Q	c.2073G>A	Unverified	AAC	CAG	TTT	3	G	A
TCGA-04-1651	RAPGEF5_EI	ENST00000344041	p.Q691Q	c.2073G>A	Unverified	AAC	CAG	TTT	3	G	A
TCGA-25-1326	RAPGEF6	NM_016340.2	p.R13K	c.38G>A	Unverified	aag	agg	acc	2	G	A
TCGA-20-0991	RAPGEFL1	ENST00000264644	p.E395E	c.1185G>A	Verified	atc	gag	aag	3	G	A
TCGA-09-2051	RARRES1	ENST00000237696	p.R122H	c.365G>A	Unverified	GGA	CGT	TTG	2	G	A
TCGA-24-1103	RASA2	ENST00000286364	p.R75H	c.224G>A	Verified	TAT	CGT	ACC	2	G	A
TCGA-23-1032	RASAL1	ENST00000261729	p.D642N	c.1924G>A	Unverified	CAG	GAC	GGC	1	G	A
TCGA-29-1693	RASAL1	ENST00000261729	p.R197Q	c.590G>A	Unverified	CTG	CGG	GTG	2	G	A
TCGA-23-1022	RASGEF1C	ENST00000361132	p.G293S	c.877G>A	Verified	ATC	GGC	AAC	1	G	A
TCGA-13-1481	RASSF2	ENST00000379400	p.R194H	c.581G>A	Verified	gtc	cgc	atc	2	G	A
TCGA-13-0885	RB1CC1	ENST00000025008	p.A683T	c.2047G>A	Verified	CCT	GCA	GTT	1	G	A
TCGA-13-0923	RBBP8	ENST00000327155	p.S696N	c.2087G>A	Unverified	gtt	agt	gaa	2	G	A
TCGA-24-0979	RBM12	ENST00000374104	p.D355N	c.1063G>A	Verified	CAA	GAT	ACA	1	G	A
TCGA-13-1481	RBM15	ENST00000369784	p.L976L	c.2928G>A	Verified	ACC	CTG	TTA	3	G	A
TCGA-61-1907	RBM26	ENST00000267229	p.Q66Q	c.198G>A	Unverified	ACA	CAG	ATA	3	G	A
TCGA-61-1907	RBM26_ENS	ENST00000327303	p.Q66Q	c.198G>A	Unverified	ACA	CAG	ATA	3	G	A
TCGA-13-0760	RBM4	ENST00000310092	p.A359T	c.1075G>A	Verified	CGG	GCG	CGG	1	G	A
TCGA-13-0807	RBM44	ENST00000316997	p.G221E	c.662G>A	Verified	TTA	GGA	AAT	2	G	A
TCGA-24-1431	RBPJL	ENST00000343694	p.A492T	c.1474G>A	Unverified	ccc	gcc	acc	1	G	A
TCGA-29-1775	RC3H1	ENST00000258349	p.Q407Q	c.1221G>A	Unverified	CAG	CAG	CCT	3	G	A
TCGA-61-1733	RCHY1	ENST00000324439	p.R54H	c.161G>A	Unverified	GAT	CGC	TTT	2	G	A
TCGA-61-1733	RCHY1	ENST00000324439	p.R54H	c.161G>A	Unverified	GAT	CGC	TTT	2	G	A
TCGA-61-1915	RCN2	ENST00000394885	p.G204E	c.611G>A	Unverified	GAT	GGA	TTT	2	G	A
TCGA-29-1775	RCOR3	ENST00000367005	p.Q342Q	c.1026G>A	Unverified	TTG	CAG	GAG	3	G	A
TCGA-13-0755	RDH5	ENST00000257895	p.E216K	c.646G>A	Verified	CTG	GAG	AGT	1	G	A
TCGA-04-1342	REEP2	ENST00000254901	p.V10M	c.28G>A	Unverified	CTG	GTG	GTG	1	G	A
TCGA-25-2392	RELN	ENST00000343529	p.A1276T	c.3826G>A	Verified	TCA	GCA	ATG	1	G	A
TCGA-30-1857	RELN	ENST00000343529	p.G106D	c.317G>A	Unverified	GGA	GGT	TCC	2	G	A
TCGA-23-1123	REP15	ENST00000310791	p.V19I	c.55G>A	Verified	CCC	GTC	GTC	1	G	A
TCGA-23-1123	REP15	ENST00000310791	p.V19I	c.55G>A	Verified	CCC	GTC	GTC	1	G	A
TCGA-61-1738	REPIN1	ENST00000397281	p.R165Q	c.494G>A	Unverified	CTG	CGG	CGG	2	G	A
TCGA-61-1738	REPIN1	ENST00000397281	p.R165Q	c.494G>A	Unverified	CTG	CGG	CGG	2	G	A
TCGA-61-1738	REPIN1_ENS	ENST00000489432	p.R222Q	c.665G>A	Unverified	CTG	CGG	CGG	2	G	A

TCGA-61-1738	REPIN1_ENS	ENST00000489432	p.R222Q	c.665G>A	Unverified	CTG	CGG	CGG	2	G	A
TCGA-24-2288	RERE	ENST00000400908	p.R358Q	c.1073G>A	Unverified	TCT	CGG	GAT	2	G	A
TCGA-04-1347	REV1	ENST00000258428	p.T331T	c.993G>A	Verified	TCT	ACG	TTT	3	G	A
TCGA-23-1110	RFX3	ENST00000358730	p.M84I	c.252G>A	Verified	CAG	ATG	TAC	3	G	A
TCGA-13-1498	RFX5	ENST00000368870	p.D610N	c.1828G>A	Verified	AAA	GAC	CCA	1	G	A
TCGA-61-1910	RGAG1	ENST00000465301	p.A1131A	c.3393G>A	Unverified	ACT	GCG	AAA	3	G	A
TCGA-13-0884	RGAG4	XM_291322.3	p.R315H	c.944G>A	Verified	att	cgc	aag	2	G	A
TCGA-13-0886	RGL3	XM_290867.2	p.S376S	c.1128G>A	Verified	agc	tcg	gag	3	G	A
TCGA-13-0887	RGL3_ENST(	ENST00000380456	p.W151*	c.452G>A	Verified	TCC	TGG	CTG	2	G	A
TCGA-04-1347	RGL3_ENST(	ENST00000380456	p.A131A	c.393G>A	Verified	ACA	GCG	GTA	3	G	A
TCGA-13-0886	RGL3_ENST(	ENST00000380456	p.S612S	c.1836G>A	Verified	AGC	TCG	GAG	3	G	A
TCGA-09-1674	RGPD2_ENS	ENST00000398146	p.V1700M	c.5098G>A	Unverified	AAC	GTG	GAA	1	G	A
TCGA-29-1711	RGPD4	ENST00000408999	p.D694N	c.2080G>A	Unverified	GAA	GAC	ATT	1	G	A
TCGA-29-1711	RGPD4	ENST00000408999	p.D694N	c.2080G>A	Unverified	GAA	GAC	ATT	1	G	A
TCGA-29-1698	RGPD4	ENST00000408999	p.K734K	c.2202G>A	Unverified	GTC	AAG	AAA	3	G	A
TCGA-13-0924	RGS18	ENST00000367460	p.R62H	c.185G>A	Verified	acc	cgc	tcc	2	G	A
TCGA-29-1768	RGS2	ENST00000235382	p.K102K	c.306G>A	Unverified	tta	aag	tcg	3	G	A
TCGA-24-1844	RGS4	ENST00000367909	p.E96K	c.286G>A	Unverified	tgt	gaa	gag	1	G	A
TCGA-24-1844	RGS4_ENST(	ENST00000421743	p.E193K	c.577G>A	Unverified	TGT	GAA	GAG	1	G	A
TCGA-23-1111	RGS7	ENST00000366565	p.E255K	c.763G>A	Unverified	ACA	GAA	GAT	1	G	A
TCGA-23-1111	RGS7_ENST(	ENST00000366565	p.E255K	c.763G>A	Unverified	ACA	GAA	GAT	1	G	A
TCGA-09-2044	RGS9	ENST00000262406	p.R569Q	c.1706G>A	Verified	AGC	CGG	CCC	2	G	A
TCGA-29-1693	RHBDF2	ENST00000313080	p.L684L	c.2052G>A	Unverified	GAC	CTG	GAG	3	G	A
TCGA-61-2012	RHBDL3	ENST00000269051	p.M270I	c.810G>A	Verified	GAC	ATG	ACC	3	G	A
TCGA-29-1695	RHOBTB1	ENST00000357917	p.V580I	c.1738G>A	Unverified	gcc	gtt	cag	1	G	A
TCGA-13-0890	RIMS1	ENST00000521978	p.P1157P	c.3471G>A	Verified	TCT	CCG	GAG	3	G	A
TCGA-29-1769	RIN2	NM_018993.2	p.R523R	c.1569G>A	Unverified	tcc	cgg	gac	3	G	A
TCGA-29-1769	RIN2_ENST0	ENST00000255006	p.R572R	c.1716G>A	Unverified	TCC	CGG	GAC	3	G	A
TCGA-04-1338	RIPK3	ENST00000216274	p.M211I	c.633G>A	Verified	CTA	ATG	TGG	3	G	A
TCGA-23-1118	RIPK4	ENST00000332512	p.L641L	c.1923G>A	Verified	CCC	CTG	CAC	3	G	A
TCGA-13-1481	RIT2	ENST00000326695	p.D55N	c.163G>A	Verified	gaa	gat	gct	1	G	A
TCGA-13-0924	RNASE12	ENST00000382999	p.E112K	c.334G>A	Verified	ATT	GAA	GGC	1	G	A
TCGA-23-1022	RNF112	ENST00000299604	p.D285N	c.853G>A	Verified	AAG	GAT	ACA	1	G	A
TCGA-13-0904	RNF115	ENST00000369291	p.G151E	c.452G>A	Verified	GCA	GGA	TTC	2	G	A

TCGA-61-1906	RNF122	ENST00000256257	p.R71Q	c.212G>A	Unverified	gag	cga	tac	2	G	A
TCGA-61-1906	RNF122	ENST00000256257	p.R71Q	c.212G>A	Unverified	gag	cga	tac	2	G	A
TCGA-13-1488	RNF149	ENST00000295317	p.M162I	c.486G>A	Verified	ATT	ATG	ATT	3	G	A
TCGA-61-1733	RNF165	ENST00000269439	p.V252M	c.754G>A	Unverified	AAT	GTG	ACT	1	G	A
TCGA-61-1733	RNF165	ENST00000269439	p.V252M	c.754G>A	Unverified	AAT	GTG	ACT	1	G	A
TCGA-24-2267	RNF20	ENST00000389120	p.E215E	c.645G>A	Verified	gtg	gag	gaa	3	G	A
TCGA-24-1604	RNF213	ENST00000336301	p.S3092N	c.9275G>A	Verified	TAC	AGC	GAT	2	G	A
TCGA-23-2078	RNF216	ENST00000389902	p.D899N	c.2695G>A	Verified	tat	gac	ttc	1	G	A
TCGA-24-1563	RNF26	ENST00000311413	p.K388K	c.1164G>A	Verified	agc	aag	aca	3	G	A
TCGA-04-1347	RNPS1	ENST00000397086	p.E217K	c.649G>A	Verified	GCC	GAG	AAG	1	G	A
TCGA-13-0885	ROM1	ENST00000278833	p.R186H	c.557G>A	Verified	AGC	CGT	TAC	2	G	A
TCGA-13-0903	ROR1	ENST00000371079	p.G183S	c.547G>A	Verified	ATT	GGC	AAC	1	G	A
TCGA-29-1784	RP1	ENST00000220676	p.G44E	c.131G>A	Unverified	AGC	GGA	GAC	2	G	A
TCGA-24-0979	RP1L1	ENST00000382483	p.G1809R	c.5425G>A	Verified	TCT	GGG	CAT	1	G	A
TCGA-13-0884	RPH3A	ENST00000389385	p.R452Q	c.1355G>A	Verified	CTG	CGG	AAT	2	G	A
TCGA-13-0760	RPIA	ENST00000283646	p.G143D	c.428G>A	Verified	TAT	GGC	TTG	2	G	A
TCGA-24-1563	RPL10L	ENST00000298283	p.A68T	c.202G>A	Verified	GCC	GCC	CGT	1	G	A
TCGA-13-0760	RPL35	ENST00000348462	p.R94R	c.282G>A	Verified	CGC	CGG	CTC	3	G	A
TCGA-24-1417	RPP40	ENST00000380051	p.W167*	c.501G>A	Unverified	TCT	TGG	TCT	3	G	A
TCGA-29-1769	RPS3	ENST00000531188	p.R40Q	c.119G>A	Unverified	GTG	CGA	GTT	2	G	A
TCGA-13-0920	RPS3A	ENST00000274065	p.E175E	c.525G>A	Verified	CGA	GAG	GTG	3	G	A
TCGA-29-1783	RPS6KA2	ENST00000265678	p.A591A	c.1773G>A	Unverified	GCG	GCG	TGT	3	G	A
TCGA-29-1783	RPS6KA2_EN	ENST00000503859	p.A599A	c.1797G>A	Unverified	GCG	GCG	TGT	3	G	A
TCGA-24-1844	RPS6KA5	ENST00000261991	p.S240N	c.719G>A	Unverified	TGG	AGT	TTG	2	G	A
TCGA-24-1844	RPS6KA5_EN	ENST00000261991	p.S240N	c.719G>A	Unverified	TGG	AGT	TTG	2	G	A
TCGA-61-1900	RPS6KB1	ENST00000225577	p.E45E	c.135G>A	Unverified	ctg	gag	gag	3	G	A
TCGA-24-1423	RPS6KC1	ENST00000366960	p.G410S	c.1228G>A	Verified	ggt	ggc	aaa	1	G	A
TCGA-04-1652	RPS9	ENST00000391752	p.V149I	c.445G>A	Unverified	ATT	GTC	CGC	1	G	A
TCGA-04-1652	RPS9	ENST00000391752	p.V149I	c.445G>A	Unverified	ATT	GTC	CGC	1	G	A
TCGA-04-1649	RPTOR	ENST00000306801	p.V1273I	c.3817G>A	Unverified	TCC	GTC	AAT	1	G	A
TCGA-13-0916	RRN3	ENST00000198767	p.E288K	c.862G>A	Verified	GAT	GAA	GAT	1	G	A
TCGA-20-1683	RRP12	ENST00000370992	p.V447M	c.1339G>A	Unverified	TGC	GTG	GCT	1	G	A
TCGA-61-1907	RRP1B	ENST00000340648	p.R323H	c.968G>A	Unverified	AAG	CGC	CTC	2	G	A
TCGA-29-1690	RSPH10B	ENST00000337579	p.R92H	c.275G>A	Unverified	GTT	CGT	GGG	2	G	A

TCGA-13-0714	RSPH10B2	ENST00000297186	p.A679T	c.2035G>A	Unverified	AGC	GCG	GTC	1	G	A
TCGA-29-1690	RSPH10B2	ENST00000297186	p.R92H	c.275G>A	Unverified	GTT	CGT	GGG	2	G	A
TCGA-24-2290	RSPH3	ENST00000252655	p.E390K	c.1168G>A	Verified	AAC	GAG	ACA	1	G	A
TCGA-24-2290	RSPH3	ENST00000252655	p.E390K	c.1168G>A	Verified	AAC	GAG	ACA	1	G	A
TCGA-24-1431	RTN2	ENST00000245923	p.V377M	c.1129G>A	Verified	ATC	GTG	TCC	1	G	A
TCGA-23-2078	RTP1	ENST00000312295	p.S226S	c.678G>A	Verified	AAG	TCG	CAG	3	G	A
TCGA-09-1674	RUNDC1	ENST00000361677	p.M470I	c.1410G>A	Unverified	GCC	ATG	CAC	3	G	A
TCGA-24-0979	RYR1	ENST00000359596	p.A3541T	c.10621G>A	Verified	TAC	GCC	CTG	1	G	A
TCGA-13-0885	RYR2	ENST00000366574	p.V613I	c.1837G>A	Verified	GAT	GTC	TTG	1	G	A
TCGA-20-1686	RYR3	ENST00000389232	p.R3485Q	c.10454G>A	Unverified	AAA	CGG	GCA	2	G	A
TCGA-61-2109	RYR3	ENST00000389232	p.L397L	c.1191G>A	Unverified	ACA	CTG	CAG	3	G	A
TCGA-25-1326	S1PR5	ENST00000333430	p.D374N	c.1120G>A	Unverified	CTG	GAC	ACC	1	G	A
TCGA-24-2280	SAGE1	ENST00000324447	p.R451Q	c.1352G>A	Unverified	caa	cga	aaa	2	G	A
TCGA-13-0919	SALL2	ENST00000327430	p.L961L	c.2883G>A	Verified	CTC	CTG	GCA	3	G	A
TCGA-04-1649	SALL3	ENST00000537592	p.R1285H	c.3854G>A	Unverified	AGC	CGC	CCA	2	G	A
TCGA-09-2051	SALL4	ENST00000217086	p.E771K	c.2311G>A	Unverified	CAG	GAG	TAT	1	G	A
TCGA-23-1111	SAMD4A	ENST00000357634	p.P413P	c.1239G>A	Unverified	ACT	CCG	ATC	3	G	A
TCGA-24-1845	SAMD9L	ENST00000318238	p.M892I	c.2676G>A	Unverified	ATC	ATG	AAA	3	G	A
TCGA-24-1435	SAMD9L	ENST00000318238	p.L1310L	c.3930G>A	Verified	CAT	TTG	GAT	3	G	A
TCGA-23-1031	SAMM50	ENST00000350028	p.E234K	c.700G>A	Unverified	CGA	GAA	CTG	1	G	A
TCGA-13-1481	SAMSN1	ENST00000400566	p.G189E	c.566G>A	Verified	AAA	GGA	GAC	2	G	A
TCGA-24-1604	SAMSN1	ENST00000400566	p.G54E	c.161G>A	Verified	AGT	GGA	GAA	2	G	A
TCGA-61-1915	SAP130	ENST00000259235	p.G372S	c.1114G>A	Unverified	ACT	GGC	ACG	1	G	A
TCGA-61-1915	SAP130_ENS	ENST00000357702	p.G372S	c.1114G>A	Unverified	ACT	GGC	ACG	1	G	A
TCGA-24-1847	SARS	ENST00000234677	p.R45Q	c.134G>A	Unverified	CGA	CGA	TGT	2	G	A
TCGA-20-1685	SARS	ENST00000234677	p.V84V	c.252G>A	Unverified	AAT	GTG	CTG	3	G	A
TCGA-24-2035	SARS	ENST00000234677	p.T399T	c.1197G>A	Unverified	TGC	ACG	GAT	3	G	A
TCGA-24-1849	SATB1	ENST00000338745	p.E654K	c.1960G>A	Unverified	gtg	gaa	gcc	1	G	A
TCGA-29-1763	SATB1	ENST00000338745	p.R410Q	c.1229G>A	Unverified	ctc	cga	aag	2	G	A
TCGA-24-1849	SATB1	ENST00000338745	p.Q694Q	c.2082G>A	Unverified	ttt	cag	aac	3	G	A
TCGA-61-2109	SBF2	ENST00000256190	p.E1586E	c.4758G>A	Unverified	GAA	GAG	ACC	3	G	A
TCGA-23-1114	SCAI	ENST00000373549	p.G115R	c.343G>A	Unverified	TTT	GGA	AGA	1	G	A
TCGA-24-1846	SCAND3	ENST00000452236	p.V112M	c.334G>A	Unverified	TGG	GTG	CGG	1	G	A
TCGA-29-1783	SCAP	ENST00000265565	p.G1218S	c.3652G>A	Unverified	GGC	GGC	CAG	1	G	A

TCGA-13-0920	SCD5	ENST00000319540	p.W97*	c.291G>A	Verified	TTG	TGG	AGC	3	G	A
TCGA-13-0795	SCGN	ENST00000377961	p.R135Q	c.404G>A	Verified	CTC	CGA	GAC	2	G	A
TCGA-13-0760	SCIN	ENST00000297029	p.A289T	c.865G>A	Verified	GGG	GCT	GCC	1	G	A
TCGA-04-1655	SCIN	ENST00000297029	p.W440*	c.1319G>A	Unverified	ACG	TGG	CAA	2	G	A
TCGA-04-1655	SCIN_ENST0	ENST00000297029	p.W440*	c.1319G>A	Unverified	ACG	TGG	CAA	2	G	A
TCGA-04-1356	SCML1	ENST00000380045	p.R25H	c.74G>A	Verified	agg	cgt	gag	2	G	A
TCGA-29-1703	SCN11A	ENST00000302328	p.T1410T	c.4230G>A	Unverified	TTT	ACG	TTA	3	G	A
TCGA-25-2400	SCN1B	ENST00000262631	p.S137N	c.410G>A	Verified	ACC	AGC	GTC	2	G	A
TCGA-24-1417	SCN2A	ENST00000375427	p.Q452Q	c.1356G>A	Verified	GAA	CAG	TTG	3	G	A
TCGA-13-1498	SCN3B	ENST00000299333	p.E188K	c.562G>A	Verified	GCC	GAA	GAG	1	G	A
TCGA-13-0714	SCN4A	ENST00000435607	p.E764K	c.2290G>A	Unverified	ATC	GAG	ACC	1	G	A
TCGA-29-1766	SCN5A	ENST00000333535	p.R53Q	c.158G>A	Unverified	CCC	CGG	CCC	2	G	A
TCGA-29-1766	SCN5A_ENS	ENST00000333535_v68	p.R53Q	c.158G>A	Unverified	CCC	CGG	CCC	2	G	A
TCGA-29-1766	SCN5A_ENS	ENST00000413689	p.R53Q	c.158G>A	Unverified	CCC	CGG	CCC	2	G	A
TCGA-04-1542	SCN8A	ENST00000354534	p.V764I	c.2290G>A	Verified	ATC	GTC	CTG	1	G	A
TCGA-61-1738	SCN8A	ENST00000354534	p.G242S	c.724G>A	Unverified	GTG	GGT	GCC	1	G	A
TCGA-61-1738	SCN8A	ENST00000354534	p.G242S	c.724G>A	Unverified	GTG	GGT	GCC	1	G	A
TCGA-23-1022	SCN8A	ENST00000354534	p.R606Q	c.1817G>A	Verified	ATC	CGG	GCC	2	G	A
TCGA-23-1122	SCN8A	ENST00000354534	p.L1845L	c.5535G>A	Verified	TGC	TTG	GAC	3	G	A
TCGA-61-1738	SCN8A_ENS	ENST00000354534	p.G242S	c.724G>A	Unverified	GTG	GGT	GCC	1	G	A
TCGA-61-1738	SCN8A_ENS	ENST00000354534	p.G242S	c.724G>A	Unverified	GTG	GGT	GCC	1	G	A
TCGA-24-1463	SCNN1B	ENST00000343070	p.V434M	c.1300G>A	Verified	AGC	GTG	GCG	1	G	A
TCGA-04-1530	SCUBE1	ENST00000360835	p.R884H	c.2651G>A	Unverified	TCC	CGC	TCC	2	G	A
TCGA-23-1022	SCYL2	ENST00000360820	p.G355R	c.1063G>A	Verified	AAA	GGA	CTG	1	G	A
TCGA-61-2012	SCYL2	ENST00000360820	p.R505H	c.1514G>A	Verified	GTT	CGT	GTA	2	G	A
TCGA-23-1116	SDC2	ENST00000302190	p.E125K	c.373G>A	Verified	GCC	GAA	GAG	1	G	A
TCGA-13-0887	SDK2	ENST00000316893	p.A814T	c.2440G>A	Verified	AAC	GCC	CCC	1	G	A
TCGA-29-1691	SDK2	ENST00000316893	p.P1406P	c.4218G>A	Unverified	AGG	CCG	ATG	3	G	A
TCGA-04-1362	SEC11A	ENST00000268220	p.R178H	c.533G>A	Verified	CAT	CGT	GAG	2	G	A
TCGA-61-2009	SEC13	ENST00000350697	p.R43H	c.128G>A	Verified	GTG	CGC	AAT	2	G	A
TCGA-23-1124	SEC14L1	ENST00000436233	p.V530I	c.1588G>A	Verified	AGC	GTC	TTC	1	G	A
TCGA-61-1998	SEC23IP	ENST00000369075	p.G770R	c.2308G>A	Verified	GCC	GGA	CAG	1	G	A
TCGA-61-2008	SEC24B	ENST00000399100	p.L476L	c.1428G>A	Verified	GGA	TTG	AGT	3	G	A
TCGA-04-1347	SEL1L	ENST00000336735	p.G304S	c.910G>A	Verified	gct	ggc	atc	1	G	A

TCGA-20-1687	SEMA5A	ENST00000382496	p.A103T	c.307G>A	Unverified	AAG	GCC	TGT	1	G	A
TCGA-13-0904	SEMA5A	ENST00000382496	p.Q166Q	c.498G>A	Verified	CCC	CAG	CAC	3	G	A
TCGA-13-0900	SEMA5A	ENST00000382496	p.P740P	c.2220G>A	Unverified	GAT	CCG	AAT	3	G	A
TCGA-23-1117	SEMA6A	ENST00000343348	p.A175T	c.523G>A	Verified	GTT	GCA	CTG	1	G	A
TCGA-23-1117	SEMA6A	ENST00000343348	p.A175T	c.523G>A	Verified	GTT	GCA	CTG	1	G	A
TCGA-20-1683	SENP2	ENST00000296257	p.R199H	c.596G>A	Unverified	AGG	CGT	CCC	2	G	A
TCGA-20-1683	SENP2_ENST	ENST00000296257	p.R199H	c.596G>A	Unverified	AGG	CGT	CCC	2	G	A
TCGA-13-0885	SENP3	NM_015670.3	p.E420K	c.1258G>A	Verified	cct	gaa	aag	1	G	A
TCGA-61-1914	SENP7	NM_020654.2	p.R95K	c.284G>A	Unverified	gaa	agg	ata	2	G	A
TCGA-61-1914	SENP7_ENST	ENST00000394095	p.R95K	c.284G>A	Unverified	GAA	AGG	CAA	2	G	A
TCGA-29-1699	SERBP1	ENST00000370995	p.G43G	c.129G>A	Unverified	GGC	GGG	GGC	3	G	A
TCGA-04-1356	SERPINB1	ENST00000380739	p.E268K	c.802G>A	Verified	att	gaa	gtt	1	G	A
TCGA-13-0761	SERPINB1	ENST00000380739	p.V229I	c.685G>A	Unverified	atg	gtc	atc	1	G	A
TCGA-13-0761	SERPINB1	ENST00000380739	p.V229I	c.685G>A	Unverified	atg	gtc	atc	1	G	A
TCGA-61-2113	SERPINB3	ENST00000283752	p.T112T	c.336G>A	Verified	aaa	acg	tat	3	G	A
TCGA-61-2095	SERPINB4	ENST00000341074	p.A104T	c.310G>A	Unverified	atc	gcc	aac	1	G	A
TCGA-61-1907	SERPINB5	ENST00000382771	p.D76N	c.226G>A	Unverified	TCG	GAT	GTA	1	G	A
TCGA-29-1777	SERPINE2	ENST00000258405	p.G330E	c.989G>A	Unverified	aca	ggg	tca	2	G	A
TCGA-29-1777	SERPINE2_E	ENST00000447280	p.R341K	c.1022G>A	Unverified	ACA	AGG	TCA	2	G	A
TCGA-25-1318	SERTAD4	ENST00000367012	p.R63K	c.188G>A	Verified	TAC	AGG	GGA	2	G	A
TCGA-25-1318	SERTAD4	ENST00000367012	p.R63K	c.188G>A	Verified	TAC	AGG	GGA	2	G	A
TCGA-24-1474	SESTD1	ENST00000428443	p.E240K	c.718G>A	Verified	GAT	GAA	CTT	1	G	A
TCGA-04-1530	SETD4	ENST00000399215	p.R90Q	c.269G>A	Verified	ATT	CGA	AGC	2	G	A
TCGA-13-0791	SETD5	ENST00000302463	p.Q960Q	c.2880G>A	Unverified	CCC	CAG	AAC	3	G	A
TCGA-04-1361	SETD5_ENST	ENST00000402198	p.S38N	c.113G>A	Verified	AAG	AGC	GTG	2	G	A
TCGA-04-1361	SETD5_ENST	ENST00000402198	p.S38N	c.113G>A	Verified	AAG	AGC	GTG	2	G	A
TCGA-13-0791	SETD5_ENST	ENST00000402198	p.Q1058Q	c.3174G>A	Unverified	CCC	CAG	AAC	3	G	A
TCGA-13-0795	SETD6	ENST00000310682	p.G328E	c.983G>A	Verified	GAG	GGA	GCC	2	G	A
TCGA-04-1343	SEZ6L	ENST00000248933	p.?	c.2212+1G>A	Verified	ATA	GAG	GTA	1	G	A
TCGA-29-1770	SEZ6L	ENST00000248933	p.R108H	c.323G>A	Unverified	GCC	CGC	CCC	2	G	A
TCGA-29-1690	SEZ6L2	ENST00000308713	p.R386H	c.1157G>A	Unverified	GGG	CGC	CGG	2	G	A
TCGA-29-1690	SEZ6L2_ENS	ENST00000350527	p.R316H	c.947G>A	Unverified	GGG	CGC	CGG	2	G	A
TCGA-29-1690	SFXN2	ENST00000369893	p.E40E	c.120G>A	Unverified	TCT	GAG	CGG	3	G	A
TCGA-29-1768	SG269_HUN	ENST00000312493	p.K1299K	c.3897G>A	Unverified	TTG	AAG	AAA	3	G	A

TCGA-29-1785	SGCD	ENST00000337851	p.R11Q	c.32G>A	Unverified	CAC	CGG	AGC	2	G	A
TCGA-29-1785	SGCD	ENST00000337851	p.R11Q	c.32G>A	Unverified	CAC	CGG	AGC	2	G	A
TCGA-29-1695	SGEF	ENST00000356448	p.E229K	c.685G>A	Unverified	CTC	GAG	AAT	1	G	A
TCGA-25-2401	SGIP1	ENST00000371037	p.K755K	c.2265G>A	Unverified	TGG	AAG	ATT	3	G	A
TCGA-29-1768	SgK269	ENST00000312493	p.K1299K	c.3897G>A	Unverified	TTG	AAG	AAA	3	G	A
TCGA-61-2095	SGPP2	ENST00000321276	p.V81M	c.241G>A	Unverified	GTC	GTG	AAG	1	G	A
TCGA-13-0807	SGSM3	ENST00000248929	p.E304K	c.910G>A	Verified	TAC	GAG	GGC	1	G	A
TCGA-30-1855	SH2D6	ENST00000340326	p.S67S	c.201G>A	Unverified	tac	tcg	ggg	3	G	A
TCGA-13-1497	SH3BP4	ENST00000344528	p.V466V	c.1398G>A	Verified	ACC	GTG	TGG	3	G	A
TCGA-24-0975	SH3BP4	ENST00000344528	p.Q535Q	c.1605G>A	Unverified	CCC	CAG	GAT	3	G	A
TCGA-29-1702	SH3BP4	ENST00000344528	p.E862E	c.2586G>A	Unverified	GCT	GAG	AAG	3	G	A
TCGA-24-2280	SH3GL3	ENST00000324537	p.V351M	c.1051G>A	Unverified	ATC	GTG	CCT	1	G	A
TCGA-09-1674	SH3PXD2A	ENST00000355946	p.A822T	c.2464G>A	Unverified	agc	gcc	tac	1	G	A
TCGA-29-1702	SH3RF3	ENST00000309415	p.C707Y	c.2120G>A	Unverified	GGA	TGC	AAA	2	G	A
TCGA-23-1120	SH3TC2	ENST00000515425	p.E1232K	c.3694G>A	Verified	ACT	GAG	TAC	1	G	A
TCGA-13-0761	SIAH2	ENST00000312960	p.G286S	c.856G>A	Verified	gac	ggt	gtg	1	G	A
TCGA-13-0761	SIAH2	ENST00000312960	p.G286S	c.856G>A	Verified	gac	ggt	gtg	1	G	A
TCGA-04-1638	SIGLEC14	ENST00000360844	p.T113T	c.339G>A	Unverified	GAC	ACG	GGA	3	G	A
TCGA-23-1117	SIPA1L3	ENST00000222345	p.G869G	c.2607G>A	Verified	GCA	GGG	GCC	3	G	A
TCGA-23-1117	SIPA1L3	ENST00000222345	p.G869G	c.2607G>A	Verified	GCA	GGG	GCC	3	G	A
TCGA-13-1512	SIRPA	ENST00000356025	p.V302I	c.904G>A	Verified	acc	gtt	aca	1	G	A
TCGA-04-1530	SIRPD	ENST00000381623	p.R72Q	c.215G>A	Unverified	AAC	CGG	AAA	2	G	A
TCGA-23-1029	SIRT3	ENST00000382743	p.E390K	c.1168G>A	Unverified	cgg	gaa	act	1	G	A
TCGA-24-1417	SKA1	ENST00000285116	p.R191K	c.572G>A	Unverified	ACC	AGA	AAT	2	G	A
TCGA-13-1498	SLC10A2	ENST00000245312	p.W118*	c.353G>A	Verified	TAT	TGG	GTC	2	G	A
TCGA-13-0760	SLC10A3	ENST00000263512	p.D280N	c.838G>A	Verified	ggg	gac	gtc	1	G	A
TCGA-25-2393	SLC11A1	ENST00000233202	p.D429N	c.1285G>A	Verified	AAT	GAT	CTG	1	G	A
TCGA-24-1846	SLC12A3	ENST00000438926	p.C645Y	c.1934G>A	Unverified	CAG	TGC	CTG	2	G	A
TCGA-23-1116	SLC12A4	ENST00000316341	p.R492R	c.1476G>A	Unverified	AGC	AGG	AAC	3	G	A
TCGA-29-1761	SLC14A2	ENST00000255226	p.A730T	c.2188G>A	Unverified	TCC	GCC	ATG	1	G	A
TCGA-23-1118	SLC14A2	ENST00000255226	p.P885P	c.2655G>A	Verified	CTC	CCG	CTC	3	G	A
TCGA-13-0916	SLC16A2	ENST00000276033	p.E582K	c.1744G>A	Verified	aaa	gag	cag	1	G	A
TCGA-24-1435	SLC16A4	ENST00000369779	p.L157L	c.471G>A	Verified	GGA	CTG	ACT	3	G	A
TCGA-04-1530	SLC16A9	ENST00000395347	p.?	c.1351+1G>A	Unverified	GTT	GGT	TGG	1	G	A

TCGA-61-1907	SLC20A2	ENST00000342228	p.G560G	c.1680G>A	Unverified	ATG	GGG	AAG	3	G	A
TCGA-61-1907	SLC25A20	ENST00000319017	p.R37Q	c.110G>A	Unverified	GTC	CGA	CTG	2	G	A
TCGA-20-1683	SLC25A23	ENST00000301454	p.R139H	c.416G>A	Unverified	TGG	CGC	GAC	2	G	A
TCGA-29-1777	SLC25A39	ENST00000225308	p.G72S	c.214G>A	Unverified	AAT	GGT	GTC	1	G	A
TCGA-24-1104	SLC25A4	ENST00000281456	p.R188K	c.563G>A	Verified	TAT	AGA	GCT	2	G	A
TCGA-29-1761	SLC25A47	ENST00000361529	p.R267Q	c.800G>A	Unverified	GTT	CGA	GAG	2	G	A
TCGA-23-1118	SLC25A5	ENST00000317881	p.G158S	c.472G>A	Verified	CTC	GGT	GAC	1	G	A
TCGA-61-1904	SLC26A3	ENST00000340010	p.V340I	c.1018G>A	Unverified	ACC	GTA	GGA	1	G	A
TCGA-13-1509	SLC26A8	ENST00000355574	p.G964S	c.2890G>A	Verified	GAG	GGC	AAC	1	G	A
TCGA-04-1362	SLC28A2	ENST00000347644	p.S64N	c.191G>A	Verified	TTC	AGC	AAA	2	G	A
TCGA-13-0795	SLC2A2	ENST00000314251	p.V197I	c.589G>A	Verified	ATC	GTC	ACG	1	G	A
TCGA-04-1530	SLC2A3	ENST00000075120	p.S363N	c.1088G>A	Verified	ATG	AGC	TTT	2	G	A
TCGA-23-1022	SLC2A5	ENST00000377424	p.V358M	c.1072G>A	Verified	TGC	GTG	CTC	1	G	A
TCGA-13-0714	SLC34A1	ENST00000324417	p.A263T	c.787G>A	Verified	GAT	GCT	CCT	1	G	A
TCGA-13-0920	SLC35B2	ENST00000393812	p.L11L	c.33G>A	Verified	GTG	CTG	GCT	3	G	A
TCGA-13-1488	SLC35F3	ENST00000366618	p.V139M	c.415G>A	Verified	GGC	GTG	GCG	1	G	A
TCGA-61-1740	SLC35F3	ENST00000366618	p.A106A	c.318G>A	Unverified	CCG	GCG	GAG	3	G	A
TCGA-13-1509	SLC37A3	ENST00000326232	p.V23M	c.67G>A	Verified	GTA	GTG	TTC	1	G	A
TCGA-13-0883	SLC38A1	ENST00000398637	p.S164N	c.491G>A	Verified	CTG	AGC	TAC	2	G	A
TCGA-61-2009	SLC38A4	ENST00000266579	p.G73R	c.217G>A	Verified	CCC	GGA	ACC	1	G	A
TCGA-29-1777	SLC38A8	ENST00000299709	p.W47*	c.141G>A	Unverified	CCC	TGG	GCC	3	G	A
TCGA-30-1714	SLC43A3	ENST00000395123	p.G77R	c.229G>A	Unverified	CTG	GGG	TCC	1	G	A
TCGA-23-2078	SLC45A1	ENST00000289877	p.G349S	c.1045G>A	Unverified	TAC	GGC	AGC	1	G	A
TCGA-61-2012	SLC4A3	ENST00000273063	p.R370H	c.1109G>A	Verified	GCC	CGC	TGG	2	G	A
TCGA-13-0919	SLC4A4	ENST00000340595	p.W781*	c.2343G>A	Verified	TTT	TGG	GTG	3	G	A
TCGA-61-1907	SLC4A8	ENST00000319957	p.W652*	c.1956G>A	Unverified	TAC	TGG	AAG	3	G	A
TCGA-61-1907	SLC4A8_ENS	ENST00000394856	p.W599*	c.1797G>A	Unverified	TAC	TGG	AAG	3	G	A
TCGA-61-1907	SLC4A8_ENS	ENST00000453097	p.W652*	c.1956G>A	Unverified	TAC	TGG	AAG	3	G	A
TCGA-23-1021	SLC50A1	ENST00000368404	p.W175*	c.524G>A	Verified	TCC	TGG	TGC	2	G	A
TCGA-25-1318	SLC5A10	ENST00000395647	p.G363S	c.1087G>A	Unverified	GTC	GGC	TGC	1	G	A
TCGA-25-1318	SLC5A10	ENST00000395647	p.G363S	c.1087G>A	Unverified	GTC	GGC	TGC	1	G	A
TCGA-13-1497	SLC5A12	ENST00000396005	p.R530K	c.1589G>A	Unverified	CAA	AGA	GGT	2	G	A
TCGA-20-0991	SLC5A2	ENST00000330498	p.V346M	c.1036G>A	Verified	TGC	GTG	GTG	1	G	A
TCGA-29-1691	SLC5A6	ENST00000310574	p.S15S	c.45G>A	Unverified	ACC	TCG	GGC	3	G	A

TCGA-25-1326	SLC5A7	ENST00000264047	p.V378I	c.1132G>A	Verified	ATC	GTT	TGG	1	G	A
TCGA-04-1338	SLC6A15	ENST00000266682	p.A30T	c.88G>A	Verified	GCA	GCT	GAT	1	G	A
TCGA-29-1764	SLC6A3	ENST00000270349	p.A161T	c.481G>A	Unverified	ATC	GCC	TGG	1	G	A
TCGA-09-1665	SLC6A3	ENST00000270349	p.V604V	c.1812G>A	Verified	CTG	GTG	GAC	3	G	A
TCGA-61-1915	SLC6A6	ENST00000360861	p.A270A	c.810G>A	Unverified	GGC	GCG	GGC	3	G	A
TCGA-24-1845	SLC6A9	ENST00000372310	p.T246T	c.738G>A	Unverified	TTC	ACG	GCC	3	G	A
TCGA-24-1845	SLC6A9_ENS	ENST00000360584	p.T319T	c.957G>A	Unverified	TTC	ACG	GCC	3	G	A
TCGA-61-2095	SLC7A10	ENST00000253188	p.C398Y	c.1193G>A	Unverified	CTC	TGC	TAC	2	G	A
TCGA-24-2262	SLC7A8	ENST00000316902	p.E228K	c.682G>A	Verified	CAG	GAA	CCT	1	G	A
TCGA-29-1699	SLC7A8	ENST00000316902	p.R339Q	c.1016G>A	Unverified	TCT	CGG	CTG	2	G	A
TCGA-61-1906	SLC8A1	ENST00000403092	p.V156M	c.466G>A	Unverified	GAA	GTG	TGT	1	G	A
TCGA-61-1906	SLC8A1	ENST00000403092	p.V156M	c.466G>A	Unverified	GAA	GTG	TGT	1	G	A
TCGA-61-2012	SLC8A2	ENST00000236877	p.T730T	c.2190G>A	Verified	CTG	ACG	GTG	3	G	A
TCGA-24-1474	SLC9A10	ENST00000305815	p.A70T	c.208G>A	Verified	AAC	GCC	ATA	1	G	A
TCGA-61-1998	SLC9A10	ENST00000305815	p.A1004T	c.3010G>A	Verified	CTC	GCT	ATT	1	G	A
TCGA-04-1530	SLC9A5	ENST00000299798	p.A183A	c.549G>A	Unverified	TCG	GCG	GTG	3	G	A
TCGA-04-1542	SLCO1C1	ENST00000266509	p.C480Y	c.1439G>A	Verified	AGA	TGC	AAA	2	G	A
TCGA-29-1691	SLCO1C1	ENST00000266509	p.C246Y	c.737G>A	Unverified	TTA	TGT	GCC	2	G	A
TCGA-29-1691	SLCO1C1_EN	ENST00000381552	p.C246Y	c.737G>A	Unverified	TTA	TGT	GCC	2	G	A
TCGA-04-1652	SLCO3A1	ENST00000318445	p.M297I	c.891G>A	Unverified	GCC	ATG	GAA	3	G	A
TCGA-04-1652	SLCO3A1	ENST00000318445	p.M297I	c.891G>A	Unverified	GCC	ATG	GAA	3	G	A
TCGA-13-0923	SLCO6A1	ENST00000379807	p.G484G	c.1452G>A	Verified	GCT	GGG	ATC	3	G	A
TCGA-13-0807	SLFN12L	ENST00000449046	p.T181T	c.543G>A	Verified	GTA	ACG	TCT	3	G	A
TCGA-13-0760	SLITRK2	ENST00000447897	p.S135N	c.404G>A	Verified	gag	agc	ctg	2	G	A
TCGA-24-1843	SLITRK4	ENST00000381779	p.K381K	c.1143G>A	Unverified	aag	aag	ctg	3	G	A
TCGA-30-1891	SMAP2	ENST00000372718	p.V161V	c.483G>A	Unverified	AAG	GTG	AAA	3	G	A
TCGA-36-1577	SMARCA1	NM_003069.2	p.R525H	c.1574G>A	Verified	tgg	cgt	ggt	2	G	A
TCGA-20-1685	SMARCA2	ENST00000349721	p.R261K	c.782G>A	Unverified	AAC	AGA	CCA	2	G	A
TCGA-29-1781	SMARCA2	ENST00000349721	p.Q676Q	c.2028G>A	Unverified	AAG	CAG	ATC	3	G	A
TCGA-20-1685	SMARCA2_E	ENST00000349721	p.R261K	c.782G>A	Unverified	AAC	AGA	CCA	2	G	A
TCGA-29-1781	SMARCA2_E	ENST00000349721	p.Q676Q	c.2028G>A	Unverified	AAG	CAG	ATC	3	G	A
TCGA-13-0924	SMARCAD1	ENST00000354268	p.S173N	c.518G>A	Verified	AGA	AGT	GAC	2	G	A
TCGA-25-2393	SMARCAL1	ENST00000357276	p.T742T	c.2226G>A	Verified	att	acg	caa	3	G	A
TCGA-04-1337	SMARCAL1	ENST00000357276	p.T592T	c.1776G>A	Verified	tac	acg	cag	3	G	A

TCGA-23-1021	SMARCD1	NM_003076.3	p.A451T	c.1351G>A	Verified	gag	gct	gtg	1	G	A
TCGA-24-1464	SMC1A	ENST00000322213	p.R895H	c.2684G>A	Verified	att	cgt	aag	2	G	A
TCGA-24-1563	SMC1A	ENST00000322213	p.R626H	c.1877G>A	Verified	gcc	cgc	cgc	2	G	A
TCGA-04-1362	SMC2	ENST00000286398	p.Q795Q	c.2385G>A	Verified	gct	cag	aaa	3	G	A
TCGA-25-2391	SMC2	ENST00000286398	p.R58R	c.174G>A	Unverified	gtt	cgg	gct	3	G	A
TCGA-23-1032	SMC4	NM_005496.2	p.D150N	c.448G>A	Verified	tct	gat	gaa	1	G	A
TCGA-29-1696	SMCHD1	ENST00000261598	p.G358D	c.1073G>A	Unverified	CCT	GGC	TTA	2	G	A
TCGA-29-1696	SMCHD1	ENST00000261598	p.G358D	c.1073G>A	Unverified	CCT	GGC	TTA	2	G	A
TCGA-29-1781	SMG7	ENST00000347615	p.R881K	c.2642G>A	Unverified	AAC	AGA	TCT	2	G	A
TCGA-29-1781	SMG7_ENST	ENST00000507469	p.R835K	c.2504G>A	Unverified	AAC	AGA	TCT	2	G	A
TCGA-24-1843	SMOC2	ENST00000354536	p.R203H	c.608G>A	Unverified	TCA	CGT	TAC	2	G	A
TCGA-61-1738	SMTNL1	ENST00000457912	p.R499H	c.1496G>A	Unverified	TAC	CGC	AGC	2	G	A
TCGA-61-1738	SMTNL1	ENST00000457912	p.R499H	c.1496G>A	Unverified	TAC	CGC	AGC	2	G	A
TCGA-61-1738	SMTNL1_EN	ENST00000457912_v65	p.R499H	c.1496G>A	Unverified	TAC	CGC	AGC	2	G	A
TCGA-61-1738	SMTNL1_EN	ENST00000457912_v65	p.R499H	c.1496G>A	Unverified	TAC	CGC	AGC	2	G	A
TCGA-29-1768	SNAP23	ENST00000249647	p.D65N	c.193G>A	Unverified	AAG	GAC	ATG	1	G	A
TCGA-30-1855	SND1	ENST00000354725	p.L323L	c.969G>A	Unverified	AGG	CTG	AGA	3	G	A
TCGA-61-1998	SNIP1	ENST00000296215	p.P46P	c.138G>A	Unverified	CGT	CCG	GAC	3	G	A
TCGA-13-0919	SNPH	ENST00000381873	p.E199E	c.597G>A	Verified	GGG	GAG	TCA	3	G	A
TCGA-61-1915	SNRNP40	ENST00000263694	p.V112V	c.336G>A	Unverified	GCA	GTG	ATG	3	G	A
TCGA-10-0930	SNRPD2P1	ENST00000277860	p.R19Q	c.56G>A	Verified	AAG	CGA	GAG	2	G	A
TCGA-10-0930	SNRPD2P1	ENST00000277860	p.R19Q	c.56G>A	Verified	AAG	CGA	GAG	2	G	A
TCGA-29-1776	SNTB1	ENST00000395601	p.E364K	c.1090G>A	Unverified	aag	gaa	gcc	1	G	A
TCGA-24-2035	SNTG1	ENST00000518864	p.C116Y	c.347G>A	Verified	AAA	TGT	AGA	2	G	A
TCGA-04-1343	SOCS5	ENST00000306503	p.R429Q	c.1286G>A	Verified	gcc	cga	att	2	G	A
TCGA-25-1313	SOCS6	ENST00000397942	p.R140Q	c.419G>A	Unverified	ctg	cgg	ccc	2	G	A
TCGA-29-1699	SORBS2	ENST00000284776	p.G831S	c.2491G>A	Unverified	ggc	ggt	gca	1	G	A
TCGA-04-1651	SORCS1	ENST00000263054	p.R337K	c.1010G>A	Unverified	GCC	AGA	ACT	2	G	A
TCGA-04-1651	SORCS1	ENST00000263054	p.R337K	c.1010G>A	Unverified	GCC	AGA	ACT	2	G	A
TCGA-04-1651	SORCS1_ENS	ENST00000344440	p.R337K	c.1010G>A	Unverified	GCC	AGA	ACT	2	G	A
TCGA-04-1651	SORCS1_ENS	ENST00000344440	p.R337K	c.1010G>A	Unverified	GCC	AGA	ACT	2	G	A
TCGA-13-0887	SORCS2	NM_020777.1	p.G443S	c.1327G>A	Verified	gag	ggc	ctc	1	G	A
TCGA-04-1331	SORCS2	NM_020777.1	p.R552Q	c.1655G>A	Unverified	tgc	cgg	gac	2	G	A
TCGA-24-2290	SORL1	ENST00000260197	p.D1267N	c.3799G>A	Verified	TCC	GAT	GAA	1	G	A

TCGA-24-2290	SORL1	ENST00000260197	p.D1267N	c.3799G>A	Verified	TCC	GAT	GAA	1	G	A
TCGA-04-1338	SOSTDC1	ENST00000307068	p.G119R	c.355G>A	Verified	TAT	GGA	ACA	1	G	A
TCGA-13-0900	SOX30	ENST00000265007	p.P726P	c.2178G>A	Verified	GCC	CCG	ACA	3	G	A
TCGA-25-2400	SPAG7	ENST00000206020	p.V194M	c.580G>A	Verified	CCC	GTG	GCC	1	G	A
TCGA-23-1117	SPATA5L1	ENST00000305560	p.P728P	c.2184G>A	Verified	AAA	CCG	TCG	3	G	A
TCGA-23-1117	SPATA5L1	ENST00000305560	p.P728P	c.2184G>A	Verified	AAA	CCG	TCG	3	G	A
TCGA-61-1740	SPEF2	ENST00000282469	p.E230K	c.688G>A	Unverified	CTC	GAG	GCC	1	G	A
TCGA-61-1740	SPEF2_ENST	ENST00000356031	p.E230K	c.688G>A	Unverified	CTC	GAG	GCC	1	G	A
TCGA-61-1907	SPEN	ENST00000375759	p.G2688R	c.8062G>A	Unverified	gct	ggg	ccc	1	G	A
TCGA-61-1910	SPG7	ENST00000268704	p.V662V	c.1986G>A	Unverified	atg	gtg	aag	3	G	A
TCGA-24-2280	SPG7	ENST00000268704	p.L65L	c.195G>A	Unverified	caa	ttg	aga	3	G	A
TCGA-13-0906	SPHKAP	XM_051221.5	p.P756P	c.2268G>A	Verified	gat	ccg	ggt	3	G	A
TCGA-24-0979	SPHKAP	XM_051221.5	p.L1387L	c.4161G>A	Verified	tct	ttg	agc	3	G	A
TCGA-04-1530	SPINT4	ENST00000279058	p.C34Y	c.101G>A	Verified	ATA	TGT	GGA	2	G	A
TCGA-13-1497	SPOCD1	ENST00000360482	p.E1003E	c.3009G>A	Verified	CTG	GAG	GTC	3	G	A
TCGA-61-1915	SPPL2A	ENST00000261854	p.?	c.585-1G>A	Unverified	gaa	ttg	gaa	3	G	A
TCGA-13-1510	SPR	ENST00000234454	p.V214M	c.640G>A	Verified	TCC	GTG	GAC	1	G	A
TCGA-13-0904	SPRR2E	ENST00000368751	p.K40K	c.120G>A	Verified	CCA	AAG	TGT	3	G	A
TCGA-04-1638	SPTA1	ENST00000368148	p.A816T	c.2446G>A	Unverified	TCA	GCT	ACT	1	G	A
TCGA-24-1843	SPTA1	ENST00000368148	p.E1396K	c.4186G>A	Unverified	CTG	GAG	TTG	1	G	A
TCGA-30-1718	SPTA1	ENST00000368148	p.R41Q	c.122G>A	Unverified	GAG	CGG	GTC	2	G	A
TCGA-29-1693	SPTA1	ENST00000368148	p.Q1401Q	c.4203G>A	Unverified	TTC	CAG	GGG	3	G	A
TCGA-13-0755	SPTB	ENST00000389722	p.R1843Q	c.5528G>A	Verified	GAG	CGG	GAG	2	G	A
TCGA-20-1686	SPTB	ENST00000389722	p.R1682H	c.5045G>A	Unverified	GAG	CGC	AAG	2	G	A
TCGA-29-1766	SPTB	ENST00000389722	p.R706H	c.2117G>A	Unverified	GCG	CGC	AAG	2	G	A
TCGA-61-1910	SPTB	ENST00000389722	p.A1884A	c.5652G>A	Unverified	GCC	GCG	TGG	3	G	A
TCGA-20-1686	SPTB_ENST(	ENST00000542895	p.R1682H	c.5045G>A	Unverified	GAG	CGC	AAG	2	G	A
TCGA-29-1766	SPTB_ENST(	ENST00000542895	p.R706H	c.2117G>A	Unverified	GCG	CGC	AAG	2	G	A
TCGA-61-1910	SPTB_ENST(	ENST00000542895	p.A1884A	c.5652G>A	Unverified	GCC	GCG	TGG	3	G	A
TCGA-23-1029	SRP72	ENST00000342756	p.G407D	c.1220G>A	Unverified	CCA	GGC	ATG	2	G	A
TCGA-13-1509	SRPK1	ENST00000373825	p.D468N	c.1402G>A	Verified	CTG	GAC	AAC	1	G	A
TCGA-29-1764	SRSF1	ENST00000258962	p.R97Q	c.290G>A	Unverified	GGC	CGA	GGC	2	G	A
TCGA-61-2012	SSH1	ENST00000326495	p.R144Q	c.431G>A	Verified	CTC	CGA	CTG	2	G	A
TCGA-20-1685	SSH3	ENST00000308127	p.T144T	c.432G>A	Unverified	GAG	ACG	GTC	3	G	A

TCGA-24-1843	SSRP1	ENST00000278412	p.A704A	c.2112G>A	Unverified	tca	gcg	tca	3	G	A
TCGA-04-1361	ST18	ENST00000276480	p.A92T	c.274G>A	Verified	ACC	GCA	GAG	1	G	A
TCGA-04-1361	ST18	ENST00000276480	p.A92T	c.274G>A	Verified	ACC	GCA	GAG	1	G	A
TCGA-25-2400	ST18	ENST00000276480	p.R557H	c.1670G>A	Verified	GGC	CGT	GCC	2	G	A
TCGA-13-0793	STARD8	ENST00000252336	p.D298N	c.892G>A	Unverified	GAT	GAT	GAG	1	G	A
TCGA-13-0793	STARD8_ENS	ENST00000252336	p.D298N	c.892G>A	Unverified	GAT	GAT	GAG	1	G	A
TCGA-13-1501	STAU1	ENST00000371856	p.A242T	c.724G>A	Verified	gcc	gcc	ata	1	G	A
TCGA-13-1501	STAU1	ENST00000371856	p.A242T	c.724G>A	Verified	gcc	gcc	ata	1	G	A
TCGA-13-0885	STAU1	ENST00000371856	p.R196Q	c.587G>A	Verified	aaa	cgg	aac	2	G	A
TCGA-13-0903	STK33	ENST00000447869	p.V490V	c.1470G>A	Verified	CCT	GTG	ACT	3	G	A
TCGA-04-1342	STON1-GTF2	ENST00000394754	p.V932M	c.2794G>A	Verified	ATC	GTG	CCT	1	G	A
TCGA-13-0919	STON1-GTF2	ENST00000394754	p.S784S	c.2352G>A	Verified	CAA	TCG	TCA	3	G	A
TCGA-09-1665	STRN3	NM_014574.2	p.L141L	c.423G>A	Unverified	gaa	ctg	aac	3	G	A
TCGA-30-1718	STT3B	ENST00000295770	p.A378T	c.1132G>A	Unverified	ATT	GCA	CCA	1	G	A
TCGA-25-2400	STX11	ENST00000367568	p.D155N	c.463G>A	Verified	CGC	GAC	AAC	1	G	A
TCGA-29-1691	STX16	ENST00000371132	p.V68I	c.202G>A	Unverified	GAT	GTT	GGC	1	G	A
TCGA-13-1507	STXBP1_ENS	ENST00000373299	p.S593N	c.1778G>A	Verified	ATA	AGC	AGT	2	G	A
TCGA-24-2035	SULT1E1	ENST00000226444	p.?	c.591+1G>A	Verified	AAA	GAG	GAT	3	G	A
TCGA-13-1488	SUPT6H	ENST00000314616	p.D18N	c.52G>A	Verified	AAT	GAT	GAA	1	G	A
TCGA-09-2044	SURF6	ENST00000372022	p.E143E	c.429G>A	Verified	TTG	GAG	AAA	3	G	A
TCGA-29-1703	SUSD4	ENST00000344029	p.E128K	c.382G>A	Unverified	ATC	GAA	GAT	1	G	A
TCGA-29-1703	SUSD4_ENST	ENST00000343846	p.E128K	c.382G>A	Unverified	ATC	GAA	GAT	1	G	A
TCGA-04-1338	SVEP1	ENST00000401783	p.V2781I	c.8341G>A	Verified	CCA	GTC	ATG	1	G	A
TCGA-13-0884	SVEP1	ENST00000401783	p.S3346N	c.10037G>A	Verified	TGG	AGC	CAC	2	G	A
TCGA-29-1691	SYNE1	ENST00000265368	p.E247K	c.739G>A	Unverified	GCC	GAA	ACA	1	G	A
TCGA-04-1649	SYNE1	ENST00000265368	p.R1165H	c.3494G>A	Unverified	AAA	CGT	GCC	2	G	A
TCGA-61-1737	SYNE1	ENST00000265368	p.K866K	c.2598G>A	Unverified	TGT	AAG	AAA	3	G	A
TCGA-29-1691	SYNE1_ENST	ENST00000265368	p.E247K	c.739G>A	Unverified	GCC	GAA	ACA	1	G	A
TCGA-04-1649	SYNE1_ENST	ENST00000265368	p.R1165H	c.3494G>A	Unverified	AAA	CGT	GCC	2	G	A
TCGA-61-1737	SYNE1_ENST	ENST00000265368	p.K866K	c.2598G>A	Unverified	TGT	AAG	AAA	3	G	A
TCGA-29-1691	SYNE1_ENST	ENST00000423061	p.E254K	c.760G>A	Unverified	GCC	GAA	ACA	1	G	A
TCGA-04-1649	SYNE1_ENST	ENST00000423061	p.R1172H	c.3515G>A	Unverified	AAA	CGT	GCC	2	G	A
TCGA-61-1737	SYNE1_ENST	ENST00000423061	p.K873K	c.2619G>A	Unverified	TGT	AAG	AAA	3	G	A
TCGA-04-1652	SYNE2	ENST00000358025	p.E448K	c.1342G>A	Unverified	TTT	GAA	AAT	1	G	A

TCGA-04-1652	SYNE2	ENST00000358025	p.E448K	c.1342G>A	Unverified	TTT	GAA	AAT	1	G	A
TCGA-61-1737	SYNE2	ENST00000358025	p.G6759R	c.20275G>A	Unverified	CAT	GGA	GAA	1	G	A
TCGA-23-1118	SYNGAP1	ENST00000418600	p.G355R	c.1063G>A	Unverified	GCT	GGG	CGC	1	G	A
TCGA-29-1701	SYNGAP1	ENST00000418600	p.G991R	c.2971G>A	Unverified	TCC	GGG	GTC	1	G	A
TCGA-23-1118	SYNGAP1_EI	ENST00000293748	p.G355R	c.1063G>A	Unverified	GCT	GGG	CGC	1	G	A
TCGA-29-1701	SYNGAP1_EI	ENST00000293748	p.G991R	c.2971G>A	Unverified	TCC	GGG	GTC	1	G	A
TCGA-13-0762	SYNPO2	ENST00000307142	p.S243S	c.729G>A	Verified	AAT	TCG	ATC	3	G	A
TCGA-13-0762	SYNPO2	ENST00000307142	p.S243S	c.729G>A	Verified	AAT	TCG	ATC	3	G	A
TCGA-23-1117	SYT9	ENST00000318881	p.K479K	c.1437G>A	Verified	CGG	AAG	CCC	3	G	A
TCGA-23-1117	SYT9	ENST00000318881	p.K479K	c.1437G>A	Verified	CGG	AAG	CCC	3	G	A
TCGA-09-0369	SZT2	ENST00000372442	p.V704I	c.2110G>A	Verified	CCA	GTT	GGA	1	G	A
TCGA-09-0369	SZT2	ENST00000372442	p.V704I	c.2110G>A	Verified	CCA	GTT	GGA	1	G	A
TCGA-29-2427	SZT2	ENST00000372442	p.R995R	c.2985G>A	Verified	TTG	CGG	ACT	3	G	A
TCGA-04-1652	TACC2	ENST00000369005	p.R606H	c.1817G>A	Unverified	AAG	CGT	GAT	2	G	A
TCGA-04-1652	TACC2	ENST00000369005	p.R606H	c.1817G>A	Unverified	AAG	CGT	GAT	2	G	A
TCGA-13-1497	TACC2	ENST00000369005	p.P100P	c.300G>A	Verified	CCA	CCG	TCC	3	G	A
TCGA-04-1343	TAF1	ENST00000373790	p.G626D	c.1877G>A	Verified	ttt	ggt	gca	2	G	A
TCGA-04-1638	TAF1L	ENST00000242310	p.D479N	c.1435G>A	Unverified	gat	gat	gac	1	G	A
TCGA-23-2077	TAF6L	ENST00000294168	p.A79T	c.235G>A	Verified	GAG	GCT	GTG	1	G	A
TCGA-04-1367	TANC1	ENST00000263635	p.L660L	c.1980G>A	Verified	GAC	CTG	CAC	3	G	A
TCGA-24-1431	TANC2	ENST00000389520	p.R1349K	c.4046G>A	Verified	AGC	AGA	CAG	2	G	A
TCGA-13-0905	TARBP1	ENST00000040877	p.W893*	c.2678G>A	Verified	CAA	TGG	GTG	2	G	A
TCGA-30-1855	TARBP1	ENST00000040877	p.L638L	c.1914G>A	Unverified	TCT	CTG	ATG	3	G	A
TCGA-23-1110	TARS	ENST00000265112	p.G43S	c.127G>A	Verified	GGA	GGT	CGA	1	G	A
TCGA-24-1849	TAS1R2	ENST00000375371	p.A658T	c.1972G>A	Unverified	TGC	GCC	TTC	1	G	A
TCGA-13-1498	TAS1R2	ENST00000375371	p.R707H	c.2120G>A	Verified	ACC	CGT	ACT	2	G	A
TCGA-13-0762	TAS2R20	ENST00000538986	p.V141I	c.421G>A	Unverified	TTG	GTT	TGT	1	G	A
TCGA-13-0762	TAS2R20	ENST00000538986	p.V141I	c.421G>A	Unverified	TTG	GTT	TGT	1	G	A
TCGA-24-2024	TAS2R40	ENST00000408947	p.T181T	c.543G>A	Verified	TCC	ACG	GAG	3	G	A
TCGA-24-1844	TBC1D14	ENST00000340816	p.A251T	c.751G>A	Unverified	AAA	GCG	CCA	1	G	A
TCGA-24-1844	TBC1D14_EI	ENST00000409757	p.A266T	c.796G>A	Unverified	AAA	GCG	CCA	1	G	A
TCGA-29-1770	TBC1D22B	ENST00000373491	p.E250K	c.748G>A	Unverified	ATT	GAA	CAG	1	G	A
TCGA-24-1563	TBC1D28	ENST00000345096	p.A110A	c.330G>A	Verified	CGG	GCG	TTG	3	G	A
TCGA-29-1693	TBC1D2B	ENST00000409931	p.R668H	c.2003G>A	Unverified	CAC	CGT	TCC	2	G	A

TCGA-29-1693	TBC1D2B_EI	ENST00000300584	p.R668H	c.2003G>A	Unverified	CAC	CGT	TCC	2	G	A
TCGA-29-1777	TBC1D3P2	ENST00000338790	p.G40E	c.119G>A	Unverified	TTT	GGA	AGC	2	G	A
TCGA-29-1769	TBP	ENST00000230354	p.Q76Q	c.228G>A	Unverified	CAG	CAG	CAG	3	G	A
TCGA-29-1698	TBRG4	ENST00000258770	p.R175H	c.524G>A	Unverified	TGG	CGC	ATG	2	G	A
TCGA-13-0886	TBX5	ENST00000310346	p.S372S	c.1116G>A	Verified	GAG	TCG	GCA	3	G	A
TCGA-24-0975	TCEAL2	ENST00000372780	p.P57P	c.171G>A	Unverified	gaa	ccg	tta	3	G	A
TCGA-29-1695	TCEB3B	ENST00000332567	p.A543T	c.1627G>A	Unverified	GAC	GCC	CTC	1	G	A
TCGA-24-1103	TCEB3B	ENST00000332567	p.L529L	c.1587G>A	Verified	ACG	CTG	CGC	3	G	A
TCGA-29-1763	TCERG1	ENST00000296702	p.A651T	c.1951G>A	Unverified	GCT	GCC	CGA	1	G	A
TCGA-61-1904	TCERG1L	ENST00000368643	p.R262Q	c.785G>A	Unverified	CTG	CGG	GCC	2	G	A
TCGA-61-2012	TCHH	ENST00000368804	p.E547E	c.1641G>A	Verified	CGC	GAG	CAG	3	G	A
TCGA-61-1737	TCN1	ENST00000257264	p.A191T	c.571G>A	Unverified	ATG	GCT	GTC	1	G	A
TCGA-24-1104	TCOF1	ENST00000323668	p.G1044R	c.3130G>A	Verified	AAA	GGG	ACC	1	G	A
TCGA-25-1313	TCTE1	ENST00000371505	p.C142Y	c.425G>A	Verified	GTG	TGC	CAC	2	G	A
TCGA-20-1683	TDRD5	ENST00000367614	p.D629N	c.1885G>A	Unverified	GTA	GAT	GGA	1	G	A
TCGA-61-1998	TDRD5	ENST00000367614	p.R925H	c.2774G>A	Verified	AAG	CGT	CAA	2	G	A
TCGA-25-2393	TECR	ENST00000215567	p.R159H	c.476G>A	Unverified	TTG	CGC	AAC	2	G	A
TCGA-20-0990	TECTA	ENST00000392793	p.P1461P	c.4383G>A	Verified	GAC	CCG	CGC	3	G	A
TCGA-29-1761	TEKT4	ENST00000295201	p.A55T	c.163G>A	Unverified	CAG	GCC	TTC	1	G	A
TCGA-09-2044	TELO2	ENST00000262319	p.L288L	c.864G>A	Unverified	AAC	CTG	GTG	3	G	A
TCGA-30-1714	TEP1	ENST00000262715	p.R1328Q	c.3983G>A	Unverified	GCC	CGG	CTG	2	G	A
TCGA-61-1737	TET1_ENST0	ENST00000373644	p.K1653K	c.4959G>A	Unverified	AGC	AAG	GAA	3	G	A
TCGA-24-2280	TET3	ENST00000409262	p.G644E	c.1931G>A	Unverified	GGA	GGA	CAG	2	G	A
TCGA-09-1665	TEX11	ENST00000374333	p.V639V	c.1917G>A	Verified	cca	gtg	atg	3	G	A
TCGA-13-0793	TEX14	ENST00000349033	p.C179Y	c.536G>A	Verified	TGG	TGT	GGG	2	G	A
TCGA-30-1718	TEX264	ENST00000395057	p.V131V	c.393G>A	Unverified	CAT	GTG	GTG	3	G	A
TCGA-24-2289	TFE3_ENST0	ENST00000336239	p.A243A	c.729G>A	Unverified	AGT	GCG	CCC	3	G	A
TCGA-23-1124	TFRC	ENST00000360110	p.R155H	c.464G>A	Verified	CCT	CGT	GAG	2	G	A
TCGA-61-1733	TG	ENST00000220616	p.E1561K	c.4681G>A	Unverified	CTT	GAG	GAC	1	G	A
TCGA-61-1733	TG	ENST00000220616	p.E1561K	c.4681G>A	Unverified	CTT	GAG	GAC	1	G	A
TCGA-61-1895	TG	ENST00000220616	p.G624R	c.1870G>A	Unverified	GAA	GGA	AGC	1	G	A
TCGA-09-2051	TG	ENST00000220616	p.R2336Q	c.7007G>A	Unverified	TAC	CGA	GTG	2	G	A
TCGA-13-0900	TG	ENST00000220616	p.C237Y	c.710G>A	Verified	CAC	TGT	GCT	2	G	A
TCGA-29-1764	TG	ENST00000220616	p.V742V	c.2226G>A	Unverified	ACG	GTG	CAG	3	G	A

TCGA-61-2009	TGFBR2	NM_003242	p.R479Q	c.1436G>A	Verified	gtg	cgg	gag	2	G	A
TCGA-24-1469	TGM3	ENST00000381458	p.R608Q	c.1823G>A	Verified	GTG	CGG	AAG	2	G	A
TCGA-30-1718	TGS1	ENST00000260129	p.M12I	c.36G>A	Unverified	GAA	ATG	TTT	3	G	A
TCGA-20-1687	THADA	ENST00000405975	p.V43M	c.127G>A	Unverified	tgt	gtg	caa	1	G	A
TCGA-61-1907	THEMIS	ENST00000368248	p.K460K	c.1380G>A	Unverified	GTG	AAG	GTG	3	G	A
TCGA-04-1362	THOC2	OTTHUMT00000058153.	p.D111N	c.331G>A	Verified	agt	gat	tta	1	G	A
TCGA-04-1652	THOC2_ENS	ENST00000245838	p.A4A	c.12G>A	Unverified	GCC	GCG	GCT	3	G	A
TCGA-04-1652	THOC2_ENS	ENST00000245838	p.A4A	c.12G>A	Unverified	GCC	GCG	GCT	3	G	A
TCGA-24-1464	THOP1	ENST00000307741	p.D109N	c.325G>A	Verified	TTC	GAC	GTG	1	G	A
TCGA-13-0886	THSD1	ENST00000258613	p.L673L	c.2019G>A	Unverified	ACT	CTG	ACT	3	G	A
TCGA-29-1691	TIGD2	ENST00000317005	p.G403R	c.1207G>A	Unverified	GAA	GGA	GCC	1	G	A
TCGA-04-1343	TIGD4	ENST00000304337	p.S159S	c.477G>A	Verified	CCT	TCG	ACT	3	G	A
TCGA-04-1530	TIMELESS	ENST00000553532	p.R831R	c.2493G>A	Unverified	CTT	CGG	GAG	3	G	A
TCGA-13-0916	TIMM44	ENST00000270538	p.R305Q	c.914G>A	Verified	CTC	CGG	GTG	2	G	A
TCGA-24-1463	TKTL1	ENST00000369915	p.D315N	c.943G>A	Verified	GGT	GAC	ACC	1	G	A
TCGA-61-2012	TKTL1	ENST00000369915	p.S43N	c.128G>A	Verified	ACG	AGC	TCC	2	G	A
TCGA-13-1507	TLN2	ENST00000306829	p.?	c.7375-1G>A	Verified	CAG	GCG	GCA	1	G	A
TCGA-24-1431	TLN2	ENST00000306829	p.D2341N	c.7021G>A	Verified	CTG	GAC	TTT	1	G	A
TCGA-04-1347	TLR1	ENST00000308979	p.A476T	c.1426G>A	Unverified	GTT	GCT	TTC	1	G	A
TCGA-29-1761	TLR2	ENST00000260010	p.E383K	c.1147G>A	Unverified	TGT	GAG	GAT	1	G	A
TCGA-13-0760	TLR4	ENST00000355622	p.A814T	c.2440G>A	Verified	AAA	GCC	CTG	1	G	A
TCGA-23-2078	TLR4	ENST00000355622	p.G830E	c.2489G>A	Verified	ACA	GGA	TGC	2	G	A
TCGA-13-1512	TM4SF18	ENST00000296059	p.A112T	c.334G>A	Verified	TCT	GCC	TTG	1	G	A
TCGA-20-1683	TM4SF20	ENST00000304568	p.G220E	c.659G>A	Unverified	TGT	GGA	GTC	2	G	A
TCGA-61-1914	TMCO7_ENS	ENST00000261778	p.R745H	c.2234G>A	Unverified	CTC	CGC	ATC	2	G	A
TCGA-13-0885	TMED1	ENST00000214869	p.E142K	c.424G>A	Verified	GTG	GAG	CCC	1	G	A
TCGA-04-1343	TMEM108	ENST00000321871	p.G288S	c.862G>A	Verified	GGT	GGT	TCT	1	G	A
TCGA-04-1652	TMEM119	ENST00000392806	p.E43E	c.129G>A	Unverified	GGG	GAG	GCC	3	G	A
TCGA-04-1652	TMEM119	ENST00000392806	p.E43E	c.129G>A	Unverified	GGG	GAG	GCC	3	G	A
TCGA-20-1683	TMEM120B	ENST00000342607	p.R174R	c.522G>A	Unverified	ATT	CGG	GAG	3	G	A
TCGA-20-1683	TMEM120B_	ENST00000449592	p.R174R	c.522G>A	Unverified	ATT	CGG	GAG	3	G	A
TCGA-09-1674	TMEM130	ENST00000339375	p.W108*	c.323G>A	Unverified	GTC	TGG	GTC	2	G	A
TCGA-29-1696	TMEM130	ENST00000339375	p.P375P	c.1125G>A	Unverified	AAC	CCG	GAG	3	G	A
TCGA-29-1696	TMEM130	ENST00000339375	p.P375P	c.1125G>A	Unverified	AAC	CCG	GAG	3	G	A

TCGA-13-1498	TMEM132D	ENST00000422113	p.E557E	c.1671G>A	Verified	GAA	GAG	GAG	3	G	A
TCGA-13-0792	TMEM136	ENST00000529187	p.W17*	c.50G>A	Verified	TTT	TGG	TCT	2	G	A
TCGA-23-1111	TMEM139	ENST00000359333	p.A137T	c.409G>A	Unverified	AGA	GCC	AAA	1	G	A
TCGA-09-1674	TMEM14A	ENST00000211314	p.R51Q	c.152G>A	Unverified	AAA	CGA	GAT	2	G	A
TCGA-13-0900	TMEM150C	ENST00000449862	p.C66Y	c.197G>A	Verified	AGC	TGT	GTG	2	G	A
TCGA-24-2024	TMEM169	ENST00000295658	p.R56H	c.167G>A	Verified	TAC	CGC	TCA	2	G	A
TCGA-13-0755	TMEM186	ENST00000333050	p.R201H	c.602G>A	Verified	GAG	CGT	TTC	2	G	A
TCGA-13-0904	TMEM2	ENST00000377044	p.P1252P	c.3756G>A	Verified	GAT	CCG	TGC	3	G	A
TCGA-30-1714	TMEM22	ENST00000446465	p.D35N	c.103G>A	Unverified	GGC	GAT	GAT	1	G	A
TCGA-20-1683	TMEM30A	ENST00000230461	p.G203D	c.608G>A	Unverified	AAA	GGT	ATT	2	G	A
TCGA-61-1907	TMEM48	ENST00000371429	p.A660T	c.1978G>A	Unverified	TCT	GCA	GAA	1	G	A
TCGA-24-2289	TMEM63B	ENST00000259746	p.A687A	c.2061G>A	Unverified	GCC	GCG	CCC	3	G	A
TCGA-04-1542	TMLHE	ENST00000334398	p.V74I	c.220G>A	Verified	tac	gtc	tgg	1	G	A
TCGA-29-1770	TMPPE	ENST00000342462	p.A11T	c.31G>A	Unverified	GGC	GCG	AAG	1	G	A
TCGA-20-0991	TNFSF14	ENST00000245912	p.G188R	c.562G>A	Unverified	tgc	gga	cgg	1	G	A
TCGA-13-1498	TNFSF8	ENST00000223795	p.D215N	c.643G>A	Verified	ata	gat	aca	1	G	A
TCGA-09-2051	TNIK	ENST00000436636	p.R1008K	c.3023G>A	Unverified	CTT	AGG	CAA	2	G	A
TCGA-20-1683	TNK2	ENST00000333602	p.E4E	c.12G>A	Unverified	CCA	GAG	GAG	3	G	A
TCGA-13-0761	TNK2	ENST00000333602	p.R99R	c.297G>A	Verified	TTC	CGG	AAG	3	G	A
TCGA-13-0761	TNK2	ENST00000333602	p.R99R	c.297G>A	Verified	TTC	CGG	AAG	3	G	A
TCGA-20-1683	TNK2_ENST(	ENST00000333602	p.E4E	c.12G>A	Unverified	CCA	GAG	GAG	3	G	A
TCGA-20-1683	TNK2_ENST(	ENST00000381916	p.E67E	c.201G>A	Unverified	CCA	GAG	GAG	3	G	A
TCGA-13-0761	TNK2_ENST(	ENST00000381916	p.R162R	c.486G>A	Verified	TTC	CGG	AAG	3	G	A
TCGA-13-0761	TNK2_ENST(	ENST00000381916	p.R162R	c.486G>A	Verified	TTC	CGG	AAG	3	G	A
TCGA-13-0916	TNN	ENST00000239462	p.G1046S	c.3136G>A	Verified	AAG	GGT	GGT	1	G	A
TCGA-13-1505	TNNI3K	ENST00000326637	p.G351S	c.1051G>A	Unverified	cac	ggt	cac	1	G	A
TCGA-61-1906	TNPO3	ENST00000265388	p.L324L	c.972G>A	Unverified	act	ctg	gag	3	G	A
TCGA-61-1906	TNPO3	ENST00000265388	p.L324L	c.972G>A	Unverified	act	ctg	gag	3	G	A
TCGA-24-1616	TNPO3	ENST00000265388	p.R797R	c.2391G>A	Verified	atg	agg	ttt	3	G	A
TCGA-61-1906	TNPO3_ENS	ENST00000393245	p.L324L	c.972G>A	Unverified	ACT	CTG	GAG	3	G	A
TCGA-61-1906	TNPO3_ENS	ENST00000393245	p.L324L	c.972G>A	Unverified	ACT	CTG	GAG	3	G	A
TCGA-25-1313	TNRC18_EN:	ENST00000430969	p.A832T	c.2494G>A	Verified	ATG	GCC	CCT	1	G	A
TCGA-13-0913	TNS4	ENST00000254051	p.G597E	c.1790G>A	Verified	ACT	GGA	GCC	2	G	A
TCGA-04-1367	TNXB_ENST(	ENST00000375247	p.S1474S	c.4422G>A	Verified	GAT	TCG	CTG	3	G	A

TCGA-13-0906	TOM1	ENST00000449058	p.E363K	c.1087G>A	Unverified	CTG	GAA	GAT	1	G	A
TCGA-25-1326	TONSL	ENST00000292520	p.R690H	c.2069G>A	Unverified	CCC	CGC	CCC	2	G	A
TCGA-24-2262	TOP3A	ENST00000321105	p.R548Q	c.1643G>A	Unverified	gcc	cgg	atg	2	G	A
TCGA-23-1809	TOP3B	ENST00000357179	p.V136I	c.406G>A	Unverified	CCC	GTC	ATG	1	G	A
TCGA-23-1809	TOP3B_ENS	ENST00000357179	p.V136I	c.406G>A	Unverified	CCC	GTC	ATG	1	G	A
TCGA-13-0755	TOPORS	ENST00000360538	p.V128I	c.382G>A	Verified	TGT	GTA	CAG	1	G	A
TCGA-04-1338	TP53	ENST00000269305	p.G245S	c.733G>A	Verified	GGC	GGC	ATG	1	G	A
TCGA-04-1362	TP53	ENST00000269305	p.?	c.673-1G>A	Verified	GAG	GTT	GGC	1	G	A
TCGA-09-2049	TP53	ENST00000269305	p.G245S	c.733G>A	Verified	GGC	GGC	ATG	1	G	A
TCGA-13-0886	TP53	ENST00000269305	p.V272M	c.814G>A	Verified	GAG	GTG	CGT	1	G	A
TCGA-24-1103	TP53	ENST00000269305	p.?	c.919+1G>A	Verified	CGA	GCA	CTG	1	G	A
TCGA-24-1435	TP53	ENST00000269305	p.V216M	c.646G>A	Verified	AGT	GTG	GTG	1	G	A
TCGA-24-2024	TP53	ENST00000269305	p.V272M	c.814G>A	Verified	GAG	GTG	CGT	1	G	A
TCGA-25-1326	TP53	ENST00000269305	p.E286K	c.856G>A	Verified	GAG	GAA	GAG	1	G	A
TCGA-61-2102	TP53	ENST00000269305	p.G266R	c.796G>A	Verified	CTG	GGA	CGG	1	G	A
TCGA-04-1530	TP53	ENST00000269305	p.R273H	c.818G>A	Verified	GTG	CGT	GTT	2	G	A
TCGA-04-1542	TP53	ENST00000269305	p.R273H	c.818G>A	Verified	GTG	CGT	GTT	2	G	A
TCGA-13-0761	TP53	ENST00000269305	p.R273H	c.818G>A	Verified	GTG	CGT	GTT	2	G	A
TCGA-13-0761	TP53	ENST00000269305	p.R273H	c.818G>A	Verified	GTG	CGT	GTT	2	G	A
TCGA-13-0795	TP53	ENST00000269305	p.C135Y	c.404G>A	Verified	TTT	TGC	CAA	2	G	A
TCGA-13-0893	TP53	ENST00000269305	p.G245D	c.734G>A	Verified	GGC	GGC	ATG	2	G	A
TCGA-13-0900	TP53	ENST00000269305	p.R273H	c.818G>A	Verified	GTG	CGT	GTT	2	G	A
TCGA-13-0904	TP53	ENST00000269305	p.G279E	c.836G>A	Verified	CCT	GGG	AGA	2	G	A
TCGA-13-1496	TP53	ENST00000269305	p.R248Q	c.743G>A	Verified	AAC	CGG	AGG	2	G	A
TCGA-13-1499	TP53	ENST00000269305	p.R248Q	c.743G>A	Verified	AAC	CGG	AGG	2	G	A
TCGA-23-1032	TP53	ENST00000269305	p.R248Q	c.743G>A	Verified	AAC	CGG	AGG	2	G	A
TCGA-23-1110	TP53	ENST00000269305	p.C176Y	c.527G>A	Verified	CGC	TGC	CCC	2	G	A
TCGA-23-1122	TP53	ENST00000269305	p.R175H	c.524G>A	Verified	AGG	CGC	TGC	2	G	A
TCGA-23-1123	TP53	ENST00000269305	p.C176Y	c.527G>A	Verified	CGC	TGC	CCC	2	G	A
TCGA-23-1123	TP53	ENST00000269305	p.C176Y	c.527G>A	Verified	CGC	TGC	CCC	2	G	A
TCGA-23-2077	TP53	ENST00000269305	p.R175H	c.524G>A	Verified	AGG	CGC	TGC	2	G	A
TCGA-24-0979	TP53	ENST00000269305	p.R248Q	c.743G>A	Verified	AAC	CGG	AGG	2	G	A
TCGA-25-1318	TP53	ENST00000269305	p.R175H	c.524G>A	Verified	AGG	CGC	TGC	2	G	A
TCGA-25-1318	TP53	ENST00000269305	p.R175H	c.524G>A	Verified	AGG	CGC	TGC	2	G	A

TCGA-29-2427	TP53	ENST00000269305	p.G244D	c.731G>A	Verified	ATG	GGC	GGC	2	G	A
TCGA-24-1845	TP53	ENST00000269305	p.W53*	c.159G>A	Unverified	CAA	TGG	TTC	3	G	A
TCGA-25-2042	TP53	ENST00000269305	p.E224E	c.672G>A	Verified	CCT	GAG	GTT	3	G	A
TCGA-13-0885	TP53	ENST00000269305	p.W146*	c.438G>A	Verified	CTG	TGG	GTT	3	G	A
TCGA-24-1845	TP53_ENST(	ENST00000269305	p.W53*	c.159G>A	Unverified	CAA	TGG	TTC	3	G	A
TCGA-24-1845	TP53_ENST(	ENST00000413465	p.W53*	c.159G>A	Unverified	CAA	TGG	TTC	3	G	A
TCGA-29-1777	TP63	ENST00000264731	p.R643Q	c.1928G>A	Unverified	gtg	cga	ttc	2	G	A
TCGA-29-1701	TPCN1	ENST00000335509	p.D284N	c.850G>A	Unverified	CCA	GAT	GTG	1	G	A
TCGA-29-1701	TPCN1_ENS	ENST00000550785	p.D356N	c.1066G>A	Unverified	CCA	GAT	GTG	1	G	A
TCGA-29-1785	TPP1	ENST00000299427	p.R185H	c.554G>A	Unverified	CAA	CGT	CCT	2	G	A
TCGA-29-1785	TPP1	ENST00000299427	p.R185H	c.554G>A	Unverified	CAA	CGT	CCT	2	G	A
TCGA-24-1435	TRAF1	ENST00000373887	p.V289I	c.865G>A	Verified	acc	gtc	agc	1	G	A
TCGA-23-1111	TRAK1	ENST00000341421	p.A682T	c.2044G>A	Unverified	TCC	GCT	CGT	1	G	A
TCGA-23-1114	TRAP1	ENST00000246957	p.D557N	c.1669G>A	Unverified	GTG	GAT	CAC	1	G	A
TCGA-04-1361	TRAPPC10	ENST00000291574	p.C23Y	c.68G>A	Verified	ACC	TGT	GCT	2	G	A
TCGA-04-1361	TRAPPC10	ENST00000291574	p.C23Y	c.68G>A	Verified	ACC	TGT	GCT	2	G	A
TCGA-09-2056	TRAPPC8	ENST00000283351	p.?	c.4073+1G>A	Verified	ACA	AGT	CCA	2	G	A
TCGA-23-1124	TRERF1	ENST00000372922	p.D1164N	c.3490G>A	Verified	gac	gac	gac	1	G	A
TCGA-23-1124	TRERF1	ENST00000372922	p.D1165N	c.3493G>A	Verified	gac	gac	gtc	1	G	A
TCGA-29-1691	TRIB3	ENST00000217233	p.E204E	c.612G>A	Unverified	CTG	GAG	GAC	3	G	A
TCGA-23-1110	TRIM10_EN!	ENST00000449742	p.R473Q	c.1418G>A	Unverified	GGC	CGA	GGG	2	G	A
TCGA-61-1904	TRIM17	ENST00000366697	p.C99Y	c.296G>A	Unverified	ctg	tgc	cag	2	G	A
TCGA-61-1904	TRIM17_EN!	ENST00000456946	p.C99Y	c.296G>A	Unverified	CTG	TGC	CAG	2	G	A
TCGA-13-0884	TRIM21	ENST00000254436	p.A437T	c.1309G>A	Verified	tgt	gcc	ttt	1	G	A
TCGA-13-1497	TRIM26	ENST00000453195	p.R202Q	c.605G>A	Verified	GAG	CGG	GAG	2	G	A
TCGA-09-2049	TRIM36	ENST00000282369	p.V429I	c.1285G>A	Verified	AAA	GTT	TAT	1	G	A
TCGA-13-0920	TRIM39	ENST00000376656	p.G436R	c.1306G>A	Verified	AAT	GGG	GAC	1	G	A
TCGA-23-1124	TRIM55	ENST00000315962	p.V38V	c.114G>A	Verified	gtg	gtg	att	3	G	A
TCGA-09-2056	TRIM58	ENST00000366481	p.L458L	c.1374G>A	Verified	ATC	TTG	CCA	3	G	A
TCGA-23-1123	TRIM67	NM_001004342.2	p.G574S	c.1720G>A	Verified	gac	ggt	ctt	1	G	A
TCGA-23-1123	TRIM67	NM_001004342.2	p.G574S	c.1720G>A	Verified	gac	ggt	ctt	1	G	A
TCGA-24-0979	TRIOBP_EN!	ENST00000406386	p.D813N	c.2437G>A	Verified	CAG	GAC	AAC	1	G	A
TCGA-61-1998	TRIP10	ENST00000313285	p.D260N	c.778G>A	Verified	gtg	gat	ccc	1	G	A
TCGA-13-0906	TRIP12	ENST00000283943	p.A179T	c.535G>A	Verified	CTG	GCT	TCA	1	G	A

TCGA-24-2280	TRIT1	ENST00000316891	p.E145K	c.433G>A	Unverified	ACT	GAG	AAA	1	G	A
TCGA-29-1764	TROVE2	ENST00000367444	p.G68S	c.202G>A	Unverified	GAT	GGC	AGA	1	G	A
TCGA-61-1914	TROVE2	ENST00000367444	p.R149Q	c.446G>A	Unverified	CTC	CGG	AAG	2	G	A
TCGA-61-1915	TRPM1	ENST00000397795	p.L573L	c.1719G>A	Unverified	CTT	CTG	GGA	3	G	A
TCGA-29-2427	TRPV1	ENST00000399756	p.V222M	c.664G>A	Verified	CTG	GTG	GAG	1	G	A
TCGA-29-1702	TRPV3	ENST00000576742	p.S518S	c.1554G>A	Unverified	CTC	TCG	GAT	3	G	A
TCGA-24-1469	TRRAP	ENST00000355540	p.V1955V	c.5865G>A	Verified	AAG	GTG	TAC	3	G	A
TCGA-20-1685	TSFM	ENST00000454289	p.S306S	c.918G>A	Unverified	GTG	TCG	GTA	3	G	A
TCGA-23-1117	TSGA13	ENST00000356588	p.D166N	c.496G>A	Verified	TCG	GAT	GAT	1	G	A
TCGA-23-1117	TSGA13	ENST00000356588	p.D166N	c.496G>A	Verified	TCG	GAT	GAT	1	G	A
TCGA-13-0893	TSHZ3	NM_020856.1	p.S702S	c.2106G>A	Verified	gag	tcg	acg	3	G	A
TCGA-29-1695	TSKS	ENST00000246801	p.M90I	c.270G>A	Unverified	GCC	ATG	GAG	3	G	A
TCGA-23-1116	TSPAN12	ENST00000222747	p.L214L	c.642G>A	Unverified	TTT	TTG	AGA	3	G	A
TCGA-29-1783	TSPAN18	ENST00000340160	p.G186R	c.556G>A	Unverified	GAC	GGG	GTC	1	G	A
TCGA-04-1649	TSSK6	ENST00000360913	p.E20K	c.58G>A	Unverified	gga	gag	ggc	1	G	A
TCGA-13-0886	TTBK1	ENST00000259750	p.S524N	c.1571G>A	Verified	ctg	agc	aac	2	G	A
TCGA-29-1775	TTC13	ENST00000366661	p.D141N	c.421G>A	Unverified	AAT	GAC	AGC	1	G	A
TCGA-13-1512	TTC13	ENST00000366661	p.S549S	c.1647G>A	Verified	AAC	TCG	AAA	3	G	A
TCGA-24-1422	TTC15	ENST00000382110	p.S52S	c.156G>A	Unverified	GCA	TCG	GAA	3	G	A
TCGA-24-2262	TTC17	ENST00000039989	p.R1003Q	c.3008G>A	Verified	ACC	CGA	ATT	2	G	A
TCGA-09-1674	TTC17	ENST00000039989	p.T1013T	c.3039G>A	Unverified	CAG	ACG	TCC	3	G	A
TCGA-23-2078	TTC21A	ENST00000431162	p.G609S	c.1825G>A	Verified	GAA	GGC	AGA	1	G	A
TCGA-13-0924	TTC25	ENST00000377543	p.Q330Q	c.990G>A	Verified	GCC	CAG	ATT	3	G	A
TCGA-29-1781	TTC27	ENST00000317907	p.V366V	c.1098G>A	Unverified	GAA	GTG	GAG	3	G	A
TCGA-04-1652	TTLL2	ENST00000239587	p.V91I	c.271G>A	Unverified	CGC	GTT	GAC	1	G	A
TCGA-04-1652	TTLL2	ENST00000239587	p.V91I	c.271G>A	Unverified	CGC	GTT	GAC	1	G	A
TCGA-13-0886	TTLL4	ENST00000392102	p.E972K	c.2914G>A	Verified	CCA	GAG	CAT	1	G	A
TCGA-09-1674	TTN	NM_003319	p.G14510S	c.43528G>A	Unverified	aaa	ggc	tct	1	G	A
TCGA-29-1711	TTN	NM_003319	p.G1325R	c.3973G>A	Unverified	aaa	gga	aat	1	G	A
TCGA-29-1711	TTN	NM_003319	p.G1325R	c.3973G>A	Unverified	aaa	gga	aat	1	G	A
TCGA-29-1761	TTN	NM_003319	p.V19301I	c.57901G>A	Unverified	gac	gtt	att	1	G	A
TCGA-29-2427	TTN	NM_003319	p.V865I	c.2593G>A	Verified	acc	gtc	cgt	1	G	A
TCGA-61-2012	TTN	NM_003319	p.A11785T	c.35353G>A	Verified	acg	gct	ggc	1	G	A
TCGA-13-0885	TTN	NM_003319	p.E23401K	c.70201G>A	Verified	acc	gaa	gga	1	G	A

TCGA-09-1674	TTN	NM_003319	p.R6038Q	c.18113G>A	Unverified	tgt	cga	ctc	2	G	A
TCGA-61-2095	TTN	NM_003319	p.Q17043Q	c.51129G>A	Verified	tta	cag	tgg	3	G	A
TCGA-09-1674	TTN_ENST0(	ENST00000342175	p.G14702S	c.44104G>A	Unverified	AAA	GGC	TCT	1	G	A
TCGA-29-1711	TTN_ENST0(	ENST00000342175	p.G1325R	c.3973G>A	Unverified	AAA	GGA	AAT	1	G	A
TCGA-29-1711	TTN_ENST0(	ENST00000342175	p.G1325R	c.3973G>A	Unverified	AAA	GGA	AAT	1	G	A
TCGA-29-1761	TTN_ENST0(	ENST00000342175	p.V19493I	c.58477G>A	Unverified	GAC	GTT	ATT	1	G	A
TCGA-61-2094	TTN_ENST0(	ENST00000342175	p.E17011K	c.51031G>A	Unverified	ATC	GAA	ATT	1	G	A
TCGA-09-1674	TTN_ENST0(	ENST00000342175	p.R6230Q	c.18689G>A	Unverified	TGT	CGA	CTC	2	G	A
TCGA-09-1674	TTN_ENST0(	ENST00000342992	p.G21007S	c.63019G>A	Unverified	AAA	GGC	TCT	1	G	A
TCGA-29-1711	TTN_ENST0(	ENST00000342992	p.G1371R	c.4111G>A	Unverified	AAA	GGA	AAT	1	G	A
TCGA-29-1711	TTN_ENST0(	ENST00000342992	p.G1371R	c.4111G>A	Unverified	AAA	GGA	AAT	1	G	A
TCGA-29-1761	TTN_ENST0(	ENST00000342992	p.V25798I	c.77392G>A	Unverified	GAC	GTT	ATT	1	G	A
TCGA-61-1906	TTN_ENST0(	ENST00000342992	p.G3938R	c.11812G>A	Unverified	GGA	GGA	CAA	1	G	A
TCGA-61-1906	TTN_ENST0(	ENST00000342992	p.G3938R	c.11812G>A	Unverified	GGA	GGA	CAA	1	G	A
TCGA-61-1907	TTN_ENST0(	ENST00000342992	p.G7484S	c.22450G>A	Unverified	GTT	GGT	TCC	1	G	A
TCGA-09-1674	TTN_ENST0(	ENST00000342992	p.R12535Q	c.37604G>A	Unverified	TGT	CGA	CTC	2	G	A
TCGA-09-1674	TTN_ENST0(	ENST00000356127	p.G21005S	c.63013G>A	Unverified	AAA	GGC	TCT	1	G	A
TCGA-29-1711	TTN_ENST0(	ENST00000356127	p.G1371R	c.4111G>A	Unverified	AAA	GGA	AAT	1	G	A
TCGA-29-1711	TTN_ENST0(	ENST00000356127	p.G1371R	c.4111G>A	Unverified	AAA	GGA	AAT	1	G	A
TCGA-29-1761	TTN_ENST0(	ENST00000356127	p.V25796I	c.77386G>A	Unverified	GAC	GTT	ATT	1	G	A
TCGA-29-2427	TTN_ENST0(	ENST00000356127	p.V911I	c.2731G>A	Verified	ACC	GTC	CGT	1	G	A
TCGA-61-2012	TTN_ENST0(	ENST00000356127	p.A18280T	c.54838G>A	Verified	ACG	GCT	GGC	1	G	A
TCGA-13-0885	TTN_ENST0(	ENST00000356127	p.E29896K	c.89686G>A	Verified	ACC	GAA	GGA	1	G	A
TCGA-61-2094	TTN_ENST0(	ENST00000356127	p.E23314K	c.69940G>A	Unverified	ATC	GAA	ATT	1	G	A
TCGA-09-1674	TTN_ENST0(	ENST00000356127	p.R12535Q	c.37604G>A	Unverified	TGT	CGA	CTC	2	G	A
TCGA-13-1496	TTN_ENST0(	ENST00000356127	p.R6745H	c.20234G>A	Verified	ATC	CGC	AAG	2	G	A
TCGA-61-2095	TTN_ENST0(	ENST00000356127	p.Q23538Q	c.70614G>A	Verified	TTA	CAG	TGG	3	G	A
TCGA-09-1674	TTN_ENST0(	ENST00000359218	p.G14635S	c.43903G>A	Unverified	AAA	GGC	TCT	1	G	A
TCGA-29-1711	TTN_ENST0(	ENST00000359218	p.G1325R	c.3973G>A	Unverified	AAA	GGA	AAT	1	G	A
TCGA-29-1711	TTN_ENST0(	ENST00000359218	p.G1325R	c.3973G>A	Unverified	AAA	GGA	AAT	1	G	A
TCGA-29-1761	TTN_ENST0(	ENST00000359218	p.V19426I	c.58276G>A	Unverified	GAC	GTT	ATT	1	G	A
TCGA-09-1674	TTN_ENST0(	ENST00000359218	p.R6163Q	c.18488G>A	Unverified	TGT	CGA	CTC	2	G	A
TCGA-29-1711	TTN_ENST0(	ENST00000360870	p.G1371R	c.4111G>A	Unverified	AAA	GGA	AAT	1	G	A
TCGA-29-1711	TTN_ENST0(	ENST00000360870	p.G1371R	c.4111G>A	Unverified	AAA	GGA	AAT	1	G	A

TCGA-29-2427	TTN_ENST0(	ENST00000360870	p.V911I	c.2731G>A	Verified	ACC	GTC	CGT	1	G	A
TCGA-04-1542	TTN_ENST0(	ENST00000360870	p.K3761K	c.11283G>A	Verified	TAT	AAG	GTA	3	G	A
TCGA-29-1763	TTPA	ENST00000260116	p.V126I	c.376G>A	Unverified	AAA	GTT	TTT	1	G	A
TCGA-24-1464	TUBA4A	ENST00000248437	p.R79Q	c.236G>A	Verified	ATC	CGA	AAT	2	G	A
TCGA-24-1464	TUBA4A_EN	ENST00000392088	p.R64Q	c.191G>A	Verified	ATC	CGA	AAT	2	G	A
TCGA-23-1117	TUBB3	ENST00000315491	p.G71R	c.211G>A	Verified	CCC	GGA	ACC	1	G	A
TCGA-23-1117	TUBB3	ENST00000315491	p.G71R	c.211G>A	Verified	CCC	GGA	ACC	1	G	A
TCGA-25-2398	TUBB4	ENST00000264071	p.E405K	c.1213G>A	Verified	GAC	GAG	ATG	1	G	A
TCGA-29-1770	TUBB8	ENST00000328974	p.R276Q	c.827G>A	Unverified	AGC	CGG	GGC	2	G	A
TCGA-61-1738	TULP4	ENST00000367097	p.A1057T	c.3169G>A	Unverified	ACC	GCC	AGC	1	G	A
TCGA-61-1738	TULP4	ENST00000367097	p.A1057T	c.3169G>A	Unverified	ACC	GCC	AGC	1	G	A
TCGA-29-1761	TYK2	ENST00000525621	p.S973S	c.2919G>A	Unverified	AAG	TCG	CTG	3	G	A
TCGA-61-1740	TYW3	ENST00000370867	p.R228H	c.683G>A	Unverified	ACA	CGT	GCC	2	G	A
TCGA-20-0990	UBA3	ENST00000361055	p.C249Y	c.746G>A	Verified	cac	tgt	att	2	G	A
TCGA-29-1693	UBA52	ENST00000442744	p.R54H	c.161G>A	Unverified	GGC	CGC	ACT	2	G	A
TCGA-23-1111	UBA7	ENST00000333486	p.R204H	c.611G>A	Unverified	ttc	cgt	gat	2	G	A
TCGA-13-0920	UBAP2	ENST00000379238	p.V344I	c.1030G>A	Verified	GCC	GTC	AAC	1	G	A
TCGA-24-1470	UBASH3A	ENST00000319294	p.P332P	c.996G>A	Verified	CTG	CCG	GAA	3	G	A
TCGA-24-1435	UBC	ENST00000339647	p.D52N	c.154G>A	Verified	GAA	GAT	GGG	1	G	A
TCGA-61-1907	UBE2D3	ENST00000357194	p.R17H	c.50G>A	Unverified	gcc	cgt	gac	2	G	A
TCGA-61-1907	UBE2D3_EN	ENST00000343106	p.R15H	c.44G>A	Unverified	GCC	CGT	GAC	2	G	A
TCGA-20-1686	UBE2Q1	ENST00000292211	p.G359D	c.1076G>A	Unverified	cag	ggc	tgg	2	G	A
TCGA-04-1361	UBE3C	ENST00000348165	p.E836E	c.2508G>A	Verified	gtg	gag	ctg	3	G	A
TCGA-04-1361	UBE3C	ENST00000348165	p.E836E	c.2508G>A	Verified	gtg	gag	ctg	3	G	A
TCGA-13-0900	UBLCP1	ENST00000296786	p.V234I	c.700G>A	Verified	GGT	GTT	ATA	1	G	A
TCGA-23-1809	UBN2	ENST00000288561	p.M1034I	c.3102G>A	Unverified	GGA	ATG	AAC	3	G	A
TCGA-29-2427	UBOX5	ENST00000217173	p.L217L	c.651G>A	Verified	aac	ctg	cct	3	G	A
TCGA-23-1029	UBR3	ENST00000392632	p.G172R	c.514G>A	Unverified	GAA	GGA	GGT	1	G	A
TCGA-23-1029	UBR3_ENST(	ENST00000418381	p.G1319R	c.3955G>A	Unverified	GAA	GGA	GGT	1	G	A
TCGA-13-0920	UBR4	ENST00000375254	p.T3554T	c.10662G>A	Verified	GAC	ACG	CGG	3	G	A
TCGA-61-1738	UBR5	ENST00000520539	p.V2687M	c.8059G>A	Unverified	aat	gtg	caa	1	G	A
TCGA-61-1738	UBR5	ENST00000520539	p.V2687M	c.8059G>A	Unverified	aat	gtg	caa	1	G	A
TCGA-13-0807	UBXN10	ENST00000375099	p.E13E	c.39G>A	Verified	CCT	GAG	TGT	3	G	A
TCGA-09-2050	UBXN2B	ENST00000399598	p.D252N	c.754G>A	Verified	GAT	GAT	TCA	1	G	A

TCGA-30-1718	UBXN6	ENST00000301281	p.E51K	c.151G>A	Unverified	AAT	GAG	GCA	1	G	A
TCGA-24-1469	UGCG	ENST00000374279	p.A242A	c.726G>A	Verified	AAA	GCG	ATA	3	G	A
TCGA-24-1845	UGGT1	ENST00000259253	p.V590I	c.1768G>A	Unverified	GTG	GTC	AGT	1	G	A
TCGA-29-1703	UGGT2	ENST00000376747	p.A1493T	c.4477G>A	Unverified	GAT	GCT	GAG	1	G	A
TCGA-23-1021	UGT1A9	ENST00000354728	p.E178K	c.532G>A	Verified	ctt	gaa	gaa	1	G	A
TCGA-29-1699	UGT2B28	ENST00000335568	p.V508I	c.1522G>A	Unverified	GTC	GTC	ACA	1	G	A
TCGA-23-1117	UIMC1	ENST00000377227	p.R122Q	c.365G>A	Verified	TGC	CGG	CCT	2	G	A
TCGA-23-1117	UIMC1	ENST00000377227	p.R122Q	c.365G>A	Verified	TGC	CGG	CCT	2	G	A
TCGA-13-0924	UNC13A	ENST00000428389	p.A125T	c.373G>A	Verified	ATC	GCG	GTG	1	G	A
TCGA-13-0795	UNC13A	ENST00000428389	p.M1183I	c.3549G>A	Verified	GAC	ATG	AAG	3	G	A
TCGA-13-0760	UNC13B	ENST00000378495	p.V1514M	c.4540G>A	Verified	TGC	GTG	AAG	1	G	A
TCGA-20-1687	UNC13C	ENST00000260323	p.Q1020Q	c.3060G>A	Unverified	CTC	CAG	GTG	3	G	A
TCGA-20-1687	UNC13C_EN	ENST00000260323	p.Q1020Q	c.3060G>A	Unverified	CTC	CAG	GTG	3	G	A
TCGA-30-1891	UNC5B	ENST00000335350	p.G287D	c.860G>A	Unverified	GAG	GGC	CAG	2	G	A
TCGA-29-1766	UNC5C	ENST00000453304	p.V356I	c.1066G>A	Unverified	CTC	GTC	TTG	1	G	A
TCGA-25-2392	UNC80	ENST00000281753	p.R253Q	c.758G>A	Unverified	AGC	CGG	ACC	2	G	A
TCGA-23-1122	UNQ1887	ENST00000353487	p.A338T	c.1012G>A	Verified	CGG	GCC	GCC	1	G	A
TCGA-25-1313	URB2	ENST00000258243	p.G955R	c.2863G>A	Verified	AAG	GGG	AAA	1	G	A
TCGA-24-1422	URGCP	ENST00000223341	p.V95M	c.283G>A	Verified	GAC	GTG	CTC	1	G	A
TCGA-24-0979	URGCP	ENST00000223341	p.G349G	c.1047G>A	Verified	CGT	GGG	AAG	3	G	A
TCGA-29-1693	UROC1	ENST00000290868	p.E302K	c.904G>A	Unverified	AGG	GAA	GCA	1	G	A
TCGA-29-1693	UROC1_ENS	ENST00000383579	p.E362K	c.1084G>A	Unverified	AGG	GAA	GCA	1	G	A
TCGA-24-1843	UROD	ENST00000246337	p.V321M	c.961G>A	Unverified	TTG	GTG	AAG	1	G	A
TCGA-23-1022	USH2A	ENST00000307340	p.G1911R	c.5731G>A	Verified	CAG	GGA	AAA	1	G	A
TCGA-13-1501	USH2A	ENST00000307340	p.S1088N	c.3263G>A	Verified	TAC	AGT	TTA	2	G	A
TCGA-13-1501	USH2A	ENST00000307340	p.S1088N	c.3263G>A	Verified	TAC	AGT	TTA	2	G	A
TCGA-23-1111	USH2A	ENST00000307340	p.R1578H	c.4733G>A	Unverified	GGA	CGT	CTT	2	G	A
TCGA-23-1809	USH2A	ENST00000307340	p.R4120H	c.12359G>A	Unverified	TTC	CGC	CGC	2	G	A
TCGA-09-2056	USH2A	ENST00000307340	p.R4302R	c.12906G>A	Verified	CAA	AGG	AAT	3	G	A
TCGA-13-0755	USHBP1	ENST00000252597	p.R431H	c.1292G>A	Verified	CAG	CGT	CTC	2	G	A
TCGA-23-1110	USHBP1	ENST00000252597	p.Q539Q	c.1617G>A	Unverified	CTC	CAG	GCT	3	G	A
TCGA-13-1498	USP11	ENST00000218348	p.V455M	c.1363G>A	Verified	atc	gtg	gac	1	G	A
TCGA-04-1638	USP13	ENST00000263966	p.G13S	c.37G>A	Unverified	GGC	GGC	AGC	1	G	A
TCGA-25-2042	USP17L2	ENST00000333796	p.D332N	c.994G>A	Unverified	CAC	GAC	GGA	1	G	A

TCGA-23-1021	USP19	XM_496642.1	p.G1144S	c.3430G>A	Verified	gcc	ggc	cac	1	G	A
TCGA-13-1488	USP19	XM_496642.1	p.E514E	c.1542G>A	Verified	gtg	gag	aag	3	G	A
TCGA-29-1770	USP2	ENST00000260187	p.G109R	c.325G>A	Unverified	AGC	GGG	GGC	1	G	A
TCGA-29-1776	USP20	ENST00000372429	p.A502T	c.1504G>A	Unverified	GGC	GCC	TGT	1	G	A
TCGA-29-1776	USP20_ENST	ENST00000315480	p.A502T	c.1504G>A	Unverified	GGC	GCC	TGT	1	G	A
TCGA-29-1776	USP20_ENST	ENST00000372429	p.A502T	c.1504G>A	Unverified	GGC	GCC	TGT	1	G	A
TCGA-04-1651	USP28	ENST00000003302	p.W888*	c.2663G>A	Unverified	AAG	TGG	CAT	2	G	A
TCGA-04-1651	USP28	ENST00000003302	p.W888*	c.2663G>A	Unverified	AAG	TGG	CAT	2	G	A
TCGA-61-1900	USP29	ENST00000254181	p.G352G	c.1056G>A	Unverified	ctt	ggg	aat	3	G	A
TCGA-30-1891	USP31	ENST00000219689	p.A857T	c.2569G>A	Unverified	TTG	GCC	GTC	1	G	A
TCGA-04-1343	USP31	ENST00000219689	p.E1098E	c.3294G>A	Verified	AAG	GAG	TCA	3	G	A
TCGA-29-1691	USP37	ENST00000258399	p.R976H	c.2927G>A	Unverified	ACC	CGT	CAG	2	G	A
TCGA-09-2050	USP37	ENST00000258399	p.E874E	c.2622G>A	Verified	GCT	GAG	GAA	3	G	A
TCGA-04-1338	USP47	ENST00000339865	p.V781V	c.2343G>A	Verified	TCT	GTG	GAA	3	G	A
TCGA-29-1784	USP49	ENST00000373006	p.G614E	c.1841G>A	Unverified	TCA	GGA	CAC	2	G	A
TCGA-04-1347	USP51	ENST00000500968	p.V643V	c.1929G>A	Verified	tgt	gtg	ccc	3	G	A
TCGA-13-0906	USP54	NM_152586.2	p.E48K	c.142G>A	Unverified	gtt	gaa	gta	1	G	A
TCGA-13-0906	USP54_ENST	ENST00000408019	p.E960K	c.2878G>A	Unverified	GTT	GAA	GTA	1	G	A
TCGA-13-1408	USP6	NM_004505.1	p.V148V	c.444G>A	Unverified	gac	gtg	agg	3	G	A
TCGA-13-1408	USP6_ENST(	ENST00000250066	p.V148V	c.444G>A	Unverified	GAC	GTG	AGG	3	G	A
TCGA-20-1685	USPL1	ENST00000255304	p.C97Y	c.290G>A	Unverified	GAA	TGT	CAC	2	G	A
TCGA-61-1899	VAC14	ENST00000261776	p.E258K	c.772G>A	Unverified	gct	gag	atg	1	G	A
TCGA-24-1464	VARS2	ENST00000321897	p.V828M	c.2482G>A	Verified	CCC	GTG	CTG	1	G	A
TCGA-04-1638	VAT1L	ENST00000302536	p.E410K	c.1228G>A	Unverified	AGC	GAG	AAC	1	G	A
TCGA-29-1696	VCAM1	ENST00000294728	p.W258*	c.774G>A	Unverified	TTC	TGG	AGT	3	G	A
TCGA-29-1696	VCAM1	ENST00000294728	p.W258*	c.774G>A	Unverified	TTC	TGG	AGT	3	G	A
TCGA-61-2012	VCAN	ENST00000265077	p.V2685I	c.8053G>A	Verified	GAC	GTT	TTA	1	G	A
TCGA-61-2094	VIL1	ENST00000248444	p.Q290Q	c.870G>A	Unverified	GAC	CAG	GGG	3	G	A
TCGA-09-2049	VNN1	ENST00000367928	p.G477G	c.1431G>A	Verified	TTT	GGG	AGG	3	G	A
TCGA-13-0885	VPS11	ENST00000300793	p.G565E	c.1694G>A	Verified	AAG	GGA	CTT	2	G	A
TCGA-23-1118	VPS18	ENST00000220509	p.A686T	c.2056G>A	Verified	CAG	GCT	GGG	1	G	A
TCGA-04-1338	VPS52	ENST00000445902	p.S657N	c.1970G>A	Verified	CTG	AGT	CAG	2	G	A
TCGA-61-1910	VRK3	ENST00000316763	p.E150K	c.448G>A	Unverified	ctt	gaa	gct	1	G	A
TCGA-24-2289	VRTN	ENST00000256362	p.L113L	c.339G>A	Unverified	ATG	CTG	CTG	3	G	A

TCGA-23-1118	VSX2	ENST00000261980	p.E169K	c.505G>A	Verified	AAC	GAA	GCC	1	G	A
TCGA-20-1686	WAS	ENST00000376701	p.V141M	c.421G>A	Unverified	ctc	gtg	cag	1	G	A
TCGA-29-1701	WASF2	ENST00000430629	p.T5T	c.15G>A	Unverified	gta	acg	agg	3	G	A
TCGA-04-1530	WBP2NL	ENST00000329620	p.A254T	c.760G>A	Unverified	GGA	GCC	CCA	1	G	A
TCGA-29-1693	WDFY2	ENST00000298125	p.R329H	c.986G>A	Unverified	AAG	CGC	TCC	2	G	A
TCGA-13-0791	WDR13	ENST00000376729	p.G150R	c.448G>A	Verified	GCC	GGG	GAC	1	G	A
TCGA-23-1114	WDR13	ENST00000376729	p.R19H	c.56G>A	Unverified	TAC	CGC	ACA	2	G	A
TCGA-23-1114	WDR13_ENS	ENST00000218056	p.R19H	c.56G>A	Unverified	TAC	CGC	ACA	2	G	A
TCGA-24-1843	WDR16	ENST00000352665	p.Q451Q	c.1353G>A	Unverified	ACC	CAG	AAG	3	G	A
TCGA-13-1509	WDR24	ENST00000293883	p.T122T	c.366G>A	Verified	CGC	ACG	GTA	3	G	A
TCGA-29-1691	WDR3	ENST00000349139	p.D319N	c.955G>A	Unverified	ATG	GAT	AAG	1	G	A
TCGA-13-0924	WDR35	ENST00000345530	p.R472Q	c.1415G>A	Verified	TCT	CGA	AAA	2	G	A
TCGA-29-1699	WDR37	ENST00000358220	p.?	c.1239-1G>A	Unverified	AAC	AGG	ATC	3	G	A
TCGA-13-0893	WDR48	ENST00000302313	p.R13R	c.39G>A	Verified	CGG	AGG	AAA	3	G	A
TCGA-61-1895	WDR61	ENST00000267973	p.?	c.829-1G>A	Unverified	CAG	GTC	TGG	1	G	A
TCGA-29-1691	WDR63	ENST00000294664	p.D399N	c.1195G>A	Unverified	GAT	GAC	ATC	1	G	A
TCGA-29-1785	WDR64	ENST00000341258	p.G385R	c.1153G>A	Unverified	GCT	GGA	ACC	1	G	A
TCGA-29-1785	WDR64	ENST00000341258	p.G385R	c.1153G>A	Unverified	GCT	GGA	ACC	1	G	A
TCGA-29-1785	WDR64_ENS	ENST00000366552	p.G665R	c.1993G>A	Unverified	GCT	GGA	ACC	1	G	A
TCGA-29-1785	WDR64_ENS	ENST00000366552	p.G665R	c.1993G>A	Unverified	GCT	GGA	ACC	1	G	A
TCGA-23-2078	WDR7	ENST00000254442	p.?	c.863+1G>A	Unverified	GCC	AGT	TGC	2	G	A
TCGA-30-1718	WDR75	ENST00000314761	p.R276H	c.827G>A	Unverified	TGG	CGC	GAT	2	G	A
TCGA-13-0795	WDR93	ENST00000268130	p.G316R	c.946G>A	Verified	TCC	GGG	CAG	1	G	A
TCGA-23-1124	WDR96	ENST00000357060	p.G492R	c.1474G>A	Verified	GTT	GGA	ACA	1	G	A
TCGA-25-2393	WDR96	ENST00000357060	p.V70I	c.208G>A	Verified	GGC	GTC	ATG	1	G	A
TCGA-23-1022	WDTC1	ENST00000361771	p.D663N	c.1987G>A	Verified	GAT	GAT	GAG	1	G	A
TCGA-24-2280	WHSC1	ENST00000382891	p.E339E	c.1017G>A	Unverified	GAG	GAG	CGG	3	G	A
TCGA-20-0990	WNT9A	ENST00000272164	p.A264T	c.790G>A	Unverified	GAG	GCA	GGT	1	G	A
TCGA-23-1124	WWC1	ENST00000265293	p.Q1062Q	c.3186G>A	Verified	CTT	CAG	ACA	3	G	A
TCGA-09-2050	WWC3	NM_015691.2	p.V184I	c.550G>A	Verified	tcc	gtt	acc	1	G	A
TCGA-04-1652	XCR1	ENST00000309285	p.A33T	c.97G>A	Unverified	CTC	GCC	ACC	1	G	A
TCGA-04-1652	XCR1	ENST00000309285	p.A33T	c.97G>A	Unverified	CTC	GCC	ACC	1	G	A
TCGA-13-1510	XPOT	ENST00000332707	p.A654T	c.1960G>A	Unverified	cat	gct	gtt	1	G	A
TCGA-29-1784	XRN2	ENST00000377191	p.S499N	c.1496G>A	Unverified	GAC	AGT	GAC	2	G	A

TCGA-13-0883	XYLT1	ENST00000261381	p.D413N	c.1237G>A	Verified	ACC	GAC	TGG	1	G	A
TCGA-13-0886	YLPM1	ENST00000238571	p.S851N	c.2552G>A	Verified	ATC	AGT	CGA	2	G	A
TCGA-13-0920	YTHDF1	ENST00000370339	p.V200I	c.598G>A	Verified	GCC	GTC	AAG	1	G	A
TCGA-30-1891	ZBTB10	ENST00000455036	p.W686*	c.2057G>A	Unverified	ACC	TGG	GAA	2	G	A
TCGA-61-1907	ZBTB25	ENST00000394715	p.A111T	c.331G>A	Unverified	ATT	GCA	ACT	1	G	A
TCGA-23-1809	ZBTB3	ENST00000394807	p.L453L	c.1359G>A	Unverified	TAC	CTG	TCT	3	G	A
TCGA-13-0885	ZBTB38	ENST00000514251	p.G652D	c.1955G>A	Verified	GAG	GGT	ACC	2	G	A
TCGA-25-2400	ZBTB48	ENST00000377674	p.M397I	c.1191G>A	Unverified	CAC	ATG	ATC	3	G	A
TCGA-23-1032	ZBTB7B	ENST00000292176	p.D155N	c.463G>A	Unverified	GAT	GAC	TGT	1	G	A
TCGA-13-0905	ZC3H12A	ENST00000373082	p.S555N	c.1664G>A	Verified	GCC	AGC	GTG	2	G	A
TCGA-24-1844	ZC3H13	ENST00000282007	p.R361Q	c.1082G>A	Unverified	CAG	CGG	ACA	2	G	A
TCGA-61-1906	ZC3H14	ENST00000251038	p.R505Q	c.1514G>A	Unverified	ACA	CGA	GAT	2	G	A
TCGA-61-1906	ZC3H14	ENST00000251038	p.R505Q	c.1514G>A	Unverified	ACA	CGA	GAT	2	G	A
TCGA-24-1431	ZC3H4	ENST00000253048	p.A1156T	c.3466G>A	Unverified	AAC	GCG	GGG	1	G	A
TCGA-13-0924	ZC3H6	ENST00000409871	p.E283K	c.847G>A	Verified	CTG	GAA	GGG	1	G	A
TCGA-04-1362	ZCCHC12	ENST00000310164	p.V149V	c.447G>A	Verified	tat	gtg	atc	3	G	A
TCGA-23-1021	ZDBF2	ENST00000374423	p.E888K	c.2662G>A	Verified	CCC	GAA	GTA	1	G	A
TCGA-24-2280	ZDHHC12	ENST00000372663	p.W22*	c.65G>A	Unverified	ACC	TGG	GGA	2	G	A
TCGA-04-1331	ZDHHC15	ENST00000373367	p.V40I	c.118G>A	Verified	tac	gtc	ttt	1	G	A
TCGA-13-0906	ZEB2	ENST00000303660	p.R779K	c.2336G>A	Verified	tcc	agg	agt	2	G	A
TCGA-13-1499	ZFP106	ENST00000263805	p.E627K	c.1879G>A	Verified	GAG	GAG	GAT	1	G	A
TCGA-23-1022	ZFP37	ENST00000374227	p.R20K	c.59G>A	Verified	AGG	AGA	AGT	2	G	A
TCGA-24-1846	ZFP41	ENST00000330701	p.R94Q	c.281G>A	Unverified	GGG	CGG	ATC	2	G	A
TCGA-04-1638	ZFYVE1	ENST00000556143	p.V692M	c.2074G>A	Unverified	GCC	GTG	GTG	1	G	A
TCGA-29-1705	ZHX2	ENST00000314393	p.R561Q	c.1682G>A	Unverified	gat	cgg	cta	2	G	A
TCGA-61-1737	ZIC5	ENST00000267294	p.A631T	c.1891G>A	Unverified	GGG	GCC	CCC	1	G	A
TCGA-04-1337	ZIM2_ENST(	ENST00000423103	p.R598H	c.1793G>A	Unverified	GAA	CGT	GAA	2	G	A
TCGA-23-1118	ZKSCAN1	ENST00000324306	p.R193Q	c.578G>A	Verified	TCA	CGA	GCT	2	G	A
TCGA-24-1563	ZKSCAN2	ENST00000328086	p.K506K	c.1518G>A	Verified	ACC	AAG	ACT	3	G	A
TCGA-24-2289	ZMAT1	NM_032441.1	p.R425Q	c.1274G>A	Verified	cat	cga	aaa	2	G	A
TCGA-13-0714	ZMYM2	ENST00000456228	p.V1028I	c.3082G>A	Verified	CCT	GTT	TTT	1	G	A
TCGA-24-1422	ZMYM4	ENST00000314607	p.D717N	c.2149G>A	Verified	TCT	GAT	GAA	1	G	A
TCGA-24-1435	ZMYND17	ENST00000372912	p.P225P	c.675G>A	Verified	GAC	CCG	GAT	3	G	A
TCGA-29-1777	ZMYND8	ENST00000461685	p.A504T	c.1510G>A	Unverified	caa	gca	ggg	1	G	A

TCGA-24-1846	ZNF182	ENST00000396965	p.P85P	c.255G>A	Unverified	tgc	ccg	gca	3	G	A
TCGA-24-1846	ZNF182_EN!	ENST00000376943	p.P66P	c.198G>A	Unverified	TGC	CCG	GCA	3	G	A
TCGA-25-2398	ZNF212	ENST00000335870	p.L484L	c.1452G>A	Verified	GGG	CTG	GCC	3	G	A
TCGA-25-2401	ZNF236	ENST00000253159	p.V1315I	c.3943G>A	Verified	TCT	GTT	CTA	1	G	A
TCGA-13-1499	ZNF236	ENST00000253159	p.G90G	c.270G>A	Verified	AGC	GGG	GAA	3	G	A
TCGA-13-1512	ZNF238	ENST00000358704	p.V273M	c.817G>A	Verified	GAC	GTG	CTG	1	G	A
TCGA-13-0924	ZNF264	ENST00000263095	p.D87N	c.259G>A	Verified	GGC	GAC	AAA	1	G	A
TCGA-13-0916	ZNF266	ENST00000361151	p.E300K	c.898G>A	Verified	AAG	GAA	TGT	1	G	A
TCGA-09-2049	ZNF280A	ENST00000302097	p.M101I	c.303G>A	Verified	ATC	ATG	CCC	3	G	A
TCGA-61-2113	ZNF280B	ENST00000360412	p.E370E	c.1110G>A	Verified	CAG	GAG	CCC	3	G	A
TCGA-13-0893	ZNF280D	ENST00000267807	p.E378K	c.1132G>A	Verified	ATC	GAA	AGT	1	G	A
TCGA-23-1114	ZNF282	ENST00000262085	p.M61I	c.183G>A	Unverified	GAC	ATG	GAC	3	G	A
TCGA-29-1705	ZNF283_EN!	ENST00000324461	p.G577G	c.1731G>A	Unverified	TGT	GGG	AAG	3	G	A
TCGA-29-1705	ZNF295	ENST00000398499	p.M917I	c.2751G>A	Unverified	AAG	ATG	TTC	3	G	A
TCGA-24-2267	ZNF318	ENST00000361428	p.D1611N	c.4831G>A	Verified	TCA	GAC	ACC	1	G	A
TCGA-24-2262	ZNF335	ENST00000322927	p.R443R	c.1329G>A	Unverified	TCC	AGG	CGC	3	G	A
TCGA-24-1469	ZNF345	ENST00000420450	p.G86S	c.256G>A	Verified	ACT	GGT	GAG	1	G	A
TCGA-23-2078	ZNF35	ENST00000396056	p.G480G	c.1440G>A	Unverified	TGT	GGG	AAG	3	G	A
TCGA-24-1435	ZNF354B	ENST00000322434	p.C216Y	c.647G>A	Verified	AAA	TGC	AGT	2	G	A
TCGA-61-2102	ZNF383	ENST00000352998	p.M66I	c.198G>A	Verified	TGG	ATG	GTT	3	G	A
TCGA-24-1417	ZNF425	ENST00000378061	p.C315Y	c.944G>A	Unverified	CAG	TGC	GAG	2	G	A
TCGA-24-1563	ZNF429	ENST00000358491	p.C539Y	c.1616G>A	Verified	AAA	TGT	GAA	2	G	A
TCGA-29-1691	ZNF434	ENST00000304926	p.G368E	c.1103G>A	Unverified	TGT	GGG	CAA	2	G	A
TCGA-13-1481	ZNF462	ENST00000277225	p.A1171T	c.3511G>A	Verified	CCC	GCC	CCC	1	G	A
TCGA-13-0886	ZNF471	ENST00000308031	p.E540E	c.1620G>A	Verified	GGA	GAG	AAA	3	G	A
TCGA-29-1761	ZNF473	ENST00000391821	p.E166K	c.496G>A	Unverified	GGA	GAA	GAT	1	G	A
TCGA-20-0990	ZNF479	ENST00000331162	p.E29K	c.85G>A	Verified	GAG	GAA	TGG	1	G	A
TCGA-29-1702	ZNF479	ENST00000331162	p.Q101Q	c.303G>A	Unverified	GAG	CAG	GGC	3	G	A
TCGA-23-1116	ZNF488	ENST00000395702	p.R334Q	c.1001G>A	Unverified	TCC	CGG	CAC	2	G	A
TCGA-04-1655	ZNF492_EN!	ENST00000456783	p.C482Y	c.1445G>A	Unverified	GAA	TGT	GGC	2	G	A
TCGA-09-1665	ZNF500	ENST00000219478	p.D364N	c.1090G>A	Verified	AGC	GAC	CGC	1	G	A
TCGA-29-1785	ZNF516	ENST00000217537	p.S181N	c.542G>A	Unverified	AAG	AGC	CAG	2	G	A
TCGA-29-1785	ZNF516	ENST00000217537	p.S181N	c.542G>A	Unverified	AAG	AGC	CAG	2	G	A
TCGA-13-1512	ZNF521	NM_015461.1	p.R967Q	c.2900G>A	Verified	gag	cgg	ttt	2	G	A

TCGA-29-1699	ZNF528	ENST00000360465	p.R426Q	c.1277G>A	Unverified	ATA	CGA	CAT	2	G	A
TCGA-30-1855	ZNF536	ENST00000355537	p.G637S	c.1909G>A	Unverified	TGC	GGC	CGG	1	G	A
TCGA-13-0883	ZNF565	ENST00000304116	p.G285S	c.853G>A	Verified	TGT	GGC	AAG	1	G	A
TCGA-29-1703	ZNF574	ENST00000359044	p.G737S	c.2209G>A	Unverified	CGG	GGT	CTA	1	G	A
TCGA-13-0755	ZNF585A	ENST00000392157	p.G463G	c.1389G>A	Verified	ACT	GGG	GAG	3	G	A
TCGA-24-1844	ZNF587	ENST00000339656	p.P6P	c.18G>A	Unverified	GTG	CCG	AGG	3	G	A
TCGA-23-1032	ZNF608	ENST00000306315	p.R479Q	c.1436G>A	Unverified	GGA	CGA	AGG	2	G	A
TCGA-24-1435	ZNF609	ENST00000326648	p.R124H	c.371G>A	Verified	GGG	CGC	TCA	2	G	A
TCGA-13-0913	ZNF610	ENST00000327920	p.V308I	c.922G>A	Verified	CTT	GTA	ATC	1	G	A
TCGA-61-1725	ZNF613	ENST00000293471	p.E593K	c.1777G>A	Unverified	GCA	GAG	AGC	1	G	A
TCGA-13-0755	ZNF614	ENST00000270649	p.E162K	c.484G>A	Verified	GGT	GAG	AAA	1	G	A
TCGA-13-1488	ZNF619	ENST00000314686	p.G112R	c.334G>A	Verified	GAG	GGA	CTG	1	G	A
TCGA-23-1118	ZNF649	ENST00000354957	p.W19*	c.56G>A	Verified	ACC	TGG	GAG	2	G	A
TCGA-04-1649	ZNF653	ENST00000293771	p.T226T	c.678G>A	Unverified	GCG	ACG	CCC	3	G	A
TCGA-61-1900	ZNF669	ENST00000343381	p.G228R	c.682G>A	Unverified	TGT	GGG	AAA	1	G	A
TCGA-23-2077	ZNF670	ENST00000366503	p.E244K	c.730G>A	Verified	CAT	GAA	AGA	1	G	A
TCGA-13-1509	ZNF687	ENST00000324048	p.E169K	c.505G>A	Verified	CCT	GAG	CCA	1	G	A
TCGA-23-1117	ZNF691	ENST00000372506	p.E15K	c.43G>A	Verified	CCT	GAG	GAA	1	G	A
TCGA-23-1117	ZNF691	ENST00000372506	p.E15K	c.43G>A	Verified	CCT	GAG	GAA	1	G	A
TCGA-23-1032	ZNF697	ENST00000421812	p.T488T	c.1464G>A	Unverified	CAC	ACG	GGC	3	G	A
TCGA-13-0760	ZNF697	ENST00000421812	p.P53P	c.159G>A	Verified	CAT	CCG	GAG	3	G	A
TCGA-24-1417	ZNF7	ENST00000325241	p.E52E	c.156G>A	Unverified	CCT	GAG	CTG	3	G	A
TCGA-29-1770	ZNF74	ENST00000400451	p.G384S	c.1150G>A	Unverified	ACG	GGC	GAG	1	G	A
TCGA-24-1422	ZNF74	ENST00000400451	p.G127D	c.380G>A	Verified	CAG	GGC	ATT	2	G	A
TCGA-61-1907	ZNF750	ENST00000269394	p.C663Y	c.1988G>A	Unverified	GCC	TGC	CGG	2	G	A
TCGA-61-1737	ZNF772	ENST00000343280	p.A10T	c.28G>A	Unverified	CCG	GCA	CAG	1	G	A
TCGA-61-1895	ZNF790	ENST00000356725	p.V535I	c.1603G>A	Unverified	TAC	GTA	TGT	1	G	A
TCGA-29-1705	ZNF80	ENST00000308095	p.V108V	c.324G>A	Unverified	TGC	GTG	GAG	3	G	A
TCGA-09-1674	ZNF804A	ENST00000302277	p.E397K	c.1189G>A	Unverified	CCC	GAG	ACA	1	G	A
TCGA-29-1770	ZP1	ENST00000278853	p.R504Q	c.1511G>A	Unverified	CAG	CGA	TTC	2	G	A
TCGA-20-1683	ZP4	ENST00000366570	p.V85M	c.253G>A	Unverified	TCC	GTG	GTG	1	G	A
TCGA-61-1740	ZPLD1	ENST00000306176	p.G393R	c.1177G>A	Unverified	TCA	GGA	ATG	1	G	A
TCGA-24-2289	ZXDA	ENST00000358697	p.A392T	c.1174G>A	Unverified	tgc	gcg	ttt	1	G	A
TCGA-09-2049	AARS	ENST00000261772	p.D261H	c.781G>C	Unverified	ACT	GAC	CTT	1	G	C

TCGA-25-2392	AASS	ENST00000393376	p.R294P	c.881G>C	Unverified	AGT	CGT	TTT	2	G	C
TCGA-24-1469	ABCA12	ENST00000272895	p.R1095P	c.3284G>C	Verified	CTC	CGG	CTT	2	G	C
TCGA-24-1846	ABCA13_EN	ENST00000435803	p.D1093H	c.3277G>C	Unverified	GAA	GAC	CTA	1	G	C
TCGA-30-1856	ABCA8	ENST00000269080	p.G1470A	c.4409G>C	Unverified	TTT	GGC	AAA	2	G	C
TCGA-25-2393	ABCA9	ENST00000340001	p.L31F	c.93G>C	Verified	ACC	TTG	TTG	3	G	C
TCGA-13-0887	ABCC1	ENST00000399410	p.G700A	c.2099G>C	Verified	GAG	GGG	CAC	2	G	C
TCGA-13-1408	ABCC5	ENST00000334444	p.V1435L	c.4303G>C	Verified	GCT	GTC	AAG	1	G	C
TCGA-13-1481	ABHD2	ENST00000352732	p.C234S	c.701G>C	Verified	gtg	tgc	cag	2	G	C
TCGA-09-2051	ABL1	ENST00000318560	p.K87N	c.261G>C	Unverified	GAA	AAG	CTC	3	G	C
TCGA-29-1764	ABLIM2	ENST00000447017	p.K161N	c.483G>C	Unverified	ATC	AAG	AAT	3	G	C
TCGA-23-1022	ACAA2	ENST00000285093	p.R371P	c.1112G>C	Verified	AGG	CGT	CGA	2	G	C
TCGA-09-2056	ACCS	ENST00000263776	p.G221R	c.661G>C	Verified	ACT	GGG	CTA	1	G	C
TCGA-13-1497	ACLY	ENST00000352035	p.L43F	c.129G>C	Verified	cgc	ttg	ctg	3	G	C
TCGA-61-2095	ACRC	ENST00000373695	p.E263Q	c.787G>C	Unverified	TCG	GAA	GCT	1	G	C
TCGA-29-1783	ACTBL2	ENST00000423391	p.A261P	c.781G>C	Unverified	GAA	GCC	ATT	1	G	C
TCGA-13-1499	ACTL8	ENST00000375406	p.W328C	c.984G>C	Verified	GTC	TGG	GAG	3	G	C
TCGA-04-1655	ACTR6	ENST00000188312	p.V389L	c.1165G>C	Unverified	AGC	GTC	TGT	1	G	C
TCGA-23-1120	ACTR8	ENST00000335754	p.D525H	c.1573G>C	Unverified	TCT	GAC	GAC	1	G	C
TCGA-13-0884	ACVR1	ENST00000263640	p.L495F	c.1485G>C	Verified	ACT	TTG	ACC	3	G	C
TCGA-24-0979	ACVR1B	NM_020328	p.?	c.979+1G>C	Verified	caa	ggg	aag	1	G	C
TCGA-25-1318	ADAM22	ENST00000265727	p.G577R	c.1729G>C	Verified	GAA	GGG	ACG	1	G	C
TCGA-25-1318	ADAM22	ENST00000265727	p.G577R	c.1729G>C	Verified	GAA	GGG	ACG	1	G	C
TCGA-25-1318	ADAM22_EN	ENST00000398209	p.G577R	c.1729G>C	Verified	GAA	GGG	ACG	1	G	C
TCGA-25-1318	ADAM22_EN	ENST00000398209	p.G577R	c.1729G>C	Verified	GAA	GGG	ACG	1	G	C
TCGA-29-1693	ADAMTS1	ENST00000284984	p.W528S	c.1583G>C	Unverified	CCG	TGG	GCG	2	G	C
TCGA-04-1337	ADAMTS13	ENST00000371929	p.A822P	c.2464G>C	Unverified	GGA	GCA	GGC	1	G	C
TCGA-61-2109	ADAMTS14	NM_139155.1	p.V272L	c.814G>C	Unverified	gtg	gtt	cgc	1	G	C
TCGA-25-2400	ADAMTS19	NM_133638.1	p.Q889H	c.2667G>C	Verified	gat	cag	aat	3	G	C
TCGA-23-1124	ADAMTS6_E	ENST00000381055	p.D837H	c.2509G>C	Verified	GGA	GAT	AAT	1	G	C
TCGA-24-1470	ADC	ENST00000373443	p.E250Q	c.748G>C	Verified	TTT	GAA	GAG	1	G	C
TCGA-61-1737	ADCY8	ENST00000286355	p.K811N	c.2433G>C	Unverified	GAC	AAG	TCG	3	G	C
TCGA-20-1687	ADCY9	ENST00000294016	p.E353Q	c.1057G>C	Unverified	GAG	GAG	AGT	1	G	C
TCGA-23-1031	ADD2	ENST00000355733	p.G591A	c.1772G>C	Verified	TCT	GGT	CCG	2	G	C
TCGA-13-1488	ADD3	ENST00000356080	p.E580D	c.1740G>C	Verified	GCT	GAG	CAG	3	G	C

TCGA-24-1604	ADRBK2	ENST00000324198	p.R659P	c.1976G>C	Verified	TTG	CGT	CGT	2	G	C
TCGA-24-1846	ADSS	ENST00000366535	p.D131H	c.391G>C	Unverified	tct	gac	aga	1	G	C
TCGA-29-1764	AEBP1	ENST00000223357	p.E805D	c.2415G>C	Unverified	CAG	GAG	ACT	3	G	C
TCGA-13-1497	AFF3	ENST00000356421	p.D1117H	c.3349G>C	Verified	ATC	GAC	TAT	1	G	C
TCGA-23-1124	AGBL2	ENST00000298861	p.K858N	c.2574G>C	Verified	CCA	AAG	AGA	3	G	C
TCGA-61-1907	AGK	ENST00000355413	p.E186Q	c.556G>C	Unverified	GGA	GAG	ACA	1	G	C
TCGA-23-1117	AGXT2	ENST00000231420	p.R182T	c.545G>C	Verified	GCC	AGG	GCG	2	G	C
TCGA-23-1117	AGXT2	ENST00000231420	p.R182T	c.545G>C	Verified	GCC	AGG	GCG	2	G	C
TCGA-13-0885	AGXT2	ENST00000231420	p.L14L	c.42G>C	Verified	TGC	CTG	GTC	3	G	C
TCGA-24-1435	AHCTF1	ENST00000391829	p.G192A	c.575G>C	Verified	ACT	GGT	ATC	2	G	C
TCGA-29-1705	AHNAK	ENST00000378024	p.G202G	c.606G>C	Unverified	TCT	GGG	AAG	3	G	C
TCGA-04-1331	AHNAK2_EN	ENST00000333244	p.E430Q	c.1288G>C	Verified	CAG	GAG	ACA	1	G	C
TCGA-61-2109	AHNAK2_EN	ENST00000333244	p.V1848V	c.5544G>C	Unverified	GAT	GTG	TCT	3	G	C
TCGA-29-2427	AIFM1	ENST00000319908	p.K589N	c.1767G>C	Verified	att	aag	gac	3	G	C
TCGA-09-2051	AIM1	ENST00000369066	p.R1294T	c.3881G>C	Unverified	TAC	AGG	GAC	2	G	C
TCGA-61-1738	AK1	ENST00000373176	p.V182L	c.544G>C	Unverified	AGT	GTC	TTC	1	G	C
TCGA-61-1738	AK1	ENST00000373176	p.V182L	c.544G>C	Unverified	AGT	GTC	TTC	1	G	C
TCGA-23-1114	AKAP12	ENST00000402676	p.V115V	c.345G>C	Unverified	gat	gtg	agc	3	G	C
TCGA-13-1481	AKAP2	ENST00000374525	p.G484R	c.1450G>C	Verified	CCC	GGT	GCC	1	G	C
TCGA-23-1124	AKAP9	NM_147171.1	p.Q1174H	c.3522G>C	Verified	ctt	cag	aaa	3	G	C
TCGA-29-1784	AKAP9	NM_147171.1	p.K3723N	c.11169G>C	Unverified	cag	aag	aaa	3	G	C
TCGA-23-1124	AKAP9_ENS	ENST00000356239	p.Q1162H	c.3486G>C	Verified	CTT	CAG	AAA	3	G	C
TCGA-29-1784	AKAP9_ENS	ENST00000356239	p.K3719N	c.11157G>C	Unverified	CAG	AAG	AAA	3	G	C
TCGA-24-1104	ALAS1	ENST00000394965	p.S311S	c.933G>C	Verified	TCC	TCG	TGC	3	G	C
TCGA-24-1104	ALCAM	ENST00000306107	p.E460Q	c.1378G>C	Verified	ACA	GAG	GAA	1	G	C
TCGA-04-1651	ALG10B	ENST00000308742	p.Q235H	c.705G>C	Unverified	CTT	CAG	TTT	3	G	C
TCGA-04-1651	ALG10B	ENST00000308742	p.Q235H	c.705G>C	Unverified	CTT	CAG	TTT	3	G	C
TCGA-29-1691	ALG2	ENST00000476832	p.R378P	c.1133G>C	Unverified	ATC	CGT	GAA	2	G	C
TCGA-24-2267	ALKBH8_EN	ENST00000428149	p.W290C	c.870G>C	Verified	CTT	TGG	ACC	3	G	C
TCGA-24-2290	ALLC	ENST00000252505	p.A115P	c.343G>C	Unverified	GCT	GCA	GCC	1	G	C
TCGA-24-2290	ALLC	ENST00000252505	p.A115P	c.343G>C	Unverified	GCT	GCA	GCC	1	G	C
TCGA-10-0930	ALPK2	NM_052947	p.E41Q	c.121G>C	Verified	tca	gag	cct	1	G	C
TCGA-10-0930	ALPK2	NM_052947	p.E41Q	c.121G>C	Verified	tca	gag	cct	1	G	C
TCGA-10-0930	ALPK2_ENS	ENST00000361673	p.E675Q	c.2023G>C	Verified	TCA	GAG	CCT	1	G	C

TCGA-10-0930	ALPK2_ENST	ENST00000361673	p.E675Q	c.2023G>C	Verified	TCA	GAG	CCT	1	G	C
TCGA-23-1022	ALPK3	ENST00000258888	p.D1160H	c.3478G>C	Verified	ATA	GAT	TCC	1	G	C
TCGA-29-1761	AMBP	ENST00000265132	p.R39P	c.116G>C	Unverified	TCT	CGG	ATC	2	G	C
TCGA-36-1577	AMHR2	ENST00000257863	p.V227L	c.679G>C	Verified	ctg	gtt	gcc	1	G	C
TCGA-24-1845	AMIGO1	ENST00000369862	p.G470R	c.1408G>C	Unverified	ACA	GGC	AAG	1	G	C
TCGA-04-1331	AMOTL2	ENST00000249883	p.S298T	c.893G>C	Unverified	GTG	AGT	GCC	2	G	C
TCGA-13-1498	ANAPC16	ENST00000299381	p.E44D	c.132G>C	Verified	GGA	GAG	ATG	3	G	C
TCGA-09-2049	ANKRD11	ENST00000301030	p.K77N	c.231G>C	Unverified	GAG	AAG	CAG	3	G	C
TCGA-25-2401	ANKRD44	NM_153697.1	p.A593P	c.1777G>C	Verified	gaa	gcg	ctt	1	G	C
TCGA-09-2050	ANKS1A	ENST00000360359	p.?	c.2994+1G>C	Verified	AAC	AAG	AAC	3	G	C
TCGA-23-1123	ANKS6	ENST00000353234	p.V126L	c.376G>C	Verified	AGT	GTG	GCA	1	G	C
TCGA-23-1123	ANKS6	ENST00000353234	p.V126L	c.376G>C	Verified	AGT	GTG	GCA	1	G	C
TCGA-24-1431	ANKZF1	ENST00000323348	p.A500P	c.1498G>C	Verified	CTT	GCT	GCT	1	G	C
TCGA-24-1463	ANLN	ENST00000265748	p.E994Q	c.2980G>C	Verified	TTT	GAA	GAT	1	G	C
TCGA-04-1655	ANO6	ENST00000320560	p.V624L	c.1870G>C	Unverified	GAA	GTA	TTA	1	G	C
TCGA-04-1655	ANO6_ENST	ENST00000425752	p.V624L	c.1870G>C	Unverified	GAA	GTA	TTA	1	G	C
TCGA-04-1362	AP1M1	ENST00000291439	p.W408C	c.1224G>C	Verified	CCC	TGG	GTG	3	G	C
TCGA-13-1488	AP1M2	ENST00000250244	p.E284Q	c.850G>C	Verified	ATT	GAG	AAG	1	G	C
TCGA-29-1783	AP1M2	ENST00000250244	p.L16F	c.48G>C	Unverified	CCA	TTG	ATC	3	G	C
TCGA-29-1769	APITD1	ENST00000556104	p.D37H	c.109G>C	Unverified	TTG	GAC	AAA	1	G	C
TCGA-29-1769	APITD1_ENS	ENST00000309048	p.D37H	c.109G>C	Unverified	TTG	GAC	AAA	1	G	C
TCGA-24-2288	APOB	ENST00000233242	p.R4446T	c.13337G>C	Unverified	CAC	AGA	AAT	2	G	C
TCGA-13-0893	APOB	ENST00000233242	p.G210G	c.630G>C	Verified	CTG	GGG	CAG	3	G	C
TCGA-13-0924	APOB	ENST00000233242	p.K3627N	c.10881G>C	Verified	ACT	AAG	AAC	3	G	C
TCGA-23-1120	AQP7	ENST00000297988	p.V82V	c.246G>C	Unverified	GGA	GTG	CAC	3	G	C
TCGA-23-1031	AQP9	ENST00000219919	p.Q109H	c.327G>C	Verified	GCC	CAG	TTC	3	G	C
TCGA-61-1915	AQR	ENST00000156471	p.R410P	c.1229G>C	Unverified	TCT	CGT	CAT	2	G	C
TCGA-24-1417	ARCN1	ENST00000264028	p.A163P	c.487G>C	Unverified	AAA	GCA	AAG	1	G	C
TCGA-24-2289	ARFGAP2	ENST00000524782	p.L484L	c.1452G>C	Unverified	GTG	CTG	CCT	3	G	C
TCGA-25-1326	ARHGAP22	ENST00000249601	p.E667Q	c.1999G>C	Verified	gcg	gag	agg	1	G	C
TCGA-20-0990	ARHGAP32_	ENST00000310343	p.G112A	c.335G>C	Verified	AAA	GGT	CAC	2	G	C
TCGA-20-1685	ARHGAP33	ENST00000314737	p.A836P	c.2506G>C	Unverified	cct	gcc	cag	1	G	C
TCGA-13-1408	ARHGEF38	ENST00000265154	p.K67N	c.201G>C	Verified	GAA	AAG	ATG	3	G	C
TCGA-13-0755	ARID2	ENST00000334344	p.G1166R	c.3496G>C	Verified	CAA	GGG	ACT	1	G	C

TCGA-23-1021	ARL10	ENST00000310389	p.L216F	c.648G>C	Verified	CTC	TTG	GCA	3	G	C
TCGA-04-1367	ARL13A_ENS	ENST00000450049	p.R230T	c.689G>C	Verified	AAA	AGA	CAG	2	G	C
TCGA-29-1781	ARNTL	ENST00000396441	p.D567H	c.1699G>C	Unverified	TCT	GAT	AGT	1	G	C
TCGA-09-1665	ARR3	ENST00000307959	p.D28H	c.82G>C	Verified	GTG	GAC	ACG	1	G	C
TCGA-13-0755	ASAP1	ENST00000357668	p.L998L	c.2994G>C	Verified	GAC	CTG	CTA	3	G	C
TCGA-24-1469	ASB11	ENST00000480796	p.R257T	c.770G>C	Unverified	cgt	aga	aat	2	G	C
TCGA-13-0791	ASCC3	ENST00000369162	p.?	c.2079+1G>C	Verified	AAT	AAG	ATG	3	G	C
TCGA-29-1768	ASMTL	ENST00000381317	p.T462T	c.1386G>C	Unverified	TGC	ACG	GGT	3	G	C
TCGA-09-2044	ASPH	ENST00000379454	p.A377P	c.1129G>C	Verified	CGA	GCA	AGA	1	G	C
TCGA-04-1347	ASZ1	ENST00000284629	p.R298T	c.893G>C	Verified	GAA	AGG	GAT	2	G	C
TCGA-29-1770	ATAD2	ENST00000287394	p.D897H	c.2689G>C	Unverified	TCT	GAC	AAA	1	G	C
TCGA-29-1775	ATAD2	ENST00000287394	p.D1054H	c.3160G>C	Unverified	ATT	GAT	CTA	1	G	C
TCGA-04-1649	ATAD2B_EN	ENST00000238789	p.R1400T	c.4199G>C	Unverified	GAG	AGA	TTG	2	G	C
TCGA-13-1497	ATAD5	ENST00000321990	p.Q1274H	c.3822G>C	Verified	ACT	CAG	ACT	3	G	C
TCGA-13-0920	ATAT1	ENST00000376483	p.E248Q	c.742G>C	Verified	GTG	GAG	CCT	1	G	C
TCGA-13-0884	ATL2	ENST00000419554	p.R104P	c.311G>C	Verified	TTT	CGT	AAA	2	G	C
TCGA-24-1850	ATL2	ENST00000419554	p.R556P	c.1667G>C	Unverified	CGT	CGA	ATG	2	G	C
TCGA-61-1907	ATL3	ENST00000398868	p.L140L	c.420G>C	Unverified	GTT	CTG	ATG	3	G	C
TCGA-13-0919	ATP2A3	ENST00000359983	p.?	c.3139+1G>C	Verified	AGG	GAA	GAA	1	G	C
TCGA-29-1763	ATP2A3	ENST00000359983	p.A240A	c.720G>C	Unverified	ATG	GCG	GCA	3	G	C
TCGA-23-1120	ATP2B2	ENST00000383800	p.E525D	c.1575G>C	Verified	ACG	GAG	TGC	3	G	C
TCGA-13-1497	ATP2B4	ENST00000357681	p.E335D	c.1005G>C	Verified	AAT	GAG	GAA	3	G	C
TCGA-13-0755	ATP5B	ENST00000262030	p.V99V	c.297G>C	Verified	GAG	GTG	GCC	3	G	C
TCGA-23-1120	ATP5G3	ENST00000392541	p.G41R	c.121G>C	Verified	GAG	GGC	TCT	1	G	C
TCGA-04-1338	ATP6V1C2	ENST00000381661	p.V118L	c.352G>C	Verified	CTC	GTG	AGT	1	G	C
TCGA-29-1762	ATRIP	ENST00000320211	p.A438P	c.1312G>C	Unverified	TTG	GCA	GCT	1	G	C
TCGA-24-1844	ATRNL1	ENST00000355044	p.C1060S	c.3179G>C	Unverified	GCT	TGT	ACA	2	G	C
TCGA-24-1847	ATXN3L	ENST00000380622	p.E26Q	c.76G>C	Unverified	gga	gaa	tat	1	G	C
TCGA-29-1691	AXL	ENST00000359092	p.K493N	c.1479G>C	Unverified	CGC	AAG	TCC	3	G	C
TCGA-24-2267	B7	ENST00000323702	p.E27Q	c.79G>C	Verified	ACA	GAG	GAG	1	G	C
TCGA-61-1904	BAI1	NM_001702.1	p.V857V	c.2571G>C	Unverified	act	gtg	aaa	3	G	C
TCGA-04-1331	BAI2	ENST00000373658	p.R1028P	c.3083G>C	Unverified	GGG	CGG	ATG	2	G	C
TCGA-23-2077	BAIAP2L1	ENST00000005260	p.G364A	c.1091G>C	Verified	CAG	GGA	GAT	2	G	C
TCGA-13-0792	BAMBI	ENST00000375533	p.Q189H	c.567G>C	Unverified	caa	cag	atg	3	G	C

TCGA-13-1488	BATF	ENST00000286639	p.L56L	c.168G>C	Verified	cac	ctg	gag	3	G	C
TCGA-24-2024	BBS1	ENST00000318312	p.V541L	c.1621G>C	Verified	CTG	GTG	CCA	1	G	C
TCGA-61-2008	BBS7	ENST00000264499	p.M284I	c.852G>C	Verified	CAG	ATG	TTG	3	G	C
TCGA-30-1857	BCAN	ENST00000329117	p.G720R	c.2158G>C	Unverified	TAC	GGC	GCG	1	G	C
TCGA-24-2288	BCHE	ENST00000264381	p.M109I	c.327G>C	Unverified	gag	atg	tgg	3	G	C
TCGA-29-1764	BCL9_ENST(	ENST00000234739	p.G97A	c.290G>C	Unverified	AAG	GGG	AAA	2	G	C
TCGA-24-1844	BCOR_ENST(	ENST00000378444	p.V1196L	c.3586G>C	Unverified	AAG	GTG	TGC	1	G	C
TCGA-61-2102	BEST4	ENST00000372207	p.A280P	c.838G>C	Verified	CCA	GCC	CTG	1	G	C
TCGA-24-1423	BID	ENST00000317361	p.S110T	c.329G>C	Unverified	CGC	AGC	AGC	2	G	C
TCGA-29-1705	BIN1	ENST00000316724	p.D113H	c.337G>C	Unverified	atg	gat	tac	1	G	C
TCGA-29-1705	BIN1_ENST0	ENST00000259238	p.D113H	c.337G>C	Unverified	ATG	GAT	TAC	1	G	C
TCGA-13-0883	BIRC6	ENST00000421745	p.R302T	c.905G>C	Verified	TAT	AGG	TGG	2	G	C
TCGA-13-0884	BMPR2	ENST00000374580	p.R974P	c.2921G>C	Verified	aaa	cgt	gtg	2	G	C
TCGA-24-1843	BNC1	ENST00000345382	p.E852D	c.2556G>C	Unverified	AGC	GAG	GGC	3	G	C
TCGA-30-1857	BOD1L	ENST00000040738	p.D1708H	c.5122G>C	Unverified	GTG	GAT	GGC	1	G	C
TCGA-23-1022	BOLA1	ENST00000369150	p.L19F	c.57G>C	Verified	TGT	TTG	TGC	3	G	C
TCGA-09-2044	BPTF	ENST00000306378	p.V920L	c.2758G>C	Verified	TTT	GTT	CCT	1	G	C
TCGA-13-1498	BRD2_ENST(	ENST00000395289	p.R528P	c.1583G>C	Verified	CTT	CGG	GCA	2	G	C
TCGA-24-1849	BRD4_ENST(	ENST00000263377	p.E1258Q	c.3772G>C	Unverified	CAG	GAG	CGC	1	G	C
TCGA-24-1844	BRD8	ENST00000254900	p.V847L	c.2539G>C	Unverified	AGT	GTC	CCA	1	G	C
TCGA-24-1844	BRD8_ENST(	ENST00000230901	p.V920L	c.2758G>C	Unverified	AGT	GTC	CCA	1	G	C
TCGA-20-0990	BST2	ENST00000252593	p.V35V	c.105G>C	Verified	ATC	GTG	ATT	3	G	C
TCGA-24-1849	BTK	ENST00000308731	p.G164A	c.491G>C	Unverified	ATG	GGC	TGC	2	G	C
TCGA-23-1031	C10orf113	ENST00000377118	p.C35S	c.104G>C	Verified	TCT	TGT	GTG	2	G	C
TCGA-23-1031	C10orf113_E	ENST00000377118	p.C35S	c.104G>C	Verified	TCT	TGT	GTG	2	G	C
TCGA-23-1117	C10orf54	ENST00000394957	p.A174P	c.520G>C	Verified	GAT	GCA	CCA	1	G	C
TCGA-23-1117	C10orf54	ENST00000394957	p.A174P	c.520G>C	Verified	GAT	GCA	CCA	1	G	C
TCGA-29-1762	C11orf2	ENST00000279281	p.G628R	c.1882G>C	Unverified	GTG	GGG	CTC	1	G	C
TCGA-04-1331	C11orf30	ENST00000334736	p.V415L	c.1243G>C	Verified	CAA	GTG	CAA	1	G	C
TCGA-13-0885	C11orf47	ENST00000354685	p.G330R	c.988G>C	Verified	TTT	GGG	ATC	1	G	C
TCGA-04-1362	C11orf63	ENST00000227349	p.A146P	c.436G>C	Verified	GAA	GCG	TTG	1	G	C
TCGA-24-1563	C11orf66	ENST00000338608	p.G320R	c.958G>C	Verified	ACA	GGG	TTC	1	G	C
TCGA-24-2289	C12orf34	ENST00000358906	p.K394N	c.1182G>C	Unverified	AGC	AAG	AGT	3	G	C
TCGA-04-1638	C12orf35	ENST00000312561	p.M168I	c.504G>C	Unverified	CAG	ATG	ATC	3	G	C

TCGA-04-1651	C12orf39	ENST00000256969	p.R71T	c.212G>C	Unverified	GAA	AGA	CGA	2	G	C
TCGA-04-1651	C12orf39	ENST00000256969	p.R71T	c.212G>C	Unverified	GAA	AGA	CGA	2	G	C
TCGA-29-1761	C12orf41	ENST00000420613	p.G164R	c.490G>C	Unverified	AGA	GGT	GAC	1	G	C
TCGA-13-0762	C12orf41	ENST00000420613	p.Q160H	c.480G>C	Verified	GAT	CAG	ACA	3	G	C
TCGA-13-0762	C12orf41	ENST00000420613	p.Q160H	c.480G>C	Verified	GAT	CAG	ACA	3	G	C
TCGA-29-1761	C12orf41_EI	ENST00000420613	p.G164R	c.490G>C	Unverified	AGA	GGT	GAC	1	G	C
TCGA-13-0906	C12orf63	ENST00000342887	p.E1042Q	c.3124G>C	Verified	ATA	GAA	CGT	1	G	C
TCGA-13-0904	C14orf135	ENST00000404681	p.V741L	c.2221G>C	Verified	ACA	GTA	ATG	1	G	C
TCGA-13-0920	C14orf149	ENST00000247194	p.A207P	c.619G>C	Verified	TCT	GCA	AAG	1	G	C
TCGA-13-0760	C14orf43	ENST00000286523	p.C442S	c.1325G>C	Verified	GAC	TGT	GGG	2	G	C
TCGA-29-1693	C14orf43	ENST00000286523	p.K205N	c.615G>C	Unverified	GCC	AAG	AAA	3	G	C
TCGA-24-1849	C15orf39	ENST00000360639	p.E244Q	c.730G>C	Unverified	TCT	GAG	GGG	1	G	C
TCGA-24-1849	C15orf39	ENST00000360639	p.E438D	c.1314G>C	Unverified	GAA	GAG	AAG	3	G	C
TCGA-04-1347	C15orf48	ENST00000396650	p.A31P	c.91G>C	Verified	TTC	GCT	GTG	1	G	C
TCGA-13-0755	C15orf57	ENST00000358005	p.A69P	c.205G>C	Verified	TGG	GCT	CCC	1	G	C
TCGA-13-0886	C17orf46	ENST00000331780	p.E96Q	c.286G>C	Verified	GAG	GAG	TCT	1	G	C
TCGA-24-1847	C17orf57	ENST00000331493	p.E564Q	c.1690G>C	Unverified	CCA	GAA	TTT	1	G	C
TCGA-24-1474	C17orf76	ENST00000325618	p.R121P	c.362G>C	Unverified	CAG	CGT	GGA	2	G	C
TCGA-30-1714	C18orf34	ENST00000300227	p.E501Q	c.1501G>C	Unverified	AAG	GAG	GCT	1	G	C
TCGA-30-1714	C18orf34_EI	ENST00000383096	p.E501Q	c.1501G>C	Unverified	AAG	GAG	GCT	1	G	C
TCGA-13-0884	C1orf127	ENST00000377008	p.R80T	c.239G>C	Verified	CAG	AGG	TGG	2	G	C
TCGA-04-1649	C1orf131	ENST00000366649	p.D162H	c.484G>C	Unverified	GTG	GAT	ACA	1	G	C
TCGA-23-1110	C1orf158	ENST00000288048	p.R115P	c.344G>C	Verified	CTC	CGC	ACT	2	G	C
TCGA-24-1563	C1orf52	ENST00000471115	p.D81H	c.241G>C	Verified	ATA	GAC	TGG	1	G	C
TCGA-20-1686	C1orf87	ENST00000371201	p.?	c.1193-1G>C	Unverified	ACA	GGG	AAG	2	G	C
TCGA-04-1338	C1QL3	ENST00000298943	p.S214T	c.641G>C	Verified	GCC	AGT	AAC	2	G	C
TCGA-29-1783	C20orf132_I	ENST00000400441	p.S343T	c.1028G>C	Unverified	GCC	AGC	ATG	2	G	C
TCGA-23-1124	C20orf54	ENST00000217254	p.E145Q	c.433G>C	Verified	GGT	GAA	GGA	1	G	C
TCGA-24-1104	C21orf7	ENST00000399947	p.K174N	c.522G>C	Verified	AAA	AAG	GAA	3	G	C
TCGA-13-0760	C2CD2	ENST00000380486	p.G376A	c.1127G>C	Verified	CTG	GGC	TCG	2	G	C
TCGA-23-1022	C2CD3	ENST00000313663	p.R371P	c.1112G>C	Verified	AAT	CGG	TTT	2	G	C
TCGA-13-1489	C2orf16	ENST00000408964	p.Q1635H	c.4905G>C	Unverified	TCT	CAG	AGG	3	G	C
TCGA-13-1489	C2orf16_EN	ENST00000408964	p.Q1635H	c.4905G>C	Unverified	TCT	CAG	AGG	3	G	C
TCGA-24-1470	C2orf63	ENST00000407122	p.G275R	c.823G>C	Verified	CAA	GGC	ATT	1	G	C

TCGA-24-1470	C2orf63_EN!	ENST00000407122	p.G275R	c.823G>C	Verified	CAA	GGC	ATT	1	G	C
TCGA-04-1331	C2orf78	ENST00000342345	p.R470S	c.1410G>C	Verified	GTC	AGG	AAG	3	G	C
TCGA-09-2051	C3orf26	ENST00000421999	p.K100N	c.300G>C	Unverified	ATG	AAG	GAC	3	G	C
TCGA-23-1117	C3orf58	ENST00000315691	p.A230P	c.688G>C	Verified	TTT	GCA	AAG	1	G	C
TCGA-23-1117	C3orf58	ENST00000315691	p.A230P	c.688G>C	Verified	TTT	GCA	AAG	1	G	C
TCGA-23-1118	C3orf63	ENST00000431842	p.D371H	c.1111G>C	Verified	GCT	GAC	ATC	1	G	C
TCGA-29-1763	C5orf64	ENST00000505642	p.E41D	c.123G>C	Unverified	ATA	GAG	ACA	3	G	C
TCGA-13-0905	C6orf168	ENST00000389677	p.G52G	c.156G>C	Verified	GGT	GGG	ATC	3	G	C
TCGA-24-1850	C6orf70	ENST00000366773	p.V68L	c.202G>C	Unverified	AGC	GTG	AGG	1	G	C
TCGA-61-2109	C7orf29	ENST00000343855	p.E202Q	c.604G>C	Unverified	TGG	GAG	AAT	1	G	C
TCGA-29-1703	C7orf41	ENST00000324453	p.E108Q	c.322G>C	Unverified	TTT	GAA	GAG	1	G	C
TCGA-24-2289	C7orf42	ENST00000341567	p.D81H	c.241G>C	Unverified	AAC	GAC	ACC	1	G	C
TCGA-24-1470	C7orf58	ENST00000310396	p.L446L	c.1338G>C	Verified	TGT	CTG	TCC	3	G	C
TCGA-24-1464	C8orf73	ENST00000398882	p.?	c.295-1G>C	Verified	CAG	GTT	CCC	1	G	C
TCGA-24-1464	C8orf73	ENST00000398882	p.?	c.295-1G>C	Verified	CAG	GTT	CCC	1	G	C
TCGA-24-1464	C8orf73_EN!	ENST00000398882	p.?	c.295-1G>C	Verified	CAG	GTT	CCC	1	G	C
TCGA-13-0893	C9orf46	ENST00000223864	p.Q46H	c.138G>C	Verified	ATG	CAG	ATT	3	G	C
TCGA-09-2044	C9orf72	ENST00000380003	p.G458R	c.1372G>C	Verified	CCA	GGC	CTA	1	G	C
TCGA-04-1651	C9orf79	ENST00000325643	p.A1169P	c.3505G>C	Unverified	TGT	GCC	CTC	1	G	C
TCGA-04-1651	C9orf79	ENST00000325643	p.A1169P	c.3505G>C	Unverified	TGT	GCC	CTC	1	G	C
TCGA-29-1783	CA10	ENST00000285273	p.Q150H	c.450G>C	Unverified	GGA	CAG	GCC	3	G	C
TCGA-13-0916	CA6	ENST00000377443	p.R132T	c.395G>C	Verified	ATC	AGA	CAT	2	G	C
TCGA-23-1116	CABS1	ENST00000273936	p.D80H	c.238G>C	Unverified	GAT	GAT	ATG	1	G	C
TCGA-04-1336	CABS1	ENST00000273936	p.K354N	c.1062G>C	Verified	CCT	AAG	ATC	3	G	C
TCGA-13-0760	CACNA1A_E	ENST00000357018	p.K1159N	c.3477G>C	Verified	GCT	AAG	ACT	3	G	C
TCGA-61-1740	CACNA1E	ENST00000367570	p.L1736L	c.5208G>C	Unverified	ATC	CTG	GGG	3	G	C
TCGA-61-1740	CACNA1E_EI	ENST00000367573	p.L1736L	c.5208G>C	Unverified	ATC	CTG	GGG	3	G	C
TCGA-29-1769	CACNA1S	ENST00000362061	p.E1693D	c.5079G>C	Unverified	GAA	GAG	ACA	3	G	C
TCGA-13-0924	CACNA2D1	ENST00000356860	p.R754T	c.2261G>C	Verified	AAA	AGG	AGC	2	G	C
TCGA-13-0755	CACNB1	ENST00000344140	p.K390N	c.1170G>C	Verified	ATC	AAG	ATC	3	G	C
TCGA-09-0369	CAD	ENST00000264705	p.V823L	c.2467G>C	Verified	TCA	GTG	GAC	1	G	C
TCGA-09-0369	CAD	ENST00000264705	p.V823L	c.2467G>C	Verified	TCA	GTG	GAC	1	G	C
TCGA-13-1481	CAD	ENST00000264705	p.M67I	c.201G>C	Verified	GAA	ATG	GAT	3	G	C
TCGA-23-1114	CALCRL	ENST00000392370	p.C436S	c.1307G>C	Unverified	GAC	TGT	CCT	2	G	C

TCGA-23-1031	CALM3	ENST00000291295	p.M145I	c.435G>C	Verified	CAG	ATG	ATG	3	G	C
TCGA-29-1696	CAMKK1	ENST00000348335	p.D285H	c.853G>C	Unverified	ggg	gat	gat	1	G	C
TCGA-29-1696	CAMKK1	ENST00000348335	p.D285H	c.853G>C	Unverified	ggg	gat	gat	1	G	C
TCGA-29-1696	CAMKK1_EN	ENST00000158166	p.D323H	c.967G>C	Unverified	GGG	GAT	GAT	1	G	C
TCGA-29-1696	CAMKK1_EN	ENST00000158166	p.D323H	c.967G>C	Unverified	GGG	GAT	GAT	1	G	C
TCGA-29-1775	CANT1	ENST00000392446	p.D150H	c.448G>C	Unverified	AAA	GAC	CAT	1	G	C
TCGA-61-2094	CAPN6	ENST00000324068	p.D37H	c.109G>C	Unverified	aat	gat	tct	1	G	C
TCGA-13-0913	CAPN7	ENST00000253693	p.D516H	c.1546G>C	Verified	ATA	GAC	AAC	1	G	C
TCGA-23-2077	CAPZA3	ENST00000317658	p.G136A	c.407G>C	Verified	AAA	GGA	AAT	2	G	C
TCGA-04-1362	CARS	ENST00000397111	p.V268L	c.802G>C	Verified	TCT	GTC	TAC	1	G	C
TCGA-09-2056	CASC3	ENST00000264645	p.R323P	c.968G>C	Verified	GGT	CGT	GCT	2	G	C
TCGA-13-0795	CASD1	ENST00000297273	p.L443F	c.1329G>C	Verified	ATT	TTG	ATT	3	G	C
TCGA-13-0924	CASP5	NM_004347.1	p.E173D	c.519G>C	Verified	aga	gag	gac	3	G	C
TCGA-13-1507	CCAR1	ENST00000265872	p.W200C	c.600G>C	Verified	AAA	TGG	AAT	3	G	C
TCGA-09-2056	CCDC116	ENST00000292779	p.A166P	c.496G>C	Verified	ATG	GCC	GGC	1	G	C
TCGA-13-0760	CCDC147	ENST00000369704	p.D22H	c.64G>C	Verified	AGA	GAT	TTT	1	G	C
TCGA-29-1781	CCDC155	ENST00000447857	p.R73P	c.218G>C	Unverified	GCA	CGC	CTC	2	G	C
TCGA-61-1910	CCDC34_EN	ENST00000328697	p.E217Q	c.649G>C	Unverified	GAG	GAA	AAA	1	G	C
TCGA-13-1497	CCDC57	ENST00000445854	p.E287D	c.861G>C	Verified	GAG	GAG	CTC	3	G	C
TCGA-13-0760	CCDC64	ENST00000397558	p.?	c.1309-1G>C	Verified	ACG	GTG	ACA	1	G	C
TCGA-30-1718	CCDC77	ENST00000239830	p.E418Q	c.1252G>C	Unverified	GTA	GAA	GGC	1	G	C
TCGA-13-0903	CCDC88B	ENST00000356786	p.V497L	c.1489G>C	Verified	CCT	GTT	CTT	1	G	C
TCGA-23-1022	CCDC88C	ENST00000389857	p.L1075L	c.3225G>C	Verified	CAG	CTG	CTA	3	G	C
TCGA-13-0887	CCKBR	ENST00000334619	p.A327P	c.979G>C	Verified	CTG	GCT	AAG	1	G	C
TCGA-13-0762	CCNG2	ENST00000316355	p.D264H	c.790G>C	Verified	cca	gat	ctt	1	G	C
TCGA-13-0762	CCNG2	ENST00000316355	p.D264H	c.790G>C	Verified	cca	gat	ctt	1	G	C
TCGA-24-2289	CCT3	ENST00000295688	p.V332L	c.994G>C	Unverified	ATA	GTC	AGC	1	G	C
TCGA-61-1998	CCT8L2	ENST00000359963	p.V362L	c.1084G>C	Unverified	GTG	GTA	TTT	1	G	C
TCGA-61-2102	CD101	ENST00000369470	p.Q223H	c.669G>C	Verified	GTA	CAG	CTC	3	G	C
TCGA-13-1489	CD2BP2	ENST00000305596	p.E288Q	c.862G>C	Verified	TGG	GAG	AAC	1	G	C
TCGA-23-2077	CD300E	ENST00000328630	p.R79T	c.236G>C	Verified	ATC	AGA	GAC	2	G	C
TCGA-13-0755	CD34	ENST00000310833	p.W317C	c.951G>C	Verified	AGC	TGG	AGC	3	G	C
TCGA-29-1769	CD37	ENST00000323906	p.K40N	c.120G>C	Unverified	GAC	AAG	ACC	3	G	C
TCGA-29-1769	CD37_ENST(	ENST00000391859	p.K40N	c.120G>C	Unverified	GAC	AAG	ACC	3	G	C

TCGA-25-1326	CD38	ENST00000226279	p.C119S	c.356G>C	Verified	CCT	TGC	AAC	2	G	C
TCGA-13-0761	CD3E	ENST00000361763	p.V134L	c.400G>C	Verified	ATT	GTC	ATA	1	G	C
TCGA-13-0761	CD3E	ENST00000361763	p.V134L	c.400G>C	Verified	ATT	GTC	ATA	1	G	C
TCGA-13-1481	CDAN1	ENST00000356231	p.E1163Q	c.3487G>C	Verified	GTG	GAG	AAG	1	G	C
TCGA-29-1761	CDC20	ENST00000310955	p.E142D	c.426G>C	Unverified	CCA	GAG	GGT	3	G	C
TCGA-29-1784	CDC27	ENST00000066544	p.R709T	c.2126G>C	Unverified	CAC	AGA	GCC	2	G	C
TCGA-29-1784	CDC27_ENST	ENST00000531206	p.R715T	c.2144G>C	Unverified	CAC	AGA	GCC	2	G	C
TCGA-29-1703	CDC42BPA	ENST00000366767	p.?	c.2344-1G>C	Unverified	aca	gat	atg	1	G	C
TCGA-13-0761	CDC42BPA	ENST00000366767	p.L1576F	c.4728G>C	Unverified	tct	ttg	tcc	3	G	C
TCGA-13-0761	CDC42BPA	ENST00000366767	p.L1576F	c.4728G>C	Unverified	tct	ttg	tcc	3	G	C
TCGA-29-1703	CDC42BPA_	ENST00000366766	p.?	c.2587-1G>C	Unverified	ACA	GAT	ATG	1	G	C
TCGA-29-1703	CDC42BPA_	ENST00000366769	p.?	c.2587-1G>C	Unverified	ACA	GAT	ATG	1	G	C
TCGA-25-2400	CDC42BPG_	ENST00000342711	p.A81P	c.241G>C	Unverified	GGA	GCC	TTT	1	G	C
TCGA-04-1367	CDC7	ENST00000234626	p.E533Q	c.1597G>C	Unverified	TTA	GAA	GGC	1	G	C
TCGA-13-1510	CDH20	ENST00000262717	p.S60T	c.179G>C	Verified	AGG	AGC	TGG	2	G	C
TCGA-13-0887	CDH6	ENST00000265071	p.G42A	c.125G>C	Verified	TCT	GGA	AAC	2	G	C
TCGA-04-1367	CDK1	ENST00000395284	p.D73H	c.217G>C	Verified	CAG	GAT	TCC	1	G	C
TCGA-04-1367	CDK1_ENST(	ENST00000395284	p.D73H	c.217G>C	Verified	CAG	GAT	TCC	1	G	C
TCGA-25-1326	CDK17	ENST00000261211	p.K343N	c.1029G>C	Verified	ACA	AAG	ACC	3	G	C
TCGA-61-2095	CDK5RAP2	ENST00000349780	p.V1290V	c.3870G>C	Verified	gat	gtg	gat	3	G	C
TCGA-24-1422	CDKAL1	ENST00000378610	p.V381L	c.1141G>C	Verified	CTT	GTT	GAA	1	G	C
TCGA-13-0884	CDSN	ENST00000376288	p.E524D	c.1572G>C	Verified	GGA	GAG	TTA	3	G	C
TCGA-23-1022	CELF1	ENST00000358597	p.A97P	c.289G>C	Verified	CCT	GCT	GAC	1	G	C
TCGA-04-1343	CELF4	ENST00000361795	p.S29T	c.86G>C	Verified	GGC	AGT	GCC	2	G	C
TCGA-29-1691	CELSR1	ENST00000262738	p.V1908L	c.5722G>C	Unverified	TGT	GTG	GAT	1	G	C
TCGA-23-1022	CELSR3	ENST00000164024	p.R1937P	c.5810G>C	Verified	CAC	CGA	GTG	2	G	C
TCGA-29-1764	CENPC1	ENST00000273853	p.D221H	c.661G>C	Unverified	ATA	GAT	AAT	1	G	C
TCGA-36-1577	CENPJ	ENST00000381884	p.E212D	c.636G>C	Verified	GGA	GAG	AGA	3	G	C
TCGA-23-1031	CEP192	ENST00000325971	p.S1794T	c.5381G>C	Verified	CAA	AGC	ATC	2	G	C
TCGA-23-1021	CEP95	ENST00000556440	p.D412H	c.1234G>C	Verified	GAG	GAT	GGA	1	G	C
TCGA-09-2049	CERKL	ENST00000410087	p.G252A	c.755G>C	Verified	GCT	GGG	ATG	2	G	C
TCGA-09-2044	CGA	ENST00000369582	p.G96A	c.287G>C	Verified	ATG	GGG	GGT	2	G	C
TCGA-13-0905	CGRRF1	ENST00000216420	p.G69A	c.206G>C	Verified	TTT	GGC	TTA	2	G	C
TCGA-24-1604	CHAD	ENST00000258969	p.L231L	c.693G>C	Verified	CCC	CTG	AAA	3	G	C

TCGA-30-1718	CHD1	ENST00000284049	p.E778D	c.2334G>C	Unverified	cag	gag	gcc	3	G	C
TCGA-24-1843	CHD3	ENST00000330494	p.V461L	c.1381G>C	Unverified	CGC	GTA	TGC	1	G	C
TCGA-61-2009	CHD8	ENST00000399982	p.A406P	c.1216G>C	Verified	cag	gct	ggc	1	G	C
TCGA-04-1367	CHMP1A	ENST00000253475	p.A227P	c.679G>C	Unverified	CCG	GCA	GCC	1	G	C
TCGA-61-1895	CHN2	ENST00000222792	p.D374H	c.1120G>C	Unverified	ata	gat	gca	1	G	C
TCGA-61-1895	CHN2_ENST	ENST00000409041	p.D238H	c.712G>C	Unverified	ATA	GAT	GCA	1	G	C
TCGA-24-2288	CHPF2	ENST00000035307	p.S467T	c.1400G>C	Unverified	GTC	AGC	CTG	2	G	C
TCGA-13-0923	CHPF2	ENST00000035307	p.Q53H	c.159G>C	Unverified	CCA	CAG	AAT	3	G	C
TCGA-29-1762	CHRDL1	ENST00000218054	p.E296D	c.888G>C	Unverified	caa	gag	tgt	3	G	C
TCGA-24-1849	CHRM3	ENST00000255380	p.M188I	c.564G>C	Unverified	GTG	ATG	ATC	3	G	C
TCGA-61-1913	CHRM5	ENST00000383263	p.L50F	c.150G>C	Unverified	GTC	TTG	GTC	3	G	C
TCGA-61-1910	CHRNA7	ENST00000306901	p.E24D	c.72G>C	Unverified	GGC	GAG	TTC	3	G	C
TCGA-04-1367	CIC	ENST00000160740	p.W37S	c.110G>C	Verified	CCT	TGG	CAC	2	G	C
TCGA-30-1718	CKAP5	ENST00000529230	p.A198P	c.592G>C	Unverified	GAT	GCT	CTG	1	G	C
TCGA-29-1769	CLCA4	ENST00000370563	p.V909L	c.2725G>C	Unverified	GTT	GTA	ATT	1	G	C
TCGA-13-0885	CLEC9A	ENST00000355819	p.G46R	c.136G>C	Verified	ATG	GGA	TTA	1	G	C
TCGA-30-1718	CLTC_ENST0	ENST00000269122	p.R967S	c.2901G>C	Unverified	TAC	AGG	AGA	3	G	C
TCGA-13-0760	CMTM2	ENST00000268595	p.C157S	c.470G>C	Verified	GCT	TGT	GCG	2	G	C
TCGA-13-0762	CMYA5	ENST00000238522	p.L3295L	c.9885G>C	Verified	AGT	CTG	GAA	3	G	C
TCGA-13-0762	CMYA5	ENST00000238522	p.L3295L	c.9885G>C	Verified	AGT	CTG	GAA	3	G	C
TCGA-04-1338	CNGA1	ENST00000358519	p.D472H	c.1414G>C	Verified	TTA	GAC	ACA	1	G	C
TCGA-25-2392	CNO	ENST00000320776	p.P119P	c.357G>C	Unverified	GTG	CCG	CGC	3	G	C
TCGA-61-1737	CNOT1	ENST00000317147	p.R964T	c.2891G>C	Unverified	AAC	AGA	TTG	2	G	C
TCGA-61-1737	CNOT1_ENS	ENST00000441024	p.R964T	c.2891G>C	Unverified	AAC	AGA	TTG	2	G	C
TCGA-13-0923	CNOT3	ENST00000221232	p.Q684H	c.2052G>C	Verified	GCA	CAG	TAT	3	G	C
TCGA-23-1031	CNTFR	ENST00000351266	p.G85A	c.254G>C	Verified	AGT	GGC	CTC	2	G	C
TCGA-13-0792	CNTN2	ENST00000331830	p.E776Q	c.2326G>C	Verified	AAC	GAG	AGC	1	G	C
TCGA-61-1906	COL13A1	ENST00000398969	p.?	c.553-1G>C	Unverified	CCG	GGA	CCC	1	G	C
TCGA-61-1906	COL13A1	ENST00000398969	p.?	c.553-1G>C	Unverified	CCG	GGA	CCC	1	G	C
TCGA-61-1906	COL13A1_EI	ENST00000398978	p.?	c.604-1G>C	Unverified	CCG	GGA	CCC	1	G	C
TCGA-61-1906	COL13A1_EI	ENST00000398978	p.?	c.604-1G>C	Unverified	CCG	GGA	CCC	1	G	C
TCGA-29-1711	COL15A1	ENST00000375001	p.W546S	c.1637G>C	Unverified	AGA	TGG	ATC	2	G	C
TCGA-29-1711	COL15A1	ENST00000375001	p.W546S	c.1637G>C	Unverified	AGA	TGG	ATC	2	G	C
TCGA-13-0883	COL1A1	ENST00000225964	p.G446A	c.1337G>C	Unverified	ACT	GGT	GCT	2	G	C

TCGA-29-1693	COL2A1	ENST00000337299	p.G456R	c.1366G>C	Unverified	GCA	GGT	CCC	1	G	C
TCGA-29-1693	COL2A1_EN:	ENST00000380518	p.G525R	c.1573G>C	Unverified	GCA	GGT	CCC	1	G	C
TCGA-30-1718	COL4A5	ENST00000361603	p.G1330A	c.3989G>C	Unverified	cca	gga	ttc	2	G	C
TCGA-24-2024	COL6A2	ENST00000300527	p.E695Q	c.2083G>C	Verified	CTC	GAG	TGG	1	G	C
TCGA-61-2094	COL6A6	ENST00000358511	p.A1179P	c.3535G>C	Unverified	ACA	GCG	GGT	1	G	C
TCGA-09-2044	COL7A1	ENST00000328333	p.G2351A	c.7052G>C	Verified	CCC	GGA	GAC	2	G	C
TCGA-24-2035	COLEC10	ENST00000332843	p.E217Q	c.649G>C	Verified	AGG	GAG	GGA	1	G	C
TCGA-24-1469	COLEC12	ENST00000400256	p.Q266H	c.798G>C	Verified	TTG	CAG	ACG	3	G	C
TCGA-09-1674	CPA3	ENST00000296046	p.T283T	c.849G>C	Unverified	GAG	ACG	AAA	3	G	C
TCGA-24-0975	CPNE2	ENST00000290776	p.Q20H	c.60G>C	Unverified	CCC	CAG	TAT	3	G	C
TCGA-23-1124	CPS1	ENST00000233072	p.S847T	c.2540G>C	Verified	CCA	AGC	AGC	2	G	C
TCGA-29-1763	CPT1B	ENST00000395650	p.E291Q	c.871G>C	Unverified	CGT	GAA	GAA	1	G	C
TCGA-23-1809	CRB1	ENST00000367400	p.G770A	c.2309G>C	Unverified	CGC	GGC	AGA	2	G	C
TCGA-24-1463	CRB1	ENST00000367400	p.G101A	c.302G>C	Verified	CCT	GGG	TAC	2	G	C
TCGA-09-2049	CREBBP	ENST00000262367	p.V1467V	c.4401G>C	Verified	TAT	GTG	ACA	3	G	C
TCGA-13-1505	CROCC	ENST00000375541	p.E89D	c.267G>C	Verified	CAG	GAG	CTG	3	G	C
TCGA-25-1313	CRYAA	ENST00000291554	p.D58H	c.172G>C	Unverified	ctg	gac	tcc	1	G	C
TCGA-24-1843	CRYBA4	ENST00000354760	p.E111D	c.333G>C	Unverified	TTC	GAG	CAA	3	G	C
TCGA-24-1845	CSE1L	ENST00000262982	p.L248F	c.744G>C	Unverified	aca	ttg	gat	3	G	C
TCGA-24-1464	CSMD3	ENST00000297405	p.W1472C	c.4416G>C	Verified	gtc	tgg	gac	3	G	C
TCGA-09-2056	CSPP1	ENST00000262210	p.D833H	c.2497G>C	Verified	AGA	GAC	AAT	1	G	C
TCGA-13-1489	CSPP1	ENST00000262210	p.K1019N	c.3057G>C	Verified	CAG	AAG	AGG	3	G	C
TCGA-25-2392	CSRNP1	ENST00000273153	p.R31P	c.92G>C	Verified	TCT	CGC	TCC	2	G	C
TCGA-29-1783	CSRNP2	ENST00000228515	p.S34T	c.101G>C	Unverified	GAT	AGT	GCT	2	G	C
TCGA-13-0905	CSRNP3	ENST00000342316	p.D337H	c.1009G>C	Verified	TTA	GAT	TGC	1	G	C
TCGA-13-0885	CSRP2BP	ENST00000278816	p.S316T	c.947G>C	Verified	TTG	AGC	TCC	2	G	C
TCGA-23-1120	CST9	ENST00000376971	p.S135T	c.404G>C	Verified	CAC	AGC	TGT	2	G	C
TCGA-10-0930	CTAGE4	ENST00000486333	p.G236R	c.706G>C	Unverified	AAA	GGA	GAA	1	G	C
TCGA-10-0930	CTAGE4	ENST00000486333	p.G236R	c.706G>C	Unverified	AAA	GGA	GAA	1	G	C
TCGA-13-1497	CTCF	ENST00000264010	p.E291D	c.873G>C	Verified	GAT	GAG	AGA	3	G	C
TCGA-36-1577	CTHRC1	ENST00000330295	p.G181R	c.541G>C	Verified	CAA	GGA	AGC	1	G	C
TCGA-36-1577	CTIF	ENST00000256413	p.Q369H	c.1107G>C	Unverified	CAG	CAG	AAC	3	G	C
TCGA-61-1907	CTNNBL1	ENST00000361383	p.E479D	c.1437G>C	Unverified	gag	gag	gag	3	G	C
TCGA-13-0762	CTSA	ENST00000372484	p.D271H	c.811G>C	Verified	AAA	GAC	CTG	1	G	C

TCGA-13-0762	CTSA	ENST00000372484	p.D271H	c.811G>C	Verified	AAA	GAC	CTG	1	G	C
TCGA-29-1764	CTSG	ENST00000216336	p.V144V	c.432G>C	Unverified	ACT	GTG	GCC	3	G	C
TCGA-13-1512	CTTN	ENST00000301843	p.G311A	c.932G>C	Verified	TAT	GGG	GTG	2	G	C
TCGA-13-0884	CTTNBP2	ENST00000160373	p.R1617T	c.4850G>C	Verified	CCT	AGA	AGT	2	G	C
TCGA-23-1031	CTTNBP2	ENST00000160373	p.S1131T	c.3392G>C	Verified	GGA	AGC	TTG	2	G	C
TCGA-23-1031	CTXN1	ENST00000318978	p.V47V	c.141G>C	Verified	ATG	GTG	CGC	3	G	C
TCGA-04-1336	CUBN	ENST00000377833	p.R1911T	c.5732G>C	Verified	TTA	AGG	ATC	2	G	C
TCGA-29-1769	CUL1	ENST00000409469	p.E457Q	c.1369G>C	Unverified	ata	gaa	gac	1	G	C
TCGA-24-2024	CUL3	ENST00000264414	p.D92H	c.274G>C	Verified	gaa	gat	gta	1	G	C
TCGA-23-2078	CUL9	ENST00000252050	p.Q381H	c.1143G>C	Verified	CAG	CAG	ACA	3	G	C
TCGA-23-1123	CUX2	ENST00000261726	p.G1243R	c.3727G>C	Verified	GGG	GGT	CCT	1	G	C
TCGA-23-1123	CUX2	ENST00000261726	p.G1243R	c.3727G>C	Verified	GGG	GGT	CCT	1	G	C
TCGA-24-2280	CUX2	ENST00000261726	p.R1457R	c.4371G>C	Unverified	CAG	CGG	CGG	3	G	C
TCGA-04-1530	CYP26A1	ENST00000371531	p.G231A	c.692G>C	Verified	TTT	GGA	GGA	2	G	C
TCGA-20-1683	CYP26A1	ENST00000371531	p.S372T	c.1115G>C	Unverified	AGG	AGC	TGT	2	G	C
TCGA-20-0991	CYP2A7	ENST00000301146	p.G336A	c.1007G>C	Verified	ATC	GGC	AAG	2	G	C
TCGA-29-1763	CYP2B6	ENST00000324071	p.L78L	c.234G>C	Unverified	ATG	CTG	TGT	3	G	C
TCGA-23-1124	CYP2C9	ENST00000260682	p.E444D	c.1332G>C	Verified	ATG	GAG	CTG	3	G	C
TCGA-61-1904	CYP3A43	ENST00000222382	p.T374T	c.1122G>C	Unverified	GTT	ACG	AGA	3	G	C
TCGA-24-1422	CYP4A11	ENST00000310638	p.L131F	c.393G>C	Verified	GGC	TTG	CTC	3	G	C
TCGA-04-1347	DAGLA	ENST00000257215	p.G799A	c.2396G>C	Verified	TCA	GGC	TTC	2	G	C
TCGA-13-0886	DCD	ENST00000293371	p.D107H	c.319G>C	Verified	CTT	GAC	TCA	1	G	C
TCGA-29-1768	DCHS2	ENST00000357232	p.L1087F	c.3261G>C	Unverified	TTT	TTG	AAC	3	G	C
TCGA-61-2095	DCTD	ENST00000438320	p.A106P	c.316G>C	Unverified	GTT	GCC	TTG	1	G	C
TCGA-24-2288	DCTN2	ENST00000434715	p.D60H	c.178G>C	Unverified	TAT	GAC	AAG	1	G	C
TCGA-25-1326	DCUN1D4	ENST00000334635	p.R49P	c.146G>C	Verified	CTG	CGG	TCT	2	G	C
TCGA-29-1695	DDR2	ENST00000367922	p.?	c.1857-1G>C	Unverified	GCC	AGG	AAT	3	G	C
TCGA-29-1695	DDR2_ENST	ENST00000367922	p.?	c.1857-1G>C	Unverified	GCC	AGG	AAT	3	G	C
TCGA-29-1777	DDX11	ENST00000542838	p.A607P	c.1819G>C	Unverified	TTT	GCC	CAA	1	G	C
TCGA-29-1777	DDX11_ENS	ENST00000407793	p.A607P	c.1819G>C	Unverified	TTT	GCC	CAA	1	G	C
TCGA-23-1124	DDX24	ENST00000330836	p.G289R	c.865G>C	Verified	cca	ggc	aag	1	G	C
TCGA-61-2102	DDX39B	ENST00000396172	p.D416H	c.1246G>C	Verified	CCT	GAT	GAG	1	G	C
TCGA-13-0760	DDX3X	ENST00000399959	p.R548T	c.1643G>C	Verified	GAG	AGG	AAC	2	G	C
TCGA-24-1463	DDX5	ENST00000225792	p.G533A	c.1598G>C	Verified	aat	ggt	gtt	2	G	C

TCGA-13-0755	DDX60L	ENST00000260184	p.V1260L	c.3778G>C	Verified	TTT	GTT	GAG	1	G	C
TCGA-23-2077	DENND2A	ENST00000275884	p.R36T	c.107G>C	Verified	GCC	AGA	GCC	2	G	C
TCGA-61-1915	DENND4C	ENST00000380437	p.R807T	c.2420G>C	Unverified	GAG	AGG	AGT	2	G	C
TCGA-30-1855	DENND5A	ENST00000328194	p.D867H	c.2599G>C	Unverified	CAG	GAT	ATG	1	G	C
TCGA-29-1784	DEPDC5	ENST00000400248	p.R1376P	c.4127G>C	Unverified	CAT	CGG	AAA	2	G	C
TCGA-29-1784	DEPDC5_EN	ENST00000535622	p.R1307P	c.3920G>C	Unverified	CAT	CGG	AAA	2	G	C
TCGA-13-0904	DGKE	ENST00000284061	p.L22L	c.66G>C	Unverified	cac	ctg	atc	3	G	C
TCGA-23-1122	DGKH	ENST00000337343	p.G734A	c.2201G>C	Verified	gct	ggg	agt	2	G	C
TCGA-30-1718	DHRS2	ENST00000250383	p.V40V	c.120G>C	Unverified	GCC	GTG	GTC	3	G	C
TCGA-30-1718	DHRS2_ENST	ENST00000344777	p.V40V	c.120G>C	Unverified	GCC	GTG	GTC	3	G	C
TCGA-61-1915	DHX29	ENST00000251636	p.R587S	c.1761G>C	Unverified	AAA	AGG	CAT	3	G	C
TCGA-23-1021	DHX34	ENST00000328771	p.?	c.1272+1G>C	Verified	gac	aag	gta	3	G	C
TCGA-29-1785	DHX57	ENST00000295373	p.R116P	c.347G>C	Unverified	ctc	cga	gac	2	G	C
TCGA-29-1785	DHX57	ENST00000295373	p.R116P	c.347G>C	Unverified	ctc	cga	gac	2	G	C
TCGA-29-1775	DHX9	ENST00000367549	p.D56H	c.166G>C	Unverified	AAA	GAT	GCA	1	G	C
TCGA-29-1770	DIDO1	ENST00000395343	p.E134Q	c.400G>C	Unverified	AAG	GAA	CGA	1	G	C
TCGA-29-1770	DIDO1_ENST	ENST00000370371	p.E134Q	c.400G>C	Unverified	AAG	GAA	CGA	1	G	C
TCGA-13-0923	DIP2B	ENST00000301180	p.?	c.797-1G>C	Verified	GAT	GGT	GTT	2	G	C
TCGA-25-2392	DIS3L	ENST00000319194	p.?	c.2608-1G>C	Verified	ACC	GTA	AGA	1	G	C
TCGA-20-1687	DIS3L	ENST00000319194	p.S297T	c.890G>C	Unverified	ATT	AGC	ACT	2	G	C
TCGA-20-1687	DIS3L_ENST(	ENST00000319212	p.S380T	c.1139G>C	Unverified	ATT	AGC	ACT	2	G	C
TCGA-09-1665	DKK2	ENST00000285311	p.C127S	c.380G>C	Verified	atc	tgt	atc	2	G	C
TCGA-23-2078	DKK3	ENST00000396505	p.V304L	c.910G>C	Unverified	gag	gtc	ccc	1	G	C
TCGA-13-1489	DLGAP4	ENST00000373913	p.E109D	c.327G>C	Verified	TTT	GAG	AAG	3	G	C
TCGA-24-1844	DMBT1	ENST00000368909	p.C2360S	c.7079G>C	Unverified	CGT	TGT	AAA	2	G	C
TCGA-24-1844	DMBT1_ENS	ENST00000368909	p.C2360S	c.7079G>C	Unverified	CGT	TGT	AAA	2	G	C
TCGA-24-1844	DMBT1_ENS	ENST00000368915	p.C2489S	c.7466G>C	Unverified	CGT	TGT	AAA	2	G	C
TCGA-13-1497	DMD_ENST(	ENST00000378677	p.Q1693H	c.5079G>C	Verified	ATT	CAG	GCT	3	G	C
TCGA-29-1693	DMXL1	ENST00000311085	p.D2282H	c.6844G>C	Unverified	TTA	GAT	GAA	1	G	C
TCGA-29-1777	DNA2	ENST00000399180	p.V135V	c.405G>C	Unverified	CTG	GTG	TTG	3	G	C
TCGA-29-1777	DNA2L	ENST00000358410	p.V49V	c.147G>C	Unverified	CTG	GTG	TTG	3	G	C
TCGA-29-1693	DNAH10	ENST00000339192	p.E263Q	c.787G>C	Unverified	ATA	GAG	GCT	1	G	C
TCGA-29-1693	DNAH10_EN	ENST00000409039	p.E445Q	c.1333G>C	Unverified	ATA	GAG	GCT	1	G	C
TCGA-24-2262	DNAH11	ENST00000328843	p.K4003N	c.12009G>C	Verified	GCC	AAG	TGG	3	G	C

TCGA-29-1711	DNAH12	ENST00000351747	p.E412D	c.1236G>C	Unverified	TAT	GAG	ACA	3	G	C
TCGA-29-1711	DNAH12	ENST00000351747	p.E412D	c.1236G>C	Unverified	TAT	GAG	ACA	3	G	C
TCGA-29-1711	DNAH12L	ENST00000311202	p.E412D	c.1236G>C	Unverified	TAT	GAG	ACA	3	G	C
TCGA-29-1711	DNAH12L	ENST00000311202	p.E412D	c.1236G>C	Unverified	TAT	GAG	ACA	3	G	C
TCGA-13-0761	DNAH2	ENST00000389173	p.Q137H	c.411G>C	Verified	AAC	CAG	CTT	3	G	C
TCGA-13-0761	DNAH2	ENST00000389173	p.Q137H	c.411G>C	Verified	AAC	CAG	CTT	3	G	C
TCGA-13-0807	DNAH3	ENST00000261383	p.E4023Q	c.12067G>C	Verified	TTA	GAA	GGT	1	G	C
TCGA-23-1031	DNAH3	ENST00000261383	p.W1961C	c.5883G>C	Verified	GTG	TGG	ACC	3	G	C
TCGA-24-1843	DNAH8	NM_001371.1	p.L4038L	c.12114G>C	Unverified	ctt	ctg	gac	3	G	C
TCGA-24-1843	DNAH8_ENS	ENST00000359357	p.L4038L	c.12114G>C	Unverified	CTT	CTG	GAC	3	G	C
TCGA-13-1408	DNAI2	ENST00000311014	p.D48H	c.142G>C	Unverified	GTG	GAC	ACG	1	G	C
TCGA-30-1857	DNAJC12	ENST00000225171	p.A32P	c.94G>C	Unverified	ctg	gca	gaa	1	G	C
TCGA-30-1857	DNAJC12_EI	ENST00000339758	p.A32P	c.94G>C	Unverified	CTG	GCA	GAA	1	G	C
TCGA-13-0885	DNHD1	ENST00000254579	p.G330R	c.988G>C	Verified	TTT	GGG	ATC	1	G	C
TCGA-24-1435	DOC2A	ENST00000350119	p.D120H	c.358G>C	Verified	ATG	GAT	TTC	1	G	C
TCGA-23-2078	DOCK2	ENST00000256935	p.W1074C	c.3222G>C	Verified	ATG	TGG	TAC	3	G	C
TCGA-13-0887	DOCK7	ENST00000340370	p.R118T	c.353G>C	Verified	ATA	AGA	AGT	2	G	C
TCGA-04-1655	DOCK8	ENST00000432829	p.G16R	c.46G>C	Unverified	CTC	GGG	GAC	1	G	C
TCGA-04-1655	DOCK8_ENS	ENST00000453981	p.G84R	c.250G>C	Unverified	CTC	GGG	GAC	1	G	C
TCGA-29-1785	DOLPP1	ENST00000372546	p.V48V	c.144G>C	Unverified	TTC	GTG	ACC	3	G	C
TCGA-29-1785	DOLPP1	ENST00000372546	p.V48V	c.144G>C	Unverified	TTC	GTG	ACC	3	G	C
TCGA-04-1530	DOPEY2	ENST00000399151	p.W881C	c.2643G>C	Verified	CTT	TGG	AAT	3	G	C
TCGA-04-1347	DROSHA	ENST00000511367	p.R930T	c.2789G>C	Verified	GAC	AGA	AAA	2	G	C
TCGA-13-0714	DSCAM	ENST00000400454	p.K767N	c.2301G>C	Verified	TGC	AAG	GTC	3	G	C
TCGA-29-1701	DST	ENST00000370765	p.Q2421H	c.7263G>C	Unverified	TAT	CAG	GAA	3	G	C
TCGA-20-1685	DST_ENST0(	ENST00000244364	p.E1850Q	c.5548G>C	Unverified	ATG	GAG	GAG	1	G	C
TCGA-29-1701	DST_ENST0(	ENST00000370765	p.Q2421H	c.7263G>C	Unverified	TAT	CAG	GAA	3	G	C
TCGA-20-1685	DST_ENST0(	ENST00000370769	p.E4264Q	c.12790G>C	Unverified	ATG	GAG	GAG	1	G	C
TCGA-24-1843	DTL	ENST00000366991	p.S679T	c.2036G>C	Unverified	CGA	AGT	CCG	2	G	C
TCGA-13-0885	DTNA_ENST	ENST00000269190	p.G277R	c.829G>C	Verified	GCC	GGT	GGT	1	G	C
TCGA-24-1843	DTX3L	ENST00000296161	p.S233T	c.698G>C	Unverified	AAA	AGC	AAC	2	G	C
TCGA-13-0761	DYNC1LI1	ENST00000273130	p.L451F	c.1353G>C	Verified	AGT	TTG	TTG	3	G	C
TCGA-13-0761	DYNC1LI1	ENST00000273130	p.L451F	c.1353G>C	Verified	AGT	TTG	TTG	3	G	C
TCGA-23-2077	DYRK1B	ENST00000348817	p.Q72H	c.216G>C	Verified	gcc	cag	cag	3	G	C

TCGA-29-1781	DYTN	ENST00000452335	p.A558P	c.1672G>C	Unverified	GGA	GCT	CAG	1	G	C
TCGA-29-1781	DYTN_ENST(	ENST00000452335	p.A558P	c.1672G>C	Unverified	GGA	GCT	CAG	1	G	C
TCGA-24-1103	DZIP1L	ENST00000327532	p.Q122H	c.366G>C	Verified	CAG	CAG	CAG	3	G	C
TCGA-13-0762	DZIP3	ENST00000361582	p.V567L	c.1699G>C	Verified	TCT	GTC	TAC	1	G	C
TCGA-13-0762	DZIP3	ENST00000361582	p.V567L	c.1699G>C	Verified	TCT	GTC	TAC	1	G	C
TCGA-23-1117	EAPP	ENST00000250454	p.R155T	c.464G>C	Verified	CAG	AGA	AGG	2	G	C
TCGA-23-1117	EAPP	ENST00000250454	p.R155T	c.464G>C	Verified	CAG	AGA	AGG	2	G	C
TCGA-20-1686	EBAG9	ENST00000395785	p.R24T	c.71G>C	Unverified	aag	aga	tta	2	G	C
TCGA-13-1510	EBI3	ENST00000221847	p.V10L	c.28G>C	Unverified	CTT	GTC	CTC	1	G	C
TCGA-29-1781	ECSIT	ENST00000270517	p.E235Q	c.703G>C	Unverified	ATG	GAG	CCT	1	G	C
TCGA-04-1338	ECT2	ENST00000232458	p.E45D	c.135G>C	Verified	gaa	gag	atg	3	G	C
TCGA-24-2290	EFNB3	ENST00000226091	p.G143G	c.429G>C	Verified	GAT	GGG	ACC	3	G	C
TCGA-24-2290	EFNB3	ENST00000226091	p.G143G	c.429G>C	Verified	GAT	GGG	ACC	3	G	C
TCGA-13-0884	EFR3A	ENST00000254624	p.V45L	c.133G>C	Verified	GCA	GTA	TCT	1	G	C
TCGA-13-0900	EGFR	ENST00000275493	p.E709Q	c.2125G>C	Unverified	AAG	GAA	ACT	1	G	C
TCGA-29-1761	EHD3	ENST00000322054	p.A465P	c.1393G>C	Unverified	GGC	GCT	AAT	1	G	C
TCGA-23-1110	EHHADH	ENST00000231887	p.V480V	c.1440G>C	Verified	TTT	GTG	GGG	3	G	C
TCGA-24-0979	EIF2AK3	ENST00000303236	p.E300Q	c.898G>C	Verified	GAA	GAA	CAG	1	G	C
TCGA-61-1740	EIF2B5	ENST00000273783	p.V435V	c.1305G>C	Unverified	CAG	GTG	GTC	3	G	C
TCGA-13-0807	EIF3E	ENST00000220849	p.E194D	c.582G>C	Verified	aaa	gag	acc	3	G	C
TCGA-13-0900	EIF4A2	ENST00000323963	p.M316I	c.948G>C	Verified	ATC	ATG	AGG	3	G	C
TCGA-13-0920	EIF4A3	ENST00000269349	p.A377P	c.1129G>C	Verified	gtg	gcc	att	1	G	C
TCGA-30-1855	EIF6	ENST00000374436	p.E127Q	c.379G>C	Unverified	gaa	gaa	att	1	G	C
TCGA-04-1362	ELK1	ENST00000247161	p.K35N	c.105G>C	Verified	TTC	AAG	CTG	3	G	C
TCGA-61-1904	ELN	ENST00000252034	p.G78G	c.234G>C	Unverified	GCA	GGG	CTC	3	G	C
TCGA-13-0900	ELP4	ENST00000350638	p.G346A	c.1037G>C	Verified	CAT	GGA	TTG	2	G	C
TCGA-30-1856	ELP4	ENST00000350638	p.S22T	c.65G>C	Unverified	GCC	AGC	AAG	2	G	C
TCGA-30-1856	ELP4_ENST0	ENST00000395934	p.S22T	c.65G>C	Unverified	GCC	AGC	AAG	2	G	C
TCGA-13-0920	EML4	ENST00000318522	p.V782L	c.2344G>C	Verified	GGT	GTC	TGG	1	G	C
TCGA-23-1022	ENC1	ENST00000302351	p.G347R	c.1039G>C	Verified	GGG	GGG	CGG	1	G	C
TCGA-23-1124	ENPP1	ENST00000360971	p.E854Q	c.2560G>C	Verified	AAA	GAG	CCA	1	G	C
TCGA-13-0885	ENPP7	ENST00000328313	p.V36L	c.106G>C	Verified	CTG	GTG	TCC	1	G	C
TCGA-23-1022	ENSG00000:	ENST00000341450	p.S26T	c.77G>C	Verified	GGG	AGT	TTG	2	G	C
TCGA-30-1857	ENSG00000;	ENST00000400226	p.E208Q	c.622G>C	Unverified	TTT	GAG	AAA	1	G	C

TCGA-04-1338	ENTPD5	ENST00000334696	p.A131P	c.391G>C	Verified	ACA	GCA	GGA	1	G	C
TCGA-25-1326	EPB41	ENST00000349460	p.W481S	c.1442G>C	Verified	GAA	TGG	GAT	2	G	C
TCGA-24-1843	EPB41L2	ENST00000368128	p.R318P	c.953G>C	Unverified	CTC	CGG	CAG	2	G	C
TCGA-24-2288	EPC1	ENST00000263062	p.E285Q	c.853G>C	Unverified	TCT	GAG	GTT	1	G	C
TCGA-61-2009	EPDR1	ENST00000559325	p.G108R	c.322G>C	Unverified	TTG	GGC	TTG	1	G	C
TCGA-24-1422	EPDR1	ENST00000559325	p.S113S	c.339G>C	Unverified	CCC	TCG	GGC	3	G	C
TCGA-23-1120	EPG5	ENST00000282041	p.E1162Q	c.3484G>C	Unverified	CGA	GAA	CAA	1	G	C
TCGA-24-1103	EPHA2	ENST00000358432	p.W912C	c.2736G>C	Verified	GAG	TGG	CTG	3	G	C
TCGA-09-2049	EPHX1	ENST00000366837	p.G245R	c.733G>C	Verified	AAA	GGC	CTG	1	G	C
TCGA-24-1563	EPHX2	ENST00000521400	p.S238T	c.713G>C	Verified	ATG	AGC	CAT	2	G	C
TCGA-04-1361	EPS15L1	ENST00000248070	p.D820H	c.2458G>C	Verified	GCT	GAC	AGC	1	G	C
TCGA-04-1361	EPS15L1	ENST00000248070	p.D820H	c.2458G>C	Verified	GCT	GAC	AGC	1	G	C
TCGA-23-1123	EPS15L1	ENST00000248070	p.G731A	c.2192G>C	Verified	TTC	GGA	AGT	2	G	C
TCGA-23-1123	EPS15L1	ENST00000248070	p.G731A	c.2192G>C	Verified	TTC	GGA	AGT	2	G	C
TCGA-29-1784	ERBB3	ENST00000267101	p.R491T	c.1472G>C	Unverified	CGC	AGA	GAC	2	G	C
TCGA-29-1784	ERBB3_ENST	ENST00000267101	p.R491T	c.1472G>C	Unverified	CGC	AGA	GAC	2	G	C
TCGA-09-2049	ERI3	ENST00000372257	p.?	c.666+1G>C	Verified	CTG	GAG	AGG	3	G	C
TCGA-23-2077	ERMP1	ENST00000339450	p.P681P	c.2043G>C	Verified	AAT	CCG	AAG	3	G	C
TCGA-13-1481	ERN1_ENST(	ENST00000433197	p.G50A	c.149G>C	Verified	ACA	GGC	TCA	2	G	C
TCGA-23-2077	ESRP2	ENST00000473183	p.E535D	c.1605G>C	Verified	AAG	GAG	CGC	3	G	C
TCGA-61-2109	ESYT1	ENST00000394048	p.W615C	c.1845G>C	Unverified	GCT	TGG	GAC	3	G	C
TCGA-13-0913	ETAA1	ENST00000272342	p.S630T	c.1889G>C	Verified	GAC	AGT	AAG	2	G	C
TCGA-29-1695	ETNK1	ENST00000266517	p.G146A	c.437G>C	Unverified	GAT	GGA	ATC	2	G	C
TCGA-29-1695	ETNK1_ENST	ENST00000335148	p.G146A	c.437G>C	Unverified	GAT	GGA	ATC	2	G	C
TCGA-61-2009	ETV6	ENST00000396373	p.G375R	c.1123G>C	Verified	aac	gga	ctg	1	G	C
TCGA-13-1505	EXOC3L4	ENST00000380069	p.E649D	c.1947G>C	Verified	CTG	GAG	ACT	3	G	C
TCGA-29-1711	EXOC6B_EN	ENST00000272427	p.E45Q	c.133G>C	Unverified	GAA	GAA	CAT	1	G	C
TCGA-29-1711	EXOC6B_EN	ENST00000272427	p.E45Q	c.133G>C	Unverified	GAA	GAA	CAT	1	G	C
TCGA-24-1422	EYA2	ENST00000327619	p.V270V	c.810G>C	Verified	CGT	GTG	TTC	3	G	C
TCGA-23-1031	EYS	ENST00000342421	p.E335D	c.1005G>C	Verified	agt	gag	ttt	3	G	C
TCGA-13-1497	F8	ENST00000360256	p.A2227P	c.6679G>C	Verified	AAA	GCT	CGA	1	G	C
TCGA-13-1497	F8_ENST000	ENST00000360256	p.A2227P	c.6679G>C	Verified	AAA	GCT	CGA	1	G	C
TCGA-04-1356	FADD	ENST00000301838	p.R114S	c.342G>C	Verified	aga	agg	ctg	3	G	C
TCGA-61-1725	FAM113B	ENST00000546455	p.R49T	c.146G>C	Unverified	ctt	aga	gca	2	G	C

TCGA-24-2288	FAM118B	ENST00000533050	p.E285D	c.855G>C	Unverified	GAT	GAG	TTC	3	G	C
TCGA-29-1769	FAM120C	ENST00000375180	p.D521H	c.1561G>C	Unverified	CCA	GAC	TCT	1	G	C
TCGA-23-1111	FAM127C	ENST00000391440	p.E108Q	c.322G>C	Unverified	tgg	gag	gag	1	G	C
TCGA-13-1501	FAM134A	ENST00000430297	p.E141D	c.423G>C	Verified	AGT	GAG	GGT	3	G	C
TCGA-13-1501	FAM134A	ENST00000430297	p.E141D	c.423G>C	Verified	AGT	GAG	GGT	3	G	C
TCGA-23-1021	FAM184A	ENST00000338891	p.E624Q	c.1870G>C	Verified	AAA	GAA	GAA	1	G	C
TCGA-25-1313	FAM189B	ENST00000361361	p.R549R	c.1647G>C	Verified	GCC	CGG	TCA	3	G	C
TCGA-30-1856	FAM196B	ENST00000377365	p.V166L	c.496G>C	Unverified	AAG	GTG	TCC	1	G	C
TCGA-13-1507	FAM19A2	ENST00000416284	p.E60Q	c.178G>C	Verified	GAA	GAA	CGG	1	G	C
TCGA-23-2077	FAM21C	ENST00000374362	p.K744N	c.2232G>C	Verified	GAT	AAG	AAG	3	G	C
TCGA-20-1686	FAM40B	ENST00000249344	p.V311L	c.931G>C	Unverified	GTG	GTG	AAG	1	G	C
TCGA-09-2050	FAM75D4	ENST00000344803	p.G730R	c.2188G>C	Verified	CAT	GGA	CCG	1	G	C
TCGA-24-1103	FAM83C	ENST00000374408	p.L713L	c.2139G>C	Verified	GAC	CTG	GTC	3	G	C
TCGA-13-0884	FAM98B	ENST00000491535	p.E78Q	c.232G>C	Verified	CTA	GAG	AGC	1	G	C
TCGA-61-2009	FASTKD2	ENST00000236980	p.V369V	c.1107G>C	Verified	AAG	GTG	GTC	3	G	C
TCGA-04-1338	FAT1	ENST00000441802	p.E3486D	c.10458G>C	Verified	GAT	GAG	AAG	3	G	C
TCGA-29-1762	FAT4	ENST00000330166	p.S3323T	c.9968G>C	Unverified	GCT	AGC	GAC	2	G	C
TCGA-29-1762	FAT4_ENST(	ENST00000394329	p.S3323T	c.9968G>C	Unverified	GCT	AGC	GAC	2	G	C
TCGA-25-1326	FBN1	ENST00000316623	p.C313S	c.938G>C	Verified	TTT	TGC	AAA	2	G	C
TCGA-25-2392	FBXL18	NM_024963.2	p.D582H	c.1744G>C	Unverified	tca	gac	atg	1	G	C
TCGA-23-1122	FBXO18	ENST00000379999	p.E995Q	c.2983G>C	Verified	ggg	gag	tac	1	G	C
TCGA-61-1913	FBXO24	ENST00000241071	p.R442P	c.1325G>C	Unverified	ggc	cgc	ctc	2	G	C
TCGA-23-1022	FBXO24	ENST00000241071	p.Q93H	c.279G>C	Verified	gat	cag	ggt	3	G	C
TCGA-61-1913	FBXO24_EN:	ENST00000427939	p.R480P	c.1439G>C	Unverified	GGC	CGC	CTC	2	G	C
TCGA-09-2050	FBXO38	ENST00000340253	p.E885Q	c.2653G>C	Verified	tca	gaa	gta	1	G	C
TCGA-23-1117	FBXW10	ENST00000395667	p.K374N	c.1122G>C	Verified	ACG	AAG	AAT	3	G	C
TCGA-23-1117	FBXW10	ENST00000395667	p.K374N	c.1122G>C	Verified	ACG	AAG	AAT	3	G	C
TCGA-13-0883	FBXW9	NM_032301.1	p.C52S	c.155G>C	Verified	gcc	tgc	tgc	2	G	C
TCGA-24-1104	FCAMR	ENST00000400962	p.E21Q	c.61G>C	Verified	AGA	GAA	GTG	1	G	C
TCGA-61-1738	FCGBP	ENST00000221347	p.S914T	c.2741G>C	Unverified	GGC	AGC	CAG	2	G	C
TCGA-61-1738	FCGBP	ENST00000221347	p.S914T	c.2741G>C	Unverified	GGC	AGC	CAG	2	G	C
TCGA-29-1711	FCGR1A	ENST00000369168	p.D324H	c.970G>C	Unverified	TGG	GAT	TTA	1	G	C
TCGA-29-1711	FCGR1A	ENST00000369168	p.D324H	c.970G>C	Unverified	TGG	GAT	TTA	1	G	C
TCGA-04-1356	FCN1	ENST00000371806	p.K23N	c.69G>C	Verified	ATC	AAG	AAC	3	G	C

TCGA-24-1849	FCRL5	ENST00000361835	p.E920Q	c.2758G>C	Unverified	GGA	GAA	AAT	1	G	C
TCGA-13-0760	FCRL6	ENST00000368106	p.V390L	c.1168G>C	Verified	ACC	GTG	GGG	1	G	C
TCGA-09-0369	FEZF1	ENST00000442488	p.Q225H	c.675G>C	Verified	GCT	CAG	CTG	3	G	C
TCGA-09-0369	FEZF1	ENST00000442488	p.Q225H	c.675G>C	Verified	GCT	CAG	CTG	3	G	C
TCGA-29-1705	FGD2	ENST00000274963	p.L173L	c.519G>C	Unverified	CGC	CTG	GAC	3	G	C
TCGA-24-0979	FGD5	NM_152536.2	p.G287R	c.859G>C	Verified	gag	ggg	aag	1	G	C
TCGA-29-1698	FGD6	ENST00000343958	p.V1251L	c.3751G>C	Unverified	ATT	GTA	TGC	1	G	C
TCGA-29-1699	FILIP1L	ENST00000331335	p.E294Q	c.880G>C	Unverified	AAG	GAA	CTG	1	G	C
TCGA-29-1699	FILIP1L_ENS	ENST00000354552	p.E294Q	c.880G>C	Unverified	AAG	GAA	CTG	1	G	C
TCGA-13-1509	FLAD1	ENST00000292180	p.E198Q	c.592G>C	Verified	GAT	GAG	CTG	1	G	C
TCGA-24-2280	FLG2	ENST00000388718	p.L572F	c.1716G>C	Unverified	GGG	TTG	GGC	3	G	C
TCGA-61-1900	FLJ43860	ENST00000599122	p.G1147R	c.3439G>C	Unverified	TTC	GGG	GAC	1	G	C
TCGA-04-1361	FLJ90650	ENST00000357872	p.?	c.1516-1G>C	Verified	AAG	GGA	GCG	1	G	C
TCGA-04-1361	FLJ90650	ENST00000357872	p.?	c.1516-1G>C	Verified	AAG	GGA	GCG	1	G	C
TCGA-61-1906	FLNB	ENST00000295956	p.D130H	c.388G>C	Unverified	GAG	GAT	GAA	1	G	C
TCGA-61-1906	FLNB	ENST00000295956	p.D130H	c.388G>C	Unverified	GAG	GAT	GAA	1	G	C
TCGA-24-1435	FLNB	ENST00000295956	p.E1305D	c.3915G>C	Verified	GTG	GAG	GTG	3	G	C
TCGA-61-1906	FLNB_ENST(	ENST00000490882	p.D130H	c.388G>C	Unverified	GAG	GAT	GAA	1	G	C
TCGA-61-1906	FLNB_ENST(	ENST00000490882	p.D130H	c.388G>C	Unverified	GAG	GAT	GAA	1	G	C
TCGA-29-1693	FLNC	ENST00000325888	p.V326V	c.978G>C	Unverified	AAG	GTG	GTT	3	G	C
TCGA-25-2391	FLT3	ENST00000241453	p.V491L	c.1471G>C	Unverified	AAA	GTG	TTT	1	G	C
TCGA-24-2262	FMN2	ENST00000319653	p.G1128A	c.3383G>C	Unverified	GCG	GGC	ATA	2	G	C
TCGA-25-2400	FMR1NB	ENST00000370467	p.V198L	c.592G>C	Verified	ctg	gta	tgt	1	G	C
TCGA-13-1497	FOSL2	ENST00000264716	p.E289Q	c.865G>C	Verified	CTG	GAG	CAG	1	G	C
TCGA-09-1665	FOXG1	ENST00000382535	p.L460F	c.1380G>C	Verified	tct	ttg	cca	3	G	C
TCGA-29-1769	FOXP4	ENST00000373063	p.T42T	c.126G>C	Unverified	ggc	acg	ggc	3	G	C
TCGA-29-1769	FOXP4_ENS1	ENST00000373060	p.T42T	c.126G>C	Unverified	GGC	ACG	GGC	3	G	C
TCGA-25-1313	FRMPD1	ENST00000377765	p.A309P	c.925G>C	Verified	CTC	GCG	GCT	1	G	C
TCGA-20-1686	FRMPD2	ENST00000374201	p.R844S	c.2532G>C	Unverified	GTT	AGG	ATG	3	G	C
TCGA-24-2289	FRYL	ENST00000503238	p.V1345L	c.4033G>C	Unverified	ATG	GTG	ACT	1	G	C
TCGA-04-1338	FSTL5	ENST00000306100	p.D212H	c.634G>C	Verified	AAG	GAT	CTC	1	G	C
TCGA-13-0919	FTSJD2	ENST00000373451	p.E370D	c.1110G>C	Verified	GTG	GAG	GGG	3	G	C
TCGA-25-2042	FUK	ENST00000288078	p.E1041D	c.3123G>C	Unverified	AAG	GAG	GCC	3	G	C
TCGA-23-1120	FYB_ENST0(	ENST00000351578	p.R232T	c.695G>C	Verified	GTC	AGG	TCC	2	G	C

TCGA-24-2289	FZD2	ENST00000315323	p.P505P	c.1515G>C	Unverified	atc	ccg	tgc	3	G	C
TCGA-29-1711	GABRA4	ENST00000264318	p.D53H	c.157G>C	Unverified	CTG	GAC	AGT	1	G	C
TCGA-29-1711	GABRA4	ENST00000264318	p.D53H	c.157G>C	Unverified	CTG	GAC	AGT	1	G	C
TCGA-13-0905	GABRA6	ENST00000274545	p.V255L	c.763G>C	Verified	ACA	GTC	ATT	1	G	C
TCGA-04-1337	GABRB1	ENST00000295454	p.D450H	c.1348G>C	Verified	ATA	GAC	AAG	1	G	C
TCGA-29-1784	GANAB	ENST00000356638	p.A534P	c.1600G>C	Unverified	TCA	GCT	CCC	1	G	C
TCGA-25-1313	GANAB	ENST00000356638	p.R493R	c.1479G>C	Verified	ACC	CGG	GAT	3	G	C
TCGA-29-1764	GARNL3	ENST00000373387	p.K1013N	c.3039G>C	Unverified	TTG	AAG	TAA	3	G	C
TCGA-04-1338	GART	ENST00000381839	p.G475R	c.1423G>C	Verified	GCT	GGT	CTT	1	G	C
TCGA-13-0920	GBF1	ENST00000369983	p.L143L	c.429G>C	Verified	ACT	CTG	CTG	3	G	C
TCGA-24-1846	GFI1	ENST00000294702	p.A47A	c.141G>C	Unverified	GGG	GCG	AAG	3	G	C
TCGA-29-1701	GFOD1	ENST00000379287	p.A101P	c.301G>C	Unverified	GAC	GCT	TTC	1	G	C
TCGA-23-1031	GGNBP2	ENST00000304718	p.D579H	c.1735G>C	Verified	AAA	GAT	ACA	1	G	C
TCGA-24-2280	GHR	ENST00000230882	p.C390S	c.1169G>C	Unverified	tgt	tgt	gaa	2	G	C
TCGA-29-1763	GIGYF2	ENST00000373563	p.G276G	c.828G>C	Unverified	AGT	GGG	AGC	3	G	C
TCGA-29-1763	GIGYF2_ENS	ENST00000409451	p.G298G	c.894G>C	Unverified	AGT	GGG	AGC	3	G	C
TCGA-23-1111	GIMAP2	ENST00000223293	p.V95L	c.283G>C	Unverified	GAG	GTG	CAG	1	G	C
TCGA-23-1124	GINS1	ENST00000262460	p.G133R	c.397G>C	Verified	GAA	GGT	TTG	1	G	C
TCGA-09-0369	GJA1	ENST00000282561	p.V289L	c.865G>C	Verified	CTG	GTT	ACT	1	G	C
TCGA-09-0369	GJA1	ENST00000282561	p.V289L	c.865G>C	Verified	CTG	GTT	ACT	1	G	C
TCGA-30-1855	GJA10	ENST00000369352	p.W78C	c.234G>C	Unverified	TTC	TGG	GTT	3	G	C
TCGA-30-1855	GLB1L	ENST00000295759	p.E184D	c.552G>C	Unverified	GTG	GAG	AAT	3	G	C
TCGA-13-0807	GLDC	ENST00000321612	p.A498P	c.1492G>C	Verified	GTT	GCT	GAA	1	G	C
TCGA-13-1510	GLIPR1L2	ENST00000320460	p.A88P	c.262G>C	Unverified	ACT	GCT	AGA	1	G	C
TCGA-13-0906	GLRA3	ENST00000274093	p.D45H	c.133G>C	Verified	TCT	GAT	TTT	1	G	C
TCGA-23-1110	GMPR2	ENST00000399440	p.V295L	c.883G>C	Verified	GAA	GTT	CCT	1	G	C
TCGA-24-0975	GMPR2	ENST00000399440	p.G242R	c.724G>C	Verified	CTG	GGT	GGC	1	G	C
TCGA-61-1998	GMPR2	ENST00000399440	p.?	c.547+1G>C	Verified	CCA	GGC	TCT	1	G	C
TCGA-24-1469	GNAL	ENST00000334049	p.S413T	c.1238G>C	Verified	ATC	AGC	ACG	2	G	C
TCGA-23-1116	GNG13	ENST00000248150	p.Q8H	c.24G>C	Unverified	CCA	CAG	ATG	3	G	C
TCGA-13-1497	GOLGA3	ENST00000204726	p.M687I	c.2061G>C	Verified	AGG	ATG	GCG	3	G	C
TCGA-24-1844	GOLGA4	ENST00000361924	p.S1308T	c.3923G>C	Unverified	AAG	AGC	ATG	2	G	C
TCGA-09-2050	GOLGA5	ENST00000163416	p.E476Q	c.1426G>C	Verified	AAA	GAG	ATG	1	G	C
TCGA-13-0761	GPNMB	ENST00000381990	p.W168S	c.503G>C	Verified	GGA	TGG	AGA	2	G	C

TCGA-13-0761	GPNMB	ENST00000381990	p.W168S	c.503G>C	Verified	GGA	TGG	AGA	2	G	C
TCGA-61-2102	GPR110	ENST00000371253	p.V215L	c.643G>C	Verified	GTT	GTT	GGC	1	G	C
TCGA-13-1501	GPR124	ENST00000412232	p.G392G	c.1176G>C	Unverified	GGC	GGG	GGT	3	G	C
TCGA-13-1501	GPR124	ENST00000412232	p.G392G	c.1176G>C	Unverified	GGC	GGG	GGT	3	G	C
TCGA-30-1718	GPR15	ENST00000284311	p.W89C	c.267G>C	Unverified	CTC	TGG	GTG	3	G	C
TCGA-29-1761	GPR155	ENST00000392551	p.E8Q	c.22G>C	Unverified	GCA	GAG	AAC	1	G	C
TCGA-61-1725	GPR160	ENST00000355897	p.R212T	c.635G>C	Unverified	ATC	AGG	ATA	2	G	C
TCGA-24-1423	GPR179	ENST00000342292	p.C387S	c.1160G>C	Unverified	GCC	TGC	CAG	2	G	C
TCGA-23-1022	GPR55	ENST00000392039	p.A230P	c.688G>C	Verified	GCA	GCC	AGC	1	G	C
TCGA-04-1361	GPR98	ENST00000405460	p.E3975Q	c.11923G>C	Verified	CTT	GAA	TTT	1	G	C
TCGA-04-1361	GPR98	ENST00000405460	p.E3975Q	c.11923G>C	Verified	CTT	GAA	TTT	1	G	C
TCGA-13-0795	GPR98	ENST00000405460	p.R3405P	c.10214G>C	Verified	GTC	CGA	GGT	2	G	C
TCGA-24-2024	GRB2	ENST00000392564	p.E130D	c.390G>C	Verified	aat	gag	ctg	3	G	C
TCGA-24-2288	GRIA4	ENST00000282499	p.A699P	c.2095G>C	Unverified	TCA	GCA	GAG	1	G	C
TCGA-09-2050	GRIN2D	ENST00000263269	p.?	c.1200+1G>C	Verified	GAG	GTG	GTG	3	G	C
TCGA-09-2050	GRK7	ENST00000264952	p.G352A	c.1055G>C	Verified	gct	gga	acc	2	G	C
TCGA-24-1604	GRM1	ENST00000282753	p.D87H	c.259G>C	Verified	TTG	GAT	AAG	1	G	C
TCGA-13-0755	GRPEL2	ENST00000329271	p.E47Q	c.139G>C	Verified	TCT	GAG	GAC	1	G	C
TCGA-25-2391	GSK3B_ENS	ENST00000316626	p.E121D	c.363G>C	Unverified	GGT	GAG	AAG	3	G	C
TCGA-24-2288	GSR	ENST00000221130	p.V266L	c.796G>C	Verified	AAG	GTA	CTT	1	G	C
TCGA-24-1563	GSTA1	ENST00000334575	p.G144R	c.430G>C	Verified	CAT	GGA	CAA	1	G	C
TCGA-23-2078	GTPBP1	ENST00000216044	p.A140P	c.418G>C	Unverified	GAA	GCT	GGG	1	G	C
TCGA-13-0913	H2AFJ	ENST00000389078	p.E93D	c.279G>C	Verified	GAG	GAG	TTA	3	G	C
TCGA-13-1498	HACE1	ENST00000262903	p.V741L	c.2221G>C	Verified	TAC	GTC	CAG	1	G	C
TCGA-30-1891	HAP1	ENST00000347901	p.M400I	c.1200G>C	Unverified	CGG	ATG	TAT	3	G	C
TCGA-61-1725	HBXIP	ENST00000256644	p.R51P	c.152G>C	Unverified	TTA	CGG	TCC	2	G	C
TCGA-23-1122	HDAC1	ENST00000373548	p.G25A	c.74G>C	Verified	tat	gga	caa	2	G	C
TCGA-24-1469	HDAC3	ENST00000305264	p.G176R	c.526G>C	Unverified	GAC	GGG	GTT	1	G	C
TCGA-09-2049	HDDC3	ENST00000330334	p.D122H	c.364G>C	Verified	GCA	GAC	AAG	1	G	C
TCGA-29-1691	HEATR1	ENST00000366582	p.E642Q	c.1924G>C	Unverified	GAA	GAA	GCT	1	G	C
TCGA-23-1022	HEATR5A	ENST00000389961	p.D378H	c.1132G>C	Verified	AAG	GAT	ATT	1	G	C
TCGA-23-2077	HEATR7B2	ENST00000399564	p.A528P	c.1582G>C	Verified	GGG	GCT	GGT	1	G	C
TCGA-24-1843	HECW2	ENST00000260983	p.R1487T	c.4460G>C	Unverified	gaa	aga	ttc	2	G	C
TCGA-25-1326	HECW2	ENST00000260983	p.Q365H	c.1095G>C	Verified	agc	cag	gtg	3	G	C

TCGA-29-1761	HEPHL1	ENST00000315765	p.W184C	c.552G>C	Unverified	ACC	TGG	GTG	3	G	C
TCGA-61-1740	HERC2	NM_004667.3	p.G635G	c.1905G>C	Unverified	tat	ggg	aaa	3	G	C
TCGA-24-1469	HEXB	ENST00000261416	p.G418R	c.1252G>C	Verified	CCG	GGC	ACA	1	G	C
TCGA-13-0760	HEXDC	ENST00000337014	p.A128P	c.382G>C	Verified	GGC	GCC	ATG	1	G	C
TCGA-25-1313	HIST1H1C	ENST00000343677	p.K109N	c.327G>C	Unverified	aac	aag	aag	3	G	C
TCGA-25-2392	HIST1H2AM	ENST00000359611	p.Q7H	c.21G>C	Verified	AAG	CAG	GGC	3	G	C
TCGA-61-1725	HIST1H2BB	ENST00000357905	p.K25N	c.75G>C	Unverified	AAG	AAG	GAT	3	G	C
TCGA-13-0905	HIST1H2BH	ENST00000356350	p.G61G	c.183G>C	Verified	ATG	GGG	ATC	3	G	C
TCGA-04-1336	HIST1H3B	ENST00000244661	p.E51Q	c.151G>C	Verified	CGC	GAG	ATC	1	G	C
TCGA-61-1900	HIST1H4C	ENST00000377803	p.G42R	c.124G>C	Unverified	CGC	GGT	GGC	1	G	C
TCGA-13-0885	HIST2H2BE	ENST00000369155	p.E77Q	c.229G>C	Verified	GGA	GAG	GCT	1	G	C
TCGA-24-1849	HLCS	ENST00000399120	p.D208H	c.622G>C	Unverified	AGA	GAC	CCG	1	G	C
TCGA-24-1847	HMGB1P1	ENST00000243919	p.R24P	c.71G>C	Unverified	TGT	CGG	GAG	2	G	C
TCGA-24-1431	HMHA1	ENST00000313093	p.C528S	c.1583G>C	Unverified	CTG	TGT	GAG	2	G	C
TCGA-24-1104	HOXA1	ENST00000343060	p.S16T	c.47G>C	Verified	CTT	AGC	AGT	2	G	C
TCGA-23-1032	HOXA11	ENST00000006015	p.D141H	c.421G>C	Unverified	TTC	GAC	CAG	1	G	C
TCGA-24-1464	HOXC4	ENST00000303406	p.G74R	c.220G>C	Verified	CAG	GGG	CCC	1	G	C
TCGA-61-1737	HOXD10	ENST00000249501	p.E220Q	c.658G>C	Unverified	CCC	GAG	GCC	1	G	C
TCGA-24-2280	HPCA	ENST00000373467	p.E120Q	c.358G>C	Unverified	GAG	GAG	ATG	1	G	C
TCGA-29-1781	HPDL	ENST00000334815	p.E271Q	c.811G>C	Unverified	ACT	GAG	GGG	1	G	C
TCGA-29-1762	HPS6	ENST00000299238	p.L335L	c.1005G>C	Unverified	CTG	CTG	GAC	3	G	C
TCGA-61-1740	HPX	ENST00000265983	p.V46V	c.138G>C	Unverified	GAC	GTG	ACT	3	G	C
TCGA-13-0893	HRH2	ENST00000231683	p.L80L	c.240G>C	Verified	CAG	CTG	TCC	3	G	C
TCGA-24-1850	HRNR	ENST00000368801	p.G933R	c.2797G>C	Unverified	TTT	GGT	CAC	1	G	C
TCGA-25-2391	HSF5	ENST00000323777	p.S199T	c.596G>C	Unverified	GAT	AGT	TTG	2	G	C
TCGA-30-1718	HSPA12B	ENST00000254963	p.R202P	c.605G>C	Unverified	gtg	cgc	tgg	2	G	C
TCGA-23-2077	HSPG2	ENST00000374695	p.G2355A	c.7064G>C	Unverified	GAA	GGG	CAG	2	G	C
TCGA-29-1696	HTATSF1	ENST00000535601	p.E665Q	c.1993G>C	Unverified	gaa	gaa	ggt	1	G	C
TCGA-29-1696	HTATSF1	ENST00000535601	p.E665Q	c.1993G>C	Unverified	gaa	gaa	ggt	1	G	C
TCGA-13-0885	HTATSF1	ENST00000535601	p.G3A	c.8G>C	Verified	agc	ggc	acc	2	G	C
TCGA-23-1031	HTR1E	ENST00000369584	p.G91R	c.271G>C	Verified	CTT	GGG	TAC	1	G	C
TCGA-29-1691	HTR2A	ENST00000378688	p.E455D	c.1365G>C	Unverified	GAA	GAG	GCT	3	G	C
TCGA-13-0885	HUWE1	XM_497119.1	p.L3002L	c.9006G>C	Verified	cgt	ctg	ttt	3	G	C
TCGA-30-1855	HYDIN_ENST	ENST00000316490_v65	p.E1326Q	c.3976G>C	Unverified	ATA	GAA	GAA	1	G	C

TCGA-24-1423	ICT1	ENST00000301585	p.R129S	c.387G>C	Verified	AAC	AGG	TTA	3	G	C
TCGA-13-1408	IFRD2	ENST00000336089	p.V247L	c.739G>C	Verified	TAT	GTG	GAC	1	G	C
TCGA-29-1770	IFT140	ENST00000426508	p.G471R	c.1411G>C	Unverified	TCT	GGA	GCC	1	G	C
TCGA-23-1110	IGSF22	ENST00000319338	p.?	c.811-1G>C	Verified	AAG	GGT	ACT	1	G	C
TCGA-24-1470	IGSF22	ENST00000319338	p.V124L	c.370G>C	Verified	CAC	GTG	CTG	1	G	C
TCGA-13-1497	IGSF22	ENST00000319338	p.E474D	c.1422G>C	Verified	GCA	GAG	CTG	3	G	C
TCGA-61-2012	IGSF3	ENST00000369486	p.R1054P	c.3161G>C	Unverified	CTT	CGC	TTC	2	G	C
TCGA-13-1510	IL10RA	ENST00000227752	p.E168D	c.504G>C	Unverified	TAT	GAG	ATT	3	G	C
TCGA-29-1762	IL1RAPL1	ENST00000378993	p.G272A	c.815G>C	Unverified	TTT	GGG	TAC	2	G	C
TCGA-29-1762	IL1RAPL1_EI	ENST00000378993	p.G272A	c.815G>C	Unverified	TTT	GGG	TAC	2	G	C
TCGA-25-2391	IL37	ENST00000263326	p.E185Q	c.553G>C	Unverified	AAT	GAG	CCT	1	G	C
TCGA-24-1563	ILKAP	ENST00000254654	p.L241F	c.723G>C	Verified	ATC	TTG	TGT	3	G	C
TCGA-13-1489	INHA	ENST00000243786	p.C291S	c.872G>C	Verified	TAC	TGT	CAT	2	G	C
TCGA-29-1696	INO80	ENST00000361937	p.E760D	c.2280G>C	Unverified	ATT	GAG	ATT	3	G	C
TCGA-29-1696	INO80	ENST00000361937	p.E760D	c.2280G>C	Unverified	ATT	GAG	ATT	3	G	C
TCGA-29-1696	INOC1	ENST00000361937	p.E760D	c.2280G>C	Unverified	att	gag	att	3	G	C
TCGA-29-1696	INOC1	ENST00000361937	p.E760D	c.2280G>C	Unverified	att	gag	att	3	G	C
TCGA-13-0761	INPP5D_ENS	ENST00000359570	p.S33T	c.98G>C	Verified	GCC	AGC	GAG	2	G	C
TCGA-13-0761	INPP5D_ENS	ENST00000359570	p.S33T	c.98G>C	Verified	GCC	AGC	GAG	2	G	C
TCGA-04-1356	INSRR	ENST00000368195	p.V139L	c.415G>C	Unverified	GCT	GTG	CGT	1	G	C
TCGA-04-1356	INSRR_ENST	ENST00000368195	p.V139L	c.415G>C	Unverified	GCT	GTG	CGT	1	G	C
TCGA-24-1470	INTS3	ENST00000318967	p.W802C	c.2406G>C	Verified	GAC	TGG	GAG	3	G	C
TCGA-61-1915	INTS5	ENST00000330574	p.L941L	c.2823G>C	Unverified	CGT	CTG	CTG	3	G	C
TCGA-29-1777	IQCC	ENST00000291358	p.R326T	c.977G>C	Unverified	CCC	AGA	GGT	2	G	C
TCGA-61-2102	IQGAP2	ENST00000274364	p.G1466A	c.4397G>C	Verified	GAT	GGA	AAA	2	G	C
TCGA-61-1733	IQGAP3	ENST00000361170	p.R1630P	c.4889G>C	Unverified	TTG	CGG	AAG	2	G	C
TCGA-61-1733	IQGAP3	ENST00000361170	p.R1630P	c.4889G>C	Unverified	TTG	CGG	AAG	2	G	C
TCGA-04-1338	IRF6	ENST00000367021	p.D225H	c.673G>C	Verified	ACT	GAC	CTG	1	G	C
TCGA-36-1577	IRS4	ENST00000372129	p.V1047L	c.3139G>C	Unverified	tat	gtg	gtc	1	G	C
TCGA-24-1463	IRX3	ENST00000329734	p.E230Q	c.688G>C	Unverified	GAG	GAG	GAG	1	G	C
TCGA-29-1699	ITGA1	ENST00000282588	p.G483A	c.1448G>C	Unverified	agt	gga	gaa	2	G	C
TCGA-24-1463	ITGA10	ENST00000369304	p.V654L	c.1960G>C	Verified	GAG	GTG	ACC	1	G	C
TCGA-61-1915	ITGA2	NM_002203.2	p.V874L	c.2620G>C	Unverified	tct	gtt	gcc	1	G	C
TCGA-61-1915	ITGA2_ENST	ENST00000296585	p.V874L	c.2620G>C	Unverified	TCT	GTT	GCC	1	G	C

TCGA-29-1777	ITGA2B	ENST00000262407	p.G823A	c.2468G>C	Unverified	cct	ggg	act	2	G	C
TCGA-20-0990	ITGAV	ENST00000261023	p.V347L	c.1039G>C	Verified	TCA	GTG	TCT	1	G	C
TCGA-04-1367	ITGB2	ENST00000397852	p.D300H	c.898G>C	Verified	TTC	GAC	TAC	1	G	C
TCGA-29-1776	ITGB4	ENST00000200181	p.E597Q	c.1789G>C	Unverified	tgt	gag	tgt	1	G	C
TCGA-23-1032	ITIH5	ENST00000256861	p.A532P	c.1594G>C	Verified	ACC	GCC	AGC	1	G	C
TCGA-13-1505	ITK	ENST00000422843	p.A261P	c.781G>C	Verified	GGA	GCC	TTC	1	G	C
TCGA-25-1318	ITPR1	ENST00000456211	p.K2409N	c.7227G>C	Unverified	TTC	AAG	GAT	3	G	C
TCGA-25-1318	ITPR1	ENST00000456211	p.K2409N	c.7227G>C	Unverified	TTC	AAG	GAT	3	G	C
TCGA-61-2109	ITSN1	ENST00000381318	p.G1195A	c.3584G>C	Unverified	AAA	GGA	GAA	2	G	C
TCGA-24-1464	IVNS1ABP	ENST00000367498	p.G600G	c.1800G>C	Verified	GCT	GGG	ATT	3	G	C
TCGA-13-0904	JAKMIP2	ENST00000265272	p.Q34H	c.102G>C	Verified	ATT	CAG	ATA	3	G	C
TCGA-24-2289	JMJD7-PLA2	ENST00000382448	p.S627T	c.1880G>C	Unverified	CCA	AGC	TGC	2	G	C
TCGA-61-2109	KALRN	ENST00000240874	p.R817R	c.2451G>C	Unverified	GAA	CGG	AAG	3	G	C
TCGA-24-1847	KANK1	ENST00000382293	p.E770Q	c.2308G>C	Unverified	CAA	GAA	GTG	1	G	C
TCGA-24-1847	KANK1_ENS	ENST00000382303	p.E928Q	c.2782G>C	Unverified	CAA	GAA	GTG	1	G	C
TCGA-24-1417	KBTBD7	ENST00000379483	p.W578S	c.1733G>C	Unverified	CAA	TGG	AAA	2	G	C
TCGA-25-2401	KCNA1	ENST00000382545	p.G6R	c.16G>C	Unverified	TCT	GGG	GAG	1	G	C
TCGA-23-1117	KCNA10	ENST00000369771	p.G39R	c.115G>C	Verified	AAA	GGC	CGG	1	G	C
TCGA-23-1117	KCNA10	ENST00000369771	p.G39R	c.115G>C	Verified	AAA	GGC	CGG	1	G	C
TCGA-13-0890	KCNA10	ENST00000369771	p.S222S	c.666G>C	Verified	GTC	TCG	GTG	3	G	C
TCGA-13-0887	KCNA2	ENST00000316361	p.E279Q	c.835G>C	Verified	CCA	GAG	GAC	1	G	C
TCGA-20-1683	KCNA6	ENST00000280684	p.G205R	c.613G>C	Unverified	CGA	GGT	GGA	1	G	C
TCGA-24-1849	KCNH8	ENST00000328405	p.L186F	c.558G>C	Unverified	AAA	TTG	AAA	3	G	C
TCGA-61-1907	KCNK10	ENST00000340700	p.E332D	c.996G>C	Unverified	GAA	GAG	GTG	3	G	C
TCGA-61-1907	KCNK10_EN	ENST00000312350	p.E337D	c.1011G>C	Unverified	GAA	GAG	GTG	3	G	C
TCGA-61-1907	KCNK10_EN	ENST00000319231	p.E337D	c.1011G>C	Unverified	GAA	GAG	GTG	3	G	C
TCGA-04-1342	KCNQ1	ENST00000155840	p.A607P	c.1819G>C	Unverified	CTG	GCA	CTC	1	G	C
TCGA-10-0930	KCNQ5	ENST00000370398	p.A351P	c.1051G>C	Verified	TTT	GCA	TTA	1	G	C
TCGA-10-0930	KCNQ5	ENST00000370398	p.A351P	c.1051G>C	Verified	TTT	GCA	TTA	1	G	C
TCGA-29-1766	KCNV2	ENST00000382082	p.R180P	c.539G>C	Unverified	CGC	CGC	TTC	2	G	C
TCGA-13-0916	KCTD7	ENST00000275532	p.V272V	c.816G>C	Verified	CTC	GTG	AAC	3	G	C
TCGA-23-1029	KDM4D	ENST00000335080	p.S430T	c.1289G>C	Unverified	ccc	agc	tca	2	G	C
TCGA-24-1604	KDM5C	ENST00000375401	p.E23Q	c.67G>C	Unverified	GCC	GAG	TTC	1	G	C
TCGA-09-1674	KHDRBS2	ENST00000281156	p.L127F	c.381G>C	Unverified	CAC	TTG	AGT	3	G	C

TCGA-24-1104	KIAA0020	ENST00000397885	p.R181P	c.542G>C	Verified	ACT	CGT	GTG	2	G	C
TCGA-23-1022	KIAA0528	ENST00000333957	p.D819H	c.2455G>C	Verified	ACA	GAT	AAT	1	G	C
TCGA-29-1705	KIAA0889	ENST00000237536	p.G966A	c.2897G>C	Unverified	ACG	GGG	GAC	2	G	C
TCGA-29-1705	KIAA0889_E	ENST00000279034	p.G728A	c.2183G>C	Unverified	ACG	GGG	GAC	2	G	C
TCGA-13-1496	KIAA0913_E	ENST00000398706	p.D1150H	c.3448G>C	Unverified	ATT	GAC	AGC	1	G	C
TCGA-13-1496	KIAA0913_E	ENST00000412131	p.D1145H	c.3433G>C	Unverified	ATT	GAC	AGC	1	G	C
TCGA-04-1655	KIAA1109	ENST00000264501	p.E1817Q	c.5449G>C	Unverified	AAA	GAA	ATT	1	G	C
TCGA-29-1691	KIAA1109	ENST00000264501	p.G3163R	c.9487G>C	Unverified	AAG	GGT	ATT	1	G	C
TCGA-29-2427	KIAA1109	ENST00000264501	p.E726Q	c.2176G>C	Verified	GTA	GAA	ATG	1	G	C
TCGA-61-1737	KIAA1109	ENST00000264501	p.M3054I	c.9162G>C	Unverified	ACC	ATG	TCA	3	G	C
TCGA-29-1693	KIAA1211	ENST00000264229	p.L676L	c.2028G>C	Unverified	ATC	CTG	AAG	3	G	C
TCGA-09-1674	KIAA1383	ENST00000418460	p.R669T	c.2006G>C	Unverified	AAA	AGA	CAA	2	G	C
TCGA-09-1674	KIAA1383_E	ENST00000418460	p.R669T	c.2006G>C	Unverified	AAA	AGA	CAA	2	G	C
TCGA-61-2008	KIAA1409	ENST00000256339	p.G1847R	c.5539G>C	Verified	CTT	GGG	CTA	1	G	C
TCGA-24-2024	KIAA1524	ENST00000295746	p.R530T	c.1589G>C	Verified	CTG	AGA	ATA	2	G	C
TCGA-04-1343	KIAA1529	ENST00000375206	p.?	c.1797+1G>C	Verified	TGT	AAG	AAG	3	G	C
TCGA-23-1124	KIAA1984	ENST00000338005	p.K192N	c.576G>C	Verified	GAC	AAG	CTG	3	G	C
TCGA-29-1764	KIAA2026_E	ENST00000399933_v61	p.D1077H	c.3229G>C	Unverified	ATA	GAT	CTT	1	G	C
TCGA-13-1488	KIF13A	ENST00000259711	p.R1097T	c.3290G>C	Verified	GAG	AGG	TGG	2	G	C
TCGA-24-2289	KIF17	ENST00000247986	p.Q387H	c.1161G>C	Unverified	GTG	CAG	GTG	3	G	C
TCGA-61-2008	KIF21B	ENST00000332129	p.K373N	c.1119G>C	Verified	AAC	AAG	GTG	3	G	C
TCGA-13-0885	KIF26B	ENST00000407071	p.K441N	c.1323G>C	Verified	AAC	AAG	GTG	3	G	C
TCGA-23-1031	KIF2B	ENST00000268919	p.Q430H	c.1290G>C	Verified	TTC	CAG	ATC	3	G	C
TCGA-13-0760	KIF3B	ENST00000375712	p.R507T	c.1520G>C	Verified	GAA	AGA	GAA	2	G	C
TCGA-29-1777	KIF3C	ENST00000264712	p.E166Q	c.496G>C	Unverified	CTA	GAG	CTG	1	G	C
TCGA-61-1737	KIFC1	ENST00000428849	p.E294Q	c.880G>C	Unverified	CTA	GAA	ATG	1	G	C
TCGA-25-2401	KLC4	ENST00000394056	p.W425C	c.1275G>C	Verified	ATC	TGG	ATG	3	G	C
TCGA-25-2391	KLHDC6	ENST00000405256	p.R288T	c.863G>C	Unverified	ATC	AGA	TCA	2	G	C
TCGA-13-0795	KLHL10	ENST00000293303	p.D268H	c.802G>C	Verified	TAT	GAC	CTC	1	G	C
TCGA-29-1696	KRAS	ENST00000311936	p.G12R	c.34G>C	Unverified	gct	ggt	ggc	1	G	C
TCGA-29-1696	KRAS	ENST00000311936	p.G12R	c.34G>C	Unverified	gct	ggt	ggc	1	G	C
TCGA-29-1696	KRAS_ENST(	ENST00000256078	p.G12R	c.34G>C	Unverified	GCT	GGT	GGC	1	G	C
TCGA-29-1696	KRAS_ENST(	ENST00000256078	p.G12R	c.34G>C	Unverified	GCT	GGT	GGC	1	G	C
TCGA-25-1313	KRBA1	ENST00000255992	p.G145A	c.434G>C	Verified	ACT	GGT	GAC	2	G	C

TCGA-29-1785	KRT10	ENST00000269576	p.Q357H	c.1071G>C	Unverified	GAA	CAG	ATA	3	G	C
TCGA-29-1785	KRT10	ENST00000269576	p.Q357H	c.1071G>C	Unverified	GAA	CAG	ATA	3	G	C
TCGA-30-1891	KRT12	ENST00000251643	p.L384L	c.1152G>C	Unverified	CAG	CTG	TCC	3	G	C
TCGA-30-1855	KRT3	ENST00000417996	p.K437N	c.1311G>C	Unverified	AAG	AAG	CAG	3	G	C
TCGA-23-1032	KRT31	ENST00000251645	p.A34P	c.100G>C	Unverified	GGG	GCC	TGC	1	G	C
TCGA-23-1032	KRT31_ENST	ENST00000251645	p.A34P	c.100G>C	Unverified	GGG	GCC	TGC	1	G	C
TCGA-13-0884	KRT6B	ENST00000252252	p.S76T	c.227G>C	Verified	GGC	AGC	TGT	2	G	C
TCGA-29-1783	KRT73	ENST00000305748	p.R55R	c.165G>C	Unverified	GCC	CGG	AGC	3	G	C
TCGA-23-1120	KRT77	ENST00000341809	p.G135A	c.404G>C	Verified	GGG	GGC	ATC	2	G	C
TCGA-20-1685	KRT83	ENST00000293670	p.C222S	c.665G>C	Unverified	GAC	TGC	GCC	2	G	C
TCGA-29-1695	KRT86	ENST00000293525	p.A11P	c.31G>C	Unverified	CGC	GCC	TTC	1	G	C
TCGA-04-1651	KRT9	ENST00000246662	p.K390N	c.1170G>C	Unverified	AGC	AAG	AAA	3	G	C
TCGA-04-1651	KRT9	ENST00000246662	p.K390N	c.1170G>C	Unverified	AGC	AAG	AAA	3	G	C
TCGA-24-1843	KRTAP10-5	ENST00000400372	p.C188S	c.563G>C	Unverified	TGC	TGT	GTG	2	G	C
TCGA-29-1696	KRTAP26-1	ENST00000360542	p.S167T	c.500G>C	Unverified	TCG	AGT	TGC	2	G	C
TCGA-29-1696	KRTAP26-1	ENST00000360542	p.S167T	c.500G>C	Unverified	TCG	AGT	TGC	2	G	C
TCGA-24-1843	KRTAP4-6	ENST00000345847	p.Q128H	c.384G>C	Unverified	TCC	CAG	TGC	3	G	C
TCGA-61-1740	KRTAP9-1	ENST00000398470	p.R226T	c.677G>C	Unverified	TAC	AGG	ACC	2	G	C
TCGA-61-1740	KRTAP9-1_E	ENST00000398470	p.R226T	c.677G>C	Unverified	TAC	AGG	ACC	2	G	C
TCGA-61-2009	KRTAP9-8	ENST00000254072	p.C116S	c.347G>C	Verified	GGT	TGC	CTA	2	G	C
TCGA-61-1738	KYNU	ENST00000264170	p.D250H	c.748G>C	Unverified	TTT	GAT	CTA	1	G	C
TCGA-61-1738	KYNU	ENST00000264170	p.D250H	c.748G>C	Unverified	TTT	GAT	CTA	1	G	C
TCGA-61-1738	KYNU_ENST	ENST00000375773	p.D250H	c.748G>C	Unverified	TTT	GAT	CTA	1	G	C
TCGA-61-1738	KYNU_ENST	ENST00000375773	p.D250H	c.748G>C	Unverified	TTT	GAT	CTA	1	G	C
TCGA-61-1998	L2HGDH	ENST00000267436	p.A62P	c.184G>C	Verified	CTT	GCC	TCT	1	G	C
TCGA-25-1318	LAMA1	ENST00000389658	p.W2285S	c.6854G>C	Unverified	CTA	TGG	AAC	2	G	C
TCGA-25-1318	LAMA1	ENST00000389658	p.W2285S	c.6854G>C	Unverified	CTA	TGG	AAC	2	G	C
TCGA-09-0369	LAMB4	ENST00000205386	p.V1494L	c.4480G>C	Verified	AAC	GTG	CCT	1	G	C
TCGA-09-0369	LAMB4	ENST00000205386	p.V1494L	c.4480G>C	Verified	AAC	GTG	CCT	1	G	C
TCGA-13-0890	LAMP3	ENST00000265598	p.?	c.760-1G>C	Verified	TCG	GTT	TTT	1	G	C
TCGA-61-1900	LAS1L	ENST00000374811	p.C186S	c.557G>C	Unverified	tgg	tgc	cgc	2	G	C
TCGA-24-1435	LAS1L	ENST00000374811	p.T395T	c.1185G>C	Verified	ttc	acg	cag	3	G	C
TCGA-04-1331	LATS1_ENST	ENST00000253339	p.R1020T	c.3059G>C	Verified	CTG	AGA	CAG	2	G	C
TCGA-61-1895	LCLAT1	ENST00000309052	p.K411N	c.1233G>C	Unverified	TCA	AAG	AAA	3	G	C

TCGA-23-1117	LCOR	ENST00000371103	p.V258L	c.772G>C	Verified	CAA	GTT	CGA	1	G	C
TCGA-23-1117	LCOR	ENST00000371103	p.V258L	c.772G>C	Verified	CAA	GTT	CGA	1	G	C
TCGA-24-1435	LCT	ENST00000264162	p.V716L	c.2146G>C	Verified	CAT	GTG	TGG	1	G	C
TCGA-24-2289	LDB3	ENST00000361373	p.G162A	c.485G>C	Unverified	CCT	GGC	CCT	2	G	C
TCGA-24-1422	LDHAL6B	ENST00000307144	p.L222F	c.666G>C	Unverified	ttc	ttg	att	3	G	C
TCGA-23-1124	LECT2	ENST00000274507	p.G76R	c.226G>C	Verified	GTG	GGC	CAG	1	G	C
TCGA-29-1768	LEKR1_ENST	ENST00000356539_v65	p.E628Q	c.1882G>C	Unverified	TCT	GAA	AAA	1	G	C
TCGA-30-1855	LMOD3	ENST00000420581	p.E38Q	c.112G>C	Unverified	TCG	GAA	ATG	1	G	C
TCGA-30-1855	LMOD3_ENS	ENST00000420581	p.E38Q	c.112G>C	Unverified	TCG	GAA	ATG	1	G	C
TCGA-61-1740	LNX1	ENST00000306888	p.G419A	c.1256G>C	Unverified	CCC	GGT	GAA	2	G	C
TCGA-61-1740	LNX1_ENST(	ENST00000263925	p.G515A	c.1544G>C	Unverified	CCC	GGT	GAA	2	G	C
TCGA-13-1496	LOC652153	ENST00000316490	p.D1816H	c.5446G>C	Verified	TTT	GAT	CAG	1	G	C
TCGA-30-1855	LOC652153	ENST00000316490	p.E1278Q	c.3832G>C	Unverified	ATA	GAA	GAA	1	G	C
TCGA-24-2288	LONRF3	ENST00000365713	p.R242P	c.725G>C	Unverified	gcg	cga	gcg	2	G	C
TCGA-24-2262	LOXL3	ENST00000264094	p.G316R	c.946G>C	Unverified	cct	gga	gag	1	G	C
TCGA-23-1032	LOXL4	ENST00000260702	p.G511R	c.1531G>C	Verified	cac	ggg	ccg	1	G	C
TCGA-23-1124	LPAR4	ENST00000435339	p.D307H	c.919G>C	Verified	ttt	gac	cct	1	G	C
TCGA-13-1497	LPCAT3	ENST00000261407	p.W344C	c.1032G>C	Verified	GCC	TGG	GTG	3	G	C
TCGA-09-1674	LPP_ENST0C	ENST00000312675	p.D559H	c.1675G>C	Unverified	TTG	GAT	CGA	1	G	C
TCGA-13-1509	LRCH3	ENST00000334859	p.R350T	c.1049G>C	Verified	CGA	AGA	GAA	2	G	C
TCGA-04-1542	LRFN5	ENST00000298119	p.A695P	c.2083G>C	Verified	AGA	GCA	CAT	1	G	C
TCGA-61-1895	LRIT2	ENST00000372113	p.A384P	c.1150G>C	Unverified	CTT	GCA	GTG	1	G	C
TCGA-61-1899	LRP1B	ENST00000389484	p.S1177S	c.3531G>C	Unverified	TGT	TCG	CTG	3	G	C
TCGA-36-1577	LRP2	ENST00000263816	p.W2523S	c.7568G>C	Unverified	TAC	TGG	GCT	2	G	C
TCGA-04-1362	LRP2	ENST00000263816	p.V4541V	c.13623G>C	Verified	CAG	GTG	ACT	3	G	C
TCGA-30-1857	LRP8	ENST00000306052	p.D862H	c.2584G>C	Unverified	TTT	GAC	AAC	1	G	C
TCGA-09-2050	LRRC16B	ENST00000342740	p.A313P	c.937G>C	Verified	ACT	GCC	ATT	1	G	C
TCGA-24-2267	LRRC23	ENST00000007969	p.E27Q	c.79G>C	Verified	ACA	GAG	GAG	1	G	C
TCGA-29-1693	LRRC3B	ENST00000396641	p.W213C	c.639G>C	Unverified	GGC	TGG	TTC	3	G	C
TCGA-29-1693	LRRC6	ENST00000250173	p.D175H	c.523G>C	Unverified	AAA	GAT	CAC	1	G	C
TCGA-24-1469	LRRC7	ENST00000035383	p.D938H	c.2812G>C	Verified	AAA	GAT	ATC	1	G	C
TCGA-23-1031	LRRC7	ENST00000035383	p.R703P	c.2108G>C	Verified	ACC	CGG	GTT	2	G	C
TCGA-29-1766	LRRC8C	ENST00000370454	p.L777L	c.2331G>C	Unverified	GCT	CTG	AAG	3	G	C
TCGA-13-1488	LRRC8D	ENST00000337338	p.L476L	c.1428G>C	Verified	CAT	CTG	TTC	3	G	C

TCGA-04-1362	LRRIQ1	ENST00000256007	p.W961C	c.2883G>C	Verified	TAC	TGG	AAT	3	G	C
TCGA-09-2049	LRRK1	SU_LRRK1	p.E1413D	c.4239G>C	Verified	aag	gag	cac	3	G	C
TCGA-61-1915	LTBP3	ENST00000322147	p.G792A	c.2375G>C	Unverified	GCT	GGG	GAC	2	G	C
TCGA-13-0883	LTK_ENST00	ENST00000263800	p.W831C	c.2493G>C	Unverified	AGC	TGG	GGA	3	G	C
TCGA-30-1857	LUC7L3	ENST00000505658	p.D203H	c.607G>C	Unverified	GGA	GAT	GCC	1	G	C
TCGA-24-1850	LYG1	ENST00000308528	p.G164R	c.490G>C	Unverified	GGG	GGT	GCT	1	G	C
TCGA-13-0890	LYN	ENST00000519728	p.V303L	c.907G>C	Verified	CTC	GTG	AGG	1	G	C
TCGA-24-2288	MACF1	ENST00000360115	p.V1920L	c.5758G>C	Unverified	AAA	GTG	TTA	1	G	C
TCGA-04-1638	MACF1	ENST00000360115	p.K3970N	c.11910G>C	Unverified	GAA	AAG	ATA	3	G	C
TCGA-04-1638	MACF1_ENS	ENST00000361689	p.K3468N	c.10404G>C	Unverified	GAA	AAG	ATA	3	G	C
TCGA-25-2400	MADD	ENST00000311027	p.V1408L	c.4222G>C	Verified	CTT	GTG	TAC	1	G	C
TCGA-24-2024	MAEA	ENST00000303400	p.K77N	c.231G>C	Verified	GAG	AAG	CTC	3	G	C
TCGA-24-1469	MAGEE1	ENST00000361470	p.E720Q	c.2158G>C	Verified	GCA	GAG	GTA	1	G	C
TCGA-04-1336	MAGI2	ENST00000354212	p.R627R	c.1881G>C	Verified	CAG	CGG	GTG	3	G	C
TCGA-25-1326	MAGI3	ENST00000369615	p.G737A	c.2210G>C	Verified	TTT	GGC	TTC	2	G	C
TCGA-24-1849	MAMDC2	ENST00000377182	p.G507A	c.1520G>C	Unverified	CCT	GGA	GAG	2	G	C
TCGA-09-2050	MAP2	ENST00000360351	p.E837Q	c.2509G>C	Verified	TCA	GAG	ACT	1	G	C
TCGA-24-1474	MAP2	ENST00000360351	p.G1678A	c.5033G>C	Verified	ATC	GGA	TCA	2	G	C
TCGA-25-2400	MAP3K2	ENST00000409947	p.D221H	c.661G>C	Verified	CTT	GAT	AGT	1	G	C
TCGA-13-1498	MAP3K5	ENST00000359015	p.?	c.806+1G>C	Verified	TCT	AGC	CAG	2	G	C
TCGA-29-1770	MAP3K6_EN	ENST00000374040	p.R178R	c.534G>C	Unverified	GGT	CGG	GTG	3	G	C
TCGA-29-1770	MAP3K6_EN	ENST00000493901	p.R186R	c.558G>C	Unverified	GGT	CGG	GTG	3	G	C
TCGA-13-0904	MAP7D1	ENST00000373151	p.Q795H	c.2385G>C	Verified	CAC	CAG	GAG	3	G	C
TCGA-23-1110	MAPKAP1	ENST00000350766	p.R81T	c.242G>C	Verified	ATT	AGA	AGA	2	G	C
TCGA-30-1857	MASP1	ENST00000337774	p.D689H	c.2065G>C	Unverified	AAG	GAC	TGG	1	G	C
TCGA-24-0979	MASP2	ENST00000400897	p.A305P	c.913G>C	Verified	ATG	GCG	CCA	1	G	C
TCGA-29-1699	MASTL	ENST00000375946	p.E403Q	c.1207G>C	Unverified	GTA	GAA	CTG	1	G	C
TCGA-24-1604	MBD4	ENST00000249910	p.R492T	c.1475G>C	Unverified	gca	aga	acc	2	G	C
TCGA-23-1116	MC3R	NM_019888.2	p.G86R	c.256G>C	Verified	ctg	ggc	atc	1	G	C
TCGA-13-1501	MCAM	ENST00000264036	p.R406P	c.1217G>C	Verified	TAT	CGC	TGC	2	G	C
TCGA-13-1501	MCAM	ENST00000264036	p.R406P	c.1217G>C	Verified	TAT	CGC	TGC	2	G	C
TCGA-29-1766	MCM6	ENST00000264156	p.R92R	c.276G>C	Unverified	tgt	cgg	gcc	3	G	C
TCGA-24-1422	MDH1B	ENST00000374412	p.S58T	c.173G>C	Verified	TGG	AGT	CAC	2	G	C
TCGA-25-1313	MDN1	ENST00000369393	p.W4189C	c.12567G>C	Verified	TCA	TGG	GAT	3	G	C

TCGA-09-2056	MED13	ENST00000397786	p.D1087H	c.3259G>C	Verified	AAA	GAC	TGT	1	G	C
TCGA-24-1470	MED29	ENST00000315588	p.D76H	c.226G>C	Verified	TTC	GAT	CCT	1	G	C
TCGA-04-1347	MEGF10	ENST00000274473	p.C191S	c.572G>C	Verified	GAC	TGT	CAT	2	G	C
TCGA-24-1846	MEMO1	ENST00000295065	p.W114S	c.341G>C	Unverified	CTG	TGG	AAG	2	G	C
TCGA-04-1337	MEP1B	ENST00000269202	p.S560T	c.1679G>C	Verified	ACC	AGT	GCC	2	G	C
TCGA-30-1855	METAP2	ENST00000323666	p.G475R	c.1423G>C	Unverified	AGA	GGA	GAT	1	G	C
TCGA-09-0369	MFGE8	ENST00000268150	p.E72Q	c.214G>C	Verified	GTC	GAG	CCA	1	G	C
TCGA-09-0369	MFGE8	ENST00000268150	p.E72Q	c.214G>C	Verified	GTC	GAG	CCA	1	G	C
TCGA-29-1695	MFSD6	ENST00000392328	p.R413S	c.1239G>C	Unverified	GAA	AGG	AAC	3	G	C
TCGA-13-0791	MGC33414	ENST00000329206	p.D36H	c.106G>C	Verified	GTG	GAT	GCT	1	G	C
TCGA-24-2288	MGC42105	ENST00000326035	p.L257F	c.771G>C	Unverified	GCC	TTG	GGG	3	G	C
TCGA-23-1120	MIA3	ENST00000344922	p.Q940H	c.2820G>C	Verified	TTC	CAG	AAG	3	G	C
TCGA-23-2077	MICAL2	ENST00000256194	p.K866N	c.2598G>C	Unverified	ACA	AAG	AAC	3	G	C
TCGA-09-2056	MID1	ENST00000453318	p.A394P	c.1180G>C	Verified	aca	gct	tca	1	G	C
TCGA-13-0762	MID1	ENST00000453318	p.E271Q	c.811G>C	Verified	att	gag	atc	1	G	C
TCGA-13-0762	MID1	ENST00000453318	p.E271Q	c.811G>C	Verified	att	gag	atc	1	G	C
TCGA-24-2024	MKL2	ENST00000318282	p.E578D	c.1734G>C	Verified	AAA	GAG	AAG	3	G	C
TCGA-13-1510	MKLN1	ENST00000352689	p.D671H	c.2011G>C	Unverified	TCA	GAC	CCA	1	G	C
TCGA-61-1910	MKNK1	ENST00000371946	p.E125Q	c.373G>C	Unverified	ttt	gag	aaa	1	G	C
TCGA-23-1124	MKRN1	ENST00000255977	p.Q401H	c.1203G>C	Verified	CCA	CAG	AGA	3	G	C
TCGA-09-1665	MLEC	ENST00000228506	p.R253P	c.758G>C	Verified	AAC	CGG	GTG	2	G	C
TCGA-13-0885	MLL	NM_005933.1	p.D1690H	c.5068G>C	Verified	cct	gat	cca	1	G	C
TCGA-24-1844	MLL	NM_005933.1	p.A1212P	c.3634G>C	Unverified	aaa	gct	gtg	1	G	C
TCGA-24-1844	MLL_ENST0(	ENST00000534358	p.A1212P	c.3634G>C	Unverified	AAA	GCT	GTG	1	G	C
TCGA-10-0930	MLPH	ENST00000264605	p.K412N	c.1236G>C	Verified	GAA	AAG	GCA	3	G	C
TCGA-10-0930	MLPH	ENST00000264605	p.K412N	c.1236G>C	Verified	GAA	AAG	GCA	3	G	C
TCGA-20-1687	MME	ENST00000360490	p.R254T	c.761G>C	Unverified	GCC	AGA	TTG	2	G	C
TCGA-13-0904	MMP2	ENST00000219070	p.V400L	c.1198G>C	Verified	CTC	GTG	GCA	1	G	C
TCGA-24-1474	MMP3	ENST00000299855	p.T277T	c.831G>C	Unverified	CCC	ACG	GAA	3	G	C
TCGA-13-1509	MMS22L	ENST00000275053	p.M945I	c.2835G>C	Verified	GGA	ATG	ATG	3	G	C
TCGA-20-1683	MNAT1	ENST00000261245	p.G270A	c.809G>C	Unverified	CTT	GGG	TAT	2	G	C
TCGA-13-0893	MPDZ	NM_003829.1	p.M1774I	c.5322G>C	Verified	tta	atg	gtg	3	G	C
TCGA-24-1469	MPP1	ENST00000369534	p.K281N	c.843G>C	Verified	ttc	aag	agg	3	G	C
TCGA-09-2050	MRPL23	ENST00000381519	p.R18P	c.53G>C	Verified	CTT	CGG	GTG	2	G	C

TCGA-29-1775	MRPL44	ENST00000258383	p.Q139H	c.417G>C	Unverified	ACA	CAG	TTT	3	G	C
TCGA-20-0990	MSH3	NM_002439.1	p.V283L	c.847G>C	Verified	ttt	gtt	cat	1	G	C
TCGA-24-1845	MSL2	ENST00000309993	p.K450N	c.1350G>C	Unverified	ACC	AAG	ACT	3	G	C
TCGA-24-1423	MTA3	ENST00000405094	p.E308Q	c.922G>C	Verified	ATT	GAA	TAT	1	G	C
TCGA-24-1849	MTF2	ENST00000370298	p.C273S	c.818G>C	Unverified	CTA	TGC	CTT	2	G	C
TCGA-13-1501	MTG1	ENST00000317502	p.V253V	c.759G>C	Unverified	TAC	GTG	CAG	3	G	C
TCGA-13-1501	MTG1	ENST00000317502	p.V253V	c.759G>C	Unverified	TAC	GTG	CAG	3	G	C
TCGA-13-0884	MTHFD1	ENST00000555709	p.G209A	c.626G>C	Verified	AAA	GGT	GAC	2	G	C
TCGA-13-0890	MTHFD1L	ENST00000367321	p.M749I	c.2247G>C	Verified	aag	atg	cat	3	G	C
TCGA-13-0919	MTL5	ENST00000255087	p.G218A	c.653G>C	Verified	GGG	GGC	ACA	2	G	C
TCGA-13-0890	MTMR12	ENST00000382142	p.E378Q	c.1132G>C	Verified	aca	gaa	tgt	1	G	C
TCGA-24-1435	MUC16	ENST00000397910	p.K5103N	c.15309G>C	Verified	GAT	AAG	ATA	3	G	C
TCGA-61-1738	MUC17	ENST00000306151	p.S1528T	c.4583G>C	Unverified	GCC	AGT	TCT	2	G	C
TCGA-61-1738	MUC17	ENST00000306151	p.S1528T	c.4583G>C	Unverified	GCC	AGT	TCT	2	G	C
TCGA-20-0990	MUC4	ENST00000346145	p.W163C	c.489G>C	Unverified	TTC	TGG	GAC	3	G	C
TCGA-20-0990	MUC4_ENST	ENST00000405167	p.W4271C	c.12813G>C	Unverified	TTC	TGG	GAC	3	G	C
TCGA-04-1655	MUSK	ENST00000374448	p.V16L	c.46G>C	Unverified	CTG	GTT	GCC	1	G	C
TCGA-23-1120	MUSK	ENST00000374448	p.E209Q	c.625G>C	Verified	GTT	GAG	GTT	1	G	C
TCGA-24-1469	MYB	ENST00000367814	p.E514Q	c.1540G>C	Unverified	gct	gag	ttt	1	G	C
TCGA-24-1469	MYB_ENST0	ENST00000341911	p.E635Q	c.1903G>C	Unverified	GCT	GAG	TTT	1	G	C
TCGA-13-0762	MYBPC1	ENST00000361466	p.D1023H	c.3067G>C	Verified	GAG	GAT	GCC	1	G	C
TCGA-13-0762	MYBPC1	ENST00000361466	p.D1023H	c.3067G>C	Verified	GAG	GAT	GCC	1	G	C
TCGA-23-1124	MYCBP2_EN	ENST00000357337	p.M4469I	c.13407G>C	Verified	TTA	ATG	AGA	3	G	C
TCGA-24-2035	MYCBPAP	NM_032133.2	p.R259P	c.776G>C	Unverified	cgg	cgt	gat	2	G	C
TCGA-61-2008	MYCBPAP	NM_032133.2	p.R215P	c.644G>C	Verified	ctg	cgg	aag	2	G	C
TCGA-04-1347	MYEF2	ENST00000324324	p.D165H	c.493G>C	Verified	TAT	GAT	CTT	1	G	C
TCGA-61-1899	MYF5	ENST00000228644	p.E132D	c.396G>C	Unverified	ATC	GAG	AGC	3	G	C
TCGA-13-0893	MYH13_ENS	ENST00000570743	p.R30P	c.89G>C	Verified	AAT	CGT	CCA	2	G	C
TCGA-04-1347	MYH4	ENST00000255381	p.G461R	c.1381G>C	Verified	ATC	GGG	GTC	1	G	C
TCGA-13-1488	MYH6	ENST00000356287	p.L1546L	c.4638G>C	Verified	GCC	CTG	GAG	3	G	C
TCGA-29-1777	MYH7	ENST00000355349	p.E1028Q	c.3082G>C	Unverified	CTG	GAG	CAG	1	G	C
TCGA-13-0904	MYH7B	ENST00000262873	p.A1239P	c.3715G>C	Unverified	GTG	GCG	GCA	1	G	C
TCGA-24-1844	MYH8	ENST00000403437	p.V1925L	c.5773G>C	Unverified	CGA	GTG	AAG	1	G	C
TCGA-04-1655	MYH8	ENST00000403437	p.R372P	c.1115G>C	Unverified	CAG	CGT	GAG	2	G	C

TCGA-04-1649	MYO16	ENST00000357550	p.R47S	c.141G>C	Unverified	AAA	AGG	CTG	3	G	C
TCGA-29-1768	MYO16	ENST00000357550	p.L645F	c.1935G>C	Unverified	GTT	TTG	AAA	3	G	C
TCGA-29-1768	MYO18A	ENST00000527372	p.G607R	c.1819G>C	Unverified	GAT	GGC	ACC	1	G	C
TCGA-04-1361	MYO18A_EN	ENST00000527372	p.E1749Q	c.5245G>C	Verified	CTG	GAG	GAA	1	G	C
TCGA-04-1361	MYO18A_EN	ENST00000527372	p.E1749Q	c.5245G>C	Verified	CTG	GAG	GAA	1	G	C
TCGA-29-1768	MYO18A_EN	ENST00000527372	p.G607R	c.1819G>C	Unverified	GAT	GGC	ACC	1	G	C
TCGA-13-1408	MYO18B	ENST00000335473	p.K140N	c.420G>C	Verified	AAA	AAG	ACT	3	G	C
TCGA-09-2044	MYO1B	ENST00000339514	p.W764C	c.2292G>C	Verified	GGT	TGG	AAG	3	G	C
TCGA-29-1777	MYO1E	ENST00000288235	p.S661S	c.1983G>C	Unverified	CAG	TCG	GTC	3	G	C
TCGA-23-1123	MYO1F	ENST00000338257	p.K15N	c.45G>C	Verified	GTG	AAG	CAG	3	G	C
TCGA-23-1123	MYO1F	ENST00000338257	p.K15N	c.45G>C	Verified	GTG	AAG	CAG	3	G	C
TCGA-61-2113	MYO3B	NM_138995	p.D776H	c.2326G>C	Verified	gag	gac	aac	1	G	C
TCGA-23-2078	MYO5A	ENST00000399231	p.W414S	c.1241G>C	Verified	AAC	TGG	ATT	2	G	C
TCGA-61-1899	MYO5B	ENST00000285039	p.?	c.1404+1G>C	Unverified	AAC	TCG	CAT	3	G	C
TCGA-13-0887	MYO7A	ENST00000409709	p.K1737N	c.5211G>C	Verified	TCC	AAG	GCC	3	G	C
TCGA-23-1021	MYO9B_ENS	ENST00000319396	p.V697L	c.2089G>C	Verified	GCA	GTG	CTT	1	G	C
TCGA-04-1336	MYT1	ENST00000328439	p.E1104Q	c.3310G>C	Verified	CCG	GAG	AAC	1	G	C
TCGA-04-1338	N4BP1_ENST	ENST00000262384	p.E274Q	c.820G>C	Verified	GAA	GAG	GCA	1	G	C
TCGA-13-0893	NARS2	ENST00000281038	p.E137D	c.411G>C	Verified	CTG	GAG	TAT	3	G	C
TCGA-24-1604	NAV1	ENST00000367296	p.V1699L	c.5095G>C	Unverified	CTG	GTA	GAG	1	G	C
TCGA-13-1488	NAV2	ENST00000360655	p.E2089Q	c.6265G>C	Verified	GGA	GAA	ATC	1	G	C
TCGA-13-0924	NBEA	ENST00000310336	p.R2219P	c.6656G>C	Verified	AGA	CGT	TAC	2	G	C
TCGA-25-1313	NBEAL1	ENST00000388944	p.L81F	c.243G>C	Verified	CAT	TTG	AGT	3	G	C
TCGA-25-1313	NBEAL1_ENS	ENST00000449802	p.L1371F	c.4113G>C	Verified	CAT	TTG	AGT	3	G	C
TCGA-20-0990	NCCRP1	ENST00000339852	p.W152S	c.455G>C	Verified	AGC	TGG	ACA	2	G	C
TCGA-13-1509	NCF2	ENST00000367536	p.W167C	c.501G>C	Verified	GTC	TGG	AAG	3	G	C
TCGA-23-1029	NCKAP1	ENST00000360982	p.W230C	c.690G>C	Unverified	CAG	TGG	AGA	3	G	C
TCGA-61-1904	NCKAP5L_EI	ENST00000335999	p.R933P	c.2798G>C	Unverified	AAC	CGC	CGC	2	G	C
TCGA-24-2280	NDUFA13	NM_015965.3	p.P95P	c.285G>C	Unverified	cct	ccg	ccg	3	G	C
TCGA-13-0923	NEB	ENST00000409198	p.E6187Q	c.18559G>C	Verified	AAG	GAA	GCA	1	G	C
TCGA-29-1776	NEU4	ENST00000325935	p.G76R	c.226G>C	Unverified	GCC	GGG	GGC	1	G	C
TCGA-13-1510	NEURL	ENST00000369780	p.Q73H	c.219G>C	Unverified	TCC	CAG	ATC	3	G	C
TCGA-25-2391	NEUROD1	ENST00000295108	p.S162T	c.485G>C	Unverified	AAA	AGC	CCA	2	G	C
TCGA-25-2401	NEUROD6	ENST00000297142	p.D226H	c.676G>C	Verified	CTT	GAT	AAT	1	G	C

TCGA-13-0900	NFKBID	ENST00000396901	p.V210L	c.628G>C	Unverified	ACA	GTT	CTG	1	G	C
TCGA-13-1488	NFRKB	ENST00000524794	p.G1147R	c.3439G>C	Verified	TCT	GGA	ACT	1	G	C
TCGA-13-1497	NHEDC1	ENST00000296422	p.K367N	c.1101G>C	Verified	CAA	AAG	ATT	3	G	C
TCGA-13-1489	NID2	ENST00000216286	p.?	c.229-1G>C	Verified	TAC	GTG	GGC	1	G	C
TCGA-61-2008	NLGN1	ENST00000457714	p.V205L	c.613G>C	Verified	atc	gtc	atc	1	G	C
TCGA-13-1510	NLRC5	ENST00000262510	p.A1443P	c.4327G>C	Unverified	TTG	GCT	CAC	1	G	C
TCGA-24-2290	NOD2	ENST00000300589	p.G78A	c.233G>C	Verified	ctg	ggc	cag	2	G	C
TCGA-24-2290	NOD2	ENST00000300589	p.G78A	c.233G>C	Verified	ctg	ggc	cag	2	G	C
TCGA-04-1338	NOS1	ENST00000317775	p.E333D	c.999G>C	Verified	ACT	GAG	TAC	3	G	C
TCGA-04-1542	NOTCH4	ENST00000375023	p.V344V	c.1032G>C	Verified	tgt	gtg	agt	3	G	C
TCGA-13-0920	NOV	ENST00000259526	p.K288N	c.864G>C	Verified	tac	aag	ccc	3	G	C
TCGA-24-2289	NPHP4	ENST00000378156	p.V298L	c.892G>C	Unverified	TTC	GTG	CAG	1	G	C
TCGA-09-1674	NPHS2	ENST00000367615	p.D244H	c.730G>C	Unverified	CAA	GAT	GCA	1	G	C
TCGA-13-0900	NRAP	ENST00000359988	p.K374N	c.1122G>C	Verified	TAT	AAG	AAG	3	G	C
TCGA-24-2290	NRIP2	ENST00000337508	p.E32Q	c.94G>C	Verified	aga	gag	gac	1	G	C
TCGA-24-2290	NRIP2	ENST00000337508	p.E32Q	c.94G>C	Verified	aga	gag	gac	1	G	C
TCGA-29-1762	NRIP3	ENST00000309166	p.V231L	c.691G>C	Unverified	aca	gtc	tct	1	G	C
TCGA-13-0884	NRP1	ENST00000265371	p.G423A	c.1268G>C	Verified	TAC	GGT	TGC	2	G	C
TCGA-61-1900	NRXN3_ENS	ENST00000428277	p.V33L	c.97G>C	Unverified	TCT	GTA	TGG	1	G	C
TCGA-25-2391	NTF3	ENST00000331010	p.G125R	c.373G>C	Unverified	gtg	ggc	agc	1	G	C
TCGA-13-0919	NTRK3	ENST00000394480	p.V501V	c.1503G>C	Verified	ACT	GTG	GTC	3	G	C
TCGA-24-2035	NUBP1	ENST00000283027	p.R145T	c.434G>C	Verified	TGG	AGG	GGA	2	G	C
TCGA-04-1356	NUP160	ENST00000378460	p.E52Q	c.154G>C	Verified	GCT	GAG	CGC	1	G	C
TCGA-20-1685	NUP188	ENST00000372577	p.K1721N	c.5163G>C	Unverified	ggc	aag	tcc	3	G	C
TCGA-24-2288	NUP210	ENST00000254508	p.E1606Q	c.4816G>C	Unverified	CCA	GAG	ACC	1	G	C
TCGA-29-1690	NUP62	ENST00000352066	p.S283T	c.848G>C	Unverified	ACC	AGC	AGC	2	G	C
TCGA-04-1337	NUP85	ENST00000245544	p.E628Q	c.1882G>C	Verified	ATA	GAG	ACC	1	G	C
TCGA-13-0900	NXF3	ENST00000395065	p.Q134H	c.402G>C	Verified	att	cag	aat	3	G	C
TCGA-09-1665	OAS1	ENST00000445409	p.V262L	c.784G>C	Verified	TTA	GTC	ATA	1	G	C
TCGA-61-1895	OAS3	ENST00000228928	p.W340C	c.1020G>C	Unverified	TCT	TGG	AAG	3	G	C
TCGA-13-0885	ODF1	ENST00000285402	p.S5T	c.14G>C	Verified	CTG	AGT	TGT	2	G	C
TCGA-25-2393	ODZ1	ENST00000371130	p.D118H	c.352G>C	Verified	CCT	GAC	CAT	1	G	C
TCGA-29-1761	ODZ1	ENST00000371130	p.G2362A	c.7085G>C	Unverified	GGA	GGA	CTC	2	G	C
TCGA-61-1907	ODZ1	ENST00000371130	p.R2204T	c.6611G>C	Unverified	ACC	AGA	TTA	2	G	C

TCGA-04-1362	ODZ2	ENST00000388903	p.E1167Q	c.3499G>C	Verified	GTA	GAC	AAG	1	G	C
TCGA-23-1114	ODZ2_ENST	ENST00000518659	p.A1232P	c.3694G>C	Unverified	CTT	GCT	GAA	1	G	C
TCGA-23-1114	ODZ2_ENST	ENST00000519204	p.A1111P	c.3331G>C	Unverified	CTT	GCT	GAA	1	G	C
TCGA-25-2401	OLA1	ENST00000284719	p.A171P	c.511G>C	Verified	GTG	GCT	GTG	1	G	C
TCGA-04-1343	OLFM3	ENST00000370103	p.E132Q	c.394G>C	Verified	GAC	GAG	CTC	1	G	C
TCGA-24-1423	OPHN1	ENST00000355520	p.D137H	c.409G>C	Verified	aag	gat	ggt	1	G	C
TCGA-09-2056	OPN4	ENST00000372071	p.K304N	c.912G>C	Verified	TGC	AAG	ATG	3	G	C
TCGA-29-1693	OPTN	ENST00000263036	p.G21R	c.61G>C	Unverified	ACA	GGA	AAT	1	G	C
TCGA-13-0885	OR10AD1	ENST00000310248	p.K140N	c.420G>C	Verified	CAG	AAG	GTC	3	G	C
TCGA-24-1845	OR10C1	ENST00000444197	p.V38L	c.112G>C	Unverified	ACC	GTG	GCA	1	G	C
TCGA-29-1691	OR11H4	ENST00000315409	p.G317A	c.950G>C	Unverified	TTT	GGA	ATG	2	G	C
TCGA-23-1114	OR1J1	ENST00000259357	p.A237P	c.709G>C	Unverified	AAA	GCC	TTG	1	G	C
TCGA-24-1469	OR1N2	ENST00000373688	p.G46A	c.137G>C	Verified	TTT	GGC	ATC	2	G	C
TCGA-61-2109	OR2T5	ENST00000366473	p.V48L	c.142G>C	Unverified	GCT	GTC	CTG	1	G	C
TCGA-13-0886	OR4B1	ENST00000309562	p.R224T	c.671G>C	Verified	TTG	AGG	AAC	2	G	C
TCGA-25-2400	OR4C15	ENST00000314644	p.V299L	c.895G>C	Verified	GCT	GTT	GTG	1	G	C
TCGA-13-0885	OR4D5	ENST00000307033	p.C240S	c.719G>C	Verified	ACC	TGT	GCC	2	G	C
TCGA-24-1850	OR4K14	ENST00000305045	p.L167F	c.501G>C	Unverified	AAT	TTG	CCT	3	G	C
TCGA-04-1342	OR51S1	ENST00000322101	p.R130R	c.390G>C	Verified	GAT	CGG	GCA	3	G	C
TCGA-10-0930	OR5D16	ENST00000378396	p.A35P	c.103G>C	Verified	CTG	GCA	GTC	1	G	C
TCGA-10-0930	OR5D16	ENST00000378396	p.A35P	c.103G>C	Verified	CTG	GCA	GTC	1	G	C
TCGA-23-1032	OR5L1	ENST00000333973	p.V281L	c.841G>C	Verified	GTC	GTG	ATT	1	G	C
TCGA-13-1499	OR6K3	ENST00000368146	p.E127D	c.381G>C	Verified	TCA	GAG	GGG	3	G	C
TCGA-29-1693	OR7A17	ENST00000327462	p.L166L	c.498G>C	Unverified	TGG	CTG	TCC	3	G	C
TCGA-29-1763	OR7D4	ENST00000308682	p.L30L	c.90G>C	Unverified	GGG	CTG	TTC	3	G	C
TCGA-13-0807	OR8B8	ENST00000328064	p.G165R	c.493G>C	Verified	ATG	GGT	GTG	1	G	C
TCGA-61-2008	OS9	ENST00000315970	p.D420H	c.1258G>C	Verified	GAG	GAT	GAG	1	G	C
TCGA-23-1031	OSBP2	ENST00000332585	p.E109Q	c.325G>C	Unverified	TCA	GAG	TCA	1	G	C
TCGA-04-1542	OSBPL1A	ENST00000319481	p.S551T	c.1652G>C	Verified	TGG	AGC	ATC	2	G	C
TCGA-30-1714	OTX1	ENST00000282549	p.S183S	c.549G>C	Unverified	ATC	TCG	CCA	3	G	C
TCGA-04-1336	P2RY11	ENST00000321826	p.G25G	c.75G>C	Verified	AGT	GGG	TTC	3	G	C
TCGA-23-1031	PABPC3	ENST00000281589	p.R287S	c.861G>C	Verified	GAT	AGG	ATC	3	G	C
TCGA-23-1123	PABPC5	ENST00000312600	p.G146A	c.437G>C	Verified	aag	ggt	tat	2	G	C
TCGA-23-1123	PABPC5	ENST00000312600	p.G146A	c.437G>C	Verified	aag	ggt	tat	2	G	C

TCGA-24-2289	PADI6	ENST00000358481	p.V682L	c.2044G>C	Unverified	CGG	GTG	CCC	1	G	C
TCGA-29-1769	PAK2	ENST00000327134	p.E115Q	c.343G>C	Unverified	CTA	GAG	CAA	1	G	C
TCGA-29-1769	PAK2_ENST(	ENST00000327134	p.E115Q	c.343G>C	Unverified	CTA	GAG	CAA	1	G	C
TCGA-13-1481	PALM2-AKA	ENST00000374530	p.G626R	c.1876G>C	Verified	CCC	GGT	GCC	1	G	C
TCGA-13-0923	PALMD	ENST00000263174	p.D200H	c.598G>C	Verified	TCA	GAT	GAC	1	G	C
TCGA-61-1904	PAN2	ENST00000440411	p.D828H	c.2482G>C	Unverified	GGG	GAT	GAG	1	G	C
TCGA-24-0979	PANK3	ENST00000239231	p.E233D	c.699G>C	Verified	GAA	GAG	GCT	3	G	C
TCGA-24-2289	PARP10	ENST00000313028	p.C514S	c.1541G>C	Unverified	TTG	TGC	CTG	2	G	C
TCGA-13-1488	PARP4	ENST00000381989	p.D597H	c.1789G>C	Verified	GAA	GAT	TAC	1	G	C
TCGA-23-1022	PAX7	ENST00000375375	p.V60L	c.178G>C	Verified	ATA	GTG	GAG	1	G	C
TCGA-23-1124	PBX4	ENST00000251203	p.E278Q	c.832G>C	Verified	GAA	GAG	GCT	1	G	C
TCGA-13-0714	PC	ENST00000393955	p.V42V	c.126G>C	Verified	ATG	GTG	GCC	3	G	C
TCGA-61-1725	PCDH1	ENST00000287008	p.G889R	c.2665G>C	Unverified	GCT	GGT	AAG	1	G	C
TCGA-23-1022	PCDH1	ENST00000287008	p.G840G	c.2520G>C	Verified	GCT	GGG	GAT	3	G	C
TCGA-23-1120	PCDH12	ENST00000231484	p.D93H	c.277G>C	Verified	CTG	GAT	CGA	1	G	C
TCGA-24-0975	PCDH12	ENST00000231484	p.L71L	c.213G>C	Verified	CAG	CTG	CCT	3	G	C
TCGA-29-1775	PCDHA10	ENST00000506939	p.E143Q	c.427G>C	Unverified	CCT	GAA	TCC	1	G	C
TCGA-29-1775	PCDHA10_E	ENST00000307360	p.E143Q	c.427G>C	Unverified	CCT	GAA	TCC	1	G	C
TCGA-13-0884	PCDHA2	ENST00000378132	p.G8R	c.22G>C	Verified	AGG	GGC	CGA	1	G	C
TCGA-13-1499	PCDHA4	ENST00000356878	p.V687V	c.2061G>C	Verified	GCT	GTG	GGT	3	G	C
TCGA-29-1763	PCDHA6	ENST00000289630	p.G97R	c.289G>C	Unverified	TGC	GGG	CGG	1	G	C
TCGA-29-1763	PCDHA6_EN	ENST00000529310	p.G97R	c.289G>C	Unverified	TGC	GGG	CGG	1	G	C
TCGA-23-1809	PCDHA8	ENST00000378123	p.G97R	c.289G>C	Unverified	TGC	GGG	CGG	1	G	C
TCGA-23-1809	PCDHA8_EN	ENST00000531613	p.G97R	c.289G>C	Unverified	TGC	GGG	CGG	1	G	C
TCGA-23-1022	PCDHB15	ENST00000231173	p.E522D	c.1566G>C	Verified	TAC	GAG	GCC	3	G	C
TCGA-29-1762	PCDHGB1_E	ENST00000523390	p.E496Q	c.1486G>C	Unverified	CGG	GAG	CTG	1	G	C
TCGA-61-1740	PCDHGC3	ENST00000308177	p.V506L	c.1516G>C	Unverified	CTA	GTG	GGT	1	G	C
TCGA-61-1740	PCDHGC3_E	ENST00000308177	p.V506L	c.1516G>C	Unverified	CTA	GTG	GGT	1	G	C
TCGA-13-0792	PCDHGC4	ENST00000306593	p.V584L	c.1750G>C	Verified	TCA	GTT	GGT	1	G	C
TCGA-61-1725	PCSK9	ENST00000302118	p.V233L	c.697G>C	Unverified	GGG	GTG	GTC	1	G	C
TCGA-23-1122	PDCD1	ENST00000334409	p.R86P	c.257G>C	Verified	GAC	CGC	AGC	2	G	C
TCGA-13-1496	PDCD5	ENST00000221784	p.E85D	c.255G>C	Verified	agt	gag	aag	3	G	C
TCGA-29-2427	PDCD8	ENST00000287295	p.K593N	c.1779G>C	Verified	att	aag	gac	3	G	C
TCGA-13-1501	PDE3A	ENST00000359062	p.S852T	c.2555G>C	Verified	ACT	AGT	GCT	2	G	C

TCGA-13-1501	PDE3A	ENST00000359062	p.S852T	c.2555G>C	Verified	ACT	AGT	GCT	2	G	C
TCGA-61-1740	PDE4D	ENST00000360047	p.E396Q	c.1186G>C	Unverified	TTA	GAG	AAC	1	G	C
TCGA-61-1740	PDE4D_ENS	ENST00000340635	p.E532Q	c.1594G>C	Unverified	TTA	GAG	AAC	1	G	C
TCGA-61-1740	PDE4D_ENS	ENST00000360047	p.E396Q	c.1186G>C	Unverified	TTA	GAG	AAC	1	G	C
TCGA-61-1740	PDE4D_ENS	ENST00000405755	p.E410Q	c.1228G>C	Unverified	TTA	GAG	AAC	1	G	C
TCGA-61-1740	PDE4D_ENS	ENST00000502484	p.E471Q	c.1411G>C	Unverified	TTA	GAG	AAC	1	G	C
TCGA-61-1740	PDE4D_ENS	ENST00000503258	p.E402Q	c.1204G>C	Unverified	TTA	GAG	AAC	1	G	C
TCGA-61-1740	PDE4D_ENS	ENST00000507116	p.E468Q	c.1402G>C	Unverified	TTA	GAG	AAC	1	G	C
TCGA-09-2049	PDLIM3	ENST00000284770	p.G215A	c.644G>C	Verified	CTA	GGG	GAA	2	G	C
TCGA-23-1022	PDSS2	ENST00000369037	p.V223L	c.667G>C	Verified	TTG	GTA	CAA	1	G	C
TCGA-10-0930	PDXP	ENST00000215904	p.E220D	c.660G>C	Verified	TTC	GAG	TGC	3	G	C
TCGA-10-0930	PDXP	ENST00000215904	p.E220D	c.660G>C	Verified	TTC	GAG	TGC	3	G	C
TCGA-13-0916	PDZRN3	NM_015009.1	p.V542L	c.1624G>C	Verified	agc	gtg	ctg	1	G	C
TCGA-29-1761	PDZRN4	ENST00000539469	p.D457H	c.1369G>C	Unverified	ATA	GAT	ATG	1	G	C
TCGA-29-1761	PDZRN4_EN	ENST00000402685	p.D715H	c.2143G>C	Unverified	ATA	GAT	ATG	1	G	C
TCGA-13-0883	PELI3	ENST00000320740	p.W397S	c.1190G>C	Unverified	CTA	TGG	CTT	2	G	C
TCGA-24-1470	PENK	ENST00000314922	p.E258Q	c.772G>C	Verified	ATG	GAA	AAA	1	G	C
TCGA-30-1718	PER1	ENST00000317276	p.R1189R	c.3567G>C	Unverified	GTC	CGG	AAG	3	G	C
TCGA-25-2391	PEX11G	ENST00000221480	p.L35L	c.105G>C	Unverified	GTT	CTG	GTT	3	G	C
TCGA-20-1683	PEX16	ENST00000241041	p.G168A	c.503G>C	Unverified	GTG	GGG	AAG	2	G	C
TCGA-30-1855	PFKFB1	ENST00000375006	p.E452Q	c.1354G>C	Unverified	CCT	GAG	AAT	1	G	C
TCGA-25-2398	PFKP	ENST00000381125	p.K627N	c.1881G>C	Verified	ATG	AAG	ACC	3	G	C
TCGA-24-2280	PFN2	ENST00000239940	p.G104R	c.310G>C	Unverified	GTC	GGC	AGA	1	G	C
TCGA-23-1122	PGLYRP1	ENST00000008938	p.A153P	c.457G>C	Verified	CTG	GCC	TGC	1	G	C
TCGA-13-1510	PGM2L1	ENST00000298198	p.E607D	c.1821G>C	Unverified	ATA	GAG	AAT	3	G	C
TCGA-13-0793	PHACTR1	ENST00000332995	p.E382Q	c.1144G>C	Unverified	CAT	GAG	TCA	1	G	C
TCGA-09-2049	PHC2	ENST00000257118	p.L750F	c.2250G>C	Verified	ccc	ttg	tca	3	G	C
TCGA-30-1718	PHF20	ENST00000374012	p.Q1005H	c.3015G>C	Unverified	CAG	CAG	ATC	3	G	C
TCGA-24-0979	PHF8	ENST00000338154	p.W786C	c.2358G>C	Verified	tac	tgg	aga	3	G	C
TCGA-09-2050	PHKA1	ENST00000373542	p.R491T	c.1472G>C	Verified	AAT	AGA	ATG	2	G	C
TCGA-29-1699	PHLDB2	ENST00000412622	p.A126P	c.376G>C	Unverified	AGA	GCA	GAC	1	G	C
TCGA-29-1699	PHLDB2_EN	ENST00000393923	p.A153P	c.457G>C	Unverified	AGA	GCA	GAC	1	G	C
TCGA-29-1699	PHLDB2_EN	ENST00000431670	p.A126P	c.376G>C	Unverified	AGA	GCA	GAC	1	G	C
TCGA-24-1435	PI4K2B	ENST00000264864	p.L381F	c.1143G>C	Unverified	AAT	TTG	ATT	3	G	C

TCGA-25-2398	PIK3IP1	ENST00000215912	p.V237V	c.711G>C	Verified	GTC	GTG	GTC	3	G	C
TCGA-04-1651	PITX2	ENST00000306732	p.S314T	c.941G>C	Unverified	ctg	agt	gct	2	G	C
TCGA-04-1651	PITX2	ENST00000306732	p.S314T	c.941G>C	Unverified	ctg	agt	gct	2	G	C
TCGA-04-1651	PITX2_ENST(	ENST00000354925	p.S307T	c.920G>C	Unverified	CTG	AGT	GCT	2	G	C
TCGA-04-1651	PITX2_ENST(	ENST00000354925	p.S307T	c.920G>C	Unverified	CTG	AGT	GCT	2	G	C
TCGA-61-2008	PIWIL3	ENST00000332271	p.R290P	c.869G>C	Verified	CTC	CGA	ATA	2	G	C
TCGA-61-1998	PKD1L2	ENST00000337114	p.R641T	c.1922G>C	Unverified	CTG	AGA	GGC	2	G	C
TCGA-61-1998	PKD1L2_ENS	ENST00000525539	p.R641T	c.1922G>C	Unverified	CTG	AGA	GGC	2	G	C
TCGA-10-0930	PKHD1	ENST00000371117	p.V712L	c.2134G>C	Verified	AAC	GTA	ACA	1	G	C
TCGA-10-0930	PKHD1	ENST00000371117	p.V712L	c.2134G>C	Verified	AAC	GTA	ACA	1	G	C
TCGA-24-1849	PKHD1L1	ENST00000378402	p.A2349P	c.7045G>C	Unverified	ATT	GCA	TCT	1	G	C
TCGA-61-1910	PKHD1L1	ENST00000378402	p.V1707L	c.5119G>C	Unverified	TCA	GTG	AAT	1	G	C
TCGA-25-1326	PKHD1L1	ENST00000378402	p.R4073T	c.12218G>C	Verified	GAA	AGG	TCT	2	G	C
TCGA-20-1685	PKP4	ENST00000389759	p.V757V	c.2271G>C	Unverified	GAG	GTG	CCC	3	G	C
TCGA-23-1118	PKP4	ENST00000389759	p.K171N	c.513G>C	Verified	AGC	AAG	GCA	3	G	C
TCGA-23-1124	PLA2G4F	ENST00000382396	p.C675S	c.2024G>C	Verified	GAC	TGC	CTG	2	G	C
TCGA-13-1510	PLB1	ENST00000327757	p.L725F	c.2175G>C	Verified	GCC	TTG	CAC	3	G	C
TCGA-25-1313	PLD5	ENST00000366545	p.E336Q	c.1006G>C	Verified	AAA	GAA	CAA	1	G	C
TCGA-30-1718	PLEKHG1	ENST00000358517	p.G647R	c.1939G>C	Unverified	TTT	GGG	TCA	1	G	C
TCGA-13-0923	PLSCR1	ENST00000342435	p.D301H	c.901G>C	Verified	ATT	GAC	TTC	1	G	C
TCGA-23-1114	PLXDC2	ENST00000377252	p.V473V	c.1419G>C	Unverified	CTT	GTG	ACA	3	G	C
TCGA-09-2049	PLXNA2	ENST00000367033	p.G632R	c.1894G>C	Verified	TTT	GGG	CTG	1	G	C
TCGA-29-1761	PLXNA4_EN:	ENST00000321063	p.A589P	c.1765G>C	Unverified	TCA	GCT	GGC	1	G	C
TCGA-29-1761	PLXNA4_EN:	ENST00000321063	p.A589P	c.1765G>C	Unverified	TCA	GCT	GGC	1	G	C
TCGA-13-1498	PLXNB1	ENST00000296440	p.L1983L	c.5949G>C	Verified	CCT	CTG	AGG	3	G	C
TCGA-04-1342	PM20D1	ENST00000367136	p.G143A	c.428G>C	Unverified	TCT	GGG	TTG	2	G	C
TCGA-24-2024	PMPCA	ENST00000371717	p.K269N	c.807G>C	Unverified	CGG	AAG	TAC	3	G	C
TCGA-13-0924	PMPCB	ENST00000249269	p.S282T	c.845G>C	Verified	GGA	AGT	GAG	2	G	C
TCGA-24-1846	POC1B	ENST00000313546	p.G198A	c.593G>C	Unverified	AGT	GGT	ACA	2	G	C
TCGA-10-0930	POGLUT1	ENST00000295588	p.?	c.639-1G>C	Verified	TCA	AGG	ACA	3	G	C
TCGA-10-0930	POGLUT1	ENST00000295588	p.?	c.639-1G>C	Verified	TCA	AGG	ACA	3	G	C
TCGA-13-0919	POLI	NM_007195.1	p.R28T	c.83G>C	Unverified	tcc	aga	gtc	2	G	C
TCGA-61-2012	POLR3E	ENST00000299853	p.V239L	c.715G>C	Verified	CTC	GTC	AAG	1	G	C
TCGA-23-1124	POU4F1	ENST00000377208	p.E348Q	c.1042G>C	Verified	CCT	GAG	CTC	1	G	C

TCGA-09-1674	POU4F1	ENST00000377208	p.M344I	c.1032G>C	Unverified	AAA	ATG	AAC	3	G	C
TCGA-04-1336	PPAN-P2RY1	ENST00000393796	p.G445G	c.1335G>C	Verified	AGT	GGG	TTC	3	G	C
TCGA-04-1651	PPFIA1	ENST00000253925	p.G667A	c.2000G>C	Unverified	agt	gga	agt	2	G	C
TCGA-04-1651	PPFIA1	ENST00000253925	p.G667A	c.2000G>C	Unverified	agt	gga	agt	2	G	C
TCGA-04-1337	PPHLN1	ENST00000358314	p.R125T	c.374G>C	Unverified	AAA	AGG	GAC	2	G	C
TCGA-29-1777	PPM1F	ENST00000263212	p.A395P	c.1183G>C	Unverified	gtg	gct	gcg	1	G	C
TCGA-25-2391	PPP1R12B	NM_032105.1	p.R114T	c.341G>C	Unverified	aac	aga	gcc	2	G	C
TCGA-29-1783	PPP1R15A	ENST00000200453	p.V513L	c.1537G>C	Unverified	TAT	GTA	CCT	1	G	C
TCGA-29-1783	PPP1R15A	ENST00000200453	p.D367H	c.1099G>C	Unverified	TCA	GAT	GAG	1	G	C
TCGA-29-1783	PPP1R15A	ENST00000200453	p.V569L	c.1705G>C	Unverified	GCT	GTC	TGG	1	G	C
TCGA-29-1783	PPP1R15A	ENST00000200453	p.E489D	c.1467G>C	Unverified	AAA	GAG	ACA	3	G	C
TCGA-30-1718	PPP1R15B	ENST00000367188	p.Q610H	c.1830G>C	Unverified	GTG	CAG	CTG	3	G	C
TCGA-13-1501	PPP1R3F	ENST00000055335	p.V382L	c.1144G>C	Verified	cag	gtt	tct	1	G	C
TCGA-13-1501	PPP1R3F	ENST00000055335	p.V382L	c.1144G>C	Verified	cag	gtt	tct	1	G	C
TCGA-24-2267	PPP1R9A	XM_374491.1	p.L602F	c.1806G>C	Verified	cag	ttg	ata	3	G	C
TCGA-23-1117	PRAMEF20	ENST00000316412	p.R47T	c.140G>C	Verified	AGG	AGA	CAC	2	G	C
TCGA-23-1117	PRAMEF20	ENST00000316412	p.R47T	c.140G>C	Verified	AGG	AGA	CAC	2	G	C
TCGA-23-1032	PRAMEF4	ENST00000235349	p.D366H	c.1096G>C	Verified	ATA	GAC	TCC	1	G	C
TCGA-24-2289	PRB3	ENST00000381842	p.G97A	c.290G>C	Unverified	CCG	GGA	AAG	2	G	C
TCGA-61-1740	PRKAA2	ENST00000371244	p.L170L	c.510G>C	Unverified	TTT	CTG	AGA	3	G	C
TCGA-61-1740	PRKAA2_EN	ENST00000371244	p.L170L	c.510G>C	Unverified	TTT	CTG	AGA	3	G	C
TCGA-20-1687	PRKACA	ENST00000308677	p.?	c.644-1G>C	Unverified	aaa	ggc	tac	2	G	C
TCGA-20-1687	PRKACA_EN	ENST00000350356	p.G207R	c.619G>C	Unverified	AAA	GGC	TAC	1	G	C
TCGA-20-1685	PRKACB	ENST00000370689	p.M232I	c.696G>C	Unverified	gaa	atg	gca	3	G	C
TCGA-20-1685	PRKACB_EN	ENST00000370685	p.M279I	c.837G>C	Unverified	GAA	ATG	GCA	3	G	C
TCGA-20-1685	PRKACB_EN	ENST00000370688	p.M232I	c.696G>C	Unverified	GAA	ATG	GCA	3	G	C
TCGA-20-1685	PRKACB_EN	ENST00000394838	p.M239I	c.717G>C	Unverified	GAA	ATG	GCA	3	G	C
TCGA-25-1326	PRKD2	ENST00000291281	p.L662F	c.1986G>C	Verified	GCT	TTG	AGA	3	G	C
TCGA-29-1781	PRPF38B	ENST00000370025	p.R442T	c.1325G>C	Unverified	AGT	AGA	AAT	2	G	C
TCGA-24-1469	PRPF8	ENST00000304992	p.R998P	c.2993G>C	Verified	CTG	CGC	CTC	2	G	C
TCGA-04-1356	PRPH2	ENST00000230381	p.K154N	c.462G>C	Verified	AAG	AAG	ACC	3	G	C
TCGA-25-2042	PRR23B	ENST00000329447	p.A65A	c.195G>C	Unverified	GCC	GCG	GGC	3	G	C
TCGA-24-1563	PRSS36	ENST00000268281	p.D115H	c.343G>C	Verified	CTG	GAC	GGC	1	G	C
TCGA-61-2009	PRUNE	ENST00000271620	p.E403D	c.1209G>C	Verified	GAG	GAG	GAC	3	G	C

TCGA-13-0904	PRUNE2_EN	ENST00000376718	p.K1733N	c.5199G>C	Verified	CCT	AAG	TCT	3	G	C
TCGA-09-2049	PSENEN	ENST00000222266	p.G89G	c.267G>C	Verified	ctt	ggg	gac	3	G	C
TCGA-29-1763	PSMB4	ENST00000290541	p.Q20H	c.60G>C	Unverified	GGA	CAG	TTT	3	G	C
TCGA-04-1338	PSMC3	ENST00000298852	p.D98H	c.292G>C	Verified	CTG	GAT	GTT	1	G	C
TCGA-29-1770	PSMC3IP	ENST00000253789	p.L171L	c.513G>C	Unverified	GAG	CTG	TCT	3	G	C
TCGA-13-0900	PSTPIP1	ENST00000558012	p.K34N	c.102G>C	Verified	AGG	AAG	ATG	3	G	C
TCGA-13-0760	PTCHD3	ENST00000438700	p.G626R	c.1876G>C	Verified	GAA	GGT	TTA	1	G	C
TCGA-13-1510	PTGR1	ENST00000309195	p.V157L	c.469G>C	Unverified	TCA	GTC	GTG	1	G	C
TCGA-24-1849	PTGS1_ENST	ENST00000362012	p.E363Q	c.1087G>C	Unverified	CCA	GAG	CTG	1	G	C
TCGA-09-2050	PTH2R	ENST00000272847	p.E527Q	c.1579G>C	Verified	ATG	GAG	AAG	1	G	C
TCGA-13-1408	PTK2B	ENST00000397501	p.E438Q	c.1312G>C	Unverified	GGG	GAG	GTC	1	G	C
TCGA-29-1693	PTK2B	ENST00000397501	p.E88Q	c.262G>C	Unverified	GCT	GAG	TGC	1	G	C
TCGA-13-1408	PTK2B_ENST	ENST00000397497	p.E184Q	c.550G>C	Unverified	GGG	GAG	GTC	1	G	C
TCGA-29-1693	PTK2B_ENST	ENST00000544172	p.E88Q	c.262G>C	Unverified	GCT	GAG	TGC	1	G	C
TCGA-04-1530	PTPDC1	ENST00000288976	p.V272L	c.814G>C	Unverified	ttt	gtg	cgg	1	G	C
TCGA-24-1845	PTPRB	NM_002837.2	p.R312T	c.935G>C	Unverified	caa	aga	cct	2	G	C
TCGA-24-1845	PTPRB_ENST	ENST00000261266	p.R312T	c.935G>C	Unverified	CAA	AGA	CCT	2	G	C
TCGA-24-1845	PTPRB_ENST	ENST00000334414	p.R530T	c.1589G>C	Unverified	CAA	AGA	CCT	2	G	C
TCGA-23-1022	PTPRN	ENST00000295718	p.V577V	c.1731G>C	Verified	tca	gtg	ctg	3	G	C
TCGA-04-1347	PTPRN2	NM_002847.2	p.A251P	c.751G>C	Verified	gct	gcc	cag	1	G	C
TCGA-13-0760	PTTG1IP	ENST00000330938	p.G106R	c.316G>C	Verified	GTC	GGG	GGA	1	G	C
TCGA-29-1761	PUM2	ENST00000338086	p.G391A	c.1172G>C	Unverified	GCA	GGT	CAG	2	G	C
TCGA-13-0760	PUS1	ENST00000376649	p.W15C	c.45G>C	Unverified	CGG	TGG	ACC	3	G	C
TCGA-61-1740	PUS3	ENST00000227474	p.Q126H	c.378G>C	Unverified	GGA	CAG	GTG	3	G	C
TCGA-24-2289	PXN	ENST00000424649	p.E241D	c.723G>C	Unverified	GGC	GAG	ATG	3	G	C
TCGA-29-1705	Q69YJ1_HUI	ENST00000290422	p.E31D	c.93G>C	Unverified	TGC	GAG	CTC	3	G	C
TCGA-29-1761	Q8N8K0_HU	ENST00000301807	p.R1700P	c.5099G>C	Unverified	TTC	CGG	TTC	2	G	C
TCGA-25-1326	Q96K91_HU	ENST00000288841	p.G419A	c.1256G>C	Verified	GCT	GGA	GCC	2	G	C
TCGA-13-0905	QSER1	ENST00000399302	p.G1180R	c.3538G>C	Verified	CCA	GGG	ACC	1	G	C
TCGA-25-2400	RABGAP1L	ENST00000251507	p.V813L	c.2437G>C	Verified	TTT	GTA	TAT	1	G	C
TCGA-13-1507	RAD18	ENST00000264926	p.R489T	c.1466G>C	Verified	CCA	AGA	AAC	2	G	C
TCGA-20-1687	RAD21	ENST00000297338	p.E34Q	c.100G>C	Unverified	ttc	gag	tgt	1	G	C
TCGA-04-1331	RAD50	NM_133482.1	p.E292D	c.876G>C	Unverified	gat	gag	ata	3	G	C
TCGA-24-1846	RALGAPA2	ENST00000202677	p.D941H	c.2821G>C	Unverified	GGA	GAT	GTG	1	G	C

TCGA-24-1846	RALGAPA2_	ENST00000202677	p.D941H	c.2821G>C	Unverified	GGA	GAT	GTG	1	G	C
TCGA-61-1910	RALGDS	ENST00000372050	p.L585L	c.1755G>C	Unverified	tat	ctg	tat	3	G	C
TCGA-29-1691	RANBP2	ENST00000283195	p.A655P	c.1963G>C	Unverified	TTT	GCT	ATA	1	G	C
TCGA-29-1691	RANBP2_EN	ENST00000283195	p.A655P	c.1963G>C	Unverified	TTT	GCT	ATA	1	G	C
TCGA-29-1769	RAP2A	ENST00000245304	p.A59P	c.175G>C	Unverified	acg	gcg	ggc	1	G	C
TCGA-24-1844	RAPGEFL1	ENST00000264644	p.R325P	c.974G>C	Unverified	ctt	cga	ctc	2	G	C
TCGA-13-1481	RARA	ENST00000254066	p.K380N	c.1140G>C	Verified	atg	aag	att	3	G	C
TCGA-29-1761	RARG	ENST00000425354	p.A313P	c.937G>C	Unverified	ttt	gcc	ttt	1	G	C
TCGA-24-1104	RASAL2	ENST00000263528	p.E526D	c.1578G>C	Verified	GGG	GAG	TTT	3	G	C
TCGA-23-1117	RASGRF1	ENST00000419573	p.E254Q	c.760G>C	Verified	gct	gag	tac	1	G	C
TCGA-23-1117	RASGRF1	ENST00000419573	p.E254Q	c.760G>C	Verified	gct	gag	tac	1	G	C
TCGA-29-1711	RASGRP3_EI	ENST00000403687	p.E622D	c.1866G>C	Unverified	TCA	GAG	GCT	3	G	C
TCGA-29-1711	RASGRP3_EI	ENST00000403687	p.E622D	c.1866G>C	Unverified	TCA	GAG	GCT	3	G	C
TCGA-04-1638	RB1	ENST00000267163	p.A562P	c.1684G>C	Unverified	CTT	GCA	TGG	1	G	C
TCGA-24-1469	RB1	ENST00000267163	p.R763T	c.2288G>C	Unverified	CAG	AGA	CTG	2	G	C
TCGA-04-1638	RB1_ENST0(	ENST00000267163	p.A562P	c.1684G>C	Unverified	CTT	GCA	TGG	1	G	C
TCGA-24-1469	RB1_ENST0(	ENST00000267163	p.R763T	c.2288G>C	Unverified	CAG	AGA	CTG	2	G	C
TCGA-10-0930	RBL1	ENST00000373664	p.K794N	c.2382G>C	Verified	aga	aag	gtc	3	G	C
TCGA-10-0930	RBL1	ENST00000373664	p.K794N	c.2382G>C	Verified	aga	aag	gtc	3	G	C
TCGA-61-2008	RBM15	ENST00000369784	p.G563R	c.1687G>C	Verified	AGG	GGT	GCT	1	G	C
TCGA-61-1740	RBM28	ENST00000223073	p.Q390H	c.1170G>C	Unverified	GCT	CAG	AAA	3	G	C
TCGA-29-1764	RBM39	ENST00000253363	p.G383A	c.1148G>C	Unverified	AGT	GGC	TCT	2	G	C
TCGA-20-0991	RBM44	ENST00000316997	p.G13R	c.37G>C	Verified	TCT	GGT	AAA	1	G	C
TCGA-23-1114	RBM45	ENST00000286070	p.G10R	c.28G>C	Unverified	AGC	GGC	GGG	1	G	C
TCGA-04-1356	RBP5	ENST00000266560	p.G68R	c.202G>C	Verified	GTG	GGA	GTG	1	G	C
TCGA-23-1124	RBPMS	ENST00000287771	p.R24R	c.72G>C	Verified	GTC	CGG	ACC	3	G	C
TCGA-61-2113	RCOR1	ENST00000570597	p.D147H	c.439G>C	Verified	TTG	GAT	GAA	1	G	C
TCGA-09-1674	RDH14	ENST00000381249	p.K152N	c.456G>C	Unverified	ATG	AAG	ACT	3	G	C
TCGA-24-2289	RECQL	ENST00000444129	p.S568S	c.1704G>C	Unverified	ATT	TCG	TAT	3	G	C
TCGA-24-1417	REL	ENST00000295025	p.E38Q	c.112G>C	Verified	ggg	gag	cac	1	G	C
TCGA-13-0760	RELA	ENST00000406246	p.E127Q	c.379G>C	Verified	CTG	GAG	CAG	1	G	C
TCGA-04-1338	REV1	ENST00000258428	p.A1035A	c.3105G>C	Verified	GCA	GCG	TAT	3	G	C
TCGA-23-1031	RFX2	ENST00000303657	p.V549L	c.1645G>C	Verified	AAC	GTG	CAG	1	G	C
TCGA-09-1674	RFX3	ENST00000358730	p.R498T	c.1493G>C	Unverified	CGA	AGA	TAC	2	G	C

TCGA-09-1674	RFX3_ENST(	ENST00000382004	p.R498T	c.1493G>C	Unverified	CGA	AGA	TAC	2	G	C
TCGA-23-2078	RG9MTD2	ENST00000394876	p.E257Q	c.769G>C	Verified	CTG	GAA	TAC	1	G	C
TCGA-13-0885	RGL1	ENST00000304685	p.S585T	c.1754G>C	Verified	GAA	AGC	ATG	2	G	C
TCGA-29-1770	RGS20	ENST00000297313	p.M369I	c.1107G>C	Unverified	ttc	atg	aac	3	G	C
TCGA-29-1770	RGS20_ENS	ENST00000276500	p.M222I	c.666G>C	Unverified	TTC	ATG	AAC	3	G	C
TCGA-10-0930	RHOT1	ENST00000358365	p.D106H	c.316G>C	Verified	ACA	GAC	AAA	1	G	C
TCGA-10-0930	RHOT1	ENST00000358365	p.D106H	c.316G>C	Verified	ACA	GAC	AAA	1	G	C
TCGA-23-1031	RHOT1	ENST00000358365	p.V418L	c.1252G>C	Verified	AAT	GTG	TTC	1	G	C
TCGA-61-1998	RIC3	ENST00000343202	p.A319P	c.955G>C	Verified	AAT	GCT	GGA	1	G	C
TCGA-61-2008	RIF1	ENST00000444746	p.D492H	c.1474G>C	Verified	aaa	gat	gcc	1	G	C
TCGA-23-1122	RIMS4	ENST00000372851	p.E222Q	c.664G>C	Verified	GAG	GAG	CTG	1	G	C
TCGA-23-1022	RIMS4	ENST00000372851	p.E222D	c.666G>C	Verified	GAG	GAG	CTG	3	G	C
TCGA-29-1693	RINT1	ENST00000257700	p.D31H	c.91G>C	Unverified	AGT	GAC	ATA	1	G	C
TCGA-13-1510	RIOK3	ENST00000339486	p.R178T	c.533G>C	Verified	GCA	AGA	ATG	2	G	C
TCGA-13-0904	RLTPR_ENST	ENST00000334583	p.G376R	c.1126G>C	Verified	GCA	GGC	ACC	1	G	C
TCGA-13-0760	RMND5A	ENST00000283632	p.M182I	c.546G>C	Verified	GAA	ATG	CTT	3	G	C
TCGA-24-1845	RNF112	ENST00000299604	p.R63S	c.189G>C	Unverified	GAG	AGG	TTG	3	G	C
TCGA-13-1496	RNF13	ENST00000392894	p.A114P	c.340G>C	Verified	AGA	GCA	GGA	1	G	C
TCGA-04-1655	RNF180	ENST00000296615	p.R392T	c.1175G>C	Unverified	GAA	AGA	TGG	2	G	C
TCGA-04-1655	RNF180_EN!	ENST00000389100	p.R392T	c.1175G>C	Unverified	GAA	AGA	TGG	2	G	C
TCGA-13-0893	RNF20	ENST00000389120	p.E427D	c.1281G>C	Verified	gat	gag	gtt	3	G	C
TCGA-29-1769	ROMO1	ENST00000336695	p.G38A	c.113G>C	Unverified	TTC	GGC	ACC	2	G	C
TCGA-23-1114	ROPN1	ENST00000184183	p.D102H	c.304G>C	Unverified	ACA	GAT	CTG	1	G	C
TCGA-13-1510	RORA	ENST00000261523	p.K107N	c.321G>C	Unverified	TGC	AAG	ATC	3	G	C
TCGA-61-2095	RP1L1	ENST00000382483	p.R692P	c.2075G>C	Unverified	GAG	CGG	CGA	2	G	C
TCGA-04-1651	RPGR_ENST(	ENST00000378505	p.G1073A	c.3218G>C	Unverified	ACA	GGC	GAA	2	G	C
TCGA-04-1651	RPGR_ENST(	ENST00000378505	p.G1073A	c.3218G>C	Unverified	ACA	GGC	GAA	2	G	C
TCGA-29-1702	RPGRIP1_EN	ENST00000400017	p.E629Q	c.1885G>C	Unverified	TTT	GAA	CTG	1	G	C
TCGA-24-1463	RPGRIP1L	ENST00000379925	p.D457H	c.1369G>C	Unverified	GCT	GAT	GAA	1	G	C
TCGA-24-1435	RPL23A	ENST00000422514	p.E133D	c.399G>C	Verified	GGA	GAG	AAG	3	G	C
TCGA-29-1785	RPLP1	ENST00000260379	p.E103D	c.309G>C	Unverified	GAG	GAG	TCT	3	G	C
TCGA-29-1785	RPLP1	ENST00000260379	p.E103D	c.309G>C	Unverified	GAG	GAG	TCT	3	G	C
TCGA-61-2095	RREB1	ENST00000379933	p.R51R	c.153G>C	Unverified	AAT	CGG	ATT	3	G	C
TCGA-23-1123	RRH	ENST00000317735	p.V319L	c.955G>C	Verified	CCT	GTG	ACA	1	G	C

TCGA-23-1123	RRH	ENST00000317735	p.V319L	c.955G>C	Verified	CCT	GTG	ACA	1	G	C
TCGA-04-1362	RTF1	ENST00000260373	p.D538H	c.1612G>C	Verified	CAC	GAT	TTT	1	G	C
TCGA-10-0930	RUFY1	ENST00000437570	p.E261Q	c.781G>C	Verified	CGA	GAA	AGA	1	G	C
TCGA-10-0930	RUFY1	ENST00000437570	p.E261Q	c.781G>C	Verified	CGA	GAA	AGA	1	G	C
TCGA-13-1498	RUFY1	ENST00000437570	p.S569T	c.1706G>C	Verified	TCC	AGC	AAC	2	G	C
TCGA-24-1422	RUSC2	ENST00000361226	p.C1328S	c.3983G>C	Unverified	GCC	TGC	CCT	2	G	C
TCGA-24-2262	RWDD3	ENST00000370202	p.V34L	c.100G>C	Verified	ACC	GTG	TTC	1	G	C
TCGA-04-1655	RYR2	ENST00000366574	p.E712Q	c.2134G>C	Unverified	GAA	GAG	TGG	1	G	C
TCGA-13-0923	RYR2	ENST00000366574	p.E2338Q	c.7012G>C	Verified	GGA	GAA	GGT	1	G	C
TCGA-29-1761	RYR2	ENST00000366574	p.D2885H	c.8653G>C	Unverified	AAG	GAT	AGA	1	G	C
TCGA-13-0885	RYR2	ENST00000366574	p.E3487D	c.10461G>C	Verified	CAG	GAG	CTC	3	G	C
TCGA-29-1769	RYR2	ENST00000366574	p.L4462F	c.13386G>C	Unverified	AAG	TTG	AGG	3	G	C
TCGA-30-1891	RYR2	ENST00000366574	p.K3471N	c.10413G>C	Unverified	CTG	AAG	CGG	3	G	C
TCGA-04-1342	S1PR3	ENST00000375846	p.D333H	c.997G>C	Unverified	CTC	GAC	CCA	1	G	C
TCGA-13-1510	SACS	NM_014363.3	p.E871D	c.2613G>C	Verified	ctt	gag	aat	3	G	C
TCGA-61-2109	SAMD9	ENST00000379958	p.R759T	c.2276G>C	Unverified	TTC	AGA	TGT	2	G	C
TCGA-24-1850	SAMD9L	ENST00000318238	p.W481S	c.1442G>C	Unverified	ATG	TGG	GAG	2	G	C
TCGA-29-1698	SAP130	ENST00000259235	p.K1045N	c.3135G>C	Unverified	CGA	AAG	GAA	3	G	C
TCGA-29-1698	SAP130_ENS	ENST00000357702	p.K1080N	c.3240G>C	Unverified	CGA	AAG	GAA	3	G	C
TCGA-61-1740	SARDH	ENST00000371872	p.K558N	c.1674G>C	Unverified	ATC	AAG	AAG	3	G	C
TCGA-24-2267	SBF1	ENST00000390679	p.R562P	c.1685G>C	Unverified	CGG	CGG	CTG	2	G	C
TCGA-13-0795	SCAMP2	ENST00000268099	p.G243A	c.728G>C	Verified	CTG	GGG	GAC	2	G	C
TCGA-13-0905	SCAMP3	ENST00000302631	p.R5T	c.14G>C	Verified	AGC	AGA	GAC	2	G	C
TCGA-13-1512	SCARA3	ENST00000301904	p.G523A	c.1568G>C	Verified	AAA	GGC	TCA	2	G	C
TCGA-23-1117	SCEL	ENST00000349847	p.D236H	c.706G>C	Verified	CTT	GAT	AAC	1	G	C
TCGA-23-1117	SCEL	ENST00000349847	p.D236H	c.706G>C	Verified	CTT	GAT	AAC	1	G	C
TCGA-13-1498	SCG3	ENST00000220478	p.V12V	c.36G>C	Verified	CTG	GTG	TTA	3	G	C
TCGA-29-1691	SCN3A	ENST00000283254	p.D1066H	c.3196G>C	Unverified	AGA	GAT	GGG	1	G	C
TCGA-29-1691	SCN3A_ENST	ENST00000409101	p.D1017H	c.3049G>C	Unverified	AGA	GAT	GGG	1	G	C
TCGA-09-1674	SCRN1	ENST00000242059	p.D231H	c.691G>C	Unverified	GAG	GAT	CAT	1	G	C
TCGA-09-1674	SCRN1_ENST	ENST00000434476	p.D251H	c.751G>C	Unverified	GAG	GAT	CAT	1	G	C
TCGA-24-1422	SCRN3	ENST00000272732	p.G252R	c.754G>C	Verified	AAA	GGA	AAT	1	G	C
TCGA-04-1338	SCUBE3	ENST00000274938	p.D279H	c.835G>C	Unverified	ATA	GAT	GAG	1	G	C
TCGA-13-1497	SDC1	ENST00000254351	p.V25L	c.73G>C	Verified	ATT	GTG	GCT	1	G	C

TCGA-23-1124	SDC1	ENST00000254351	p.G36A	c.107G>C	Verified	GAT	GGC	TCT	2	G	C
TCGA-13-0903	SDCBP2_EN!	ENST00000381812	p.D10H	c.28G>C	Verified	GAG	GAC	CTA	1	G	C
TCGA-04-1651	SEC31A_EN!	ENST00000395310	p.L1153L	c.3459G>C	Unverified	TTT	CTG	TAT	3	G	C
TCGA-04-1651	SEC31A_EN!	ENST00000395310	p.L1153L	c.3459G>C	Unverified	TTT	CTG	TAT	3	G	C
TCGA-04-1651	SEC31A_EN!	ENST00000432794	p.L1166L	c.3498G>C	Unverified	TTT	CTG	TAT	3	G	C
TCGA-04-1651	SEC31A_EN!	ENST00000432794	p.L1166L	c.3498G>C	Unverified	TTT	CTG	TAT	3	G	C
TCGA-24-1417	SEC62	ENST00000337002	p.V24L	c.70G>C	Verified	GCT	GTG	GCC	1	G	C
TCGA-04-1367	SECISBP2	ENST00000375807	p.Q110H	c.330G>C	Verified	TCC	CAG	TAT	3	G	C
TCGA-20-1683	SELENBP1	ENST00000368868	p.G161A	c.482G>C	Unverified	AAA	GGG	GGT	2	G	C
TCGA-23-1122	SEMA3D	ENST00000284136	p.R634P	c.1901G>C	Verified	CAT	CGA	GAG	2	G	C
TCGA-13-0923	SEMA3E	ENST00000307792	p.V72L	c.214G>C	Verified	TTC	GTG	GGA	1	G	C
TCGA-13-0714	SEMA4F	ENST00000357877	p.A230P	c.688G>C	Verified	GCA	GCC	GTG	1	G	C
TCGA-24-1847	SEMG2	ENST00000372769	p.V223L	c.667G>C	Unverified	AAT	GTG	GTT	1	G	C
TCGA-24-1474	SERINC1	ENST00000368454	p.V79L	c.235G>C	Verified	GTT	GTC	CCT	1	G	C
TCGA-13-0792	SERPINA4	ENST00000557004	p.K193N	c.579G>C	Verified	GGG	AAG	ATT	3	G	C
TCGA-13-0905	SERPINA5	ENST00000553780	p.D51H	c.151G>C	Verified	TTT	GAC	CTC	1	G	C
TCGA-29-1764	SERPINB13	ENST00000344731	p.E199D	c.597G>C	Unverified	gaa	gag	aaa	3	G	C
TCGA-29-1785	SERPINB2	ENST00000299502	p.A135A	c.405G>C	Unverified	tct	gcg	agc	3	G	C
TCGA-29-1785	SERPINB2	ENST00000299502	p.A135A	c.405G>C	Unverified	tct	gcg	agc	3	G	C
TCGA-30-1856	SERPINB8	ENST00000397985	p.G151A	c.452G>C	Unverified	gct	ggg	aca	2	G	C
TCGA-24-1845	SERPIND1	ENST00000215727	p.V177L	c.529G>C	Unverified	CAA	GTG	CAC	1	G	C
TCGA-24-1845	SERPIND1_E	ENST00000215727	p.V177L	c.529G>C	Unverified	CAA	GTG	CAC	1	G	C
TCGA-25-1318	SESN1	ENST00000436639	p.G104R	c.310G>C	Verified	cta	gga	cag	1	G	C
TCGA-25-1318	SESN1	ENST00000436639	p.G104R	c.310G>C	Verified	cta	gga	cag	1	G	C
TCGA-61-1907	SETD1A	ENST00000262519	p.T213T	c.639G>C	Unverified	GAG	ACG	GCC	3	G	C
TCGA-09-1674	SETD2	ENST00000330022	p.V1694L	c.5080G>C	Unverified	CCA	GTG	TGT	1	G	C
TCGA-09-1674	SETD2_ENST	ENST00000409792	p.V2197L	c.6589G>C	Unverified	CCA	GTG	TGT	1	G	C
TCGA-23-1029	SETDB1	ENST00000271640	p.L205L	c.615G>C	Unverified	ATT	CTG	GGC	3	G	C
TCGA-23-1029	SETDB1_EN!	ENST00000271640	p.L205L	c.615G>C	Unverified	ATT	CTG	GGC	3	G	C
TCGA-24-2288	SFMBT2	ENST00000361972	p.Q71H	c.213G>C	Unverified	ATT	CAG	AGC	3	G	C
TCGA-13-1512	SFRP2	ENST00000274063	p.S272S	c.816G>C	Verified	ACC	TCG	GTG	3	G	C
TCGA-09-2050	SGCA	ENST00000262018	p.E190D	c.570G>C	Verified	ATT	GAG	GGC	3	G	C
TCGA-13-0887	SGSM3	ENST00000248929	p.E672Q	c.2014G>C	Verified	CTG	GAG	GTG	1	G	C
TCGA-13-0900	SGTA	ENST00000221566	p.E187D	c.561G>C	Verified	CTG	GAG	CTG	3	G	C

TCGA-29-1696	SH2B1	ENST00000337120	p.S430T	c.1289G>C	Unverified	GAG	AGC	AAT	2	G	C
TCGA-29-1696	SH2B1	ENST00000337120	p.S430T	c.1289G>C	Unverified	GAG	AGC	AAT	2	G	C
TCGA-29-1696	SH2B1_ENST	ENST00000322610	p.S430T	c.1289G>C	Unverified	GAG	AGC	AAT	2	G	C
TCGA-29-1696	SH2B1_ENST	ENST00000322610	p.S430T	c.1289G>C	Unverified	GAG	AGC	AAT	2	G	C
TCGA-29-1696	SH2B1_ENST	ENST00000359285	p.S430T	c.1289G>C	Unverified	GAG	AGC	AAT	2	G	C
TCGA-29-1696	SH2B1_ENST	ENST00000359285	p.S430T	c.1289G>C	Unverified	GAG	AGC	AAT	2	G	C
TCGA-61-1910	SH3GLB1	ENST00000212369	p.V79L	c.235G>C	Unverified	TTT	GTT	TAT	1	G	C
TCGA-61-1910	SH3GLB1_EI	ENST00000482504	p.V79L	c.235G>C	Unverified	TTT	GTT	TAT	1	G	C
TCGA-25-1313	SH3PXD2B	ENST00000311601	p.R671T	c.2012G>C	Verified	CTC	AGG	CCT	2	G	C
TCGA-13-0886	SHC2	XM_375550.2	p.E771D	c.2313G>C	Unverified	cac	gag	gag	3	G	C
TCGA-23-1122	SHCBP1	ENST00000303383	p.G653G	c.1959G>C	Verified	GTT	GGG	ATT	3	G	C
TCGA-10-0930	SHKBP1	ENST00000291842	p.G344A	c.1031G>C	Verified	AAC	GGC	TCC	2	G	C
TCGA-10-0930	SHKBP1	ENST00000291842	p.G344A	c.1031G>C	Verified	AAC	GGC	TCC	2	G	C
TCGA-13-1497	SHROOM2	ENST00000380913	p.S983T	c.2948G>C	Verified	ccg	agt	cag	2	G	C
TCGA-29-1781	SHROOM3	ENST00000296043	p.R718P	c.2153G>C	Unverified	GAC	CGG	CAG	2	G	C
TCGA-29-1769	SIDT1	ENST00000264852	p.M578I	c.1734G>C	Unverified	TTC	ATG	TAC	3	G	C
TCGA-24-1469	SIM1	ENST00000262901	p.E579D	c.1737G>C	Verified	GAA	GAG	AAC	3	G	C
TCGA-29-1696	SIN3B	ENST00000379803	p.D769H	c.2305G>C	Unverified	CTG	GAC	GAT	1	G	C
TCGA-29-1696	SIN3B	ENST00000379803	p.D769H	c.2305G>C	Unverified	CTG	GAC	GAT	1	G	C
TCGA-24-1849	SIX4	ENST00000216513	p.E232D	c.696G>C	Unverified	AAG	GAG	AAG	3	G	C
TCGA-25-2401	SKIV2L2	ENST00000230640	p.V729L	c.2185G>C	Verified	GTT	GTC	CCA	1	G	C
TCGA-61-1915	SLC22A2	ENST00000366953	p.G328A	c.983G>C	Unverified	ACT	GGC	AAG	2	G	C
TCGA-24-1846	SLC22A23	ENST00000380302	p.S157S	c.471G>C	Unverified	AAC	TCG	CTG	3	G	C
TCGA-24-1846	SLC22A23_E	ENST00000406686	p.S438S	c.1314G>C	Unverified	AAC	TCG	CTG	3	G	C
TCGA-23-1021	SLC22A8	ENST00000336232	p.G166A	c.497G>C	Verified	TCC	GGT	GCA	2	G	C
TCGA-13-0903	SLC23A2	ENST00000338244	p.S78T	c.233G>C	Verified	GAT	AGC	ACT	2	G	C
TCGA-25-1326	SLC25A44	ENST00000359511	p.Q264H	c.792G>C	Verified	AGA	CAG	CTG	3	G	C
TCGA-13-0916	SLC26A3	ENST00000340010	p.E334Q	c.1000G>C	Verified	GTG	GAG	ACT	1	G	C
TCGA-13-0916	SLC26A8	ENST00000355574	p.R63P	c.188G>C	Verified	TGC	CGC	TGC	2	G	C
TCGA-25-2393	SLC2A12	ENST00000275230	p.K327N	c.981G>C	Verified	GTC	AAG	GTC	3	G	C
TCGA-24-2267	SLC2A4	ENST00000317370	p.G207R	c.619G>C	Unverified	CTG	GGC	CTC	1	G	C
TCGA-20-1685	SLC2A9	ENST00000309065	p.E162D	c.486G>C	Unverified	AGT	GAG	ATC	3	G	C
TCGA-20-1685	SLC2A9_ENS	ENST00000264784	p.E191D	c.573G>C	Unverified	AGT	GAG	ATC	3	G	C
TCGA-13-1497	SLC30A8	ENST00000456015	p.V330L	c.988G>C	Verified	GTG	GTT	CGG	1	G	C

TCGA-13-0884	SLC34A2	ENST00000382051	p.G257A	c.770G>C	Verified	AAT	GGA	GAA	2	G	C
TCGA-23-1118	SLC35D3	ENST00000331858	p.G327A	c.980G>C	Verified	AGT	GGA	GAC	2	G	C
TCGA-04-1649	SLC36A2	ENST00000335244	p.G280R	c.838G>C	Unverified	ATT	GGT	GTG	1	G	C
TCGA-04-1338	SLC36A3	ENST00000335230	p.E422Q	c.1264G>C	Verified	TCT	GAG	GAC	1	G	C
TCGA-25-2042	SLC40A1	ENST00000261024	p.Q194H	c.582G>C	Verified	GGC	CAG	ATT	3	G	C
TCGA-29-1775	SLC44A5	ENST00000370855	p.D537H	c.1609G>C	Unverified	TTG	GAC	CAC	1	G	C
TCGA-04-1338	SLC44A5	ENST00000370855	p.?	c.1266+1G>C	Verified	CCA	GAG	ATT	3	G	C
TCGA-29-1775	SLC44A5_EN	ENST00000370859	p.D537H	c.1609G>C	Unverified	TTG	GAC	CAC	1	G	C
TCGA-61-2012	SLC4A7	ENST00000295736	p.G811A	c.2432G>C	Verified	TTG	GGG	TCA	2	G	C
TCGA-13-0923	SLC5A6	ENST00000310574	p.R394P	c.1181G>C	Verified	GCC	CGG	GCC	2	G	C
TCGA-24-1435	SLC6A6	ENST00000360861	p.E569Q	c.1705G>C	Verified	ACT	GAG	GGG	1	G	C
TCGA-29-1777	SLC7A13	ENST00000297524	p.S172T	c.515G>C	Unverified	CTT	AGC	TTC	2	G	C
TCGA-09-2050	SLC7A4	ENST00000382932	p.A373A	c.1119G>C	Verified	CTG	GCG	TTC	3	G	C
TCGA-30-1718	SLC8A3	ENST00000381269	p.R483T	c.1448G>C	Unverified	GTA	AGG	TTG	2	G	C
TCGA-30-1718	SLC8A3_ENS	ENST00000357887	p.R483T	c.1448G>C	Unverified	GTA	AGG	TTG	2	G	C
TCGA-23-1022	SLC9A11	ENST00000367714	p.M563I	c.1689G>C	Verified	TAT	ATG	AGA	3	G	C
TCGA-04-1342	SLCO4A1	ENST00000370507	p.R448S	c.1344G>C	Unverified	CTC	AGG	CTC	3	G	C
TCGA-24-1846	SLIT2	ENST00000504154	p.Q1042H	c.3126G>C	Unverified	GCC	CAG	GAC	3	G	C
TCGA-24-1843	SLITRK5	ENST00000325089	p.P943P	c.2829G>C	Unverified	GAG	CCG	GAC	3	G	C
TCGA-30-1718	SLX4	ENST00000294008	p.Q1216H	c.3648G>C	Unverified	CAG	CAG	GAG	3	G	C
TCGA-13-0714	SMAD1	ENST00000302085	p.W420C	c.1260G>C	Verified	ggc	tgg	gga	3	G	C
TCGA-23-1114	SMARCA1	NM_003069.2	p.W860C	c.2580G>C	Unverified	aac	tgg	act	3	G	C
TCGA-29-1761	SMC1A	ENST00000322213	p.Q702H	c.2106G>C	Unverified	gtg	cag	tct	3	G	C
TCGA-13-0793	SMC1B	NM_148674.1	p.E283Q	c.847G>C	Unverified	aaa	gaa	tta	1	G	C
TCGA-29-1703	SMG1	SU_SMG1	p.R888T	c.2663G>C	Unverified	cag	aga	ctg	2	G	C
TCGA-29-1703	SMG1_ENST	ENST00000446231	p.R892T	c.2675G>C	Unverified	CAG	AGA	CTG	2	G	C
TCGA-24-2262	SMR3A	ENST00000226460	p.A15A	c.45G>C	Unverified	GCA	GCG	TGT	3	G	C
TCGA-29-1761	SMR3B	ENST00000304915	p.E21D	c.63G>C	Unverified	GGT	GAG	AGT	3	G	C
TCGA-13-1510	SNAI1	ENST00000244050	p.E48D	c.144G>C	Unverified	ccg	gag	atc	3	G	C
TCGA-13-0793	SND1	ENST00000354725	p.T794T	c.2382G>C	Verified	GCC	ACG	GAG	3	G	C
TCGA-13-0919	SNRNP200	ENST00000323853	p.G1353R	c.4057G>C	Unverified	ACG	GGC	AGC	1	G	C
TCGA-13-0761	SNX16	ENST00000396330	p.A197P	c.589G>C	Verified	GTA	GCT	CAC	1	G	C
TCGA-13-0761	SNX16	ENST00000396330	p.A197P	c.589G>C	Verified	GTA	GCT	CAC	1	G	C
TCGA-24-1423	SOAT2	ENST00000301466	p.D154H	c.460G>C	Verified	TTT	GAC	CTA	1	G	C

TCGA-29-1705	SOGA1	ENST00000237536	p.G966A	c.2897G>C	Unverified	ACG	GGG	GAC	2	G	C
TCGA-29-1705	SOGA1_ENS	ENST00000237536_v68	p.G966A	c.2897G>C	Unverified	ACG	GGG	GAC	2	G	C
TCGA-24-1469	SOS2	ENST00000216373	p.E647Q	c.1939G>C	Verified	TTT	GAA	ATT	1	G	C
TCGA-61-2095	SOX7	ENST00000304501	p.G151R	c.451G>C	Unverified	AGC	GGC	AGC	1	G	C
TCGA-61-1725	SP100	ENST00000264052	p.E206D	c.618G>C	Unverified	TCA	GAG	CAC	3	G	C
TCGA-61-2102	SP100_ENST	ENST00000340126	p.V786L	c.2356G>C	Verified	AAG	GTC	TAC	1	G	C
TCGA-61-1725	SP100_ENST	ENST00000340126	p.E206D	c.618G>C	Unverified	TCA	GAG	CAC	3	G	C
TCGA-25-2401	SP140_ENST	ENST00000392045	p.P739P	c.2217G>C	Verified	TCT	CCG	GGA	3	G	C
TCGA-29-1784	SPATA7	ENST00000393545	p.V538V	c.1614G>C	Unverified	TCT	GTG	AAT	3	G	C
TCGA-24-1423	SPDYE1	ENST00000258704	p.G234G	c.702G>C	Verified	TAT	GGG	AAG	3	G	C
TCGA-61-1998	SPECC1L	ENST00000314328	p.E563Q	c.1687G>C	Verified	TTA	GAG	GAA	1	G	C
TCGA-61-1737	SPEG	SU_SPEG	p.G2851R	c.8551G>C	Unverified	agg	ggc	ctg	1	G	C
TCGA-09-2051	SPEN	ENST00000375759	p.G2788R	c.8362G>C	Unverified	cca	ggg	tcc	1	G	C
TCGA-24-1469	SPEN	ENST00000375759	p.R251T	c.752G>C	Verified	tct	aga	aat	2	G	C
TCGA-61-1904	SPEN	ENST00000375759	p.G2813G	c.8439G>C	Unverified	gaa	ggg	gtt	3	G	C
TCGA-24-2288	SPHKAP	XM_051221.5	p.E1671Q	c.5011G>C	Unverified	att	gaa	aag	1	G	C
TCGA-13-0760	SPINK5	ENST00000256084	p.V1013L	c.3037G>C	Verified	CCT	GTC	TGT	1	G	C
TCGA-04-1347	SPINT1	ENST00000344051	p.Q221H	c.663G>C	Verified	TTC	CAG	CTG	3	G	C
TCGA-13-0923	SPOCD1	ENST00000360482	p.D443H	c.1327G>C	Verified	TCA	GAC	AAC	1	G	C
TCGA-04-1356	SPTA1	ENST00000368148	p.V164L	c.490G>C	Verified	TAT	GTA	CAG	1	G	C
TCGA-20-1683	SPTA1	ENST00000368148	p.W246S	c.737G>C	Unverified	GCC	TGG	GAG	2	G	C
TCGA-29-1763	SPTA1	ENST00000368148	p.R1608P	c.4823G>C	Unverified	AGT	CGT	CAA	2	G	C
TCGA-29-1703	SPTA1	ENST00000368148	p.W1097C	c.3291G>C	Unverified	GAA	TGG	ATT	3	G	C
TCGA-20-0991	SPTBN5	ENST00000320955	p.A331P	c.991G>C	Unverified	GAG	GCG	CGG	1	G	C
TCGA-24-2035	SRPK2	ENST00000357311	p.Q315H	c.945G>C	Verified	gac	cag	gat	3	G	C
TCGA-61-2109	SRSF11	ENST00000370950	p.R343T	c.1028G>C	Unverified	GAG	AGA	CGA	2	G	C
TCGA-29-1777	SRSF11_ENS	ENST00000370950	p.R363T	c.1088G>C	Unverified	AAA	AGA	AAA	2	G	C
TCGA-24-2024	ST8SIA1	ENST00000396037	p.K22N	c.66G>C	Verified	TGG	AAG	TTC	3	G	C
TCGA-24-1469	STAG1	ENST00000383202	p.V1075L	c.3223G>C	Verified	TCA	GTA	AGG	1	G	C
TCGA-13-0884	STAT1	ENST00000361099	p.V734L	c.2200G>C	Verified	gag	gtg	tct	1	G	C
TCGA-13-1498	STAU1	ENST00000371856	p.E310Q	c.928G>C	Verified	aca	gag	cga	1	G	C
TCGA-29-1763	STK36	ENST00000295709	p.E894D	c.2682G>C	Unverified	GAG	GAG	GCA	3	G	C
TCGA-10-0930	STK38L	ENST00000389032	p.V104L	c.310G>C	Verified	GAG	GTG	CGG	1	G	C
TCGA-10-0930	STK38L	ENST00000389032	p.V104L	c.310G>C	Verified	GAG	GTG	CGG	1	G	C

TCGA-61-1998	STOML1	ENST00000316900	p.D358H	c.1072G>C	Unverified	GCA	GAC	CTG	1	G	C
TCGA-61-1907	STT3B	ENST00000295770	p.D782H	c.2344G>C	Unverified	TTA	GAT	CAC	1	G	C
TCGA-13-0887	STXBP3	ENST00000370008	p.L252L	c.756G>C	Verified	GAA	CTG	ACC	3	G	C
TCGA-61-1737	SUFU	ENST00000369902	p.R339P	c.1016G>C	Unverified	GAC	CGG	GCC	2	G	C
TCGA-04-1343	SUPT5H	ENST00000359191	p.E362Q	c.1084G>C	Verified	TTT	GAG	GGG	1	G	C
TCGA-61-1740	SUPT6H	ENST00000314616	p.C527S	c.1580G>C	Unverified	ATC	TGC	CAG	2	G	C
TCGA-29-1784	SUSD1	ENST00000374270	p.Q582H	c.1746G>C	Unverified	ACC	CAG	ATA	3	G	C
TCGA-24-1469	SV2B	ENST00000394232	p.L142L	c.426G>C	Unverified	TGT	CTG	TCC	3	G	C
TCGA-29-1696	SV2B	ENST00000394232	p.G601G	c.1803G>C	Unverified	TGT	GGG	ACA	3	G	C
TCGA-29-1696	SV2B	ENST00000394232	p.G601G	c.1803G>C	Unverified	TGT	GGG	ACA	3	G	C
TCGA-24-1469	SYCP2L	ENST00000283141	p.D141H	c.421G>C	Verified	GAA	GAT	TTC	1	G	C
TCGA-29-1761	SYNE1	ENST00000265368	p.E6380Q	c.19138G>C	Unverified	TTG	GAG	CAG	1	G	C
TCGA-29-1761	SYNE1_ENST	ENST00000265368	p.E6380Q	c.19138G>C	Unverified	TTG	GAG	CAG	1	G	C
TCGA-29-1761	SYNE1_ENST	ENST00000423061	p.E6309Q	c.18925G>C	Unverified	TTG	GAG	CAG	1	G	C
TCGA-25-1326	SYNE2	ENST00000358025	p.E3636Q	c.10906G>C	Verified	TTT	GAG	AAT	1	G	C
TCGA-29-1690	SYNE2	ENST00000358025	p.G6423A	c.19268G>C	Unverified	ACA	GGC	GAC	2	G	C
TCGA-25-2042	SYNM	ENST00000328642	p.E1117Q	c.3349G>C	Verified	CTG	GAG	GAT	1	G	C
TCGA-61-1998	SYP	ENST00000263233	p.Q185H	c.555G>C	Verified	CGC	CAG	ACA	3	G	C
TCGA-61-1998	SYP_ENST00	ENST00000263233	p.Q185H	c.555G>C	Verified	CGC	CAG	ACA	3	G	C
TCGA-61-1998	SYT7	ENST00000263846	p.R291R	c.873G>C	Verified	GCC	CGG	AAC	3	G	C
TCGA-13-1501	SYTL3	ENST00000360448	p.K61N	c.183G>C	Unverified	GAG	AAG	TGC	3	G	C
TCGA-13-1501	SYTL3	ENST00000360448	p.K61N	c.183G>C	Unverified	GAG	AAG	TGC	3	G	C
TCGA-13-0920	SYTL4	ENST00000372989	p.E367D	c.1101G>C	Verified	TAT	GAG	CAG	3	G	C
TCGA-24-1423	TACR2	ENST00000373306	p.G2A	c.5G>C	Unverified	ATG	GGG	ACC	2	G	C
TCGA-24-2024	TACR3	ENST00000304883	p.K368N	c.1104G>C	Verified	TTC	AAG	AGA	3	G	C
TCGA-20-1683	TAGAP	ENST00000367066	p.V156L	c.466G>C	Unverified	GCT	GTG	GTC	1	G	C
TCGA-25-2392	TANC1	ENST00000263635	p.S1564T	c.4691G>C	Unverified	CCA	AGT	CCC	2	G	C
TCGA-24-1563	TAS1R1	ENST00000333172	p.M166I	c.498G>C	Verified	CCC	ATG	ATT	3	G	C
TCGA-25-2042	TBC1D10C	ENST00000312390	p.G136A	c.407G>C	Unverified	ATT	GGC	AGG	2	G	C
TCGA-13-0760	TBC1D14	ENST00000340816	p.?	c.799-1G>C	Verified	AAG	GAA	TAT	1	G	C
TCGA-04-1530	TBC1D21	ENST00000300504	p.A222P	c.664G>C	Verified	TTT	GCT	GAG	1	G	C
TCGA-29-1777	TBC1D4	ENST00000377636	p.E814Q	c.2440G>C	Unverified	GAG	GAG	GAA	1	G	C
TCGA-13-0890	TBC1D5	ENST00000253692	p.S477T	c.1430G>C	Verified	GGC	AGT	GCA	2	G	C
TCGA-24-1423	TBC1D8B	ENST00000357242	p.A965P	c.2893G>C	Verified	caa	gca	tat	1	G	C

TCGA-24-1422	TBCK	ENST00000273980	p.S609T	c.1826G>C	Verified	CTG	AGT	AAT	2	G	C
TCGA-04-1542	TCERG1L	ENST00000368643	p.R368T	c.1103G>C	Verified	AAC	AGG	GAA	2	G	C
TCGA-23-1809	TDRD1	ENST00000251864	p.A859P	c.2575G>C	Unverified	CAA	GCC	AGA	1	G	C
TCGA-24-2280	TECTA	ENST00000392793	p.R1827T	c.5480G>C	Unverified	GAG	AGG	GAG	2	G	C
TCGA-13-1498	TEDDM1	ENST00000367565	p.K89N	c.267G>C	Verified	AGC	AAG	AAT	3	G	C
TCGA-29-1761	TEF	ENST00000266304	p.A266P	c.796G>C	Unverified	GCA	GCC	TTC	1	G	C
TCGA-25-2392	TEP1	ENST00000262715	p.E1811Q	c.5431G>C	Verified	CCA	GAG	GGG	1	G	C
TCGA-24-2035	TFAP2D	ENST00000008391	p.L292F	c.876G>C	Verified	TCC	TTG	GTT	3	G	C
TCGA-24-0975	TFDP3	NM_016521.2	p.A83P	c.247G>C	Verified	aga	gct	gcc	1	G	C
TCGA-23-1110	TFEB	ENST00000230323	p.A214A	c.642G>C	Verified	cct	gcg	gac	3	G	C
TCGA-09-1665	TG	ENST00000220616	p.C1543S	c.4628G>C	Verified	TTC	TGT	GTG	2	G	C
TCGA-61-1740	TGFBR1	ENST00000374994	p.G217A	c.650G>C	Unverified	ttt	gga	gaa	2	G	C
TCGA-10-0930	TGFBR3	ENST00000212355	p.S767T	c.2300G>C	Verified	CCA	AGC	ATG	2	G	C
TCGA-10-0930	TGFBR3	ENST00000212355	p.S767T	c.2300G>C	Verified	CCA	AGC	ATG	2	G	C
TCGA-61-1904	TGM7	ENST00000452443	p.G586A	c.1757G>C	Unverified	TCT	GGC	ATC	2	G	C
TCGA-29-1763	THAP11	ENST00000303596	p.Q124H	c.372G>C	Unverified	CAG	CAG	CAG	3	G	C
TCGA-13-1496	THBS2	ENST00000366787	p.R189P	c.566G>C	Verified	AGC	CGG	ATG	2	G	C
TCGA-13-0900	THBS3	ENST00000368378	p.E210D	c.630G>C	Verified	GAC	GAG	TCC	3	G	C
TCGA-29-1784	THOC5	ENST00000397871	p.Q253H	c.759G>C	Unverified	GAC	CAG	GCT	3	G	C
TCGA-13-0762	THOC6	ENST00000326266	p.A200P	c.598G>C	Verified	ACA	GCC	AAG	1	G	C
TCGA-13-0762	THOC6	ENST00000326266	p.A200P	c.598G>C	Verified	ACA	GCC	AAG	1	G	C
TCGA-24-0975	THSD7A	ENST00000423059	p.S1526S	c.4578G>C	Verified	CAC	TCG	TAC	3	G	C
TCGA-13-0893	TIMELESS	ENST00000553532	p.A119P	c.355G>C	Verified	CAG	GCC	TAC	1	G	C
TCGA-24-1616	TIMM17B	ENST00000376582	p.G120R	c.358G>C	Verified	ggg	ggc	atc	1	G	C
TCGA-23-1809	TIMM9	ENST00000395159	p.R66T	c.197G>C	Unverified	ATG	AGA	TTT	2	G	C
TCGA-25-1313	TKTL1	ENST00000369915	p.G377A	c.1130G>C	Verified	ATA	GGA	GGC	2	G	C
TCGA-29-1701	TLL1	ENST00000061240	p.R218P	c.653G>C	Unverified	GGT	CGG	CGA	2	G	C
TCGA-61-1915	TLR4	ENST00000355622	p.L785L	c.2355G>C	Unverified	GAG	CTG	TAC	3	G	C
TCGA-25-2401	TLR6	ENST00000381950	p.R446T	c.1337G>C	Verified	TTC	AGA	TGT	2	G	C
TCGA-29-1761	TM4SF1	ENST00000305366	p.C21S	c.62G>C	Unverified	CTG	TGC	ATC	2	G	C
TCGA-23-1124	TM7SF2	ENST00000279263	p.G200R	c.598G>C	Verified	ATC	GGC	TGG	1	G	C
TCGA-61-2094	TM7SF4	ENST00000297581	p.D255H	c.763G>C	Unverified	TTT	GAT	GAA	1	G	C
TCGA-29-1691	TMC2	ENST00000358864	p.E151Q	c.451G>C	Unverified	GAG	GAG	GAA	1	G	C
TCGA-61-1725	TMCC1	ENST00000393238	p.L601F	c.1803G>C	Unverified	CTT	TTG	GTC	3	G	C

TCGA-61-1725	TMCC1_ENS	ENST00000393238	p.L601F	c.1803G>C	Unverified	CTT	TTG	GTC	3	G	C
TCGA-13-1488	TMEM116	ENST00000354825	p.R140P	c.419G>C	Verified	GTG	CGC	TTC	2	G	C
TCGA-61-1740	TMEM127	ENST00000258439	p.E213Q	c.637G>C	Unverified	TCA	GAG	ATG	1	G	C
TCGA-24-2280	TMEM211	ENST00000382744	p.G5A	c.14G>C	Unverified	GGA	GGC	TGG	2	G	C
TCGA-24-2288	TMEM39B	ENST00000373634	p.G228R	c.682G>C	Unverified	CTG	GGC	TGT	1	G	C
TCGA-24-1104	TMEM71	ENST00000356838	p.S96T	c.287G>C	Verified	ACC	AGC	GTT	2	G	C
TCGA-29-1764	TMEM74	ENST00000297459	p.W141C	c.423G>C	Unverified	ggc	tgg	gaa	3	G	C
TCGA-36-1577	TMOD2	ENST00000249700	p.E33Q	c.97G>C	Verified	TTG	GAA	AAT	1	G	C
TCGA-30-1855	TMTC4	ENST00000342624	p.S676S	c.2028G>C	Unverified	TTC	TCG	TTG	3	G	C
TCGA-04-1342	TNFSF13B	ENST00000375887	p.A88P	c.262G>C	Unverified	cac	gcg	gag	1	G	C
TCGA-13-0900	TNIK	ENST00000436636	p.R43P	c.128G>C	Verified	GGT	CGT	CAT	2	G	C
TCGA-13-1499	TNKS2	ENST00000371627	p.D668H	c.2002G>C	Verified	CCT	GAT	AAT	1	G	C
TCGA-13-1512	TNN	ENST00000239462	p.Q269H	c.807G>C	Verified	CTG	CAG	CTG	3	G	C
TCGA-13-0923	TNNT1	ENST00000291901	p.D196H	c.586G>C	Verified	ATT	GAC	TAC	1	G	C
TCGA-61-1914	TNR	ENST00000367674	p.Q1354H	c.4062G>C	Unverified	CGG	CAG	TCC	3	G	C
TCGA-13-1498	TOB2	ENST00000327492	p.L150L	c.450G>C	Verified	TCC	CTG	TCC	3	G	C
TCGA-13-0919	TOPBP1	NM_007027.2	p.E636Q	c.1906G>C	Verified	ttg	gag	act	1	G	C
TCGA-13-0807	TP53	ENST00000269305	p.A276P	c.826G>C	Verified	TGT	GCC	TGT	1	G	C
TCGA-13-0905	TP53	ENST00000269305	p.R273P	c.818G>C	Verified	GTG	CGT	GTT	2	G	C
TCGA-23-1124	TP53	ENST00000269305	p.R156P	c.467G>C	Verified	ACC	CGC	GTC	2	G	C
TCGA-10-0930	TP53	ENST00000269305	p.?	c.375+5G>C	Verified	TGC	ACG	TAC	3	G	C
TCGA-10-0930	TP53	ENST00000269305	p.?	c.375+5G>C	Verified	TGC	ACG	TAC	3	G	C
TCGA-10-0930	TP53_ENST(	ENST00000269305	p.?	c.375+5G>C	Verified	TGC	ACG	TAC	3	G	C
TCGA-10-0930	TP53_ENST(	ENST00000269305	p.?	c.375+5G>C	Verified	TGC	ACG	TAC	3	G	C
TCGA-10-0930	TP53_ENST(	ENST00000413465	p.?	c.375+5G>C	Verified	TGC	ACG	TAC	3	G	C
TCGA-10-0930	TP53_ENST(	ENST00000413465	p.?	c.375+5G>C	Verified	TGC	ACG	TAC	3	G	C
TCGA-24-1422	TPBG	ENST00000369750	p.P115P	c.345G>C	Unverified	CGG	CCG	CCG	3	G	C
TCGA-61-2102	TPM2	ENST00000360958	p.V64L	c.190G>C	Verified	TCC	GTG	AAG	1	G	C
TCGA-13-1507	TPP2	ENST00000376065	p.S328T	c.983G>C	Verified	TAC	AGT	TAC	2	G	C
TCGA-09-0369	TRA2A	ENST00000297071	p.G35R	c.103G>C	Verified	TCA	GGA	TCT	1	G	C
TCGA-09-0369	TRA2A	ENST00000297071	p.G35R	c.103G>C	Verified	TCA	GGA	TCT	1	G	C
TCGA-29-1775	TRAM2	ENST00000182527	p.E186Q	c.556G>C	Unverified	AAG	GAG	GAA	1	G	C
TCGA-29-1761	TRANK1	ENST00000428977	p.R1707P	c.5120G>C	Unverified	TTC	CGG	TTC	2	G	C
TCGA-29-1761	TRANK1_EN	ENST00000429976	p.R2257P	c.6770G>C	Unverified	TTC	CGG	TTC	2	G	C

TCGA-09-1674	TRIM2	ENST00000437508	p.K31N	c.93G>C	Unverified	tac	aag	aat	3	G	C
TCGA-09-1674	TRIM2_ENST	ENST00000338700	p.K58N	c.174G>C	Unverified	TAC	AAG	AAT	3	G	C
TCGA-04-1338	TRIM21	ENST00000254436	p.V250L	c.748G>C	Verified	att	gtc	ctg	1	G	C
TCGA-13-0893	TRIM21	ENST00000254436	p.E192Q	c.574G>C	Verified	gaa	gaa	gaa	1	G	C
TCGA-09-2050	TRIM34	ENST00000514226	p.G60R	c.178G>C	Verified	tgt	ggt	atc	1	G	C
TCGA-04-1362	TRIM39	ENST00000376656	p.V351L	c.1051G>C	Verified	CTA	GTC	CTG	1	G	C
TCGA-24-1417	TRIM43	ENST00000272395	p.E207D	c.621G>C	Verified	CAA	GAG	ATT	3	G	C
TCGA-29-1784	TRIM44	ENST00000299413	p.E172D	c.516G>C	Unverified	gca	gag	aga	3	G	C
TCGA-24-1422	TRIM50	ENST00000333149	p.K390N	c.1170G>C	Unverified	CTG	AAG	GAG	3	G	C
TCGA-29-1777	TRIM56	ENST00000306085	p.S567T	c.1700G>C	Unverified	GCT	AGC	GCA	2	G	C
TCGA-29-1777	TRIM56_ENS	ENST00000306085	p.S567T	c.1700G>C	Unverified	GCT	AGC	GCA	2	G	C
TCGA-09-2050	TRIM6-TRIM	ENST00000337072	p.G414R	c.1240G>C	Verified	TGT	GGT	ATC	1	G	C
TCGA-24-1463	TRIM8	ENST00000302424	p.W524S	c.1571G>C	Verified	GAC	TGG	CTT	2	G	C
TCGA-61-1914	TRIO	ENST00000344204	p.Q1066H	c.3198G>C	Unverified	gag	cag	aag	3	G	C
TCGA-09-2051	TRIOBP	ENST00000407319	p.V137L	c.409G>C	Unverified	GCG	GTG	CAG	1	G	C
TCGA-09-2051	TRIOBP_ENS	ENST00000406386	p.V1850L	c.5548G>C	Unverified	GCG	GTG	CAG	1	G	C
TCGA-61-1904	TRIOBP_ENS	ENST00000406386	p.C26S	c.77G>C	Unverified	AAC	TGC	TTC	2	G	C
TCGA-30-1855	TRPM7	ENST00000313478	p.V136L	c.406G>C	Unverified	CTT	GTT	ATC	1	G	C
TCGA-24-1103	TRRAP	ENST00000355540	p.E3420Q	c.10258G>C	Verified	TTG	GAG	GCC	1	G	C
TCGA-23-2077	TSEN54	ENST00000333213	p.D451H	c.1351G>C	Verified	TTT	GAT	GTT	1	G	C
TCGA-29-1693	TSEN54	ENST00000333213	p.S204T	c.611G>C	Unverified	AGC	AGC	TCC	2	G	C
TCGA-61-1740	TSPAN5	ENST00000305798	p.G155G	c.465G>C	Unverified	TGT	GGG	GCT	3	G	C
TCGA-13-0890	TSR1	ENST00000301364	p.K384N	c.1152G>C	Verified	TTG	AAG	GAA	3	G	C
TCGA-24-1845	TTC13	ENST00000366661	p.C856S	c.2567G>C	Unverified	CGT	TGT	CTT	2	G	C
TCGA-24-1616	TTC37	ENST00000358746	p.D686H	c.2056G>C	Verified	GTT	GAT	TAT	1	G	C
TCGA-24-2289	TTC39B	NM_152574.1	p.A47A	c.141G>C	Unverified	cag	gcg	ccc	3	G	C
TCGA-23-1029	TTC39C	ENST00000304621	p.V335L	c.1003G>C	Unverified	GCA	GTT	TGT	1	G	C
TCGA-23-1029	TTC39C_ENS	ENST00000317571_v68	p.V396L	c.1186G>C	Unverified	GCA	GTT	TGT	1	G	C
TCGA-09-1665	TTI1	ENST00000373448	p.L205F	c.615G>C	Verified	GAT	TTG	TTT	3	G	C
TCGA-13-0923	TTLL9	ENST00000375938	p.R157P	c.470G>C	Verified	TTT	CGC	AAA	2	G	C
TCGA-29-1785	TTN	NM_003319	p.D25277H	c.75829G>C	Unverified	gaa	gac	agc	1	G	C
TCGA-29-1785	TTN	NM_003319	p.D25277H	c.75829G>C	Unverified	gaa	gac	agc	1	G	C
TCGA-24-1423	TTN	NM_003319	p.R11749T	c.35246G>C	Verified	aca	aga	tca	2	G	C
TCGA-30-1718	TTN	NM_003319	p.C5671S	c.17012G>C	Unverified	aac	tgt	cgc	2	G	C

TCGA-23-1111	TTN	NM_003319	p.K4571N	c.13713G>C	Unverified	gcc	aag	cca	3	G	C
TCGA-29-1785	TTN_ENST0(	ENST00000342175	p.D25469H	c.76405G>C	Unverified	GAA	GAC	AGC	1	G	C
TCGA-29-1785	TTN_ENST0(	ENST00000342175	p.D25469H	c.76405G>C	Unverified	GAA	GAC	AGC	1	G	C
TCGA-30-1718	TTN_ENST0(	ENST00000342175	p.C5863S	c.17588G>C	Unverified	AAC	TGT	CGC	2	G	C
TCGA-23-1111	TTN_ENST0(	ENST00000342175	p.K4763N	c.14289G>C	Unverified	GCC	AAG	CCA	3	G	C
TCGA-29-1785	TTN_ENST0(	ENST00000342992	p.D31774H	c.95320G>C	Unverified	GAA	GAC	AGC	1	G	C
TCGA-29-1785	TTN_ENST0(	ENST00000342992	p.D31774H	c.95320G>C	Unverified	GAA	GAC	AGC	1	G	C
TCGA-30-1718	TTN_ENST0(	ENST00000342992	p.C12168S	c.36503G>C	Unverified	AAC	TGT	CGC	2	G	C
TCGA-23-1111	TTN_ENST0(	ENST00000342992	p.K11068N	c.33204G>C	Unverified	GCC	AAG	CCA	3	G	C
TCGA-13-1408	TTN_ENST0(	ENST00000356127	p.D7159H	c.21475G>C	Verified	GAT	GAT	GCT	1	G	C
TCGA-29-1785	TTN_ENST0(	ENST00000356127	p.D31772H	c.95314G>C	Unverified	GAA	GAC	AGC	1	G	C
TCGA-29-1785	TTN_ENST0(	ENST00000356127	p.D31772H	c.95314G>C	Unverified	GAA	GAC	AGC	1	G	C
TCGA-24-1423	TTN_ENST0(	ENST00000356127	p.R18244T	c.54731G>C	Verified	ACA	AGA	TCA	2	G	C
TCGA-30-1718	TTN_ENST0(	ENST00000356127	p.C12168S	c.36503G>C	Unverified	AAC	TGT	CGC	2	G	C
TCGA-13-0887	TTN_ENST0(	ENST00000356127	p.Q7912H	c.23736G>C	Verified	TCT	CAG	AGG	3	G	C
TCGA-23-1111	TTN_ENST0(	ENST00000356127	p.K11068N	c.33204G>C	Unverified	GCC	AAG	CCA	3	G	C
TCGA-29-1785	TTN_ENST0(	ENST00000359218	p.D25402H	c.76204G>C	Unverified	GAA	GAC	AGC	1	G	C
TCGA-29-1785	TTN_ENST0(	ENST00000359218	p.D25402H	c.76204G>C	Unverified	GAA	GAC	AGC	1	G	C
TCGA-30-1718	TTN_ENST0(	ENST00000359218	p.C5796S	c.17387G>C	Unverified	AAC	TGT	CGC	2	G	C
TCGA-23-1111	TTN_ENST0(	ENST00000359218	p.K4696N	c.14088G>C	Unverified	GCC	AAG	CCA	3	G	C
TCGA-13-0887	TUBA4A	ENST00000248437	p.D218H	c.652G>C	Verified	CTA	GAC	ATC	1	G	C
TCGA-13-0887	TUBA4A_EN	ENST00000392088	p.D203H	c.607G>C	Verified	CTA	GAC	ATC	1	G	C
TCGA-23-1032	TUBG2	ENST00000251412	p.S259S	c.777G>C	Verified	GCC	TCG	CTC	3	G	C
TCGA-13-0905	TULP1	ENST00000229771	p.G414R	c.1240G>C	Verified	CTG	GGC	TTC	1	G	C
TCGA-23-1123	TULP1	ENST00000229771	p.E204Q	c.610G>C	Verified	GGG	GAG	GCC	1	G	C
TCGA-23-1123	TULP1	ENST00000229771	p.E204Q	c.610G>C	Verified	GGG	GAG	GCC	1	G	C
TCGA-24-1846	TXNDC12	ENST00000371626	p.V59L	c.175G>C	Unverified	ATG	GTG	ATT	1	G	C
TCGA-61-1907	TYK2	ENST00000525621	p.E636D	c.1908G>C	Unverified	CAG	GAG	CTA	3	G	C
TCGA-24-1104	UBAP2	ENST00000379238	p.D156H	c.466G>C	Verified	ATT	GAT	TGC	1	G	C
TCGA-23-1123	UBAP2L	ENST00000361546	p.Q483H	c.1449G>C	Verified	CAG	CAG	AAA	3	G	C
TCGA-23-1123	UBAP2L	ENST00000361546	p.Q483H	c.1449G>C	Verified	CAG	CAG	AAA	3	G	C
TCGA-61-1740	UBE3C	ENST00000348165	p.L670F	c.2010G>C	Unverified	ggt	ttg	gag	3	G	C
TCGA-13-0920	UBE4B	ENST00000253251	p.G1065G	c.3195G>C	Verified	gca	ggg	atc	3	G	C
TCGA-13-0904	UCHL5	ENST00000367455	p.D75H	c.223G>C	Verified	CTT	GAC	ACG	1	G	C

TCGA-61-1904	UCP3	ENST00000314032	p.L207L	c.621G>C	Unverified	AAG	CTG	CTG	3	G	C
TCGA-29-1768	UEVLD	ENST00000543987	p.G340R	c.1018G>C	Unverified	TCA	GGC	AAA	1	G	C
TCGA-29-1769	UEVLD	ENST00000543987	p.W174C	c.522G>C	Unverified	AGC	TGG	GCA	3	G	C
TCGA-29-1768	UEVLD_ENST	ENST00000396197	p.G340R	c.1018G>C	Unverified	TCA	GGC	AAA	1	G	C
TCGA-29-1769	UEVLD_ENST	ENST00000396197	p.W174C	c.522G>C	Unverified	AGC	TGG	GCA	3	G	C
TCGA-24-2267	UGT1A3	ENST00000482026	p.C281S	c.842G>C	Verified	AAC	TGT	GCC	2	G	C
TCGA-25-1326	UHRF1BP1	ENST00000192788	p.L661L	c.1983G>C	Verified	CTT	CTG	CAC	3	G	C
TCGA-13-0916	UNC13C	ENST00000260323	p.K1913N	c.5739G>C	Verified	CTA	AAG	CGA	3	G	C
TCGA-25-2392	USH2A	ENST00000307340	p.G3754A	c.11261G>C	Verified	ACT	GGA	GGT	2	G	C
TCGA-29-1775	USP14	ENST00000261601	p.E490D	c.1470G>C	Unverified	gaa	gag	gaa	3	G	C
TCGA-13-0883	USP2	ENST00000260187	p.Q355H	c.1065G>C	Verified	AAT	CAG	CAG	3	G	C
TCGA-13-0795	USP25	ENST00000285679	p.G946R	c.2836G>C	Verified	tct	gga	gag	1	G	C
TCGA-29-1761	USP28	ENST00000003302	p.R428T	c.1283G>C	Unverified	GAA	AGG	TAT	2	G	C
TCGA-13-0883	USP34	ENST00000398571	p.Q282H	c.846G>C	Verified	GAT	CAG	GAG	3	G	C
TCGA-13-1488	USP40	ENST00000450966	p.R730T	c.2189G>C	Verified	CTC	AGA	AAT	2	G	C
TCGA-09-1674	USP42	XM_374396.2	p.S416S	c.1248G>C	Unverified	aac	tcg	tac	3	G	C
TCGA-09-1674	USP42_ENST	ENST00000306177	p.S288S	c.864G>C	Unverified	AAC	TCG	TAC	3	G	C
TCGA-13-1498	USP53	ENST00000274030	p.D398H	c.1192G>C	Verified	GGT	GAT	CAG	1	G	C
TCGA-61-1899	USP7	ENST00000344836	p.R153P	c.458G>C	Unverified	CGT	CGT	ATT	2	G	C
TCGA-23-1022	UST	ENST00000367463	p.D311H	c.931G>C	Verified	AAA	GAC	CCA	1	G	C
TCGA-29-1761	UTS2	ENST00000054668	p.E78Q	c.232G>C	Unverified	GCA	GAA	AGA	1	G	C
TCGA-29-1761	UTS2_ENST(	ENST00000361696	p.E63Q	c.187G>C	Unverified	GCA	GAA	AGA	1	G	C
TCGA-29-1761	UTS2_ENST(	ENST00000377516	p.E63Q	c.187G>C	Unverified	GCA	GAA	AGA	1	G	C
TCGA-23-2077	VAV2	ENST00000406606	p.G168R	c.502G>C	Unverified	GGA	GGG	GAC	1	G	C
TCGA-25-1326	VCPIP1	ENST00000310421	p.V480L	c.1438G>C	Verified	cat	gtt	cct	1	G	C
TCGA-30-1718	VCX	ENST00000381059	p.P5P	c.15G>C	Unverified	AAG	CCG	AGA	3	G	C
TCGA-29-1784	VGLL3	ENST00000398399	p.A290P	c.868G>C	Unverified	TCT	GCT	ACC	1	G	C
TCGA-23-2078	VNN1	ENST00000367928	p.C509S	c.1526G>C	Verified	GTA	TGC	TCA	2	G	C
TCGA-04-1337	VSIG2	ENST00000326621	p.G305R	c.913G>C	Unverified	AAG	GGG	TTC	1	G	C
TCGA-30-1718	VSIG4	ENST00000374737	p.G48A	c.143G>C	Unverified	caa	ggc	tac	2	G	C
TCGA-09-2056	WAPAL	ENST00000298767	p.G1173R	c.3517G>C	Verified	GTT	GGA	ACA	1	G	C
TCGA-30-1857	WAPAL	ENST00000298767	p.K60N	c.180G>C	Unverified	CCG	AAG	AAA	3	G	C
TCGA-29-1784	WBP2	ENST00000254806	p.G101R	c.301G>C	Unverified	GCG	GGA	GGT	1	G	C
TCGA-09-1674	WDR11	ENST00000263461	p.E982Q	c.2944G>C	Unverified	CTA	GAA	AGG	1	G	C

TCGA-29-1775	WDR23	ENST00000559115	p.R487P	c.1460G>C	Unverified	GTG	CGT	GAC	2	G	C
TCGA-29-1776	WDR33	ENST00000322313	p.V52L	c.154G>C	Unverified	GCT	GTG	AAC	1	G	C
TCGA-29-1776	WDR33_ENS	ENST00000409658	p.V52L	c.154G>C	Unverified	GCT	GTG	AAC	1	G	C
TCGA-24-1463	WDR43	ENST00000296126	p.D586H	c.1756G>C	Verified	CCT	GAC	CTG	1	G	C
TCGA-24-1844	WDR6	ENST00000315574	p.V956L	c.2866G>C	Unverified	ACT	GTC	CTG	1	G	C
TCGA-24-2289	WDR66	ENST00000288912	p.S433T	c.1298G>C	Unverified	CAC	AGT	GCC	2	G	C
TCGA-04-1530	WDR75	ENST00000314761	p.D794H	c.2380G>C	Verified	CAG	GAT	ACA	1	G	C
TCGA-13-1507	WFIKKN2	ENST00000311378	p.E162D	c.486G>C	Verified	GCC	GAG	GCC	3	G	C
TCGA-04-1338	WHSC1	ENST00000382891	p.E1087Q	c.3259G>C	Verified	GGA	GAA	TTT	1	G	C
TCGA-24-2288	WHSC1L1	ENST00000316985	p.W275S	c.824G>C	Unverified	GTG	TGG	TCC	2	G	C
TCGA-20-0990	WIF1	ENST00000286574	p.?	c.288+1G>C	Unverified	GGG	CAG	GCA	3	G	C
TCGA-20-0990	WIF1_ENST(	ENST00000286574	p.?	c.288+1G>C	Unverified	GGG	CAG	GCA	3	G	C
TCGA-13-0913	WNT7A	ENST00000285018	p.E199Q	c.595G>C	Verified	CTG	GAA	TGT	1	G	C
TCGA-13-1498	WNT8A	ENST00000398754	p.V25L	c.73G>C	Verified	TCA	GTG	AAC	1	G	C
TCGA-13-0884	XIRP1	ENST00000340369	p.G545A	c.1634G>C	Verified	GCT	GGG	GAC	2	G	C
TCGA-29-1775	XIRP2	ENST00000295237	p.T2475T	c.7425G>C	Unverified	CAC	ACG	GAG	3	G	C
TCGA-24-1422	XPC	ENST00000285021	p.G757R	c.2269G>C	Unverified	TTT	GGG	AAT	1	G	C
TCGA-29-1770	XPC	ENST00000285021	p.E518D	c.1554G>C	Unverified	GGT	GAG	AAG	3	G	C
TCGA-04-1530	XPNPEP3	ENST00000357137	p.S504T	c.1511G>C	Unverified	TGC	AGC	CAG	2	G	C
TCGA-13-0714	XPO6	ENST00000304658	p.G801R	c.2401G>C	Verified	TCG	GGG	GAG	1	G	C
TCGA-24-0979	XPOT	ENST00000332707	p.C268S	c.803G>C	Verified	ttg	tgt	caa	2	G	C
TCGA-24-2024	XPOT	ENST00000332707	p.V775V	c.2325G>C	Verified	gaa	gtg	ctg	3	G	C
TCGA-13-1488	XRCC1	ENST00000262887	p.R246T	c.737G>C	Verified	AAG	AGG	AAG	2	G	C
TCGA-61-1907	XRCC1	ENST00000262887	p.P514P	c.1542G>C	Unverified	GAC	CCG	TAT	3	G	C
TCGA-29-1761	XYLT2	ENST00000017003	p.C581S	c.1742G>C	Unverified	CTC	TGC	AGG	2	G	C
TCGA-09-2050	YME1L1	ENST00000326799	p.L211F	c.633G>C	Verified	ACT	TTG	AAA	3	G	C
TCGA-24-2290	ZBP1	ENST00000371173	p.K148N	c.444G>C	Verified	ATG	AAG	AGC	3	G	C
TCGA-24-2290	ZBP1	ENST00000371173	p.K148N	c.444G>C	Verified	ATG	AAG	AGC	3	G	C
TCGA-29-1763	ZBTB11	ENST00000312938	p.V1042L	c.3124G>C	Unverified	ACT	GTT	AAA	1	G	C
TCGA-13-0923	ZBTB20	ENST00000357258	p.V272L	c.814G>C	Verified	AGG	GTG	CAG	1	G	C
TCGA-13-0920	ZBTB49	ENST00000337872	p.D291H	c.871G>C	Verified	TCA	GAC	GCC	1	G	C
TCGA-09-2051	ZC3H13	ENST00000282007	p.K385N	c.1155G>C	Unverified	AGA	AAG	CAG	3	G	C
TCGA-13-1501	ZC3H14	ENST00000251038	p.R278T	c.833G>C	Verified	TTT	AGA	AAC	2	G	C
TCGA-13-1501	ZC3H14	ENST00000251038	p.R278T	c.833G>C	Verified	TTT	AGA	AAC	2	G	C

TCGA-04-1338	ZCCHC17	ENST00000373714	p.R80T	c.239G>C	Verified	GAT	AGA	ATA	2	G	C
TCGA-23-1031	ZCCHC5	ENST00000321110	p.S260T	c.779G>C	Verified	tat	agt	tac	2	G	C
TCGA-13-0919	ZFHX3	ENST00000268489	p.V815V	c.2445G>C	Unverified	AAC	GTG	GCC	3	G	C
TCGA-23-1021	ZFP2	ENST00000361362	p.E190D	c.570G>C	Verified	AAA	GAG	TGT	3	G	C
TCGA-13-0883	ZFP91	ENST00000316059	p.L499F	c.1497G>C	Verified	CCC	TTG	GGA	3	G	C
TCGA-13-0883	ZFP91-CNTF	ENST00000389919	p.L499F	c.1497G>C	Verified	CCC	TTG	GGA	3	G	C
TCGA-29-1691	ZFYVE20	ENST00000253699	p.G607R	c.1819G>C	Unverified	GTT	GGC	CAG	1	G	C
TCGA-23-2077	ZGLP1	ENST00000403903	p.K255N	c.765G>C	Verified	CCC	AAG	AGG	3	G	C
TCGA-24-2035	ZHX2	ENST00000314393	p.G601A	c.1802G>C	Verified	aaa	ggc	caa	2	G	C
TCGA-29-1781	ZKSCAN2	ENST00000328086	p.K563N	c.1689G>C	Unverified	CGC	AAG	GTG	3	G	C
TCGA-20-1683	ZKSCAN4	ENST00000377294	p.G183R	c.547G>C	Unverified	CTG	GGA	TCC	1	G	C
TCGA-25-1313	ZKSCAN4	ENST00000377294	p.G523A	c.1568G>C	Verified	AAA	GGT	TTC	2	G	C
TCGA-25-1326	ZKSCAN5	ENST00000394170	p.K337N	c.1011G>C	Verified	CCT	AAG	CAA	3	G	C
TCGA-30-1718	ZMAT2	ENST00000274712	p.G4R	c.10G>C	Unverified	TCG	GGC	AGC	1	G	C
TCGA-13-0762	ZMYM4	ENST00000314607	p.L845F	c.2535G>C	Unverified	ATC	TTG	ATG	3	G	C
TCGA-13-0762	ZMYM4	ENST00000314607	p.L845F	c.2535G>C	Unverified	ATC	TTG	ATG	3	G	C
TCGA-13-1510	ZMYND15	ENST00000269289	p.R639T	c.1916G>C	Unverified	CTC	AGA	GCG	2	G	C
TCGA-29-1766	ZNF138	ENST00000359735	p.E218Q	c.652G>C	Unverified	ACT	GAA	GAG	1	G	C
TCGA-29-1766	ZNF138_ENS	ENST00000440155	p.E249Q	c.745G>C	Unverified	ACT	GAA	GAG	1	G	C
TCGA-24-2267	ZNF142	ENST00000396788	p.L397L	c.1191G>C	Verified	GTG	CTG	AGC	3	G	C
TCGA-24-1845	ZNF160	ENST00000429604	p.K454N	c.1362G>C	Unverified	TAC	AAG	TGT	3	G	C
TCGA-13-1481	ZNF169	ENST00000395395	p.S226T	c.677G>C	Verified	TCA	AGC	CTG	2	G	C
TCGA-13-1501	ZNF184	ENST00000377419	p.G442A	c.1325G>C	Verified	ACT	GGG	GAG	2	G	C
TCGA-13-1501	ZNF184	ENST00000377419	p.G442A	c.1325G>C	Verified	ACT	GGG	GAG	2	G	C
TCGA-61-2109	ZNF20_ENST	ENST00000334213	p.C204S	c.611G>C	Unverified	TTT	TGT	GGG	2	G	C
TCGA-25-2398	ZNF217	ENST00000371471	p.C999S	c.2996G>C	Verified	ACT	TGT	GTG	2	G	C
TCGA-61-1907	ZNF236	ENST00000253159	p.L839F	c.2517G>C	Unverified	CAG	TTG	GCA	3	G	C
TCGA-61-1907	ZNF236_ENS	ENST00000543926	p.L839F	c.2517G>C	Unverified	CAG	TTG	GCA	3	G	C
TCGA-25-1313	ZNF25	ENST00000302609	p.C263S	c.788G>C	Verified	GAG	TGT	GGG	2	G	C
TCGA-23-1022	ZNF324B	ENST00000336614	p.S298S	c.894G>C	Verified	ACG	TCG	CAC	3	G	C
TCGA-29-1785	ZNF335	ENST00000322927	p.A1104P	c.3310G>C	Unverified	TTT	GCA	TGC	1	G	C
TCGA-29-1785	ZNF335	ENST00000322927	p.A1104P	c.3310G>C	Unverified	TTT	GCA	TGC	1	G	C
TCGA-24-1463	ZNF341	ENST00000342427	p.K461N	c.1383G>C	Verified	CAT	AAG	AAT	3	G	C
TCGA-29-1776	ZNF343	ENST00000278772	p.E385Q	c.1153G>C	Unverified	CTG	GAG	TGT	1	G	C

TCGA-13-0791	ZNF354C	ENST00000315475	p.E131Q	c.391G>C	Verified	TTT	GAG	AGC	1	G	C
TCGA-13-0890	ZNF429	ENST00000358491	p.R67P	c.200G>C	Verified	AAG	CGA	CAT	2	G	C
TCGA-25-2398	ZNF441_EN	ENST00000357901	p.E395Q	c.1183G>C	Unverified	TGT	GAA	TGT	1	G	C
TCGA-61-2109	ZNF45	ENST00000269973	p.G452A	c.1355G>C	Unverified	AAG	GGC	TTC	2	G	C
TCGA-29-1769	ZNF473	ENST00000391821	p.G155R	c.463G>C	Unverified	AGA	GGA	CTC	1	G	C
TCGA-29-1781	ZNF473	ENST00000391821	p.Q198H	c.594G>C	Unverified	AGC	CAG	CAG	3	G	C
TCGA-23-1021	ZNF479	ENST00000331162	p.R345T	c.1034G>C	Unverified	AAG	AGA	ATT	2	G	C
TCGA-23-1117	ZNF496	ENST00000294753	p.E344Q	c.1030G>C	Verified	CTA	GAG	AAC	1	G	C
TCGA-23-1117	ZNF496	ENST00000294753	p.E344Q	c.1030G>C	Verified	CTA	GAG	AAC	1	G	C
TCGA-24-1469	ZNF516	ENST00000217537	p.G720R	c.2158G>C	Verified	TCT	GGG	GGA	1	G	C
TCGA-29-1763	ZNF521	NM_015461.1	p.E109Q	c.325G>C	Unverified	gga	gag	gaa	1	G	C
TCGA-29-1763	ZNF521	NM_015461.1	p.G793A	c.2378G>C	Unverified	ttt	ggc	acc	2	G	C
TCGA-25-2393	ZNF536	ENST00000355537	p.G871R	c.2611G>C	Verified	TCG	GGG	CAG	1	G	C
TCGA-29-1762	ZNF552	ENST00000391701	p.R322T	c.965G>C	Unverified	GAA	AGG	CCA	2	G	C
TCGA-20-0991	ZNF572	ENST00000319286	p.C417S	c.1250G>C	Verified	GAG	TGC	TGG	2	G	C
TCGA-13-0903	ZNF592	ENST00000299927	p.E1062Q	c.3184G>C	Verified	CTG	GAG	AGC	1	G	C
TCGA-25-2401	ZNF605	ENST00000360187	p.E9Q	c.25G>C	Verified	TTT	GAG	GAT	1	G	C
TCGA-61-1733	ZNF608	ENST00000306315	p.E1278D	c.3834G>C	Unverified	AAA	GAG	AGT	3	G	C
TCGA-61-1733	ZNF608	ENST00000306315	p.E1278D	c.3834G>C	Unverified	AAA	GAG	AGT	3	G	C
TCGA-61-1915	ZNF618	ENST00000288466	p.E232Q	c.694G>C	Unverified	AAA	GAA	GTT	1	G	C
TCGA-61-1915	ZNF618_EN	ENST00000288466	p.E232Q	c.694G>C	Unverified	AAA	GAA	GTT	1	G	C
TCGA-20-1686	ZNF638	ENST00000264447	p.D1539H	c.4615G>C	Unverified	TTG	GAT	GAA	1	G	C
TCGA-61-1913	ZNF645	ENST00000323684	p.R69P	c.206G>C	Unverified	ggg	cga	ata	2	G	C
TCGA-24-1844	ZNF665	ENST00000396424	p.G455R	c.1363G>C	Unverified	GGA	GAA	AAG	1	G	C
TCGA-24-1844	ZNF665_EN	ENST00000396424	p.G520R	c.1558G>C	Unverified	TGT	GGC	AAA	1	G	C
TCGA-61-1910	ZNF673	ENST00000298190	p.K107N	c.321G>C	Unverified	GGC	AAG	GAA	3	G	C
TCGA-23-1022	ZNF724P	ENST00000418100	p.G390A	c.1169G>C	Verified	ACT	GGA	GAA	2	G	C
TCGA-29-1775	ZNF732_EN	ENST00000419098	p.C423S	c.1268G>C	Unverified	GAG	TGT	GGC	2	G	C
TCGA-25-1326	ZNF746	ENST00000340622	p.R346P	c.1037G>C	Unverified	CTG	CGA	GAG	2	G	C
TCGA-61-1900	ZNF804A	ENST00000302277	p.?	c.255+1G>C	Unverified	AAG	CAG	AGG	3	G	C
TCGA-04-1542	ZNF828	ENST00000361283	p.D89H	c.265G>C	Unverified	CCA	GAC	AAA	1	G	C
TCGA-09-1665	ZNF828	ENST00000361283	p.G474R	c.1420G>C	Verified	CGT	GGT	GGT	1	G	C
TCGA-23-1111	ZNF828	ENST00000361283	p.R769P	c.2306G>C	Unverified	CCA	CGT	TGT	2	G	C
TCGA-24-1470	ZNF91_ENS	ENST00000300619	p.R171T	c.512G>C	Verified	AAC	AGA	CAT	2	G	C

TCGA-13-0920	ZSCAN2	ENST00000448803	p.G144G	c.432G>C	Verified	AAT	GGG	GAG	3	G	C
TCGA-04-1638	ZSWIM1	ENST00000372523	p.E140Q	c.418G>C	Unverified	TGG	GAG	AGA	1	G	C
TCGA-61-1740	ZSWIM2	ENST00000295131	p.K305N	c.915G>C	Unverified	GAA	AAG	ATG	3	G	C
TCGA-13-0762	ZW10	ENST00000200135	p.?	c.1584-1G>C	Unverified	cac	aag	gag	3	G	C
TCGA-13-0762	ZW10	ENST00000200135	p.?	c.1584-1G>C	Unverified	cac	aag	gag	3	G	C
TCGA-24-2262	ZXDB	ENST00000374888	p.R598T	c.1793G>C	Unverified	cag	aga	cag	2	G	C
TCGA-09-2051	ZZEF1	ENST00000381638	p.G1953A	c.5858G>C	Unverified	CAG	GGA	AAA	2	G	C
TCGA-25-2393	AACS	ENST00000316519	p.D523Y	c.1567G>T	Unverified	GGC	GAC	TAC	1	G	T
TCGA-24-1103	AASDH	ENST00000205214	p.S641I	c.1922G>T	Verified	AAG	AGT	TGT	2	G	T
TCGA-24-1435	ABCA4	ENST00000370225	p.V552L	c.1654G>T	Verified	GTG	GTA	TTC	1	G	T
TCGA-61-2009	ABCA8	ENST00000269080	p.D726Y	c.2176G>T	Verified	CTT	GAT	AGC	1	G	T
TCGA-23-2077	ABCB1	ENST00000265724	p.G722V	c.2165G>T	Verified	aat	gga	ggc	2	G	T
TCGA-20-1683	ABCC1	ENST00000399410	p.V987V	c.2961G>T	Unverified	CAT	GTG	TCC	3	G	T
TCGA-13-0920	ABCC10	ENST00000244533	p.A1367S	c.4099G>T	Verified	AGG	GCT	CTC	1	G	T
TCGA-23-1110	ABCC11	ENST00000356608	p.E782*	c.2344G>T	Verified	GAG	GAG	ATG	1	G	T
TCGA-25-2392	ABCC8	ENST00000389817	p.D1354Y	c.4060G>T	Verified	TAC	GAC	AGC	1	G	T
TCGA-04-1356	ABCG5	ENST00000260645	p.L394L	c.1182G>T	Verified	AAT	CTG	ATC	3	G	T
TCGA-61-1725	ABHD10	ENST00000273359	p.V175L	c.523G>T	Unverified	GGT	GTA	GCT	1	G	T
TCGA-29-1763	ACACB	ENST00000377848	p.E593*	c.1777G>T	Unverified	ACA	GAA	ATG	1	G	T
TCGA-13-0903	ACACB	ENST00000377848	p.M1253I	c.3759G>T	Verified	GAC	ATG	TAC	3	G	T
TCGA-04-1367	ACAD11	ENST00000264990	p.A5S	c.13G>T	Unverified	GGT	GCT	ACT	1	G	T
TCGA-13-0923	ACAD9	ENST00000308982	p.G267V	c.800G>T	Verified	CGG	GGC	TCC	2	G	T
TCGA-61-1906	ACCN2	ENST00000228468	p.V362L	c.1084G>T	Unverified	TGC	GTG	TGT	1	G	T
TCGA-61-1906	ACCN2	ENST00000228468	p.V362L	c.1084G>T	Unverified	TGC	GTG	TGT	1	G	T
TCGA-23-2077	ACOT12	ENST00000307624	p.C40F	c.119G>T	Unverified	GCC	TGC	CTG	2	G	T
TCGA-24-1431	ACOT9	ENST00000336430	p.V280F	c.838G>T	Verified	cga	gtt	tta	1	G	T
TCGA-24-2289	ACSM5	ENST00000331849	p.C343F	c.1028G>T	Unverified	CAC	TGT	CTG	2	G	T
TCGA-30-1857	ACTA1	ENST00000366684	p.G50C	c.148G>T	Unverified	ATG	GGT	CAG	1	G	T
TCGA-24-1423	ACTL6B	ENST00000160382	p.E385*	c.1153G>T	Verified	ATG	GAG	CGC	1	G	T
TCGA-20-1685	ACTN2	ENST00000366578	p.A476S	c.1426G>T	Unverified	GAC	GCT	GTG	1	G	T
TCGA-13-1497	ACTN2	ENST00000366578	p.R407M	c.1220G>T	Verified	TTC	AGG	CAG	2	G	T
TCGA-25-2391	ACTR3B	ENST00000256001	p.E402D	c.1206G>T	Unverified	GAA	GAG	TAC	3	G	T
TCGA-13-0904	ACVR1	ENST00000263640	p.G69V	c.206G>T	Verified	AAA	GGC	TGC	2	G	T
TCGA-13-0886	ADAM19	NM_023038.2	p.L720L	c.2160G>T	Verified	atg	ctg	atg	3	G	T

TCGA-61-1906	ADAM2	ENST00000265708	p.G424V	c.1271G>T	Unverified	gcc	ggt	tca	2	G	T
TCGA-61-1906	ADAM2	ENST00000265708	p.G424V	c.1271G>T	Unverified	gcc	ggt	tca	2	G	T
TCGA-04-1347	ADAM29	ENST00000359240	p.G335V	c.1004G>T	Verified	CTA	GGT	CAT	2	G	T
TCGA-29-1769	ADAMTS18	ENST00000282849	p.T65T	c.195G>T	Unverified	gtc	acg	cca	3	G	T
TCGA-04-1652	ADAMTS2	ENST00000251582	p.A540S	c.1618G>T	Unverified	TGT	GCA	CCT	1	G	T
TCGA-04-1652	ADAMTS2	ENST00000251582	p.A540S	c.1618G>T	Unverified	TGT	GCA	CCT	1	G	T
TCGA-13-0793	ADAMTS4	ENST00000367996	p.V785F	c.2353G>T	Verified	CAA	GTC	CTA	1	G	T
TCGA-61-1900	ADAMTS4	ENST00000367996	p.V714F	c.2140G>T	Unverified	CTT	GTC	CGG	1	G	T
TCGA-61-1900	ADAMTS4_E	ENST00000367996	p.V714F	c.2140G>T	Unverified	CTT	GTC	CGG	1	G	T
TCGA-24-1845	ADAMTS7	ENST00000388820	p.A1675S	c.5023G>T	Unverified	GGC	GCC	CCC	1	G	T
TCGA-13-0913	ADAMTSL1_	ENST00000380548	p.K868N	c.2604G>T	Verified	AGG	AAG	GTC	3	G	T
TCGA-29-1711	ADAMTSL3	ENST00000286744	p.R1207M	c.3620G>T	Unverified	AAA	AGG	ACA	2	G	T
TCGA-29-1711	ADAMTSL3	ENST00000286744	p.R1207M	c.3620G>T	Unverified	AAA	AGG	ACA	2	G	T
TCGA-61-1998	ADAR	ENST00000368474	p.A319S	c.955G>T	Verified	TTG	GCT	AAA	1	G	T
TCGA-61-1740	ADCK1	ENST00000341211	p.G162W	c.484G>T	Unverified	gaa	ggg	agg	1	G	T
TCGA-61-1740	ADCK1_ENS	ENST00000238561	p.G230W	c.688G>T	Unverified	GAA	GGG	AGG	1	G	T
TCGA-25-2391	ADCY10	ENST00000367851	p.G408C	c.1222G>T	Unverified	ATT	GGT	CAA	1	G	T
TCGA-61-1740	ADCY2	ENST00000338316	p.G298C	c.892G>T	Unverified	GTT	GGC	TTT	1	G	T
TCGA-24-1469	ADCY9	ENST00000294016	p.C583F	c.1748G>T	Verified	AGC	TGT	GCA	2	G	T
TCGA-24-1844	ADH5	ENST00000296412	p.C174F	c.521G>T	Unverified	ggt	tgt	ggc	2	G	T
TCGA-61-2095	ADNP2	ENST00000262198	p.V622F	c.1864G>T	Unverified	TCT	GTC	ACT	1	G	T
TCGA-24-1469	ADORA3	ENST00000241356	p.M174I	c.522G>T	Verified	AGA	ATG	GAC	3	G	T
TCGA-24-1846	ADRA2A	ENST00000280155	p.G364W	c.1090G>T	Unverified	CGC	GGG	CGG	1	G	T
TCGA-13-0885	ADRA2B	ENST00000409345	p.Q154H	c.462G>T	Verified	GAC	CAG	GGC	3	G	T
TCGA-23-1120	AFF2	ENST00000370460	p.D1073Y	c.3217G>T	Verified	GCT	GAT	TAT	1	G	T
TCGA-13-0714	AFM	ENST00000226355	p.G225W	c.673G>T	Verified	TGT	GGG	GCA	1	G	T
TCGA-61-1910	AGBL1	ENST00000421325	p.?	c.1849+1G>T	Unverified	TAT	GGG	ATG	1	G	T
TCGA-29-1761	AGBL1	ENST00000421325	p.S400I	c.1199G>T	Unverified	AAT	AGT	CTC	2	G	T
TCGA-13-0886	AHCTF1	ENST00000391829	p.A1182S	c.3544G>T	Verified	AAA	GCT	AAA	1	G	T
TCGA-29-1766	AHNAK2_EN	ENST00000333244	p.D3694Y	c.11080G>T	Unverified	GCC	GAC	CTG	1	G	T
TCGA-24-1431	AIFM3	ENST00000399167	p.G217V	c.650G>T	Unverified	GAG	GGC	TTC	2	G	T
TCGA-23-1124	AKAP13	ENST00000361243	p.S1750I	c.5249G>T	Verified	gtc	agt	cgt	2	G	T
TCGA-04-1342	AKAP8	ENST00000269701	p.R206L	c.617G>T	Unverified	atg	cgc	agc	2	G	T
TCGA-13-0887	AKAP9	NM_147171.1	p.E3156*	c.9466G>T	Verified	cag	gag	cag	1	G	T

TCGA-13-0887	AKAP9_ENST	ENST00000356239	p.E3152*	c.9454G>T	Verified	CAG	GAG	CAG	1	G	T
TCGA-24-0979	AKR7A3	ENST00000361640	p.A258S	c.772G>T	Unverified	GGC	GCC	AGC	1	G	T
TCGA-24-1845	ALDH1L1	ENST00000393434	p.V860F	c.2578G>T	Unverified	TTT	GTC	AAC	1	G	T
TCGA-24-1422	ALG10B	ENST00000308742	p.K215N	c.645G>T	Unverified	CAA	AAG	AAG	3	G	T
TCGA-13-1510	ALMS1	ENST00000264448	p.L214L	c.642G>T	Unverified	CCA	CTG	CTA	3	G	T
TCGA-13-1510	ALOX15	ENST00000570836	p.G117C	c.349G>T	Verified	gtg	ggc	gag	1	G	T
TCGA-24-1846	ALOX15	ENST00000570836	p.D199Y	c.595G>T	Unverified	aag	gat	cta	1	G	T
TCGA-29-1761	ALPK2_ENST	ENST00000361673	p.V417F	c.1249G>T	Unverified	AGA	GTC	TCC	1	G	T
TCGA-25-1318	AMICA1	ENST00000292067	p.D201Y	c.601G>T	Verified	GGG	GAC	ATT	1	G	T
TCGA-25-1318	AMICA1	ENST00000292067	p.D201Y	c.601G>T	Verified	GGG	GAC	ATT	1	G	T
TCGA-29-1702	AMOTL1	ENST00000433060	p.V85L	c.253G>T	Unverified	GAG	GTG	GAA	1	G	T
TCGA-23-1124	AMY2A	ENST00000414303	p.G435V	c.1304G>T	Verified	TTT	GGG	AGA	2	G	T
TCGA-30-1857	ANAPC1	ENST00000341068	p.A1214S	c.3640G>T	Unverified	TCT	GCT	GCA	1	G	T
TCGA-25-2401	ANAPC2	ENST00000323927	p.K625N	c.1875G>T	Verified	aag	aag	tat	3	G	T
TCGA-29-1763	ANK2	ENST00000357077	p.D1558Y	c.4672G>T	Unverified	GAG	GAC	TTA	1	G	T
TCGA-25-2042	ANKRD20A2	ENST00000377601	p.Q35H	c.105G>T	Unverified	CTG	CAG	AAG	3	G	T
TCGA-61-1914	ANO6	ENST00000320560	p.M749I	c.2247G>T	Unverified	GAC	ATG	ATC	3	G	T
TCGA-61-1914	ANO6_ENST	ENST00000425752	p.M749I	c.2247G>T	Unverified	GAC	ATG	ATC	3	G	T
TCGA-23-1120	ANXA11	ENST00000372234	p.G38G	c.114G>T	Unverified	ATC	GGG	CTG	3	G	T
TCGA-13-0792	AP3B2	ENST00000261722	p.E717D	c.2151G>T	Verified	AGT	GAG	TCC	3	G	T
TCGA-24-1422	AP4B1	ENST00000369569	p.R512R	c.1536G>T	Verified	GTA	CGG	GAC	3	G	T
TCGA-29-1691	AP4E1	ENST00000261842	p.A371S	c.1111G>T	Unverified	CTG	GCT	CTT	1	G	T
TCGA-29-1781	APC	ENST00000457016	p.S2794I	c.8381G>T	Unverified	AGC	AGC	GCA	2	G	T
TCGA-04-1347	APP	ENST00000346798	p.L28L	c.84G>T	Verified	GGC	CTG	CTG	3	G	T
TCGA-29-1695	AQP2	ENST00000199280	p.E3*	c.7G>T	Unverified	TGG	GAG	CTC	1	G	T
TCGA-25-1326	ARG1	ENST00000368087	p.V24L	c.70G>T	Verified	GGG	GTG	GAA	1	G	T
TCGA-20-1685	ARG2	ENST00000261783	p.E205*	c.613G>T	Unverified	CCT	GAA	CAT	1	G	T
TCGA-23-1110	ARHGAP15	ENST00000295095	p.V162L	c.484G>T	Verified	ACA	GTA	TCA	1	G	T
TCGA-24-2280	ARHGAP32	ENST00000392657	p.V675F	c.2023G>T	Unverified	CCT	GTC	AGT	1	G	T
TCGA-24-2280	ARHGAP32_	ENST00000310343	p.V1024F	c.3070G>T	Unverified	CCT	GTC	AGT	1	G	T
TCGA-13-1481	ARHGAP5	ENST00000345122	p.V224L	c.670G>T	Verified	CTT	GTA	GTG	1	G	T
TCGA-30-1891	ARHGAP9	NM_032496.1	p.W11C	c.33G>T	Unverified	tcc	tgg	ggg	3	G	T
TCGA-29-1784	ARHGEF11	ENST00000368194	p.E1188D	c.3564G>T	Unverified	cta	gag	gac	3	G	T
TCGA-29-1770	ARHGEF15	ENST00000361926	p.V425V	c.1275G>T	Unverified	gag	gtg	gtg	3	G	T

TCGA-24-1563	ARHGEF18	ENST00000319670	p.M405I	c.1215G>T	Verified	gcg	atg	ttt	3	G	T
TCGA-29-1703	ARHGEF38	ENST00000265154	p.V168L	c.502G>T	Unverified	CAA	GTA	ATT	1	G	T
TCGA-29-1703	ARHGEF38_	ENST00000420470	p.V168L	c.502G>T	Unverified	CAA	GTA	ATT	1	G	T
TCGA-20-1687	ARHGEF40_	ENST00000298694	p.G359*	c.1075G>T	Unverified	GGA	GGA	GGA	1	G	T
TCGA-13-0760	ARHGEF9	XM_377014.1	p.W190C	c.570G>T	Verified	ttc	tgg	ata	3	G	T
TCGA-24-2267	ARL11	ENST00000282026	p.V16L	c.46G>T	Unverified	GTG	GTG	ATG	1	G	T
TCGA-23-1122	ARL13A	XM_373358.3	p.G107W	c.319G>T	Verified	cat	ggg	ctt	1	G	T
TCGA-04-1362	ARL15	ENST00000332313	p.V48F	c.142G>T	Verified	AAC	GTC	GTG	1	G	T
TCGA-04-1530	ARL8A	ENST00000272217	p.?	c.373-1G>T	Verified	ccg	gtc	tta	1	G	T
TCGA-04-1338	ARMC4	ENST00000305242	p.D1019Y	c.3055G>T	Verified	CAG	GAT	CTC	1	G	T
TCGA-29-1768	ARPC1B	ENST00000252725	p.R97R	c.291G>T	Unverified	AAC	CGG	GCT	3	G	T
TCGA-13-1497	ARSI	ENST00000328668	p.G534W	c.1600G>T	Verified	GAA	GGG	AGG	1	G	T
TCGA-09-1665	ASAP3	ENST00000336689	p.A679A	c.2037G>T	Verified	CAG	GCG	GGG	3	G	T
TCGA-23-1118	ASNSD1	ENST00000260952	p.G329C	c.985G>T	Verified	GGG	GGC	ATT	1	G	T
TCGA-61-1915	ASNSD1	ENST00000260952	p.E66*	c.196G>T	Unverified	GTG	GAA	GAT	1	G	T
TCGA-24-1845	ASPA	ENST00000263080	p.D66Y	c.196G>T	Unverified	ATT	GAC	TGT	1	G	T
TCGA-13-0900	ATG9B	ENST00000397266	p.P254P	c.762G>T	Unverified	GGG	CCG	TTC	3	G	T
TCGA-24-1423	ATG9B	ENST00000397266	p.W718C	c.2154G>T	Unverified	CTC	TGG	CGC	3	G	T
TCGA-24-2267	ATP10A	ENST00000356865	p.A1211S	c.3631G>T	Verified	ATC	GCG	CTG	1	G	T
TCGA-61-2012	ATP11B	ENST00000323116	p.A846S	c.2536G>T	Verified	CAG	GCT	GCA	1	G	T
TCGA-13-0887	ATP11B	ENST00000323116	p.K532N	c.1596G>T	Verified	GAA	AAG	GCT	3	G	T
TCGA-20-1685	ATP11C	ENST00000327569	p.G521*	c.1561G>T	Unverified	AAT	GGA	TAT	1	G	T
TCGA-24-1422	ATP11C	ENST00000327569	p.L1132F	c.3396G>T	Verified	GTA	TTG	TAA	3	G	T
TCGA-20-1685	ATP11C_EN!	ENST00000361648	p.G521*	c.1561G>T	Unverified	AAT	GGA	TAT	1	G	T
TCGA-61-2008	ATP12A	ENST00000381946	p.D587Y	c.1759G>T	Verified	TTT	GAC	ATA	1	G	T
TCGA-13-0755	ATP13A3	ENST00000256031	p.A774S	c.2320G>T	Verified	ATT	GCT	GAA	1	G	T
TCGA-13-0906	ATP13A4	ENST00000342695	p.V133V	c.399G>T	Verified	AAA	GTG	CAG	3	G	T
TCGA-13-1501	ATP1A3	ENST00000302102	p.Q920H	c.2760G>T	Verified	GTC	CAG	TGG	3	G	T
TCGA-13-1501	ATP1A3	ENST00000302102	p.Q920H	c.2760G>T	Verified	GTC	CAG	TGG	3	G	T
TCGA-24-1463	ATP2B2	ENST00000383800	p.E1131*	c.3391G>T	Unverified	ATC	GAA	GAT	1	G	T
TCGA-04-1542	ATP5EP2	ENST00000381026	p.V46L	c.136G>T	Verified	ATT	GTG	AAA	1	G	T
TCGA-13-0904	ATP8A2	ENST00000381655	p.E499D	c.1497G>T	Verified	CAG	GAG	TTC	3	G	T
TCGA-04-1338	ATP8B1	ENST00000283684	p.G1001W	c.3001G>T	Verified	ATG	GGG	CTG	1	G	T
TCGA-24-2289	ATRNL1	ENST00000355044	p.G646V	c.1937G>T	Unverified	TCT	GGG	AAT	2	G	T

TCGA-61-2109	AWAT2	ENST00000276101	p.G239V	c.716G>T	Unverified	cct	ggt	ggc	2	G	T
TCGA-13-0903	AZI1	ENST00000374782	p.A327S	c.979G>T	Verified	AAG	GCC	CGG	1	G	T
TCGA-61-2102	B3GALT2	ENST00000367434	p.R336L	c.1007G>T	Verified	CGC	CGT	TTG	2	G	T
TCGA-24-1470	B3GALT4	ENST00000451237	p.G29V	c.86G>T	Verified	TTG	GGG	GAG	2	G	T
TCGA-13-0904	BAI1	NM_001702.1	p.R1473R	c.4419G>T	Unverified	cgg	cgg	aag	3	G	T
TCGA-23-1031	BARX2	ENST00000281437	p.E120D	c.360G>T	Unverified	AGC	GAG	TCA	3	G	T
TCGA-13-0791	BBS9	ENST00000242067	p.E375*	c.1123G>T	Verified	CGA	GAA	CTA	1	G	T
TCGA-13-0905	BBX	ENST00000415149	p.G737G	c.2211G>T	Verified	AGT	GGG	GAT	3	G	T
TCGA-13-0904	BCHE	ENST00000264381	p.G311V	c.932G>T	Verified	tat	ggg	act	2	G	T
TCGA-13-1498	BCL9	NM_004326.1	p.S890S	c.2670G>T	Verified	cca	tcg	cag	3	G	T
TCGA-61-1900	BCORL1	ENST00000218147	p.A325S	c.973G>T	Unverified	tca	gct	ccg	1	G	T
TCGA-61-2008	BDH1	ENST00000392378	p.G26V	c.77G>T	Verified	AAT	GGA	GCA	2	G	T
TCGA-23-1021	BMS1	ENST00000374518	p.G402W	c.1204G>T	Verified	CTT	GGG	TCA	1	G	T
TCGA-61-2008	BPTF	ENST00000306378	p.K1250N	c.3750G>T	Verified	TCC	AAG	AGT	3	G	T
TCGA-24-1847	BRCA1	ENST00000357654	p.E111*	c.331G>T	Unverified	aag	gaa	aat	1	G	T
TCGA-29-1762	BRCA2	ENST00000380152	p.E2906*	c.8716G>T	Unverified	TAT	GAA	GCA	1	G	T
TCGA-29-1762	BRCA2_ENST	ENST00000544455	p.E2906*	c.8716G>T	Unverified	TAT	GAA	GCA	1	G	T
TCGA-24-1847	BRD8	ENST00000254900	p.V698F	c.2092G>T	Unverified	TCT	GTC	TGT	1	G	T
TCGA-24-1847	BRD8_ENST(	ENST00000230901	p.V771F	c.2311G>T	Unverified	TCT	GTC	TGT	1	G	T
TCGA-13-1510	BRD9	ENST00000323510	p.D437Y	c.1309G>T	Unverified	CAG	GAC	CTG	1	G	T
TCGA-24-1474	BRSK1_ENST	ENST00000309383	p.D383Y	c.1147G>T	Unverified	GTG	GAT	TCT	1	G	T
TCGA-10-0930	BRWD1	ENST00000342449	p.W395C	c.1185G>T	Verified	ATT	TGG	AGA	3	G	T
TCGA-10-0930	BRWD1	ENST00000342449	p.W395C	c.1185G>T	Verified	ATT	TGG	AGA	3	G	T
TCGA-23-1124	BSN	ENST00000296452	p.E3557D	c.10671G>T	Verified	GAG	GAG	GGC	3	G	T
TCGA-13-0904	BTBD8	ENST00000342818	p.K136N	c.408G>T	Verified	AAA	AAG	ATA	3	G	T
TCGA-24-1104	C10orf140	ENST00000449193	p.G862G	c.2586G>T	Verified	GAT	GGG	GAT	3	G	T
TCGA-24-2262	C10orf140_E	ENST00000449193	p.K831N	c.2493G>T	Verified	AAA	AAG	GTA	3	G	T
TCGA-04-1338	C11orf30	ENST00000334736	p.V220F	c.658G>T	Verified	GAA	GTT	CCA	1	G	T
TCGA-29-1698	C11orf49	ENST00000378615	p.R6L	c.17G>T	Unverified	GAG	CGC	CTA	2	G	T
TCGA-29-1698	C11orf49_EI	ENST00000378618	p.R6L	c.17G>T	Unverified	GAG	CGC	CTA	2	G	T
TCGA-24-1844	C11orf54	ENST00000528288	p.E61*	c.181G>T	Unverified	GCA	GAA	GTT	1	G	T
TCGA-24-1474	C11orf94	ENST00000449465	p.D59Y	c.175G>T	Unverified	GAT	GAC	TAT	1	G	T
TCGA-61-1910	C12orf23	ENST00000280756	p.V102L	c.304G>T	Unverified	GTT	GTA	AAC	1	G	T
TCGA-23-1031	C12orf40	ENST00000324616	p.S366I	c.1097G>T	Verified	ACA	AGT	TAT	2	G	T

TCGA-23-1809	C12orf51	ENST00000377560	p.?	c.9514-1G>T	Unverified	ATC	GGA	GGC	1	G	T
TCGA-04-1331	C12orf51_EI	ENST00000377560_v62	p.E3835D	c.11505G>T	Unverified	CCT	GAG	ATC	3	G	T
TCGA-13-1499	C14orf178	ENST00000355883	p.A120S	c.358G>T	Verified	GAT	GCC	ATG	1	G	T
TCGA-29-1762	C14orf79	ENST00000547315	p.R198I	c.593G>T	Unverified	TGG	AGA	GCC	2	G	T
TCGA-61-1998	C15orf2	ENST00000329468	p.R107S	c.321G>T	Verified	CCG	AGG	TTT	3	G	T
TCGA-23-1122	C15orf42	ENST00000268138	p.E1598*	c.4792G>T	Verified	GGA	GAA	GAG	1	G	T
TCGA-13-0883	C15orf55	ENST00000333756	p.G807*	c.2419G>T	Verified	CCA	GGA	CCT	1	G	T
TCGA-61-1900	C16orf73	ENST00000325962	p.G160V	c.479G>T	Unverified	TTG	GGC	TGC	2	G	T
TCGA-61-1900	C16orf73_EI	ENST00000412554	p.G404V	c.1211G>T	Unverified	TTG	GGC	TGC	2	G	T
TCGA-23-1110	C16orf91	ENST00000310355	p.G23G	c.69G>T	Unverified	ACA	GGG	ACA	3	G	T
TCGA-24-1844	C18orf26	ENST00000321600	p.E12*	c.34G>T	Unverified	ATT	GAA	AAA	1	G	T
TCGA-24-1849	C1orf101	ENST00000366533	p.C71F	c.212G>T	Unverified	CGT	TGT	TCC	2	G	T
TCGA-24-1849	C1orf101_EI	ENST00000366534	p.C71F	c.212G>T	Unverified	CGT	TGT	TCC	2	G	T
TCGA-23-1122	C1orf106	ENST00000367342	p.G271G	c.813G>T	Verified	GAT	GGG	CTC	3	G	T
TCGA-23-1114	C1orf187	ENST00000294485	p.?	c.757+1G>T	Unverified	AAA	GAG	AAA	1	G	T
TCGA-25-2392	C1orf50	ENST00000372525	p.D98Y	c.292G>T	Verified	GAA	GAT	GCT	1	G	T
TCGA-61-1914	C1orf87	ENST00000371201	p.L391F	c.1173G>T	Unverified	GAT	TTG	TTA	3	G	T
TCGA-13-0884	C20orf152	ENST00000349339	p.D102Y	c.304G>T	Unverified	GAG	GAT	GAG	1	G	T
TCGA-29-1761	C20orf194	ENST00000252032	p.?	c.1468+1G>T	Unverified	AAG	GAT	GGC	1	G	T
TCGA-13-1489	C21orf62	ENST00000290186	p.M60I	c.180G>T	Verified	CTG	ATG	TGC	3	G	T
TCGA-24-1103	C21orf63	ENST00000300255	p.S402I	c.1205G>T	Verified	TAC	AGT	TCC	2	G	T
TCGA-04-1530	C2orf18	ENST00000344420	p.A211S	c.631G>T	Unverified	CGG	GCA	GTT	1	G	T
TCGA-29-1691	C2orf28	ENST00000380171	p.E222D	c.666G>T	Unverified	CCT	GAG	AAT	3	G	T
TCGA-29-1711	C2orf29	ENST00000289382	p.A331S	c.991G>T	Unverified	ATG	GCC	AAA	1	G	T
TCGA-29-1711	C2orf29	ENST00000289382	p.A331S	c.991G>T	Unverified	ATG	GCC	AAA	1	G	T
TCGA-25-2042	C2orf57	ENST00000313965	p.V73F	c.217G>T	Verified	GCA	GTT	GTT	1	G	T
TCGA-13-1505	C3orf20	ENST00000253697	p.G794W	c.2380G>T	Verified	TTT	GGG	GGC	1	G	T
TCGA-29-1781	C3orf20	ENST00000253697	p.R522L	c.1565G>T	Unverified	AAA	CGG	CTG	2	G	T
TCGA-61-2009	C3orf30	ENST00000295622	p.G88C	c.262G>T	Verified	GCT	GGC	CGC	1	G	T
TCGA-13-0919	C3orf32	ENST00000341795	p.R120I	c.359G>T	Unverified	caa	aga	ggc	2	G	T
TCGA-25-2401	C3orf35	ENST00000328376	p.G114V	c.341G>T	Unverified	CGT	GGA	ATC	2	G	T
TCGA-23-1117	C4BPB	ENST00000367076	p.S161I	c.482G>T	Verified	ATT	AGT	TAT	2	G	T
TCGA-23-1117	C4BPB	ENST00000367076	p.S161I	c.482G>T	Verified	ATT	AGT	TAT	2	G	T
TCGA-13-1498	C5AR1	ENST00000355085	p.L323F	c.969G>T	Verified	GTG	TTG	ACT	3	G	T

TCGA-04-1336	C6orf106	ENST00000374023	p.T196T	c.588G>T	Verified	GAA	ACG	GAG	3	G	T
TCGA-04-1338	C6orf81	ENST00000288065	p.V278L	c.832G>T	Unverified	GAG	GTA	CTG	1	G	T
TCGA-23-1123	C7orf42	ENST00000341567	p.G128V	c.383G>T	Verified	GGA	GGG	TAT	2	G	T
TCGA-23-1123	C7orf42	ENST00000341567	p.G128V	c.383G>T	Verified	GGA	GGG	TAT	2	G	T
TCGA-13-0885	C7orf49	ENST00000361743	p.A57S	c.169G>T	Verified	CTG	GCG	GGG	1	G	T
TCGA-24-2280	C8orf45	ENST00000396592	p.E429*	c.1285G>T	Unverified	CTG	GAG	AGC	1	G	T
TCGA-04-1338	C8orf76	ENST00000276704	p.R359I	c.1076G>T	Verified	ttc	aga	aag	2	G	T
TCGA-25-1313	C9orf21	ENST00000375234	p.?	c.315+1G>T	Unverified	ATT	GAG	CCT	3	G	T
TCGA-24-2280	C9orf7	ENST00000316948	p.L140L	c.420G>T	Unverified	GCT	CTG	GGC	3	G	T
TCGA-13-0807	C9orf84	ENST00000394779	p.K677N	c.2031G>T	Verified	AAA	AAG	CCT	3	G	T
TCGA-13-0904	CA5A	ENST00000309893	p.E250D	c.750G>T	Unverified	AAG	GAG	CCC	3	G	T
TCGA-25-1313	CA9	ENST00000378357	p.S352I	c.1055G>T	Verified	CTG	AGT	GCT	2	G	T
TCGA-13-1497	CACNA1A_E	ENST00000357018	p.W597L	c.1790G>T	Verified	TAC	TGG	GCA	2	G	T
TCGA-13-0887	CACNA1C	ENST00000402845	p.A1777S	c.5329G>T	Verified	AGG	GCC	GGT	1	G	T
TCGA-13-0762	CACNA1C	ENST00000402845	p.R1407L	c.4220G>T	Verified	TTC	CGC	CTG	2	G	T
TCGA-13-0762	CACNA1C	ENST00000402845	p.R1407L	c.4220G>T	Verified	TTC	CGC	CTG	2	G	T
TCGA-13-1489	CACNA1D	ENST00000288139	p.G29V	c.86G>T	Verified	AGA	GGC	ACC	2	G	T
TCGA-24-2289	CACNA1F	ENST00000376265	p.A1504S	c.4510G>T	Unverified	gtg	gcc	tgc	1	G	T
TCGA-25-2400	CACNA1H_E	ENST00000358590	p.G1321V	c.3962G>T	Unverified	CCC	GGC	AGC	2	G	T
TCGA-24-2289	CACNA1S	ENST00000362061	p.S506I	c.1517G>T	Unverified	TGT	AGC	GGT	2	G	T
TCGA-29-1763	CACNA2D1	ENST00000356860	p.A913S	c.2737G>T	Unverified	GTA	GCA	GAC	1	G	T
TCGA-23-2078	CACNA2D1	ENST00000356860	p.S936I	c.2807G>T	Verified	TTG	AGT	TTG	2	G	T
TCGA-13-1498	CADM1	ENST00000452722	p.V383L	c.1147G>T	Verified	GCG	GTG	GTG	1	G	T
TCGA-61-2113	CADPS2	ENST00000449022	p.M828I	c.2484G>T	Unverified	ACC	ATG	AAC	3	G	T
TCGA-10-0930	CAGE1_ENS	ENST00000338150	p.V435L	c.1303G>T	Verified	TCT	GTA	AGT	1	G	T
TCGA-10-0930	CAGE1_ENS	ENST00000338150	p.V435L	c.1303G>T	Verified	TCT	GTA	AGT	1	G	T
TCGA-24-1846	CALCOCO1	ENST00000550804	p.E132*	c.394G>T	Unverified	GAG	GAG	GCT	1	G	T
TCGA-25-1326	CAMSAP1	ENST00000389532	p.V1594V	c.4782G>T	Unverified	GCA	GTG	CCA	3	G	T
TCGA-13-0906	CAMSAP1L1	ENST00000358823	p.R754M	c.2261G>T	Verified	CTT	AGG	ATG	2	G	T
TCGA-29-1696	CAMSAP3	ENST00000446248	p.C1194F	c.3581G>T	Unverified	AGC	TGC	CAG	2	G	T
TCGA-29-1696	CAMSAP3	ENST00000446248	p.C1194F	c.3581G>T	Unverified	AGC	TGC	CAG	2	G	T
TCGA-29-1696	CAMSAP3_E	ENST00000446248_v68	p.C1194F	c.3581G>T	Unverified	AGC	TGC	CAG	2	G	T
TCGA-29-1696	CAMSAP3_E	ENST00000446248_v68	p.C1194F	c.3581G>T	Unverified	AGC	TGC	CAG	2	G	T
TCGA-13-0885	CAP2	ENST00000229922	p.G368V	c.1103G>T	Verified	AAA	GGG	AAA	2	G	T

TCGA-24-1422	CARD6	ENST00000254691	p.V218F	c.652G>T	Verified	TCT	GTT	GAC	1	G	T
TCGA-20-0990	CARS	ENST00000397111	p.G626V	c.1877G>T	Verified	CTT	GGG	GTG	2	G	T
TCGA-29-1777	CASC1	ENST00000320267	p.V445L	c.1333G>T	Unverified	AAT	GTA	ATC	1	G	T
TCGA-25-1318	CASC1	ENST00000320267	p.E238D	c.714G>T	Verified	TTT	GAG	ATT	3	G	T
TCGA-25-1318	CASC1	ENST00000320267	p.E238D	c.714G>T	Verified	TTT	GAG	ATT	3	G	T
TCGA-29-1777	CASC1_ENST	ENST00000354189	p.V509L	c.1525G>T	Unverified	AAT	GTA	ATC	1	G	T
TCGA-13-0924	CASK	ENST00000378166	p.E414*	c.1240G>T	Verified	TTG	GAA	GAA	1	G	T
TCGA-13-0762	CASP7	ENST00000369321	p.V324L	c.970G>T	Verified	tgt	gtg	gtc	1	G	T
TCGA-13-0762	CASP7	ENST00000369321	p.V324L	c.970G>T	Verified	tgt	gtg	gtc	1	G	T
TCGA-29-1781	CASR	ENST00000296154	p.C585F	c.1754G>T	Unverified	AAG	TGC	CCA	2	G	T
TCGA-29-1763	CATSPERG	ENST00000312265	p.V675L	c.2023G>T	Unverified	TTG	GTG	AGC	1	G	T
TCGA-13-0924	CBLN4	ENST00000064571	p.D51Y	c.151G>T	Verified	acg	gac	tcc	1	G	T
TCGA-29-1764	CBWD1	ENST00000356521	p.S68I	c.203G>T	Unverified	CAT	AGT	AAA	2	G	T
TCGA-29-1698	CCDC108	ENST00000341552	p.E157D	c.471G>T	Unverified	TGG	GAG	CTA	3	G	T
TCGA-29-1698	CCDC108_EI	ENST00000295729	p.E92D	c.276G>T	Unverified	TGG	GAG	CTA	3	G	T
TCGA-24-1422	CCDC13	ENST00000310232	p.E52D	c.156G>T	Verified	TTG	GAG	GTT	3	G	T
TCGA-61-1899	CCDC141	ENST00000343876	p.G684V	c.2051G>T	Unverified	CCA	GGG	GAT	2	G	T
TCGA-04-1338	CCDC141	ENST00000343876	p.R494S	c.1482G>T	Verified	GAA	AGG	ATT	3	G	T
TCGA-61-1899	CCDC141_EI	ENST00000420890	p.G1259V	c.3776G>T	Unverified	CCA	GGG	GAT	2	G	T
TCGA-30-1891	CCDC147	ENST00000369704	p.A508S	c.1522G>T	Unverified	GAG	GCT	CAG	1	G	T
TCGA-23-1123	CCDC148	ENST00000283233	p.V475L	c.1423G>T	Verified	GAA	GTG	GCC	1	G	T
TCGA-23-1123	CCDC148	ENST00000283233	p.V475L	c.1423G>T	Verified	GAA	GTG	GCC	1	G	T
TCGA-29-1763	CCDC155	ENST00000447857	p.Q507H	c.1521G>T	Unverified	GGC	CAG	CTC	3	G	T
TCGA-04-1343	CCDC165	ENST00000359865	p.C1504F	c.4511G>T	Verified	ATG	TGC	AGG	2	G	T
TCGA-04-1343	CCDC165	ENST00000359865	p.G1508V	c.4523G>T	Verified	GAA	GGG	GGA	2	G	T
TCGA-13-0887	CCDC39	ENST00000273654	p.E268*	c.802G>T	Verified	TTG	GAA	TGT	1	G	T
TCGA-13-1499	CCDC42	ENST00000293845	p.K92N	c.276G>T	Verified	CAG	AAG	TCT	3	G	T
TCGA-61-1910	CCDC88B	ENST00000356786	p.R64R	c.192G>T	Unverified	CTC	CGG	GTG	3	G	T
TCGA-24-1847	CCIN	ENST00000335119	p.L139F	c.417G>T	Unverified	TTC	TTG	GCT	3	G	T
TCGA-24-1844	CCL11	ENST00000305869	p.G21G	c.63G>T	Unverified	CAG	GGG	CTC	3	G	T
TCGA-13-1488	CCNB3	ENST00000376042	p.K233N	c.699G>T	Verified	AAG	AAG	AAG	3	G	T
TCGA-13-1488	CCNB3_ENST	ENST00000376042	p.K233N	c.699G>T	Verified	AAG	AAG	AAG	3	G	T
TCGA-09-2050	CCR3	ENST00000357422	p.V56L	c.166G>T	Verified	gtg	gtg	atg	1	G	T
TCGA-30-1714	CCR9	ENST00000357632	p.K245N	c.735G>T	Unverified	gcc	aag	aag	3	G	T

TCGA-13-1498	CD1B	ENST00000368168	p.W295L	c.884G>T	Verified	TAC	TGG	AGA	2	G	T
TCGA-09-0369	CD1C	ENST00000368170	p.G252C	c.754G>T	Verified	CAT	GGT	GAT	1	G	T
TCGA-09-0369	CD1C	ENST00000368170	p.G252C	c.754G>T	Verified	CAT	GGT	GAT	1	G	T
TCGA-29-1696	CD226	ENST00000280200	p.W204L	c.611G>T	Unverified	AGG	TGG	AGC	2	G	T
TCGA-29-1696	CD226	ENST00000280200	p.W204L	c.611G>T	Unverified	AGG	TGG	AGC	2	G	T
TCGA-23-1122	CD300A	ENST00000360141	p.A138A	c.414G>T	Verified	ACT	GCG	GCC	3	G	T
TCGA-24-0975	CD4	ENST00000011653	p.W359C	c.1077G>T	Unverified	GTG	TGG	GTG	3	G	T
TCGA-61-1913	CD40	ENST00000372285	p.V218L	c.652G>T	Unverified	aag	gtg	gcc	1	G	T
TCGA-09-2056	CD5L	ENST00000368174	p.R298L	c.893G>T	Verified	GAC	CGG	AAA	2	G	T
TCGA-29-1691	CD80	ENST00000264246	p.D205Y	c.613G>T	Unverified	CTG	GAT	TTC	1	G	T
TCGA-61-1900	CD93	ENST00000246006	p.G113V	c.338G>T	Unverified	AAG	GGC	TTC	2	G	T
TCGA-23-1117	CDC6	ENST00000209728	p.R445S	c.1335G>T	Verified	aac	agg	atg	3	G	T
TCGA-23-1117	CDC6	ENST00000209728	p.R445S	c.1335G>T	Verified	aac	agg	atg	3	G	T
TCGA-29-1770	CDCA8	ENST00000373055	p.M122I	c.366G>T	Unverified	GAA	ATG	ATA	3	G	T
TCGA-24-1470	CDH10	ENST00000264463	p.D324Y	c.970G>T	Verified	AAG	GAC	ACA	1	G	T
TCGA-24-1435	CDH17	ENST00000027335	p.S484I	c.1451G>T	Verified	GGG	AGT	TCT	2	G	T
TCGA-61-2094	CDH7	ENST00000323011	p.V499F	c.1495G>T	Unverified	CAG	GTT	ATC	1	G	T
TCGA-61-2094	CDH7_ENST	ENST00000323011	p.V499F	c.1495G>T	Unverified	CAG	GTT	ATC	1	G	T
TCGA-23-1117	CDH8	ENST00000577390	p.P389P	c.1167G>T	Verified	CCT	CCG	GTC	3	G	T
TCGA-23-1117	CDH8	ENST00000577390	p.P389P	c.1167G>T	Verified	CCT	CCG	GTC	3	G	T
TCGA-13-0906	CDHR2	ENST00000261944	p.S83I	c.248G>T	Verified	GCC	AGC	GCT	2	G	T
TCGA-25-2392	CDK12	NM_016507	p.R882L	c.2645G>T	Verified	gct	cgg	ctc	2	G	T
TCGA-23-1110	CDK13	ENST00000181839	p.G1050C	c.3148G>T	Verified	CTG	GGC	TTG	1	G	T
TCGA-29-1781	CDK13	ENST00000181839	p.L884L	c.2652G>T	Unverified	GAA	CTG	CTA	3	G	T
TCGA-13-0807	CDON	ENST00000263577	p.M655I	c.1965G>T	Verified	TTG	ATG	GTA	3	G	T
TCGA-25-2392	CECR1	ENST00000399837	p.E234D	c.702G>T	Unverified	CAG	GAG	TTC	3	G	T
TCGA-09-1674	CELA2A	ENST00000359621	p.V30F	c.88G>T	Unverified	GTG	GTT	GGC	1	G	T
TCGA-29-1690	CELA3A	ENST00000290122	p.G243V	c.728G>T	Unverified	TTT	GGC	TGC	2	G	T
TCGA-13-1510	CELSR3	ENST00000164024	p.A2235S	c.6703G>T	Unverified	CTG	GCC	CAC	1	G	T
TCGA-04-1367	CELSR3	ENST00000164024	p.L897L	c.2691G>T	Verified	CTC	CTG	GAG	3	G	T
TCGA-29-1699	CENPN_ENS	ENST00000393335	p.P335P	c.1005G>T	Unverified	GAC	CCG	AGC	3	G	T
TCGA-04-1347	CEP135	ENST00000257287	p.R924I	c.2771G>T	Verified	GAA	AGA	GTG	2	G	T
TCGA-13-0791	CEP290	ENST00000552810	p.S1594I	c.4781G>T	Verified	GAT	AGT	TCA	2	G	T
TCGA-23-1111	CEP350	ENST00000367607	p.E2234*	c.6700G>T	Unverified	AAA	GAA	TTA	1	G	T

TCGA-23-1111	CEP350_ENS	ENST00000367607	p.E2234*	c.6700G>T	Unverified	AAA	GAA	TTA	1	G	T
TCGA-04-1347	CES1_ENST0	ENST00000360526	p.G143G	c.429G>T	Verified	GGA	GGG	GGG	3	G	T
TCGA-13-1488	CES3	ENST00000303334	p.D219Y	c.655G>T	Verified	GGT	GAC	CTC	1	G	T
TCGA-29-1703	CFH	ENST00000367429	p.G967W	c.2899G>T	Unverified	gat	ggg	cct	1	G	T
TCGA-29-1761	CFH	ENST00000367429	p.W1096C	c.3288G>T	Unverified	aac	tgg	acg	3	G	T
TCGA-13-0760	CHAF1B	ENST00000314103	p.D38Y	c.112G>T	Verified	GTG	GAC	ACC	1	G	T
TCGA-13-0887	CHD1	ENST00000284049	p.K204N	c.612G>T	Verified	aag	aag	att	3	G	T
TCGA-61-1895	CHD3	ENST00000330494	p.R170S	c.510G>T	Unverified	ATG	AGG	CCC	3	G	T
TCGA-61-2109	CHD4_ENST	ENST00000309577	p.G1042C	c.3124G>T	Unverified	GAT	GGC	AGT	1	G	T
TCGA-25-2042	CHD4_ENST	ENST00000309577	p.R1434L	c.4301G>T	Verified	GCC	CGT	GTT	2	G	T
TCGA-36-1577	CHD4_ENST	ENST00000309577	p.E698D	c.2094G>T	Unverified	TTG	GAG	AGG	3	G	T
TCGA-24-2289	CHI3L1	ENST00000255409	p.G248G	c.744G>T	Unverified	CTG	GGG	GCT	3	G	T
TCGA-61-1900	CHI3L2	ENST00000369748	p.G250V	c.749G>T	Unverified	GTG	GGG	TAC	2	G	T
TCGA-24-1469	CHRM2	ENST00000320658	p.R387S	c.1161G>T	Verified	ACC	AGG	ACA	3	G	T
TCGA-20-1687	CHRNA3	ENST00000326828	p.W341C	c.1023G>T	Unverified	TCA	TGG	GTG	3	G	T
TCGA-13-0887	CILP	ENST00000261883	p.L74L	c.222G>T	Verified	CGG	CTG	GAC	3	G	T
TCGA-29-1763	CKAP4	ENST00000378026	p.S396S	c.1188G>T	Unverified	CAC	TCG	GAA	3	G	T
TCGA-09-2049	CLCN1	ENST00000343257	p.G222C	c.664G>T	Verified	GCG	GGC	CTG	1	G	T
TCGA-13-0913	CLCN5	ENST00000376108	p.L719F	c.2157G>T	Verified	cga	ttg	ctt	3	G	T
TCGA-13-0807	CLDN10	ENST00000376873	p.R62L	c.185G>T	Verified	TGC	CGA	CCG	2	G	T
TCGA-23-1029	CLDN17	ENST00000286808	p.G120*	c.358G>T	Unverified	CTG	GGA	ACT	1	G	T
TCGA-24-1469	CLDN7	ENST00000360325	p.V113V	c.339G>T	Verified	AAA	GTG	AAG	3	G	T
TCGA-13-1497	CLEC14A	ENST00000342213	p.G254V	c.761G>T	Verified	GCT	GGC	AAA	2	G	T
TCGA-04-1343	CLIP1	ENST00000358808	p.G816G	c.2448G>T	Verified	CAG	GGG	AGA	3	G	T
TCGA-24-1103	CLPTM1	ENST00000337392	p.W136C	c.408G>T	Verified	GAC	TGG	ACT	3	G	T
TCGA-23-1021	CLTCL1	ENST00000263200	p.L1471L	c.4413G>T	Verified	CTG	CTG	ACA	3	G	T
TCGA-23-1029	CLVS1	ENST00000325897	p.D291Y	c.871G>T	Unverified	CCC	GAC	TAC	1	G	T
TCGA-13-0883	CMYA5	ENST00000238522	p.E3089*	c.9265G>T	Verified	GAA	GAA	ACT	1	G	T
TCGA-13-0761	CNGA3	ENST00000272602	p.G634V	c.1901G>T	Verified	CTG	GGG	TCC	2	G	T
TCGA-13-0761	CNGA3	ENST00000272602	p.G634V	c.1901G>T	Verified	CTG	GGG	TCC	2	G	T
TCGA-24-2280	CNGB1	ENST00000251102	p.E521*	c.1561G>T	Unverified	GAT	GAG	GCT	1	G	T
TCGA-13-0920	CNNM4	ENST00000377075	p.G239V	c.716G>T	Verified	AAG	GGC	AAC	2	G	T
TCGA-13-0923	CNOT3	ENST00000221232	p.S358I	c.1073G>T	Unverified	GCC	AGT	CCA	2	G	T
TCGA-61-1740	CNTFR	ENST00000351266	p.L103L	c.309G>T	Unverified	CTG	CTG	CAT	3	G	T

TCGA-24-1563	COBLL1	ENST00000342193	p.G437V	c.1310G>T	Verified	CCT	GGG	ACA	2	G	T
TCGA-13-0893	COL14A1	ENST00000297848	p.E581*	c.1741G>T	Verified	gtt	gaa	gtc	1	G	T
TCGA-61-2008	COL15A1	ENST00000375001	p.R1266M	c.3797G>T	Verified	GTG	AGG	AAA	2	G	T
TCGA-24-2267	COL15A1	ENST00000375001	p.L1218L	c.3654G>T	Verified	GCT	CTG	CAT	3	G	T
TCGA-24-0979	COL16A1	ENST00000373672	p.G1561V	c.4682G>T	Verified	CCT	GGC	ATC	2	G	T
TCGA-13-0916	COL16A1	ENST00000373672	p.R201S	c.603G>T	Verified	ATG	AGG	CCT	3	G	T
TCGA-13-1505	COL22A1	ENST00000303045	p.G1045G	c.3135G>T	Verified	ATT	GGG	GTT	3	G	T
TCGA-13-1510	COL23A1	ENST00000390654	p.?	c.1494+1G>T	Unverified	GAG	AAG	GGA	3	G	T
TCGA-29-1691	COL23A1	ENST00000390654	p.E302D	c.906G>T	Unverified	GGC	GAG	CAG	3	G	T
TCGA-13-1498	COL24A1	ENST00000370571	p.G600C	c.1798G>T	Verified	CCT	GGC	AGG	1	G	T
TCGA-23-1123	COL27A1	ENST00000356083	p.C1731F	c.5192G>T	Verified	ACG	TGT	CTC	2	G	T
TCGA-23-1123	COL27A1	ENST00000356083	p.C1731F	c.5192G>T	Verified	ACG	TGT	CTC	2	G	T
TCGA-23-1117	COL4A4	ENST00000396625	p.G695C	c.2083G>T	Verified	CAA	GGT	GCC	1	G	T
TCGA-23-1117	COL4A4	ENST00000396625	p.G695C	c.2083G>T	Verified	CAA	GGT	GCC	1	G	T
TCGA-30-1714	COL4A4	ENST00000396625	p.D371Y	c.1111G>T	Unverified	GGG	GAC	CCA	1	G	T
TCGA-13-1497	COL4A6	ENST00000334504	p.G1442*	c.4324G>T	Unverified	CCA	GGA	TTT	1	G	T
TCGA-29-1702	COL5A1	ENST00000371817	p.D1761Y	c.5281G>T	Unverified	TAC	GAC	AAG	1	G	T
TCGA-24-2280	COL5A3	ENST00000264828	p.G1190V	c.3569G>T	Unverified	CCA	GGG	CTG	2	G	T
TCGA-61-1895	COL6A3	ENST00000295550	p.G1842V	c.5525G>T	Unverified	CTG	GGG	TTT	2	G	T
TCGA-04-1367	COL6A3	ENST00000295550	p.Q788H	c.2364G>T	Verified	GAG	CAG	ATT	3	G	T
TCGA-10-0930	COL6A3	ENST00000295550	p.?	c.5838+1G>T	Verified	GTG	AAG	GTG	3	G	T
TCGA-10-0930	COL6A3	ENST00000295550	p.?	c.5838+1G>T	Verified	GTG	AAG	GTG	3	G	T
TCGA-24-1469	COL6A3	ENST00000295550	p.Q1479H	c.4437G>T	Unverified	GTG	CAG	TTC	3	G	T
TCGA-24-2289	COL9A1	ENST00000357250	p.G481V	c.1442G>T	Unverified	AAA	GGG	GAA	2	G	T
TCGA-29-1770	COL9A2	ENST00000372748	p.P160P	c.480G>T	Unverified	CGC	CCG	GGA	3	G	T
TCGA-20-0990	COQ5	ENST00000288532	p.G276*	c.826G>T	Verified	GCT	GGA	GAC	1	G	T
TCGA-13-0913	COX11	ENST00000576370	p.R6L	c.17G>T	Verified	TGG	CGT	CCT	2	G	T
TCGA-24-1844	CPA2	ENST00000222481	p.K294N	c.882G>T	Unverified	ATC	AAG	AGT	3	G	T
TCGA-24-0979	CPAMD8	ENST00000291440	p.V513F	c.1537G>T	Verified	CAG	GTT	GGG	1	G	T
TCGA-13-0904	CPN2	ENST00000323830	p.G217C	c.649G>T	Verified	CTG	GGC	AGC	1	G	T
TCGA-04-1331	CPOX	ENST00000264193	p.G226V	c.677G>T	Unverified	AGA	GGA	AAA	2	G	T
TCGA-04-1331	CPSF2	ENST00000298875	p.M443I	c.1329G>T	Verified	ATG	ATG	AAA	3	G	T
TCGA-24-2280	CPT1A	ENST00000265641	p.A478S	c.1432G>T	Unverified	GAT	GCG	CCG	1	G	T
TCGA-13-0760	CPZ	ENST00000360986	p.V357L	c.1069G>T	Verified	AAG	GTG	GCC	1	G	T

TCGA-24-1844	CR1	ENST00000400960	p.V208L	c.622G>T	Unverified	CTT	GTG	GGT	1	G	T
TCGA-24-1844	CR1_ENST0(	ENST00000367049	p.V208L	c.622G>T	Unverified	CTT	GTG	GGT	1	G	T
TCGA-09-2044	CR2	ENST00000367057	p.G99*	c.295G>T	Verified	gga	gga	tac	1	G	T
TCGA-04-1347	CRADD	ENST00000332896	p.V22V	c.66G>T	Verified	ttg	gtg	gag	3	G	T
TCGA-10-0930	CREB3L1	ENST00000529193	p.?	c.1131+1G>T	Verified	CTC	ATG	GTG	3	G	T
TCGA-10-0930	CREB3L1	ENST00000529193	p.?	c.1131+1G>T	Verified	CTC	ATG	GTG	3	G	T
TCGA-29-1703	CREBBP	ENST00000262367	p.?	c.4280+1G>T	Unverified	ACG	AGG	CGT	2	G	T
TCGA-29-1699	CREBBP	ENST00000262367	p.Q286H	c.858G>T	Unverified	GGG	CAG	CCA	3	G	T
TCGA-13-0760	CREBZF	ENST00000490820	p.R222L	c.665G>T	Verified	AAT	CGA	CTG	2	G	T
TCGA-23-1118	CRTAC1	ENST00000370597	p.R436L	c.1307G>T	Verified	TTC	CGG	GGC	2	G	T
TCGA-23-1122	CRTC2	ENST00000368633	p.G626C	c.1876G>T	Unverified	CCA	GGT	TTC	1	G	T
TCGA-61-2094	CSDE1	ENST00000358528	p.L656F	c.1968G>T	Unverified	caa	ttg	tgt	3	G	T
TCGA-09-2051	CSE1L	ENST00000262982	p.V637F	c.1909G>T	Unverified	gct	gtt	gta	1	G	T
TCGA-24-1563	CSF2RA	ENST00000381529	p.E311*	c.931G>T	Verified	agt	gaa	gcc	1	G	T
TCGA-04-1338	CSF2RB	ENST00000403662	p.A85S	c.253G>T	Unverified	TCA	GCC	TGC	1	G	T
TCGA-29-1691	CSMD1_ENS	ENST00000318252	p.A2406S	c.7216G>T	Unverified	TGC	GGT	TCC	1	G	T
TCGA-29-1691	CSMD1_ENS	ENST00000537824	p.A2677S	c.8029G>T	Unverified	CTG	GCT	GGC	1	G	T
TCGA-61-2109	CSMD2	ENST00000241312	p.G2166V	c.6497G>T	Unverified	CAT	GGC	ACC	2	G	T
TCGA-04-1361	CSMD3	ENST00000297405	p.R2601L	c.7802G>T	Verified	ttc	cga	ctt	2	G	T
TCGA-04-1361	CSMD3	ENST00000297405	p.R2601L	c.7802G>T	Verified	ttc	cga	ctt	2	G	T
TCGA-09-1674	CSNK1D	ENST00000314028	p.R98S	c.294G>T	Unverified	TCC	AGG	AAA	3	G	T
TCGA-09-1674	CSNK1D_EN	ENST00000314028	p.R98S	c.294G>T	Unverified	TCC	AGG	AAA	3	G	T
TCGA-09-1674	CSNK1D_EN	ENST00000392334	p.R98S	c.294G>T	Unverified	TCC	AGG	AAA	3	G	T
TCGA-04-1652	CSNK1E	ENST00000359867	p.R127R	c.381G>T	Unverified	cac	cgg	gac	3	G	T
TCGA-04-1652	CSNK1E	ENST00000359867	p.R127R	c.381G>T	Unverified	cac	cgg	gac	3	G	T
TCGA-04-1652	CSNK1E_EN:	ENST00000400206	p.R127R	c.381G>T	Unverified	CAC	CGG	GAC	3	G	T
TCGA-04-1652	CSNK1E_EN:	ENST00000400206	p.R127R	c.381G>T	Unverified	CAC	CGG	GAC	3	G	T
TCGA-04-1652	CSNK1E_EN:	ENST00000403904	p.R127R	c.381G>T	Unverified	CAC	CGG	GAC	3	G	T
TCGA-04-1652	CSNK1E_EN:	ENST00000403904	p.R127R	c.381G>T	Unverified	CAC	CGG	GAC	3	G	T
TCGA-13-0920	CSPP1	ENST00000262210	p.D288Y	c.862G>T	Verified	CAA	GAT	CCT	1	G	T
TCGA-23-1022	CSRP2	ENST00000311083	p.V190F	c.568G>T	Verified	CTT	GTT	CAT	1	G	T
TCGA-61-1913	CST9L	ENST00000376979	p.A67S	c.199G>T	Unverified	TAT	GCC	TAC	1	G	T
TCGA-61-1907	CTAGE1	ENST00000391403	p.L672F	c.2016G>T	Unverified	TTA	TTG	TTT	3	G	T
TCGA-61-1907	CTAGE1_EN:	ENST00000391403	p.L672F	c.2016G>T	Unverified	TTA	TTG	TTT	3	G	T

TCGA-29-1775	CTAGE4	ENST00000486333	p.G638V	c.1913G>T	Unverified	TCT	GGA	CCA	2	G	T
TCGA-29-1783	CTDSP2	ENST00000398073	p.R101S	c.303G>T	Unverified	GGA	AGG	ATC	3	G	T
TCGA-29-1781	CTGF	ENST00000367976	p.V303L	c.907G>T	Unverified	CCG	GTG	GAG	1	G	T
TCGA-29-1761	CTR9	ENST00000361367	p.D924Y	c.2770G>T	Unverified	GAT	GAC	ACT	1	G	T
TCGA-24-1847	CTSL1	ENST00000343150	p.A146S	c.436G>T	Unverified	GGT	GCT	CTT	1	G	T
TCGA-61-1910	CTTNBP2	ENST00000160373	p.D1529Y	c.4585G>T	Unverified	GCA	GAT	CTT	1	G	T
TCGA-61-2012	CTTNBP2NL	ENST00000271277	p.R121L	c.362G>T	Verified	CAG	CGG	CAT	2	G	T
TCGA-04-1347	CUBN	ENST00000377833	p.T1841T	c.5523G>T	Verified	GGC	ACG	GGC	3	G	T
TCGA-23-1022	CUL5	ENST00000393094	p.C188F	c.563G>T	Verified	CTT	TGT	TCT	2	G	T
TCGA-04-1649	CXCR3	ENST00000373693	p.E293*	c.877G>T	Unverified	cga	gaa	agc	1	G	T
TCGA-04-1649	CXCR3_ENST	ENST00000373691	p.E340*	c.1018G>T	Unverified	CGA	GAA	AGC	1	G	T
TCGA-29-1701	CYLC2	ENST00000374798	p.G118V	c.353G>T	Unverified	AAA	GGT	GAA	2	G	T
TCGA-23-1122	CYP11B1	ENST00000292427	p.L373F	c.1119G>T	Verified	ACC	TTG	CGG	3	G	T
TCGA-29-1705	CYP1A1	ENST00000395048	p.C20F	c.59G>T	Unverified	TTC	TGT	CTG	2	G	T
TCGA-29-1691	CYP20A1	ENST00000356079	p.A8S	c.22G>T	Unverified	TTC	GCC	GTT	1	G	T
TCGA-29-2427	CYP26B1	ENST00000001146	p.G98V	c.293G>T	Unverified	ACC	GGC	GCG	2	G	T
TCGA-04-1347	CYP2C18	ENST00000285979	p.G79V	c.236G>T	Verified	CAT	GGA	TAT	2	G	T
TCGA-13-1489	CYP3A43	ENST00000222382	p.?	c.1026+1G>T	Verified	AAT	AAG	GCA	3	G	T
TCGA-13-0762	CYP4A11	ENST00000310638	p.V185F	c.553G>T	Unverified	GAG	GTC	TTT	1	G	T
TCGA-13-0762	CYP4A11	ENST00000310638	p.V185F	c.553G>T	Unverified	GAG	GTC	TTT	1	G	T
TCGA-61-1740	CYP4B1	ENST00000271153	p.T38T	c.114G>T	Unverified	CAG	ACG	TTG	3	G	T
TCGA-23-1022	CYP4F12	ENST00000324632	p.K177N	c.531G>T	Verified	GAC	AAG	TGG	3	G	T
TCGA-30-1718	CYSLTR1	ENST00000373304	p.V81F	c.241G>T	Unverified	gtg	gtc	tat	1	G	T
TCGA-61-1899	CYTL1	ENST00000307746	p.E52*	c.154G>T	Unverified	TCG	GAG	CCA	1	G	T
TCGA-29-1770	DAB1	ENST00000371233	p.D507Y	c.1519G>T	Unverified	GAT	GAC	ATC	1	G	T
TCGA-13-1496	DAB1	ENST00000371233	p.S519I	c.1556G>T	Verified	AAA	AGC	GAA	2	G	T
TCGA-13-1497	DACH2	ENST00000373125	p.?	c.1750+1G>T	Verified	caa	gga	ggt	1	G	T
TCGA-13-1497	DACH2_ENS	ENST00000373131	p.?	c.1711+1G>T	Verified	CAA	GCC	TGA	1	G	T
TCGA-24-1844	DAG1	ENST00000308775	p.V277L	c.829G>T	Unverified	GGT	GTA	GAG	1	G	T
TCGA-20-1686	DAP3	ENST00000368336	p.A184S	c.550G>T	Unverified	gag	gct	tca	1	G	T
TCGA-29-1699	DAP3	ENST00000368336	p.E285*	c.853G>T	Unverified	ccc	gag	gaa	1	G	T
TCGA-61-1907	DARS	ENST00000264161	p.R399I	c.1196G>T	Unverified	GTA	AGA	CCT	2	G	T
TCGA-13-1510	DCAF12L2	ENST00000538699	p.V418L	c.1252G>T	Verified	tgg	gtg	aac	1	G	T
TCGA-30-1891	DCAF12L2	ENST00000538699	p.A62A	c.186G>T	Unverified	gga	gcg	cgg	3	G	T

TCGA-24-1103	DCBLD1	ENST00000296955	p.G230G	c.690G>T	Verified	GAA	GGG	ATT	3	G	T
TCGA-13-0885	DCBLD2	ENST00000326840	p.R178L	c.533G>T	Verified	GGA	CGC	GGA	2	G	T
TCGA-25-1318	DCST2	ENST00000368424	p.R487L	c.1460G>T	Unverified	CGG	CGT	TGT	2	G	T
TCGA-25-1318	DCST2	ENST00000368424	p.R487L	c.1460G>T	Unverified	CGG	CGT	TGT	2	G	T
TCGA-30-1857	DCT	ENST00000377028	p.G112C	c.334G>T	Unverified	TTT	GGC	TGG	1	G	T
TCGA-30-1857	DCT_ENST0(	ENST00000446125	p.G112C	c.334G>T	Unverified	TTT	GGC	TGG	1	G	T
TCGA-23-1118	DDA1	ENST00000359866	p.L36L	c.108G>T	Verified	TAC	CTG	CCT	3	G	T
TCGA-29-1777	DDHD2	ENST00000397166	p.R434I	c.1301G>T	Unverified	CCA	AGA	AAG	2	G	T
TCGA-13-1509	DDX23	ENST00000308025	p.S541I	c.1622G>T	Verified	ctg	agc	cgc	2	G	T
TCGA-23-1031	DDX47	ENST00000358007	p.G128V	c.383G>T	Unverified	gta	ggt	gga	2	G	T
TCGA-10-0930	DDX52	ENST00000349699	p.A247S	c.739G>T	Verified	ctt	gcc	agc	1	G	T
TCGA-10-0930	DDX52	ENST00000349699	p.A247S	c.739G>T	Verified	ctt	gcc	agc	1	G	T
TCGA-24-1843	DDX60L	ENST00000260184	p.V896F	c.2686G>T	Unverified	TTG	GTT	CTT	1	G	T
TCGA-29-1761	DEFB129	ENST00000246105	p.A156S	c.466G>T	Unverified	TCT	GCC	ACT	1	G	T
TCGA-25-1318	DENND5B	ENST00000389082	p.G1200W	c.3598G>T	Verified	ATT	GGG	AAG	1	G	T
TCGA-25-1318	DENND5B	ENST00000389082	p.G1200W	c.3598G>T	Verified	ATT	GGG	AAG	1	G	T
TCGA-09-2049	DEPTOR	NM_022783.1	p.?	c.1102-1G>T	Verified	aag	gtc	tgt	1	G	T
TCGA-24-1845	DFFB	ENST00000378209	p.E248D	c.744G>T	Unverified	AGG	GAG	AGC	3	G	T
TCGA-23-1123	DGAT2L6	ENST00000333026	p.V184L	c.550G>T	Verified	gtg	gtg	ggt	1	G	T
TCGA-23-1123	DGAT2L6	ENST00000333026	p.V184L	c.550G>T	Verified	gtg	gtg	ggt	1	G	T
TCGA-24-1469	DGCR14	ENST00000252137	p.S147I	c.440G>T	Verified	CCC	AGC	CTA	2	G	T
TCGA-04-1342	DGCR2	ENST00000263196	p.R492L	c.1475G>T	Unverified	CGG	CGC	CTG	2	G	T
TCGA-23-1021	DGKA	ENST00000331886	p.D283Y	c.847G>T	Verified	tgc	gac	cgc	1	G	T
TCGA-09-1674	DGKD	ENST00000264057	p.R900L	c.2699G>T	Unverified	tgt	cgc	acg	2	G	T
TCGA-23-1122	DGKE	ENST00000284061	p.G61W	c.181G>T	Unverified	cac	ggg	tgg	1	G	T
TCGA-30-1857	DGKI	ENST00000288490	p.G1057V	c.3170G>T	Unverified	ATT	GGC	CAT	2	G	T
TCGA-30-1857	DGKI_ENST(	ENST00000288490	p.G1057V	c.3170G>T	Unverified	ATT	GGC	CAT	2	G	T
TCGA-25-1313	DHX32	ENST00000284690	p.G654C	c.1960G>T	Verified	tct	ggt	tac	1	G	T
TCGA-04-1530	DHX8	ENST00000262415	p.E361*	c.1081G>T	Verified	gag	gag	acc	1	G	T
TCGA-24-2289	DIAPH2	ENST00000324765	p.G697W	c.2089G>T	Unverified	act	ggg	cct	1	G	T
TCGA-61-1904	DIAPH2	ENST00000324765	p.A147S	c.439G>T	Unverified	act	gcc	aaa	1	G	T
TCGA-29-1691	DISP2	ENST00000267889	p.T489T	c.1467G>T	Unverified	GAC	ACG	GTG	3	G	T
TCGA-61-1910	DKKL1_ENST	ENST00000221498	p.G96V	c.287G>T	Unverified	CTG	GGG	AAC	2	G	T
TCGA-25-2392	DLG2	ENST00000398309	p.V161F	c.481G>T	Verified	GAG	GTT	TCC	1	G	T

TCGA-23-1120	DLG5	ENST00000372391	p.E508D	c.1524G>T	Verified	CAG	GAG	CTG	3	G	T
TCGA-04-1652	DLK2	ENST00000357338	p.L307F	c.921G>T	Unverified	AGC	TTG	GTG	3	G	T
TCGA-04-1652	DLK2	ENST00000357338	p.L307F	c.921G>T	Unverified	AGC	TTG	GTG	3	G	T
TCGA-13-0913	DLX6	ENST00000007660	p.Q152H	c.456G>T	Verified	CTC	CAG	GCT	3	G	T
TCGA-13-0905	DMBT1_ENS	ENST00000368915	p.G2033V	c.6098G>T	Verified	CAT	GGT	GGC	2	G	T
TCGA-10-0930	DMD_ENST(	ENST00000378677	p.G1770*	c.5308G>T	Verified	ACT	GGA	AAG	1	G	T
TCGA-10-0930	DMD_ENST(	ENST00000378677	p.G1770*	c.5308G>T	Verified	ACT	GGA	AAG	1	G	T
TCGA-29-1696	DMXL2	ENST00000251076	p.?	c.1618-1G>T	Unverified	CAG	GTT	TCT	1	G	T
TCGA-29-1696	DMXL2	ENST00000251076	p.?	c.1618-1G>T	Unverified	CAG	GTT	TCT	1	G	T
TCGA-13-0923	DNA2	ENST00000399180	p.G1077C	c.3229G>T	Unverified	GTT	GGT	GAA	1	G	T
TCGA-13-0923	DNA2L	ENST00000358410	p.G991C	c.2971G>T	Unverified	GTT	GGT	GAA	1	G	T
TCGA-23-1122	DNAH1_ENS	ENST00000420323	p.V1494F	c.4480G>T	Verified	ATC	GTC	ATT	1	G	T
TCGA-13-1497	DNAH17	ENST00000300671	p.G1810V	c.5429G>T	Verified	CTG	GGC	AAC	2	G	T
TCGA-24-1463	DNAH3	ENST00000261383	p.E3502*	c.10504G>T	Verified	ATC	GAA	GCC	1	G	T
TCGA-13-0904	DNAH5	ENST00000265104	p.L3284L	c.9852G>T	Verified	AAA	CTG	GAA	3	G	T
TCGA-20-1687	DNAH8	NM_001371.1	p.W1362L	c.4085G>T	Unverified	cac	tgg	gat	2	G	T
TCGA-20-1687	DNAH8_ENS	ENST00000359357	p.W1362L	c.4085G>T	Unverified	CAC	TGG	GAT	2	G	T
TCGA-13-0903	DNAH9	ENST00000262442	p.D3868Y	c.11602G>T	Verified	CGA	GAT	TTT	1	G	T
TCGA-04-1649	DNAH9	ENST00000262442	p.L2122L	c.6366G>T	Unverified	GAT	CTG	AAG	3	G	T
TCGA-24-2288	DNAJB4	ENST00000370763	p.V329L	c.985G>T	Unverified	gaa	gta	ctt	1	G	T
TCGA-13-1499	DNAJC6	ENST00000395325	p.E379*	c.1135G>T	Verified	tgg	gaa	cat	1	G	T
TCGA-24-1469	DNM1L	ENST00000549701	p.M439I	c.1317G>T	Verified	GAA	ATG	CAA	3	G	T
TCGA-23-2077	DNMT3B	ENST00000328111	p.V719L	c.2155G>T	Verified	cca	gtg	atg	1	G	T
TCGA-24-2267	DNTT	ENST00000371174	p.A56S	c.166G>T	Verified	CTG	GCC	CGC	1	G	T
TCGA-09-2044	DNTTIP1	ENST00000372622	p.G160*	c.478G>T	Verified	CGA	GGA	AGC	1	G	T
TCGA-24-1844	DOCK2	ENST00000256935	p.A1538S	c.4612G>T	Unverified	CCT	GCT	GTC	1	G	T
TCGA-04-1331	DOCK5	ENST00000276440	p.M800I	c.2400G>T	Verified	AAT	ATG	CTG	3	G	T
TCGA-24-1474	DOLK	ENST00000372586	p.V369F	c.1105G>T	Unverified	GCG	GTC	TTC	1	G	T
TCGA-29-1768	DPP8	ENST00000300141	p.E546*	c.1636G>T	Unverified	GGA	GAG	GTG	1	G	T
TCGA-29-1761	DSC1	ENST00000257197	p.K624N	c.1872G>T	Unverified	GAA	AAG	GAT	3	G	T
TCGA-29-1761	DSC1_ENST(	ENST00000257198	p.K624N	c.1872G>T	Unverified	GAA	AAG	GAT	3	G	T
TCGA-24-1616	DSC3	ENST00000360428	p.?	c.475-1G>T	Verified	CAA	GTT	GAA	1	G	T
TCGA-13-0886	DSCAM	ENST00000400454	p.E873*	c.2617G>T	Verified	GGG	GAG	GAC	1	G	T
TCGA-09-2056	DSCAML1	ENST00000321322	p.R610L	c.1829G>T	Verified	CAC	CGC	CAG	2	G	T

TCGA-24-2280	DSG1	ENST00000257192	p.V586F	c.1756G>T	Unverified	CCT	GTT	CCC	1	G	T
TCGA-29-1698	DSG4	ENST00000308128	p.R139S	c.417G>T	Unverified	GAA	AGG	CCT	3	G	T
TCGA-29-1698	DSG4_ENST(	ENST00000359747	p.R139S	c.417G>T	Unverified	GAA	AGG	CCT	3	G	T
TCGA-13-1499	DSN1	ENST00000373750	p.E130*	c.388G>T	Verified	GCA	GAA	AGC	1	G	T
TCGA-30-1714	DST_ENST0(	ENST00000244364	p.?	c.9072+1G>T	Unverified	CAA	AAG	CTT	3	G	T
TCGA-13-0886	DST_ENST0(	ENST00000370754	p.W338C	c.1014G>T	Verified	CTC	TGG	ACG	3	G	T
TCGA-13-0886	DST_ENST0(	ENST00000370769	p.W160C	c.480G>T	Verified	CTC	TGG	ACG	3	G	T
TCGA-30-1714	DST_ENST0(	ENST00000370769	p.?	c.16314+1G>T	Unverified	CAA	AAG	CTT	3	G	T
TCGA-29-1763	DTNA	ENST00000399097	p.R170I	c.509G>T	Unverified	CTC	AGA	CAG	2	G	T
TCGA-29-1763	DTNA_ENST	ENST00000269190	p.R523I	c.1568G>T	Unverified	CTC	AGA	CAG	2	G	T
TCGA-29-1763	DTNA_ENST	ENST00000444659	p.R522I	c.1565G>T	Unverified	CTC	AGA	CAG	2	G	T
TCGA-13-0761	DTNBP1	ENST00000344537	p.K26N	c.78G>T	Unverified	GAC	AAG	TCA	3	G	T
TCGA-13-0761	DTNBP1	ENST00000344537	p.K26N	c.78G>T	Unverified	GAC	AAG	TCA	3	G	T
TCGA-04-1542	DTX2	ENST00000324432	p.D118Y	c.352G>T	Verified	GAC	GAT	GGC	1	G	T
TCGA-30-1855	DYNC1I1	ENST00000324972	p.A622S	c.1864G>T	Unverified	TTT	GCC	AGG	1	G	T
TCGA-13-0807	DYSF	ENST00000258104	p.W857L	c.2570G>T	Verified	CTG	TGG	TTT	2	G	T
TCGA-04-1337	E2F2	ENST00000361729	p.L413L	c.1239G>T	Verified	tac	ctg	tgg	3	G	T
TCGA-61-1740	EBAG9	ENST00000395785	p.E154*	c.460G>T	Unverified	cag	gaa	aat	1	G	T
TCGA-04-1362	EBF2	ENST00000520164	p.E82D	c.246G>T	Verified	GTG	GAG	ATC	3	G	T
TCGA-13-1481	EBF2	ENST00000520164	p.R332S	c.996G>T	Verified	GGA	AGG	TTC	3	G	T
TCGA-24-1463	EEF1G	ENST00000329251	p.G4W	c.10G>T	Unverified	GCT	GGG	ACC	1	G	T
TCGA-09-2044	EEFSEC	ENST00000254730	p.G301G	c.903G>T	Verified	CGC	GGG	TTG	3	G	T
TCGA-13-0887	EFEMP1	ENST00000394554	p.D44Y	c.130G>T	Verified	AAA	GAT	ATT	1	G	T
TCGA-61-1906	EFEMP1	ENST00000394554	p.G100W	c.298G>T	Unverified	ACC	GGG	GTT	1	G	T
TCGA-61-1906	EFEMP1	ENST00000394554	p.G100W	c.298G>T	Unverified	ACC	GGG	GTT	1	G	T
TCGA-13-1408	EFEMP1	ENST00000394554	p.Q211H	c.633G>T	Verified	GAG	CAG	TGC	3	G	T
TCGA-24-1850	EGFR	ENST00000275493	p.K261N	c.783G>T	Unverified	TGC	AAG	GAC	3	G	T
TCGA-24-1850	EGFR_ENST(	ENST00000344576	p.K261N	c.783G>T	Unverified	TGC	AAG	GAC	3	G	T
TCGA-29-1693	EGR2	ENST00000242480	p.V36L	c.106G>T	Unverified	TCG	GTG	ACC	1	G	T
TCGA-13-0904	EHD2	ENST00000263277	p.E38*	c.112G>T	Unverified	GAG	GAG	CAC	1	G	T
TCGA-29-1691	EHD2	ENST00000263277	p.A179S	c.535G>T	Unverified	TTC	GCG	GAG	1	G	T
TCGA-29-1784	EHMT2	ENST00000375537	p.E77D	c.231G>T	Unverified	GAT	GAG	GGG	3	G	T
TCGA-20-0990	EIF2A_ENST(	ENST00000487799	p.C264F	c.791G>T	Verified	TTT	TGT	GCT	2	G	T
TCGA-13-0795	EIF2C1	ENST00000373204	p.G443V	c.1328G>T	Verified	aat	ggg	att	2	G	T

TCGA-23-1022	EIF4G1	ENST00000319274	p.R893R	c.2679G>T	Verified	CGG	CGG	CGC	3	G	T
TCGA-29-1691	EIF4G3	ENST00000264211	p.E879*	c.2635G>T	Unverified	CTG	GAA	GAA	1	G	T
TCGA-29-1691	EIF4G3_ENS	ENST00000374937	p.E885*	c.2653G>T	Unverified	CTG	GAA	GAA	1	G	T
TCGA-13-0906	ELFN2	ENST00000349653	p.L311L	c.933G>T	Verified	ACC	CTG	GTG	3	G	T
TCGA-09-2050	ELK1	ENST00000247161	p.C99F	c.296G>T	Verified	GAC	TGC	CCG	2	G	T
TCGA-25-2042	ELMO3	ENST00000393997	p.L421L	c.1263G>T	Unverified	GCC	CTG	GAC	3	G	T
TCGA-09-2049	ELN	ENST00000252034	p.A461S	c.1381G>T	Verified	GCA	GCT	GCT	1	G	T
TCGA-23-1031	ELOVL4	ENST00000369816	p.W169C	c.507G>T	Verified	TTG	TGG	TGG	3	G	T
TCGA-25-2400	EMID2	ENST00000397927	p.G350W	c.1048G>T	Verified	AAG	GGG	GAA	1	G	T
TCGA-24-1604	EML5	ENST00000554922	p.D957Y	c.2869G>T	Verified	GAA	GAT	AAT	1	G	T
TCGA-13-0887	EMR2	ENST00000315576	p.A656S	c.1966G>T	Verified	CCA	GCT	GTG	1	G	T
TCGA-29-1761	ENSG00000	ENST00000329762	p.V51V	c.153G>T	Unverified	GGT	GTG	GAT	3	G	T
TCGA-20-1683	ENSG00000	ENST00000402510	p.R1164M	c.3491G>T	Unverified	AAG	AGG	TCT	2	G	T
TCGA-23-1114	ENSG00000	ENST00000445286	p.Q318H	c.954G>T	Unverified	TTC	AGG	CGC	3	G	T
TCGA-13-0760	EOMES	ENST00000295743	p.E613*	c.1837G>T	Verified	GAG	GAA	ATT	1	G	T
TCGA-04-1651	EPB42	ENST00000300215	p.V308V	c.924G>T	Unverified	ACA	GTG	CTG	3	G	T
TCGA-04-1651	EPB42	ENST00000300215	p.V308V	c.924G>T	Unverified	ACA	GTG	CTG	3	G	T
TCGA-13-0887	EPC2	ENST00000258484	p.A599S	c.1795G>T	Verified	CTT	GCC	CAG	1	G	T
TCGA-61-1998	EPG5	ENST00000282041	p.?	c.7226+1G>T	Verified	CCA	AGC	TCC	2	G	T
TCGA-13-0923	EPHA7	NM_004440	p.E482D	c.1446G>T	Verified	tac	gag	aaa	3	G	T
TCGA-13-1408	EPHA7	NM_004440	p.R676S	c.2028G>T	Unverified	caa	agg	aga	3	G	T
TCGA-24-0979	EPS15L1	ENST00000248070	p.?	c.1107+1G>T	Verified	GGC	CCG	GAC	3	G	T
TCGA-61-2095	ERBB3_ENS	ENST00000267101	p.L49L	c.147G>T	Unverified	ACA	CTG	TAC	3	G	T
TCGA-61-2008	ERCC6	ENST00000355832	p.?	c.423-1G>T	Unverified	CTC	ACG	TCA	3	G	T
TCGA-61-1914	ERI1	ENST00000250263	p.K217N	c.651G>T	Unverified	TTG	AAG	GAA	3	G	T
TCGA-23-1031	ESD	ENST00000378720	p.L48L	c.144G>T	Verified	GCA	CTG	TAT	3	G	T
TCGA-29-1770	ESR2	ENST00000341099	p.G218C	c.652G>T	Unverified	tgt	ggc	tcc	1	G	T
TCGA-29-1770	ESR2_ENST0	ENST00000554572	p.G218C	c.652G>T	Unverified	TGT	GGC	TCC	1	G	T
TCGA-04-1338	ESYT1	ENST00000394048	p.G79V	c.236G>T	Verified	GTG	GGT	TTC	2	G	T
TCGA-29-1761	ETS1	ENST00000319397	p.Q71H	c.213G>T	Unverified	cgg	cag	tgg	3	G	T
TCGA-29-1761	ETS1_ENST0	ENST00000392668	p.Q115H	c.345G>T	Unverified	CGG	CAG	TGG	3	G	T
TCGA-23-1117	ETS2	ENST00000360214	p.G356C	c.1066G>T	Verified	GCC	GGC	TTC	1	G	T
TCGA-23-1117	ETS2	ENST00000360214	p.G356C	c.1066G>T	Verified	GCC	GGC	TTC	1	G	T
TCGA-04-1361	EVC	ENST00000264956	p.R194R	c.582G>T	Verified	CTC	CGG	GTG	3	G	T

TCGA-04-1361	EVC	ENST00000264956	p.R194R	c.582G>T	Verified	CTC	CGG	GTG	3	G	T
TCGA-13-1408	EVC2	ENST00000344408	p.A786S	c.2356G>T	Unverified	GCT	GCA	CGG	1	G	T
TCGA-29-1766	EVI2A	ENST00000247270	p.R244I	c.731G>T	Unverified	GAA	AGA	AAA	2	G	T
TCGA-61-2095	EVPL	ENST00000301607	p.Q1098H	c.3294G>T	Unverified	CTG	CAG	ATG	3	G	T
TCGA-13-0903	EVX2	ENST00000308618	p.S156S	c.468G>T	Verified	ACG	TCG	GCG	3	G	T
TCGA-04-1347	EXOC2	ENST00000230449	p.A57S	c.169G>T	Verified	tct	gca	agt	1	G	T
TCGA-24-2035	EXOC2	ENST00000230449	p.Q462H	c.1386G>T	Verified	agc	cag	ctg	3	G	T
TCGA-23-1117	EXOSC2	ENST00000372358	p.E232D	c.696G>T	Verified	CGA	GAG	GTG	3	G	T
TCGA-23-1117	EXOSC2	ENST00000372358	p.E232D	c.696G>T	Verified	CGA	GAG	GTG	3	G	T
TCGA-13-0792	EYA1	ENST00000388742	p.W430C	c.1290G>T	Verified	gac	tgg	atg	3	G	T
TCGA-13-1408	F11R	ENST00000289779	p.G120W	c.358G>T	Verified	TAT	GGG	GAG	1	G	T
TCGA-25-1318	F13A1	ENST00000264870	p.G330V	c.989G>T	Verified	CTT	GGA	ATA	2	G	T
TCGA-25-1318	F13A1	ENST00000264870	p.G330V	c.989G>T	Verified	CTT	GGA	ATA	2	G	T
TCGA-61-1914	F5	ENST00000367797	p.D843Y	c.2527G>T	Unverified	CCA	GAT	GTC	1	G	T
TCGA-24-1103	F8	ENST00000360256	p.L1862L	c.5586G>T	Verified	GAC	CTG	GAA	3	G	T
TCGA-24-1103	F8_ENST000	ENST00000360256	p.L1862L	c.5586G>T	Verified	GAC	CTG	GAA	3	G	T
TCGA-61-1914	FAM102A	ENST00000373095	p.G148*	c.442G>T	Unverified	TCT	GGA	GAT	1	G	T
TCGA-09-2049	FAM105B	ENST00000284274	p.R121R	c.363G>T	Verified	ATA	CGG	CGA	3	G	T
TCGA-13-1512	FAM117A	ENST00000240364	p.G291V	c.872G>T	Verified	CGG	GGT	GCC	2	G	T
TCGA-04-1367	FAM123A	ENST00000357816	p.G24V	c.71G>T	Unverified	GTG	GGG	GTC	2	G	T
TCGA-13-0893	FAM126B	ENST00000418596	p.A347S	c.1039G>T	Verified	GAT	GCA	GAT	1	G	T
TCGA-24-1422	FAM135B	ENST00000395297	p.V12L	c.34G>T	Verified	TCG	GTA	GAG	1	G	T
TCGA-04-1338	FAM171A1	ENST00000378116	p.E158*	c.472G>T	Verified	CCT	GAG	AAC	1	G	T
TCGA-13-0920	FAM171B	ENST00000304698	p.G766*	c.2296G>T	Verified	GAT	GGA	GGG	1	G	T
TCGA-13-0893	FAM183A	ENST00000335282	p.K81N	c.243G>T	Verified	AAG	AAG	TAC	3	G	T
TCGA-13-1488	FAM204A	ENST00000369183	p.E168*	c.502G>T	Verified	ATT	GAG	AAG	1	G	T
TCGA-04-1361	FAM20B	ENST00000263733	p.G269V	c.806G>T	Verified	TCT	GGC	CCG	2	G	T
TCGA-04-1361	FAM20B	ENST00000263733	p.G269V	c.806G>T	Verified	TCT	GGC	CCG	2	G	T
TCGA-24-1844	FAM26F	ENST00000368605	p.V297L	c.889G>T	Unverified	ACG	GTG	ATT	1	G	T
TCGA-29-1769	FAM35A	ENST00000358313	p.C317F	c.950G>T	Unverified	GTT	TGT	CCT	2	G	T
TCGA-13-1498	FAM53C	ENST00000239906	p.V364V	c.1092G>T	Verified	GCT	GTG	CGG	3	G	T
TCGA-13-1510	FAM69C	ENST00000400291	p.K120N	c.360G>T	Unverified	GAG	AAG	TAG	3	G	T
TCGA-24-2280	FAM75D4	ENST00000344803	p.V642V	c.1926G>T	Unverified	TCT	GTG	GTT	3	G	T
TCGA-29-1784	FAM83B	ENST00000306858	p.R459S	c.1377G>T	Unverified	GAC	AGG	AAT	3	G	T

TCGA-24-1103	FAM83D	ENST00000217429	p.K506N	c.1518G>T	Verified	TTG	AAG	TCT	3	G	T
TCGA-24-2280	FAM92B	ENST00000539556	p.L163L	c.489G>T	Unverified	CAG	CTG	GAG	3	G	T
TCGA-13-1499	FANCC_ENST	ENST00000289081	p.C96F	c.287G>T	Verified	TGT	TGT	CTA	2	G	T
TCGA-23-1123	FANCD2	ENST00000287647	p.E650*	c.1948G>T	Verified	ctg	gaa	tgg	1	G	T
TCGA-23-1123	FANCD2	ENST00000287647	p.E650*	c.1948G>T	Verified	ctg	gaa	tgg	1	G	T
TCGA-24-2024	FANCM	ENST00000267430	p.R1666I	c.4997G>T	Verified	TCC	AGA	ATT	2	G	T
TCGA-30-1891	FARSA	ENST00000314606	p.V430L	c.1288G>T	Unverified	TGG	GTG	GAG	1	G	T
TCGA-13-0886	FAT1	ENST00000441802	p.V2267L	c.6799G>T	Unverified	GAA	GTA	TTT	1	G	T
TCGA-23-1022	FAT3_ENST(	ENST00000298047_v61	p.K224N	c.672G>T	Verified	GAA	AAG	AAT	3	G	T
TCGA-24-2280	FAT3_ENST(	ENST00000298047_v61	p.Q1253H	c.3759G>T	Verified	CCC	CAG	TTC	3	G	T
TCGA-30-1718	FAT3_ENST(	ENST00000298047_v61	p.K2803N	c.8409G>T	Unverified	ATC	AAG	GTA	3	G	T
TCGA-23-1022	FAT3_ENST(	ENST00000409404	p.K224N	c.672G>T	Verified	GAA	AAG	AAT	3	G	T
TCGA-24-2280	FAT3_ENST(	ENST00000409404	p.Q1253H	c.3759G>T	Verified	CCC	CAG	TTC	3	G	T
TCGA-30-1718	FAT3_ENST(	ENST00000409404	p.K2803N	c.8409G>T	Unverified	ATC	AAG	GTA	3	G	T
TCGA-13-1512	FBL	ENST00000221801	p.G47V	c.140G>T	Verified	GGA	GGA	GGT	2	G	T
TCGA-23-1118	FBRS	ENST00000287468	p.G381C	c.1141G>T	Verified	GCG	GGT	GAG	1	G	T
TCGA-25-2398	FBXO15	ENST00000269500	p.L403L	c.1209G>T	Verified	gag	ctg	gtg	3	G	T
TCGA-04-1362	FBXO40	ENST00000338040	p.E407*	c.1219G>T	Verified	AGA	GAA	CTC	1	G	T
TCGA-24-0979	FBXO42	ENST00000375592	p.A565S	c.1693G>T	Unverified	aaa	gcg	atg	1	G	T
TCGA-09-1674	FBXO7	ENST00000266087	p.D136Y	c.406G>T	Unverified	AAT	GAC	GAC	1	G	T
TCGA-09-1674	FBXO7_ENST	ENST00000266087	p.D136Y	c.406G>T	Unverified	AAT	GAC	GAC	1	G	T
TCGA-09-1674	FBXO7_ENST	ENST00000382058	p.D57Y	c.169G>T	Unverified	AAT	GAC	GAC	1	G	T
TCGA-25-2392	FBXW8	ENST00000309909	p.S470S	c.1410G>T	Verified	ctg	tcg	ctc	3	G	T
TCGA-29-1691	FCAMR	ENST00000400962	p.G253*	c.757G>T	Unverified	TCT	GGA	GAA	1	G	T
TCGA-29-1691	FCAMR_ENS	ENST00000324852	p.W520L	c.1559G>T	Unverified	CTC	TGG	AGA	2	G	T
TCGA-09-1665	FCF1	ENST00000341162	p.R167I	c.500G>T	Verified	AGA	AGA	ATC	2	G	T
TCGA-09-2044	FCHO2	ENST00000430046	p.S508I	c.1523G>T	Verified	GAA	AGT	TCT	2	G	T
TCGA-24-1417	FCRL4	ENST00000271532	p.G171V	c.512G>T	Verified	TAT	GGA	GAC	2	G	T
TCGA-04-1367	FER1L6	ENST00000399018	p.D1559Y	c.4675G>T	Verified	AAG	GAT	AAG	1	G	T
TCGA-24-2289	FERMT2	ENST00000395631	p.A542S	c.1624G>T	Unverified	GAG	GCC	CAT	1	G	T
TCGA-13-1507	FGB	ENST00000302068	p.D419Y	c.1255G>T	Verified	TCA	GAT	CCC	1	G	T
TCGA-20-1685	FGB	ENST00000302068	p.W279L	c.836G>T	Unverified	GGA	TGG	ACA	2	G	T
TCGA-04-1331	FGD1	ENST00000375135	p.A818S	c.2452G>T	Verified	gtg	gct	gca	1	G	T
TCGA-09-2051	FGD6	ENST00000343958	p.C256F	c.767G>T	Unverified	ACT	TGC	CAG	2	G	T

TCGA-24-1604	FGF6	ENST00000228837	p.S76I	c.227G>T	Verified	GAA	AGT	GGC	2	G	T
TCGA-23-1123	FIGNL1	ENST00000395556	p.D71Y	c.211G>T	Verified	ATT	GAT	TCT	1	G	T
TCGA-23-1123	FIGNL1	ENST00000395556	p.D71Y	c.211G>T	Verified	ATT	GAT	TCT	1	G	T
TCGA-61-2009	FLG	ENST00000368799	p.D3492Y	c.10474G>T	Verified	AAT	GAC	GAA	1	G	T
TCGA-24-1844	FLG	ENST00000368799	p.R1203S	c.3609G>T	Unverified	GGA	AGG	TCT	3	G	T
TCGA-24-0979	FLG2	ENST00000388718	p.G1065V	c.3194G>T	Verified	TTT	GGA	CAA	2	G	T
TCGA-13-1510	FLJ20273	ENST00000381795	p.G43C	c.127G>T	Unverified	ACG	GGC	TAC	1	G	T
TCGA-23-1117	FLJ90650	ENST00000357872	p.A341S	c.1021G>T	Verified	AGT	GCA	GAC	1	G	T
TCGA-23-1117	FLJ90650	ENST00000357872	p.A341S	c.1021G>T	Verified	AGT	GCA	GAC	1	G	T
TCGA-29-1698	FLNB	ENST00000295956	p.R73L	c.218G>T	Unverified	TTT	CGC	CAG	2	G	T
TCGA-29-1698	FLNB_ENST(	ENST00000490882	p.R73L	c.218G>T	Unverified	TTT	CGC	CAG	2	G	T
TCGA-23-1117	FLNC	ENST00000325888	p.V1054L	c.3160G>T	Verified	GCT	GTG	GAG	1	G	T
TCGA-23-1117	FLNC	ENST00000325888	p.V1054L	c.3160G>T	Verified	GCT	GTG	GAG	1	G	T
TCGA-13-0795	FMR1	ENST00000370475	p.?	c.270+1G>T	Verified	ggt	gag	ttt	3	G	T
TCGA-25-1326	FNDC1	ENST00000297267	p.R830M	c.2489G>T	Verified	GGG	AGG	TCT	2	G	T
TCGA-61-1899	FNDC9	ENST00000312349	p.A118S	c.352G>T	Unverified	ATG	GCC	ATT	1	G	T
TCGA-13-0903	FOXA2	ENST00000377115	p.Q173H	c.519G>T	Verified	atc	cag	cag	3	G	T
TCGA-13-0916	FOXJ3	ENST00000372573	p.A153S	c.457G>T	Verified	tgg	gca	ata	1	G	T
TCGA-25-1313	FPR3	ENST00000339223	p.S168I	c.503G>T	Unverified	ATA	AGT	ACT	2	G	T
TCGA-04-1347	FREM1	ENST00000422223	p.W418C	c.1254G>T	Verified	TCC	TGG	AAT	3	G	T
TCGA-13-0916	FRMD4A	ENST00000357447	p.D152Y	c.454G>T	Verified	GGA	GAT	TTT	1	G	T
TCGA-13-0793	FRMD4B	ENST00000264546	p.G202V	c.605G>T	Verified	TAC	GGT	GTC	2	G	T
TCGA-09-2049	FRY	ENST00000380250	p.G1989C	c.5965G>T	Verified	TTT	GGT	GTC	1	G	T
TCGA-13-0916	FRY	ENST00000380250	p.D2489Y	c.7465G>T	Verified	CTG	GAC	AGC	1	G	T
TCGA-24-1844	FSCN3	ENST00000265825	p.E231*	c.691G>T	Unverified	GGA	GAA	GGA	1	G	T
TCGA-13-0792	FSIP1	ENST00000350221	p.E455*	c.1363G>T	Verified	AAT	GAA	TCA	1	G	T
TCGA-09-1665	FTSJ3	ENST00000582115	p.D50Y	c.148G>T	Verified	CTG	GAC	CTG	1	G	T
TCGA-25-1318	FUK	ENST00000288078	p.R35R	c.105G>T	Verified	AAG	CGG	GAG	3	G	T
TCGA-25-1318	FUK	ENST00000288078	p.R35R	c.105G>T	Verified	AAG	CGG	GAG	3	G	T
TCGA-24-2267	G6PD	ENST00000291567	p.R393L	c.1178G>T	Verified	ATC	CGC	GTG	2	G	T
TCGA-29-1762	GAD2	ENST00000259271	p.G413*	c.1237G>T	Unverified	GAG	GGA	TTG	1	G	T
TCGA-61-2109	GAD2	ENST00000259271	p.G383V	c.1148G>T	Unverified	AGT	GGC	GTG	2	G	T
TCGA-29-1693	GAL3ST1	ENST00000338911	p.G291W	c.871G>T	Unverified	TCG	GGG	GAG	1	G	T
TCGA-29-1703	GALNT13	ENST00000392825	p.V135F	c.403G>T	Unverified	ACT	GTT	TAC	1	G	T

TCGA-24-2267	GARS	ENST00000389266	p.L375F	c.1125G>T	Verified	TAT	TTG	TAT	3	G	T
TCGA-09-2051	GCA	ENST00000437150	p.G37*	c.109G>T	Unverified	GAT	GGA	TAC	1	G	T
TCGA-61-1998	GCC1	ENST00000321407	p.A610S	c.1828G>T	Verified	GTG	GCC	TTG	1	G	T
TCGA-24-0975	GCM1	ENST00000259803	p.?	c.441+1G>T	Unverified	TTC	CAG	TCA	3	G	T
TCGA-29-1761	GCNT4	ENST00000322348	p.W220L	c.659G>T	Unverified	CAG	TGG	AAA	2	G	T
TCGA-13-1501	GCNT4	ENST00000322348	p.R399S	c.1197G>T	Verified	TTA	AGG	TGG	3	G	T
TCGA-13-1501	GCNT4	ENST00000322348	p.R399S	c.1197G>T	Verified	TTA	AGG	TGG	3	G	T
TCGA-24-1604	GEMIN7	ENST00000391951	p.A47S	c.139G>T	Verified	ATA	GCT	CAA	1	G	T
TCGA-61-2008	GFOD2	ENST00000268797	p.V7V	c.21G>T	Verified	GGA	GTG	GGC	3	G	T
TCGA-13-0755	GFRA1	ENST00000369236	p.R221L	c.662G>T	Verified	AGG	CGA	CAG	2	G	T
TCGA-09-2049	GFRAL	ENST00000340465	p.G124V	c.371G>T	Verified	CAT	GGA	TTC	2	G	T
TCGA-29-1698	GGA3	ENST00000245541	p.L102L	c.306G>T	Unverified	TAC	CTG	GGG	3	G	T
TCGA-04-1347	GGT5	ENST00000327365	p.G585V	c.1754G>T	Verified	GCA	GGC	TAC	2	G	T
TCGA-23-1809	GGTLC2	ENST00000215938	p.R149L	c.446G>T	Unverified	GGC	CGT	GCC	2	G	T
TCGA-04-1338	GHRL	ENST00000335542	p.G88V	c.263G>T	Verified	TCA	GGG	GTT	2	G	T
TCGA-23-1117	GIOT-1	ENST00000396893	p.R240M	c.719G>T	Verified	TTT	AGG	CAT	2	G	T
TCGA-23-1117	GIOT-1	ENST00000396893	p.R240M	c.719G>T	Verified	TTT	AGG	CAT	2	G	T
TCGA-29-1781	GJA8	ENST00000369235	p.W78C	c.234G>T	Unverified	CTC	TGG	GTG	3	G	T
TCGA-24-1469	GJB4	ENST00000339480	p.G242V	c.725G>T	Verified	CAG	GGA	GGG	2	G	T
TCGA-29-1781	GJB5	ENST00000338513	p.M93I	c.279G>T	Unverified	GTC	ATG	CAC	3	G	T
TCGA-24-2267	GK2	ENST00000358842	p.C283F	c.848G>T	Verified	GGT	TGC	TTC	2	G	T
TCGA-61-2102	GLI2	ENST00000361492	p.A325S	c.973G>T	Verified	CTG	GCC	CAG	1	G	T
TCGA-20-0991	GLI2	ENST00000361492	p.S1482I	c.4445G>T	Verified	GAC	AGC	ATC	2	G	T
TCGA-13-0795	GLIPR1	ENST00000266659	p.G161V	c.482G>T	Verified	TCT	GGC	TTT	2	G	T
TCGA-09-2050	GLO1	ENST00000373365	p.G10C	c.28G>T	Unverified	GGC	GGC	CTC	1	G	T
TCGA-09-2049	GLRA1	ENST00000274576	p.A203S	c.607G>T	Verified	GGA	GCC	GTG	1	G	T
TCGA-29-1777	GLUD1	ENST00000277865	p.P43P	c.129G>T	Unverified	CAG	CCG	GGG	3	G	T
TCGA-61-1740	GMPS	ENST00000496455	p.G142C	c.424G>T	Unverified	agg	ggc	ctt	1	G	T
TCGA-29-1702	GNAI3	ENST00000369851	p.Q164H	c.492G>T	Unverified	tcc	cag	tct	3	G	T
TCGA-29-1761	GNAL	ENST00000334049	p.V181F	c.541G>T	Unverified	CCA	GTT	CCG	1	G	T
TCGA-13-1497	GNPAT	ENST00000366647	p.V510F	c.1528G>T	Verified	GAT	GTC	TAC	1	G	T
TCGA-23-1809	GNPAT	ENST00000366647	p.E290*	c.868G>T	Unverified	GAA	GAA	ACT	1	G	T
TCGA-61-1725	GNPTG	ENST00000204679	p.L259L	c.777G>T	Unverified	AGG	CTG	AAA	3	G	T
TCGA-13-0923	GOLGB1	ENST00000340645	p.E2564*	c.7690G>T	Verified	AAG	GAG	CTG	1	G	T

TCGA-24-1847	GOLGB1	ENST00000340645	p.G1301V	c.3902G>T	Unverified	GGC	GGA	ACT	2	G	T
TCGA-23-1122	GPAM	ENST00000348367	p.G154V	c.461G>T	Verified	GAT	GGT	TCT	2	G	T
TCGA-25-2391	GPR124	ENST00000412232	p.G98G	c.294G>T	Unverified	ACG	GGG	CTC	3	G	T
TCGA-29-1777	GPR126	ENST00000296932	p.D709Y	c.2125G>T	Unverified	GAA	GAT	TCT	1	G	T
TCGA-29-1777	GPR126_ENS	ENST00000367609	p.D737Y	c.2209G>T	Unverified	GAA	GAT	TCT	1	G	T
TCGA-24-2289	GPR137C	ENST00000321662	p.G313V	c.938G>T	Unverified	AGT	GGA	GAA	2	G	T
TCGA-23-1118	GPR139	ENST00000570682	p.A296S	c.886G>T	Verified	ATG	GCA	GCC	1	G	T
TCGA-13-1510	GPR149	ENST00000389740	p.G50C	c.148G>T	Unverified	GTG	GGC	AGC	1	G	T
TCGA-25-2042	GPR50	NM_004224.1	p.G167C	c.499G>T	Verified	att	ggc	acc	1	G	T
TCGA-13-1507	GPR77	ENST00000257267	p.R300L	c.899G>T	Verified	CTC	CGC	CGG	2	G	T
TCGA-04-1362	GPR78	ENST00000382487	p.K242N	c.726G>T	Verified	AGG	AAG	ATT	3	G	T
TCGA-61-2095	GPRC5D	ENST00000228887	p.R49L	c.146G>T	Unverified	ATG	CGA	AAG	2	G	T
TCGA-61-2102	GPRIN1	ENST00000303991	p.G549C	c.1645G>T	Verified	GTG	GGC	AAG	1	G	T
TCGA-13-0884	GPX6	ENST00000361902	p.G105*	c.313G>T	Verified	TTT	GGA	AAA	1	G	T
TCGA-13-0885	GRB14	ENST00000263915	p.G497C	c.1489G>T	Verified	GAC	GGT	GAA	1	G	T
TCGA-24-1845	GRIA3	NM_000828.2	p.D484Y	c.1450G>T	Unverified	agg	gat	cca	1	G	T
TCGA-24-1845	GRIA3_ENST	ENST00000264357	p.D484Y	c.1450G>T	Unverified	AGG	GAT	CCA	1	G	T
TCGA-24-1845	GRIA3_ENST	ENST00000371256	p.D484Y	c.1450G>T	Unverified	AGG	GAT	CCA	1	G	T
TCGA-23-1122	GRID2	ENST00000282020	p.R297R	c.891G>T	Verified	CAG	CGG	TGT	3	G	T
TCGA-23-1120	GRIN3A	ENST00000361820	p.W907C	c.2721G>T	Verified	AAG	TGG	TAC	3	G	T
TCGA-13-0904	GRK1	NM_002929.2	p.K40N	c.120G>T	Verified	ctc	aag	ctg	3	G	T
TCGA-23-1123	GRLF1_ENST	ENST00000317082_v61	p.S752I	c.2255G>T	Verified	ATC	AGT	CAA	2	G	T
TCGA-23-1123	GRLF1_ENST	ENST00000317082_v61	p.S752I	c.2255G>T	Verified	ATC	AGT	CAA	2	G	T
TCGA-13-0883	GRM1	ENST00000282753	p.W224C	c.672G>T	Verified	AAT	TGG	ACC	3	G	T
TCGA-25-2400	GRM1	ENST00000282753	p.R78S	c.234G>T	Unverified	CAG	AGG	GTG	3	G	T
TCGA-13-0885	GRM4	ENST00000374181	p.V824F	c.2470G>T	Verified	ACG	GTC	TCG	1	G	T
TCGA-13-0885	GRM4_ENST	ENST00000374177	p.V708F	c.2122G>T	Verified	ACG	GTC	TCG	1	G	T
TCGA-13-0884	GSPT1	NM_002094.1	p.P66P	c.198G>T	Verified	gca	ccg	cca	3	G	T
TCGA-24-2267	GSTP1	ENST00000398606	p.L161L	c.483G>T	Verified	CTG	CTG	ATC	3	G	T
TCGA-61-2008	GTF2H4	ENST00000259895	p.V343V	c.1029G>T	Verified	GTG	GTG	GCG	3	G	T
TCGA-13-0905	GYLTL1B	ENST00000325468	p.A448S	c.1342G>T	Verified	GAA	GCC	CTG	1	G	T
TCGA-24-1616	HAS2	ENST00000303924	p.G491C	c.1471G>T	Verified	GGT	GGT	GTG	1	G	T
TCGA-13-0887	HAS2	ENST00000303924	p.G139V	c.416G>T	Verified	ATG	GGC	AGA	2	G	T
TCGA-29-1768	HAUS3	ENST00000243706	p.L40L	c.120G>T	Unverified	TTT	CTG	AAG	3	G	T

TCGA-13-0885	HCRTR1	ENST00000373706	p.V106L	c.316G>T	Verified	CTG	GTG	GAC	1	G	T
TCGA-13-0903	HDAC3	ENST00000305264	p.?	c.420+1G>T	Verified	TTT	GAG	GCC	3	G	T
TCGA-24-1470	HEATR3	ENST00000299192	p.Q212H	c.636G>T	Verified	TTG	CAG	ACA	3	G	T
TCGA-61-1998	HEATR6	ENST00000184956	p.C232F	c.695G>T	Verified	TTT	TGC	ATG	2	G	T
TCGA-30-1714	HEATR8	ENST00000421030	p.R736L	c.2207G>T	Unverified	TAC	CGC	CAC	2	G	T
TCGA-30-1714	HEATR8_EN:	ENST00000421030	p.R736L	c.2207G>T	Unverified	TAC	CGC	CAC	2	G	T
TCGA-61-1915	HECTD3	NM_024602.4	p.D500Y	c.1498G>T	Unverified	gag	gac	cgg	1	G	T
TCGA-61-1915	HECTD3_EN:	ENST00000372168	p.D394Y	c.1180G>T	Unverified	GAG	GAC	CGG	1	G	T
TCGA-61-1915	HECTD3_EN:	ENST00000372172	p.D784Y	c.2350G>T	Unverified	GAG	GAC	CGG	1	G	T
TCGA-61-2009	HELZ	ENST00000358691	p.E122D	c.366G>T	Verified	GGA	GAG	TCC	3	G	T
TCGA-04-1362	HEMGN	ENST00000375112	p.A154S	c.460G>T	Verified	ATA	GCA	GTA	1	G	T
TCGA-20-1685	HERC2	NM_004667.3	p.L3391L	c.10173G>T	Unverified	aat	ctg	gcc	3	G	T
TCGA-13-0924	HGF	ENST00000222390	p.G520*	c.1558G>T	Verified	TGC	GGA	GGA	1	G	T
TCGA-30-1718	HGS	ENST00000329138	p.A301S	c.901G>T	Unverified	TCA	GCG	CCC	1	G	T
TCGA-24-1844	HIP1	ENST00000336926	p.A911S	c.2731G>T	Unverified	ATT	GCT	GCT	1	G	T
TCGA-24-1417	HIST1H2AG	ENST00000359193	p.G3V	c.8G>T	Unverified	TCT	GGA	CGT	2	G	T
TCGA-04-1655	HIST1H2BE	ENST00000356530	p.V19V	c.57G>T	Unverified	GCC	GTG	ACC	3	G	T
TCGA-13-0755	HIST1H3A	ENST00000357647	p.Q20H	c.60G>T	Verified	AAA	CAG	TTG	3	G	T
TCGA-24-1604	HIST1H3H	ENST00000369163	p.V90V	c.270G>T	Unverified	GCG	GTG	ATG	3	G	T
TCGA-61-1733	HIST1H4G	ENST00000244537	p.K78N	c.234G>T	Unverified	GCC	AAG	CGC	3	G	T
TCGA-61-1733	HIST1H4G	ENST00000244537	p.K78N	c.234G>T	Unverified	GCC	AAG	CGC	3	G	T
TCGA-13-1481	HIST1H4J	ENST00000355057	p.R96L	c.287G>T	Verified	GGC	CGC	ACC	2	G	T
TCGA-29-1784	HIVEP3	ENST00000372583	p.M699I	c.2097G>T	Unverified	ATG	ATG	CAT	3	G	T
TCGA-23-1022	HK2	ENST00000290573	p.G74W	c.220G>T	Verified	gat	ggg	aca	1	G	T
TCGA-61-1998	HLA-E	ENST00000376630	p.W81C	c.243G>T	Unverified	TAT	TGG	GAC	3	G	T
TCGA-13-1481	HLA-G	ENST00000360323	p.D338Y	c.1012G>T	Verified	TCA	GAT	TGA	1	G	T
TCGA-09-2049	HMCN1	ENST00000271588	p.G506C	c.1516G>T	Verified	GCA	GGT	ACT	1	G	T
TCGA-23-1117	HMCN1	ENST00000271588	p.D5165Y	c.15493G>T	Verified	TGT	GAC	AAT	1	G	T
TCGA-23-1117	HMCN1	ENST00000271588	p.D5165Y	c.15493G>T	Verified	TGT	GAC	AAT	1	G	T
TCGA-25-1318	HMCN1	ENST00000271588	p.V4990L	c.14968G>T	Verified	GTT	GTG	AGT	1	G	T
TCGA-25-1318	HMCN1	ENST00000271588	p.V4990L	c.14968G>T	Verified	GTT	GTG	AGT	1	G	T
TCGA-04-1338	HNF1B	ENST00000225893	p.G349*	c.1045G>T	Verified	TCA	GGA	GTG	1	G	T
TCGA-24-1103	HNRNPA3	ENST00000392524	p.G295V	c.884G>T	Verified	CCA	GGA	TAT	2	G	T
TCGA-24-2267	HNRNPA3	ENST00000392524	p.G288V	c.863G>T	Verified	GGG	GGC	TAT	2	G	T

TCGA-29-1781	HNRNPH2	ENST00000316594	p.G149W	c.445G>T	Unverified	cag	ggg	cga	1	G	T
TCGA-13-1498	HNRNPL	ENST00000221419	p.L109L	c.327G>T	Unverified	GGC	CTG	ATT	3	G	T
TCGA-61-2113	HOXA1	ENST00000343060	p.G84G	c.252G>T	Unverified	TCC	GGG	AAC	3	G	T
TCGA-25-2400	HR	ENST00000381418	p.A295S	c.883G>T	Unverified	TGG	GCT	GGG	1	G	T
TCGA-13-0900	HRH1	ENST00000397056	p.A23S	c.67G>T	Verified	ATG	GCC	AGC	1	G	T
TCGA-24-1845	HRH2	ENST00000231683	p.G5C	c.13G>T	Unverified	AAT	GGC	ACA	1	G	T
TCGA-24-1845	HRH2_ENST(	ENST00000377291	p.G5C	c.13G>T	Unverified	AAT	GGC	ACA	1	G	T
TCGA-24-1431	HRNR	ENST00000368801	p.R567S	c.1701G>T	Unverified	GGG	AGG	TCT	3	G	T
TCGA-23-1118	HSD3B1	ENST00000369413	p.A349S	c.1045G>T	Verified	GAA	GCC	AAG	1	G	T
TCGA-29-1769	HSF4	ENST00000264009	p.M212I	c.636G>T	Unverified	CTG	ATG	CTG	3	G	T
TCGA-61-2109	HSPA12B	ENST00000254963	p.S117I	c.350G>T	Unverified	cac	agc	ttt	2	G	T
TCGA-25-2393	HSPA4L	ENST00000508776	p.A754S	c.2260G>T	Verified	aat	gca	cag	1	G	T
TCGA-13-1488	HSPA5	ENST00000324460	p.Q343H	c.1029G>T	Verified	gtc	cag	aaa	3	G	T
TCGA-30-1857	HTR5A	ENST00000287907	p.G110C	c.328G>T	Unverified	CTA	GGT	CGG	1	G	T
TCGA-23-1122	HTT	ENST00000355072	p.?	c.9054+1G>T	Unverified	TAT	AAG	GTG	3	G	T
TCGA-13-0887	HUWE1	XM_497119.1	p.?	c.8767-1G>T	Verified	gaa	gta	ctg	1	G	T
TCGA-61-1900	HUWE1	XM_497119.1	p.V131L	c.391G>T	Unverified	gtg	gtg	ctg	1	G	T
TCGA-24-2289	HUWE1	XM_497119.1	p.Q2206H	c.6618G>T	Unverified	atg	cag	gtt	3	G	T
TCGA-61-1900	HUWE1	XM_497119.1	p.E2930D	c.8790G>T	Unverified	gct	gag	cag	3	G	T
TCGA-61-1900	HUWE1_ENS	ENST00000342160	p.V131L	c.391G>T	Unverified	GTG	GTG	CTG	1	G	T
TCGA-61-1900	HUWE1_ENS	ENST00000342160	p.E3040D	c.9120G>T	Unverified	GCT	GAG	CAG	3	G	T
TCGA-24-1422	IBTK	ENST00000306270	p.V619V	c.1857G>T	Unverified	TTT	GTG	GTA	3	G	T
TCGA-24-1417	ICK	ENST00000350082	p.Q365H	c.1095G>T	Verified	ctc	cag	gag	3	G	T
TCGA-29-1763	ICMT	ENST00000343813	p.S118I	c.353G>T	Unverified	aaa	agt	ctg	2	G	T
TCGA-24-0979	IDO2	ENST00000389060	p.E24D	c.72G>T	Verified	GAA	GAG	TAT	3	G	T
TCGA-09-2044	IFI35	ENST00000438323	p.V173F	c.517G>T	Verified	GAC	GTT	CGG	1	G	T
TCGA-61-2012	IFNA13	ENST00000449498	p.V13L	c.37G>T	Verified	CTG	GTG	GTG	1	G	T
TCGA-24-1422	IFT122	ENST00000296266	p.V819L	c.2455G>T	Verified	GCC	GTG	GAG	1	G	T
TCGA-24-2288	IFT122	ENST00000296266	p.?	c.1300+1G>T	Unverified	AAA	GTT	CGG	1	G	T
TCGA-24-1563	IGDCC3	ENST00000327987	p.G644V	c.1931G>T	Verified	ATC	GGC	ATC	2	G	T
TCGA-61-1737	IGF2R	ENST00000356956	p.A380S	c.1138G>T	Unverified	GCT	GCA	GTT	1	G	T
TCGA-24-2280	IK_ENST000(	ENST00000417647	p.A50S	c.148G>T	Unverified	TCT	GCA	CCA	1	G	T
TCGA-20-1687	IL12RB1	ENST00000322153	p.E266*	c.796G>T	Unverified	CTG	GAG	CTT	1	G	T
TCGA-20-1687	IL12RB1_EN	ENST00000430026	p.E266*	c.796G>T	Unverified	CTG	GAG	CTT	1	G	T

TCGA-09-2044	IL18RAP	ENST00000264260	p.V238F	c.712G>T	Verified	GTT	GTT	CAA	1	G	T
TCGA-29-1777	IL1F8	ENST00000327407	p.A7S	c.19G>T	Unverified	GAG	GCA	GCA	1	G	T
TCGA-25-2391	IL1RN	ENST00000361779	p.K62N	c.186G>T	Unverified	GTC	AAG	TCT	3	G	T
TCGA-24-1844	IL24	ENST00000294984	p.Q29H	c.87G>T	Unverified	ATG	CAG	ATG	3	G	T
TCGA-13-1510	IL27RA	ENST00000263379	p.R514M	c.1541G>T	Unverified	CTG	AGG	TGG	2	G	T
TCGA-29-1777	IL36B	ENST00000259213	p.A7S	c.19G>T	Unverified	GAG	GCA	GCA	1	G	T
TCGA-29-1777	IL36B_ENST(	ENST00000259213	p.A7S	c.19G>T	Unverified	GAG	GCA	GCA	1	G	T
TCGA-24-1844	IMPA2	ENST00000269159	p.R178L	c.533G>T	Unverified	aaa	cgt	gac	2	G	T
TCGA-13-0760	ING2	ENST00000302327	p.W253C	c.759G>T	Verified	AAA	TGG	TAT	3	G	T
TCGA-61-2012	INMT	ENST00000013222	p.E220*	c.658G>T	Unverified	GAG	GAG	GTG	1	G	T
TCGA-24-1470	INPP1	ENST00000392329	p.G187C	c.559G>T	Verified	ATT	GGT	GTC	1	G	T
TCGA-13-1509	INPP4B	NM_003866.1	p.D674Y	c.2020G>T	Verified	agc	gat	gaa	1	G	T
TCGA-29-1781	INPP5B	XM_375718.1	p.K246N	c.738G>T	Unverified	gag	aag	aca	3	G	T
TCGA-29-1781	INPP5B_ENS	ENST00000373026	p.K209N	c.627G>T	Unverified	GAG	AAG	ACA	3	G	T
TCGA-13-0890	INPP5F	ENST00000361976	p.V1055L	c.3163G>T	Verified	cat	gta	act	1	G	T
TCGA-13-1498	INSR_ENST0	ENST00000302850	p.D1293Y	c.3877G>T	Unverified	GAC	GAC	CTG	1	G	T
TCGA-13-0883	INSRR	ENST00000368195	p.S197I	c.590G>T	Verified	TTC	AGC	GGG	2	G	T
TCGA-24-2262	INTS10	ENST00000397977	p.E491D	c.1473G>T	Unverified	CTG	GAG	CAT	3	G	T
TCGA-13-0920	INTS2	ENST00000444766	p.C998F	c.2993G>T	Verified	ATC	TGT	TGT	2	G	T
TCGA-24-1847	INTS9	ENST00000521022	p.V16L	c.46G>T	Unverified	AAT	GTG	CTC	1	G	T
TCGA-09-2051	INVS	ENST00000262457	p.V370F	c.1108G>T	Unverified	CAT	GTC	AGC	1	G	T
TCGA-61-1914	IPO9	ENST00000361565	p.E887*	c.2659G>T	Unverified	GAG	GAG	ATC	1	G	T
TCGA-13-0761	IQSEC2	XM_291345.2	p.V1002L	c.3004G>T	Verified	ctt	gtg	gtc	1	G	T
TCGA-13-0761	IQSEC2	XM_291345.2	p.V1002L	c.3004G>T	Verified	ctt	gtg	gtc	1	G	T
TCGA-24-1431	IQSEC2	XM_291345.2	p.E734D	c.2202G>T	Unverified	cgg	gag	aca	3	G	T
TCGA-13-0887	IQUB	ENST00000324698	p.A10S	c.28G>T	Verified	GAA	GCT	CAG	1	G	T
TCGA-23-1031	IQUB	ENST00000324698	p.V88F	c.262G>T	Verified	CAA	GTT	TCA	1	G	T
TCGA-13-0906	IRAK3	ENST00000261233	p.M314I	c.942G>T	Verified	GCC	ATG	GCA	3	G	T
TCGA-13-0885	IRF9	ENST00000396864	p.R142S	c.426G>T	Verified	GAG	AGG	AAG	3	G	T
TCGA-04-1367	IRS1	ENST00000305123	p.G1154V	c.3461G>T	Verified	cct	ggg	gag	2	G	T
TCGA-13-1501	ITFG1	ENST00000320640	p.G54W	c.160G>T	Verified	TTC	GGG	GAC	1	G	T
TCGA-13-1501	ITFG1	ENST00000320640	p.G54W	c.160G>T	Verified	TTC	GGG	GAC	1	G	T
TCGA-13-0906	ITGA2B	ENST00000262407	p.R889L	c.2666G>T	Verified	gat	cgc	aga	2	G	T
TCGA-13-1488	ITGA4	ENST00000397033	p.G490*	c.1468G>T	Verified	AAT	GGA	TGG	1	G	T

TCGA-24-1850	ITGAE	ENST00000263087	p.S567I	c.1700G>T	Unverified	CTG	AGT	GGG	2	G	T
TCGA-30-1891	ITGAM	ENST00000287497	p.G607G	c.1821G>T	Unverified	cag	ggg	cac	3	G	T
TCGA-13-0923	ITK	ENST00000422843	p.A349S	c.1045G>T	Verified	ACA	GCA	GGG	1	G	T
TCGA-13-0920	ITLN1	ENST00000326245	p.L179L	c.537G>T	Verified	ACA	CTG	GGA	3	G	T
TCGA-20-0991	JMJD8_ENST	ENST00000293882_v62	p.G254G	c.762G>T	Unverified	CCC	GGG	TAC	3	G	T
TCGA-61-1915	KANK1	ENST00000382293	p.V358V	c.1074G>T	Unverified	GTG	GTG	GAG	3	G	T
TCGA-61-1915	KANK1_ENST	ENST00000382303	p.V516V	c.1548G>T	Unverified	GTG	GTG	GAG	3	G	T
TCGA-29-1693	KBTBD5	ENST00000287777	p.E294*	c.880G>T	Unverified	GAG	GAG	GAT	1	G	T
TCGA-09-2050	KBTBD5	ENST00000287777	p.L104F	c.312G>T	Verified	GAT	TTG	TTC	3	G	T
TCGA-24-1845	KCNA4	ENST00000328224	p.V276L	c.826G>T	Unverified	TTT	GTG	AGA	1	G	T
TCGA-13-0887	KCNAB1_EN	ENST00000490337	p.L63L	c.189G>T	Verified	CTG	CTG	CGC	3	G	T
TCGA-25-1313	KCNB2	ENST00000523207	p.L524L	c.1572G>T	Verified	CAG	CTG	AAC	3	G	T
TCGA-13-0904	KCND2	ENST00000331113	p.T234T	c.702G>T	Verified	GAC	ACG	GCC	3	G	T
TCGA-24-2267	KCNE3	ENST00000310128	p.R88L	c.263G>T	Verified	AAG	CGT	AGT	2	G	T
TCGA-13-0760	KCNH1	ENST00000271751	p.Q344H	c.1032G>T	Verified	AGT	CAG	GGC	3	G	T
TCGA-13-0900	KCNH5	ENST00000322893	p.S161I	c.482G>T	Unverified	CGA	AGT	GTT	2	G	T
TCGA-24-2262	KCNH5	ENST00000322893	p.G46V	c.137G>T	Verified	GAC	GGT	TTT	2	G	T
TCGA-04-1342	KCNH6	ENST00000583023	p.A83A	c.249G>T	Unverified	CTA	GCG	CAG	3	G	T
TCGA-24-1616	KCNH8	ENST00000328405	p.D132Y	c.394G>T	Verified	AAA	GAT	ATA	1	G	T
TCGA-29-1761	KCNK2	ENST00000391894	p.?	c.431-1G>T	Unverified	ATA	GGA	TTT	2	G	T
TCGA-29-1761	KCNK2_ENST	ENST00000444842	p.?	c.476-1G>T	Unverified	ATA	GGA	TTT	2	G	T
TCGA-13-0885	KCNK3	ENST00000302909	p.G231V	c.692G>T	Verified	ACG	GGC	CTC	2	G	T
TCGA-29-1785	KCNQ1	ENST00000155840	p.M476I	c.1428G>T	Unverified	AGC	ATG	CCC	3	G	T
TCGA-29-1785	KCNQ1	ENST00000155840	p.M476I	c.1428G>T	Unverified	AGC	ATG	CCC	3	G	T
TCGA-30-1856	KCNU1	ENST00000399881	p.D991Y	c.2971G>T	Unverified	TTA	GAT	CTT	1	G	T
TCGA-30-1856	KCNU1_ENS	ENST00000399881	p.D991Y	c.2971G>T	Unverified	TTA	GAT	CTT	1	G	T
TCGA-61-2102	KCTD18	ENST00000359878	p.R32L	c.95G>T	Verified	TGC	CGC	TTC	2	G	T
TCGA-13-1497	KDM2B	NM_032590.2	p.D863Y	c.2587G>T	Verified	ctg	gac	atc	1	G	T
TCGA-13-1481	KEL	ENST00000355265	p.A473S	c.1417G>T	Verified	GTT	GCT	CAA	1	G	T
TCGA-13-0793	KHNYN	ENST00000251343	p.?	c.1788-1G>T	Verified	GCC	AGG	ACA	3	G	T
TCGA-24-0979	KIAA0232	ENST00000307659	p.A295S	c.883G>T	Verified	GAA	GCA	GGC	1	G	T
TCGA-61-1910	KIAA0319	ENST00000378214	p.E246*	c.736G>T	Unverified	GGA	GAG	GTG	1	G	T
TCGA-61-1914	KIAA0319L	ENST00000335883	p.?	c.666+1G>T	Unverified	GCA	GAG	GTC	3	G	T
TCGA-61-1914	KIAA0319L_	ENST00000325722	p.?	c.666+1G>T	Unverified	GCA	GAG	GTC	3	G	T

TCGA-13-0906	KIAA0430	ENST00000396368	p.G235V	c.704G>T	Unverified	CCA	GGG	CAT	2	G	T
TCGA-29-1777	KIAA0467_E	ENST00000372442	p.D634Y	c.1900G>T	Unverified	TCA	GAC	GTA	1	G	T
TCGA-29-1781	KIAA0556	ENST00000261588	p.S890I	c.2669G>T	Unverified	CGC	AGT	GAG	2	G	T
TCGA-24-1417	KIAA0556	ENST00000261588	p.V1520V	c.4560G>T	Verified	CTG	GTG	GAC	3	G	T
TCGA-24-2262	KIAA0556	ENST00000261588	p.V1497V	c.4491G>T	Verified	ACC	GTG	TCA	3	G	T
TCGA-29-1781	KIAA0556_E	ENST00000261588	p.S890I	c.2669G>T	Unverified	CGC	AGT	GAG	2	G	T
TCGA-13-1510	KIAA1024	ENST00000305428	p.V536L	c.1606G>T	Unverified	GGA	GTG	ATA	1	G	T
TCGA-20-1683	KIAA1210	XM_172801.3	p.R988M	c.2963G>T	Unverified	aag	agg	tct	2	G	T
TCGA-25-2401	KIAA1217	ENST00000376454	p.E795D	c.2385G>T	Verified	AAG	GAG	GAG	3	G	T
TCGA-09-2050	KIAA1244	NM_020340.2	p.Q41H	c.123G>T	Verified	ctg	cag	gtg	3	G	T
TCGA-13-1497	KIAA1274	ENST00000263563	p.W453C	c.1359G>T	Verified	CGC	TGG	CTG	3	G	T
TCGA-61-1906	KIAA1310	ENST00000431828	p.?	c.1933+1G>T	Unverified	GAA	GCT	GCA	1	G	T
TCGA-61-1906	KIAA1310	ENST00000431828	p.?	c.1933+1G>T	Unverified	GAA	GCT	GCA	1	G	T
TCGA-29-1696	KIAA1409	ENST00000256339	p.C623F	c.1868G>T	Unverified	AAT	TGT	TTC	2	G	T
TCGA-29-1696	KIAA1409	ENST00000256339	p.C623F	c.1868G>T	Unverified	AAT	TGT	TTC	2	G	T
TCGA-24-1464	KIAA1462	ENST00000375377	p.E577*	c.1729G>T	Verified	AAC	GAG	ACT	1	G	T
TCGA-20-0990	KIAA1468	ENST00000398130	p.V324V	c.972G>T	Verified	GAT	GTG	GCC	3	G	T
TCGA-29-1696	KIAA1543	ENST00000160298	p.C1167F	c.3500G>T	Unverified	AGC	TGC	CAG	2	G	T
TCGA-29-1696	KIAA1543	ENST00000160298	p.C1167F	c.3500G>T	Unverified	AGC	TGC	CAG	2	G	T
TCGA-09-2056	KIAA1609	ENST00000343629	p.V138L	c.412G>T	Verified	GTG	GTG	CAC	1	G	T
TCGA-24-2267	KIAA1614	ENST00000367588	p.V339L	c.1015G>T	Unverified	GAT	GTG	GAC	1	G	T
TCGA-13-0900	KIAA1614	ENST00000367588	p.G285V	c.854G>T	Verified	CTG	GGC	GCT	2	G	T
TCGA-24-1422	KIAA1614	ENST00000367588	p.R1061L	c.3182G>T	Verified	CGT	CGG	AAA	2	G	T
TCGA-61-1910	KIAA1614	ENST00000367588	p.V96V	c.288G>T	Unverified	ACA	GTG	GCC	3	G	T
TCGA-23-1022	KIAA1797	ENST00000380249	p.G792W	c.2374G>T	Verified	CGT	GGG	ATA	1	G	T
TCGA-61-1910	KIF16B	ENST00000354981	p.V1111F	c.3331G>T	Unverified	ctg	gtt	ccc	1	G	T
TCGA-61-1910	KIF16B_ENS	ENST00000408042	p.V1111F	c.3331G>T	Unverified	CTG	GTT	CCC	1	G	T
TCGA-24-1845	KIF16B_ENS	ENST00000408042	p.C1184F	c.3551G>T	Unverified	GCC	TGC	GCT	2	G	T
TCGA-23-1116	KIF1A	ENST00000320389	p.G997C	c.2989G>T	Unverified	CTG	GGC	AAC	1	G	T
TCGA-29-1777	KIF1A	ENST00000320389	p.W57L	c.170G>T	Unverified	TAC	TGG	TCG	2	G	T
TCGA-13-0761	KIF1B	ENST00000263934	p.C853F	c.2558G>T	Verified	GGC	TGT	GTG	2	G	T
TCGA-13-0761	KIF1B	ENST00000263934	p.C853F	c.2558G>T	Verified	GGC	TGT	GTG	2	G	T
TCGA-13-0904	KIF25	ENST00000354419	p.W3C	c.9G>T	Verified	ACA	TGG	ACC	3	G	T
TCGA-61-2009	KIF3B	ENST00000375712	p.E609*	c.1825G>T	Verified	GAG	GAA	GAT	1	G	T

TCGA-24-1463	KIF4B	ENST00000435029	p.G748V	c.2243G>T	Unverified	CTT	GGA	AAT	2	G	T
TCGA-13-0755	KIF4B_ENST	ENST00000435029	p.G283C	c.847G>T	Verified	AAG	GGT	AGC	1	G	T
TCGA-24-1463	KIF4B_ENST	ENST00000435029	p.G748V	c.2243G>T	Unverified	CTT	GGA	AAT	2	G	T
TCGA-13-0761	KIF6	ENST00000287152	p.L275F	c.825G>T	Unverified	AAC	TTG	TCA	3	G	T
TCGA-13-0761	KIF6	ENST00000287152	p.L275F	c.825G>T	Unverified	AAC	TTG	TCA	3	G	T
TCGA-24-2290	KIRREL3	ENST00000278934	p.E420D	c.1260G>T	Verified	CTG	GAG	TCG	3	G	T
TCGA-24-2290	KIRREL3	ENST00000278934	p.E420D	c.1260G>T	Verified	CTG	GAG	TCG	3	G	T
TCGA-24-1423	KLF15	ENST00000296233	p.G327C	c.979G>T	Unverified	CCT	GGC	TGC	1	G	T
TCGA-09-2049	KLF17	ENST00000372299	p.Q242H	c.726G>T	Verified	AGT	CAG	CCA	3	G	T
TCGA-29-1711	KLF5	ENST00000377687	p.S133I	c.398G>T	Unverified	TAC	AGT	ATC	2	G	T
TCGA-29-1711	KLF5	ENST00000377687	p.S133I	c.398G>T	Unverified	TAC	AGT	ATC	2	G	T
TCGA-23-1031	KLF5	ENST00000377687	p.M281I	c.843G>T	Verified	GGC	ATG	CCC	3	G	T
TCGA-61-2095	KLHL14	ENST00000359358	p.E558D	c.1674G>T	Unverified	TTG	GAG	GGT	3	G	T
TCGA-29-1784	KLHL8	ENST00000273963	p.R404L	c.1211G>T	Unverified	AGG	CGA	GGA	2	G	T
TCGA-36-1577	KLK10	ENST00000391805	p.G106V	c.317G>T	Unverified	cag	gga	gag	2	G	T
TCGA-24-1844	KLRG1	ENST00000356986	p.W132L	c.395G>T	Unverified	TGC	TGG	ATT	2	G	T
TCGA-24-2288	KNCN	ENST00000481882	p.L13L	c.39G>T	Unverified	GGC	CTG	CAG	3	G	T
TCGA-10-0930	KNTC1	ENST00000333479	p.W539L	c.1616G>T	Verified	tct	tgg	att	2	G	T
TCGA-10-0930	KNTC1	ENST00000333479	p.W539L	c.1616G>T	Verified	tct	tgg	att	2	G	T
TCGA-24-2035	KRT4	ENST00000293774	p.E465*	c.1393G>T	Verified	GGT	GAG	AAT	1	G	T
TCGA-13-1497	KRT4	ENST00000293774	p.S560I	c.1679G>T	Verified	AGT	AGT	GGC	2	G	T
TCGA-13-1501	KRT6A	ENST00000330722	p.S540I	c.1619G>T	Verified	CTC	AGC	TCT	2	G	T
TCGA-13-1501	KRT6A	ENST00000330722	p.S540I	c.1619G>T	Verified	CTC	AGC	TCT	2	G	T
TCGA-04-1655	KRT78	ENST00000304620	p.D192Y	c.574G>T	Unverified	CGG	GAC	CAG	1	G	T
TCGA-09-2050	KRT78	ENST00000304620	p.E291*	c.871G>T	Verified	GAG	GAG	ATC	1	G	T
TCGA-61-2095	KSR1_ENST(	ENST00000319524	p.?	c.2157+1G>T	Unverified	ATC	AAG	GGC	3	G	T
TCGA-13-0906	KTI12	ENST00000371614	p.A288S	c.862G>T	Unverified	GAA	GCG	CAG	1	G	T
TCGA-13-0886	L3MBTL3	ENST00000368136	p.A351S	c.1051G>T	Verified	AAA	GCT	CAA	1	G	T
TCGA-24-0979	LAMA1	ENST00000389658	p.D691Y	c.2071G>T	Verified	CTG	GAC	ATA	1	G	T
TCGA-13-0900	LAMA2	ENST00000421865	p.R369L	c.1106G>T	Verified	ATA	CGT	GGA	2	G	T
TCGA-61-2012	LAMA2	ENST00000421865	p.R1366L	c.4097G>T	Verified	GGA	CGT	GGA	2	G	T
TCGA-13-0924	LAMA3	ENST00000313654	p.S2834I	c.8501G>T	Verified	ggc	agc	cca	2	G	T
TCGA-23-1118	LAMB3	ENST00000367030	p.E1065*	c.3193G>T	Verified	GCG	GAA	GGT	1	G	T
TCGA-29-1775	LAMC3	ENST00000361069	p.V654F	c.1960G>T	Unverified	GAG	GTC	CGG	1	G	T

TCGA-13-0791	LAMP2	ENST00000371335	p.?	c.556+1G>T	Verified	aat	gag	ttc	1	G	T
TCGA-09-2050	LARP1	ENST00000377643	p.?	c.2233+1G>T	Verified	CAA	GTT	CCT	1	G	T
TCGA-13-0904	LASS3	ENST00000284382	p.E114*	c.340G>T	Verified	GTG	GAA	AGA	1	G	T
TCGA-29-1762	LCE1F	ENST00000334371	p.G64C	c.190G>T	Unverified	GGG	GGT	GGT	1	G	T
TCGA-04-1347	LCT	ENST00000264162	p.V971L	c.2911G>T	Verified	AAG	GTG	AAG	1	G	T
TCGA-20-0990	LCT	ENST00000264162	p.G910V	c.2729G>T	Verified	TGG	GGC	GTG	2	G	T
TCGA-13-0885	LDLRAP1	ENST00000374338	p.L31L	c.93G>T	Verified	AAG	CTG	CCT	3	G	T
TCGA-29-1777	LEF1	ENST00000265165	p.?	c.280+1G>T	Unverified	gac	gga	aag	1	G	T
TCGA-29-1777	LEF1_ENST0	ENST00000510624	p.?	c.76+1G>T	Unverified	GAC	GGA	AAG	1	G	T
TCGA-24-1469	LGALS3BP	ENST00000262776	p.D262Y	c.784G>T	Verified	CTG	GAC	CTG	1	G	T
TCGA-24-1431	LGR4	ENST00000379214	p.R941L	c.2822G>T	Verified	GTG	CGC	TAT	2	G	T
TCGA-61-1913	LGR6	ENST00000367278	p.L816L	c.2448G>T	Unverified	GTG	CTG	CCC	3	G	T
TCGA-24-2289	LIAS	ENST00000261434	p.A222S	c.664G>T	Unverified	AAA	GCA	ATA	1	G	T
TCGA-23-1021	LINS	ENST00000314742	p.V15L	c.43G>T	Verified	AAG	GTA	CTT	1	G	T
TCGA-13-0795	LL0XNC01-2	ENST00000218249	p.T70T	c.210G>T	Verified	gat	acg	tcg	3	G	T
TCGA-24-0975	LMAN1	ENST00000251047	p.G71G	c.213G>T	Unverified	GCG	GGG	AAT	3	G	T
TCGA-20-0990	LMTK3_ENS	ENST00000270238	p.A1484S	c.4450G>T	Unverified	CCC	GCA	GGC	1	G	T
TCGA-13-0883	LNPEP	ENST00000231368	p.E40*	c.118G>T	Verified	cta	gag	cct	1	G	T
TCGA-13-0923	LNPEP	ENST00000231368	p.R515L	c.1544G>T	Verified	gct	cga	ttt	2	G	T
TCGA-61-1899	LOC347421	XM_498372.1	p.G37C	c.109G>T	Unverified	ggg	ggt	acc	1	G	T
TCGA-30-1857	LOC388972	XM_371511.3	p.A199S	c.595G>T	Unverified	tct	gct	gca	1	G	T
TCGA-24-1422	LOC51059	ENST00000395297	p.V12L	c.34G>T	Verified	TCG	GTA	GAG	1	G	T
TCGA-24-1463	LOC651503	ENST00000377164	p.C110F	c.329G>T	Unverified	GAA	TGT	ATG	2	G	T
TCGA-13-0883	LOC728378	ENST00000357462	p.K100N	c.300G>T	Verified	GGC	AAG	TGG	3	G	T
TCGA-29-1695	LONRF2	ENST00000393437	p.E381*	c.1141G>T	Unverified	TTT	GAA	GAG	1	G	T
TCGA-29-1699	LPA	ENST00000316300	p.R820S	c.2460G>T	Unverified	CAG	AGG	CCT	3	G	T
TCGA-24-1470	LPAR4	ENST00000435339	p.D307Y	c.919G>T	Verified	ttt	gac	cct	1	G	T
TCGA-24-1463	LPIN3	ENST00000373257	p.D336Y	c.1006G>T	Verified	GGG	GAC	ATG	1	G	T
TCGA-09-2050	LRFN5	ENST00000298119	p.E717*	c.2149G>T	Verified	CTG	GAG	TTA	1	G	T
TCGA-61-1914	LRIG1	AF381545.1	p.A984S	c.2950G>T	Unverified	act	gcc	gct	1	G	T
TCGA-13-0714	LRP1	ENST00000243077	p.G4316V	c.12947G>T	Unverified	TTT	GGC	ACA	2	G	T
TCGA-61-1907	LRP1	ENST00000243077	p.C2800F	c.8399G>T	Unverified	GAC	TGT	GCT	2	G	T
TCGA-09-1665	LRP1	ENST00000243077	p.V4081V	c.12243G>T	Verified	ATT	GTG	GCT	3	G	T
TCGA-30-1857	LRP10	ENST00000359591	p.G168V	c.503G>T	Unverified	GAT	GGC	TCT	2	G	T

TCGA-29-1783	LRRC2	ENST00000296144	p.E52*	c.154G>T	Unverified	GCC	GAA	TGC	1	G	T
TCGA-13-1497	LRRC30	ENST00000383467	p.E143*	c.427G>T	Verified	CTG	GAG	GTC	1	G	T
TCGA-24-1422	LRRC33	ENST00000328557	p.R645R	c.1935G>T	Unverified	GAG	CGG	CTG	3	G	T
TCGA-23-1110	LRRC52	ENST00000294818	p.G10G	c.30G>T	Verified	CCT	GGG	TGG	3	G	T
TCGA-20-0990	LRRC55	ENST00000497933	p.E133D	c.399G>T	Unverified	ATG	GAG	CTC	3	G	T
TCGA-13-1497	LRRC7_ENST	ENST00000370958	p.R23L	c.68G>T	Verified	GTT	CGT	GCA	2	G	T
TCGA-13-0760	LRRK2	SU_LRRK2	p.V2163F	c.6487G>T	Verified	atg	gtt	gct	1	G	T
TCGA-13-0760	LRRK2_ENST	ENST00000298910	p.V2156F	c.6466G>T	Verified	ATG	GTT	GCT	1	G	T
TCGA-24-1843	LRRN3	ENST00000308478	p.V233F	c.697G>T	Unverified	TTG	GTT	GGA	1	G	T
TCGA-04-1356	LRRTM1	ENST00000295057	p.S251S	c.753G>T	Verified	AGC	TCG	CTG	3	G	T
TCGA-29-1699	LTA4H	ENST00000228740	p.V196F	c.586G>T	Unverified	AAA	GTT	CCA	1	G	T
TCGA-29-1763	LYPLA1	ENST00000316963	p.V186V	c.558G>T	Unverified	ACG	GTG	GAA	3	G	T
TCGA-23-1123	LYST	ENST00000389793	p.Q1208H	c.3624G>T	Verified	TCT	CAG	TGT	3	G	T
TCGA-23-1123	LYST	ENST00000389793	p.Q1208H	c.3624G>T	Verified	TCT	CAG	TGT	3	G	T
TCGA-30-1891	MAD1L1	AF083811.1	p.L269L	c.807G>T	Unverified	cac	ctg	cgg	3	G	T
TCGA-29-1711	MAGEB3	ENST00000378986	p.V210L	c.628G>T	Unverified	ggc	gtg	atc	1	G	T
TCGA-29-1711	MAGEB3	ENST00000378986	p.V210L	c.628G>T	Unverified	ggc	gtg	atc	1	G	T
TCGA-20-1683	MAGEC1	ENST00000285879	p.Q582H	c.1746G>T	Unverified	cct	cag	agc	3	G	T
TCGA-20-1686	MAGED2	ENST00000375068	p.G403V	c.1208G>T	Unverified	cct	ggg	ata	2	G	T
TCGA-20-1685	MAN2A2	ENST00000360468	p.G1150V	c.3449G>T	Unverified	TTG	GGT	TAG	2	G	T
TCGA-24-2289	MAN2A2	ENST00000360468	p.E244D	c.732G>T	Unverified	CTG	GAG	ATT	3	G	T
TCGA-23-2077	MAN2B1	ENST00000221363	p.V279V	c.837G>T	Verified	CTG	GTG	GAG	3	G	T
TCGA-61-2094	MAP1A	ENST00000300231	p.G1052G	c.3156G>T	Unverified	CCA	GGG	CCT	3	G	T
TCGA-29-1711	MAP1S	ENST00000324096	p.G815V	c.2444G>T	Unverified	GAG	GGC	TTT	2	G	T
TCGA-29-1711	MAP1S	ENST00000324096	p.G815V	c.2444G>T	Unverified	GAG	GGC	TTT	2	G	T
TCGA-61-1906	MAP2	ENST00000360351	p.A341S	c.1021G>T	Unverified	TTT	GCC	CCT	1	G	T
TCGA-61-1906	MAP2	ENST00000360351	p.A341S	c.1021G>T	Unverified	TTT	GCC	CCT	1	G	T
TCGA-29-1770	MAP3K13	ENST00000424227	p.E323*	c.967G>T	Unverified	CCA	GAG	GTG	1	G	T
TCGA-29-1770	MAP3K13_E	ENST00000424227	p.E323*	c.967G>T	Unverified	CCA	GAG	GTG	1	G	T
TCGA-04-1338	MAP3K7	ENST00000369332	p.G45V	c.134G>T	Verified	aga	gga	gcc	2	G	T
TCGA-61-1907	MAP4	ENST00000360240	p.D604Y	c.1810G>T	Unverified	GAG	GAT	TCC	1	G	T
TCGA-61-1907	MAP4_ENST	ENST00000395734_v68	p.D604Y	c.1810G>T	Unverified	GAG	GAT	TCC	1	G	T
TCGA-13-0883	MAP9	ENST00000311277	p.D325Y	c.973G>T	Verified	GAT	GAT	GAC	1	G	T
TCGA-29-1785	MAPRE2	ENST00000300249	p.?	c.396+1G>T	Unverified	gat	aag	gta	3	G	T

TCGA-29-1785	MAPRE2	ENST00000300249	p.?	c.396+1G>T	Unverified	gat	aag	gta	3	G	T
TCGA-09-2050	MARCO	ENST00000327097	p.R332L	c.995G>T	Verified	GGG	CGA	GCA	2	G	T
TCGA-23-1120	MARK4	ENST00000300843	p.E350D	c.1050G>T	Unverified	AAA	GAG	TCC	3	G	T
TCGA-04-1342	MARS2	ENST00000282276	p.R489S	c.1467G>T	Unverified	CAA	AGG	CAT	3	G	T
TCGA-23-1110	MASP1	ENST00000337774	p.V240V	c.720G>T	Verified	GAG	GTG	CCC	3	G	T
TCGA-29-1691	MAST2_ENS	ENST00000361297	p.R530R	c.1590G>T	Unverified	GTG	CGG	CAC	3	G	T
TCGA-04-1638	MAST4_ENS	ENST00000403625	p.G1236V	c.3707G>T	Unverified	ACT	GGA	CCA	2	G	T
TCGA-13-1507	MATN2	ENST00000520016	p.E743*	c.2227G>T	Verified	TTT	GAG	AGA	1	G	T
TCGA-24-0979	MBD5	ENST00000407073	p.D1082Y	c.3244G>T	Verified	GGT	GAT	GCT	1	G	T
TCGA-30-1856	MBNL1	ENST00000282486	p.A132S	c.394G>T	Unverified	GCC	GCC	TTT	1	G	T
TCGA-30-1856	MBNL1_ENS	ENST00000324196	p.A132S	c.394G>T	Unverified	GCC	GCC	TTT	1	G	T
TCGA-23-1110	MBTD1	ENST00000393180	p.G249V	c.746G>T	Verified	GAC	GGA	TTC	2	G	T
TCGA-13-0900	MCM10	ENST00000361282	p.S237I	c.710G>T	Verified	GGA	AGT	TCT	2	G	T
TCGA-13-1499	MDN1	ENST00000369393	p.G2433V	c.7298G>T	Verified	ATG	GGA	CTG	2	G	T
TCGA-13-0893	MED26	ENST00000263390	p.V256V	c.768G>T	Verified	AGG	GTG	GAC	3	G	T
TCGA-24-1104	MED4	ENST00000258648	p.G60V	c.179G>T	Verified	gct	gga	gag	2	G	T
TCGA-04-1530	MEF2C	ENST00000514028	p.D432Y	c.1294G>T	Unverified	tac	gac	ggg	1	G	T
TCGA-24-2289	MEFV	ENST00000219596	p.S312I	c.935G>T	Unverified	GCG	AGT	CCC	2	G	T
TCGA-61-2102	MEOX2	ENST00000262041	p.A143A	c.429G>T	Verified	GCC	GCG	TGC	3	G	T
TCGA-30-1855	MEP1B	ENST00000269202	p.Q631H	c.1893G>T	Unverified	TGC	CAG	TCA	3	G	T
TCGA-13-0920	METTL19	ENST00000285635	p.R45M	c.134G>T	Verified	GCC	AGG	TCT	2	G	T
TCGA-23-1114	MFN2	ENST00000235329	p.W626L	c.1877G>T	Unverified	GTG	TGG	AAG	2	G	T
TCGA-23-1114	MFN2_ENST	ENST00000376337	p.W324L	c.971G>T	Unverified	GTG	TGG	AAG	2	G	T
TCGA-23-1124	MFSD11	ENST00000336509	p.G422V	c.1265G>T	Verified	TTT	GGG	TTT	2	G	T
TCGA-13-0903	MFSD2B	ENST00000406420	p.A93S	c.277G>T	Verified	GCG	GCT	GCT	1	G	T
TCGA-29-1762	MFSD6	ENST00000392328	p.M379I	c.1137G>T	Unverified	CTC	ATG	ACC	3	G	T
TCGA-13-1512	MGC99813	ENST00000373883	p.V113L	c.337G>T	Verified	CTA	GTG	CGC	1	G	T
TCGA-13-0887	MGEA5	ENST00000361464	p.E898*	c.2692G>T	Verified	TTT	GAA	ATT	1	G	T
TCGA-09-2051	MGST1	ENST00000396207	p.L95F	c.285G>T	Unverified	TCC	TTG	AGT	3	G	T
TCGA-04-1342	MICALL1	ENST00000215957	p.E346*	c.1036G>T	Unverified	CAC	GAA	CTG	1	G	T
TCGA-04-1655	MKNK1	ENST00000371946	p.R32R	c.96G>T	Unverified	aag	cgg	agg	3	G	T
TCGA-04-1331	MKX	ENST00000375790	p.G180V	c.539G>T	Verified	GAA	GGG	GGC	2	G	T
TCGA-13-0885	MLL4	ENST00000222270	p.V2041V	c.6123G>T	Verified	CCT	GTG	ACT	3	G	T
TCGA-23-1124	MLXIP	ENST00000319080	p.W832L	c.2495G>T	Verified	AAT	TGG	AAG	2	G	T

TCGA-04-1342	MMEL1	ENST00000288709	p.Q581H	c.1743G>T	Unverified	CTC	CAG	CCC	3	G	T
TCGA-61-1915	MMP13	ENST00000260302	p.A385S	c.1153G>T	Unverified	gca	gct	gtt	1	G	T
TCGA-10-0930	MMP8	ENST00000236826	p.R292L	c.875G>T	Verified	ctc	cgt	gga	2	G	T
TCGA-10-0930	MMP8	ENST00000236826	p.R292L	c.875G>T	Verified	ctc	cgt	gga	2	G	T
TCGA-13-0904	MMRN1	ENST00000394980	p.E64*	c.190G>T	Verified	GCG	GAG	ATA	1	G	T
TCGA-24-2289	MMRN1	ENST00000394980	p.V168F	c.502G>T	Unverified	GGC	GTT	GGA	1	G	T
TCGA-23-1120	MOCS3	ENST00000244051	p.V176L	c.526G>T	Unverified	GTG	GTG	GCT	1	G	T
TCGA-23-1110	MORC1	ENST00000232603	p.?	c.223+1G>T	Verified	CCT	GAG	GAA	1	G	T
TCGA-13-1509	MOV10L1	ENST00000262794	p.V570F	c.1708G>T	Verified	GAG	GTC	CCA	1	G	T
TCGA-13-0923	MPDZ	NM_003829.1	p.G100V	c.299G>T	Verified	aat	ggg	aat	2	G	T
TCGA-24-2267	MPDZ	NM_003829.1	p.G777G	c.2331G>T	Verified	tca	ggg	act	3	G	T
TCGA-09-2050	MPG	ENST00000219431	p.D211Y	c.631G>T	Verified	AAG	GAC	CGC	1	G	T
TCGA-23-1124	MPI	ENST00000352410	p.G265V	c.794G>T	Verified	CCT	GGG	GAG	2	G	T
TCGA-29-1766	MPP4	ENST00000409474	p.E314*	c.940G>T	Unverified	CGG	GAA	TTC	1	G	T
TCGA-29-1766	MPP4_ENST	ENST00000409474	p.E314*	c.940G>T	Unverified	CGG	GAA	TTC	1	G	T
TCGA-24-2035	MPRIP	ENST00000341712	p.P522P	c.1566G>T	Unverified	GAG	CCG	GAC	3	G	T
TCGA-23-1122	MRGPRX3	ENST00000396275	p.V190F	c.568G>T	Verified	GTG	GTT	CTC	1	G	T
TCGA-13-0885	MRPL30	ENST00000338148	p.M134I	c.402G>T	Verified	AAC	ATG	TCT	3	G	T
TCGA-24-0979	MRPL49	ENST00000279242	p.L132L	c.396G>T	Unverified	CTG	CTG	GGG	3	G	T
TCGA-25-1313	MRPS18B	ENST00000259873	p.G132C	c.394G>T	Verified	ACG	GGT	ATC	1	G	T
TCGA-09-2056	MS4A1	ENST00000389939	p.G68V	c.203G>T	Verified	GGG	GGT	CTT	2	G	T
TCGA-13-0885	MS4A1	ENST00000389939	p.G60G	c.180G>T	Verified	AAT	GGG	CTC	3	G	T
TCGA-24-1435	MS4A15_EN	ENST00000405633	p.L60F	c.180G>T	Verified	GAC	TTG	CGG	3	G	T
TCGA-13-0906	MS4A3	ENST00000278865	p.G95C	c.283G>T	Verified	TGG	GGT	GCT	1	G	T
TCGA-13-0903	MSH4	ENST00000263187	p.E742*	c.2224G>T	Verified	aaa	gag	ata	1	G	T
TCGA-20-0990	MSH5	NM_025259.3	p.M583I	c.1749G>T	Verified	ctg	atg	gaa	3	G	T
TCGA-24-1464	MSH5_ENST	ENST00000375742	p.?	c.863+1G>T	Verified	CTC	AGG	CTA	2	G	T
TCGA-13-0883	MST1R	ENST00000296474	p.V977F	c.2929G>T	Verified	CTG	GTC	TTC	1	G	T
TCGA-09-1665	MST1R	ENST00000296474	p.R669R	c.2007G>T	Verified	TTC	CGG	GTA	3	G	T
TCGA-29-1775	MSTN	ENST00000260950	p.T115T	c.345G>T	Unverified	aca	acg	gaa	3	G	T
TCGA-30-1891	MT1B	ENST00000334346	p.A14S	c.40G>T	Unverified	TGT	GCC	TGC	1	G	T
TCGA-25-2400	MTA2	ENST00000278823	p.E113D	c.339G>T	Verified	AAT	GAG	ACA	3	G	T
TCGA-23-1122	MTFMT	ENST00000220058	p.D326Y	c.976G>T	Verified	AAG	GAT	GGT	1	G	T
TCGA-13-0893	MTHFD2L	ENST00000325278	p.S27I	c.80G>T	Verified	CTC	AGT	ATA	2	G	T

TCGA-24-0979	MTIF2	ENST00000394600	p.D350Y	c.1048G>T	Verified	GCA	GAT	CCC	1	G	T
TCGA-25-2398	MTIF3	ENST00000381116	p.L161L	c.483G>T	Verified	GAA	CTG	ATT	3	G	T
TCGA-24-1604	MTMR12	ENST00000382142	p.L215L	c.645G>T	Unverified	gaa	ctg	gaa	3	G	T
TCGA-61-1907	MTOR	ENST00000361445	p.V1461L	c.4381G>T	Unverified	ctt	gtg	gcc	1	G	T
TCGA-04-1342	MTRR	ENST00000264668	p.R612S	c.1836G>T	Verified	GAT	AGG	GAT	3	G	T
TCGA-23-1032	MTUS2_ENS	ENST00000431530	p.R160M	c.479G>T	Verified	AAA	AGG	GAT	2	G	T
TCGA-24-2024	MUC16	ENST00000397910	p.W1384C	c.4152G>T	Verified	GCA	TGG	ACC	3	G	T
TCGA-61-2012	MUC16	ENST00000397910	p.L3262L	c.9786G>T	Verified	CAC	CTG	CCC	3	G	T
TCGA-23-1120	MUC17	ENST00000306151	p.E4380*	c.13138G>T	Verified	GGG	GAG	ACC	1	G	T
TCGA-61-1913	MUC17	ENST00000306151	p.S1763I	c.5288G>T	Unverified	CTC	AGT	TCT	2	G	T
TCGA-61-2102	MUC17	ENST00000306151	p.G1265V	c.3794G>T	Verified	GAA	GGT	ACC	2	G	T
TCGA-29-1695	MUC20	ENST00000447234	p.R649S	c.1947G>T	Unverified	ACG	AGG	CCG	3	G	T
TCGA-29-1695	MUC20_ENS	ENST00000381954	p.R460S	c.1380G>T	Unverified	ACG	AGG	CCG	3	G	T
TCGA-25-1313	MUC4	ENST00000346145	p.E395*	c.1183G>T	Unverified	CGT	GAA	GGC	1	G	T
TCGA-25-1313	MUC4_ENST	ENST00000405167	p.E4503*	c.13507G>T	Unverified	CGT	GAA	GGC	1	G	T
TCGA-25-2401	MX2	ENST00000330714	p.E217D	c.651G>T	Verified	CCT	GAG	GTT	3	G	T
TCGA-13-1496	MXRA5	ENST00000381114	p.E325D	c.975G>T	Verified	GAC	GAG	CAC	3	G	T
TCGA-61-2094	MYBBP1A	ENST00000254718	p.L193F	c.579G>T	Unverified	ACA	TTG	CAG	3	G	T
TCGA-04-1337	MYH1	ENST00000226207	p.D1656Y	c.4966G>T	Verified	AAG	GAT	ACC	1	G	T
TCGA-09-2044	MYH14	ENST00000301415	p.V1149L	c.3445G>T	Unverified	CGT	GTG	GCC	1	G	T
TCGA-29-1777	MYH2	ENST00000245503	p.E1078*	c.3232G>T	Unverified	AAT	GAG	AAA	1	G	T
TCGA-13-0885	MYH4	ENST00000255381	p.E1709D	c.5127G>T	Verified	CAA	GAG	CTT	3	G	T
TCGA-13-1510	MYH4	ENST00000255381	p.K1899N	c.5697G>T	Verified	GCC	AAG	TTC	3	G	T
TCGA-61-1737	MYH9	ENST00000216181	p.A420S	c.1258G>T	Unverified	AAG	GCG	ACC	1	G	T
TCGA-13-1496	MYO18B	ENST00000335473	p.R1254L	c.3761G>T	Verified	CTG	CGT	CTG	2	G	T
TCGA-30-1856	MYO3A	ENST00000265944	p.E291D	c.873G>T	Unverified	ATT	GAG	GGC	3	G	T
TCGA-29-1764	MYO7A	ENST00000409709	p.V1918L	c.5752G>T	Unverified	GAA	GTG	GAG	1	G	T
TCGA-24-1463	MYO7A	ENST00000409709	p.G1191V	c.3572G>T	Verified	CGG	GGC	TGG	2	G	T
TCGA-23-1031	MYO7B	ENST00000272666	p.V1458L	c.4372G>T	Unverified	CAG	GTG	CGG	1	G	T
TCGA-30-1891	MYOC	ENST00000037502	p.W373L	c.1118G>T	Unverified	tct	tgg	ggt	2	G	T
TCGA-13-0923	MYOM1	ENST00000356443	p.R31L	c.92G>T	Unverified	CAG	CGG	GAG	2	G	T
TCGA-23-1114	MYOZ1	ENST00000359322	p.G146C	c.436G>T	Unverified	GGT	GGT	CCC	1	G	T
TCGA-24-2035	MYRIP	ENST00000302541	p.Q57H	c.171G>T	Verified	CAC	CAG	CAG	3	G	T
TCGA-13-1489	MYST4	ENST00000287239	p.S1618I	c.4853G>T	Verified	CCA	AGT	GTC	2	G	T

TCGA-25-2042	NARF	NM_031968.1	p.E238*	c.712G>T	Unverified	cag	gaa	agc	1	G	T
TCGA-13-0760	NAT14	ENST00000205194	p.M158I	c.474G>T	Unverified	GGC	ATG	GGG	3	G	T
TCGA-29-1703	NAV2	ENST00000360655	p.?	c.4900+1G>T	Unverified	GAA	GAA	TCA	1	G	T
TCGA-23-1124	NCAPD3	ENST00000534548	p.?	c.4174+1G>T	Verified	cag	gtg	tct	1	G	T
TCGA-61-1738	NCK2	ENST00000233154	p.R28R	c.84G>T	Unverified	GAG	CGG	CTG	3	G	T
TCGA-61-1738	NCK2	ENST00000233154	p.R28R	c.84G>T	Unverified	GAG	CGG	CTG	3	G	T
TCGA-61-1738	NCK2_ENST(	ENST00000233154	p.R28R	c.84G>T	Unverified	GAG	CGG	CTG	3	G	T
TCGA-61-1738	NCK2_ENST(	ENST00000233154	p.R28R	c.84G>T	Unverified	GAG	CGG	CTG	3	G	T
TCGA-61-1915	NCKAP1L	ENST00000293373	p.R102R	c.306G>T	Unverified	TTT	CGG	GAT	3	G	T
TCGA-09-2051	NCOA1	ENST00000406961	p.G131W	c.391G>T	Unverified	GAA	GGG	AGA	1	G	T
TCGA-13-0904	NCOA3	ENST00000371998	p.D566Y	c.1696G>T	Verified	cag	gat	tcc	1	G	T
TCGA-13-0714	NDRG1	ENST00000323851	p.V17L	c.49G>T	Verified	ttg	gtg	gag	1	G	T
TCGA-29-1691	NDST2	ENST00000309979	p.M526I	c.1578G>T	Unverified	TTT	ATG	ACC	3	G	T
TCGA-29-1763	NDST2	ENST00000309979	p.K860N	c.2580G>T	Unverified	TCG	AAG	CTG	3	G	T
TCGA-29-1766	NEB	ENST00000409198	p.Q3994H	c.11982G>T	Unverified	ATC	CAG	GCT	3	G	T
TCGA-29-1766	NEB_ENST0(	ENST00000397345	p.Q5695H	c.17085G>T	Unverified	ATC	CAG	GCT	3	G	T
TCGA-29-1766	NEB_ENST0(	ENST00000427231	p.Q5695H	c.17085G>T	Unverified	ATC	CAG	GCT	3	G	T
TCGA-61-1907	NEDD1	ENST00000266742	p.V204L	c.610G>T	Unverified	AGT	GTA	CAC	1	G	T
TCGA-13-1497	NEK10	SU_NEK10	p.D875Y	c.2623G>T	Verified	aaa	gac	gaa	1	G	T
TCGA-24-2024	NEK3	NM_002498	p.D413Y	c.1237G>T	Verified	cct	gac	act	1	G	T
TCGA-04-1649	NETO1	ENST00000327305	p.V236V	c.708G>T	Unverified	GCT	GTG	TAT	3	G	T
TCGA-04-1347	NEUROD4	ENST00000242994	p.G320V	c.959G>T	Verified	CAT	GGT	ATT	2	G	T
TCGA-61-2102	NEXN	ENST00000334785	p.R42M	c.125G>T	Verified	GCC	AGG	GAA	2	G	T
TCGA-13-0920	NFE2L3	ENST00000056233	p.A299S	c.895G>T	Verified	TCA	GCA	CAT	1	G	T
TCGA-29-1777	NFX1	ENST00000379540	p.V211L	c.631G>T	Unverified	CCT	GTG	GGG	1	G	T
TCGA-04-1649	NFYC	ENST00000447388	p.G176W	c.526G>T	Unverified	cct	ggg	cag	1	G	T
TCGA-04-1343	NGEF	ENST00000264051	p.A286S	c.856G>T	Verified	GAG	GCG	TCC	1	G	T
TCGA-23-1032	NGFR	ENST00000172229	p.A355S	c.1063G>T	Verified	TCT	GCG	GGG	1	G	T
TCGA-24-1469	NINL	ENST00000278886	p.V253V	c.759G>T	Verified	AAA	GTG	AGT	3	G	T
TCGA-09-2051	NKX6-1	ENST00000295886	p.D331Y	c.991G>T	Unverified	CTG	GAT	CCC	1	G	T
TCGA-24-2267	NLE1	ENST00000442241	p.W422C	c.1266G>T	Verified	GCG	TGG	TCA	3	G	T
TCGA-24-1435	NLRC4	ENST00000360906	p.V499L	c.1495G>T	Verified	tct	gtg	gaa	1	G	T
TCGA-25-1326	NLRC5	ENST00000262510	p.V1659F	c.4975G>T	Unverified	CTC	GTT	CTT	1	G	T
TCGA-13-1481	NLRC5	ENST00000262510	p.S1409I	c.4226G>T	Verified	GGC	AGT	GTC	2	G	T

TCGA-04-1361	NLRP13	ENST00000342929	p.M1027I	c.3081G>T	Verified	AAG	ATG	CTA	3	G	T
TCGA-04-1361	NLRP13	ENST00000342929	p.M1027I	c.3081G>T	Verified	AAG	ATG	CTA	3	G	T
TCGA-24-1422	NLRP5	ENST00000390649	p.G1157V	c.3470G>T	Unverified	ATT	GGG	CTG	2	G	T
TCGA-23-1118	NLRP8	ENST00000291971	p.?	c.2381+1G>T	Verified	CTC	AGG	TTG	2	G	T
TCGA-13-0920	NMS	ENST00000376865	p.G113C	c.337G>T	Verified	CGA	GGC	TCG	1	G	T
TCGA-23-1021	NOL4	ENST00000261592	p.E44D	c.132G>T	Verified	TCC	GAG	TCG	3	G	T
TCGA-61-1904	NOL9	ENST00000377705	p.G593G	c.1779G>T	Unverified	AAT	GGG	CCC	3	G	T
TCGA-61-1900	NONO	ENST00000276079	p.G448C	c.1342G>T	Unverified	att	ggt	gga	1	G	T
TCGA-61-1725	NOP2	ENST00000399466	p.A367S	c.1099G>T	Unverified	GGA	GCC	TCC	1	G	T
TCGA-23-1120	NOTCH2	ENST00000256646	p.A1340S	c.4018G>T	Verified	ggg	gca	agg	1	G	T
TCGA-24-1474	NOTCH3	ENST00000263388	p.L1768L	c.5304G>T	Unverified	GCA	CTG	ACA	3	G	T
TCGA-13-1501	NOTUM	ENST00000330917	p.M395I	c.1185G>T	Unverified	GAG	ATG	AAC	3	G	T
TCGA-13-1501	NOTUM	ENST00000330917	p.M395I	c.1185G>T	Unverified	GAG	ATG	AAC	3	G	T
TCGA-61-1740	NPAS4	ENST00000311034	p.G432V	c.1295G>T	Unverified	CCA	GGA	GGC	2	G	T
TCGA-13-1497	NPC1	ENST00000269228	p.A764A	c.2292G>T	Verified	TTT	GCG	GGA	3	G	T
TCGA-13-1498	NPHS1	ENST00000378910	p.G779*	c.2335G>T	Verified	CTG	GGA	GAA	1	G	T
TCGA-24-2289	NPHS1	ENST00000378910	p.G54W	c.160G>T	Unverified	TGT	GGG	GTC	1	G	T
TCGA-10-0930	NPPB	ENST00000376468	p.E60*	c.178G>T	Verified	GTG	GAG	CAG	1	G	T
TCGA-10-0930	NPPB	ENST00000376468	p.E60*	c.178G>T	Verified	GTG	GAG	CAG	1	G	T
TCGA-13-1481	NPR2	ENST00000342694	p.V960F	c.2878G>T	Verified	GGG	GTC	CAT	1	G	T
TCGA-29-1784	NPSR1	ENST00000360581	p.E34*	c.100G>T	Unverified	ACT	GAA	GTG	1	G	T
TCGA-29-1784	NPSR1_ENST	ENST00000359791	p.E34*	c.100G>T	Unverified	ACT	GAA	GTG	1	G	T
TCGA-29-1691	NR1I3	ENST00000367983	p.R306R	c.918G>T	Unverified	gat	cgg	ttt	3	G	T
TCGA-13-0760	NR4A2	ENST00000339562	p.L410L	c.1230G>T	Verified	ctc	ctg	act	3	G	T
TCGA-13-1481	NR6A1	ENST00000487099	p.E282*	c.844G>T	Verified	gca	gaa	cta	1	G	T
TCGA-24-1844	NRBF2	ENST00000277746	p.G283V	c.848G>T	Unverified	AAA	GGA	TTT	2	G	T
TCGA-24-1846	NRP1	ENST00000265371	p.R143I	c.428G>T	Unverified	AAG	AGA	GGT	2	G	T
TCGA-23-1029	NRSN2	ENST00000382285	p.G140C	c.418G>T	Unverified	ATA	GGC	TGG	1	G	T
TCGA-25-2391	NRXN2	ENST00000377551	p.G702G	c.2106G>T	Unverified	aat	ggg	ggc	3	G	T
TCGA-10-0930	NSUN6	ENST00000377304	p.E25*	c.73G>T	Verified	AAG	GAG	ATT	1	G	T
TCGA-10-0930	NSUN6	ENST00000377304	p.E25*	c.73G>T	Verified	AAG	GAG	ATT	1	G	T
TCGA-09-2050	NUMA1	ENST00000393695	p.R985R	c.2955G>T	Verified	ctg	cgg	gcc	3	G	T
TCGA-61-2009	NUP210	ENST00000254508	p.A394S	c.1180G>T	Verified	CCT	GCT	GAG	1	G	T
TCGA-04-1361	NUP93	ENST00000308159	p.V421L	c.1261G>T	Verified	CAA	GTG	TGT	1	G	T

TCGA-04-1361	NUP93	ENST00000308159	p.V421L	c.1261G>T	Verified	CAA	GTG	TGT	1	G	T
TCGA-24-1616	NXPH2	ENST00000272641	p.G140*	c.418G>T	Verified	AAT	GGA	ACC	1	G	T
TCGA-13-0923	OBSCN	SU_OBSCN.1	p.G850W	c.2548G>T	Verified	gtg	ggg	acg	1	G	T
TCGA-13-0923	OBSCN_ENS	ENST00000359599	p.G850W	c.2548G>T	Verified	GTG	GGG	ACG	1	G	T
TCGA-04-1336	OC90	XM_070277.6	p.E255*	c.763G>T	Verified	cct	gag	gag	1	G	T
TCGA-04-1336	OC90_ENST(	ENST00000262283	p.E493*	c.1477G>T	Verified	CCT	GAG	GAG	1	G	T
TCGA-13-0791	OCA2	ENST00000354638	p.V833L	c.2497G>T	Verified	CAT	GTG	GTG	1	G	T
TCGA-13-0884	ODZ1	ENST00000371130	p.E2290D	c.6870G>T	Verified	TCG	GAG	ATT	3	G	T
TCGA-25-2392	OR10A7	ENST00000326258	p.R234L	c.701G>T	Verified	GGC	CGC	CAG	2	G	T
TCGA-30-1718	OR10G8	ENST00000431524	p.V271L	c.811G>T	Unverified	GTT	GTG	GCC	1	G	T
TCGA-24-2290	OR10G8	ENST00000431524	p.V294V	c.882G>T	Verified	GAG	GTG	AAG	3	G	T
TCGA-24-2290	OR10G8	ENST00000431524	p.V294V	c.882G>T	Verified	GAG	GTG	AAG	3	G	T
TCGA-23-1114	OR10H1	ENST00000334920	p.L33L	c.99G>T	Unverified	CTG	CTG	ATG	3	G	T
TCGA-23-1123	OR11H12	ENST00000550708	p.K95N	c.285G>T	Verified	CCC	AAG	ATG	3	G	T
TCGA-23-1123	OR11H12	ENST00000550708	p.K95N	c.285G>T	Verified	CCC	AAG	ATG	3	G	T
TCGA-13-1481	OR13C5	ENST00000374779	p.M144I	c.432G>T	Verified	CCC	ATG	GCA	3	G	T
TCGA-24-2024	OR1N2	ENST00000373688	p.W240L	c.719G>T	Verified	TTC	TGG	GCT	2	G	T
TCGA-13-1509	OR2F1	ENST00000392899	p.W149C	c.447G>T	Verified	TCC	TGG	GTC	3	G	T
TCGA-23-1116	OR2M5	ENST00000366476	p.L30L	c.90G>T	Verified	TTT	CTG	GTC	3	G	T
TCGA-13-1498	OR4A13P	ENST00000314689	p.G73V	c.218G>T	Verified	TGT	GGC	CCC	2	G	T
TCGA-30-1855	OR4C11	ENST00000302231	p.V144F	c.430G>T	Unverified	ATT	GTT	CTT	1	G	T
TCGA-04-1652	OR4C12	ENST00000335238	p.D68Y	c.202G>T	Unverified	ATA	GAC	ACA	1	G	T
TCGA-04-1652	OR4C12	ENST00000335238	p.D68Y	c.202G>T	Unverified	ATA	GAC	ACA	1	G	T
TCGA-61-2012	OR4C13	ENST00000555099	p.A117S	c.349G>T	Verified	ATG	GCC	TAT	1	G	T
TCGA-61-1910	OR4E2	ENST00000408935	p.A236S	c.706G>T	Unverified	AAA	GCC	CTG	1	G	T
TCGA-13-0903	OR4L1	ENST00000315683	p.R302L	c.905G>T	Verified	TTA	CGG	TTC	2	G	T
TCGA-61-1895	OR4S2	ENST00000312422	p.L45L	c.135G>T	Unverified	ATG	CTG	ACA	3	G	T
TCGA-23-1022	OR51E2	ENST00000396950	p.R301L	c.902G>T	Verified	ACA	CGG	GTG	2	G	T
TCGA-24-2280	OR52N5	ENST00000317093	p.E120*	c.358G>T	Unverified	GTG	GAG	TCT	1	G	T
TCGA-24-2267	OR5B2	ENST00000302581	p.K78N	c.234G>T	Verified	CCC	AAG	GTC	3	G	T
TCGA-29-1781	OR5H1	ENST00000354565	p.A237S	c.709G>T	Unverified	AAA	GCC	TTT	1	G	T
TCGA-25-2042	OR5R1	ENST00000312253	p.Q232H	c.696G>T	Verified	ACT	CAG	GGG	3	G	T
TCGA-29-1763	OR5V1	ENST00000377154	p.C241F	c.722G>T	Unverified	ACA	TGT	GCC	2	G	T
TCGA-30-1718	OR7G3	ENST00000305444	p.V276V	c.828G>T	Unverified	TCA	GTG	ATG	3	G	T

TCGA-23-1022	OR8U1	ENST00000302270	p.M108I	c.324G>T	Verified	TTC	ATG	ATA	3	G	T
TCGA-09-1665	OXCT1	ENST00000196371	p.G62V	c.185G>T	Verified	GTT	GGT	GGT	2	G	T
TCGA-36-1577	OXSR1_ENST	ENST00000311806	p.V201F	c.601G>T	Unverified	CAG	GTC	CGT	1	G	T
TCGA-13-1510	P2RY12	ENST00000302632	p.R231S	c.693G>T	Unverified	CCC	AGG	AAA	3	G	T
TCGA-13-1497	P2RY6	ENST00000393590	p.A280S	c.838G>T	Verified	TTT	GCA	GCG	1	G	T
TCGA-20-1686	PADI1	ENST00000375471	p.E244*	c.730G>T	Unverified	GGG	GAG	CAG	1	G	T
TCGA-13-0924	PAGE2	ENST00000374968	p.E74*	c.220G>T	Verified	CAG	GAA	CTG	1	G	T
TCGA-13-1497	PAGE2	ENST00000374968	p.G65W	c.193G>T	Verified	CAA	GGG	CCT	1	G	T
TCGA-25-2401	PAIP1	ENST00000306846	p.D260Y	c.778G>T	Verified	GGG	GAT	GAA	1	G	T
TCGA-24-1469	PAK3	ENST00000446737	p.R49L	c.146G>T	Verified	ctt	cgc	tct	2	G	T
TCGA-13-0913	PAK3	ENST00000446737	p.W339C	c.1017G>T	Verified	cta	tgg	gta	3	G	T
TCGA-13-1481	PAPD4	ENST00000296783	p.A442S	c.1324G>T	Verified	ACA	GCC	AGA	1	G	T
TCGA-13-0903	PAPPA2	ENST00000367662	p.V1020L	c.3058G>T	Verified	GGG	GTG	AAA	1	G	T
TCGA-13-0793	PAPPA2	ENST00000367662	p.G1148V	c.3443G>T	Verified	GAA	GGT	TTC	2	G	T
TCGA-13-0795	PARP14	ENST00000310276	p.L1209L	c.3627G>T	Verified	TTT	CTG	CCT	3	G	T
TCGA-25-1313	PAX6	ENST00000419022	p.G190V	c.569G>T	Verified	gat	ggc	tgc	2	G	T
TCGA-23-1029	PCDHB2	ENST00000194155	p.V777F	c.2329G>T	Unverified	TTC	GTT	GCT	1	G	T
TCGA-25-1313	PCDHB2	ENST00000194155	p.P11P	c.33G>T	Verified	GTT	CCG	AAA	3	G	T
TCGA-61-1725	PCDHB7	ENST00000231137	p.V232F	c.694G>T	Unverified	CTG	GTT	CTA	1	G	T
TCGA-29-1696	PCDHGA10	ENST00000398610	p.A641A	c.1923G>T	Unverified	GAC	GCG	CTC	3	G	T
TCGA-29-1696	PCDHGA10	ENST00000398610	p.A641A	c.1923G>T	Unverified	GAC	GCG	CTC	3	G	T
TCGA-61-1895	PCDHGA10	ENST00000398610	p.V596V	c.1788G>T	Unverified	GTG	GTG	GCG	3	G	T
TCGA-61-1910	PCDHGB6	ENST00000520790	p.A472S	c.1414G>T	Unverified	ATT	GCG	CAA	1	G	T
TCGA-13-0893	PCSK5	ENST00000376752	p.Q816H	c.2448G>T	Verified	GTG	CAG	AGC	3	G	T
TCGA-25-1326	PCSK9	ENST00000302118	p.R167L	c.500G>T	Verified	TAC	CGG	GCG	2	G	T
TCGA-29-1777	PDE1C	ENST00000396184	p.V210F	c.628G>T	Unverified	CTT	GTC	TCA	1	G	T
TCGA-29-1777	PDE1C_ENST	ENST00000396191	p.V210F	c.628G>T	Unverified	CTT	GTC	TCA	1	G	T
TCGA-29-1777	PDE1C_ENST	ENST00000396193	p.V270F	c.808G>T	Unverified	CTT	GTC	TCA	1	G	T
TCGA-23-2078	PDE2A	ENST00000334456	p.?	c.1183-1G>T	Verified	CAG	GCT	CTT	1	G	T
TCGA-13-0905	PDE4B	ENST00000329654	p.S417S	c.1251G>T	Verified	CAG	TCG	ACC	3	G	T
TCGA-13-0905	PDE4B_ENST	ENST00000423207	p.S402S	c.1206G>T	Verified	CAG	TCG	ACC	3	G	T
TCGA-24-2024	PDE4DIP	ENST00000369354	p.S1389S	c.4167G>T	Verified	TAT	TCG	TCT	3	G	T
TCGA-23-1029	PDE6C	ENST00000371447	p.L179L	c.537G>T	Unverified	AAC	CTG	CTG	3	G	T
TCGA-13-1489	PDIA3	ENST00000300289	p.L254F	c.762G>T	Verified	GAT	TTG	ATA	3	G	T

TCGA-25-2392	PDLIM1	ENST00000329399	p.G171V	c.512G>T	Unverified	AGC	GGG	GTG	2	G	T
TCGA-61-1906	PDP1	ENST00000297598	p.V430F	c.1288G>T	Unverified	gtg	gtt	agg	1	G	T
TCGA-61-1906	PDP1	ENST00000297598	p.V430F	c.1288G>T	Unverified	gtg	gtt	agg	1	G	T
TCGA-61-1906	PDP1_ENST(	ENST00000396200	p.V455F	c.1363G>T	Unverified	GTG	GTT	AGG	1	G	T
TCGA-61-1906	PDP1_ENST(	ENST00000396200	p.V455F	c.1363G>T	Unverified	GTG	GTT	AGG	1	G	T
TCGA-24-1464	PDS5B	ENST00000315596	p.L999F	c.2997G>T	Verified	CTT	TTG	GCA	3	G	T
TCGA-24-1847	PDYN	ENST00000217305	p.K235N	c.705G>T	Unverified	TTC	AAG	GTG	3	G	T
TCGA-09-1674	PDZD7	ENST00000370215	p.R476L	c.1427G>T	Unverified	GGG	CGG	CAG	2	G	T
TCGA-09-1674	PDZD7_ENS	ENST00000393462	p.R476L	c.1427G>T	Unverified	GGG	CGG	CAG	2	G	T
TCGA-13-1488	PDZRN4	ENST00000539469	p.K378N	c.1134G>T	Verified	TGC	AAG	ATT	3	G	T
TCGA-61-1998	PEG3	ENST00000326441	p.G1266C	c.3796G>T	Verified	CCA	GGC	TTA	1	G	T
TCGA-13-1408	PELI1	ENST00000358912	p.G348*	c.1042G>T	Verified	CTT	GGA	TGT	1	G	T
TCGA-61-2012	PER1	ENST00000317276	p.A712A	c.2136G>T	Verified	AAG	GCG	GAG	3	G	T
TCGA-29-1702	PFAS	ENST00000314666	p.A984S	c.2950G>T	Unverified	GAG	GCC	GGG	1	G	T
TCGA-13-0923	PFKFB2	ENST00000367080	p.A109S	c.325G>T	Verified	GTG	GCG	CTG	1	G	T
TCGA-09-2056	PGK2	ENST00000304801	p.A102S	c.304G>T	Verified	GGC	GCA	GAA	1	G	T
TCGA-04-1542	PGLYRP2	ENST00000340880	p.V111L	c.331G>T	Verified	GTG	GTG	CTG	1	G	T
TCGA-13-1507	PGM5	ENST00000396396	p.V357L	c.1069G>T	Verified	CCT	GTA	TAT	1	G	T
TCGA-13-0807	PHEX	ENST00000379374	p.?	c.933+1G>T	Verified	CCC	CAG	TTC	3	G	T
TCGA-24-1464	PHF20L1	ENST00000337920	p.G215*	c.643G>T	Verified	TTT	GGA	CTT	1	G	T
TCGA-29-1691	PIAS2	ENST00000398654	p.D204Y	c.610G>T	Unverified	AGA	GAT	TAT	1	G	T
TCGA-29-1691	PIAS2_ENST	ENST00000324794	p.D204Y	c.610G>T	Unverified	AGA	GAT	TAT	1	G	T
TCGA-13-0714	PIGM	ENST00000368090	p.T237T	c.711G>T	Verified	ctc	acg	ttt	3	G	T
TCGA-61-2012	PIGR	ENST00000356495	p.K577N	c.1731G>T	Verified	GCG	AAG	GCA	3	G	T
TCGA-13-1488	PIGV	ENST00000374145	p.S244S	c.732G>T	Verified	CTG	TCG	GTG	3	G	T
TCGA-61-1738	PIK3CA	NM_006218.1	p.R115L	c.344G>T	Unverified	aat	cga	gaa	2	G	T
TCGA-61-1738	PIK3CA	NM_006218.1	p.R115L	c.344G>T	Unverified	aat	cga	gaa	2	G	T
TCGA-61-1738	PIK3CA_ENS	ENST00000263967	p.R115L	c.344G>T	Unverified	AAT	CGA	GAA	2	G	T
TCGA-61-1738	PIK3CA_ENS	ENST00000263967	p.R115L	c.344G>T	Unverified	AAT	CGA	GAA	2	G	T
TCGA-13-0792	PIK3CG	ENST00000440650	p.A445S	c.1333G>T	Unverified	AAG	GCC	TCT	1	G	T
TCGA-13-0795	PIP4K2B	ENST00000269554	p.V157L	c.469G>T	Verified	gac	gtg	gcg	1	G	T
TCGA-61-1895	PKD2L1	ENST00000318222	p.V399L	c.1195G>T	Unverified	ATT	GTG	GCT	1	G	T
TCGA-24-1469	PKHD1	ENST00000371117	p.A2521S	c.7561G>T	Verified	CAT	GCA	GCA	1	G	T
TCGA-13-0920	PKHD1	ENST00000371117	p.R1390R	c.4170G>T	Verified	CCT	CGG	ATA	3	G	T

TCGA-09-2049	PKLR	ENST00000342741	p.R289L	c.866G>T	Verified	GTG	CGG	AAA	2	G	T
TCGA-13-0906	PKN1	ENST00000242783	p.E764D	c.2292G>T	Unverified	AAG	GAG	GGG	3	G	T
TCGA-24-2289	PKP1	ENST00000367324	p.R317M	c.950G>T	Unverified	TTC	AGG	AGC	2	G	T
TCGA-24-0975	PLA2G2F	ENST00000375102	p.G30V	c.89G>T	Verified	TTC	GGG	GCC	2	G	T
TCGA-29-2427	PLAC8	ENST00000311507	p.?	c.119-1G>T	Verified	GTC	TGT	CTC	2	G	T
TCGA-61-2009	PLAU	ENST00000372764	p.C91F	c.272G>T	Verified	CCC	TGC	CTG	2	G	T
TCGA-13-0886	PLB1	ENST00000327757	p.R890I	c.2669G>T	Unverified	CAT	AGA	GAG	2	G	T
TCGA-23-1123	PLCXD3	ENST00000377801	p.E316*	c.946G>T	Verified	gat	gaa	gga	1	G	T
TCGA-23-1123	PLCXD3	ENST00000377801	p.E316*	c.946G>T	Verified	gat	gaa	gga	1	G	T
TCGA-23-1124	PLCZ1	ENST00000266505	p.G592C	c.1774G>T	Verified	atg	ggt	gag	1	G	T
TCGA-04-1336	PLD5	ENST00000366545	p.W191L	c.572G>T	Verified	ACC	TGG	TCC	2	G	T
TCGA-10-0930	PLEC	ENST00000322810	p.R3439M	c.10316G>T	Verified	GAG	AGG	CTG	2	G	T
TCGA-10-0930	PLEC	ENST00000322810	p.R3439M	c.10316G>T	Verified	GAG	AGG	CTG	2	G	T
TCGA-24-1846	PLEKHA5	ENST00000299275	p.Q747H	c.2241G>T	Unverified	GTT	CAG	ACG	3	G	T
TCGA-24-1846	PLEKHA5_EN	ENST00000429027	p.Q850H	c.2550G>T	Unverified	GTT	CAG	ACG	3	G	T
TCGA-13-1507	PLEKHB1	ENST00000354190	p.L51L	c.153G>T	Verified	acc	ctg	gga	3	G	T
TCGA-04-1347	PLEKHF2	ENST00000315367	p.K139N	c.417G>T	Verified	GGG	AAG	ACA	3	G	T
TCGA-13-1499	PLEKHG4B_I	ENST00000283426	p.V412F	c.1234G>T	Verified	ACC	GTC	CTG	1	G	T
TCGA-13-1499	PLXDC1	ENST00000315392	p.V55L	c.163G>T	Verified	CAT	GTG	TCA	1	G	T
TCGA-13-0916	PLXNA4	ENST00000378539	p.D89Y	c.265G>T	Verified	CCG	GAC	GAG	1	G	T
TCGA-04-1362	PLXNA4_EN:	ENST00000321063	p.E1568*	c.4702G>T	Verified	GAT	GAA	GAC	1	G	T
TCGA-13-0887	PLXNB2	XM_371474.2	p.D970Y	c.2908G>T	Verified	gag	gac	gtg	1	G	T
TCGA-29-1770	PMS2	ENST00000265849	p.G750C	c.2248G>T	Unverified	AAT	GGC	TTT	1	G	T
TCGA-61-1740	POLA1_ENST	ENST00000379059	p.V1059F	c.3175G>T	Unverified	CTG	GTT	GTT	1	G	T
TCGA-23-1114	POLA2	ENST00000265465	p.E106*	c.316G>T	Unverified	GAA	GAG	GAA	1	G	T
TCGA-23-1114	POLA2_ENST	ENST00000265465	p.E106*	c.316G>T	Unverified	GAA	GAG	GAA	1	G	T
TCGA-24-1469	POLR3B	ENST00000228347	p.L29L	c.87G>T	Verified	AGG	CTG	CTT	3	G	T
TCGA-61-1998	POMT2	ENST00000261534	p.V342V	c.1026G>T	Verified	TCT	GTG	ATC	3	G	T
TCGA-23-1114	POP1	ENST00000349693	p.W631C	c.1893G>T	Unverified	TTC	TGG	ATT	3	G	T
TCGA-09-2044	POTED	ENST00000299443	p.G239*	c.715G>T	Verified	TAT	GGA	AAT	1	G	T
TCGA-13-0883	POTEF	ENST00000409914	p.K100N	c.300G>T	Verified	GGC	AAG	TGG	3	G	T
TCGA-24-1422	POU6F1	ENST00000333640	p.A248S	c.742G>T	Verified	GAG	GCT	CTC	1	G	T
TCGA-23-1114	PPAPDC2	ENST00000381883	p.D93Y	c.277G>T	Unverified	ATG	GAC	TTG	1	G	T
TCGA-23-1022	PPBP	ENST00000296028	p.G48V	c.143G>T	Verified	AAA	GGC	AAA	2	G	T

TCGA-61-1895	PPL	ENST00000345988	p.E1044*	c.3130G>T	Unverified	GAA	GAG	AAG	1	G	T
TCGA-24-2280	PPM1E	ENST00000308249	p.G523V	c.1568G>T	Unverified	cat	gga	gag	2	G	T
TCGA-61-1900	PPP1CB	ENST00000358506	p.G173V	c.518G>T	Unverified	cat	gga	gga	2	G	T
TCGA-13-1498	PPP1R11	ENST00000376772	p.A69S	c.205G>T	Verified	cgg	gcc	ttt	1	G	T
TCGA-04-1356	PPP1R13L	ENST00000360957	p.P59P	c.177G>T	Unverified	GGC	CCG	CAG	3	G	T
TCGA-29-1783	PPP1R15A	ENST00000200453	p.D406Y	c.1216G>T	Unverified	AGT	GAT	ACA	1	G	T
TCGA-13-1488	PPP1R3B	ENST00000310455	p.M243I	c.729G>T	Verified	GGA	ATG	ACC	3	G	T
TCGA-24-1616	PPP2R1A	ENST00000322088	p.W257C	c.771G>T	Verified	tcc	tgg	cgc	3	G	T
TCGA-04-1530	PPP3R2	ENST00000374806	p.G89C	c.265G>T	Verified	aag	ggc	gac	1	G	T
TCGA-23-2078	PPP6R1_ENS	ENST00000412770	p.A632S	c.1894G>T	Unverified	GAG	GCC	CAG	1	G	T
TCGA-36-1577	PRC1	ENST00000394249	p.G543V	c.1628G>T	Unverified	CAT	GGA	GCC	2	G	T
TCGA-61-2012	PRDM7	ENST00000325921	p.A58S	c.172G>T	Unverified	GAG	GCA	TCT	1	G	T
TCGA-13-0795	PREPL	ENST00000260648	p.?	c.2094+1G>T	Verified	AAA	AAG	ATT	3	G	T
TCGA-24-0979	PREX1	ENST00000371941	p.C234F	c.701G>T	Verified	GTT	TGC	TCC	2	G	T
TCGA-25-2393	PRKCQ	ENST00000263125	p.G499*	c.1495G>T	Verified	aaa	gga	ata	1	G	T
TCGA-29-1701	PRKG1	ENST00000373980	p.D508Y	c.1522G>T	Unverified	cta	gat	cac	1	G	T
TCGA-29-1701	PRKG1_ENST	ENST00000401604	p.D493Y	c.1477G>T	Unverified	CTA	GAT	CAC	1	G	T
TCGA-24-1845	PRKG2	ENST00000395578	p.G602G	c.1806G>T	Unverified	ATA	GGG	TCT	3	G	T
TCGA-24-1845	PRKG2_ENST	ENST00000395578	p.G602G	c.1806G>T	Unverified	ATA	GGG	TCT	3	G	T
TCGA-09-2050	PRPF19	ENST00000227524	p.E197*	c.589G>T	Verified	GAG	GAG	CTG	1	G	T
TCGA-04-1651	PRR14L_ENS	ENST00000327423	p.V186L	c.556G>T	Unverified	AAT	GTA	CAG	1	G	T
TCGA-04-1651	PRR14L_ENS	ENST00000327423	p.V186L	c.556G>T	Unverified	AAT	GTA	CAG	1	G	T
TCGA-04-1651	PRR14L_ENS	ENST00000397493	p.V186L	c.556G>T	Unverified	AAT	GTA	CAG	1	G	T
TCGA-04-1651	PRR14L_ENS	ENST00000397493	p.V186L	c.556G>T	Unverified	AAT	GTA	CAG	1	G	T
TCGA-61-1738	PRSS35	ENST00000369700	p.G218C	c.652G>T	Unverified	GAG	GGT	ACC	1	G	T
TCGA-61-1738	PRSS35	ENST00000369700	p.G218C	c.652G>T	Unverified	GAG	GGT	ACC	1	G	T
TCGA-29-1691	PRSS55	ENST00000328655	p.W186C	c.558G>T	Unverified	ACA	TGG	CGC	3	G	T
TCGA-09-1674	PRX	ENST00000324001	p.E628D	c.1884G>T	Unverified	CCA	GAG	ATG	3	G	T
TCGA-23-1022	PSMD3	ENST00000264639	p.G383V	c.1148G>T	Verified	TTT	GGG	GAG	2	G	T
TCGA-13-1481	PSME1	ENST00000206451	p.V150L	c.448G>T	Verified	GGA	GTG	GCT	1	G	T
TCGA-25-1326	PTCH1	ENST00000331920	p.A1101S	c.3301G>T	Verified	GTT	GCT	TTG	1	G	T
TCGA-25-1326	PTCH1_ENST	ENST00000331920	p.A1101S	c.3301G>T	Verified	GTT	GCT	TTG	1	G	T
TCGA-10-0930	PTEN	ENST00000371953	p.V175L	c.523G>T	Verified	tat	gtg	tat	1	G	T
TCGA-10-0930	PTEN	ENST00000371953	p.V175L	c.523G>T	Verified	tat	gtg	tat	1	G	T

TCGA-13-1507	PTGDR	ENST00000306051	p.W131L	c.392G>T	Verified	TGC	TGG	CTC	2	G	T
TCGA-04-1347	PTGFR	ENST00000370756	p.L296F	c.888G>T	Verified	ACA	TTG	GAA	3	G	T
TCGA-04-1347	PTGFR_ENST	ENST00000370758	p.G273*	c.817G>T	Verified	ATT	GGA	ATA	1	G	T
TCGA-23-1022	PTGIS	ENST00000244043	p.A307S	c.919G>T	Verified	CTG	GCT	GCT	1	G	T
TCGA-23-1110	PTK2B	ENST00000397501	p.R770R	c.2310G>T	Verified	CAC	CGG	CAC	3	G	T
TCGA-04-1638	PTOV1	ENST00000391842	p.C359F	c.1076G>T	Unverified	tcg	tgt	gag	2	G	T
TCGA-13-1408	PTPRG	ENST00000474889	p.V237F	c.709G>T	Verified	TTC	GTC	CTC	1	G	T
TCGA-13-1505	PTPRH	ENST00000376350	p.A403S	c.1207G>T	Verified	ATC	GCC	CTA	1	G	T
TCGA-09-2051	PTPRN	ENST00000295718	p.L338L	c.1014G>T	Unverified	agg	ctg	gcc	3	G	T
TCGA-29-1775	PTPRN	ENST00000295718	p.L493L	c.1479G>T	Unverified	atc	ctg	gct	3	G	T
TCGA-61-1740	PTPRT	NM_133170.2	p.E287*	c.859G>T	Unverified	aaa	gag	cct	1	G	T
TCGA-04-1652	PTPRZ1	ENST00000393386	p.D866Y	c.2596G>T	Unverified	GGT	GAT	TTG	1	G	T
TCGA-04-1652	PTPRZ1	ENST00000393386	p.D866Y	c.2596G>T	Unverified	GGT	GAT	TTG	1	G	T
TCGA-13-1498	PTPRZ1	ENST00000393386	p.G667*	c.1999G>T	Verified	GTT	GGA	TCA	1	G	T
TCGA-04-1652	PTPRZ1_ENS	ENST00000393386	p.D866Y	c.2596G>T	Unverified	GGT	GAT	TTG	1	G	T
TCGA-04-1652	PTPRZ1_ENS	ENST00000393386	p.D866Y	c.2596G>T	Unverified	GGT	GAT	TTG	1	G	T
TCGA-23-1031	PUS10	ENST00000316752	p.E408*	c.1222G>T	Verified	GAA	GAA	GAA	1	G	T
TCGA-24-1846	PVALB	ENST00000216200	p.D91Y	c.271G>T	Unverified	GGA	GAC	AAA	1	G	T
TCGA-61-1910	PXN	ENST00000424649	p.L7L	c.21G>T	Unverified	GCC	CTG	CTG	3	G	T
TCGA-61-1910	PXN_ENST0(	ENST00000228307	p.L7L	c.21G>T	Unverified	GCC	CTG	CTG	3	G	T
TCGA-24-1850	Q5T7C0_HU	ENST00000255320	p.C106F	c.317G>T	Unverified	TTC	TGC	TCT	2	G	T
TCGA-13-0887	Q6YL47_HU	ENST00000322367	p.A1234S	c.3700G>T	Verified	AGG	GCC	GGT	1	G	T
TCGA-13-0762	Q6YL47_HU	ENST00000322367	p.R864L	c.2591G>T	Verified	TTC	CGC	CTG	2	G	T
TCGA-13-0762	Q6YL47_HU	ENST00000322367	p.R864L	c.2591G>T	Verified	TTC	CGC	CTG	2	G	T
TCGA-24-1464	Q86U89_HU	ENST00000220847	p.G215*	c.643G>T	Verified	TTT	GGA	CTT	1	G	T
TCGA-61-1900	Q8NH77_HU	ENST00000316517	p.W29L	c.86G>T	Unverified	GAG	TGG	CAG	2	G	T
TCGA-24-1463	QDPR	ENST00000281243	p.K79N	c.237G>T	Verified	GAG	AAG	GTG	3	G	T
TCGA-29-1699	QRFPR	ENST00000394427	p.K303N	c.909G>T	Unverified	GAA	AAG	GAA	3	G	T
TCGA-24-1103	QRICH2	ENST00000262765	p.G769V	c.2306G>T	Verified	CCT	GGT	GCC	2	G	T
TCGA-13-0885	RAB15	ENST00000267512	p.R169S	c.507G>T	Verified	atg	agg	agc	3	G	T
TCGA-30-1718	RAB20	ENST00000267328	p.R209I	c.626G>T	Unverified	CAG	AGA	GCT	2	G	T
TCGA-30-1718	RAB20_ENST	ENST00000267328	p.R209I	c.626G>T	Unverified	CAG	AGA	GCT	2	G	T
TCGA-13-1488	RAB37	ENST00000402449	p.Q198H	c.594G>T	Verified	TTC	CAG	ATC	3	G	T
TCGA-61-1895	RAB3GAP1	ENST00000264158	p.?	c.2289+1G>T	Unverified	GAA	AAG	GTG	3	G	T

TCGA-13-0795	RAB40AL	ENST00000218249	p.T70T	c.210G>T	Verified	GAT	ACG	TCG	3	G	T
TCGA-24-1850	RAE1	ENST00000395841	p.R159L	c.476G>T	Unverified	act	cga	tcg	2	G	T
TCGA-29-1762	RALB	ENST00000272519	p.V125F	c.373G>T	Unverified	ctc	gtc	gtg	1	G	T
TCGA-61-1895	RANBP2	ENST00000283195	p.S1592I	c.4775G>T	Unverified	ACA	AGC	AAG	2	G	T
TCGA-61-1895	RANBP2_EN	ENST00000283195	p.S1592I	c.4775G>T	Unverified	ACA	AGC	AAG	2	G	T
TCGA-61-2095	RANBP3	ENST00000340578	p.G141V	c.422G>T	Unverified	agt	ggc	ttc	2	G	T
TCGA-13-0887	RAPGEF1	ENST00000372190	p.G846W	c.2536G>T	Verified	cgg	ggg	gta	1	G	T
TCGA-29-1698	RAPGEFL1	ENST00000264644	p.G106C	c.316G>T	Unverified	cgg	ggc	tct	1	G	T
TCGA-25-2391	RASA1	ENST00000274376	p.G700C	c.2098G>T	Verified	aaa	ggt	att	1	G	T
TCGA-25-2392	RASAL1	ENST00000261729	p.G50V	c.149G>T	Verified	CTG	GGC	CCC	2	G	T
TCGA-09-2044	RASAL2	ENST00000263528	p.E120*	c.358G>T	Verified	TCC	GAG	GGT	1	G	T
TCGA-20-1686	RASSF8	ENST00000381352	p.E290D	c.870G>T	Unverified	GAA	GAG	GTT	3	G	T
TCGA-20-1686	RASSF8_ENS	ENST00000541490	p.E290D	c.870G>T	Unverified	GAA	GAG	GTT	3	G	T
TCGA-13-1498	RB1CC1	ENST00000025008	p.E1387*	c.4159G>T	Verified	GAA	GAA	GAA	1	G	T
TCGA-24-2024	RB1CC1	ENST00000025008	p.G1411*	c.4231G>T	Verified	TAT	GGA	GCT	1	G	T
TCGA-25-2393	RB1CC1	ENST00000025008	p.E1388*	c.4162G>T	Verified	GAA	GAA	GTC	1	G	T
TCGA-29-1764	RB1CC1	ENST00000025008	p.R550L	c.1649G>T	Unverified	AAT	CGT	CTG	2	G	T
TCGA-09-2051	RBBP7	ENST00000380087	p.V297L	c.889G>T	Unverified	acc	gta	gct	1	G	T
TCGA-23-1118	RBL2	ENST00000262133	p.S1090I	c.3269G>T	Verified	AAT	AGT	ATG	2	G	T
TCGA-24-1846	RBM12	ENST00000374104	p.V13L	c.37G>T	Unverified	ATT	GTG	GCG	1	G	T
TCGA-13-1510	RBM47	ENST00000319592	p.G43C	c.127G>T	Unverified	ACG	GGC	TAC	1	G	T
TCGA-13-0885	RBM6	ENST00000266022	p.A77S	c.229G>T	Verified	GGA	GCT	AGA	1	G	T
TCGA-04-1338	RBPJL	ENST00000343694	p.C208F	c.623G>T	Verified	ctg	tgc	ata	2	G	T
TCGA-61-1904	RBPJL	ENST00000343694	p.R220L	c.659G>T	Unverified	aac	cgc	ctg	2	G	T
TCGA-25-1313	REEP6	ENST00000233596	p.P142P	c.426G>T	Unverified	CGT	CCG	CTG	3	G	T
TCGA-25-1313	REEP6_ENST	ENST00000395484	p.A210S	c.628G>T	Unverified	TCC	GCT	GTT	1	G	T
TCGA-29-1769	RELN	ENST00000343529	p.G2603C	c.7807G>T	Unverified	GGA	GGC	ATT	1	G	T
TCGA-24-2262	REPS2	NM_004726.1	p.V417L	c.1249G>T	Unverified	gat	gta	ctg	1	G	T
TCGA-25-2042	RFTN1	ENST00000334133	p.G489V	c.1466G>T	Verified	GCT	GGA	GAC	2	G	T
TCGA-10-0930	RFXAP	ENST00000255476	p.E200*	c.598G>T	Verified	CTC	GAG	GAA	1	G	T
TCGA-10-0930	RFXAP	ENST00000255476	p.E200*	c.598G>T	Verified	CTC	GAG	GAA	1	G	T
TCGA-23-1022	RGL4	ENST00000290691	p.A363S	c.1087G>T	Verified	aag	gcg	ggg	1	G	T
TCGA-61-1998	RGS22	ENST00000360863	p.R1037L	c.3110G>T	Verified	CAA	CGT	TTT	2	G	T
TCGA-13-0883	RHBG	ENST00000368249	p.G306V	c.917G>T	Verified	TTT	GGG	GCT	2	G	T

TCGA-61-2095	RIMS1	ENST00000521978	p.A340A	c.1020G>T	Unverified	GCG	GCG	GAT	3	G	T
TCGA-23-1110	RIN2	NM_018993.2	p.G10C	c.28G>T	Verified	cgc	ggt	ctg	1	G	T
TCGA-13-0904	RLIM	ENST00000332687	p.R117I	c.350G>T	Verified	caa	aga	gga	2	G	T
TCGA-09-2051	RND1	ENST00000309739	p.E150D	c.450G>T	Unverified	tat	gag	cag	3	G	T
TCGA-13-1505	RNF112	ENST00000299604	p.V345V	c.1035G>T	Verified	AAG	GTG	CAG	3	G	T
TCGA-13-0714	RNF113B	ENST00000267291	p.E232D	c.696G>T	Verified	GAA	GAG	GGT	3	G	T
TCGA-24-2280	RNF133	ENST00000340112	p.R262L	c.785G>T	Unverified	gaa	cgc	tat	2	G	T
TCGA-61-1737	RNF19A	ENST00000519449	p.G86C	c.256G>T	Unverified	aat	ggc	ggg	1	G	T
TCGA-13-1481	RNF213	ENST00000336301	p.K741N	c.2223G>T	Verified	TCC	AAG	TCC	3	G	T
TCGA-04-1342	RNPS1	ENST00000397086	p.E232D	c.696G>T	Unverified	CAG	GAG	ATC	3	G	T
TCGA-20-0990	RORA	ENST00000261523	p.E14D	c.42G>T	Verified	ACT	GAG	GCA	3	G	T
TCGA-61-2094	ROS1	ENST00000368508	p.V707V	c.2121G>T	Unverified	AAT	GTG	TCA	3	G	T
TCGA-04-1367	RP1	ENST00000220676	p.T1796T	c.5388G>T	Verified	CCA	ACG	ATG	3	G	T
TCGA-24-2289	RP1	ENST00000220676	p.Q2077H	c.6231G>T	Unverified	TTC	CAG	GGC	3	G	T
TCGA-24-1417	RP1L1	ENST00000382483	p.G2369C	c.7105G>T	Verified	GAA	GGT	TAT	1	G	T
TCGA-29-1690	RP1L1	ENST00000382483	p.G1335V	c.4004G>T	Unverified	GAG	GGG	GTG	2	G	T
TCGA-23-1124	RPGRIP1L	ENST00000379925	p.G103V	c.308G>T	Verified	CTG	GGA	CGA	2	G	T
TCGA-13-1498	RPL15	ENST00000354811	p.G175*	c.523G>T	Verified	CTT	GGA	AAG	1	G	T
TCGA-13-0905	RPP38	ENST00000378203	p.V21V	c.63G>T	Verified	GTT	GTG	AAG	3	G	T
TCGA-23-1120	RPS6KA2	ENST00000265678	p.R685L	c.2054G>T	Verified	AGC	CGA	CAG	2	G	T
TCGA-09-1674	RPTN	ENST00000316073	p.G464V	c.1391G>T	Unverified	TAT	GGT	CAG	2	G	T
TCGA-13-0760	RREB1	ENST00000379933	p.G782V	c.2345G>T	Verified	GGC	GGG	GGC	2	G	T
TCGA-04-1331	RRP9	ENST00000232888	p.K44N	c.132G>T	Verified	GGC	AAG	ATG	3	G	T
TCGA-25-1318	RUSC2	ENST00000361226	p.K970N	c.2910G>T	Verified	GAG	AAG	CCT	3	G	T
TCGA-25-1318	RUSC2	ENST00000361226	p.K970N	c.2910G>T	Verified	GAG	AAG	CCT	3	G	T
TCGA-24-1422	RWDD4	ENST00000326397	p.L100F	c.300G>T	Verified	ACA	TTG	TTT	3	G	T
TCGA-13-0761	RYR1	ENST00000359596	p.G1184W	c.3550G>T	Unverified	ATT	GGG	GAC	1	G	T
TCGA-13-0761	RYR1	ENST00000359596	p.G1184W	c.3550G>T	Unverified	ATT	GGG	GAC	1	G	T
TCGA-24-1423	RYR2	ENST00000366574	p.A1798S	c.5392G>T	Verified	GAA	GCT	GTT	1	G	T
TCGA-23-1114	RYR2	ENST00000366574	p.G253V	c.758G>T	Unverified	CAT	GGT	GAA	2	G	T
TCGA-09-2044	RYR3	ENST00000389232	p.G609G	c.1827G>T	Verified	AAT	GGG	GTT	3	G	T
TCGA-61-2012	RYR3	ENST00000389232	p.M2040I	c.6120G>T	Verified	CTC	ATG	ATC	3	G	T
TCGA-20-1683	S100A7A	ENST00000329256	p.G99V	c.296G>T	Unverified	GGG	GGA	AGC	2	G	T
TCGA-24-1843	S1PR2	ENST00000317726	p.E109D	c.327G>T	Unverified	CGG	GAG	GGC	3	G	T

TCGA-23-1022	S1PR3	ENST00000375846	p.R311L	c.932G>T	Verified	TTC	CGT	CTG	2	G	T
TCGA-29-1705	SALL1	ENST00000251020	p.G895V	c.2684G>T	Unverified	GAG	GGG	GAT	2	G	T
TCGA-13-0807	SALL3	ENST00000537592	p.G1254C	c.3760G>T	Verified	CTG	GGC	AGC	1	G	T
TCGA-23-1116	SAMD10	ENST00000369886	p.V178L	c.532G>T	Unverified	CAG	GTG	CTC	1	G	T
TCGA-13-1481	SAMD9	ENST00000379958	p.A651S	c.1951G>T	Verified	ACT	GCT	CTG	1	G	T
TCGA-24-1469	SAMD9	ENST00000379958	p.R1040L	c.3119G>T	Verified	CAC	CGC	GAT	2	G	T
TCGA-25-2400	SAMD9L	ENST00000318238	p.E1358*	c.4072G>T	Verified	ATG	GAA	AGT	1	G	T
TCGA-24-2262	SBF1	ENST00000390679	p.G688W	c.2062G>T	Unverified	TAT	GGG	GAT	1	G	T
TCGA-61-1740	SBF1	ENST00000390679	p.G409V	c.1226G>T	Unverified	CGT	GGG	CTG	2	G	T
TCGA-61-1740	SBF1_ENST0	ENST00000380817	p.G409V	c.1226G>T	Unverified	CGT	GGG	CTG	2	G	T
TCGA-61-1913	SBK2	ENST00000344158	p.G85C	c.253G>T	Unverified	AAA	GGC	ACA	1	G	T
TCGA-25-2400	SBSN	ENST00000518157	p.G154G	c.462G>T	Verified	CAA	GGG	GTC	3	G	T
TCGA-13-0919	SCAF8	ENST00000367178	p.E1101D	c.3303G>T	Verified	GAT	GAG	AGA	3	G	T
TCGA-61-1740	SCIN	ENST00000297029	p.E666*	c.1996G>T	Unverified	GTT	GAG	AAA	1	G	T
TCGA-61-1740	SCIN_ENST0	ENST00000297029	p.E666*	c.1996G>T	Unverified	GTT	GAG	AAA	1	G	T
TCGA-24-2289	SCN4B	ENST00000324727	p.G171C	c.511G>T	Unverified	GTG	GGC	GGG	1	G	T
TCGA-24-2267	SCTR	ENST00000019103	p.W112C	c.336G>T	Verified	GGC	TGG	TCA	3	G	T
TCGA-24-1104	SDC1	ENST00000254351	p.R155M	c.464G>T	Verified	CAC	AGG	GAC	2	G	T
TCGA-61-2095	SDHA	ENST00000264932	p.R195R	c.585G>T	Unverified	GAT	CGG	ACT	3	G	T
TCGA-04-1367	SDHAF2	ENST00000301761	p.L16L	c.48G>T	Verified	GCT	CTG	TCA	3	G	T
TCGA-24-2280	SDK1	ENST00000404826	p.V361F	c.1081G>T	Unverified	TAC	GTC	TGC	1	G	T
TCGA-23-1117	SDK2	ENST00000316893	p.V1698F	c.5092G>T	Verified	ATG	GTC	AGC	1	G	T
TCGA-23-1117	SDK2	ENST00000316893	p.V1698F	c.5092G>T	Verified	ATG	GTC	AGC	1	G	T
TCGA-13-1497	SELE	ENST00000333360	p.C394F	c.1181G>T	Verified	AGC	TGT	GAG	2	G	T
TCGA-04-1338	SEMA3A	ENST00000265362	p.V295L	c.883G>T	Verified	TCA	GTG	CCA	1	G	T
TCGA-13-0760	SEMA5B	ENST00000357599	p.G42C	c.124G>T	Verified	AGG	GGT	CTT	1	G	T
TCGA-13-0883	SEPN1	ENST00000361547	p.E62*	c.184G>T	Verified	CAG	GAA	CTG	1	G	T
TCGA-13-0760	SERPINA10	ENST00000393096	p.R437M	c.1310G>T	Verified	ggc	agg	gtg	2	G	T
TCGA-24-2267	SERPINA9_E	ENST00000337425	p.M414I	c.1242G>T	Verified	ATG	ATG	ATT	3	G	T
TCGA-04-1649	SERPINB3	ENST00000283752	p.T155T	c.465G>T	Unverified	caa	acg	aat	3	G	T
TCGA-24-2024	SERPINE3	ENST00000521255	p.R201I	c.602G>T	Verified	AAG	AGA	TTC	2	G	T
TCGA-24-1423	SETD1A	ENST00000262519	p.V1094V	c.3282G>T	Verified	GAG	GTG	CCA	3	G	T
TCGA-13-0893	SETD2	ENST00000330022	p.G1156V	c.3467G>T	Verified	TCA	GGC	TCA	2	G	T
TCGA-13-0893	SETD2_ENST	ENST00000409792	p.G1659V	c.4976G>T	Verified	TCA	GGC	TCA	2	G	T

TCGA-24-1470	SETD7	ENST00000274031	p.G343V	c.1028G>T	Verified	CCC	GGG	AAG	2	G	T
TCGA-13-1510	SF3A1	ENST00000215793	p.D286Y	c.856G>T	Unverified	GTG	GAC	TTC	1	G	T
TCGA-24-2024	SF3A1	ENST00000215793	p.V711L	c.2131G>T	Verified	CAG	GTG	CCC	1	G	T
TCGA-23-1124	SF3A1	ENST00000215793	p.G478V	c.1433G>T	Verified	TTC	GGT	GTA	2	G	T
TCGA-13-0900	SFI1	ENST00000400288	p.E1154D	c.3462G>T	Unverified	CTT	GAG	GAG	3	G	T
TCGA-61-1913	SgK069	ENST00000413299	p.G85C	c.253G>T	Unverified	AAA	GGC	ACA	1	G	T
TCGA-20-1686	SGOL2	ENST00000357799	p.E92*	c.274G>T	Unverified	TTT	GAG	AAC	1	G	T
TCGA-23-1110	SGOL2	ENST00000357799	p.K1106N	c.3318G>T	Verified	GGA	AAG	TCT	3	G	T
TCGA-09-1674	SGSM2	ENST00000268989	p.A60S	c.178G>T	Unverified	GCC	GCT	GGC	1	G	T
TCGA-13-0923	SH2D3C	ENST00000314830	p.G365V	c.1094G>T	Verified	GAT	GGG	CTC	2	G	T
TCGA-61-2095	SH3GLB2	ENST00000372559	p.D131Y	c.391G>T	Unverified	AGG	GAT	TTT	1	G	T
TCGA-24-1845	SH3PXD2B	ENST00000311601	p.L587L	c.1761G>T	Unverified	AGA	CTG	TTC	3	G	T
TCGA-13-1499	SHANK2	ENST00000294018	p.D807Y	c.2419G>T	Verified	TTG	GAT	GAG	1	G	T
TCGA-04-1356	SHE	ENST00000304760	p.E324D	c.972G>T	Verified	GCA	GAG	TAC	3	G	T
TCGA-61-1725	SLAIN1	ENST00000267219	p.S300I	c.899G>T	Unverified	CGC	AGT	GCA	2	G	T
TCGA-04-1336	SLC12A3	ENST00000438926	p.G493V	c.1478G>T	Verified	ATC	GGC	TTC	2	G	T
TCGA-13-1510	SLC12A8_EN	ENST00000393469	p.D43Y	c.127G>T	Unverified	TGG	GAT	GGT	1	G	T
TCGA-13-1481	SLC13A3	ENST00000279027	p.K166N	c.498G>T	Verified	CAG	AAG	GAG	3	G	T
TCGA-24-1845	SLC16A10	ENST00000368851	p.V356L	c.1066G>T	Unverified	GGT	GTG	AAG	1	G	T
TCGA-23-1022	SLC16A11	ENST00000308009	p.S420I	c.1259G>T	Verified	GGC	AGC	TTC	2	G	T
TCGA-24-1474	SLC16A14	ENST00000295190	p.E64*	c.190G>T	Unverified	GAA	GAA	TTC	1	G	T
TCGA-13-1497	SLC16A14	ENST00000295190	p.A207A	c.621G>T	Verified	GGG	GCG	CTC	3	G	T
TCGA-23-1029	SLC16A7	ENST00000261187	p.?	c.217+1G>T	Unverified	GGA	GGT	CCT	1	G	T
TCGA-23-1114	SLC17A7	ENST00000221485	p.G421V	c.1262G>T	Unverified	TCT	GGG	TTC	2	G	T
TCGA-13-1505	SLC17A8	ENST00000323346	p.E271D	c.813G>T	Verified	TAT	GAG	TGC	3	G	T
TCGA-25-2393	SLC19A3	ENST00000258403	p.V116F	c.346G>T	Verified	ATG	GTC	ACC	1	G	T
TCGA-13-1499	SLC1A2	ENST00000278379	p.V122V	c.366G>T	Verified	ATG	GTG	TAT	3	G	T
TCGA-24-1845	SLC1A6	ENST00000221742	p.V496V	c.1488G>T	Unverified	GCC	GTG	GAC	3	G	T
TCGA-09-2049	SLC20A2	ENST00000342228	p.W518C	c.1554G>T	Verified	TTG	TGG	CTG	3	G	T
TCGA-61-2113	SLC27A5	ENST00000263093	p.L662L	c.1986G>T	Unverified	CCT	CTG	TTT	3	G	T
TCGA-24-2024	SLC2A3	ENST00000075120	p.G109V	c.326G>T	Verified	ATG	GGA	CTG	2	G	T
TCGA-09-0369	SLC2A9	ENST00000309065	p.G332G	c.996G>T	Verified	ACA	GGG	GGC	3	G	T
TCGA-09-0369	SLC2A9	ENST00000309065	p.G332G	c.996G>T	Verified	ACA	GGG	GGC	3	G	T
TCGA-04-1338	SLC30A5	ENST00000396591	p.L423F	c.1269G>T	Unverified	TTC	TTG	TGC	3	G	T

TCGA-13-1489	SLC36A2	ENST00000335244	p.G302V	c.905G>T	Verified	TTG	GGA	ATG	2	G	T
TCGA-24-2288	SLC45A2	ENST00000296589	p.V274F	c.820G>T	Unverified	AAA	GTT	AAA	1	G	T
TCGA-09-2049	SLC4A5	ENST00000394019	p.G469V	c.1406G>T	Verified	GGC	GGA	ACA	2	G	T
TCGA-24-1422	SLC4A8	ENST00000319957	p.L667L	c.2001G>T	Verified	AAC	CTG	ACT	3	G	T
TCGA-04-1367	SLC5A11	ENST00000347898	p.S222I	c.665G>T	Verified	TAC	AGT	TTT	2	G	T
TCGA-23-1124	SLC5A11	ENST00000347898	p.P272P	c.816G>T	Verified	TGG	CCG	GGG	3	G	T
TCGA-13-1496	SLC6A2	ENST00000379906	p.V10L	c.28G>T	Unverified	CAG	GTG	CAG	1	G	T
TCGA-61-1906	SLC6A2	ENST00000379906	p.?	c.1023-1G>T	Unverified	TAC	AGG	GAT	3	G	T
TCGA-61-1906	SLC6A2	ENST00000379906	p.?	c.1023-1G>T	Unverified	TAC	AGG	GAT	3	G	T
TCGA-61-1906	SLC6A2_ENS	ENST00000219833	p.?	c.1023-1G>T	Unverified	TAC	AGG	GAT	3	G	T
TCGA-61-1906	SLC6A2_ENS	ENST00000219833	p.?	c.1023-1G>T	Unverified	TAC	AGG	GAT	3	G	T
TCGA-24-1846	SLC6A4	ENST00000261707	p.G146*	c.436G>T	Unverified	AAT	GGA	TGC	1	G	T
TCGA-29-1769	SLC7A2	ENST00000494857	p.D166Y	c.496G>T	Unverified	CCC	GAT	TTT	1	G	T
TCGA-29-1769	SLC7A2_ENS	ENST00000470360	p.D206Y	c.616G>T	Unverified	CCC	GAT	TTT	1	G	T
TCGA-13-0900	SLC8A1	ENST00000403092	p.R267L	c.800G>T	Verified	TAT	CGA	GCT	2	G	T
TCGA-24-1850	SLC8A1	ENST00000403092	p.E148D	c.444G>T	Unverified	CCT	GAG	ATT	3	G	T
TCGA-23-1123	SLC8A3	ENST00000381269	p.K677N	c.2031G>T	Verified	TTC	AAG	ACT	3	G	T
TCGA-23-1123	SLC8A3	ENST00000381269	p.K677N	c.2031G>T	Verified	TTC	AAG	ACT	3	G	T
TCGA-29-1777	SLC9A5	ENST00000299798	p.G592V	c.1775G>T	Unverified	AGC	GGC	CGG	2	G	T
TCGA-09-2050	SLC9A6	ENST00000370698	p.A304S	c.910G>T	Verified	ttt	gca	atg	1	G	T
TCGA-23-1029	SLCO1A2	ENST00000307378	p.G206C	c.616G>T	Unverified	ATT	GGT	CCT	1	G	T
TCGA-09-2044	SLCO6A1	ENST00000379807	p.R60S	c.180G>T	Verified	ATA	AGG	TTC	3	G	T
TCGA-30-1857	SLIT2	ENST00000504154	p.A1470S	c.4408G>T	Unverified	GCT	GCT	TGC	1	G	T
TCGA-13-1510	SLITRK1	ENST00000377084	p.Q517H	c.1551G>T	Unverified	GAC	CAG	TTA	3	G	T
TCGA-61-2113	SLITRK3	ENST00000241274	p.E968*	c.2902G>T	Verified	CTC	GAA	GTC	1	G	T
TCGA-13-0923	SLK	NM_014720	p.E912*	c.2734G>T	Verified	caa	gag	aaa	1	G	T
TCGA-24-1470	SLX4	ENST00000294008	p.W1428C	c.4284G>T	Verified	TGC	TGG	CAC	3	G	T
TCGA-29-1693	SMARCD3	ENST00000392811	p.Q314H	c.942G>T	Unverified	ccc	cag	cgc	3	G	T
TCGA-29-1693	SMARCD3_E	ENST00000262188	p.Q327H	c.981G>T	Unverified	CCC	CAG	CGC	3	G	T
TCGA-13-1498	SMC1B	NM_148674.1	p.S482I	c.1445G>T	Verified	aga	agt	gaa	2	G	T
TCGA-24-1469	SMEK1_ENS	ENST00000417249	p.M597I	c.1791G>T	Verified	CTG	ATG	AAC	3	G	T
TCGA-04-1367	SMPDL3B	ENST00000373894	p.G185G	c.555G>T	Verified	AGC	GGG	GCT	3	G	T
TCGA-23-1117	SNAI2	ENST00000020945	p.G209G	c.627G>T	Verified	acg	ggg	gag	3	G	T
TCGA-23-1117	SNAI2	ENST00000020945	p.G209G	c.627G>T	Verified	acg	ggg	gag	3	G	T

TCGA-10-0930	SNAP91	ENST00000369694	p.A838S	c.2512G>T	Verified	GGA	GCA	GGA	1	G	T
TCGA-10-0930	SNAP91	ENST00000369694	p.A838S	c.2512G>T	Verified	GGA	GCA	GGA	1	G	T
TCGA-61-1737	SNCAIP	ENST00000261368	p.G246C	c.736G>T	Unverified	TGT	GGC	TCT	1	G	T
TCGA-61-1737	SNCAIP_ENS	ENST00000379533	p.G293C	c.877G>T	Unverified	TGT	GGC	TCT	1	G	T
TCGA-61-1737	SNCAIP_ENS	ENST00000503116	p.G293C	c.877G>T	Unverified	TGT	GGC	TCT	1	G	T
TCGA-30-1856	SNTG1	ENST00000518864	p.?	c.219+1G>T	Unverified	ATA	AAG	GGA	3	G	T
TCGA-30-1856	SNTG1_ENS	ENST00000518864	p.?	c.219+1G>T	Unverified	ATA	AAG	GGA	3	G	T
TCGA-09-0369	SNX15	ENST00000377244	p.R51R	c.153G>T	Verified	aag	cgg	tac	3	G	T
TCGA-09-0369	SNX15	ENST00000377244	p.R51R	c.153G>T	Verified	aag	cgg	tac	3	G	T
TCGA-30-1857	SNX15	ENST00000377244	p.R19S	c.57G>T	Unverified	ccc	agg	act	3	G	T
TCGA-13-1489	SNX19	ENST00000265909	p.V263L	c.787G>T	Verified	ctt	gta	ctc	1	G	T
TCGA-24-1844	SON	ENST00000356577	p.L276L	c.828G>T	Unverified	GTG	CTG	AAA	3	G	T
TCGA-24-1844	SON_ENST0	ENST00000300278	p.L276L	c.828G>T	Unverified	GTG	CTG	AAA	3	G	T
TCGA-24-2024	SORL1	ENST00000260197	p.G622*	c.1864G>T	Verified	TTG	GGA	GTT	1	G	T
TCGA-23-1117	SOS2	ENST00000216373	p.L1200L	c.3600G>T	Verified	CCT	CTG	CAT	3	G	T
TCGA-23-1117	SOS2	ENST00000216373	p.L1200L	c.3600G>T	Verified	CCT	CTG	CAT	3	G	T
TCGA-29-1777	SOX5	ENST00000451604	p.V433F	c.1297G>T	Unverified	tct	gtc	cca	1	G	T
TCGA-24-1843	SPAG5	ENST00000321765	p.V682V	c.2046G>T	Unverified	GAT	GTG	GCA	3	G	T
TCGA-09-2044	SPANXN4	ENST00000446864	p.G91*	c.271G>T	Verified	gca	gga	tct	1	G	T
TCGA-29-2427	SPATA5L1	ENST00000305560	p.L702L	c.2106G>T	Verified	TTG	CTG	GCT	3	G	T
TCGA-24-2035	SPDEF	ENST00000374037	p.K30N	c.90G>T	Unverified	gag	aag	gcg	3	G	T
TCGA-61-1907	SPDYC	ENST00000377185	p.P288P	c.864G>T	Unverified	CCT	CCG	GCA	3	G	T
TCGA-61-1913	SPEN	ENST00000375759	p.V3250F	c.9748G>T	Unverified	ccc	gtc	cct	1	G	T
TCGA-29-1785	SPHKAP	XM_051221.5	p.S1209I	c.3626G>T	Unverified	gaa	agt	gcc	2	G	T
TCGA-29-1785	SPHKAP	XM_051221.5	p.S1209I	c.3626G>T	Unverified	gaa	agt	gcc	2	G	T
TCGA-29-1785	SPHKAP_EN	ENST00000392056	p.S1209I	c.3626G>T	Unverified	GAA	AGT	GCC	2	G	T
TCGA-29-1785	SPHKAP_EN	ENST00000392056	p.S1209I	c.3626G>T	Unverified	GAA	AGT	GCC	2	G	T
TCGA-29-1690	SPIRE1	ENST00000309836	p.M92I	c.276G>T	Unverified	GAA	ATG	GAA	3	G	T
TCGA-29-1690	SPIRE1_ENS	ENST00000409402	p.M251I	c.753G>T	Unverified	GAA	ATG	GAA	3	G	T
TCGA-29-1777	SPNS3	ENST00000355530	p.V361F	c.1081G>T	Unverified	CTC	GTC	CTG	1	G	T
TCGA-24-1843	SPRR2F	ENST00000468739	p.C45F	c.134G>T	Unverified	TCC	TGC	CCA	2	G	T
TCGA-24-1469	SPRY4	ENST00000344120	p.R53L	c.158G>T	Verified	agc	cgg	ctc	2	G	T
TCGA-13-1408	SPTBN2	ENST00000309996	p.E1918*	c.5752G>T	Unverified	CGG	GAA	CTG	1	G	T
TCGA-24-1845	SPTBN2	ENST00000309996	p.V1627L	c.4879G>T	Unverified	GAG	GTG	AAG	1	G	T

TCGA-61-1737	SPTBN4	ENST00000352632	p.Q250H	c.750G>T	Unverified	GAG	CAG	CAC	3	G	T
TCGA-30-1718	SRRM2	ENST00000301740	p.G1819V	c.5456G>T	Unverified	TCT	GGT	TAT	2	G	T
TCGA-24-1423	SRSF11	ENST00000370950	p.E478*	c.1432G>T	Verified	GAA	GAA	GAC	1	G	T
TCGA-23-1122	SSH3	ENST00000308127	p.G218C	c.652G>T	Verified	AGC	GGC	CTT	1	G	T
TCGA-24-1431	SSTR3	ENST00000328544	p.V260V	c.780G>T	Unverified	GTG	GTG	GCC	3	G	T
TCGA-20-1683	ST18	ENST00000276480	p.E944*	c.2830G>T	Unverified	ATT	GAA	GCA	1	G	T
TCGA-23-2077	ST6GALNAC	ENST00000328299	p.R7I	c.20G>T	Verified	AAG	AGA	AAG	2	G	T
TCGA-61-1740	STARD4	ENST00000296632	p.K141N	c.423G>T	Unverified	gaa	aag	aga	3	G	T
TCGA-13-1512	STAT5B	ENST00000293328	p.A91S	c.271G>T	Verified	tat	gcc	aca	1	G	T
TCGA-04-1331	STK36	ENST00000295709	p.G811C	c.2431G>T	Verified	CTT	GGT	CAG	1	G	T
TCGA-13-0795	STK38	ENST00000229812	p.K354N	c.1062G>T	Verified	gcc	aag	gat	3	G	T
TCGA-29-1769	STOX1	ENST00000399169	p.L537F	c.1611G>T	Unverified	TCC	TTG	GAT	3	G	T
TCGA-29-1777	SUGP2	ENST00000337018	p.V412V	c.1236G>T	Unverified	GCT	GTG	AAG	3	G	T
TCGA-09-2044	SULF2	ENST00000359930	p.A128S	c.382G>T	Verified	ttt	gcc	gtg	1	G	T
TCGA-04-1367	SUPV3L1	ENST00000359655	p.G248C	c.742G>T	Verified	gct	ggt	gtg	1	G	T
TCGA-29-1763	SUSD4_ENST	ENST00000343846	p.E262D	c.786G>T	Unverified	TGT	GAG	CGC	3	G	T
TCGA-13-0760	SUV420H1	NM_017635.3	p.K761N	c.2283G>T	Verified	gca	aag	ctt	3	G	T
TCGA-13-0791	SV2A	ENST00000369146	p.G319W	c.955G>T	Verified	TAT	GGG	TGG	1	G	T
TCGA-13-1507	SV2A	ENST00000369146	p.T399T	c.1197G>T	Verified	AAG	ACG	ATT	3	G	T
TCGA-24-1563	SV2A	ENST00000369146	p.W454C	c.1362G>T	Verified	GTG	TGG	TTC	3	G	T
TCGA-61-1733	SV2C	ENST00000502798	p.D119Y	c.355G>T	Unverified	AAG	GAC	CGG	1	G	T
TCGA-61-1733	SV2C	ENST00000502798	p.D119Y	c.355G>T	Unverified	AAG	GAC	CGG	1	G	T
TCGA-61-1910	SV2C	ENST00000502798	p.V30L	c.88G>T	Unverified	AAG	GTG	AAT	1	G	T
TCGA-29-1777	SWAP70	ENST00000318950	p.C94F	c.281G>T	Unverified	ATG	TGT	TGG	2	G	T
TCGA-23-1124	SYN2	ENST00000341648	p.L349L	c.1047G>T	Verified	CTG	CTG	TCC	3	G	T
TCGA-13-1498	SYNE1	ENST00000265368	p.E3409*	c.10225G>T	Unverified	ATG	GAA	GCC	1	G	T
TCGA-13-0919	SYNE1	ENST00000265368	p.E5246D	c.15738G>T	Verified	GCA	GAG	CTC	3	G	T
TCGA-13-1498	SYNE1_ENST	ENST00000265368	p.E3409*	c.10225G>T	Unverified	ATG	GAA	GCC	1	G	T
TCGA-13-0919	SYNE1_ENST	ENST00000265368	p.E5246D	c.15738G>T	Verified	GCA	GAG	CTC	3	G	T
TCGA-13-0893	SYNGAP1	ENST00000418600	p.S1088I	c.3263G>T	Unverified	TCC	AGC	GGG	2	G	T
TCGA-13-0893	SYNGAP1_EI	ENST00000293748	p.S1088I	c.3263G>T	Unverified	TCC	AGC	GGG	2	G	T
TCGA-04-1343	SYT9	ENST00000318881	p.L403L	c.1209G>T	Verified	CGA	CTG	AAG	3	G	T
TCGA-29-1777	SZT2	ENST00000372442	p.D634Y	c.1900G>T	Unverified	TCA	GAC	GTA	1	G	T
TCGA-29-1777	SZT2_ENST0	ENST00000562955	p.D1476Y	c.4426G>T	Unverified	TCA	GAC	GTA	1	G	T

TCGA-13-0791	TAAR6	ENST00000275198	p.R254I	c.761G>T	Verified	GAG	AGA	AAA	2	G	T
TCGA-13-0755	TACC2	ENST00000369005	p.A1755S	c.5263G>T	Verified	AAG	GCT	CCG	1	G	T
TCGA-24-1431	TACR2	ENST00000373306	p.D278Y	c.832G>T	Unverified	GAG	GAC	ATC	1	G	T
TCGA-29-1763	TAGAP	ENST00000367066	p.G180V	c.539G>T	Unverified	ATG	GGT	GCT	2	G	T
TCGA-24-2288	TANC1	ENST00000263635	p.G1449V	c.4346G>T	Unverified	CCT	GGC	TTA	2	G	T
TCGA-09-1674	TARDBP	ENST00000240185	p.G245*	c.733G>T	Unverified	TGT	GGA	GAG	1	G	T
TCGA-20-1687	TATDN2	ENST00000287652	p.A690S	c.2068G>T	Unverified	GAG	GCC	CGG	1	G	T
TCGA-04-1347	TBC1D8	ENST00000409318	p.M425I	c.1275G>T	Verified	GAC	ATG	GCT	3	G	T
TCGA-13-0906	TBCCD1	ENST00000338733	p.V115L	c.343G>T	Verified	TCA	GTG	GAC	1	G	T
TCGA-13-0923	TBX18	ENST00000369663	p.C395F	c.1184G>T	Verified	TCT	TGC	TCC	2	G	T
TCGA-61-2012	TBX3	ENST00000257566	p.D27Y	c.79G>T	Verified	CCG	GAC	TTC	1	G	T
TCGA-29-1761	TBX5	ENST00000310346	p.R246I	c.737G>T	Unverified	CAC	AGA	ATG	2	G	T
TCGA-29-1761	TBX5_ENST(	ENST00000526441	p.R246I	c.737G>T	Unverified	CAC	AGA	ATG	2	G	T
TCGA-23-1117	TCF4	ENST00000356073	p.G658*	c.1972G>T	Verified	ATG	GGA	GAC	1	G	T
TCGA-23-1117	TCF4	ENST00000356073	p.G658*	c.1972G>T	Verified	ATG	GGA	GAC	1	G	T
TCGA-09-2044	TCN1	ENST00000257264	p.K121N	c.363G>T	Verified	GAC	AAG	CTA	3	G	T
TCGA-09-1665	TCP11	ENST00000373979	p.G334C	c.1000G>T	Verified	ATG	GGC	CTT	1	G	T
TCGA-13-0807	TCP11	ENST00000373979	p.V270F	c.808G>T	Verified	ACC	GTC	ATG	1	G	T
TCGA-23-1022	TDG	ENST00000392872	p.D202Y	c.604G>T	Verified	AAA	GAT	CTC	1	G	T
TCGA-30-1718	TDRD1	ENST00000251864	p.?	c.2770+1G>T	Unverified	CAA	GCT	ACC	1	G	T
TCGA-23-1118	TDRD9	ENST00000339063	p.A478A	c.1434G>T	Verified	ATG	GCG	GTG	3	G	T
TCGA-13-0916	TDRKH	ENST00000368822	p.G22V	c.65G>T	Verified	CTT	GGG	ATC	2	G	T
TCGA-24-0979	TECTA	ENST00000392793	p.V1618L	c.4852G>T	Verified	AAA	GTG	TGC	1	G	T
TCGA-20-1687	TECTA	ENST00000392793	p.Q945H	c.2835G>T	Unverified	TGC	CAG	AGT	3	G	T
TCGA-13-1481	TEK	ENST00000380036	p.V1014L	c.3040G>T	Verified	AGT	GTG	TAC	1	G	T
TCGA-09-1665	TEKT1	ENST00000338694	p.R119M	c.356G>T	Verified	TAC	AGG	GAG	2	G	T
TCGA-30-1855	TESK2	NM_007170	p.D2Y	c.4G>T	Unverified	atg	gat	cgg	1	G	T
TCGA-29-1777	TEX14	ENST00000349033	p.R1355I	c.4064G>T	Unverified	GAG	AGA	GCT	2	G	T
TCGA-29-1777	TEX14_ENST	ENST00000240361	p.R1401I	c.4202G>T	Unverified	GAG	AGA	GCT	2	G	T
TCGA-25-2393	TF	ENST00000402696	p.W363C	c.1089G>T	Verified	AAG	TGG	TGT	3	G	T
TCGA-13-0761	TFDP1	ENST00000375370	p.E208*	c.622G>T	Verified	gtg	gaa	aga	1	G	T
TCGA-13-0761	TFDP1	ENST00000375370	p.E208*	c.622G>T	Verified	gtg	gaa	aga	1	G	T
TCGA-13-0883	TG	ENST00000220616	p.G2341V	c.7022G>T	Verified	TTC	GGC	TTC	2	G	T
TCGA-25-2393	TGFBR1	ENST00000374994	p.S241S	c.723G>T	Verified	cgt	tcg	tgg	3	G	T

TCGA-24-2267	TGIF1	ENST00000330513	p.V176L	c.526G>T	Verified	tct	gtg	cag	1	G	T
TCGA-24-1563	TGM6	ENST00000202625	p.R293R	c.879G>T	Verified	ACA	CGG	GTC	3	G	T
TCGA-24-1849	TGM7	ENST00000452443	p.L508L	c.1524G>T	Unverified	CAG	CTG	CTG	3	G	T
TCGA-29-1777	THAP9	ENST00000302236	p.R524S	c.1572G>T	Unverified	AGT	AGG	AAC	3	G	T
TCGA-29-1762	THEM5	ENST00000368817	p.R96R	c.288G>T	Unverified	ATC	CGG	GGA	3	G	T
TCGA-13-1488	THEMIS	ENST00000368248	p.R19S	c.57G>T	Verified	CCC	AGG	GTT	3	G	T
TCGA-13-0755	THSD1	ENST00000258613	p.V125L	c.373G>T	Verified	AAG	GTG	GAA	1	G	T
TCGA-24-2280	TIE1	ENST00000372476	p.V1041F	c.3121G>T	Unverified	GGA	GTC	CTT	1	G	T
TCGA-09-2044	TKTL2	ENST00000280605	p.C447F	c.1340G>T	Verified	AAT	TGT	ACT	2	G	T
TCGA-61-2113	TLL1	ENST00000061240	p.V239F	c.715G>T	Verified	GTT	GTT	CAT	1	G	T
TCGA-13-0904	TLN1	ENST00000314888	p.L152L	c.456G>T	Verified	TTG	CTG	CGA	3	G	T
TCGA-23-1031	TLN1	ENST00000314888	p.Q2259H	c.6777G>T	Unverified	CTG	CAG	AAG	3	G	T
TCGA-24-1843	TLR1	ENST00000308979	p.R18I	c.53G>T	Unverified	ATC	AGA	ATA	2	G	T
TCGA-13-0760	TLR4	ENST00000355622	p.C40F	c.119G>T	Verified	CAA	TGC	ATG	2	G	T
TCGA-30-1891	TM4SF4	ENST00000305354	p.G181C	c.541G>T	Unverified	AAT	GGC	CTC	1	G	T
TCGA-29-1693	TMEFF2	ENST00000272771	p.E126D	c.378G>T	Unverified	AGT	GAG	ATA	3	G	T
TCGA-29-1769	TMEM131_E	ENST00000186436	p.A664S	c.1990G>T	Unverified	AAG	GCT	GTG	1	G	T
TCGA-23-1118	TMEM132B	ENST00000299308	p.L431F	c.1293G>T	Verified	GTT	TTG	AAC	3	G	T
TCGA-04-1347	TMEM132D	ENST00000422113	p.E953*	c.2857G>T	Verified	TTC	GAG	GAG	1	G	T
TCGA-09-2051	TMEM156	ENST00000381938	p.M171I	c.513G>T	Unverified	ATC	ATG	GAG	3	G	T
TCGA-13-1497	TMEM181	ENST00000367090	p.S191I	c.572G>T	Verified	AAT	AGC	AAA	2	G	T
TCGA-13-1512	TMEM198	ENST00000344458	p.V113L	c.337G>T	Verified	CTA	GTG	CGC	1	G	T
TCGA-24-1845	TMEM61	ENST00000371268	p.A51S	c.151G>T	Unverified	CTG	GCC	CCA	1	G	T
TCGA-25-2042	TMEM86A	ENST00000280734	p.V123L	c.367G>T	Verified	CTG	GTG	ATG	1	G	T
TCGA-13-0886	TMEM97	ENST00000226230	p.S20I	c.59G>T	Unverified	CTC	AGC	CAC	2	G	T
TCGA-23-1117	TMOD4	ENST00000295314	p.G237V	c.710G>T	Verified	AGT	GGT	GAC	2	G	T
TCGA-23-1117	TMOD4	ENST00000295314	p.G237V	c.710G>T	Verified	AGT	GGT	GAC	2	G	T
TCGA-61-1998	TMPO_ENST	ENST00000266732	p.E496*	c.1486G>T	Verified	CAT	GAA	TCT	1	G	T
TCGA-25-1313	TMPRSS15	ENST00000284885	p.C354F	c.1061G>T	Verified	TTT	TGT	TTC	2	G	T
TCGA-24-1464	TMTC2	ENST00000321196	p.S7I	c.20G>T	Verified	GTG	AGC	AGC	2	G	T
TCGA-61-2012	TMTC2	ENST00000321196	p.K614N	c.1842G>T	Verified	GGA	AAG	CTG	3	G	T
TCGA-24-2289	TNFRSF10A	ENST00000221132	p.C132F	c.395G>T	Unverified	TTG	TGT	CCA	2	G	T
TCGA-13-0890	TNIK	ENST00000436636	p.Q457H	c.1371G>T	Verified	AGA	CAG	TTA	3	G	T
TCGA-09-2051	TNKS1BP1	ENST00000358252	p.D922Y	c.2764G>T	Unverified	CAG	GAT	GCC	1	G	T

TCGA-30-1855	TNNI3K	ENST00000326637	p.M798I	c.2394G>T	Unverified	gag	atg	aaa	3	G	T
TCGA-61-1737	TNPO3	ENST00000265388	p.C665F	c.1994G>T	Unverified	agg	tgc	ctg	2	G	T
TCGA-61-1737	TNPO3_ENS	ENST00000393245	p.C699F	c.2096G>T	Unverified	AGG	TGC	CTG	2	G	T
TCGA-23-2078	TNR	ENST00000367674	p.K11N	c.33G>T	Verified	CTG	AAG	AAC	3	G	T
TCGA-24-1435	TNRC6A	ENST00000395799	p.G1061V	c.3182G>T	Verified	TGG	GGT	GAG	2	G	T
TCGA-23-1118	TNRC6C	ENST00000301624	p.D566Y	c.1696G>T	Verified	GAG	GAC	AAG	1	G	T
TCGA-13-1488	TNS1	ENST00000171887	p.K249N	c.747G>T	Verified	CTG	AAG	TGC	3	G	T
TCGA-13-0916	TNXB_ENST(	ENST00000375247	p.A1070S	c.3208G>T	Verified	ACC	GCC	CAG	1	G	T
TCGA-24-1469	TOP1	ENST00000361337	p.A96S	c.286G>T	Verified	cga	gcc	tct	1	G	T
TCGA-04-1651	TP53	ENST00000269305	p.E343*	c.1027G>T	Unverified	CGA	GAG	CTG	1	G	T
TCGA-04-1651	TP53	ENST00000269305	p.E343*	c.1027G>T	Unverified	CGA	GAG	CTG	1	G	T
TCGA-09-1665	TP53	ENST00000269305	p.D259Y	c.775G>T	Verified	GAA	GAC	TCC	1	G	T
TCGA-13-0919	TP53	ENST00000269305	p.V157F	c.469G>T	Verified	CGC	GTC	CGC	1	G	T
TCGA-61-1915	TP53	ENST00000269305	p.E221*	c.661G>T	Unverified	TAT	GAG	CCG	1	G	T
TCGA-61-2009	TP53	ENST00000269305	p.V157F	c.469G>T	Verified	CGC	GTC	CGC	1	G	T
TCGA-13-0791	TP53	ENST00000269305	p.R273L	c.818G>T	Verified	GTG	CGT	GTT	2	G	T
TCGA-13-1512	TP53	ENST00000269305	p.R273L	c.818G>T	Verified	GTG	CGT	GTT	2	G	T
TCGA-23-1120	TP53	ENST00000269305	p.R110L	c.329G>T	Verified	TTC	CGT	CTG	2	G	T
TCGA-61-2113	TP53	ENST00000269305	p.G266V	c.797G>T	Verified	CTG	GGA	CGG	2	G	T
TCGA-13-0755	TP53	ENST00000269305	p.T125T	c.375G>T	Verified	TGC	ACG	TAC	3	G	T
TCGA-24-2262	TP53	ENST00000269305	p.K132N	c.396G>T	Verified	AAC	AAG	ATG	3	G	T
TCGA-25-2400	TP53	ENST00000269305	p.?	c.672+1G>T	Verified	CCT	GAG	GTT	3	G	T
TCGA-36-1577	TP53	ENST00000269305	p.?	c.672+1G>T	Verified	CCT	GAG	GTT	3	G	T
TCGA-04-1651	TP53_ENST(	ENST00000269305	p.E343*	c.1027G>T	Unverified	CGA	GAG	CTG	1	G	T
TCGA-04-1651	TP53_ENST(	ENST00000269305	p.E343*	c.1027G>T	Unverified	CGA	GAG	CTG	1	G	T
TCGA-61-1915	TP53_ENST(	ENST00000269305	p.E221*	c.661G>T	Unverified	TAT	GAG	CCG	1	G	T
TCGA-23-1120	TP53_ENST(	ENST00000269305	p.R110L	c.329G>T	Verified	TTC	CGT	CTG	2	G	T
TCGA-36-1577	TP53_ENST(	ENST00000269305	p.?	c.672+1G>T	Verified	CCT	GAG	GTT	3	G	T
TCGA-61-1915	TP53_ENST(	ENST00000413465	p.E221*	c.661G>T	Unverified	TAT	GAG	CCG	1	G	T
TCGA-23-1120	TP53_ENST(	ENST00000413465	p.R110L	c.329G>T	Verified	TTC	CGT	CTG	2	G	T
TCGA-36-1577	TP53_ENST(	ENST00000413465	p.?	c.672+1G>T	Verified	CCT	GAG	GTT	3	G	T
TCGA-61-1915	TP53_ENST(	ENST00000545858	p.E128*	c.382G>T	Unverified	TAT	GAG	CCG	1	G	T
TCGA-36-1577	TP53_ENST(	ENST00000545858	p.?	c.393+1G>T	Verified	CCT	GAG	GTT	3	G	T
TCGA-13-0807	TP73	ENST00000378295	p.Q133H	c.399G>T	Verified	CAG	CAG	TCC	3	G	T

TCGA-61-2095	TPBG	ENST00000369750	p.G136C	c.406G>T	Unverified	GCG	GGC	GCC	1	G	T
TCGA-09-0369	TRAF3IP3	NM_025228.1	p.?	c.855+1G>T	Verified	aca	cag	cga	3	G	T
TCGA-09-0369	TRAF3IP3	NM_025228.1	p.?	c.855+1G>T	Verified	aca	cag	cga	3	G	T
TCGA-29-1695	TRDMT1	ENST00000377799	p.V291V	c.873G>T	Unverified	tcc	gtg	tgc	3	G	T
TCGA-13-0885	TRIM13	ENST00000420995	p.V401L	c.1201G>T	Verified	ttt	gtg	tgc	1	G	T
TCGA-29-1777	TRIM17	ENST00000366697	p.C224F	c.671G>T	Unverified	gcc	tgc	ctg	2	G	T
TCGA-29-1777	TRIM17_ENS	ENST00000456946	p.C224F	c.671G>T	Unverified	GCC	TGC	CTG	2	G	T
TCGA-24-1616	TRIM43_ENS	ENST00000272395	p.C30F	c.89G>T	Unverified	TGC	TGT	GGG	2	G	T
TCGA-13-0913	TRIM63	ENST00000374272	p.A157S	c.469G>T	Unverified	GTG	GCC	CCA	1	G	T
TCGA-29-1777	TRIO	ENST00000344204	p.V706L	c.2116G>T	Unverified	gcc	gtg	cag	1	G	T
TCGA-61-1737	TRIOBP_ENS	ENST00000406386	p.E876D	c.2628G>T	Unverified	AAG	GAG	AAT	3	G	T
TCGA-23-1022	TRIP4	ENST00000261884	p.?	c.618+1G>T	Verified	ACT	CTG	GTG	3	G	T
TCGA-29-1703	TRPC4	ENST00000379705	p.V839L	c.2515G>T	Unverified	TTT	GTG	ACC	1	G	T
TCGA-30-1855	TRPC4	ENST00000379705	p.E648*	c.1942G>T	Unverified	TTT	GAA	GAA	1	G	T
TCGA-29-1703	TRPC4_ENST	ENST00000379681	p.V844L	c.2530G>T	Unverified	TTT	GTG	ACC	1	G	T
TCGA-30-1855	TRPC4_ENST	ENST00000379681	p.E648*	c.1942G>T	Unverified	TTT	GAA	GAA	1	G	T
TCGA-25-2392	TRPM3	ENST00000377106	p.V971V	c.2913G>T	Verified	TTA	GTG	GCA	3	G	T
TCGA-13-0913	TRPV5	ENST00000265310	p.R355L	c.1064G>T	Verified	TTT	CGT	GGT	2	G	T
TCGA-23-1118	TRRAP	ENST00000355540	p.V3329V	c.9987G>T	Verified	GCG	GTG	TCC	3	G	T
TCGA-24-1845	TRRAP	ENST00000355540	p.Q1381H	c.4143G>T	Unverified	CCT	CAG	TCC	3	G	T
TCGA-24-1845	TRRAP_ENST	ENST00000359863	p.Q1381H	c.4143G>T	Unverified	CCT	CAG	TCC	3	G	T
TCGA-24-0975	TSEN54	ENST00000333213	p.V498F	c.1492G>T	Unverified	GAT	GTC	CCT	1	G	T
TCGA-61-2012	TSHZ2	ENST00000371497	p.K887N	c.2661G>T	Verified	TCT	AAG	TTT	3	G	T
TCGA-24-2267	TSPAN10	ENST00000328585	p.V254F	c.760G>T	Verified	TCT	GTC	AAC	1	G	T
TCGA-13-1497	TSPAN9	ENST00000011898	p.G29*	c.85G>T	Verified	CTG	GGA	GTG	1	G	T
TCGA-61-1738	TSPEAR	ENST00000323084	p.A44S	c.130G>T	Unverified	GGC	GCC	ACA	1	G	T
TCGA-61-1738	TSPEAR	ENST00000323084	p.A44S	c.130G>T	Unverified	GGC	GCC	ACA	1	G	T
TCGA-04-1337	TSPYL1	ENST00000368608	p.E225*	c.673G>T	Unverified	CAT	GAG	GCT	1	G	T
TCGA-61-1910	TTC12	ENST00000529221	p.L413L	c.1239G>T	Unverified	GCT	CTG	GAA	3	G	T
TCGA-29-1691	TTC21B	ENST00000243344	p.?	c.552+1G>T	Unverified	GGT	AAG	GCA	3	G	T
TCGA-29-1777	TTC30A	ENST00000355689	p.G623V	c.1868G>T	Unverified	TAT	GGC	ACA	2	G	T
TCGA-24-1616	TTC37	ENST00000358746	p.A680S	c.2038G>T	Verified	ATG	GCA	AAA	1	G	T
TCGA-24-1616	TTC37	ENST00000358746	p.E630*	c.1888G>T	Verified	CCA	GAA	TCC	1	G	T
TCGA-24-2290	TTF1	ENST00000334270	p.K747N	c.2241G>T	Verified	ACC	AAG	AGG	3	G	T

TCGA-24-2290	TTF1	ENST00000334270	p.K747N	c.2241G>T	Verified	ACC	AAG	AGG	3	G	T
TCGA-13-0886	TTLL4	ENST00000392102	p.R1008R	c.3024G>T	Unverified	GTT	CGG	ATT	3	G	T
TCGA-13-0904	TTLL8	ENST00000266182	p.V374V	c.1122G>T	Verified	TGG	GTG	GTC	3	G	T
TCGA-25-2042	TTN	NM_003319	p.E3995*	c.11983G>T	Verified	att	gag	tct	1	G	T
TCGA-30-1855	TTN	NM_003319	p.E8152*	c.24454G>T	Unverified	aaa	gag	tac	1	G	T
TCGA-61-1737	TTN	NM_003319	p.V23577L	c.70729G>T	Unverified	aaa	gta	gat	1	G	T
TCGA-09-0369	TTN	NM_003319	p.G5678V	c.17033G>T	Verified	ggt	ggg	gtg	2	G	T
TCGA-09-0369	TTN	NM_003319	p.G5678V	c.17033G>T	Verified	ggt	ggg	gtg	2	G	T
TCGA-13-0755	TTN	NM_003319	p.E3925D	c.11775G>T	Verified	tat	gag	ccc	3	G	T
TCGA-23-1021	TTN	NM_003319	p.V11423V	c.34269G>T	Verified	acc	gtg	aga	3	G	T
TCGA-30-1855	TTN_ENST0(	ENST00000342175	p.E8344*	c.25030G>T	Unverified	AAA	GAG	TAC	1	G	T
TCGA-61-1737	TTN_ENST0(	ENST00000342175	p.V23769L	c.71305G>T	Unverified	AAA	GTA	GAT	1	G	T
TCGA-30-1891	TTN_ENST0(	ENST00000342175	p.R19550L	c.58649G>T	Unverified	AGA	CGA	GAC	2	G	T
TCGA-24-1846	TTN_ENST0(	ENST00000342992	p.E6708*	c.20122G>T	Unverified	GCC	GAA	ATG	1	G	T
TCGA-30-1855	TTN_ENST0(	ENST00000342992	p.E14649*	c.43945G>T	Unverified	AAA	GAG	TAC	1	G	T
TCGA-61-1737	TTN_ENST0(	ENST00000342992	p.V30074L	c.90220G>T	Unverified	AAA	GTA	GAT	1	G	T
TCGA-61-1740	TTN_ENST0(	ENST00000342992	p.?	c.14857+1G>T	Unverified	AAA	GAA	CCC	1	G	T
TCGA-30-1855	TTN_ENST0(	ENST00000356127	p.E14649*	c.43945G>T	Unverified	AAA	GAG	TAC	1	G	T
TCGA-61-1737	TTN_ENST0(	ENST00000356127	p.V30072L	c.90214G>T	Unverified	AAA	GTA	GAT	1	G	T
TCGA-09-0369	TTN_ENST0(	ENST00000356127	p.G12175V	c.36524G>T	Verified	GGT	GGG	GTG	2	G	T
TCGA-09-0369	TTN_ENST0(	ENST00000356127	p.G12175V	c.36524G>T	Verified	GGT	GGG	GTG	2	G	T
TCGA-30-1891	TTN_ENST0(	ENST00000356127	p.R25853L	c.77558G>T	Unverified	AGA	CGA	GAC	2	G	T
TCGA-13-1497	TTN_ENST0(	ENST00000356127	p.K3558N	c.10674G>T	Verified	CCT	AAG	TCC	3	G	T
TCGA-23-1021	TTN_ENST0(	ENST00000356127	p.V17918V	c.53754G>T	Verified	ACC	GTG	AGA	3	G	T
TCGA-30-1855	TTN_ENST0(	ENST00000359218	p.E8277*	c.24829G>T	Unverified	AAA	GAG	TAC	1	G	T
TCGA-61-1737	TTN_ENST0(	ENST00000359218	p.V23702L	c.71104G>T	Unverified	AAA	GTA	GAT	1	G	T
TCGA-30-1855	TUBA1B	ENST00000336023	p.?	c.226+1G>T	Unverified	ATT	GAT	GAA	1	G	T
TCGA-30-1855	TUBB1	ENST00000217133	p.E383D	c.1149G>T	Unverified	TCT	GAG	CAT	3	G	T
TCGA-24-2288	TUBB8	ENST00000328974	p.G308C	c.922G>T	Unverified	CAC	GGC	CGC	1	G	T
TCGA-24-1470	TUBG1	ENST00000251413	p.R124L	c.371G>T	Verified	GAC	CGG	GAG	2	G	T
TCGA-04-1361	TXLNA	ENST00000373609	p.G509V	c.1526G>T	Verified	CCT	GGG	GCT	2	G	T
TCGA-04-1361	TXLNA	ENST00000373609	p.G509V	c.1526G>T	Verified	CCT	GGG	GCT	2	G	T
TCGA-09-0369	TXNDC3	ENST00000199447	p.G265V	c.794G>T	Verified	CCT	GGA	ATG	2	G	T
TCGA-09-0369	TXNDC3	ENST00000199447	p.G265V	c.794G>T	Verified	CCT	GGA	ATG	2	G	T

TCGA-04-1367	TXNL4B	ENST00000268483	p.L115F	c.345G>T	Verified	GAT	TTG	ATT	3	G	T
TCGA-23-1117	UBE2E3	ENST00000392415	p.A22A	c.66G>T	Verified	gat	gcg	gac	3	G	T
TCGA-23-1117	UBE2E3	ENST00000392415	p.A22A	c.66G>T	Verified	gat	gcg	gac	3	G	T
TCGA-13-1501	UBE2Q2	ENST00000267938	p.?	c.448-1G>T	Verified	GAA	GAT	ATA	1	G	T
TCGA-13-1501	UBE2Q2	ENST00000267938	p.?	c.448-1G>T	Verified	GAA	GAT	ATA	1	G	T
TCGA-24-2288	UBP1	ENST00000283629	p.S36I	c.107G>T	Unverified	TAC	AGC	ATG	2	G	T
TCGA-24-1470	UBQLN2	ENST00000338222	p.G289V	c.866G>T	Verified	GGG	GGT	AAT	2	G	T
TCGA-24-1422	UBR3	ENST00000392632	p.A328S	c.982G>T	Unverified	GGT	GCA	AGC	1	G	T
TCGA-24-1422	UBR3_ENST(	ENST00000418381	p.A1475S	c.4423G>T	Unverified	GGT	GCA	AGC	1	G	T
TCGA-24-1469	UBR4	ENST00000375254	p.R3611L	c.10832G>T	Unverified	GCT	CGC	TGG	2	G	T
TCGA-61-1914	UBXN6	ENST00000301281	p.E107*	c.319G>T	Unverified	TCT	GAG	CCC	1	G	T
TCGA-13-0884	UBXN7	ENST00000296328	p.D246Y	c.736G>T	Verified	TTA	GAT	GTA	1	G	T
TCGA-09-1674	UGT1A1	ENST00000305208	p.G493C	c.1477G>T	Unverified	att	ggt	ttc	1	G	T
TCGA-09-1674	UGT1A10	ENST00000344644	p.G490C	c.1468G>T	Unverified	ATT	GGT	TTC	1	G	T
TCGA-09-1674	UGT1A3	ENST00000482026	p.G494C	c.1480G>T	Unverified	ATT	GGT	TTC	1	G	T
TCGA-09-1674	UGT1A4	ENST00000373409	p.G494C	c.1480G>T	Unverified	ATT	GGT	TTC	1	G	T
TCGA-09-1674	UGT1A5	ENST00000373414	p.G494C	c.1480G>T	Unverified	ATT	GGT	TTC	1	G	T
TCGA-09-1674	UGT1A6	ENST00000305139	p.G492C	c.1474G>T	Unverified	ATT	GGT	TTC	1	G	T
TCGA-09-1674	UGT1A7	ENST00000373426	p.G490C	c.1468G>T	Unverified	att	ggt	ttc	1	G	T
TCGA-09-1674	UGT1A8	ENST00000373450	p.G490C	c.1468G>T	Unverified	ATT	GGT	TTC	1	G	T
TCGA-09-1674	UGT1A9	ENST00000354728	p.G490C	c.1468G>T	Unverified	att	ggt	ttc	1	G	T
TCGA-04-1651	UGT2B4	ENST00000305107	p.G522V	c.1565G>T	Unverified	ACA	GGA	AAG	2	G	T
TCGA-04-1651	UGT2B4	ENST00000305107	p.G522V	c.1565G>T	Unverified	ACA	GGA	AAG	2	G	T
TCGA-13-0924	UGT3A1	ENST00000274278	p.L455L	c.1365G>T	Verified	CGG	CTG	GTG	3	G	T
TCGA-24-2280	ULK2_ENST(	ENST00000361658	p.Q527H	c.1581G>T	Unverified	CTG	CAG	AGC	3	G	T
TCGA-24-2024	UNC13B	ENST00000378495	p.A926S	c.2776G>T	Verified	AAG	GCC	TGT	1	G	T
TCGA-29-1696	UNC79	ENST00000553484	p.C800F	c.2399G>T	Unverified	AAT	TGT	TTC	2	G	T
TCGA-29-1696	UNC79	ENST00000553484	p.C800F	c.2399G>T	Unverified	AAT	TGT	TTC	2	G	T
TCGA-24-1563	USH1C	ENST00000005226	p.S174I	c.521G>T	Verified	AAA	AGC	TCT	2	G	T
TCGA-13-0904	USH2A	ENST00000307340	p.V2829L	c.8485G>T	Verified	TCT	GTG	ATT	1	G	T
TCGA-13-1507	USH2A	ENST00000307340	p.R3258R	c.9774G>T	Verified	AAT	CGG	GTT	3	G	T
TCGA-24-1843	USH2A	ENST00000307340	p.G3879G	c.11637G>T	Unverified	CTG	GGG	TCA	3	G	T
TCGA-24-1844	USH2A	ENST00000307340	p.L4656F	c.13968G>T	Unverified	CTT	TTG	TGG	3	G	T
TCGA-13-1498	USP19	XM_496642.1	p.L1182L	c.3546G>T	Verified	cag	ctg	ttg	3	G	T

TCGA-04-1338	USP34	ENST00000398571	p.G584G	c.1752G>T	Verified	AGT	GGG	CAT	3	G	T
TCGA-29-1761	USP35	XM_290527.3	p.L580L	c.1740G>T	Unverified	atc	ctg	gat	3	G	T
TCGA-29-1761	USP35_ENS⁻	ENST00000529308	p.L824L	c.2472G>T	Unverified	ATC	CTG	GAT	3	G	T
TCGA-29-1691	USP40	ENST00000450966	p.A626S	c.1876G>T	Unverified	ATT	GCT	GAT	1	G	T
TCGA-29-1691	USP40_ENS⁻	ENST00000450966	p.A626S	c.1876G>T	Unverified	ATT	GCT	GAT	1	G	T
TCGA-04-1347	USP43	ENST00000285199	p.R788R	c.2364G>T	Verified	GTA	CGG	GGC	3	G	T
TCGA-24-1474	USP45	ENST00000500704	p.G525*	c.1573G>T	Verified	agt	gga	tcc	1	G	T
TCGA-29-1698	USP6NL	ENST00000277575	p.A484S	c.1450G>T	Unverified	cac	gca	gct	1	G	T
TCGA-61-1904	USP7	ENST00000344836	p.K1022N	c.3066G>T	Unverified	ATG	AAG	CGA	3	G	T
TCGA-20-0990	UTP14A	ENST00000394422	p.E269*	c.805G>T	Verified	ttt	gag	cag	1	G	T
TCGA-23-1110	VARS2	ENST00000321897	p.R754L	c.2261G>T	Verified	CTT	CGC	TTT	2	G	T
TCGA-24-1103	VAV3	ENST00000370056	p.E205*	c.613G>T	Verified	GAA	GAA	AAA	1	G	T
TCGA-24-1847	VAV3	ENST00000370056	p.R103L	c.308G>T	Unverified	GTT	CGT	GAC	2	G	T
TCGA-13-0924	VCPIP1	ENST00000310421	p.G670C	c.2008G>T	Verified	gat	ggt	gct	1	G	T
TCGA-24-2288	VDR	ENST00000395324	p.D149Y	c.445G>T	Unverified	tcc	gac	ttc	1	G	T
TCGA-29-1777	VIPAR	ENST00000343765	p.A254S	c.760G>T	Unverified	CTT	GCG	CTA	1	G	T
TCGA-04-1651	VPS13B	ENST00000358544	p.A1013S	c.3037G>T	Unverified	cag	gcc	tct	1	G	T
TCGA-04-1651	VPS13B	ENST00000358544	p.A1013S	c.3037G>T	Unverified	cag	gcc	tct	1	G	T
TCGA-13-1408	VPS13B	ENST00000358544	p.M3637I	c.10911G>T	Unverified	gca	atg	cac	3	G	T
TCGA-04-1651	VPS13B_EN!	ENST00000357162	p.A1013S	c.3037G>T	Unverified	CAG	GCC	TCT	1	G	T
TCGA-04-1651	VPS13B_EN!	ENST00000357162	p.A1013S	c.3037G>T	Unverified	CAG	GCC	TCT	1	G	T
TCGA-04-1649	VPS33B	ENST00000333371	p.A142S	c.424G>T	Unverified	TGG	GCC	TTC	1	G	T
TCGA-25-1313	VSNL1	ENST00000295156	p.E14D	c.42G>T	Verified	ATG	GAG	GAC	3	G	T
TCGA-13-1501	VWA2	ENST00000298715	p.S223I	c.668G>T	Verified	CTC	AGC	AGC	2	G	T
TCGA-13-1501	VWA2	ENST00000298715	p.S223I	c.668G>T	Verified	CTC	AGC	AGC	2	G	T
TCGA-13-0890	WAC	ENST00000354911	p.?	c.1437+1G>T	Verified	CCA	AAG	GTT	3	G	T
TCGA-13-0916	WDFY3	ENST00000295888	p.A2831S	c.8491G>T	Unverified	GAG	GCC	TGG	1	G	T
TCGA-09-2050	WDPCP	ENST00000272321	p.G355C	c.1063G>T	Verified	CTG	GGC	TGT	1	G	T
TCGA-13-1489	WDR45L	ENST00000392325	p.P296P	c.888G>T	Verified	TCT	CCG	TGC	3	G	T
TCGA-23-1809	WDR49	ENST00000308378	p.E450D	c.1350G>T	Unverified	ATA	GAG	GAG	3	G	T
TCGA-04-1338	WFDC1	ENST00000219454	p.G189V	c.566G>T	Unverified	GAT	GGG	CGA	2	G	T
TCGA-04-1342	WHSC2	ENST00000333877	p.C471F	c.1412G>T	Unverified	CCG	TGC	CAG	2	G	T
TCGA-29-1766	WIZ	ENST00000263381	p.G39G	c.117G>T	Unverified	GAG	GGG	GAA	3	G	T
TCGA-29-1766	WIZ_ENST0(	ENST00000389282	p.G39G	c.117G>T	Unverified	GAG	GGG	GAA	3	G	T

TCGA-25-2042	WNT2B	ENST00000369684	p.A272S	c.814G>T	Verified	GGG	GCT	GTG	1	G	T
TCGA-04-1331	WNT7A	ENST00000285018	p.E171D	c.513G>T	Verified	CGG	GAG	ATC	3	G	T
TCGA-61-1904	WRAP73	ENST00000270708	p.V274L	c.820G>T	Unverified	GTG	GTG	TAT	1	G	T
TCGA-61-1733	WSCD1	ENST00000317744	p.C178F	c.533G>T	Unverified	GCG	TGT	GCT	2	G	T
TCGA-61-1733	WSCD1	ENST00000317744	p.C178F	c.533G>T	Unverified	GCG	TGT	GCT	2	G	T
TCGA-23-2078	WWC3	NM_015691.2	p.S355I	c.1064G>T	Verified	aaa	agc	ctg	2	G	T
TCGA-13-0762	XIRP1	ENST00000340369	p.D334Y	c.1000G>T	Verified	CCA	GAC	CTT	1	G	T
TCGA-13-0762	XIRP1	ENST00000340369	p.D334Y	c.1000G>T	Verified	CCA	GAC	CTT	1	G	T
TCGA-29-1770	XRRA1	ENST00000340360	p.K590N	c.1770G>T	Unverified	CAA	AAG	AAA	3	G	T
TCGA-13-0807	XYLB	ENST00000207870	p.G155V	c.464G>T	Verified	GGT	GGT	GCT	2	G	T
TCGA-25-2398	YBX1	ENST00000321358	p.G71V	c.212G>T	Verified	AAC	GGA	TAT	2	G	T
TCGA-24-2035	YSK4_ENSTC	ENST00000375845	p.G353V	c.1058G>T	Verified	CAT	GGT	AGT	2	G	T
TCGA-13-0913	YWHAE	ENST00000264335	p.E15*	c.43G>T	Verified	GCC	GAG	CAG	1	G	T
TCGA-25-1313	ZAN	ENST00000348028	p.R2122S	c.6366G>T	Verified	AGC	AGG	AGA	3	G	T
TCGA-04-1343	ZBTB20	ENST00000357258	p.A430S	c.1288G>T	Verified	ACA	GCT	GGC	1	G	T
TCGA-61-2012	ZBTB24	ENST00000230122	p.D417Y	c.1249G>T	Verified	ATG	GAT	GTG	1	G	T
TCGA-24-0975	ZBTB3	ENST00000394807	p.G27*	c.79G>T	Unverified	CGT	GGA	GCC	1	G	T
TCGA-25-2401	ZBTB46	ENST00000245663	p.E199*	c.595G>T	Verified	AAA	GAG	GAT	1	G	T
TCGA-29-1693	ZBTB46	ENST00000245663	p.V35V	c.105G>T	Unverified	TGC	GTG	GTC	3	G	T
TCGA-61-1906	ZC3H6	ENST00000409871	p.G216V	c.647G>T	Unverified	GTT	GGT	CGT	2	G	T
TCGA-61-1906	ZC3H6	ENST00000409871	p.G216V	c.647G>T	Unverified	GTT	GGT	CGT	2	G	T
TCGA-23-1116	ZCWPW2	ENST00000383768	p.S46I	c.137G>T	Verified	TCA	AGT	GAG	2	G	T
TCGA-04-1530	ZDHHC23	ENST00000330212	p.G201V	c.602G>T	Verified	AGC	GGT	GAC	2	G	T
TCGA-24-1423	ZEB2	ENST00000303660	p.G1120G	c.3360G>T	Verified	cag	ggg	tac	3	G	T
TCGA-13-1509	ZFAND3	ENST00000287218	p.R156R	c.468G>T	Verified	CGT	CGG	TGC	3	G	T
TCGA-09-1674	ZFHX4	ENST00000521891	p.A572S	c.1714G>T	Unverified	GCT	GCT	CAT	1	G	T
TCGA-24-2290	ZFHX4	ENST00000521891	p.G612V	c.1835G>T	Verified	AGT	GGC	ATC	2	G	T
TCGA-24-2290	ZFHX4	ENST00000521891	p.G612V	c.1835G>T	Verified	AGT	GGC	ATC	2	G	T
TCGA-20-1687	ZFHX4	ENST00000521891	p.M279I	c.837G>T	Unverified	TTA	ATG	TGT	3	G	T
TCGA-25-2391	ZFP106	ENST00000263805	p.E115D	c.345G>T	Unverified	AGA	GAG	AGT	3	G	T
TCGA-24-1563	ZFYVE26	ENST00000347230	p.K2236N	c.6708G>T	Verified	GGA	AAG	TAC	3	G	T
TCGA-13-0760	ZHX1	ENST00000395571	p.E326*	c.976G>T	Verified	GAG	GAA	CAG	1	G	T
TCGA-13-1489	ZNF101	ENST00000318110	p.S379I	c.1136G>T	Verified	TGC	AGT	TCC	2	G	T
TCGA-13-0893	ZNF12	ENST00000405858	p.?	c.238+1G>T	Unverified	CCA	GAT	GAA	1	G	T

TCGA-13-0923	ZNF222	ENST00000187879	p.Q102H	c.306G>T	Verified	TCC	CAG	ATT	3	G	T
TCGA-13-1507	ZNF224	ENST00000336976	p.G452V	c.1355G>T	Verified	ACA	GGA	GAG	2	G	T
TCGA-25-1326	ZNF232	ENST00000250076	p.E221*	c.661G>T	Verified	ACA	GAA	GAT	1	G	T
TCGA-29-1777	ZNF234_ENS	ENST00000454662	p.C197F	c.590G>T	Unverified	TTA	TGT	CAA	2	G	T
TCGA-20-1687	ZNF256	ENST00000282308	p.G59V	c.176G>T	Unverified	GCA	GGG	GAT	2	G	T
TCGA-20-1687	ZNF256_ENS	ENST00000282308	p.G59V	c.176G>T	Unverified	GCA	GGG	GAT	2	G	T
TCGA-04-1530	ZNF292	ENST00000339907	p.L1422F	c.4266G>T	Verified	AGC	TTG	CCT	3	G	T
TCGA-24-1435	ZNF292	ENST00000339907	p.M2561I	c.7683G>T	Verified	GAT	ATG	TCT	3	G	T
TCGA-23-1124	ZNF326	ENST00000340281	p.D350Y	c.1048G>T	Verified	TTT	GAT	AAA	1	G	T
TCGA-29-1784	ZNF343	ENST00000278772	p.S503I	c.1508G>T	Unverified	TTT	AGC	CAG	2	G	T
TCGA-04-1651	ZNF345	ENST00000420450	p.A378S	c.1132G>T	Unverified	AAG	GCC	TTT	1	G	T
TCGA-04-1651	ZNF345	ENST00000420450	p.A378S	c.1132G>T	Unverified	AAG	GCC	TTT	1	G	T
TCGA-13-0923	ZNF385D	ENST00000281523	p.S352I	c.1055G>T	Verified	GTG	AGT	TCC	2	G	T
TCGA-61-2094	ZNF423_ENS	ENST00000262383	p.D207Y	c.619G>T	Unverified	AGC	GAC	CAC	1	G	T
TCGA-23-2078	ZNF491	ENST00000323169	p.G239V	c.716G>T	Verified	ACA	GGA	GAA	2	G	T
TCGA-09-2051	ZNF549	ENST00000240719	p.G12V	c.35G>T	Unverified	CAG	GGC	CAT	2	G	T
TCGA-61-1740	ZNF549	ENST00000240719	p.G410G	c.1230G>T	Unverified	TGT	GGG	AAG	3	G	T
TCGA-61-1740	ZNF549_ENS	ENST00000376233	p.G423G	c.1269G>T	Unverified	TGT	GGG	AAG	3	G	T
TCGA-61-1998	ZNF551	ENST00000282296	p.G633W	c.1897G>T	Verified	TGT	GGG	AAA	1	G	T
TCGA-24-2035	ZNF560	ENST00000301480	p.G150C	c.448G>T	Verified	GTA	GGT	TAC	1	G	T
TCGA-24-2290	ZNF582	ENST00000301310	p.R387I	c.1160G>T	Verified	CAG	AGA	ATT	2	G	T
TCGA-24-2290	ZNF582	ENST00000301310	p.R387I	c.1160G>T	Verified	CAG	AGA	ATT	2	G	T
TCGA-23-1022	ZNF583	ENST00000291598	p.V47F	c.139G>T	Verified	GGA	GTT	TCT	1	G	T
TCGA-61-2012	ZNF589	ENST00000354698	p.G340V	c.1019G>T	Verified	CGA	GGC	TTT	2	G	T
TCGA-61-1998	ZNF628_ENS	ENST00000391718	p.R86L	c.257G>T	Unverified	GAG	CGG	CCC	2	G	T
TCGA-29-1770	ZNF662	ENST00000440367	p.G145V	c.434G>T	Unverified	GGG	GGA	CGT	2	G	T
TCGA-29-1770	ZNF662_ENS	ENST00000328199_v68	p.G171V	c.512G>T	Unverified	GGG	GGA	CGT	2	G	T
TCGA-25-2392	ZNF671	ENST00000317398	p.A159S	c.475G>T	Verified	ATG	GCA	GAG	1	G	T
TCGA-24-1844	ZNF679	ENST00000421025	p.D20Y	c.58G>T	Unverified	AGA	GAT	GTA	1	G	T
TCGA-20-1683	ZNF699	ENST00000308650	p.G229V	c.686G>T	Unverified	TGT	GGG	AAG	2	G	T
TCGA-25-1326	ZNF710	ENST00000268154	p.K583N	c.1749G>T	Unverified	AGC	AAG	TTT	3	G	T
TCGA-04-1649	ZNF727	ENST00000550760	p.A326S	c.976G>T	Unverified	TCG	GCC	CTT	1	G	T
TCGA-09-0369	ZNF729	ENST00000357491	p.G1100W	c.3298G>T	Verified	ACT	GGG	GAG	1	G	T
TCGA-09-0369	ZNF729	ENST00000357491	p.G1100W	c.3298G>T	Verified	ACT	GGG	GAG	1	G	T

TCGA-23-1122	ZNF765_ENS	ENST00000396408	p.R477R	c.1431G>T	Verified	AGC	CGG	ACG	3	G	T
TCGA-23-1029	ZNF772	ENST00000343280	p.C439F	c.1316G>T	Unverified	GAA	TGT	GGA	2	G	T
TCGA-23-1110	ZNF777	ENST00000247930	p.C602F	c.1805G>T	Unverified	GGC	TGC	GTC	2	G	T
TCGA-24-1463	ZNF862	ENST00000223210	p.G246G	c.738G>T	Unverified	CCA	GGG	CCT	3	G	T
TCGA-24-0979	ZNF99	ENST00000397104	p.A398S	c.1192G>T	Verified	AAA	GCT	TTT	1	G	T
TCGA-29-1761	ZP2	ENST00000219593	p.Q412H	c.1236G>T	Unverified	TCT	CAG	GGG	3	G	T
TCGA-24-1845	ZSCAN20	ENST00000326544	p.E228*	c.682G>T	Unverified	CAG	GAA	GCC	1	G	T
TCGA-24-1847	ZSCAN22	ENST00000329665	p.W233L	c.698G>T	Unverified	GCG	TGG	CCA	2	G	T
TCGA-29-1775	A4GNT	ENST00000236709	p.V53E	c.158T>A	Unverified	ATT	GTG	TTT	2	T	A
TCGA-61-1725	ABCA12	ENST00000272895	p.P961P	c.2883T>A	Unverified	CTT	CCT	TCT	3	T	A
TCGA-13-0762	ABCD2	ENST00000308666	p.V593D	c.1778T>A	Verified	ATA	GTT	CAA	2	T	A
TCGA-13-0762	ABCD2	ENST00000308666	p.V593D	c.1778T>A	Verified	ATA	GTT	CAA	2	T	A
TCGA-29-1781	ABHD8	ENST00000247706	p.A231A	c.693T>A	Unverified	CTG	GCT	GAG	3	T	A
TCGA-61-1740	ABP1	ENST00000360937	p.S560T	c.1678T>A	Unverified	TAC	TCC	TGG	1	T	A
TCGA-23-1124	AC093393.1	ENST00000322472	p.Y236N	c.706T>A	Verified	GTG	TAC	TCT	1	T	A
TCGA-29-1784	ACADM	ENST00000370841	p.D68E	c.204T>A	Unverified	TAT	GAT	AAA	3	T	A
TCGA-24-1417	ACCN5	ENST00000537611	p.H140Q	c.420T>A	Verified	CTC	CAT	CTT	3	T	A
TCGA-13-0913	ACRC	ENST00000373695	p.S532T	c.1594T>A	Verified	AGA	TCC	GTC	1	T	A
TCGA-24-1417	ACTG1	ENST00000331925	p.S233T	c.697T>A	Verified	TCC	TCC	TCT	1	T	A
TCGA-04-1338	ACTR8	ENST00000335754	p.P197P	c.591T>A	Verified	GGC	CCT	GGG	3	T	A
TCGA-24-1849	ADAM18	ENST00000265707	p.S352R	c.1056T>A	Unverified	GCC	AGT	GGT	3	T	A
TCGA-61-1910	ADCK2	ENST00000072869	p.L101H	c.302T>A	Unverified	tgg	ctt	cgc	2	T	A
TCGA-04-1361	ADORA3	ENST00000241356	p.F80Y	c.239T>A	Verified	CAC	TTC	TAC	2	T	A
TCGA-04-1361	ADORA3	ENST00000241356	p.F80Y	c.239T>A	Verified	CAC	TTC	TAC	2	T	A
TCGA-24-1469	ADSSL1	ENST00000332972	p.F432I	c.1294T>A	Verified	TAT	TTC	CCA	1	T	A
TCGA-25-2398	AGAP8	ENST00000425119	p.I650N	c.1949T>A	Unverified	TGC	ATC	AAC	2	T	A
TCGA-24-1422	AGPAT3	ENST00000398058	p.V120D	c.359T>A	Unverified	AAG	GTC	CTC	2	T	A
TCGA-13-0905	AGTPBP1	ENST00000376083	p.L520L	c.1560T>A	Verified	AGT	CTT	CCT	3	T	A
TCGA-13-0887	AHCTF1	ENST00000391829	p.L1411H	c.4232T>A	Verified	TCT	CTT	TGT	2	T	A
TCGA-13-1481	AHCTF1	ENST00000391829	p.S1647R	c.4941T>A	Verified	ACT	AGT	GAC	3	T	A
TCGA-13-0890	AKAP1	ENST00000337714	p.V815D	c.2444T>A	Verified	ttt	gtc	acc	2	T	A
TCGA-29-1761	ANK1	ENST00000347528	p.F16Y	c.47T>A	Unverified	agc	ttt	ctg	2	T	A
TCGA-29-1761	ANK1_ENST(	ENST00000265709	p.F49Y	c.146T>A	Unverified	AGC	TTT	CTG	2	T	A
TCGA-25-2392	ANK3	ENST00000280772	p.C3752S	c.11254T>A	Verified	AGT	TGT	CAG	1	T	A

TCGA-29-1711	ANK3	ENST00000280772	p.P2191P	c.6573T>A	Unverified	GAG	CCT	GTG	3	T	A
TCGA-29-1711	ANK3	ENST00000280772	p.P2191P	c.6573T>A	Unverified	GAG	CCT	GTG	3	T	A
TCGA-13-0904	ANKRD30A	ENST00000361713	p.C285S	c.853T>A	Verified	CAA	TGT	TTG	1	T	A
TCGA-23-1032	ANKRD30A	ENST00000361713	p.I327N	c.980T>A	Verified	AAG	ATC	GCA	2	T	A
TCGA-13-0923	ANO5	ENST00000324559	p.L695L	c.2085T>A	Verified	CCT	CTT	CTT	3	T	A
TCGA-04-1342	ANO8	ENST00000159087	p.V447D	c.1340T>A	Unverified	TTT	GTC	AAC	2	T	A
TCGA-20-1686	ANXA5	ENST00000296511	p.S305T	c.913T>A	Unverified	ACA	TCT	GGG	1	T	A
TCGA-24-2024	APBB3	ENST00000354402	p.S248R	c.744T>A	Verified	GCC	AGT	GCC	3	T	A
TCGA-24-2289	APOB	ENST00000233242	p.F2957I	c.8869T>A	Unverified	TCC	TTT	GGA	1	T	A
TCGA-24-1435	APOB	ENST00000233242	p.L1527H	c.4580T>A	Verified	TAC	CTC	CAA	2	T	A
TCGA-24-0979	APOB	ENST00000233242	p.D4049E	c.12147T>A	Verified	TCT	GAT	GAG	3	T	A
TCGA-29-1770	AQP10	ENST00000324978	p.A275A	c.825T>A	Unverified	CCA	GCT	CAG	3	T	A
TCGA-13-0760	AR	ENST00000374690	p.L729I	c.2185T>A	Verified	aac	tta	cac	1	T	A
TCGA-13-0885	ARHGAP20	ENST00000260283	p.F551L	c.1653T>A	Verified	ata	ttt	gga	3	T	A
TCGA-13-1512	ARHGEF1	ENST00000337665	p.S3T	c.7T>A	Verified	gct	tct	ctt	1	T	A
TCGA-13-1501	ARHGEF11	ENST00000368194	p.G1162G	c.3486T>A	Verified	cat	ggt	gaa	3	T	A
TCGA-13-1501	ARHGEF11	ENST00000368194	p.G1162G	c.3486T>A	Verified	cat	ggt	gaa	3	T	A
TCGA-24-1417	ARMC2	ENST00000392644	p.D198E	c.594T>A	Unverified	GAT	GAT	GGA	3	T	A
TCGA-04-1356	ARMCX3	ENST00000341189	p.L266H	c.797T>A	Unverified	aaa	ctc	ctt	2	T	A
TCGA-29-1696	ARR3	ENST00000307959	p.H355Q	c.1065T>A	Unverified	TCT	CAT	GAG	3	T	A
TCGA-29-1696	ARR3	ENST00000307959	p.H355Q	c.1065T>A	Unverified	TCT	CAT	GAG	3	T	A
TCGA-09-1665	ATAD2	ENST00000287394	p.S1275T	c.3823T>A	Verified	GCT	TCT	AGC	1	T	A
TCGA-13-1507	ATF1	ENST00000262053	p.L71I	c.211T>A	Verified	GAC	TTA	TCT	1	T	A
TCGA-24-2290	ATG9B	ENST00000397266	p.L317Q	c.950T>A	Verified	GCC	CTG	CAC	2	T	A
TCGA-24-2290	ATG9B	ENST00000397266	p.L317Q	c.950T>A	Verified	GCC	CTG	CAC	2	T	A
TCGA-23-1032	ATRN	ENST00000262919	p.I1161I	c.3483T>A	Verified	CTT	ATT	GAC	3	T	A
TCGA-13-1512	BAG4	ENST00000287322	p.L411L	c.1233T>A	Verified	tgg	ctt	ctg	3	T	A
TCGA-13-0755	BAZ2B	ENST00000392783	p.S1229R	c.3687T>A	Verified	GTC	AGT	GAA	3	T	A
TCGA-23-1123	BCLAF1	ENST00000531224	p.D451E	c.1353T>A	Verified	AGT	GAT	GGA	3	T	A
TCGA-23-1123	BCLAF1	ENST00000531224	p.D451E	c.1353T>A	Verified	AGT	GAT	GGA	3	T	A
TCGA-29-1763	BICC1	ENST00000373886	p.V239E	c.716T>A	Unverified	TCA	GTA	TCA	2	T	A
TCGA-24-2289	BTRC	ENST00000370187	p.F514Y	c.1541T>A	Unverified	cga	ttt	gat	2	T	A
TCGA-61-1914	C10orf71_EI	ENST00000374144	p.S732T	c.2194T>A	Unverified	AGC	TCT	TCA	1	T	A
TCGA-61-1740	C14orf135	ENST00000404681	p.P758P	c.2274T>A	Unverified	AGT	CCT	GGT	3	T	A

TCGA-61-1740	C14orf135_I	ENST00000406854	p.P992P	c.2976T>A	Unverified	AGT	CCT	GGT	3	T	A
TCGA-04-1542	C18orf1	ENST00000361205	p.C15S	c.43T>A	Verified	gag	tgc	aaa	1	T	A
TCGA-24-1845	C20orf103	ENST00000246070	p.V248D	c.743T>A	Unverified	CTC	GTC	ATC	2	T	A
TCGA-04-1530	C20orf185	ENST00000375494	p.V358D	c.1073T>A	Unverified	TTG	GTC	TCC	2	T	A
TCGA-29-1775	C2orf47	ENST00000392290	p.F11I	c.31T>A	Unverified	CAG	TTC	CTG	1	T	A
TCGA-29-1770	C6orf138	ENST00000339488	p.F226I	c.676T>A	Unverified	GAC	TTT	CAT	1	T	A
TCGA-13-0905	C6orf47	ENST00000375911	p.L155*	c.464T>A	Verified	CTG	TTG	GGC	2	T	A
TCGA-29-1768	C9orf11	ENST00000380032	p.A93A	c.279T>A	Unverified	TTT	GCT	CTA	3	T	A
TCGA-61-1900	CACNA2D3	ENST00000474759	p.A455A	c.1365T>A	Unverified	GAA	GCT	TAC	3	T	A
TCGA-24-1850	CACNG4	ENST00000262138	p.F306I	c.916T>A	Unverified	GAC	TTT	TTC	1	T	A
TCGA-24-1435	CADM1	ENST00000452722	p.I247N	c.740T>A	Verified	CAC	ATT	CAG	2	T	A
TCGA-13-1510	CADPS	ENST00000283269	p.V721D	c.2162T>A	Unverified	GGA	GTC	CGG	2	T	A
TCGA-13-0919	CaMK1b	SU_CaMK1b	p.I310N	c.929T>A	Verified	cac	atc	cgg	2	T	A
TCGA-13-0885	CAMTA2	ENST00000348066	p.P69P	c.207T>A	Verified	AGG	CCT	CAG	3	T	A
TCGA-13-1497	CAPN6	ENST00000324068	p.T205T	c.615T>A	Verified	tac	act	gag	3	T	A
TCGA-13-0714	CASP14	ENST00000427043	p.F195Y	c.584T>A	Verified	gtg	ttc	acg	2	T	A
TCGA-20-1687	CATSPERB	ENST00000256343	p.D27E	c.81T>A	Unverified	AAA	GAT	GAT	3	T	A
TCGA-13-0924	CCDC148	ENST00000283233	p.L301M	c.901T>A	Verified	GAT	TTG	GTT	1	T	A
TCGA-30-1855	CCDC149	ENST00000324309	p.S224T	c.670T>A	Unverified	CTA	TCT	GAG	1	T	A
TCGA-30-1855	CCDC149_EI	ENST00000504487	p.S279T	c.835T>A	Unverified	CTA	TCT	GAG	1	T	A
TCGA-30-1856	CCDC158	ENST00000388914	p.L837I	c.2509T>A	Unverified	CGC	TTA	AAA	1	T	A
TCGA-09-0369	CCNB1IP1	ENST00000358932	p.N168K	c.504T>A	Verified	CGC	AAT	CGT	3	T	A
TCGA-09-0369	CCNB1IP1	ENST00000358932	p.N168K	c.504T>A	Verified	CGC	AAT	CGT	3	T	A
TCGA-13-0885	CCNB3	ENST00000376042	p.V680V	c.2040T>A	Verified	CAT	GTT	AAG	3	T	A
TCGA-13-0885	CCNB3_ENS	ENST00000376042	p.V680V	c.2040T>A	Verified	CAT	GTT	AAG	3	T	A
TCGA-29-1693	CCT8L2	ENST00000359963	p.V205E	c.614T>A	Unverified	GGG	GTG	TGC	2	T	A
TCGA-29-1763	CD1D	ENST00000368171	p.F36Y	c.107T>A	Unverified	TCC	TTC	GCC	2	T	A
TCGA-13-1510	CD80	ENST00000264246	p.V225E	c.674T>A	Verified	AGA	GTG	AAT	2	T	A
TCGA-13-0792	CDH17	ENST00000027335	p.L13L	c.39T>A	Unverified	CTT	CTT	ATG	3	T	A
TCGA-13-0920	CDKAL1	ENST00000378610	p.C221S	c.661T>A	Verified	TAC	TGC	AAA	1	T	A
TCGA-13-1505	CEACAM5	ENST00000221992	p.L549Q	c.1646T>A	Unverified	CAG	CTG	TCC	2	T	A
TCGA-23-1118	CEBPE	ENST00000206513	p.F20I	c.58T>A	Verified	GAG	TTC	TCA	1	T	A
TCGA-61-1738	CEP164	ENST00000278935	p.H543Q	c.1629T>A	Unverified	CAG	CAT	TCC	3	T	A
TCGA-61-1738	CEP164	ENST00000278935	p.H543Q	c.1629T>A	Unverified	CAG	CAT	TCC	3	T	A

TCGA-23-1110	CHM	ENST00000357749	p.F52I	c.154T>A	Verified	agc	ttt	tca	1	T	A
TCGA-13-0893	CHN1	ENST00000409900	p.Y70N	c.208T>A	Verified	agc	tac	ctc	1	T	A
TCGA-24-1846	CINP	ENST00000216756	p.S73T	c.217T>A	Unverified	GCC	TCG	AAG	1	T	A
TCGA-13-0900	CLCA4	ENST00000370563	p.S421T	c.1261T>A	Verified	AGT	TCT	TGT	1	T	A
TCGA-13-1408	CLCN4	ENST00000380833	p.I33N	c.98T>A	Verified	acc	atc	gac	2	T	A
TCGA-23-1021	CLCN5	ENST00000376108	p.V262E	c.785T>A	Verified	gga	gta	tta	2	T	A
TCGA-13-0793	CLP1	ENST00000533682	p.V65V	c.195T>A	Verified	GCT	GTT	TTC	3	T	A
TCGA-24-1843	CNTNAP5	ENST00000431078	p.I623N	c.1868T>A	Unverified	AAT	ATC	ACT	2	T	A
TCGA-30-1718	COG5	ENST00000297135	p.P713P	c.2139T>A	Unverified	AGA	CCT	CTT	3	T	A
TCGA-13-0760	COL11A1	ENST00000358392	p.S1674T	c.5020T>A	Verified	TCA	TCA	TGG	1	T	A
TCGA-24-1844	COL4A1	ENST00000375820	p.C1616*	c.4848T>A	Unverified	GAG	TGT	CAC	3	T	A
TCGA-24-1844	COL4A1_EN	ENST00000375815	p.C1259*	c.3777T>A	Unverified	GAG	TGT	CAC	3	T	A
TCGA-09-2049	COL6A6	ENST00000358511	p.T1235T	c.3705T>A	Verified	GAG	ACT	CAG	3	T	A
TCGA-23-1022	COL9A1	ENST00000357250	p.F10I	c.28T>A	Verified	GTT	TTC	TTC	1	T	A
TCGA-30-1855	CPA6	ENST00000297770	p.W200R	c.598T>A	Unverified	GAA	TGG	ATT	1	T	A
TCGA-13-0791	CPN2	ENST00000323830	p.W468R	c.1402T>A	Verified	AGC	TGG	GAT	1	T	A
TCGA-13-0887	CRELD1	ENST00000326434	p.I342N	c.1025T>A	Verified	GGC	ATC	TGT	2	T	A
TCGA-24-2289	CRELD2	ENST00000328268	p.A336A	c.1008T>A	Unverified	GAG	GCT	GAA	3	T	A
TCGA-24-2280	CRTAM	ENST00000227348	p.C177*	c.531T>A	Unverified	AAA	TGT	AAT	3	T	A
TCGA-13-0923	CSMD2	ENST00000241312	p.F1314I	c.3940T>A	Verified	CAC	TTC	CTG	1	T	A
TCGA-13-0760	CSMD3	ENST00000297405	p.L2512H	c.7535T>A	Verified	gtt	ctt	cag	2	T	A
TCGA-61-1899	CSMD3	ENST00000297405	p.A3230A	c.9690T>A	Unverified	aga	gct	gtt	3	T	A
TCGA-61-1899	CSMD3_ENS	ENST00000343508	p.A3190A	c.9570T>A	Unverified	AGA	GCT	GTT	3	T	A
TCGA-04-1331	CSRNP1	ENST00000273153	p.P315P	c.945T>A	Verified	GCC	CCT	GCC	3	T	A
TCGA-24-1847	CTNND2	ENST00000304623	p.C127S	c.379T>A	Unverified	tcc	tgt	att	1	T	A
TCGA-24-1847	CYP11A1	ENST00000268053	p.L490H	c.1469T>A	Unverified	CAC	CTC	AGC	2	T	A
TCGA-61-1907	CYP11B1	ENST00000292427	p.S388T	c.1162T>A	Unverified	AGC	TCA	GAC	1	T	A
TCGA-13-0893	CYP11B1	ENST00000292427	p.L53Q	c.158T>A	Unverified	CTG	CTG	CAG	2	T	A
TCGA-09-2044	CYP2C19	ENST00000371321	p.L366Q	c.1097T>A	Verified	AGC	CTG	CCC	2	T	A
TCGA-13-0760	CYP2C19	ENST00000371321	p.L445Q	c.1334T>A	Verified	GAG	CTG	TTT	2	T	A
TCGA-13-1496	CYP39A1	ENST00000275016	p.D313E	c.939T>A	Verified	AAA	GAT	AAG	3	T	A
TCGA-25-2400	DACH2	ENST00000373125	p.A296A	c.888T>A	Verified	ggt	gct	cca	3	T	A
TCGA-25-2400	DACH2_ENS	ENST00000373131	p.A283A	c.849T>A	Verified	GGT	GCT	CCA	3	T	A
TCGA-23-1122	DCAF4	ENST00000358377	p.S291T	c.871T>A	Verified	TGG	TCC	CTG	1	T	A

TCGA-24-2267	DCHS1	ENST00000299441	p.Y538N	c.1612T>A	Verified	GAC	TAT	GAG	1	T	A
TCGA-24-1616	DHRS4L2	ENST00000335125	p.H89Q	c.267T>A	Verified	TGC	CAT	GTG	3	T	A
TCGA-29-1763	DIRAS3	ENST00000370981	p.L20Q	c.59T>A	Unverified	ctt	ctg	ccc	2	T	A
TCGA-61-2012	DLX2	ENST00000234198	p.M87K	c.260T>A	Unverified	CAC	ATG	GGT	2	T	A
TCGA-25-1313	DMRT3	ENST00000190165	p.V254E	c.761T>A	Verified	GAA	GTG	TTA	2	T	A
TCGA-13-0760	DNAH10	ENST00000339192	p.P784P	c.2352T>A	Verified	AAC	CCT	CAG	3	T	A
TCGA-30-1891	DNAH3	ENST00000261383	p.L1949H	c.5846T>A	Unverified	TTT	CTC	TGG	2	T	A
TCGA-30-1891	DNAH3_ENS	ENST00000261383	p.L1949H	c.5846T>A	Unverified	TTT	CTC	TGG	2	T	A
TCGA-20-1687	DNAH7	ENST00000312428	p.S996S	c.2988T>A	Unverified	AGC	TCT	CCA	3	T	A
TCGA-29-1703	DOCK8	ENST00000432829	p.L1978H	c.5933T>A	Unverified	GAA	CTC	AAA	2	T	A
TCGA-29-1703	DOCK8_ENS	ENST00000453981	p.L2046H	c.6137T>A	Unverified	GAA	CTC	AAA	2	T	A
TCGA-61-2008	DOPEY1	ENST00000349129	p.H2141Q	c.6423T>A	Verified	ACA	CAT	GAT	3	T	A
TCGA-24-1103	DPP10	ENST00000310323	p.V172D	c.515T>A	Verified	TCC	GTC	TTG	2	T	A
TCGA-04-1331	DSEL	ENST00000310045	p.F1143Y	c.3428T>A	Verified	CAT	TTT	CCT	2	T	A
TCGA-13-0920	DSG1	ENST00000257192	p.I290N	c.869T>A	Verified	GAG	ATT	AGA	2	T	A
TCGA-13-0893	DST	ENST00000370765	p.I549I	c.1647T>A	Verified	ATA	ATT	CAA	3	T	A
TCGA-13-0893	DST_ENST0(	ENST00000370769	p.I875I	c.2625T>A	Verified	ATA	ATT	CAA	3	T	A
TCGA-13-0923	DUOX1	ENST00000389037	p.Y1206*	c.3618T>A	Verified	ATG	TAT	GTC	3	T	A
TCGA-30-1857	DYNC2LI1	ENST00000260605	p.I184N	c.551T>A	Unverified	ATA	ATT	GGA	2	T	A
TCGA-23-1021	DZIP1	ENST00000361396	p.L315*	c.944T>A	Verified	GAA	TTA	ACT	2	T	A
TCGA-29-1764	E2F2	ENST00000361729	p.L374Q	c.1121T>A	Unverified	agc	ctg	ctg	2	T	A
TCGA-09-0369	E2F5	ENST00000416274	p.L314H	c.941T>A	Verified	CCT	CTC	TTA	2	T	A
TCGA-09-0369	E2F5	ENST00000416274	p.L314H	c.941T>A	Verified	CCT	CTC	TTA	2	T	A
TCGA-23-2078	E2F7	ENST00000322886	p.A592A	c.1776T>A	Unverified	TCA	GCT	CAG	3	T	A
TCGA-13-1488	EFHC1	ENST00000371068	p.D230E	c.690T>A	Verified	TTT	GAT	CAA	3	T	A
TCGA-13-0923	EFTUD2	ENST00000426333	p.M603K	c.1808T>A	Verified	AAG	ATG	CTT	2	T	A
TCGA-29-1770	EGFLAM	ENST00000322350	p.D436E	c.1308T>A	Unverified	GGG	GAT	TTC	3	T	A
TCGA-29-1770	EGFLAM_EN	ENST00000354891	p.D436E	c.1308T>A	Unverified	GGG	GAT	TTC	3	T	A
TCGA-23-2077	EIF3E	ENST00000220849	p.L205H	c.614T>A	Verified	cca	ctt	cag	2	T	A
TCGA-13-0883	ELMOD3	ENST00000315658	p.I178N	c.533T>A	Verified	ACC	ATC	TAT	2	T	A
TCGA-23-1117	EML4	ENST00000318522	p.V412V	c.1236T>A	Verified	GTT	GTT	TTG	3	T	A
TCGA-23-1117	EML4	ENST00000318522	p.V412V	c.1236T>A	Verified	GTT	GTT	TTG	3	T	A
TCGA-24-1474	ENSG00001	ENST00000315797	p.M217K	c.650T>A	Unverified	ACC	ATG	TGC	2	T	A
TCGA-04-1652	ENSG00001	ENST00000340731	p.L268H	c.803T>A	Unverified	CAC	CTC	TGG	2	T	A

TCGA-04-1652	ENSG00001	ENST00000340731	p.L268H	c.803T>A	Unverified	CAC	CTC	TGG	2	T	A
TCGA-13-0903	ENSG00002	ENST00000260257	p.F377L	c.1131T>A	Verified	GTC	TTT	CAG	3	T	A
TCGA-61-1895	EPB41	ENST00000349460	p.N497K	c.1491T>A	Unverified	ATC	AAT	GGG	3	T	A
TCGA-61-1895	EPB41_ENST	ENST00000343067	p.N720K	c.2160T>A	Unverified	ATC	AAT	GGG	3	T	A
TCGA-23-1110	EPB42	ENST00000300215	p.M364K	c.1091T>A	Verified	TGG	ATG	ACG	2	T	A
TCGA-04-1367	EPHA3	ENST00000336596	p.I564N	c.1691T>A	Verified	ttg	att	ggg	2	T	A
TCGA-25-2400	EPHA7	NM_004440	p.A517A	c.1551T>A	Verified	cgg	gct	ttt	3	T	A
TCGA-29-1699	EPHX1	ENST00000366837	p.V180D	c.539T>A	Unverified	GAA	GTC	ATC	2	T	A
TCGA-23-1114	EPS8	ENST00000281172	p.M367K	c.1100T>A	Unverified	AAT	ATG	GTG	2	T	A
TCGA-13-1497	ERBB3_ENST	ENST00000267101	p.D1194E	c.3582T>A	Verified	GAA	GAT	GAG	3	T	A
TCGA-20-1685	ESF1	ENST00000202816	p.S190T	c.568T>A	Unverified	GAC	TCA	GGC	1	T	A
TCGA-61-1910	ESYT1	ENST00000394048	p.L763M	c.2287T>A	Unverified	CAC	TTG	CGC	1	T	A
TCGA-61-2109	EXOC5	ENST00000413566	p.I364N	c.1091T>A	Unverified	ATG	ATC	CTA	2	T	A
TCGA-61-1907	EXPH5	ENST00000265843	p.L144Q	c.431T>A	Unverified	TCA	CTG	GGA	2	T	A
TCGA-25-1313	F8	ENST00000360256	p.W87R	c.259T>A	Verified	CCC	TGG	ATG	1	T	A
TCGA-25-1313	F8_ENST000	ENST00000360256	p.W87R	c.259T>A	Verified	CCC	TGG	ATG	1	T	A
TCGA-13-1496	FABP2	ENST00000274024	p.N104K	c.312T>A	Verified	CTG	AAT	ACT	3	T	A
TCGA-61-1910	FAM131B	ENST00000409346	p.L198Q	c.593T>A	Unverified	TGT	CTG	GGG	2	T	A
TCGA-61-1910	FAM131B_E	ENST00000443739	p.L226Q	c.677T>A	Unverified	TGT	CTG	GGG	2	T	A
TCGA-24-1847	FAM186B	ENST00000257894	p.V230D	c.689T>A	Unverified	GAG	GTC	AGG	2	T	A
TCGA-13-0793	FAM190A	ENST00000509176	p.D515E	c.1545T>A	Verified	CTG	GAT	GAA	3	T	A
TCGA-20-1687	FAM22F	ENST00000341207	p.P485P	c.1455T>A	Unverified	TCT	CCT	GTC	3	T	A
TCGA-20-1687	FAM22F_EN	ENST00000253262	p.P619P	c.1857T>A	Unverified	TCT	CCT	GTC	3	T	A
TCGA-09-1665	FAM71B	ENST00000302938	p.A257A	c.771T>A	Verified	GGG	GCT	GCT	3	T	A
TCGA-13-0760	FAM98A	ENST00000238823	p.L77H	c.230T>A	Verified	CAG	CTT	GAG	2	T	A
TCGA-20-0990	FANCG	ENST00000378643	p.V471E	c.1412T>A	Verified	aaa	gtg	gca	2	T	A
TCGA-30-1857	FASTKD3	ENST00000264669	p.G480G	c.1440T>A	Unverified	CAA	GGT	AAA	3	T	A
TCGA-09-2044	FAT4	ENST00000330166	p.G1453G	c.4359T>A	Verified	AAT	GGT	CAA	3	T	A
TCGA-09-2044	FAT4_ENST0	ENST00000394329	p.G1453G	c.4359T>A	Verified	AAT	GGT	CAA	3	T	A
TCGA-61-2102	FBN2	ENST00000262464	p.M692K	c.2075T>A	Verified	GGC	ATG	GAT	2	T	A
TCGA-23-1117	FBXO34	ENST00000313833	p.Y654N	c.1960T>A	Verified	ccc	tat	tgc	1	T	A
TCGA-23-1117	FBXO34	ENST00000313833	p.Y654N	c.1960T>A	Verified	ccc	tat	tgc	1	T	A
TCGA-13-1501	FBXO38	ENST00000340253	p.S444T	c.1330T>A	Verified	gac	tct	ttt	1	T	A
TCGA-13-1501	FBXO38	ENST00000340253	p.S444T	c.1330T>A	Verified	gac	tct	ttt	1	T	A

TCGA-13-1408	FBXO6	ENST00000376753	p.L183H	c.548T>A	Unverified	caa	ctc	aaa	2	T	A
TCGA-13-1509	FCRL3	ENST00000368184	p.M207K	c.620T>A	Verified	CCC	ATG	ACC	2	T	A
TCGA-23-1110	FGA	ENST00000302053	p.L699Q	c.2096T>A	Verified	AGC	CTG	AAT	2	T	A
TCGA-24-2280	FGA	ENST00000302053	p.V541D	c.1622T>A	Unverified	TTT	GTC	AGT	2	T	A
TCGA-04-1338	FGF12	ENST00000454309	p.G91G	c.273T>A	Verified	GAT	GGT	ACC	3	T	A
TCGA-29-1696	FGF14	ENST00000376131	p.G75G	c.225T>A	Unverified	AAG	GGT	ATA	3	T	A
TCGA-29-1696	FGF14	ENST00000376131	p.G75G	c.225T>A	Unverified	AAG	GGT	ATA	3	T	A
TCGA-29-1696	FGF14_ENST	ENST00000376143	p.G70G	c.210T>A	Unverified	AAG	GGT	ATA	3	T	A
TCGA-29-1696	FGF14_ENST	ENST00000376143	p.G70G	c.210T>A	Unverified	AAG	GGT	ATA	3	T	A
TCGA-13-0760	FIP1L1	ENST00000337488	p.W165R	c.493T>A	Verified	CCA	TGG	CGT	1	T	A
TCGA-13-0920	FLJ45831	ENST00000379640	p.C98*	c.294T>A	Verified	GGC	TGT	GAA	3	T	A
TCGA-13-0884	FLT1	ENST00000282397	p.N212K	c.636T>A	Verified	GTC	AAT	GGG	3	T	A
TCGA-23-1124	FOLR4	ENST00000328458	p.N122K	c.366T>A	Verified	GTG	AAT	GTG	3	T	A
TCGA-09-1665	FOXN4	NM_213596.1	p.F263Y	c.788T>A	Verified	gga	ttc	agc	2	T	A
TCGA-13-0893	FOXS1	ENST00000375978	p.P186P	c.558T>A	Verified	AGG	CCT	CGG	3	T	A
TCGA-29-1781	FPR3	ENST00000339223	p.Y175N	c.523T>A	Unverified	ACA	TAC	TGT	1	T	A
TCGA-20-1687	FRAS1	ENST00000264895	p.I2666I	c.7998T>A	Unverified	GTC	ATT	ATC	3	T	A
TCGA-20-1687	FRAS1_ENST	ENST00000264895	p.I2666I	c.7998T>A	Unverified	GTC	ATT	ATC	3	T	A
TCGA-25-1326	FRK	ENST00000368626	p.S145R	c.435T>A	Verified	gaa	agt	gaa	3	T	A
TCGA-04-1649	FRMPD3	XM_042978.4	p.P442P	c.1326T>A	Unverified	ctt	cct	acc	3	T	A
TCGA-04-1649	FRMPD3_EN	ENST00000276185	p.P393P	c.1179T>A	Unverified	CTT	CCT	ACC	3	T	A
TCGA-61-1998	FSHR	ENST00000406846	p.V556E	c.1667T>A	Verified	ACA	GTG	CGG	2	T	A
TCGA-29-1764	FSHR	ENST00000406846	p.S26S	c.78T>A	Unverified	TGC	TCT	AAC	3	T	A
TCGA-25-1313	GAB4	ENST00000400588	p.S468T	c.1402T>A	Verified	TCC	TCC	CAA	1	T	A
TCGA-13-1481	GABRE_ENST	ENST00000370328	p.P108P	c.324T>A	Verified	GGT	CCT	CTC	3	T	A
TCGA-24-2262	GAS2L1_ENS	ENST00000333679	p.L516Q	c.1547T>A	Unverified	CAG	CTG	TTC	2	T	A
TCGA-13-1505	GAS2L2	NM_139285.1	p.V235E	c.704T>A	Verified	cgt	gtg	ggt	2	T	A
TCGA-24-1845	GCM2	ENST00000379491	p.P233P	c.699T>A	Unverified	TGC	CCT	TCC	3	T	A
TCGA-23-1031	GDE1	ENST00000353258	p.T120T	c.360T>A	Verified	ACG	ACT	GAT	3	T	A
TCGA-04-1338	GJB6	ENST00000400065	p.I193N	c.578T>A	Verified	ACC	ATT	TTT	2	T	A
TCGA-13-1481	GLI1	ENST00000228682	p.N632K	c.1896T>A	Verified	ccc	aat	gca	3	T	A
TCGA-13-1507	GLYATL2	ENST00000287275	p.D126E	c.378T>A	Verified	GTA	GAT	TAC	3	T	A
TCGA-23-2077	GMPR	ENST00000259727	p.A234A	c.702T>A	Verified	GGA	GCT	GGA	3	T	A
TCGA-24-1104	GOLPH3L	ENST00000271732	p.D120E	c.360T>A	Verified	CTG	GAT	GAA	3	T	A

TCGA-24-1847	GPAM	ENST00000348367	p.Y705N	c.2113T>A	Unverified	TGC	TAC	CTG	1	T	A
TCGA-23-1110	GPER	ENST00000397088	p.F146Y	c.437T>A	Verified	GTC	TTC	TTC	2	T	A
TCGA-13-1488	GPHN	ENST00000478722	p.I377N	c.1130T>A	Verified	GAA	ATC	ATC	2	T	A
TCGA-13-0714	GPR101	ENST00000298110	p.F256I	c.766T>A	Verified	gag	ttc	cag	1	T	A
TCGA-24-1470	GPR128	ENST00000273352	p.V453D	c.1358T>A	Verified	ACA	GTT	ATA	2	T	A
TCGA-23-1022	GPR133	ENST00000261654	p.F36Y	c.107T>A	Verified	GGA	TTT	CAG	2	T	A
TCGA-23-1029	GPR149	ENST00000389740	p.D490E	c.1470T>A	Unverified	AAG	GAT	GCG	3	T	A
TCGA-61-1915	GPR37	ENST00000303921	p.F360I	c.1078T>A	Unverified	CGC	TTC	CGT	1	T	A
TCGA-23-1032	GPRC6A	ENST00000310357	p.I99N	c.296T>A	Verified	GAA	ATC	TAT	2	T	A
TCGA-29-1693	GRASP	ENST00000293662	p.D108E	c.324T>A	Unverified	GAG	GAT	AAC	3	T	A
TCGA-20-1687	GRIN3A	ENST00000361820	p.F463I	c.1387T>A	Unverified	AAC	TTT	TTC	1	T	A
TCGA-04-1356	GRLF1_ENST	ENST00000317082_v61	p.L671Q	c.2012T>A	Verified	TCG	CTA	TCC	2	T	A
TCGA-04-1347	GRM3	ENST00000361669	p.S496T	c.1486T>A	Verified	AAC	TCT	ATC	1	T	A
TCGA-13-0795	GRM8	ENST00000339582	p.L555L	c.1665T>A	Verified	GAA	CTT	TGC	3	T	A
TCGA-13-0923	GRPR	ENST00000380289	p.N243K	c.729T>A	Verified	TAC	AAT	CTT	3	T	A
TCGA-24-1469	GRXCR1	ENST00000399770	p.L2H	c.5T>A	Verified	ATG	CTT	AAA	2	T	A
TCGA-29-1691	hCG_38941	ENST00000328828	p.S139T	c.415T>A	Unverified	TAC	TCC	CCA	1	T	A
TCGA-13-1501	HCN4	ENST00000261917	p.L386I	c.1156T>A	Verified	CTC	TTA	CGC	1	T	A
TCGA-13-1501	HCN4	ENST00000261917	p.L386I	c.1156T>A	Verified	CTC	TTA	CGC	1	T	A
TCGA-29-2427	HEATR7B2	ENST00000399564	p.I783I	c.2349T>A	Verified	CTG	ATT	GGT	3	T	A
TCGA-13-0714	HECTD1	ENST00000399332	p.F2123Y	c.6368T>A	Verified	GAG	TTT	CGA	2	T	A
TCGA-23-1123	HEPACAM2	ENST00000341723	p.Y226*	c.678T>A	Verified	ATA	TAT	TAT	3	T	A
TCGA-23-1123	HEPACAM2	ENST00000341723	p.Y226*	c.678T>A	Verified	ATA	TAT	TAT	3	T	A
TCGA-24-1845	HEPHL1	ENST00000315765	p.F11I	c.31T>A	Unverified	ATC	TTT	CTC	1	T	A
TCGA-23-1029	HERC3	ENST00000402738	p.S232T	c.694T>A	Unverified	gaa	tct	cca	1	T	A
TCGA-04-1337	HINT2	ENST00000259667	p.V152E	c.455T>A	Unverified	CAT	GTA	CTT	2	T	A
TCGA-24-2262	HIVEP1	ENST00000379388	p.V1644D	c.4931T>A	Verified	AGT	GTT	CCT	2	T	A
TCGA-29-1691	HMMR	ENST00000358715	p.A563A	c.1689T>A	Unverified	AAA	GCT	GAA	3	T	A
TCGA-09-2049	HNMT	ENST00000280097	p.S201T	c.601T>A	Verified	ACA	TCA	GAT	1	T	A
TCGA-29-1703	HRNR	ENST00000368801	p.I88N	c.263T>A	Unverified	AAA	ATC	ATT	2	T	A
TCGA-13-0919	HSF5	ENST00000323777	p.S500T	c.1498T>A	Verified	TCT	TCA	GTA	1	T	A
TCGA-25-2400	HTR1F	ENST00000319595	p.L230H	c.689T>A	Verified	GTC	CTT	TTG	2	T	A
TCGA-13-0920	HTR2C	ENST00000371951	p.W55R	c.163T>A	Unverified	aac	tgg	cca	1	T	A
TCGA-24-2288	HTT	ENST00000355072	p.W2420R	c.7258T>A	Unverified	GGA	TGG	TCA	1	T	A

TCGA-13-0886	HUWE1	XM_497119.1	p.S1503T	c.4507T>A	Verified	gat	tct	gcc	1	T	A
TCGA-23-1118	HYDIN	ENST00000321489	p.I595N	c.1784T>A	Verified	TTG	ATC	CCC	2	T	A
TCGA-24-1604	IARS2	ENST00000302637	p.V709D	c.2126T>A	Verified	GAA	GTT	GCA	2	T	A
TCGA-13-1509	IFNB1	ENST00000380232	p.N111K	c.333T>A	Verified	GCT	AAT	GTC	3	T	A
TCGA-20-1685	IGHMBP2	ENST00000255078	p.L59Q	c.176T>A	Unverified	GGG	CTG	TAC	2	T	A
TCGA-09-0369	IL18RAP	ENST00000264260	p.L265H	c.794T>A	Verified	GAA	CTT	GGA	2	T	A
TCGA-09-0369	IL18RAP	ENST00000264260	p.L265H	c.794T>A	Verified	GAA	CTT	GGA	2	T	A
TCGA-30-1891	IL24	ENST00000294984	p.F170I	c.508T>A	Unverified	CGG	TTT	CTG	1	T	A
TCGA-24-1563	IL8	ENST00000307407	p.L32H	c.95T>A	Verified	GAA	CTT	AGA	2	T	A
TCGA-24-1422	ITIH1	ENST00000273283	p.V669E	c.2006T>A	Unverified	GGC	GTG	GAC	2	T	A
TCGA-24-1563	ITLN1	ENST00000326245	p.Y66N	c.196T>A	Verified	ATC	TAC	CAG	1	T	A
TCGA-23-2078	ITPR1	ENST00000456211	p.S421S	c.1263T>A	Verified	GTT	TCT	CCT	3	T	A
TCGA-23-1110	JMJD1C	ENST00000402544	p.V2071E	c.6212T>A	Verified	TTT	GTA	CGT	2	T	A
TCGA-61-2008	KANK4	ENST00000371153	p.M413K	c.1238T>A	Verified	GTG	ATG	GTG	2	T	A
TCGA-24-2290	KARS	ENST00000302445	p.I551N	c.1652T>A	Verified	GGC	ATT	GAT	2	T	A
TCGA-24-2290	KARS	ENST00000302445	p.I551N	c.1652T>A	Verified	GGC	ATT	GAT	2	T	A
TCGA-04-1649	KCNA6	ENST00000280684	p.P485P	c.1455T>A	Unverified	CAG	CCT	GCG	3	T	A
TCGA-13-0904	KCNH7	ENST00000332142	p.L828H	c.2483T>A	Verified	GCC	CTC	ACA	2	T	A
TCGA-20-1687	KCNMB1	ENST00000274629	p.F148I	c.442T>A	Unverified	CTA	TTC	CAG	1	T	A
TCGA-23-1124	KDELR3	ENST00000216014	p.F194Y	c.581T>A	Verified	GAC	TTC	TTC	2	T	A
TCGA-30-1857	KDM4D	ENST00000335080	p.L377H	c.1130T>A	Unverified	caa	ctc	cct	2	T	A
TCGA-13-0755	KIAA1109	ENST00000264501	p.P1460P	c.4380T>A	Verified	CAT	CCT	TCT	3	T	A
TCGA-04-1361	KIAA1210	XM_172801.3	p.L189M	c.565T>A	Verified	aac	ttg	gta	1	T	A
TCGA-04-1361	KIAA1210	XM_172801.3	p.L189M	c.565T>A	Verified	aac	ttg	gta	1	T	A
TCGA-29-1784	KIAA1274	ENST00000263563	p.I749N	c.2246T>A	Unverified	ATC	ATC	TGC	2	T	A
TCGA-13-0913	KIAA1919	ENST00000368847	p.F81Y	c.242T>A	Verified	CCT	TTT	TGC	2	T	A
TCGA-24-1469	KIR2DL3	ENST00000342376	p.V11V	c.33T>A	Unverified	TGT	GTT	GGG	3	T	A
TCGA-25-1318	KIR3DL1	ENST00000391728	p.L133Q	c.398T>A	Verified	CCC	CTG	GTG	2	T	A
TCGA-25-1318	KIR3DL1	ENST00000391728	p.L133Q	c.398T>A	Verified	CCC	CTG	GTG	2	T	A
TCGA-13-0760	KIR3DL3	ENST00000291860	p.L202I	c.604T>A	Verified	CAC	TTA	CCC	1	T	A
TCGA-04-1367	KIRREL3	ENST00000278934	p.M296K	c.887T>A	Verified	CGG	ATG	ACC	2	T	A
TCGA-24-1844	KLHL3	ENST00000309755	p.D545E	c.1635T>A	Unverified	GGG	GAT	GAT	3	T	A
TCGA-23-1031	KRT79	ENST00000330553	p.F105L	c.315T>A	Verified	ACG	TTT	GGG	3	T	A
TCGA-29-1711	KRTAP4-2	ENST00000377726	p.C35S	c.103T>A	Unverified	ACC	TGC	TGC	1	T	A

TCGA-29-1711	KRTAP4-2	ENST00000377726	p.C35S	c.103T>A	Unverified	ACC	TGC	TGC	1	T	A
TCGA-04-1649	KRTDAP	ENST00000338897	p.I3N	c.8T>A	Unverified	AAG	ATC	CCG	2	T	A
TCGA-13-0792	LAIR1	ENST00000391742	p.L175H	c.524T>A	Verified	TTC	CTC	TTC	2	T	A
TCGA-13-0885	LAMA1	ENST00000389658	p.C1041S	c.3121T>A	Verified	GGG	TGC	CAG	1	T	A
TCGA-24-1422	LAMA3	ENST00000313654	p.S2220R	c.6660T>A	Verified	ctg	agt	tcc	3	T	A
TCGA-09-1674	LAMC1	ENST00000258341	p.C316S	c.946T>A	Unverified	GAC	TGT	GAA	1	T	A
TCGA-04-1336	LAMC1	ENST00000258341	p.D835E	c.2505T>A	Verified	ATC	GAT	CCC	3	T	A
TCGA-61-2113	LARP7	ENST00000324052	p.D302E	c.906T>A	Unverified	GAA	GAT	GCA	3	T	A
TCGA-29-1699	LATS1_ENST	ENST00000253339	p.L894I	c.2680T>A	Unverified	CCA	TTA	GAG	1	T	A
TCGA-29-1699	LATS1_ENST	ENST00000543571	p.L894I	c.2680T>A	Unverified	CCA	TTA	GAG	1	T	A
TCGA-04-1331	LDOC1L	ENST00000341255	p.I153N	c.458T>A	Unverified	GCT	ATC	CCC	2	T	A
TCGA-09-1665	LHFPL4	ENST00000287585	p.L190H	c.569T>A	Verified	ATC	CTC	TCC	2	T	A
TCGA-13-0761	LHFPL4	ENST00000287585	p.V241E	c.722T>A	Verified	CCC	GTG	GCT	2	T	A
TCGA-13-0761	LHFPL4	ENST00000287585	p.V241E	c.722T>A	Verified	CCC	GTG	GCT	2	T	A
TCGA-23-1120	LIX1L	ENST00000369308	p.S197S	c.591T>A	Verified	GCA	TCT	TTT	3	T	A
TCGA-24-1843	LMO7	ENST00000341547	p.D821E	c.2463T>A	Unverified	ACT	GAT	TTC	3	T	A
TCGA-24-1843	LMO7_ENST	ENST00000357063	p.D1155E	c.3465T>A	Unverified	ACT	GAT	TTC	3	T	A
TCGA-24-1843	LMO7_ENST	ENST00000465261	p.D870E	c.2610T>A	Unverified	ACT	GAT	TTC	3	T	A
TCGA-23-1118	LOC652153	ENST00000316490	p.I595N	c.1784T>A	Verified	TTG	ATC	CCC	2	T	A
TCGA-13-0887	LOC653192	ENST00000272598	p.F371I	c.1111T>A	Unverified	ATA	TTT	CTT	1	T	A
TCGA-23-1022	LPHN3	ENST00000512091	p.S261T	c.781T>A	Verified	AAA	TCT	GAC	1	T	A
TCGA-04-1356	LPPR4	ENST00000370185	p.S444T	c.1330T>A	Verified	CGA	TCA	AAG	1	T	A
TCGA-61-2012	LPPR4	ENST00000370185	p.F286L	c.858T>A	Verified	ACA	TTT	ATC	3	T	A
TCGA-20-1687	LPXN	ENST00000395074	p.F294L	c.882T>A	Unverified	TGC	TTT	GTT	3	T	A
TCGA-20-1687	LPXN_ENST(	ENST00000528954	p.F299L	c.897T>A	Unverified	TGC	TTT	GTT	3	T	A
TCGA-09-1665	LRP1B	ENST00000389484	p.F2895L	c.8685T>A	Verified	TCA	TTT	TTT	3	T	A
TCGA-13-1498	LRRC4C	ENST00000278198	p.I530I	c.1590T>A	Verified	ATC	ATT	GGG	3	T	A
TCGA-10-0930	LRRC7	ENST00000035383	p.V1364E	c.4091T>A	Verified	GAT	GTA	CCT	2	T	A
TCGA-10-0930	LRRC7	ENST00000035383	p.V1364E	c.4091T>A	Verified	GAT	GTA	CCT	2	T	A
TCGA-13-0795	LRRK2	SU_LRRK2	p.S1283T	c.3847T>A	Verified	aga	tcc	ttt	1	T	A
TCGA-23-1032	LRRK2	SU_LRRK2	p.C1152*	c.3456T>A	Unverified	gct	tgt	cct	3	T	A
TCGA-13-0795	LRRK2_ENST	ENST00000298910	p.S1283T	c.3847T>A	Verified	AGA	TCC	TTT	1	T	A
TCGA-23-1032	LRRK2_ENST	ENST00000298910	p.C1152*	c.3456T>A	Unverified	GCT	TGT	CCT	3	T	A
TCGA-13-0923	LZTS1	ENST00000381569	p.L338H	c.1013T>A	Verified	CAG	CTT	CAG	2	T	A

TCGA-24-1474	LZTS2	ENST00000370220	p.M354K	c.1061T>A	Unverified	ACC	ATG	TGC	2	T	A
TCGA-04-1542	MACC1	ENST00000332878	p.S115T	c.343T>A	Verified	GAT	TCC	TCC	1	T	A
TCGA-04-1362	MAGEB16	XM_498354.1	p.D268E	c.804T>A	Verified	gag	gat	aca	3	T	A
TCGA-09-1665	MAGEB6	ENST00000379034	p.Y229N	c.685T>A	Verified	gag	tac	aag	1	T	A
TCGA-09-2049	MAGEC1	ENST00000285879	p.S857T	c.2569T>A	Verified	atc	tcc	ttc	1	T	A
TCGA-13-0714	MAN2A1	ENST00000261483	p.S1010T	c.3028T>A	Verified	ACT	TCT	TCT	1	T	A
TCGA-23-2078	MAN2B2	ENST00000285599	p.T426T	c.1278T>A	Unverified	GGG	ACT	GAG	3	T	A
TCGA-13-0916	MAP2	ENST00000360351	p.S1227S	c.3681T>A	Verified	GTA	TCT	GAA	3	T	A
TCGA-13-1498	MAST2	NM_015112	p.Y625N	c.1873T>A	Verified	gag	tac	tta	1	T	A
TCGA-13-1498	MAST2_ENS	ENST00000361297	p.Y625N	c.1873T>A	Verified	GAG	TAC	TTA	1	T	A
TCGA-13-1505	MC2R	ENST00000399821	p.L66M	c.196T>A	Verified	AGC	TTG	GCC	1	T	A
TCGA-29-1693	MC3R	NM_019888.2	p.V166E	c.497T>A	Unverified	ctg	gtg	gcc	2	T	A
TCGA-29-1784	MCF2	ENST00000370576	p.I377K	c.1130T>A	Unverified	TTT	ATA	AAT	2	T	A
TCGA-29-1784	MCF2_ENST	ENST00000370573	p.I377K	c.1130T>A	Unverified	TTT	ATA	AAT	2	T	A
TCGA-29-1784	MCF2_ENST	ENST00000519895	p.I437K	c.1310T>A	Unverified	TTT	ATA	AAT	2	T	A
TCGA-09-2051	MDN1	ENST00000369393	p.V4613D	c.13838T>A	Unverified	CCG	GTC	CTC	2	T	A
TCGA-09-2056	MEF2A	ENST00000346108	p.S379T	c.1135T>A	Verified	AGC	TCT	CTT	1	T	A
TCGA-24-1417	MEIG1	ENST00000378240	p.Y32*	c.96T>A	Unverified	GGA	TAT	CGG	3	T	A
TCGA-13-0904	MEMO1	ENST00000295065	p.M144K	c.431T>A	Verified	GCC	ATG	GAA	2	T	A
TCGA-23-1110	MFN1	ENST00000263969	p.F35Y	c.104T>A	Verified	CAT	TTT	GTT	2	T	A
TCGA-13-0887	MFSD2B	ENST00000406420	p.I246N	c.737T>A	Verified	TGC	ATC	AGT	2	T	A
TCGA-23-1029	MKLN1	ENST00000352689	p.L179L	c.537T>A	Unverified	CGC	CTT	TGC	3	T	A
TCGA-04-1530	MLL5	ENST00000257745	p.S1471T	c.4411T>A	Verified	CCT	TCA	CAG	1	T	A
TCGA-24-1604	MMP27	ENST00000260229	p.V373E	c.1118T>A	Verified	CGT	GTG	AAG	2	T	A
TCGA-61-1900	MON2	ENST00000393632	p.M470K	c.1409T>A	Unverified	GAA	ATG	TTG	2	T	A
TCGA-29-1784	MRC1	ENST00000239761	p.A85A	c.255T>A	Unverified	GTT	GCT	ATC	3	T	A
TCGA-29-1698	MSH5_ENST	ENST00000375703	p.I590N	c.1769T>A	Unverified	GTC	ATC	ACT	2	T	A
TCGA-29-1698	MSH5_ENST	ENST00000375742	p.I607N	c.1820T>A	Unverified	GTC	ATC	ACT	2	T	A
TCGA-61-2012	MSLNL	ENST00000293892	p.L753Q	c.2258T>A	Unverified	GCT	CTG	CTG	2	T	A
TCGA-04-1651	MSX2	ENST00000239243	p.Y166N	c.496T>A	Unverified	CAG	TAC	CTC	1	T	A
TCGA-04-1651	MSX2	ENST00000239243	p.Y166N	c.496T>A	Unverified	CAG	TAC	CTC	1	T	A
TCGA-23-1122	MTOR	ENST00000361445	p.F370Y	c.1109T>A	Verified	aaa	ttt	gat	2	T	A
TCGA-13-0924	MUC16	ENST00000397910	p.V769D	c.2306T>A	Verified	GCC	GTT	CTT	2	T	A
TCGA-04-1530	MUC16	ENST00000397910	p.P1368P	c.4104T>A	Verified	AGT	CCT	AAA	3	T	A

TCGA-13-0923	MUC17	ENST00000306151	p.T2314T	c.6942T>A	Unverified	TCT	ACT	GAA	3	T	A
TCGA-29-1699	MUC17	ENST00000306151	p.P1201P	c.3603T>A	Unverified	CCT	CCT	CCA	3	T	A
TCGA-13-0920	MYCT1	ENST00000367245	p.L68H	c.203T>A	Verified	GAC	CTT	ATC	2	T	A
TCGA-23-2078	MYH13	ENST00000252172	p.Y195*	c.585T>A	Verified	CAG	TAT	TTT	3	T	A
TCGA-13-0755	MYH15	ENST00000273353	p.A396A	c.1188T>A	Verified	AAA	GCT	GCT	3	T	A
TCGA-13-0887	MYLK	NM_053025	p.L761H	c.2282T>A	Verified	gcc	ctc	tgc	2	T	A
TCGA-23-1110	MYO15A	ENST00000205890	p.F2569I	c.7705T>A	Verified	CAC	TTC	CCA	1	T	A
TCGA-61-1910	MYO1B_ENS	ENST00000392318	p.Y847N	c.2539T>A	Unverified	GCT	TAC	TGG	1	T	A
TCGA-04-1651	MYOM2	ENST00000262113	p.S58T	c.172T>A	Unverified	GCC	TCC	AGC	1	T	A
TCGA-04-1651	MYOM2	ENST00000262113	p.S58T	c.172T>A	Unverified	GCC	TCC	AGC	1	T	A
TCGA-23-1122	MYOM2	ENST00000262113	p.I1092I	c.3276T>A	Verified	CAG	ATT	CAT	3	T	A
TCGA-09-2049	NAT8	ENST00000272425	p.M121K	c.362T>A	Verified	GGC	ATG	GTA	2	T	A
TCGA-24-2035	NAV2	ENST00000360655	p.M881K	c.2642T>A	Unverified	AGC	ATG	GAT	2	T	A
TCGA-13-1498	NCAN	ENST00000252575	p.Y1102N	c.3304T>A	Unverified	CGC	TAT	TTT	1	T	A
TCGA-13-0923	NCL	ENST00000322723	p.A210A	c.630T>A	Verified	gaa	gct	atg	3	T	A
TCGA-29-1691	NCOA2	ENST00000452400	p.S444T	c.1330T>A	Unverified	ggt	tct	ggg	1	T	A
TCGA-13-1501	NDFIP2	ENST00000218652	p.S153R	c.459T>A	Verified	AGT	AGT	ATT	3	T	A
TCGA-13-1501	NDFIP2	ENST00000218652	p.S153R	c.459T>A	Verified	AGT	AGT	ATT	3	T	A
TCGA-24-1844	NDUFB5	ENST00000259037	p.F73Y	c.218T>A	Unverified	AGA	TTC	TAC	2	T	A
TCGA-24-2024	NELL1	ENST00000357134	p.I435N	c.1304T>A	Verified	GAT	ATT	GAT	2	T	A
TCGA-20-1686	NLRP14_ENS	ENST00000299481	p.G856G	c.2568T>A	Unverified	TTG	GGT	GAT	3	T	A
TCGA-13-0884	NLRP9	ENST00000332836	p.P657P	c.1971T>A	Verified	CAG	CCT	GTT	3	T	A
TCGA-24-1604	NMD3	ENST00000351193	p.L164*	c.491T>A	Verified	ACT	TTG	CAT	2	T	A
TCGA-24-2289	NMU	ENST00000264218	p.L42*	c.125T>A	Unverified	ata	tta	cct	2	T	A
TCGA-29-1784	NR1H4_ENS	ENST00000551379	p.I12N	c.35T>A	Unverified	CCA	ATT	CAA	2	T	A
TCGA-29-1777	NSD1	ENST00000439151	p.F97Y	c.290T>A	Unverified	TCC	TTT	CAA	2	T	A
TCGA-29-1777	NSD1_ENST(	ENST00000439151	p.F97Y	c.290T>A	Unverified	TCC	TTT	CAA	2	T	A
TCGA-13-1512	NT5C3	ENST00000242210	p.L192H	c.575T>A	Verified	AAG	CTC	CAA	2	T	A
TCGA-20-1687	NUP210L	ENST00000368559	p.F358I	c.1072T>A	Unverified	GGA	TTT	TTA	1	T	A
TCGA-24-1470	NUP210L	ENST00000368559	p.V1383E	c.4148T>A	Verified	CGA	GTG	AGC	2	T	A
TCGA-13-0755	NXPH2	ENST00000272641	p.F108I	c.322T>A	Verified	AAA	TTT	AAG	1	T	A
TCGA-13-0755	NXPH2	ENST00000272641	p.C20*	c.60T>A	Verified	TTT	TGT	GAC	3	T	A
TCGA-04-1542	ODF4	ENST00000328248	p.S176T	c.526T>A	Verified	GTA	TCC	ATC	1	T	A
TCGA-13-1498	ODZ1	ENST00000371130	p.N1155K	c.3465T>A	Verified	GGG	AAT	GGA	3	T	A

TCGA-13-1498	OLFML1	ENST00000329293	p.T135T	c.405T>A	Verified	CGG	ACT	CTG	3	T	A
TCGA-13-0883	OR10A5	ENST00000299454	p.L195Q	c.584T>A	Verified	GCA	CTG	TTT	2	T	A
TCGA-13-0807	OR10G7	ENST00000330487	p.L12H	c.35T>A	Verified	ATC	CTC	ACG	2	T	A
TCGA-24-1844	OR10S1	ENST00000531945	p.L180Q	c.539T>A	Unverified	CGC	CTG	CTC	2	T	A
TCGA-09-2049	OR12D3	ENST00000377143	p.L113Q	c.338T>A	Verified	TTA	CTG	GCT	2	T	A
TCGA-09-2056	OR1D2	ENST00000331459	p.Y176N	c.526T>A	Verified	CAC	TAC	ATC	1	T	A
TCGA-23-1123	OR2AK2	ENST00000366480	p.S22R	c.66T>A	Verified	CAA	AGT	TTT	3	T	A
TCGA-23-1123	OR2AK2	ENST00000366480	p.S22R	c.66T>A	Verified	CAA	AGT	TTT	3	T	A
TCGA-24-2267	OR2F2	ENST00000408955	p.L32M	c.94T>A	Verified	TTC	TTG	GTC	1	T	A
TCGA-30-1857	OR2J3	ENST00000377169	p.F254I	c.760T>A	Unverified	CTC	TTT	TTC	1	T	A
TCGA-61-1998	OR2L2	ENST00000366479	p.L13I	c.37T>A	Verified	ATC	TTA	TTG	1	T	A
TCGA-23-1114	OR2T11	ENST00000330803	p.F154I	c.460T>A	Unverified	GGC	TTT	CTG	1	T	A
TCGA-25-1313	OR2T12	ENST00000317996	p.L148H	c.443T>A	Verified	TGG	CTC	CTG	2	T	A
TCGA-61-1910	OR4E2	ENST00000408935	p.F250I	c.748T>A	Unverified	CTC	TTC	TTT	1	T	A
TCGA-13-1481	OR4K2	ENST00000298642	p.I285K	c.854T>A	Verified	CCA	ATA	ATC	2	T	A
TCGA-29-1764	OR4K2	ENST00000298642	p.V146E	c.437T>A	Unverified	GTG	GTG	GCT	2	T	A
TCGA-13-0924	OR4K2	ENST00000298642	p.N309K	c.927T>A	Verified	TTT	AAT	AAG	3	T	A
TCGA-13-0900	OR5M9	ENST00000279791	p.V246D	c.737T>A	Verified	GCT	GTT	TCT	2	T	A
TCGA-24-1435	OR6C75	ENST00000343399	p.I133N	c.398T>A	Verified	ATC	ATC	ATG	2	T	A
TCGA-04-1530	OR7D2	ENST00000344248	p.G204G	c.612T>A	Verified	ACG	GGT	GTG	3	T	A
TCGA-61-1740	OR8H1	ENST00000313022	p.S310T	c.928T>A	Unverified	GAC	TCC	AGG	1	T	A
TCGA-29-1768	OR8K3	ENST00000312711	p.I163N	c.488T>A	Unverified	AAG	ATT	TTT	2	T	A
TCGA-23-1123	P4HA1	ENST00000263556	p.G243G	c.729T>A	Verified	AAT	GGT	AAC	3	T	A
TCGA-23-1123	P4HA1	ENST00000263556	p.G243G	c.729T>A	Verified	AAT	GGT	AAC	3	T	A
TCGA-29-2427	PAAF1	ENST00000310571	p.M154K	c.461T>A	Verified	GGA	ATG	GAT	2	T	A
TCGA-24-2289	PAPLN	ENST00000340738	p.M315K	c.944T>A	Unverified	CAC	ATG	TGC	2	T	A
TCGA-23-1029	PCDHB4	ENST00000194152	p.F177I	c.529T>A	Unverified	CAT	TTT	CAC	1	T	A
TCGA-61-2009	PCDHGC5	ENST00000252087	p.I695N	c.2084T>A	Verified	CTC	ATT	GTG	2	T	A
TCGA-61-2009	PCDHGC5_E	ENST00000252087	p.I695N	c.2084T>A	Verified	CTC	ATT	GTG	2	T	A
TCGA-13-0905	PDE10A	ENST00000366882	p.L254H	c.761T>A	Verified	CTA	CTC	GAC	2	T	A
TCGA-13-0793	PDE4A	ENST00000344979	p.L332H	c.995T>A	Verified	GTC	CTC	CTG	2	T	A
TCGA-04-1361	PDIA5	ENST00000316218	p.I449I	c.1347T>A	Verified	AAG	ATT	GCC	3	T	A
TCGA-04-1361	PDIA5	ENST00000316218	p.I449I	c.1347T>A	Verified	AAG	ATT	GCC	3	T	A
TCGA-61-1910	PDPN	ENST00000294489	p.W78R	c.232T>A	Unverified	ATG	TGG	AAG	1	T	A

TCGA-24-1463	PEX3	ENST00000367591	p.D174E	c.522T>A	Unverified	GGA	GAT	GGC	3	T	A
TCGA-29-1781	PGAP1	ENST00000354764	p.L343I	c.1027T>A	Unverified	GAC	TTA	ACA	1	T	A
TCGA-30-1891	PHF20	ENST00000374012	p.V816E	c.2447T>A	Unverified	GCA	GTG	GAG	2	T	A
TCGA-23-1114	PHKA1	ENST00000373542	p.F594I	c.1780T>A	Unverified	TAT	TTT	GGT	1	T	A
TCGA-29-1703	PHRF1	ENST00000416188	p.C186S	c.556T>A	Unverified	TTC	TGT	GAG	1	T	A
TCGA-29-1703	PHRF1_ENST	ENST00000416188	p.C186S	c.556T>A	Unverified	TTC	TGT	GAG	1	T	A
TCGA-13-0885	PIP4K2A	ENST00000376573	p.I401N	c.1202T>A	Verified	ttt	att	ggc	2	T	A
TCGA-29-1783	PKHD1L1	ENST00000378402	p.F2614Y	c.7841T>A	Unverified	TTT	TTT	AAC	2	T	A
TCGA-25-1326	PLAC1L	ENST00000278855	p.L148I	c.442T>A	Verified	GAG	TTA	GGA	1	T	A
TCGA-04-1347	PLEKHA3	ENST00000234453	p.W19R	c.55T>A	Verified	cgt	tgg	ttt	1	T	A
TCGA-23-1031	PNN	ENST00000216832	p.D436E	c.1308T>A	Verified	CCA	GAT	AAA	3	T	A
TCGA-29-1784	POLR1A	ENST00000263857	p.S1689T	c.5065T>A	Unverified	GGA	TCC	CAC	1	T	A
TCGA-29-1705	POLR2B	ENST00000381227	p.C357S	c.1069T>A	Unverified	TTT	TGT	GAG	1	T	A
TCGA-24-1103	POLR2B	ENST00000381227	p.S250R	c.750T>A	Verified	AAG	AGT	GCT	3	T	A
TCGA-23-1022	PPFIA4	XM_046751.7	p.W1178R	c.3532T>A	Verified	gtc	tgg	acc	1	T	A
TCGA-61-1738	PPP1R3B	ENST00000310455	p.M243K	c.728T>A	Unverified	GGA	ATG	ACC	2	T	A
TCGA-61-1738	PPP1R3B	ENST00000310455	p.M243K	c.728T>A	Unverified	GGA	ATG	ACC	2	T	A
TCGA-29-1784	PPP2R3A	ENST00000264977	p.F1089I	c.3265T>A	Unverified	cgg	ttt	gcc	1	T	A
TCGA-09-1665	PPP2R3A	ENST00000264977	p.S723R	c.2169T>A	Verified	tgt	agt	aat	3	T	A
TCGA-29-1784	PPP2R3A_EN	ENST00000334546	p.F468I	c.1402T>A	Unverified	CGG	TTT	GCC	1	T	A
TCGA-24-1844	PRAMEF12	ENST00000357726	p.L9H	c.26T>A	Unverified	AGA	CTC	CTG	2	T	A
TCGA-29-1761	PRDM2	ENST00000235372	p.D924E	c.2772T>A	Unverified	CCA	GAT	CCT	3	T	A
TCGA-13-0904	PRKAA2_EN	ENST00000371244	p.H73Q	c.219T>A	Verified	CGT	CAT	CCT	3	T	A
TCGA-13-0905	PRKCB	ENST00000303531	p.C386S	c.1156T>A	Verified	GAG	TGC	ACT	1	T	A
TCGA-29-1702	PRKCB	ENST00000303531	p.C67S	c.199T>A	Unverified	CAG	TGC	CAA	1	T	A
TCGA-29-1702	PRKCB_ENST	ENST00000303531	p.C67S	c.199T>A	Unverified	CAG	TGC	CAA	1	T	A
TCGA-29-1702	PRKCB_ENST	ENST00000321728	p.C67S	c.199T>A	Unverified	CAG	TGC	CAA	1	T	A
TCGA-13-0885	PRMT10	ENST00000322396	p.L157L	c.471T>A	Verified	ATG	CTT	AAT	3	T	A
TCGA-13-0916	PROX1	ENST00000498508	p.S275T	c.823T>A	Verified	ctg	tct	gaa	1	T	A
TCGA-24-2035	PRPF6	ENST00000266079	p.V392V	c.1176T>A	Verified	CGG	GTT	CTT	3	T	A
TCGA-04-1343	PRR5L	ENST00000378867	p.L89H	c.266T>A	Verified	GAA	CTT	GGA	2	T	A
TCGA-23-2078	PSG1_ENST(	ENST00000449000	p.W67R	c.199T>A	Unverified	ATC	TGG	TAC	1	T	A
TCGA-23-2078	PSG2	ENST00000406487	p.W67R	c.199T>A	Unverified	ATC	TGG	TAC	1	T	A
TCGA-25-1326	PTCRA	ENST00000304672	p.V52D	c.155T>A	Verified	GAT	GTT	GCA	2	T	A

TCGA-23-1114	PTPN13	ENST00000436978	p.L1793H	c.5378T>A	Unverified	CTC	CTC	ATT	2	T	A
TCGA-13-0920	PTPN4	ENST00000263708	p.T39T	c.117T>A	Verified	aac	act	gta	3	T	A
TCGA-24-1847	PTPRJ	ENST00000418331	p.M1281K	c.3842T>A	Unverified	TTA	ATG	GTG	2	T	A
TCGA-24-1847	PTPRJ_ENST	ENST00000418331	p.M1281K	c.3842T>A	Unverified	TTA	ATG	GTG	2	T	A
TCGA-13-0887	PTPRK	ENST00000368226	p.S1304S	c.3912T>A	Verified	atg	tct	tgt	3	T	A
TCGA-24-2280	RAB11FIP1	ENST00000330843	p.S190T	c.568T>A	Unverified	GCC	TCC	GCC	1	T	A
TCGA-29-1693	RAGE	ENST00000361847	p.W166R	c.496T>A	Unverified	cgc	tgg	tac	1	T	A
TCGA-30-1855	RAPGEF4	NM_007023.1	p.Y395N	c.1183T>A	Unverified	tgg	tac	att	1	T	A
TCGA-04-1361	RDH8	ENST00000171214	p.L87Q	c.260T>A	Verified	GTG	CTG	GTG	2	T	A
TCGA-04-1361	RDH8	ENST00000171214	p.L87Q	c.260T>A	Verified	GTG	CTG	GTG	2	T	A
TCGA-30-1857	RFC5	ENST00000454402	p.I326N	c.977T>A	Unverified	ctc	att	gct	2	T	A
TCGA-25-1313	RGS22	ENST00000360863	p.Y407*	c.1221T>A	Verified	ACT	TAT	GAC	3	T	A
TCGA-13-0893	RGS4	ENST00000367909	p.L107Q	c.320T>A	Verified	aaa	cta	agt	2	T	A
TCGA-13-0807	RGS7	ENST00000366565	p.L145H	c.434T>A	Verified	GAG	CTC	GCA	2	T	A
TCGA-24-1103	RIPK2	ENST00000220751	p.I208N	c.623T>A	Verified	AGT	ATC	AAG	2	T	A
TCGA-29-1783	RNF148	ENST00000434824	p.F262I	c.784T>A	Unverified	TGC	TTT	GAC	1	T	A
TCGA-29-1783	RNF148_EN!	ENST00000434824	p.F262I	c.784T>A	Unverified	TGC	TTT	GAC	1	T	A
TCGA-61-1915	RNF157	XM_290732.4	p.S428R	c.1284T>A	Unverified	gat	agt	ggc	3	T	A
TCGA-09-2056	RNF19A	ENST00000519449	p.I309K	c.926T>A	Verified	ata	ata	aag	2	T	A
TCGA-29-1770	RNF213	ENST00000336301	p.W1733R	c.5197T>A	Unverified	CCC	TGG	GCA	1	T	A
TCGA-04-1336	RNF213	ENST00000336301	p.L2463H	c.7388T>A	Unverified	AGC	CTC	CAC	2	T	A
TCGA-29-1770	RNF213_EN!	ENST00000411702	p.W3709R	c.11125T>A	Unverified	CCC	TGG	GCA	1	T	A
TCGA-04-1336	RNF213_EN!	ENST00000411702	p.L4439H	c.13316T>A	Unverified	AGC	CTC	CAC	2	T	A
TCGA-23-1116	ROBO1_ENS	ENST00000464233	p.S1609T	c.4825T>A	Unverified	TCA	TCA	AGA	1	T	A
TCGA-23-1116	ROBO1_ENS	ENST00000464233	p.S1609T	c.4825T>A	Unverified	TCA	TCA	AGA	1	T	A
TCGA-61-2094	ROBO2	ENST00000461745	p.Y388N	c.1162T>A	Unverified	GGT	TAC	TAC	1	T	A
TCGA-23-1032	ROBO2	ENST00000461745	p.I973N	c.2918T>A	Verified	GCC	ATT	TAT	2	T	A
TCGA-61-2094	ROBO2_ENS	ENST00000487694	p.Y404N	c.1210T>A	Unverified	GGT	TAC	TAC	1	T	A
TCGA-24-1422	RP1	ENST00000220676	p.V1264E	c.3791T>A	Verified	ACT	GTA	AAT	2	T	A
TCGA-29-1781	RRAGA	ENST00000380527	p.S257T	c.769T>A	Unverified	AAT	TCC	AAC	1	T	A
TCGA-24-2288	RSBN1L	ENST00000334955	p.S683R	c.2049T>A	Unverified	TGC	AGT	TTA	3	T	A
TCGA-23-1122	RSPRY1	ENST00000394420	p.P416P	c.1248T>A	Verified	AAG	CCT	CAC	3	T	A
TCGA-25-2398	RYR1	ENST00000359596	p.M80K	c.239T>A	Unverified	GAG	ATG	CTG	2	T	A
TCGA-04-1347	RYR2	ENST00000366574	p.V3045E	c.9134T>A	Verified	ACA	GTG	ATG	2	T	A

TCGA-24-1845	S100A11	ENST00000271638	p.F77Y	c.230T>A	Unverified	GAT	TTC	TCA	2	T	A
TCGA-13-0904	SACS	NM_014363.3	p.V1543E	c.4628T>A	Verified	agt	gta	aag	2	T	A
TCGA-25-1313	SCAF1	ENST00000360565	p.I227N	c.680T>A	Unverified	GAT	ATC	TAT	2	T	A
TCGA-61-1998	SCARF1	ENST00000263071	p.C41S	c.121T>A	Unverified	CAG	TGC	TGC	1	T	A
TCGA-04-1343	SCN3A	ENST00000283254	p.L719H	c.2156T>A	Verified	GAA	CTT	GAA	2	T	A
TCGA-13-1408	SCN4A	ENST00000435607	p.V1203D	c.3608T>A	Unverified	GAG	GTC	AAC	2	T	A
TCGA-25-2400	SCN7A_ENST	ENST00000409855	p.L152L	c.456T>A	Unverified	TTG	CTT	GGA	3	T	A
TCGA-25-2401	SELP	ENST00000367790	p.S596T	c.1786T>A	Verified	GGC	TCC	ACC	1	T	A
TCGA-13-0884	SENP8	ENST00000542035	p.C175*	c.525T>A	Verified	TTG	TGT	CAG	3	T	A
TCGA-13-0760	SEPN1	ENST00000361547	p.L210L	c.630T>A	Verified	TTC	CTT	CCC	3	T	A
TCGA-30-1856	SERPINA4	ENST00000557004	p.I396N	c.1187T>A	Unverified	CAC	ATC	CTG	2	T	A
TCGA-24-2280	SERPINA7	ENST00000327674	p.F82Y	c.245T>A	Unverified	TCC	TTT	GGG	2	T	A
TCGA-29-1784	SETBP1	ENST00000282030	p.S1330T	c.3988T>A	Unverified	TCC	TCC	ATG	1	T	A
TCGA-13-0923	SETD2	ENST00000330022	p.I1297N	c.3890T>A	Verified	GAG	ATT	ATA	2	T	A
TCGA-24-2290	SETD2	ENST00000330022	p.Y1673*	c.5019T>A	Verified	GCC	TAT	GTG	3	T	A
TCGA-24-2290	SETD2	ENST00000330022	p.Y1673*	c.5019T>A	Verified	GCC	TAT	GTG	3	T	A
TCGA-13-0923	SETD2_ENST	ENST00000409792	p.I1800N	c.5399T>A	Verified	GAG	ATT	ATA	2	T	A
TCGA-24-2290	SETD2_ENST	ENST00000409792	p.Y2176*	c.6528T>A	Verified	GCC	TAT	GTG	3	T	A
TCGA-24-2290	SETD2_ENST	ENST00000409792	p.Y2176*	c.6528T>A	Verified	GCC	TAT	GTG	3	T	A
TCGA-13-0920	SEZ6L2	ENST00000308713	p.S48T	c.142T>A	Verified	GCC	TCT	GAG	1	T	A
TCGA-29-1691	SH2D7	ENST00000328828	p.S275T	c.823T>A	Unverified	TAC	TCC	CCA	1	T	A
TCGA-24-1847	SHROOM4	ENST00000289292	p.L106Q	c.317T>A	Unverified	aag	ctg	ctg	2	T	A
TCGA-13-1408	SIM1	ENST00000262901	p.C605S	c.1813T>A	Verified	CTG	TGT	TTT	1	T	A
TCGA-13-0886	SLC17A1	ENST00000244527	p.L22M	c.64T>A	Verified	GGA	TTG	TCT	1	T	A
TCGA-23-1110	SLC22A10	ENST00000332793	p.S81T	c.241T>A	Verified	GAC	TCA	AAT	1	T	A
TCGA-04-1655	SLC22A14	ENST00000273173	p.I542N	c.1625T>A	Unverified	CCC	ATC	TTT	2	T	A
TCGA-61-1737	SLC22A9	ENST00000279178	p.S126T	c.376T>A	Unverified	ATC	TCC	TTC	1	T	A
TCGA-13-0900	SLC24A4	ENST00000298877	p.V422D	c.1265T>A	Verified	TGC	GTC	ACC	2	T	A
TCGA-04-1651	SLC24A5	ENST00000341459	p.S84T	c.250T>A	Unverified	ATA	TCT	ATT	1	T	A
TCGA-04-1651	SLC24A5	ENST00000341459	p.S84T	c.250T>A	Unverified	ATA	TCT	ATT	1	T	A
TCGA-23-1124	SLC38A11	ENST00000303735	p.L99H	c.296T>A	Verified	AAG	CTT	GGA	2	T	A
TCGA-29-1764	SLC39A12	ENST00000377374	p.L401*	c.1202T>A	Unverified	ATC	TTA	CAG	2	T	A
TCGA-61-1904	SLC39A9	ENST00000336643	p.I24N	c.71T>A	Unverified	ATC	ATT	CCC	2	T	A
TCGA-13-0913	SLC4A11	ENST00000380056	p.F68Y	c.203T>A	Verified	GCC	TTC	GAC	2	T	A

TCGA-23-1120	SLCO4A1	ENST00000370507	p.S560T	c.1678T>A	Unverified	TCC	TCT	GGT	1	T	A
TCGA-29-1698	SLMAP	ENST00000295951	p.N281K	c.843T>A	Unverified	AGT	AAT	ACT	3	T	A
TCGA-24-1422	SMC2	ENST00000286398	p.V1072D	c.3215T>A	Verified	aag	gtt	gcc	2	T	A
TCGA-25-2391	SNCA	ENST00000394986	p.A19A	c.57T>A	Unverified	GCT	GCT	GAG	3	T	A
TCGA-25-1313	SNTG1	ENST00000518864	p.L186*	c.557T>A	Verified	ACA	TTA	TCA	2	T	A
TCGA-09-2050	SNTG2	ENST00000308624	p.N70K	c.210T>A	Verified	AGG	AAT	CGC	3	T	A
TCGA-24-1844	SNX31	ENST00000311812	p.F347I	c.1039T>A	Unverified	AGA	TTT	CAA	1	T	A
TCGA-24-2280	SOCS2	ENST00000340600	p.I72N	c.215T>A	Unverified	ttg	att	aga	2	T	A
TCGA-61-1738	SORCS1	ENST00000263054	p.Y536N	c.1606T>A	Unverified	CCC	TAC	ACA	1	T	A
TCGA-61-1738	SORCS1	ENST00000263054	p.Y536N	c.1606T>A	Unverified	CCC	TAC	ACA	1	T	A
TCGA-61-1738	SORCS1_EN!	ENST00000344440	p.Y536N	c.1606T>A	Unverified	CCC	TAC	ACA	1	T	A
TCGA-61-1738	SORCS1_EN!	ENST00000344440	p.Y536N	c.1606T>A	Unverified	CCC	TAC	ACA	1	T	A
TCGA-24-1845	SP140_ENST	ENST00000392045	p.C478S	c.1432T>A	Unverified	GCG	TGT	GGC	1	T	A
TCGA-13-0923	SP4	ENST00000222584	p.D388E	c.1164T>A	Verified	CAG	GAT	CAA	3	T	A
TCGA-24-1847	SPARC	ENST00000231061	p.L8H	c.23T>A	Unverified	TTT	CTC	CTT	2	T	A
TCGA-13-0791	SPATS2L	ENST00000409718	p.I136K	c.407T>A	Verified	TCG	ATA	CTT	2	T	A
TCGA-25-2400	SPERT	ENST00000310521	p.L257Q	c.770T>A	Unverified	GCG	CTG	CAG	2	T	A
TCGA-24-1844	SPINK14	ENST00000356972	p.V7E	c.20T>A	Unverified	CCA	GTA	TTC	2	T	A
TCGA-13-0883	SPRED2	ENST00000356388	p.V19E	c.56T>A	Verified	GCT	GTG	GTT	2	T	A
TCGA-13-0919	SPTBN1	ENST00000356805	p.L1314*	c.3941T>A	Unverified	TGG	TTG	AAG	2	T	A
TCGA-29-1698	SPTBN2	ENST00000309996	p.S2119T	c.6355T>A	Unverified	GCT	TCT	GAC	1	T	A
TCGA-13-1510	SQLE	ENST00000265896	p.T3T	c.9T>A	Verified	TGG	ACT	TTT	3	T	A
TCGA-13-1510	SRRM2	ENST00000301740	p.S554S	c.1662T>A	Unverified	AGG	TCT	AGG	3	T	A
TCGA-24-0979	SSBP3	ENST00000371319	p.M285K	c.854T>A	Unverified	CCG	ATG	GGT	2	T	A
TCGA-13-0714	STAB2	ENST00000388887	p.V1128D	c.3383T>A	Verified	CTG	GTC	CCA	2	T	A
TCGA-13-0890	STAB2	ENST00000388887	p.V52D	c.155T>A	Verified	GGA	GTC	AAG	2	T	A
TCGA-13-1498	STAR	ENST00000276449	p.V28E	c.83T>A	Verified	GCT	GTG	ATG	2	T	A
TCGA-23-1118	STARD13	ENST00000336934	p.V123E	c.368T>A	Verified	GAT	GTG	AAC	2	T	A
TCGA-13-1481	STEAP2	ENST00000394621	p.F363I	c.1087T>A	Verified	TCC	TTT	GGC	1	T	A
TCGA-13-0714	STK32C	ENST00000298630	p.Y256N	c.766T>A	Unverified	CCG	TAC	ATG	1	T	A
TCGA-20-1685	STK33	ENST00000447869	p.F323I	c.967T>A	Unverified	CCC	TTT	TTG	1	T	A
TCGA-20-1685	STK33_ENST	ENST00000447869	p.F323I	c.967T>A	Unverified	CCC	TTT	TTG	1	T	A
TCGA-29-1777	SUPT6H	ENST00000314616	p.Y693N	c.2077T>A	Unverified	TTT	TAC	TAC	1	T	A
TCGA-29-1761	SVEP1	ENST00000401783	p.F2102Y	c.6305T>A	Unverified	AAA	TTT	GCA	2	T	A

TCGA-20-1687	SYNE2	ENST00000358025	p.L187L	c.561T>A	Unverified	GCC	CTT	CTT	3	T	A
TCGA-04-1655	SYT12	ENST00000393946	p.L405M	c.1213T>A	Unverified	ATG	TTG	GCC	1	T	A
TCGA-09-1665	SYT7	ENST00000263846	p.W384R	c.1150T>A	Verified	CAC	TGG	AAG	1	T	A
TCGA-61-1910	TAS2R42	ENST00000334266	p.I56N	c.167T>A	Unverified	ACA	ATT	GGA	2	T	A
TCGA-23-1124	TAS2R8	ENST00000240615	p.L146H	c.437T>A	Verified	AGC	CTT	ATA	2	T	A
TCGA-29-1769	TCEA1_ENST	ENST00000521604	p.L28Q	c.83T>A	Unverified	TTG	CTA	AAG	2	T	A
TCGA-13-0913	THBS1	ENST00000260356	p.P315P	c.945T>A	Verified	cgg	cct	ccc	3	T	A
TCGA-13-1499	THRAP3	ENST00000354618	p.G735G	c.2205T>A	Verified	CGA	GGT	AAA	3	T	A
TCGA-61-1910	THSD1	ENST00000258613	p.I494N	c.1481T>A	Unverified	GGC	ATC	CCT	2	T	A
TCGA-13-0793	THSD1	ENST00000258613	p.G199G	c.597T>A	Verified	CAA	GGT	CAG	3	T	A
TCGA-13-0900	TIMD4	ENST00000274532	p.C111S	c.331T>A	Verified	TAC	TGC	TGC	1	T	A
TCGA-23-1110	TMC7	ENST00000304381	p.W487R	c.1459T>A	Verified	TGC	TGG	GAG	1	T	A
TCGA-13-0760	TMEM108	ENST00000321871	p.L430H	c.1289T>A	Verified	TTC	CTC	AAC	2	T	A
TCGA-25-1318	TMEM180	ENST00000238936	p.F435Y	c.1304T>A	Unverified	CTC	TTC	CAG	2	T	A
TCGA-25-1318	TMEM180	ENST00000238936	p.F435Y	c.1304T>A	Unverified	CTC	TTC	CAG	2	T	A
TCGA-13-0885	TMEM209	ENST00000397622	p.S425T	c.1273T>A	Verified	AGC	TCA	TTT	1	T	A
TCGA-13-0906	TMEM48	ENST00000371429	p.V455E	c.1364T>A	Verified	AGT	GTA	ATG	2	T	A
TCGA-61-1915	TNFRSF8	ENST00000263932	p.L52Q	c.155T>A	Unverified	GGG	CTG	TTC	2	T	A
TCGA-25-2398	TNFRSF9	ENST00000377507	p.V9E	c.26T>A	Verified	ata	gta	gcc	2	T	A
TCGA-24-1844	TP53	ENST00000269305	p.C176S	c.526T>A	Unverified	CGC	TGC	CCC	1	T	A
TCGA-09-2050	TP53	ENST00000269305	p.L257Q	c.770T>A	Verified	ACA	CTG	GAA	2	T	A
TCGA-13-1501	TP53	ENST00000269305	p.L145Q	c.434T>A	Verified	CAG	CTG	TGG	2	T	A
TCGA-13-1501	TP53	ENST00000269305	p.L145Q	c.434T>A	Verified	CAG	CTG	TGG	2	T	A
TCGA-24-1463	TP53	ENST00000269305	p.V274D	c.821T>A	Verified	CGT	GTT	TGT	2	T	A
TCGA-04-1347	TP53	ENST00000269305	p.S215R	c.645T>A	Verified	CAT	AGT	GTG	3	T	A
TCGA-24-1844	TP53_ENST(	ENST00000269305	p.C176S	c.526T>A	Unverified	CGC	TGC	CCC	1	T	A
TCGA-24-1844	TP53_ENST(	ENST00000413465	p.C176S	c.526T>A	Unverified	CGC	TGC	CCC	1	T	A
TCGA-24-1844	TP53_ENST(	ENST00000414315	p.C44S	c.130T>A	Unverified	CGC	TGC	CCC	1	T	A
TCGA-24-1844	TP53_ENST(	ENST00000545858	p.C83S	c.247T>A	Unverified	CGC	TGC	CCC	1	T	A
TCGA-24-1469	TPD52L2	ENST00000217121	p.S110S	c.330T>A	Verified	ACT	TCT	GAG	3	T	A
TCGA-24-1843	TRHDE	ENST00000261180	p.L727Q	c.2180T>A	Unverified	AGC	CTA	GCC	2	T	A
TCGA-13-0923	TRIM29	ENST00000341846	p.S388T	c.1162T>A	Verified	TAC	TCT	CTC	1	T	A
TCGA-29-1761	TRPC1	ENST00000273482	p.Y476N	c.1426T>A	Unverified	AGT	TAT	CTT	1	T	A
TCGA-61-1998	TTC7B	ENST00000328459	p.T605T	c.1815T>A	Verified	CTG	ACT	TGT	3	T	A

TCGA-23-2077	TTLL5	ENST00000298832	p.P487P	c.1461T>A	Verified	TTT	CCT	ACA	3	T	A
TCGA-13-1505	TTN	NM_003319	p.S11069T	c.33205T>A	Verified	tca	tca	gta	1	T	A
TCGA-13-0760	TTN	NM_003319	p.V5150D	c.15449T>A	Verified	caa	gtc	aag	2	T	A
TCGA-29-1699	TTN	NM_003319	p.I20435N	c.61304T>A	Unverified	ctc	atc	aaa	2	T	A
TCGA-24-2290	TTN	NM_003319	p.T3310T	c.9930T>A	Verified	gtc	act	tct	3	T	A
TCGA-24-2290	TTN	NM_003319	p.T3310T	c.9930T>A	Verified	gtc	act	tct	3	T	A
TCGA-61-1910	TTN	NM_003319	p.P25577P	c.76731T>A	Unverified	tct	cct	gat	3	T	A
TCGA-29-1699	TTN_ENST0(	ENST00000342175	p.I20627N	c.61880T>A	Unverified	CTC	ATC	AAA	2	T	A
TCGA-61-1910	TTN_ENST0(	ENST00000342175	p.P25769P	c.77307T>A	Unverified	TCT	CCT	GAT	3	T	A
TCGA-29-1699	TTN_ENST0(	ENST00000342992	p.I26932N	c.80795T>A	Unverified	CTC	ATC	AAA	2	T	A
TCGA-61-1910	TTN_ENST0(	ENST00000342992	p.P32074P	c.96222T>A	Unverified	TCT	CCT	GAT	3	T	A
TCGA-13-1505	TTN_ENST0(	ENST00000356127	p.S17564T	c.52690T>A	Verified	TCA	TCA	GTA	1	T	A
TCGA-13-0760	TTN_ENST0(	ENST00000356127	p.V11647D	c.34940T>A	Verified	CAA	GTC	AAG	2	T	A
TCGA-29-1699	TTN_ENST0(	ENST00000356127	p.I26930N	c.80789T>A	Unverified	CTC	ATC	AAA	2	T	A
TCGA-24-2290	TTN_ENST0(	ENST00000356127	p.T3356T	c.10068T>A	Verified	GTC	ACT	TCT	3	T	A
TCGA-24-2290	TTN_ENST0(	ENST00000356127	p.T3356T	c.10068T>A	Verified	GTC	ACT	TCT	3	T	A
TCGA-61-1910	TTN_ENST0(	ENST00000356127	p.P32072P	c.96216T>A	Unverified	TCT	CCT	GAT	3	T	A
TCGA-29-1699	TTN_ENST0(	ENST00000359218	p.I20560N	c.61679T>A	Unverified	CTC	ATC	AAA	2	T	A
TCGA-61-1910	TTN_ENST0(	ENST00000359218	p.P25702P	c.77106T>A	Unverified	TCT	CCT	GAT	3	T	A
TCGA-24-2290	TTN_ENST0(	ENST00000360870	p.T3356T	c.10068T>A	Verified	GTC	ACT	TCT	3	T	A
TCGA-24-2290	TTN_ENST0(	ENST00000360870	p.T3356T	c.10068T>A	Verified	GTC	ACT	TCT	3	T	A
TCGA-25-1313	TTN_ENST0(	ENST00000360870	p.V4863V	c.14589T>A	Verified	GAA	GTT	CAA	3	T	A
TCGA-29-1690	TUBB	ENST00000327892	p.T149T	c.447T>A	Unverified	GGC	ACT	CTC	3	T	A
TCGA-09-2050	TUBB2C	ENST00000340384	p.F408I	c.1222T>A	Verified	GAG	TTC	ACC	1	T	A
TCGA-29-1768	TXLNB	ENST00000358430	p.D99E	c.297T>A	Unverified	GAG	GAT	GGG	3	T	A
TCGA-13-0906	TYK2	ENST00000525621	p.V298E	c.893T>A	Unverified	GGG	GTG	GCC	2	T	A
TCGA-25-2400	UBA7	ENST00000333486	p.V35D	c.104T>A	Unverified	agg	gtc	ctg	2	T	A
TCGA-04-1336	UHRF1BP1	ENST00000192788	p.V654D	c.1961T>A	Verified	AAG	GTT	CCA	2	T	A
TCGA-23-2077	UNC13A	ENST00000428389	p.V116E	c.347T>A	Verified	AAA	GTG	CAG	2	T	A
TCGA-23-1114	UNC80	ENST00000281753	p.L187H	c.560T>A	Unverified	GAG	CTC	TTC	2	T	A
TCGA-23-1114	UNC80_ENS	ENST00000439458	p.L187H	c.560T>A	Unverified	GAG	CTC	TTC	2	T	A
TCGA-13-0920	USH2A	ENST00000307340	p.C3359*	c.10077T>A	Verified	TGC	TGT	GAG	3	T	A
TCGA-13-1501	USP24	XM_371254.3	p.S1211T	c.3631T>A	Verified	tca	tcc	tac	1	T	A
TCGA-13-1501	USP24	XM_371254.3	p.S1211T	c.3631T>A	Verified	tca	tcc	tac	1	T	A

TCGA-23-1123	VCAN	ENST00000265077	p.L1799I	c.5395T>A	Verified	ACA	TTA	GAA	1	T	A
TCGA-23-1123	VCAN	ENST00000265077	p.L1799I	c.5395T>A	Verified	ACA	TTA	GAA	1	T	A
TCGA-13-0885	VPS13B	ENST00000358544	p.S1189S	c.3567T>A	Verified	acg	tct	caa	3	T	A
TCGA-24-1474	VPS13D	ENST00000358136	p.V732V	c.2196T>A	Verified	TTA	GTT	GTC	3	T	A
TCGA-24-1847	VWF	ENST00000261405	p.C1927S	c.5779T>A	Unverified	TCG	TGC	CCT	1	T	A
TCGA-29-1785	WDR20	ENST00000335263	p.L146Q	c.437T>A	Unverified	AGA	CTA	ATA	2	T	A
TCGA-29-1785	WDR20	ENST00000335263	p.L146Q	c.437T>A	Unverified	AGA	CTA	ATA	2	T	A
TCGA-24-2035	WDR78	ENST00000371026	p.W19R	c.55T>A	Unverified	GCT	TGG	GGG	1	T	A
TCGA-29-1711	WDYHV1	ENST00000287387	p.I32I	c.96T>A	Unverified	AAT	ATT	TGG	3	T	A
TCGA-29-1711	WDYHV1	ENST00000287387	p.I32I	c.96T>A	Unverified	AAT	ATT	TGG	3	T	A
TCGA-29-1768	WHSC2	ENST00000333877	p.V451D	c.1352T>A	Unverified	AAA	GTC	ACG	2	T	A
TCGA-23-1022	WNT2	ENST00000265441	p.A256A	c.768T>A	Verified	GTG	GCT	AAC	3	T	A
TCGA-13-1481	YAP1	ENST00000526343	p.L450I	c.1348T>A	Verified	TGG	TTA	TAG	1	T	A
TCGA-29-1784	YSK4	AK131216.1	p.S45T	c.133T>A	Unverified	cca	tca	ggc	1	T	A
TCGA-25-1326	YSK4	AK131216.1	p.C533*	c.1599T>A	Verified	aac	tgt	gtg	3	T	A
TCGA-29-1784	YSK4_ENST0	ENST00000375845	p.S693T	c.2077T>A	Unverified	CCA	TCA	GGC	1	T	A
TCGA-25-1326	YSK4_ENST0	ENST00000375845	p.C1181*	c.3543T>A	Verified	AAC	TGT	GTG	3	T	A
TCGA-13-0714	ZBTB40	ENST00000404138	p.V766E	c.2297T>A	Verified	CAG	GTG	CAG	2	T	A
TCGA-04-1336	ZFHX3	ENST00000268489	p.S3432T	c.10294T>A	Verified	GTG	TCC	CCC	1	T	A
TCGA-04-1336	ZFHX4	ENST00000521891	p.N1528K	c.4584T>A	Verified	CCC	AAT	TTT	3	T	A
TCGA-61-1900	ZFP57	ENST00000376883	p.T116T	c.348T>A	Unverified	GGG	ACT	AGT	3	T	A
TCGA-29-1777	ZFPM2	ENST00000407775	p.C770*	c.2310T>A	Unverified	CCT	TGT	ACC	3	T	A
TCGA-61-1906	ZFYVE9	ENST00000287727	p.Y957*	c.2871T>A	Unverified	aat	tat	gtg	3	T	A
TCGA-61-1906	ZFYVE9	ENST00000287727	p.Y957*	c.2871T>A	Unverified	aat	tat	gtg	3	T	A
TCGA-13-1408	ZMIZ2	ENST00000309315	p.C639*	c.1917T>A	Unverified	AGG	TGT	CCT	3	T	A
TCGA-25-2391	ZNF14	ENST00000344099	p.F531L	c.1593T>A	Unverified	CCT	TTT	GAG	3	T	A
TCGA-23-1110	ZNF235	ENST00000291182	p.L558H	c.1673T>A	Verified	AAT	CTT	CAC	2	T	A
TCGA-13-0923	ZNF354B	ENST00000322434	p.I538K	c.1613T>A	Verified	CTT	ATA	CAA	2	T	A
TCGA-29-1762	ZNF418	ENST00000396147	p.S159S	c.477T>A	Unverified	TCA	TCT	ATC	3	T	A
TCGA-13-0916	ZNF432_ENS	ENST00000354939	p.F656I	c.1966T>A	Verified	CGA	TTT	CAC	1	T	A
TCGA-24-1103	ZNF491	ENST00000323169	p.F135I	c.403T>A	Verified	AAG	TTT	TGT	1	T	A
TCGA-13-0887	ZNF521	NM_015461.1	p.L575L	c.1725T>A	Verified	gtt	ctt	aaa	3	T	A
TCGA-24-1417	ZNF536	ENST00000355537	p.F607I	c.1819T>A	Verified	GAT	TTT	TTG	1	T	A
TCGA-13-0893	ZNF549	ENST00000240719	p.V320E	c.959T>A	Verified	TAT	GTG	TGC	2	T	A

TCGA-13-0916	ZNF615	ENST00000376716	p.F645I	c.1933T>A	Verified	CGA	TTT	CAC	1	T	A
TCGA-29-1693	ZNF638	ENST00000264447	p.I423K	c.1268T>A	Unverified	AGA	ATA	TTT	2	T	A
TCGA-61-2008	ZNF652	ENST00000362063	p.S216R	c.648T>A	Verified	AAG	AGT	GTA	3	T	A
TCGA-29-1691	ZNF766	ENST00000439461	p.P28P	c.84T>A	Unverified	GAC	CCT	GTG	3	T	A
TCGA-13-0887	ZNF804A	ENST00000302277	p.T177T	c.531T>A	Verified	AAT	ACT	AAA	3	T	A
TCGA-23-1122	ZNF804B	ENST00000333190	p.D385E	c.1155T>A	Verified	CTG	GAT	GAG	3	T	A
TCGA-10-0930	ZNF833P	ENST00000344893	p.S167R	c.501T>A	Verified	CAT	AGT	CAT	3	T	A
TCGA-10-0930	ZNF833P	ENST00000344893	p.S167R	c.501T>A	Verified	CAT	AGT	CAT	3	T	A
TCGA-23-1022	ZNF90	ENST00000300996	p.T64T	c.192T>A	Verified	TTC	ACT	GTG	3	T	A
TCGA-23-1022	ZNF90_ENST	ENST00000418063	p.T64T	c.192T>A	Verified	TTC	ACT	GTG	3	T	A
TCGA-13-0887	ZSCAN21	ENST00000292450	p.D198E	c.594T>A	Verified	CGA	GAT	TGC	3	T	A
TCGA-23-1118	ZSWIM3	ENST00000255152	p.L91Q	c.272T>A	Verified	AGA	CTA	TTT	2	T	A
TCGA-24-1844	A2ML1	ENST00000299698	p.V1394A	c.4181T>C	Unverified	AAG	GTT	GAA	2	T	C
TCGA-13-1509	AASDH	ENST00000205214	p.I1079T	c.3236T>C	Verified	CTC	ATT	ATT	2	T	C
TCGA-13-1501	ABCA12	ENST00000272895	p.F1623L	c.4867T>C	Verified	CCA	TTC	AGC	1	T	C
TCGA-13-1501	ABCA12	ENST00000272895	p.F1623L	c.4867T>C	Verified	CCA	TTC	AGC	1	T	C
TCGA-04-1347	ABCA12	ENST00000272895	p.I1732T	c.5195T>C	Verified	GCT	ATA	CTC	2	T	C
TCGA-25-2400	ABCA12	ENST00000272895	p.M1281T	c.3842T>C	Unverified	GGT	ATG	GCA	2	T	C
TCGA-13-0714	ABCA3	ENST00000301732	p.I888T	c.2663T>C	Verified	CTC	ATC	GAG	2	T	C
TCGA-23-1022	ABP1	ENST00000360937	p.I660T	c.1979T>C	Verified	AAC	ATT	GAA	2	T	C
TCGA-24-1563	AC013469_8	ENST00000408934	p.H51H	c.153T>C	Unverified	CTT	CAT	CCA	3	T	C
TCGA-09-2044	ACAT1	ENST00000265838	p.F261F	c.783T>C	Verified	GAT	TTT	AGC	3	T	C
TCGA-13-0884	ACP2	ENST00000256997	p.W204R	c.610T>C	Verified	GTC	TGG	AAT	1	T	C
TCGA-61-2012	ACSL3	ENST00000357430	p.V658A	c.1973T>C	Verified	GAG	GTA	CTT	2	T	C
TCGA-13-0884	ACSM5	ENST00000331849	p.V497A	c.1490T>C	Verified	GCT	GTC	CTG	2	T	C
TCGA-29-1775	ACTB	ENST00000331789	p.Y166H	c.496T>C	Unverified	atc	tac	gag	1	T	C
TCGA-25-2042	ADAM18	ENST00000265707	p.D330D	c.990T>C	Verified	TAT	GAT	GAC	3	T	C
TCGA-23-1122	ADAM22	ENST00000265727	p.I378T	c.1133T>C	Verified	AAT	ATT	GGT	2	T	C
TCGA-25-1313	ADAM22	ENST00000265727	p.V327V	c.981T>C	Verified	GCA	GTT	CAC	3	T	C
TCGA-23-1122	ADAM22_EN	ENST00000398209	p.I378T	c.1133T>C	Verified	AAT	ATT	GGT	2	T	C
TCGA-25-1313	ADAM22_EN	ENST00000398209	p.V327V	c.981T>C	Verified	GCA	GTT	CAC	3	T	C
TCGA-61-1900	ADAM7	ENST00000175238	p.F395L	c.1183T>C	Unverified	CCA	TTT	CCT	1	T	C
TCGA-24-1463	ADAMTS16	NM_139056.1	p.C274R	c.820T>C	Unverified	tct	tgc	tta	1	T	C
TCGA-24-1463	ADAMTS16_	ENST00000274181	p.C274R	c.820T>C	Unverified	TCT	TGC	TTA	1	T	C

TCGA-04-1336	ADAMTS20	ENST00000389420	p.T607T	c.1821T>C	Verified	AAT	ACT	GAT	3	T	C
TCGA-61-1915	ADCY6	ENST00000307885	p.M820T	c.2459T>C	Unverified	AAC	ATG	CTG	2	T	C
TCGA-13-0923	ADNP	ENST00000349014	p.F38F	c.114T>C	Verified	GAT	TTT	AAA	3	T	C
TCGA-25-1326	ADNP2	ENST00000262198	p.I1102T	c.3305T>C	Verified	GCA	ATA	AAA	2	T	C
TCGA-23-1122	ADSL	ENST00000216194	p.I106T	c.317T>C	Verified	ATT	ATT	CAC	2	T	C
TCGA-24-1417	AFF2	ENST00000370460	p.L1050L	c.3150T>C	Unverified	CCC	CTT	CTA	3	T	C
TCGA-61-1740	AFF3	ENST00000356421	p.Y45H	c.133T>C	Unverified	GTC	TAT	GAA	1	T	C
TCGA-29-1691	AGBL5	ENST00000360131	p.Y861H	c.2581T>C	Unverified	TGT	TAC	AGC	1	T	C
TCGA-24-0975	AHI1	ENST00000367800	p.L71P	c.212T>C	Verified	aat	ctt	ccc	2	T	C
TCGA-61-2012	AHNAK	ENST00000378024	p.I5712T	c.17135T>C	Verified	AAG	ATC	AAA	2	T	C
TCGA-13-1510	AICDA	ENST00000229335	p.Y144H	c.430T>C	Unverified	GAT	TAT	TTT	1	T	C
TCGA-09-1674	ALG10	ENST00000266483	p.L220P	c.659T>C	Unverified	AGA	CTT	CCA	2	T	C
TCGA-20-1683	ALK	ENST00000389048	p.S342S	c.1026T>C	Unverified	agc	agt	gag	3	T	C
TCGA-24-1469	ALS2CR8	ENST00000320443	p.N56N	c.168T>C	Verified	AAT	AAT	TCA	3	T	C
TCGA-04-1347	AMPD1	NM_000036.1	p.I506T	c.1517T>C	Verified	cat	atc	act	2	T	C
TCGA-13-1498	AMPH	ENST00000356264	p.D639D	c.1917T>C	Verified	TCT	GAT	GAA	3	T	C
TCGA-30-1857	ANGEL1	ENST00000251089	p.C611R	c.1831T>C	Unverified	TCC	TGT	GAG	1	T	C
TCGA-04-1638	ANK2	ENST00000357077	p.L1121P	c.3362T>C	Unverified	ATT	CTT	AAC	2	T	C
TCGA-13-1510	ANKRD12	ENST00000262126	p.I1120T	c.3359T>C	Unverified	AAA	ATA	AAA	2	T	C
TCGA-04-1342	ANKRD32	ENST00000255667	p.M419T	c.1256T>C	Verified	GTC	ATG	GAG	2	T	C
TCGA-29-1783	ANUBL1	ENST00000344646	p.N217N	c.651T>C	Unverified	GAA	AAT	TCA	3	T	C
TCGA-29-1761	AP4B1	ENST00000369569	p.L499S	c.1496T>C	Unverified	TTG	TTG	TAT	2	T	C
TCGA-29-1761	APBB1IP	ENST00000376236	p.M290T	c.869T>C	Unverified	aaa	atg	aat	2	T	C
TCGA-13-0793	APC	ENST00000457016	p.L852S	c.2555T>C	Unverified	AGT	TTG	GAG	2	T	C
TCGA-29-1777	APC	ENST00000457016	p.I858I	c.2574T>C	Unverified	GGA	ATT	GGT	3	T	C
TCGA-13-1497	APOB	ENST00000233242	p.T151T	c.453T>C	Verified	CCT	ACT	TAC	3	T	C
TCGA-20-0991	APOD	ENST00000343267	p.I172T	c.515T>C	Verified	AAC	ATT	GAT	2	T	C
TCGA-24-1843	ARHGAP36	NM_144967.2	p.S304P	c.910T>C	Unverified	gat	tct	ctg	1	T	C
TCGA-30-1857	ARNT2	ENST00000303329	p.D147D	c.441T>C	Unverified	GCT	GAT	GGA	3	T	C
TCGA-29-1769	ARRB2	ENST00000269260	p.V336A	c.1007T>C	Unverified	GAT	GTC	TCT	2	T	C
TCGA-25-1326	ASH1L	ENST00000392403	p.F1227S	c.3680T>C	Verified	TCT	TTT	GAG	2	T	C
TCGA-23-1120	ASPH	ENST00000379454	p.I294T	c.881T>C	Verified	GTA	ATT	GTA	2	T	C
TCGA-24-1469	ATF6	ENST00000367942	p.I471T	c.1412T>C	Unverified	CTA	ATT	AAC	2	T	C
TCGA-20-1683	ATL1	ENST00000358385	p.F293L	c.877T>C	Unverified	GAA	TTC	ATC	1	T	C

TCGA-04-1338	ATP1A2	ENST00000361216	p.V652A	c.1955T>C	Verified	CAA	GTC	AAC	2	T	C
TCGA-13-0755	ATP2A2	ENST00000539276	p.S72S	c.216T>C	Verified	ATA	TCT	TTT	3	T	C
TCGA-24-1846	ATP2C1	ENST00000359644	p.N841N	c.2523T>C	Unverified	AGT	AAT	AGA	3	T	C
TCGA-23-1122	ATP6AP1	ENST00000369762	p.L437P	c.1310T>C	Unverified	ATG	CTC	TTC	2	T	C
TCGA-29-1781	ATP6V1C2	ENST00000381661	p.L181P	c.542T>C	Unverified	TAT	CTC	GTC	2	T	C
TCGA-29-1781	ATP6V1C2_E	ENST00000272238	p.L181P	c.542T>C	Unverified	TAT	CTC	GTC	2	T	C
TCGA-24-0979	ATP8B2	ENST00000368489	p.L590P	c.1769T>C	Verified	CGA	CTC	TAC	2	T	C
TCGA-24-1844	ATP8B2	ENST00000368489	p.I323T	c.968T>C	Unverified	GTG	ATC	CTG	2	T	C
TCGA-24-1844	ATP8B2_ENS	ENST00000368487	p.I290T	c.869T>C	Unverified	GTG	ATC	CTG	2	T	C
TCGA-61-1906	ATP8B4	ENST00000284509	p.W328R	c.982T>C	Unverified	TTC	TGG	TCA	1	T	C
TCGA-61-1906	ATP8B4	ENST00000284509	p.W328R	c.982T>C	Unverified	TTC	TGG	TCA	1	T	C
TCGA-13-1497	ATRNL1	ENST00000355044	p.S226S	c.678T>C	Verified	GTC	TCT	GTT	3	T	C
TCGA-13-0906	B4GALNT4	ENST00000329962	p.S894P	c.2680T>C	Unverified	CGC	TCC	GCC	1	T	C
TCGA-04-1342	BACE1	ENST00000313005	p.F60L	c.178T>C	Unverified	AGC	TTT	GTG	1	T	C
TCGA-13-1507	BACH2	ENST00000257749	p.C830C	c.2490T>C	Verified	AAG	TGT	ACA	3	T	C
TCGA-13-0807	BAI3	ENST00000370598	p.I805T	c.2414T>C	Verified	GAG	ATA	GAA	2	T	C
TCGA-13-0793	BAZ1A	ENST00000382422	p.F442S	c.1325T>C	Unverified	ctt	ttt	gat	2	T	C
TCGA-24-2289	BBX	ENST00000415149	p.I438T	c.1313T>C	Unverified	ATC	ATT	GAG	2	T	C
TCGA-13-0893	BCAS1	ENST00000395961	p.A279A	c.837T>C	Verified	GCA	GCT	ATA	3	T	C
TCGA-23-1114	BCAS3	ENST00000390652	p.Y504H	c.1510T>C	Unverified	TCC	TAT	AAC	1	T	C
TCGA-24-1470	BCHE	ENST00000264381	p.F472L	c.1414T>C	Verified	gaa	ttt	gtc	1	T	C
TCGA-29-1769	BCLAF1_ENS	ENST00000531224	p.F509L	c.1525T>C	Unverified	CTC	TTT	GAT	1	T	C
TCGA-13-1510	BDKRB1	ENST00000216629	p.F49S	c.146T>C	Unverified	TGT	TTC	TTC	2	T	C
TCGA-13-0906	BMP2K_ENS	ENST00000335016	p.D359D	c.1077T>C	Verified	ACA	GAT	ACC	3	T	C
TCGA-25-1318	BOD1L	ENST00000040738	p.S484S	c.1452T>C	Verified	GAT	TCT	GAT	3	T	C
TCGA-25-1318	BOD1L	ENST00000040738	p.S484S	c.1452T>C	Verified	GAT	TCT	GAT	3	T	C
TCGA-23-2078	BOK	ENST00000318407	p.L92P	c.275T>C	Verified	cag	ctg	cac	2	T	C
TCGA-24-1104	BOLL	ENST00000392296	p.L18S	c.53T>C	Verified	CCT	TTG	AAT	2	T	C
TCGA-13-0760	BPI	ENST00000262865	p.L287P	c.860T>C	Verified	TAC	CTG	GGC	2	T	C
TCGA-29-1693	C10orf12	ENST00000286067	p.D70D	c.210T>C	Unverified	CAG	GAT	TTA	3	T	C
TCGA-29-1763	C10orf129	ENST00000327739	p.L119S	c.356T>C	Unverified	TCT	TTA	TCC	2	T	C
TCGA-29-1763	C10orf129_E	ENST00000341686	p.L274S	c.821T>C	Unverified	TCT	TTA	TCC	2	T	C
TCGA-13-0890	C11orf30	ENST00000334736	p.F263L	c.787T>C	Verified	ACC	TTC	ACT	1	T	C
TCGA-24-1464	C11orf40	ENST00000307616	p.N52N	c.156T>C	Verified	AAA	AAT	ATC	3	T	C

TCGA-13-1510	C11orf80	ENST00000360962	p.H383H	c.1149T>C	Unverified	TAC	CAT	TTG	3	T	C
TCGA-61-2109	C17orf104	ENST00000409122	p.P720P	c.2160T>C	Unverified	TAC	CCT	TAT	3	T	C
TCGA-61-2095	C19orf57	ENST00000346736	p.L147P	c.440T>C	Unverified	CTG	CTA	GTG	2	T	C
TCGA-23-2077	C20orf185	ENST00000375494	p.A62A	c.186T>C	Verified	ACA	GCT	GTG	3	T	C
TCGA-29-1776	C21orf70	ENST00000291634	p.F47L	c.139T>C	Unverified	GCG	TTC	ATC	1	T	C
TCGA-24-0979	C2CD3	ENST00000313663	p.V431A	c.1292T>C	Verified	GAT	GTG	TAT	2	T	C
TCGA-24-2280	C2orf56	ENST00000002125	p.I282T	c.845T>C	Unverified	ATC	ATC	GAG	2	T	C
TCGA-61-1737	C2orf71	ENST00000331664	p.C75C	c.225T>C	Unverified	CTT	TGT	CAG	3	T	C
TCGA-13-0760	C3orf28	ENST00000477892	p.D81D	c.243T>C	Verified	GAA	GAT	GAA	3	T	C
TCGA-04-1338	C3orf30	ENST00000295622	p.F432S	c.1295T>C	Verified	AAC	TTC	CAA	2	T	C
TCGA-29-1785	C5orf48	ENST00000357147	p.L89L	c.267T>C	Unverified	AGG	CTT	TGC	3	T	C
TCGA-29-1785	C5orf48	ENST00000357147	p.L89L	c.267T>C	Unverified	AGG	CTT	TGC	3	T	C
TCGA-23-1122	C6orf70	ENST00000366773	p.M353T	c.1058T>C	Verified	GCT	ATG	GAA	2	T	C
TCGA-13-1496	C7orf16	ENST00000342032	p.F81F	c.243T>C	Verified	GTG	TTT	TCA	3	T	C
TCGA-20-1686	C7orf31	ENST00000283905	p.H390H	c.1170T>C	Unverified	ACC	CAT	AAA	3	T	C
TCGA-23-1022	C7orf62	ENST00000297203	p.Y114Y	c.342T>C	Verified	GAT	TAT	ATT	3	T	C
TCGA-13-0920	C8orf34_EN	ENST00000518698	p.L25L	c.73T>C	Verified	GAA	TTA	ATG	1	T	C
TCGA-09-1665	C9orf3	ENST00000297979	p.L143S	c.428T>C	Verified	TTT	TTG	CTA	2	T	C
TCGA-04-1542	CCDC36	ENST00000296449	p.N579N	c.1737T>C	Verified	AAG	AAT	TTG	3	T	C
TCGA-61-1895	CCDC53	ENST00000240079	p.S115P	c.343T>C	Unverified	GAC	TCT	GGA	1	T	C
TCGA-04-1367	CCDC6	ENST00000263102	p.L217P	c.650T>C	Verified	CGC	CTC	TGG	2	T	C
TCGA-09-2050	CCDC77	ENST00000239830	p.D351D	c.1053T>C	Verified	AAA	GAT	AAG	3	T	C
TCGA-13-1481	CCDC88A	ENST00000263630	p.L189P	c.566T>C	Verified	CCA	CTC	TTG	2	T	C
TCGA-24-2289	CCL27	ENST00000259631	p.L10P	c.29T>C	Unverified	AGC	CTC	CTG	2	T	C
TCGA-04-1338	CCNF_ENST	ENST00000397066	p.F326S	c.977T>C	Verified	GAC	TTC	ACA	2	T	C
TCGA-04-1347	CCPG1	ENST00000310958	p.F566S	c.1697T>C	Verified	GTT	TTT	AGT	2	T	C
TCGA-29-1761	CCR10	ENST00000332438	p.S15S	c.45T>C	Unverified	TAC	TCT	GGG	3	T	C
TCGA-24-1435	CCR3	ENST00000357422	p.F316S	c.947T>C	Verified	ttc	ttc	cac	2	T	C
TCGA-13-1501	CD163L1	ENST00000313599	p.A123A	c.369T>C	Verified	TCA	GCT	CTC	3	T	C
TCGA-13-1501	CD163L1	ENST00000313599	p.A123A	c.369T>C	Verified	TCA	GCT	CTC	3	T	C
TCGA-13-0887	CD180	ENST00000256447	p.H101H	c.303T>C	Verified	CAT	CAT	CAA	3	T	C
TCGA-29-1691	CD248	ENST00000311330	p.V453A	c.1358T>C	Unverified	GTG	GTC	TCT	2	T	C
TCGA-04-1337	CDC2L2	ENST00000357760	p.W109R	c.325T>C	Unverified	AAA	TGG	AAG	1	T	C
TCGA-29-1696	CDKL2	ENST00000429927	p.V157A	c.470T>C	Unverified	gag	gtt	tat	2	T	C

TCGA-29-1696	CDKL2	ENST00000429927	p.V157A	c.470T>C	Unverified	gag	gtt	tat	2	T	C
TCGA-13-0923	CEBPE	ENST00000206513	p.I267T	c.800T>C	Verified	CAG	ATT	CCT	2	T	C
TCGA-10-0930	CELF5	ENST00000292672	p.F272L	c.814T>C	Verified	GCC	TTC	TCA	1	T	C
TCGA-10-0930	CELF5	ENST00000292672	p.F272L	c.814T>C	Verified	GCC	TTC	TCA	1	T	C
TCGA-29-1703	CENPE	ENST00000265148.1	p.L2462L	c.7384T>C	Unverified	cta	ttg	aga	1	T	C
TCGA-13-1509	CENPE	ENST00000265148.1	p.F2608S	c.7823T>C	Verified	ttt	ttt	gat	2	T	C
TCGA-24-2289	CENPJ	ENST00000381884	p.L575S	c.1724T>C	Unverified	GAA	TTA	GAT	2	T	C
TCGA-13-0883	CEP250	ENST00000397527	p.S1187P	c.3559T>C	Verified	TAC	TCT	GCC	1	T	C
TCGA-10-0930	CEP290	ENST00000552810	p.L2158S	c.6473T>C	Verified	ATA	TTG	ACT	2	T	C
TCGA-10-0930	CEP290	ENST00000552810	p.L2158S	c.6473T>C	Verified	ATA	TTG	ACT	2	T	C
TCGA-23-1022	CEP290	ENST00000552810	p.D1972D	c.5916T>C	Verified	GTT	GAT	CAG	3	T	C
TCGA-25-2391	CEPT1	ENST00000357172	p.D180D	c.540T>C	Unverified	CCT	GAT	TGG	3	T	C
TCGA-04-1638	CFH	ENST00000367429	p.Y271H	c.811T>C	Unverified	cct	tat	att	1	T	C
TCGA-13-0760	CGB5	ENST00000301408	p.S101P	c.301T>C	Unverified	GTC	TCC	TAC	1	T	C
TCGA-09-1674	CHD4	ENST00000357008	p.D282D	c.846T>C	Unverified	CCT	GAT	GCC	3	T	C
TCGA-09-1674	CHD4_ENST	ENST00000309577	p.D282D	c.846T>C	Unverified	CCT	GAT	GCC	3	T	C
TCGA-09-1674	CHD4_ENST	ENST00000357008	p.D282D	c.846T>C	Unverified	CCT	GAT	GCC	3	T	C
TCGA-23-1122	CHRNA10	ENST00000250699	p.S360P	c.1078T>C	Verified	CAG	TCC	AGG	1	T	C
TCGA-04-1342	CHRNB1	ENST00000306071	p.L10P	c.29T>C	Unverified	CTG	CTG	GGG	2	T	C
TCGA-29-1785	CLEC4D	ENST00000299665	p.P85P	c.255T>C	Unverified	TGT	CCT	ATT	3	T	C
TCGA-29-1785	CLEC4D	ENST00000299665	p.P85P	c.255T>C	Unverified	TGT	CCT	ATT	3	T	C
TCGA-30-1855	CLIP1	ENST00000358808	p.I798I	c.2394T>C	Unverified	GAG	ATT	GAA	3	T	C
TCGA-20-1686	CNGA2	ENST00000329903	p.V8A	c.23T>C	Unverified	ggt	gtg	aag	2	T	C
TCGA-13-0760	CNOT1	ENST00000317147	p.L781P	c.2342T>C	Verified	GGT	CTT	GGC	2	T	C
TCGA-61-1910	CNST	ENST00000366513	p.S172P	c.514T>C	Unverified	CAG	TCT	CTG	1	T	C
TCGA-25-1326	CNTNAP1	ENST00000264638	p.F503S	c.1508T>C	Unverified	GCA	TTC	CAT	2	T	C
TCGA-29-1775	CNTNAP2	ENST00000361727	p.F366F	c.1098T>C	Unverified	AGC	TTT	TCT	3	T	C
TCGA-23-1031	CNTNAP5	ENST00000431078	p.V859A	c.2576T>C	Verified	CCT	GTG	GAG	2	T	C
TCGA-24-1104	CNTRL	ENST00000238341	p.L2042L	c.6124T>C	Verified	CTG	TTG	GCC	1	T	C
TCGA-24-2289	CNTRL	ENST00000238341	p.I1663T	c.4988T>C	Unverified	CAG	ATT	AGT	2	T	C
TCGA-25-2392	CNTRL	ENST00000238341	p.A480A	c.1440T>C	Verified	GAA	GCT	ATA	3	T	C
TCGA-04-1530	COL14A1	ENST00000297848	p.L835S	c.2504T>C	Unverified	aac	ttg	cgg	2	T	C
TCGA-24-2035	COL14A1	ENST00000297848	p.V742A	c.2225T>C	Verified	cta	gtt	gta	2	T	C
TCGA-61-2012	COL18A1	ENST00000355480	p.L356P	c.1067T>C	Unverified	GCC	CTC	TAC	2	T	C

TCGA-13-0885	COL22A1	ENST00000303045	p.V721A	c.2162T>C	Verified	GGT	GTC	CCA	2	T	C
TCGA-61-1904	COL4A2	ENST00000360467	p.L27P	c.80T>C	Unverified	TTC	CTC	GCC	2	T	C
TCGA-29-1770	COX8C	ENST00000342144	p.Y61H	c.181T>C	Unverified	GCA	TAT	GTG	1	T	C
TCGA-13-1509	CP	ENST00000264613	p.D103D	c.309T>C	Verified	GGA	GAT	AAA	3	T	C
TCGA-29-1777	CTAGE4	ENST00000486333	p.D709D	c.2127T>C	Unverified	GTG	GAT	ACA	3	T	C
TCGA-23-1031	CTCFL	ENST00000243914	p.I591T	c.1772T>C	Verified	acc	atc	ctg	2	T	C
TCGA-24-1469	CTNNBL1	ENST00000361383	p.L444L	c.1330T>C	Verified	gag	ttg	cat	1	T	C
TCGA-13-0913	CUBN	ENST00000377833	p.T517T	c.1551T>C	Verified	TTC	ACT	TTT	3	T	C
TCGA-04-1347	CXorf67	NM_203407.1	p.L469P	c.1406T>C	Verified	agc	ctt	agg	2	T	C
TCGA-29-1698	CYB5R4	ENST00000369681	p.Y326H	c.976T>C	Unverified	CCA	TAT	ACA	1	T	C
TCGA-13-0791	CYP11A1	ENST00000268053	p.H266H	c.798T>C	Verified	GAC	CAT	GTG	3	T	C
TCGA-25-2042	DCBLD2	ENST00000326840	p.F406L	c.1216T>C	Verified	ATA	TTT	CAA	1	T	C
TCGA-04-1356	DCUN1D3	ENST00000324344	p.N116N	c.348T>C	Verified	TGC	AAT	GAC	3	T	C
TCGA-13-1509	DDX18	ENST00000263239	p.L568S	c.1703T>C	Verified	aaa	ttg	att	2	T	C
TCGA-29-1781	DDX58	ENST00000379883	p.S125P	c.373T>C	Unverified	att	tct	gat	1	T	C
TCGA-23-1122	DDX59	ENST00000367349	p.I381T	c.1142T>C	Verified	ACC	ATT	TTG	2	T	C
TCGA-13-0923	DDX60	ENST00000393743	p.L477L	c.1429T>C	Verified	GAT	TTG	CCT	1	T	C
TCGA-13-0923	DDX60_ENS	ENST00000393743	p.L477L	c.1429T>C	Verified	GAT	TTG	CCT	1	T	C
TCGA-13-1499	DHX29	ENST00000251636	p.D432D	c.1296T>C	Verified	GAA	GAT	GGC	3	T	C
TCGA-13-1481	DIAPH2	ENST00000324765	p.L898P	c.2693T>C	Verified	gaa	ctg	gaa	2	T	C
TCGA-24-1422	DISP2	ENST00000267889	p.W946R	c.2836T>C	Unverified	GGT	TGG	TTC	1	T	C
TCGA-04-1542	DLL1	ENST00000366756	p.C204C	c.612T>C	Verified	ACC	TGT	GGG	3	T	C
TCGA-04-1343	DMXL2	ENST00000251076	p.F782S	c.2345T>C	Verified	TGC	TTT	GTT	2	T	C
TCGA-23-1124	DMXL2	ENST00000251076	p.L964P	c.2891T>C	Verified	AAT	CTT	CAA	2	T	C
TCGA-24-2280	DNAH1_ENS	ENST00000420323	p.M1986T	c.5957T>C	Unverified	ATG	ATG	TTC	2	T	C
TCGA-29-1691	DNAH11	ENST00000328843	p.S160P	c.478T>C	Unverified	GTA	TCT	GCT	1	T	C
TCGA-24-2035	DNAH12L	ENST00000311202	p.D80D	c.240T>C	Verified	CCT	GAT	TAT	3	T	C
TCGA-04-1361	DNAH3	ENST00000261383	p.Y1393Y	c.4179T>C	Verified	GGC	TAT	GAG	3	T	C
TCGA-04-1361	DNAH3	ENST00000261383	p.Y1393Y	c.4179T>C	Verified	GGC	TAT	GAG	3	T	C
TCGA-13-1510	DNAH7	ENST00000312428	p.Y2073Y	c.6219T>C	Unverified	TGG	TAT	GAT	3	T	C
TCGA-13-0924	DNMT3A	ENST00000321117	p.F414S	c.1241T>C	Verified	ggc	ttc	cag	2	T	C
TCGA-13-1488	DOCK5	ENST00000276440	p.I81T	c.242T>C	Verified	GTG	ATT	CCT	2	T	C
TCGA-25-1326	DOPEY1	ENST00000349129	p.Y1335H	c.4003T>C	Verified	TGG	TAT	AGC	1	T	C
TCGA-24-2288	DOPEY1	ENST00000349129	p.N1043N	c.3129T>C	Unverified	AGC	AAT	GTG	3	T	C

TCGA-61-1900	DPP10	ENST00000310323	p.S238P	c.712T>C	Unverified	TGG	TCA	CCA	1	T	C
TCGA-13-0885	DPP10	ENST00000310323	p.M355T	c.1064T>C	Verified	GAG	ATG	ACA	2	T	C
TCGA-61-1900	DPP10_ENST	ENST00000410059	p.S245P	c.733T>C	Unverified	TGG	TCA	CCA	1	T	C
TCGA-25-2042	DPP8	ENST00000300141	p.L120L	c.358T>C	Unverified	CTT	TTG	GAT	1	T	C
TCGA-09-1665	DRD1	ENST00000329144	p.I438T	c.1313T>C	Verified	CCC	ATC	ACA	2	T	C
TCGA-29-1770	DSCAM	ENST00000400454	p.S1806S	c.5418T>C	Unverified	GAA	AGT	GCC	3	T	C
TCGA-23-1122	DSCAML1	ENST00000321322	p.I388T	c.1163T>C	Unverified	GGC	ATT	GGC	2	T	C
TCGA-25-2392	DSCAML1	ENST00000321322	p.I1244T	c.3731T>C	Unverified	TAC	ATC	CAG	2	T	C
TCGA-24-0975	DSG3	ENST00000257189	p.I142T	c.425T>C	Unverified	CTT	ATA	CTA	2	T	C
TCGA-24-2280	DSP	ENST00000379802	p.S989S	c.2967T>C	Unverified	CCT	TCT	GGG	3	T	C
TCGA-24-1563	DST	ENST00000370765	p.I108T	c.323T>C	Verified	TAT	ATA	CTT	2	T	C
TCGA-30-1714	DST	ENST00000370765	p.I435T	c.1304T>C	Unverified	GAG	ATA	GCA	2	T	C
TCGA-20-1686	DST_ENST0(	ENST00000244364	p.M2592T	c.7775T>C	Unverified	GAA	ATG	GAC	2	T	C
TCGA-30-1714	DST_ENST0(	ENST00000244364	p.I435T	c.1304T>C	Unverified	GAG	ATA	GCA	2	T	C
TCGA-24-1563	DST_ENST0(	ENST00000370754	p.I612T	c.1835T>C	Verified	TAT	ATA	CTT	2	T	C
TCGA-30-1714	DST_ENST0(	ENST00000370765	p.I435T	c.1304T>C	Unverified	GAG	ATA	GCA	2	T	C
TCGA-20-1686	DST_ENST0(	ENST00000370769	p.M5006T	c.15017T>C	Unverified	GAA	ATG	GAC	2	T	C
TCGA-24-1563	DST_ENST0(	ENST00000370769	p.I434T	c.1301T>C	Verified	TAT	ATA	CTT	2	T	C
TCGA-30-1714	DST_ENST0(	ENST00000370769	p.I761T	c.2282T>C	Unverified	GAG	ATA	GCA	2	T	C
TCGA-61-1733	DUSP16	ENST00000228862	p.Y558Y	c.1674T>C	Unverified	tgg	tat	ttt	3	T	C
TCGA-61-1733	DUSP16	ENST00000228862	p.Y558Y	c.1674T>C	Unverified	tgg	tat	ttt	3	T	C
TCGA-13-0887	DUSP19	ENST00000354221	p.S178P	c.532T>C	Verified	ttt	tct	ttg	1	T	C
TCGA-23-1122	DYNC1H1	ENST00000360184	p.Y3841H	c.11521T>C	Verified	CTA	TAC	GAG	1	T	C
TCGA-25-2042	DYNC1H1	ENST00000360184	p.Y1172Y	c.3516T>C	Verified	ACC	TAT	GTG	3	T	C
TCGA-29-1784	E2F5	ENST00000416274	p.I303T	c.908T>C	Unverified	ATC	ATT	GAT	2	T	C
TCGA-30-1855	EDN3	ENST00000311585	p.Y127Y	c.381T>C	Unverified	CCC	TAT	GGA	3	T	C
TCGA-30-1855	EDN3_ENST(	ENST00000337938	p.Y127Y	c.381T>C	Unverified	CCC	TAT	GGA	3	T	C
TCGA-13-1505	EI24	ENST00000343678	p.I105T	c.314T>C	Verified	ATT	ATC	GGT	2	T	C
TCGA-29-1693	EIF2A	ENST00000460851	p.W390R	c.1168T>C	Unverified	ATT	TGG	CAT	1	T	C
TCGA-29-1693	EIF2A_ENST(	ENST00000487799	p.W365R	c.1093T>C	Unverified	ATT	TGG	CAT	1	T	C
TCGA-23-1122	EIF2C1	ENST00000373204	p.Y418H	c.1252T>C	Verified	cag	tac	ggc	1	T	C
TCGA-29-1691	ELL3	ENST00000319359	p.V73A	c.218T>C	Unverified	ATA	GTG	TCC	2	T	C
TCGA-61-1907	ELOVL6	ENST00000394607	p.W219R	c.655T>C	Unverified	TGC	TGG	ATG	1	T	C
TCGA-23-1123	EMR3	ENST00000253673	p.D261D	c.783T>C	Verified	AAA	GAT	CAA	3	T	C

TCGA-23-1123	EMR3	ENST00000253673	p.D261D	c.783T>C	Verified	AAA	GAT	CAA	3	T	C
TCGA-13-0905	ENPP3	ENST00000357639	p.L651L	c.1951T>C	Verified	CAG	TTG	GGA	1	T	C
TCGA-24-1469	ENPP4	ENST00000371401	p.N165N	c.495T>C	Verified	CTA	AAT	AAT	3	T	C
TCGA-13-1488	ENSG00000[illegible]	ENST00000334042	p.S280S	c.840T>C	Verified	TGC	TCT	TTC	3	T	C
TCGA-13-0884	ENSG00000[illegible]	ENST00000433532	p.M50T	c.149T>C	Verified	CGC	ATG	CAG	2	T	C
TCGA-61-1738	ENSG00000[illegible]	ENST00000430863	p.F80L	c.238T>C	Unverified	ATG	TTC	ATG	1	T	C
TCGA-61-1738	ENSG00000[illegible]	ENST00000430863	p.F80L	c.238T>C	Unverified	ATG	TTC	ATG	1	T	C
TCGA-24-2280	EPB41	ENST00000349460	p.S569P	c.1705T>C	Unverified	ACA	TCT	GAG	1	T	C
TCGA-24-1470	EPHA1	ENST00000275815	p.L710P	c.2129T>C	Verified	GCC	CTG	GAT	2	T	C
TCGA-29-1763	EPS15L1	ENST00000248070	p.L139S	c.416T>C	Unverified	CTC	TTG	CCC	2	T	C
TCGA-13-0884	EPSTI1	ENST00000313640	p.L333L	c.997T>C	Verified	ATA	TTG	ACT	1	T	C
TCGA-13-1496	ERI3	ENST00000372257	p.L149P	c.446T>C	Verified	GTG	CTG	GAC	2	T	C
TCGA-29-1781	ERO1L	ENST00000395686	p.V318A	c.953T>C	Unverified	AAA	GTG	TTA	2	T	C
TCGA-24-1846	ERP44	ENST00000262455	p.F60L	c.178T>C	Unverified	CGT	TTC	AGT	1	T	C
TCGA-13-0924	ESRRB	NM_004452.2	p.F45S	c.134T>C	Verified	ggc	ttt	ggc	2	T	C
TCGA-61-2009	EVI2A	ENST00000247270	p.F39L	c.115T>C	Verified	GCC	TTT	CTG	1	T	C
TCGA-25-2392	EXOC5	ENST00000413566	p.L145L	c.433T>C	Verified	GAA	TTG	AAA	1	T	C
TCGA-25-2401	EXPH5	ENST00000265843	p.F287S	c.860T>C	Verified	ACC	TTT	TCT	2	T	C
TCGA-23-1022	FAM135B	ENST00000395297	p.F1392S	c.4175T>C	Verified	CTC	TTC	CTG	2	T	C
TCGA-13-0760	FAM162A	ENST00000477892	p.D81D	c.243T>C	Verified	gaa	gat	gaa	3	T	C
TCGA-09-2044	FAM199X	ENST00000493442	p.F365L	c.1093T>C	Verified	ctc	ttc	ctt	1	T	C
TCGA-13-0884	FAM24A	ENST00000368894	p.C82R	c.244T>C	Verified	TGC	TGT	GAA	1	T	C
TCGA-61-1740	FAM46B	ENST00000289166	p.C181R	c.541T>C	Unverified	GTG	TGC	ACA	1	T	C
TCGA-25-2042	FAM48B1	XM_093087.4	p.P634P	c.1902T>C	Unverified	aag	cct	gtg	3	T	C
TCGA-24-1422	FAM55D	ENST00000375478	p.F275L	c.823T>C	Verified	CTC	TTT	GAA	1	T	C
TCGA-24-1422	FAM83B	ENST00000306858	p.N410N	c.1230T>C	Verified	ACC	AAT	AAT	3	T	C
TCGA-23-1032	FANCM	ENST00000267430	p.G873G	c.2619T>C	Unverified	CAC	GGT	ATT	3	T	C
TCGA-04-1655	FASTKD3	ENST00000264669	p.F351F	c.1053T>C	Unverified	TTT	TTT	ACA	3	T	C
TCGA-13-0924	FASTKD3	ENST00000264669	p.P368P	c.1104T>C	Verified	GAT	CCT	GAA	3	T	C
TCGA-24-1103	FAT1	ENST00000441802	p.S157P	c.469T>C	Verified	GTT	TCT	TTA	1	T	C
TCGA-09-2050	FAT4	ENST00000330166	p.I1429T	c.4286T>C	Verified	TCT	ATT	GTT	2	T	C
TCGA-13-1505	FAT4	ENST00000330166	p.Y1137Y	c.3411T>C	Verified	AGG	TAT	TCT	3	T	C
TCGA-09-2050	FAT4_ENST[illegible]	ENST00000394329	p.I1429T	c.4286T>C	Verified	TCT	ATT	GTT	2	T	C
TCGA-13-1505	FAT4_ENST[illegible]	ENST00000394329	p.Y1137Y	c.3411T>C	Verified	AGG	TAT	TCT	3	T	C

TCGA-29-1763	FBLN2	ENST00000404922	p.C508R	c.1522T>C	Unverified	ACC	TGC	CAC	1	T	C
TCGA-29-1763	FBLN2_ENST	ENST00000492059	p.C1089R	c.3265T>C	Unverified	ACC	TGC	CAC	1	T	C
TCGA-13-1481	FBXW8	ENST00000309909	p.I125T	c.374T>C	Verified	gca	atc	aat	2	T	C
TCGA-24-1469	FCGBP	ENST00000221347	p.L242P	c.725T>C	Verified	CAG	CTG	CTA	2	T	C
TCGA-10-0930	FCGR1A	ENST00000369168	p.Y245H	c.733T>C	Verified	GAA	TAC	CAA	1	T	C
TCGA-10-0930	FCGR1A	ENST00000369168	p.Y245H	c.733T>C	Verified	GAA	TAC	CAA	1	T	C
TCGA-09-2044	FCHSD1	ENST00000435817	p.V309A	c.926T>C	Verified	cag	gtg	tgt	2	T	C
TCGA-04-1347	FCRL1	ENST00000368176	p.S235S	c.705T>C	Verified	GGC	TCT	CCT	3	T	C
TCGA-04-1342	FCRL5	ENST00000361835	p.F787F	c.2361T>C	Unverified	TTT	TTT	CAT	3	T	C
TCGA-23-1029	FGF10	ENST00000264664	p.N162N	c.486T>C	Unverified	TAC	AAT	ACC	3	T	C
TCGA-09-2049	FGF12	ENST00000454309	p.F49L	c.145T>C	Unverified	GTG	TTC	AGC	1	T	C
TCGA-09-2044	FGF14	ENST00000376131	p.L180L	c.538T>C	Verified	GGA	TTA	AAT	1	T	C
TCGA-25-2393	FILIP1L	ENST00000331335	p.V822A	c.2465T>C	Verified	GCA	GTC	ATC	2	T	C
TCGA-29-1690	FILIP1L	ENST00000331335	p.I764T	c.2291T>C	Unverified	GAG	ATT	GAA	2	T	C
TCGA-29-1690	FILIP1L_ENS	ENST00000354552	p.I764T	c.2291T>C	Unverified	GAG	ATT	GAA	2	T	C
TCGA-30-1855	FLG	ENST00000368799	p.S3915S	c.11745T>C	Unverified	CAG	TCT	TCA	3	T	C
TCGA-61-1738	FLJ43860	ENST00000599122	p.F80L	c.238T>C	Unverified	ATG	TTC	ATG	1	T	C
TCGA-61-1738	FLJ43860	ENST00000599122	p.F80L	c.238T>C	Unverified	ATG	TTC	ATG	1	T	C
TCGA-61-1910	FLRT3	ENST00000378053	p.S284S	c.852T>C	Unverified	CTA	AGT	AAT	3	T	C
TCGA-13-1499	FLT1	ENST00000282397	p.F490L	c.1468T>C	Verified	TCC	TTT	ATC	1	T	C
TCGA-09-0369	FLT3	ENST00000241453	p.V615A	c.1844T>C	Verified	AAG	GTA	CTA	2	T	C
TCGA-09-0369	FLT3	ENST00000241453	p.V615A	c.1844T>C	Verified	AAG	GTA	CTA	2	T	C
TCGA-23-1032	FMNL2	ENST00000288670	p.P559P	c.1677T>C	Unverified	CCC	CCT	CCT	3	T	C
TCGA-29-1784	FN1	ENST00000336916	p.P1212P	c.3636T>C	Unverified	ACC	CCT	ACA	3	T	C
TCGA-29-1784	FN1_ENST0(	ENST00000354785	p.P1212P	c.3636T>C	Unverified	ACC	CCT	ACA	3	T	C
TCGA-13-1510	FNBP4	ENST00000263773	p.L686P	c.2057T>C	Unverified	TCA	CTC	ATG	2	T	C
TCGA-29-1699	FNDC7_ENS	ENST00000370017	p.N475N	c.1425T>C	Unverified	ATT	AAT	GTG	3	T	C
TCGA-30-1857	FOXP3	ENST00000376207	p.V283A	c.848T>C	Unverified	atc	gta	gct	2	T	C
TCGA-23-1022	FREM2	ENST00000280481	p.I1963T	c.5888T>C	Verified	CGG	ATA	GTC	2	T	C
TCGA-61-1915	FRMPD3	XM_042978.4	p.L924P	c.2771T>C	Unverified	tct	ctc	caa	2	T	C
TCGA-61-1915	FRMPD3_EN	ENST00000276185	p.L875P	c.2624T>C	Unverified	TCT	CTC	CAA	2	T	C
TCGA-61-2113	GABBR1	ENST00000377034	p.T369T	c.1107T>C	Verified	GAG	ACT	GAA	3	T	C
TCGA-23-2078	GABBR2	ENST00000259455	p.I879T	c.2636T>C	Verified	CCT	ATA	GAA	2	T	C
TCGA-24-1464	GAN	ENST00000248272	p.I73T	c.218T>C	Verified	AAG	ATT	GAA	2	T	C

TCGA-30-1714	GATM	ENST00000396659	p.V245A	c.734T>C	Unverified	TTT	GTG	ACA	2	T	C
TCGA-29-1776	GBA2	ENST00000378103	p.V201A	c.602T>C	Unverified	ACT	GTG	TAC	2	T	C
TCGA-29-1690	GBF1	ENST00000369983	p.L1426P	c.4277T>C	Unverified	AGT	CTG	AAT	2	T	C
TCGA-23-1123	GC	ENST00000273951	p.M185T	c.554T>C	Verified	TCT	ATG	GTA	2	T	C
TCGA-23-1123	GC	ENST00000273951	p.M185T	c.554T>C	Verified	TCT	ATG	GTA	2	T	C
TCGA-29-1768	GCNT3	ENST00000396065	p.F119S	c.356T>C	Unverified	AAG	TTC	ATA	2	T	C
TCGA-61-1895	GCNT3	ENST00000396065	p.S47S	c.141T>C	Unverified	GAA	TCT	CAA	3	T	C
TCGA-30-1855	GDAP2	ENST00000369443	p.F205F	c.615T>C	Unverified	GTA	TTT	GCT	3	T	C
TCGA-24-1844	GJA3	ENST00000241125	p.L93P	c.278T>C	Unverified	TAC	CTG	GGC	2	T	C
TCGA-13-1501	GNL1	ENST00000376621	p.A233A	c.699T>C	Verified	CCA	GCT	CTT	3	T	C
TCGA-13-1501	GNL1	ENST00000376621	p.A233A	c.699T>C	Verified	CCA	GCT	CTT	3	T	C
TCGA-61-1907	GNL3L	ENST00000336470	p.S563S	c.1689T>C	Unverified	ctg	tct	gat	3	T	C
TCGA-09-2049	GNRHR	ENST00000226413	p.T42T	c.126T>C	Verified	GTT	ACT	TTC	3	T	C
TCGA-24-1843	GOLGA6B	ENST00000421285	p.V351A	c.1052T>C	Unverified	GAG	GTG	CAG	2	T	C
TCGA-04-1347	GOT1L1	ENST00000307599	p.L306P	c.917T>C	Verified	GCT	CTG	CTG	2	T	C
TCGA-29-1711	GPR116	ENST00000265417	p.L1260S	c.3779T>C	Unverified	GGA	TTA	TTC	2	T	C
TCGA-29-1711	GPR116	ENST00000265417	p.L1260S	c.3779T>C	Unverified	GGA	TTA	TTC	2	T	C
TCGA-13-1509	GPR158	ENST00000376351	p.G1017G	c.3051T>C	Verified	CCT	GGT	CCT	3	T	C
TCGA-25-2401	GPR162	ENST00000311268	p.V548A	c.1643T>C	Verified	GCC	GTT	GGA	2	T	C
TCGA-09-2050	GPR97	ENST00000333493	p.S416S	c.1248T>C	Verified	ACC	TCT	CTG	3	T	C
TCGA-29-1761	GPR98	ENST00000405460	p.V2288A	c.6863T>C	Unverified	GTT	GTC	TCA	2	T	C
TCGA-09-2050	GRIN3A	ENST00000361820	p.L727L	c.2179T>C	Verified	CTC	TTG	TTT	1	T	C
TCGA-13-0755	GRM3	ENST00000361669	p.C641C	c.1923T>C	Verified	ATC	TGT	GCA	3	T	C
TCGA-61-1740	GTF2IRD2	ENST00000405086	p.S188S	c.564T>C	Unverified	GAG	AGT	CAG	3	T	C
TCGA-13-0761	GUCY2C	ENST00000261170	p.C685C	c.2055T>C	Verified	AGC	TGT	CGG	3	T	C
TCGA-13-0761	GUCY2C	ENST00000261170	p.C685C	c.2055T>C	Verified	AGC	TGT	CGG	3	T	C
TCGA-24-1616	GULP1	ENST00000359135	p.I220T	c.659T>C	Verified	ATG	ATT	CCA	2	T	C
TCGA-29-1691	HABP2	ENST00000351270	p.I412I	c.1236T>C	Unverified	GAT	ATT	GCA	3	T	C
TCGA-09-1665	HCFC2	ENST00000229330	p.S203S	c.609T>C	Verified	GAT	TCT	GGA	3	T	C
TCGA-13-0904	HDAC6	ENST00000334136	p.L508L	c.1522T>C	Verified	atc	ttg	cgg	1	T	C
TCGA-24-1435	HEATR7B2	ENST00000399564	p.I783T	c.2348T>C	Verified	CTG	ATT	GGT	2	T	C
TCGA-24-2035	HERC1	ENST00000443617	p.P1418P	c.4254T>C	Verified	CCA	CCT	CCT	3	T	C
TCGA-04-1336	HERC5	NM_016323.1	p.T616T	c.1848T>C	Verified	atg	act	gtg	3	T	C
TCGA-04-1652	HIPK3	ENST00000303296	p.V351A	c.1052T>C	Unverified	cat	gta	tca	2	T	C

TCGA-04-1652	HIPK3	ENST00000303296	p.V351A	c.1052T>C	Unverified	cat	gta	tca	2	T	C
TCGA-23-1114	HIVEP1	ENST00000379388	p.A268A	c.804T>C	Unverified	TCT	GCT	GCT	3	T	C
TCGA-61-2109	HIVEP2	ENST00000012134	p.F577L	c.1729T>C	Unverified	GTA	TTC	TAT	1	T	C
TCGA-24-1470	HIVEP3	ENST00000372583	p.I858T	c.2573T>C	Verified	GAG	ATC	CTA	2	T	C
TCGA-29-1695	HNRNPD	ENST00000313899	p.C252C	c.756T>C	Unverified	AAA	TGT	GAA	3	T	C
TCGA-29-1695	HNRPD	ENST00000353341	p.C252C	c.756T>C	Unverified	AAA	TGT	GAA	3	T	C
TCGA-24-2267	HSD3B2	ENST00000369416	p.F275F	c.825T>C	Verified	GAG	TTT	GGC	3	T	C
TCGA-23-1021	HSPA4L	ENST00000508776	p.L41L	c.121T>C	Verified	tca	ttg	gga	1	T	C
TCGA-23-2078	HSPA5	ENST00000324460	p.D578D	c.1734T>C	Unverified	gga	gat	aaa	3	T	C
TCGA-13-0791	HTR1F	ENST00000319595	p.L114P	c.341T>C	Verified	CAT	CTC	TCA	2	T	C
TCGA-24-1104	HTR2A	ENST00000378688	p.F186L	c.556T>C	Verified	CGC	TTC	AAC	1	T	C
TCGA-23-1122	HTR3E	ENST00000335304	p.L99P	c.296T>C	Verified	CAC	CTC	TTG	2	T	C
TCGA-29-1691	HUWE1	XM_497119.1	p.L976L	c.2926T>C	Unverified	ggg	tta	tct	1	T	C
TCGA-29-1691	HUWE1_ENS	ENST00000342160	p.L1113L	c.3337T>C	Unverified	GGG	TTA	TCT	1	T	C
TCGA-29-1761	HYDIN	ENST00000321489	p.T905T	c.2715T>C	Unverified	TCC	ACT	ATT	3	T	C
TCGA-29-1761	HYDIN_ENST	ENST00000316490_v65	p.T905T	c.2715T>C	Unverified	TCC	ACT	ATT	3	T	C
TCGA-29-1761	HYDIN_ENST	ENST00000538248	p.T932T	c.2796T>C	Unverified	TCC	ACT	ATT	3	T	C
TCGA-29-1693	IGSF9	ENST00000361509	p.L320P	c.959T>C	Unverified	GTG	CTC	TGC	2	T	C
TCGA-61-2113	IKZF3	ENST00000346872	p.S497S	c.1491T>C	Unverified	tcg	tct	cac	3	T	C
TCGA-04-1343	IL13RA2	ENST00000371936	p.N194N	c.582T>C	Verified	caa	aat	ata	3	T	C
TCGA-61-1907	IL6ST	ENST00000381298	p.Y334H	c.1000T>C	Unverified	tgg	tat	aaa	1	T	C
TCGA-29-1781	IMPDH2	ENST00000326739	p.Y258Y	c.774T>C	Unverified	aag	tat	agg	3	T	C
TCGA-20-1685	INPPL1	ENST00000298229	p.Y62Y	c.186T>C	Unverified	CTG	TAT	CAG	3	T	C
TCGA-24-0979	INSR	ENST00000302850	p.F1298S	c.3893T>C	Verified	AGC	TTT	CCA	2	T	C
TCGA-04-1342	INTS5	ENST00000330574	p.L462P	c.1385T>C	Verified	GTG	CTA	GGC	2	T	C
TCGA-09-2050	IQGAP2	ENST00000274364	p.I1308T	c.3923T>C	Verified	ATA	ATT	GAT	2	T	C
TCGA-10-0930	IRF2	ENST00000393593	p.W58R	c.172T>C	Verified	AAC	TGG	GCA	1	T	C
TCGA-10-0930	IRF2	ENST00000393593	p.W58R	c.172T>C	Verified	AAC	TGG	GCA	1	T	C
TCGA-13-0904	ISG20L2	ENST00000368219	p.V325A	c.974T>C	Verified	TCT	GTG	GAA	2	T	C
TCGA-30-1856	ITGA8	ENST00000378076	p.F276L	c.826T>C	Unverified	GAG	TTT	ACT	1	T	C
TCGA-24-1417	ITGAD	ENST00000389202	p.I155T	c.464T>C	Unverified	CTG	ATT	GAC	2	T	C
TCGA-61-2008	ITGAL	ENST00000356798	p.L510P	c.1529T>C	Verified	GAG	CTG	CAG	2	T	C
TCGA-61-1907	ITGAM	ENST00000287497	p.F457L	c.1369T>C	Unverified	tac	ttc	ggg	1	T	C
TCGA-29-1781	ITIH1	ENST00000273283	p.I677T	c.2030T>C	Unverified	ATC	ATC	CAC	2	T	C

TCGA-25-2393	JMJD1C	ENST00000402544	p.Y1855H	c.5563T>C	Verified	GTA	TAT	TCA	1	T	C
TCGA-24-1431	KCNC2	ENST00000298972	p.L37P	c.110T>C	Unverified	CTT	CTT	GCC	2	T	C
TCGA-24-1435	KCND3	ENST00000369697	p.V403V	c.1209T>C	Verified	ATT	GTT	TCC	3	T	C
TCGA-13-1497	KCNH1	ENST00000271751	p.L516P	c.1547T>C	Verified	ATG	CTC	AAC	2	T	C
TCGA-61-1900	KCNH5	ENST00000322893	p.L50P	c.149T>C	Unverified	AAA	CTC	TCT	2	T	C
TCGA-24-1463	KCNJ8	ENST00000240662	p.L132P	c.395T>C	Unverified	TTT	CTC	TTC	2	T	C
TCGA-61-1907	KERA	ENST00000266719	p.L254P	c.761T>C	Unverified	GGT	CTC	CCA	2	T	C
TCGA-29-1777	KHDRBS2	ENST00000281156	p.D165D	c.495T>C	Unverified	AAT	GAT	GAA	3	T	C
TCGA-04-1655	KIAA0355	ENST00000299505	p.I164T	c.491T>C	Unverified	GAC	ATT	GAG	2	T	C
TCGA-04-1331	KIAA1109	ENST00000264501	p.S4903P	c.14707T>C	Verified	AAT	TCC	TCT	1	T	C
TCGA-04-1542	KIAA1486	ENST00000272907	p.I36T	c.107T>C	Verified	gtt	att	cag	2	T	C
TCGA-61-2094	KIAA1751	ENST00000270720	p.L450S	c.1349T>C	Unverified	ACG	TTA	GCT	2	T	C
TCGA-24-2267	KIAA1797	ENST00000380249	p.S126S	c.378T>C	Verified	CAG	AGT	ATA	3	T	C
TCGA-04-1530	KIAA2018	ENST00000316407	p.H901H	c.2703T>C	Unverified	GAA	CAT	TTT	3	T	C
TCGA-20-0990	KIF20B	ENST00000260753	p.P495P	c.1485T>C	Verified	GGA	CCT	GTC	3	T	C
TCGA-04-1342	KIT	ENST00000288135	p.I957T	c.2870T>C	Verified	cgg	atc	aat	2	T	C
TCGA-25-1326	KRT28	ENST00000306658	p.L377P	c.1130T>C	Verified	CTC	CTC	GAT	2	T	C
TCGA-61-1910	KRT85	ENST00000257901	p.Y299H	c.895T>C	Unverified	CAG	TAT	GAC	1	T	C
TCGA-13-0893	KRTAP10-11	ENST00000334670	p.S166P	c.496T>C	Verified	TCC	TCT	TCA	1	T	C
TCGA-24-1474	KRTAP27-1	ENST00000382835	p.F151S	c.452T>C	Verified	AAT	TTC	GAA	2	T	C
TCGA-04-1356	KRTAP5-3	ENST00000399685	p.C17C	c.51T>C	Verified	GGC	TGT	GGC	3	T	C
TCGA-29-1775	KRTAP5-6	ENST00000382160	p.S72P	c.214T>C	Unverified	GGC	TCT	TGT	1	T	C
TCGA-25-2398	KRTAP9-3	ENST00000411528	p.C76C	c.228T>C	Verified	TGC	TGT	AGC	3	T	C
TCGA-13-0793	LAMA4	ENST00000389463	p.S964S	c.2892T>C	Verified	GAC	TCT	CTG	3	T	C
TCGA-24-1422	LCLAT1	ENST00000309052	p.I8T	c.23T>C	Verified	GAA	ATT	GTG	2	T	C
TCGA-09-2050	LHCGR	ENST00000294954	p.M582T	c.1745T>C	Verified	TGC	ATG	GCA	2	T	C
TCGA-24-1843	LHX8	ENST00000294638	p.S101P	c.301T>C	Unverified	CTC	TCC	TGC	1	T	C
TCGA-29-1703	LMBRD1	ENST00000370577	p.I390T	c.1169T>C	Unverified	GGC	ATA	TGG	2	T	C
TCGA-24-1846	LOC441504	XM_497127.1	p.A127A	c.381T>C	Unverified	cta	gct	caa	3	T	C
TCGA-23-1022	LOC51059	ENST00000395297	p.F1392S	c.4175T>C	Verified	CTC	TTC	CTG	2	T	C
TCGA-29-1761	LOC652153	ENST00000316490	p.T905T	c.2715T>C	Unverified	TCC	ACT	ATT	3	T	C
TCGA-13-0795	LOXL4	ENST00000260702	p.I678T	c.2033T>C	Verified	gac	att	gat	2	T	C
TCGA-24-1846	LPAL2	ENST00000335388	p.S54S	c.162T>C	Unverified	TCA	TCT	ATG	3	T	C
TCGA-29-1781	LPAR3	ENST00000370611	p.M242T	c.725T>C	Unverified	GTG	ATG	ACT	2	T	C

TCGA-25-1318	LPHN2	ENST00000359929	p.F13L	c.37T>C	Verified	TGG	TTT	ATC	1	T	C
TCGA-25-1318	LPHN2	ENST00000359929	p.F13L	c.37T>C	Verified	TGG	TTT	ATC	1	T	C
TCGA-61-1915	LRCH1	ENST00000389798	p.L612P	c.1835T>C	Unverified	GTC	CTC	TGC	2	T	C
TCGA-61-1915	LRCH1_ENST	ENST00000311191	p.L612P	c.1835T>C	Unverified	GTC	CTC	TGC	2	T	C
TCGA-61-1915	LRIT2	ENST00000372113	p.F521F	c.1563T>C	Unverified	TCC	TTT	AGA	3	T	C
TCGA-09-2056	LRP1B	ENST00000389484	p.W1749R	c.5245T>C	Verified	TAT	TGG	ATC	1	T	C
TCGA-30-1855	LRP2	ENST00000263816	p.Y2601H	c.7801T>C	Unverified	CTC	TAT	GGC	1	T	C
TCGA-04-1331	LRP2	ENST00000263816	p.L2297S	c.6890T>C	Unverified	AAT	TTG	AAA	2	T	C
TCGA-25-2391	LRRC15	ENST00000347624	p.I431T	c.1292T>C	Unverified	GAC	ATC	CTT	2	T	C
TCGA-30-1891	LRRC16A	ENST00000399313	p.F616F	c.1848T>C	Unverified	GGC	TTT	CAG	3	T	C
TCGA-23-1031	LRRIQ1	ENST00000256007	p.L1096P	c.3287T>C	Verified	GAT	CTT	AAA	2	T	C
TCGA-24-2290	LRRIQ3	ENST00000395089	p.L170L	c.510T>C	Verified	CAT	CTT	CCT	3	T	C
TCGA-24-2290	LRRIQ3	ENST00000395089	p.L170L	c.510T>C	Verified	CAT	CTT	CCT	3	T	C
TCGA-29-1764	LRRK1	SU_LRRK1	p.I1825T	c.5474T>C	Unverified	tcc	atc	gcg	2	T	C
TCGA-29-1764	LRRK1_ENST	ENST00000388948	p.I1813T	c.5438T>C	Unverified	TCC	ATC	GCG	2	T	C
TCGA-09-0369	LRRK2	SU_LRRK2	p.I952T	c.2855T>C	Verified	aaa	ata	tta	2	T	C
TCGA-09-0369	LRRK2	SU_LRRK2	p.I952T	c.2855T>C	Verified	aaa	ata	tta	2	T	C
TCGA-61-2012	LRRK2	SU_LRRK2	p.I1192T	c.3575T>C	Verified	gca	att	tta	2	T	C
TCGA-09-0369	LRRK2_ENST	ENST00000298910	p.I952T	c.2855T>C	Verified	AAA	ATA	TTA	2	T	C
TCGA-09-0369	LRRK2_ENST	ENST00000298910	p.I952T	c.2855T>C	Verified	AAA	ATA	TTA	2	T	C
TCGA-61-2012	LRRK2_ENST	ENST00000298910	p.I1192T	c.3575T>C	Verified	GCA	ATT	TTA	2	T	C
TCGA-23-1114	MAG	ENST00000361922	p.V469A	c.1406T>C	Unverified	CTC	GTG	CTC	2	T	C
TCGA-23-1114	MAG_ENST(	ENST00000392213	p.V469A	c.1406T>C	Unverified	CTC	GTG	CTC	2	T	C
TCGA-24-1847	MAGEB4	ENST00000378982	p.N298N	c.894T>C	Unverified	CCC	AAT	AAC	3	T	C
TCGA-29-1783	MAGEB6	ENST00000379034	p.V342A	c.1025T>C	Unverified	tac	gtg	gtt	2	T	C
TCGA-29-1770	MAMDC2	ENST00000377182	p.V6A	c.17T>C	Unverified	GGC	GTC	CTC	2	T	C
TCGA-24-2289	MAN2A1	ENST00000261483	p.V318V	c.954T>C	Unverified	GCA	GTT	AAA	3	T	C
TCGA-24-2289	MAP2	ENST00000360351	p.V1476A	c.4427T>C	Unverified	GAA	GTT	CAG	2	T	C
TCGA-61-1738	MAP3K1	XM_042066	p.C547R	c.1639T>C	Unverified	ctg	tgc	aaa	1	T	C
TCGA-61-1738	MAP3K1	XM_042066	p.C547R	c.1639T>C	Unverified	ctg	tgc	aaa	1	T	C
TCGA-61-1738	MAP3K1_EN	ENST00000399503	p.C710R	c.2128T>C	Unverified	CTG	TGC	AAA	1	T	C
TCGA-61-1738	MAP3K1_EN	ENST00000399503	p.C710R	c.2128T>C	Unverified	CTG	TGC	AAA	1	T	C
TCGA-29-1699	MAP3K10	ENST00000253055	p.L391P	c.1172T>C	Unverified	GAC	CTT	CGG	2	T	C
TCGA-23-1111	MAP4	ENST00000360240	p.V192A	c.575T>C	Unverified	TCT	GTG	GAA	2	T	C

TCGA-23-1111	MAP4_ENST	ENST00000395734_v68	p.V192A	c.575T>C	Unverified	TCT	GTG	GAA	2	T	C
TCGA-13-0916	MAS1L	ENST00000377127	p.L131L	c.391T>C	Verified	TTC	TTA	CAG	1	T	C
TCGA-30-1855	MCART1	ENST00000377716	p.Y173Y	c.519T>C	Unverified	TAT	TAT	CGA	3	T	C
TCGA-13-1488	MCOLN2	ENST00000370608	p.L188P	c.563T>C	Verified	GAG	CTC	GAT	2	T	C
TCGA-61-2102	MCOLN2	ENST00000370608	p.F466S	c.1397T>C	Verified	ATG	TTT	GCA	2	T	C
TCGA-61-2008	MCOLN2	ENST00000370608	p.D463D	c.1389T>C	Verified	GGT	GAT	GAC	3	T	C
TCGA-25-2392	MDN1	ENST00000369393	p.L4459P	c.13376T>C	Verified	GCA	CTA	GTT	2	T	C
TCGA-24-1469	MDN1	ENST00000369393	p.G4842G	c.14526T>C	Unverified	GAT	GGT	GGA	3	T	C
TCGA-13-0905	MEP1A	ENST00000230588	p.F37F	c.111T>C	Verified	GAT	TTT	GGT	3	T	C
TCGA-24-2288	MFN1	ENST00000263969	p.L484P	c.1451T>C	Unverified	TTA	CTT	CCA	2	T	C
TCGA-29-1763	MKNK1	ENST00000371946	p.L218L	c.652T>C	Unverified	ata	ttg	tgt	1	T	C
TCGA-24-0979	MLH3	NM_014381.1	p.F491S	c.1472T>C	Verified	tct	ttc	ctg	2	T	C
TCGA-61-1998	MLL4	ENST00000222270	p.H332H	c.996T>C	Unverified	CAA	CAT	GAG	3	T	C
TCGA-24-1474	MLXIP	ENST00000319080	p.F185L	c.553T>C	Unverified	CAC	TTT	GTG	1	T	C
TCGA-09-2049	MMAA	ENST00000281317	p.I275T	c.824T>C	Verified	GGT	ATC	AAA	2	T	C
TCGA-13-0903	MMP8	ENST00000236826	p.L291P	c.872T>C	Verified	aca	ctc	cgt	2	T	C
TCGA-24-1104	MMRN1	ENST00000394980	p.L9P	c.26T>C	Verified	GTC	CTT	CTT	2	T	C
TCGA-29-1784	MOBKL1A	ENST00000309395	p.M119T	c.356T>C	Unverified	ttg	atg	act	2	T	C
TCGA-04-1331	MRPL11	ENST00000310999	p.L91P	c.272T>C	Unverified	TTC	CTG	AAG	2	T	C
TCGA-23-1117	MS4A2	ENST00000278888	p.M238T	c.713T>C	Verified	GAA	ATG	TCT	2	T	C
TCGA-23-1117	MS4A2	ENST00000278888	p.M238T	c.713T>C	Verified	GAA	ATG	TCT	2	T	C
TCGA-29-1695	MT-ATP6	ENST00000361899	p.L137L	c.411T>C	Unverified	CCC	CTT	ATC	3	T	C
TCGA-29-1695	MT-ATP6_EI	ENST00000361899_v68	p.L137L	c.411T>C	Unverified	CCC	CTT	ATC	3	T	C
TCGA-24-2290	MTBP	ENST00000305949	p.C853C	c.2559T>C	Verified	gaa	tgt	ttc	3	T	C
TCGA-24-2290	MTBP	ENST00000305949	p.C853C	c.2559T>C	Verified	gaa	tgt	ttc	3	T	C
TCGA-20-1683	MT-CO1	ENST00000361624	p.F8S	c.23T>C	Unverified	CTA	TTC	TCT	2	T	C
TCGA-20-1683	MT-CO1_EN	ENST00000361624_v68	p.F8S	c.23T>C	Unverified	CTA	TTC	TCT	2	T	C
TCGA-04-1651	MT-CO3	ENST00000362079	p.I167I	c.501T>C	Unverified	ACA	ATT	TTA	3	T	C
TCGA-04-1651	MT-CO3	ENST00000362079	p.I167I	c.501T>C	Unverified	ACA	ATT	TTA	3	T	C
TCGA-04-1651	MT-CO3_EN	ENST00000362079_v68	p.I167I	c.501T>C	Unverified	ACA	ATT	TTA	3	T	C
TCGA-04-1651	MT-CO3_EN	ENST00000362079_v68	p.I167I	c.501T>C	Unverified	ACA	ATT	TTA	3	T	C
TCGA-04-1651	MT-CYB	ENST00000361789	p.T7T	c.21T>C	Unverified	AAA	ACT	AAC	3	T	C
TCGA-04-1651	MT-CYB	ENST00000361789	p.T7T	c.21T>C	Unverified	AAA	ACT	AAC	3	T	C
TCGA-61-1907	MT-CYB	ENST00000361789	p.I188I	c.564T>C	Unverified	TTC	ATT	ATT	3	T	C

TCGA-04-1651	MT-CYB_EN:	ENST00000361789_v68	p.T7T	c.21T>C	Unverified	AAA	ACT	AAC	3	T	C
TCGA-04-1651	MT-CYB_EN:	ENST00000361789_v68	p.T7T	c.21T>C	Unverified	AAA	ACT	AAC	3	T	C
TCGA-61-1907	MT-CYB_EN:	ENST00000361789_v68	p.I188I	c.564T>C	Unverified	TTC	ATT	ATT	3	T	C
TCGA-04-1338	MTERFD1	ENST00000287025	p.V391A	c.1172T>C	Verified	CTA	GTA	TCT	2	T	C
TCGA-29-1698	MT-ND3	ENST00000361227	p.L5S	c.14T>C	Unverified	CTC	TTA	TTA	2	T	C
TCGA-24-1431	MTR	ENST00000366577	p.L568L	c.1702T>C	Verified	ACA	TTA	CCT	1	T	C
TCGA-13-1501	MTUS2_ENS	ENST00000431530	p.L91P	c.272T>C	Verified	CAA	CTT	CAG	2	T	C
TCGA-13-1501	MTUS2_ENS	ENST00000431530	p.L91P	c.272T>C	Verified	CAA	CTT	CAG	2	T	C
TCGA-04-1542	MYBL1	ENST00000522677	p.M307T	c.920T>C	Verified	aac	atg	tct	2	T	C
TCGA-09-1665	MYBL2	ENST00000217026	p.L631P	c.1892T>C	Unverified	TTG	CTC	AAC	2	T	C
TCGA-23-1124	MYH11	ENST00000338282	p.I786T	c.2357T>C	Verified	AAG	ATC	ACC	2	T	C
TCGA-25-2401	MYL4	ENST00000393450	p.L111P	c.332T>C	Verified	ATG	CTG	GAC	2	T	C
TCGA-13-0714	MYO5A	ENST00000399231	p.S1797S	c.5391T>C	Verified	GTC	TCT	GTG	3	T	C
TCGA-61-2012	NAALADL1	ENST00000358658	p.A738A	c.2214T>C	Unverified	GTG	GCT	GAC	3	T	C
TCGA-13-1497	NALCN	ENST00000251127	p.L1576P	c.4727T>C	Verified	TGG	CTC	AAG	2	T	C
TCGA-23-1029	NARG2	ENST00000261520	p.N729N	c.2187T>C	Unverified	GGA	AAT	TTT	3	T	C
TCGA-13-1488	NAT2	ENST00000286479	p.L225S	c.674T>C	Verified	TCC	TTG	CAG	2	T	C
TCGA-13-1408	NAV3	NM_014903.3	p.S1075P	c.3223T>C	Verified	ggg	tca	tct	1	T	C
TCGA-61-2012	NBEA	ENST00000310336	p.I1735I	c.5205T>C	Verified	AGC	ATT	AGT	3	T	C
TCGA-24-0979	NCKAP1L	ENST00000293373	p.F375L	c.1123T>C	Verified	TCC	TTC	ATT	1	T	C
TCGA-24-1464	NEB	ENST00000409198	p.D4895D	c.14685T>C	Verified	ACA	GAT	ACA	3	T	C
TCGA-61-1910	NEDD4L	ENST00000382850	p.F128F	c.384T>C	Unverified	ACA	TTT	AAG	3	T	C
TCGA-61-1910	NEDD4L_EN	ENST00000400345	p.F128F	c.384T>C	Unverified	ACA	TTT	AAG	3	T	C
TCGA-61-1913	NEK10	SU_NEK10	p.L459L	c.1377T>C	Unverified	aga	ctt	ttc	3	T	C
TCGA-61-1913	NEK10_ENS	ENST00000341435	p.L459L	c.1377T>C	Unverified	AGA	CTT	TTC	3	T	C
TCGA-61-1913	NEK10_ENS	ENST00000429845	p.L459L	c.1377T>C	Unverified	AGA	CTT	TTC	3	T	C
TCGA-13-0762	NFATC2	ENST00000396009	p.I518T	c.1553T>C	Verified	GGG	ATC	TTG	2	T	C
TCGA-13-0762	NFATC2	ENST00000396009	p.I518T	c.1553T>C	Verified	GGG	ATC	TTG	2	T	C
TCGA-23-1110	NHEDC1	ENST00000296422	p.P121P	c.363T>C	Unverified	GTG	CCT	CCA	3	T	C
TCGA-24-0979	NHLH1	ENST00000302101	p.M8T	c.23T>C	Verified	ACC	ATG	GAG	2	T	C
TCGA-29-1701	NHLRC1	ENST00000340650	p.H104H	c.312T>C	Unverified	GCC	CAT	CGC	3	T	C
TCGA-29-1691	NLGN3	ENST00000374051	p.F416S	c.1247T>C	Unverified	AAT	TTT	GTG	2	T	C
TCGA-29-1691	NLGN3_ENS	ENST00000358741	p.F436S	c.1307T>C	Unverified	AAT	TTT	GTG	2	T	C
TCGA-29-1777	NLRP13	ENST00000342929	p.Y458Y	c.1374T>C	Unverified	GCC	TAT	TTT	3	T	C

TCGA-61-1914	NMNAT2	ENST00000287713	p.C165R	c.493T>C	Unverified	tgc	tgt	gtc	1	T	C
TCGA-25-2400	NONO	ENST00000276079	p.F213L	c.637T>C	Verified	tcc	ttc	ctg	1	T	C
TCGA-61-1740	NOS2	ENST00000313735	p.S758P	c.2272T>C	Unverified	CTC	TCC	TGT	1	T	C
TCGA-09-2050	NOTCH3	ENST00000263388	p.V356A	c.1067T>C	Unverified	TGT	GTC	AGC	2	T	C
TCGA-36-1577	NOVA1	ENST00000539517	p.V221A	c.662T>C	Unverified	gtt	gtc	act	2	T	C
TCGA-13-1488	NPFFR2	ENST00000308744	p.C455R	c.1363T>C	Verified	CTC	TGC	CAA	1	T	C
TCGA-09-2044	NPPA	ENST00000376480	p.Y151H	c.451T>C	Verified	CGG	TAC	TGA	1	T	C
TCGA-29-1762	NPR1	ENST00000368680	p.Y614H	c.1840T>C	Unverified	gag	tac	tgt	1	T	C
TCGA-29-1784	NR1D2	ENST00000312521	p.L318P	c.953T>C	Unverified	CAT	CTC	AAT	2	T	C
TCGA-29-1691	NR5A2	ENST00000367362	p.S205S	c.615T>C	Unverified	ccc	tct	gac	3	T	C
TCGA-24-0979	NSA2	ENST00000296802	p.V135V	c.405T>C	Verified	AAA	GTT	ATT	3	T	C
TCGA-24-1103	NTRK1	ENST00000524377	p.C152C	c.456T>C	Verified	CAC	TGT	TCT	3	T	C
TCGA-29-1702	NUF2	ENST00000367900	p.L334L	c.1000T>C	Unverified	AAA	TTG	AAG	1	T	C
TCGA-29-1702	NUF2_ENST(	ENST00000271452	p.L334L	c.1000T>C	Unverified	AAA	TTG	AAG	1	T	C
TCGA-13-1488	O5AK3_HUN	ENST00000326876	p.F101L	c.301T>C	Verified	CAA	TTC	TTA	1	T	C
TCGA-24-2289	OCA2	ENST00000354638	p.N279N	c.837T>C	Unverified	TTA	AAT	CCG	3	T	C
TCGA-13-0762	ODF1	ENST00000285402	p.I125T	c.374T>C	Verified	AAC	ATT	TTA	2	T	C
TCGA-13-0762	ODF1	ENST00000285402	p.I125T	c.374T>C	Verified	AAC	ATT	TTA	2	T	C
TCGA-24-2289	OPHN1	ENST00000355520	p.F87S	c.260T>C	Unverified	tcc	ttc	aag	2	T	C
TCGA-13-1509	OPRK1	ENST00000265572	p.C345R	c.1033T>C	Verified	TTC	TGC	TTT	1	T	C
TCGA-29-1698	OR1L4	ENST00000259466	p.F69S	c.206T>C	Unverified	TCT	TTC	ATG	2	T	C
TCGA-61-2113	OR1L8	ENST00000304865	p.V143A	c.428T>C	Verified	TGT	GTC	CTG	2	T	C
TCGA-23-2077	OR2A7	ENST00000493325	p.S242P	c.724T>C	Verified	TTC	TCC	CAC	1	T	C
TCGA-24-1846	OR2B11	ENST00000318749	p.S155S	c.465T>C	Unverified	CTC	AGT	GGC	3	T	C
TCGA-61-1733	OR2L3	ENST00000359959	p.S152P	c.454T>C	Unverified	GGC	TCG	ATC	1	T	C
TCGA-61-1733	OR2L3	ENST00000359959	p.S152P	c.454T>C	Unverified	GGC	TCG	ATC	1	T	C
TCGA-04-1343	OR2T6	ENST00000355728	p.L55P	c.164T>C	Verified	CAT	CTC	CAC	2	T	C
TCGA-29-1784	OR2W5	ENST00000366492	p.F157L	c.469T>C	Unverified	TCC	TTC	ATC	1	T	C
TCGA-13-1497	OR4C12	ENST00000335238	p.I67T	c.200T>C	Verified	TTG	ATA	GAC	2	T	C
TCGA-09-1674	OR4C13	ENST00000555099	p.Y63H	c.187T>C	Unverified	GCC	TAT	CTC	1	T	C
TCGA-24-1435	OR4D10	ENST00000313265	p.I74T	c.221T>C	Verified	TCC	ATC	ACA	2	T	C
TCGA-24-2262	OR4D10	ENST00000313265	p.F100F	c.300T>C	Verified	ATG	TTT	CTA	3	T	C
TCGA-24-1616	OR4K1	ENST00000285600	p.F26L	c.76T>C	Verified	CTT	TTC	TTT	1	T	C
TCGA-24-2288	OR4K13	ENST00000315693	p.Y275H	c.823T>C	Unverified	TTT	TAC	ACA	1	T	C

TCGA-24-1844	OR4K17	ENST00000315543	p.V277A	c.830T>C	Unverified	ACA	GTG	GTG	2	T	C
TCGA-29-1761	OR4K2	ENST00000298642	p.F27S	c.80T>C	Unverified	TTT	TTC	TTT	2	T	C
TCGA-25-2393	OR5AS1	ENST00000313555	p.P183P	c.549T>C	Verified	CCA	CCT	CTT	3	T	C
TCGA-13-1489	OR5B21	ENST00000360374	p.G137G	c.411T>C	Verified	GCA	GGT	GTG	3	T	C
TCGA-04-1342	OR5D14	ENST00000335605	p.Y134Y	c.402T>C	Verified	CTT	TAT	ACA	3	T	C
TCGA-13-0885	OR5T1	ENST00000313033	p.Y213H	c.637T>C	Verified	TTC	TAC	TTT	1	T	C
TCGA-24-2280	OR6A2	ENST00000332601	p.L34P	c.101T>C	Unverified	CTG	CTG	GCC	2	T	C
TCGA-29-1691	OR6N1	ENST00000335094	p.T38T	c.114T>C	Unverified	ATG	ACT	GTG	3	T	C
TCGA-13-0762	OR8B12	ENST00000306842	p.V204A	c.611T>C	Verified	GCT	GTT	GAC	2	T	C
TCGA-13-0762	OR8B12	ENST00000306842	p.V204A	c.611T>C	Verified	GCT	GTT	GAC	2	T	C
TCGA-24-1423	OSMR	ENST00000274276	p.M949T	c.2846T>C	Verified	CAA	ATG	GCA	2	T	C
TCGA-13-0791	OVCH1	ENST00000318184	p.V102A	c.305T>C	Verified	ACT	GTG	ACT	2	T	C
TCGA-29-1764	OVCH1	ENST00000318184	p.L109P	c.326T>C	Unverified	AGC	CTC	TTT	2	T	C
TCGA-13-0893	P2RY13	ENST00000325602	p.M291T	c.872T>C	Verified	AGA	CTG	CAA	2	T	C
TCGA-20-1683	PACS1	ENST00000320580	p.S242S	c.726T>C	Unverified	GTC	TCT	GTG	3	T	C
TCGA-25-1313	PAK3	ENST00000446737	p.S509P	c.1525T>C	Verified	gga	tct	gcc	1	T	C
TCGA-09-1674	PALB2	ENST00000261584	p.S500P	c.1498T>C	Unverified	TTG	TCT	AGG	1	T	C
TCGA-30-1891	PAOX	ENST00000278060	p.F208S	c.623T>C	Verified	CCC	TTT	GGG	2	T	C
TCGA-30-1891	PAOX_ENST(	ENST00000357296	p.F208S	c.623T>C	Verified	CCC	TTT	GGG	2	T	C
TCGA-13-0885	PARG	ENST00000402038	p.S654S	c.1962T>C	Verified	TAT	TCT	AGT	3	T	C
TCGA-23-2078	PCDHA3	ENST00000378129	p.C759R	c.2275T>C	Unverified	GTG	TGC	TCT	1	T	C
TCGA-24-2024	PCDHB11	ENST00000354757	p.L140P	c.419T>C	Verified	CTC	CTA	GAA	2	T	C
TCGA-29-1766	PCDHB4	ENST00000194152	p.L490P	c.1469T>C	Unverified	CTG	CTG	CCG	2	T	C
TCGA-24-2267	PCDHB7	ENST00000231137	p.L301P	c.902T>C	Verified	AGT	CTT	CAT	2	T	C
TCGA-09-2049	PCOLCE2	ENST00000295992	p.G289G	c.867T>C	Verified	ACG	GGT	TTA	3	T	C
TCGA-24-1845	PDE11A	ENST00000286063	p.P516P	c.1548T>C	Unverified	GTC	CCT	ATT	3	T	C
TCGA-24-1845	PDE11A_EN:	ENST00000358450	p.P266P	c.798T>C	Unverified	GTC	CCT	ATT	3	T	C
TCGA-04-1361	PDE4C	ENST00000355502	p.L457P	c.1370T>C	Verified	GTG	CTG	GAG	2	T	C
TCGA-04-1361	PDE4C	ENST00000355502	p.L457P	c.1370T>C	Verified	GTG	CTG	GAG	2	T	C
TCGA-13-0761	PDGFRA	ENST00000257290	p.F238L	c.712T>C	Unverified	GTT	TTT	AAC	1	T	C
TCGA-13-0761	PDGFRA	ENST00000257290	p.F238L	c.712T>C	Unverified	GTT	TTT	AAC	1	T	C
TCGA-13-0762	PDGFRA	ENST00000257290	p.F238L	c.712T>C	Unverified	GTT	TTT	AAC	1	T	C
TCGA-13-0762	PDGFRA	ENST00000257290	p.F238L	c.712T>C	Unverified	GTT	TTT	AAC	1	T	C
TCGA-29-1784	PER1	ENST00000317276	p.V1184A	c.3551T>C	Unverified	GCT	GTG	CAC	2	T	C

TCGA-29-1781	PEX12	ENST00000225873	p.L65P	c.194T>C	Unverified	ACT	CTG	CTA	2	T	C
TCGA-30-1855	PGBD2	ENST00000355360	p.V239A	c.716T>C	Unverified	TAT	GTC	ATT	2	T	C
TCGA-30-1855	PGBD2_ENS	ENST00000329291	p.V490A	c.1469T>C	Unverified	TAT	GTC	ATT	2	T	C
TCGA-29-1711	PGLYRP3	ENST00000290722	p.L15P	c.44T>C	Unverified	GGT	CTC	CAG	2	T	C
TCGA-29-1711	PGLYRP3	ENST00000290722	p.L15P	c.44T>C	Unverified	GGT	CTC	CAG	2	T	C
TCGA-13-0906	PHACTR1	ENST00000332995	p.L72P	c.215T>C	Unverified	TAC	CTC	GCA	2	T	C
TCGA-20-1685	PHIP	ENST00000275034	p.V771A	c.2312T>C	Unverified	ACT	GTC	TCA	2	T	C
TCGA-13-1509	PIGM	ENST00000368090	p.F259F	c.777T>C	Verified	tac	ttt	tat	3	T	C
TCGA-29-1693	PIK3C3	ENST00000262039	p.M787T	c.2360T>C	Unverified	gga	atg	ggg	2	T	C
TCGA-61-1899	PIK3CB	ENST00000477593	p.F876L	c.2626T>C	Unverified	gcc	ttc	aac	1	T	C
TCGA-04-1331	PIP5K1C	ENST00000335312	p.?	c.1345+2T>C	Unverified	TCC	TCC	CTG	1	T	C
TCGA-24-2267	PISD	ENST00000382151	p.N357N	c.1071T>C	Verified	TTC	AAT	TTC	3	T	C
TCGA-23-1120	PKD1L1	ENST00000289672	p.V1371A	c.4112T>C	Verified	TCT	GTT	CTT	2	T	C
TCGA-13-1496	PKHD1	ENST00000371117	p.Y3631Y	c.10893T>C	Verified	CAT	TAT	AGA	3	T	C
TCGA-24-2280	PKP4	ENST00000389759	p.L442S	c.1325T>C	Unverified	GCG	TTG	TAT	2	T	C
TCGA-29-1768	PLAA	ENST00000380086	p.V553A	c.1658T>C	Unverified	ATT	GTC	TTT	2	T	C
TCGA-20-1687	PLB1	ENST00000327757	p.W1127R	c.3379T>C	Unverified	TCT	TGG	AGC	1	T	C
TCGA-20-1687	PLB1_ENST(	ENST00000422425	p.W1116R	c.3346T>C	Unverified	TCT	TGG	AGC	1	T	C
TCGA-24-1604	PLEK	ENST00000234313	p.F224L	c.670T>C	Verified	GGG	TTC	TTC	1	T	C
TCGA-61-1740	PLVAP	ENST00000252590	p.L278P	c.833T>C	Unverified	AGC	CTC	ATG	2	T	C
TCGA-24-0979	POLK	ENST00000241436	p.N98N	c.294T>C	Verified	CGA	AAT	TTG	3	T	C
TCGA-30-1855	PON3	ENST00000265627	p.T349T	c.1047T>C	Unverified	AAA	ACT	CTG	3	T	C
TCGA-29-1777	POSTN	ENST00000379747	p.C44C	c.132T>C	Unverified	GTC	TGT	GCC	3	T	C
TCGA-61-2109	POT1	ENST00000357628	p.I78T	c.233T>C	Unverified	GAT	ATT	GTT	2	T	C
TCGA-29-1693	PPEF2	ENST00000286719	p.V274A	c.821T>C	Unverified	gat	gtt	ttc	2	T	C
TCGA-13-1509	PPM1F	ENST00000263212	p.F242S	c.725T>C	Verified	atg	ttt	ctc	2	T	C
TCGA-24-1469	PPP1R12A	ENST00000261207	p.I276T	c.827T>C	Verified	gac	att	tta	2	T	C
TCGA-24-1844	PPP1R3A	NM_002711.2	p.F1100L	c.3298T>C	Unverified	aca	ttc	tac	1	T	C
TCGA-29-1784	PPP1R3A	NM_002711.2	p.D345D	c.1035T>C	Unverified	aca	gat	cca	3	T	C
TCGA-29-1705	PPP1R3F	ENST00000055335	p.N656N	c.1968T>C	Unverified	atg	aat	agg	3	T	C
TCGA-13-0904	PPP1R7	ENST00000407025	p.Y258H	c.772T>C	Verified	ctg	tac	ctt	1	T	C
TCGA-24-2035	PPP1R7	ENST00000407025	p.S177S	c.531T>C	Verified	atc	agt	aaa	3	T	C
TCGA-04-1649	PPP2R2A_EI	ENST00000380737	p.F77L	c.229T>C	Unverified	ACC	TTC	CAG	1	T	C
TCGA-29-1691	PRG2_ENST(	ENST00000311862	p.V74A	c.221T>C	Unverified	GCT	GTT	GAG	2	T	C

TCGA-24-2289	PRKD3	ENST00000379066	p.F307S	c.920T>C	Unverified	AAA	TTC	AAC	2	T	C
TCGA-24-2289	PRKD3_ENST	ENST00000379066	p.F307S	c.920T>C	Unverified	AAA	TTC	AAC	2	T	C
TCGA-24-1563	PRR21	ENST00000408934	p.H51H	c.153T>C	Unverified	CTT	CAT	CCA	3	T	C
TCGA-04-1638	PSME4	NM_014614.1	p.D931D	c.2793T>C	Unverified	cat	gat	tgg	3	T	C
TCGA-04-1638	PSME4_ENS	ENST00000404125	p.D1045D	c.3135T>C	Unverified	CAT	GAT	TGG	3	T	C
TCGA-13-0760	PTER	ENST00000378000	p.L14P	c.41T>C	Verified	GGC	CTT	GTA	2	T	C
TCGA-13-0890	PTH	ENST00000282091	p.T110T	c.330T>C	Verified	TTA	ACT	AAA	3	T	C
TCGA-13-0920	PTPN13	ENST00000436978	p.I2373T	c.7118T>C	Verified	CAT	ATT	TCT	2	T	C
TCGA-13-0904	PTPN18	ENST00000175756	p.Y144H	c.430T>C	Verified	cgg	tac	tgg	1	T	C
TCGA-04-1652	PTPN21	ENST00000328736	p.V844A	c.2531T>C	Unverified	CGA	GTA	GAT	2	T	C
TCGA-04-1652	PTPN21	ENST00000328736	p.V844A	c.2531T>C	Unverified	CGA	GTA	GAT	2	T	C
TCGA-29-1693	PTPRD	ENST00000381196	p.D1064D	c.3192T>C	Unverified	GTG	GAT	GGC	3	T	C
TCGA-29-1693	PTPRD_ENST	ENST00000355233	p.D653D	c.1959T>C	Unverified	GTG	GAT	GGC	3	T	C
TCGA-29-1693	PTPRD_ENST	ENST00000381196	p.D1064D	c.3192T>C	Unverified	GTG	GAT	GGC	3	T	C
TCGA-23-1809	PTPRH	ENST00000376350	p.M1002T	c.3005T>C	Unverified	AGG	ATG	CTT	2	T	C
TCGA-24-0979	PXDNL_ENST	ENST00000356297	p.L1126P	c.3377T>C	Verified	AGG	CTC	TTC	2	T	C
TCGA-13-0792	PYGM	ENST00000164139	p.I276T	c.827T>C	Verified	AAC	ATC	TCT	2	T	C
TCGA-24-1470	Q8N8K0_HU	ENST00000301807	p.M740T	c.2219T>C	Verified	AAA	ATG	ACC	2	T	C
TCGA-24-1422	RAB11B	ENST00000328024	p.V134A	c.401T>C	Unverified	GCT	GTG	CCC	2	T	C
TCGA-24-1563	RAB27A	ENST00000396307	p.L84L	c.250T>C	Verified	agc	tta	acg	1	T	C
TCGA-24-0979	RABGEF1	ENST00000510829	p.D313D	c.939T>C	Verified	gcg	gat	gac	3	T	C
TCGA-24-2289	RAD21	ENST00000297338	p.S195P	c.583T>C	Unverified	gag	tct	gaa	1	T	C
TCGA-29-1762	RAD54B	ENST00000336148	p.S26S	c.78T>C	Unverified	aga	agt	aat	3	T	C
TCGA-20-0990	RALYL	ENST00000522455	p.S107P	c.319T>C	Verified	CTT	TCT	GCA	1	T	C
TCGA-29-1777	RASA3	ENST00000334062	p.S444S	c.1332T>C	Unverified	gag	tct	ggg	3	T	C
TCGA-13-0762	RASGEF1A	NM_145313.1	p.L120L	c.358T>C	Unverified	tcc	ttg	gct	1	T	C
TCGA-13-0762	RASGEF1A	NM_145313.1	p.L120L	c.358T>C	Unverified	tcc	ttg	gct	1	T	C
TCGA-24-0975	RASGEF1A	NM_145313.1	p.V164A	c.491T>C	Unverified	ctg	gtg	ctg	2	T	C
TCGA-24-1103	RBAK	ENST00000353796	p.Y233Y	c.699T>C	Verified	ccc	tat	gag	3	T	C
TCGA-13-0755	RBM34_ENS	ENST00000408888	p.G51G	c.153T>C	Verified	AGA	GGT	GGC	3	T	C
TCGA-61-2012	RBM39	ENST00000253363	p.S246S	c.738T>C	Verified	GGA	AGT	GCT	3	T	C
TCGA-24-1422	RBM44	ENST00000316997	p.W786R	c.2356T>C	Verified	GAC	TGG	TCT	1	T	C
TCGA-25-2042	RBM44	ENST00000316997	p.V902A	c.2705T>C	Verified	CCT	GTT	AAA	2	T	C
TCGA-13-0903	RC3H2	ENST00000373670	p.Y589H	c.1765T>C	Verified	GTA	TAT	CCT	1	T	C

TCGA-13-0762	RELN	ENST00000343529	p.M702T	c.2105T>C	Verified	GAG	ATG	GCA	2	T	C
TCGA-13-0762	RELN	ENST00000343529	p.M702T	c.2105T>C	Verified	GAG	ATG	GCA	2	T	C
TCGA-29-1763	RGAG1	ENST00000465301	p.A814A	c.2442T>C	Unverified	CCA	GCT	TAT	3	T	C
TCGA-61-2012	RGP1	ENST00000456972	p.V279A	c.836T>C	Verified	GAC	GTG	GTG	2	T	C
TCGA-25-1313	RGS20	ENST00000297313	p.I386I	c.1158T>C	Verified	tct	att	gaa	3	T	C
TCGA-61-1915	RICTOR	ENST00000357387	p.A130A	c.390T>C	Unverified	ATA	GCT	AGG	3	T	C
TCGA-36-1577	RIMBP2	ENST00000261655	p.I940T	c.2819T>C	Verified	AAA	ATA	GAG	2	T	C
TCGA-23-1117	RIMS1	ENST00000521978	p.S989S	c.2967T>C	Verified	CGT	TCT	CCA	3	T	C
TCGA-23-1117	RIMS1	ENST00000521978	p.S989S	c.2967T>C	Verified	CGT	TCT	CCA	3	T	C
TCGA-20-0991	RIMS2	ENST00000507740	p.L580P	c.1739T>C	Verified	TTT	CTT	CCA	2	T	C
TCGA-20-0991	RIMS2_ENS	ENST00000436393	p.L566P	c.1697T>C	Verified	TTT	CTT	CCA	2	T	C
TCGA-61-1740	RMND5A	ENST00000283632	p.I303T	c.908T>C	Unverified	GTG	ATT	GAA	2	T	C
TCGA-13-1488	RNASE2	ENST00000304625	p.W37R	c.109T>C	Verified	CAA	TGG	TTT	1	T	C
TCGA-24-1847	RNF130	ENST00000521389	p.Y271H	c.811T>C	Unverified	agc	tat	aag	1	T	C
TCGA-24-1470	RNF182	ENST00000537663	p.V235V	c.705T>C	Verified	TGT	GTT	TGT	3	T	C
TCGA-23-1032	RPLP0	ENST00000392514	p.S240S	c.720T>C	Verified	CAT	TCT	ATC	3	T	C
TCGA-24-0979	RPN1	ENST00000296255	p.I454T	c.1361T>C	Verified	GTT	ATC	ATC	2	T	C
TCGA-24-2262	RPS27A	ENST00000272317	p.Y105H	c.313T>C	Verified	AAA	TAT	TAT	1	T	C
TCGA-61-1914	RTDR1	ENST00000216036	p.L113P	c.338T>C	Unverified	GCC	CTG	TCC	2	T	C
TCGA-24-1104	RYK	NM_002958	p.I341T	c.1022T>C	Verified	cgt	att	ttc	2	T	C
TCGA-09-2044	RYR2	ENST00000366574	p.L730P	c.2189T>C	Verified	GGC	CTT	CAT	2	T	C
TCGA-29-1781	SAP18	NM_005870.3	p.N50N	c.150T>C	Unverified	gga	aat	gta	3	T	C
TCGA-25-1326	SBNO1	ENST00000267176	p.L1083P	c.3248T>C	Verified	GGC	CTG	ATA	2	T	C
TCGA-61-1915	SCAND3	ENST00000452236	p.L1038L	c.3112T>C	Unverified	AGA	TTA	TTC	1	T	C
TCGA-13-1510	SCN3A	ENST00000283254	p.V1432V	c.4296T>C	Verified	GAT	GTT	AAA	3	T	C
TCGA-13-1499	SCN5A	ENST00000333535	p.L921L	c.2763T>C	Verified	TTG	CTT	GTT	3	T	C
TCGA-29-1769	SCN8A	ENST00000354534	p.F273L	c.817T>C	Unverified	CTG	TTC	ATG	1	T	C
TCGA-29-1769	SCN8A_ENS	ENST00000354534	p.F273L	c.817T>C	Unverified	CTG	TTC	ATG	1	T	C
TCGA-23-1120	SCRN1	ENST00000242059	p.L127S	c.380T>C	Verified	GCC	TTA	GAT	2	T	C
TCGA-24-2262	SDAD1	ENST00000356260	p.V269A	c.806T>C	Verified	AAA	GTG	CTC	2	T	C
TCGA-04-1347	SEC23A	ENST00000307712	p.L637P	c.1910T>C	Verified	CTT	CTT	GAT	2	T	C
TCGA-09-2051	SELP	ENST00000367790	p.S781S	c.2343T>C	Unverified	GCT	TCT	ACG	3	T	C
TCGA-13-0893	SEMA3D	ENST00000284136	p.D465D	c.1395T>C	Verified	TAC	GAT	GTA	3	T	C
TCGA-13-1496	SERPINA3	ENST00000393078	p.S302P	c.904T>C	Verified	gac	tct	ctg	1	T	C

TCGA-25-2392	SETD5	ENST00000302463	p.S1229S	c.3687T>C	Verified	CAT	TCT	GGA	3	T	C
TCGA-61-1900	SFI1	ENST00000400288	p.L754P	c.2261T>C	Unverified	TGT	CTG	CGG	2	T	C
TCGA-23-1123	SFXN1	ENST00000321442	p.V229A	c.686T>C	Verified	CAA	GTT	GTC	2	T	C
TCGA-23-1123	SFXN1	ENST00000321442	p.V229A	c.686T>C	Verified	CAA	GTT	GTC	2	T	C
TCGA-04-1356	SHC4	ENST00000332408	p.N313N	c.939T>C	Verified	GTT	AAT	CAA	3	T	C
TCGA-29-1698	SIGLEC7	ENST00000317643	p.S271S	c.813T>C	Unverified	CAG	TCT	CTG	3	T	C
TCGA-29-1699	SLC12A7	ENST00000264930	p.C163R	c.487T>C	Unverified	ACA	TGT	ACA	1	T	C
TCGA-13-0887	SLC15A1	ENST00000376503	p.V366A	c.1097T>C	Verified	ATG	GTC	CTG	2	T	C
TCGA-24-1563	SLC16A9	ENST00000395347	p.A299A	c.897T>C	Verified	GTG	GCT	CTT	3	T	C
TCGA-13-0792	SLC22A10	ENST00000332793	p.L180L	c.538T>C	Verified	TGT	TTG	CTC	1	T	C
TCGA-23-1032	SLC22A25	ENST00000306494	p.I75T	c.224T>C	Verified	AGA	ATC	TCC	2	T	C
TCGA-30-1718	SLC25A43	ENST00000217909	p.P336P	c.1008T>C	Unverified	AAG	CCT	AAA	3	T	C
TCGA-23-1031	SLC28A2	ENST00000347644	p.V284A	c.851T>C	Verified	TGG	GTA	GTT	2	T	C
TCGA-23-2078	SLC35E4	ENST00000343605	p.L162P	c.485T>C	Unverified	CCA	CTT	CAG	2	T	C
TCGA-13-1512	SLC38A7	ENST00000219320	p.D18D	c.54T>C	Verified	ACG	GAT	GCC	3	T	C
TCGA-24-1849	SLC4A9	ENST00000230993	p.S81P	c.241T>C	Unverified	GCA	TCT	CTG	1	T	C
TCGA-24-1849	SLC4A9_ENS	ENST00000230993_v65	p.S81P	c.241T>C	Unverified	GCA	TCT	CTG	1	T	C
TCGA-04-1638	SLC5A2	ENST00000330498	p.V298A	c.893T>C	Unverified	ATC	GTG	CAG	2	T	C
TCGA-29-1699	SLC5A9	ENST00000236495	p.W648R	c.1942T>C	Unverified	CTC	TGG	AGC	1	T	C
TCGA-23-1032	SLC6A5	ENST00000525748	p.L478S	c.1433T>C	Verified	TCT	TTA	TCT	2	T	C
TCGA-13-0906	SLC7A10	ENST00000253188	p.I285T	c.854T>C	Verified	AAC	ATT	GCC	2	T	C
TCGA-20-0990	SLCO1B3	ENST00000381545	p.F620F	c.1860T>C	Verified	GTA	TTT	TTT	3	T	C
TCGA-20-1683	SLCO3A1	ENST00000318445	p.V364A	c.1091T>C	Unverified	GCA	GTG	GTG	2	T	C
TCGA-24-1845	SLITRK4	ENST00000381779	p.I607T	c.1820T>C	Unverified	ccc	att	cga	2	T	C
TCGA-24-2288	SLITRK4	ENST00000381779	p.I772T	c.2315T>C	Unverified	agc	ata	ggt	2	T	C
TCGA-20-1683	SMAD4	ENST00000342988	p.F310L	c.928T>C	Unverified	gca	ttc	cag	1	T	C
TCGA-24-2289	SMARCA1	NM_003069.2	p.I804I	c.2412T>C	Unverified	gaa	att	ctt	3	T	C
TCGA-29-1693	SMARCC1	ENST00000254480	p.S748P	c.2242T>C	Unverified	GCC	TCT	GGG	1	T	C
TCGA-61-1907	SMC1A	ENST00000322213	p.I249T	c.746T>C	Unverified	gag	atc	gag	2	T	C
TCGA-23-1031	SMC5	ENST00000361138	p.S792P	c.2374T>C	Verified	GCA	TCT	TCA	1	T	C
TCGA-29-1711	SMC6	ENST00000448223	p.D594D	c.1782T>C	Unverified	ata	gat	aat	3	T	C
TCGA-29-1711	SMC6	ENST00000448223	p.D594D	c.1782T>C	Unverified	ata	gat	aat	3	T	C
TCGA-23-1124	SMG8	ENST00000300917	p.L532L	c.1596T>C	Verified	GCT	CTT	CGA	3	T	C
TCGA-61-2008	SMS	ENST00000404933	p.D167D	c.501T>C	Verified	ggg	gat	gtt	3	T	C

TCGA-13-0885	SNRPD3	ENST00000215829	p.G4G	c.12T>C	Verified	ATT	GGT	GTG	3	T	C
TCGA-13-0920	SP100_ENST	ENST00000340126	p.L815L	c.2443T>C	Verified	TGG	TTA	AAC	1	T	C
TCGA-30-1856	SPAM1	ENST00000340011	p.S74P	c.220T>C	Unverified	TTC	TCT	TTC	1	T	C
TCGA-30-1856	SPAM1_ENS	ENST00000439500	p.S74P	c.220T>C	Unverified	TTC	TCT	TTC	1	T	C
TCGA-23-1124	SPATA8	ENST00000328504	p.C69R	c.205T>C	Verified	AGC	TGT	TCT	1	T	C
TCGA-25-2392	SPCS3	ENST00000503362	p.I66T	c.197T>C	Verified	TTT	ATC	ACA	2	T	C
TCGA-23-1031	SPG11	ENST00000261866	p.F118F	c.354T>C	Verified	GAA	TTT	AAT	3	T	C
TCGA-24-1469	SPHKAP	XM_051221.5	p.V440A	c.1319T>C	Verified	gtg	gtt	tct	2	T	C
TCGA-13-0924	SPINK6	ENST00000325630	p.C30C	c.90T>C	Verified	GAC	TGT	GGT	3	T	C
TCGA-13-0900	SPRED2	ENST00000356388	p.S129P	c.385T>C	Unverified	ACG	TCA	TCT	1	T	C
TCGA-29-1761	SRFBP1	ENST00000339397	p.V105A	c.314T>C	Unverified	GCA	GTA	CAT	2	T	C
TCGA-25-1318	SRPRB	ENST00000466490	p.L129S	c.386T>C	Verified	TTC	TTA	GAG	2	T	C
TCGA-25-1318	SRPRB	ENST00000466490	p.L129S	c.386T>C	Verified	TTC	TTA	GAG	2	T	C
TCGA-29-1691	SRRM2	ENST00000301740	p.Y2390H	c.7168T>C	Unverified	GCC	TAC	GAG	1	T	C
TCGA-29-1699	SSTR3	ENST00000328544	p.A157A	c.471T>C	Unverified	ACA	GCT	CCG	3	T	C
TCGA-04-1331	SSX2IP	ENST00000342203	p.G245G	c.735T>C	Verified	ACT	GGT	AAA	3	T	C
TCGA-13-0760	STAT2	ENST00000314128	p.V286A	c.857T>C	Verified	ctg	gtt	agc	2	T	C
TCGA-24-1431	STEAP2	ENST00000394621	p.F146L	c.436T>C	Verified	GGA	TTT	AAT	1	T	C
TCGA-23-1120	STXBP5L	ENST00000273666	p.F4F	c.12T>C	Verified	AAG	TTT	AAT	3	T	C
TCGA-13-0760	SULF1	ENST00000260128	p.Y838Y	c.2514T>C	Verified	GGA	TAT	AAG	3	T	C
TCGA-29-1777	SULT1A1	ENST00000314752	p.I4T	c.11T>C	Unverified	CTG	ATC	CAG	2	T	C
TCGA-29-1703	SUSD4	ENST00000344029	p.L54L	c.162T>C	Unverified	GAC	CTT	CAA	3	T	C
TCGA-29-1703	SUSD4_ENST	ENST00000343846	p.L54L	c.162T>C	Unverified	GAC	CTT	CAA	3	T	C
TCGA-23-1110	SYNE2	ENST00000358025	p.L2603P	c.7808T>C	Verified	CAA	CTT	GAA	2	T	C
TCGA-24-1423	SYNJ1	NM_003895.2	p.L1328P	c.3983T>C	Verified	gat	ctg	tca	2	T	C
TCGA-09-1665	SYT1	ENST00000393240	p.F77F	c.231T>C	Verified	TGC	TTT	TGT	3	T	C
TCGA-24-2035	TACC2	ENST00000369005	p.L2915P	c.8744T>C	Unverified	CAG	CTG	CGA	2	T	C
TCGA-24-1847	TARBP1	ENST00000040877	p.V1168A	c.3503T>C	Unverified	AGA	GTG	AAA	2	T	C
TCGA-13-0887	TARBP1	ENST00000040877	p.A1198A	c.3594T>C	Verified	CAG	GCT	GGT	3	T	C
TCGA-29-1691	TAS2R9	ENST00000240691	p.T177T	c.531T>C	Unverified	GGT	ACT	TTC	3	T	C
TCGA-29-1691	TCEANC	OTTHUMT00000055796.	p.H42H	c.126T>C	Unverified	gag	cat	ctc	3	T	C
TCGA-61-1725	TCF12	ENST00000438423	p.S508S	c.1524T>C	Unverified	CTG	TCT	AGT	3	T	C
TCGA-61-1725	TCF12_ENST	ENST00000343827	p.S314S	c.942T>C	Unverified	CTG	TCT	AGT	3	T	C
TCGA-61-1725	TCF12_ENST	ENST00000438423	p.S508S	c.1524T>C	Unverified	CTG	TCT	AGT	3	T	C

TCGA-61-1725	TCF12_ENST	ENST00000452095	p.S504S	c.1512T>C	Unverified	CTG	TCT	AGT	3	T	C
TCGA-61-2095	TCHP	ENST00000312777	p.L402P	c.1205T>C	Unverified	TCC	CTG	AAA	2	T	C
TCGA-61-2095	TCIRG1	ENST00000265686	p.V595A	c.1784T>C	Unverified	TGT	GTC	TGG	2	T	C
TCGA-29-1777	TCN2	ENST00000215838	p.M42T	c.125T>C	Unverified	TGG	ATG	GAC	2	T	C
TCGA-13-1498	TCP11L2	ENST00000299045	p.V173A	c.518T>C	Verified	GCT	GTT	GAC	2	T	C
TCGA-13-0884	TDRD6	ENST00000316081	p.F976L	c.2926T>C	Verified	TAC	TTC	TAT	1	T	C
TCGA-24-2289	TECTB	ENST00000369422	p.V143A	c.428T>C	Unverified	CAC	GTG	AAG	2	T	C
TCGA-61-2009	TEX14	ENST00000349033	p.H1038H	c.3114T>C	Verified	GAG	CAT	AGT	3	T	C
TCGA-23-1032	TEX15	ENST00000256246	p.S160P	c.478T>C	Verified	ATT	TCT	GTG	1	T	C
TCGA-13-0916	TFAP2D	ENST00000008391	p.T365T	c.1095T>C	Verified	CCC	ACT	CCA	3	T	C
TCGA-29-1698	TFDP1	ENST00000375370	p.C202C	c.606T>C	Unverified	gaa	tgt	cag	3	T	C
TCGA-29-1691	TFSM1_HUI	ENST00000314720	p.H42H	c.126T>C	Unverified	GAG	CAT	CTC	3	T	C
TCGA-29-1777	TG	ENST00000220616	p.L1152P	c.3455T>C	Unverified	GTG	CTC	AAG	2	T	C
TCGA-61-2113	TG	ENST00000220616	p.V1511A	c.4532T>C	Verified	TGT	GTC	ACT	2	T	C
TCGA-24-1422	TG	ENST00000220616	p.H892H	c.2676T>C	Verified	GCA	CAT	TTT	3	T	C
TCGA-24-1417	THPO	ENST00000204615	p.L195P	c.584T>C	Verified	GAG	CTC	CCA	2	T	C
TCGA-13-0885	THUMPD3	ENST00000452837	p.N160N	c.480T>C	Verified	AAT	AAT	GGA	3	T	C
TCGA-24-2288	TIGD6	ENST00000296736	p.T97T	c.291T>C	Unverified	GTG	ACT	GGT	3	T	C
TCGA-25-2398	TIMELESS	ENST00000553532	p.S756S	c.2268T>C	Verified	CTT	AGT	GAC	3	T	C
TCGA-04-1652	TLR7	ENST00000380659	p.F580F	c.1740T>C	Unverified	tat	ttt	caa	3	T	C
TCGA-04-1652	TLR7	ENST00000380659	p.F580F	c.1740T>C	Unverified	tat	ttt	caa	3	T	C
TCGA-61-1737	TM9SF2	ENST00000376387	p.Y150Y	c.450T>C	Unverified	AAT	TAT	CAA	3	T	C
TCGA-04-1638	TMEFF1	ENST00000374879	p.I336T	c.1007T>C	Unverified	ATT	ATT	GGA	2	T	C
TCGA-13-0924	TMEM126A	ENST00000304511	p.V60A	c.179T>C	Verified	AAT	GTG	ACA	2	T	C
TCGA-23-1809	TMEM63C	ENST00000298351	p.S248S	c.744T>C	Unverified	GGC	AGT	GTC	3	T	C
TCGA-13-0886	TNFRSF8	ENST00000263932	p.C122C	c.366T>C	Verified	TCC	TGT	GCC	3	T	C
TCGA-29-1781	TNFSF4	ENST00000281834	p.S134S	c.402T>C	Unverified	agg	tct	gtc	3	T	C
TCGA-04-1337	TP53	ENST00000269305	p.C135R	c.403T>C	Verified	TTT	TGC	CAA	1	T	C
TCGA-04-1649	TP53	ENST00000269305	p.F270S	c.809T>C	Unverified	AGC	TTT	GAG	2	T	C
TCGA-13-0760	TP53	ENST00000269305	p.L265P	c.794T>C	Verified	CTA	CTG	GGA	2	T	C
TCGA-24-1104	TP53	ENST00000269305	p.I195T	c.584T>C	Verified	CTT	ATC	CGA	2	T	C
TCGA-24-1469	TP53	ENST00000269305	p.I195T	c.584T>C	Verified	CTT	ATC	CGA	2	T	C
TCGA-24-1563	TP53	ENST00000269305	p.I195T	c.584T>C	Verified	CTT	ATC	CGA	2	T	C
TCGA-04-1649	TP53_ENST(	ENST00000269305	p.F270S	c.809T>C	Unverified	AGC	TTT	GAG	2	T	C

TCGA-04-1347	TPX2	ENST00000300403	p.I151T	c.452T>C	Verified	atc	atc	gat	2	T	C
TCGA-24-1470	TRANK1	ENST00000428977	p.M740T	c.2219T>C	Verified	AAA	ATG	ACC	2	T	C
TCGA-23-1118	TRIM23	ENST00000231524	p.T442T	c.1326T>C	Verified	gaa	act	gta	3	T	C
TCGA-29-1775	TRIM31	ENST00000376734	p.S384S	c.1152T>C	Unverified	att	tct	ggt	3	T	C
TCGA-13-0906	TRIM42	ENST00000286349	p.V420A	c.1259T>C	Unverified	AAA	GTG	AAC	2	T	C
TCGA-29-1763	TRIOBP_ENS	ENST00000406386	p.S516P	c.1546T>C	Unverified	ACC	TCC	TCT	1	T	C
TCGA-24-2267	TRMT1L	ENST00000367506	p.Y251H	c.751T>C	Verified	TCC	TAT	TTT	1	T	C
TCGA-25-2393	TRPA1	NM_007332.1	p.F278S	c.833T>C	Verified	cat	ttt	gct	2	T	C
TCGA-24-1563	TRPA1	NM_007332.1	p.N143N	c.429T>C	Verified	atg	aat	aat	3	T	C
TCGA-23-1117	TRPC3	ENST00000264811	p.V710A	c.2129T>C	Verified	CTA	GTT	CCT	2	T	C
TCGA-23-1117	TRPC3	ENST00000264811	p.V710A	c.2129T>C	Verified	CTA	GTT	CCT	2	T	C
TCGA-23-1021	TRPM4	ENST00000252826	p.L641P	c.1922T>C	Verified	CGC	CTC	CTC	2	T	C
TCGA-04-1338	TRPV3	ENST00000576742	p.L541L	c.1621T>C	Verified	TAC	TTG	TTT	1	T	C
TCGA-20-0991	TSGA10	ENST00000355053	p.V321A	c.962T>C	Verified	ATT	GTG	TGT	2	T	C
TCGA-24-1469	TSHZ3	NM_020856.1	p.L255P	c.764T>C	Verified	ctg	ctg	gat	2	T	C
TCGA-29-1761	TSNARE1	ENST00000307180	p.L386P	c.1157T>C	Unverified	GAG	CTG	GCT	2	T	C
TCGA-23-1110	TSPAN19	ENST00000532498	p.F97F	c.291T>C	Verified	ACC	TTT	GCT	3	T	C
TCGA-29-1768	TSPYL5	ENST00000322128	p.L224L	c.672T>C	Unverified	TAC	CTT	CGG	3	T	C
TCGA-30-1857	TTC29	ENST00000325106	p.R460R	c.1380T>C	Unverified	GAA	CGT	TTG	3	T	C
TCGA-29-1761	TTC6	ENST00000267368	p.I520T	c.1559T>C	Unverified	GTT	ATA	TGA	2	T	C
TCGA-29-1761	TTC6_ENST(	ENST00000553443	p.I1886T	c.5657T>C	Unverified	GTT	ATA	TGA	2	T	C
TCGA-13-0762	TTLL3	ENST00000336674	p.F361S	c.1082T>C	Unverified	CGC	TTT	TCC	2	T	C
TCGA-13-0762	TTLL3	ENST00000336674	p.F361S	c.1082T>C	Unverified	CGC	TTT	TCC	2	T	C
TCGA-23-1110	TTN	NM_003319	p.Y18577H	c.55729T>C	Verified	gaa	tat	aac	1	T	C
TCGA-25-1326	TTN	NM_003319	p.I18141T	c.54422T>C	Verified	tat	att	gta	2	T	C
TCGA-29-1777	TTN	NM_003319	p.L3252P	c.9755T>C	Unverified	ttg	cta	att	2	T	C
TCGA-61-1910	TTN	NM_003319	p.T559T	c.1677T>C	Unverified	gaa	act	agg	3	T	C
TCGA-29-1777	TTN_ENST0(	ENST00000342175	p.L3252P	c.9755T>C	Unverified	TTG	CTA	ATT	2	T	C
TCGA-61-1910	TTN_ENST0(	ENST00000342175	p.T559T	c.1677T>C	Unverified	GAA	ACT	AGG	3	T	C
TCGA-29-1777	TTN_ENST0(	ENST00000342992	p.L3298P	c.9893T>C	Unverified	TTG	CTA	ATT	2	T	C
TCGA-61-1910	TTN_ENST0(	ENST00000342992	p.T605T	c.1815T>C	Unverified	GAA	ACT	AGG	3	T	C
TCGA-23-1110	TTN_ENST0(	ENST00000356127	p.Y25072H	c.75214T>C	Verified	GAA	TAT	AAC	1	T	C
TCGA-25-1326	TTN_ENST0(	ENST00000356127	p.I24636T	c.73907T>C	Verified	TAT	ATT	GTA	2	T	C
TCGA-29-1777	TTN_ENST0(	ENST00000356127	p.L3298P	c.9893T>C	Unverified	TTG	CTA	ATT	2	T	C

TCGA-61-1910	TTN_ENST0(	ENST00000356127	p.T605T	c.1815T>C	Unverified	GAA	ACT	AGG	3	T	C
TCGA-29-1777	TTN_ENST0(	ENST00000359218	p.L3252P	c.9755T>C	Unverified	TTG	CTA	ATT	2	T	C
TCGA-61-1910	TTN_ENST0(	ENST00000359218	p.T559T	c.1677T>C	Unverified	GAA	ACT	AGG	3	T	C
TCGA-29-1777	TTN_ENST0(	ENST00000360870	p.L3298P	c.9893T>C	Unverified	TTG	CTA	ATT	2	T	C
TCGA-13-0760	TTN_ENST0(	ENST00000360870	p.D4524D	c.13572T>C	Verified	TCA	GAT	TCA	3	T	C
TCGA-61-1910	TTN_ENST0(	ENST00000360870	p.T605T	c.1815T>C	Unverified	GAA	ACT	AGG	3	T	C
TCGA-13-1497	TUBA3C	ENST00000400113	p.V115A	c.344T>C	Verified	ATC	GTC	GAC	2	T	C
TCGA-13-0913	TUBB3	ENST00000315491	p.F85S	c.254T>C	Verified	CTC	TTC	AGG	2	T	C
TCGA-24-2267	TUBD1	ENST00000325752	p.I423T	c.1268T>C	Verified	tac	att	cat	2	T	C
TCGA-13-1481	TUBGCP4	ENST00000564079	p.H65H	c.195T>C	Verified	GGC	CAT	GTG	3	T	C
TCGA-61-1998	TXLNB	ENST00000358430	p.A632A	c.1896T>C	Unverified	CCT	GCT	CCA	3	T	C
TCGA-23-1122	TYR	ENST00000263321	p.F207F	c.621T>C	Verified	GCT	TTT	CTG	3	T	C
TCGA-29-1770	U2SURP	ENST00000319822	p.S26P	c.76T>C	Unverified	GGA	TCT	TCA	1	T	C
TCGA-13-1509	UBR2	ENST00000372899	p.T245T	c.735T>C	Verified	TAT	ACT	CTT	3	T	C
TCGA-24-2035	UBTF	ENST00000302904	p.A488A	c.1464T>C	Unverified	AGA	GCT	GAG	3	T	C
TCGA-24-1417	UCK2	ENST00000367879	p.F196L	c.586T>C	Verified	GAA	TTC	TGC	1	T	C
TCGA-13-0885	UCP1	ENST00000262999	p.F289L	c.865T>C	Verified	TGC	TTT	GAA	1	T	C
TCGA-09-2044	UGT1A10	ENST00000344644	p.F219L	c.655T>C	Verified	TTA	TTT	TGC	1	T	C
TCGA-23-1031	UGT2B10_EI	ENST00000265403_v61	p.V130V	c.390T>C	Verified	GTA	GTT	TCA	3	T	C
TCGA-13-0887	UGT2B17	ENST00000317746	p.P196P	c.588T>C	Verified	GTA	CCT	GTT	3	T	C
TCGA-20-0991	UNC13A	ENST00000428389	p.Y804Y	c.2412T>C	Unverified	ATC	TAT	GGG	3	T	C
TCGA-13-0886	UNC5CL	ENST00000373164	p.W33R	c.97T>C	Verified	CGA	TGG	CAC	1	T	C
TCGA-13-0760	USH2A	ENST00000307340	p.Y2277H	c.6829T>C	Verified	TTA	TAT	CTA	1	T	C
TCGA-20-1686	USP15	NM_006313.1	p.S232P	c.694T>C	Unverified	aac	tca	aat	1	T	C
TCGA-10-0930	USP24	XM_371254.3	p.Y2338H	c.7012T>C	Verified	gtg	tac	tgc	1	T	C
TCGA-10-0930	USP24	XM_371254.3	p.Y2338H	c.7012T>C	Verified	gtg	tac	tgc	1	T	C
TCGA-24-1423	USP46	ENST00000441222	p.I208T	c.623T>C	Verified	tcc	att	acc	2	T	C
TCGA-61-1740	USP54	NM_152586.2	p.Y206Y	c.618T>C	Unverified	acc	tat	agg	3	T	C
TCGA-61-1740	USP54_ENS⁻	ENST00000408019	p.Y1118Y	c.3354T>C	Unverified	ACC	TAT	AGG	3	T	C
TCGA-23-1120	VAV3	ENST00000370056	p.D374D	c.1122T>C	Verified	AGA	GAT	AAT	3	T	C
TCGA-09-2056	VCAN	ENST00000265077	p.T2265T	c.6795T>C	Verified	CCT	ACT	CTA	3	T	C
TCGA-29-1777	VCAN	ENST00000265077	p.S729S	c.2187T>C	Unverified	ACA	TCT	CTC	3	T	C
TCGA-29-1695	VEZF1	ENST00000581208	p.C179R	c.535T>C	Unverified	ATG	TGT	GGG	1	T	C
TCGA-29-1702	VNN2	ENST00000326499	p.L299L	c.897T>C	Unverified	AAA	CTT	CTC	3	T	C

TCGA-29-1777	WDPCP	ENST00000272321	p.I164T	c.491T>C	Unverified	ACC	ATC	AGT	2	T	C
TCGA-30-1718	WDR13	ENST00000376729	p.S330P	c.988T>C	Unverified	TTC	TCT	TTC	1	T	C
TCGA-30-1718	WDR13_ENS	ENST00000218056	p.S330P	c.988T>C	Unverified	TTC	TCT	TTC	1	T	C
TCGA-23-1032	WDR17	ENST00000280190	p.I259T	c.776T>C	Verified	TGC	ATA	ACA	2	T	C
TCGA-30-1855	WDR89	ENST00000394942	p.L346L	c.1036T>C	Unverified	TTG	TTA	CTT	1	T	C
TCGA-13-1497	WFDC10B	ENST00000330523	p.I41T	c.122T>C	Verified	AGC	ATA	GAT	2	T	C
TCGA-24-1435	XG	ENST00000381174	p.M139T	c.416T>C	Verified	aat	atg	gta	2	T	C
TCGA-30-1718	XIAP	ENST00000434753	p.I370T	c.1109T>C	Unverified	acc	atc	ttc	2	T	C
TCGA-23-1022	XKR3	ENST00000331428	p.I219T	c.656T>C	Verified	GCC	ATC	CAG	2	T	C
TCGA-29-1693	XKR3	ENST00000331428	p.M183T	c.548T>C	Unverified	CAG	ATG	TAT	2	T	C
TCGA-29-1768	XKR5	ENST00000409172	p.W79R	c.235T>C	Unverified	GTT	TGG	AAG	1	T	C
TCGA-23-1124	XKR9	ENST00000408926	p.P320P	c.960T>C	Verified	ATA	CCT	ATC	3	T	C
TCGA-61-2009	ZDBF2	ENST00000374423	p.D232D	c.696T>C	Verified	CCA	GAT	GGG	3	T	C
TCGA-61-1914	ZDHHC17	ENST00000426126	p.L605P	c.1814T>C	Unverified	GGC	CTC	TTT	2	T	C
TCGA-13-0883	ZEB1	ENST00000320985	p.A499A	c.1497T>C	Verified	GTC	GCT	ACA	3	T	C
TCGA-24-2262	ZFP14	ENST00000270001	p.I145I	c.435T>C	Verified	AAA	ATT	ACC	3	T	C
TCGA-13-0793	ZFP57	ENST00000376883	p.F373F	c.1119T>C	Verified	ACT	TTT	AGC	3	T	C
TCGA-09-2049	ZFYVE26	ENST00000347230	p.F678F	c.2034T>C	Verified	GGA	TTT	CTG	3	T	C
TCGA-13-0904	ZG16B	ENST00000382280	p.S153P	c.457T>C	Verified	ATC	TCC	TCT	1	T	C
TCGA-61-1915	ZMYM2	ENST00000456228	p.T254T	c.762T>C	Unverified	AAG	ACT	GGA	3	T	C
TCGA-61-1915	ZMYM2_ENS	ENST00000456228	p.T254T	c.762T>C	Unverified	AAG	ACT	GGA	3	T	C
TCGA-61-1915	ZNF198	ENST00000382869	p.T254T	c.762T>C	Unverified	AAG	ACT	GGA	3	T	C
TCGA-30-1718	ZNF239	ENST00000374446	p.I27I	c.81T>C	Unverified	GAT	ATT	TCC	3	T	C
TCGA-04-1361	ZNF304	ENST00000282286	p.Y217H	c.649T>C	Verified	GAC	TAT	GAT	1	T	C
TCGA-04-1361	ZNF304	ENST00000282286	p.Y217H	c.649T>C	Verified	GAC	TAT	GAT	1	T	C
TCGA-13-1496	ZNF334	ENST00000347606	p.S505S	c.1515T>C	Verified	TGC	AGT	CAG	3	T	C
TCGA-23-1110	ZNF365	ENST00000395254	p.Y102H	c.304T>C	Verified	TTG	TAC	AGC	1	T	C
TCGA-13-1505	ZNF41	ENST00000313116	p.G694G	c.2082T>C	Verified	tgt	ggt	gac	3	T	C
TCGA-61-1913	ZNF417	ENST00000312026	p.C385R	c.1153T>C	Unverified	GAA	TGT	GGA	1	T	C
TCGA-30-1856	ZNF43	ENST00000354959	p.L469L	c.1407T>C	Unverified	AAC	CTT	ACT	3	T	C
TCGA-24-2289	ZNF438	ENST00000361310	p.I539T	c.1616T>C	Verified	CGG	ATT	TGT	2	T	C
TCGA-61-1907	ZNF451	ENST00000357489	p.I729T	c.2186T>C	Unverified	TAC	ATC	TGT	2	T	C
TCGA-61-1907	ZNF451_ENS	ENST00000370706	p.I729T	c.2186T>C	Unverified	TAC	ATC	TGT	2	T	C
TCGA-23-1029	ZNF462	ENST00000277225	p.G998G	c.2994T>C	Unverified	GGT	GGT	GGT	3	T	C

TCGA-09-1674	ZNF551	ENST00000282296	p.S70P	c.208T>C	Unverified	GTA	TCT	ATA	1	T	C
TCGA-24-2280	ZNF572	ENST00000319286	p.R339R	c.1017T>C	Unverified	AGT	CGT	AGT	3	T	C
TCGA-23-1022	ZNF613	ENST00000293471	p.L585L	c.1753T>C	Verified	TCA	TTA	ACT	1	T	C
TCGA-23-1122	ZNF641	ENST00000301042	p.V146A	c.437T>C	Verified	ATA	GTA	GTC	2	T	C
TCGA-25-2391	ZNF654	ENST00000309495	p.P96P	c.288T>C	Unverified	ACA	CTT	ACT	3	T	C
TCGA-25-2391	ZNF654_EN	ENST00000309495	p.L204L	c.612T>C	Unverified	GCT	CTT	GGT	3	T	C
TCGA-61-2009	ZNF70	ENST00000341976	p.I407T	c.1220T>C	Unverified	CTC	ATT	GAG	2	T	C
TCGA-24-1849	ZNF701	ENST00000391785	p.L341L	c.1023T>C	Unverified	CAC	CTT	GAA	3	T	C
TCGA-13-1509	ZNF711	NM_021998.2	p.S621P	c.1861T>C	Verified	ata	tct	gtc	1	T	C
TCGA-29-1775	ZNF76	ENST00000373953	p.D127D	c.381T>C	Unverified	GAT	GAT	GAG	3	T	C
TCGA-29-1691	ZNF773	ENST00000282292	p.L39P	c.116T>C	Unverified	CTC	CTC	TAC	2	T	C
TCGA-61-1899	ZNF778	ENST00000433976	p.L321P	c.962T>C	Unverified	GGA	CTC	TCA	2	T	C
TCGA-24-1104	ZNF799	ENST00000357979	p.Y52H	c.154T>C	Verified	GAT	TAC	AGT	1	T	C
TCGA-13-0920	ZSCAN5B	ENST00000358992	p.N121N	c.363T>C	Verified	CGA	AAT	AAC	3	T	C
TCGA-23-1022	ZXDC	ENST00000336332	p.S331P	c.991T>C	Verified	TGC	TCC	TTT	1	T	C
TCGA-29-1770	AADAC	ENST00000232892	p.A308A	c.924T>G	Unverified	CTG	GCT	AAA	3	T	G
TCGA-30-1714	ABCA13	ENST00000319379	p.T2043T	c.6129T>G	Unverified	GAA	TAT	GCA	3	T	G
TCGA-30-1714	ABCA13_EN	ENST00000435803	p.T2098T	c.6294T>G	Unverified	ATA	ACT	TTG	3	T	G
TCGA-04-1338	ABCA9	ENST00000340001	p.F998L	c.2994T>G	Verified	ATT	TTT	AAT	3	T	G
TCGA-24-1422	ACAD10	ENST00000313698	p.F210C	c.629T>G	Verified	ATC	TTT	CTT	2	T	G
TCGA-61-2009	ACTR6	ENST00000188312	p.L270W	c.809T>G	Verified	CGT	TTG	GCC	2	T	G
TCGA-61-1915	ACTRT1	ENST00000371124	p.C286G	c.856T>G	Unverified	aag	tgt	gac	1	T	G
TCGA-61-1738	ADAM17	ENST00000310823	p.I284S	c.851T>G	Unverified	CGC	ATT	CTC	2	T	G
TCGA-61-1738	ADAM17	ENST00000310823	p.I284S	c.851T>G	Unverified	CGC	ATT	CTC	2	T	G
TCGA-25-2392	ADAMTS17	ENST00000268070	p.L287R	c.860T>G	Verified	CTG	CTA	CGA	2	T	G
TCGA-61-1910	ADAMTS9	ENST00000498707	p.V628G	c.1883T>G	Unverified	tgt	gta	gga	2	T	G
TCGA-25-1326	ADK	ENST00000286621	p.?	c.194+2T>G	Verified	gaa	ctg	ttt	2	T	G
TCGA-13-0920	AHNAK	ENST00000378024	p.L5588V	c.16762T>G	Verified	CAT	TTG	AGT	1	T	G
TCGA-61-1913	AKAP12	ENST00000402676	p.G1027G	c.3081T>G	Unverified	gaa	ggt	ggc	3	T	G
TCGA-13-0886	ALDH3A1	ENST00000225740	p.V261G	c.782T>G	Verified	att	gtg	gag	2	T	G
TCGA-29-1764	ALKBH1	ENST00000216489	p.F101C	c.302T>G	Unverified	ATT	TTT	ATC	2	T	G
TCGA-29-1711	AMHR2	ENST00000257863	p.P124P	c.372T>G	Unverified	ctg	cct	cct	3	T	G
TCGA-29-1711	AMHR2	ENST00000257863	p.P124P	c.372T>G	Unverified	ctg	cct	cct	3	T	G
TCGA-61-2113	ANK2	ENST00000357077	p.I536S	c.1607T>G	Unverified	CAC	ATC	TCT	2	T	G

TCGA-24-1845	ANK3	ENST00000280772	p.V1047G	c.3140T>G	Unverified	CTG	GTA	GAA	2	T	G
TCGA-24-1845	ANK3_ENST(	ENST00000355288	p.V181G	c.542T>G	Unverified	CTG	GTA	GAA	2	T	G
TCGA-24-1845	ANK3_ENST(	ENST00000395293	p.V682G	c.2045T>G	Unverified	CTG	GTA	GAA	2	T	G
TCGA-04-1652	ANKRD18B	ENST00000290943	p.I129M	c.387T>G	Unverified	AAC	ATT	AAG	3	T	G
TCGA-04-1652	ANKRD18B	ENST00000290943	p.I129M	c.387T>G	Unverified	AAC	ATT	AAG	3	T	G
TCGA-13-1501	ANKRD29	ENST00000322980	p.F101C	c.302T>G	Verified	GGA	TTT	GGA	2	T	G
TCGA-13-1501	ANKRD29	ENST00000322980	p.F101C	c.302T>G	Verified	GGA	TTT	GGA	2	T	G
TCGA-24-1563	ANKRD30A	ENST00000361713	p.L1017R	c.3050T>G	Verified	GCT	CTC	AGA	2	T	G
TCGA-13-0893	ANKRD30A	ENST00000361713	p.D270E	c.810T>G	Verified	CCT	GAT	GAG	3	T	G
TCGA-24-1850	ANKRD57	ENST00000356454	p.H441Q	c.1323T>G	Unverified	CAC	CAT	CAC	3	T	G
TCGA-29-1691	ANKS1A	ENST00000360359	p.L468V	c.1402T>G	Unverified	GTG	TTG	GTG	1	T	G
TCGA-09-2049	ANO10	ENST00000292246	p.S386A	c.1156T>G	Verified	ACT	TCA	TGG	1	T	G
TCGA-13-0887	AOX1	ENST00000374700	p.L381V	c.1141T>G	Verified	AAC	TTG	CTA	1	T	G
TCGA-13-1481	AP4B1	ENST00000369569	p.V156G	c.467T>G	Verified	GAA	GTA	GAT	2	T	G
TCGA-09-2051	APLF	ENST00000303795	p.C324G	c.970T>G	Verified	AGC	TGT	TCT	1	T	G
TCGA-13-0885	ARID5B	ENST00000279873	p.S304A	c.910T>G	Verified	GTC	TCA	AAT	1	T	G
TCGA-30-1855	ATP8A1	ENST00000381668	p.T413T	c.1239T>G	Unverified	GGT	ACT	CTG	3	T	G
TCGA-30-1855	ATP8A1_ENS	ENST00000264449	p.T413T	c.1239T>G	Unverified	GGT	ACT	CTG	3	T	G
TCGA-04-1347	AURKC	ENST00000448930	p.S227A	c.679T>G	Verified	CTA	TCA	ATG	1	T	G
TCGA-13-1512	AXL	ENST00000359092	p.C24G	c.70T>G	Verified	GCG	TGC	ATG	1	T	G
TCGA-23-1032	BAG5	ENST00000299204	p.V27G	c.80T>G	Verified	caa	gtt	atc	2	T	G
TCGA-13-0886	BANK1	ENST00000322953	p.N25K	c.75T>G	Verified	GGA	AAT	ACA	3	T	G
TCGA-61-1904	BDP1	ENST00000358731	p.S1915S	c.5745T>G	Unverified	GTT	TCT	GCT	3	T	G
TCGA-13-0890	BOLL	ENST00000392296	p.S167A	c.499T>G	Verified	AGC	TCC	CCT	1	T	G
TCGA-29-2427	BRCA1	ENST00000357654	p.L431*	c.1292T>G	Verified	gac	tta	ctg	2	T	G
TCGA-61-1914	BRCA1	ENST00000357654	p.L474*	c.1421T>G	Unverified	aac	tta	agc	2	T	G
TCGA-13-1509	BRDT	ENST00000362005	p.F172V	c.514T>G	Verified	GTA	TTT	AAG	1	T	G
TCGA-24-1849	BRDT	ENST00000362005	p.F373L	c.1119T>G	Unverified	CAT	TTT	TCA	3	T	G
TCGA-23-1031	C12orf40	ENST00000324616	p.C571G	c.1711T>G	Verified	CAG	TGC	AAT	1	T	G
TCGA-24-0975	C12orf56	ENST00000333722	p.L348V	c.1042T>G	Unverified	GGA	TTG	GGA	1	T	G
TCGA-13-0760	C14orf102	ENST00000354366	p.W25G	c.73T>G	Verified	GAC	TGG	CTG	1	T	G
TCGA-29-1776	C17orf42	ENST00000581216	p.L164L	c.492T>G	Unverified	AGA	CTT	AAG	3	T	G
TCGA-13-0792	C17orf75	ENST00000225805	p.Y345*	c.1035T>G	Verified	CAT	TAT	GCT	3	T	G
TCGA-13-0905	C18orf1	ENST00000361205	p.I221S	c.662T>G	Verified	gac	att	gct	2	T	G

TCGA-61-1914	C1orf101	ENST00000366533	p.L676L	c.2028T>G	Unverified	GGT	CTT	GTG	3	T	G
TCGA-61-1914	C1orf101_EI	ENST00000366534	p.L676L	c.2028T>G	Unverified	GGT	CTT	GTG	3	T	G
TCGA-29-1770	C20orf177	ENST00000358293	p.V171G	c.512T>G	Unverified	GCC	GTG	CCC	2	T	G
TCGA-04-1651	C20orf194	ENST00000252032	p.F242C	c.725T>G	Unverified	AGC	TTC	TTC	2	T	G
TCGA-04-1651	C20orf194	ENST00000252032	p.F242C	c.725T>G	Unverified	AGC	TTC	TTC	2	T	G
TCGA-24-1463	C7orf28B	ENST00000316731	p.N94K	c.282T>G	Unverified	TTC	AAT	GAA	3	T	G
TCGA-09-2051	CA3	ENST00000285381	p.S48S	c.144T>G	Unverified	TGG	TCT	GTG	3	T	G
TCGA-10-0930	CALHM1	ENST00000329905	p.V115G	c.344T>G	Verified	TGG	GTG	GCC	2	T	G
TCGA-10-0930	CALHM1	ENST00000329905	p.V115G	c.344T>G	Verified	TGG	GTG	GCC	2	T	G
TCGA-13-1512	CCDC132_EI	ENST00000305866	p.L393R	c.1178T>G	Verified	CAG	CTA	AAA	2	T	G
TCGA-29-1761	CCDC149	ENST00000324309	p.A105A	c.315T>G	Unverified	CGA	GCT	AAG	3	T	G
TCGA-29-1761	CCDC149_EI	ENST00000504487	p.A160A	c.480T>G	Unverified	CGA	GCT	AAG	3	T	G
TCGA-13-0760	CCDC18	ENST00000334652	p.I564S	c.1691T>G	Verified	AGA	ATT	AAG	2	T	G
TCGA-29-1764	CCDC73	ENST00000335185	p.F451L	c.1353T>G	Unverified	TCA	TTT	ATA	3	T	G
TCGA-61-2094	CCDC73	ENST00000335185	p.P639P	c.1917T>G	Unverified	AAT	CCT	GTT	3	T	G
TCGA-25-2393	CCNT2	ENST00000264157	p.V603G	c.1808T>G	Verified	CCT	GTT	GGC	2	T	G
TCGA-61-1740	CCR3	ENST00000357422	p.C294G	c.880T>G	Unverified	cac	tgc	tgc	1	T	G
TCGA-13-0885	CD109	ENST00000287097	p.T1112T	c.3336T>G	Verified	CTG	ACT	TGG	3	T	G
TCGA-13-1510	CD86	ENST00000330540	p.L63V	c.187T>G	Unverified	AAC	TTG	GTT	1	T	G
TCGA-04-1337	CDC27	ENST00000066544	p.V550G	c.1649T>G	Unverified	GAT	GTT	GCT	2	T	G
TCGA-13-0919	CDC27	ENST00000066544	p.D549E	c.1647T>G	Unverified	AAA	GAT	GTT	3	T	G
TCGA-61-1914	CDH11	ENST00000268603	p.F527C	c.1580T>G	Unverified	AGA	TTT	ATC	2	T	G
TCGA-23-1111	CDSN	ENST00000376288	p.S303S	c.909T>G	Unverified	TCC	TCT	GAC	3	T	G
TCGA-24-1847	CEBPE	ENST00000206513	p.V200G	c.599T>G	Unverified	GCA	GTG	AAC	2	T	G
TCGA-04-1367	CELSR1	ENST00000262738	p.W1958G	c.5872T>G	Unverified	TGG	TGG	GGG	1	T	G
TCGA-61-1907	CERKL	ENST00000410087	p.F318C	c.953T>G	Unverified	CGC	TTT	GGG	2	T	G
TCGA-61-1907	CERKL_ENST	ENST00000339098	p.F344C	c.1031T>G	Unverified	CGC	TTT	GGG	2	T	G
TCGA-04-1347	CHD7	ENST00000423902	p.L1229L	c.3687T>G	Verified	TTT	CTT	TCC	3	T	G
TCGA-23-1022	CHRM2	ENST00000320658	p.V95G	c.284T>G	Verified	GTG	GTG	TGT	2	T	G
TCGA-61-1910	CLCA1	ENST00000234701	p.S440A	c.1318T>G	Unverified	CCC	TCT	GCA	1	T	G
TCGA-23-1032	CLDN17	ENST00000286808	p.L167R	c.500T>G	Verified	TTC	CTT	GGC	2	T	G
TCGA-23-1032	CLLU1	ENST00000378485	p.S53A	c.157T>G	Verified	TTT	TCT	CAA	1	T	G
TCGA-04-1655	CLUAP1	ENST00000576634	p.L130R	c.389T>G	Unverified	GAT	CTT	GGC	2	T	G
TCGA-13-0887	CMYA5	ENST00000238522	p.V1522G	c.4565T>G	Verified	GAG	GTG	TTA	2	T	G

TCGA-04-1356	CNBD1	ENST00000518476	p.V194G	c.581T>G	Verified	GTG	GTT	GCA	2	T	G
TCGA-24-1844	COASY	ENST00000393818	p.F431C	c.1292T>G	Unverified	GTG	TTT	GGG	2	T	G
TCGA-09-1674	COL4A5	ENST00000361603	p.G1309G	c.3927T>G	Unverified	cag	ggt	aat	3	T	G
TCGA-24-1847	COL5A3	ENST00000264828	p.R550R	c.1650T>G	Unverified	GAT	CGT	GGC	3	T	G
TCGA-25-1313	COPE	ENST00000262812	p.V16G	c.47T>G	Unverified	GAG	GTA	GAC	2	T	G
TCGA-24-1422	CPA6	ENST00000297770	p.I176S	c.527T>G	Unverified	TTT	ATT	TTA	2	T	G
TCGA-04-1338	CREBBP	ENST00000262367	p.F1379V	c.4135T>G	Verified	CGG	TTT	GTG	1	T	G
TCGA-36-1577	CSMD3	ENST00000297405	p.T2365T	c.7095T>G	Verified	agt	act	tca	3	T	G
TCGA-29-1691	CTR9	ENST00000361367	p.L820V	c.2458T>G	Unverified	GAC	TTA	CTG	1	T	G
TCGA-30-1855	CUL9	ENST00000252050	p.C1077G	c.3229T>G	Unverified	TGC	TGC	CTG	1	T	G
TCGA-23-1117	CUX1	ENST00000292535	p.R602R	c.1806T>G	Verified	GTT	CGT	GGC	3	T	G
TCGA-23-1117	CUX1	ENST00000292535	p.R602R	c.1806T>G	Verified	GTT	CGT	GGC	3	T	G
TCGA-10-0930	DAND5	ENST00000317060	p.L12R	c.35T>G	Verified	TGC	CTG	CTT	2	T	G
TCGA-10-0930	DAND5	ENST00000317060	p.L12R	c.35T>G	Verified	TGC	CTG	CTT	2	T	G
TCGA-20-1686	DBC1	ENST00000373969	p.S572A	c.1714T>G	Unverified	CAT	TCG	GAG	1	T	G
TCGA-23-1122	DCLK2	ENST00000296550	p.C524W	c.1572T>G	Verified	GTG	TGT	GAA	3	T	G
TCGA-04-1347	DCST2	ENST00000368424	p.I390S	c.1169T>G	Verified	TAC	ATC	CCA	2	T	G
TCGA-04-1331	DDIT3	ENST00000547303	p.L25R	c.74T>G	Verified	gac	ctg	caa	2	T	G
TCGA-13-0793	DDO	ENST00000368924	p.I182R	c.545T>G	Unverified	AGG	ATA	AAG	2	T	G
TCGA-23-1022	DENND2A	ENST00000275884	p.S310A	c.928T>G	Verified	TCC	TCT	CCC	1	T	G
TCGA-24-2024	DENND4C	ENST00000380437	p.I952M	c.2856T>G	Verified	GGT	ATT	GCT	3	T	G
TCGA-20-1685	DEPDC5	ENST00000400248	p.S681A	c.2041T>G	Unverified	ATG	TCC	TTC	1	T	G
TCGA-25-1326	DHX34	ENST00000328771	p.F149L	c.447T>G	Verified	gca	ttt	ggg	3	T	G
TCGA-61-1904	DIAPH3	ENST00000400324	p.G584G	c.1752T>G	Unverified	TCT	GGT	GGA	3	T	G
TCGA-23-1032	DLG5	ENST00000372391	p.I540S	c.1619T>G	Verified	AGC	ATC	CGG	2	T	G
TCGA-09-2056	DMXL2	ENST00000251076	p.I568R	c.1703T>G	Verified	TGT	ATA	AAT	2	T	G
TCGA-29-1768	DMXL2	ENST00000251076	p.L2971W	c.8912T>G	Unverified	AGA	TTG	ACA	2	T	G
TCGA-13-0884	DNAH3	ENST00000261383	p.C3478W	c.10434T>G	Verified	CGA	TGT	TTG	3	T	G
TCGA-29-1691	DNM3	ENST00000358155	p.H267Q	c.801T>G	Unverified	AGA	CAT	ATC	3	T	G
TCGA-20-1683	DOPEY2	ENST00000399151	p.F2073C	c.6218T>G	Unverified	ATG	TTT	CTT	2	T	G
TCGA-24-1846	DPRX	ENST00000376650	p.L7R	c.20T>G	Unverified	GAT	CTT	CGT	2	T	G
TCGA-61-1998	DUXA	ENST00000554048	p.F63C	c.188T>G	Verified	TGG	TTT	CAG	2	T	G
TCGA-29-1768	DYSF	ENST00000258104	p.I2048S	c.6143T>G	Unverified	ATC	ATC	CTC	2	T	G
TCGA-61-1900	DYSF	ENST00000258104	p.L2015R	c.6044T>G	Unverified	AAG	CTT	GAG	2	T	G

TCGA-29-1768	DYSF_ENST(	ENST00000410020	p.I2087S	c.6260T>G	Unverified	ATC	ATC	CTC	2	T	G
TCGA-61-1900	DYSF_ENST(	ENST00000410020	p.L2054R	c.6161T>G	Unverified	AAG	CTT	GAG	2	T	G
TCGA-04-1356	ECE1	ENST00000374893	p.F405V	c.1213T>G	Verified	TCC	TTC	CTT	1	T	G
TCGA-24-1844	ECHDC2	ENST00000358358	p.I215M	c.645T>G	Unverified	GCC	ATT	GAC	3	T	G
TCGA-13-0791	EDA	ENST00000374552	p.L42R	c.125T>G	Verified	CTG	CTC	TTC	2	T	G
TCGA-24-2035	EDEM1	ENST00000256497	p.V447G	c.1340T>G	Verified	CAG	GTG	CTG	2	T	G
TCGA-20-0991	EHMT2	ENST00000375537	p.L618L	c.1854T>G	Unverified	TGC	CTT	TCA	3	T	G
TCGA-13-0886	EIF3M	ENST00000531120	p.R19R	c.57T>G	Verified	CTT	CGT	GCT	3	T	G
TCGA-13-1497	ENSG00000:	ENST00000308911	p.V419G	c.1256T>G	Verified	CTG	GTG	TCT	2	T	G
TCGA-24-2280	EOMES	ENST00000295743	p.Y679*	c.2037T>G	Verified	GGG	TAT	TAT	3	T	G
TCGA-29-1711	EPM2A	ENST00000367519	p.W230G	c.688T>G	Unverified	ATC	TGG	ATG	1	T	G
TCGA-29-1711	EPM2A	ENST00000367519	p.W230G	c.688T>G	Unverified	ATC	TGG	ATG	1	T	G
TCGA-13-0760	ERC1	ENST00000397203	p.L1016L	c.3048T>G	Verified	gaa	ctt	gac	3	T	G
TCGA-10-0930	ERVFRD-1	ENST00000472091	p.I74S	c.221T>G	Verified	CAT	ATT	TCC	2	T	G
TCGA-10-0930	ERVFRD-1	ENST00000472091	p.I74S	c.221T>G	Verified	CAT	ATT	TCC	2	T	G
TCGA-24-1103	EXOC1	ENST00000346134	p.V709G	c.2126T>G	Verified	GGA	GTA	TTT	2	T	G
TCGA-13-0885	EXOC3L1	ENST00000314586	p.I374M	c.1122T>G	Verified	AAC	ATT	GAG	3	T	G
TCGA-24-1469	F2RL2	ENST00000296641	p.F6V	c.16T>G	Verified	atc	ttt	gca	1	T	G
TCGA-13-1510	FABP3	ENST00000373713	p.L6R	c.17T>G	Verified	TTC	CTG	GGC	2	T	G
TCGA-25-2392	FAHD1	ENST00000427358	p.L148R	c.443T>G	Unverified	AAG	CTC	TGG	2	T	G
TCGA-29-1763	FAM105A	ENST00000274217	p.F149V	c.445T>G	Unverified	ATA	TTC	AGC	1	T	G
TCGA-30-1891	FAM161A	ENST00000307507	p.D107E	c.321T>G	Unverified	AAA	GAT	ACT	3	T	G
TCGA-13-0885	FAM20B	ENST00000263733	p.V87G	c.260T>G	Verified	GCA	GTC	ATG	2	T	G
TCGA-23-1031	FAM48B2	XM_293352.4	p.V630V	c.1890T>G	Unverified	cag	gtt	ttg	3	T	G
TCGA-23-1111	FAM53C	ENST00000239906	p.T283T	c.849T>G	Unverified	AAA	ACT	GGG	3	T	G
TCGA-61-1725	FASTKD3	ENST00000264669	p.T395T	c.1185T>G	Unverified	GAA	ACT	TTT	3	T	G
TCGA-13-1509	FAT3_ENST(	ENST00000298047_v61	p.F2235V	c.6703T>G	Verified	GAC	TTT	GAC	1	T	G
TCGA-09-2044	FAT3_ENST(	ENST00000298047_v61	p.V870G	c.2609T>G	Unverified	TCA	GTC	TTG	2	T	G
TCGA-09-2044	FAT3_ENST(	ENST00000409404	p.V870G	c.2609T>G	Unverified	TCA	GTC	TTG	2	T	G
TCGA-04-1331	FCRL5	ENST00000361835	p.I66R	c.197T>G	Unverified	GAA	ATA	CTA	2	T	G
TCGA-13-0755	FCRL5	ENST00000361835	p.A368A	c.1104T>G	Verified	AAG	GCT	GTG	3	T	G
TCGA-13-0893	FER1L6	ENST00000399018	p.M331R	c.992T>G	Verified	AGC	ATG	AAT	2	T	G
TCGA-09-2051	FGD2	ENST00000274963	p.V375G	c.1124T>G	Unverified	AAG	GTG	CGG	2	T	G
TCGA-29-1763	FGF5	ENST00000312465	p.L101R	c.302T>G	Unverified	CAT	CTG	CAG	2	T	G

TCGA-61-2009	FIGNL1	ENST00000395556	p.F440C	c.1319T>G	Verified	CTC	TTT	GGT	2	T	G
TCGA-24-1431	FKBP10	ENST00000321562	p.S389S	c.1167T>G	Verified	CCA	TCT	GAG	3	T	G
TCGA-23-1031	FNBP4	ENST00000263773	p.F543V	c.1627T>G	Verified	GAG	TTT	CTA	1	T	G
TCGA-29-1781	FNIP2_ENST	ENST00000264433	p.V865G	c.2594T>G	Unverified	CTC	GTG	GCT	2	T	G
TCGA-13-1498	FOSB	ENST00000353609	p.S114R	c.342T>G	Verified	AGC	AGT	GGC	3	T	G
TCGA-13-1498	FUT10	ENST00000327671	p.L315L	c.945T>G	Verified	TGG	CTT	CCA	3	T	G
TCGA-29-1701	GAD2	ENST00000259271	p.F330V	c.988T>G	Unverified	CCT	TTC	CTC	1	T	G
TCGA-10-0930	GAPVD1	ENST00000394105	p.L540R	c.1619T>G	Verified	CAG	CTT	TCG	2	T	G
TCGA-10-0930	GAPVD1	ENST00000394105	p.L540R	c.1619T>G	Verified	CAG	CTT	TCG	2	T	G
TCGA-61-1740	GBP6	ENST00000370456	p.N18K	c.54T>G	Unverified	AAC	AAT	GAG	3	T	G
TCGA-24-0979	GCC2	ENST00000309863	p.V645G	c.1934T>G	Verified	AAG	GTA	AAT	2	T	G
TCGA-09-2044	GCFC1	ENST00000331923	p.S720A	c.2158T>G	Verified	CCT	TCA	GTA	1	T	G
TCGA-04-1338	GCFC1	ENST00000331923	p.I219M	c.657T>G	Verified	GAA	ATT	CCA	3	T	G
TCGA-61-1900	GDA	ENST00000358399	p.D266E	c.798T>G	Unverified	tat	gat	aaa	3	T	G
TCGA-61-1900	GDA_ENST0	ENST00000238018	p.D266E	c.798T>G	Unverified	TAT	GAT	AAA	3	T	G
TCGA-23-2078	GHRH	ENST00000373614	p.V44G	c.131T>G	Verified	AAG	GTG	CTG	2	T	G
TCGA-29-1761	GJA8	ENST00000369235	p.T120T	c.360T>G	Unverified	GGG	ACT	AAC	3	T	G
TCGA-61-2095	GLIS3	ENST00000324333	p.Y356D	c.1066T>G	Unverified	CTG	TAC	GAC	1	T	G
TCGA-24-2267	GPR112	ENST00000394143	p.V2871G	c.8612T>G	Verified	agt	gtg	aaa	2	T	G
TCGA-61-2095	GPR114	ENST00000340339	p.D144E	c.432T>G	Unverified	AAG	GAT	GAA	3	T	G
TCGA-13-0890	GPR149	ENST00000389740	p.S268A	c.802T>G	Verified	TGC	TCT	CCG	1	T	G
TCGA-13-1498	GPRASP2	ENST00000543253	p.F508C	c.1523T>G	Verified	ggc	ttc	cga	2	T	G
TCGA-30-1855	GRIA2	ENST00000296526	p.W481G	c.1441T>G	Unverified	ATT	TGG	AAT	1	T	G
TCGA-30-1855	GRIA2_ENST	ENST00000264426	p.W481G	c.1441T>G	Unverified	ATT	TGG	AAT	1	T	G
TCGA-30-1855	GRIN2B	ENST00000279593	p.I365S	c.1094T>G	Unverified	ATA	ATT	CTT	2	T	G
TCGA-13-0755	GRM8	ENST00000339582	p.C64W	c.192T>G	Verified	CCT	TGT	GGG	3	T	G
TCGA-61-1740	GSPT1	NM_002094.1	p.L3R	c.8T>G	Unverified	gaa	ctt	tca	2	T	G
TCGA-61-1740	GSPT1_ENST	ENST00000434724	p.L141R	c.422T>G	Unverified	GAA	CTT	TCA	2	T	G
TCGA-13-1512	GTF3C1	ENST00000356183	p.W145G	c.433T>G	Verified	AGG	TGG	GGG	1	T	G
TCGA-29-1784	HAT1	ENST00000264108	p.F202C	c.605T>G	Unverified	tac	ttt	cta	2	T	G
TCGA-29-1763	hCG_38941	ENST00000328828	p.S51A	c.151T>G	Unverified	TCT	TCT	CCA	1	T	G
TCGA-09-1674	HCRTR2	ENST00000370862	p.L108L	c.324T>G	Unverified	TGC	CTT	CCA	3	T	G
TCGA-04-1362	HERC1	ENST00000443617	p.L3096*	c.9287T>G	Verified	GAA	TTA	CTT	2	T	G
TCGA-61-1914	HERC1	ENST00000443617	p.I616M	c.1848T>G	Unverified	TTC	ATT	CGC	3	T	G

TCGA-61-1914	HERC1_ENST	ENST00000443617	p.I616M	c.1848T>G	Unverified	TTC	ATT	CGC	3	T	G
TCGA-13-0923	HERC5	NM_016323.1	p.F793C	c.2378T>G	Verified	ctg	ttt	aag	2	T	G
TCGA-24-1563	HKDC1	ENST00000354624	p.Y587D	c.1759T>G	Verified	GAC	TAC	ATG	1	T	G
TCGA-13-0900	HRG	ENST00000232003	p.N137K	c.411T>G	Verified	GCC	AAT	ACC	3	T	G
TCGA-29-1764	HSPG2	ENST00000374695	p.F386V	c.1156T>G	Unverified	AGC	TTC	CAC	1	T	G
TCGA-23-1124	HTR5A	ENST00000287907	p.C297G	c.889T>G	Verified	CTC	TGC	TGG	1	T	G
TCGA-13-1497	HTRA3	ENST00000307358	p.P396P	c.1188T>G	Verified	TCA	CCT	TCT	3	T	G
TCGA-20-1687	HYDIN_ENST	ENST00000316490_v65	p.Y3442D	c.10324T>G	Unverified	AAC	TAC	CAG	1	T	G
TCGA-24-1423	IER2	ENST00000292433	p.G26G	c.78T>G	Unverified	cgc	ggt	ggc	3	T	G
TCGA-61-2113	IFT140	ENST00000426508	p.W386G	c.1156T>G	Unverified	CAG	TGG	GGT	1	T	G
TCGA-61-1915	IGF2BP3	ENST00000258729	p.T394T	c.1182T>G	Unverified	ATG	ACT	CCT	3	T	G
TCGA-04-1542	IL12RB2	ENST00000262345	p.F99C	c.296T>G	Verified	TTG	TTT	GTC	2	T	G
TCGA-13-0904	IL17RA	ENST00000319363	p.V579G	c.1736T>G	Unverified	CAG	GTC	CGC	2	T	G
TCGA-29-1781	IL26	ENST00000229134	p.L16R	c.47T>G	Unverified	ACT	CTG	TCT	2	T	G
TCGA-25-1318	IRAK3	ENST00000261233	p.L421V	c.1261T>G	Verified	TGT	TTG	GCA	1	T	G
TCGA-25-1318	IRAK3	ENST00000261233	p.L421V	c.1261T>G	Verified	TGT	TTG	GCA	1	T	G
TCGA-13-0886	ISPD	ENST00000407010	p.M102R	c.305T>G	Verified	AAC	ATG	GAA	2	T	G
TCGA-30-1718	ITK	ENST00000422843	p.L254V	c.760T>G	Unverified	CTT	TTG	GAC	1	T	G
TCGA-61-1910	ITPR2	ENST00000381340	p.S1126A	c.3376T>G	Unverified	AAG	TCT	GAG	1	T	G
TCGA-13-0793	JARID2	ENST00000341776	p.I557S	c.1670T>G	Unverified	gag	atc	ccc	2	T	G
TCGA-04-1342	KATNB1	ENST00000379661	p.L326R	c.977T>G	Unverified	CCA	CTG	CCC	2	T	G
TCGA-24-1104	KBTBD6	ENST00000379485	p.M494R	c.1481T>G	Verified	CGC	ATG	TTC	2	T	G
TCGA-13-0791	KCNE4	ENST00000281830	p.S131A	c.391T>G	Verified	TCC	TCC	CCG	1	T	G
TCGA-24-1103	KCNMA1	ENST00000286627	p.F213C	c.638T>G	Verified	GAT	TTC	ACA	2	T	G
TCGA-09-2044	KCNT2	ENST00000294725	p.C948W	c.2844T>G	Verified	TTG	TGT	TCT	3	T	G
TCGA-13-1499	KCTD3	ENST00000259154	p.F29C	c.86T>G	Verified	AGA	TTT	AGT	2	T	G
TCGA-23-1124	KDELR3	ENST00000216014	p.F194V	c.580T>G	Verified	GAC	TTC	TTC	1	T	G
TCGA-61-1913	KIAA0528	ENST00000333957	p.L521*	c.1562T>G	Unverified	CGC	TTA	AAA	2	T	G
TCGA-61-1913	KIAA0528_E	ENST00000544930	p.L336*	c.1007T>G	Unverified	CGC	TTA	AAA	2	T	G
TCGA-13-0760	KIAA0922	ENST00000361316	p.V107V	c.321T>G	Verified	GAT	GTT	TTG	3	T	G
TCGA-29-2427	KIAA1404	ENST00000396105	p.F1453C	c.4358T>G	Verified	TGC	TTC	GAA	2	T	G
TCGA-13-0890	KIAA1468	ENST00000398130	p.L290*	c.869T>G	Verified	GAA	TTA	TGG	2	T	G
TCGA-13-0795	KIAA1529	ENST00000375206	p.L887*	c.2660T>G	Verified	TCC	TTA	AAC	2	T	G
TCGA-23-1122	KIF2C	ENST00000372224	p.I56M	c.168T>G	Verified	GAG	ATT	GAT	3	T	G

TCGA-24-2280	KIR3DL3	ENST00000291860	p.I326M	c.978T>G	Unverified	CTG	ATT	GGG	3	T	G
TCGA-24-1464	KMO	ENST00000366559	p.S392A	c.1174T>G	Verified	CCA	TCG	ACC	1	T	G
TCGA-04-1655	KRTAP5-6	ENST00000382160	p.C125G	c.373T>G	Unverified	TGC	TGC	CAG	1	T	G
TCGA-61-1904	L1CAM	ENST00000370060	p.V850G	c.2549T>G	Unverified	AAT	GTG	ACG	2	T	G
TCGA-61-1914	L1TD1	ENST00000498273	p.Y713D	c.2137T>G	Unverified	AGT	TAT	GAG	1	T	G
TCGA-23-2078	L3MBTL2	ENST00000216237	p.S115A	c.343T>G	Unverified	TGC	TCC	AGG	1	T	G
TCGA-04-1356	LARP7	ENST00000324052	p.S413A	c.1237T>G	Verified	GAG	TCA	GAA	1	T	G
TCGA-25-2400	LAS1L	ENST00000374811	p.C256G	c.766T>G	Unverified	cat	tgc	aaa	1	T	G
TCGA-13-0913	LCE2C	ENST00000368783	p.L73R	c.218T>G	Unverified	TCC	CTG	AGC	2	T	G
TCGA-29-1770	LCN12	ENST00000371633	p.L48R	c.143T>G	Unverified	GGC	CTG	GCG	2	T	G
TCGA-61-1915	LIPI	ENST00000344577	p.V227G	c.680T>G	Unverified	TTT	GTG	GAT	2	T	G
TCGA-23-1022	LMNA	ENST00000368300	p.V256G	c.767T>G	Verified	cag	gtg	gag	2	T	G
TCGA-13-0885	LOC283849	ENST00000314586	p.I374M	c.1122T>G	Verified	AAC	ATT	GAG	3	T	G
TCGA-20-1687	LOC652153	ENST00000316490	p.Y3394D	c.10180T>G	Unverified	AAC	TAC	CAG	1	T	G
TCGA-24-2262	LPCAT1	ENST00000283415	p.Y138D	c.412T>G	Unverified	TCC	TAC	TTC	1	T	G
TCGA-61-1910	LRCH2	NM_020871.2	p.M205R	c.614T>G	Unverified	tta	atg	gaa	2	T	G
TCGA-61-1910	LRCH2_ENST	ENST00000317135	p.M205R	c.614T>G	Unverified	TTA	ATG	GAA	2	T	G
TCGA-13-0904	LRIG2	ENST00000361127	p.S1062R	c.3186T>G	Verified	GGT	AGT	GAG	3	T	G
TCGA-61-1913	LRP1B	ENST00000389484	p.I262S	c.785T>G	Unverified	TGT	ATC	CAG	2	T	G
TCGA-13-0920	LRP2	ENST00000263816	p.C3519G	c.10555T>G	Verified	CTG	TGC	GCT	1	T	G
TCGA-29-1701	LRPPRC	ENST00000260665	p.F1372C	c.4115T>G	Unverified	CCT	TTC	ATT	2	T	G
TCGA-23-1032	LRRC4C	ENST00000278198	p.G15G	c.45T>G	Verified	ATA	GGT	CCT	3	T	G
TCGA-61-1895	LRRC58	ENST00000295628	p.L287L	c.861T>G	Unverified	CTT	CTT	AGA	3	T	G
TCGA-61-1740	LRRCC1	ENST00000360375	p.S687S	c.2061T>G	Unverified	TCC	TCT	TTA	3	T	G
TCGA-61-1740	LRRCC1_ENS	ENST00000414626	p.S667S	c.2001T>G	Unverified	TCC	TCT	TTA	3	T	G
TCGA-61-1910	LRRK2	SU_LRRK2	p.F671L	c.2013T>G	Unverified	tca	ttt	gac	3	T	G
TCGA-61-1910	LRRK2_ENST	ENST00000298910	p.F671L	c.2013T>G	Unverified	TCA	TTT	GAC	3	T	G
TCGA-13-0913	LYG2	ENST00000333017	p.P79P	c.237T>G	Verified	AAA	CCT	TAC	3	T	G
TCGA-61-2095	MAPKAPK3_	ENST00000446044	p.V39G	c.116T>G	Unverified	GCA	GTG	ACC	2	T	G
TCGA-29-1761	MATN3	ENST00000407540	p.L82W	c.245T>G	Unverified	CCC	TTG	GAC	2	T	G
TCGA-29-1777	MCM3AP	ENST00000397708	p.S1466R	c.4398T>G	Unverified	aag	agt	gag	3	T	G
TCGA-29-1777	MDGA2	ENST00000357362	p.V440G	c.1319T>G	Unverified	ATT	GTT	GCA	2	T	G
TCGA-29-1777	MDGA2_EN	ENST00000399232	p.V738G	c.2213T>G	Unverified	ATT	GTT	GCA	2	T	G
TCGA-29-1777	MDGA2_EN	ENST00000426342	p.V440G	c.1319T>G	Unverified	ATT	GTT	GCA	2	T	G

[truncated: 33,649 more chars]
