# Supplementary material for: Association between targeted somatic mutation (TSM) signatures and HGS‐OvCa progression
Source: Cancer Med. 2016 Aug 3;5(9):2629–40. doi: 10.1002/cam4.825 (PMC5055158; doi:10.1002/cam4.825)
Supplement: Supplementary file 3 — Table S3. Mutation Data. (Lindley et al., 2015) TSM Mutations. [file CAM4-5-2629-s003.pdf]

Supporting Information Table S2.
Mutation Data. (Lindley et al, 2015)

TSM MUTATIONS

AID

WRCGSS	MC1	MC2	MC3
C>A	1	3	4
C>G	5	4	5
C>T	**45**	13	45

P-value 5.18E-39

APOBEC3B

TCGA	MC1	MC2	MC3
C>A	3	0	13
C>G	14	1	6
C>T	**21**	12	**25**

P-value 5.8E-12

**ATCS**	MC1	MC2	MC3
C>A	12	8	19
C>G	10	7	20
C>T	25	12	**63**

P-value 9.76E-26

APOBEC3G

**GCGGC**	MC1	MC2	MC3
C>A	1	0	1
C>G	3	0	0
C>T	**30**	13	13

P-value 1.06E-25

CCGX	MC1	MC2	MC3
C>A	11	4	12
C>G	14	4	11
C>T	**92**	34	59

P-value 1.78E-57

ZCCG	MC1	MC2	MC3
C>A	13	5	5
C>G	9	6	9
C>T	**100**	45	71

P-value 2.64E-71

SGGRR	MC1	MC2	MC3
G>A	56	**75**	70
G>C	13	22	10
G>T	19	26	18

P-value 1.62E-31

**GCGC**	MC1	MC2	MC3
G>A	17	**59**	17
G>C	3	3	1
G>T	5	5	5

P-value 2.63E-44

**CCGGC**	MC1	MC2	MC3
G>A	5	**16**	6
G>C	0	3	0
G>T	2	1	1

P-value 5.57E-11

TCCG	MC1	MC2	MC3
C>A	5	2	2

C>G	3	0	3
C>T	54	18	**37**

P-value 3.28E-45

ADARS

RAWA	MC1	MC2	MC3
A>C	12	13	14
A>G	37	21	25
A>T	**46**	19	18

P-value 1.37E-09

WTAW	MC1	MC2	MC3
A>C	10	2	10
A>G	**30**	16	11
A>T	21	4	10

P-value 1.6E-09

SARA	MC1	MC2	MC3
A>C	13	9	18
A>G	**56**	38	24
A>T	43	44	29

P-value 1.47E-13

TWTY	MC1	MC2	MC3
T>A	22	11	21
T>C	11	**26**	**36**
T>G	4	12	6

P-value 1.11E-10
